# Enantioselective Nitrene Transfer to Hydrocinnamyl Alcohols and Allylic Alcohols Enabled by Systematic Exploration of the Structure of Ion-Paired Rhodium Catalysts

**DOI:** 10.1021/jacs.4c07117

**Published:** 2024-07-31

**Authors:** Nicholas J. Hodson, Shotaro Takano, Alexander Fanourakis, Robert J. Phipps

**Affiliations:** Yusuf Hamied Department of Chemistry, University of Cambridge, Lensfield Road, Cambridge CB2 1EW, United Kingdom

## Abstract

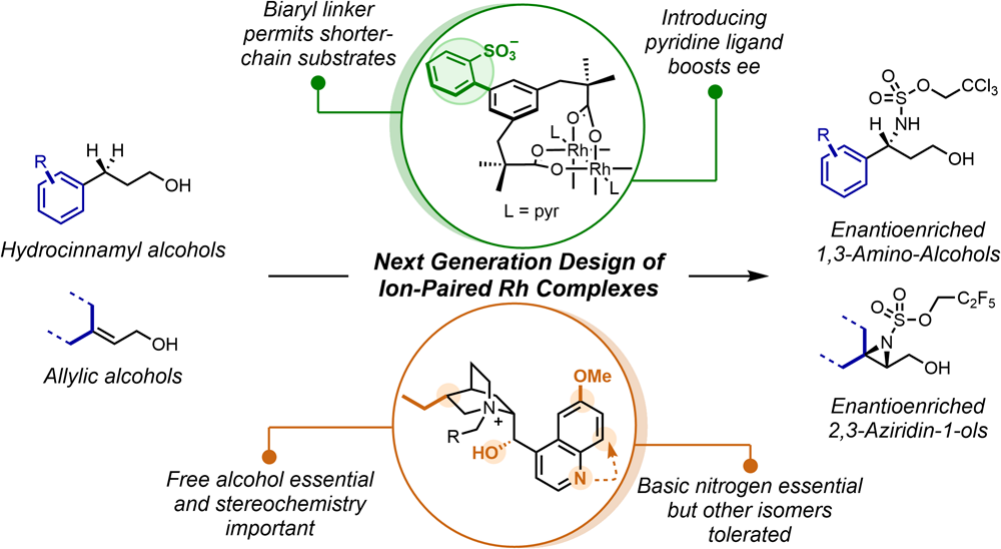

This work describes highly enantioselective nitrene transfer to hydrocinnamyl alcohols (benzylic C–H amination) and allylic alcohols (aziridination) using ion-paired Rh (II,II) complexes based on anionic variants of Du Bois’ esp ligand that are associated with cinchona alkaloid-derived chiral cations. Directed by a substrate hydroxyl group, our previous work with these complexes had not been able to achieve high enantioselectivity on these most useful short-chain compounds, and we overcame this challenge through a combination of catalyst design and modified conditions. A hypothesis that modulation of the linker between the anionic sulfonate group and the central arene spacer might provide a better fit for shorter chain length substrates led to the development of a new biaryl-containing scaffold, which has allowed a broad scope for both substrate classes to be realized for the first time. Furthermore, we describe a systematic structural “knockout” study on the cinchona alkaloid-derived chiral cation to elucidate which features are crucial for high enantioinduction. *De novo* synthesis of modified scaffolds led to the surprising finding that for high ee the quinoline nitrogen of the alkaloid is crucial, although its location within the heterocycle could be varied, even leading to a superior catalyst. The free hydroxyl is also crucial and should possess the naturally occurring diastereomeric configuration of the alkaloid. These findings underline the privileged nature of the cinchona alkaloid scaffold and provide insight into how these cations might be used in other catalysis contexts.

## 1 Introduction

**Figure 1:**
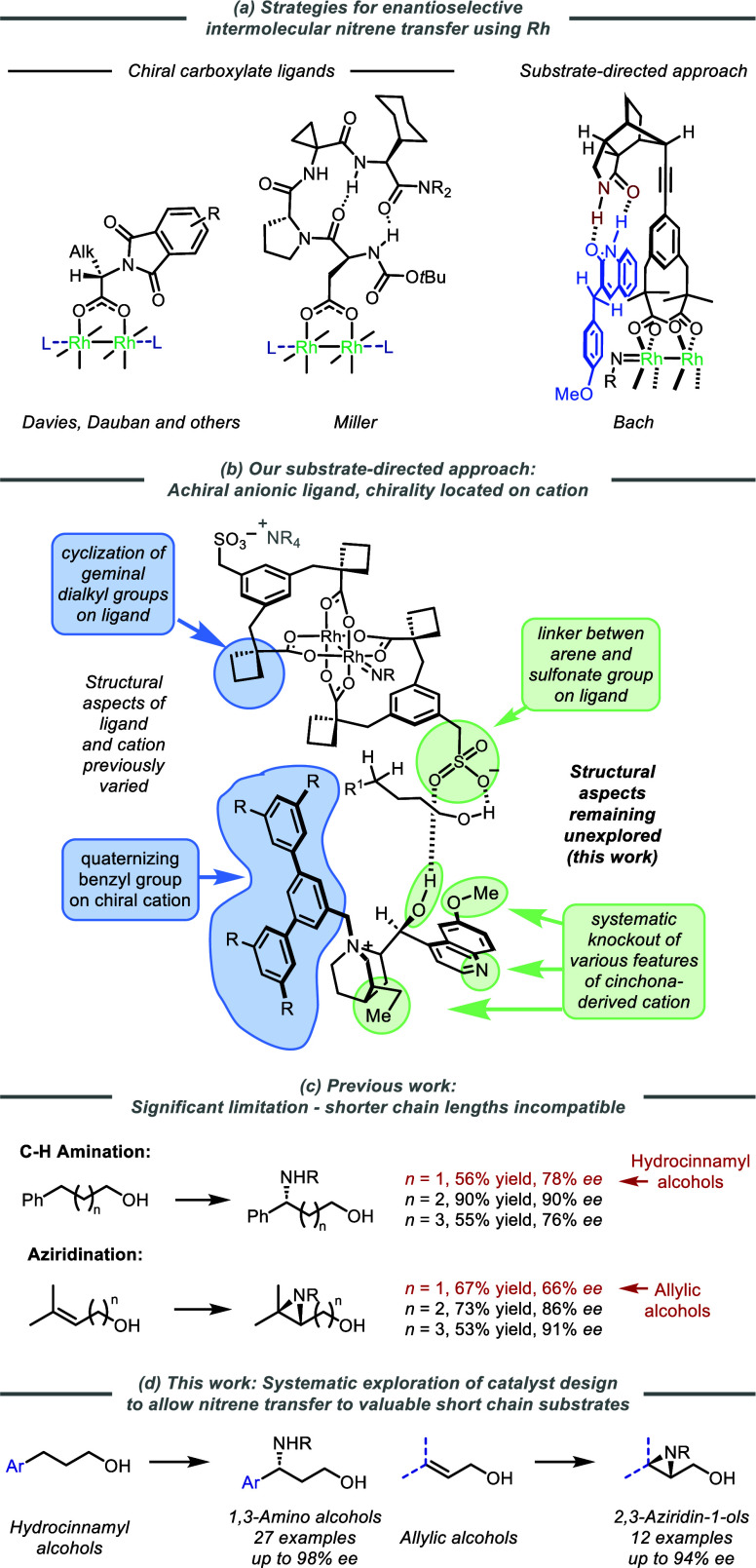
Background and approach in this study.

Nitrogen-containing molecules are ubiquitous, and it is important to continue to develop and refine amination methods. Nitrenes are, in principle, attractive reactive nitrogen-based intermediates, which can engage in a range of reactions.^1^ Free nitrenes themselves, however, are typically too reactive to allow controlled, selective intermolecular processes. In contrast, catalytic approaches that proceed via metal nitrenoids have proven incredibly versatile in permitting a broad range of C–H amination and alkene aziridination processes through insertion into C–H bonds and addition to alkenes, respectively.^2^ A number of metals are proficient at forming metal nitrenoids, and among these, rhodium (II,II) paddlewheel complexes have proven very effective, and Rh-catalyzed amination and aziridination have become mainstream synthetic methods, aided by the popularization of Du Bois’ Rh_2_(esp)_2_ as an active and functional group tolerant catalyst.^3^ A continuing challenge in the field is that of developing generally applicable ligand strategies for asymmetric nitrene transfer.^2^ An established approach for rhodium paddlewheels involves the replacement of the achiral carboxylate ligands with chiral variants,^4^ as demonstrated in early work on amination of benzylic C–H bonds from Hashimoto et al.,^5^ Müller et al.,^6^ Davies and Reddy,^7^ and Du Bois and Zalatan^8^ and followed by recent important intermolecular contributions from Dauban et al. (Figure 1a, left).^9^ Very recently, Miller et al. have developed novel peptide-derived carboxylate ligands, which, through a well-defined hydrogen bonding network, form a chiral pocket in which benzylic C–H amination occurs (Figure 1a, center).^10−12^ Applied to aziridination, chiral Rh carboxylate complexes have been recently showcased by Dauban and co-workers as being very effective on trisubstituted styrenes and terminal aliphatic alkenes.^13,14^ Using a distinct approach, Rh(III) Cp* complexes have also been used productively in enantioselective allylic amination^15^ and aziridination^16^ reactions. Stereoinduction using chiral carboxylate complexes generally relies on repulsive interactions alone. In comparison, substrate-directed catalysis involves a functional group on the substrate interacting with the catalyst to assist the organization at the transition state and can offer substantial advantages so long as the functional group is common and synthetically useful.^17^ This has been explored by Bach and co-workers in the context of Rh-catalyzed enantioselective nitrene transfer: a dual hydrogen bonding interaction was designed between a lactam substrate and a modified version of Rh_2_(esp)_2_ appended with its own chiral lactam (Figure 1a, right).^18^ This permitted C–H amination with a maximum 74% *ee*(18a) and was subsequently also applied to aziridination for which the *ee* values were higher, but the substrate variability was limited to specific motifs.^18b^ We recently disclosed a conceptually distinct strategy for substrate-directed asymmetric nitrene transfer whereby a derivative of Rh_2_(esp)_2_, rendered anionic by the addition of sulfonate groups, is ion-paired with a chiral cinchona alkaloid-derived cation.^19^ It is the latter that provides the chiral environment in which nitrene transfer occurs, and we envisioned that the quaternized cinchona scaffold would provide rich opportunities for engaging in attractive noncovalent interactions with the substrate (Figure 1b).^20,21^ We anticipated that a hydroxyl group of the substrate may hydrogen bond with the sulfonate group of the ligand to create a high level of organization in this ternary system. In our initial proof-of-concept study, we demonstrated that such ion-paired Rh complexes were effective for enantioselective C–H amination at the benzylic position of 4-phenylbutanol and analogues (as depicted in Figure 1b).^19a^ However, shortening or lengthening the alkyl chain separating the alcohol and the arene was detrimental. In particular, amination of hydrocinnamyl alcohol occurred in only 78% *ee*, a serious limitation given that amination of this broader substrate class leads to γ-amino alcohols, important chiral building blocks present in various pharmaceutical agents, as well as direct precursors to β-amino acids upon oxidation (Figure 1c, upper). We subsequently applied our ion-paired catalysts to the aziridination of alkenyl alcohols and obtained excellent results on a range of substrates including homoallylic alcohols and those with longer chains between the alkene and the alcohol.^19b^ As before, a significant limitation was that the shortest chain length substrates, allylic alcohols, gave only a moderate *ee* (Figure 1c, lower). Yet, these would arguably be the most useful to produce small molecule chiral building blocks for synthesis (in analogy with the Sharpless asymmetric epoxidation for oxygen transfer, as opposed to nitrogen transfer). We hypothesized that these limitations might be addressed through judicious catalyst modification. The modularity of our ion-paired catalysts bestows several opportunities for variation both on the chiral cation and the achiral anionic dicarboxylate ligand. Until this point, we had made relatively limited variations, which focused on (1) the quaternizing benzyl group on the cation and (2) the geminal dialkyl groups adjacent to the carboxylate on the anionic ligand scaffold (Figure 1b, blue). Two key areas of the catalyst remain unexplored (Figure 1b, green). The first was the linker between the added sulfonate group and the backbone benzene ring in the “esp” ligand, which had always been a simple methylene group. We hypothesized that modifying this to reduce the distance between the sulfonate group and a rhodium center may enable shorter chain substrates to be better accommodated. The second is related to the array of structural features provided by nature in the cinchona alkaloid scaffold. Until this point, these had not been altered substantially and it was unclear which of these were crucial for selectivity and whether some could even be detrimental. These included the free hydroxyl group, the methoxy on the quinoline, the ethyl group on the quinuclidine, and, perhaps most intriguingly, the basic nitrogen of the quinoline ring. We herein describe a detailed study where these features are systematically knocked out to gain insight into those essential for high *ee*. Ultimately, we have been successful in identifying a modified catalyst scaffold, which allows valuable shorter-chain substrates to deliver high enantioselectivities in both nitrene-transfer processes: amination of hydrocinnamyl alcohols and aziridination of allylic alcohols (Figure 1d). During this process, we gained valuable insights into the features of the cation important for stereoinduction and identified an isomerized cation, which was combined with the new ligand scaffold to obtain an additive superior *ee* outcome. We envisage these insights will be of use to others who apply this privileged catalyst motif to enantioselective transition metal catalysis.

## 2 Results and Discussion

### 2.1 Anionic Ligand Design and Effect of Additives

**Figure 2:**
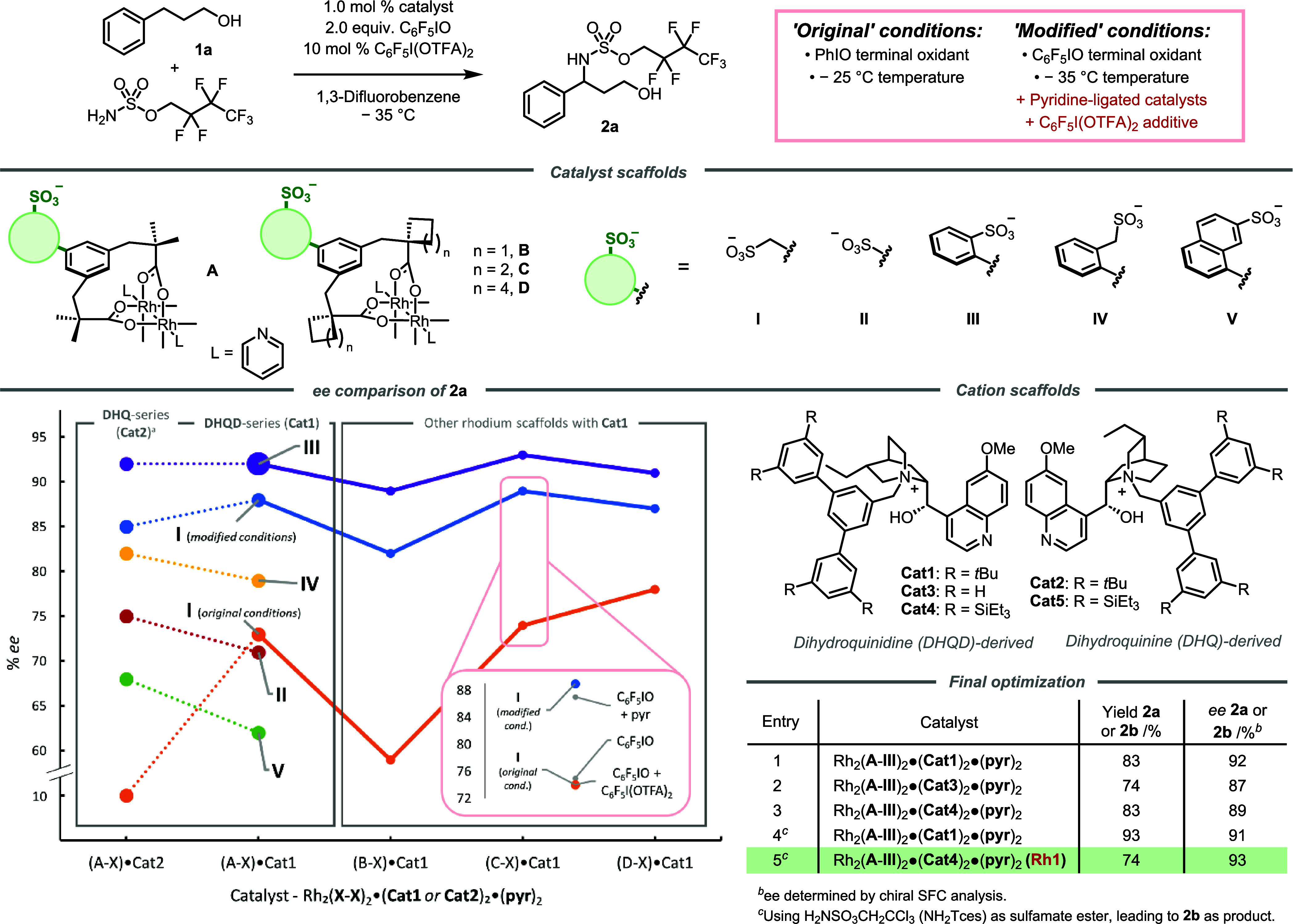
Schematic detail of structural variations in anionic Rh dimer complexes, chiral cations used in this study, and effects of previously used versus modified reaction conditions. ^a^Antipode of **2a** obtained with DHQ-derived **Cat2**.

Our prior studies on aziridination uncovered several improvements to the original reaction conditions used in our C–H amination of aryl butanols.^19a^ This included the use of a more soluble oxidant (C_6_F_5_IO vs PhIO), which allowed a lower reaction temperature (− 35 °C vs −25 °C). We also identified the beneficial impact on *ee* of a substoichiometric additive, C_6_F_5_I(OTFA)_2_, which served to release trifluoroacetic acid as the reaction progressed. Finally, the use of a Rh complex ligated by two pyridine molecules also gave an important increase in *ee*.^19b^ Collectively these changes comprise what we now refer to as the “modified reaction conditions” (Figure 2, top scheme). Although the exact role of the latter two alterations was unclear, we speculated that they may modulate a binding equilibrium between the cation-based quinoline nitrogen and the axial coordination sites on the Rh-dimer. Throughout this article, we use a catalyst naming convention that highlights the four variables for a given Rh complex (Figure 2): (1) letters **A**-**D** represent the substitution α to the ligand carboxylate groups, (2) numerals **I**–**V** represent the different linkers between sulfonate and arene, (3) **Cat1**–**5** represents which chiral cations are ion-paired to the Rh complex, and (4) “pyr” indicates that the complex is axially ligated by two pyridine molecules. Before evaluating novel ligand scaffolds, we first examined the modified reaction conditions on the amination of hydrocinnamyl alcohol using our previously reported catalysts (Figure 2). Using Rh_2_(**A**-**I**)_2_·(**Cat1**)_2_·(**pyr**)_2_, the aminated product **2a** was obtained in 88% *ee* (Figure 2, chart, **I** (modified conditions)), considerably higher than the 73% *ee* obtained with the analogous complex under the original C–H amination conditions (Figure 2, chart, **I** (original conditions)). We evaluated the modified conditions with the corresponding complexes in which the α-carboxylate alkyl groups are cyclized in differing ring sizes (**B**–**D**, right-hand box of the chart) and saw an *ee* increase of between 10 and 20% in all cases compared with the original conditions (blue line versus orange line), peaking with scaffold **C** at 89% *ee*. For this complex Rh_2_(**C**–**I**)_2_·(**Cat1**)_2_·(**pyr**)_2_ a detailed analysis of the effect of each change moving from the original to modified conditions was conducted (*chart*, pink insert). The use of C_6_F_5_IO at lower temperatures gave only a slight increase in *ee* (73 to 75%). The inclusion of C_6_F_5_I(OTFA)_2_ as an additive afforded a similar *ee* value (75 to 74%); however, using a pyridine-ligated Rh complex in addition to C_6_F_5_IO afforded a large increase (75 to 87% *ee*). Finally, the inclusion of the C_6_F_5_I(OTFA)_2_ additive to afford the full modified conditions gave a small further increase to 89% *ee*.

Although we were pleased that the modified reaction conditions significantly improved *ee* in the C–H amination of hydrocinnamyl alcohol, we sought further improvement through catalyst design to allow the highest levels of selectivity to be obtained. In our prior work, the methylene linker between the central arene and sulfonate group was consistent across all catalysts (Figure 2, *catalyst scaffolds*, **I**). We hypothesized that modulation of the distance between the pendant sulfonate group and the Rh–Rh axis may enable shorter chain substrates to be accommodated with high selectivity. To probe this, we designed a series of scaffolds in which the linker between the arene and the sulfonate was systematically varied. The linker was removed entirely in **II**, directly attaching the sulfonate group to the arene. In **III** and **IV** an *ortho*-substituted arene is used as the linker, with **IV** featuring an additional methylene spacer before the sulfonate. A naphthyl group is used to extend the aromatic linker in **V** while retaining rigidity. Once the dicarboxylic acids of each were accessed (using prototypical scaffold **A** featuring gem-dimethyl groups), the dimer assembly and ion-exchange steps were carried out to obtain the final ion-paired catalysts. For completeness, we synthesized dihydroquinidine (DHQD)- and dihydroquinine (DHQ)-derived cations (**Cat1** and **Cat2**) for all scaffolds using the same quaternizing group to probe any enantioselectivity gap between the two pseudoenantiomeric cations for each. The outcomes of these catalysts in the amination of hydrocinnamyl alcohol are shown in the left-hand box of the chart. The poorest outcome was obtained with naphthyl-linked **V** which afforded **2a** in only 62% *ee* using **Cat1** (vs 88% with the original methylene-linked scaffold **I**). Scaffold **II**, bearing no linker, also performed poorly giving only 71% *ee*. We were pleased to observe that biaryl scaffold **III** gave an increase in *ee* to 92%, constituting the best catalyst so far. Extending this linker by an additional methylene in **IV** resulted in a drop in *ee* to 79%. Interestingly, the relative performance of DHQD- and DHQ-derived catalysts varied significantly between scaffolds: for **V**, **II**, and **IV** the DHQ-derived complexes gave higher *ee* than the DHQD-derived, in contrast to original scaffold **I** where DHQD was superior (the relative ordering of complexes in terms of *ee* remained unchanged). Gratifyingly, the new biaryl scaffold **III** was unique in exhibiting no *ee* difference between the two pseudoenantiomeric complexes: 92% *ee* with cation **Cat1** and (−)92% *ee* with cation **Cat2**. This represents a considerable advantage, as each product enantiomer is now accessible with equally high *ee* (a tedious process of devinylation of the alkaloid was required to access the same magnitude for substrates examined in our previous work). With a new optimal scaffold identified in **III**, the various cyclic surrogates for the gem-dimethyl group (**B**–**D**) were synthesized and tested (right-hand box, purple line). Only the cyclopentyl analogue **C** was competitive in *ee*, but a lower yield of **2a** meant that the simpler gem-dimethyl complex (Rh_2_(**A**-**III**)_2_·(**Cat1**)_2_·(**pyr**)_2_) was taken forward. A brief survey of quaternizing groups on the cation (inset Table) showed that removal of the peripherial *t*Bu groups (**Cat3,** entry 2) and enlargement to SiEt_3_ groups (**Cat4**, entry 3) were both slighly detrimental to *ee*. Interestingly, the use of NH_2_Tces as the amine source in place of the perfluorinated sulfamate ester used thus far gave similarly high levels of enantioselectivity (entry 4), which could be slightly improved to 93% *ee* by switching to cation **Cat4** (entry 5). The use of NH_2_Tces as an alternative aminating agent is advantageous due to its simple and mild deprotection conditions.^22^ The optimal ion-paired catalyst Rh_2_(**A**-**III**)_2_·(**Cat4**)_2_·(**pyr**)_2_ shall now be referred to as **Rh1** for brevity.

### 2.2 Scope Evaluation for C–H Amination

**Scheme 1:**
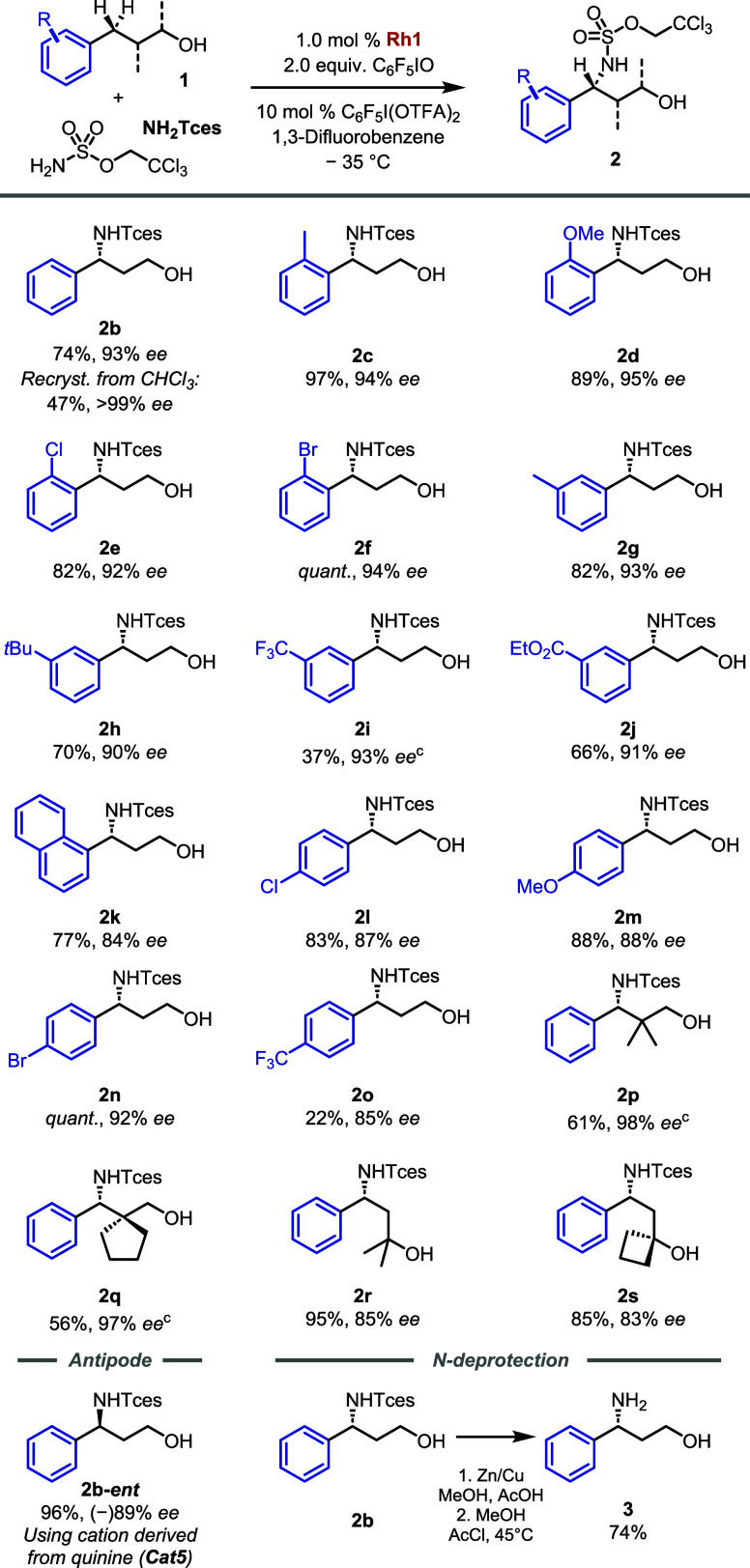
Enantioselective C–H Amination of Hydrocinnamyl Alcohols, *ee* determined by chiral SFC analysis of the purified amino alcohol product. NHTces = HNSO_3_CH_2_CCl_3_. Using 2.0 mol % catalyst at −25 °C.

With NH_2_Tces as an aminating agent, we evaluated the scope of substituted hydrocinnamyl alcohols using **Rh1** (Scheme 1). We were pleased to observe that high reactivity and enantioselectivity could be obtained for a range of *ortho*-substituted substrates, which have proven very challenging in other benzylic C–H amination protocols, including in our previous work. This encompassed *ortho* methyl, methoxy, chloro, and bromo substituents (**2c**–**2f**). *Meta*-substitution was well tolerated, including alkyl groups (**2g**, **2h**) and inductively (**2i**) as well as conjugatively (**2j**) withdrawing groups. A naphthyl substrate was compatible (**2k**) as were a range of *para*-substituted hydrocinnamyl alcohols (**2l**-**2o**). Lower yields for **2i** and **2o** can be attributed to the strongly withdrawing CF_3_ group deactivating the benzylic C–H bonds toward nitrene insertion. Substitution on the alkyl chain could be incorporated on the central carbon, with **2p** and **2q** obtained in very high *ee* (98 and 97%) despite the amination occurring on a very hindered methylene. In contrast, trace amounts of **2p** and **2q** were afforded when Rh_2_(esp)_2_ was used as the catalyst. Tertiary alcohol directing groups could also be used with only a minimal impact on *ee* (**2r** and **2s**). The antipodal product **2b-*ent*** was obtained using the DHQ-derived version of **Rh1**, Rh_2_(**A**-**III**)_2_·(**Cat5**)_2_·(**pyr**)_2_ (from herein abbreviated as **Rh2**), in high *ee*. Finally, the Tces protecting group was removed to give **3** from which the absolute configuration could be determined by comparison with the literature and all others assigned by analogy. A benefit of the Tces protecting group was that amino alcohols **2** were often solids, and a simple recrystallization procedure could access **2b** in >99% *ee*.

**Scheme 2:**
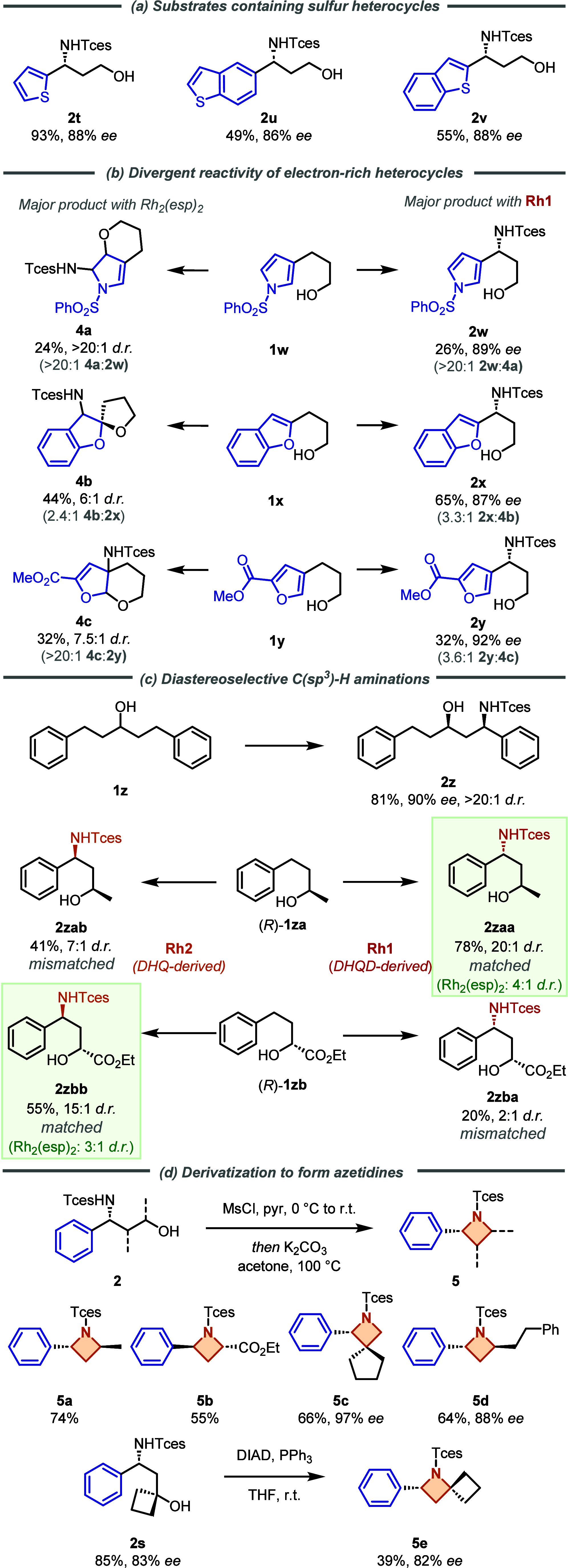
Heterocyclic and Secondary Alcohol Substrates

We next investigated heterocyclic substrates, focusing first on sulfur heterocycles (Scheme 2a). A 2-substituted thiophene gave excellent results (**2t**) as did both 5- and 2-substituted benzothiophenes (**2u**, **2v**). In moving to more electron-rich heteroarenes, we observed an intriguing switch in chemoselectivity using **Rh1** compared with Rh_2_(esp)_2_ (Scheme 2b). For pyrrole-containing **1w**, aziridination on the heteroarene followed by ring-opening afforded **4a** as the major product with Rh_2_(esp)_2_. In contrast, **Rh1** gave benzylic amination as the major product in high *ee* (**2w**). The low mass balance of the reaction is attributed to the apparent decomposition of side products. The same trend was observed for benzofuran-containing **1x** and furan-containing **1y**. In both cases, the benzylic amination products **2x** and **2y** were obtained with **Rh1**, in contrast to the dearomatized products **4b** and **4c** obtained with Rh_2_(esp)_2_. These divergent chemoselectivity outcomes highlight the powerful control our ion-paired catalysts have on the reaction outcome is not limited to enantioselectivity, a benefit of the substrate-directed strategy employed which presumably directs the reaction to a site that would not be typically functionalized using a nondirected approach. We next sought to challenge the control of diastereoselectivity as well as enantioselectivity in achiral secondary alcohol **1z** (Scheme 2c). Control of both metrics was very high, with essentially a single diastereomer obtained in excellent yield and *ee* (**2z**). Next, we examined how the catalyst might deal with substrates featuring an existing, defined stereocenter. Commercially available (*R*)-**1za**, which gave **2zaa** in moderate *d.r.* of 4:1 using Rh_2_(esp)_2_, gave **2zaa** in >20:1 *d.r.* using **Rh1**, indicating a strong match between intrinsic selectivity and the catalyst. In contrast, the use of pseudoenantiomeric **Rh2** (featuring the DHQ-derived cation **Cat5** rather than DHQD-derived **Cat4**) gave **2zab** in 7:1 *d.r.* in favor of the previously minor diastereomer, demonstrating the powerful ability of our ion-paired catalyst to override an existing stereocenter. We also evaluated (*R*)-**1zb,** featuring an ester rather than a methyl at the alcohol stereocenter and with the opposite absolute stereochemistry. In this case, the matched catalyst was **Rh2**, which gave **2zbb** in 15:1 *d.r.* in the same sense as Rh_2_(esp)_2_ (3:1 *d.r*.). With **Rh1**, the overriding effect of the catalyst was not as strong, but the previously minor diastereomer **2zba** could be obtained in 2:1 *d.r*. The 1,3-amino alcohol relationship in our products provides the opportunity to access valuable azetidine scaffolds through the facile intramolecular displacement of the alcohol. To showcase this, we utilized a mesylation/cyclization procedure to access several azetidines **5a**–**5d** bearing different substitution patterns (Scheme 2d). Tertiary alcohol **2s** was cyclized under Mitsunobu conditions to afford the spirocycle **5e**, with a lower yield in this case due to competing elimination pathways.

**Scheme 3:**
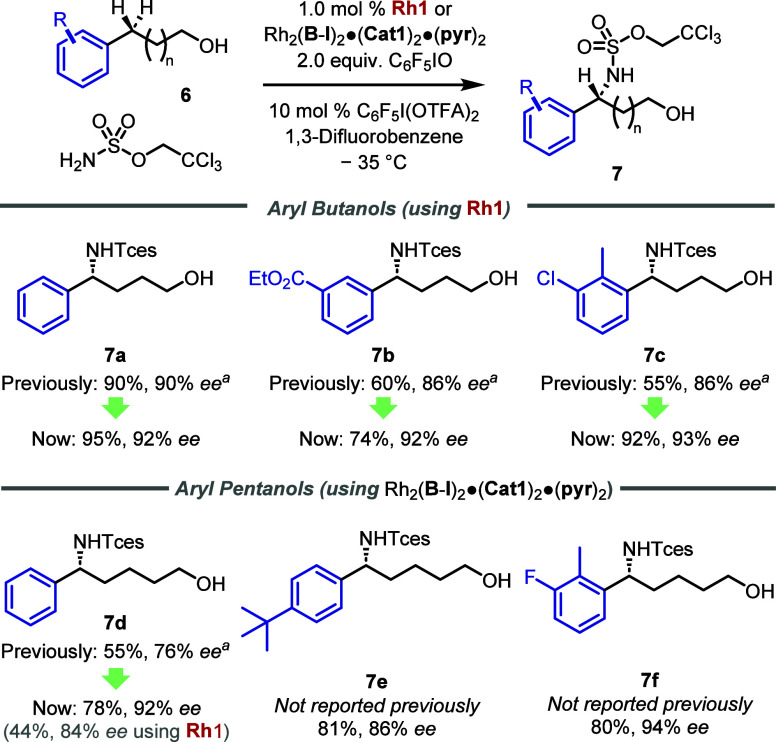
Application of Modified Catalytic System to Aryl Butanols and Aryl Pentanols Previously reported with H_2_NSO_3_CH_2_C_3_F_7_ as the aminating agent instead of H_2_NTces, under the “original” reaction conditions: catalyst (1.0 mol %), PhIO (2.0 equiv), H_2_NSO_3_CH_2_C_3_F_7_ (1.2 equiv), 1,3-difluorobenzene (0.2 M), −25 °C.

As the new catalyst scaffold **III** combined with the modified reaction conditions had been successful on hydrocinnamyl alcohols, we questioned whether other chain lengths from our initial study may also be improved.^19a^ We found that the updated conditions together with the new catalyst **Rh1** improved both yield and *ee* on 4-arylbutanol substrates (Scheme 3, **7a**–**7c**). For the longer chain 5-phenylpentanol, the “original” conditions and catalyst gave **7d** with only 76% *ee*.^19a^ Use of the new biaryl catalyst **Rh1** and modified conditions increased this to 84% *ee*. However, we found that *ee* could be improved to 92% by using the previously identified benzyl sulfonated catalyst Rh_2_(**B**-**I**)_2_·(**Cat1**)_2_·(**pyr**)_2_. Several substrates were examined successfully (**7d**–**7f**). This catalyst matching reinforces that the biaryl linker is most suitable for shorter chain lengths, while the original benzyl linker is still optimal for longer chains, in line with our original hypothesis.

### 2.3 Aziridination of Allylic Alcohols

**Scheme 4:**
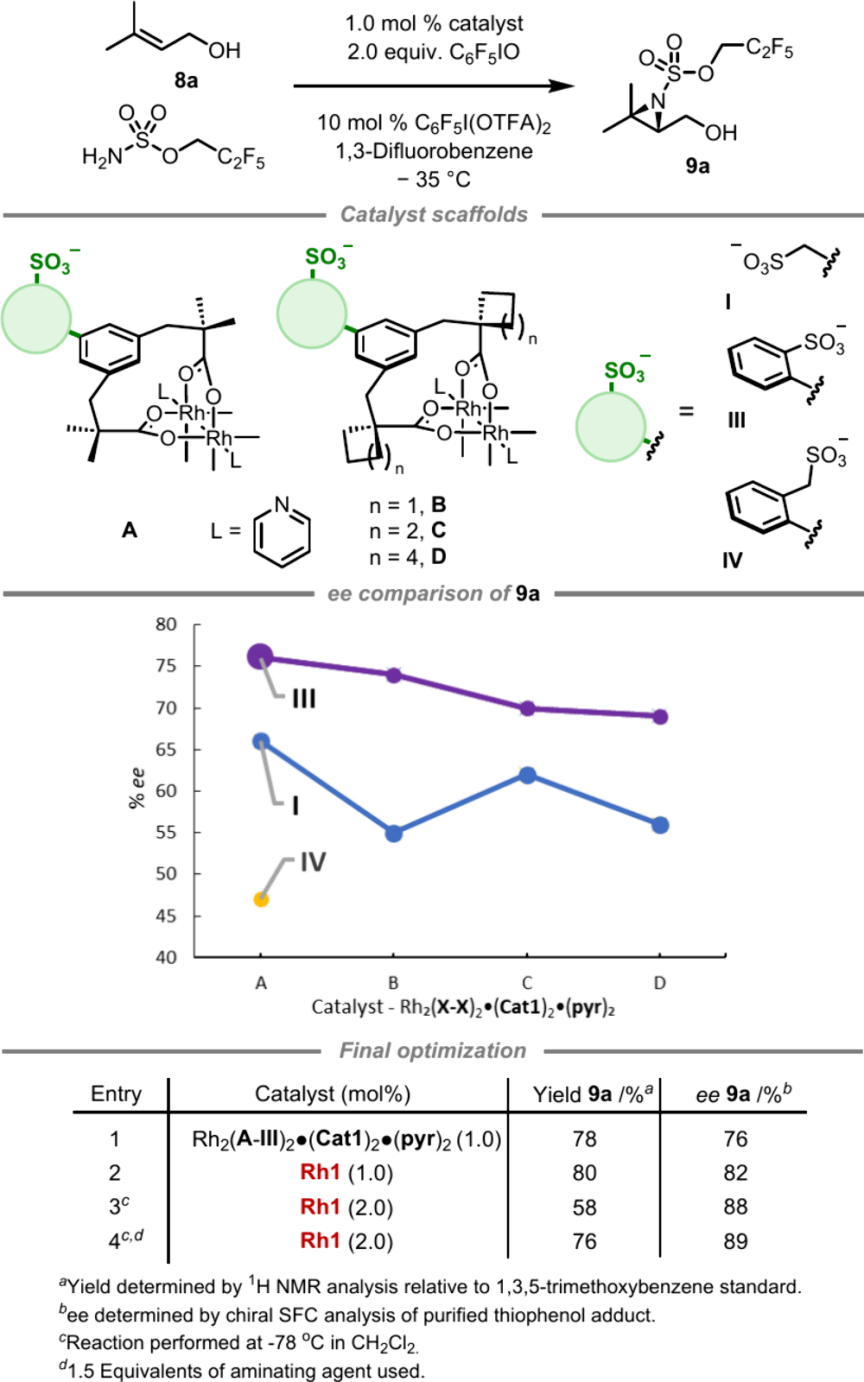
Catalyst Comparison on Allylic Alcohol Aziridination

In our previous aziridination work, Rh_2_(**B**-**I**)_2_·(**Cat4**)_2_·(**pyr**)_2_ was the optimal catalyst for nitrene transfer to homoallylic, bishomoallylic, and trishomoallylic alkenyl alcohol substrates.^19b^ Disappointingly, we observed a significant drop in *ee* for allylic alcohols, arguably the most useful class of the set (Figure 1c). Given the prevalence and utility of the allylic alcohol motif, we decided to evaluate the most promising of our new catalyst scaffolds for their enantioselective aziridination and selected the trisubstituted alkene prenol for optimization (**8a**) (Scheme 4). Across α-carboxylate groups **A**–**D**, our previous benzyl sulfonate scaffold **I** delivered aziridine **9a** in the range 55–66% *ee* (Scheme 4, chart). We thus explored the biaryl scaffold **III** and immediately observed an improvement. Across groups **A**–**D**, **III** consistently gave 10–20% higher *ee* than the counterparts with **I**, peaking with **A**-**III**, which gave 76% *ee*. This greater increase compared to the shorter-chain C–H amination may reflect the lower flexibility of the allylic alcohol, meaning that the biaryl linker is more important to effectively position the chiral pocket for allylic alcohol aziridination. The use of Tces as aminating agent gave inferior enantioselectivity in this case (see SI for details). As in the C–H amination, extending **III** to give scaffold **IV** resulted in poorer performance, giving **9a** in only 47% *ee*. Returning to **III**, the slightly larger chiral cation **Cat4** gave a small improvement in *ee* (Table, entry 2 vs 1). Employing 2.0 mol % of catalyst at −78 °C using **Rh1** enabled an ee of 88% (entry 3), and we found that high reactivity could be maintained by using a larger excess of aminating agent and a longer reaction time (entry 4).

**Scheme 5:**
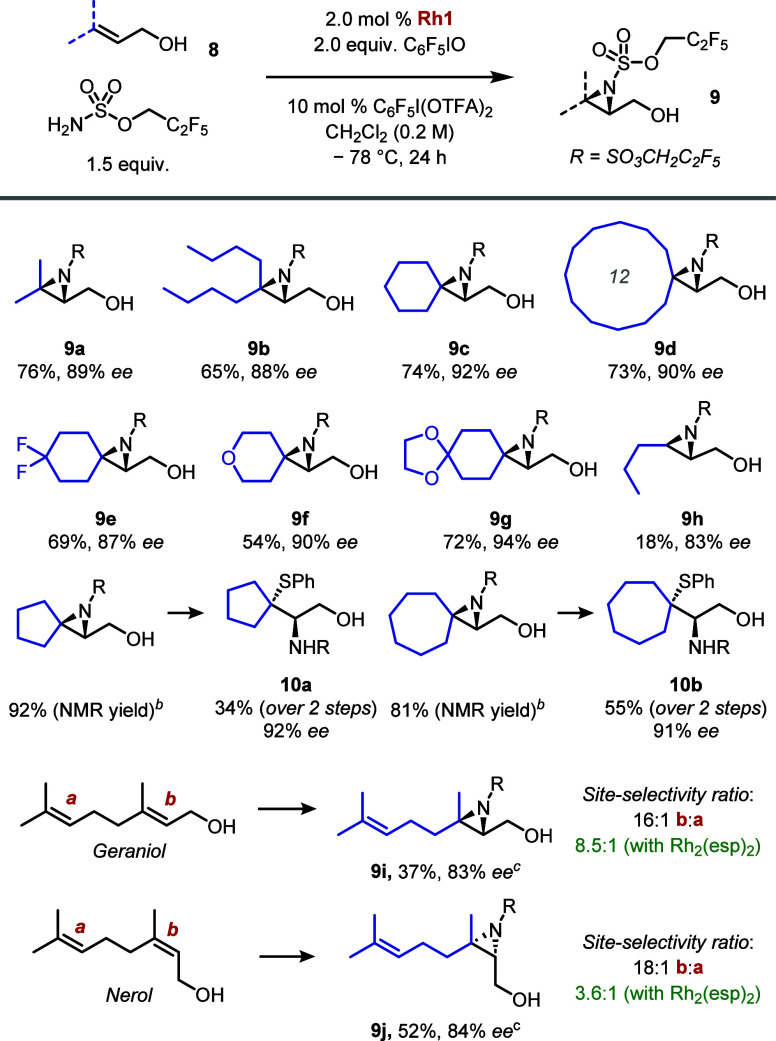
Scope of Allylic Alcohol Aziridination In all cases, ee was determined by chiral SFC analysis of the purified thiophenol adduct. Degradation of these aziridines occurred upon attempted purification, see the Supporting Information for details. 1.05 equiv of sulfamate ester used.

Having identified suitable conditions for the aziridination of prenol, we next explored the scope of the reaction on related trisubstituted allylic alcohols using the optimal catalyst **Rh1** (Scheme 5). Longer alkyl chains were well tolerated (**9b**) as well as a cyclohexyl ring (**9c**) and a larger cyclododecyl (**9d**) ring. Additional functional groups could be included at the 4-position of the cyclohexyl ring; a gemdifluoro (**9e**), 4*H*-pyran (**9f**), and protected ketone (**9g**) also gave highly enantioenriched aziridines. **Rh1** could accommodate a *trans*-alkyl substrate with good levels of *ee* (**9h**); however, competing oxidation of the allylic alcohol to the corresponding enal, presumably occurring due to slower aziridination, precluded a high yield under the present conditions. The corresponding *cis* isomer also gave poor yield but lower *ee* (see SI for details). Although yields were high, styrenyl allylic alcohols did not give good enantioselectivity outcomes under similar conditions (see SI for details). High enantioselectivity was maintained for cyclopentyl and cycloheptyl examples; however, degradation of the aziridine upon isolation was observed, and these were transformed to the corresponding thiophenol adducts **10a** and **10b** to enable characterization. Next, we examined the ability of **Rh1** to control site selectivity as well as enantioselectivity in the aziridination of nerol and geraniol. Although Rh_2_(esp)_2_ also has a slight preference for the proximal alkene ***b*** in both cases, **Rh1** gave considerably higher ratios of 16:1 (geraniol) and 18:1 (nerol) while affording the desired aziridines (**9i** and **9j**) in high *ee*.^23^

### 2.4 Evaluation of Cation Structural Features

**Figure 3:**
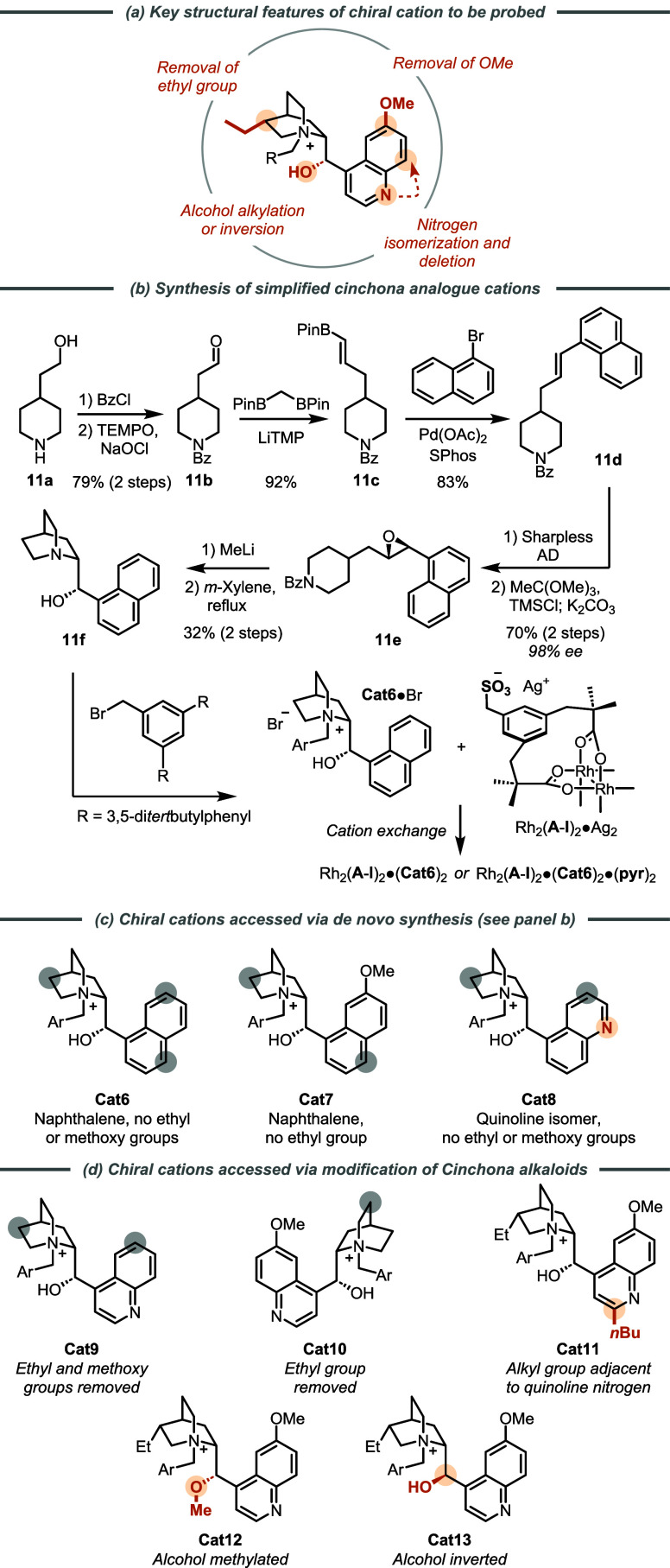
Synthesis of structurally varied Cinchona alkaloid analogues.

Having thoroughly evaluated changes to the achiral portion of the complex, we turned our attention to the systematic variation of the chiral cation. Throughout our studies so far using ion-paired catalysts, we have relied on chiral cations derived from naturally occurring quinine and quinidine. Due to the good performance in a range of substrates our ventures into scaffold variation had been mainly limited to the nature of the aromatic *N*-quaternizing group. However, there are several distinctive structural features possessed by these alkaloids, and we were intrigued to probe their importance in a systematic manner by “building up” the features from a simpler version to gauge the impact on selectivity. These features comprise the following: the methoxy group on the quinoline ring, the ethyl group on the quinuclidine, the free hydroxyl group adjacent to the quaternized quinuclidine, and, perhaps most challengingly, the basic nitrogen of the quinoline (Figure 3a). The latter in particular demands critical assessment, as the inclusion of a basic heteroarene into the cation architecture would certainly be a counterintuitive move in any *de novo* design due to the possibility of deleterious ligation of the metal center. In our first study involving these catalysts, we established that axial ligation of the Rh dimer (via quinoline *N*-ligation) was occurring in solution, as evidenced by distinctive bands in the UV–visible spectra.^19a^ In subsequent work on asymmetric aziridination, we found that the use of a Rh complex ligated by two molecules of pyridine increased *ee* by ∼10%.^19b^ We found a similar increase in enantioselectivity through the addition of a substoichiometric amount of a weak acid additive, specifically C_6_F_5_I(OTFA)_2_, which releases trifluoroacetic acid *in situ*. We supposed that these two factors together might influence a putative binding equilibrium between the Rh complex and cation. For this reason, we were particularly intrigued to remove the quinoline nitrogen from the cation to assess whether this would negate the requirement for the various additives and even improve selectivity; perhaps, these were needed to “correct” problems stemming from the presence of the quinoline nitrogen in the parent alkaloid?

Addressing this question necessitated a *de novo* synthetic approach to nonheteroaromatic cinchona analogues. Lygo and co-workers previously asked similar questions in the context of enolate alkylation using phase transfer catalysis and devised a route, based on Sharpless Asymmetric Dihydroxylation (AD),^24^ to a simplified cinchona scaffold, which lacked the vinyl group and permitted the quinoline to be replaced with various arenes at an early stage.^25,26^ We have modified Lygo’s approach to use Suzuki-Miyaura coupling for the introduction of the aromatic fragment,^27^ with the final route shown in detail for the synthesis of cation **Cat6** in which the 6-methoxyquinoline of the quinine/quinidine alkaloids is replaced by a naphthalene (Figure 3b). This route allowed access to the following further cations, depicted in Figure 3c: **Cat7**, which lacks the quinoline nitrogen but retains the methoxy on the naphthalene, and **Cat8**, which isomerizes the 4-substituted quinoline of the alkaloids to a 5-substituted analogue. We attempted to access the corresponding isoquinoline analogue of **Cat8** but the Sharpless AD delivered only moderate *ee* in this case, precluding its evaluation. In addition to these *de novo* synthesized scaffolds, we accessed others of interest from the derivatization of the alkaloids (Figure 3d, see the SI for details). This included versions in which the ethyl and methoxy groups are removed (**Cat9**), just the ethyl is (**Cat10**), a variant in which an *n*-butyl group is introduced at the quinoline C2 position (**Cat11**), one in which the hydroxyl group is methylated (**Cat12**) and one in which the hydroxyl stereochemistry is inverted (**Cat13**).

For consistent comparison, all of the above were quaternized using the 3,5-bis-*tert*butyl-substituted *m*-terphenyl benzylating group possessed by original cations **Cat1** and **Cat2** at the outset of this work. Similarly, all were paired with the anionic Rh dimer scaffold Rh_2_(**A**-**I**)_2_ which bears a gem-dimethyl as the geminal dialkyl group and a benzyl sulfonate linker. However, the method of chiral ion-pairing required variation depending on the cation used (Figure 3b, lower). Typically, we perform cation exchange by simply stirring a slight excess of the sodium salt of the bis-sulfonated Rh complex with chiral cation bromide in a biphasic solvent mixture. The correct ratio between the two is usually easily obtained in the organic layer and the exchange is associated with a striking solution color change from green to red, consistent with the axial ligation of the Rh complex, presumably by the quinoline nitrogen of the cation. This proceeded as normal for cations in which the heteroaromatic nitrogen was present and unencumbered. In contrast, cations **Cat6**, **Cat7**, and **Cat11** did not give complete ion exchange under these conditions and necessitated the formation of the silver salt of the Rh complex to drive the exchange to completion by precipitation of silver bromide. Furthermore, the solutions for the latter remained green after the exchange. These observations together suggest that when the quinoline nitrogen is not present (**Cat6**, **Cat7**) or is encumbered (**Cat11**), axial ligation by the cation is precluded.

**Figure 4:**
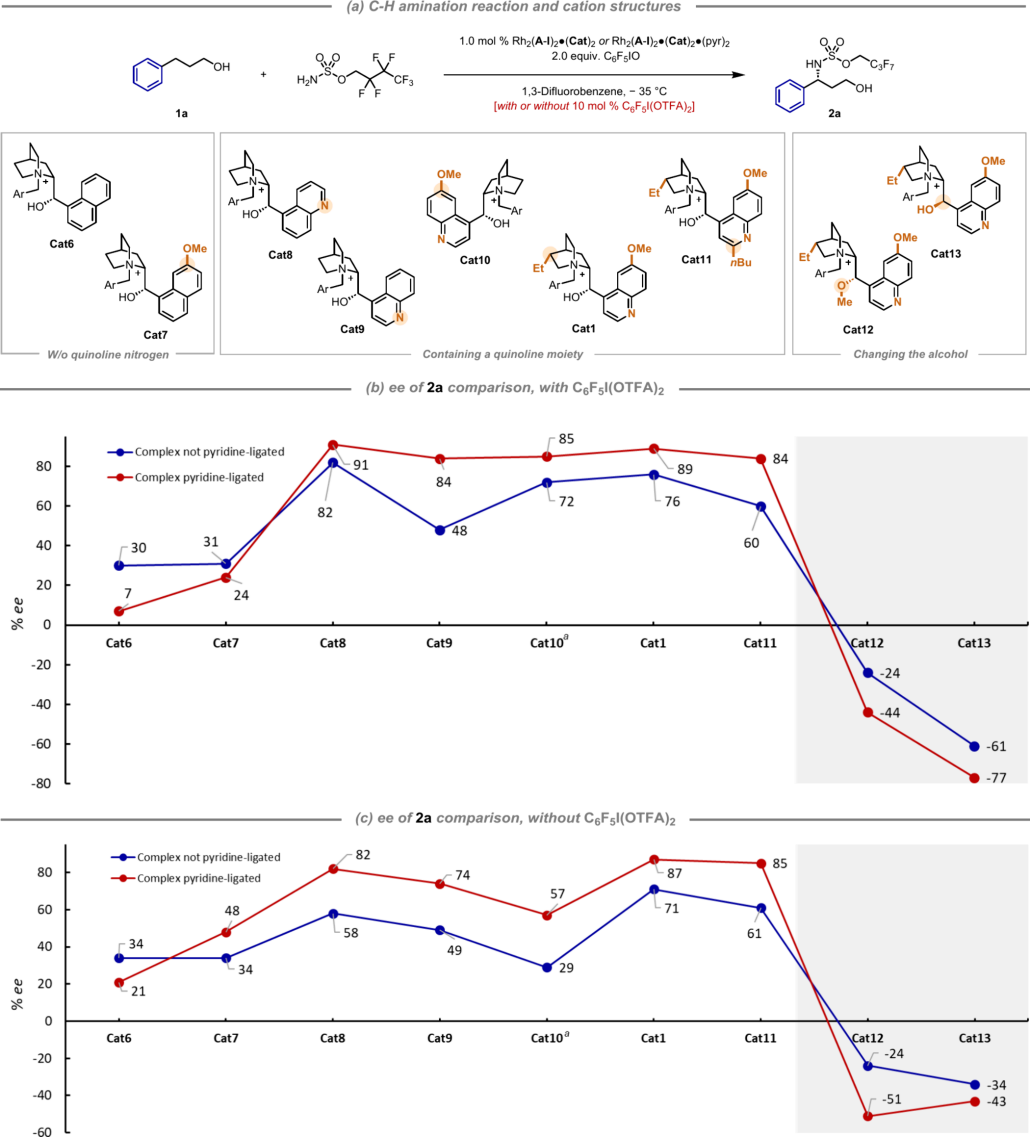
Evaluation of Rh complexes containing (a) cations **Cat1** & **Cat6**-**Cat13**. (b) C–H Amination of hydrocinnamyl alcohol with 10 mol % of C_6_F_5_I(OTFA)_2_ as additive. (c) C–H Amination of hydrocinnamyl alcohol without C_6_F_5_I(OTFA)_2_ additive. ^*a*^Antipode of **2a** obtained due to **Cat10** having the opposite aminoalcohol configuration compared with the others.

We evaluated the Rh complexes bearing these cations in C–H amination of hydrocinnamyl alcohol, evaluating both pyridine-ligated and nonligated Rh complexes for each to examine if there was any divergence (Figure 4). In the first instance, the trifluoroacetic acid-releasing additive C_6_F_5_I(OTFA)_2_ was included (Figure 4b). We began with **Cat6**, the most simple cation scaffold in our study, in which the three intrinsic alkaloid features are “knocked out”: the basic quinoline nitrogen, the methoxy group at the quinoline 6-position and the quinuclidine-located ethyl. This complex gave a very poor outcome, delivering only 7% *ee* for the pyridine-ligated catalyst and 30% for the nonligated. Interestingly for the pyridine-ligated catalyst, the yield was also unusually low, suggesting that the pyridine may remain strongly bound in the absence of competitive binding from the cation (see the SI for yield information). We next introduced the methoxy group to the naphthalene in **Cat7** and saw a small improvement with the ligated complex but overall was still poor. We next introduced the basic nitrogen to form a quinoline, but now, a 5-substituted quinoline than the 4-isomer of the natural alkaloids (**Cat8**). This gave a very large jump (91% *ee* with ligated complex and 82% with nonligated) and was in fact higher than the natural quinoline isomer **Cat9**, which delivered 84 and 48% *ee* for ligated and nonligated, respectively.^28^ Returning the methoxy group to the 6-position of the quinoline (**Cat10**) and adding the ethyl group to the quinuclidine (**Cat1**) gave minor increases to reach 89% *ee* (ligated) and 76% (nonligated). Having established that the quinoline nitrogen was crucial for high *ee*, seemingly irrespective of position, we next evaluated the 2-*n*-butyl-substituted cation **Cat11**. We anticipated that binding to Rh would be severely impaired (as evidenced by the lack of color change of the solution upon cation ion exchange) but that the quinoline should retain its basicity, potentially allowing a distinction between a binding effect and a basicity effect. Very interestingly, the *ee* was only slightly reduced in direct comparison with **Cat1**, at 84% (pyridine-ligated), which could be attributed to the quinoline nitrogen acting beneficially as a base. Finally, we evaluated cation **Cat12** in which the hydroxyl is methylated and were surprised to see that this favored formation of the opposite product enantiomer with moderate *ee* (−44% and −24%, ligated and nonligated). Such a dramatic shift hints at a very important role for the cation hydroxyl group in the presumed network of noncovalent interactions at the transition state.^29^**Cat13** differs from **Cat1** by being epimeric at the hydroxyl group and this also gave the opposite enantiomer in −77 and −61% *ee* for ligated and nonligated, respectively. Although relatively high, these *ee* values are still 10–15% lower than those with the natural diastereomeric configuration possessed by the alkaloids.

We also performed an analogous analysis for aziridination, using a bishomoallylic alcohol that had been optimized extensively in our previous study.^19b^ Very similar trends were observed across the cations, confirming for this reaction too the clear need for the quinoline nitrogen, irrespective of the specific isomer (**Cat8** or **Cat9**) used (see the SI for details).

**Scheme 6:**
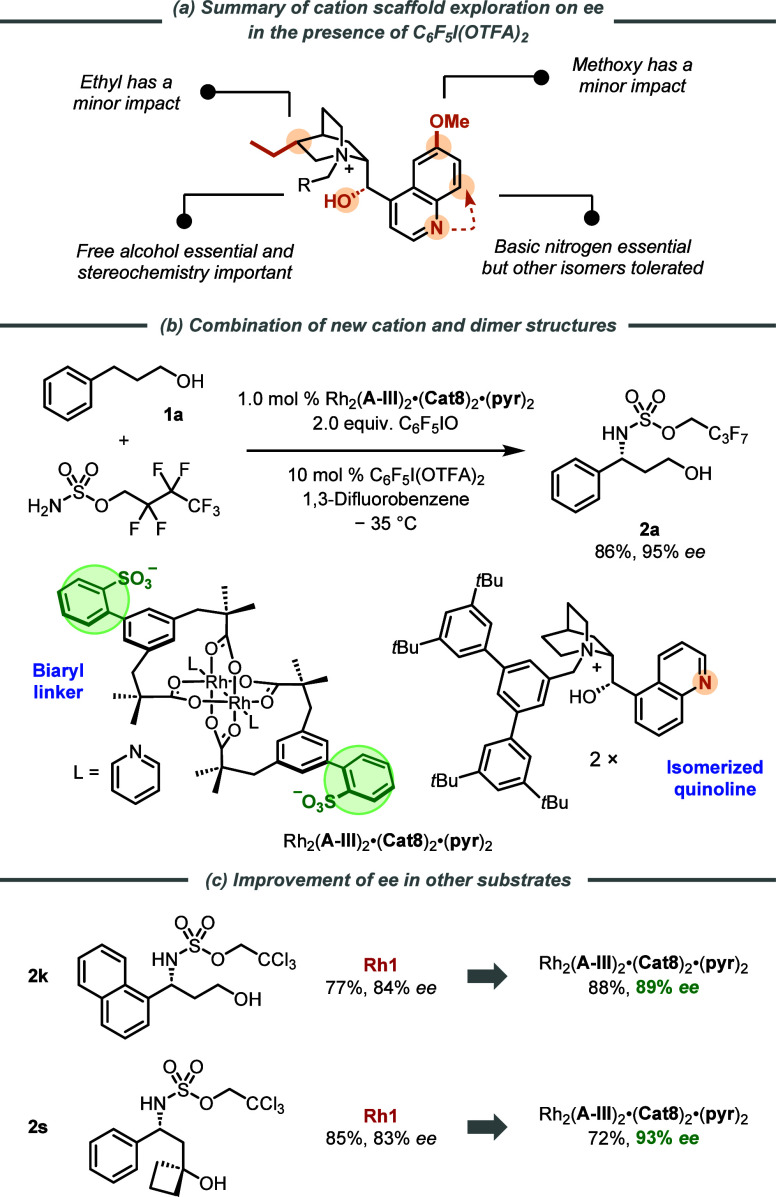
Summary of Cation Exploration Findings and Amination Using a Catalyst Containing **Cat8** and Biaryl Linker

The outcome of this investigation demonstrated that the quinoline nitrogen possessed by the cation is essential for obtaining high *ee* in this chemistry. Furthermore, the precise location of this basic nitrogen seems unimportant as complexes containing **Cat8** and **Cat9** provided very similar enantioselectivities in direct comparisons, with the non-natural isomer **Cat8** even providing slightly better results (*vide infra*). The free hydroxyl with the correct stereochemistry was similarly crucial. The remaining features of the natural cinchona scaffold (methoxy on the quinoline ring, ethyl on the quinuclidine) provided small degrees of tuning, which varies from one substrate to another, the effect being nowhere near as dramatic. The tolerance of the location of the quinoline nitrogen combined with the fact that *ee* is largely retained when an alkyl group is introduced adjacent to it led us to suppose that the quinoline nitrogen effect may be acting as a weak base, which is needed to increase *ee*. In line with this, we have established that the acid-releasing additive C_6_F_5_I(OTFA)_2_ in substoichiometric amounts boosts *ee* in this system and others.^19c^ For this reason, we returned to the original C–H amination reaction and evaluated all of the complexes in the absence of C_6_F_5_I(OTFA)_2_ (Figure 4c). Informative trends here are more challenging to elucidate, but it is clear that inclusion of the quinoline nitrogen has a less pronounced effect on *ee* under these conditions. The quinoline effect is substantial in direct comparison between **Cat6** and **Cat9**, albeit only for the pyridine-ligated complex. For the analogous comparison between **Cat7** and **Cat10**, there was little impact on *ee* with nitrogen inclusion and this held true for both ligated and nonligated complexes. What we had previously deemed “ancillary” features of the cation in the presence of C_6_F_5_I(OTFA)_2_ now seem to have a larger effect in its absence. For instance, the inclusion of the quinoline methoxy reduces *ee* by around 20% (**Cat9** to **Cat10**), but the inclusion of the quinuclidine ethyl group increased it by a remarkable 30% (**Cat10** to **Cat1**). Although high *ee* values can be obtained in the absence of the C_6_F_5_I(OTFA)_2_ additive, tolerance of variations in the cation structure is greater in its presence, allowing high *ee* values to be obtained more generally. This leads to a more general, highly enantioselective reaction in its presence, and we believe that protonation of the quinoline nitrogen seems likely to play a role, potentially impacting the conformation of the cation.^30^ Our studies have shown that introducing the Rh-complex with axially ligating pyridines has a consistently beneficial effect (as seen during optimization, Figure 2 graph inset, and reinforced here) of typically 10–20% *ee* in most cases.^31^ A summary of the key findings in this cation exploration is given in Scheme 6a.

Finally, we were intrigued by the observation that the complex containing **Cat8**, in which the nitrogen of the quinoline has been transposed to the non-natural position, provided an edge in *ee* terms compared with **Cat9**, its direct comparator containing the quinoline isomer produced by nature. In the amination of **2a** it gave 91% *ee* when combined with scaffold **I,** which possessed the original benzyl sulfonate linker. Given the increase we had seen in the present work with the improved biaryl scaffold **III**, we wondered whether combining novel cation **Cat8** with the new linker might raise enantioselectivity even further. Upon testing this, we were pleased to find that **2a** could be obtained in 95% *ee*, the highest we have obtained for this compound so far throughout our studies, demonstrating that the improvements to scaffold and cation identified during this study are additive (Scheme 6b). We then selected two substrates from our main scope, **2k** and **2s**, that gave slightly lower than optimal *ee* using **Rh1** and re-examined them with the complex incorporating **Cat8**. We were very happy to find that, in both cases, the *ee* values were increased, from 84 to 89% and 83 to 93% (Scheme 6c). While the synthesis of **Cat8** is significantly longer compared with the alkaloid-derived **Cat4** that is possessed by **Rh1**, we hope that this demonstration will be of value in certain circumstances where **Rh1** may provide insufficient selectivity.

## 3 Conclusions

In this study, we have developed an effective catalyst system for enantioselective nitrene transfer to hydrocinnamyl alcohols (C–H amination) and allylic alcohols (aziridination). This expansion of highly enantioselective C–H amination and aziridination allows access to small, densely functionalized chiral building blocks that we anticipate will be of great utility in synthesis. These reactions are enabled by the directing effect of the substrate alcohol combined with highly effective ion-paired Rh complexes that we have developed that incorporate cinchona alkaloid-derived cations. In our previous work, these shorter chain length substrates gave inadequate enantioselectivity and we have developed a superior catalyst scaffold in which the sulfonate group is separated from the catalyst backbone by an *ortho*-biaryl linker. Both reactions lead to valuable chiral building blocks with excellent enantioselectivities which are amenable to further functionalization for a range of possible applications, for example, in the synthesis of highly substituted azetidines. A key component of the ion-paired catalysts, which had not been previously explored, beyond variation of the *N*-quaternizing group, was the cinchona alkaloid-derived chiral cation; we had until this point retained many natural features of the alkaloid without rigorous analysis. We carried out a “knockout” and systematic reinclusion study, which included investigating the quinoline nitrogen of the alkaloid through *de novo* asymmetric synthesis. While features such as the 6-methoxy on the quinoline and the ethyl of the quinuclidine had a minor impact, the free hydroxyl group, with the stereochemistry possessed by the natural alkaloid, was found to be profoundly important. Just as important was the presence of the quinoline nitrogen although its precise position was unimportant and steric obstruction of it was inconsequential. While these observations are difficult to reconcile into a unified stereochemical model, it is clear that the complex environment provided by the quaternized alkaloid provides rich opportunities for productive interactions with the substrate during enantiodetermining nitrene transfer and under the optimal conditions the basic nitrogen of the cation plays an important role. It is somewhat remarkable that the chemical structure provided by nature turns out to have several key features required for a purely synthetic reaction. On the other hand, the cinchona alkaloids have surprised practitioners of asymmetric catalysis time and time again with the diversity of situations in which they have been highly effective. We hope these insights will be of use in the further application of these complexes to asymmetric nitrene transfer and beyond, for example in carbene transfer methodology.

## Supporting Information Available

Additional optimization, full experimental details, and characterization data for compounds (PDF)

## References

[ref1] aDequirez G.; Pons V.; Dauban P. Nitrene Chemistry in Organic Synthesis: Still in Its Infancy?. Angew. Chem., Int. Ed. 2012, 51, 7384–7395. 10.1002/anie.201201945. 22730346

[ref2] aMüller P.; Fruit C. Enantioselective Catalytic Aziridinations and Asymmetric Nitrene Insertions into CH Bonds. Chem. Rev. 2003, 103, 2905–2920. 10.1021/cr020043t. 12914485

[ref3] aEspino C. G.; Fiori K. W.; Kim M.; Du Bois J. Expanding the Scope of C–H Amination through Catalyst Design. J. Am. Chem. Soc. 2004, 126, 15378–15379. 10.1021/ja0446294. 15563154

[ref4] Hansen J.; Davies H. M. L. High symmetry dirhodium(II) paddlewheel complexes as chiral catalysts. Coord. Chem. Rev. 2008, 252, 545–555. 10.1016/j.ccr.2007.08.019. 19255604 PMC2390838

[ref5] Yamawaki M.; Tsutsui H.; Kitagaki S.; Anada M.; Hashimoto S. Dirhodium(II) tetrakis[N-tetrachlorophthaloyl-(S)-tert-leucinate]: a new chiral Rh(II) catalyst for enantioselective amidation of C-H bonds. Tetrahedron Lett. 2002, 43, 9561–9564. 10.1016/S0040-4039(02)02432-2.

[ref6] aNägeli I.; Baud C.; Bernardinelli G.; Jacquier Y.; Moraon M.; Müller P. Rhodium(II)-Catalyzed CH Insertions with ^6a^phenyl-λ3-iodane. Helv. Chim. Act. 1997, 80, 1087–1105.

[ref7] Reddy R. P.; Davies H. M. L. Dirhodium Tetracarboxylates Derived from Adamantylglycine as Chiral Catalysts for Enantioselective C–H Aminations. Org. Lett. 2006, 8, 5013–5016. 10.1021/ol061742l. 17048831

[ref8] Zalatan D. N.; Du Bois J. A Chiral Rhodium Carboxamidate Catalyst for Enantioselective C–H Amination. J. Am. Chem. Soc. 2008, 130, 9220–9221. 10.1021/ja8031955. 18582043 PMC2597189

[ref9] aNasrallah A.; Boquet V.; Hecker A.; Retailleau P.; Darses B.; Dauban P. Catalytic Enantioselective Intermolecular Benzylic C(sp3)–H Amination. Angew. Chem., Int. Ed. 2019, 58, 8192–8196. 10.1002/anie.201902882. 30968491

[ref10] van den Heuvel N.; Mason S. M.; Mercado B. Q.; Miller S. J. Aspartyl β-Turn-Based Dirhodium(II) Metallopeptides for Benzylic C(sp3)–H Amination: Enantioselectivity and X-ray Structural Analysis. J. Am. Chem. Soc. 2023, 145, 12377–12385. 10.1021/jacs.3c03587. 37216431 PMC10330621

[ref11] aSambasivan R.; Ball Z. T. Metallopeptides for Asymmetric Dirhodium Catalysis. J. Am. Chem. Soc. 2010, 132, 9289–9291. 10.1021/ja103747h. 20518468

[ref12] aZhou X.-G.; Yu X.-Q.; Huang J.-S.; Che C.-M. Asymmetric amidation of saturated C–H bonds catalysed by chiral ruthenium and manganese porphyrins. Chem. Commun. 1999, 2377–2378. 10.1039/a907653k.

[ref13] Boquet V.; Nasrallah A.; Dana A. L.; Brunard E.; Di Chenna P. H.; Duran F. J.; Retailleau P.; Darses B.; Sircoglou M.; Dauban P. Rhodium(II)-Catalyzed Enantioselective Intermolecular Aziridination of Alkenes. J. Am. Chem. Soc. 2022, 144, 17156–17164. 10.1021/jacs.2c07337. 36094904

[ref14] aEvans D. A.; Woerpel K. A.; Hinman M. M.; Faul M. M. Bis(oxazolines) as chiral ligands in metal-catalyzed asymmetric reactions. Catalytic, asymmetric cyclopropanation of olefins. J. Am. Chem. Soc. 1991, 113, 726–728. 10.1021/ja00002a080.

[ref15] aFukagawa S.; Kojima M.; Yoshino T.; Matsunaga S. Catalytic Enantioselective Methylene C(sp3)–H Amidation of 8-Alkylquinolines Using a Cp*RhIII/Chiral Carboxylic Acid System. Angew. Chem., Int. Ed. 2019, 58, 18154–18158. 10.1002/anie.201911268. 31593365

[ref16] aWang J.; Luo M.-P.; Gu Y.-J.; Liu Y.-Y.; Yin Q.; Wang S.-G. Chiral CpxRhodium(III)-Catalyzed Enantioselective Aziridination of Unactivated Terminal Alkenes. Angew. Chem., Int. Ed. 2024, 63, e202400502 10.1002/anie.202400502. 38279683

[ref17] aHoveyda A. H.; Evans D. A.; Fu G. C. Substrate-directable chemical reactions. Chem. Rev. 1993, 93, 1307–1370. 10.1021/cr00020a002.

[ref18] aHöke T.; Herdtweck E.; Bach T. Hydrogen-bond mediated regio- and enantioselectivity in a C–H amination reaction catalysed by a supramolecular Rh(ii) complex. Chem. Commun. 2013, 49, 8009–8011. 10.1039/c3cc44197k. 23917404

[ref19] aFanourakis A.; Williams B. D.; Paterson K. J.; Phipps R. J. Enantioselective Intermolecular C–H Amination Directed by a Chiral Cation. J. Am. Chem. Soc. 2021, 143, 10070–10076. 10.1021/jacs.1c05206. 34181401 PMC8283762

[ref20] aGenov G. R.; Douthwaite J. L.; Lahdenperä A. S. K.; Gibson D. C.; Phipps R. J. Enantioselective remote C–H activation directed by a chiral cation. Science 2020, 367, 1246–1251. 10.1126/science.aba1120. 32165586

[ref21] aOhmatsu K.; Ito M.; Kunieda T.; Ooi T. Ion-paired chiral ligands for asymmetric palladium catalysis. Nat. Chem. 2012, 4, 473–477. 10.1038/nchem.1311. 22614382

[ref22] Guthikonda K.; Du Bois J. A Unique and Highly Efficient Method for Catalytic Olefin Aziridination. J. Am. Chem. Soc. 2002, 124, 13672–13673. 10.1021/ja028253a. 12431086

[ref23] Additionally, significant amounts of over-amination were found in the reactions with Rh_2_(esp)_2_ which complicated analysis of site-selectivity.

[ref24] Kolb H. C.; VanNieuwenhze M. S.; Sharpless K. B. Catalytic Asymmetric Dihydroxylation. Chem. Rev. 1994, 94, 2483–2547. 10.1021/cr00032a009.

[ref25] aLygo B.; Crosby J.; Lowdon T. R.; Wainwright P. G. Enantio- and diastereoselective synthesis of all four possible stereoisomers of 2-(phenylhydroxymethyl)quinuclidine. Tetrahedron Lett. 1997, 38, 2343–2346. 10.1016/S0040-4039(97)00310-9.

[ref26] Furukawa K.; Katsukawa M.; Nuruzzaman M.; Kobayashi Y. Synthesis of a Series of Structural Analogues of the Cinchona Alkaloids. Heterocycles 2007, 74, 159–166. 10.3987/COM-07-S(W)28.

[ref27] aRaheem I. T.; Goodman S. N.; Jacobsen E. N. Catalytic Asymmetric Total Syntheses of Quinine and Quinidine. J. Am. Chem. Soc. 2004, 126, 706–707. 10.1021/ja039550y. 14733531

[ref28] The ee value obtained for the nonligated complex seemed abnormally low but repitition of the reaction twice yielded identical outcomes therefore we conclude that this is a genuine effect.

[ref29] In our studies using similar chiral cations in enantioselective borylation we had observed decreased ee using the methylated cation, but the decrease was much smaller than that seen here (ref (20a)).

[ref30] We recently found that enantioselective C-H amination using amide directing groups could be perfomed with high ee, for which the presence of the C_6_F_5_I(OTFA)_2_ additive was crucial (ref (19c)). Control experiments suggested that the presence of the additive allowed hydrogen bond acceptor groups to be effective directors, including a methyl ether. We have evaluated amination of the corresponding methyl ether of **2a** and found that very low reactivity (<5% yield) was obtained under the optimized conditions using the new biaryl catalyst **Rh1** suggesting that this is not an effective directing group in this case (see the SI for details).

[ref31] Sharland J. C.; Wei B.; Hardee D. J.; Hodges T. R.; Gong W.; Voight E. A.; Davies H. M. L. Asymmetric synthesis of pharmaceutically relevant 1-aryl-2-heteroaryl- and 1,2-diheteroarylcyclopropane-1-carboxylates. Chem. Sci. 2021, 12, 11181–11190. 10.1039/D1SC02474D. 34522315 PMC8386643

