# Supplementary material for: Enantioselective Nitrene Transfer to Hydrocinnamyl Alcohols and Allylic Alcohols Enabled by Systematic Exploration of the Structure of Ion-Paired Rhodium Catalysts
Source: J Am Chem Soc. 2024 Jul 31;146(32):22629–41. doi: 10.1021/jacs.4c07117 (PMC11328136; doi:10.1021/jacs.4c07117)

Enantioselective Nitrene Transfer to Hydrocinnamyl Alcohols and Allylic Alcohols Enabled by Systematic Exploration of the Structure of Ion-Paired Rhodium Catalysts

Nicholas J. Hodson, Shotaro Takano, Alexander Fanourakis and Robert J. Phipps*

Yusuf Hamied Department of Chemistry, University of Cambridge, Lensfield Road,

Cambridge, CB2 1EW, United Kingdom.

Corresponding Author

*rjp71@cam.ac.uk

Table of Contents

General Information S3

General Protocols S5

Additional Optimisation Data S12

Evaluation of Diverse Cation Structures in C-H Amination and Aziridination Reactions S16

Synthesis of Chiral Cation Bromide Salts S20

Synthesis of Achiral Rh(II,II) Tetracarboxylate Dimers S49

Synthesis of Chiral Rh(II,II) Tetracarboxylate Dimers S77

Synthesis of Hydrocinnamyl Alcohol Substrates S99

Synthesis of Other Chain Length Substrates for C-H Amination S114

Synthesis of Control Substrate S1 .... S116

Synthesis of Allylic Alcohol Substrates S117

Synthesis of Aminating Agents and Oxidants S125

Enantioselective Intermolecular C-H Amination Products S126

Enantioselective Intermolecular Aziridination Products S158

Determination of the Absolute Stereochemistry of Allylic Alcohol-Derived Aziridines S182

References S185

Chiral SFC, HPLC and GC Traces S188

NMR Spectra S246

General Information

Reaction Setup, Solvents and Reagents: Excluding the enantioselective intermolecular C-H amination and aziridination, reactions were carried out under an inert argon or nitrogen atmosphere using standard Schlenk-septa techniques in oven-dried glassware. The enantioselective intermolecular C-H amination and aziridination reactions were conducted under an atmosphere of air with minimal precaution taken against moisture. Reactions performed in 4.0 mL crimp-top vials that required cooling were placed either in a Polar Bear Cub (by Cambridge Reactor Design) featuring a deep-welled heating block for – 35 °C, or in a deep metal dewar with a shallow-welled heated block surrounded by dry ice for – 78 °C. All reagents were used as supplied from commercial sources without further purification unless otherwise stated. NaOH was finely ground using a pestle and mortar. THF, Et_2O, MeOH, MeCN, CH_2Cl_2, n-hexane and toluene were purified by distillation on site under inert atmosphere via the following processes: THF and Et_2O were pre-dried over sodium wire then distilled from calcium hydride and lithium aluminium hydride. MeOH, MeCN, CH_2Cl_2, n-hexane and toluene were distilled from calcium hydride. 1,3-Difluorobenzene was purchased from Fluorochem or Apollo Scientific and was used as received. In the text below, "Petrol" refers to petroleum ether 40–60 °C.

Chiral HPLC Analysis: Performed on a Waters ARC system with YMC CHIRAL ART SA, SC or SJ columns (4.6 x 250 mm, 3.0 µm) in a mixed solvent system of hexane and *i*PrOH.

Chiral SFC Analysis: Performed on a Waters ACQUITY UPC2 System with YMC CHIRAL ART SA, SB, SC, or SJ columns (4.6 x 250 mm, 3.0 µm), or DAICEL CHIRALPAK IA, IJ, IK, IG, IH or IE columns (4.6 x 250 mm, 3.0 µm) in a mixed solvent system of supercritical CO_2 and MeOH or *i*PrOH. A system backpressure of 138 bar was used in all cases.

Chiral GC Analysis: GC analyses were obtained on a Shimadzu GC-2010 Plus instrument equipped with a Cyclosil-B column (30 m x 0.25 mm ID x 0.25 µm film) and an FID detector.

Chromatography: Analytical thin-layer chromatography was performed using precoated Merck glass backed silica gel plates (Silica gel 60 F254). Visualisation was by ultraviolet fluorescence (λ = 254 and 365 nm) and/or staining with potassium permanganate ($KMnO_4$) or Ceric Ammonium Molybdate (CAM). Flash column chromatography was performed using silica gel 60 (pore size: 60 Å, mesh: 40-63 µm) from Material Harvest® or Fluorochem.

High Resolution Mass Spectrometry (HRMS): Recorded on a Waters Micromass LCT Premier, a Waters Xevo G2-S QTOF, a Waters Synapt G2-Si, a Waters Vion IMS QTOF and AGILENT 6230 LC/TOF at the Department of Chemistry at the University of Cambridge. The ionisation method is noted as either positive or negative electrospray ionisation (+/–ESI). Measured values are reported to 4 decimal places and are within ± 5 ppm of the calculated value. The calculated values are based on the most abundant isotope unless otherwise stated in the chemical formula. For ions bearing more than a single unit of charge, the masses reported as 'found' and 'required' are the mass/charge ratios.

NMR Spectroscopy: ^{1}H NMR spectra were recorded on 700 MHz TXO Cryoprobe, 600 MHz Bruker Avance DRX-600, 500 MHz Bruker DCH Cryoprobe, 400 MHz Bruker DPX-400 Dual, 400 MHz Avance III HD, 400 MHz Avance III HD Smart Probe or 400 MHz Neo Prodigy spectrometers. Chemical shifts are reported in parts per million (ppm) and the spectra are calibrated to the resonance resulting from incomplete deuteration of the solvent ($CDCl_3$: 7.26 ppm; CD_3CN: 1.94 ppm, p; $(CD_3)_2CO$: 2.05 ppm, p; D_2O: 4.79 ppm; $(CD_3)_2SO$: 2.50 ppm, p; CD_3OD: 3.31 ppm, p; C_5D_5N (H–C–N): 8.74 ppm).[1] ^{13}C NMR spectra were recorded on the same spectrometers with complete proton decoupling. ^{13}C NMR experiments of compounds containing poly-fluorination were performed using a UDEFT sequence to increase the signal:noise ratio for ^{13}C signals of carbon nuclei along the poly-fluorinated chain.[2] Chemical shifts are reported in ppm with the solvent resonance as the internal standard ($^{13}CDCl_3$: 77.16 ppm, t; $^{13}CD_3CN$: 1.32 ppm, sept; $(^{13}CD_3)_2CO$: 29.84 ppm, sept; $(^{13}CD_3)_2SO$: 39.52 ppm, sept; $^{13}CD_3OD$: 49.00 ppm, sept; $^{13}C_5D_5N$ (C–N): 150.35 ppm, t).[1] ^{19}F NMR spectra were recorded on 400 MHz Avance III HD or 400 MHz Avance III HD Smart Probe spectrometers. Chemical shifts are reported in ppm with $CFCl_3$ as the external standard ($CFCl_3$: 0.00 ppm). Data are reported as follows: chemical shift δ, multiplicity (s = singlet, d = doublet, t = triplet, q = quartet, p = pentet, sext = sextet, sept = septet, br = broad, m = multiplet or combinations thereof (^{13}C and all other nuclides except ^{1}H are singlets unless otherwise stated)), coupling constants *J*, number of nuclides (signals for all other nuclides except ^{1}H refer to one nuclide unless otherwise stated), assignment. ^{1}H NMR spectra are assigned as fully as possible, using ^{1}H-^{1}H COSY, ^{1}H-^{1}H NOESY, DEPT-135, ^{1}H-^{13}C HSQC and ^{1}H-^{13}C HMBC where appropriate to facilitate structural determination. Assignments either follow the numbering system shown on the structures or are described unambiguously. ^{1}H NMR signals are reported in ppm to 2 decimal places and all other nuclide signals are typically reported to 1 decimal place. Coupling constants are reported in Hz to a maximum of 3 significant figures. For cinchona alkaloid-derived compounds the appearance and chemical shifts of the peaks in the NMR spectra can vary significantly depending on sample concentration and other factors. For spectra acquired in C_5D_5N the residual water peak is often visible at approximately 4.9 ppm in the ^{1}H NMR spectrum.

Optical Rotations: Measured in spectrophotometric grade $CHCl_3$, MeOH or C_5H_5N or in LiChrosolv® hypergrade CH_3CN for LC-MS on a Perkin Elmer 343 Polarimeter using a sodium lamp (λ = 589 nm, D-line). $[\alpha]_D$ values are reported at the stated temperature, with concentration in g /100mL.

Naming and Numbering of compounds: Systematic names were generated by the computer program ChemDraw according to the guidelines specified by the IUPAC. However, the numbering on the structures does not correspond to the systematic name.

General Protocols

General procedure for the quaternisation of cinchona alkaloid derivatives (**GP1**):

The protocol described by Phipps and co-workers was employed.[3] A crimp-top vial was charged with the cinchona alkaloid derivative (1.0 equiv.) and the benzyl bromide (1.0 equiv.). The vial was sealed and evacuated and backfilled with nitrogen three times, following which dry THF (0.04 M) was added. The reaction mixture was then heated at 75 °C overnight following which it was allowed to cool and the THF removed under reduced pressure. The crude residue was taken up in CH_2Cl_2 (20 mL) and washed twice with H_2O (2 x 10 mL). The organic layer was dried ($MgSO_4$) and the solvent removed under reduced pressure. Purification by flash column chromatography afforded the title compounds.

General procedure for the assembly of achiral Rh(II,II) dimers (**GP2**):

A variant of the protocol described by Du Bois and co-workers was employed.[4] An oven-dried 2-necked round-bottomed flask fitted with a reflux condenser was charged with $Rh_2(TFA)_4$ (1.0 equiv.) and ligand (1.03 equiv.) and placed under a nitrogen atmosphere. Chlorobenzene (approx. 0.02 M) was added and the mixture was evacuated until solvent started to boil and then backfilled with nitrogen. This was repeated a further four times following which the mixture was heated at reflux for 5 hours. The flask was allowed to cool slightly, and a second portion of ligand (1.03 equiv.) was added and the resulting mixture was heated at reflux for an additional 5 hours. The reaction mixture was then cooled to room temperature and concentrated under a stream of nitrogen. Purification by flash column chromatography (SiO_2, 0-100% v/v EtOH in $CHCl_3$) afforded the semi-purified achiral dimer. At this stage, ^{1}H NMR often revealed an under-integration of the tetrabutylammonium cations relative to the dimer. As a result, ^{1}H NMR with an internal standard was used to determine the extra number of mmol (τ) of tetrabutylammonium cation required to ensure a 1:2.0 ratio of dimer:tetrabutylammonium cation. Thus, the crude product was taken up in water (10 mL) and 3:1 $CHCl_3$/*i*PrOH (10 mL) following which the appropriate number of mmol of tetrabutylammonium hydrogensulfate (0.9 × τ) and NaOH (0.9 × τ) were added and the resulting biphasic mixture was shaken vigorously in a separating funnel, upon which the blue colour present in the aqueous layer was observed to move into the organic layer. The layers were separated and the aqueous layer was extracted with 3:1 $CHCl_3$/*i*PrOH (2 × 10 mL). The combined organic layers were dried over $MgSO_4$, concentrated under reduced pressure and further dried by heating at 90 °C overnight under vacuum to afford the title compounds.

Note: (0.9 × τ) is used to determine the mmol of tetrabutylammonium hydrogensulfate and NaOH added to avoid over-integration of tetrabutylammonium in the final product.

General procedure for the synthesis of chiral ion-paired Rh(II,II) dimers (**GP3**):

A sinter funnel was loosely packed with a pad of Amberlite® IRC120 H hydrogen form beads (approx. 6 cm column height) and equilibrated by flushing through with three column volumes of MeOH. Following this, a solution of the achiral rhodium dimer (1.0 equiv.) in MeOH (25 mL) was loaded onto the pad and eluted. The eluent was then recycled through the pad a further four times. Following this, the eluent was evaporated to dryness and taken up in water (10 mL). Drops of 10% aqueous NaOH solution were added until the solution was basic. The chiral cation bromide salt (1.8 equiv.) was added followed by 3:1 $CHCl_3$/*i*PrOH (20 mL). The biphasic mixture was stirred rapidly overnight during which the blue colour of the aqueous layer disappeared and a deep red colour was observed to move into the organic layer. The layers were separated and the aqueous phase extracted with 3:1 $CHCl_3$/*i*PrOH (3 × 20 mL). The combined organic layers were dried over $MgSO_4$, concentrated under reduced pressure and further dried by heating at 90 °C overnight under vacuum to afford the title compounds.

Formation of the pyridine-ligated catalyst is achieved by taking a portion of the desolvated material and dissolving it fully in pyridine to give a bright-pink solution. The excess pyridine is then removed under a stream of nitrogen and the oily residue dried thoroughly under vacuum to a free-flowing pink powder. Following this the pyridine-ligated catalyst is ready for use.

General protocol for synthesis of chiral ion-paired Rh(II,II) dimers using chiral cation bromide salts **Cat6•Br**, **Cat7•Br** and **Cat11•Br** (**GP4**):

A sinter funnel was loosely packed with a pad of Amberlite® IRC120 H hydrogen form beads (approx. 6 cm column height) and equilibrated by flushing through with three column volumes of MeOH. Following this, a solution of Bis[rhodium tetrabutylammonium (3,5-bis(2-carboxy-2-methylpropyl)phenyl)methanesulfonate)] (Rh_2(**A-I**)$_2$•$(Bu_4N)_2$) (1.0 equiv.) in MeOH (25 mL) was loaded onto the pad and eluted. The eluent was then recycled through the pad a further four times. After the eluent was evaporated to dryness, the residue was dissolved in MeOH (10 mL) and then Ag_2CO_3 (1.5 equiv.) was added to the solution. The reaction mixture was heated at 50 °C for 1 hour. After cooling to room temperature, water (7.5 mL) was added to the mixture. The resulting mixture was passed through a Celite pad to remove the excess amount of Ag_2CO_3 and then the solvent was removed under reduced pressure. The residue was dissolved in water (10 mL) and a 3:1 mixture of $CHCl_3$/*i*PrOH (20 mL) was added, followed by the corresponding chiral cation bromide salt (1.8 equiv.). The biphasic mixture was stirred rapidly overnight during which the blue colour of the aqueous phase disappeared, and the blue colour was observed to move into the organic phase. The layers were separated and the aqueous phase extracted with a 3:1 mixture of $CHCl_3$/*i*PrOH (3 × 20 mL). The organic fractions were dried ($MgSO_4$) and the solvent removed under reduced pressure. The obtained solid was dissolved in CH_2Cl_2 and passed through a Celite pad. After removing the solvent, desolvation of the catalyst was achieved by heating at 90 °C overnight under vacuum to afford the title compounds.

Formation of the pyridine-ligated catalyst is achieved by taking a portion of the desolvated material and dissolving it fully in pyridine to give a bright-pink solution. The excess pyridine is then removed under a stream of

nitrogen and the oily residue dried thoroughly under vacuum to a free-flowing pink powder. Following this the pyridine-ligated catalyst is ready for use.

General protocol for the BH_3•THF reduction of carboxylic acids (**GP5**):

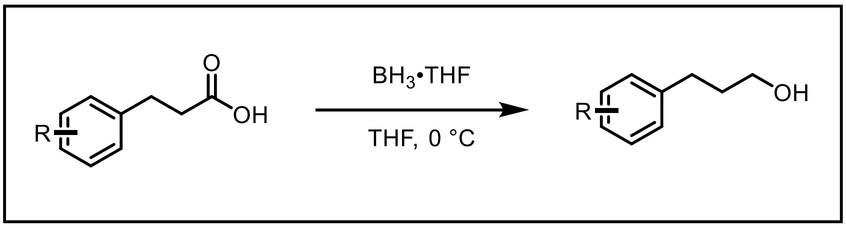

To a solution of the carboxylic acid (3.0 mmol) in THF (20 mL) cooled to 0 °C was added dropwise BH_3•THF (9.0 mL of a 1.0 M solution in THF, 9.0 mmol). The reaction mixture was stirred at 0 °C for a further 20 minutes following which it was removed from the ice bath and stirred at room temperature overnight. The solution was then cooled to 0 °C and quenched *via* the portionwise addition of ice. The volatiles were subsequently removed under reduced pressure and a small amount of brine was added to the aqueous layer following which it was extracted with Et_2O. The combined organic fractions were dried ($MgSO_4$) and the solvent removed under reduced pressure. Purification by flash column chromatography afforded the title compounds.

General protocol for the Sonogashira coupling of aryl bromides (**GP6**):

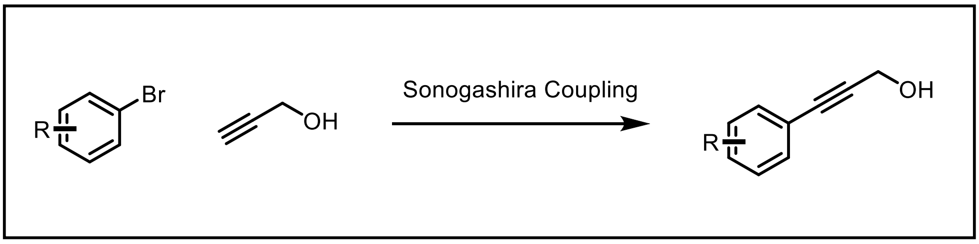

To an oven dried three-necked round-bottomed flask equipped with a reflux condenser was added $Pd(PPh_3)_2Cl_2$ (4 mol %), CuI (8 mol %) and aryl bromide (*if solid*, 1.0 equiv.). The flask was placed under a nitrogen atmosphere and THF (0.30 M), diisopropylamine (10 equiv.) and aryl bromide (*if liquid*, 1.0 equiv.) were added. After stirring at room temperature for 15 minutes, propargyl alcohol (1.5 equiv.) was added and the resulting mixture was heated at reflux overnight. After cooling to room temperature, the reaction mixture was diluted in EtOAc, filtered over Celite® and concentrated under reduced pressure. Purification of the crude residue by flash column chromatography afforded the title compounds.

General protocol for the hydrogenation of aryl alkynes (**GP7**):

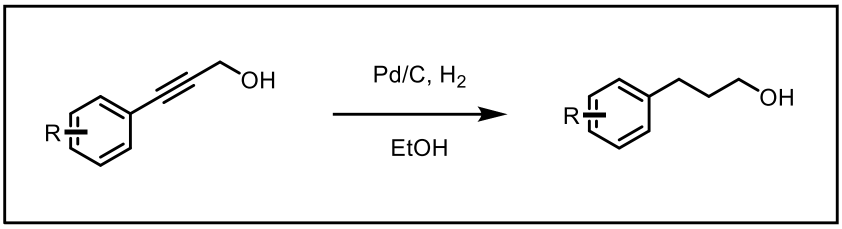

To a microwave vial charged with alkyne (1.0 equiv.) and Pd/C (10 wt%, 10 mol %) was added EtOH (0.12 M). The vial was sealed and then evacuated and backfilled with hydrogen three times. The resulting mixture was stirred at room temperature under a hydrogen balloon overnight. After completion, the mixture was filtered over Celite® eluting with EtOAc. The filtrate was collected, the solvent removed under reduced pressure and purification of the crude residue by flash column chromatography afforded the title compounds.

Note: we observed significant formation of deoxygenated products due to over reduction of the alcohol group. These side-products were typically much less polar than the desired compounds so could be easily separated out.

General protocol for Horner-Wadsworth-Emmons reactions (**GP8**):

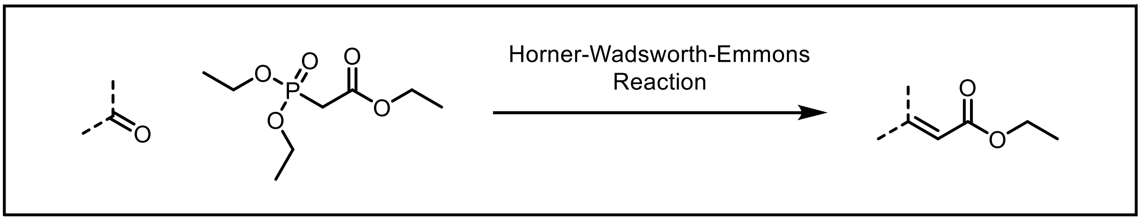

An oven dried round-bottomed flask was charged with NaH (1.1 equiv.) and placed under a nitrogen atmosphere. THF (0.75 M) was added and the resulting suspension was cooled to 0 °C. Triethyl phosphonoacetate (1.1 equiv.) was added dropwise over 10 minutes and the mixture was stirred at room temperature for 30 minutes. Ketone (1.0 equiv.) was added dropwise and the mixture was stirred at room temperature for 2 hours. In the case that the ketone was a solid at room temperature, the ketone was dissolved in THF (5 mL) and added dropwise. The reaction was quenched with saturated aqueous $NaHCO_3$ solution, extracted with EtOAc and dried over $MgSO_4$. The filtrate was collected, the solvent removed under reduced pressure and purification of the crude residue by flash column chromatography afforded the title compounds.

General protocol for DIBAL-H reductions (**GP9**):

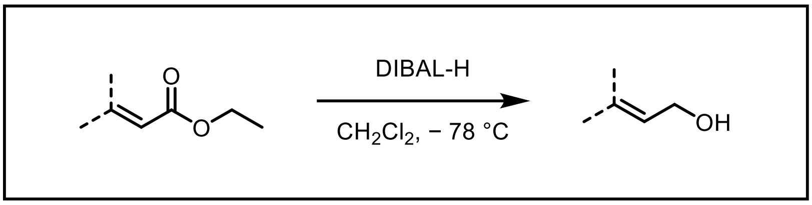

An oven dried round-bottomed flask was charged with the ester (1.0 equiv.) and placed under a nitrogen atmosphere. CH_2Cl_2 (0.25 M) was added and the resulting mixture was cooled to − 78 °C. DIBAL-H (1.0 M in hexanes, 3 equiv.) was added dropwise and the mixture was stirred overnight, with the reaction temperature slowly raised to room temperature. The reaction was cooled to – 78 °C, slowly quenched with saturated aqueous NH_4Cl solution (2 mL) and warmed to room temperature over 2 hours. Following this, it was filtered through Celite® and $MgSO_4$ eluting with CH_2Cl_2. The filtrate was collected, the solvent removed under reduced pressure and purification of the crude residue by flash column chromatography afforded the title compounds.

General protocol for intermolecular C-H amination reactions (**GP10**):

Under an atmosphere of air, a 4.0 mL crimp-top vial was charged with the substrate (0.1 mmol, 1.0 equiv.), Rh_2(**A-III**)$_2$•(**Cat4**)$_2$•(**pyr**)$_2$ (**Rh1**) (1.0 mol %) and **NH$_2$Tces** (2,2,2-trichloroethyl sulfamate) (1.2 equiv.). 1,3-difluorobenzene (0.5 mL, 0.2 M) was then added via syringe. The vial was then cooled to – 35 °C over 10 minutes. Following this, pentafluoroiodsobenzene (C_6F_5IO) (0.2 mmol, 2.0 equiv.) and (perfluorophenyl)-λ^3-iodanediyl bis(2,2,2-trifluoroacetate) ($C_6F_5I(OTFA)_2$) (10 mol %) were added together in a single portion. The vial was then capped and the reaction mixture stirred at the same temperature for 16 hours. Saturated aqueous thiourea (1.5 mL) and $CHCl_3$ (1.5 mL) were then added and the biphasic mixture was stirred vigorously for 5 minutes at – 35 °C. The mixture was then allowed to warm to room temperature and stirred for a further 15 minutes. The layers were separated and the aqueous layer extracted with $CHCl_3$ (3 x 2 mL). The combined organic layers were dried ($MgSO_4$) and the products purified by flash column chromatography.

Note on intermolecular amination reactions performed using $Rh_2(esp)_2$: In order to obtain racemic SFC/HPLC traces, racemic intermolecular amination reactions were run alongside the corresponding chiral ones. The racemic reactions were performed according to **GP10** with the following procedural modification: $Rh_2(esp)_2$ was used as the catalyst at 2 mol % loading and the 10 mol % of (perfluorophenyl)-λ^3-iodanediyl bis(2,2,2-trifluoroacetate) ($C_6F_5I(OTFA)_2$) was omitted.

General protocol for azetidine formation (**GP11**):

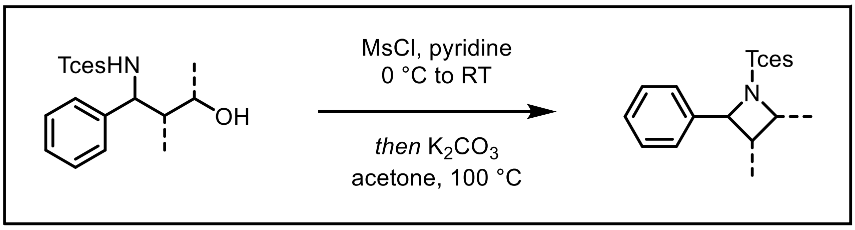

To a vial charged with amino alcohol (1.0 equiv.) under a nitrogen atmosphere was added pyridine (anhydrous, 0.025 M). The solution was cooled to 0 °C, methanesulfonyl chloride (2.0 equiv.) was added dropwise and the resulting mixture was stirred at room temperature for 3 hours. The reaction was diluted in CH_2Cl_2 and washed with aqueous 1 M HCl, saturated aqueous $NaHCO_3$, brine and dried over $MgSO_4$. The filtrate was collected and the solvent was removed under reduced pressure to reveal the crude mesylated alcohol.

The mesylate was transferred to a microwave vial and the solution concentrated under a stream of nitrogen. K_2CO_3 (3.0 equiv.) and acetone (0.025 M) were added, the vial was sealed under air and the resulting suspension was stirred at 100 °C for 2 hours. After cooling to room temperature, the solvent was removed under a stream of nitrogen and purification of the crude residue by flash column chromatography afforded the title compounds.

General protocol for intermolecular aziridination reactions (**GP12**):

Under an atmosphere of air, a 4.0 mL crimp-top vial was charged with the substrate (0.1 mmol, 1.0 equiv.), Rh_2(**A-III**)$_2$•(**Cat4**)$_2$•(**pyr**)$_2$ (**Rh1**) (2.0 mol %) and 2,2,3,3,3-pentafluoropropyl sulfamate (1.5 equiv.). CH_2Cl_2 (0.5 mL, 0.2 M) was then added via syringe. The vial was then cooled to – 78 °C over 10 minutes. This was achieved through placing the vial holder in a large dewar and surrounding the holder with dry ice. Following this, pentafluoroiodsobenzene (C_6F_5IO) (0.2 mmol, 2.0 equiv.) and (perfluorophenyl)-λ^3-iodanediyl bis(2,2,2-trifluoroacetate) ($C_6F_5I(OTFA)_2$) (10 mol %) were added together in a single portion. The vial was then capped, the dewar filled with dry ice and the reaction mixture stirred at the same temperature for 24 hours. After removing from the – 78 °C environment and allowing to warm slightly over 2 minutes, a needle outlet is placed in the vial seal, saturated aqueous thiourea (1.5 mL) and $CHCl_3$ (1.5 mL) were then added and the biphasic mixture was stirred vigorously for 10 minutes at room temperature. The needle and quench solutions should be added slowly to prevent uncontrolled effervescence of the reaction mixture. The layers were separated and the aqueous layer extracted with $CHCl_3$ (3 x 2 mL). The combined organic layers were dried ($MgSO_4$) and the products purified by flash column chromatography.

Note on intermolecular amination reactions performed using $Rh_2(esp)_2$: In order to obtain racemic SFC/HPLC traces, racemic intermolecular amination reactions were run alongside the corresponding chiral ones. The racemic reactions were performed according to **GP12** with the following procedural modification: $Rh_2(esp)_2$ was used as the catalyst at 2 mol % loading and the 10 mol % of (perfluorophenyl)-λ^3-iodanediyl bis(2,2,2-trifluoroacetate) ($C_6F_5I(OTFA)_2$) was omitted.

General procedure for thiophenol-opening of aziridine products (**GP13**):

To a vial charged with the aziridine (1.0 equiv.) under a nitrogen atmosphere was added MeOH (2.0 mL), thiophenol (4 drops) and triethylamine (8 drops). The resulting mixture was stirred at room temperature for 16 hours and concentrated under a stream of nitrogen. Purification of the crude residue by flash column chromatography afforded the title compounds.

Note: thiophenol-opening of aziridines often afforded a mixture of regioisomeric products. In most cases these were only partially separable by column chromatography. Where appropriate, the mixture of thiophenol adducts is noted. In all cases, separation of all enantiomeric peaks was achieved via chiral HPLC or SFC analysis.

Additional Optimisation Data

Supplementary Table 1: Summary of yields and *ee* values for **2a** using various catalysts (to accompany Figure 2.)

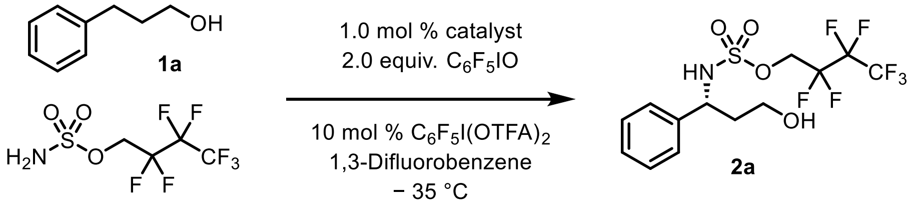

Entry	Catalyst	Yield of 2a / %[a]	*ee* of 2a / %[b]
1	Rh_2(**A-I**)$_2$●(**Cat1**)$_2$●(**pyr**)$_2$	86	88
2	Rh_2(**A-I**)$_2$●(**Cat2**)$_2$●(**pyr**)$_2$	81	85[c]
3	Rh_2(**A-II**)$_2$●(**Cat1**)$_2$●(**pyr**)$_2$	31	71
4	Rh_2(**A-II**)$_2$●(**Cat2**)$_2$●(**pyr**)$_2$	50	75[c]
5	Rh_2(**A-III**)$_2$●(**Cat1**)$_2$●(**pyr**)$_2$	83	92
6	Rh_2(**A-III**)$_2$●(**Cat2**)$_2$●(**pyr**)$_2$	72	92[c]
7	Rh_2(**A-IV**)$_2$●(**Cat1**)$_2$●(**pyr**)$_2$	78	79
8	Rh_2(**A-IV**)$_2$●(**Cat2**)$_2$●(**pyr**)$_2$	59	82[c]
9	Rh_2(**A-V**)$_2$●(**Cat1**)$_2$●(**pyr**)$_2$	12	62
10	Rh_2(**A-V**)$_2$●(**Cat2**)$_2$●(**pyr**)$_2$	21	68[c]
11	Rh_2(**B-I**)$_2$●(**Cat1**)$_2$●(**pyr**)$_2$	91	82
12	Rh_2(**C-I**)$_2$●(**Cat1**)$_2$●(**pyr**)$_2$	73	89
13	Rh_2(**D-I**)$_2$●(**Cat1**)$_2$●(**pyr**)$_2$	52	87
14	Rh_2(**B-III**)$_2$●(**Cat1**)$_2$●(**pyr**)$_2$	64	89
15	Rh_2(**C-III**)$_2$●(**Cat1**)$_2$●(**pyr**)$_2$	70	93
16	Rh_2(**D-III**)$_2$●(**Cat1**)$_2$●(**pyr**)$_2$	57	91

[a]Reactions performed on a 0.1 mmol scale with respect to **1a** and using 1.2 equivalents of sulfamate ester. Yields refer to isolated yields of **2a**. [b]*ee* determined by chiral SFC analysis of purified **2a**. [c]The antipode **2a-ent** was obtained in these cases, but the magnitude of *ee* is shown for clarity.

Supplementary Table 2: Summary of yields and *ee* values for **2a** using various reaction conditions (to accompany Figure 2, insert)

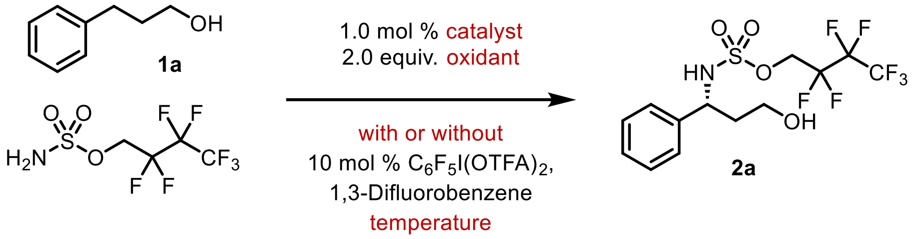

Entry	Catalyst	Oxidant	Additive	Temperature / ° C	Yield of 2a / %[a]	*ee* of 2a / %[b]
1	Rh_2(**C-I**)$_2$●(**Cat1**)$_2$	C_6H_5IO	×	− 25	56	74
2	Rh_2(**C-I**)$_2$●(**Cat1**)$_2$	C_6F_5IO	×	− 35	88	75
3	Rh_2(**C-I**)$_2$●(**Cat1**)$_2$	C_6F_5IO	✓	− 35	54	74
4	Rh_2(**C-I**)$_2$●(**Cat1**)$_2$●(**pyr**)$_2$	C_6F_5IO	×	− 35	77	87
5	Rh_2(**C-I**)$_2$●(**Cat1**)$_2$●(**pyr**)$_2$	C_6F_5IO	✓	− 35	73	89

[a]Reactions performed on a 0.1 mmol scale with respect to **1a** and using 1.2 equivalents of sulfamate ester. Yields refer to isolated yields of **2a**. [b]*ee* determined by chiral SFC analysis of purified **2a**.

Supplementary Scheme 1: Unsuccessful C-H amination of methyl ether **S1**

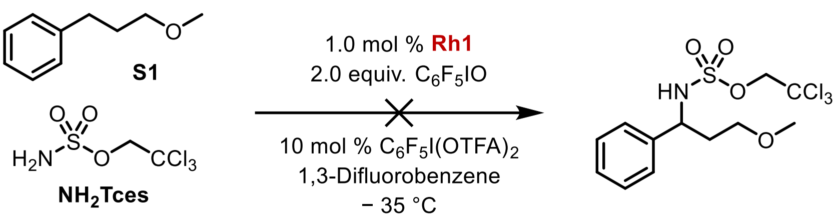

Using the optimal catalyst for the C-H amination of hydrocinnamyl alcohols (**Rh1**), the C-H amination of methylated substrate **S1** was unsuccessful, affording <5% NMR yield of the desired product (tentatively assigned based on similar ^{1}H NMR shifts to **2b**). The remaining mass balance (90%) was attributed to unreacted substrate.

Supplementary Table 3: Summary of yields and *ee* values for **9a** using various catalysts (to accompany Scheme 4)

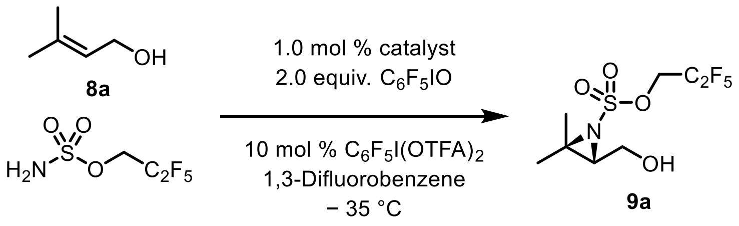

Entry	Catalyst	Yield of 9a / %[a]	*ee* / %[b]
1	Rh_2(**A-I**)$_2$●(**Cat1**)$_2$●(**pyr**)$_2$	67	66
2	Rh_2(**A-III**)$_2$●(**Cat1**)$_2$●(**pyr**)$_2$	78	76
3	Rh_2(**A-IV**)$_2$●(**Cat1**)$_2$●(**pyr**)$_2$	37	47
4	Rh_2(**B-I**)$_2$●(**Cat1**)$_2$●(**pyr**)$_2$	64	55
5	Rh_2(**C-I**)$_2$●(**Cat1**)$_2$●(**pyr**)$_2$	72	62
6	Rh_2(**D-I**)$_2$●(**Cat1**)$_2$●(**pyr**)$_2$	66	56
7	Rh_2(**B-III**)$_2$●(**Cat1**)$_2$●(**pyr**)$_2$	68	74

8	Rh_2(**C**-**III**)$_2$●(**Cat1**)$_2$●(**pyr**)$_2$	64	70
9	Rh_2(**D**-**III**)$_2$●(**Cat1**)$_2$●(**pyr**)$_2$	61	69

[a]Reactions performed on a 0.1 mmol scale with respect to **8a** and using 1.2 equivalents of sulfamate ester. Yields determined by ^{1}H NMR with reference to a 1,3,5-trimethoxybenzene internal standard. [b]*ee* determined by chiral SFC analysis of the purified thiophenol adduct of **9a**.

Supplementary Table 4: Aziridination of **8a** using **NH_2Tces** as sulfamate ester

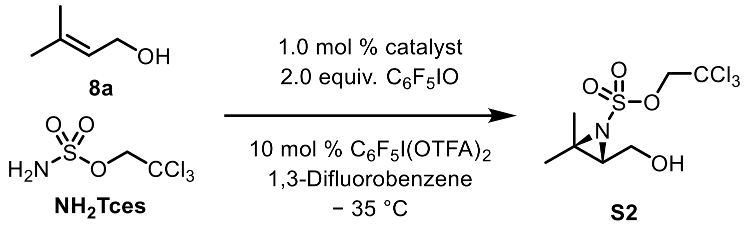

Entry	Catalyst	Yield of S2 / %[a]	*ee* / %[b]
1	Rh_2(**A**-**I**)$_2$●(**Cat1**)$_2$●(**pyr**)$_2$	67	51
2	Rh_2(**A**-**III**)$_2$●(**Cat1**)$_2$●(**pyr**)$_2$	69	62
3	Rh_2(**B**-**III**)$_2$●(**Cat1**)$_2$●(**pyr**)$_2$	64	59
4	Rh_2(**C**-**III**)$_2$●(**Cat1**)$_2$●(**pyr**)$_2$	69	59
5	Rh_2(**D**-**III**)$_2$●(**Cat1**)$_2$●(**pyr**)$_2$	62	57

[a]Reactions performed on a 0.1 mmol scale with respect to **8a** and using 1.2 equivalents of **NH_2Tces**. Yields determined by ^{1}H NMR with reference to a 1,3,5-trimethoxybenzene internal standard. [b]*ee* determined by chiral SFC analysis of the purified thiophenol adduct of **S2**.

Use of **NH_2Tces** as sulfamate ester affords aziridine **S2** in 62% *ee* using Rh_2(**A**-**III**)$_2$●(**Cat1**)$_2$●(**pyr**)$_2$ as catalyst. When the pentafluorinated aminating agent is used (to give aziridine **9a**), the corresponding *ee* value is 76%. Indeed, across all catalysts shown in Supplementary Table 4, the *ee* values for **S2** are approximately 10-15% lower than for **9a** (c.f. Supplementary Table 3, entries 1-2, 7-9). Hence, **NH_2Tces** was not used in further optimization on the aziridination of **8a**.

Supplementary Table 5: Aziridination of *cis*-cinnamyl alcohol

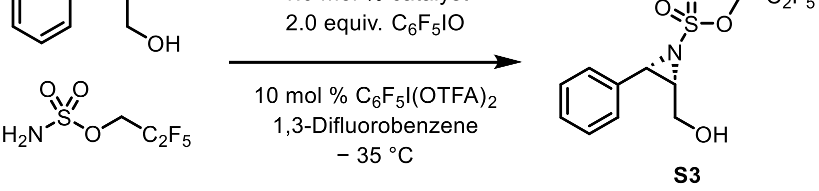

Entry	Catalyst	Yield of S2 / %[a]	*ee* of S2 / %[b]
1	Rh_2(**A**-**I**)$_2$●(**Cat1**)$_2$●(**pyr**)$_2$	53	27
2	Rh_2(**A**-**III**)$_2$●(**Cat1**)$_2$●(**pyr**)$_2$	50	33
3[c]	Rh_2(**B**-**I**)$_2$●(**Cat4**)$_2$●(**pyr**)$_2$	34	45

[a]Reactions performed on a 0.1 mmol scale with respect to *cis*-cinnamyl alcohol and using 1.2 equivalents of sulfamate ester. Yields refer to isolated yields of **S3**. [b]*ee* determined by chiral HPLC analysis of purified **S3**. [c]This result was reported in our previous work.[5] See this report for characterisation data for **S3**.

Evaluation of Diverse Cation Structures in C-H Amination and Aziridination Reactions

Supplementary Table 6: Summary of yields and *ee* values for **2a** using various catalysts (to accompany Figure 4b.)

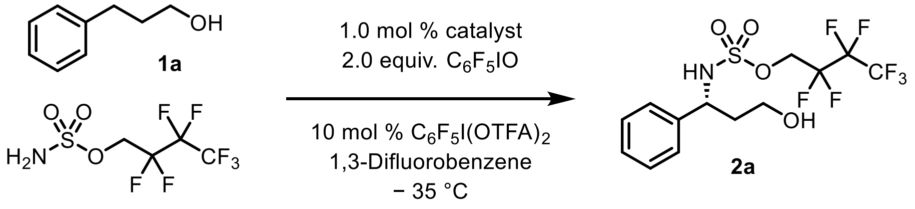

Entry	Catalyst	Yield of 2a / %[a]	*ee* of 2a / %[b]
1	Rh_2(**A-I**)$_2$●(**Cat1**)$_2$●(**pyr**)$_2$	89	89
2	Rh_2(**A-I**)$_2$●(**Cat6**)$_2$●(**pyr**)$_2$	14	7
3	Rh_2(**A-I**)$_2$●(**Cat7**)$_2$●(**pyr**)$_2$	8	24
4	Rh_2(**A-I**)$_2$●(**Cat8**)$_2$●(**pyr**)$_2$	90	91
5	Rh_2(**A-I**)$_2$●(**Cat9**)$_2$●(**pyr**)$_2$	92	84
6	Rh_2(**A-I**)$_2$●(**Cat10**)$_2$●(**pyr**)$_2$	85	85[c]
7	Rh_2(**A-I**)$_2$●(**Cat11**)$_2$●(**pyr**)$_2$	68	84
8	Rh_2(**A-I**)$_2$●(**Cat12**)$_2$●(**pyr**)$_2$	59	44[c]
9	Rh_2(**A-I**)$_2$●(**Cat13**)$_2$●(**pyr**)$_2$	40	77[c]
10	Rh_2(**A-I**)$_2$●(**Cat1**)$_2$	62	76
11	Rh_2(**A-I**)$_2$●(**Cat6**)$_2$	38	30
12	Rh_2(**A-I**)$_2$●(**Cat7**)$_2$	56	31
13	Rh_2(**A-I**)$_2$●(**Cat8**)$_2$	80	82
14	Rh_2(**A-I**)$_2$●(**Cat9**)$_2$	82	48
15	Rh_2(**A-I**)$_2$●(**Cat10**)$_2$	54	72[c]
16	Rh_2(**A-I**)$_2$●(**Cat11**)$_2$	59	60
17	Rh_2(**A-I**)$_2$●(**Cat12**)$_2$	79	24[c]
18	Rh_2(**A-I**)$_2$●(**Cat13**)$_2$	64	61[c]

[a]Reactions performed on a 0.1 mmol scale with respect to **1a** and using 1.2 equivalents of sulfamate ester. Yields refer to NMR yields of **2a** using 1,2-dimethoxyethane as an internal standard. [b]*ee* determined by chiral SFC analysis of purified **2a**. [c]The antipode **2a-ent** was obtained in these cases, but the magnitude of *ee* is shown for clarity.

Supplementary Table 7: Summary of yields and *ee* values for **2a** using various catalysts (to accompany Figure 4c.)

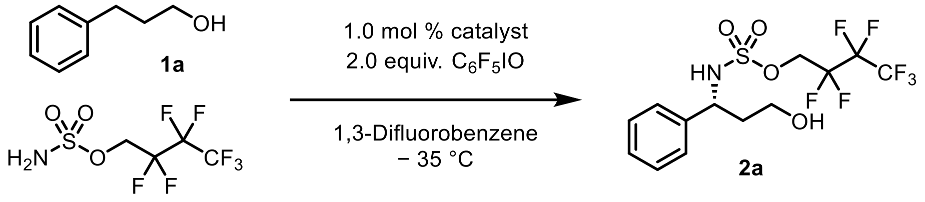

Entry	Catalyst	Yield of 2a / %[a]	*ee* of 2a / %[b]
1	Rh_2(**A-I**)$_2$●(**Cat1**)$_2$●(**pyr**)$_2$	88	87
2	Rh_2(**A-I**)$_2$●(**Cat6**)$_2$●(**pyr**)$_2$	25	21
3	Rh_2(**A-I**)$_2$●(**Cat7**)$_2$●(**pyr**)$_2$	7	48
4	Rh_2(**A-I**)$_2$●(**Cat8**)$_2$●(**pyr**)$_2$	69	82
5	Rh_2(**A-I**)$_2$●(**Cat9**)$_2$●(**pyr**)$_2$	60	74
6	Rh_2(**A-I**)$_2$●(**Cat10**)$_2$●(**pyr**)$_2$	77	57[c]
7	Rh_2(**A-I**)$_2$●(**Cat11**)$_2$●(**pyr**)$_2$	69	85
8	Rh_2(**A-I**)$_2$●(**Cat12**)$_2$●(**pyr**)$_2$	82	51[c]
9	Rh_2(**A-I**)$_2$●(**Cat13**)$_2$●(**pyr**)$_2$	82	43[c]
10	Rh_2(**A-I**)$_2$●(**Cat1**)$_2$	88	71
11	Rh_2(**A-I**)$_2$●(**Cat6**)$_2$	79	34
12	Rh_2(**A-I**)$_2$●(**Cat7**)$_2$	84	34
13	Rh_2(**A-I**)$_2$●(**Cat8**)$_2$	81	58
14	Rh_2(**A-I**)$_2$●(**Cat9**)$_2$	78	49
15	Rh_2(**A-I**)$_2$●(**Cat10**)$_2$	81	29[c]
16	Rh_2(**A-I**)$_2$●(**Cat11**)$_2$	57	61
17	Rh_2(**A-I**)$_2$●(**Cat12**)$_2$	86	24[c]
18	Rh_2(**A-I**)$_2$●(**Cat13**)$_2$	76	34[c]

[a]Reactions performed on a 0.1 mmol scale with respect to **1a** and using 1.2 equivalents of sulfamate ester. Yields refer to NMR yields of **2a** using 1,2-dimethoxyethane as an internal standard. [b]*ee* determined by chiral SFC analysis of purified **2a**. [c]The antipode **2a-ent** was obtained in these cases, but the magnitude of *ee* is shown for clarity.

Supplementary Table 8: Aziridination of a bishomoallylic alcohol

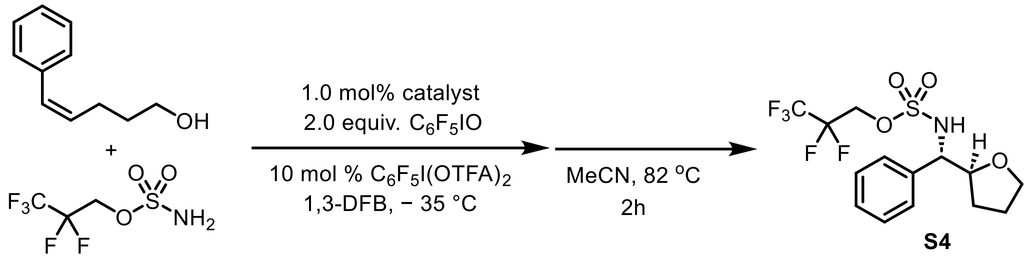

Entry	Catalyst	Yield of S4 / %[a]	*ee* of S4 / %[b]
1[d]	Rh_2(**A-I**)$_2$●(**Cat1**)$_2$●(**pyr**)$_2$	82	90
2	Rh_2(**A-I**)$_2$●(**Cat6**)$_2$●(**pyr**)$_2$	20	9
3	Rh_2(**A-I**)$_2$●(**Cat7**)$_2$●(**pyr**)$_2$	9	18
4	Rh_2(**A-I**)$_2$●(**Cat8**)$_2$●(**pyr**)$_2$	47	83
5	Rh_2(**A-I**)$_2$●(**Cat9**)$_2$●(**pyr**)$_2$	68	80
6	Rh_2(**A-I**)$_2$●(**Cat10**)$_2$●(**pyr**)$_2$	75	92[c]
7	Rh_2(**A-I**)$_2$●(**Cat11**)$_2$●(**pyr**)$_2$	69	74
8	Rh_2(**A-I**)$_2$●(**Cat12**)$_2$●(**pyr**)$_2$	60	36[c]
9[d]	Rh_2(**A-I**)$_2$●(**Cat1**)$_2$	78	80
10	Rh_2(**A-I**)$_2$●(**Cat6**)$_2$	82	7
11	Rh_2(**A-I**)$_2$●(**Cat7**)$_2$	62	22
12	Rh_2(**A-I**)$_2$●(**Cat8**)$_2$	60	68
13	Rh_2(**A-I**)$_2$●(**Cat9**)$_2$	68	68
14	Rh_2(**A-I**)$_2$●(**Cat10**)$_2$	71	76[c]
15	Rh_2(**A-I**)$_2$●(**Cat11**)$_2$	68	32
16	Rh_2(**A-I**)$_2$●(**Cat12**)$_2$	38	22[c]

[a]Reactions performed on a 0.1 mmol scale with respect to (*Z*)-5-phenylpent-4-en-1-ol and using 1.2 equivalents of sulfamate ester. Reactions were performed according to the general procedure below. Yields refer to NMR yields of **S4** using 1,2-dimethoxyethane as an internal standard. [b]*ee* determined by chiral SFC analysis of purified **S4**. [c]The antipode **S4-ent** was obtained in these cases, but the magnitude of *ee* is shown for clarity. [d]This result was reported in our previous work.[5] See this report for characterisation data for **S4**.

General procedure for aziridination and cyclisation reactions in **Supplementary Table 8**:

Under an atmosphere of air, a 4.0 mL crimp-top vial was charged with (*Z*)-5-phenylpent-4-en-1-ol (0.1 mmol, 1.0 equiv.) and catalyst (1.0 mol %). A solution of 2,2,3,3-pentafluoropropyl sulfamate (0.12 mmol, 1.2 equiv.) in 1,3-difluorobenzene (0.5 mL, 0.2 M) was then added via syringe. The vial was then cooled to – 35 °C over 10 minutes. Following this, pentafluoroiodsobenzene (C_6F_5IO) (0.2 mmol, 2.0 equiv.) and (perfluorophenyl)-λ^3-iodanediyl bis(2,2,2-trifluoroacetate) ($C_6F_5I(OTFA)_2$) (10 mol %) were added together in a single portion. The vial was then capped and the reaction mixture stirred at the same temperature for 14 hours. Saturated aqueous

solution of thiourea (0.5 mL) and $CHCl_3$ (0.5 mL) were then added and the biphasic mixture was stirred vigorously for 5 minutes at – 35 °C. The mixture was then allowed to warm to room temperature and stirred for a further 15 minutes. The layers were separated and the aqueous layer extracted with $CHCl_3$ (4 x 1.0 mL). The combined organic layer was dried over $MgSO_4$, filtered, concentrated and re-dissolved in MeCN (3.5 mL). The solution was then transferred to a crimp-top vial and heated at 82 °C for 2 hours. Following this, the MeCN was removed under reduced pressure. After confirming NMR yield, purification of the crude residue by flash column chromatography (SiO_2, 0-3% v/v Et_2O in CH_2Cl_2) afforded the compound **S4**.

Supplementary Figure 1: Comparison of *ee* values for **S4** for various catalysts (to accompany **Supplementary Table 8**).

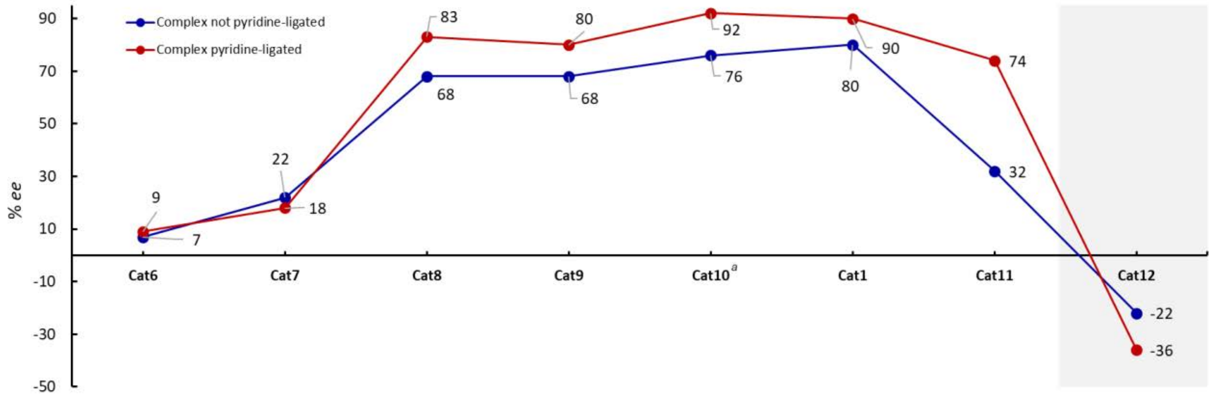

[a]The antipode **S4-ent** was obtained in this case, but the magnitude of *ee* is shown for clarity.

Synthesis of Chiral Cation Bromide Salts

The syntheses of chiral cation bromide salts **Cat1●**Br-**Cat5●**Br, **Cat10●**Br and **Cat13●**Br has been reported previously.[5, 6]

Those reported in *J. Am. Chem. Soc.* **2021**, 143, 27, 10070–10076:

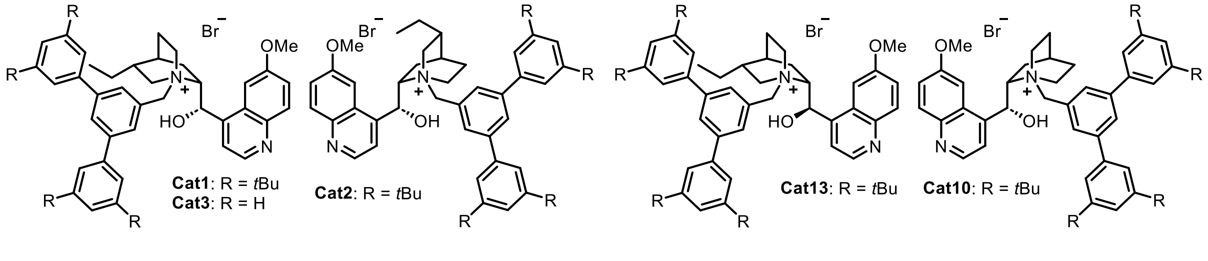

Those reported in *J. Am. Chem. Soc.* **2023**, 145, 13, 7516–7527:

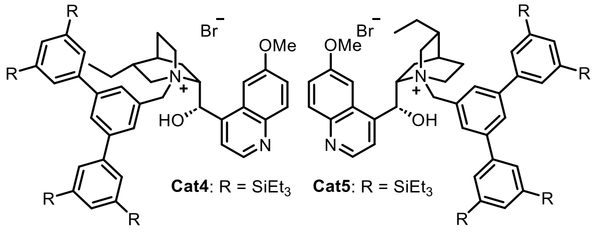

Synthesis of Chiral Cation **Cat6•Br** *from* **11a**

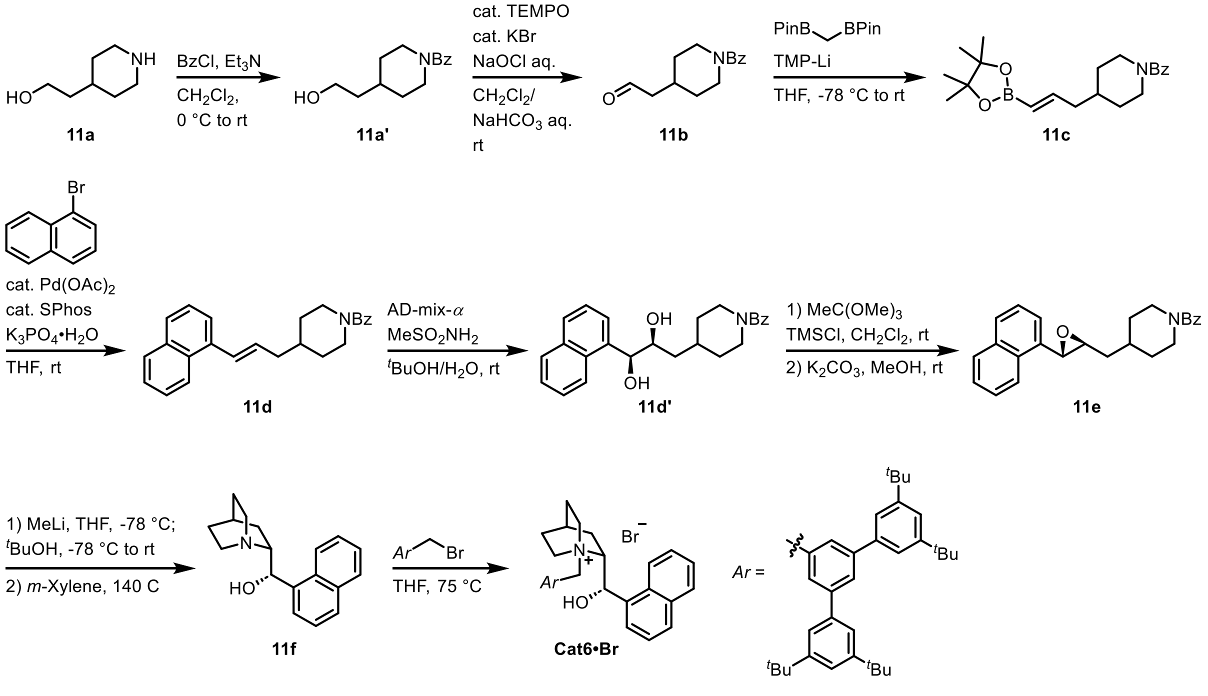

The compound **11c** was prepared according to the protocol described by Carbó, Fernández and co-workers.[7] The Suzuki-Miyaura coupling conditions for making compound **11d** was followed by the protocol described by

Jacobsen and co-workers.[8] The synthesis of the compound **11f** from **11d** was followed by the protocol described by Lygo and co-workers.[9]

(4-(2-hydroxyethyl)piperidin-1-yl)(phenyl)methanone (**11a'**)

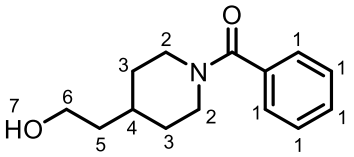

To a solution of Et_3N (2.54 mL, 18.2 mmol, 2.0 equiv) and 4-piperidine-ethanol (**11a**) (1.29 g, 10.0 mmol, 1.1 equiv) in dry CH_2Cl_2 (40 mL), benzoyl chloride (1.06 mL, 9.09 mmol, 1.0 equiv) was added dropwise at 0 °C under an inert atmosphere of nitrogen. The reaction mixture was allowed to warm to room temperature and stirred for 17 hours. After that, MeOH (5 mL) was added to the mixture and then all the volatile compounds were removed under reduced pressure. Purification by flash column chromatography (SiO_2, ethyl acetate only) afforded the title compound (**11a'**) as a sticky colorless oil (1.95 g, 8.36 mmol, 92% yield).

***R$_f$* value**: 0.29 (ethyl acetate only);

^{1}H NMR (400 MHz, $CDCl_3$): δ 7.38-7.33 (m, 5 H, H-1), 4.65 (br s, 1 H, H-2a), 3.70-3.62 (m, 3 H, H-2b, H-6), 2.98-2.92 (m, 1 H, H-2c), 2.76-2.70 (m, 1 H, H-2d), 2.14 (br s, 1 H, H-7), 1.81-1.62 (m, 3 H, H-3a, H-3b, H-4), 1.49 (q, *J* = 6.6 Hz, 2 H, H-5), 1.23-1.16 (m, 2 H, H-3c, H-3d) ppm;

^{13}C NMR (101 MHz, $CDCl_3$): δ 170.4, 136.4, 129.5, 128.5, 126.9, 60.0, 48.1*, 42.6*, 39.1, 32.9*, 32.8, 32.0* ppm;

* Observed as broad signals.

HRMS (+ESI): *m/z* found $[M+H]^+$ 234.1487, $[C_{14}H_{20}NO_2]^+$ requires 234.1489, (δ = - 0.9 ppm).

The ^{1}H NMR data is in agreement with that reported in the literature.[9]

2-(1-benzoylpiperidin-4-yl)acetaldehyde (**11b**)

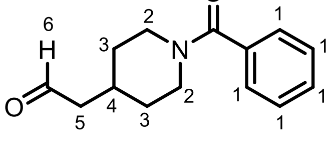

To a solution of the compound **11a'** (4.83 g, 20.7 mmol, 1.0 equiv), 2,2,6,6-tetramethylpiperidine 1-oxyl (TEMPO, 25.9 mg, 0.167 mmol, 0.8 mol %), and KBr (39.4 mg, 0.331 mmol, 1.6 mol %) in CH_2Cl_2 (100 mL) and 5% aqueous $NaHCO_3$ solution (50 mL), 14% aqueous NaOCl solution (19 mL, 41.4 mmol, 2.0 equiv) was added dropwise at room temperature and then the mixture was stirred for 1 hour. After that, saturated aqueous $Na_2S_2O_5$ solution was added to the reaction mixture until the orange color of organic layer disappeared. The resulting mixture

was diluted with water and CH_2Cl_2. The aqueous layer was thrice extracted with CH_2Cl_2 and the combined organic layer was dried over $MgSO_4$, filtered and concentrated. Purification by flash column chromatography (SiO_2, ethyl acetate only) afforded the title compound (**11b**) as a sticky colorless oil (4.11 g, 17.8 mmol, 86% yield). This oil was changed to a white solid upon cooling.

R

R_f value: 0.24 (33% v/v hexane in ethyl acetate);

^{1}H NMR (400 MHz, $CDCl_3$): δ 9.75 (t, *J* = 1.6 Hz, 1 H, H-6), 7.38-7.33 (m, 5 H, H-1), 4.67 (br s, 1 H, H-2a), 3.71 (br s, 1 H, H-2b), 3.00 (br s, 1 H, H-2c), 2.79 (br s, 1 H, H-2d), 2.41-2.39 (m, 2 H, H-5), 2.21-2.10 (m, 1 H, H-4), 1.80 (br s, 1 H, H-3a), 1.66 (br s, 1 H, H-3b), 1.27-1.16 (m, 2 H, H-3c, H-3d) ppm;

^{13}C NMR (101 MHz, $CDCl_3$): δ 201.2, 170.4, 136.2, 129.6, 128.5, 126.9, 50.2, 47.8*, 42.3*, 32.6*, 31.8*, 30.7 ppm;

* Observed as broad signals.

HRMS (+ESI): *m/z* found $[M+H]^+$ 232.1334, $[C_{14}H_{18}NO_2]^+$ requires 232.1332, (δ = + 0.9 ppm).

The ^{1}H NMR data is in agreement with that reported in the literature.[9]

(E)-phenyl(4-(3-(4,4,5,5-tetramethyl-1,3,2-dioxaborolan-2-yl)allyl)piperidin-1-yl)methanone (**11c**)

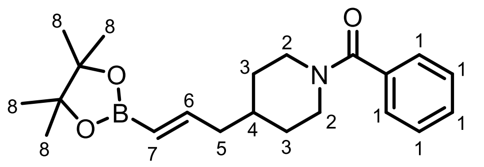

To a solution of 2,2,6,6-tetramethylpiperidine (TMP, 0.371 mL, 2.20 mmol, 1.1 equiv) in dry THF (10 mL), ^{n}BuLi (1.6 M hexane solution, 1.38 mL, 2.21 mmol, 1.1 equiv) was added dropwise at 0 °C under an inert atmosphere of nitrogen and the reaction mixture was stirred for 40 minutes at 0 °C. After that, the reaction mixture was cooled to -78 °C and a solution of bis(pinacolboryl)methane (643 mg, 2.40 mmol, 1.2 equiv) in dry THF (6.0 mL) was added dropwise to the mixture. After the reaction mixture was stirred for 30 minutes at -78 °C, a solution of the compound **11b** (467 mg, 2.02 mmol, 1.0 equiv) in dry THF (3.0 mL) was added dropwise to the mixture and then the reaction mixture was allowed to warm to room temperature and stirred for 16 hours. The reaction mixture was quenched with saturated aqueous NH_4Cl solution (10 mL) and diluted with water and ethyl acetate. The aqueous layer was thrice extracted with ethyl acetate and the combined organic layer was dried over $MgSO_4$, filtered and concentrated. Purification by flash column chromatography (SiO_2, 40% v/v ethyl acetate in hexane) afforded the title compound (**11c**) as a white solid (660 mg, 1.86 mmol, 92% yield).

R_f value: 0.30 (40% v/v ethyl acetate in hexane);

^{1}H NMR (400 MHz, $CDCl_3$): δ 7.40-7.36 (m, 5 H, H-1), 6.56 (dt, *J* = 17.8, 7.0 Hz, 1 H, H-6), 5.44 (dt, *J* = 17.8, 1.4 Hz, 1 H, H-7), 4.69 (br s, 1 H, H-2a), 3.72 (br s, 1 H, H-2b), 2.98-2.71 (m, 2 H, H-2c, H-2d), 2.16-2.12 (m, 2 H, H-5), 1.81

(br s, 1 H, H-3a), 1.72-1.61 (m, 2 H, H-3b, H-4, overlapped with the signal of H_2O), 1.26-1.13 (m, 14 H, H-3c, H-3d, H-8) ppm;

^{13}C NMR (101 MHz, $CDCl_3$): δ 170.4, 151.6, 136.5, 129.5, 128.5, 126.9, 121.1†, 83.2, 48.1*, 42.9, 42.5*, 35.7, 32.8*, 31.9*, 24.9 ppm;

* Observed as broad signals.

† Weak signal but clearly visible in the ^{1}H-^{13}C HSQC.

HRMS (+ESI): *m/z* found $[M+H]^+$ 356.2392, $[C_{21}H_{31}BNO_3]^+$ requires 356.2392, (δ = ± 0.0 ppm).

(E)-(4-(3-(naphthalen-1-yl)allyl)piperidin-1-yl)(phenyl)methanone (**11d**)

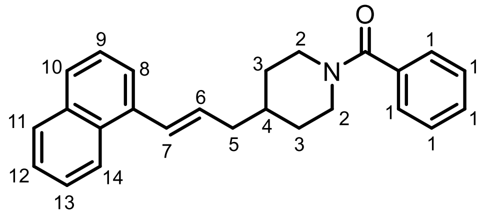

An oven-dried round bottom flask was charged with $Pd(OAc)_2$ (22.4 mg, 0.10 mmol, 2.5 mol %), SPhos (82.1 mg, 0.20 mmol, 5 mol %), $K_3PO_4{\bullet}H_2O$ (1.84 g, 8.0 mmol, 2.0 equiv), and the compound **11c** (1.44 g, 4.05 mmol, 1.0 equiv), and thrice evacuated and backfilled with nitrogen. After the addition of dry THF (10 mL), the mixture was stirred for 5 minutes at room temperature and then 1-bromonaphthalene (0.62 mL, 4.40 mmol, 1.1 equiv) was added to the mixture. The reaction mixture was stirred at room temperature for 14 hours. After the reaction, ethyl acetate and water were added to the mixture. The aqueous layer was thrice extracted with ethyl acetate and the combined organic layer was dried over $MgSO_4$, filtered and concentrated. Purification by flash column chromatography (SiO_2, 33-50% v/v ethyl acetate in hexane) afforded the title compound (**11d**) as a pale-yellow sticky oil (1.19 g, 3.35 mmol, 83% yield).

***R_f* value**: 0.54 (50% v/v ethyl acetate in hexane);

^{1}H NMR (400 MHz, $CDCl_3$): δ 8.10 (d, *J* = 7.9 Hz, 1 H, H-14), 7.85 (d, *J* = 7.6 Hz, 1 H, H-11), 7.76 (d, *J* = 8.2 Hz, 1 H, H-10), 7.57-7.44 (m, 4 H, H-8, H-9, H-12, H-13), 7.41 (s, 5 H, H-1), 7.14 (d, *J* = 15.3 Hz, 1 H, H-7), 6.20 (dt, *J* = 15.3, 7.4 Hz, 1 H, H-6), 4.76 (br s, 1 H, H-2a), 3.78 (br s, 1 H, H-2b), 3.00 (br s, 1 H, H-2c), 2.80 (br s, 1 H, H-2d), 2.34-2.31 (m, 2 H, H-5), 1.92-1.75 (m, 3 H, H-3a, H-3b, H-4), 1.37-1.23 (m, 2 H, H-3c, H-3d) ppm;

^{13}C NMR (101 MHz, $CDCl_3$): δ 170.4, 136.5, 135.4, 133.7, 131.4, 131.1, 129.5, 129.1, 128.6, 128.5, 127.6, 126.9, 126.0, 125.8, 125.7, 123.9, 123.7, 48.1*, 42.6*, 40.4, 36.7, 32.8*, 32.0* ppm;

* Observed as broad signals.

HRMS (+ESI): *m/z* found $[M+H]^+$ 356.2009, $[C_{25}H_{26}NO]^+$ requires 356.2009, (δ = ± 0.0 ppm).

The ^{1}H NMR data is in agreement with that reported in the literature.[9]

(4-((2S,3S)-2,3-dihydroxy-3-(naphthalen-1-yl)propyl)piperidin-1-yl)(phenyl)methanone (**11d'**)

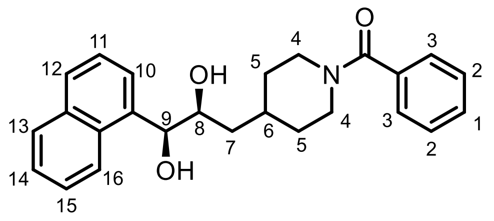

To a solution of AD-mix-*α* (3.0 g) and methanesulfonamide (713 mg, 7.50 mmol, 5.0 equiv) in ^{t}BuOH/H_2O (20 mL, v/v 1:1), the compound **11d** (533 mg, 1.50 mmol, 1.0 equiv) in ^{t}BuOH (5 mL) was added at room temperature. The reaction mixture was stirred for 62 hours. After the reaction was quenched with saturated aqueous Na_2SO_3 solution (10 mL), the resulting mixture was diluted with ethyl acetate and water. The aqueous layer was thrice extracted with ethyl acetate and the combined organic layer was dried over $MgSO_4$, filtered and concentrated. Purification by flash column chromatography (SiO_2, 75-100% v/v ethyl acetate in hexane) afforded the title compound (**11d'**) as a colorless sticky oil (490 mg, 1.26 mmol, 88% yield, >99% *ee*).

***R_f* value**: 0.30 (ethyl acetate only);

^{1}H NMR (400 MHz, $CDCl_3$): δ 8.05-8.02 (m, 1 H, H-16), 7.85-7.82 (m, 1 H, H-13), 7.76 (d, *J* = 8.0 Hz, 1 H, H-12), 7.53-7.40 (m, 4 H, H-10, H-11, H-14, H-15), 7.37-7.28 (m, 3 H, H-1, H-2), 7.24-7.22 (m, 2 H, H-3), 5.12 (dd, *J* = 5.8, 4.5 Hz, 1 H, H-9), 4.50 (br s, 1 H, H-4a), 3.99 (br s, 1 H, H-8), 3.56 (br s, 1 H, H-4b), 3.41 (br s, 1 H, O*H*), 3.03 (br s, 1 H, O*H*), 2.88-2.77 (m, 1 H, H-4c), 2.60-2.58 (m, 1 H, H-4d), 1.75-1.52 (m, 4 H, H-5a, H-5b, H-6, H-7a), 1.04-0.77 (m, 3 H, H-5c, H-5d, H-7b) ppm;

^{13}C NMR (101 MHz, $CDCl_3$): δ 170.3, 137.2, 136.2, 134.0, 131.0, 129.5, 129.1, 128.6, 128.4, 126.8, 126.3, 125.8, 125.4, 124.7, 123.3, 74.8, 72.6, (48.2, 48.0, 42.6, 42.4)*, 39.7, (33.8, 32.8, 31.8, 30.9)*, 32.6 ppm;

* Observed as broad signals. 2D NMR experiments clearly showed the signals in parentheses are the same position carbons of the piperidine structure.

HRMS (+ESI): *m/z* found $[M+H]^+$ 390.2069, $[C_{25}H_{28}NO_3]^+$ requires 390.2064, (δ = + 1.3 ppm);

$[\alpha]_D^{25.0}$ = + 12.8 (c = 0.86, $CHCl_3$);

Chiral SFC Analysis: CHIRALPAK IG (CO_2:MeOH, 60:40, 2.5 mL min^{-1}, 40 °C) t_R = 8.99 (major), 10.27 (minor) minutes.

The ^{1}H NMR data is in agreement with that reported in the literature.[9]

(4-(((2S,3S)-3-(naphthalen-1-yl)oxiran-2-yl)methyl)piperidin-1-yl)(phenyl)methanone (**11e**)

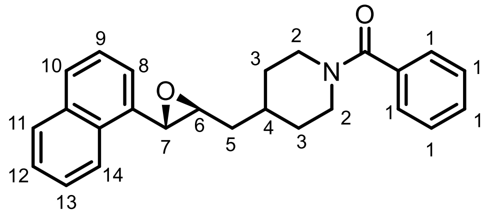

To a solution of compound **11d'** (1.02 g, 2.62 mmol, 1.0 equiv) and trimethyl orthoacetate (0.43 mL, 3.38 mmol, 1.3 equiv) in dry CH_2Cl_2 (15 mL), trimethylsilyl chloride (0.43 mL, 3.39 mmol, 1.3 equiv) was added dropwise at room temperature under an inert atmosphere of nitrogen. The reaction mixture was stirred at room temperature for 3 hours and then concentrated under reduced pressure. The resulting oil was dissolved in methanol (15 mL) and K_2CO_3 (471 mg, 3.41 mmol, 1.3 equiv) was added to the mixture. The reaction mixture was stirred at room temperature for 1.5 hours. After addition of saturated aqueous NH_4Cl solution (10 mL), the resulting mixture was diluted with ethyl acetate and water. The aqueous layer was thrice extracted with ethyl acetate and the combined organic layer was dried over $MgSO_4$, filtered and concentrated. Purification by flash column chromatography (SiO_2, 40% v/v ethyl acetate in hexane) afforded the title compound (**11e**) as a colorless sticky oil (778 mg, 2.09 mmol, 80% yield, 98% *ee*).

***R_f* value**: 0.38 (50% v/v ethyl acetate in hexane);

^{1}H NMR (700 MHz, $CDCl_3$): δ 8.07 (d, *J* = 8.3 Hz, 1 H, H-14), 7.88 (d, *J* = 8.1 Hz, 1 H, H-11), 7.80-7.78 (m, 1 H, H-10), 7.56-7.51 (m, 2 H, H-12, H-13), 7.45-7.43 (m, 2 H, H-8, H-9), 7.40 (s, 5 H, H-1), 4.75 (br s, 1 H, H-2a), 4.25 (br s, 1 H, H-7), 3.77 (br s, 1 H, H-2b), 3.03-2.99 (m, 2 H, H-2c, H-6), 2.79 (br s, 1 H, H-2d), 1.95-1.89 (m, 3 H, H-3a, H-4, H-5a), 1.79-1.72 (m, 2 H, H-3b, H-5b), 1.41-1.29 (m, 2 H, H-3c, H-3d) ppm;

^{13}C NMR (176 MHz, $CDCl_3$): δ 170.3, 136.3, 133.4, 133.3, 131.3, 129.5, 128.8, 128.4, 128.1, 126.8, 126.4, 125.9, 125.6, 122.7, 122.1*, (60.7, 60.5)†, 56.7, 47.9*, 42.3*, 39.1, 34.6, (33.1, 32.7, 32.2, 31.9)† ppm;

* Observed as broad signals.

† Observed as broad signals. 2D NMR experiments clearly showed the signals in parentheses are the same position carbons of the piperidine structure.

HRMS (+ESI): *m/z* found $[M+H]^+$ 372.1960, $[C_{25}H_{26}NO_2]^+$ requires 372.1958, (δ = + 0.5 ppm);

$[\alpha]_D^{25.0}$ = + 28.0 (c = 1.2, $CHCl_3$);

Chiral SFC Analysis: CHIRALPAK IA (CO_2:MeOH, 65:35, 2.5 mL min^{-1}, 40 °C, 220 nm) indicated 98% *ee*, t_R = 4.94 (major), 5.58 (minor) minutes.

The ^{1}H NMR data is in agreement with that reported in the literature.[9]

Note on racemic dihydroxylation and subsequent epoxidation reaction performed: In order to obtain racemic SFC trace, racemic dihydroxylation was conducted using the compound **11d**. The corresponding racemic epoxide was also prepared from the racemic *cis*-diol **11d'** by same procedure when making the chiral one.

Dihydroxylation: To a solution of the compound **11d** (178 mg, 0.50 mmol, 1.0 equiv) in acetone/H_2O (12.5 mL, v/v 4:1), K_2OsO_4•H_2O (3.6 mg, 0.01 mmol, 2 mol %) in H_2O (2.5 mL) and *N*-methylmorpholine *N*-oxide (50% wt in H_2O, 0.156 mL, 0.75 mmol, 1.5 equiv) was added at room temperature. The reaction mixture was stirred for 8 hours. After the reaction was quenched with saturated aqueous Na_2SO_3 solution (20 mL), the resulting mixture was diluted with ethyl acetate and water. The aqueous layer was thrice extracted with ethyl acetate and the combined organic layer was dried over $MgSO_4$, filtered and concentrated. Purification by flash column chromatography (SiO_2, ethyl acetate only) afforded the racemic *cis*-diol **11d'** as a colorless sticky oil (193 mg, 0.494 mmol, 99% yield).

Epoxidation: To a solution of racemic *cis*-diol **11d'** (117 mg, 0.301 mmol, 1.0 equiv) and trimethyl orthoacetate (0.050 mL, 0.393 mmol, 1.3 equiv) in dry CH_2Cl_2 (10 mL), trimethylsilyl chloride (0.050 mL, 0.393 mmol, 1.3 equiv) was added dropwise at room temperature under an inert atmosphere of nitrogen. The reaction mixture was stirred at room temperature for 4 hours and then concentrated under reduced pressure. The resulting oil was dissolved in methanol (10 mL) and K_2CO_3 (53.9 mg, 0.390 mmol, 1.3 equiv) was added to the mixture. The reaction mixture was stirred at room temperature for 3 hours. After addition of saturated aqueous NH_4Cl solution (5 mL), the resulting mixture was diluted with ethyl acetate and water. The aqueous layer was thrice extracted with ethyl acetate and the combined organic layer was dried over $MgSO_4$, filtered and concentrated. Purification by flash column chromatography (SiO_2, 50% v/v ethyl acetate in hexane) afforded the racemic epoxide **11e** as a pale-yellow sticky oil (91.9 mg, 0.247 mmol, 82% yield).

(S)-naphthalen-1-yl((1S,2R,4S)-quinuclidin-2-yl)methanol (**11f**)

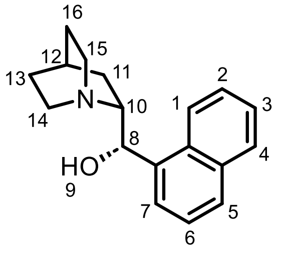

To a solution of compound **11e** (778.0 mg, 2.09 mmol, 1.0 equiv) in dry THF (30 mL), MeLi (1.6 M Et_2O solution, 1.31 mL, 2.09 mmol, 1.0 equiv) was added dropwise at -78 °C under an inert atmosphere of nitrogen. The reaction mixture was stirred at -78 °C for 30 minutes and then ^{t}BuOH (0.60 mL, 6.27 mmol, 3.0 equiv) was added to the mixture. The resulting mixture was allowed to warm to room temperature and concentrated under reduced pressure. The resulting oil was dissolved in dry *m*-xylene (20 mL) under an inert atmosphere of nitrogen and the solution was heated at 140 °C for 64 hours. After cooling to room temperature, the solvent was removed

under reduced pressure. The resulting solid was dissolved in small amount of ethyl acetate and the solution was added dropwise to hexane (500 mL). The residue was collected by filtration and washed with hexane. The residue was purified by flash column chromatography (SiO_2, 20% v/v 70:29:1 CH_2Cl_2/MeOH/NH_4OH (aq.) in CH_2Cl_2 and then 10-20% v/v (2 M NH_3 in MeOH) in CH_2Cl_2) and the obtained solid was dissolved in ethyl acetate. The saturated aqueous $NaHCO_3$ solution was added to the solution and the aqueous layer was thrice extracted with ethyl acetate. The combined organic layer was dried over K_2CO_3, filtered and concentrated. The title compound (**11f**) was afforded as a white solid (177 mg, 0.663 mmol, 32% yield).

***R_f* value**: 0.52 (20% v/v (2 M NH_3 in MeOH) in CH_2Cl_2);

^{1}H NMR (400 MHz, $CDCl_3$): δ 8.09-8.07 (m, 1 H, H-1), 7.88-7.84 (m, 1 H, H-4), 7.76 (d, *J* = 8.2 Hz, 1 H, H-5), 7.69 (d, *J* = 7.1 Hz, 1 H, H-7), 7.50-7.43 (m, 3 H, H-2, H-3, H-6), 5.71 (d, *J* = 4.2 Hz, 1 H, H-8), 3.51-3.42 (m, 1 H, H-14a), 3.24-3.18 (m, 1 H, H-10), 2.94-2.77 (m, 3 H, H-9, H-15), 2.73-2.66 (m, 1 H, H-14b), 1.88-1.83 (m, 2 H, H-11a, H-12), 1.64-1.56 (m, 1 H, H-13a), 1.50-1.37 (m, 4 H, H-11b, H-13b, H-16) ppm;

^{13}C NMR (101 MHz, $CDCl_3$): δ 139.5, 133.9, 130.6, 129.1, 128.1, 126.2, 125.6, 125.5, 123.6, 123.3, 73.3, 60.2, 50.9, 44.0, 27.0, 26.8, 25.9, 22.2 ppm;

HRMS (+ESI): *m/z* found $[M+H]^+$ 268.1699, $[C_{18}H_{22}NO]^+$ requires 268.1696, (δ = + 1.3 ppm);

$[\alpha]_D^{25.0}$ = + 113.0 (c = 0.49, $CHCl_3$).

The ^{1}H NMR data is in agreement with that reported in the literature.[9]

(1S,2R,4S)-2-((S)-hydroxy(naphthalen-1-yl)methyl)-1-((3,3'',5,5''-tetra-tert-butyl-[1,1':3',1''-terphenyl]-5'-yl)methyl)quinuclidin-1-ium bromide (**Cat6•Br**)

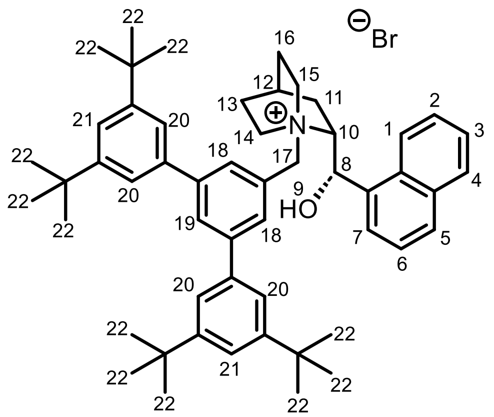

Prepared according to **GP1** on a 0.30 mmol scale with respect to the compound **11f** and using 5'-(bromomethyl)-3,3'',5,5''-tetra-tert-butyl-1,1':3',1''-terphenyl as the benzyl bromide. Purification by flash column chromatography (SiO_2, 0-8 % v/v MeOH in CH_2Cl_2) afforded the title compound (**Cat6•Br**) as an off-white solid (207 mg, 0.253 mmol, 84% yield).

***R*$_f$ value**: 0.31 (10% v/v MeOH in CH_2Cl_2);

^{1}H NMR (700 MHz, $CDCl_3$): δ 8.04 (br s, 2 H, H-18), 8.00 (d, *J* = 8.3 Hz, 1 H, H-1), 7.97 (d, *J* = 7.2 Hz, 1 H, H-7), 7.87 (br s, 1 H, H-19), 7.76 (d, *J* = 8.3 Hz, 1 H, H-4), 7.71 (d, *J* = 8.0 Hz, 1 H, H-5), 7.52-7.50 (m, 7 H, H-2, H-20, H-21), 7.46-7.44 (m, 1 H, H-6), 7.33 (t, *J* = 7.4 Hz, 1 H, H-3), 6.87 (br d, *J* = 4.2 Hz, 1 H, H-8), 6.54 (br d, *J* = 5.6 Hz, 1 H, H-9), 6.24 (d, *J* = 12.5 Hz, 1 H, H-17a), 5.06 (d, *J* = 12.5 Hz, 1 H, H-17b), 4.99-4.95 (m, 1 H, H-14a), 3.84 (t, *J* = 8.9 Hz, 1 H, H-10), 3.64-3.60 (m, 1 H, H-15a), 3.48-3.44 (m, 1 H, H-14b), 3.40-3.36 (m, 1 H, H-15b), 2.37-2.34 (m, 1 H, H-11a), 2.11-2.08 (m, 1 H, H-13a), 2.04 (br s, 1 H, H-12), 1.77-1.71 (m, 2 H, H-16), 1.61 (br s, 1 H, H-13b, overlapped with the signal of H_2O), 1.41 (s, 36 H, H-22), 1.15-1.12 (m, 1 H, H-11b) ppm;

^{13}C NMR (176 MHz, $CDCl_3$): δ 151.7, 144.1, 139.7, 134.8, 133.6, 131.5, 129.3, 129.2, 128.8, 128.5, 128.2, 127.1, 126.1, 125.55, 125.53, 122.5, 122.3, 122.1, 69.9, 64.5, 64.0, 57.6, 51.6, 35.2, 31.7, 26.1, 24.3, 23.9, 20.8 ppm;

HRMS (+ESI): *m/z* For cation found 734.5296, $[C_{53}H_{68}NO]^+$ requires 734.5295, (δ = + 0.1 ppm);

$[\alpha]_D^{25.0}$ = + 66.3 (c = 0.47, $CHCl_3$).

Note: the synthesis of 5'-(bromomethyl)-3,3'',5,5''-tetra-tert-butyl-1,1':3',1''-terphenyl has been reported previously.[3]

<u>*Synthesis of Chiral Cation* **Cat7•Br** *from* **11c**</u>

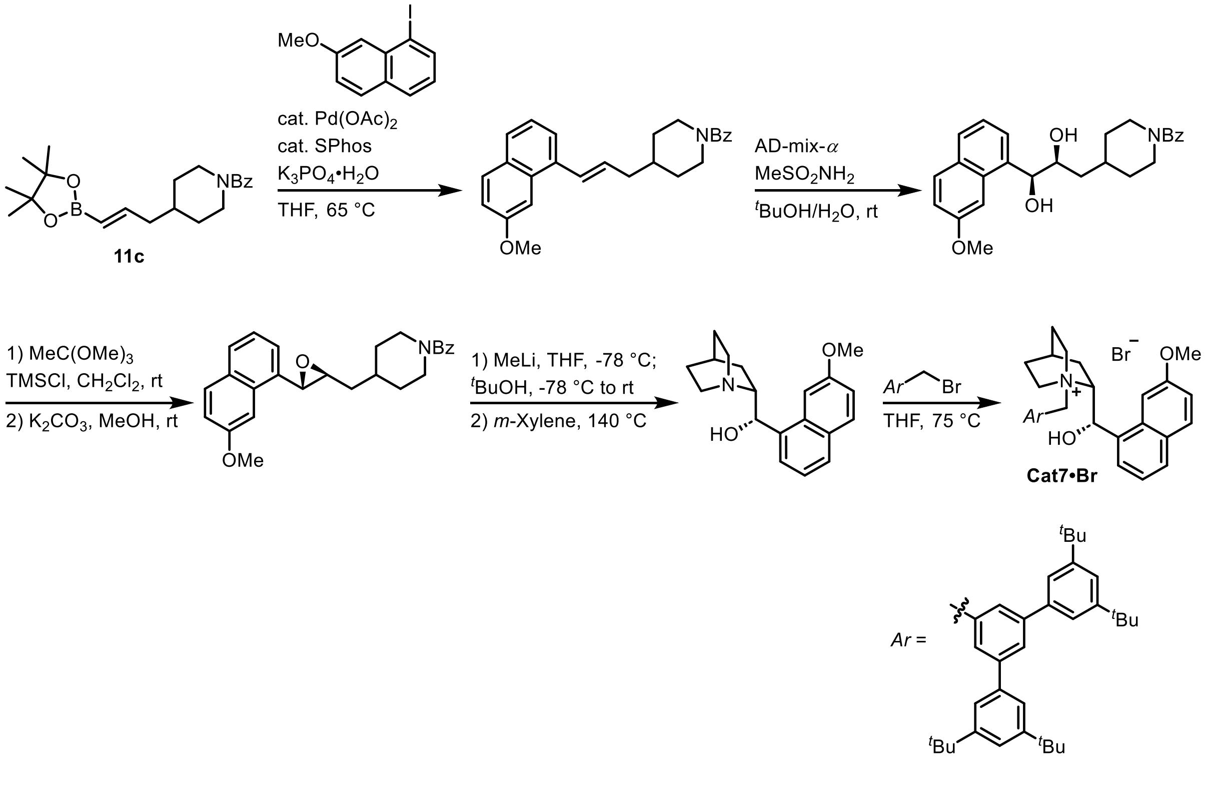

The Suzuki-Miyaura coupling of **11c** was conducted using a protocol described by Jacobsen and co-workers.[8] The remaining steps in the sequence were conducted using protocols described by Lygo and co-workers.[9]

7-Methoxynaphthalen-1-amine

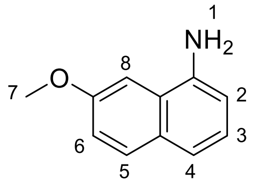

Prepared according to the protocol described by Jin and co-workers.[10] An oven-dried three-necked round bottom flask was charged with NaH (60% dispersion in mineral oil, 580 mg, 14.5 mmol, 1.2 equiv) and thrice evacuated and backfilled with nitrogen. After the addition of dry DMF (40 mL), the resulting slurry was stirred at 0 °C and 8-aminonaphthalen-2-ol (1.92 g, 12.1 mmol, 1.0 equiv) was added to the slurry. The mixture was allowed to warm to room temperature and stirred for 20 minutes. After the reaction mixture was cooled to 0 °C again, iodomethane (0.74 mL, 12.1 mmol, 1.0 equiv) was added dropwise to the mixture. The reaction mixture was allowed to warm to room temperature and stirred for 1 hour. After the addition of water (10 mL), the mixture was diluted with ethyl acetate (200 mL) and water (200 mL). The aqueous layer was thrice extracted with ethyl acetate and the combined organic layer was washed twice with water and brine, dried over $MgSO_4$, filtered and concentrated. Purification by flash column chromatography (SiO_2, 33% v/v ethyl acetate in hexane) afforded the title compound as a black solid (1.76 g, 10.2 mmol, 84% yield).

***R$_f$* value**: 0.53 (50% v/v ethyl acetate in hexane);

^{1}H NMR (400 MHz, $CDCl_3$): δ 7.73 (d, *J* = 8.9 H, 1 H, H-5), 7.30 (d, *J* = 8.1 Hz, 1 H, H-4), 7.20-7.15 (m, 2 H, H-3, H-6), 7.08 (d, *J* = 2.4 Hz, 1 H, H-8), 6.80 (dd, *J* = 7.3, 1.0, 1 H, H-2), 3.94 (s, 3 H, H-7, overlapped with the signal of H-1), 3.89 (br s, 2 H, H-1, overlapped with the signal of H-7) ppm;

^{13}C NMR (101 MHz, $CDCl_3$): δ 157.3, 141.0, 130.3, 129.9, 124.8, 124.0, 119.3, 118.4, 110.9, 99.8, 55.5 ppm.

The spectroscopic data is in agreement with that reported in the literature.[11]

1-Iodo-7-methoxynaphthalene

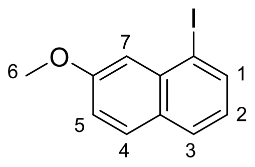

To a solution of 7-methoxynaphthalen-1-amine (866 mg, 5.0 mmol, 1.0 equiv) and TsOH•H_2O (2.85 g, 15.0 mmol, 3.0 equiv) in MeCN (50 mL) at 0 °C, $NaNO_2$ (690 mg, 10.0 mmol, 2.0 equiv) and KI (2.08 g, 12.5 mmol, 2.5 equiv) were added. The reaction mixture was allowed to warm to room temperature and stirred for 2 hours. After the addition of saturated aqueous Na_2SO_3 solution (10 mL), the resulting mixture was diluted with ethyl acetate and water. The aqueous layer was thrice extracted with ethyl acetate and the combined organic layer was washed

with water and brine, dried over $MgSO_4$, filtered and concentrated. Purification by flash column chromatography (SiO_2, 3% v/v ethyl acetate in hexane) afforded the title compound as an orange solid (0.912 g, 3.21 mmol, 63% yield).

***R_f* value**: 0.29 (hexane only);

^{1}H NMR (400 MHz, $CDCl_3$): δ 8.05 (dd, *J* = 7.4, 1.0 Hz, 1 H, H-1), 7.76 (d, *J* = 8.1 Hz, 1 H, H-3), 7.67 (d, *J* = 8.9 Hz, 1 H, H-4), 7.40 (d, *J* = 2.4 Hz, 1 H, H-7), 7.17 (dd, *J* = 8.1, 2.4 Hz, 1 H, H-5), 7.05 (dd, *J* = 8,1, 7.4 Hz, 1 H, H-2), 3.99 (s, 3 H, H-6) ppm;

^{13}C NMR (101 MHz, $CDCl_3$): δ 159.4, 138.0, 135.8, 130.4, 129.6, 128.8, 124.7, 119.7, 110.7, 98.3, 55.6 ppm.

The spectroscopic data is in agreement with that reported in the literature.[12]

(E)-(4-(3-(7-methoxynaphthalen-1-yl)allyl)piperidin-1-yl)(phenyl)methanone

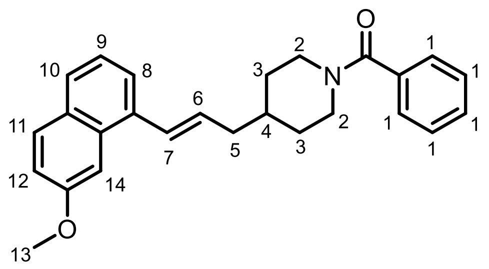

An oven-dried round bottom flask was charged with $Pd(OAc)_2$ (18.2 mg, 0.08 mmol, 2.4 mol %), SPhos (66.1 mg, 0.16 mmol, 5 mol %), K_3PO_4•H_2O (1.48 g, 6.44 mmol, 1.9 equiv), and the compound **11c** (1.21 g, 3.40 mmol, 1.0 equiv), and thrice evacuated and backfilled with nitrogen. After the addition of dry THF (20 mL), the mixture was stirred for 5 minutes at room temperature and then 1-iodo-7-methoxynaphthalene (967 mg, 3.40 mmol, 1.0 equiv) was added to the mixture. The reaction mixture was warmed to 65 °C and stirred for 22 hours. After cooling to room temperature, ethyl acetate and water were added to the mixture. The aqueous layer was thrice extracted with ethyl acetate and the combined organic layer was dried over $MgSO_4$, filtered and concentrated. Purification by flash column chromatography (SiO_2, 33% v/v ethyl acetate in hexane) afforded the title compound as a pale-yellow sticky oil (1.12 g, 2.90 mmol, 85% yield).

***R_f* value**: 0.46 (50% v/v ethyl acetate in hexane);

^{1}H NMR (400 MHz, $CDCl_3$): δ 7.75 (d, *J* = 9.0 Hz, 1 H, H-11), 7.69 (d, *J* = 8.1 Hz, 1 H, H-10), 7.53 (d, *J* = 7.1 Hz, 1 H, H-8), 7.43-7.39 (m, 5 H, H-1), 7.36 (d, *J* = 2.3 Hz, 1 H, H-14), 7.30 (t, *J* = 7.5 Hz, 1 H, H-9), 7.17 (dd, *J* = 9.0, 2.3 Hz, 1 H, H-12), 7.05 (d, *J* = 15.3 Hz, 1 H, H-7), 6.18 (dt, *J* = 15.3, 7.4 Hz, 1 H, H-6), 4.77 (br s, 1 H, H-2a), 3.94 (s, 3 H, H-13), 3.78 (br s, 1 H, H-2b), 3.00-2.80 (m, 2 H, H-2c, H-2d), 2.33 (t, *J* = 6.8 Hz, 2 H, H-5), 1.94 (br s, 1 H, H-3a), 1.80-1.69 (m, 2 H, H-3b, H-4), 1.37-1.26 (m, 2 H, H-3c, H-3d) ppm;

^{13}C NMR (101 MHz, $CDCl_3$): δ 170.3, 157.7, 136.4, 134.2, 132.2, 131.1, 130.1, 129.5, 129.2, 129.1, 128.4, 127.3, 126.9, 124.3, 123.4, 118.1, 102.6, 55.3, 48.1*, 42.5*, 40.3, 36.7, 32.8*, 31.9* ppm;

* Observed as broad signals.

HRMS (+ESI): *m/z* found $[M+H]^+$ 386.2118, $[C_{26}H_{28}NO_2]^+$ requires 386.2115, (δ = + 0.8 ppm).

(4-((2S,3S)-2,3-dihydroxy-3-(7-methoxynaphthalen-1-yl)propyl)piperidin-1-yl)(phenyl)methanone

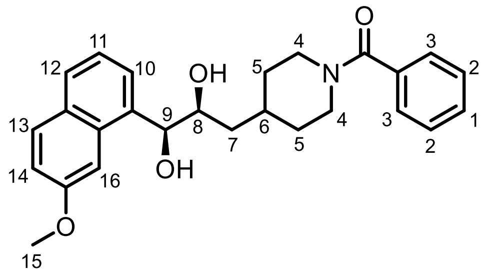

To a solution of AD-mix-*α* (0.5 g) and methanesulfonamide (123 mg, 1.29 mmol, 5.0 equiv) in ^{t}BuOH/H_2O (10 mL, v/v 1:1), (*E*)-(4-(3-(7-methoxynaphthalen-1-yl)allyl)piperidin-1-yl)(phenyl)methanone (98.9 g, 0.257 mmol, 1.0 equiv) was added at room temperature. The reaction mixture was stirred for 16 hours. After the reaction was quenched with saturated aqueous Na_2SO_3 solution (10 mL), the resulting mixture was diluted with ethyl acetate and water. The aqueous layer was thrice extracted with ethyl acetate and the combined organic layer was dried over $MgSO_4$, filtered and concentrated. Purification by flash column chromatography (SiO_2, ethyl acetate only) afforded the title compound as a pale-yellow sticky oil (79.2 mg, 0.189 mmol, 74% yield, >99% *ee*).

***R_f* value**: 0.35 (ethyl acetate only);

^{1}H NMR (700 MHz, $CDCl_3$): δ 7.69 (d, *J* = 9.0 Hz, 1 H, H-13), 7.64 (d, *J* = 8.1 Hz, 1 H, H-10), 7.44 (br s, 1 H, H-12), 7.34-7.32 (m, 2 H, H-1, H-16), 7.28 (t, *J* = 7.3 Hz, 2 H, H-2), 7.26-7.23 (m, 1 H, H-11, overlapped with the signal of residual chloroform), 7.19 (br d, *J* = 7.1 Hz, 2 H, H-3), 7.13-7.11 (m, 1 H, H-14), 4.99 (d, *J* = 4.8 Hz, 1 H, H-9), 4.48-4.44 (m, 1 H, H-4a), 3.99-3.94 (m, 1 H, H-8), 3.84 (s, 3 H, H-15), 3.77-3.76 (m, 1 H, O*H*), 3.55-3.50 (m, 1 H, H-4b), 3.32-3.31 (m, 1 H, O*H*), 2.83-2.75 (m, 1 H, H-4c), 2.57-2.52 (m, 1 H, H-4d), 1.69-1.45 (m, 4 H, H-5a, H-5b, H-6, H-7a), 0.99-0.88 (m, 2 H, H-5c, H-7b), 0.82-0.67 (m, 1 H, H-5d) ppm;

^{13}C NMR (176 MHz, $CDCl_3$): δ 170.3, 157.7, 136.0, 135.8, 132.2, 130.4, 129.5, 129.4, 128.4, 128.2, 126.7, 125.2*, 123.1, 118.0, (102.51, 102.45)†, 75.0, 72.3, 55.3, (48.1, 47.9) †, (42.5, 42.3) †, 39.7, (33.7, 32.7, 31.7, 30.8) †, 32.5 ppm;

* Observed as broad signals.

† Observed as broad signals. 2D NMR experiments clearly showed the signals in parentheses are the same position carbons of the piperidine structure.

HRMS (+ESI): *m/z* found $[M+H]^+$ 420.2170, $[C_{26}H_{30}NO_4]^+$ requires 420.2169, (δ = + 0.2 ppm);

$[\alpha]_D^{25.0}$ = + 21.8 (c = 1.9, $CHCl_3$);

Chiral SFC Analysis: CHIRALPAK IG (CO_2:MeOH, 60:40, 2.5 mL min^{-1}, 40 °C) t_R = 8.23 (minor), 10.46 (major) minutes.

(4-(((2S,3S)-3-(7-methoxynaphthalen-1-yl)oxiran-2-yl)methyl)piperidin-1-yl)(phenyl)methanone

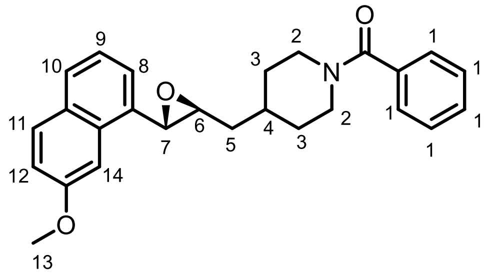

To a solution of the compound (4-((2S,3S)-2,3-dihydroxy-3-(7-methoxynaphthalen-1-yl)propyl)piperidin-1-yl)(phenyl)methanone (75.8 g, 0.181 mmol, 1.0 equiv) and trimethyl orthoacetate (0.030 mL, 0.235 mmol, 1.3 equiv) in dry CH_2Cl_2 (5 mL), trimethylsilyl chloride (0.030 mL, 0.235 mmol, 1.3 equiv) was added dropwise at room temperature under an inert atmosphere of nitrogen. The reaction mixture was stirred at room temperature for 3.5 hours and then concentrated under reduced pressure. The resulting oil was dissolved in methanol (5 mL) and K_2CO_3 (32.5 mg, 0.235 mmol, 1.3 equiv) was added to the mixture. The reaction mixture was stirred at room temperature for 2 hours. After addition of saturated aqueous NH_4Cl solution (5 mL), the resulting mixture was diluted with ethyl acetate and water. The aqueous layer was thrice extracted with ethyl acetate and the combined organic layer was dried over $MgSO_4$, filtered and concentrated. Purification by flash column chromatography (SiO_2, 50% v/v ethyl acetate in hexane) afforded the title compound as a colorless sticky oil (45.7 mg, 0.114 mmol, 63% yield, >99% *ee*).

***R_f* value**: 0.65 (25% v/v hexane in ethyl acetate);

^{1}H NMR (700 MHz, $CDCl_3$): δ 7.78 (d, *J* = 8.9 Hz, 1 H, H-11), 7.72 (d, *J* = 8.1 Hz, 1 H, H-10), 7.41-7.40 (m, 6 H, H-1, H-8), 7.32-7.29 (m, 2 H, H-9, H-14), 7.19 (br d, *J* = 8.3 Hz, 1 H, H-12), 4.75 (br s, 1 H, H-2a), 4.16 (br s, 1 H, H-7), 3.94-3.91 (m, 3 H, H-13), 3.78 (br s, 1 H, H-2b), 3.03-3.02 (m, 2 H, H-2c, H-6), 2.80 (br s, 1 H, H-2d), 1.99-1.73 (m, 5 H, H-3a, H-3b, H-4, H-5), 1.43-1.29 (m, 2 H, H-3c, H-3d) ppm;

^{13}C NMR (176 MHz, $CDCl_3$): δ 170.4, 158.0, 136.3, 132.6, 132.1, 130.4, 129.6, 128.8, 128.5, 128.0, 126.9, 123.3, 122.9, 118.3, 101.7, (60.3, 60.2)†, 57.1*, 55.4, 48.0*, 42.4*, 39.3, (34.7, 34.6)†, (33.3, 32.9, 32.3, 32.0)† ppm;

* Observed as broad signals.

† Observed as broad signals. 2D NMR experiments clearly showed the signals in parentheses are the same position carbons of the piperidine structure.

HRMS (+ESI): *m/z* found $[M+H]^+$ 402.2062, $[C_{26}H_{28}NO_3]^+$ requires 402.2064, (δ = - 0.5 ppm);

$[\alpha]_D^{25.0}$ = + 32.9 (c = 2.0, $CHCl_3$);

Chiral SFC Analysis: CHIRALPAK IG (CO_2:MeOH, 60:40, 2.5 mL min^{-1}, 40 °C) t_R = 23.83 (major), 31.25 (minor) minutes.

Note on racemic dihydroxylation and subsequent epoxidation reaction performed: In order to obtain racemic SFC trace, racemic dihydroxylation was conducted on (*E*)-(4-(3-(7-methoxynaphthalen-1-yl)allyl)piperidin-1-yl)(phenyl)methanone. The corresponding racemic epoxide was also prepared from the racemic *cis*-diol by the same procedure when making the enantioenriched version.

Dihydroxylation: To a solution of (*E*)-(4-(3-(7-methoxynaphthalen-1-yl)allyl)piperidin-1-yl)(phenyl)methanone (879 mg, 0.879 mmol, 1.0 equiv) in acetone/H_2O (6.25 mL, v/v 4:1), K_2OsO_4•H_2O (6.5 mg, 0.0176 mmol, 2 mol %) in H_2O (1.25 mL) and *N*-methylmorpholine *N*-oxide (50% wt in H_2O, 0.137 mL, 1.32 mmol, 1.5 equiv) was added at room temperature. The reaction mixture was stirred for 16 hours. After the reaction was quenched with saturated aqueous Na_2SO_3 solution (10 mL), the resulting mixture was diluted with ethyl acetate and water. The aqueous layer was thrice extracted with ethyl acetate and the combined organic layer was dried over $MgSO_4$, filtered and concentrated. Purification by flash column chromatography (SiO_2, 50-100% v/v ethyl acetate in hexane) afforded the racemic *cis*-diol as a white solid (242 mg, 0.576 mmol, 66% yield).

Epoxidation: To a solution of racemic *cis*-diol (129 mg, 0.307 mmol, 1.0 equiv) and trimethyl orthoacetate (0.050 mL, 0.393 mmol, 1.3 equiv) in dry CH_2Cl_2 (2 mL), trimethylsilyl chloride (0.050 mL, 0.393 mmol, 1.3 equiv) was added dropwise at room temperature under an inert atmosphere of nitrogen. The reaction mixture was stirred at room temperature for 3.5 hours and then concentrated under reduced pressure. The resulting oil was dissolved in methanol (2 mL) and K_2CO_3 (51.8 mg, 0.375 mmol, 1.2 equiv) was added to the mixture. The reaction mixture was stirred at room temperature for 1 hour. After addition of saturated aqueous NH_4Cl solution (5 mL), the resulting mixture was diluted with ethyl acetate and water. The aqueous layer was thrice extracted with ethyl acetate and the combined organic layer was dried over $MgSO_4$, filtered and concentrated. Purification by flash column chromatography (SiO_2, 66% v/v ethyl acetate in hexane) afforded the racemic epoxide as a pale-yellow sticky oil (104.7 mg, 0.261 mmol, 85% yield).

(S)-(7-methoxynaphthalen-1-yl)((1S,2R,4S)-quinuclidin-2-yl)methanol

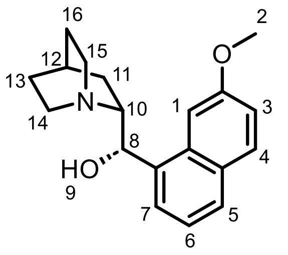

To a solution of (4-(((2S,3S)-3-(7-methoxynaphthalen-1-yl)oxiran-2-yl)methyl)piperidin-1-yl)(phenyl)methanone (1.21 g, 3.04 mmol, 1.0 equiv) in dry THF (40 mL), MeLi (1.6 M Et_2O solution, 1.90 mL, 3.04 mmol, 1.0 equiv) was added dropwise at -78 °C under an inert atmosphere of nitrogen. The reaction mixture was stirred at -78 °C for 30 minutes and then ^{t}BuOH (0.87 mL, 9.12 mmol, 3.0 equiv) was added to the mixture. The resulting mixture was allowed to warm to room temperature and concentrated under reduced pressure. The resulting oil was dissolved in dry *m*-xylene (30 mL) under an inert atmosphere of nitrogen and the solution was heated at 140 °C for 20 hours. After cooling to room temperature, the solvent was removed under reduced pressure. The resulting oil was dissolved in small amount of ethyl acetate and the solution was added dropwise to hexane (600 mL). The residue was removed by filtration and the filtrate was concentrated. The resulting solid from filtrate was purified by flash column chromatography (SiO_2, 30-50% v/v 70:29:1 CH_2Cl_2/MeOH/NH_4OH (aq.) in CH_2Cl_2) and then obtained solid was dissolved in ethyl acetate. The saturated aqueous $NaHCO_3$ solution was added to the solution and the aqueous layer was thrice extracted with ethyl acetate. The combined organic layer was dried over K_2CO_3, filtered and concentrated. The title compound was afforded as a white powder (307 mg, 1.03 mmol, 34% yield).

***R$_f$* value**: 0.31 (40% v/v 70:29:1 CH_2Cl_2/MeOH/NH_4OH (aq.) in CH_2Cl_2);

^{1}H NMR (400 MHz, $CDCl_3$): δ 7.75 (d, *J* = 9.0 Hz, 1 H, H-4), 7.68 (d, *J* = 8.1 Hz, 1 H, H-5), 7.64 (d, *J* = 7.1 Hz, 1 H, H-7), 7.35 (d, *J* = 2.4 Hz, 1 H, H-1), 7.33-7.29 (m, 1 H, H-6), 7,14 (dd, *J* = 9.0, 2.4 Hz, 1 H, H-3), 5.60 (d, *J* = 4.4 Hz, 1 H, H-8), 3.89 (s, 3 H, H-2), 3.48-3.40 (m, 1 H, H-14a), 3.23-3.12 (m, 2 H, H-9, H-10), 2.93-2.86 (m, 1 H, H-15a), 2.84-2.76 (m, 1 H, H-15b), 2.71-2.64 (m, 1 H, H-14b), 1.86-1.80 (m, 2 H, H-11a, H-12), 1.61-1.53 (m, 1 H, H-13a), 1.47-1.33 (m, 4 H, H-11b, H-13b, H-16) ppm;

^{13}C NMR (101 MHz, $CDCl_3$): δ 157.8, 138.2, 131.7, 130.5, 129.3, 127.7, 124.1, 123.2, 118.1, 102.1, 73.3, 59.9, 55.6, 50.9, 44.1, 26.9, 26.8, 25.8, 22.2 ppm;

HRMS (+ESI): *m/z* found $[M+H]^+$ 298.1809, $[C_{19}H_{24}NO]^+$ requires 298.1802, (δ = + 2.6 ppm);

$[\alpha]_D^{25.0}$ = + 126.3 (c = 0.51, $CHCl_3$).

(1S,2R,4S)-2-((S)-hydroxy(7-methoxynaphthalen-1-yl)methyl)-1-((3,3'',5,5''-tetra-tert-butyl-[1,1':3',1''-terphenyl]-5'-yl)methyl)quinuclidin-1-ium bromide (**Cat7•Br**)

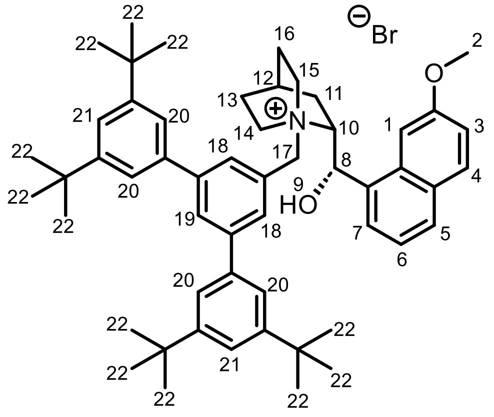

Prepared according to **GP1** on a 0.30 mmol scale with respect to (S)-(7-methoxynaphthalen-1-yl)((1S,2R,4S)-quinuclidin-2-yl)methanol and using 5'-(bromomethyl)-3,3'',5,5''-tetra-tert-butyl-1,1':3',1''-terphenyl as the benzyl bromide. Purification by flash column chromatography (SiO_2, 0-7 % v/v MeOH in CH_2Cl_2) afforded the title compound (**Cat7•Br**) as an off-white solid (231 mg, 0.273 mmol, 91% yield).

***R**$_f$* **value**: 0.29 (10% v/v MeOH in CH_2Cl_2);

^{1}H NMR (400 MHz, $CDCl_3$): δ 7.96 (br s, 2 H, H-18), 7.92-7.90 (m, 2 H, H-7, H-19), 7.80 (d, *J* = 9.0 Hz, 1 H, H-4), 7.70 (d, *J* = 8.0 Hz, 1 H, H-5), 7.53-7.51 (m, 6 H, H-20, H-21), 7.33 (dd, *J* = 8.0, 7.4 Hz, 1 H, H-6), 7.25 (d, *J* = 2.2 Hz, 1 H, H-1, overlapped with the signal of residual chloroform), 7.18 (dd, *J* = 9.0, 2.2 Hz, 1 H, H-3), 6.82 (d, *J* = 6.8 Hz, 1 H, H-8), 6.71 (d, *J* = 12.4 Hz, 1 H, H-17a), 6.65 (d, *J* = 6.9 Hz, 1 H, H-9), 5.19-5.12 (m, 1 H, H-14a), 4.39 (d, *J* = 12.4 Hz, 1 H, H-17b), 3.97 (s, 3 H, H-2), 3.72 (t, *J* = 8.7 Hz, 1 H, H-10), 3.44-3.25 (m, 3 H, H-14b, H-15), 2.56-2.51 (m, 1 H, H-11a), 2.25-2.19 (m, 1 H, H-13a), 2.13 (br s, 1 H, H-12), 1.82-1.78 (m, 2 H, H-16), 1.68-1.61 (m, 1 H, H-13b, overlapped with the signal of H_2O), 1.41 (s, 36 H, H-22), 1.36-1.30 (m, 1 H, H-11b) ppm;

^{13}C NMR (101 MHz, $CDCl_3$): δ 158.1, 151.8, 144.4, 139.6, 133.5, 131.4, 131.2, 130.6, 129.3, 129.2, 128.4, 127.8, 126.7, 123.5, 122.5, 122.1, 117.2, 102.3, 71.4, 64.8, 63.8, 57.8, 56.0, 51.1, 35.2, 31.7, 26.1, 24.11, 24.08, 20.7 ppm;

HRMS (+ESI): *m/z* For cation found 764.5408, $[C_{54}H_{70}NO_2]^+$ requires 764.5401, (δ = + 1.0 ppm);

$[\alpha]_D^{25.0}$ = + 114.2 (c = 0.57, $CHCl_3$).

Note: the synthesis of 5'-(bromomethyl)-3,3'',5,5''-tetra-tert-butyl-1,1':3',1''-terphenyl has been reported previously.[3]

Synthesis of Chiral Cation **Cat8•Br** *from* **11c**

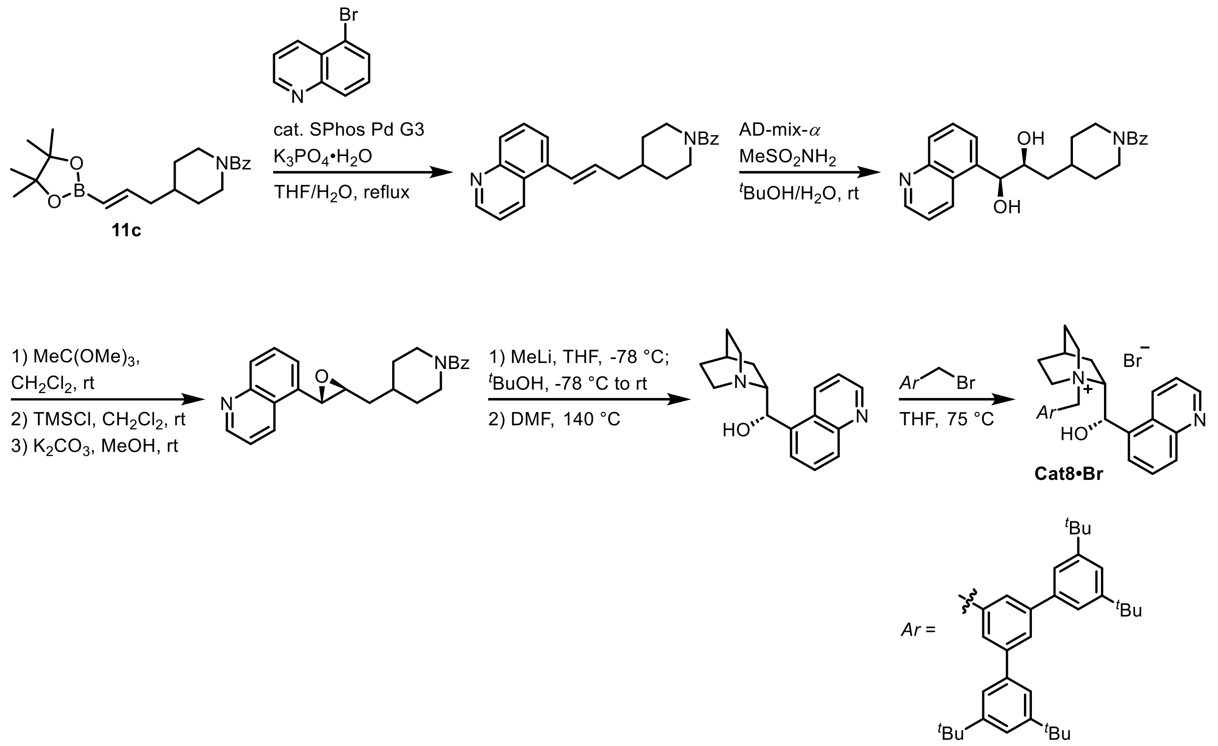

The Suzuki-Miyaura coupling of **11c** was conducted using a protocol described by Tudge, Buchwald and co-worker.[13] The remaining steps in the sequence were conducted using similar protocols described by Hatakeyama and co-workers.[14]

(E)-phenyl(4-(3-(quinolin-5-yl)allyl)piperidin-1-yl)methanone

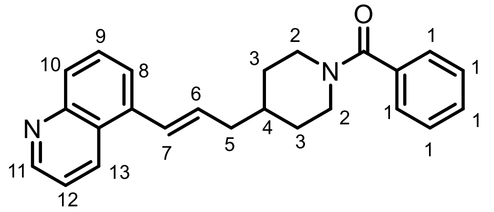

A three-necked round bottom flask was charged with Shos Pd G3(117 mg, 0.15 mmol, 2 mol %), K_3PO_4•H_2O (3.45 g, 15.0 mmol, 3.0 equiv), and the compound **11c** (2.67 g, 7.51 mmol, 1.0 equiv), and thrice evacuated and backfilled with nitrogen. After the addition of dry THF (60 mL) and H_2O (6 mL), 5-bromoquinoline (1.93 g, 9.28 mmol, 1.24 equiv) was added to the mixture. The reaction mixture was refluxed for 16 hours. After cooling to room temperature, ethyl acetate and water were added to the mixture. The aqueous layer was thrice extracted with ethyl acetate and the combined organic layer was dried over $MgSO_4$, filtered and concentrated. Purification by flash column chromatography (SiO_2, 50-100% v/v ethyl acetate in hexane) afforded the title compound as a white solid (2.33 g, 6.53 mmol, 87% yield).

***R_f* value**: 0.13 (50% v/v ethyl acetate in hexane);

^{1}H NMR (400 MHz, $CDCl_3$): δ 8.90 (d, *J* = 4.0 Hz, 1 H, H-11), 8.41 (d, *J* = 8.5 Hz, 1 H, H-13), 8.00 (d, *J* = 8.3 Hz, 1 H, H-10), 7.64 (t, *J* = 7.6 Hz, 1 H, H-9), 7.59 (d, *J* = 7.1 Hz, 1 H, H-8), 7.41-7.38 (m, 6 H, H-1, H-12), 7.04 (d, *J* = 15.5 Hz, 1 H, H-7), 6.22 (dt, *J* = 15.5, 7.7 Hz, 1 H, H-6), 4.74 (br s, 1 H, H-2a), 3.77 (br s, 1 H, H-2b), 3.03-2.74 (m, 2 H, H-2c, H-2d), 2.31 (br t, *J* = 6.6 Hz, 2 H, H-5), 1.92-1.69 (m, 3 H, H-3a, H-3b, H-4), 1.34-1.22 (m, 2 H, H-3c, H-3d) ppm;

^{13}C NMR (101 MHz, $CDCl_3$): δ 170.4, 150.3, 148.5, 136.4, 135.7, 132.7, 132.3, 129.5, 129.2, 128.9, 128.5, 127.7, 126.9, 126.2, 123.9, 120.9, 48.1*, 42.6*, 40.4, 36.6, 32.9*, 32.0* ppm;

* Observed as broad signals.

HRMS (+ESI): *m/z* found $[M+H]^+$ 357.1963, $[C_{24}H_{25}N_2O]^+$ requires 357.1961, (δ = + 0.6 ppm).

(4-((2S,3S)-2,3-dihydroxy-3-(quinolin-5-yl)propyl)piperidin-1-yl)(phenyl)methanone

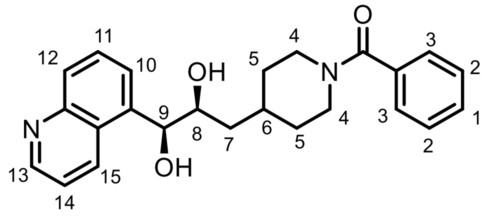

To a solution of AD-mix-*α* (10 g) and methanesulfonamide (2.38 g, 25.0 mmol, 5 equiv) in ^{t}BuOH/H_2O (30 mL, v/v 1:1), (*E*)-phenyl(4-(3-(quinolin-5-yl)allyl)piperidin-1-yl)methanone (1.79 g, 5.02 mmol, 1.0 equiv) in ^{t}BuOH (15 mL) was added at room temperature. The reaction mixture was stirred for 90 hours. After the reaction was quenched with saturated aqueous Na_2SO_3 solution (10 mL), ^{t}BuOH was removed under reduced pressure and then ethyl acetate was added to the mixture. The aqueous layer was thrice extracted with ethyl acetate and the combined organic layer was dried over $MgSO_4$, filtered and concentrated. Purification by flash column chromatography (SiO_2, 0-5% v/v MeOH in CH_2Cl_2) afforded the title compound as a colorless sticky oil (1.81 g, 4.64 mmol, 92% yield, >99% *ee*).

***R_f* value**: 0.45 (10% v/v MeOH in CH_2Cl_2);

^{1}H NMR (700 MHz, $CDCl_3$): *δ* 8.85 (dd, *J* = 4.0, 1.1 Hz, 1 H, H-13), 8.49 (d, *J* = 8.5 Hz, 1 H, H-15), 8.01 (d, *J* = 8.4 Hz, 1 H, H-12), 7.65 (t, *J* = 7.7 Hz, 1 H, H-11), 7.59 (d, *J* = 7.0 Hz, 1 H, H-10), 7.39-7.30 (m, 4 H, H-1, H-2, H-14), 7.26-7.25 (m, 2 H, H-3, overlapped with the signal of residual chloroform), 5.07 (d, *J* = 6.7 Hz, 1 H, H-9), 4.57-4.53 (m, 1 H, H-4a), 4.05-4.00 (m, 1 H, H-8), 3.65-3.61 (m, 2 H, H-4b, O*H*), 3.18-3.14 (m, 1 H, O*H*), 2.91-2.84 (m, 1 H, H-4c), 2.66-2.60 (m, 1 H, H-4d), 1.77-1.66 (m, 2 H, H-5a, H-6, overlapped with the signal of H_2O), 1.52 (br s, 2 H, H-5b, H-7a), 1.09-0.77 (m, 3 H, H-5c, H-5d, H-7b) ppm;

^{13}**C NMR** (176 MHz, $CDCl_3$): *δ* 170.4, 150.2, 148.6, 137.6, 136.2, 132.3, 129.9, 129.6, 129.1, 128.5, 126.9, 126.3, 125.6, 121.1, 75.5, 72.6, (48.2, 47.9)*, (42.6, 42.4)*, 39.8, (33.9, 32.9, 31.9, 31.0)*, 32.7 ppm;

* Observed as broad signals. 2D NMR experiments clearly showed the signals in parentheses are the same position carbons of the piperidine structure.

HRMS (+ESI): *m/z* found $[M+H]^+$ 391.2020, $[C_{24}H_{27}N_2O_3]^+$ requires 391.2016, (δ = + 1.0 ppm);

$[\alpha]_D^{25.0}$ = + 1.2 (c = 0.67, $CHCl_3$);

Chiral SFC Analysis: CHIRALPAK IG (CO_2:MeOH, 60:40, 2.5 mL min^{-1}, 40 °C) t_R = 6.73 (minor), 7.72 (major) minutes.

phenyl(4-(((2S,3S)-3-(quinolin-5-yl)oxiran-2-yl)methyl)piperidin-1-yl)methanone

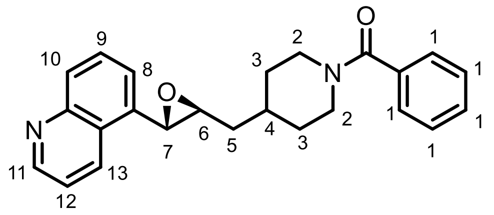

To a solution of (4-((2S,3S)-2,3-dihydroxy-3-(quinolin-5-yl)propyl)piperidin-1-yl)(phenyl)methanone (1.57 g, 4.01 mmol, 1.0 equiv) and pyridinium *p*-toluenesulfonate (101 mg, 0.4 mmol, 10 mol %) in dry CH_2Cl_2 (40 mL), trimethyl orthoacetate (2.55 mL, 20.0 mmol, 5.0 equiv) was added dropwise at room temperature under an inert atmosphere of nitrogen. The reaction mixture was stirred at room temperature for 15 hours and then concentrated under reduced pressure. The resulting oil was dissolved in dry CH_2Cl_2 (40 mL) under an inert atmosphere of nitrogen and trimethylsilyl chloride (2.54 mL, 20.0 mmol, 5.0 equiv) was added to the mixture. The reaction mixture was stirred at room temperature for 4 hours and then concentrated under reduced pressure. The resulting oil was dissolved in methanol (40 mL) and potassium carbonate (1.66 g, 12.0 mmol, 3.0 equiv) was added to the mixture. The reaction mixture was stirred at room temperature for 2 hours. After addition of saturated aqueous NH_4Cl solution (50 mL), the resulting mixture was diluted with ethyl acetate and water. The aqueous layer was thrice extracted with ethyl acetate and the combined organic layer was dried over $MgSO_4$, filtered and concentrated. Purification by flash column chromatography (SiO_2, 0-3% v/v MeOH in CH_2Cl_2) afforded the title compound as a pale-yellow sticky oil (1.25 g, 3.35 mmol, 84% yield, >99% *ee*).

***R_f* value**: 0.62 (10% v/v MeOH in CH_2Cl_2);

^{1}**H NMR** (700 MHz, $CDCl_3$): δ 8.94 (dd, *J* = 4.1, 1.3 Hz, 1 H, H-11), 8.42 (ddd, *J* = 8.5, 1.4, 0.6 Hz, 1 H, H-13), 8.05 (d, *J* = 8.5 Hz, 1 H, H-10), 7.67 (dd, *J* = 8.3, 7.3 Hz, 1 H, H-9), 7.48-7.44 (m, 2 H, H-8, H-12), 7.40-7.37 (m, 5 H, H-1), 4.74 (br s, 1 H, H-2a), 4.19 (d, *J* = 1.8 Hz, 1 H, H-7), 3.77 (br s, 1 H, H-2b), 3.03-3.02 (m, 2 H, H-2c, H-6), 2.79 (br s, 1 H, H-2d), 1.96-1.70 (m, 5 H, H-3a, H-3b, H-4, H-5), 1.41-1.27 (m, 2 H, H-3c, H-3d) ppm;

^{13}C NMR (176 MHz, $CDCl_3$): δ 170.4, 150.5, 148.2, 136.3, 133.9, 131.2, 129.7, 129.6, 129.2, 128.5, 126.9, 126.6, 123.0, 121.3, (60.8, 60.5)†, 56.3, 48.0*, 42.4*, 39.2, 34.7, (33.3, 32.8, 32.3, 32.0)† ppm;

* Observed as broad signals.

† Observed as broad signals. 2D NMR experiments clearly showed the signals in parentheses are the same position carbons of the piperidine structure.

HRMS (+ESI): *m/z* found $[M+H]^+$ 373.1916, $[C_{24}H_{25}N_2O_2]^+$ requires 373.1911, (δ = + 1.3 ppm);

$[\alpha]_D^{25.0}$ = + 17.2 (c = 0.72, $CHCl_3$);

Chiral SFC Analysis: CHIRALPAK IG (CO_2:MeOH, 60:40, 2.5 mL min^{-1}, 40 °C) t_R = 13.26 (major), 20.65 (minor) minutes.

Note on racemic dihydroxylation and subsequent epoxidation reaction performed: In order to obtain racemic SFC trace, racemic dihydroxylation was conducted on (*E*)-phenyl(4-(3-(quinolin-5-yl)allyl)piperidin-1-yl)methanone. The corresponding racemic epoxide was also prepared from the racemic *cis*-diol by the same procedure when making the enantioenriched version.

Dihydroxylation: To a solution of (*E*)-phenyl(4-(3-(quinolin-5-yl)allyl)piperidin-1-yl)methanone (366 mg, 1.03 mmol, 1.0 equiv) in ^{t}BuOH/H_2O (60 mL, v/v 5:1), a solution of $KMnO_4$ (198 mg, 1.25 mmol, 1.25 equiv) and NaOH (44 mg, 1.10 mmol, 1.1 equiv) in H_2O (40 mL) was added dropwise at 0 °C. The reaction mixture was stirred at 0 °C for 1 hour. After saturated aqueous $Na_2S_2O_5$ solution was added to the mixture until the brown color of mixture disappeared, ^{t}BuOH was removed under reduced pressure and ethyl acetate was added to the solution. The aqueous layer was thrice extracted with ethyl acetate and the combined organic layer was dried over $MgSO_4$, filtered and concentrated. Purification by flash column chromatography (SiO_2, 0-5% v/v MeOH in CH_2Cl_2) afforded the racemic *cis*-diol as a colorless sticky oil (180 mg, 0.461 mmol, 45% yield).

Epoxidation: To a solution of racemic *cis*-diol (169 mg, 0.434 mmol, 1.0 equiv) and pyridinium *p*-toluenesulfonate (10.9 mg, 0.0434 mmol, 10 mol %) in dry CH_2Cl_2 (10 mL), trimethyl orthoacetate (0.276 mL, 2.17 mmol, 5.0 equiv) was added dropwise at room temperature under an inert atmosphere of nitrogen. The reaction mixture was stirred at room temperature for 16 hours and then concentrated under reduced pressure. The resulting oil was dissolved in dry CH_2Cl_2 (10 mL) under an inert atmosphere of nitrogen and trimethylsilyl chloride (0.276 mL, 2.17 mmol, 5.0 equiv) was added to the mixture. The reaction mixture was stirred at room temperature for 4 hours and then concentrated under reduced pressure. The resulting oil was dissolved in methanol (10 mL) and K_2CO_3 (180 mg, 1.30 mmol, 3.0 equiv) was added to the mixture. The reaction mixture was stirred at room temperature for 3 hours. After addition of saturated aqueous NH_4Cl solution (20 mL), the resulting mixture was diluted with ethyl acetate and water. The aqueous layer was thrice extracted with ethyl acetate and the combined organic layer was dried over $MgSO_4$, filtered and concentrated. Purification by flash column chromatography (SiO_2, 0-

3% v/v MeOH in CH_2Cl_2) afforded the racemic epoxide as a pale-yellow sticky oil (99.4 mg, 0.267 mmol, 62% yield).

(S)-quinolin-5-yl((1S,2R,4S)-quinuclidin-2-yl)methanol

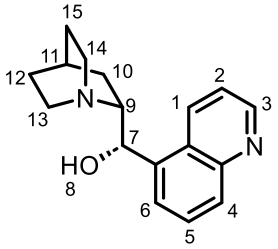

To a solution of phenyl(4-(((2S,3S)-3-(quinolin-5-yl)oxiran-2-yl)methyl)piperidin-1-yl)methanone (1.13 g, 3.04 mmol, 1.0 equiv) in dry THF (40 mL), MeLi (1.6 M Et_2O solution, 1.90 mL, 3.04 mmol, 1.0 equiv) was added dropwise at -78 °C under an inert atmosphere of nitrogen. The reaction mixture was stirred at -78 °C for 30 minutes and then ^{t}BuOH (0.87 mL, 9.12 mmol, 3.0 equiv) was added to the mixture. The resulting mixture was allowed to warm to room temperature and concentrated under reduced pressure. The resulting oil was dissolved in dry DMF (40 mL) under an inert atmosphere of nitrogen and the solution was heated at 140 °C for 17 hours. After cooling to room temperature, the solvent was removed under reduced pressure. The resulting solid was purified by flash column chromatography (SiO_2, (1) 0-10% MeOH in CH_2Cl_2, (2) 10-30% v/v 70:29:1 CH_2Cl_2/MeOH/NH_4OH (aq.) in CH_2Cl_2, (3) 10-30% v/v (2 M NH_3 in MeOH) in CH_2Cl_2) and then obtained solid was recrystallized from $CHCl_3$. After the obtained white solid was dissolved in ethyl acetate, saturated aqueous $NaHCO_3$ solution was added to the solution and the aqueous layer was thrice extracted with ethyl acetate. The combined organic layer was dried over K_2CO_3, filtered and concentrated. The title compound was afforded as a white powder (183 mg, 0.683 mmol, 22% yield).

***R_f* value**: 0.19 (30% v/v (2 M NH_3 in MeOH) in CH_2Cl_2);

^{1}H NMR (700 MHz, $CDCl_3$): δ 8.89 (dd, *J* = 4.1, 1.5 Hz, 1 H, H-3), 8.48 (d, *J* = 8.5 Hz, 1 H, H-1), 8.02 (d, *J* = 8.1 Hz, 1 H, H-4), 7.72-7.68 (m, 2 H, H-5, H-6), 7.35 (dd, *J* = 8.5, 4.1 Hz, 1 H, H-2), 5.58 (d, *J* = 5.4 Hz, 1 H, H-7), 3.37-3.32 (m, 1 H, H-13a), 3.21-3.17 (m, 1 H, H-9), 2.88-2.78 (m, 3 H, H-8, H-14), 2.71-2.67 (m, 1 H, H-13b), 1.87-1.84 (m, 1 H, H-11), 1.82-1.79 (m, 1 H, H-10a), 1.61-1.43 (m, 5 H, H-10b, H-12, H-15) ppm;

^{13}C NMR (176 MHz, $CDCl_3$): δ 150.1, 148.7, 140.0, 132.0, 129.5, 129.1, 126.0, 124.4, 120.9, 73.2, 60.7, 50.8, 43.8, 27.8, 26.8, 25.8, 22.1 ppm;

HRMS (+ESI): *m/z* found $[M+H]^+$ 269.1658, $[C_{17}H_{21}N_2O]^+$ requires 269.1648, (δ = + 3.7 ppm);

$[\alpha]_D^{25.0}$ = + 95.9 (c = 0.70, $CHCl_3$).

(1S,2R,4S)-2-((S)-hydroxy(quinolin-5-yl)methyl)-1-((3,3'',5,5''-tetra-tert-butyl-[1,1':3',1''-terphenyl]-5'-yl)methyl)quinuclidin-1-ium bromide (**Cat8•Br**)

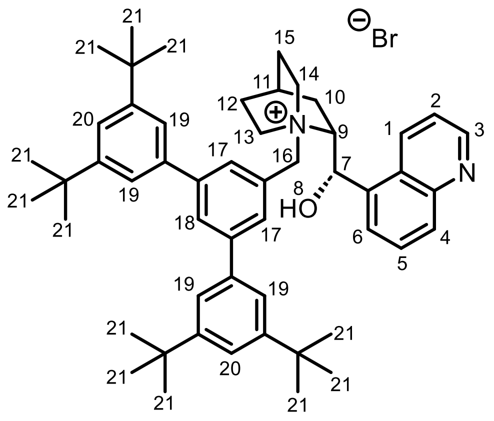

Prepared according to **GP1** on a 0.30 mmol scale with respect to (S)-quinolin-5-yl((1S,2R,4S)-quinuclidin-2-yl)methanol and using 5'-(bromomethyl)-3,3'',5,5''-tetra-tert-butyl-1,1':3',1''-terphenyl as the benzyl bromide. Purification by flash column chromatography (SiO_2, 0-8 % v/v MeOH in CH_2Cl_2) afforded the title compound (**Cat8•Br**) as an off-white solid (201 mg, 0.246 mmol, 82% yield).

***R_f* value**: 0.29 (5% v/v MeOH in CH_2Cl_2);

^{1}H NMR (700 MHz, $CDCl_3$): δ 8.63 (br d, *J* = 8.2 Hz, 1 H, H-1), 8.45 (d, *J* = 3.4 Hz, 1 H, H-3), 8.12 (br s, 2 H, H-17), 7.97 (d, *J* = 7.2 Hz, 1 H, H-6), 7.92 (d, *J* = 8.5 Hz, 1 H, H-4), 7.73 (br s, 1 H, H-18), 7.63 (t, *J* = 7.6 Hz, 1 H, H-5), 7.47 (t, *J* = 1.5 Hz, 2 H, H-20), 7.43 (br s, 4 H, H-19), 7.03 (dd, *J* = 8.5, 4.1 Hz, 1 H, H-2), 6.78 (br s, 1 H, H-7), 6.63 (d, *J* = 5.4 Hz, 1 H, H-8), 5.95 (d, *J* = 12.5 Hz, 1 H, H-16a), 5.74 (d, *J* = 12.5 Hz, 1 H, H-16b), 4.76-4.71 (m, 1 H, H-13a), 4.09-4.05 (m, 1 H, H-14a), 4.00 (t, *J* = 9.0 Hz, 1 H, H-9), 3.62-3.58 (m, 1 H, H-13b), 3.17-3.13 (m, 1 H, H-14b), 2.13-2.10 (m, 1 H, H-10a), 1.96 (br s, 1 H, H-11), 1.93-1.89 (m, 1 H, H-12a), 1.67 (t, *J* = 7.2 Hz, 2 H, H-15), 1.57-1.53 (m, 1 H, H-12b), 1.36 (s, 36 H, H-21), 1.01-0.98 (m, 1 H, H-10b) ppm;

^{13}C NMR (176 MHz, $CDCl_3$): δ 151.6, 149.8, 148.0, 143.9, 139.6, 135.5, 132.1, 131.3, 129.7, 128.7, 128.5, 128.3, 126.2, 124.6, 122.4, 122.0, 121.8, 68.0, 65.4, 62.9, 57.4, 51.9, 35.2, 31.7, 26.5, 24.5, 23.6, 20.9 ppm;

HRMS (+ESI): *m/z* For cation found 735.5249, $[C_{52}H_{67}N_2O]^+$ requires 735.5248, (δ = + 0.1 ppm);

$[\alpha]_D^{25.0}$ = + 60.0 (c = 0.69, $CHCl_3$).

Note: the synthesis of 5'-(bromomethyl)-3,3'',5,5''-tetra-tert-butyl-1,1':3',1''-terphenyl has been reported previously.[3]

Synthesis of Chiral Cation **Cat9•Br**

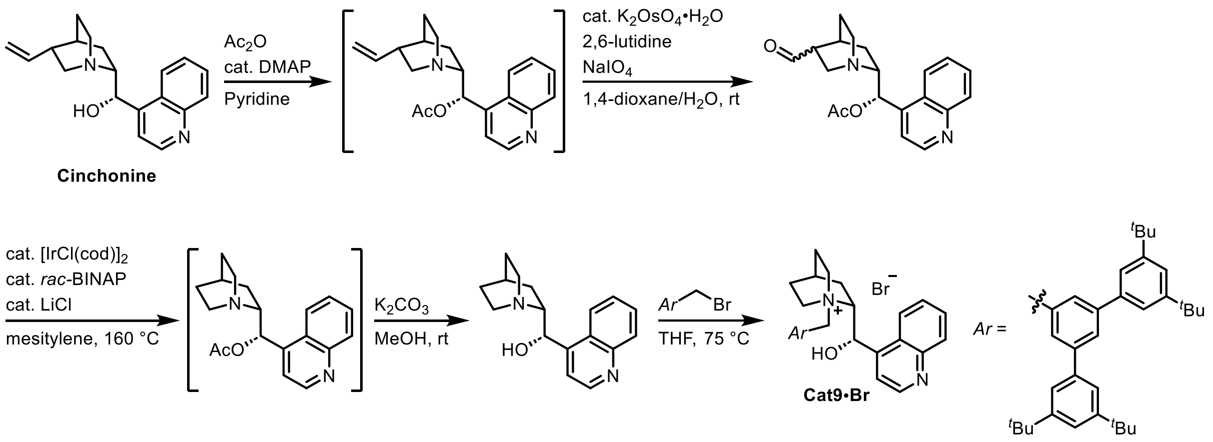

The oxidative cleavage of vinyl group was followed according to the protocol described by Jin and co-workers.[15] The Iridium-catalyzed decarbonylation was followed according to the protocol described by Madsen and co-worker.[16]

Synthesis of Chiral Cation **Cat11•Br**

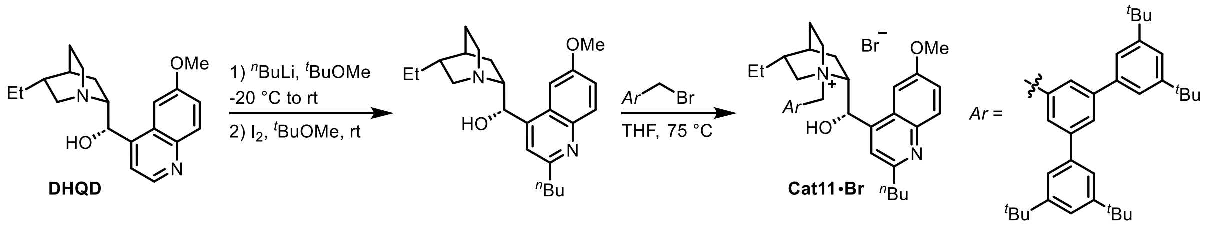

The precursor to **Cat11•Br** was prepared according to the protocol described by Hintermann and co-workers.[17]

(S)-((1S,2R,4S)-5-formylquinuclidin-2-yl)(quinolin-4-yl)methyl acetate

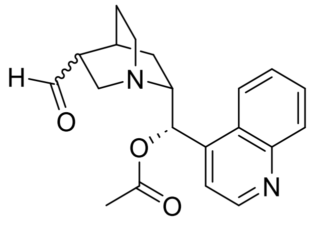

Cinchonine (2.98 g, 10.1 mmol, 1.0 equiv) was dissolved in a 1:1 mixture of Ac_2O/pyridine (20 mL). 4-Dimethylaminopyridine (224 mg, 2.0 mmol, 20 mol %) was added and the resulting mixture was stirred at room temperature for 68 hours. The mixture was evaporated to dryness in a well-ventilated fume hood under a stream of compressed nitrogen and the crude residue was dissolved in CH_2Cl_2 and washed with saturated aqueous $NaHCO_3$ solution. The aqueous layer was thrice extracted with CH_2Cl_2 and the combined organic layer was

washed with brine, dried over $MgSO_4$, filtered and concentrated. The crude residue was dissolved in 1,4-dioxane/water (100 mL, v/v 3:1) and added to K_2OsO_4•H_2O (73.7 mg, 0.20 mmol, 2 mol %) in water (2 mL), 2,6-lutidine (2.32 mL, 20.0 mmol, 2.0 equiv), and $NaIO_4$ (4.71 g, 22.0 mmol, 2.2 equiv) at room temperature. After stirring for 4 hours, the reaction mixture was diluted with ethyl acetate and water. The aqueous layer was thrice extracted with ethyl acetate and the combined organic layer was washed with brine, dried over $MgSO_4$, filtered and concentrated. The residue was purified by flash column chromatography (SiO_2, 0-60% v/v 70:29:1 CH_2Cl_2/MeOH/NH_4OH (aq.) in CH_2Cl_2) and then obtained solid was dissolved in ethyl acetate. The saturated aqueous $NaHCO_3$ solution was added to the solution and the aqueous layer was thrice extracted with ethyl acetate. The combined organic layer was dried over K_2CO_3, filtered and concentrated. The title compound was afforded as a brown foamy solid and as an inconsequential mixture of diastereomers (1.57 g, 4.63 mmol, 46%).

Note: This product still contained traces of dichloromethane due to the difficulty in drying the sticky oil.

***R$_f$* value**: 0.23 (20% v/v 70:29:1 CH_2Cl_2/MeOH/NH_4OH (aq.) in CH_2Cl_2);

^{1}H NMR (700 MHz, $CDCl_3$): δ 9.86-9.79 (m, 1 H), 8.89-8.86 (m, 1 H), 8.22-8.17 (m, 1 H), 8.14-8.11 (m, 1 H), 7.73-7.71 (m, 1 H), 7.62-7.58 (m, 1 H), 7.37-7.33 (m, 1 H), 6.57-6.54 (m, 1 H), 3.65-3.39 (m, 1 H), 3.25-3.22 (m, 1 H), 3.15-2.84 (m, 1 H), 2.77-2.69 (m, 2 H), 2.62-2.43 (m, 2 H), 2.14-2.13 (m, 3 H), 1.65-1.36 (m, 4 H) ppm;

Note: Unassigned in order to avoid confusion between the diastereomeric protons within each diastereomer.

^{13}C NMR (176 MHz, $CDCl_3$): δ 203.7, 203.5, 170.0, 169.9, 150.1, 148.8, 148.7, 145.4, 145.1, 130.7, 130.6, 129.40, 129.36, 127.1, 126.0, 123.4, 123.3, 118.6, 118.3, 74.2, 74.0, 59.3, 59.1, 50.6, 50.2, 49.6, 49.1, 42.6, 42.3, 29.6, 26.1, 24.6, 23.7, 23.6, 21.6, 21.24, 21.15 ppm;

HRMS (+ESI): *m/z* found $[M+H]^+$ 339.1706, $[C_{20}H_{22}N_2O_3]^+$ requires 339.1703, (δ = + 0.9 ppm);

$[\alpha]_D^{25.0}$ = + 110.9 (c = 0.73, MeOH).

(S)-quinolin-4-yl((1S,2R,4S)-quinuclidin-2-yl)methanol

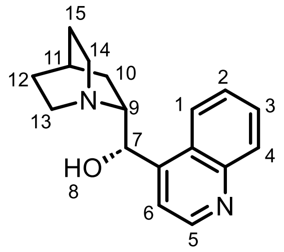

A three-necked round bottom flask was charged with (*S*)-((*1S,2R,4S*)-5-formylquinuclidin-2-yl)(quinolin-4-yl)methyl acetate (1.03 g, 3.06 mmol, 1 equiv), $[IrCl(cod)]_2$ (134 mg, 0.20 mmol, 6.5 mol %), *rac*-BINAP (249 mg, 0.40 mmol, 13 mol %), LiCl (33.9 mg, 0.80 mmol, 0.26 equiv) and thrice evacuated and backfilled with nitrogen. After the addition of mesitylene (15 mL, saturated with water), the reaction mixture was heated at 165 °C for 17

hours. After cooling to room temperature, the solvent was removed under reduced pressure. The crude was purified by flash column chromatography (SiO_2, 0-60% v/v 70:29:1 CH_2Cl_2/MeOH/NH_4OH (aq.) in CH_2Cl_2) to remove the unreacted aldehyde. The obtained solid was dissolved in MeOH (40 mL) and K_2CO_3 (2.11 g, 15.3 mmol, 5.0 equiv) was added to the solution at room temperature. After the reaction mixture was stirred for 4 hours, the solvent was removed under reduced pressure. The residue was dissolved in CH_2Cl_2 and added to a water. The aqueous layer was thrice extracted with CH_2Cl_2 and the combined organic layer was dried over $MgSO_4$, filtered and concentrated. The crude was purified by flash column chromatography (SiO_2, 0-24% v/v (2 M NH_3 in MeOH) in CH_2Cl_2) and the obtained solid was recrystallized from ethyl acetate. After the obtained white solid was dissolved in ethyl acetate, saturated aqueous $NaHCO_3$ solution was added to the solution and the aqueous layer was thrice extracted with ethyl acetate. The combined organic layer was dried over K_2CO_3, filtered and concentrated. The title compound was afforded as a white powder (204 mg, 0.761 mmol, 25% yield).

***R_f* value**: 0.54 (30% v/v (2 M NH_3 in MeOH) in CH_2Cl_2);

^{1}H NMR (400 MHz, $CDCl_3$): δ 8.80 (d, *J* = 4.4 Hz, 1 H, H-5), 8.07 (d, *J* = 8.5 Hz, 1 H, H-4), 7.87 (d, *J* = 8.4 Hz, 1 H, H-1), 7.62-7.59 (m, 2 H, H-3, H-6), 7.27-7.23 (m, 1 H, H-2, overlapped with the signal of residual chloroform), 5.69 (d, *J* = 3.6 Hz, 1 H, H-7), 4.93 (br s, 1 H, H-8), 3.56-3.46 (m, 1 H, H-13a), 3.10-3.05 (m, 1 H, H-9), 2.86-2.61 (m, 3 H, H-13b, H-14), 1.86-1.80 (m, 2 H, H-10a, H-11), 1.62-1.56 (m, 1 H, H-12a), 1.45-1.33 (m, 3 H, H-12b, H-15), 1.28-1.22 (m, 1 H, H-10b) ppm;

^{13}C NMR (101 MHz, $CDCl_3$): δ 150.2, 149.7, 148.2, 130.3, 129.1, 126.6, 125.8, 123.1, 118.4, 72.0, 60.2, 50.9, 44.1, 26.6, 26.2, 25.7, 22.1 ppm;

HRMS (+ESI): *m/z* found $[M+H]^+$ 269.1651, $[C_{17}H_{21}N_2O]^+$ requires 269.1648, (δ = + 1.1 ppm);

$[\alpha]_D^{25.0}$ = + 131.0 (c = 0.40, $CHCl_3$).

(1S,2R,4S)-2-((S)-hydroxy(quinolin-4-yl)methyl)-1-((3,3'',5,5''-tetra-tert-butyl-[1,1':3',1''-terphenyl]-5'-yl)methyl)quinuclidin-1-ium bromide (**Cat9•Br**)

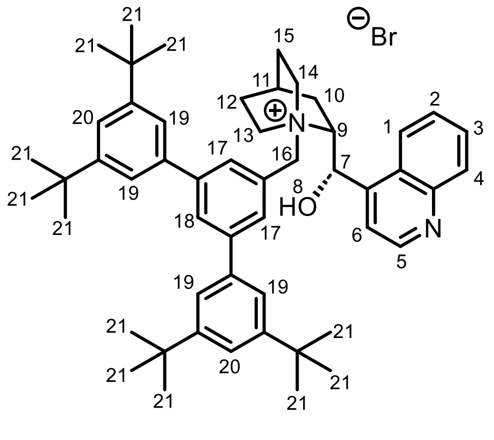

Prepared according to **GP1** on a 0.30 mmol scale with respect to (S)-quinolin-4-yl((1S,2R,4S)-quinuclidin-2-yl)methanol and using 5'-(bromomethyl)-3,3'',5,5''-tetra-tert-butyl-1,1':3',1''-terphenyl as the benzyl bromide. Purification by flash column chromatography (SiO_2, 0-8.5 % v/v MeOH in CH_2Cl_2) afforded the title compound (**Cat9•Br**) as an off-white solid (201 mg, 0.246 mmol, 82% yield).

***R**f* **value**: 0.21 (5% v/v Methanol in CH_2Cl_2);

^{1}H NMR (700 MHz, $CDCl_3$): δ 8.87 (d, *J* = 4.4 Hz, 1 H, H-5), 8.12 (br s, 2 H, H-17), 8.02 (br d, *J* = 8.1 Hz, 1 H, H-1), 7.89 (d, *J* = 8.3 Hz, 1 H, H-4), 7.86 (d, *J* = 4.4 Hz, 1 H, H-6), 7.78 (br s, 1 H, H-18), 7.49 (t, *J* = 1.5 Hz, 2 H, H-20), 7.46 (d, *J* = 1.5 Hz, 4 H, H-19), 7.32 (t, *J* = 7.7 Hz, 1 H, H-3), 7.27-7.24 (m, 1 H, H-2, overlapped with the signal of residual chloroform), 6.90 (br d, *J* = 5.9 Hz, 1 H, H-8), 6.83-6.82 (m, 1 H, H-7), 5.96 (d, *J* = 12.4 Hz, 1 H, H-16a), 5.70 (d, *J* = 12.4 Hz, 1 H, H-16b), 4.82-4.78 (m, 1 H, H-13a), 4.00 (t, *J* = 9.0 Hz, 1 H, H-9), 3.93-3.89 (m, 1 H, H-14a), 3.61-3.57 (m, 1 H, H-13b), 3.30-3.26 (m, 1 H, H-14b), 2.14-2.11 (m, 1 H, H-10a), 1.99-1.94 (m, 2 H, H-11, H-12a), 1.74-1.68 (m, 2 H, H-15, overlapped with the signal of H_2O), 1.60-1.56 (m, 1 H, H-12b), 1,37 (s, 36 H, H-21), 1.05-1.02 (m, 1 H, H-10b) ppm;

^{13}C NMR (176 MHz, $CDCl_3$): δ 151.7, 150.1, 147.9, 145.1, 144.1, 139.6, 131.4, 130.2, 129.0, 128.6, 128.3, 127.6, 124.5, 123.1, 122.4, 122.0, 120.4, 68.1, 64.8, 63.4, 57.7, 52.1, 35.2, 31.7, 26.3, 24.4, 23.7, 20.7 ppm;

HRMS (+ESI): *m/z* For cation found 735.5249, $[C_{52}H_{67}N_2O]^+$ requires 735.5248, (δ = + 0.1 ppm);

$[\alpha]_D^{25.0}$ = + 73.2 (c = 0.45, $CHCl_3$).

Note: the synthesis of 5'-(bromomethyl)-3,3'',5,5''-tetra-tert-butyl-1,1':3',1''-terphenyl has been reported previously.[3]

(S)-(2-butyl-6-methoxyquinolin-4-yl)((1S,2R,4S,5R)-5-ethylquinuclidin-2-yl)methanol

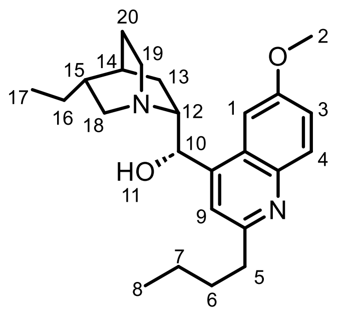

To a solution of Dihydroquinidine (DHQD, 1.64 g, 5.01 mmol, 1.0 equiv) in dry ^{t}BuOMe (30 mL) at -20 °C, ^{n}BuLi (1.6 M hexane solution, 9.40 mL, 15.0 mmol, 3.0 equiv) was added dropwise under an inert atmosphere of nitrogen and the reaction mixture was allowed to warm to room temperature and stirred for 1.5 hours. After the reaction mixture was quenched with AcOH (1.5 mL), solid iodine was added to the mixture until remaining the iodine color (ca. 570 mg, 2.25 mmol). The excess amount of iodine was quenched with saturated aqueous

$Na_2S_2O_5$ solution and then the organic layer was collected. The aqueous layer was thrice extracted with CH_2Cl_2 and the combined organic layer was dried over $MgSO_4$, filtered and concentrated. Purification by flash column chromatography (SiO_2, 0-10% v/v (2 M NH_3 in MeOH) in CH_2Cl_2) and recrystallization from hexane afforded the title compound as a white solid (270 mg, 0.706 mmol, 14% yield).

***R$_f$* value**: 0.41 (10% v/v (2 M NH_3 in MeOH) in CH_2Cl_2);

^{1}H NMR (400 MHz, $CDCl_3$): δ 7.90 (d, *J* = 9.2 Hz, 1 H, H-4), 7.42 (s, 1 H, H-9), 7.25 (dd, *J* = 9.2, 2.7 H, 1 H, H-3, overlapped with the signal of residual chloroform), 7.11 (d, *J* = 2.7 Hz, 1 H, H-1), 5.54 (d, *J* = 3.8 Hz, 1 H, H-10), 4.43 (br s, 1 H, H-11), 3.84 (s, 3 H, H-2), 3.07-2.97 (m, 2 H, H-12, H-18a), 2.91-2.68 (m, 5 H, H-5, H-18b, H-19), 1.95-1.89 (m, 1 H, H-13a), 1.68-1.60 (m, 3 H, H-6, H-14), 1.51-1.25 (m, 7 H, H-7, H-15, H-16, H-20), 1.09-1.02 (m, 1 H, H-13b), 0.88-0.84 (m, 6 H, H-8, H-17) ppm;

^{13}C NMR (101 MHz, $CDCl_3$): δ 160.2, 157.1, 148.2, 143.9, 130.9, 125.0, 121.2, 118.7, 101.5, 72.0, 60.0, 55.6, 51.5, 50.5, 38.9, 37.6, 32.4, 27.4, 26.6, 25.3, 22.8, 20.9, 14.1, 12.1 ppm;

HRMS (+ESI): *m/z* found $[M+H]^+$ 383.2693, $[C_{24}H_{35}N_2O_2]^+$ requires 383.2693, (δ = ± 0.0 ppm);

$[\alpha]_D^{25.0}$ = + 141.7 (c = 0.57, $CHCl_3$).

(1S,2R,4S,5R)-2-((S)-(2-butyl-6-methoxyquinolin-4-yl)(hydroxy)methyl)-5-ethyl-1-((3,3'',5,5''-tetra-tert-butyl-[1,1':3',1''-terphenyl]-5'-yl)methyl)quinuclidin-1-ium bromide (**Cat11•Br**)

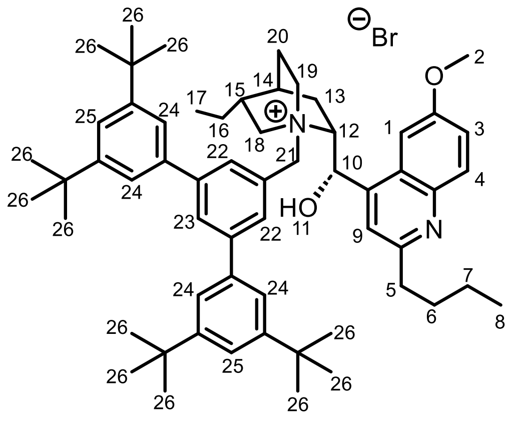

Prepared according to **GP1** on a 0.30 mmol scale with respect to (S)-(2-butyl-6-methoxyquinolin-4-yl)((1S,2R,4S,5R)-5-ethylquinuclidin-2-yl)methanol and using 5'-(bromomethyl)-3,3'',5,5''-tetra-tert-butyl-1,1':3',1''-terphenyl as the benzyl bromide. Purification by flash column chromatography (SiO_2, 0-7 % v/v MeOH in CH_2Cl_2) afforded the title compound (**Cat11•Br**) as an off-white solid (200 mg, 0.215 mmol, 72% yield).

***R$_f$* value**: 0.43 (5% v/v MeOH in CH_2Cl_2);

^{1}H NMR (700 MHz, $CDCl_3$): δ 8.01 (d, *J* = 9.2 Hz, 1 H, H-4), 7.90 (br s, 2 H, H-22), 7.87 (br t, *J* = 1.4 Hz, 1 H, H-23), 7.76 (br s, 1 H, H-9), 7.53 (t, *J* = 1.7 Hz, 2 H, H-25), 7.48 (d, *J* = 1.7 Hz, 4 H, H-24), 7.35 (dd, *J* = 9.3, 2.6 Hz, 1 H, H-

H-3), 7.21 (br d, J = 1.8 Hz, 1 H, H-1), 6.95 (br s, 1 H, H-11), 6.70 (br s, 1 H, H-10), 6.47 (br d, J = 11.4 Hz, 1 H, H-21a), 4.65 (d, J = 12.2 Hz, 1 H, H-21b), 4.54-4.50 (m, 1 H, H-18a), 3.93 (s, 3 H, H-2), 3.71-3.67 (m, 2 H, H-12, H-18b), 3.52-3.48 (m, 1 H, H-19a), 3.20-3.16 (m, 1 H, H-19b), 2.93-2.91 (m, 2 H, H-5), 2.57-2.54 (m, 1 H, H-13a), 1.96 (br s, 1 H, H-14), 1.82-1.60 (m, 7 H, H-6, H-15, H-16, H-20, overlapped with the signal of H_2O), 1.43-1.39 (m, 38 H, H-7, H-26), 1.10-1.06 (m, 1 H, H-13b), 0.96 (t, J = 7.4 Hz, 3 H, H-8), 0.92 (t, J = 7.2 Hz, 3 H, H-17) ppm;

^{13}C NMR (176 MHz, $CDCl_3$): δ 160.8, 157.5, 151.8, 144.4, 144.1, 143.3, 139.6, 131.8, 131.3, 129.2, 127.7, 124.2, 122.5, 122.1, 121.0, 120.1, 102.3, 69.9, 64.6, 64.4, 57.8, 56.1, 56.0, 39.0, 36.4, 35.2, 32.3, 31.7, 25.0, 24.7, 24.4, 22.8, 21.3, 14.1, 11.7 ppm;

HRMS (+ESI): *m/z* For cation found 849.6296, $[C_{59}H_{81}N_2O_2]^+$ requires 849.6293, (δ = + 0.4 ppm);

$[\alpha]_D^{25.0}$ = + 99.4 (c = 0.57, $CHCl_3$).

Note: the synthesis of 5'-(bromomethyl)-3,3'',5,5''-tetra-tert-butyl-1,1':3',1''-terphenyl has been reported previously.[3]

(1S,2R,4S,5R)-5-ethyl-2-((S)-methoxy(6-methoxyquinolin-4-yl)methyl)-1-((3,3'',5,5''-tetra-tert-butyl-[1,1':3',1''-terphenyl]-5'-yl)methyl)quinuclidin-1-ium bromide (**Cat12•Br**)

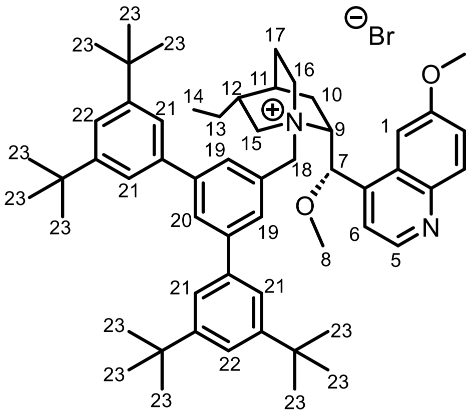

Prepared according to **GP1** on a 0.25 mmol scale with respect to (1S,2R,4S,5R)-5-ethyl-2-((S)-methoxy(6-methoxyquinolin-4-yl)methyl)quinuclidine* and using 5'-(bromomethyl)-3,3'',5,5''-tetra-tert-butyl-1,1':3',1''-terphenyl as the benzyl bromide. Purification by flash column chromatography (SiO_2, 0-5 % v/v MeOH in CH_2Cl_2) afforded the title compound (**Cat12•Br**) as an off-white solid (114 mg, 0.129 mmol, 51% yield).

** This compound was obtained as a gift from Dr Kieran J. Paterson for which we are very grateful. The synthetic route and full characterization data for this compound have been reported previously.*[18]

***R_f* value**: 0.30 (5% v/v Methanol in CH_2Cl_2);

^{1}H NMR (700 MHz, DMSO-d_6): δ 8.86 (d, J = 4.4 Hz, 1 H, H-5), 8.06 (d, J = 9.1 Hz, 1 H, H-4), 7.93-7.92 (m, 1 H, H-20), 7.89 (br s, 2 H, H-19), 7.68 (d, J = 4.4 Hz, 1 H, H-6), 7.54 (dd, J = 9.1, 2.6 Hz, 1 H, H-3), 7.51-7.50 (m, 2 H, H-

22), 7.49-7.48 (m, 5 H, H-1, H-21), 6.27 (br s, 1 H, H-7), 4.97 (d, *J* = 12.5 Hz, 1 H, H-18a), 4.86 (d, *J* = 12.5 Hz, 1 H, H-18b), 4.11 (s, 3 H, H-2), 4.06-4.03 (m, 1 H, H-16a), 3.91-3.88 (m, 1 H, H-15a), 3.85-3.79 (m, 2 H, H-9, H-15b), 3.49 (s, 3 H, H-8), 3.01-2.96 (m, 1 H, H-16b), 2.49-2.45 (m, 1 H, H-10a, overlapped with the signal of residual DMSO), 1.94-1.89 (m, 2 H, H-11, H-12), 1.76-1.69 (m, 2 H, H-17), 1.62-1.49 (m, 2 H, H-13), 1.38 (s, 36 H, H-23), 1.27-1.23 (m, 1 H, H-10b), 0.92 (t, *J* = 7.3 Hz, 3 H, H-14) ppm;

^{13}C NMR (176 MHz, DMSO-d_6): δ 157.5, 151.0, 147.5, 144.2, 143.2, 139.4, 139.1, 131.5, 131.1, 128.9, 127.9, 126.3, 121.8†, 121.7, 120.0*, 102.3, 74.4*, 67.8, 63.8, 56.7, 56.6, 56.0, 55.4, 34.7, 34.6, 31.3, 24.6, 24.2, 23.6, 20.6, 11.2 ppm;

* Observed as broad signals.

† There is an additional ^{13}C resonance underneath this peak.

HRMS (+ESI): *m/z* For cation found 807.5829, $[C_{56}H_{75}N_2O_2]^+$ requires 807.5823, (δ = + 0.7 ppm);

$[\alpha]_D^{25.0}$ = + 105.0 (c = 0.39, $CHCl_3$).

Note: the synthesis of 5'-(bromomethyl)-3,3'',5,5''-tetra-tert-butyl-1,1':3',1''-terphenyl has been reported previously.[3]

Synthesis of Achiral Rh(II,II) Tetracarboxylate Dimers

The syntheses of $Rh_2(\mathbf{A}\text{-}\mathbf{I})_2{\bullet}(NBu_4)_2$, $Rh_2(\mathbf{B}\text{-}\mathbf{I})_2{\bullet}(NBu_4)_2$, $Rh_2(\mathbf{C}\text{-}\mathbf{I})_2{\bullet}(NBu_4)_2$ and $Rh_2(\mathbf{D}\text{-}\mathbf{I})_2{\bullet}(NBu_4)_2$ has been reported previously.[6]

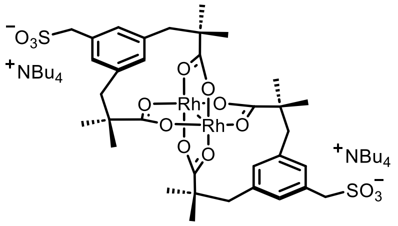

$Rh_2(\mathbf{A}\text{-}\mathbf{I})_2{\bullet}(NBu_4)_2$

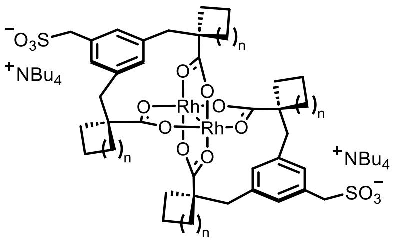

n = 1: $Rh_2(\mathbf{B}\text{-}\mathbf{I})_2{\bullet}(NBu_4)_2$
n = 2: $Rh_2(\mathbf{C}\text{-}\mathbf{I})_2{\bullet}(NBu_4)_2$
n = 3: $Rh_2(\mathbf{D}\text{-}\mathbf{I})_2{\bullet}(NBu_4)_2$

Dimethyl 5-(phenoxysulfonyl)isophthalate

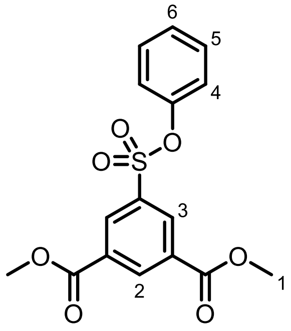

To a solution of dimethyl 5-sulfoisophthalate sodium salt (10.0 g, 33.8 mmol) in MeCN (80 mL) was added $POCl_3$ (6.3 mL, 67.5 mmol) and the white suspension heated to 55 °C for 16 hours. The reaction mixture was then allowed to cool to room temperature following which the volatiles were removed under reduced pressure. H_2O (100 mL) was added and the intermediate sulfonyl chloride was extracted with CH_2Cl_2. The combined organics were dried ($MgSO_4$) and the solvent removed under reduced pressure. In a separate round-bottomed flask a solution of PhOH (3.2 g, 33.8 mmol) and Et_3N (5.2 mL, 37.2 mmol) in CH_2Cl_2 (50 mL) was cooled to 0 °C following which a solution of the crude sulfonyl chloride prepared above in CH_2Cl_2 (20 mL), was added dropwise over 30 minutes. Transfer of the sulfonyl chloride was made quantitative with CH_2Cl_2 (10 mL). The mixture was slowly allowed to warm to room temperature and stirred for a further 36 hours. Following this the reaction was re-cooled to 0 °C and quenched with H_2O (25 mL) and 3 M HCl (aq) (25 mL). The aqueous layer was extracted with CH_2Cl_2 and the combined organic extracts dried ($MgSO_4$) and the solvent removed under reduced pressure. Recrystallisation from hot MeOH afforded the title compound as white needles (9.0 g, 25.7 mmol, 76%).

R_f value = 0.26 (20% v/v EtOAc in Hexane);

^{1}H NMR (400 MHz, $CDCl_3$) δ 8.93 (t, J = 1.5 Hz, 1H, H-2), 8.65 (d, J = 1.5 Hz, 2H, H-3), 7.33-7.24 (m, 3H, H-5, H-6), 7.00 (m, 2H, H-4), 3.98 (s, 6H, H-1) ppm;

^{13}C NMR (101 MHz, $CDCl_3$) δ 164.4, 149.4, 137.1, 135.8, 133.2, 132.2, 130.1, 127.7, 122.3, 53.1 ppm;

The spectroscopic data is in agreement with that reported in the literature.[19]

Phenyl 3,5-bis(hydroxymethyl)benzenesulfonate

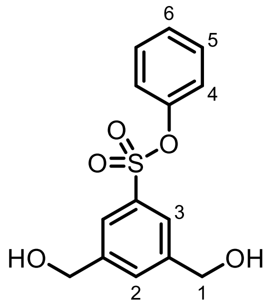

We *are grateful to Dr Kieran J. Paterson for the preparation of this compound.*

To a suspension of lithium borohydride (1.49 g, 68.5 mmol, 6.00 equiv.) in Et_2O (80 ml) was added dimethyl 5-(phenoxysulfonyl)isophthalate (4.00 g, 11.4 mmol, 1.00 equiv.) followed by MeOH (2.80 ml, 68.5 mmol, 6.00 equiv.), and the resulting solution stirred at reflux for 24 hours. Upon completion the reaction was cooled to room temperature, diluted with CH_2Cl_2, and quenched with 2.5 M NaOH. The layers were then separated, the aqueous layer extracted with CH_2Cl_2, and the combined organic extracts dried over $MgSO_4$, filtered, concentrated under reduced pressure, and the product purified by flash column chromatography (100% EtOAc) to afford the title compound as a white solid (2.39 g, 8.12 mmol, 71%).

^{1}H NMR (400 MHz, $CDCl_3$): δ 7.70 (br. s, 2H, H-3), 7.65 (br. s, 1H, H-2), 7.32-7.22 (m, 3H, H-5, H-6), 6.99 (m, 2H, H-4), 4.73 (s, 4H, H-1) ppm;

^{13}C NMR (101 MHz, $CDCl_3$): δ 149.6, 143.0, 135.9, 130.5, 129.8, 127.4, 125.5, 122.5, 64.1 ppm;

HRMS (+ESI) *m/z* found $[M+Na]^+$ 317.0457, $[C_{14}H_{14}NaO_5S]^+$ requires 317.0454 (δ = + 0.9 ppm);

The spectroscopic data is in agreement with that reported in the literature.[19]

Phenyl 3,5-bis(bromomethyl)benzenesulfonate

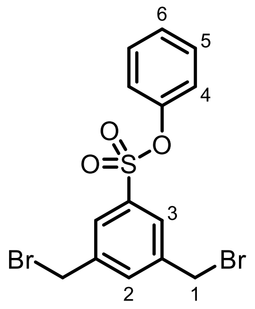

We *are grateful to Dr Kieran J. Paterson for the preparation of this compound.*

To a solution of phenyl 3,5-bis(hydroxymethyl)benzenesulfonate (2.00 g, 6.80 mmol, 1.00 equiv.) in THF (45 mL) was added tetrabromomethane (5.60 g, 17.0 mmol, 2.50 equiv.). Triphenylphosphine (4.50 g, 17.0 mmol, 2.50 equiv.) was then added portion-wise, and the resulting mixture stirred at room temperature for 2 hours. Upon

completion the volatiles were removed *in vacuo*, and the resulting residue purified by flash column chromatography (SiO_2, 60:40 Hexane:CH_2Cl_2) followed by recrystallisation from EtOH to afford the title compound as a white solid (1.60 g, 56%)

***R$_f$* value**: 0.79 (40% v/v EtOAc in hexane);

^{1}H NMR (400 MHz, $CDCl_3$): *δ* 7.75 (d, *J* = 1.7 Hz, 2H, H-3), 7.69 (t, *J* = 1.7 Hz, 1H, H-2), 7.35-7.25 (m, 3H, H-5, H-6), 6.98 (m, 2H, H-4), 4.45 (s, 4H, H-1) ppm;

^{13}C NMR (101 MHz, $CDCl_3$): *δ* 149.5, 140.3, 136.7, 135.1, 130.0, 128.7, 127.6, 122.5, 30.8 ppm;

HRMS (ESI) *this compound did not ionise.*

Dimethyl 3,3'-(5-(phenoxysulfonyl)-1,3-phenylene)bis(2,2-dimethylpropanoate)

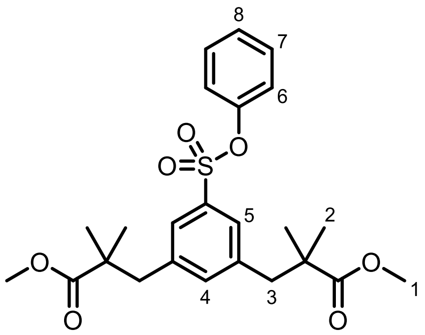

To a solution of *i*Pr_2NH (1.1 mL, 7.7 mmol) in THF (8 mL) was added n-BuLi (4.8 mL of a 1.6 M solution in Hexanes, 7.7 mmol) at 0 °C dropwise. The solution was stirred at this temperature for 5 minutes following which methyl isobutyrate (0.87 mL, 7.7 mmol) was added dropwise. The above solution was then added dropwise to a solution of phenyl 3,5-bis(bromomethyl)benzenesulfonate (1.61 g, 3.8 mmol) in THF (10 mL) at 0 °C. The reaction mixture was stirred at 0 °C for 10 minutes and then quenched by the addition of saturated aqueous NH_4Cl (10 mL) and H_2O (5 mL). The volatiles were removed under reduced pressure and the product was extracted with EtOAc (4 × 20 mL). The combined organic extracts were dried ($MgSO_4$) and the solvent removed under reduced pressure. Purification by flash column chromatography (SiO_2, 0-16% v/v EtOAc in Hexane) afforded the title compound as a yellow oil (1.35 g, 2.9 mmol, 76%).

R$_f$ value: 0.36 (20% v/v EtOAc in Hexane);

^{1}H NMR (400 MHz, CD_3OD): *δ* 7.40 (d, *J* = 1.6 Hz, 2H, H-5), 7.33-7.24 (m, 4H, H-4, H-7, H-8), 6.94-6.90 (m, 2H, H-6), 3.63 (s, 6H, H-1), 2.90 (s, 4H, H-3), 1.11 (s, 12H, H-2) ppm;

^{13}C NMR (101 MHz, CD_3OD): *δ* 178.7, 151.1, 141.1, 139.2, 136.0, 130.9, 129.0, 128.5, 123.4, 52.4, 46.5, 44.9, 25.4 ppm;

HRMS (+ESI) *m/z* found $[M+Na]^+$ 485.1601, $[C_{24}H_{30}NaO_7S]^+$ requires 485.1604, (*δ* = – 0.6 ppm).

Tetrabutylammonium 3,5-bis(2-carboxy-2-methylpropyl)benzenesulfonate

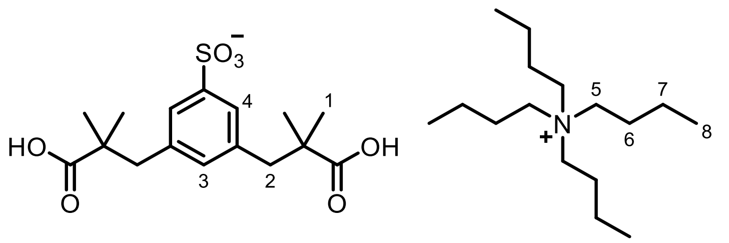

A round-bottomed flask fitted with a condenser was charged with dimethyl 3,3'-(5-(phenoxysulfonyl)-1,3-phenylene)bis(2,2-dimethylpropanoate) (673 mg, 1.5 mmol) and freshly ground NaOH (406 mg, 10.2 mmol). The apparatus was evacuated and backfilled with argon three times. A 1:1 mixture of MeOH/H_2O (15 mL) was added and the reaction mixture stirred at 50 °C overnight. The MeOH was removed under reduced pressure and the pH of the remaining aqueous solution was adjusted to pH 7 through the addition of aqueous 3 M HCl. The aqueous layer was then washed with ether to remove the phenol by-product. The aqueous layer was reserved and following this, Bu_4NHSO_4 (345 mg, 1.0 mmol) was added along with a 3:1 mixture of $CHCl_3$/*i*PrOH (15 mL). The biphasic mixture was shaken vigorously in a separating funnel and the layers separated. The aqueous layer was extracted twice more with a 3:1 mixture $CHCl_3$/*i*PrOH (2 x 15 mL). The organic extracts were then washed with H_2O to remove excess tetrabutylammonium (checked by ^1H NMR). The organic fractions were then combined, dried ($MgSO_4$) and the solvent removed under reduced pressure to afford the title compound as an off white amorphous solid (326 mg, 0.54 mmol, 37%).

^{1}H NMR (400 MHz, CD_3OD): δ 7.55 (d, J = 1.6 Hz, 2H, H-4), 7.11 (br. s, 1H, H-3), 3.26-3.21 (m, 8H, H-5), 2.88 (s, 4H, H-2), 1.70-1.62 (m, 8H, H-6), 1.42 (sext, J = 7.5 Hz, 8H, H-7), 1.16 (s, 12H, H-1), 1.03 (t, J = 7.3 Hz, 12H, H-8) ppm;

^{13}C NMR (101 MHz, CD_3OD): δ 181.2, 145.7, 139.4, 135.1, 127.0, 59.5 (ap. t, $J_{C\text{-}N}$ = 2.9 Hz), 46.9, 44.4, 25.5, 24.8, 20.7 (ap. t, $J_{C\text{-}N}$ = 1.5 Hz), 13.9 ppm;

HRMS (+ESI) *m/z* for cation found 242.2840, $[C_{16}H_{36}N]^+$ requires 242.2842, (δ = − 0.7 ppm);

HRMS (−ESI) *m/z* for anion found 357.1013, $[C_{16}H_{21}O_7S]^-$ requires 357.1013, (δ = + 0.0 ppm).

Bis[rhodium tetrabutylammonium 3,5-bis(2-carboxy-2-methylpropyl)benzenesulfonate] (Rh_2(**A-II**)$_2$•(**NBu$_4$**)$_2$)

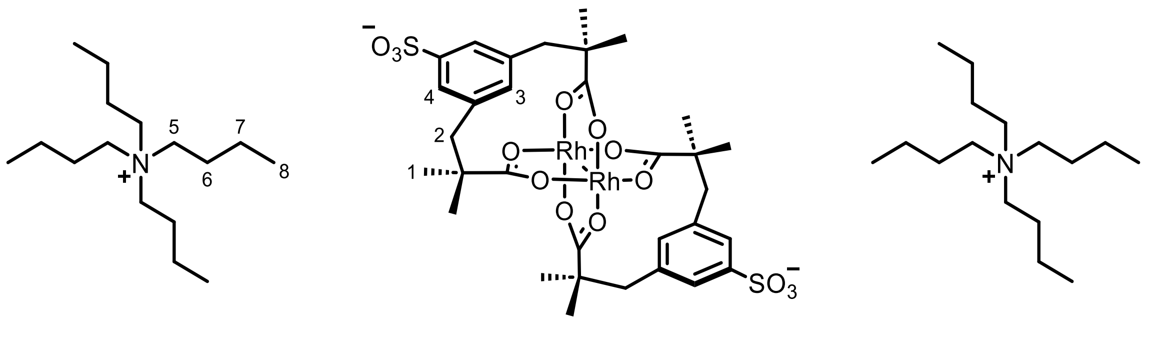

Prepared according to **GP2** on a 0.26 mmol scale with respect to $Rh_2(TFA)_4$ and using tetrabutylammonium 3,5-bis(2-carboxy-2-methylpropyl)benzenesulfonate as the ligand. The title compound was obtained as a green solid (153 mg, 0.11 mmol, 42%).

^{1}H NMR (400 MHz, CD_3OD): δ 7.32 (d, J = 1.6 Hz, 4H, H-4), 7.08 (t, J = 1.6 Hz, 2H, H-3), 3.25-3.21 (m, 16H, H-5), 2.66 (s, 8H, H-2), 1.70-1.62 (m, 16H, H-6), 1.41 (quint, J = 7.4 Hz, 16H, H-7), 1.02 (t, J = 7.3 Hz, 24H, H-8), 0.98 (s, 24H, H-1) ppm;

^{13}C NMR (101 MHz, CD_3OD): δ 196.7, 144.8, 139.8, 134.2, 126.5, 59.5 (ap. t, $J_{C\text{-}N}$ = 2.8 Hz), 47.8, 46.8, 26.2, 24.8, 20.7 (ap. t, $J_{C\text{-}N}$ = 1.5 Hz), 13.9 ppm;

HRMS (+ESI) *m/z* for cation found 242.2837, $[C_{16}H_{36}N]^+$ requires 242.2842, (δ = − 2.1 ppm);

HRMS (−ESI) *m/z* for dianion found 457.9915, $[C_{32}H_{38}O_{14}Rh_2S_2]^{2-}$ requires 457.9912, (δ = + 0.7 ppm).

1-Bromo-3,5-bis(bromomethyl)benzene

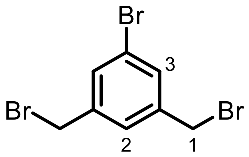

To an oven-dried three-necked round-bottomed flask fitted with a reflux condenser was added *N*-bromosuccinimide (25.0 g, 140.6 mmol) and 2,2'-azobis(isobutyronitrile) (578 mg, 3.52 mmol). After placing under a nitrogen atmosphere, 5-bromo-*m*-xylene (9.55 mL, 70.3 mmol) and MeCN (170 mL) were added, and the resulting mixture was heated at reflux for 3 hours then cooled to room temperature. Then reaction mixture was concentrated under reduced pressure, treated with $CHCl_3$ (100 mL) and heated to fully dissolve the crude solids. The solution was cooled to room temperature and then further in ice and the insoluble by-product was filtered out and washed with ice cold $CHCl_3$. The filtrate was collected, concentrated under reduced pressure and recrystallized from hot EtOH (15 mL) to afford the title compound as a white solid (9.64 g, 28.1 mmol, 40%).

***R_f* value**: 0.30 (hexane);

^{1}H NMR (400 MHz, $CDCl_3$): δ 7.47 (d, J = 1.5 Hz, 2H, H-3), 7.34 (t, J = 1.4 Hz, 1H, H-2), 4.40 (s, 4H, H-1) ppm;

^{13}C NMR (101 MHz, $CDCl_3$): δ 140.4, 132.1, 128.4, 122.8, 31.6 ppm;

HRMS (ESI): *this compound did not ionise.*

Dimethyl 3,3'-(5-bromo-1,3-phenylene)bis(2,2-dimethylpropanoate)

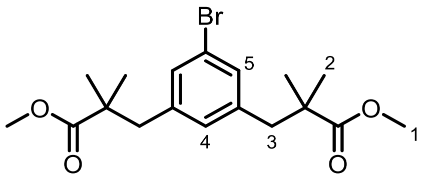

To a solution of *i*Pr_2NH (8.82 mL, 62.5 mmol) in THF (40 mL) was added nBuLi (39.1 mL of a 1.6 M solution in hexanes, 62.5 mmol) at 0 °C dropwise. The solution was stirred at this temperature for 1 hour and cooled to − 40 °C following which methyl isobutyrate (7.16 mL, 62.5 mmol) in THF (20 mL) was added dropwise. The solution was stirred at 0 °C for 1 hour then re-cooled to − 40 °C following which 1-bromo-3,5-bis(bromomethyl)benzene (8.57 g, 25.0 mmol) was added in a single portion. At this point, the mixture becomes cloudy due to poor solubility at − 40 °C, however, after gradually warming to room temperature over 1 hour the reaction becomes homogeneous. After stirring at room temperature for 1 hour, the reaction is quenched with saturated aqueous NH_4Cl, extracted with EtOAc × 3 and the organic layers combined. The organic layers were washed with brine × 2, dried over $MgSO_4$ and concentrated under reduced pressure to afford the title compound as a white solid (7.40 g, 19.2 mmol, 77%).

***R_f* value**: 0.16 (5% v/v ethyl acetate in hexane);

^{1}H NMR (400 MHz, $CDCl_3$): δ 7.10 (d, *J* = 1.3 Hz, 2H, H-5), 6.77 (t, *J* = 1.3 Hz, 1H, H-4), 3.66 (s, 6H, H-1), 2.77 (s, 4H, H-3), 1.16 (s, 12H, H-2) ppm;

^{13}C NMR (101 MHz, $CDCl_3$): δ 177.7, 139.8, 131.3, 131.0, 121.8, 51.9, 45.9, 43.7, 25.1 ppm;

HRMS (+ESI): *m/z* Found $[M+Na]^+$ 407.0830, $[C_{18}H_{25}BrNaO_4]^+$ requires 407.0828 (δ = + 0.5 ppm).

Dimethyl 3,3'-(5-(4,4,5,5-tetramethyl-1,3,2-dioxaborolan-2-yl)-1,3-phenylene)bis(2,2-dimethylpropanoate)

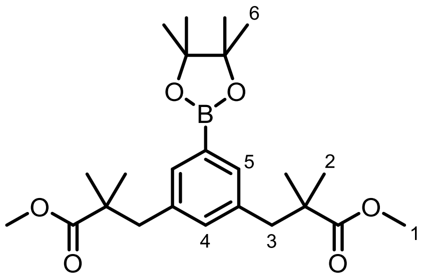

To an oven-dried round-bottomed flask fitted with a reflux condenser was added dimethyl 3,3'-(5-bromo-1,3-phenylene)bis(2,2-dimethylpropanoate) (4.82 g, 12.5 mmol), bis(pinacolato)diboron (3.17 g, 12.5 mmol), KOAc (2.45 g, 25 mmol) and ([1,1'-bis(diphenylphosphino)ferrocene]dichloropalladium(II), complex with CH_2Cl_2) (510 mg, 0.625 mmol). The combined solids were placed under a nitrogen atmosphere, dissolved in 1,4-dioxane (75 mL), sparged with N_2 for 30 minutes and heated at 80 °C overnight. The reaction mixture was cooled to room

temperature, diluted in EtOAc, filtered over Celite® and the filtrate concentrated under reduced pressure. Purification by flash column chromatography (SiO_2, 0-15% v/v EtOAc in hexane) afforded the title compound as a white solid (3.41 g, 7.88 mmol, 63%).

***R_f* value**: 0.24 (10% v/v ethyl acetate in hexane);

^{1}H NMR (500 MHz, $CDCl_3$) δ 7.39 (d, J = 1.7 Hz, 2H, H-5), 6.94 (t, J = 1.7 Hz, 1H, H-4), 3.67 (s, 6H, H_1), 2.82 (s, 4H, H-3), 1.32 (s, 12H, H-6), 1.16 (s, 12H, H-2) ppm;

^{13}C NMR (126 MHz, $CDCl_3$) δ 178.1, 137.0, 135.0, 134.8, 83.8, 51.8, 46.2, 43.8, 25.1, 25.0 ppm;

Carbon resonance belonging to the borylated carbon was not observed.

HRMS (+ESI) *m/z* found $[M+Na]^+$ 455.2567, $[C_{24}H_{37}BNaO_6]$ requires 455.2575, (δ = − 1.8 ppm);

Phenyl 2-bromobenzenesulfonate

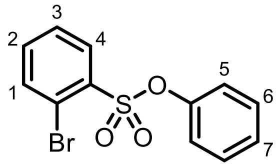

Triethylamine (7.0 mL, 50 mmol) was added to a stirred suspension of 2-bromobenzensulfonyl chloride (2.55 g, 10.0 mmol) and phenol (988 mg, 10.5 mmol) in CH_2Cl_2 (10.0 mL) at room temperature. After stirring at room temperature overnight, H_2O (10 mL) was added, and the resulting biphasic mixture was separated and extracted with CH_2Cl_2 (3 × 10 mL). The organic layers were combined, washed with saturated aqueous Na_2CO_3 solution (3 × 10 mL), 10% aqueous NaOH solution (10 mL), dried over $MgSO_4$ and concentrated *in vacuo* to afford the title compound as a white solid (2.976 g, 95%, 9.540 mmol).

***R_f* value**: 0.45 (20% v/v EtOAc in hexane);

^{1}H NMR (400 MHz, $(CD_3)_2SO$) δ 8.05 (dd, J = 7.9, 1.2 Hz, 1H, H-1), 7.92 (dd, J = 7.9, 1.7 Hz, 1H, H-4), 7.71 (td, J = 7.6, 1.7 Hz, 1H, H-2), 7.60 (td, J = 7.7, 1.2 Hz, 1H, H-3), 7.41 (m, 2H, H-6), 7.33 (tt, J = 7.5, 1.1 Hz, 1H, H-7), 7.11 (m, 2H, H-5) ppm;

^{13}C NMR (101 MHz, $(CD_3)_2SO$) δ 148.9, 136.4, 136.1, 134.1, 132.5, 130.3, 128.6, 127.7, 121.7, 120.4 ppm;

The spectroscopic data is in agreement with that reported in the literature.[20]

Dimethyl 3,3'-(2'-(phenoxysulfonyl)-[1,1'-biphenyl]-3,5-diyl)bis(2,2-dimethylpropanoate)

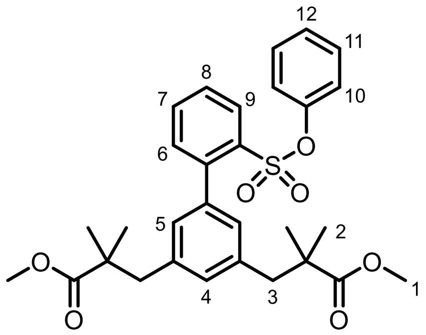

Between 4 oven-dried microwave vials was equally added phenyl 2-bromobenzenesulfonate (3.45 g, 11.0 mmol), dimethyl 3,3'-(5-(4,4,5,5-tetramethyl-1,3,2-dioxaborolan-2-yl)-1,3-phenylene)bis(2,2-dimethylpropanoate) (3.40 g, 7.86 mmol), palladium acetate (88 mg, 0.39 mmol), SPhos (323 mg, 0.786 mmol) and K_3PO_4 (5.01 g, 23.6 mmol). The vials were placed under a nitrogen atmosphere and to each was added THF (15 mL) and H_2O (1.67 mL). The resulting mixtures were heated at 90 °C overnight. After cooling to room temperature, the crude mixtures were combined, diluted in EtOAc, filtered over Celite® and the filtrate concentrated under reduced pressure. Purification by flash column chromatography (SiO_2, 0-23% v/v EtOAc in hexane) afforded the title compound as a colourless oil (3.21 g, 5.97 mmol, 76%).

***R_f* value**: 0.16 (15% v/v ethyl acetate in hexane);

^{1}H NMR (400 MHz, $CDCl_3$) δ 8.00 (d, *J* = 7.9 Hz, 1H, H-9), 7.65 (t, *J* = 7.7 Hz, 1H, H-7), 7.47 (t, *J* = 7.8 Hz, 1H, H-8), 7.37 (d, *J* = 7.8 Hz, 1H, H-6), 7.29-7.19 (m, 3H, H-11, H-12), 7.04 (s, 2H, H-5), 6.93 (s, 1H, H-4), 6.83 (d, *J* = 7.5 Hz, 2H, H-10), 3.62 (s, 6H, H-1), 2.86 (s, 4H, H-3), 1.15 (s, 12H, H-2) ppm;

^{13}C NMR (101 MHz, $CDCl_3$) δ 178.1, 149.3, 142.9, 138.4, 137.1, 134.6, 133.7, 133.2, 132.0, 130.3, 129.7, 129.6, 127.7, 127.1, 122.4, 51.9, 46.1, 43.7, 25.0 ppm.

HRMS (+ESI) *m/z* found $[M+Na]^+$ 561.1924, $[C_{30}H_{34}NaO_7S]$ requires 561.1917, (δ = + 1.2 ppm).

Tetrabutylammonium 3',5'-bis(2-carboxy-2-methylpropyl)-[1,1'-biphenyl]-2-sulfonate

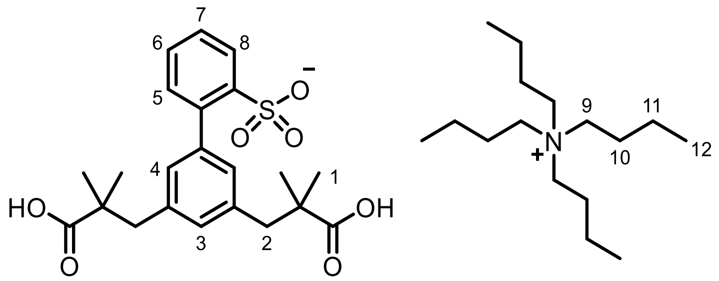

A three-necked round-bottomed flask fitted with a reflux condenser charged with dimethyl 3,3'-(2'-(phenoxysulfonyl)-[1,1'-biphenyl]-3,5-diyl)bis(2,2-dimethylpropanoate) (155 mg, 0.288 mmol) and NaOH (81

mg, 2.0 mmol) in 1:1 MeOH/H_2O (4.0 mL) was placed under a nitrogen atmosphere and heated at reflux for 18 hours. After cooling to room temperature, the resulting residue was concentrated under a stream of nitrogen and acidified to pH ~7 with 3 M aqueous HCl solution. The suspension was washed with Et_2O (5 × 5 mL) to remove the phenol by-product, the aqueous acidified further to pH ~2 and NBu_4HSO_4 (69 mg, 0.20 mmol) and 3:1 $CHCl_3$/*i*PrOH (10 mL) were added. The phases were separated, the aqueous washed with 3:1 $CHCl_3$/*i*PrOH (2 × 10 mL), organic phases combined, washed with H_2O (15 mL), dried over $MgSO_4$ and concentrated under reduced pressure. The collected oil was triturated with Et_2O and further freeze dried to afford the title compound as a white solid (120 mg, 0.178 mmol, 62%).

^{1}H NMR (400 MHz, CD_3OD) δ 8.08 (d, J = 7.6 Hz, 1H, H-8), 7.42 (t, J = 7.6 Hz, 1H, H-6), 7.36 (t, J = 7.5 Hz, 1H, H-7), 7.23-7.17 (m, 3H, H-4, H-5), 6.96 (s, 1H, H-3), 3.23 (m, 8.0H, H-9), 2.88 (s, 4H, H-2), 1.65 (p, J = 7.3 Hz, 8.0H, H-10), 1.40 (sext, J = 7.4 Hz, 8.0H, H-11), 1.17 (s, 12H, H-1), 1.02 (t, J = 7.3 Hz, 12.1H, H-12) ppm;

^{13}C NMR (101 MHz, CD_3OD) δ 181.7, 144.3, 142.3, 142.2, 137.6, 133.4, 131.9, 131.0, 130.8, 128.9, 127.6, 59.5 (ap. t, $^1J_{C\text{-}N}$ = 2.7 Hz), 47.1, 44.4, 25.5, 24.8, 20.7, 13.9 ppm;

HRMS (+ESI) *m/z* for cation found 242.2840, $[C_{16}H_{36}N]^+$ requires 242.2842, (δ = − 0.7 ppm);

HRMS (−ESI) *m/z* for anion found 433.1319, $[C_{22}H_{25}O_7S]^-$ requires 433.1326, (δ = − 1.6 ppm).

Bis[rhodium tetrabutylammonium (3',5'-bis(2-carboxy-2-methylpropyl)-[1,1'-biphenyl]-2-sulfonate)] (Rh_2(**A-III**)$_2$●(**NBu$_4$**)$_2$)

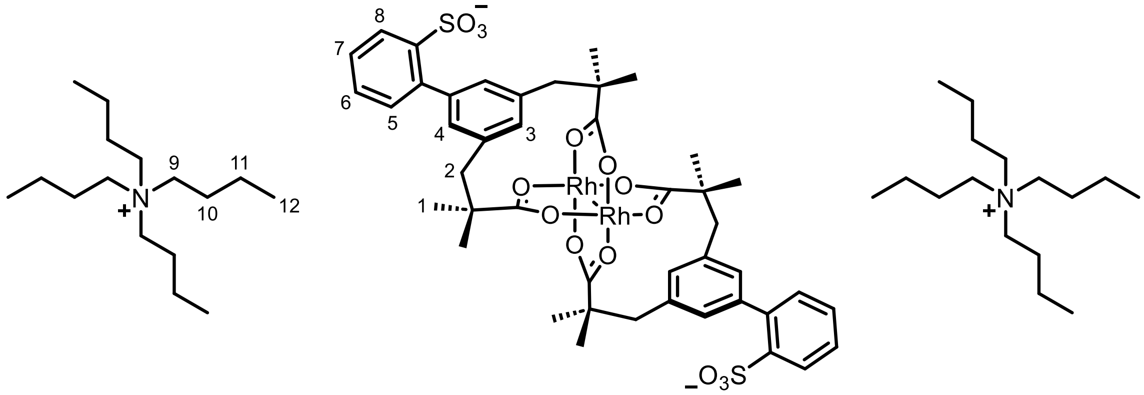

Prepared according to **GP2** on a 0.283 mmol scale with respect to $Rh_2(TFA)_4$ and tetrabutylammonium 3',5'-bis(2-carboxy-2-methylpropyl)-[1,1'-biphenyl]-2-sulfonate as the ligand. The title compound was obtained as a green solid (272 mg, 0.175 mmol, 62%).

^{1}H NMR (500 MHz, CD_3OD) δ 8.06 (d, J = 7.7, 1.2 Hz, 2H, H-8), 7.43 (td, J = 7.6, 1.2 Hz, 2H, H-6), 7.35 (td, J = 7.5, 1.3 Hz, 2H, H-7), 7.15 (dd, J = 7.6, 1.2 Hz, 2H, H-5), 7.00-6.96 (m, 6H, H-4, H-3), 3.22 (m, 16.4H, H-9), 2.67 (s, 8H, H-2), 1.65 (m, 16.3H, H-10), 1.40 (sext, J = 7.4 Hz, 16.4H, H-11), 1.02 (t, J = 7.3 Hz, 26.1H, H-12), 1.00 (s, 24H, H-1) ppm;

^{13}C NMR (126 MHz, CD_3OD) δ 197.1, 144.2, 142.6, 141.2, 138.2, 133.3, 131.0, 130.8, 130.4, 129.0, 59.5 (ap. t, J_{C-N} = 2.9 Hz), 48.0, 46.8, 26.3, 24.8, 20.7 (ap. t, J_{C-N} = 1.4 Hz), 13.9 ppm;

HRMS (−ESI) *m/z* for dianion found 534.0210, $[C_{44}H_{46}O_{14}Rh_2S_2]^{2-}$ requires 534.0225, (δ = − 2.8 ppm);

HRMS (+ESI) *m/z* for cation found 242.2846, $[C_{16}H_{36}N]^+$ requires 242.2842, (δ = + 1.7 ppm).

Diethyl 1,1'-((5-bromo-1,3-phenylene)bis(methylene))bis(cyclobutane-1-carboxylate)

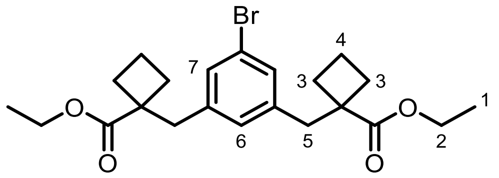

To a solution of *i*Pr_2NH (1.57 mL, 11.2 mmol) in THF (25 mL) at 0 °C was added nBuLi (7.0 mL of a 1.6 M solution in hexanes, 11.2 mmol) dropwise. The solution was stirred at this temperature for 1 hour and cooled to − 40 °C following which ethyl cyclobutanecarboxylate (1.55 mL, 11.2 mmol) was added dropwise. The solution was stirred at 0 °C for 1 hour then re-cooled to − 40 °C following which 1-bromo-3,5-bis(bromomethyl)benzene (1.73 g, 5.09 mmol) was added in a single portion. At this point, the mixture becomes cloudy due to poor solubility at − 40 °C, however, after gradually warming to room temperature over 1 hour the reaction becomes homogeneous. After stirring at room temperature for 1 hour, the reaction is quenched with saturated aqueous NH_4Cl, extracted with EtOAc × 3 and the organic layers combined. The organic layers were washed with brine × 2, dried over $MgSO_4$, concentrated under reduced pressure and purified by flash column chromatography (SiO_2, 0-10% v/v EtOAc in hexane) to afford the title compound as a white solid (1.35 g, 3.09 mmol, 61%).

***R_f* value**: 0.50 (5% v/v ethyl acetate in hexane);

^{1}H NMR (400 MHz, $CDCl_3$): δ 7.12 (d, *J* = 1.2 Hz, 2H, H-7), 6.84 (t, *J* = 1.5 Hz, 1H, H-6), 4.11 (q, *J* = 7.2 Hz, 4H, H-2), 2.99 (s, 4H, H-5), 2.40 (m, 4H, H-3_A), 2.01 (m, 4H, H-3_B), 1.87 (m, 4H, H-4), 1.21 (t, *J* = 7.1 Hz, 6H, H-1) ppm;

^{13}C NMR (101 MHz, $CDCl_3$): δ 176.4, 140.3, 130.5, 129.1, 122.0, 60.7, 48.7, 42.9, 30.0, 15.7, 14.3 ppm;

HRMS (+ESI): *m/z* found $[M+Na]^+$ 459.1141, $[C_{22}H_{29}BrNaO_4]^+$ requires 459.1141, (δ = + 0.0 ppm);

Diethyl 1,1'-((5-(4,4,5,5-tetramethyl-1,3,2-dioxaborolan-2-yl)-1,3-phenylene)bis(methylene))bis(cyclobutane-1-carboxylate)

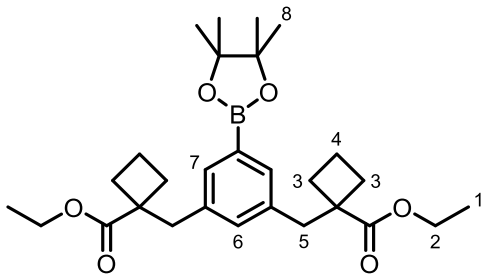

To an oven-dried round-bottomed flask fitted with a reflux condenser was added diethyl 1,1'-((5-bromo-1,3-phenylene)bis(methylene))bis(cyclobutane-1-carboxylate) (1.27 g, 2.90 mmol), bis(pinacolato)diboron (810 mg, 3.19 mmol), KOAc (569 mg, 5.80 mmol) and ([1,1'-bis(diphenylphosphino)ferrocene]dichloropalladium(II), complex with CH_2Cl_2) (118 mg, 0.145 mmol). The combined solids were placed under a nitrogen atmosphere, dissolved in 1,4-dioxane (25 mL), sparged with N_2 for 30 minutes and heated at 80 °C overnight. The reaction mixture was cooled to room temperature, diluted in EtOAc, filtered over Celite® and the filtrate concentrated under reduced pressure. Purification by flash column chromatography (SiO_2, 0-15% v/v EtOAc in hexane) afforded the title compound as a pale-yellow oil (1.16 g, 2.39 mmol, 82%).

***R_f* value**: 0.47 (10% v/v ethyl acetate in hexane);

^{1}H NMR (400 MHz, $CDCl_3$): δ 7.43 (d, J = 1.5 Hz, 2H, H-7), 7.00 (t, J = 1.5 Hz, 1H, H-6), 4.11 (q, J = 7.1 Hz, 4H, H-2), 3.03 (s, 4H, H-5), 2.37 (m, 4H, H-3_A), 2.02 (m, 4H, H-3_B), 1.83 (m, 4H, H-4), 1.31 (s, 12H, H-8), 1.22 (t, J = 7.1 Hz, 6H, H-1) ppm;

^{13}C NMR (101 MHz, $CDCl_3$): δ 176.9, 137.4, 134.2, 133.3, 83.7, 60.5, 48.8, 43.1, 29.8, 25.0, 15.6, 14.3 ppm;

The carbon resonance belonging to the borylated carbon was not detected.

HRMS (+ESI): *m/z* found $[M+Na]^+$ 507.2894, $[C_{28}H_{41}BNaO_6]^+$ requires 507.2888, (δ = + 1.2 ppm);

Diethyl 1,1'-((2'-(phenoxysulfonyl)-[1,1'-biphenyl]-3,5-diyl)bis(methylene))bis(cyclobutane-1-carboxylate)

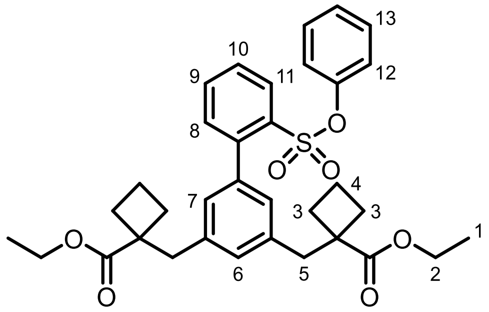

Between 2 oven-dried microwave vials was equally added phenyl 2-bromobenzenesulfonate (1.53 g, 4.89 mmol) diethyl 1,1'-((5-(4,4,5,5-tetramethyl-1,3,2-dioxaborolan-2-yl)-1,3-phenylene)bis(methylene))bis(cyclobutane-1-carboxylate) (1.19 g, 2.45 mmol), palladium acetate (27 mg, 0.12 mmol), SPhos (101 mg, 0.25 mmol) and K_3PO_4 (1.56 g, 7.34 mmol). The vials were placed under a nitrogen atmosphere and to each was added THF (13.5 mL) and H_2O (1.5 mL). The resulting mixtures were heated at 90 °C overnight. After cooling to room temperature, the crude mixtures were combined, diluted in EtOAc, filtered over Celite® and the filtrate concentrated under reduced pressure. Purification by flash column chromatography (SiO_2, 0-20% v/v EtOAc in hexane) afforded the title compound as a colourless oil (1.30 g, 2.19 mmol, 89%). *Note: this compound contained traces of an aromatic impurity which is fully separated in the remaining steps in the synthesis of* Rh_2(**B**-**III**)$_2$●(**NBu$_4$**)$_2$ *below.*

***R$_f$* value**: 0.36 (20% v/v ethyl acetate in hexane);

^{1}H NMR (500 MHz, $CDCl_3$): *δ* 8.03 (dd, *J* = 8.1, 1.2 Hz, 1H, H-11), 7.64 (td, *J* = 7.5, 1.3 Hz, 1H, H-9), 7.47 (td, *J* = 7.7, 1.3 Hz, 1H, H-10), 7.35 (dd, *J* = 7.7, 1.3 Hz, 1H, H-8), 7.26 (m, 2H, H-13), 7.21 (m, 1H, H-14), 7.08 (d, *J* = 1.6 Hz, 2H, H-7), 7.00 (t, *J* = 1.4 Hz, 1H, H-6), 6.80 (m, 2H, H-12), 4.08 (q, *J* = 7.1 Hz, 4H, H-2), 3.07 (s, 4H, H-5), 2.37 (m, 4H, H-3$_A$), 2.03 (m, 4H, H-3$_B$), 1.81 (m, 4H, H-4), 1.17 (t, *J* = 7.1 Hz, 6H, H-1) ppm;

^{13}C NMR (126 MHz, $CDCl_3$): *δ* 176.8, 149.1, 142.9, 138.7, 137.6, 134.5, 133.7, 133.2, 130.3, 129.7, 128.9, 127.6, 127.0, 122.4, 60.5, 48.6, 43.0, 29.8, 15.7, 14.3 ppm;

Note: there is an additional ^{13}C resonance underneath the peak at 130.3 ppm.

HRMS (+ESI): *m/z* found $[M+Na]^+$ 613.2234, $[C_{34}H_{38}NaO_7S]^+$ requires 613.2230, (*δ* = + 0.7 ppm).

Tetrabutylammonium 3',5'-bis((1-carboxycyclobutyl)methyl)-[1,1'-biphenyl]-2-sulfonate

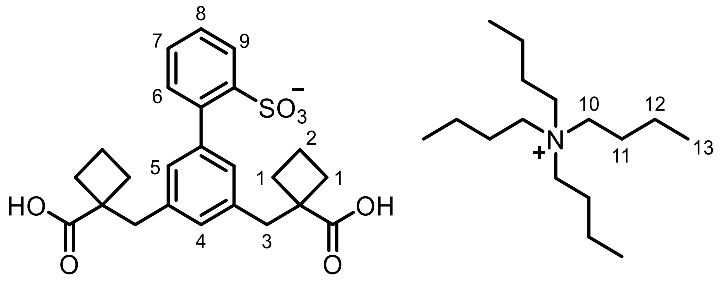

A three-necked round-bottomed flask fitted with a reflux condenser charged with diethyl 1,1'-((2'-(phenoxysulfonyl)-[1,1'-biphenyl]-3,5-diyl)bis(methylene))bis(cyclobutane-1-carboxylate) (1.30 g, 2.14 mmol) and NaOH (1.71 g, 42.8 mmol) in 1:1:1 MeOH/H_2O/THF (30 mL) was placed under a nitrogen atmosphere and heated at reflux for 18 hours. After cooling to room temperature, the resulting residue was concentrated under a stream of nitrogen, washed with CH_2Cl_2 and the aqueous acidified to pH ~7 with 3 M aqueous HCl solution. The suspension was washed with Et_2O to remove the phenol by-product, the aqueous acidified further to pH ~2 and NBu_4HSO_4 (545 mg, 1.61 mmol) and 3:1 $CHCl_3$/*i*PrOH were added. The phases were separated, the aqueous washed with 3:1 $CHCl_3$/*i*PrOH, organic phases combined, washed with H_2O, dried over $MgSO_4$ and concentrated

under reduced pressure. The collected oil was triturated with Et_2O and further freeze dried to afford the title compound as a white solid (1.06 g, 1.52 mmol, 71%).

^{1}H NMR (400 MHz, CD_3OD): δ 8.08 (dd, J = 7.7, 1.5 Hz, 1H, H-9), 7.42 (td, J = 7.5, 1.5 Hz, 1H, H-7), 7.36 (td, J = 7.7, 1.6 Hz, 1H, H-8), 7.24 (d, J = 1.3 Hz, 2H, H-5), 7.18 (dd, J = 7.5, 1.5 Hz, 1H, H-6), 7.00 (t, J = 1.4 Hz, 1H, H-4), 3.22 (m, 7.8H, H-10), 3.09 (s, 4H, H-3), 2.37 (m, 4H, H-1_A), 2.11 (m, 4H, H-1_B), 1.86 (m, 4H, H-2), 1.65 (m, 7.9H, H-11), 1.40 (sext, J = 7.4 Hz, 8.0H, H-12), 1.01 (t, J = 7.3 Hz, 11.8H, H-13) ppm;

^{13}C NMR (101 MHz, CD_3OD): δ 180.9, 144.3, 142.7, 142.2, 138.0, 133.4, 130.8, 130.3, 130.2, 128.9, 127.7, 59.5 (ap. t, $J_{C\text{-}N}$ = 2.8 Hz), 50.0, 44.1, 30.7, 24.8, 20.7 (ap. t, $J_{C\text{-}N}$ = 1.5 Hz), 16.2, 14.0 ppm;

HRMS (–ESI): *m/z* for anion found 457.1330, $[C_{24}H_{25}O_7S]^-$ requires 457.1326, (δ = + 0.9 ppm).

Bis[rhodium tetrabutylammonium 3',5'-bis((1-carboxycyclobutyl)methyl)-[1,1'-biphenyl]-2-sulfonate] (Rh_2(**B-III**)$_2$●(**NBu_4**)$_2$)

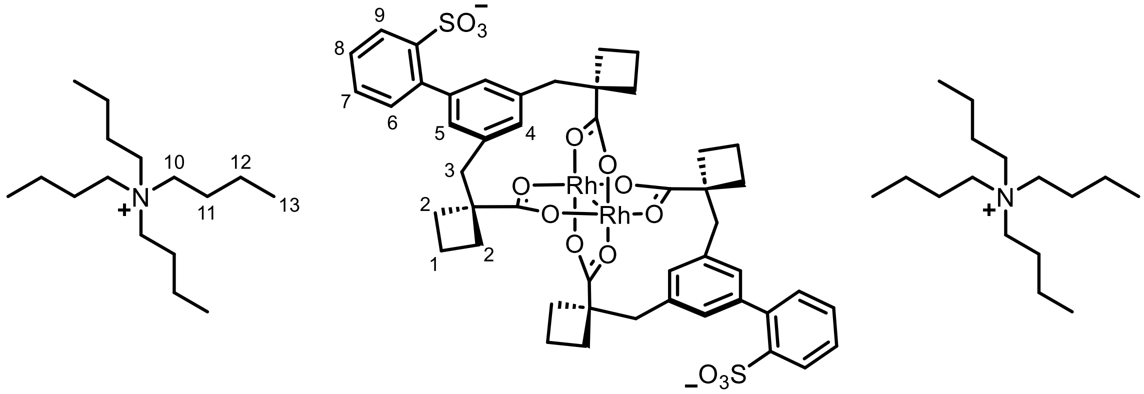

Prepared according to **GP2** on a 0.30 mmol scale with respect to $Rh_2(TFA)_4$ and tetrabutylammonium 3',5'-bis((1-carboxycyclobutyl)methyl)-[1,1'-biphenyl]-2-sulfonate as the ligand. The title compound was obtained as a green solid (391 mg, 0.244 mmol, 81%).

^{1}H NMR (500 MHz, CD_3OD): δ 8.07 (dd, J = 7.9, 1.4 Hz, 2H, H-9), 7.44 (td, J = 7.4, 1.3 Hz, 2H, H-7), 7.36 (td, J = 7.7, 1.4 Hz, 2H, H-8), 7.15 (dd, J = 7.6, 1.3 Hz, 2H, H-6), 7.09 (d, J = 1.6 Hz, 4H, H-5), 6.89 (t, J = 1.5 Hz, 2H, H-4), 3.23 (m, 15.7H, H-10), 2.94 (s, 8H, H-3), 2.18 (m, 8H, H-2_A), 1.95-1.81 (m, 12H, H-1_A, H-2_B), 1.71-1.60 (m, 19.8H, H-1_B, H-11), 1.41 (sext, J = 7.4 Hz, 16.0H, H-12), 1.02 (t, J = 7.4 Hz, 23.8H, H-13), ppm;

^{13}C NMR (126 MHz, CD_3OD): δ 196.6, 144.2, 142.4, 141.8, 138.0, 133.3, 130.9, 130.6, 129.6, 129.0, 127.6, 59.5, (ap. t, $J_{C\text{-}N}$ = 2.8 Hz), 52.8, 44.9, 31.4, 24.8, 20.7 (ap. t, $J_{C\text{-}N}$ = 1.5 Hz), 16.3, 13.9 ppm;

HRMS (–ESI) *m/z* for dianion found 558.0226, $[C_{48}H_{46}O_{14}Rh_2S_2]^{2-}$ requires 558.0225, (δ = + 0.2 ppm);

HRMS (+ESI) *m/z* for cation found 242.2850, $[C_{16}H_{36}N]^+$ requires 242.2842, (δ = + 3.3 ppm).

Dimethyl 1,1'-((5-bromo-1,3-phenylene)bis(methylene))bis(cyclopentane-1-carboxylate)

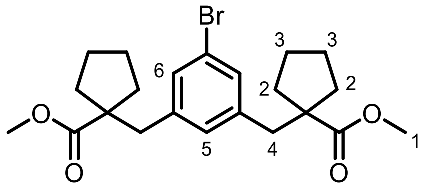

To a solution of *i*Pr_2NH (4.78 mL, 34.1 mmol) in THF (60 mL) at 0 °C was added nBuLi (21.3 mL of a 1.6 M solution in hexanes, 34.1 mmol) dropwise. The solution was stirred at this temperature for 1 hour and cooled to − 40 °C following which methyl cyclopentanecarboxylate (4.46 mL, 34.1 mmol) in THF (10 mL) was added dropwise. The solution was stirred at 0 °C for 1 hour then re-cooled to − 40 °C following which 1-bromo-3,5-bis(bromomethyl)benzene (4.64 g, 13.7 mmol) was added in a single portion. At this point, the mixture becomes cloudy due to poor solubility at − 40 °C, however, after gradually warming to room temperature over 1 hour the reaction becomes homogeneous. After stirring at room temperature for 1 hour, the reaction is quenched with saturated aqueous NH_4Cl, extracted with EtOAc × 3 and the organic layers combined. The organic layers were washed with brine × 2, dried over $MgSO_4$, concentrated under reduced pressure and purified by flash column chromatography (SiO_2, 0-10% v/v EtOAc in hexane) to afford the title compound as a white solid (4.60 g, 10.5 mmol, 77%).

***R_f* value**: 0.24 (5% v/v ethyl acetate in hexane);

^{1}H NMR (400 MHz, $CDCl_3$): δ 7.08 (d, *J* = 1.4 Hz, 2H, H-6), 6.77 (t, *J* = 1.3 Hz, 1H, H-5), 3.65 (s, 6H, H-1), 2.85 (s, 4H, H-4), 2.05 (m, 4H, H-2_A), 1.70-1.50 (m, 12H, H-2_B, H-3) ppm;

^{13}C NMR (101 MHz, $CDCl_3$): δ 177.4, 140.7, 130.7, 129.8, 122.0, 53.6, 51.9, 43.7, 35.7, 24.5 ppm;

HRMS (+ESI): *m/z* found $[M+Na]^+$ 459.1133, $[C_{22}H_{29}BrNaO_4]^+$ requires 459.1141, (δ = − 1.7 ppm).

Dimethyl 1,1'-((5-(4,4,5,5-tetramethyl-1,3,2-dioxaborolan-2-yl)-1,3-phenylene)bis(methylene))bis(cyclopentane-1-carboxylate)

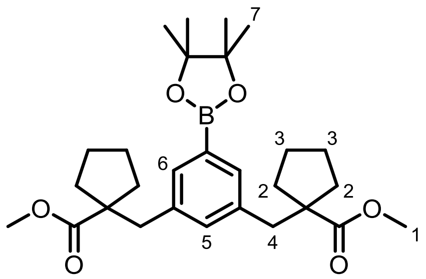

To an oven-dried round-bottomed flask fitted with a reflux condenser was added dimethyl 1,1'-((5-bromo-1,3-phenylene)bis(methylene))bis(cyclopentane-1-carboxylate) (3.49 g, 8.0 mmol), bis(pinacolato)diboron (2.13 g, 8.40 mmol), KOAc (1.57 g, 16.0 mmol) and ([1,1'-bis(diphenylphosphino)ferrocene]dichloropalladium(II), complex with CH_2Cl_2) (327 mg, 0.40 mmol). The combined solids were placed under a nitrogen atmosphere,

dissolved in 1,4-dioxane (64 mL), sparged with N_2 for 30 minutes and heated at 80 °C overnight. The reaction mixture was cooled to room temperature, diluted in EtOAc, filtered over Celite® and the filtrate concentrated under reduced pressure. Purification by flash column chromatography (SiO_2, 0-15% v/v EtOAc in hexane) afforded the title compound as a white solid (2.80 g, 5.79 mmol, 72%).

***R**$_f$* **value**: 0.28 (10% v/v ethyl acetate in hexane);

^{1}H NMR (400 MHz, $CDCl_3$): δ 7.37 (d, J = 1.7 Hz, 2H, H-6), 6.93 (t, J = 1.7 Hz, 1H, H-5), 3.65 (s, 6H, H-1), 2.90 (s, 4H, H-4), 2.04 (m, 4H, H-2_A), 1.69-1.53 (m, 12H, H-2_B, H-3), 1.32 (s, 12H, H-7), ppm;

^{13}C NMR (101 MHz, $CDCl_3$): δ 177.8, 137.8, 134.3, 133.8, 83.7, 55.7, 51.7, 44.1, 35.6, 25.0, 24.5 ppm;

The carbon resonance belonging to the borylated carbon was not detected.

HRMS (+ESI): *m/z* found $[M+Na]^+$ 507.2899, $[C_{28}H_{41}BNaO_6]^+$ requires 507.2888, (δ = + 2.2 ppm).

Dimethyl 1,1'-((2'-(phenoxysulfonyl)-[1,1'-biphenyl]-3,5-diyl)bis(methylene))bis(cyclopentane-1-carboxylate)

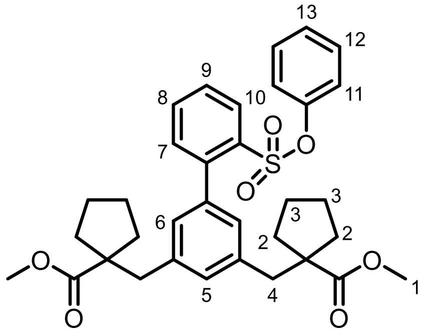

Between 3 oven-dried microwave vials was equally added phenyl 2-bromobenzenesulfonate (2.34 g, 7.50 mmol) dimethyl 1,1'-((5-(4,4,5,5-tetramethyl-1,3,2-dioxaborolan-2-yl)-1,3-phenylene)bis(methylene))bis(cyclopentane-1-carboxylate) (2.42 g, 5.00 mmol), palladium acetate (56 mg, 0.25 mmol), SPhos (205 mg, 0.50 mmol) and K_3PO_4 (3.18 g, 15.0 mmol). The vials were placed under a nitrogen atmosphere and to each was added THF (14.4 mL) and H_2O (1.6 mL). The resulting mixtures were heated at 90 °C overnight. After cooling to room temperature, the crude mixtures were combined, diluted in EtOAc, filtered over Celite® and the filtrate concentrated under reduced pressure. Purification by flash column chromatography (SiO_2, 0-20% v/v EtOAc in hexane) afforded the title compound as a thick colourless oil which was taken directly into the hydrolysis step (see below). *A quantitative yield was assumed for this step and the reaction conditions for the subsequent hydrolysis were scaled accordingly.*

Tetrabutylammonium 3',5'-bis((1-carboxycyclopentyl)methyl)-[1,1'-biphenyl]-2-sulfonate

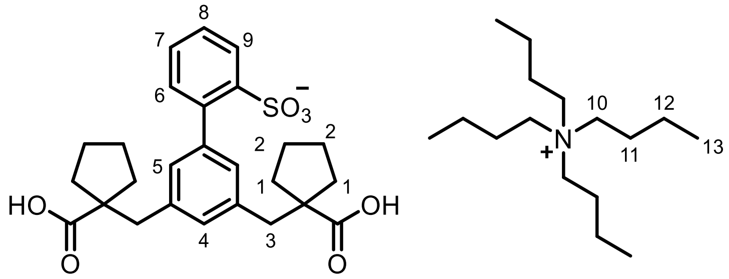

A three-necked round-bottomed flask fitted with a reflux condenser charged with dimethyl 1,1'-((2'-(phenoxysulfonyl)-[1,1'-biphenyl]-3,5-diyl)bis(methylene))bis(cyclopentane-1-carboxylate) (5.0 mmol) and NaOH (4.00 g, 100 mmol) in 2.5:2.5:1 MeOH/H_2O/THF (60 mL) was placed under a nitrogen atmosphere and heated at reflux for 18 hours. After cooling to room temperature, the resulting residue was concentrated under a stream of nitrogen, washed with CH_2Cl_2 and the aqueous acidified to pH ~7 with 3 M aqueous HCl solution. The suspension was washed with Et_2O to remove the phenol by-product, the aqueous acidified further to pH ~2 and NBu_4HSO_4 (1.02 g, 3.0 mmol) and 3:1 $CHCl_3$/*i*PrOH were added. The phases were separated, the aqueous washed with 3:1 $CHCl_3$/*i*PrOH, organic phases combined, washed with H_2O, dried over $MgSO_4$ and concentrated under reduced pressure. The collected oil was triturated with Et_2O and further freeze dried to afford the title compound as a white solid (2.41 g, 3.31 mmol, 66%).

^{1}H NMR (400 MHz, CD_3OD): δ 8.08 (dd, *J* = 7.8, 1.4 Hz, 1H, H-9), 7.42 (td, *J* = 7.5, 1.5 Hz, 1H, H-7), 7.36 (td, *J* = 7.6, 1.5 Hz, 1H, H-8), 7.22-7.17 (m, 3H, H-5, H-6), 7.00 (t, *J* = 1.4 Hz, 1H, H-4), 3.22 (m, 6.7H, H-10), 2.97 (s, 4H, H-3), 2.06 (m, 4H, H-1_A), 1.73-1.58 (m, 20H, H-1_B, H-2, H-11), 1.39 (sext, *J* = 7.4 Hz, 6.8H, H-12), 1.01 (t, *J* = 7.3 Hz, 10.1H, H-13) ppm;

^{13}C NMR (101 MHz, CD_3OD): δ 181.3, 144.2, 142.5, 142.2, 138.4, 133.4, 130.9, 130.8, 130.5, 128.9, 127.6, 59.5 (ap. t, J_{C-N} = 2.9 Hz), 56.7, 44.9, 36.5, 25.4, 24.8, 20.7 (ap. t, J_{C-N} = 1.5 Hz), 14.0 ppm;

HRMS (–ESI): *m/z* for anion found 485.1639, $[C_{26}H_{29}O_7S]^-$ requires 485.1639, (δ = 0.0 ppm).

Bis[rhodium tetrabutylammonium 3',5'-bis((1-carboxycyclopentyl)methyl)-[1,1'-biphenyl]-2-sulfonate] (Rh_2(**C-III**)$_2$●(**NBu$_4$**)$_2$)

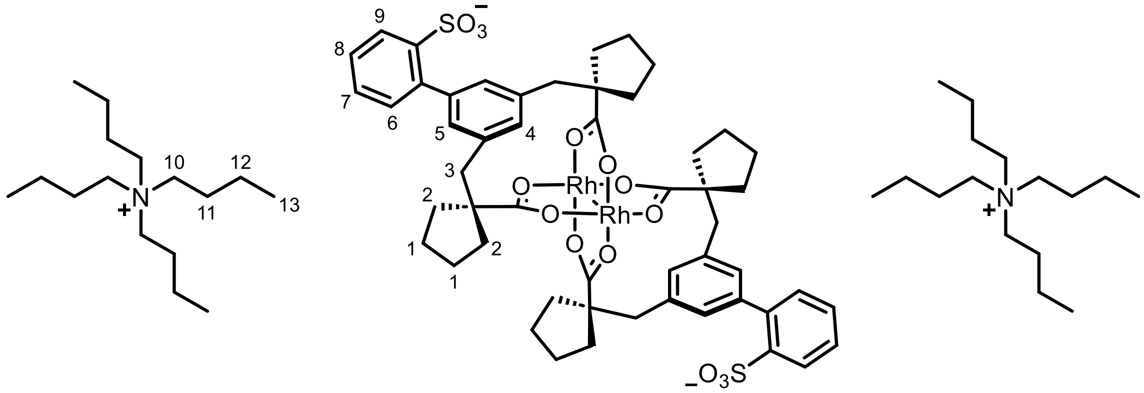

Prepared according to **GP2** on a 0.30 mmol scale with respect to $Rh_2(TFA)_4$ and tetrabutylammonium 3',5'-bis((1-carboxycyclopentyl)methyl)-[1,1'-biphenyl]-2-sulfonate as the ligand. The title compound was obtained as a green solid (319 mg, 0.192 mmol, 64%).

^{1}H NMR (500 MHz, d_6-DMSO): δ 7.90 (dd, J = 7.6, 1.3 Hz, 2H, H-9), 7.32 (td, J = 7.4, 1.5 Hz, 2H, H-7), 7.26 (td, J = 7.6, 1.4 Hz, 2H, H-8), 7.07-7.02 (m, 6H, H-5, H-6), 6.59 (t, J = 1.4 Hz, 2H, H-4), 3.16 (m, 15.0H, H-10), 2.65 (s, 8H, H-3), 1.70 (m, 8H, H-2_A), 1.60-1.47 (m, 23H, H-1_A, H-11), 1.42 (m, 8H, H-2_B), 1.35-1.26 (m, 23H, H-1_B, H-12), 0.93 (t, J = 7.4 Hz, 22.7H, H-13) ppm;

^{13}C NMR (126 MHz, d_6-DMSO): δ 196.8, 146.0, 141.2, 139.5, 136.2, 131.6, 128.4, 128.3, 128.2, 127.7, 126.0, 57.9, 57.5 (ap. t, $J_{C\text{-}N}$ = 2.5 Hz), 44.5, 35.7, 24.1, 23.1, 19.2 (ap. t, $J_{C\text{-}N}$ = 1.3 Hz), 13.5 ppm;

HRMS (−ESI): *m/z* for dianion found 586.0536, $[C_{52}H_{54}O_{14}Rh_2S_2]^{2-}$ requires 586.0538, (δ = − 0.3 ppm);

HRMS (+ESI): *m/z* for cation found 242.2848, $[C_{16}H_{36}N]^+$ requires 242.2842, (δ = + 2.5 ppm).

Dimethyl 1,1'-(1,3-phenylenebis(methylene))bis(cycloheptane-1-carboxylate)

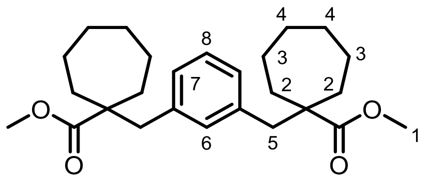

To a solution of *i*Pr_2NH (1.66 mL, 11.82 mmol) in THF (20 mL) at 0 °C was added nBuLi (7.39 mL of a 1.6 M solution in hexanes, 11.82 mmol) dropwise. The solution was stirred at this temperature for 1 hour and cooled to − 40 °C following which methyl cycloheptanecarboxylate (1.85 g, 11.82 mmol) in THF (5 mL) was added dropwise. The solution was stirred at 0 °C for 1 hour then re-cooled to − 40 °C following which 1,3-bis(bromomethyl)benzene (1.42 g, 5.37 mmol) was added in a single portion. At this point, the mixture becomes cloudy due to poor solubility at − 40 °C, however, after gradually warming to room temperature over 1 hour the reaction becomes

homogeneous. After stirring at room temperature for 1 hour, the reaction is quenched with saturated aqueous NH_4Cl, extracted with EtOAc × 3 and the organic layers combined. The organic layers were washed with brine × 2, dried over $MgSO_4$, concentrated under reduced pressure and purified by flash column chromatography (SiO_2, 0-10% v/v EtOAc in hexane) to afford the title compound as a colourless oil (1.50 g, 3.62 mmol, 67%).

***R_f* value**: 0.74 (20% v/v EtOAc in hexane);

^{1}H NMR (500 MHz, $CDCl_3$): δ 7.10 (t, *J* = 7.6 Hz, 1H, H-8), 6.87 (dd, *J* = 7.6, 1.7 Hz, 2H, H-7), 6.75 (t, *J* = 1.5 Hz, 1H, H-6), 3.65 (s, 6H, H-1), 2.78 (s, 4H, H-5), 2.01 (m, 4H, H-2_A), 1.58-1.41 (m, 20H, H-2_B, H-3, H-4) ppm;

^{13}C NMR (126 MHz, $CDCl_3$): δ 177.8, 137.6, 132.1, 128.1, 127.7, 51.6, 51.5, 46.9, 36.2, 30.0, 23.6 ppm;

HRMS (+ESI): *m/z* found $[M+Na]^+$ 437.2676, $[C_{26}H_{38}NaO_4]^+$ requires 437.2662, (δ = + 3.2 ppm).

Dimethyl 1,1'-((5-(4,4,5,5-tetramethyl-1,3,2-dioxaborolan-2-yl)-1,3-phenylene)bis(methylene))bis(cycloheptane-1-carboxylate)

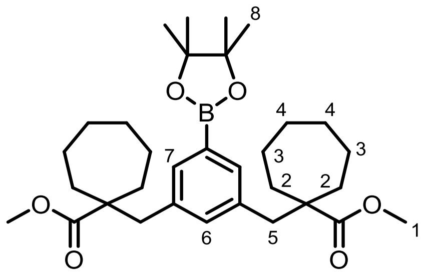

To an oven-dried microwave vial was added dimethyl 1,1'-(1,3-phenylenebis(methylene))bis(cycloheptane-1-carboxylate) (166 mg, 0.40 mmol), bis(pinacolato)diboron (203 mg, 0.80 mmol), 3,4,7,8-tetramethyl-1,10-phenanthroline (2.8 mg, 0.012 mmol) and (1,5-cyclooctadiene)(methoxy)iridium(I) dimer (4.0 mg, 0.006 mmol). The combined reagents were immediately placed under an argon atmosphere through thorough evacuation and backfill cycles. 1,4-dioxane (sparged with argon for 30 minutes) (2.5 mL) was added and the resulting mixture was heated at 90 °C overnight then cooled to room temperature and concentrated under a stream of nitrogen. Purification by flash column chromatography (0-10% v/v EtOAc in hexane) afforded the title compound as a colourless oil (80 mg, 0.15 mmol, 37%).

***R_f* value**: 0.63 (20% v/v EtOAc in hexane);

^{1}H NMR (500 MHz, $CDCl_3$): δ 7.32 (d, *J* = 1.6 Hz, 2H, H-7), 6.82 (t, *J* = 1.6 Hz, 1H, H-6), 3.66 (s, 6H, H-1), 2.78 (s, 4H, H-5), 2.00 (m, 4H, H-2_A), 1.57-1.40 (m, 20H, H-2_B, H-3, H-4), 1.31 (s, 12H, H-8) ppm;

^{13}C NMR (126 MHz, $CDCl_3$): δ 177.8, 136.9, 134.7, 134.6, 83.7, 51.51, 51.50, 47.0, 36.2, 29.9, 25.0, 23.6 ppm;

The carbon resonance belonging to the borylated carbon was not detected.

HRMS (+ESI): *m/z* found $[M+Na]^+$ 563.3537, $[C_{32}H_{49}BNaO_6]^+$ requires 563.3514, (δ = + 4.1 ppm).

Dimethyl 1,1'-((2'-(phenoxysulfonyl)-[1,1'-biphenyl]-3,5-diyl)bis(methylene))bis(cycloheptane-1-carboxylate)

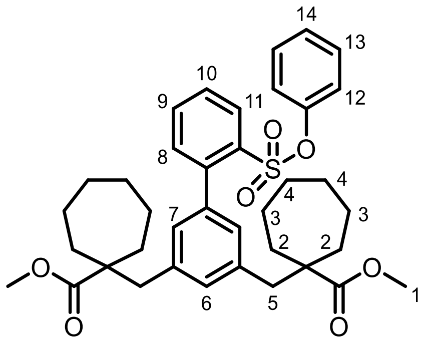

To an oven-dried microwave vial was added phenyl 2-bromobenzenesulfonate (283 mg, 0.91 mmol), dimethyl 1,1'-((5-(4,4,5,5-tetramethyl-1,3,2-dioxaborolan-2-yl)-1,3-phenylene)bis(methylene))bis(cycloheptane-1-carboxylate) (245 mg, 0.45 mmol), palladium acetate (5.2 mg, 0.023 mmol), SPhos (19 mg, 0.045 mmol) and K_3PO_4 (289 mg, 1.36 mmol). The mixture was placed under a nitrogen atmosphere, dissolved in THF (3.6 mL) and H_2O (0.4 mL) and heated at 90 °C overnight. After cooling to room temperature the crude mixture was diluted in EtOAc, filtered over Celite® and the filtrate concentrated under reduced pressure. Purification by flash column chromatography (SiO_2, 0-15% v/v EtOAc in hexane) afforded the title compound as a colourless oil (186 mg, 0.29 mmol, 64%).

***R_f* value**: 0.79 (40% v/v EtOAc in hexane);

^{1}H NMR (400 MHz, $CDCl_3$): *δ* 8.02 (d, *J* = 8.0 Hz, 1H, H-11), 7.65 (t, *J* = 7.5 Hz, 1H, H-9), 7.47 (t, *J* = 7.9 Hz, 1H, H-10), 7.36 (d, *J* = 7.5 Hz, 1H, H-8), 7.30-7.19 (m, 4H, H-6, H-13, H-14), 6.97 (s, 2H, H-7), 6.83 (m, 2H, H-12), 3.61 (s, 6H, H-1), 2.82 (s, 4H, H-5), 2.00 (m, 4H, H-2_A), 1.55-1.36 (m, 20H, H-2_B, H-3, H-4) ppm;

^{13}C NMR (101 MHz, $CDCl_3$): *δ* 177.9, 149.2, 143.0, 138.3, 137.1, 134.6, 133.7, 133.3, 131.7, 130.3, 129.7, 129.5, 127.6, 127.1, 122.4, 51.6, 51.5, 46.8, 36.2, 29.9, 23.5 ppm;

HRMS (−ESI): *m/z* found $[M-H]^-$ 645.2894, $[C_{38}H_{45}O_7S]^-$ requires 645.2891, (*δ* = + 0.5 ppm).

Tetrabutylammonium 3',5'-bis((1-carboxycycloheptyl)methyl)-[1,1'-biphenyl]-2-sulfonate

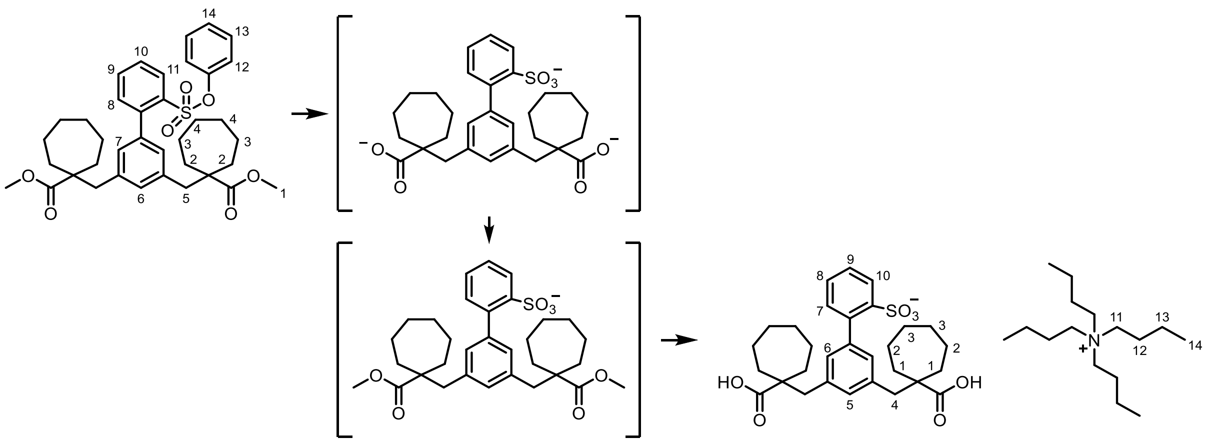

A three-necked round-bottomed flask fitted with a reflux condenser charged with dimethyl 1,1'-((2'-(phenoxysulfonyl)-[1,1'-biphenyl]-3,5-diyl)bis(methylene))bis(cycloheptane-1-carboxylate) (390 mg, 0.60 mmol) and NaOH (480 mg, 12 mmol) in 1:1:1 MeOH/H_2O/THF (15 mL) was placed under a nitrogen atmosphere and heated at reflux for 18 hours. After cooling to room temperature, the resulting residue was concentrated under a stream of nitrogen and acidified to pH ~7 with 3 M aqueous HCl solution. The suspension was washed with Et_2O × 3 and the organic washings were combined and concentrated under reduced pressure.

At this stage, we were surprised to observe a mixture of the desired trianionic ligand and phenol in the organic washings. To enable separation of the phenolate by-product, the dicarboxylate was esterified:

To a microwave vial charged with MeOH (5 mL) and c. H_2SO_4 (5 drops) was added the crude material. The resulting mixture was heated at 60 °C for 2 hours. After cooling to room temperature and concentration under reduced pressure, the mixture was dissolved in CH_2Cl_2 and washed with 10% aqueous NaOH solution × 3 to remove the phenolate by-product. The organic layer was dried over $MgSO_4$ and concentrated under reduced pressure to obtain the dimethyl ester (0.32 mmol, as determined through comparison of ^{1}H integration relative to 1,3,5-trimethoxybenzene).

To obtain the final ligand, the semi-purified dimethyl ester was fully hydrolysed:

A three-necked round-bottomed flask fitted with a reflux condenser charged with the dimethyl ester (0.32 mmol) and NaOH (253 mg, 6.32 mmol) in 1:1:1 MeOH/H_2O/THF (15 mL) was placed under a nitrogen atmosphere and heated at reflux for 18 hours. After cooling to room temperature, the resulting residue was concentrated under a stream of nitrogen and acidified to pH ~2 and NBu_4HSO_4 (80 mg, 0.24 mmol) and 3:1 $CHCl_3$/*i*PrOH were added. The phases were separated, the aqueous washed with 3:1 $CHCl_3$/*i*PrOH, organic phases combined, washed with H_2O, dried over $MgSO_4$ and concentrated under reduced pressure. The collected oil was triturated with Et_2O and further freeze dried to afford the title compound as a white solid (155 mg, 0.22 mmol, 37%).

^{1}H NMR (500 MHz, CD_3OD): δ 8.07 (dd, *J* = 7.7, 1.3 Hz, 1H, H-10), 7.42 (td, *J* = 7.4, 1.5 Hz, 1H, H-8), 7.36 (td, *J* = 7.7, 1.4 Hz, 1H, H-9), 7.20-7.16 (m, 3H, H-6, H-7), 6.91 (t, *J* = 1.5 Hz, 1H, H-5), 3.23 (m, 7.9H, H-11), 2.86 (s, 4H, H-

4), 2.03 (m, 4H, H-1A), 1.70-1.46 (m, 28H, H-1B, H-2, H-3, H-12), 1.41 (sext, *J* = 7.3 Hz, 10.5H, H-13), 1.02 (t, *J* = 7.4 Hz, 13.2H, H-14) ppm;

^{13}C NMR (126 MHz, CD_3OD): *δ* 181.4, 144.1, 142.31, 142.27, 137.5, 133.5, 131.9, 130.9, 130.8, 128.9, 127.7, 59.5 (ap. t, J_{C-N} = 2.9 Hz), 52.3, 47.8, 37.2, 31.1, 24.8, 24.6, 20.7 (ap. t, J_{C-N} = 1.5 Hz), 14.0 ppm;

HRMS (−ESI): *m/z* for dianion found 541.2273, $[C_{30}H_{37}O_7S]^{2-}$ requires 541.2265, (*δ* = + 1.5 ppm).

Bis[rhodium tetrabutylammonium 3',5'-bis((1-carboxycycloheptyl)methyl)-[1,1'-biphenyl]-2-sulfonate] (Rh_2(**D-III**)$_2$●(**NBu$_4$**)$_2$)

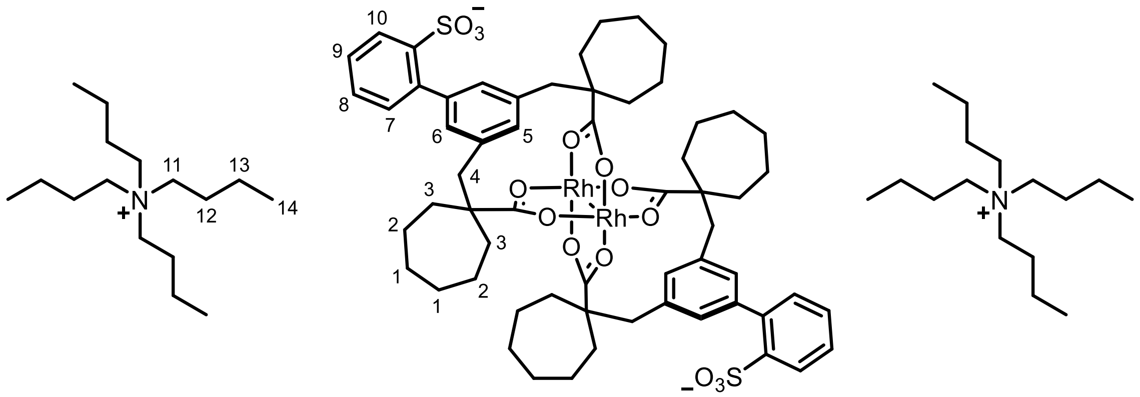

Prepared according to **GP2** on a 0.14 mmol scale with respect to $Rh_2(TFA)_4$ and tetrabutylammonium 3',5'-bis((1-carboxycycloheptyl)methyl)-[1,1'-biphenyl]-2-sulfonate as the ligand. The title compound was obtained as a green solid (43 mg, 0.024 mmol, 17%).

^{1}H NMR (500 MHz, CD_3OD): *δ* 8.06 (dd, *J* = 7.9, 1.2 Hz, 2H, H-10), 7.44 (td, *J* = 7.5, 1.3 Hz, 2H, H-8), 7.36 (td, *J* = 7.6, 1.4 Hz, 2H, H-9), 7.14 (dd, *J* = 7.5, 1.3 Hz, 2H, H-7), 6.97 (d, *J* = 1.6 Hz, 4H, H-6), 6.92 (t, *J* = 1.6 Hz, 2H, H-5), 3.23 (m, 17.3H, H-11), 2.72 (s, 8H, H-4), 1.84 (m, 8H, H-3A), 1.66 (m, 17.5H, H-12), 1.55-1.34 (m, 56H, H-1, H-2, H-3B, H-13), 1.02 (t, *J* = 7.4 Hz, 26.0H, H-14) ppm;

^{13}C NMR (126 MHz, CD_3OD): *δ* 197.5, 144.1, 142.6, 141.1, 137.9, 133.3, 131.6, 130.9, 130.0, 129.0, 127.6, 59.5 (ap. t, J_{C-N} = 2.9 Hz), 54.2, 47.3, 37.9, 32.3, 24.9, 24.8, 20.7 (ap. t, J_{C-N} = 1.4 Hz), 13.9 ppm;

HRMS (−ESI): *m/z* for dianion found 642.1152, $[C_{60}H_{70}O_{14}Rh_2S_2]^{2-}$ requires 642.1164, (*δ* = − 1.9 ppm);

HRMS (+ESI): *m/z* for cation found 242.2852, $[C_{16}H_{36}N]^+$ requires 242.2842, (*δ* = + 4.1 ppm).

Dimethyl 3,3'-(2'-(hydroxymethyl)-[1,1'-biphenyl]-3,5-diyl)bis(2,2-dimethylpropanoate)

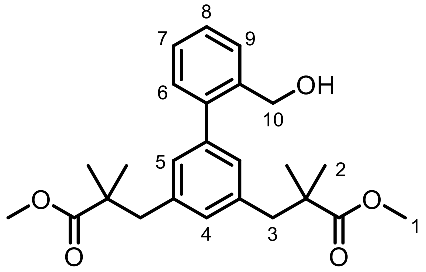

Between 2 oven-dried microwave vials was equally added 2-bromobenzyl alcohol (898 mg, 4.80 mmol), dimethyl 3,3'-(5-(4,4,5,5-tetramethyl-1,3,2-dioxaborolan-2-yl)-1,3-phenylene)bis(2,2-dimethylpropanoate) (1.38 g, 3.20 mmol), palladium acetate (36 mg, 0.16 mmol), SPhos (131 mg, 0.32 mmol) and K_3PO_4 (2.04 g, 9.60 mmol). The vials were placed under a nitrogen atmosphere and to each was added THF (8.0 mL) and H_2O (0.9 mL). The resulting mixtures were heated at 90 °C overnight. After cooling to room temperature, the crude mixtures were combined, diluted in EtOAc, filtered over Celite® and the filtrate concentrated under reduced pressure. Purification by flash column chromatography (SiO_2, 0-25% v/v EtOAc in hexane) afforded the title compound as a pale-yellow oil (1.21 g, 2.94 mmol, 92%).

***R_f* value**: 0.35 (30% v/v EtOAc in hexane);

^{1}H NMR (400 MHz, $CDCl_3$): δ 7.53 (dd, *J* = 7.5, 1.4 Hz, 1H, H-9), 7.37 (td, *J* = 7.4, 1.6 Hz, 1H, H-8), 7.33 (td, *J* = 7.5, 1.6 Hz, 1H, H-7), 7.21 (dd, *J* = 7.3, 1.5 Hz, 1H, H-6), 6.95 (d, *J* = 1.6 Hz, 2H, H-5), 6.87 (t, *J* = 1.6 Hz, 1H, H-4), 4.55 (d, *J* = 5.2 Hz, 2H, H-10), 3.66 (s, 6H, H-1), 2.86 (s, 4H, H-3), 1.88 (t, *J* = 5.1 Hz, 1H, O-H), 1.19 (s, 12H, H-2) ppm;

^{13}C NMR (101 MHz, $CDCl_3$): δ 178.1, 141.4, 140.3, 138.3, 137.6, 131.2, 130.0, 129.2, 128.7, 127.7, 127.6, 63.1, 51.9, 46.4, 43.8, 25.2 ppm;

HRMS (+ESI): *m/z* found $[M+Na]^+$ 435.2149, $[C_{25}H_{32}NaO_5]^+$ requires 435.2142, (δ = + 1.6 ppm).

Dimethyl 3,3'-(2'-(bromomethyl)-[1,1'-biphenyl]-3,5-diyl)bis(2,2-dimethylpropanoate)

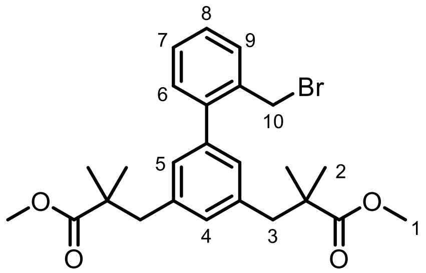

A round-bottomed flask was charged with tetrabromomethane (1.89 g, 5.70 mmol), placed under a nitrogen atmosphere and cooled to 0 °C. A solution of dimethyl 3,3'-(2'-(hydroxymethyl)-[1,1'-biphenyl]-3,5-diyl)bis(2,2-dimethylpropanoate) (1.17 g, 2.85 mmol) in CH_2Cl_2 (30 mL) was added following which triphenylphosphine (1.49 g, 5.70 mmol) in CH_2Cl_2 (10 mL) was added dropwise. The resulting mixture was gradually warmed to room

temperature overnight, concentrated under reduced pressure and purified by flash column chromatography (SiO_2, 0-15% v/v EtOAc in hexane) to afford the title compound as a colourless oil (748 mg, 1.57 mmol, 55%).

***R**$_f$* **value**: 0.24 (10% v/v EtOAc in hexane);

^{1}H NMR (400 MHz, $CDCl_3$): *δ* 7.51 (m, 1H, H-9), 7.37-7.30 (m, 2H, H-7, H-8), 7.19 (m, 1H, H-6), 7.04 (br. d, *J* = 1.3 Hz, 2H, H-5), 6.89 (br. t, *J* = 1.3 Hz, 1H, H-4), 4.45 (s, 2H, H-10), 3.66 (s, 6H, H-1), 2.88 (s, 4H, H-3), 1.20 (s, 12H, H-2) ppm;

^{13}C NMR (101 MHz, $CDCl_3$): *δ* 178.0, 142.2, 139.7, 137.8, 135.3, 131.3, 131.0, 130.5, 129.2, 128.6, 128.0, 51.9, 46.3, 43.8, 32.3, 25.2 ppm;

HRMS (+ESI): *m/z* found $[M+Na]^+$ 497.1296, $[C_{25}H_{31}BrNaO_4]^+$ requires 497.1298, (*δ* = − 0.4 ppm).

Tetrabutylammonium (3',5'-bis(3-methoxy-2,2-dimethyl-3-oxopropyl)-[1,1'-biphenyl]-2-yl)methanesulfonate

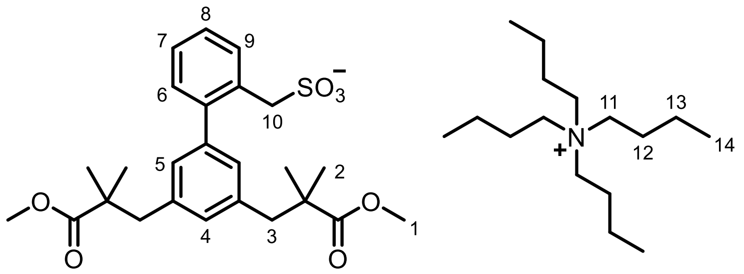

To a round-bottomed flask fitted with a reflux condenser was added dimethyl 3,3'-(2'-(bromomethyl)-[1,1'-biphenyl]-3,5-diyl)bis(2,2-dimethylpropanoate) (692 mg, 1.46 mmol), Na_2SO_3 (221 mg, 1.75 mmol) and 2:3 acetone/H_2O (15 mL). The resulting mixture was heated at reflux overnight, cooled to room temperature and concentrated under a stream of nitrogen. The aqueous residue was diluted in H_2O, transferred to a separating funnel and washed with Et_2O. NaOH (47 mg, 1.17 mmol) and NBu_4HSO_4 (397 mg, 1.17 mmol) were added to the remaining aqueous layer following which 3:1 $CHCl_3$/*i*PrOH was added. The phases were separated, the aqueous extracted with 3:1 $CHCl_3$/*i*PrOH, the organic phases combined, washed with H_2O, dried over $MgSO_4$ and concentrated under reduced pressure to afford the title compound as a colourless oil (700 mg, 0.97 mmol, 67%).

^{1}H NMR (400 MHz, $CDCl_3$): *δ* 7.95 (dd, *J* = 7.6, 1.6 Hz, 1H, H-9), 7.25-7.19 (m, 4H, H-5, H-6, H-8), 7.16 (td, *J* = 7.4, 1.7 Hz, 1H, H-7), 6.79 (t, *J* = 1.3 Hz, 1H, H-4), 4.07 (s, 2H, H-10), 3.64 (s, 6H, H-1), 3.22 (m, 8.1H, H-11), 2.85 (s, 4H, H-3), 1.58 (m, 8.6H, H-12), 1.37 (sext, *J* = 7.4 Hz, 8.7H, H-13), 1.16 (s, 12H, H-2), 0.96 (t, *J* = 7.4 Hz, 12.5H, H-14) ppm;

^{13}C NMR (101 MHz, $CDCl_3$): *δ* 178.4, 143.1, 140.8, 137.0, 132.6, 131.4, 130.43, 130.40, 130.0, 126.6, 126.3, 58.9, 54.0, 51.8, 46.2, 43.7, 25.0, 24.2, 19.8, 13.8 ppm;

HRMS (−ESI): *m/z* for anion found 475.1796, $[C_{25}H_{31}O_7S]^-$ requires 475.1796, (*δ* = 0.0 ppm).

Tetrabutylammonium (3',5'-bis(2-carboxy-2-methylpropyl)-[1,1'-biphenyl]-2-yl)methanesulfonate

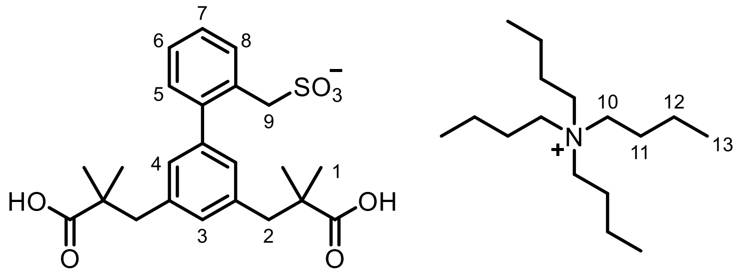

A three-necked round-bottomed flask fitted with a reflux condenser charged with tetrabutylammonium (3',5'-bis(3-methoxy-2,2-dimethyl-3-oxopropyl)-[1,1'-biphenyl]-2-yl)methanesulfonate (680 mg, 0.948 mmol) and NaOH (569 g, 14.2 mmol) in 1:1 MeOH/H_2O (20 mL) was placed under a nitrogen atmosphere and heated at reflux for 18 hours. After cooling to room temperature, the resulting residue was acidified to pH ~2 with 3 M aqueous HCl solution and extracted with 3:1 $CHCl_3$/*i*PrOH × 3. The organic phases were combined, dried over $MgSO_4$ and concentrated under reduced pressure. The collected oil was triturated with Et_2O and further freeze dried to afford the title compound as a white solid (500 mg, 0.73 mmol, 77%).

^{1}H NMR (400 MHz, CD_3OD): δ 7.78 (m, 1H, H-8), 7.33-7.25 (m, 2H, H-6, H-7), 7.24-7.19 (m, 3H, H-4, H-5), 7.01 (t, *J* = 1.4 Hz, 1H, H-3), 4.11 (s, 2H, H-9), 3.22 (m, 8.4H, H-10), 2.89 (s, 4H, H-2), 1.65 (m, 8.8H, H-11), 1.40 (sext, *J* = 7.4 Hz, 9.1H, H-12), 1.19 (s, 12H, H-1), 1.01 (t, *J* = 7.4 Hz, 13.0H, H-13) ppm;

^{13}C NMR (101 MHz, CD_3OD): δ 181.3, 144.5, 141.9, 138.9, 132.0, 131.9, 131.8, 131.3, 131.0, 128.1, 127.9, 59.5 (ap. t, $J_{C\text{-}N}$ = 2.8 Hz), 54.9, 47.2, 44.5, 25.6, 24.8, 20.7 (ap. t, $J_{C\text{-}N}$ = 1.5 Hz), 14.0 ppm;

HRMS (–ESI): *m/z* for anion found 447.1481, $[C_{23}H_{27}O_7S]^-$ requires 447.1483, (δ = – 0.4 ppm).

Bis[rhodium tetrabutylammonium (3',5'-bis(2-carboxy-2-methylpropyl)-[1,1'-biphenyl]-2-yl)methanesulfonate] (Rh_2(**A-IV**)$_2$●(**NBu$_4$**)$_2$)

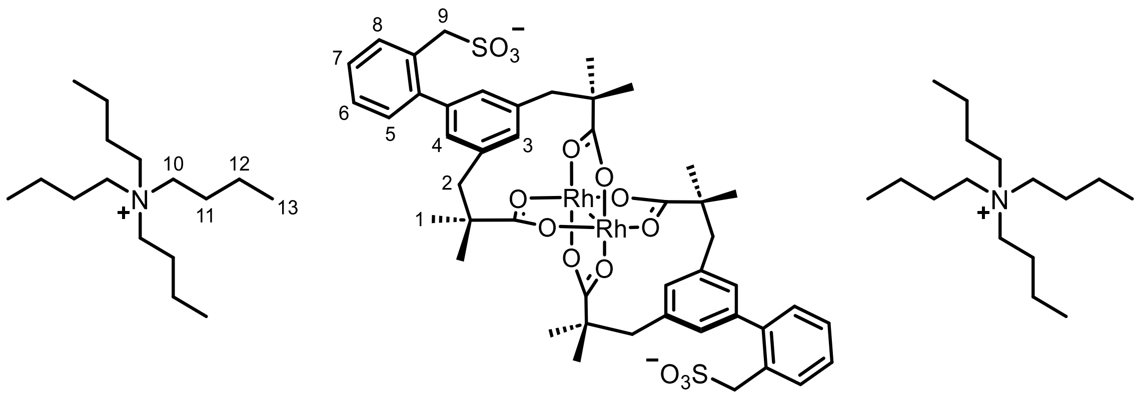

Prepared according to **GP2** on a 0.25 mmol scale with respect to $Rh_2(TFA)_4$ and tetrabutylammonium (3',5'-bis(2-carboxy-2-methylpropyl)-[1,1'-biphenyl]-2-yl)methanesulfonate as the ligand. The title compound was obtained as a green solid (218 mg, 0.138 mmol, 55%).

^{1}H NMR (700 MHz, CD_3OD) δ 7.76 (m, 2H, H-8), 7.29-7.25 (m, 4H, H-6, H-7), 7.15 (m, 2H, H-5), 7.01 (s, 6H, H-3, H-4), 4.03 (s, 4H, H-9), 3.22 (m, 15.9H, H-10), 2.68 (s, 8H, H-2), 1.65 (m, 16.0H, H-11), 1.40 (sext, J = 7.4 Hz, 16.1H, H-12), 1.02 (t, J = 7.4 Hz, 24.5H, H-13), 1.00 (s, 24H, H-1) ppm;

^{13}C NMR (176 MHz, CD_3OD) δ 197.2, 144.8, 140.8, 139.4, 132.1, 131.8, 131.2, 130.9, 130.6, 128.1, 127.7, 59.5 (ap. t, $J_{C\text{-}N}$ = 2.6 Hz), 54.8, 47.8, 46.9, 26.4, 24.8, 20.7, 13.9 ppm;

HRMS (−ESI): *m/z* for dianion found 548.0381, $[C_{46}H_{50}O_{14}Rh_2S_2]^{2-}$ requires 548.0382, (δ = − 0.2 ppm);

HRMS (+ESI): *m/z* for cation found 242.2847, $[C_{16}H_{36}N]^+$ requires 242.2842, (δ = + 2.1 ppm).

Phenyl 8-iodonaphthalene-2-sulfonate

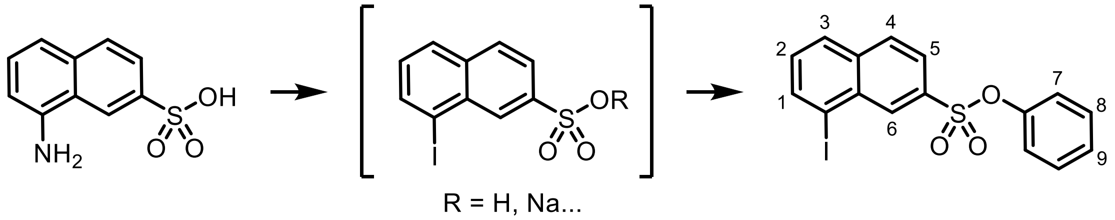

We are very grateful to Dr James L. Douthwaite who conducted the first part of the procedure below.

To a solution of KOH (3.36 g, 60 mmol) in H_2O (96 mL) was added 8-amino-2-naphthalenesulfonic acid (13.4 g, 60 mmol). The mixture was cooled to 0 °C and $NaNO_2$ (4.14 g, 60 mmol) was added portion-wise over 30 minutes. This solution was slowly added to a separate round-bottomed flask containing H_2SO_4 (1.0 M, 84 mL) at 0 °C. Slow addition is required to maintain the temperature at 0 °C. After completion of the addition (approx. 2 hours), KI (12.0 g, 72.2 mmol) in H_2O (60 mL) was added slowly at 0 °C following which the resulting mixture was warmed to room temperature and stirred for 2 hours, then heated to 100 °C and stirred for 1 hour. The reaction mixture was cooled to room temperature, treated with EtOH, concentrated to a slurry and filtered. The solids were washed with additional EtOH and the filtrate was collected and concentrated under reduced pressure. The product was filtered over a small silica pad eluting with EtOH and concentrated under reduced pressure to afford the intermediate aryl iodides as a brown solid (40% wt. purity, 25.1 mmol, 42%).

An oven-dried round-bottomed flask fitted with a reflux condenser was charged with the intermediate aryl iodides (4.43 mmol) and placed under a nitrogen atmosphere. MeCN (40 mL) and $POCl_3$ (1.24 mL, 13.3 mmol) were added and the reaction mixture was heated at 55 °C for 3 hours. After cooling to room temperature, the reaction was concentrated under a stream of nitrogen, diluted in H_2O and extracted with CH_2Cl_2 × 2. The combined organic layers were dried over $MgSO_4$ and concentrated under reduced pressure. To the crude sulfonyl chloride was added phenol (541 mg, 5.75 mmol) and the mixture was placed under a nitrogen

atmosphere. CH_2Cl_2 (40 mL) and NEt_3 (3.1 mL, 22.2 mmol) were added and the resulting mixture was stirred at room temperature overnight. The reaction was washed with saturated aqueous Na_2CO_3 solution, 10% aqueous NaOH solution, brine, the organic layer dried over $MgSO_4$ and concentrated under reduced pressure. Purification by flash column chromatography (SiO_2, 0-15% v/v EtOAc in hexane) afforded the title compound as an orange solid (1.36 g, 3.32 mmol, 75% *for this step*).

***R$_f$* value**: 0.42 (20% v/v EtOAc in hexane);

^{1}H NMR (500 MHz, $CDCl_3$): δ 8.63 (m, 1H, H-6), 8.21 (dd, *J* = 7.4, 0.9 Hz, 1H, H-1), 7.94-7.90 (m, 2H, H-3, H-4), 7.86 (dd, *J* = 8.6, 1.7 Hz, 1H, H-5), 7.38 (dd, *J* = 8.1, 7.6 Hz, 1H, H-2), 7.31-7.22 (m, 3H, H-8, H-9), 7.03 (m, 2H, H-7) ppm;

^{13}C NMR (101 MHz, $CDCl_3$): δ 149.8, 139.5, 136.4, 134.7, 134.4, 133.5, 130.5, 130.4, 129.9, 129.0, 127.4, 124.1, 122.4, 100.7 ppm;

HRMS (+ESI): *m/z* found $[M+Na]^+$ 432.9355, $[C_{16}H_{11}INaO_3S]^+$ requires 432.9366, (δ = − 2.5 ppm).

Dimethyl 3,3'-(5-(7-(phenoxysulfonyl)naphthalen-1-yl)-1,3-phenylene)bis(2,2-dimethylpropanoate)

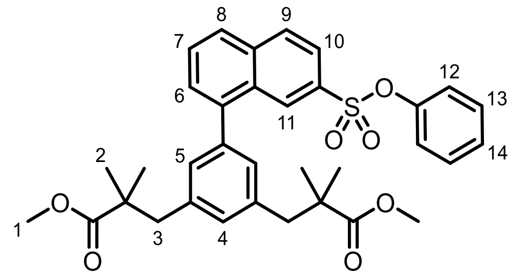

Between 2 oven-dried microwave vials was equally added phenyl 8-iodonaphthalene-2-sulfonate (957 mg, 2.33 mmol), dimethyl 3,3'-(5-(4,4,5,5-tetramethyl-1,3,2-dioxaborolan-2-yl)-1,3-phenylene)bis(2,2-dimethylpropanoate) (674 mg, 1.56 mmol), palladium acetate (18 mg, 0.078 mmol), SPhos (64 mg, 0.156 mmol) and K_3PO_4 (991 mg, 4.67 mmol). The vials were placed under a nitrogen atmosphere and to each was added THF (9.0 mL) and H_2O (1.0 mL). The resulting mixtures were heated at 90 °C overnight. After cooling to room temperature, the crude mixtures were combined, diluted in EtOAc, filtered over Celite® and the filtrate concentrated under reduced pressure. Purification by flash column chromatography (SiO_2, 0-20% v/v EtOAc in hexane) afforded the title compound as an off white solid (744 mg, 1.26 mmol, 81%).

***R$_f$* value**: 0.74 (40% v/v EtOAc in hexane);

^{1}H NMR (400 MHz, $CDCl_3$): δ 8.40 (d, *J* = 1.3 Hz, 1H, H-11), 8.04 (d, *J* = 8.8 Hz, 1H, H-9), 7.93 (d, *J* = 8.3 Hz, 1H, H-8), 7.86 (dd, *J* = 8.7, 1.8 Hz, 1H, H-10), 7.71 (dd, *J* = 8.1, 7.2 Hz, 1H, H-7), 7.51 (dd, *J* = 7.1, 0.8 Hz, 1H, H-6), 7.29-7.20 (m, 3H, H-13, H-14), 6.97-6.92 (m, 5H, H-4, H-5, H-12), 3.64 (s, 6H, H-1), 2.86 (s, 4H, H-3), 1.19 (s, 12H, H-2) ppm;

^{13}C NMR (101 MHz, $CDCl_3$): δ 178.1, 149.8, 142.3, 138.4, 138.2, 136.0, 132.6, 131.9, 130.3, 130.08, 130.06, 129.8, 129.2, 129.0, 128.9, 127.6, 127.3, 123.1, 122.5, 51.9 (d, J = 3.6 Hz), 46.2, 43.8, 25.1 ppm;

HRMS (+ESI): m/z found $[M+Na]^+$ 611.2080, $[C_{34}H_{36}NaO_7S]^+$ requires 611.2074, (δ = + 1.0 ppm).

Tetrabutylammonium 8-(3,5-bis(2-carboxy-2-methylpropyl)phenyl)naphthalene-2-sulfonate

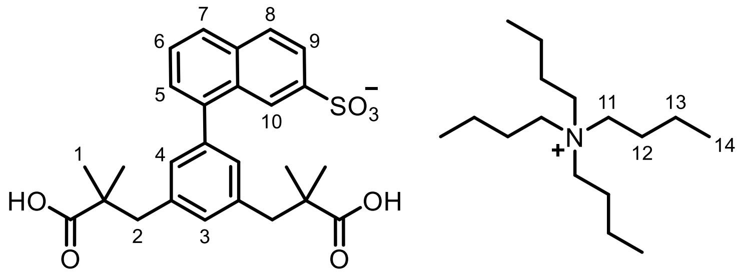

A three-necked round-bottomed flask fitted with a reflux condenser charged with dimethyl 3,3'-(5-(7-(phenoxysulfonyl)naphthalen-1-yl)-1,3-phenylene)bis(2,2-dimethylpropanoate) (684 mg, 1.16 mmol) and NaOH (326 mg, 8.14 mmol) in 1:1:1 MeOH/H_2O/THF (30 mL) was placed under a nitrogen atmosphere and heated at reflux for 18 hours. After cooling to room temperature, the resulting residue was concentrated under a stream of nitrogen and acidified to pH ~7 with 3 M aqueous HCl solution. The suspension was washed with Et_2O to remove the phenol by-product, the aqueous acidified further to pH ~2 and NBu_4HSO_4 (276 mg, 0.814 mmol) and 3:1 $CHCl_3$/*i*PrOH were added. The phases were separated, the aqueous extracted with 3:1 $CHCl_3$/*i*PrOH, the organic phases combined, washed with H_2O, dried over $MgSO_4$ and concentrated under reduced pressure. The collected oil was triturated with Et_2O and further freeze dried to afford the title compound as a white solid (600 mg, 0.826 mmol, 71%).

^{1}H NMR (400 MHz, CD_3OD): δ 8.47 (s, 1H, H-10), 7.99 (d, J = 8.7 Hz, 1H, H-8), 7.93-7.88 (m, 2H, H-7, H-9), 7.59 (dd, J = 8.1, 7.2 Hz, 1H, H-6), 7.45 (dd, J = 7.0, 0.8 Hz, 1H, H-5), 7.20 (d, J = 1.3 Hz, 2H, H-4), 7.11 (t, J = 1.4 Hz, 1H, H-3), 3.22 (m, 7.9H, H-11), 2.93 (s, 4H, H-2), 1.64 (m, 8.0H, H-12), 1.39 (sext, J = 7.5 Hz, 8.2H, H-13), 1.23 (s, 12H, H-1), 1.01 (t, J = 7.5 Hz, 12.0H, H-14) ppm;

^{13}C NMR (101 MHz, CD_3OD): δ 181.2, 143.7, 142.7, 140.9, 139.4, 135.9, 132.6, 131.8, 131.1, 129.8, 129.1, 128.4, 127.9, 124.7, 124.2, 59.5 (ap. t, J_{C-N} = 2.9 Hz), 47.3, 44.6, 25.7, 24.8, 20.7 (ap. t, J_{C-N} = 1.4 Hz), 13.9 ppm;

HRMS (–ESI): m/z found $[M]^-$ 483.1480, $[C_{26}H_{27}O_7S]^-$ requires 483.1483, (δ = – 0.6 ppm).

Bis[rhodium tetrabutylammonium 8-(3,5-bis(2-carboxy-2-methylpropyl)phenyl)naphthalene-2-sulfonate] (Rh_2(**A**-**V**)$_2$●(**NBu$_4$**)$_2$)

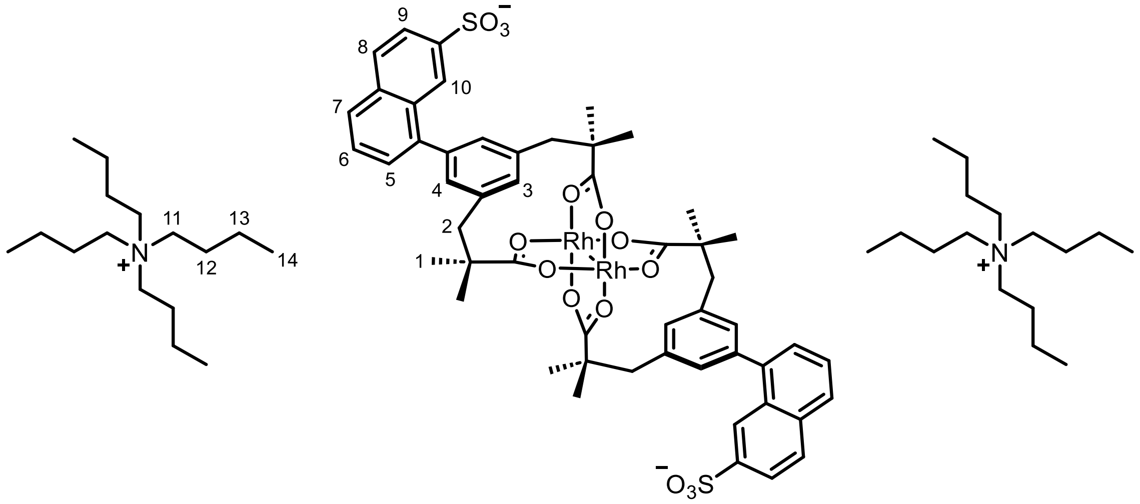

Prepared according to **GP2** on a 0.25 mmol scale with respect to $Rh_2(TFA)_4$ and tetrabutylammonium 8-(3,5-bis(2-carboxy-2-methylpropyl)phenyl)naphthalene-2-sulfonate as the ligand. The title compound was obtained as a green solid (175 mg, 0.106 mmol, 42%).

^{1}H NMR (700 MHz, CD_3OD): δ 8.43 (br. s, 2H, H-10), 7.97 (d, J = 8.6 Hz, 2H, H-8), 7.90-7.88 (m, 4H, H-7, H-9), 7.59 (dd, J = 8.2, 7.1 Hz, 2H, H-6), 7.40 (dd, J = 7.0, 1.0 Hz, 2H, H-5), 7.12 (t, J = 1.5 Hz, 2H, H-3), 6.97 (d, J = 1.5 Hz, 4H, H-4), 3.23 (m, 21.0H, H-11), 2.73 (br. s, 8H, H-2), 1.65 (m, 21.4H, H-12), 1.41 (sext, J = 7.5 Hz, 21.3H, H-13), 1.03 (s, 24H, H-1), 1.02 (t, J = 7.4 Hz, 31.5H, H-14) ppm;

^{13}C NMR (176 MHz, CD_3OD): δ 197.0, 143.6, 143.0, 139.9, 139.8, 135.9, 131.9, 131.7, 130.7, 129.7, 128.9, 128.3, 127.9, 124.9, 124.1, 59.5 (ap. t, $J_{C\text{-}N}$ = 2.5 Hz), 47.9, 46.8, 26.4, 24.8, 20.7, 13.9 ppm;

HRMS (−ESI): *m/z* for dianion found 584.0385, $[C_{52}H_{50}O_{14}Rh_2S_2]^{2-}$ requires 584.0382, (δ = + 0.5 ppm);

HRMS (+ESI): *m/z* for cation found 242.2845, $[C_{16}H_{36}N]^+$ requires 242.2842, (δ = + 1.2 ppm).

Synthesis of Chiral Rh(II,II) Tetracarboxylate Dimers

The syntheses of Rh_2(**A**-**I**)$_2$●(**Cat1**)$_2$(**pyr**)$_2$, Rh_2(**A**-**I**)$_2$●(**Cat2**)$_2$(**pyr**)$_2$, Rh_2(**B**-**I**)$_2$●(**Cat1**)$_2$(**pyr**)$_2$, Rh_2(**C**-**I**)$_2$●(**Cat1**)$_2$(**pyr**)$_2$, Rh_2(**D**-**I**)$_2$●(**Cat1**)$_2$(**pyr**)$_2$ and Rh_2(**A**-**I**)$_2$●(**Cat13**)$_2$ has been reported previously.[6]

Bis[rhodium (1S,2R,4S,5R)-5-ethyl-2-((S)-hydroxy(6-methoxyquinolin-4-yl)methyl)-1-((3,3'',5,5''-tetra-tert-butyl-[1,1':3',1''-terphenyl]-5'-yl)methyl)quinuclidin-1-ium (3,5-bis(2-carboxy-2-methylpropyl)benzenesulfonate)] (Rh_2(**A**-**II**)$_2$●(**Cat1**)$_2$)

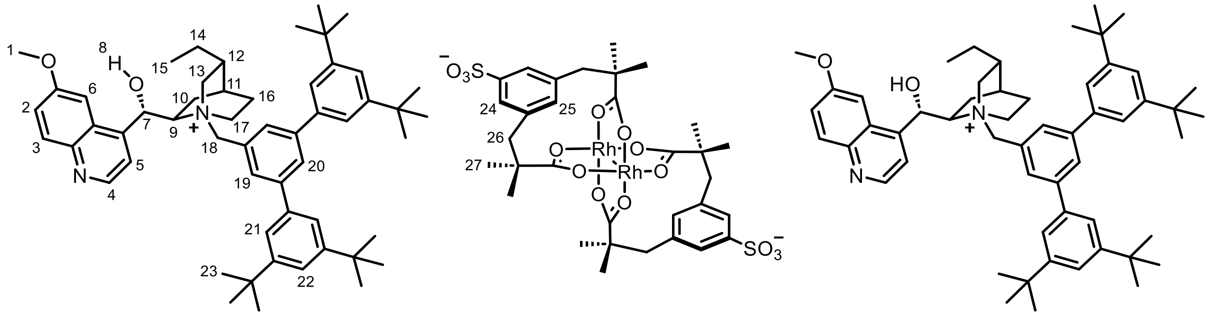

Prepared according to **GP3** on a 0.041 mmol scale with respect to Rh_2(**A**-**II**)$_2$●(NBu_4)$_2$ and using **Cat1**●Br as the chiral cation bromide salt. The title compound was isolated as a light brown powder (89 mg, 0.036 mmol, 87%).

^{1}H NMR (500 MHz, C_5D_5N): δ 9.06 (d, J = 4.5 Hz, 2H, H-4), 8.80 (d, J = 4.2 Hz, 2H, H-8), 8.62 (br. s, 4H, H-19), 8.36 (d, J = 9.2 Hz, 2H, H-3), 8.29 (br. s, 2H, H-20), 8.13 (d, J = 4.5 Hz, 2H, H-5), 7.93-7.94 (m, 14H, H-6, H-21, H-24), 7.72 (t, J = 1.6 Hz, 4H, H-22), 7.52 (dd, J = 9.2 Hz, 2.6 Hz, 2H, H-2), 7.46 (br. s, 2H, H-25), 7.35 (br. s, 2H, H-7), 6.28 (d, J = 12.4 Hz, 2H, H-18a), 5.72 (d, J = 12.4 Hz, 2H, H-18b), 4.60-4.67 (m, 4H, H-13a, H-17a), 4.41 (t, J = 9.3 Hz, 2H, H-9), 3.93-3.98 (m, 8H, H-1, H-13b), 3.45 (q, J = 10.1 Hz, 2H, H-17b), 2.62-2.70 (m, 10H, H-10a, H-26), 1.85 (q, J = 9.1 Hz, 2H, H-16a), 1.74 (br. s, 2H, H-11), 1.58-1.63 (m, 2H, H-16b), 1.42-1.52 (m, 78H, H-12, H-14, H-23), 1.12-1.18 (m, 2H, H-10b), 0.97 (s, 12H, H-27a), 0.95 (s, 12H, H-27b), 0.78 (t, J = 7.0 Hz, 6H, H-15) ppm;

^{13}C NMR (126 MHz, C_5D_5N): δ 197.3, 159.1, 152.5, 148.5, 147.8, 145.7, 145.1, 145.0, 141.1, 139.2, 133.0, 132.9, 132.1, 130.4, 129.4, 127.4, 126.9, 123.1, 122.9, 122.2, 121.7, 103.3, 69.5, 65.5, 64.5, 57.7, 57.0, 56.4, 47.5, 46.4, 36.5, 35.7, 32.1, 26.5-26.6 (m), 25.6, 25.3, 25.0, 22.0, 12.0 ppm;

HRMS (+ESI) *m/z* for cation found 793.5669, $[C_{55}H_{73}N_2O_2]^+$ requires 793.5667, (δ = + 0.3 ppm);

HRMS (−ESI) *m/z* for monoanion found 916.9881, $[C_{32}H_{38}O_{14}Rh_2S_2^{2-}+H^+]$ requires 916.9897, (δ = − 1.7 ppm);

$[\alpha]_D^{25.0}$ = + 31.9 (c. 0.09, C_5H_5N).

Bis[rhodium (1S,2S,4S,5R)-5-ethyl-2-((R)-hydroxy(6-methoxyquinolin-4-yl)methyl)-1-((3,3'',5,5''-tetra-tert-butyl-[1,1':3',1''-terphenyl]-5'-yl)methyl)quinuclidin-1-ium (3,5-bis(2-carboxy-2-methylpropyl)benzenesulfonate)] (Rh_2(**A-II**)$_2$●(**Cat2**)$_2$)

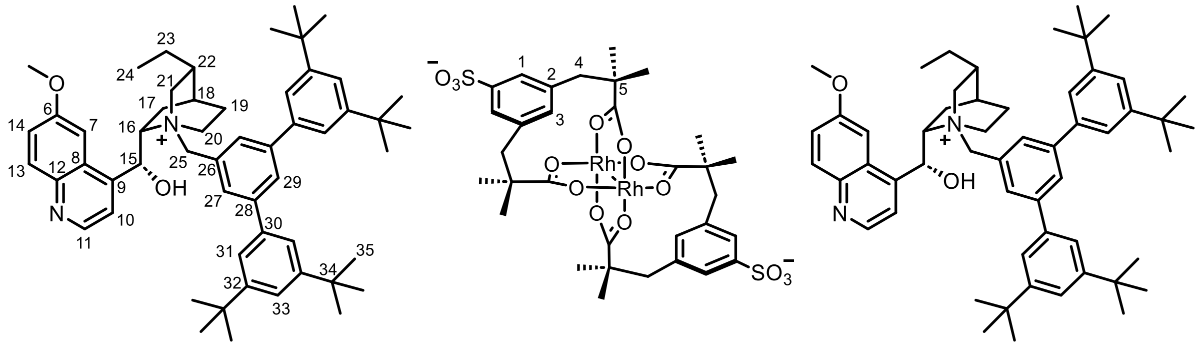

We are grateful to Dr Benjamin D. Williams for the preparation and characterisation of this compound.

Prepared according to **GP3** on a 0.050 mmol scale with respect to Rh_2(**A-II**)$_2$●(**NBu_4**)$_2$ and using **Cat2**●Br as the chiral cation bromide salt. The title compound was isolated as a brown powder (105 mg, 0.042 mmol, 84%).

^{1}H NMR (700 MHz, C_5D_5N): δ 9.08 (d, J = 4.4 Hz, 2H, H-11), 8.58 (s, 4H, H-27), 8.38 (br. d, J = 5.5 Hz, 2H, O-H), 8.33 (d, J = 9.2 Hz, 2H, H-13), 8.27 (t, J = 1.6 Hz, 2H, H-29), 8.05 (d, J = 4.4 Hz, 2H, H-10), 7.94 (d, J = 1.3 Hz, 8H, H-31), 7.91 (d, J = 1.2 Hz, 4H, H-1), 7.76 (d, J = 2.7 Hz, 2H, H-7), 7.74 (t, J = 1.7 Hz, 4H, H-33), 7.62 (d, J = 5.9 Hz, 2H, H-15), 7.46 (dd, J = 9.3, 2.6 Hz, 2H, H-14), 7.43 (t, J = 1.6 Hz, 2H, H-3), 6.90 (d, J = 12.0 Hz, 2H, H-25), 5.11 (d, J = 11.9 Hz, 2H, H-25'), 4.95–4.86 (m, 2H, H-20), 4.11 (dd, J = 11.0, 6.1 Hz, 2H, H-16), 4.02 (t, J = 11.5 Hz, 2H, H-21), 3.77 (s, 6H, OC<u>H</u>$_3$), 3.64–3.55 (m, 2H, H-21'), 3.32 (q, J = 10.4 Hz, 2H, H-20'), 2.59 (s, 8H, H-4), 2.52 (d, J = 14.0 Hz, 2H, H-17), 2.36 (q, J = 10.4 Hz, 2H, H-19), 1.82 (s, 2H, H-18), 1.69–1.61 (m, 2H, H-17'), 1.59–1.54 (m, 2H, H-22), 1.47 (s, 72H, H-35), 1.43–1.39 (m, 2H, H-19'), 1.27–1.15 (m, 4H, H-23), 0.93 (d, J = 5.7 Hz, 24H, C5C<u>H</u>$_3$), 0.72 (t, J = 7.3 Hz, 6H, H-24) ppm;

^{13}C NMR (176 MHz, C_5D_5N): δ 197.3, 159.0, 152.6, 148.6, 147.3, 145.7, 145.6, 145.2, 141.0, 139.3, 133.1, 132.8, 132.2, 130.1, 129.5, 127.0, 126.8, 123.1, 122.9, 122.2, 121.5, 102.6, 71.6, 65.3, 64.4, 63.0, 56.1, 51.8, 47.4, 46.3, 36.5, 35.7, 32.1, 26.6, 26.4, 26.1, 25.6, 21.1, 11.8 ppm;

HRMS (+ESI) *m/z* for cation found 793.5686, $[C_{55}H_{73}N_2O_2]^+$ requires 793.5667, (δ = + 2.4 ppm);

HRMS (−ESI) *m/z* for dianion found 457.9905, $[C_{32}H_{38}O_{14}Rh_2S_2]^{2-}$ requires 457.9912, (δ = − 1.5 ppm);

$[\alpha]_D^{25.0}$ = − 14.2 (c. 0.91, C_5H_5N).

Bis[rhodium (1S,2R,4S,5R)-5-ethyl-2-((S)-hydroxy(6-methoxyquinolin-4-yl)methyl)-1-((3,3'',5,5''-tetra-tert-butyl-[1,1':3',1''-terphenyl]-5'-yl)methyl)quinuclidin-1-ium (3',5'-bis(2-carboxy-2-methylpropyl)-[1,1'-biphenyl]-2-sulfonate)] (Rh_2(**A-III**)$_2$●(**Cat1**)$_2$)

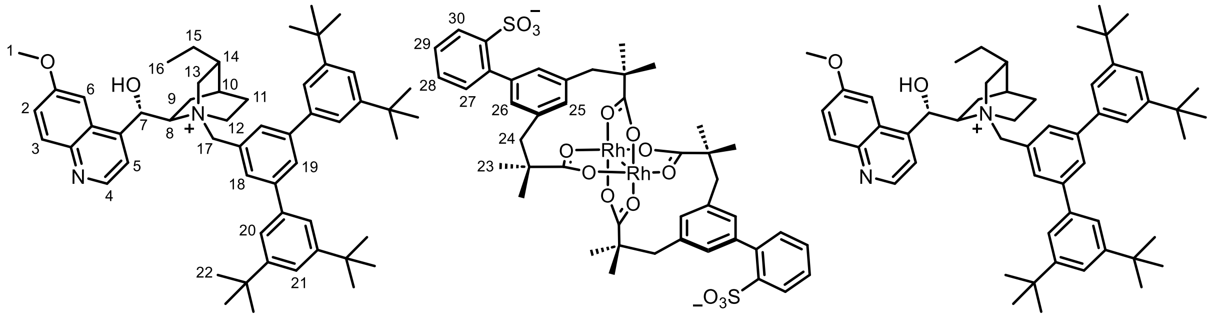

Prepared according to **GP3** on a 0.040 mmol scale with respect to Rh_2(**A-III**)$_2$●(**NBu$_4$**)$_2$ and using **Cat1**●Br as the chiral cation bromide salt. The title compound was isolated as a brown powder (77 mg, 0.029 mmol, 72%).

^{1}H NMR (400 MHz, C_5D_5N): δ 9.06 (d, J = 4.5 Hz, 2H, H-4), 8.81 (d, J = 7.6 Hz, 2H, H-30), 8.58 (m, 2H, O-H), 8.49 (s, 4H, H-18), 8.36 (d, J = 9.4 Hz, 2H, H-3), 8.29 (s, 2H, H-19), 8.06 (d, J = 4.5 Hz, 2H, H-5), 7.91 (s, 8H, H-20), 7.78 (s, 2H, H-6), 7.73 (s, 4H, H-21), 7.50 (d, J = 9.2 Hz, 2H, H-2), 7.44 (s, 4H, H-26), 7.30-7.19 (m, 6H, H-25, H-28, H-29), 7.16-7.12 (m, 4H, H-7, H-27), 6.13 (d, J = 12.3 Hz, H-17$_A$), 5.41 (d, J = 12.3 Hz, H-17$_B$), 4.58 (m, 2H, H-13$_A$), 4.40 (t, J = 10.7 Hz, 2H, H-12$_A$), 4.16 (t, J = 9.4 Hz, 2H, H-8), 3.91 (m, 2H, H-13$_B$), 3.77 (s, 6H, H-1), 3.39 (q, J = 9.8 Hz, 2H, H-12$_B$), 2.69 (t, J = 11.5 Hz, 2H, H-9$_A$), 2.56 (s, 8H, H-24), 1.83-1.72 (m, 4H, H-10, H-11$_A$), 1.65-1.48 (m, 8H, H-11$_B$, H-14, H-15), 1.44 (s, 72H, H-22), 1.16 (m, 2H, H-9$_B$), 1.02 (s, 12H, H-23$_A$), 1.00 (s, 12H, H-23$_B$), 0.80 (t, J = 6.3 Hz, 6H, H-16) ppm;

^{13}C NMR (101 MHz, C_5D_5N): δ 197.4, 158.9, 152.5, 148.5, 146.7, 145.7, 145.1, 145.0, 142.0, 141.1, 137.6, 133.0, 132.93, 132.86, 130.6, 130.3, 130.1, 129.41, 129.39, 129.35, 127.3, 126.9, 123.1, 122.9, 121.9, 121.7, 103.5, 69.9, 66.4, 64.5, 57.6, 56.8, 56.2, 47.6, 46.4, 36.5, 35.7, 32.1, 26.5 (d, J = 5.5 Hz), 25.6, 25.3, 25.0, 21.8, 12.0 ppm;

Note: an additional ^{13}C resonance is hidden underneath the solvent peak at 136.0 ppm;

HRMS (−ESI) *m/z* for dianion found 534.0218, $[C_{44}H_{46}O_{14}Rh_2S_2]^{2-}$ requires 534.0225, (δ = − 1.3 ppm);

HRMS (+ESI) *m/z* for cation found 793.5640, $[C_{55}H_{73}N_2O_2]^+$ requires 793.5667, (δ = − 3.4 ppm).

Optical rotation data was acquired as the pyridine-ligated catalyst:

$[\alpha]_D^{25.0}$ = + 184 (c. 0.1, $CHCl_3$).

Bis[rhodium (1S,2S,4S,5R)-5-ethyl-2-((R)-hydroxy(6-methoxyquinolin-4-yl)methyl)-1-((3,3'',5,5''-tetra-tert-butyl-[1,1':3',1''-terphenyl]-5'-yl)methyl)quinuclidin-1-ium (3',5'-bis(2-carboxy-2-methylpropyl)-[1,1'-biphenyl]-2-sulfonate)] (Rh_2(**A-III**)$_2$●(**Cat2**)$_2$)

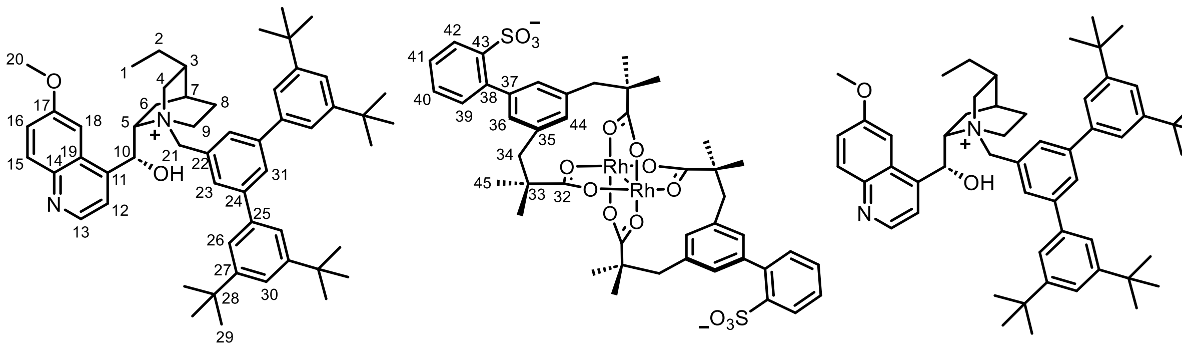

Prepared according to **GP3** on a 0.040 mmol scale with respect to Rh_2(**A-III**)$_2$●(**NBu$_4$**)$_2$ and using **Cat2**●Br as the chiral cation bromide salt. The title compound was isolated as a brown powder (73 mg, 0.027 mmol, 69%).

^{1}H NMR (500 MHz, C_5D_5N): δ 9.06 (d, J = 4.6 Hz, 2H, H-13), 8.74 (m, 2H, H-42), 8.48 (br. s, 2H, H-23), 8.31 (d, J = 9.2 Hz, 2H, H-15), 8.25 (br. s, 2H, H-31), 7.93 (d, J = 4.4 Hz, 2H, H-12), 7.87-7.83 (br. s, 10H, H-26, O-H), 7.70 (br. t, J = 1.7 Hz, 4H, H-30), 7.51 (d, J = 2.5 Hz, 2H, H-18), 7.44-7.40 (m, 4H, H-10, H-16), 7.40 (s, 4H, H-36), 7.24-7.21 (m, 4H, H-40, H-41), 7.12 (s, 2H, H-44), 7.06 (m, 2H, H-39), 6.89 (d, J = 12.1 Hz, 2H, H-21$_A$), 4.78-4.69 (m, 4H, H-9$_A$, H-21$_B$), 3.93 (t, J = 12.0 Hz, 2H, H-4$_A$), 3.81 (m, 2H, H-5), 3.61 (s, 6H, H-20), 3.32 (m, 2H, H-4$_B$), 3.23 (m, 2H, H-9$_B$), 2.56–2.43 (m, 10H, H-6$_A$, H-34), 2.30 (m, 2H, H-8$_A$), 1.78 (br. s, 2H, H-7), 1.59 (t, J = Hz, 2H, H-6$_B$), 1.51 (m, 2H, H-3), 1.42 -1.39 (m, 74H, H-8$_B$, H-29), 1.16 (p, J = 7.1 Hz, 4H, H-2), 1.00 (ap. d, J = 7.5 Hz, 24H, H-45), 0.71 (t, J = 7.3 Hz, 6H, H-1) ppm;

^{13}C NMR (126 MHz, C_5D_5N): δ 196.5, 158.0, 151.7, 147.6, 145.1, 145.1, 144.7, 144.3, 141.1, 141.0, 140.1, 136.7, 132.2, 132.1, 132.0, 129.6, 129.4, 129.4, 129.3, 128.8, 128.6, 128.5, 128.4, 126.1, 126.1, 122.2, 121.3, 120.5, 101.6, 71.4, 64.5, 62.9, 61.9, 55.2, 50.7, 46.7, 45.5, 35.5, 34.8, 31.2, 25.7, 25.1, 24.8, 20.0, 10.9 ppm;

HRMS (−ESI): *m/z* for dianion found 534.0199, $[C_{44}H_{46}O_{14}Rh_2S_2]^{2-}$ requires 534.0225, (δ = − 4.9 ppm);

HRMS (+ESI): *m/z* for cation found 793.5640, $[C_{55}H_{73}N_2O_2]^+$ requires 793.5667, (δ = − 3.4 ppm);

Optical rotation data was acquired as the pyridine-ligated catalyst:

$[\alpha]_D^{25.0}$ = − 7.6 (c. 0.11, $CHCl_3$).

Bis[rhodium (1S,2R,4S,5R)-5-ethyl-2-((S)-hydroxy(6-methoxyquinolin-4-yl)methyl)-1-((3,3'',5,5''-tetra-tert-butyl-[1,1':3',1''-terphenyl]-5'-yl)methyl)quinuclidin-1-ium (3',5'-bis((1-carboxycyclobutyl)methyl)-[1,1'-biphenyl]-2-sulfonate)] (Rh_2(**B-III**)$_2$●(**Cat1**)$_2$)

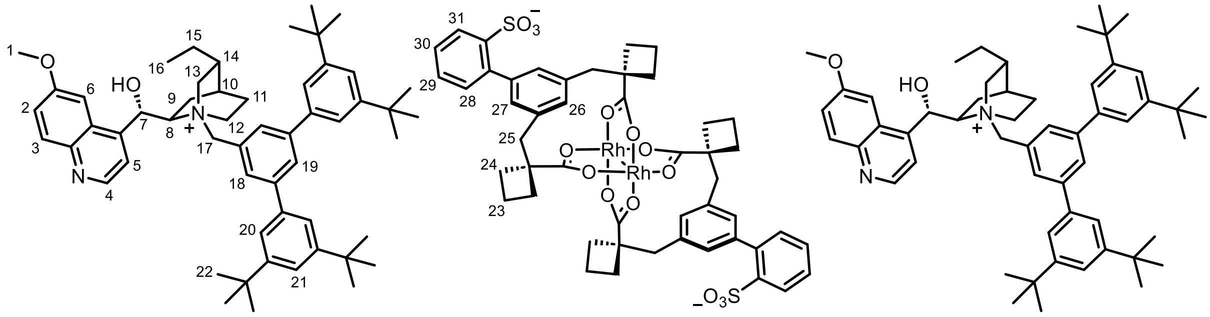

Prepared according to **GP3** on a 0.040 mmol scale with respect to Rh_2(**B-III**)$_2$●(**NBu$_4$**)$_2$ and using **Cat1**●Br as the chiral cation bromide salt. The title compound was isolated as a brown powder (78 mg, 0.029 mmol, 72%).

^{1}H NMR (500 MHz, C_5D_5N): δ 9.10 (d, J = 4.4 Hz, 2H, H-4), 8.81 (dd, J = 7.7, 1.1 Hz, 2H, H-31), 8.61 (br. s, 2H, O-H), 8.50 (d, J = 1.1 Hz, 4H, H-18), 8.39 (d, J = 9.2 Hz, 2H, H-3), 8.30 (t, J = 1.3 Hz, 2H, H-19), 8.08 (d, J = 4.5 Hz, 2H, H-5), 7.91 (d, J = 1.8 Hz, 8H, H-20), 7.78 (d, J = 2.5 Hz, 2H, H-6), 7.73 (t, J = 1.7 Hz, 4H, H-21), 7.54 (dd, J = 9.3, 2.6 Hz, 2H, H-2), 7.51 (d, J = 1.3 Hz, 4H, H-27), 7.26 (s, 2H, H-26), 7.25 (m, 2H, H-30), 7.21 (m, 2H, H-7), 7.16 (td, J = 7.5, 1.3 Hz, 2H, H-29), 6.84 (d, J = 7.6 Hz, 2H, H-28), 6.18 (d, J = 12.3 Hz, 2H, H-17$_A$), 5.44 (d, J = 12.3 Hz, 2H, H-17$_B$), 4.60 (m, 2H, H-13$_A$), 4.40 (m, 2H, H-12$_A$), 4.16 (t, J = 9.5 Hz, 2H, H-8), 3.94 (m, 2H, H-13$_B$), 3.81 (s, 6H, H-1), 3.42 (m, 2H, H-12$_B$), 2.88 (s, 8H, H-25), 2.71 (m, 2H, H-9$_A$), 2.30 (p, J = 8.3 Hz, 8H, H-24$_A$), 1.84-1.71 (m, 12H, H-10, H-11$_A$, H-24$_B$), 1.71-1.61 (m, 6H, H-11$_B$, H-23$_A$), 1.60-1.50 (m, 10H, H-14, H-15, H-23$_B$), 1.45 (s, 72H, H-22), 1.18 (m, 2H, H-9$_B$), 0.81 (t, J = 7.1 Hz, 6H, H-16) ppm;

^{13}C NMR (126 MHz, C_5D_5N): δ 196.7, 159.0, 152.5, 148.6, 146.7, 145.7, 145.11, 145.08, 142.6, 141.8, 141.1, 137.5, 133.03, 132.97, 132.8, 130.3, 129.9, 129.44, 129.39, 129.3, 129.2, 127.3, 126.8, 123.1, 122.9, 121.9, 121.7, 103.5, 69.9, 66.2, 64.7, 57.7, 56.9, 56.3, 52.0, 44.5, 36.5, 35.7, 32.1, 31.2, 25.7, 25.3, 25.1, 21.8, 16.2, 12.0 ppm;

HRMS (–ESI): *m/z* for dianion found 558.0218 $[C_{48}H_{46}O_{14}Rh_2S_2]^{2-}$ requires 558.0225, (δ = – 1.3 ppm);

HRMS (+ESI): *m/z* for cation found 793.5640, $[C_{55}H_{73}N_2O_2]^+$ requires 793.5667, (δ = – 3.4 ppm);

Optical rotation data was acquired as the pyridine-ligated catalyst:

$[\alpha]_D^{25.0}$ = + 112 (c. 0.1, $CHCl_3$).

Bis[rhodium (1S,2R,4S,5R)-5-ethyl-2-((S)-hydroxy(6-methoxyquinolin-4-yl)methyl)-1-((3,3'',5,5''-tetra-tert-butyl-[1,1':3',1''-terphenyl]-5'-yl)methyl)quinuclidin-1-ium (3',5'-bis((1-carboxycyclopentyl)methyl)-[1,1'-biphenyl]-2-sulfonate)] (Rh_2(**C-III**)$_2$●(**Cat1**)$_2$)

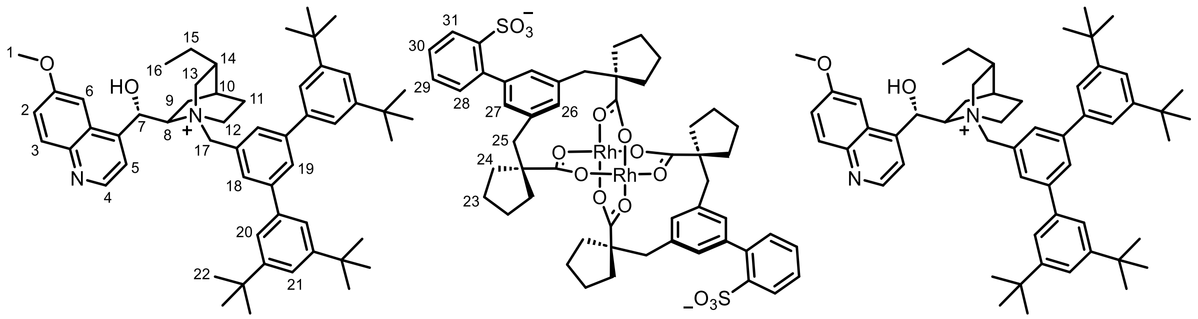

Prepared according to **GP3** on a 0.040 mmol scale with respect to Rh_2(**C-III**)$_2$●(**NBu$_4$**)$_2$ and using **Cat1**●Br as the chiral cation bromide salt. The title compound was isolated as a brown powder (85 mg, 0.031 mmol, 77%).

^{1}H NMR (500 MHz, C_5D_5N): δ 9.06 (d, J = 4.6 Hz, 2H, H-4), 8.81 (dd, J = 7.8, 1.2 Hz, 2H, H-31), 8.57 (d, J = 4.2 Hz, 2H, O-H), 8.46 (s, 4H, H-18), 8.37 (d, J = 9.2 Hz, 2H, H-3), 8.29 (s, 2H, H-19), 8.04 (d, J = 4.5 Hz, 2H, H-5), 7.91 (d, J = 1.8 Hz, 8H, H-20), 7.77 (d, J = 2.6 Hz, 2H, H-6), 7.73 (t, J = 1.7 Hz, 4H, H-21), 7.51 (dd, J = 9.3, 2.6 Hz, 2H, H-2), 7.44 (s, 4H, H-27), 7.28-7.24 (m, 4H, H-26, H-30), 7.18-7.12 (m, 4H, H-7, H-29), 7.01 (d, J = 7.6 Hz, 2H, H-28), 6.11 (d, J = 12.5 Hz, 2H, H-17$_A$), 5.39 (d, J = 12.5 Hz, 2H, H-17$_B$), 4.57 (m, 2H, H-13$_A$), 4.39 (t, J = 11.2 Hz, 2H, H-12$_A$), 4.14 (t, J = 9.3 Hz, 2H, H-8), 3.91 (m, 2H, H-13$_B$), 3.77 (s, 6H, H-1), 3.38 (q, J = 9.0 Hz, 2H, H-12$_B$), 2.74-2.62 (m, 10H, H-9$_A$, H-25), 1.99 (m, 8H, H-24$_A$), 1.83-1.73 (m, 4H, H-10, H-11$_A$), 1.60 (m, 2H, H-11$_B$), 1.51 (m, 6H, H-14, H-15), 1.48-1.30 (m, 96H, H-22, H-23, H-24$_B$), 1.17 (m, 2H, H-9$_B$), 0.80 (t, J = 7.4 Hz, 6H, H-16) ppm;

^{13}C NMR (126 MHz, C_5D_5N): δ 197.4, 158.9, 152.5, 148.5, 146.6, 145.7, 145.04, 144.99, 142.4, 142.0, 141.1, 138.2, 133.1, 132.9, 132.8, 130.3, 130.0, 129.8, 129.4, 129.34, 129.31, 127.3, 126.9, 123.1, 122.9, 121.9, 121.7, 103.5, 69.9, 66.3, 64.5, 58.7, 57.6, 56.8, 56.2, 45.7, 37.0, 36.5, 35.7, 32.1, 25.7, 25.32, 25.29, 25.1, 21.8, 12.0 ppm;

HRMS (−ESI): *m/z* for dianion found 586.0520 $[C_{52}H_{54}O_{14}Rh_2S_2]^{2-}$ requires 586.0538, (δ = − 1.3 ppm);

HRMS (+ESI): *m/z* for cation found 793.5640, $[C_{55}H_{73}N_2O_2]^+$ requires 793.5667, (δ = − 3.4 ppm);

Optical rotation data was acquired as pyridine-ligated catalyst:

$[\alpha]_D^{25.0}$ = + 128 (c. 0.1, $CHCl_3$).

Bis[rhodium (1S,2R,4S,5R)-5-ethyl-2-((S)-hydroxy(6-methoxyquinolin-4-yl)methyl)-1-((3,3'',5,5''-tetra-tert-butyl-[1,1':3',1''-terphenyl]-5'-yl)methyl)quinuclidin-1-ium (3',5'-bis((1-carboxycycloheptyl)methyl)-[1,1'-biphenyl]-2-sulfonate)] (Rh_2(**D-III**)$_2$●(**Cat1**)$_2$)

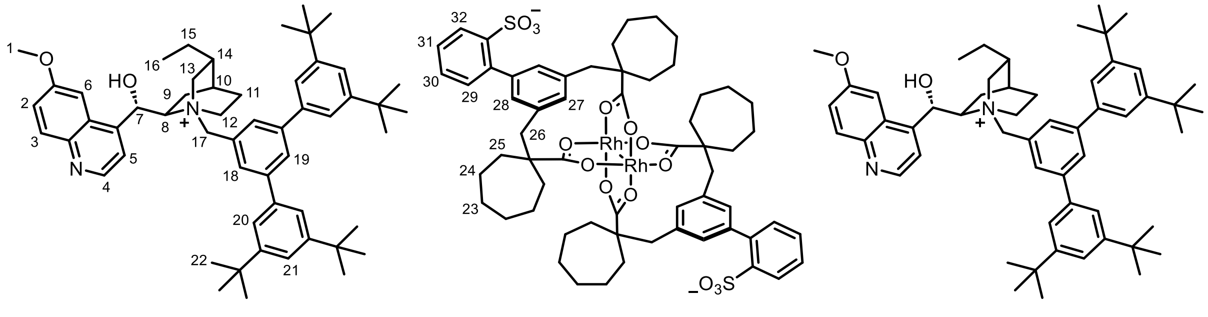

Prepared according to **GP3** on a 0.016 mmol scale with respect to Rh_2(**D-III**)$_2$●(**NBu$_4$**)$_2$ and using **Cat1**●Br as the chiral cation bromide salt. The title compound was isolated as a brown powder (36 mg, 0.013 mmol, 78%).

^{1}H NMR (500 MHz, C_5D_5N): δ 9.06 (d, J = 4.4 Hz, 2H, H-4), 8.81 (dd, J = 7.7, 1.2 Hz, 2H, H-32), 8.45 (s, 4H, H-18), 8.36 (d, J = 9.1 Hz, 2H, H-3), 8.30 (t, J = 1.4 Hz, 2H, H-19), 8.03 (d, J = 4.5 Hz, 2H, H-5), 7.90 (d, J = 1.7 Hz, 8H, H-20), 7.78 (d, J = 2.6 Hz, 2H, H-6), 7.74 (t, J = 1.7 Hz, 4H, H-21), 7.50 (dd, J = 9.2, 2.6 Hz, 2H, H-2), 7.41 (s, 4H, H-28), 7.25 (m, 2H, H-31), 7.22 (m, 2H, H-27), 7.15-7.09 (m, 4H, H-7, H-30), 6.96 (d, J = 7.6 Hz, 2H, H-29), 6.11 (d, J = 12.4 Hz, 2H, H-17$_A$), 5.36 (d, J = 12.4 Hz, 2H, H-17$_B$), 4.59 (m, 2H, H-13$_A$), 4.39 (m, 2H, H-12$_A$), 4.16 (t, J = 9.2 Hz, 2H, H-8), 3.90 (m, 2H, H-13$_B$), 3.79 (s, 6H, H-1), 3.37 (q, J = 9.5 Hz, 2H, H-12$_B$), 2.71-2.58 (m, 10H, H-9$_A$, H-26), 1.99 (m, 8H, H-25$_A$), 1.83-1.70 (m, 4H, H-10, H-11$_A$), 1.63-1.23 (m, 120H, H-11$_B$, H-14, H-15, H-22, H-23, H-24, H-25$_B$), 1.15 (m, 2H, H-9$_B$), 0.80 (t, J = 6.8 Hz, 6H, H-16) ppm;

^{13}C NMR (126 MHz, C_5D_5N): δ 197.6, 158.9, 152.5, 148.5, 146.7, 145.7, 145.1, 145.0, 142.0, 141.9, 141.1, 137.3, 133.1, 133.0, 132.8, 130.9, 130.4, 130.1, 129.3, 127.3, 126.8, 123.1, 122.9, 121.8, 121.7, 103.6, 69.9, 66.3, 64.5, 57.6, 56.8, 56.2, 53.7, 47.3, 37.7, 36.5, 35.7, 32.1, 31.6, 25.7, 25.3, 25.1, 24.5, 21.8, 12.0 ppm;

Note: there are two additional ^{13}C resonances underneath the peak at 129.3 ppm;

HRMS (−ESI): *m/z* for dianion found 642.1160 $[C_{60}H_{70}O_{14}Rh_2S_2]^{2-}$ requires 642.1164, (δ = − 0.6 ppm);

HRMS (+ESI): *m/z* for cation found 793.5640, $[C_{55}H_{73}N_2O_2]^+$ requires 793.5667, (δ = − 3.4 ppm);

Optical rotation data was acquired as the pyridine-ligated catalyst:

$[\alpha]_D^{25.0}$ = + 88.3 (c. 0.1, $CHCl_3$).

Bis[rhodium (1S,2R,4S,5R)-1-([1,1':3',1''-terphenyl]-5'-ylmethyl)-5-ethyl-2-((S)-hydroxy(6-methoxyquinolin-4-yl)methyl)quinuclidin-1-ium (3',5'-bis(2-carboxy-2-methylpropyl)-[1,1'-biphenyl]-2-sulfonate)] (Rh_2(**A-III**)$_2$●(**Cat3**)$_2$)

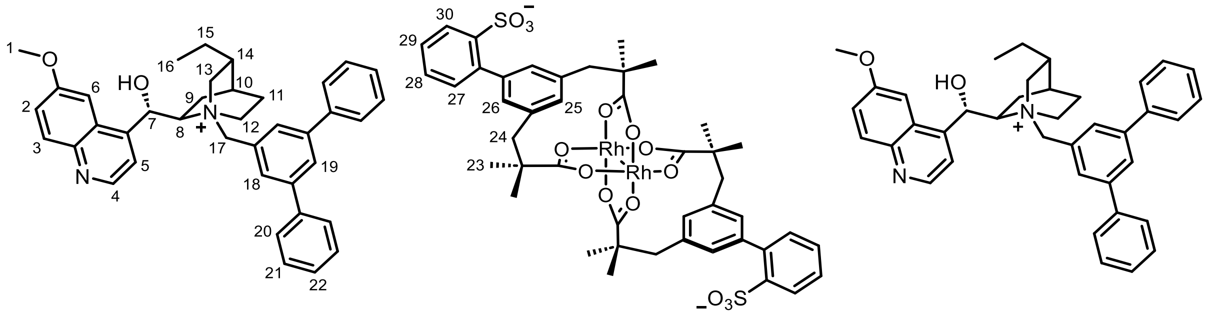

Prepared according to **GP3** on a 0.032 mmol scale with respect to Rh_2(**A-III**)$_2$●(**NBu$_4$**)$_2$ and using **Cat3**●Br as the chiral cation bromide salt. The title compound was isolated as a brown powder (57 mg, 0.026 mmol, 81%).

^{1}H NMR (700 MHz, C_5D_5N): δ 9.07 (d, J = 4.1 Hz, 2H, H-4), 8.76 (m, 2H, H-30), 8.61 (d, J = 3.3 Hz, O-H), 8.36 (d, J = 9.3 Hz, 2H, H-3), 8.34 (s, 4H, H-18), 8.15 (s, 2H, H-19), 8.09 (d, J = 4.2 Hz, 2H, H-5), 7.94 (d, J = 7.6 Hz, 8H, H-20), 7.84 (s, 2H, H-6), 7.54 (t, J = 7.6 Hz, 8H, H-21), 7.51 (dd, J = 9.2, 2.6 Hz, 2H, H-2), 7.48 (s, 4H, H-26), 7.46 (t, J = 7.4 Hz, 4H, H-22), 7.30 (s, 2H, H-25), 7.20 (m, 6H, H-7 H-28, H-29), 7.15 (m, 2H, H-27), 6.01 (d, J = 12.3 Hz, 2H, H-17$_A$), 5.42 (d, J = 12.3 Hz, 2H, H-17$_B$), 4.55 (m, 2H, H-13$_A$), 4.38 (t, J = 11.1 Hz, 2H, H-12$_A$), 4.22 (t, J = 9.0 Hz, 2H, H-8), 3.84-3.79 (m, 8H, H-1, H-13$_B$), 3.37 (q, J = 9.8 Hz, 2H, H-12$_B$), 2.66 (t, J = 10.9 Hz, 2H, H-9$_A$), 2.60 (m, 8H, H-24), 1.85 (q, J = 9.9 Hz, 2H, H-11$_A$), 1.77 (s, 2H, H-10), 1.65 (t, J = 10.2 Hz, 2H, H-11$_B$), 1.57-1.46 (m, 6H, H-14, H-15), 1.16 (m, 2H, H-9$_B$), 1.02 (s, 12H, H-23$_A$), 1.02 (s, 12H, H-23$_B$), 0.76 (t, J = 7.3 Hz, 6H, H-16) ppm;

^{13}C NMR (176 MHz, C_5D_5N): δ 197.4, 159.0, 148.6, 146.8, 145.7, 144.8, 143.4, 142.0, 141.9, 140.7, 137.7, 133.1, 133.0, 132.4, 130.6, 130.4, 130.2, 130.0, 129.5, 129.3, 128.9, 128.4, 128.3, 127.4, 126.9, 121.8, 121.7, 103.5, 69.7, 66.6, 64.5, 57.8, 57.0, 56.3, 47.7, 46.4, 36.5, 26.6, 25.29, 25.25, 24.8, 21.8, 12.0 ppm;

HRMS (−ESI): *m/z* for dianion found 534.0226, $[C_{44}H_{46}O_{14}Rh_2S_2]^{2-}$ requires 534.0225, (δ = + 0.2 ppm);

HRMS (+ESI): *m/z* for cation found 569.3165, $[C_{39}H_{41}N_2O_2]^+$ requires 569.3163, (δ = + 0.4 ppm).

Optical rotation data was acquired as the pyridine-ligated catalyst:

$[\alpha]_D^{25.0}$ = + 57.9 (c. 0.1, pyridine).

Bis[rhodium (1S,2R,4S,5R)-5-ethyl-2-((S)-hydroxy(6-methoxyquinolin-4-yl)methyl)-1-((3,3'',5,5''-tetrakis(triethylsilyl)-[1,1':3',1''-terphenyl]-5'-yl)methyl)quinuclidin-1-ium (3',5'-bis(2-carboxy-2-methylpropyl)-[1,1'-biphenyl]-2-sulfonate)] (Rh_2(**A-III**)$_2$●(**Cat4**)$_2$) (**Rh1**)

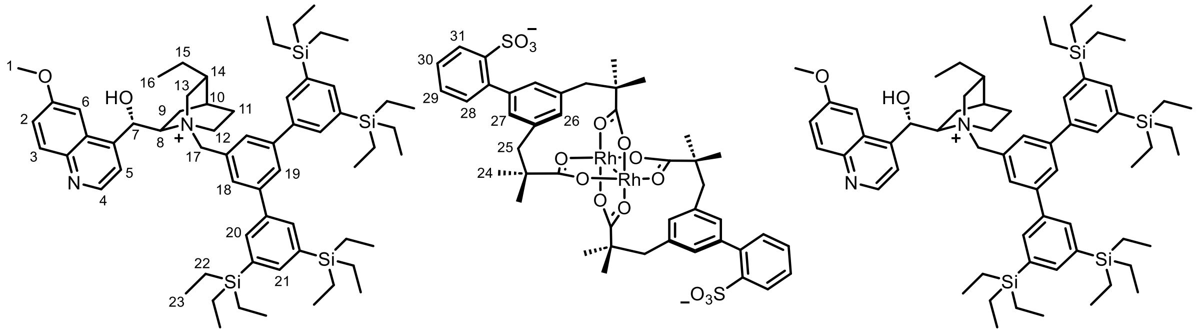

Prepared according to **GP3** on a 0.040 mmol scale with respect to Rh_2(**A-III**)$_2$●(**NBu$_4$**)$_2$ and using **Cat4**●Br as the chiral cation bromide salt. The title compound was isolated as a brown powder (107 mg, 0.034 mmol, 86%).

^{1}H NMR (700 MHz, C_5D_5N): δ 9.05 (d, J = 4.5 Hz, 2H, H-4), 8.82 (d, J = 7.6 Hz, 2H, H-31), 8.62 (br. s, 2H, O-H), 8.56 (s, 4H, H-18), 8.35 (d, J = 9.2 Hz, 2H, H-3), 8.30 (s, 2H, H-19), 8.25 (s, 8H, H-20), 8.03 (m, 2H, H-5), 8.00 (s, 4H, H-21), 7.79 (s, 2H, H-6), 7.50 (dd, J = 8.9, 2.2 Hz, 2H, H-2), 7.44 (s, 4H, H-27), 7.30 (t, J = 7.3 Hz, 2H, H-30), 7.25 (t, J = 7.4 Hz, 2H, H-29), 7.22 (s, 2H, H-26), 7.15 (d, J = 7.3 Hz, 2H, H-28), 7.09 (br. s, 2H, H-7), 6.06 (d, J = 12.1 Hz, 2H, H-17$_A$), 5.44 (d, J = 12.2 Hz, 2H, H-17$_B$), 4.56 (m, 2H, H-13$_A$), 4.38 (t, J = 10.7 Hz, 2H, H-12$_A$), 4.19 (t, J = 8.7 Hz, 2H, H-8), 3.90 (t, J = 9.3 Hz, 2H, H-13$_B$), 3.81 (s, 6H, H-1), 3.36 (q, J = 9.8 Hz, 2H, H-12$_B$), 2.67 (t, J = 12.0 Hz, 2H, H-9$_A$), 2.54 (m, 8H, H-25), 1.80-1.72 (m, 4H, H-10, H-11$_A$), 1.59-1.48 (m, 8H, H-11$_B$, H-14, H-15), 1.16 (m, 2H, H-9$_B$), 1.08 (t, J = 7.5 Hz, 72H, H-23), 1.03-0.94 (m, 72H, H-22, H-24), 0.83 (t, J = 7.0 Hz, 6H, H-16) ppm;

^{13}C NMR (176 MHz, C_5D_5N): δ 197.3, 158.9, 148.5, 146.7, 145.7, 144.9, 144.4, 142.1, 142.0, 140.8, 140.0, 138.2, 137.6, 134.9, 133.0, 132.9, 132.7, 130.7, 130.6, 130.0, 129.4, 127.4, 122.0, 121.7, 103.5, 69.7, 66.5, 64.5, 57.6, 56.9, 56.3, 47.7, 46.4, 36.5, 26.5 (d, J = 11.0 Hz), 25.6, 25.3, 25.0, 21.9, 12.0, 8.2, 4.2 ppm;

Note: there are two additional ^{13}C resonances underneath the signal at 129.4 ppm;

HRMS (−ESI): *m/z* for dianion found 534.0233, $[C_{44}H_{46}O_{14}Rh_2S_2]^{2-}$ requires 534.0225, (δ = + 1.5 ppm);

HRMS (+ESI): *m/z* for cation found 1025.6632, $[C_{63}H_{97}N_2O_2Si_4]^+$ requires 1025.6622, (δ = + 1.0 ppm).

Optical rotation data was acquired as the pyridine-ligated catalyst:

$[\alpha]_D^{25.0}$ = + 162 (c. 0.1, $CHCl_3$).

Bis[rhodium (1S,2S,4S,5R)-5-ethyl-2-((R)-hydroxy(6-methoxyquinolin-4-yl)methyl)-1-((3,3'',5,5''-tetrakis(triethylsilyl)-[1,1':3',1''-terphenyl]-5'-yl)methyl)quinuclidin-1-ium (3',5'-bis(2-carboxy-2-methylpropyl)-[1,1'-biphenyl]-2-sulfonate)] (Rh_2(**A-III**)$_2$●(**Cat5**)$_2$) (**Rh2**)

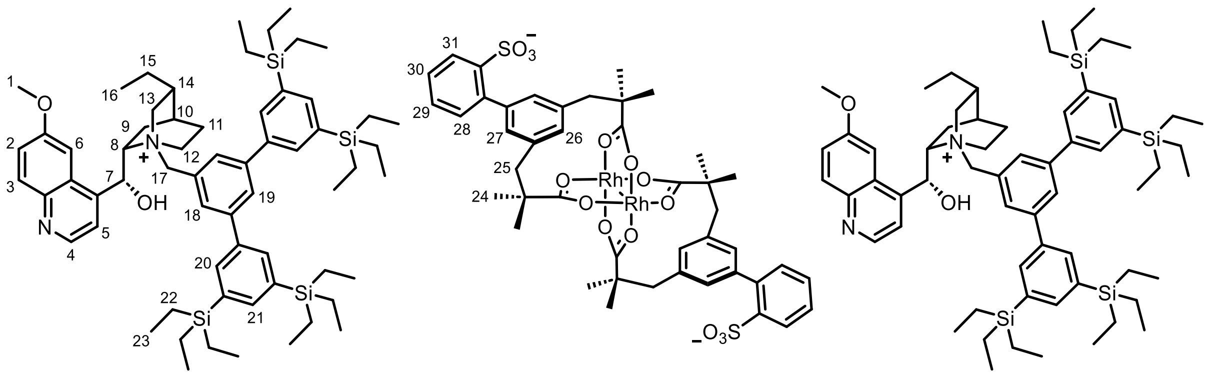

Prepared according to **GP3** on a 0.040 mmol scale with respect to Rh_2(**A-III**)$_2$●(**NBu**$_4$)$_2$ and using **Cat5**●Br as the chiral cation bromide salt. The title compound was isolated as a brown powder (113 mg, 0.036 mmol, 90%).

^{1}H NMR (700 MHz, C_5D_5N): δ 9.08 (d, J = 4.4 Hz, 2H, H-4), 8.80 (dd, J = 7.8, 1.5 Hz, 2H, H-31), 8.59 (br. s, 4H, H-18), 8.33 (d, J = 9.2 Hz, H-3), 8.29 (t, J = 1.5 Hz, 2H, H-19), 8.24 (s, 8H, H-20), 8.00 (t, J = 1.0 Hz, 4H, H-21), 7.93-7.89 (m, 4H, H-5, O-H), 7.55 (d, J = 2.6 Hz, 2H, H-6), 7.45 (dd, J = 9.1, 2.7 Hz, 2H, H-2), 7.42 (d, J = 6.0 Hz, 2H, H-7), 7.40 (d, J = 1.2 Hz, 4H, H-27), 7.30 (td, J = 7.5, 1.3 Hz, 2H, H-30), 7.26 (td, J = 7.4, 1.3 Hz, 2H, H-29), 7.11 (dd, J = 7.3, 1.1 Hz, 2H, H-28), 7.09 (t, J = 1.4 Hz, 2H, H-26), 6.82 (d, J = 12.3 Hz, 2H, H-17$_A$), 4.83 (d, J = 12.3 Hz, 2H, H-17$_B$), 4.78 (t, J = 11.2 Hz, 2H, H-12$_A$), 3.93 (m, 2H, H-13$_A$), 3.87 (m, 2H, H-8), 3.63 (s, 6H, H-1), 3.39 (m, 2H, H-13$_B$), 3.31 (m, 2H, H-12$_B$), 2.55-2.44 (m, 10H, H-9$_A$, H-25), 2.30 (m, 2H, H-11$_A$), 1.81 (br. s, 2H, H-10), 1.63 (t, J = 11.8 Hz, 2H, H-9$_B$), 1.53 (m, 2H, H-14), 1.39 (m, 2H, H-11$_B$), 1.20 (p, J = 7.5 Hz, 4H, H-15), 1.07 (t, J = 7.5 Hz, 72H, H-23), 1.02 (s, 12H, H-24$_A$), 1.02 (s, 12H, H-24$_B$), 0.96 (q, J = 7.6 Hz, 48H, H-22), 0.74 (t, J = 7.4 Hz, 6H, H-16) ppm;

^{13}C NMR (176 MHz, C_5D_5N): δ 197.2, 158.9, 148.4, 146.0, 145.8, 145.6, 144.5, 142.1, 141.7, 140.9, 140.0, 138.2, 137.6, 134.8, 133.1, 132.9, 132.6, 130.6, 130.4, 130.2, 129.63, 129.59, 129.3, 127.02, 126.98, 122.1, 121.3, 102.6, 72.2, 65.5, 63.9, 62.9, 56.1, 51.7, 47.6, 46.3, 36.5, 26.6, 26.4, 26.0, 25.4, 20.9, 11.8, 8.2, 4.2 ppm;

HRMS (−ESI): *m/z* for dianion found 534.0214, $[C_{44}H_{46}O_{14}Rh_2S_2]^{2-}$ requires 534.0225, (δ = − 2.1 ppm);

HRMS (+ESI): *m/z* for cation found 1025.6632, $[C_{63}H_{97}N_2O_2Si_4]^+$ requires 1025.6622, (δ = + 1.0 ppm).

Optical rotation data was acquired as the pyridine-ligated catalyst:

$[\alpha]_D^{25.0}$ = − 48.6 (c. 0.06, $CHCl_3$).

Bis[rhodium (1S,2R,4S,5R)-5-ethyl-2-((S)-hydroxy(6-methoxyquinolin-4-yl)methyl)-1-((3,3'',5,5''-tetra-tert-butyl-[1,1':3',1''-terphenyl]-5'-yl)methyl)quinuclidin-1-ium ((3',5'-bis(2-carboxy-2-methylpropyl)-[1,1'-biphenyl]-2-yl)methanesulfonate)] (Rh_2(**A-IV**)$_2$●(**Cat1**)$_2$)

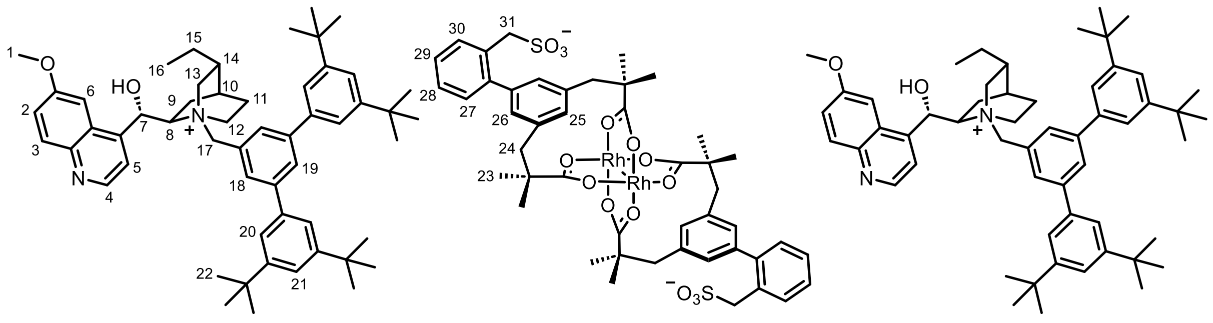

Prepared according to **GP3** on a 0.030 mmol scale with respect to Rh_2(**A-IV**)$_2$●(**NBu$_4$**)$_2$ and using **Cat1●**Br as the chiral cation bromide salt. The title compound was isolated as a brown powder (67 mg, 0.025 mmol, 83%).

^{1}H NMR (700 MHz, C_5D_5N): δ 9.05 (d, J = 4.2 Hz, 2H, H-4), 8.67 (s, 2H, O-H), 8.55 (s, 4H, H-18), 8.38-8.32 (m, 4H, H-3, H-30), 8.28 (s, 2H, H-19), 8.08 (s, 2H, H-5), 7.92 (s, 8H, H-20), 7.82 (s, 2H, H-6), 7.74 (s, 4H, H-21), 7.49-7.44 (m, 8H, H-2, H-25, H-26), 7.22 (m, 2H, H-27), 7.19 (s, 2H, H-7), 7.14 (t, J = 7.2 Hz, 2H, H-28), 7.08 (m, 2H, H-29), 6.27 (d, J = 11.7 Hz, 2H, H-17$_A$), 5.42 (d, J = 11.9 Hz, 2H, H-17$_B$), 4.64-4.57 (m, 6H, H-31, H-13$_A$), 4.39 (t, J = 11.0 Hz, 2H, H-12$_A$), 4.23 (t, J = 8.5 Hz, 2H, H-8), 3.91 (t, J = 11.0 Hz, 2H, H-13$_B$), 3.83 (s, 6H, H-1), 3.40 (q, J = 9.6 Hz, 2H, H-12$_B$), 2.76 (s, 8H, H-24), 2.68 (t, J = 11.6 Hz, 2H, H-9$_A$), 1.80 (q, J = 9.3 Hz, 2H, H-11$_A$), 1.73 (s, 2H, H-10), 1.57 (m, 2H, H-11$_B$), 1.53-1.40 (m, 78H, H-14, H-15, H-22), 1.16 (m, 2H, H-9$_B$), 1.07 (s, 12H, H-23$_A$), 1.06 (s, 12H, H-23$_B$), 0.77 (t, J = 6.6 Hz, 6H, H-16) ppm;

^{13}C NMR (176 MHz, C_5D_5N): δ 197.4, 158.9, 152.5, 148.6, 145.7, 145.1, 145.0, 144.1, 141.1, 140.8, 139.1, 133.7, 133.0, 132.9, 132.4, 130.9, 130.6, 130.4, 130.3, 129.4, 127.3, 127.1, 127.0, 123.1, 122.9, 121.7, 103.5, 70.0, 66.4, 64.6, 57.7, 56.8, 56.2, 55.3, 47.7, 46.5, 36.5, 35.7, 32.1, 26.6, 25.6, 25.4, 25.0, 21.8, 12.0 ppm;

Note: there is an additional ^{13}C resonance underneath the peak at 121.7 ppm.

HRMS (−ESI): *m/z* for dianion found 548.0380, $[C_{46}H_{50}O_{14}Rh_2S_2]^{2-}$ requires 548.0382, (δ = − 0.4 ppm);

HRMS (+ESI): *m/z* for cation found 793.5640, $[C_{55}H_{73}N_2O_2]^+$ requires 793.5667, (δ = − 3.4 ppm);

$[\alpha]_D^{25.0}$ = + 125 (c. 0.1, $CHCl_3$).

Bis[rhodium (1S,2S,4S,5R)-5-ethyl-2-((R)-hydroxy(6-methoxyquinolin-4-yl)methyl)-1-((3,3'',5,5''-tetra-tert-butyl-[1,1':3',1''-terphenyl]-5'-yl)methyl)quinuclidin-1-ium ((3',5'-bis(2-carboxy-2-methylpropyl)-[1,1'-biphenyl]-2-yl)methanesulfonate)] (Rh_2(**A-IV**)$_2$●(**Cat2**)$_2$)

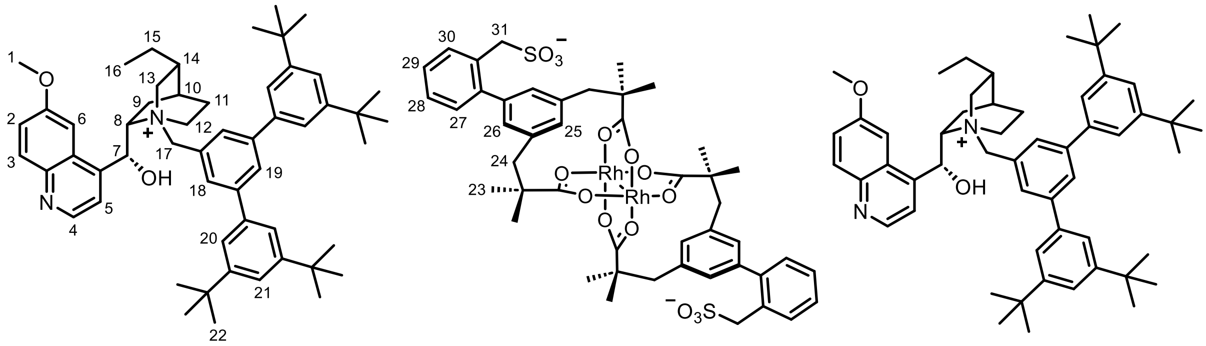

Prepared according to **GP3** on a 0.030 mmol scale with respect to Rh_2(**A-IV**)$_2$●(**NBu_4**)$_2$ and using **Cat2**●Br as the chiral cation bromide salt. The title compound was isolated as a brown powder (62 mg, 0.023 mmol, 77%).

^{1}H NMR (700 MHz, C_5D_5N): δ 9.09 (d, J = 4.3 Hz, 2H, H-4), 8.52 (br. s, 4H, H-18), 8.34 (d, J = 9.3 Hz, 2H, H-3), 8.29 (s, 2H, H-19), 8.24 (d, J = 7.8 Hz, 2H, H-30), 8.18 (d, J = 5.3 Hz, 2H, O-H), 8.01 (m, 2H, H-5), 7.91 (s, 8H, H-20), 7.76 (s, 4H, H-21), 7.50-7.47 (s, 4H, H-6, H-25), 7.46-7.41 (m, 6H, H-2, H-26), 7.33 (m, 2H, H-7), 7.14 (d, J = 7.6 Hz, 2H, H-27), 6.93 (t, J = 7.1 Hz, H-28), 6.89 (d, J = 11.5 Hz, 2H, H-17$_A$), 6.85 (t, J = 7.2 Hz, 2H, H-29), 4.89 (t, J = 11.2 Hz, 2H, H-12$_A$), 4.85 (d, J = 12.0 Hz, 2H, H-17$_B$), 4.63 (d, J = 13.6 Hz, 2H, H-31$_A$), 4.58 (d, J = 13.6 Hz, 2H, H-31$_B$), 3.96 (t, J = 11.8 Hz, 2H, H-13$_A$), 3.91 (m, 2H, H-8), 3.67 (s, 6H, H-1), 3.41 (m, 2H, H-13$_B$), 3.19 (q, J = 7.2 Hz, 2H, H-12$_B$), 2.78 (br. s, 8H, H-24), 2.46 (m, 2H, H-9$_A$), 2.32 (m, 2H, H-11$_A$), 1.76 (s, 2H, H-10), 1.58 (m, 2H, H-9$_B$), 1.53-1.41 (m, 76H, H-11$_B$, H-14, H-22), 1.16 (m, 4H, H-15), 1.05 (s, 24H, H-23), 0.71 (t, J = 7.3 Hz, 6H, H-16) ppm;

^{13}C NMR (176 MHz, C_5D_5N): δ 197.4, 158.8, 152.6, 148.6, 145.92, 145.87, 145.5, 145.2, 144.0, 141.1, 140.8, 139.1, 133.2, 133.0, 132.8, 132.1, 130.8, 130.6, 130.4, 130.2, 129.5, 127.0, 126.9, 123.1, 123.0, 121.9, 121.5, 102.6, 72.0, 65.1, 63.9, 62.9, 56.0, 55.2, 51.5, 47.7, 46.5, 36.4, 35.7, 32.1, 26.6, 26.5, 25.9, 25.6, 20.9, 11.7 ppm;

HRMS (−ESI): *m/z* for dianion found 548.0392, $[C_{46}H_{50}O_{14}Rh_2S_2]^{2-}$ requires 548.0382, (δ = + 1.8 ppm);

HRMS (+ESI): *m/z* for cation found 793.5640, $[C_{55}H_{73}N_2O_2]^+$ requires 793.5667, (δ = − 3.4 ppm);

Optical rotation data was acquired as the pyridine-ligated catalyst:

$[\alpha]_D^{25.0}$ = − 42.4 (c. 0.06, $CHCl_3$).

Bis[rhodium (1S,2R,4S,5R)-5-ethyl-2-((S)-hydroxy(6-methoxyquinolin-4-yl)methyl)-1-((3,3'',5,5''-tetra-tert-butyl-[1,1':3',1''-terphenyl]-5'-yl)methyl)quinuclidin-1-ium (8-(3,5-bis(2-carboxy-2-methylpropyl)phenyl)naphthalene-2-sulfonate)] (Rh_2(**A**-**V**)$_2$●(**Cat1**)$_2$)

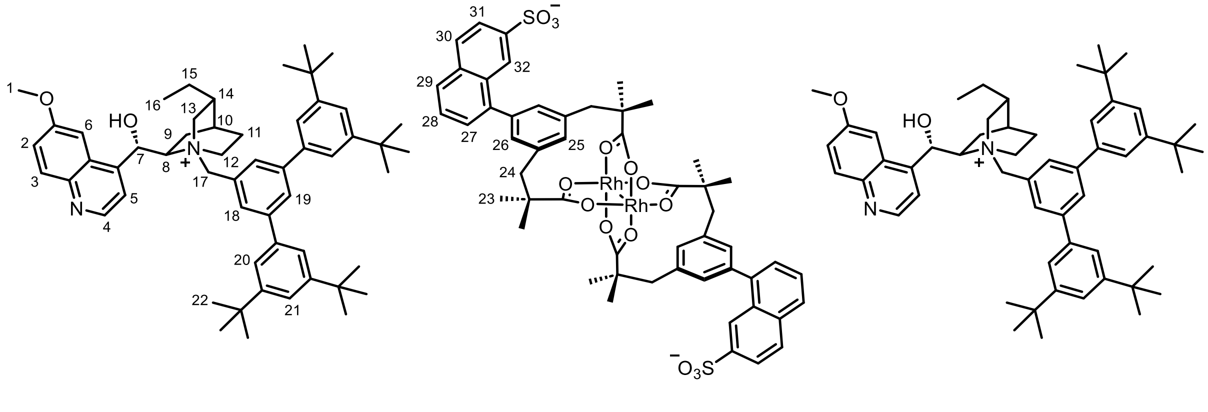

Prepared according to **GP3** on a 0.035 mmol scale with respect to Rh_2(**A**-**V**)$_2$●(**NBu₄**)$_2$ and using **Cat1**●Br as the chiral cation bromide salt. The title compound was isolated as a brown powder (88 mg, 0.032 mmol, 91%).

^{1}H NMR (700 MHz, C_5D_5N): δ 9.21 (s, 2H, H-32), 9.03 (d, J = 4.3 Hz, 2H, H-4), 8.80 (s, 2H, O-H), 8.60-8.54 (m, 6H, H-18, H-31), 8.33 (d, J = 8.9 Hz, 2H, H-3), 8.29 (s, 2H, H-19), 8.13 (s, 2H, H-5), 7.94-7.89 (m, 12H, H-6, H-20, H-30), 7.83 (d, J = 8.1 Hz, 2H, H-29), 7.73 (t, J = 1.6 Hz, 4H, H-21), 7.51-7.44 (m, 6H, H-2, H-25, H-28), 7.31-7.26 (m, 4H, H-7, H-27), 7.12 (s, 4H, H-26), 6.19 (d, J = 12.1 Hz, 2H, H-17$_A$), 5.68 (d, J = 12.1 Hz, 2H, H-17$_B$), 4.65 (m, 2H, H-13$_A$), 4.57 (t, J = 10.5 Hz, 2H, H-12$_A$), 4.38 (t, J = 8.7 Hz, 2H, H-8), 3.99 (t, J = 10.8 Hz, 2H, H-13$_B$), 3.87 (s, 6H, H-1), 3.46 (q, J = 9.3 Hz, 2H, H-12$_B$), 3.08-2.41 (m, 10H, H-9$_A$, H-24), 1.88 (q, J = 10.0 Hz, 2H, H-11$_A$), 1.77 (s, 2H, H-10), 1.62 (m, 2H, H-11$_B$), 1.57-1.50 (m, 6H, H-14, H-15), 1.44 (s, 72H, H-22), 1.19-1.06 (s, 26H, H-9$_B$, H-23), 0.80 (t, J = 7.2 Hz, 6H, H-16) ppm;

^{13}C NMR (176 MHz, C_5D_5N): δ 197.4, 158.9, 152.5, 148.5, 146.9, 145.7, 145.1, 144.7, 142.4, 141.1, 139.7, 139.5, 134.9, 133.0, 132.8, 131.9, 131.1, 130.5, 130.3, 129.5, 128.9, 128.6, 127.9, 127.3, 126.8, 125.6, 124.5, 123.1, 122.9, 121.8, 121.7, 103.4, 69.7, 66.9, 64.5, 57.8, 57.1, 56.3, 47.6, 46.5, 36.5, 35.7, 32.0, 26.6 (br. s), 25.7, 25.3, 25.1, 21.9, 12.0 ppm;

HRMS (−ESI): *m/z* for dianion found 584.0386, $[C_{52}H_{50}O_{14}Rh_2S_2]^{2-}$ requires 584.0382, (δ = + 0.7 ppm);

HRMS (+ESI): *m/z* for cation found 793.5640, $[C_{55}H_{73}N_2O_2]^+$ requires 793.5667, (δ = − 3.4 ppm);

Optical rotation data was acquired as the pyridine-ligated catalyst:

$[\alpha]_D^{25.0}$ = + 72.9 (c. 0.09, $CHCl_3$).

Bis[rhodium (1S,2S,4S,5R)-5-ethyl-2-((R)-hydroxy(6-methoxyquinolin-4-yl)methyl)-1-((3,3'',5,5''-tetra-tert-butyl-[1,1':3',1''-terphenyl]-5'-yl)methyl)quinuclidin-1-ium (8-(3,5-bis(2-carboxy-2-methylpropyl)phenyl)naphthalene-2-sulfonate)] (Rh_2(**A-V**)$_2$●(**Cat2**)$_2$)

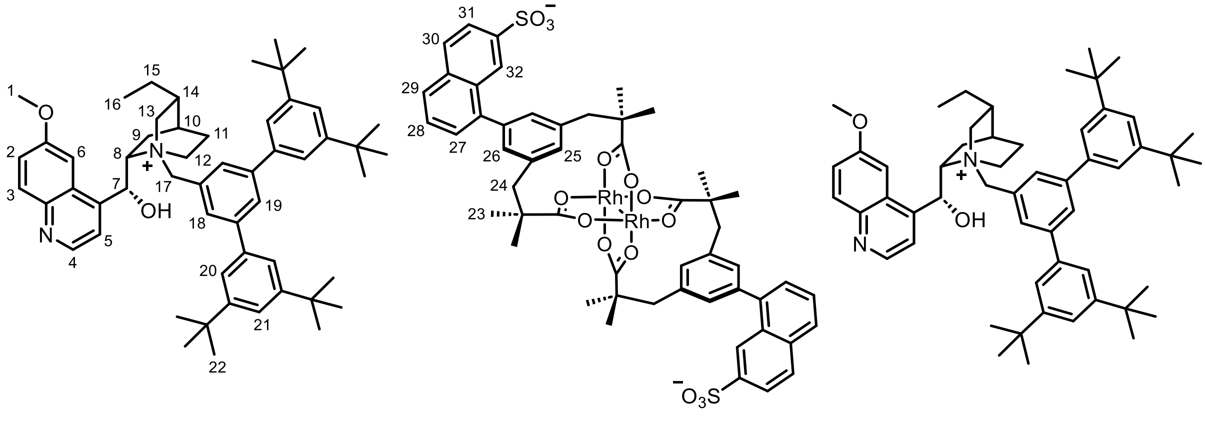

Prepared according to **GP3** on a 0.040 mmol scale with respect to Rh_2(**A-V**)$_2$●(**NBu$_4$**)$_2$ and using **Cat2**●Br as the chiral cation bromide salt. The title compound was isolated as a brown powder (75 mg, 0.027 mmol, 68%).

^{1}H NMR (500 MHz, C_5D_5N): δ 9.22 (s, 2H, H-32), 9.07 (d, J = 4.4 Hz, 2H, H-4), 8.95-8.52 (m, 6H, H-18, H-31), 8.34-8.30 (m, 4H, H-3, O-H), 8.27 (m, 2H, H-19), 8.03 (d, J = 4.4 Hz, 2H, H-5), 7.93-7.89 (m, 10H, H-20, H-30), 7.83 (d, J = 8.4 Hz, 2H, H-29), 7.76-7.72 (m, 6H, H-6, H-21), 7.62 (d, J = 5.2 Hz, 2H, H-7), 7.51 (s, 2H, H-25), 7.48-7.41 (m, 4H, H-2, H-28), 7.29 (d, J = 7.0 Hz, 2H, H-27), 7.11 (s, 4H, H-26), 6.86 (d, J = 12.3 Hz, 2H, H-17$_A$), 5.11 (d, J = 12.3 Hz, 2H, H-17$_B$), 5.00 (m, 2H, H-12$_A$), 4.10 (m, 2H, H-8), 4.03 (t, J = 11.6 Hz, 2H, H-13$_A$), 3.75 (s, 6H, H-1), 3.58 (m, 2H, H-13$_B$), 3.37 (m, 2H, H-12$_B$), 2.78 (br. s, 8H, H-24), 2.53 (m, 2H, H-9$_A$), 2.38 (m, 2H, H-11$_A$), 1.84 (s, 2H, H-10), 1.65 (t, J = 11.7 Hz, 2H, H-9$_B$), 1.60 (m, 2H, H-14), 1.51-1.38 (s, 74H, H-11$_B$, H-22), 1.22 (m, 4H, H-15), 1.12 (s, 24H, H-23), 0.72 (t, J = 7.4 Hz, 6H, H-16) ppm;

^{13}C NMR (126 MHz, C_5D_5N): δ 197.4, 159.0, 152.6, 148.6, 146.2, 145.54, 145.52, 145.2, 142.4, 141.0, 139.54, 139.49, 135.0, 133.1, 132.8, 131.8, 131.1, 130.5, 130.0, 129.5, 129.1, 128.6, 127.9, 127.0, 126.9, 125.4, 124.6, 123.1, 122.9, 122.0, 121.5, 102.5, 71.8, 65.5, 64.5, 63.1, 56.1, 51.9, 47.6, 46.5, 36.5, 35.7, 32.0, 26.6, 26.5 (br. s), 26.1, 25.6, 21.1, 11.8 ppm;

HRMS (−ESI): *m/z* for dianion found 584.0374, $[C_{52}H_{50}O_{14}Rh_2S_2]^{2-}$ requires 584.0382, (δ = − 1.4 ppm);

HRMS (+ESI): *m/z* for cation found 793.5640, $[C_{55}H_{73}N_2O_2]^+$ requires 793.5667, (δ = − 3.4 ppm);

Optical rotation data was acquired as the pyridine-ligated catalyst:

$[\alpha]_D^{25.0}$ = − 73.2 (c. 0.08, $CHCl_3$).

Bis[rhodium (1S,2R,4S)-2-((S)-hydroxy(naphthalen-1-yl)methyl)-1-((3,3'',5,5''-tetra-tert-butyl-[1,1':3',1''-terphenyl]-5'-yl)methyl)quinuclidin-1-ium (3,5-bis(2-carboxy-2-methylpropyl)benzenesulfonate)] (Rh_2(**A-I**)$_2$•(**Cat6**)$_2$)

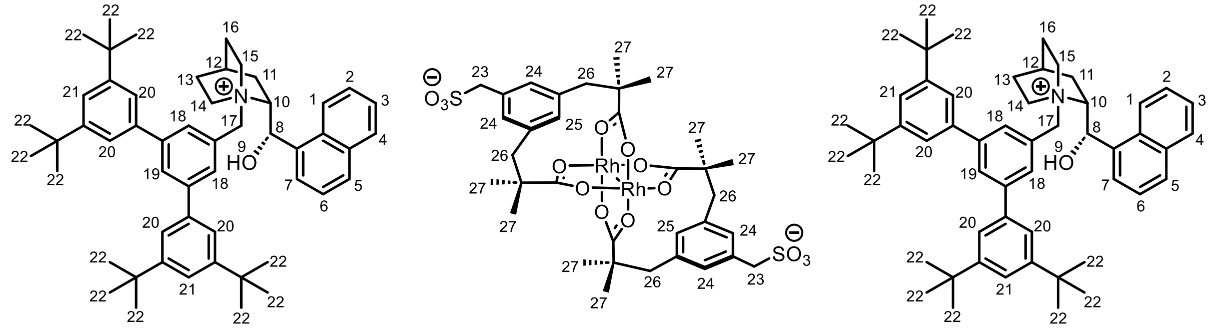

Prepared according to **GP4** on a 0.020 mmol scale with respect to Rh_2(**A-I**)$_2$•(Bu_4N)$_2$ and using (**Cat6•Br**) as the chiral bromide salt. The title compound was obtained as a green powder (39.6 mg, 0.0164 mmol, 91% yield).

^{1}H NMR (700 MHz, C_5D_5N): δ 8.50 (br s, 4 H, H-18), 8.37 (br s, 2 H, H-9), 8.27 (d, *J* = 8.5 Hz, 2 H, H-1), 8.25-8.24 (m, 2 H, H-19), 8.19 (d, *J* = 7.1 Hz, 2 H, H-7), 7.96 (d, *J* = 8.1 Hz, 2 H, H-4), 7.92-7.90 (m, 10 H, H-5, H-20), 7.73 (t, *J* = 1.7 Hz, 4 H, H-21), 7.61-7.59 (m, 2 H, H-6, overlapped with the signal of residual Pyridine), 7.45-7.43 (m, 2 H, H-3), 7.29-7.28 (m, 4 H, H-24), 7.25-7.24 (m, 2 H, H-25), 7.21-7.19 (m, 2 H, H-2, overlapped with the signal of residual Pyridine), 7.06 (br s, 2 H, H-8), 6.14 (d, *J* = 12.2 Hz, 2 H, H-17a), 5.36 (d, *J* = 12.2 Hz, 2 H, H-17b), 4.76-4.72 (m, 2 H, H-14a), 4.45 (s, 4 H, H-23), 4.16-4.09 (m, 4 H, H-15a, H-10), 3.44-3.36 (m, 4 H, H-14b, H-15b), 2.56-2.51 (m, 8 H, H-26), 2.34-2.31 (m, 2 H, H-11a), 1.89-1.86 (m, 2 H, H-13a), 1.78 (br s, 2 H, H-12), 1.59-1.56 (m, 2 H, H-16a), 1.52-1.39 (m, 76 H, H-13b, H-16b, H-22), 1.04-0.97 (m, 26 H, H-11b, H-27) ppm;

^{13}C NMR (176 MHz, C_5D_5N): δ 197.2, 152.5, 144.8, 141.2, 138.7, 137.0, 134.9, 134.5, 132.9, 131.3, 130.51, 130.50, 130.3, 129.8, 129.4, 129.2, 127.5, 126.7, 126.5, 126.3, 123.8*, 123.1, 122.8, 70.2, 65.6, 64.1, 59.4, 57.5, 51.7, 47.6, 46.4, 35.7, 32.1, 26.53, 26.50, 26.4, 24.8, 24.2, 21.6 ppm;

* Obscured by the solvent peak but clearly visible in the HSQC and HMBC spectra.

HRMS (+ESI): *m/z* For cation found 734.5331, $[C_{53}H_{68}NO]^+$ requires 734.5295, (δ = + 4.9 ppm);

HRMS (-ESI): *m/z* For dianion found 472.0068, $[C_{34}H_{42}O_{14}Rh_2S_2]^{2-}$ requires 472.0069, (δ = - 0.2 ppm);

$[\alpha]_D^{25.0}$ = + 91.9 (c = 0.053, $CHCl_3$) (*acquired as the pyridine-ligated catalyst*).

Bis[rhodium (1S,2R,4S)-2-((S)-hydroxy(7-methoxynaphthalen-1-yl)methyl)-1-((3,3'',5,5''-tetra-tert-butyl-[1,1':3',1''-terphenyl]-5'-yl)methyl)quinuclidin-1-ium (3,5-bis(2-carboxy-2-methylpropyl)benzenesulfonate)](Rh_2(**A-I**)$_2$•(**Cat7**)$_2$)

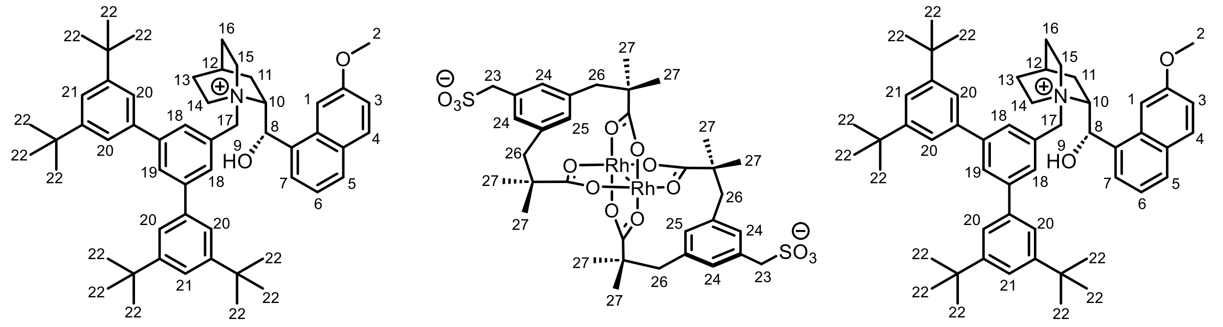

Prepared according to **GP4** on a 0.030 mmol scale with respect to Rh_2(**A-I**)$_2$•(Bu_4N)$_2$ and using (**Cat7•Br**) as the chiral bromide salt. The title compound was obtained as a green powder (57.9 mg, 0.0234 mmol, 87% yield).

^{1}H NMR (700 MHz, C_5D_5N): δ 8.45 (br s, 4 H, H-18), 8.25-8.23 (m, 4 H, H-9, H-19), 8.14 (d, *J* = 7.1 Hz, 2 H, H-7), 7.93-7.92 (m, 10 H, H-4, H-20), 7.89 (d, *J* = 8.1 Hz, 2 H, H-5), 7.735-7.730 (m, 4 H, H-21), 7.69 (br s, 2 H, H-1), 7.51-7.48 (m, 2 H, H-6), 7.30-7.28 (m, 2 H, H-3), 7.22 (6 H, H-8, H-24)*, 7.19 (br s, 2 H, H-25), 6.51 (d, *J* = 12.1 Hz, 2 H, H-17a), 5.02 (d, *J* = 12.1 Hz, 2 H, H-17b, overlapped with the signal of H_2O), 4.75-4.71 (m, 2 H, H-14a), 4.41-4.37 (m, 4 H, H-23), 4.03-3.98 (m, 4 H, H-10, H-15a), 3.82 (s, 6 H, H-2), 3.56-3.52 (m, 2 H, H-15b), 3.37-3.33 (m, 2 H, H-14b), 2.57-2.47 (m, 10 H, H-11a, H-26), 2.01-1.98 (m, 2 H, H-13a), 1.84 (br s, 2 H, H-12), 1.59-1.24 (m, 80 H, H-11b, H-13b, H-16, H-22), 1.02 (s, 12 H, H-27a), 0.99 (s, 12 H, H-27b) ppm;

* Obscured by the residual solvent peak but clearly visible in the ^{1}H-^{1}H COSY and HSQC spectra.

^{13}C NMR (176 MHz, C_5D_5N): δ 197.2, 159.1, 152.5, 145.0, 141.1, 138.7, 136.1*, 134.7, 132.7, 131.9, 131.5, 131.2, 130.4, 130.3, 130.1, 129.5, 129.0, 127.3, 124.0*, 123.1, 122.9, 118.5, 103.3, 71.1, 65.2, 64.8, 59.3, 57.4, 55.9, 51.7, 47.5, 46.4, 35.7, 32.1, 26.53, 26.48, 26.2, 24.7, 24.4, 21.6 ppm;

* Obscured by the solvent peak but clearly visible in the HSQC spectrum.

HRMS (+ESI): *m/z* For cation found 764.5380, $[C_{54}H_{70}NO_2]^+$ requires 764.5401, (δ = - 2.7 ppm);

HRMS (-ESI): *m/z* For dianion found 472.0067, $[C_{34}H_{42}O_{14}Rh_2S_2]^{2-}$ requires 472.0069, (δ = - 0.4 ppm);

$[\alpha]_D^{25.0}$ = + 94.0 (c = 0.10, $CHCl_3$) (*acquired as the pyridine-ligated catalyst*).

Bis[rhodium (1S,2R,4S)-2-((S)-hydroxy(quinolin-5-yl)methyl)-1-((3,3'',5,5''-tetra-tert-butyl-[1,1':3',1''-terphenyl]-5'-yl)methyl)quinuclidin-1-ium (3,5-bis(2-carboxy-2-methylpropyl)benzenesulfonate)] (Rh_2(**A-I**)$_2$•(**Cat8**)$_2$)

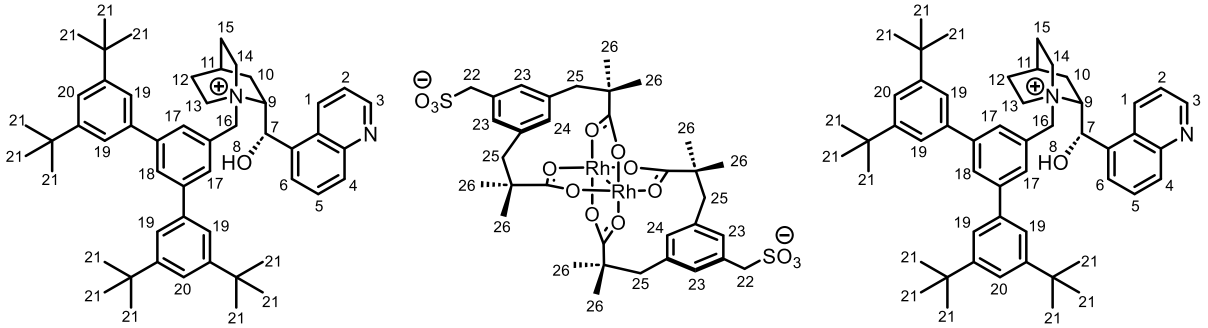

Prepared according to **GP3** on a 0.030 mmol scale with respect to Rh_2(**A-I**)$_2$•(Bu_4N)$_2$ and using (**Cat8•Br**) as the chiral bromide salt. The title compound was obtained as a brown powder (51.5 mg, 0.0213 mmol, 79% yield).

^{1}H NMR (700 MHz, C_5D_5N): δ 8.97 (dd, *J* = 4.0, 1.1 Hz, 2 H, H-3), 8.81 (d, *J* = 8.5 Hz, 2 H, H-1), 8.53 (br s, 4 H, H-17), 8.40 (d, *J* = 4.4 Hz, 2 H, H-8), 8.33 (d, *J* = 8.3 Hz, 2 H, H-4), 8.25-8.24 (m, 2 H, H-18), 8.18 (d, *J* = 7.2 Hz, 2 H, H-6), 7.94 (d, *J* = 1.5 Hz, 8 H, H-19), 7.81 (dd, *J* = 8.2, 7.4 Hz, 2 H, H-5), 7.73 (t, *J* = 1.6 Hz, 4 H, H-20), 7.27 (br s, 4 H, H-23), 7.234-7.230 (m, 2 H, H-24, overlapped with the signal of residual Pyridine), 7.17 (dd, *J* = 8.5, 4.0 Hz, 2 H, H-2), 7.02-7.01 (m, 2 H, H-7), 6.05 (d, *J* = 12.2 Hz, 2 H, H-16a), 5.55 (d, *J* = 12.2 Hz, 2 H, H-16b), 4.73-4.69 (m, 2 H, H-13a), 4.46-4.42 (m, 4 H, H-22), 4.29-4.26 (m, 2 H, H-14a), 4.20 (t, *J* = 9.2 Hz, 2 H, H-9), 3.43-3.35 (m, 4 H, H-13b, H-14b), 2.55-2.50 (m, 8 H, H-25), 2.31-2.28 (m, 2 H, H-10a), 1.88-1.85 (m, 2 H, H-12a), 1.79 (br s, 2 H, H-11), 1.62—1.56 (m, 2 H, H-15a), 1.38 (m, 76 H, H-12b, H-15b, H-21), 1.07-1.00 (m, 26 H, H-10b, H-26) ppm;

^{13}C NMR (176 MHz, C_5D_5N): δ 197.2, 152.5, 150.9, 149.5, 144.7, 141.2, 138.7, 137.8, 134.8, 132.9, 132.5, 131.3, 130.7, 130.5, 130.3, 129.5, 129.3, 127.0, 125.8, 123.1, 122.8, 122.4, 69.8, 65.6, 63.9, 59.4, 57.4, 51.7, 47.5, 46.4, 35.7, 32.1, 26.54, 26.50, 26.4, 24.8, 24.1, 21.6 ppm;

HRMS (+ESI): *m/z* For cation found 735.5256, $[C_{52}H_{67}N_2O]^+$ requires 735.5248, (δ = + 1.1 ppm);

HRMS (-ESI): *m/z* For dianion found 472.0060, $[C_{34}H_{42}O_{14}Rh_2S_2]^{2-}$ requires 472.0069, (δ = - 1.9 ppm);

$[\alpha]_D^{25.0}$ = + 18.3 (c = 0.12, C_5H_5N) (*acquired as the pyridine-ligated catalyst*).

Bis[rhodium (1S,2R,4S)-2-((S)-hydroxy(quinolin-4-yl)methyl)-1-((3,3'',5,5''-tetra-tert-butyl-[1,1':3',1''-terphenyl]-5'-yl)methyl)quinuclidin-1-ium (3,5-bis(2-carboxy-2-methylpropyl)benzenesulfonate)] (Rh_2(**A-I**)$_2$•(**Cat9**)$_2$)

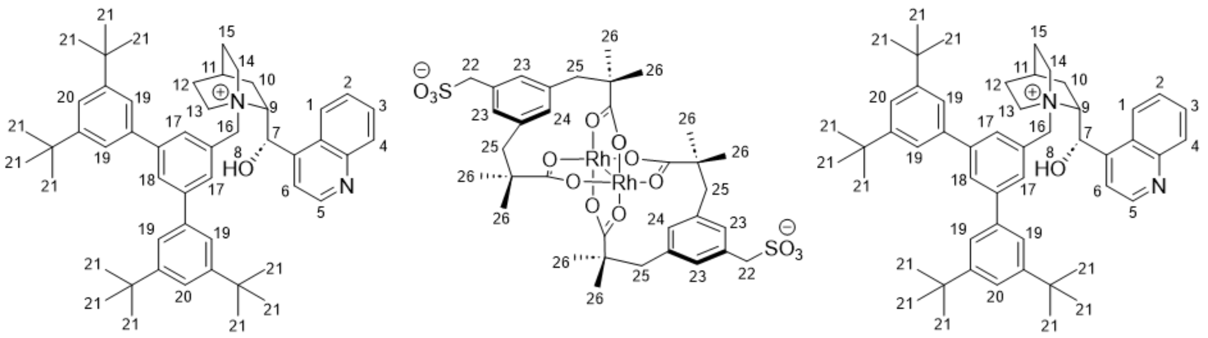

Prepared according to **GP3** on a 0.03 mmol scale with respect to Rh_2(**A-I**)$_2$•(Bu_4N)$_2$ and using (**Cat9•Br**) as the chiral bromide salt. The title compound was obtained as a brown powder (56.7 mg, 0.0235 mmol, 87% yield).

^{1}H NMR (700 MHz, C_5D_5N): δ 9.14 (d, *J* = 4.4 Hz, 2 H, H-5), 8.53 (br s, 4 H, H-17), 8.45 (br d, *J* = 4.8 Hz, 2 H, H-8), 8.38 (d, *J* = 8.4 Hz, 2 H, H-4), 8.28 (d, *J* = 8.4 Hz, 2 H, H-1), 8.242-8.237 (m, 2 H, H-18), 8.00 (d, *J* = 4.4 Hz, 2 H, H-6), 7.94 (d, *J* = 0.9 Hz, 8 H, H-19), 7.73 (t, *J* = 1.6 Hz, 4 H, H-20), 7.65-7.63 (m, 2 H, H-3), 7.25-7.23 (m, 6 H, H-2, H-23, overlapped with the signal of residual Pyridine), 7.20 (br s, 2 H, H-24), 7.04 (d, *J* = 4.6 Hz, 2 H, H-7), 6.20 (d, *J* = 12.2, 2 H, H-16a), 5.40 (d, *J* = 12.2 Hz, 2 H, H-16b), 4.70-4.66 (m, 2 H, H-13a), 4.43 (br s, 4 H, H-22), 4.17-4.12 (m, 4 H, H-9, H-14a), 3.48-3.44 (m, 2 H, H-14b), 3.39-3.35 (m, 2 H, H-13b), 2.54-2.48 (m, 8 H, H-25), 2.25-2.22 (m, 2 H, H-10a), 1.90-1.87 (m, 2 H, H-12a), 1.80 (br s, 2 H, H-11), 1.63-1.59 (m, 2 H, H-15a), 1.56-1.37 (m, 76 H, H-12b, H-15b, H-21), 1.10- 0.98 (m, 26 H, H-10b, H-26) ppm;

^{13}C NMR (176 MHz, C_5D_5N): δ 197.2, 152.5, 151.1, 149.3, 146.8, 144.7, 141.2, 138.7, 134.6, 132.9, 131.34, 131.31, 130.42, 130.36, 129.8, 129.3, 127.8, 125.8, 124.2*, 123.1, 122.8, 121.4, 69.7, 65.1, 64.1, 59.3, 57.6, 51.8, 47.5, 46.4, 35.7, 32.1, 26.54, 26.51, 26.2, 24.7, 24.2, 21.5 ppm;

* Obscured by the solvent peak but clearly visible in the HSQC and HMBC spectra.

HRMS (+ESI): *m/z* For cation found 735.5250, $[C_{52}H_{67}N_2O]^+$ requires 735.5248, (δ = + 0.3 ppm);

HRMS (-ESI): *m/z* For dianion found 472.0069, $[C_{34}H_{42}O_{14}Rh_2S_2]^{2-}$ requires 472.0069, (δ = ± 0.0 ppm);

$[\alpha]_D^{25.0}$ = + 27.5 (c = 0.12, C_5H_5N) (*acquired as the pyridine-ligated catalyst*).

Bis[rhodium (1R,2S,4R)-2-((R)-hydroxy(6-methoxyquinolin-4-yl)methyl)-1-((3,3'',5,5''-tetra-tert-butyl-[1,1':3',1''-terphenyl]-5'-yl)methyl)quinuclidin-1-ium (3,5-bis(2-carboxy-2-methylpropyl)benzenesulfonate)] (Rh_2(**A-I**)$_2$•(**Cat10**)$_2$)

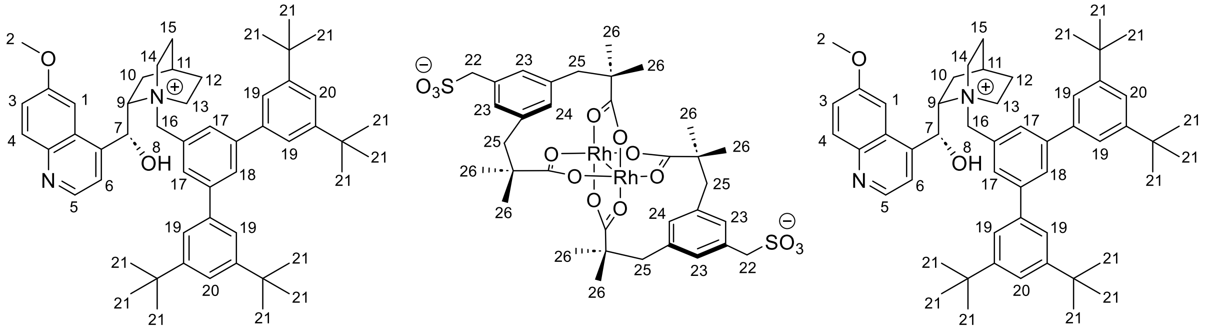

Prepared according to **GP3** on a 0.040 mmol scale with respect to Rh_2(**A-I**)$_2$•(Bu_4N)$_2$ and using (**Cat10•Br**) as the chiral bromide salt. The title compound was obtained as a brown powder (85.5 mg, 0.0345 mmol, 96% yield).

^{1}H NMR (700 MHz, C_5D_5N): δ 9.05 (d, *J* = 4.4 Hz, 2 H, H-5), 8.47 (br s, 4 H, H-17), 8.36 (d, *J* = 9.1 Hz, 2 H, H-4), 8.24-8.23 (m, 4 H, H-8, H-18), 7.94-7.93 (m, 10 H, H-6, H-19), 7.73 (t, *J* = 1.7 Hz, 4 H, H-20), 7.66 (d, *J* = 2.6 Hz, 2 H, H-1), 7.50 (dd, *J* = 9.1, 2.6 Hz, 2 H, H-3), 7.19-7.18 (m, 6 H, H-7, H-23), 7.10 (br s, 2 H, H-24), 6.54 (d, *J* = 12.2 Hz, 2 H, H-16a), 5.04 (d, *J* = 12.2 Hz, 2 H, H-16b), 4.70-4.66 (m, 2 H, H-13a), 4.41-4.37 (m, 4 H, H-22), 4.05-4.01 (m, 4 H, H-9, H-14a), 3.83 (s, 6 H, H-2), 3.60-3.56 (m, 2 H, H-14b), 3.35-3.31 (m, 2 H, H-13b), 2.51-2.45 (m, 10 H, H-10a, H-25), 2.02-1.99 (m, 2 H, H-12a), 1.88-1.86 (m, 2 H, H-11), 1.64-1.59 (m, 2 H, H-15a), 1.56-1.38 (m, 76 H, H-12b, H-15b, H-21), 1.28-1.24 (m, 2 H, H-10b), 1.01 (s, 12 H, H-26a), 0.99 (s, 12 H, H-26b) ppm;

^{13}C NMR (176 MHz, C_5D_5N): δ 197.2, 158.9, 152.5, 148.5, 145.54, 145.47, 145.0, 141.1, 138.7, 134.4, 133.0, 132.7, 131.2, 130.3, 130.2, 129.5, 127.0, 123.1, 122.9, 121.9, 121.5, 103.0, 70.6, 64.8, 64.7, 59.2, 57.5, 56.3, 51.6, 47.5, 46.3, 35.7, 32.1, 26.51, 26.47, 26.0, 24.7, 24.3, 21.5 ppm;

HRMS (+ESI): *m/z* For cation found 765.5338, $[C_{53}H_{69}N_2O_2]^+$ requires 765.5354, (δ = - 2.1 ppm);

HRMS (-ESI): *m/z* For dianion found 472.0055, $[C_{34}H_{42}O_{14}Rh_2S_2]^{2-}$ requires 472.0069, (δ = - 3.0 ppm);

$[\alpha]_D^{25.0}$ = - 95.0 (c = 0.080, C_5H_5N) (*acquired as the pyridine-ligated catalyst*).

Bis[rhodium (1S,2R,4S,5R)-2-((S)-(2-butyl-6-methoxyquinolin-4-yl)(hydroxy)methyl)-5-ethyl-1-((3,3'',5,5''-tetra-tert-butyl-[1,1':3',1''-terphenyl]-5'-yl)methyl)quinuclidin-1-ium (3,5-bis(2-carboxy-2-methylpropyl)benzenesulfonate)] (Rh_2(**A-I**)$_2$•(**Cat11**)$_2$)

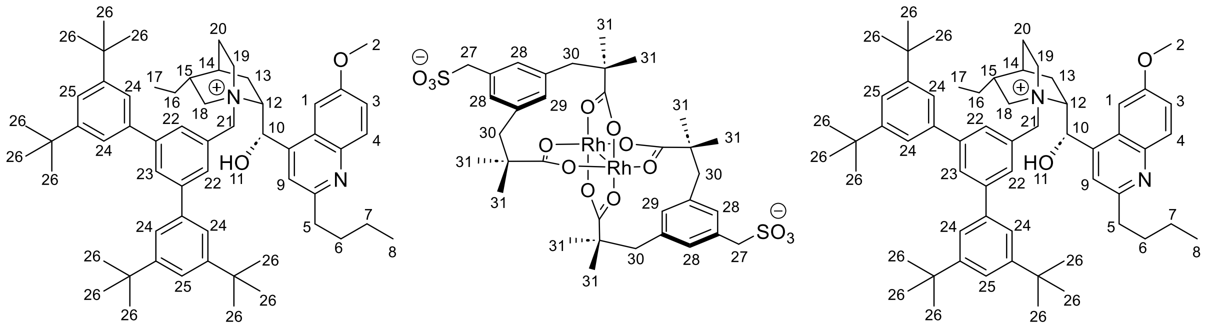

Prepared according to **GP4** on a 0.030 mmol scale with respect to Rh_2(**A-I**)$_2$•(Bu_4N)$_2$ and using (**Cat11•Br**) as the chiral bromide salt. The title compound was obtained as a green powder (67.1 mg, 0.0254 mmol, 94% yield).

^{1}H NMR (700 MHz, C_5D_5N): δ 8.72 (2 H, H-11)*, 8.53 (br s, 4 H, H-22), 8.34-8.33 (m, 2 H, H-4), 8.27 (br s, 2 H, H-23), 8.13 (br s, 2 H, H-9), 7.93-7.92 (m, 10 H, H-1, H-24), 7.73-7.72 (m, 4 H, H-25), 7.53-7.51 (m, 2 H, H-3), 7.18-7.17 (m, 6 H, H-28, H-29), 7.12 (br s, 2 H, H-10), 6.06 (d, *J* = 12.3 Hz, 2 H, H-21a), 5.51 (d, *J* = 12.3 Hz, 2 H, H-21b), 4.60-4.57 (m, 2 H, H-18a), 4.48-4.45 (m, 2 H, H-19a), 4.40-4.35 (m, 4 H, H-27), 4.28-4.26 (m, 2 H, H-12), 3.96-3.95 (m, 6 H, H-2), 3.90-3.86 (m, 2 H, H-18b), 3.36-3.32 (m, 2 H, H-19b), 3.09 (t, *J* = 7.7 Hz, 4 H, H-5), 2.79-2.76 (m, 2 H, H-13a), 2.53-2.44 (m, 8 H, H-30), 1.92-1.88 (m, 4 H, H-6), 1.71 (br s, 2 H, H-14), 1.65-1.61 (m, 2 H, H-20a), 1.55-1.38 (m, 84 H, H-7, H-15, H-16, H-20b, H-26), 1.24-1.20 (m, 2 H, H-13b), 1.03 (s, 12 H, H-31a), 1.02 (s, 12 H, H-31b), 0.91-0.88 (m, 6 H, H-8), 0.75-0.73 (m, 6 H, H-17) ppm;

* Obscured by the residual solvent peak but clearly visible in the ^{1}H-^{1}H COSY spectrum.

^{13}C NMR (176 MHz, C_5D_5N): δ 197.2, 160.7, 158.4, 152.5, 145.4, 145.1, 145.0, 141.1, 138.7, 134.7, 132.8, 132.4, 131.2, 130.4, 130.2, 129.4, 125.8, 123.1, 122.9, 122.0, 121.6, 103.8, 69.6, 66.7, 64.2, 59.4, 57.5, 56.8, 56.4, 47.5, 46.4, 39.3, 36.5, 35.7, 32.5, 32.1, 26.51, 26.49, 25.6, 25.1, 25.0, 23.3, 21.8, 14.6, 11.9 ppm;

HRMS (+ESI): *m/z* For cation found 849.6329, $[C_{59}H_{81}N_2O_2]^+$ requires 849.6293, (δ = + 4.2 ppm);

HRMS (-ESI): *m/z* For dianion found 472.0052, $[C_{34}H_{42}O_{14}Rh_2S_2]^{2-}$ requires 472.0069, (δ = - 3.6 ppm);

$[\alpha]_D^{25.0}$ = + 67.5 (c = 0.067, $CHCl_3$) (*acquired as the pyridine-ligated catalyst*);

Bis[rhodium (1S,2R,4S,5R)-5-ethyl-2-((S)-methoxy(6-methoxyquinolin-4-yl)methyl)-1-((3,3'',5,5''-tetra-tert-butyl-[1,1':3',1''-terphenyl]-5'-yl)methyl)quinuclidin-1-ium (3,5-bis(2-carboxy-2-methylpropyl)benzenesulfonate)]
(Rh_2(**A-I**)$_2$•(**Cat12**)$_2$)

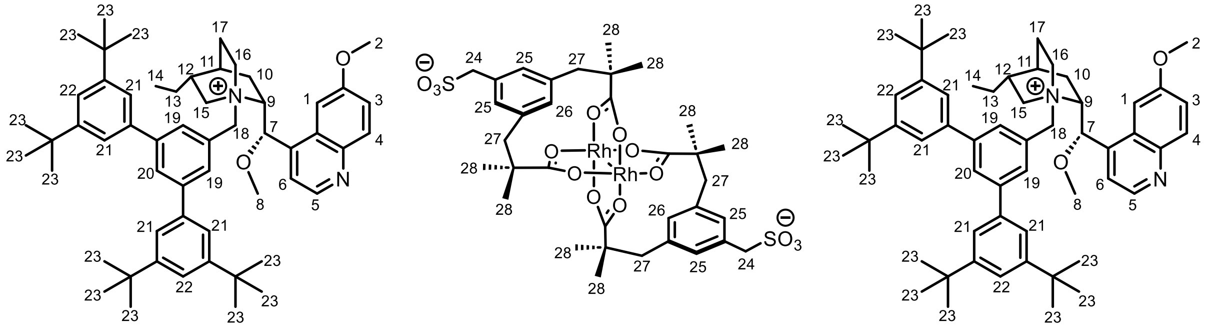

Prepared according to **GP3** on a 0.03 mmol scale with respect to Rh_2(**A-I**)$_2$•(Bu_4N)$_2$ and using (**Cat12•Br**) as the chiral bromide salt. The title compound was obtained as a brown powder (60.2 mg, 0.0235 mmol, 87% yield).

^{1}H NMR (700 MHz, C_5D_5N): δ 9.06 (br s, 2 H, H-5), 8.61 (br s, 4 H, H-19), 8.39 (d, *J* = 8.5 Hz, 2 H, H-4), 8.28 (br s, 2 H, H-20), 8.19 (br s, 2 H, H-1), 7.96 (br s, 10 H, H-6, H-21), 7.73 (br s, 4 H, H-22), 7.63-7.61 (m, 2 H, H-3), 7.22 (2 H, H-26)*, 7.12 (br s, 4 H, H-25), 6.21 (br d, *J* = 11.7 Hz, 4 H, H-7, H-18a), 4.90-4.81 (m, 4 H, H-16a, H-18b), 4.63 (br s, 2 H, H-9), 4.40-4.32 (m, 4 H, H-24), 4.21 (br s, 6 H, H-8), 4.03-4.00 (m, 2 H, H-15a), 3.92-3.89 (m, 2 H, H-15b), 3.27 (s, 6 H, H-2), 3.01-2.96 (m, 2 H, H-16b), 2.53 (d, *J* = 12.1 Hz, 4 H, H-27a), 2.38 (t, *J* = 11.7 Hz, 2 H, H-10a), 2.32 (d, *J* = 12.1 Hz, 4 H, H-27b), 1.54-1.38 (m, 80 H, H-11, H-12, H-13, H-23), 1.28-1.25 (m, 4 H, H-17), 1.04 (s, 24 H, H-28), 1.00-0.96 (m, 2 H, H-10b), 0.82 (t, *J* = 6.4 Hz, 6 H, H-14) ppm;

* Obscured by the residual solvent peak but clearly visible in the ^{1}H-^{1}H COSY, HSQC and HMBC spectra.

^{13}C NMR (176 MHz, C_5D_5N): δ 197.2, 159.5, 152.5, 148.4, 146.2, 144.9, 141.0, 139.4, 138.7, 135.3, 133.0, 132.8, 131.4, 130.6, 130.0, 129.2, 128.6, 123.1†, 122.9, 103.8, 67.7, 63.0, 59.5, 57.4, 56.9†, 56.7, 47.4, 46.4, 36.2, 35.7, 32.1, 26.5, 25.7, 25.1, 24.3, 22.5, 11.9 ppm;

† There are additional ^{13}C resonances underneath these peaks.

Note: the ^{13}C resonance belonging to C7 was not detected due having a broad and weak signal.

HRMS (+ESI): *m/z* For cation found 807.5820, $[C_{56}H_{75}N_2O_2]^+$ requires 807.5823, (δ = - 0.4 ppm);

HRMS (-ESI): *m/z* For dianion found 472.0057, $[C_{34}H_{42}O_{14}Rh_2S_2]^{2-}$ requires 472.0069, (δ = - 2.5 ppm);

$[\alpha]_D^{25.0}$ = + 106.9 (c = 0.053, $CHCl_3$) (*acquired as the pyridine-ligated catalyst*).

Bis[rhodium (1S,2R,4S)-2-((S)-hydroxy(quinolin-5-yl)methyl)-1-((3,3'',5,5''-tetra-tert-butyl-[1,1':3',1''-terphenyl]-5'-yl)methyl)quinuclidin-1-ium (3',5'-bis(2-carboxy-2-methylpropyl)-[1,1'-biphenyl]-2-sulfonate)] (Rh_2(**A-III**)$_2$•(**Cat8**)$_2$)

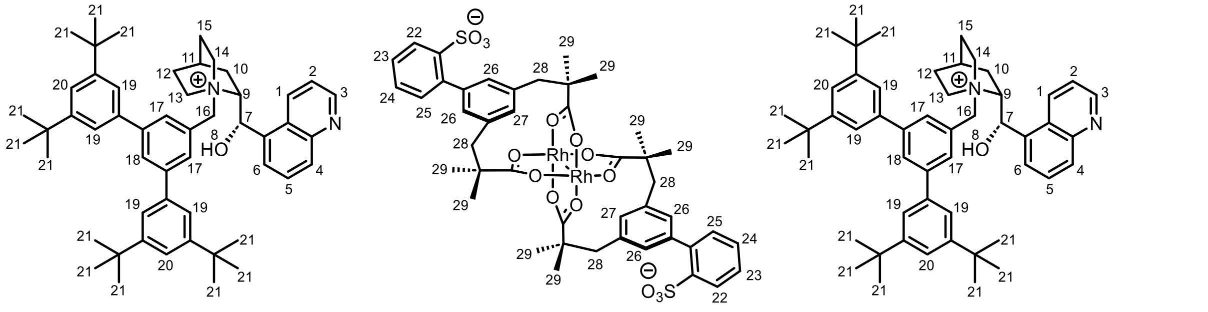

Prepared according to **GP3** on a 0.030 mmol scale with respect to Rh_2(**A-III**)$_2$•(Bu_4N)$_2$ and using (**Cat8•Br**) as the chiral bromide salt. The title compound was obtained as a brown powder (54.8 mg, 0.0216 mmol, 80% yield).

^{1}H NMR (700 MHz, C_5D_5N): δ 8.93-8.92 (m, 2 H, H-3), 8.84-8.83 (m, 2 H, H-22), 8.79 (d, *J* = 8.6 Hz, 2 H, H-1), 8.46 (s, 4 H, H-17), 8.33 (d, *J* = 8.3 Hz, 2 H, H-4), 8.25-8.24 (m, 4 H, H-8, H-18), 8.17 (d, *J* = 7.2 Hz, 2 H, H-6), 7.91 (d, *J* = 1.6 Hz, 8 H, H-19), 7.82 (dd, *J* = 8.3, 7.2 Hz, 2 H, H-5), 7.73 (t, *J* = 1.6 Hz, 4 H, H-20), 7.46 (br d, *J* = 1.1 Hz, 4 H, H-26), 7.30-7.24 (m, 6 H, H-23, H-24, H-27), 7.14-7.13 (m, 2 H, H-25), 7.09 (dd, *J* = 8.6, 4.0 Hz, 2 H, H-2), 7.01 (br s, 2 H, H-7), 5.94 (d, *J* = 12.2 Hz, 2 H, H-16a), 5.56 (d, *J* = 12.2 Hz, 2 H, H-16b), 4.80-4.76 (m, 2 H, H-13a), 4.29 (t, *J* = 11.3 Hz, 2 H, H-14a), 4.18 (t, *J* = 9.2 Hz, 2 H, H-9), 3.53-3.49 (m, 2 H, H-13b), 3.39-3.35 (m, 2 H, H-14b), 2.58 (br s, 8 H, H-28), 2.32-2.29 (m, 2 H, H-10a), 1.93-1.90 (m, 2 H, H-12a), 1.82 (br s, 2 H, H-11), 1.65-1.61 (m, 2 H, H-15a), 1.50-1.44 (m, 76 H, H-12b, H-15b, H-21), 1.07-0.98 (m, 26 H, H-10b, H-29) ppm;

^{13}C NMR (176 MHz, C_5D_5N): δ 197.4, 152.5, 151.0, 149.4, 146.7, 144.9, 142.2, 142.0, 141.3, 137.73, 137.68, 133.1, 132.9, 132.6, 130.8, 130.7, 130.5, 130.1, 129.4,* 127.0, 126.9, 125.9, 123.1, 122.9, 122.5, 69.6, 65.8, 63.9, 57.3, 51.8, 47.7, 46.4, 35.7, 32.1, 26.6, 26.5, 26.4, 24.8, 24.1, 21.7 ppm;

* There is an additional ^{13}C resonance underneath this peak.

HRMS (+ESI): *m/z* For cation found 735.5256, $[C_{52}H_{67}N_2O]^+$ requires 735.5248, (δ = + 1.1 ppm);

HRMS (-ESI): *m/z* For dianion found 534.0208, $[C_{44}H_{46}O_{14}Rh_2S_2]^{2-}$ requires 534.0225, (δ = - 3.2 ppm);

$[\alpha]_D^{25.0}$ = + 39.6 (c = 0.093, C_5H_5N) (*acquired as the pyridine-ligated catalyst*).

Synthesis of Hydrocinnamyl Alcohol Substrates

The following hydrocinnamyl alcohol substrates were commercially available: **1b**, **1f**, **1i**, **1l**, **1n**, and **1r**.

3-(o-tolyl)propan-1-ol (**1c**)

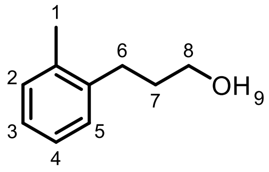

To a solution of 3-(2-methylphenyl)propionic acid (500 mg, 3.1 mmol) in THF (8 mL) at 0 °C was added $LiAlH_4$ (1.5 mL of a 2.4 M solution in THF, 3.6 mmol) dropwise. The reaction mixture was allowed to warm to room temperature and stirred for a further 8 hours and quenched through the careful portion-wise addition of sodium sulfate decahydrate. After the effervescence had ceased, the quenched mixture was filtered, eluting with plenty of EtOAc. The filtrate was collected and the solvent removed under reduced pressure. Purification by flash column chromatography (SiO_2, 0-30% v/v EtOAc in Hexane) afforded the title compound as a colourless oil (428 mg, 2.9 mmol, 92%).

***R_f* value** = 0.41 (30% v/v EtOAc in Hexane);

^{1}H NMR (400 MHz, $CDCl_3$) δ 7.09-7.17 (m, 4H, H-2, H-3, H-4, H-5), 3.72 (t, J = 6.4 Hz, 2H, H-8), 2.68-2.72 (m, 2H, H-6), 2.33 (s, 3H, H-1), 1.83-1.90 (m, 2H, H-7), 1.43 (s, 1H, H-9) ppm;

^{13}C NMR (101 MHz, $CDCl_3$) δ 140.1, 136.1, 130.4, 128.9, 126.2, 126.1, 62.7, 33.2, 29.6, 19.4 ppm;

The spectroscopic data is in agreement with that reported in the literature.[21]

3-(2-methoxyphenyl)propan-1-ol (**1d**)

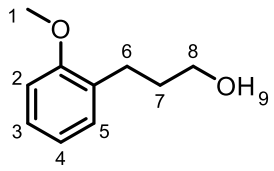

To a round bottomed flask containing 3-(2-Methoxyphenyl)propionic acid (541 mg, 3.0 mmol), and THF (8.0 mL), was added lithium aluminium hydride (1.5 mL of a 2.4 M solution in THF, 3.6 mmol) dropwise. The reaction was stirred at 0 °C for 20 min, and then at room temperature for a further 40 min. The reaction was then quenched by the cautious addition of sodium sulphate decahydrate at 0 °C until all effervescence had stopped. The reaction mixture was then filtered, eluting with plenty of EtOAc, and the solvent removed under reduced pressure. Purification by flash column chromatography (SiO_2, 30% v/v EtOAc in Hexane) afforded the title compound as a colourless oil (446 mg, 2.68 mmol, 89%).

R_f value = 0.36 (30% v/v EtOAc in Hexane);

^{1}H NMR (400 MHz, $CDCl_3$) δ 7.19 (t, J = 7.9 Hz, 1H, H-3), 7.15 (d, J = 7.3 Hz, 1H, H-5), 6.91 (t, J = 7.4 Hz, 1H, H-4), 6.86 (d, J = 8.1 Hz, 1H, H-2), 3.84 (s, 3H, H-1), 3.60 (t, J = 6.3 Hz, 2H, H-8), 2.73 (t, J = 7.4 Hz, 2H, H-6), 1.85 (pent, J = 6.7 Hz, 2H, H-7), 1.75 (br s, 1H, H-9) ppm;

^{13}C NMR (101 MHz, $CDCl_3$) δ 157.5, 130.3, 130.1, 127.3, 120.9, 110.5, 62.1, 55.5, 33.1, 26.0 ppm;

The spectroscopic data is in agreement with that reported in the literature.[22]

3-(2-chlorophenyl)propan-1-ol (**1e**)

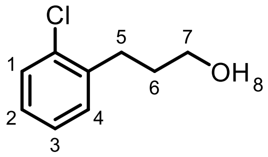

Prepared according to **GP5** using 3-(2-chlorophenyl)propanoic acid as the carboxylic acid. Purification by flash column chromatography (SiO_2, 0-40% v/v EtOAc in Hexane) afforded the title compound as a colourless oil (251 mg, 1.5 mmol, 49%).

***R_f* value** = 0.33 (30% v/v EtOAc in Hexane);

^{1}H NMR (400 MHz, $CDCl_3$) δ 7.34 (dd, J = 7.6 Hz, 1.2 Hz, 1H, H-1), 7.24 (dd, J = 7.5 Hz, 1.8 Hz, 1H, H-4), 7.19 (td, J = 7.3 Hz, 1.4 Hz, 1H, H-3), 7.14 (td, J = 7.5 Hz, 1.8 Hz, 1H, H-2), 3.70 (t, J = 6.4 Hz, 2H, H-7), 2.81-2.85 (m, 2H, H-5), 1.86-1.94 (m, 2H, H-6), 1.49 (s, 1H, H-8) ppm;

^{13}C NMR (101 MHz, $CDCl_3$) δ 139.5, 134.1, 130.6, 129.6, 127.5, 126.9, 62.3, 32.7, 30.0 ppm;

The ^{1}H NMR data is in agreement with that reported in the literature.[23]

3-(m-tolyl)propan-1-ol (**1g**)

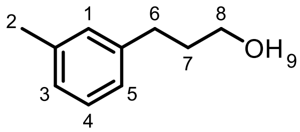

Prepared according to **GP5** using 3-(*m*-tolyl)propanoic acid as the carboxylic acid. Purification by flash column chromatography (SiO_2, 0-26% v/v Acetone in Petrol) afforded the title compound as a yellow oil (527 mg, Quant.)

***R_f* value** = 0.36 (20% v/v Acetone in Petrol);

^{1}**H NMR** (400 MHz, $CDCl_3$) δ 7.18 (t, J = 7.5 Hz, 1H, H-4), 7.00-7.03 (m, 3H, H-1, H-3, H-5), 3.68 (t, J = 6.4 Hz, 2H, H-8), 2.66-2.70 (m, 2H, H-6), 2.34 (s, 3H, H-2), 1.86-1.93 (m, 2H, H-7), 1.43 (s, 1H, H-9) ppm;

^{13}**C NMR** (101 MHz, $CDCl_3$) δ 141.9, 138.1, 129.4, 128.4, 126.7, 125.6, 62.5, 34.4, 32.1, 21.5 ppm;

The spectroscopic data is in agreement with that reported in the literature.[24]

3-(3-(tert-butyl)phenyl)prop-2-yn-1-ol

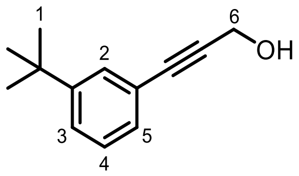

Prepared according to **GP6** on a 5.0 mmol scale with respect to 1-bromo-3-tert-butylbenzene. Purification by flash column chromatography (SiO_2, 0-7% v/v acetone in $CHCl_3$) afforded the title compound as a brown oil (162 mg, 0.858 mmol, 17%).

***R$_f$* value**: 0.62 (5% acetone in $CHCl_3$);

^{1}**H NMR** (400 MHz, $CDCl_3$): δ 7.49 (m, 1H, H-2), 7.36 (m, 1H, H-3), 7.28-7.22 (m, 2H, H-4, H-5), 4.51 (d, J = 3.9 Hz, 2H, H-6), 1.81 (br. s, 1H, O-H), 1.31 (s, 9H, H-1) ppm;

^{13}**C NMR** (101 MHz, $CDCl_3$): δ 151.4, 128.94, 128.88, 128.2, 125.9, 122.2, 86.7, 86.4, 51.8, 34.8, 31.3 ppm;

HRMS (+ESI) *m/z* Found $[M+H]^+$ 189.1268, $[C_{13}H_{17}O]^+$ requires 189.1274, (δ = − 3.2 ppm).

3-(3-(tert-butyl)phenyl)propan-1-ol (**1h**)

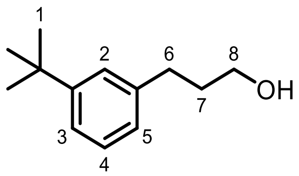

Prepared according to **GP7** on a 0.798 mmol scale with respect to 3-(3-(tert-butyl)phenyl)prop-2-yn-1-ol. Purification by flash column chromatography (SiO_2, 0-30% v/v acetone in hexane) afforded the title compound as a yellow oil (60 mg, 0.314 mmol, 40%).

***R$_f$* value**: 0.42 (30% acetone in hexane);

^{1}**H NMR** (400 MHz, $CDCl_3$): δ 7.29-7.23 (m, 3H, H-2, H-3, H-4), 7.05 (m, 1H, H-5), 3.71 (t, J = 6.5 Hz, 2H, H-8), 2.73 (m, 2H, H-6), 1.93 (m, 2H, H-7), 1.88 (br. s, 1H, O-H), 1.35 (s, 9H, H-1) ppm;

^{13}C NMR (101 MHz, $CDCl_3$): δ 151.3, 141.5, 128.2, 125.6, 122.9, 62.5, 34.7, 34.5, 32.5, 31.5 ppm;

There is an additional ^{13}C resonance underneath the peak at 125.6 ppm.

HRMS (+ESI) *m/z* Found $[M+H]^+$ 193.1588, $[C_{13}H_{21}O]^+$ requires 193.1587, (δ = + 0.5 ppm).

Ethyl 3-(3-hydroxyprop-1-yn-1-yl)benzoate

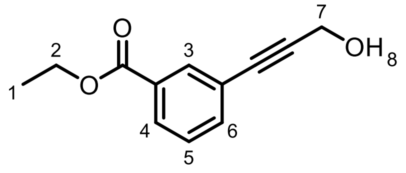

To an oven dried three-necked round-bottomed flask was added $Pd(PPh_3)_2Cl_2$ (154 mg, 0.22 mmol) and CuI (84 mg, 0.44 mmol). The flask was placed under a nitrogen atmosphere and MeCN (15 mL), triethylamine (10.0 mmol, 1.39 mL) and ethyl 3-iodobenzoate (0.84 mL, 5.0 mmol) were added. After stirring at room temperature for 15 minutes, propargyl alcohol (0.35 mL, 6.0 mmol) was added and the resulting mixture was stirred at room temperature overnight. The reaction mixture was diluted in EtOAc, filtered over Celite® and concentrated under reduced pressure. Purification of the crude residue by flash column chromatography (SiO_2, 0-10% v/v acetone in $CHCl_3$) afforded the title compound as a yellow oil (1.07 g, quant.%)

***R_f* value** = 0.54 (10% v/v Acetone in $CHCl_3$);

^{1}H NMR (400 MHz, $CDCl_3$) δ 8.11 (t, *J* = 1.5 Hz, 1H, H-3), 7.99 (dt, *J* = 7.9 Hz, 1.9 Hz, 1H, H-4), 7.59 (dt, *J* = 7.7 Hz, 1.4 Hz, 1H, H-6), 7.38 (t, *J* = 7.8 Hz, 1H, H-5), 4.51 (s, 2H, H-7), 4.37 (q, *J* = 7.1 Hz, 2H, H-2), 1.83 (s, 1H, H-8), 1.39 (t, *J* = 7.1 Hz, 3H, H-1) ppm;

^{13}C NMR (101 MHz, $CDCl_3$) δ 166.0, 135.8, 132.9, 130.9, 129.6, 128.6, 123.1, 88.3, 84.9, 61.4, 51.7, 14.4 ppm;

The ^{1}H NMR data is in agreement with that reported in the literature.[25]

Ethyl 3-(3-hydroxypropyl)benzoate (**1j**)

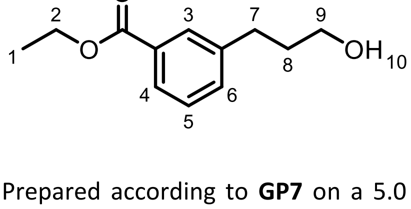

Prepared according to **GP7** on a 5.0 mmol scale with respect to ethyl 3-(3-hydroxyprop-1-yn-1-yl)benzoate. Purification by flash column chromatography (SiO_2, 0-20% v/v acetone in petrol) afforded the title compound as a colourless oil (327 mg, 1.57 mmol, 31%).

***R_f* value** = 0.33 (20% v/v Acetone in Petrol);

^{1}**H NMR** (400 MHz, $CDCl_3$) δ 7.86-7.88 (m, 2H, H-3, H-4), 7.39 (dt, J = 7.6 Hz, 1.6 Hz, 1H, H-6), 7.35 (td, J = 7.0 Hz, 0.8 Hz, 1H, H-5), 4.37 (q, J = 7.1 Hz, 2H, H-2), 3.68 (t, J = 6.4 Hz, 2H, H-9), 2.76 (dd, J = 8.8 Hz, 7.6 Hz, 2H, H-7), 1.88-1.95 (m, 2H, H-8), 1.53 (s, 1H, H-10), 1.39 (t, J = 7.1 Hz, 3H, H-1) ppm;

^{13}**C NMR** (101 MHz, $CDCl_3$) δ 166.9, 142.3, 133.1, 130.7, 130.0, 128.5, 127.3, 62.2, 61.1, 34.2, 32.0, 14.5 ppm;

The spectroscopic data is in agreement with that reported in the literature.[26]

3-(naphthalen-1-yl)propan-1-ol (**1k**)

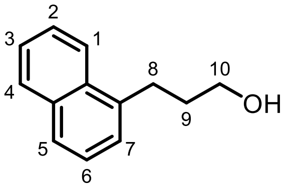

Prepared according to **GP5** using 3-(1-naphthyl)propanoic acid as the carboxylic acid. In this case the following procedural modification was employed: 2.0 mmol of acid was used and the other reagents were scaled accordingly. Purification by flash column chromatography (SiO_2, 0-40% v/v acetone in hexane) afforded the title compound as a colourless oil (320 mg, 1.72 mmol, 86%).

***R_f* value**: 0.41 (30% v/v acetone in hexane);

^{1}**H NMR** (400 MHz, $CDCl_3$): δ 8.08 (d, J = 8.0 Hz, 1H, H-1), 7.87 (m, 1H, H-4), 7.74 (d, J = 8.0 Hz, 1H, H-5), 7.55-7.45 (m, 2H, H-2, H-3), 7.41 (m, 1H, H-6), 7.36 (d, J = 6.8 Hz, 1H, H-7), 3.76 (t, J = 6.4 Hz, 2H, H-10), 3.19 (dd, J = 7.6, 7.5 Hz, 2H, H-8), 2.04 (m, 2H, H-9), 1.50 (m, 1H, O-H) ppm;

^{13}**C NMR** (101 MHz, $CDCl_3$): δ 138.1, 134.1, 132.0, 128.9, 126.8, 126.1, 125.9, 125.7, 125.6, 123.9, 62.6, 33.7, 29.3 ppm;

The spectroscopic data is in agreement with that reported in the literature.[27]

3-(4-methoxyphenyl)propan-1-ol (**1m**)

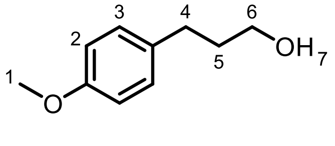

To a round bottomed flask containing 3-(4-Methoxyphenyl) propionic acid (901 mg, 5.0 mmol), and THF (12.0 mL), was added lithium aluminium hydride (2.5 mL of a 2.4 M solution in THF, 6.0 mmol) dropwise. The reaction was stirred at 0 °C for 20 min, and then at room temperature overnight. The reaction was then quenched by the cautious addition of sodium sulphate decahydrate at 0 °C until all effervescence had stopped. The reaction mixture was then filtered, eluting with plenty of EtOAc, and the solvent removed under

reduced pressure. Purification by flash column chromatography (SiO_2, 40% v/v EtOAc in Hexane) afforded the title compound as a colourless oil (508 mg, 3.1 mmol, 61%).

***R*F value** = 0.38 (40% v/v EtOAc in Hexane);

^{1}H NMR (400 MHz, $CDCl_3$) δ 7.12 (d, J = 8.2 Hz, 2H, H-3), 6.84 (d, J = 8.2 Hz, 2H, H-2), 3.79 (s, 3H, H-1), 3.67 (t, J = 6.4 Hz, 2H, H-6), 2.66 (t, J = 7.5 Hz, 2H, H-4), 1.87 (pent, J = 7.0 Hz, 2H, H-5) ppm, 1.42 (s, 1H, H-7) ppm;

^{13}C NMR (400 MHz, $CDCl_3$) δ 158.0, 134.0, 129.4, 114.0, 62.4, 55.4, 34.6, 31.3 ppm;

The spectroscopic data is in agreement with that reported in the literature.[28]

3-(4-(trifluoromethyl)phenyl)propan-1-ol (**1o**)

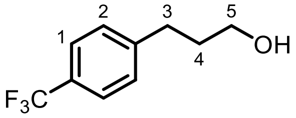

Prepared according to **GP5** using 3-[4-(trifluoromethyl)phenyl]propanoic acid as the carboxylic acid. Purification by flash column chromatography (SiO_2, 0-30% v/v acetone in hexane) afforded the title compound as a colourless oil (472 mg, 2.31 mmol, 77%).

R_f value: 0.22 (20% v/v acetone in hexane);

^{1}H NMR (400 MHz, $CDCl_3$): δ 7.54 (d, J = 8.3 Hz, 2H, H-1), 7.31 (d, J = 8.0 Hz, 2H, H-2), 3.67 (t, J = 6.4 Hz, 2H, H-5), 2.77 (dd, J = 7.5, 7.5 Hz, 2H, H-3), 1.90 (m, 2H, H-4), 1.54 (br. s, 1H, O-H) ppm;

^{13}C NMR (101 MHz, $CDCl_3$): δ 146.1, 128.9, 128.4 (q, J = 32.3 Hz), 125.4 (q, J = 3.7 Hz), 124.5 (q, J = 271 Hz), 62.0, 34.0, 32.0 ppm;

^{19}F NMR (376 MHz, $CDCl_3$): δ – 62.3 ppm

The spectroscopic data is in agreement with that reported in the literature.[29]

2,2-dimethyl-3-phenylpropan-1-ol (**1p**)

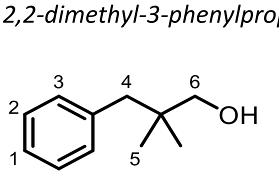

To an oven dried round-bottomed flask charged with lithium diisopropylamide (2 M in THF, 7.5 mL, 15 mmol) and additional THF (10 mL) at – 10 °C under a nitrogen atmosphere was added methyl isobutyrate (1.15 mL, 10 mmol) dropwise. After stirring at this temperature for 1 hour, benzyl bromide (1.78 mL, 15 mmol) in THF (10 mL) was added dropwise and the resulting mixture was gradually warmed to room temperature over 2 hours. The

reaction was quenched with saturated aqueous NH_4Cl, extracted with EtOAc, dried over $MgSO_4$ and concentrated under reduced pressure. The crude product was placed under a nitrogen atmosphere, cooled to 0 °C and re-dissolved in Et_2O (20 mL). $LiAlH_4$ (pellets, 570 mg, 15 mmol) was added and the reaction mixture was gradually warmed to room temperature overnight. The reaction mixture was then re-cooled to 0 °C and quenched through the careful portion-wise addition of sodium sulfate decahydrate. After the effervescence had ceased, the quenched mixture was filtered, eluting with plenty of EtOAc. The filtrate was collected and the solvent removed under reduced pressure. Purification by flash column chromatography (SiO_2, 0-20% v/v EtOAc in hexane) afforded the title compound as a yellow oil (324 mg, 1.98 mmol, 20%).

***R_f* value**: 0.43 (20% v/v ethyl acetate in hexane);

^{1}H NMR (400 MHz, $CDCl_3$): δ 7.29 (m, 2H, H-2), 7.21 (m, 1H, H-1), 7.17 (m, 2H, H-3), 3.33 (s, 2H, H-6), 2.59 (s, 2H, H-4), 1.50 (br. s, 1H, O-H), 0.90 (s, 6H, H-5) ppm;

^{13}C NMR (101 MHz, $CDCl_3$): δ 138.9, 130.6, 128.0, 126.1, 71.3, 44.8, 36.6, 24.1 ppm;

HRMS (+ESI) *m/z* Found $[M+H]^+$ 165.1276, $[C_{11}H_{17}O]^+$ requires 165.1274, (δ = + 1.2 ppm);

The spectroscopic data is in agreement with that reported in the literature.[30]

(1-benzylcyclopentyl)methanol (**1q**)

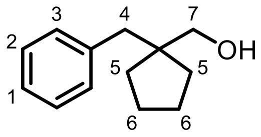

To an oven dried round-bottom flask charged with freshly distilled diisopropylamine (2.75 mL, 19.5 mmol) and THF (20 mL) at 0 °C under a nitrogen atmosphere was added n-butyllithium (1.6 M in hexanes, 12.2 mL, 19.5 mmol) dropwise. After stirring at this temperature for 1 hour, the reaction was cooled to – 40 °C and methyl cyclopentanecarboxylate (2.5 g, 19.5 mmol) dissolved in THF (10 mL) was added dropwise. After 30 minutes the reaction was warmed to 0 °C and stirred at this temperature for an additional 1 hour. Benzyl bromide (1.78 mL, 15 mmol) was added dropwise and the resulting mixture was gradually warmed to room temperature overnight. The reaction was quenched with saturated aqueous NH_4Cl, extracted with EtOAc, dried over $MgSO_4$ and concentrated under reduced pressure. The crude product was placed under a nitrogen atmosphere, cooled to 0 °C and re-dissolved in THF (40 mL). $LiAlH_4$ (pellets, 740 mg, 19.5 mmol) was added and the reaction mixture was gradually warmed to room temperature overnight. The reaction mixture was then re-cooled to 0 °C and quenched through the careful portion-wise addition of sodium sulfate decahydrate. After the effervescence had ceased, the quenched mixture was filtered, eluting with plenty of EtOAc. The filtrate was collected and the solvent removed under reduced pressure. Purification by flash column chromatography (SiO_2, 0-20% v/v EtOAc in hexane) afforded the title compound as a colourless oil (2.28 g, 12.0 mmol, 80%).

***R_f* value**: 0.39 (20% v/v ethyl acetate in hexane);

^{1}H NMR (700 MHz, $CDCl_3$): *δ* 7.30 (m, 2H, H-2), 7.52-7.21 (m, 3H, H-1, H-3), 3.35 (d, *J* = 3.3 Hz, 2H, H-7), 2.73 (s, 2H, H-4), 1.70-1.58 (m, 5H, H-6, O-H), 1.53 (m, 2H, H-5_A), 1.43 (m, 2H, H-5_B) ppm;

^{13}C NMR (176 MHz, $CDCl_3$): *δ* 139.6, 130.4, 128.1, 126.0, 67.7, 48.7, 42.4, 34.2, 24.6 ppm;

HRMS (+ESI) *m/z* Found $[M{-}(OH)]^+$ 173.1317, $[C_{13}H_{17}]^+$ requires 173.1325, (*δ* = – 4.6 ppm).

1-phenethylcyclobutan-1-ol (**1s**)

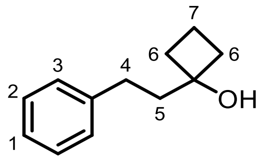

To an oven dried round-bottomed flask under a nitrogen atmosphere at 0 °C was added phenethylmagnesium chloride (1.0 M in THF, 6.5 mL, 6.5 mmol) and THF (5 mL). Cyclobutanone (0.37 mL, 5 mmol) dissolved in THF (5 mL) was added dropwise and the resulting mixture was gradually warmed to room temperature over 2 hours. The reaction was quenched with saturated aqueous NH_4Cl, extracted with Et_2O, dried over $MgSO_4$ and concentrated under reduced pressure. Purification by flash column chromatography (SiO_2, 0-20% v/v EtOAc in hexane) afforded the title compound as a colourless oil (676 mg, 3.83 mmol, 77%).

***R_f* value**: 0.21 (10% v/v ethyl acetate in hexane);

^{1}H NMR (400 MHz, $CDCl_3$): *δ* 7.33-7.17 (m, 5H, H-1, H-2, H-3), 2.73 (m, 2H, H-4), 2.09 (m, 4H, H-6), 1.94 (m, 2H, H-5), 1.85-1.69 (m, 2H, H-7_A, O-H), 1.57 (m, 1H, H-7_B) ppm;

^{13}C NMR (101 MHz, $CDCl_3$): *δ* 142.7, 128.6, 128.5, 125.9, 75.4, 41.5, 36.2, 30.1, 12.3 ppm;

HRMS (+ESI) *m/z* Found $[M{-}(OH)]^+$ 159.1165, $[C_{12}H_{15}]^+$ requires 159.1168, (*δ* = – 1.9 ppm);

The spectroscopic data is in agreement with that reported in the literature.[31]

Ethyl (E)-2-(thiophen-3-yl)acrylate

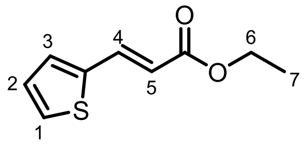

To a suspension of NaH (60 wt% suspension in mineral oil, 480 mg, 12 mmol) in THF (30 mL) at 0 °C was added triethyl phosphonoacetate (2.2 mL, 11 mmol) dropwise and the reaction mixture was stirred at the same temperature for a further 15 mins. Following this, a solution of thiophene-2-carbaldehyde (0.93 mL, 10 mmol)

in THF (7 mL) was added dropwise and the addition of the aldehyde was made quantitative with THF (2 mL). The reaction mixture was allowed to warm to room temperature overnight following which it was quenched with saturated aqueous NH_4Cl. The THF was then removed under reduced pressure and the aqueous layer was extracted with Et_2O. The combined organic extracts were washed with brine, dried ($MgSO_4$) and the solvent removed under reduced pressure. Purification by flash column chromatography (SiO_2, 0-20% v/v Acetone in Petrol) afforded the title compound as a yellow oil (1.44 g, 7.9 mmol, 79%).

***R_f* value** = 0.67 (20% v/v Acetone in Petrol);

^{1}H NMR (400 MHz, $CDCl_3$) δ 7.78 (d, J = 15.7 Hz, 1H, H-4), 7.36 (d, J = 5.1 Hz, 1H, H-1), 7.25 (d, J = 3.6 Hz, 1H, H-3), 7.05 (dd, J = 5.1 Hz, 3.6 Hz, 1H, H-2), 6.24 (d, J = 15.7 Hz, 1H, H-5), 4.25 (q, J = 7.1 Hz, 2H, H-6), 1.32 (t, J = 7.1 Hz, 3H, H-7) ppm;

^{13}C NMR (101 MHz, $CDCl_3$) δ 167.0, 139.7, 137.2, 130.9, 128.5, 128.2, 117.2, 60.6, 14.5 ppm.

The spectroscopic data is in agreement with that reported in the literature.[32]

3-(thiophen-2-yl)propan-1-ol (**1t**)

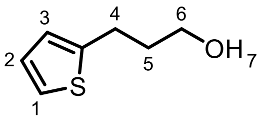

Prepared according to **GP7** on a 3.0 mmol scale with respect to ethyl (*E*)-2-(thiophen-3-yl)acrylate. In this case the following procedural modification was employed: The reaction was warmed to 50 °C. Following the filtration through celite and evaporation of the solvent, the crude residue was dissolved in THF (25 mL) and cooled to 0 °C following which $LiAlH_4$ (1.5 mL of a 2.4 M solution in THF, 3.6 mmol) was added dropwise. The reaction mixture was stirred at the same temperature for 20 minutes following which the ice bath was removed and the reaction stirred for a further 40 minutes. The reaction mixture was then re-cooled to 0 °C and quenched through the careful portion-wise addition of sodium sulfate decahydrate. After the effervescence had ceased, the quenched mixture was filtered, eluting with plenty of acetone. The filtrate was collected and the solvent removed under reduced pressure. Purification by two consecutive flash column chromatography procedures (SiO_2, 0-19% v/v Acetone in Petrol followed by 0-10% v/v Et_2O in CH_2Cl_2) afforded the title compound as a colourless oil (287 mg, 2.0 mmol, 67%).

***R_f* value** = 0.33 (20% v/v Acetone in Petrol);

^{1}H NMR (400 MHz, $CDCl_3$) δ 7.12 (dd, J = 5.1 Hz, 1.1 Hz, 1H, H-1), 6.92 (dd, J = 5.1 Hz, 3.4 Hz, 1H, H-2), 6.81-6.82 (m, 1H, H-3), 3.71 (t, J = 6.3 Hz, 2H, H-6), 2.95 (q, J = 5.0 Hz, 2H, H-4), 1.92-1.99 (m, 2H, H-5) ppm;

^{13}C NMR (101 MHz, $CDCl_3$) δ 144.7, 126.9, 124.5, 123.2, 62.0, 34.6, 26.3 ppm;

HRMS (+ESI) *m/z* Found $[M+H]^+$ 143.0531, $[C_7H_{11}OS]^+$ requires 143.0525, (δ = + 4.2 ppm).

The spectroscopic data is in agreement with that reported in the literature.[33]

ethyl (E)-3-(benzo[b]thiophen-5-yl)acrylate

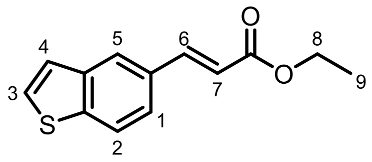

To a suspension of NaH (60 wt% suspension in mineral oil, 228 mg, 5.7 mmol) in THF (10 mL) at 0 °C was added triethyl phosphonoacetate (1.0 mL, 5.2 mmol) dropwise and the reaction mixture was stirred at the same temperature for a further 15 minutes. Following this, a solution of 1-benzothiophene-5-carbaldehyde (769 mg, 4.7 mmol) in THF (5 mL) was added dropwise and the addition of the aldehyde was made quantitative with THF (2 mL). The reaction mixture was allowed to warm to room temperature overnight following which it was quenched with saturated aqueous NH_4Cl. The THF was then removed under reduced pressure and the aqueous layer was extracted with Et_2O. The combined organic extracts were washed with brine, dried ($MgSO_4$) and the solvent removed under reduced pressure. Purification by flash column chromatography (SiO_2, 0-20% v/v EtOAc in hexane) afforded the title compound as an off-white solid (920 mg, 3.96 mmol, 84%).

***R*$_f$ value**: 0.65 (20% v/v ethyl acetate in hexane);

^{1}H NMR (700 MHz, $CDCl_3$): *δ* 7.95 (d, *J* = 1.5 Hz, 1H, H-5), 7.88 (d, *J* = 8.4 Hz, 1H, H-2), 7.81 (d, *J* = 16.0 Hz, 1H, H-6), 7.54 (dd, *J* = 8.4, 1.6 Hz, 1H, H-1), 7.49 (d, *J* = 5.4 Hz, 1H, H-3), 7.36 (dd, *J* = 5.4, 0.6 Hz, 1H, H-4), 6.51 (d, *J* = 16.0 Hz, 1H, H-7), 4.28 (q, *J* = 7.2 Hz, 2H, H-8), 1.35 (t, *J* = 7.2 Hz, 3H, H-9) ppm;

^{13}C NMR (176 MHz, $CDCl_3$): *δ* 167.3, 145.0, 141.6, 140.2, 131.0, 127.7, 124.3, 124.2, 123.2, 123.1, 117.9, 60.7, 14.5 ppm;

HRMS (ESI): *this compound did not ionise.*

3-(benzo[b]thiophen-5-yl)propan-1-ol (**1u**)

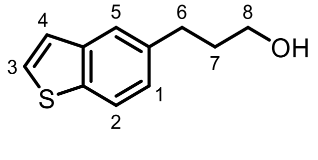

Prepared according to **GP7** on a 3.72 mmol scale with respect to ethyl (*E*)-3-(benzo[b]thiophen-5-yl)acrylate. Following the filtration through celite and evaporation of the solvent, the crude residue was dissolved in THF (25 mL) and cooled to 0 °C following which $LiAlH_4$ (2.4 M solution in THF, 3.6 mmol, 2.32 mL) was added dropwise. The reaction mixture was stirred at the same temperature for 20 minutes following which the ice bath was removed and the reaction stirred for a further 40 minutes. The reaction mixture was then re-cooled to 0 °C

and quenched through the careful portion-wise addition of sodium sulfate decahydrate. After the effervescence had ceased, the quenched mixture was filtered, eluting with plenty of acetone. The filtrate was collected and the solvent removed under reduced pressure. Purification by two consecutive chromatographic procedures (SiO_2, 0-7% v/v Et_2O in CH_2Cl_2 followed by preparative HPLC) afforded the title compound as a white solid (65 mg, 0.339 mmol, 9%).

R

R_f value: 0.66 (20% v/v acetone in $CHCl_3$);

^{1}H NMR (700 MHz, $CDCl_3$): δ 7.70 (d, *J* = 8.2 Hz, 1H, H-2), 7.65 (s, 1H, H-5), 7.42 (d, *J* = 5.4 Hz, 1H, H-3), 7.28 (d, *J* = 5.4 Hz, 1H, H-4), 7.21 (dd, *J* = 8.3, 1.3 Hz, 1H, H-1), 3.70 (t, *J* = 6.3 Hz, 2H, H-8), 2.84 (dd, *J* = 7.5, 7.5 Hz, 2H, H-6), 1.96 (m, 2H, H-7), 1.40 (br. s, 1H, O-H) ppm;

^{13}C NMR (176 MHz, $CDCl_3$): δ 140.1, 138.0, 137.5, 126.7, 125.5, 123.7, 123.2, 122.5, 62.4, 34.7, 32.1 ppm;

HRMS (ESI): *this compound did not ionise.*

We are very grateful to Thomas Wharton for assistance in the purification of this compound by preparative HPLC.

3-(benzo[b]thiophen-2-yl)propan-1-ol (**1v**)

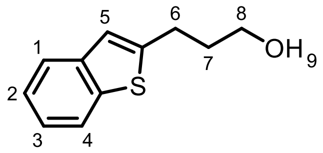

To a round bottomed flask containing 3-(benzo[*b*]thiophen-2-yl)propanoic acid (500 mg, 2.4 mmol), and THF (12 mL), was added lithium aluminium hydride (1.2 mL of a 2.4 M solution in THF, 6.0 mmol) dropwise. The reaction was stirred at 0 °C and allowed to heat up to room temperature overnight. The reaction was then quenched by the cautious addition of sodium sulphate decahydrate at 0 °C until all effervescence had stopped. The reaction mixture was then filtered, eluting with plenty of EtOAc, and the solvent removed under reduced pressure. Purification by flash column chromatography (SiO_2, 0-40 % v/v EtOAc in Hexane) afforded the title compound as compound as a white amorphous solid (420 mg, 2.2 mmol, 91%).

R_f value = 0.27 (30% v/v EtOAc in Hexane);

^{1}H NMR (400 MHz, $CDCl_3$) δ 7.77 (d, *J* = 8.0 Hz, 1H, H-4), 7.67 (d, *J* = 7.4 Hz, 1H, H-1), 7.32 (td, *J* = 7.3 Hz, 1.2 Hz, 1H, H-2), 7.26 (m, *J* = 7.8 Hz, 1.3 Hz, 1H, H-3), 7.04 (d, *J* = 0.6 Hz, 1H, H-5), 3.74 (t, *J* = 6.3 Hz, 2H, H-8), 3.00-3.04 (m, 2H, H-6), 1.99-2.06 (m, 2H, H-7), 1.49 (s, 1H, H-9) ppm;

^{13}C NMR (101 MHz, $CDCl_3$) δ 145.8, 140.3, 139.5, 124.3, 123.6, 122.9, 122.3, 121.0, 61.9, 33.9, 27.2 ppm;

HRMS (+ESI) *m/z* Found $[M+H]^+$ 193.0686, $[C_{11}H_{13}OS]^+$ requires 193.0682, (δ = + 2.1 ppm).

3-(1-(phenylsulfonyl)-1H-pyrrol-3-yl)prop-2-yn-1-ol

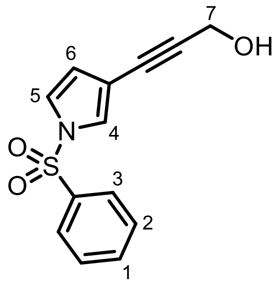

Prepared according to **GP6** on an 8.0 mmol scale with respect to 1-(benzenesulfonyl)-3-bromo-1H-pyrrole. Purification by flash column chromatography (SiO_2, 0-9% v/v acetone in $CHCl_3$) afforded the title compound as a brown oil (534 mg, 2.04 mmol, 26%).

***R_f* value**: 0.35 (10% v/v acetone in $CHCl_3$);

^{1}H NMR (400 MHz, $CDCl_3$): δ 7.85 (m, 2H, H-3), 7.62 (m, 1H, H-1), 7.51 (m, 2H, H-2), 7.31 (t, *J* = 1.8 Hz, 1H, H-4), 7.08 (dd, *J* = 3.2, 2.2 Hz, 1H, H-5), 6.32 (dd, *J* = 3.2, 1.6 Hz, 1H, H-6), 4.42 (d, *J* = 5.8 Hz, 2H, H-7), 1.71 (t, *J* = 6.0 Hz, 1H, O-H) ppm;

^{13}C NMR (101 MHz, $CDCl_3$): δ 138.6, 134.4, 129.7, 127.1, 124.1, 120.9, 116.5, 109.6, 88.4, 78.9, 51.7 ppm;

HRMS (+ESI) *m/z* Found $[M+H]^+$ 262.0536, $[C_{13}H_{12}NO_3S]^+$ requires 262.0532, (δ = + 1.5 ppm).

3-(1-(phenylsulfonyl)-1H-pyrrol-3-yl)propan-1-ol (**1w**)

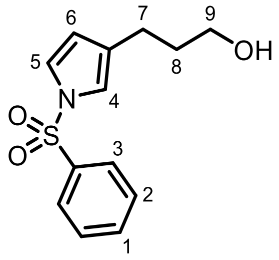

Prepared according to **GP7** on a 1.91 mmol scale with respect to 3-(1-(phenylsulfonyl)-1H-pyrrol-3-yl)prop-2-yn-1-ol. Purification by flash column chromatography (SiO_2, 0-9% v/v acetone in $CHCl_3$) afforded the title compound as a green oil (146 mg, 0.552 mmol, 29%).

***R_f* value**: 0.50 (40% v/v acetone in hexane);

^{1}H NMR (400 MHz, $CDCl_3$): δ 7.83 (m, 2H, H-3), 7.58 (m, 1H, H-1), 7.49 (m, 2H, H-2), 7.08 (dd, *J* = 3.1, 2.5 Hz, 1H, H-5), 6.92 (m, 1H, H-4), 6.17 (dd, *J* = 3.1, 1.6 Hz, 1H, H-6), 3.62 (t, *J* = 6.3 Hz, 2H, H-9), 2.48 (t, *J* = 7.5 Hz, 2H, H-7), 1.78 (m, 2H, H-8), 1.42 (br. s, 1H, O-H) ppm;

^{13}C NMR (101 MHz, $CDCl_3$): δ 139.3, 133.8, 129.4, 129.3, 126.8, 121.2, 117.5, 114.9, 62.2, 32.9, 23.1 ppm;

HRMS (+ESI) *m/z* Found $[M+H]^+$ 266.0840, $[C_{13}H_{16}NO_3S]^+$ requires 266.0845, (δ = − 1.9 ppm).

3-(benzofuran-2-yl)propan-1-ol (**1x**)

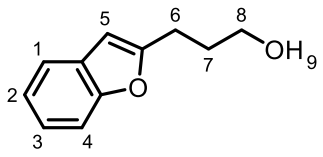

A round-bottomed flask was charged with 2-iodophenol (1.10 g, 5.0 mmol), $PdOAc_2$ (56.0 mg, 0.25 mmol) CuI (48 mg, 0.25 mmol) and PPh_3 (66.0 mg, 0.25 mmol). The flask was thrice evacuated and backfilled with nitrogen. Following this, dry Et_3N (10 mL) was added and the reaction mixture stirred at room temperature for 20 minutes after which 4-pentyn-1-ol (0.51 mL, 5.5 mmol) was added dropwise and the reaction mixture stirred at room temperature overnight. The solvent was removed under reduced pressure and the crude residue diluted with EtOAc (30 mL). The organic layer was washed with water (15 mL), brine (15 mL) and then dried ($MgSO_4$). Purification by flash column chromatography (SiO_2, 0-30% v/v EtOAc in Hexane) afforded the title compound as a dark red oil (800 mg, 4.5 mmol, 90%).

R*f* value = 0.29 (30% v/v Et_2O in Hexane);

^{1}H NMR (400 MHz, $CDCl_3$) δ 7.48-7.50 (m, 1H, H-1), 7.40-7.43 (m, 1H, H-4), 7.16-7.24 (m, 2H, H-2, H-3), 6.42 (q, *J* = 1.0 Hz, 1H, H-5), 3.75 (t, *J* = 6.3 Hz, 2H, H-8), 2.89 (t, *J* = 7.4 Hz, 2H, H-6), 1.98-2.05 (m, 2H, H-7), 1.52 (s, 1H, H-9) ppm;

^{13}C NMR (101 MHz, $CDCl_3$) δ 158.8, 154.8, 129.0, 123.4, 122.6, 120.4, 110.9, 102.4, 62.1, 30.8, 24.9 ppm;

The spectroscopic data is in agreement with that reported in the literature.[34]

methyl 4-(3-hydroxyprop-1-yn-1-yl)furan-2-carboxylate

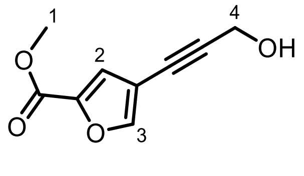

Prepared according to **GP6** on a 2.50 mmol scale with respect to methyl 4-bromofuran-2-carboxylate. Purification by flash column chromatography (SiO_2, 0-50% v/v acetone in hexane) afforded the title compound as a brown oil (116 mg, 0.643 mmol, 26%).

***R*f value**: 0.16 (20% v/v acetone in hexane);

^{1}H NMR (700 MHz, $CDCl_3$): δ 7.70 (s, 1H, H-3), 7.16 (s, 1H, H-2), 4.46 (d, *J* = 4.9 Hz, 2H, H-4), 3.90 (s, 3H, H-1), 1.77 (br. s, 1H, O-H) ppm;

^{13}**C NMR** (176 MHz, $CDCl_3$): δ 158.7, 148.9, 144.7, 120.0, 109.4, 90.4, 75.5, 52.3, 51.7 ppm;

HRMS (+ESI) *m/z* Found $[M+H]^+$ 181.0498, $[C_9H_9O_4]^+$ requires 181.0495, (δ = + 1.7 ppm).

methyl 4-(3-hydroxypropyl)furan-2-carboxylate (**1y**)

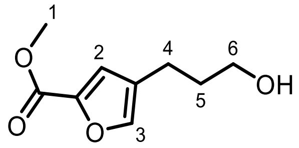

Prepared according to **GP7** on 0.60 mmol scale with respect to methyl 4-(3-hydroxyprop-1-yn-1-yl)furan-2-carboxylate. In this case the following procedural modification was employed: MeOH was used instead of EtOH. Purification by flash column chromatography (SiO_2, 0-40% v/v acetone in hexane) afforded the title compound as a colourless oil (42 mg, 0.230 mmol, 38%).

***R$_f$* value**: 0.22 (30% v/v acetone in hexane);

^{1}**H NMR** (400 MHz, $CDCl_3$): δ 7.34 (s, 1H, H-3), 7.04 (s, 1H, H-2), 3.84 (s, 3H, H-1), 3.63 (t, *J* = 6.3 Hz, 2H, H-6), 2.51 (t, *J* = 7.5 Hz, 2H, H-4), 2.04 (br. s, 1H, O-H), 1.79 (m, 2H, H-5) ppm;

^{13}**C NMR** (101 MHz, $CDCl_3$): δ 159.4, 144.5, 143.1, 127.0, 119.2, 61.7, 52.0, 32.6, 20.8 ppm;

HRMS (+ESI) *m/z* Found $[M+H]^+$ 185.0809, $[C_9H_{13}O_4]^+$ requires 185.0808, (δ = + 0.5 ppm).

1,5-diphenylpentan-3-ol ((**1z**)

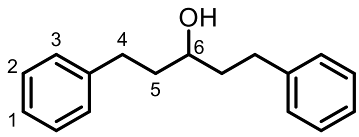

To a solution of phenethyl magnesium chloride (7.5 mL of a 1.0 M solution in THF, 7.5 mmol) in THF (20 mL) at 0 °C was added 3-phenylpropan-1-al (1.1 mL, 8.3 mmol) dropwise. The ice bath was then removed and the reaction mixture heated to 50 °C and stirred for 1.5 hours. The reaction mixture was then allowed to cool to room temperature and quenched with saturated aqueous NH_4Cl. The THF was removed under reduced pressure and the aqueous layer was extracted with Et_2O. The combined organics were washed with brine, dried ($MgSO_4$) and the solvent removed under reduced pressure. Purification by flash column chromatography (SiO_2, 0-15% v/v Acetone in Petrol) afforded the title compound as a white amorphous solid (1.21 g, 5.0 mmol, 67%).

***R$_f$* value** = 0.30 (15% v/v Acetone in Petrol);

^{1}H NMR (400 MHz, $CDCl_3$) δ 7.28-7.31 (m, 4H, H-2), 7.18-7.22 (m, 6H, H-1, H-3), 3.68 (tt, *J* = 7.8 Hz, 4.5 Hz, 1H, H-6), 2.77-2.84 (m, 2H, H-4a), 2.65-2.72 (m, 2H, H-4b), 1.74-1.89 (m, 4H, H-5a, H-5b) ppm;

^{13}C NMR (101 MHz, $CDCl_3$) δ 142.2, 128.6, 128.5, 126.0, 71.0, 39.4, 32.2 ppm;

The spectroscopic data is in agreement with that reported in the literature.[35]

Synthesis of Other Chain Length Substrates for C-H Amination

Note: the synthesis of aryl butanol substrates **6b** *and* **6c** *were reported previously.*[6]

Note: the synthesis of alkene intermediates below for **6e** *and* **6f** *were reported previously.*[5]

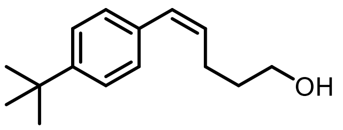

(Z)-5-(4-(tert-butyl)phenyl)pent-4-en-1-ol
(for **6e**)

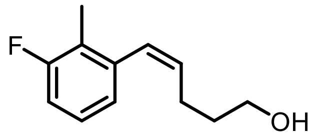

(Z)-5-(3-fluoro-2-methylphenyl)pent-4-en-1-ol
(for **6f**)

5-(4-(tert-butyl)phenyl)pentan-1-ol (**6e**)

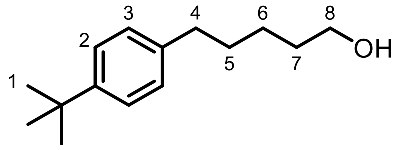

To a microwave vial charged with (*Z*)-5-(4-(*tert*-butyl)phenyl)pent-4-en-1-ol (278 mg, 1.27 mmol) and Pd/C (10 wt %, 74 mg) was added EtOH (15 mL). The vial was sealed and then evacuated and backfilled with hydrogen three times. The resulting mixture was stirred at room temperature under a hydrogen balloon overnight. After completion, the mixture was filtered over Celite® eluting with EtOAc. The filtrate was collected, the solvent removed under reduced pressure and purification of the crude residue by flash column chromatography (20% EtOAc in hexane) afforded the title compound as a colourless oil (229 mg, 1.04 mmol, 82%).

***R_f* value**: 0.30 (20% v/v ethyl acetate in hexane);

^{1}H NMR (400 MHz, $CDCl_3$): δ 7.31 (m, 2H, H-2), 7.13 (m, 2H, H-3), 3.65 (t, *J* = 6.6 Hz, 2H, H-8), 2.61 (dd, *J* = 8.0, 7.6 Hz, 2H, H-4), 1.71-1.57 (m, 4H, H-5, H-7), 1.43 (m, 2H, H-6), 1.35 (br. s, 1H, O-H), 1.32 (s, 9H, H-1) ppm;

^{13}C NMR (101 MHz, $CDCl_3$): δ 148.6, 139.6, 128.2, 125.3, 63.1, 35.5, 34.5, 32.8, 31.6, 31.4, 25.6 ppm;

HRMS (+ESI): *m/z* found $[M+H]^+$ 221.1889, $[C_{15}H_{25}O]^+$ requires 221.1900, (δ = − 5.0 ppm).

5-(3-fluoro-2-methylphenyl)pentan-1-ol (**6f**)

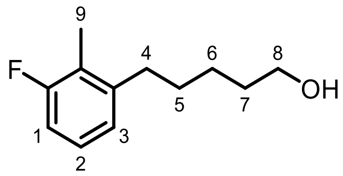

To a microwave vial charged with (*Z*)-5-(3-fluoro-2-methylphenyl)pent-4-en-1-ol (474 mg, 2.44 mmol) and Pd/C (10 wt %, 142 mg) was added EtOH (18 mL). The vial was sealed and then evacuated and backfilled with hydrogen three times. The resulting mixture was stirred at room temperature under a hydrogen balloon overnight. After completion, the mixture was filtered over Celite® eluting with EtOAc. The filtrate was collected, the solvent removed under reduced pressure and purification of the crude residue by flash column chromatography (20-40% EtOAc in hexane) afforded the title compound as a colourless oil (422 mg, 2.149 mmol, 88%).

***R_f* value**: 0.29 (20% v/v ethyl acetate in hexane);

^{1}H NMR (400 MHz, $CDCl_3$): *δ* 7.06 (m, 1H, H-2), 6.91 (d, *J* = 7.5 Hz, 1H, H-3), 6.86 (t, *J* = 9.2 Hz, 1H, H-1), 3.65 (t, *J* = 6.5 Hz, 2H, H-8), 2.63 (dd, *J* = 7.9, 7.7 Hz, 2H, H-4), 2.21 (d, *J* = 2.2 Hz, 3H, H-9), 1.65-1.56 (m, 4H, H-5, H-7), 1.49-1.31 (m, 3H, H-6, O-H) ppm;

^{13}C NMR (101 MHz, $CDCl_3$): *δ* 161.5 (d, J_{C-F} = 243 Hz), 143.4 (d, J_{C-F} = 4.0 Hz), 126.5 (d, J_{C-F} = 9.1 Hz), 124.4 (d, J_{C-F} = 3.0 Hz), 123.0 (d, J_{C-F} = 15.7 Hz), 112.6 (d, J_{C-F} = 23.3 Hz), 63.0, 33.3 (d, J_{C-F} = 2.7 Hz), 32.8, 30.3, 25.8, 10.5 (d, J_{C-F} = 6.0 Hz) ppm;

^{19}F NMR (376 MHz, $CDCl_3$): *δ* – 117.7 ppm;

HRMS (ESI): *The compound did not ionise.*

Synthesis of Control Substrate S1

(3-methoxypropyl)benzene (**S1**)

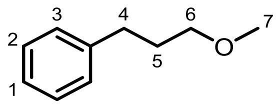

To an oven dried microwave vial charged with NaH (60% wt. 800 mg, 20.0 mmol) under a nitrogen atmosphere at 0 °C was added THF (8 mL). 3-phenyl-1-propanol (1.36 g, 10.0 mmol) in THF (8 mL) was added dropwise at the same temperature and the mixture was allowed to warm to room temperature over 1 hour following which methyl iodide (2.49 mL, 40 mmol) was added dropwise. After stirring at room temperature overnight, the reaction was quenched with H_2O and extracted with EtOAc × 3. The combined organic layers were dried over $MgSO_4$ and purified by flash column chromatography (SiO_2, 0-10% v/v EtOAc in hexane) to afford the title compound as a pale-yellow oil (921 mg, 6.13 mmol, 61%).

***R$_f$* value**: 0.59 (10% v/v ethyl acetate in hexane);

^{1}H NMR (400 MHz, $CDCl_3$): *δ* 7.32-7.16 (m, 5H, H-1, H-2, H-3), 3.40 (t, *J* = 6.3 Hz, 2H, H-6), 3.35 (s, 3H, H-7), 2.70 (t, *J* = 7.8 Hz, 2H, H-4), 1.90 (pent, *J* = 7.6 Hz, 2H, H-5) ppm;

^{13}C NMR (101 MHz, $CDCl_3$): *δ* 142.1, 128.6, 128.5, 125.9, 72.1, 58.7, 32.5, 31.4 ppm;

The spectroscopic data is in agreement with that reported in the literature.[36]

Synthesis of Allylic Alcohol Substrates

Ethyl 3-butylhept-2-enoate

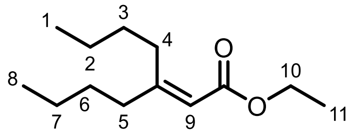

Prepared according to **GP8** on a 15 mmol scale with respect to 5-nonanone. In this case the following procedural modification was used: after addition of the ketone, the reaction was heated at reflux for 16 hours instead of room temperature for 2 hours. Purification by flash column chromatography (SiO_2, 0-3% v/v Et_2O in hexane) afforded the title compound as a colourless oil (276 mg, 1.301 mmol, 9%).

***R_f* value**: 0.61 (5% v/v Et_2O in hexane);

^{1}H NMR (400 MHz, $CDCl_3$): δ 5.61 (s, 1H, H-9), 4.13 (q, *J* = 7.0 Hz, 2H, H-10), 2.59 (m, 2H, C$\underline{H}_2$C=C), 2.13 (m, 2H, C$\underline{H}_2$C=C), 1.48-1.29 (m, 8H, H-2, H-3, H-6, H-7), 1.27 (t, *J* = 7.1 Hz, 3H, H-11), 0.93-0.88 (m, 6H, H-1, H-8) ppm;

^{13}C NMR (101 MHz, $CDCl_3$): δ 166.8, 165.0, 115.2, 59.5, 38.3, 32.1, 31.0, 30.0, 23.2, 22.6, 14.5, 14.09, 14.05 ppm;

The spectroscopic data is in agreement with that reported in the literature.[37]

Note: a particularly low yield in this instance is attributed to a challenging separation between the title compound and considerable amounts of unreacted 5-nonanone.

3-Butylhept-2-en-1-ol (**8b**)

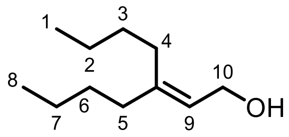

Prepared according to **GP9** on a 1.18 mmol scale with respect to ethyl 3-butylhept-2-enoate. In this case the following procedural modification was used: the title compound was obtained as a colourless oil (166 mg, 0.974 mmol, 82%) without purification by flash column chromatography.

***R_f* value**: 0.19 (20% v/v Et_2O in hexane);

^{1}H NMR (400 MHz, $CDCl_3$): δ 5.39 (t, *J* = 7.1 Hz, 1H, H-9), 4.15 (d, *J* = 6.6 Hz, 2H, H-10), 2.08-1.98 (m, 4H, H-4, H-5), 1.45-1.24 (m, 8H, H-2, H-3, H-6, H-7), 1.07 (br. s, 1H, O-H), 0.90 (t, *J* = 7.2 Hz, 6H, H-1, H-8) ppm;

^{13}C NMR (101 MHz, $CDCl_3$): δ 144.8, 123.4, 59.4, 36.7, 31.3, 30.33, 30.27, 22.9, 22.7, 14.15, 14.13 ppm;

The spectroscopic data is in agreement with that reported in the literature.[37]

Ethyl cyclohexylideneacetate

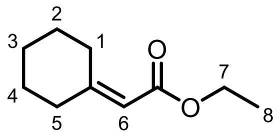

We are very grateful to Hannah K. Adams for the preparation of this compound.

Prepared according to **GP8** on a 10 mmol scale with respect to cyclohexanone. Purification by flash column chromatography (SiO_2, 0-20% v/v Et_2O in hexane) afforded the title compound as a pale-yellow oil (1.67 g, 9.93 mmol, quant.%).

^{1}H NMR (700 MHz, $CDCl_3$): δ 5.57 (br. s, 1H, H-6), 4.11 (q, J = 7.1 Hz, 2H, H-7), 2.80 (m, 2H, C<u>H</u>$_2$C=C), 2.17 (m, 2H, C<u>H</u>$_2$C=C), 1.65-1.55 (m, 6H, H-2, H-3, H-4), 1.25 (t, J = 7.1 Hz, 3H, H-8) ppm;

^{13}C NMR (176 MHz, $CDCl_3$): δ 166.9, 163.6, 113.1, 59.5, 38.1, 29.9, 28.7, 27.9, 26.4, 14.4 ppm;

The spectroscopic data is in agreement with that reported in the literature.[38]

2-Cyclohexylideneethan-1-ol (**8c**)

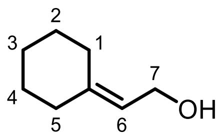

Prepared according to **GP9** on a 2.5 mmol scale with respect to ethyl cyclohexylideneacetate. In this case the following procedural modification was used: the title compound was obtained as a colourless oil (202 mg, 1.60 mmol, 64%) without purification by flash column chromatography.

^{1}H NMR (700 MHz, $CDCl_3$): δ 5.33 (t, J = 7.1 Hz, 1H, H-6), 4.10 (d, J = 7.2 Hz, 2H, H-7), 2.15 (m, 2H, C<u>H</u>$_2$C=C), 2.08 (m, 2H, C<u>H</u>$_2$C=C), 1.71 (br. s, 1H, O-H), 1.55-1.47 (m, 6H, H-2, H-3, H-4) ppm;

^{13}C NMR (176 MHz, $CDCl_3$): δ 144.4, 120.2, 58.5, 37.1, 28.9, 28.4, 27.9, 26.7 ppm;

The spectroscopic data is in agreement with that reported in the literature.[39]

Ethyl 2-cyclododecylideneacetate

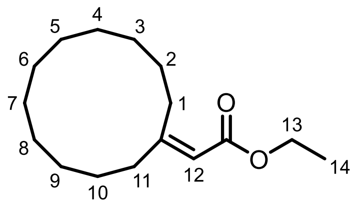

Prepared according to **GP8** on a 15 mmol scale with respect to cyclododecanone. Purification by flash column chromatography (SiO_2, 0-5% v/v Et_2O in hexane) afforded the title compound as a pale-yellow oil (378 mg, 1.498 mmol, 10%).

***R_f* value**: 0.56 (5% v/v Et_2O in hexane);

^{1}H NMR (700 MHz, $CDCl_3$): *δ* 5.74 (s, 1H, H-12), 4.14 (q, *J* = 7.2 Hz, 2H, H-13), 2.71 (t, *J* = 6.8 Hz, 2H, C$\underline{H}_2$C=C), 2.21 (td, *J* = 6.9, 1.2 Hz, 2H, C$\underline{H}_2$C=C), 1.62 (m, 2H, C$\underline{H}_2$CH$_2$C=C), 1.55 (m, 2H, C$\underline{H}_2$CH$_2$C=C), 1.38-1.25 (m, 17H, H-3, H-4, H-5, H-6, H-7, H-8, H-9, H-14) ppm;

^{13}C NMR (176 MHz, $CDCl_3$): *δ* 167.0, 163.2, 116.4, 59.6, 33.0, 29.9, 25.2, 25.0, 24.3, 24.16, 24.15, 23.9, 23.6, 23.1, 22.3, 14.5 ppm;

The spectroscopic data is in agreement with that reported in the literature.[40]

Note: a particularly low yield in this instance is attributed to a challenging separation between the title compound and considerable amounts of unreacted cyclododecanone.

2-Cyclododecylideneethan-1-ol (**8d**)

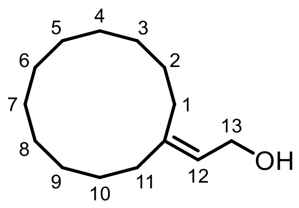

Prepared according to **GP9** on a 1.20 mmol scale with respect to ethyl 2-cyclododecylideneacetate. In this case the following procedural modification was used: the title compound was obtained as a colourless oil (194 mg, 0.922 mmol, 77%) without purification by flash column chromatography.

***R_f* value**: 0.42 (20% v/v EtOAc in hexane);

^{1}H NMR (400 MHz, $CDCl_3$): *δ* 5.50 (t, *J* = 7.0 Hz, 1H, H-12), 4.18 (d, *J* = 7.0 Hz, 2H, H-13), 2.13-2.04 (m, 4H, H-1, H-11), 1.57 (m, 2H, C$\underline{H}_2$CH$_2$C=C), 1.45 (m, 2H, C$\underline{H}_2$CH$_2$C=C), 1.38-1.23 (m, 14H, H-3, H-4, H-5, H-6, H-7, H-8, H-9), 1.19 (br. s, 1H, O-H) ppm;

^{13}C NMR (101 MHz, $CDCl_3$): *δ* 142.4, 124.2, 59.5, 31.7, 29.1, 25.2, 25.1, 24.3, 24.14, 24.05, 24.0, 23.4, 23.2, 22.3 ppm;

The spectroscopic data is in agreement with that reported in the literature.[41]

Ethyl 2-(4,4-difluorocyclohexylidene)acetate

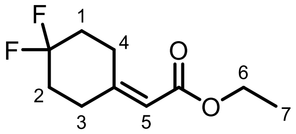

Prepared according to **GP8** on a 12 mmol scale with respect to 4,4-difluorocyclohexanone. Purification by flash column chromatography (SiO_2, 0-7% v/v Et_2O in hexane) afforded the title compound as a colourless oil (1.480 g, 7.246 mmol, 60%).

***Rf* value**: 0.31 (5% v/v Et_2O in hexane);

^{1}H NMR (400 MHz, $CDCl_3$): δ 5.72 (s, 1H, H-5), 4.15 (q, J = 7.1 Hz, 2H, H-6), 3.03 (dd, J = 6.8, 6.7 Hz, 2H, C<u>H</u>$_2$C=C), 2.40 (dd, J = 6.8, 6.5 Hz, 2H, C<u>H</u>$_2$C=C), 2.02 (m, 4H, H-1, H-2), 1.27 (t, J = 7.1 Hz, 3H, H-7) ppm;

^{13}C NMR (101 MHz, $CDCl_3$): δ 166.3, 156.7 (t, $J_{C\text{-}F}$ = 1.4 Hz), 122.6 (t, $J_{C\text{-}F}$ = 241 Hz), 116.1 (t, $J_{C\text{-}F}$ = 0.9 Hz), 60.0, 34.7 (t, $J_{C\text{-}F}$ = 24.5 Hz), 33.9 (t, $J_{C\text{-}F}$ = 24.4 Hz), 33.2 (t, $J_{C\text{-}F}$ = 5.4 Hz), 24.7 (t, $J_{C\text{-}F}$ = 5.3 Hz), 14.4 ppm;

^{19}F NMR (376 MHz, $CDCl_3$): δ – 98.3 ppm;

The ^{1}H NMR data is in agreement with that reported in the literature.[42]

2-(4,4-Difluorocyclohexylidene)ethan-1-ol (**8e**)

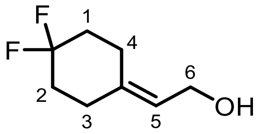

Prepared according to **GP9** on a 5 mmol scale with respect to ethyl 2-(4,4-difluorocyclohexylidene)acetate. Purification by flash column chromatography (SiO_2, 0-50% v/v EtOAc in hexane) afforded the title compound as a colourless oil (567 mg, 3.497 mmol, 70%).

***Rf* value**: 0.50 (50% v/v ethyl acetate in hexane);

^{1}H NMR (400 MHz, $CDCl_3$): δ 5.49 (t, J = 6.9 Hz, 1H, H-5), 4.16 (d, J = 6.9 Hz, 2H, H-6), 2.39-2.26 (m, 4H, H-3, H-4), 2.02-1.87 (m, 4H, H-1, H-2), 1.44 (br. s, 1H, O-H) ppm;

^{13}C NMR (101 MHz, $CDCl_3$): δ 138.7 (t, $J_{C\text{-}F}$ = 1.4 Hz), 123.7 (t, $J_{C\text{-}F}$ = 1.1 Hz), 123.3 (t, $J_{C\text{-}F}$ = 240 Hz), 58.7, 34.9 (t, $J_{C\text{-}F}$ = 23.7 Hz), 34.4 (t, $J_{C\text{-}F}$ = 24.1 Hz), 32.2 (t, $J_{C\text{-}F}$ = 5.4 Hz), 24.1 (t, $J_{C\text{-}F}$ = 5.5 Hz) ppm;

^{19}F NMR (376 MHz, $CDCl_3$): δ – 98.5 ppm;

HRMS (ESI): *this compound did not ionise.*

Ethyl 2-(tetrahydro-4H-pyran-4-ylidene)acetate

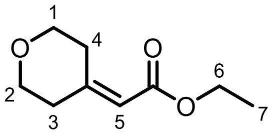

Prepared according to **GP8** on a 15 mmol scale with respect to tetrahydro-4*H*-pyran-4-one. Purification by flash column chromatography (SiO_2, 0-22% v/v Et_2O in hexane) afforded the title compound as a colourless oil (1.760 g, 10.3 mmol, 69%).

***R_f* value**: 0.33 (20% v/v Et_2O in hexane);

^{1}H NMR (400 MHz, $CDCl_3$): *δ* 5.66 (s, 1H, H-5), 4.13 (q, *J* = 7.1 Hz, 2H, H-6), 3.77-3.69 (m, 4H, H-1, H-2), 2.99 (t, *J* = 5.4 Hz, 2H, C<u>H</u>₂C=C), 2.31 (t, *J* = 5.4 Hz, 2H, C<u>H</u>₂C=C), 1.26 (t, *J* = 7.1 Hz, 3H, H-7) ppm;

^{13}C NMR (101 MHz, $CDCl_3$): *δ* 166.5, 157.3, 114.7, 69.2, 68.6, 59.8, 37.7, 31.1, 14.4 ppm;

The spectroscopic data is in agreement with that reported in the literature.[43]

2-(Tetrahydro-4H-pyran-4-ylidene)ethan-1-ol (**8f**)

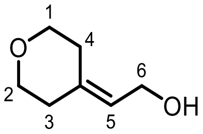

Prepared according to **GP9** on a 5 mmol scale with respect to ethyl 2-(tetrahydro-4H-pyran-4-ylidene)acetate. In this case the following procedural modification was used: the title compound was obtained as a white solid (521 mg, 4.065 mmol, 81%) without purification by flash column chromatography.

***R_f* value**: 0.21 (40% v/v EtOAc in hexane);

^{1}H NMR (700 MHz, $CDCl_3$): *δ* 5.43 (tt, *J* = 7.1, 1.1 Hz, 1H, H-5), 4.13 (d, *J* = 6.7 Hz, 2H, H-6), 3.69-3.63 (m, 4H, H-1, H-2), 2.30 (t, *J* = 5.4 Hz, 2H, C<u>H</u>₂C=C), 2.22 (t, *J* = 5.4 Hz, 2H, C<u>H</u>₂C=C), 1.78 (br. s, 1H, O-H) ppm;

^{13}C NMR (176 MHz, $CDCl_3$): *δ* 138.6, 122.4, 69.4, 68.7, 58.1, 36.8, 29.9 ppm;

The spectroscopic data is in agreement with that reported in the literature.[44]

Ethyl 2-(1,4-dioxaspiro[4.5]decan-8-ylidene)acetate

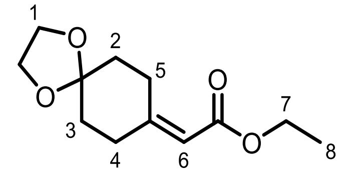

Prepared according to **GP8** on a 15 mmol scale with respect to 1,4-cyclohexanedione monoethylene acetal. Purification by flash column chromatography (SiO_2, 0-26% v/v Et_2O in hexane) afforded the title compound as a pale-yellow oil (2.197 g, 9.709 mmol, 65%).

***R_f* value**: 0.22 (20% v/v Et_2O in hexane);

^{1}H NMR (400 MHz, $CDCl_3$): δ 5.64 (s, 1H, H-6), 4.12 (q, *J* = 7.1 Hz, 2H, H-7), 3.95 (s, 4H, H-1), 2.98 (t, *J* = 6.8 Hz, 2H, C<u>H</u>$_2$C=C), 2.35 (t, *J* = 6.7 Hz, 2H, C<u>H</u>$_2$C=C), 1.78-1.70 (m, 4H, H-2, H-3), 1.25 (t, *J* = 7.0 Hz, 3H, H-8) ppm;

^{13}C NMR (101 MHz, $CDCl_3$): δ 166.6, 160.3, 114.4, 108.1, 64.5, 59.7, 35.9, 35.1, 34.7, 26.1, 14.4 ppm;

The spectroscopic data is in agreement with that reported in the literature.[45]

2-(1,4-Dioxaspiro[4.5]decan-8-ylidene)ethan-1-ol (**8g**)

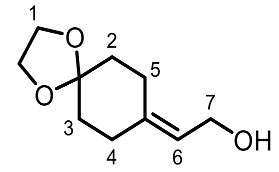

Prepared according to **GP9** on a 5 mmol scale with respect to ethyl 2-(1,4-dioxaspiro[4.5]decan-8-ylidene)acetate. Purification by flash column chromatography (SiO_2, 0-80% v/v EtOAc in hexane) afforded the title compound as a pale-yellow oil (257 mg, 1.393 mmol, 28%).

***R_f* value**: 0.30 (50% v/v ethyl acetate in hexane);

^{1}H NMR (400 MHz, $CDCl_3$): δ 5.43 (tt, *J* = 7.0, 1.1 Hz, 1H, H-6), 4.15 (d, *J* = 7.1 Hz, 2H, H-7), 3.96 (s, 4H, H-1), 2.23 (t, *J* = 6.4 Hz, 2H, C<u>H</u>$_2$C=C), 2.27 (t, *J* = 6.1 Hz, 2H, C<u>H</u>$_2$C=C), 1.73-1.65 (m, 4H, H-2, H-3), 1.26 (br. s, 1H, O-H) ppm;

^{13}C NMR (101 MHz, $CDCl_3$): δ 141.5, 122.0, 108.8, 64.5, 58.9, 36.1, 35.5, 33.6, 25.4 ppm;

The spectroscopic data is in agreement with that reported in the literature.[46]

*Note: in this reaction, we observed significant amounts of an acetal-opened product, which were easily separable from the desired compound. The low yield for **8g** is attributed to this.*

Ethyl 2-cyclopentylideneacetate

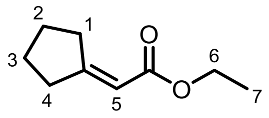

Prepared according to **GP8** on a 12 mmol scale with respect to cyclopentanone. In this case the following procedural modification was used: after addition of the ketone, the reaction was heated at 50 °C for 16 hours instead of room temperature for 2 hours. Purification by flash column chromatography (SiO_2, 0-10% v/v Et_2O in hexane) afforded the title compound as a colourless oil (1.652 g, 10.713 mmol, 89%).

***R_f* value**: 0.57 (10% v/v Et_2O in hexane);

^{1}H NMR (400 MHz, $CDCl_3$): δ 5.77 (s, 1H, H-5), 4.12 (q, J = 7.3 Hz, 2H, H-6), 2.75 (t, J = 7.1 Hz, 2H, C$\underline{H}_2$C=C), 2.41 (t, J = 7.0 Hz, 2H, C$\underline{H}_2$C=C), 1.77-1.60 (m, 4H, H-2, H-3), 1.25 (t, J = 7.3 Hz, 3H, H-7) ppm;

^{13}C NMR (101 MHz, $CDCl_3$): δ 169.1, 167.0, 111.8, 59.5, 36.1, 32.7, 26.5, 25.6, 14.5 ppm;

The spectroscopic data is in agreement with that reported in the literature.[47]

*Note: this compound contained 10% of the internal alkene isomer, presumably also formed in the Horner-Wadsworth-Emmons reaction. This isomer was partially separable at this stage but is fully separable in compound **8i** (below). The isolated yield in this instance refers to the mixture of isomers.*

2-Cyclopentylideneethan-1-ol (**8i**)

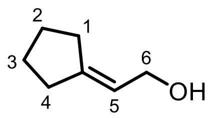

Prepared according to **GP9** on a 7 mmol scale with respect to ethyl 2-cyclopentylideneacetate. Purification by flash column chromatography (SiO_2, 0-20% v/v EtOAc in hexane) afforded the title compound as a colourless oil (346 mg, 3.085 mmol, 44%).

***R_f* value**: 0.24 (20% v/v ethyl acetate in hexane);

^{1}H NMR (700 MHz, $CDCl_3$): δ 5.49 (m, 1H, H-5), 4.11 (t, J = 6.2 Hz, 2H, H-6), 2.29-2.22 (m, 4H, H-1, H-4), 1.70-1.57 (m, 4H, H-2, H-3), 1.40 (br. s, 1H, O-H) ppm;

^{13}C NMR (176 MHz, $CDCl_3$): δ 147.9, 119.2, 61.1, 33.8, 28.7, 26.4, 26.1 ppm;

The spectroscopic data is in agreement with that reported in the literature.[47]

Ethyl 2-cycloheptylideneacetate

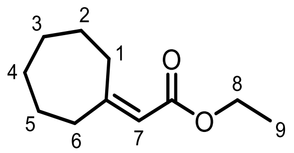

Prepared according to **GP8** on a 12 mmol scale with respect to cycloheptanone. In this case the following procedural modification was used: after addition of the ketone, the reaction was heated at 50 °C for 16 hours instead of room temperature for 2 hours. Purification by flash column chromatography (SiO_2, 0-10% v/v Et_2O in hexane) afforded the title compound as a pale-yellow oil (1.951 g, 10.704 mmol, 89%).

***R_f* value**: 0.69 (10% v/v Et_2O in hexane);

^{1}H NMR (400 MHz, $CDCl_3$): *δ* 5.67 (s, 1H, H-7), 4.14 (q, *J* = 7.2 Hz, 2H, H-8), 2.88 (m, 2H, C$\underline{H}_2$C=C), 2.38 (m, 2H, C$\underline{H}_2$C=C), 1.74-1.49 (m, 8H, H-2, H-3, H-4, H-5), 1.28 (t, *J* = 7.2 Hz, 3H, H-9) ppm;

^{13}C NMR (101 MHz, $CDCl_3$): *δ* 166.78, 166.77, 115.7, 59.5, 39.1, 32.2, 30.0, 29.1, 28.2, 26.7, 14.5 ppm;

The spectroscopic data is in agreement with that reported in the literature.[47]

2-Cycloheptylideneethan-1-ol (**8j**)

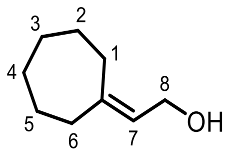

Prepared according to **GP9** on a 5 mmol scale with respect to ethyl 2-cycloheptylideneacetate. In this case the following procedural modification was used: the title compound was obtained as a colourless oil (557 mg, 3.973 mmol, 79%) without purification by flash column chromatography.

***R_f* value**: 0.34 (20% v/v EtOAc in hexane);

^{1}H NMR (400 MHz, $CDCl_3$): *δ* 5.39 (tpent, *J* = 6.9, 1.3 Hz, 1H, H-7), 4.13 (d, *J* = 6.9 Hz, 2H, H-8), 2.31-2.20 (m, 4H, H-1, H-6), 1.61-1.46 (m, 8H, H-2, H-3, H-4, H-5), 1.30 (br. s, 1H, O-H) ppm;

^{13}C NMR (101 MHz, $CDCl_3$): *δ* 145.8, 124.0, 59.3, 37.9, 30.1, 29.9, 29.1, 29.0, 27.5 ppm;

The spectroscopic data is in agreement with that reported in the literature.[47]

Synthesis of Aminating Agents and Oxidants

The synthesis of 2,2,3,3,4,4,4-heptafluorobutyl sulfamate,[6] *2,2,2-trichloroethyl sulfamate*[48] *and 2,2,3,3,3-pentafluoropropyl sulfamate*[5] *has been reported previously.*

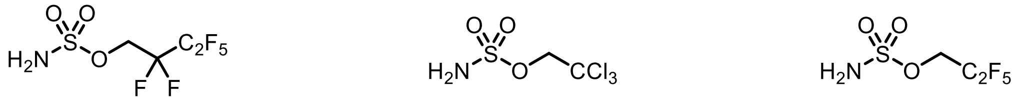

2,2,3,3,4,4,4-heptafluorobutyl sulfamate *2,2,2-trichloroethyl sulfamate* *2,2,3,3,3-pentafluoropropyl sulfamate*

The synthesis of $C_6F_5I(OTFA)_2$ and C_6F_5IO has been reported previously.[5]

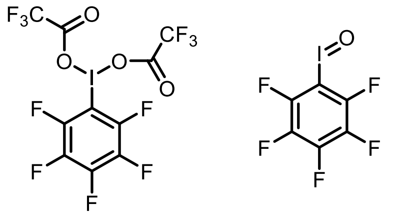

$C_6F_5I(OTFA)_2$ C_6F_5IO

Note: we have found it very important to thoroughly remove excess trifluoroacetic acid when preparing $C_6F_5I(OTFA)_2$ since any excess trifluoroacetic acid will adversely affect the reaction outcome.

Enantioselective Intermolecular C-H Amination Products

2,2,3,3,4,4,4-heptafluorobutyl (R)-(3-hydroxy-1-phenylpropyl)sulfamate (**2a**)

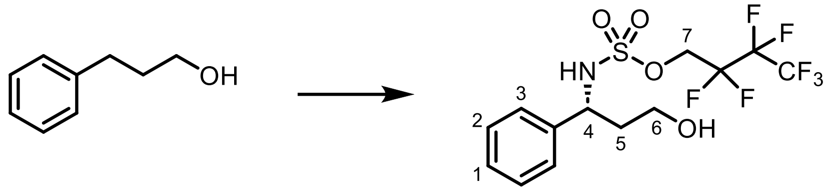

Prepared according to **GP10** using 3-phenylpropan-1-ol as the starting material. Purification by flash column chromatography (SiO_2, 0-20% acetone in $CHCl_3$) afforded the title compound as a white solid (34.2 mg, 0.083 mmol, 83%, 92% *ee*).

***R_f* value**: 0.41 (20% v/v acetone in $CHCl_3$);

^{1}H NMR (700 MHz, $CDCl_3$): *δ* 7.37 (m, 2H, H-2), 7.34-7.29 (m, 3H, H-1, H-3), 6.40 (d, *J* = 6.6 Hz, 1H, N-H), 4.75 (m, 1H, H-4), 4.24 (q, *J* = 12.6 Hz, 1H, H-7_A), 4.18 (q, *J* = 12.6 Hz, 1H, H-7_B), 3.78 (m, 2H, H-6), 2.12 (m, 1H, H-5_A), 2.07-1.85 (m, 2H, H-5_B, O-H) ppm;

^{13}C NMR (176 MHz, $CDCl_3$): *δ* 140.1, 129.0, 128.3, 126.6, 117.5 (qt, *J* = 287.4, 33.7 Hz), 113.2 (tt, *J* = 258.4, 31.0 Hz), 110.3-106.6 (m), 64.1 (t, *J* = 27.1 Hz), 60.0, 58.1, 38.2 ppm;

^{19}F NMR (376 MHz, $CDCl_3$): *δ* – 80.9 (t, *J* = 9.3 Hz), – 120.7 (m), – 127.5 ppm;

The following data was obtained from a sample of **2a** when using Rh_2(**A**-**III**)$_2$●(**Cat8**)$_2$●(**pyr**)$_2$ as the catalyst (*i.e.* the best performing catalyst in terms of *ee* for **2a**):

$[\alpha]_D^{25.0}$ = + 39.8 (c. 1.7, $CHCl_3$);

Chiral SFC Analysis: CHIRALPAK IG (CO_2:MeOH, 97.5:2.5, 2.50 mL min^{-1}, 40 °C) indicated 95% *ee*, t_R = 7.6 (minor), 8.2 (major) minutes.

The spectroscopic data is in agreement with the previous literature report.[6]

2,2,2-trichloroethyl (R)-(3-hydroxy-1-phenylpropyl)sulfamate (**2b**)

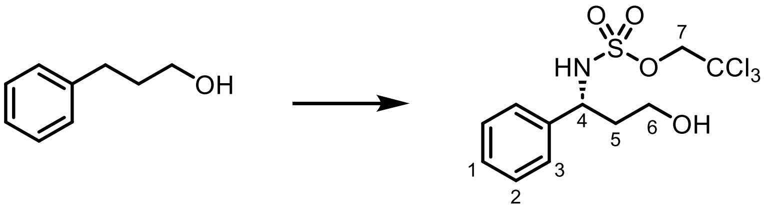

Prepared according to **GP10** using 3-phenylpropan-1-ol as the starting material. Purification by flash column chromatography (SiO_2, 0-23% acetone in $CHCl_3$) afforded the title compound as a white solid (26.9 mg, 0.074 mmol, 74%, 93% *ee*). Recrystallization of **2a** (18.6 mg, 0.051 mmol) from hot $CHCl_3$ afforded a more enantioenriched product (8.7 mg, 0.024 mmol, 47%, >99% *ee*).

***R_f* value**: 0.44 (20% v/v acetone in $CHCl_3$);

^{1}H NMR (700 MHz, CD_3OD): δ 7.39-7.34 (m, 4H, H-2, H-3), 7.28 (m, 1H, H-1), 4.63 (t, *J* = 7.6 Hz, 1H, H-4), 4.30 (d, *J* = 10.9 Hz, 1H, H-7A), 4.12 (d, *J* = 10.8 Hz, 1H, H-7B), 3.62 (dt, *J* = 10.9, 6.2 Hz, 1H, H-6A), 3.48 (dt, *J* = 10.9, 6.3 Hz, 1H, H-6B), 2.11 (sext, *J* = 6.5 Hz, 1H, H-5A), 1.94 (sext, *J* = 6.8 Hz, 1H, H-5B) ppm;

^{13}C NMR (176 MHz, CD_3OD): δ 143.0, 129.8, 128.8, 127.9, 94.8, 78.8, 59.3, 57.1, 40.7 ppm;

HRMS (−ESI) *m/z* Found $[M-(CH_2CCl_3)]^-$ 230.0493, $[C_9H_{12}NO_4S]^-$ requires 230.0493, (*δ* = + 0.0 ppm);

For **2b** after recrystallization:

$[\alpha]_D^{25.0}$ = + 17.6 (c. 0.14, MeOH);

Chiral SFC Analysis: CHIRALPAK IG (CO_2:MeOH, 85.0:15.0, 2.50 mL min^{-1}, 40 °C) t_R = 4.6 (minor), 6.2 (major) minutes.

2,2,2-trichloroethyl (S)-(3-hydroxy-1-phenylpropyl)sulfamate (**2b-ent**)

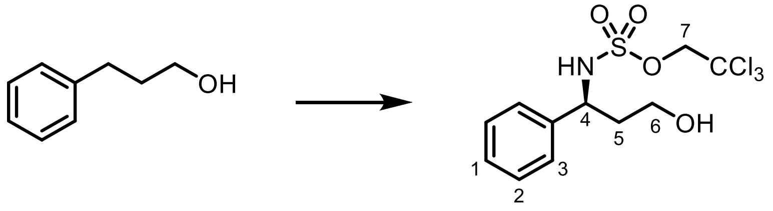

Prepared according to **GP10** using 3-phenylpropan-1-ol as the starting material. Purification by flash column chromatography (SiO_2, 0-23% acetone in $CHCl_3$) afforded the title compound as a white solid (35.0 mg, 0.096 mmol, 96%, (−)89% *ee*).

$[\alpha]_D^{25.0}$ = − 14.7 (c. 2.0, MeOH);

Chiral SFC Analysis: CHIRALPAK IG (CO_2:MeOH, 85.0:15.0, 2.50 mL min^{-1}, 40 °C) t_R = 4.6 (major), 6.3 (minor) minutes.

2,2,2-trichloroethyl (R)-(3-hydroxy-1-(o-tolyl)propyl)sulfamate (**2c**)

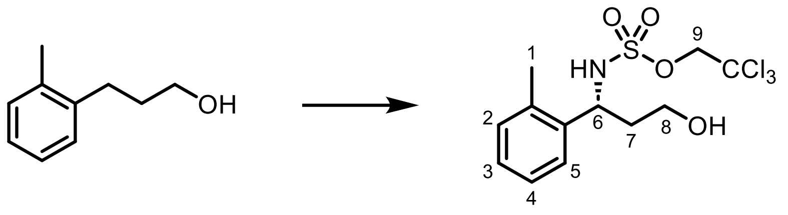

Prepared according to **GP10** using 3-(o-tolyl)propan-1-ol as the starting material. Purification by flash column chromatography (SiO_2, 0-20% acetone in $CHCl_3$) afforded the title compound as a colourless oil (36.7 mg, 0.097 mmol, 97%, 94% *ee*).

***Rf* value**: 0.42 (20% v/v acetone in $CHCl_3$);

^{1}H NMR (700 MHz, $CDCl_3$): δ 7.37 (d, *J* = 7.7 Hz, 1H, H-5), 7.25 (t, *J* = 7.4 Hz, 1H, H-4), 7.19 (t, *J* = 7.4 Hz, 1H, H-3), 7.16 (d, *J* = 7.3 Hz, 1H, H-2), 6.30 (d, *J* = 6.7 Hz, 1H, N-H), 5.09 (m, 1H, H-6), 4.33 (d, *J* = 10.8 Hz, 1H, H-9$_A$), 4.26 (d, *J* = 10.8 Hz, 1H, H-9$_B$), 3.83 (m, 1H, H-8$_A$), 3.77 (m, 1H, H-8$_B$), 2.40 (s, 3H, H-1), 2.09 (m, 1H, H-7$_A$), 2.05-1.05 (m, 2H, H-7$_B$, O-H) ppm;

^{13}C NMR (176 MHz, $CDCl_3$): δ 138.6, 135.0, 131.0, 128.0, 126.7, 126.1, 93.41 (d, *J* = 0.7 Hz), 78.1, 60.0, 53.9, 37.6, 19.2 ppm;

HRMS (−ESI) *m/z* Found $[M-H]^-$ 373.9791, $[C_{12}H_{16}Cl_3NO_4S]^-$ requires 373.9793, (δ = – 0.5 ppm);

$[\alpha]_D^{25.0}$ = + 29.3 (c. 2.2, $CHCl_3$);

Chiral SFC Analysis: CHIRALPAK IG (CO_2:MeOH, 90.0:10.0, 2.50 mL min^{-1}, 40 °C) t_R = 6.2 (minor), 6.9 (major) minutes.

2,2,2-trichloroethyl (R)-(3-hydroxy-1-(2-methoxyphenyl)propyl)sulfamate (**2d**)

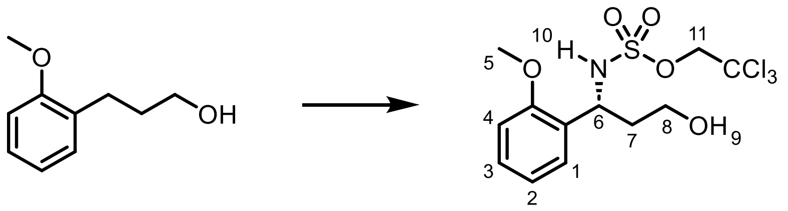

Prepared according to **GP10** using 3-(2-methoxyphenyl)propan-1-ol as the starting material. Purification by flash column chromatography (SiO_2, 0-30% v/v Acetone in Petrol) afforded the title compound as white needles (35.1 mg, 0.089 mmol, 89%, 95% *ee*).

***Rf* value** = 0.42 (30% v/v Acetone in Petrol);

^{1}H NMR (400 MHz, $CDCl_3$) δ 7.25-7.30 (m, 2H, H-1, H-3), 6.96 (d, J = 7.4 Hz, 1H, H-4), 6.93 (t, J = 8.5 Hz, 1H, H-2), 6.26 (d, J = 9.3 Hz, 1H, H-10), 4.85 (td, J = 8.7 Hz, 6.1 Hz, 1H, H-6), 4.38 (d, J = 10.8 Hz, 1H, H-11a), 4.28 (d, J = 10.7 Hz, 1H, H-11b), 3.88 (s, 3H, H-5), 3.73-3.82 (m, 1H, H-8a), 3.63-3.69 (m, 1H, H-8b), 2.14-2.22 (m, 1H, H-7a), 2.02-2.10 (m, 1H, H-7b), 1.88 (s, 1H, H-9) ppm;

^{13}C NMR (101 MHz, $CDCl_3$) δ 156.7, 129.5, 129.1, 127.9, 121.3, 111.3, 93.4, 78.1, 59.5, 55.6, 55.4, 37.5 ppm;

HRMS (–ESI) *m/z* Found $[M{-}H]^-$ 389.9743, $[C_{12}H_{15}Cl_3NO_5S]^-$ requires 389.9742, (δ = + 0.3 ppm);

$[\alpha]_D^{25.0}$ = + 18.0 (c. 1.2, CH_3CN);

Chiral HPLC Analysis: CHIRAL ART SC (Hexane: ^{i}PrOH, 88:12, 1.5 mL min^{-1}, 40 °C) t_R = 10.8 (minor), 11.6 (major) minutes.

2,2,2-trichloroethyl (R)-(1-(2-chlorophenyl)-3-hydroxypropyl)sulfamate (**2e**)

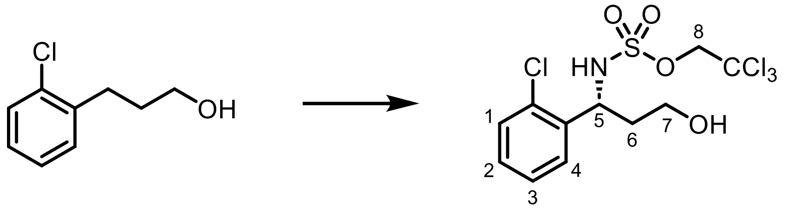

Prepared according to **GP10** using 3-(2-chlorophenyl)propan-1-ol as the starting material. Purification by flash column chromatography (SiO_2, 0-20% v/v acetone in $CHCl_3$) afforded the title compound as a colorless oil (32.6 mg, 0.082 mmol, 82%, 92% *ee*).

***R$_f$* value**: 0.47 (20% v/v acetone in $CHCl_3$);

^{1}H NMR (700 MHz, $CDCl_3$): δ 7.52 (dd, J = 7.7, 1.6 Hz, 1H, H-4), 7.36 (dd, J = 7.9, 1.4 Hz, 1H, H-1), 7.31 (td, J = 7.6, 1.4 Hz, 1H, H-3), 7.24 (td, J = 7.6, 1.7 Hz, 1H, H-2), 6.72 (d, J = 6.9 Hz, 1H, N-H), 5.25 (m, 1H, H-5), 4.47 (m, 2H, H-8_A, H-8_B), 3.80 (m, 2H, H-7_A, H-7_B), 2.22 (m, 1H, H-6_A), 2.06 (m, 1H, H-6_B), 1.93 (br. s, 1H, O-H) ppm;

^{13}C NMR (176 MHz, $CDCl_3$): δ 137.6, 132.1, 130.2, 129.3, 128.7, 127.4, 93.5, 78.2, 60.1, 55.6, 36.1 ppm;

HRMS (–ESI) *m/z* Found $[M{-}H]^-$ 393.9248, $[C_{11}H_{12}Cl_4NO_4S]^-$ requires 393.9247, (δ = + 0.3 ppm);

$[\alpha]_D^{25.0}$ = + 37.3 (c. 1.9, $CHCl_3$);

Chiral SFC Analysis: CHIRALPAK IG (CO_2:MeOH, 88.0:12.0, 2.50 mL min^{-1}, 40 °C) t_R = 5.4 (minor), 5.7 (major) minutes.

2,2,2-trichloroethyl (R)-(1-(2-bromophenyl)-3-hydroxypropyl)sulfamate (**2f**)

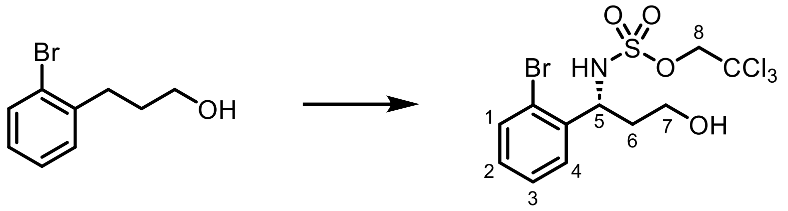

Prepared according to **GP10** using 3-(2-bromophenyl)propan-1-ol as the starting material. Purification by flash column chromatography (SiO_2, 0-18% v/v Et_2O in CH_2Cl_2) afforded the title compound as a colorless oil (45.4 mg, quant.%, 94% *ee*).

***R_f* value**: 0.39 (15% v/v Et_2O in CH_2Cl_2);

^{1}H NMR (700 MHz, $CDCl_3$): δ 7.56-7.52 (m, 2H, H-1, H-4), 7.36 (t, *J* = 7.6 Hz, 1H, H-3), 7.16 (td, *J* = 7.5, 1.5 Hz, 1H, H-2), 6.78 (d, *J* = 6.6 Hz, 1H, N-H), 5.24 (m, 1H, H-5), 4.48 (s, 2H, H-8), 3.85 (m, 1H, H-7_A), 3.77 (m, 1H, H-7_B), 2.21 (m, 1H, H-6_A), 2.04 (m, 1H, H-6_B), 1.84 (br. s, 1H, O-H) ppm;

^{13}C NMR (176 MHz, $CDCl_3$): δ 139.2, 133.5, 129.5, 128.7, 128.0, 122.2, 93.5, 78.2, 60.2, 57.7, 36.1 ppm;

HRMS (−ESI) *m/z* Found $[M−H]^−$ 437.8748, $[C_{11}H_{12}BrCl_3NO_4S]^−$ requires 437.8741, (*δ* = + 1.6 ppm);

$[\alpha]_D^{25.0}$ = + 33.5 (c. 2.0, $CHCl_3$);

Chiral SFC Analysis: CHIRALPAK IG (CO_2:*i*PrOH, 83.0:17.0, 2.50 mL min^{-1}, 40 °C) t_R = 4.9 (major), 5.9 (minor) minutes.

2,2,2-trichloroethyl (R)-(3-hydroxy-1-(m-tolyl)propyl)sulfamate (**2g**)

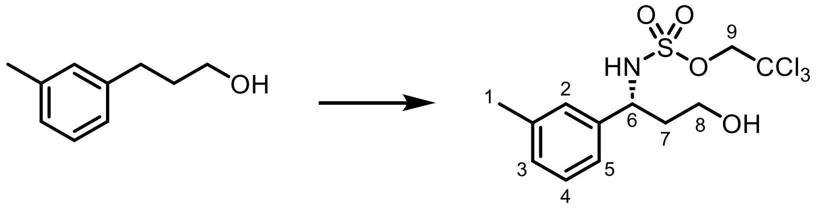

Prepared according to **GP10** using 3-(m-tolyl)propan-1-ol as the starting material. Purification by flash column chromatography (SiO_2, 0-20% v/v acetone in $CHCl_3$) afforded the title compound as a white solid (30.9 mg, 0.082 mmol, 82%, 93% *ee*).

***R_f* value**: 0.45 (20% v/v acetone in $CHCl_3$);

^{1}H NMR (700 MHz, CD_3OD): δ 7.24 (t, *J* = 7.6 Hz, 1H, H-4), 7.20 (s, 1H, H-2), 7.16 (d, *J* = 7.5 Hz, 1H, H-5), 7.11 (d, *J* = 7.4 Hz, 1H, H-3), 4.58 (t, *J* = 7.7 Hz, 1H, H-6), 4.28 (d, *J* = 10.8 Hz, 1H, H-9_A), 4.08 (d, *J* = 10.8 Hz, 1H, H-9_B), 3.61 (dt, J = 10.8, 6.3 Hz, 1H, H-8_A), 3.48 (dt, *J* = 10.7, 6.3 Hz, 1H, H-8_B), 2.34 (s, 3H, H-1), 2.10 (m, 1H, H-7_A), 1.92 (sext, *J* = 6.7 Hz, 1H, H-7_B) ppm;

^{13}C NMR (176 MHz, CD_3OD): δ 143.0, 139.6, 129.7, 129.5, 128.7, 125.0, 94.9 (d, *J* = 0.7 Hz), 78.9, 59.4, 57.2, 40.8, 21.5 ppm;

HRMS (−ESI) *m/z* Found $[M-H]^-$ 373.9795, $[C_{12}H_{15}Cl_3NO_4S]^-$ requires 373.9793, (*δ* = + 0.5 ppm);

$[\alpha]_D^{25.0}$ = + 13.2 (c. 1.4, MeOH);

Chiral SFC Analysis: CHIRALPAK IG (CO_2:MeOH, 88.0:12.0, 2.50 mL min^{-1}, 40 °C) t_R = 5.0 (minor), 5.8 (major) minutes.

2,2,2-trichloroethyl (R)-(1-(3-(tert-butyl)phenyl)-3-hydroxypropyl)sulfamate (**2h**)

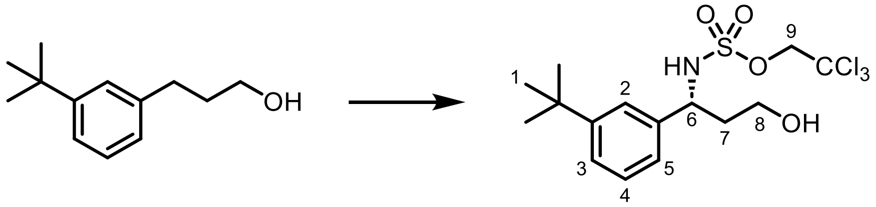

Prepared according to **GP10** using 3-(3-(tert-butyl)phenyl)propan-1-ol as the starting material. Purification by flash column chromatography (SiO_2, 0-18% v/v acetone in $CHCl_3$) afforded the title compound as a white solid (29.4 mg, 0.070 mmol, 70%, 90% *ee*).

***R_f* value**: 0.55 (20% v/v acetone in $CHCl_3$);

^{1}H NMR (700 MHz, CD_3OD): δ 7.44 (s, 1H, H-2), 7.34 (d, *J* = 7.9 Hz, 1H, H-3), 7.29 (t, *J* = 7.7 Hz, 1H, H-4), 7.17 (d, *J* = 7.6 Hz, 1H, H-5), 4.61 (t, *J* = 7.5 Hz, 1H, H-6), 4.20 (d, *J* = 10.8 Hz, 1H, H-9_A), 4.02 (d, *J* = 10.8 Hz, 1H, H-9_B), 3.62 (m, 1H, H-8_A), 3.49 (m, 1H, H-8_B), 2.10 (sext, *J* = 6.5 Hz, 1H, H-7_A), 1.92 (sext, *J* = 6.7 Hz, 1H, H-7_B), 1.33 (s, 9H, H-1) ppm;

^{13}C NMR (176 MHz, CD_3OD): δ 153.0, 142.9, 129.6, 125.9, 125.2, 124.8, 94.9 (d, *J* = 0.7 Hz), 78.9, 59.4, 57.4, 41.1, 35.6, 31.8 ppm;

HRMS (−ESI) *m/z* Found $[M-(CH_2CCl_3)]^-$ 286.1117, $[C_{13}H_{20}NO_4S]^-$ requires 286.1119, (*δ* = − 0.7 ppm);

$[\alpha]_D^{25.0}$ = + 28.5 (c. 1.9, $CHCl_3$);

Chiral HPLC Analysis: CHIRAL ART SC (hexane:*i*PrOH, 92.0:8.0, 1.25 mL min^{-1}, 40 °C) t_R = 9.6 (minor), 10.6 (major) minutes.

2,2,2-trichloroethyl (R)-(3-hydroxy-1-(3-(trifluoromethyl)phenyl)propyl)sulfamate (**2i**)

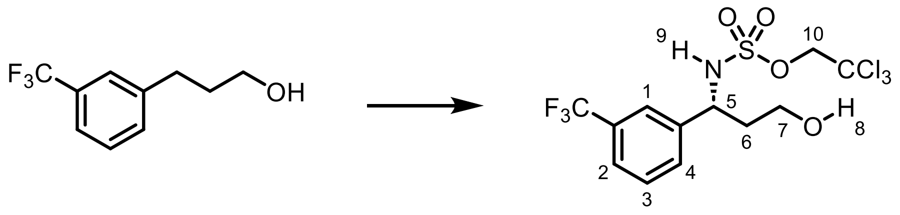

Prepared according to **GP10** using 3-(3-(trifluoromethyl)phenyl)propan-1-ol as the starting material. In this case the following procedural modification was used: The reaction was performed using 2 mol % of catalyst and the reaction temperature was – 25 °C. Purification by flash column chromatography (SiO_2, 0-20% v/v Acetone in $CHCl_3$) afforded the title compound as a colourless oil (16.0 mg, 0.037 mmol, 37%, 93% *ee*).

***Rf* value** = 0.45 (20% v/v Acetone in $CHCl_3$);

^{1}H NMR (700 MHz, $CDCl_3$) δ 7.63 (br s, 1H, H-1), 7.57-7.58 (m, 2H, H-2, H-4), 7.51 (t, *J* = 7.7 Hz, 1H, H-3), 6.55 (d, *J* = 6.2 Hz, 1H, H-9), 4.92 (td, *J* = 7.4 Hz, 4.6 Hz, 1H, H-5), 4.49 (d, *J* = 10.8 Hz, 1H, H-10a), 4.45 (d, *J* = 10.8 Hz, 1H, H-10b), 3.83-3.86 (m, 1H, H-7a), 3.79-3.82 (m, 1H, H-7b), 2.17-2.23 (m, 1H, H-6a), 2.00-2.05 (m, 1H, H-6b) ppm;

^{13}C NMR (176 MHz, $CDCl_3$) δ 141.4, 131.4 (q, J_{C-F} = 32.4 Hz), 130.2 (q, J_{C-F} = 1.1 Hz), 129.5, 125.0 (q, J_{C-F} = 3.7 Hz), 124.1 (q, J_{C-F} = 272.4 Hz), 123.4 Hz (q, J_{C-F} = 3.8 Hz), 93.35-93.36 (m), 78.1, 60.0, 58.0, 37.9 ppm;

^{19}F NMR (376 MHz, $CDCl_3$) δ – 62.6 (s) ppm;

HRMS (+ESI) *m/z* Found $[M+Na]^+$ 451.9477, $[C_{12}H_{13}Cl_3F_3NNaO_4S]^+$ requires 451.9475, (δ = + 0.4 ppm);

$[\alpha]_D^{25.0}$ = + 37.4 (c. 1.3, $CHCl_3$);

Chiral SFC Analysis: CHIRALPAK IG (CO_2: MeOH, 93:7, 2.5 mL min^{-1}, 40 °C) t_R = 4.5 (minor), 4.9 (major) minutes.

ethyl (R)-3-(3-hydroxy-1-(((2,2,2-trichloroethoxy)sulfonyl)amino)propyl)benzoate (**2j**)

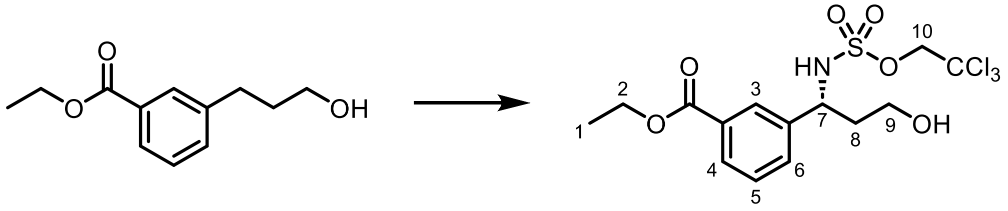

Prepared according to **GP10** using ethyl 3-(3-hydroxypropyl)benzoate as the starting material. Purification by flash column chromatography (SiO_2, 0-20% v/v acetone in $CHCl_3$) afforded the title compound as a colorless oil (28.5 mg, 0.066 mmol, 66%, 91% *ee*).

***Rf* value**: 0.47 (20% v/v acetone in $CHCl_3$);

^{1}H NMR (700 MHz, $CDCl_3$): δ 8.05 (s, 1H, H-3), 7.98 (d, *J* = 7.8 Hz, 1H, H-4), 7.57 (d, *J* = 7.7 Hz, 1H, H-6), 7.45 (t, *J* = 7.7 Hz, 1H, H-5), 6.61 (d, *J* = 6.8 Hz, 1H, N-H), 4.90 (m, 1H, H-7), 4.47 (d, *J* = 10.7 Hz, 1H, H-10_A), 4.43 (d, *J* = 10.7 Hz, 1H, H-10_B), 4.38 (q, *J* = 7.1 Hz, 2H, H-2), 3.80 (br. t, *J* = 5.5 Hz, 2H, H-9), 2.20-2.10 (m, 2H, H-8_A, O-H), 2.05 (m, 1H, H-8_B), 1.40 (t, *J* = 7.2 Hz, 3H, H-1) ppm;

^{13}C NMR (176 MHz, $CDCl_3$): δ 166.6, 140.9, 131.4, 131.2, 129.3, 129.1, 127.6, 93.4 (d, *J* = 0.8 Hz), 78.1, 61.5, 59.9, 57.8, 38.2, 14.5 ppm;

HRMS (−ESI) *m/z* Found $[M-H]^-$ 431.9847, $[C_{14}H_{17}Cl_3NO_6S]^-$ requires 431.9848, (δ = − 0.2 ppm);

$[\alpha]_D^{25.0}$ = + 48.2 (c. 1.6, $CHCl_3$);

Chiral SFC Analysis: CHIRALPAK IG (CO_2:MeOH, 85.0:15.0, 2.50 mL min^{-1}, 40 °C) t_R = 6.4 (minor), 6.8 (major) minutes.

2,2,2-trichloroethyl (R)-(3-hydroxy-1-(naphthalen-1-yl)propyl)sulfamate (**2k**)

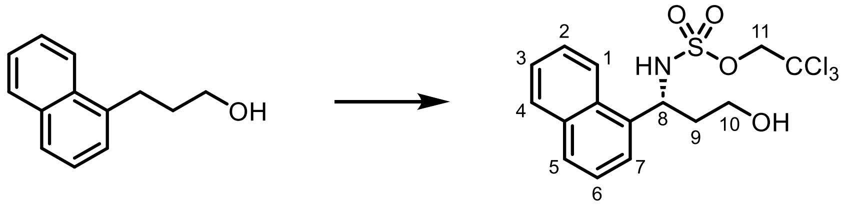

Prepared according to **GP10** using 3-(naphthalen-1-yl)propan-1-ol as the starting material. Purification by flash column chromatography (SiO_2, 0-20% v/v acetone in $CHCl_3$) afforded the title compound as a colorless oil (31.8 mg, 0.077 mmol, 77%, 84% *ee*).

***R_f* value**: 0.51 (20% v/v acetone in $CHCl_3$);

^{1}H NMR (400 MHz, $CDCl_3$): δ 8.07 (d, *J* = 8.4 Hz, 1H, H-1), 7.89 (d, *J* = 7.9 Hz, 1H, H-4), 7.81 (d, *J* = 8.2 Hz, 1H, H-5), 7.65 (d, *J* = 7.2 Hz, 1H, H-7), 7.58-7.48 (m, 3H, H-2, H-3, H-6), 6.63 (d, *J* = 6.9 Hz, 1H, N-H), 5.69 (m, 1H, H-8), 4.37 (q, *J* = 10.8 Hz, 2H, H-11), 3.79 (m, 2H, H-10), 2.36 (m, 1H, H-9_A), 2.16 (m, 1H, H-9_B) 2.03 (br. s, 1H, O-H) ppm;

^{13}C NMR (101 MHz, $CDCl_3$): δ 135.8, 134.1, 130.1, 129.3, 128.7, 126.8, 126.0, 125.4, 124.3, 122.5, 93.4, 78.2, 60.1, 54.3, 37.6 ppm;

HRMS (−ESI): *m/z* found $[M-H]^-$ 409.9795, $[C_{15}H_{15}Cl_3NO_4S]^-$ requires 409.9793, (δ = + 0.5 ppm).

2k was also prepared according to **GP10** with the following procedural modification: Rh_2(**A-III**)$_2$•(**Cat8**)$_2$•(**pyr**)$_2$ was used as the chiral catalyst. Purification by flash column chromatography (SiO_2, 0-20% v/v acetone in $CHCl_3$) afforded the title compound as a colorless oil (36.4 mg, 0.088 mmol, 88%, 89% *ee*).

$[\alpha]_D^{25.0}$ = + 26.9 (c. 1.9, $CHCl_3$);

Chiral SFC Analysis: CHIRALPAK IK (CO_2:MeOH, 85.0:15.0, 2.50 mL min^{-1}, 40 °C) t_R = 5.6 (minor), 6.1 (major) minutes.

2,2,2-trichloroethyl (R)-(1-(4-chlorophenyl)-3-hydroxypropyl)sulfamate (**2l**)

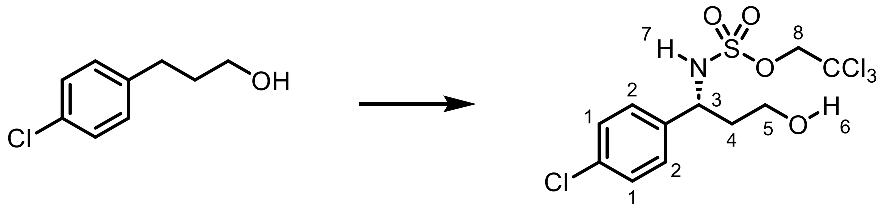

Prepared according to **GP10** using 3-(4-chlorophenyl)propan-1-ol as the starting material. In this case the following procedural modifications were used: The reaction time was 36 hours. The reaction was performed on 0.11 mmol scale and all reagent and solvent amounts were scaled proportionally. Purification by flash column chromatography (SiO_2, 0-20% v/v Acetone in $CHCl_3$) afforded the title compound as a white amorphous solid (36.4 mg, 0.092 mmol, 83%, 87% *ee*).

***R**$_f$* **value** = 0.46 (20% v/v Acetone in $CHCl_3$);

^{1}H NMR (700 MHz, $(CD_3)_2CO$) δ 7.73 (br s, 1H, H-7), 7.48 (d, J = 8.4 Hz, 2H, H-1), 7.41 (d, J = 8.5 Hz, 2H, H-2), 4.80 (t, J = 7.2 Hz, 1H, H-3), 4.56 (d, J = 11.0 Hz, 1H, H-8a), 4.46 (d, J = 11.0 Hz, 1H, H-8b), 3.93 (br s, 1H, H-6), 3.67-3.71 (m, 1H, H-5a), 3.52-3.55 (m, 1H, H-5b), 2.14-2.19 (m, 1H, H-4a), 1.98-2.03 (m, 1H, H-4b) ppm;

^{13}C NMR (176 MHz, $(CD_3)_2CO$) δ 141.8, 133.7, 129.5, 129.4, 94.63 (m), 78.6, 58.9, 56.8, 40.3 ppm;

HRMS (+ESI) *m/z* Found $[M+Na]^+$ 417.9214, $[C_{11}H_{13}Cl_4NNaO_4S]^+$ requires 417.9212, (δ = + 0.5 ppm);

$[\alpha]_D^{25.0}$ = + 20.3 (c. 1.3, MeOH);

Chiral SFC Analysis: CHIRALPAK IG (CO_2:MeOH, 85.0:15.0, 2.5 mL min^{-1}, 40 °C) t_R = 5.1 (minor), 7.3 (major) minutes.

2,2,2-trichloroethyl (R)-(3-hydroxy-1-(4-methoxyphenyl)propyl)sulfamate (**2m**)

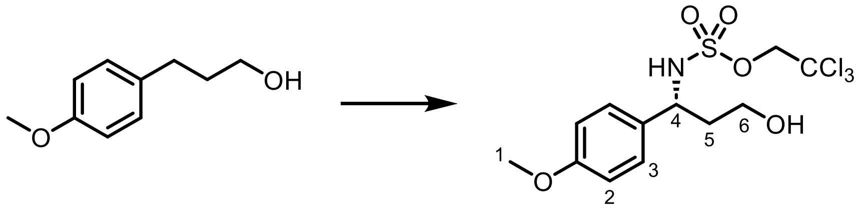

Prepared according to **GP10** using 3-(4-methoxyphenyl)propan-1-ol as the starting material. Purification by flash column chromatography (SiO_2, 0-20% v/v acetone in $CHCl_3$) afforded the title compound as a white solid (34.5 mg, 0.088 mmol, 88%, 88% *ee*).

***R_f* value**: 0.46 (20% v/v acetone in $CHCl_3$);

^{1}H NMR (400 MHz, CD_3OD): δ 7.29 (m, 2H, H-3), 6.91 (m, 2H, H-2), 4.57 (t, *J* = 7.6 Hz, 1H, H-4), 4.30 (d, *J* = 11.0 Hz, 1H, H-7A), 4.10 (d, *J* = 11.0 Hz, 1H, H-7B), 3.78 (s, 3H, H-1), 3.60 (dt, *J* = 10.9, 6.1 Hz, 1H, H-6A), 3.47 (dt, *J* = 10.9, 6.3 Hz, 1H, H-6B), 2.12 (m, 1H, H-5A), 1.92 (m, 1H, H-5B) ppm;

^{13}C NMR (101 MHz, CD_3OD): δ 160.8, 135.0, 129.2, 115.2, 94.9, 78.9, 59.4, 56.7, 55.8, 40.7 ppm;

HRMS (–ESI): *m/z* found $[M-(CH_2CCl_3)]^-$ 260.0598, $[C_{10}H_{14}NO_5S]^-$ requires 260.0598, (δ = + 0.0 ppm);

$[\alpha]_D^{25.0}$ = + 13.8 (c. 1.9, MeOH);

Chiral SFC Analysis: CHIRALPAK IG (CO_2:MeOH, 83.0:17.0, 2.50 mL min^{-1}, 40 °C) t_R = 4.8 (minor), 7.6 (major) minutes.

2,2,2-trichloroethyl (R)-(1-(4-bromophenyl)-3-hydroxypropyl)sulfamate (**2n**)

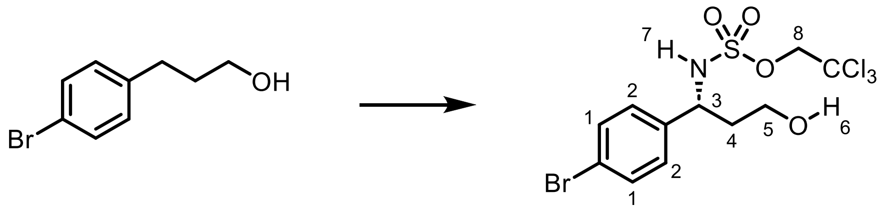

Prepared according to **GP10** using 3-(4-bromophenyl)propan-1-ol as the starting material. Purification by flash column chromatography (SiO_2, 0-24% v/v Acetone in $CHCl_3$) afforded the title compound as an amorphous white powder (45.5 mg, 0.10 mmol, Quant., 92% *ee*).

***R_f* value** = 0.41 (20% v/v Acetone in $CHCl_3$);

^{1}H NMR (400 MHz, $(CD_3)_2CO$) δ 7.75 (br s, 1H, H-7), 7.54-7.58 (m, 2H, H-1), 7.41-7.44 (m, 2H, H-2), 4.79 (t, *J* = 7.3 Hz, 1H, H-3), 4.56 (d, *J* = 11.0 Hz, 1H, H-8a), 4.46 (d, *J* = 11.0 Hz, 1H, H-8b), 3.95 (br s, 1H, H-6), 3.66-3.72 (m, 1H, H-5a), 3.51-3.56 (m, 1H, H-5b), 2.12-2.21 (m, 1H, H-4a), 1.96-2.09 (m, 1H, H-4b) ppm;

^{13}C NMR (101 MHz, $(CD_3)_2CO$) δ 142.2, 132.4, 129.9, 121.8, 94.6, 78.6, 58.9, 56.8, 40.2 ppm;

HRMS (–ESI) *m/z* Found $[M-H]^-$ 437.8741, $[C_{11}H_{12}BrCl_3NO_4S]^-$ requires 437.8741, (δ = + 0.0 ppm);

$[\alpha]_D^{25.0}$ = + 25.1 (c. 1.2, CH_3CN);

Chiral SFC Analysis: CHIRALPAK IG (CO_2:MeOH, 85.0:15.0, 2.5 mL min^{-1}, 40 °C) t_R = 5.7 (minor), 9.1 (major) minutes.

2,2,2-trichloroethyl (R)-(3-hydroxy-1-(4-(trifluoromethyl)phenyl)propyl)sulfamate (**2o**)

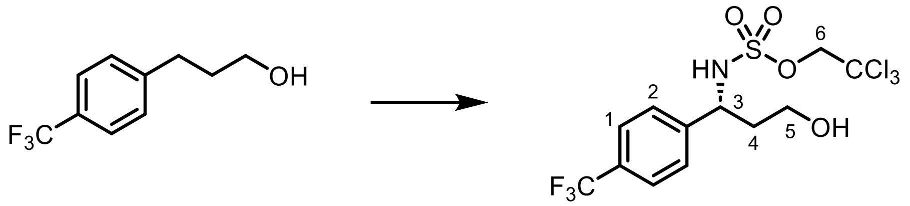

Prepared according to **GP10** using 3-(4-(trifluoromethyl)phenyl)propan-1-ol as the starting material. Purification by flash column chromatography (SiO_2, 0-20% v/v acetone in $CHCl_3$) afforded the title compound as an amorphous white solid (9.5 mg, 0.022 mmol, 22%, 85% *ee*).

***Rf* value**: 0.39 (20% v/v acetone in $CHCl_3$);

^{1}H NMR (700 MHz, CD_3OD): δ 7.67 (d, *J* = 8.1 Hz, 2H, H-1), 7.57 (d, *J* = 8.1 Hz, 2H, H-2), 4.75 (t, *J* = 7.5 Hz, 1H, H-3), 4.44 (d, *J* = 10.9 Hz, 1H, H-6_A), 4.30 (d, *J* = 10.9 Hz, 1H, H-6_B), 3.66 (m, 1H, H-5_A), 3.49 (m, 1H, H-5_B), 2.11 (m, 1H, H-4_A), 1.94 (m, 1H, H-4_B) ppm;

^{13}C NMR (176 MHz, CD_3OD): *δ* 147.8, 130.9 (q, J_{C-F} = 32.3 Hz), 128.7, 126.7 (q, J_{C-F} = 3.8 Hz), 125.6 (q, J_{C-F} = 271 Hz), 94.8, 78.9, 59.1, 56.7, 40.6 ppm;

^{19}F NMR (376 MHz, $CDCl_3$): *δ* – 64.1 ppm;

HRMS (−ESI): *m/z* found $[M-H]^-$ 427.9512, $[C_{12}H_{12}Cl_3F_3NO_4S]^-$ requires 427.9510, (*δ* = + 0.5 ppm);

$[\alpha]_D^{25.0}$ = + 23.5 (c. 0.5, MeOH);

Chiral SFC Analysis: CHIRALPAK IG (CO_2:MeOH, 93.0:7.0, 2.50 mL min^{-1}, 40 °C) t_R = 6.6 (minor), 11.3 (major) minutes.

2,2,2-trichloroethyl (S)-(3-hydroxy-2,2-dimethyl-1-phenylpropyl)sulfamate (**2o**)

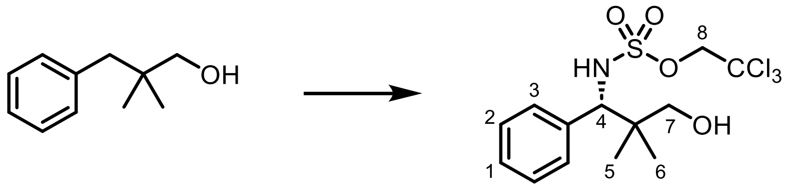

Prepared according to **GP10** using 2,2-dimethyl-3-phenylpropan-1-ol as the starting material. In this case the following procedural modification was used: The reaction was performed using 2 mol % of catalyst and the reaction temperature was – 25 °C. Purification by flash column chromatography (SiO_2, 0-6% v/v acetone in $CHCl_3$) afforded the title compound as a colorless oil (24.0 mg, 0.061 mmol, 61%, 98% *ee*).

R_f value: 0.47 (10% v/v acetone in $CHCl_3$);

^{1}H NMR (400 MHz, $CDCl_3$): δ 7.38-7.27 (m, 5H, H-1, H-2, H-3), 6.93 (br. s, 1H, N-H), 4.38 (br. s, 1H, H-4), 4.15 (d, J = 10.8 Hz, 1H, H-8$_A$), 4.12 (d, J = 10.8 Hz, 1H, H-8$_B$), 3.67 (d, J = 10.8 Hz, 1H, H-7$_A$), 3.32 (d, J = 10.8 Hz, 1H, H-7$_B$), 2.08 (br. s, 1H, O-H), 1.26 (s, 3H, H-5), 0.74 (s, 3H, H-6) ppm;

^{13}C NMR (101 MHz, $CDCl_3$): δ 138.4, 128.5, 128.4, 128.1, 93.4, 78.0, 69.4, 67.2, 38.8, 23.9, 22.7 ppm;

HRMS (−ESI): *m/z* found $[M-(CH_2CCl_3)]^-$ 258.0806, $[C_{11}H_{16}NO_4S]^-$ requires 258.0806, (δ = + 0.0 ppm);

$[\alpha]_D^{25.0}$ = + 22.8 (c. 1.3, $CHCl_3$);

Chiral SFC Analysis: CHIRALPAK IG (CO_2:MeOH, 90.0:10.0, 2.50 mL min^{-1}, 40 °C) t_R = 6.6 (minor), 8.8 (major) minutes.

2,2,2-trichloroethyl (S)-((1-(hydroxymethyl)cyclopentyl)(phenyl)methyl)sulfamate (**2q**)

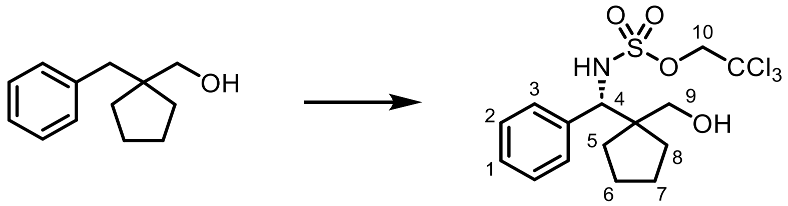

Prepared according to **GP10** using (1-benzylcyclopentyl)methanol as the starting material. In this case the following procedural modification was used: The reaction was performed using 2 mol % of catalyst and the reaction temperature was – 25 °C. Purification by flash column chromatography (SiO_2, 0-1.5% v/v Et_2O in CH_2Cl_2) afforded the title compound as a colorless oil (23.2 mg, 0.056 mmol, 56%, 97% *ee*).

***R$_f$* value**: 0.45 (2% v/v Et_2O in CH_2Cl_2);

^{1}H NMR (400 MHz, $CDCl_3$): δ 7.41-7.32 (m, 4H, H-2, H-3), 7.30 (m, 1H, H-1), 7.15 (d, J = 8.1 Hz, 1H, N-H), 4.40 (d, J = 7.9 Hz, 1H, H-4), 4.16 (d, J = 10.6 Hz, 1H, H-10$_A$), 4.09 (d, J = 10.6 Hz, 1H, H-10$_B$), 3.68 (d, J = 10.9 Hz, 1H, H-9$_A$), 3.37 (d, J = 10.8 Hz, 1H, H-9$_B$), 2.06 (m, 1H, H-5$_A$), 1.98-1.89 (m, 2H, H-5$_B$, O-H), 1.83 (m, 1H, H-6$_A$), 1.74-1.47 (m, 4H, H-6$_B$, H-7, H-8$_A$), 0.80 (m, 1H, H-8$_B$) ppm;

^{13}C NMR (101 MHz, $CDCl_3$): δ 139.1, 128.5, 128.4, 128.0, 93.5, 77.9, 67.2, 66.9, 50.9, 33.9, 33.5, 24.7, 24.6 ppm;

HRMS (−ESI): *m/z* found $[M-(CH_2CCl_3)]^-$ 284.0965, $[C_{13}H_{18}NO_4S]^-$ requires 284.0962, (δ = + 1.1 ppm);

$[\alpha]_D^{25.0}$ = + 14.1 (c. 0.4, $CHCl_3$);

Chiral SFC Analysis: CHIRALPAK IG (CO_2:MeOH, 85.0:15.0, 1.25 mL min^{-1}, 40 °C) t_R = 12.8 (minor), 13.9 (major) minutes.

2,2,2-trichloroethyl (R)-(3-hydroxy-3-methyl-1-phenylbutyl)sulfamate (**2r**)

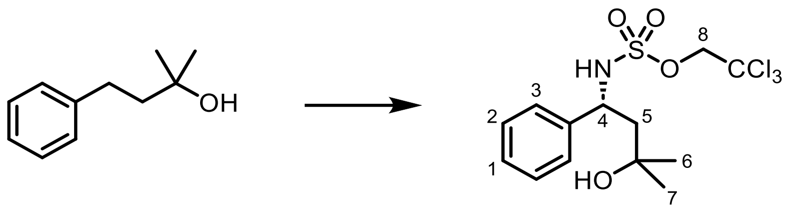

Prepared according to **GP10** using 2-methyl-4-phenylbutan-2-ol as the starting material. Purification by flash column chromatography (SiO_2, 0-9% v/v acetone in $CHCl_3$) afforded the title compound as a colorless oil (37.2 mg, 0.095 mmol, 95%, 85% *ee*).

***R_f* value**: 0.60 (10% v/v acetone in $CHCl_3$);

^{1}H NMR (700 MHz, $CDCl_3$): δ 7.39-7.34 (m, 4H, H-2, H-3), 7.28 (tt, *J* = 7.2, 1.5 Hz, 1H, H-1), 4.78 (dd, *J* = 11.5, 3.3 Hz, 1H, H-4), 4.33 (d, *J* = 10.8 Hz, 1H, H-8_A), 4.28 (d, *J* = 10.8 Hz, 1H, H-8_B), 2.07 (dd, *J* = 15.1, 11.5 Hz, 1H, H-5_A), 1.83 (br. s, 1H, O-H), 1.72 (dd, *J* = 15.1, 3.4 Hz, 1H, H-5_B), 1.49 (s, 3H, H-6), 1.30 (s, 3H, H-7) ppm;

^{13}C NMR (176 MHz, $CDCl_3$): δ 141.6, 128.9, 128.2, 126.9, 93.6 (d, *J* = 0.8 Hz), 77.9, 72.5, 57.4, 48.3, 32.8, 27.4 ppm;

HRMS (−ESI): *m/z* found $[M-H]^-$ 387.9947, $[C_{13}H_{17}Cl_3NO_4S]^-$ requires 387.9949, (*δ* = − 0.5 ppm);

$[\alpha]_D^{25.0}$ = + 34.1 (c. 1.6, $CHCl_3$);

Chiral SFC Analysis: CHIRALPAK IG (CO_2:MeOH, 90.0:10.0, 2.50 mL min^{-1}, 40 °C) t_R = 5.8 (minor), 6.5 (major) minutes.

2,2,2-trichloroethyl (R)-(2-(1-hydroxycyclobutyl)-1-phenylethyl)sulfamate (**2s**)

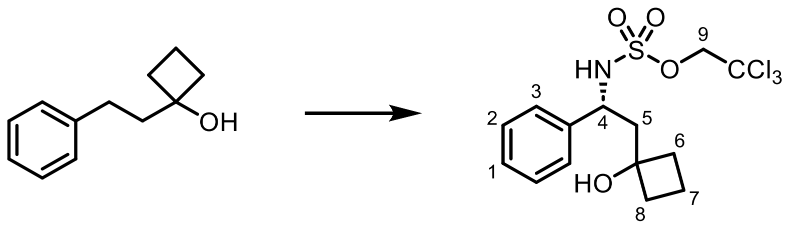

Prepared according to **GP10** using 1-phenethylcyclobutan-1-ol as the starting material. In this case the following procedural modification was used: 0.124 mmol of starting material was used and all the other reagents were scaled accordingly. Purification by flash column chromatography (SiO_2, 0-6% v/v acetone in $CHCl_3$) afforded the title compound as a white solid (42.6 mg, 0.106 mmol, 85%, 83% *ee*).

***R_f* value**: 0.47 (5% acetone in $CHCl_3$);

^{1}H NMR (400 MHz, $CDCl_3$): δ 7.42-7.33 (m, 4H, H-2, H-3), 7.29 (tt, *J* = 7.0, 1.6 Hz, 1H, H-1), 6.95 (br. s, 1H, N-H), 4.77 (m, 1H, H-4), 4.34 (d, *J* = 10.8 Hz, 1H, H-9_A), 4.30 (d, *J* = 10.8 Hz, 1H, H-9_B), 2.40-2.30 (m, 2H, H-6_A, O-H), 2.20-1.94 (m, 5H, H-5, H-6_B, H-8), 1.79 (m, 1H, H-7_A), 1.52 (m, 1H, H-7_B) ppm;

^{13}C NMR (101 MHz, $CDCl_3$): δ 141.3, 128.9, 128.2, 126.9, 93.5, 78.0, 76.2, 57.1, 44.6, 38.7, 35.8, 12.2 ppm;

HRMS (−ESI): *m/z* found $[M-H]^-$ 399.9952, $[C_{14}H_{17}Cl_3NO_4S]^-$ requires 399.9949, (*δ* = + 0.8 ppm).

2s was also prepared according to **GP10** with the following procedural modification: Rh_2(**A-III**)$_2$•(**Cat8**)$_2$•(**pyr**)$_2$ was used as the chiral catalyst. Purification by flash column chromatography (SiO_2, 0-6% v/v acetone in $CHCl_3$) afforded the title compound as a white solid (28.9 mg, 0.072 mmol, 72%, 93% *ee*).

$[\alpha]_D^{25.0}$ = + 26.7 (c. 1.8, $CHCl_3$);

Chiral SFC Analysis: CHIRALPAK IC (CO_2:MeOH, 90.0:10.0, 2.50 mL min^{-1}, 40 °C) t_R = 4.4 (minor), 4.8 (major) minutes.

(R)-3-amino-3-phenylpropan-1-ol (**3**)

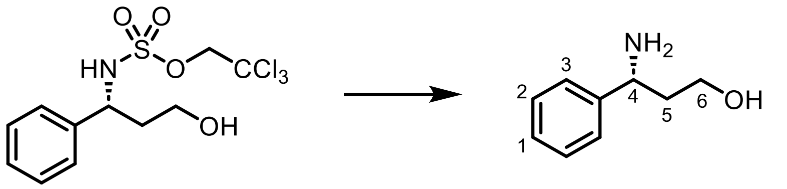

Prepared according to the protocol described by Guthikonda and Du Bois.[48] To a vial charged with 2,2,2-trichloroethyl (*R*)-(4-hydroxy-1-phenylbutyl)sulfamate (63.5 mg, 0.175 mmol) and Zn/Cu couple (57.1 mg, 0.875 mmol) under a nitrogen atmosphere was added MeOH (1.5 mL) and AcOH (glacial, 1.5 mL). After stirring at room temperature overnight, the suspension was filtered over Celite® and washed with MeOH. The filtrate was collected, concentrated under reduced pressure and further dried with a benzene azeotrope. The crude material was transferred to a microwave vial and placed under a nitrogen atmosphere. MeOH (2.0 mL) and acetyl chloride (94 µL, 1.32 mmol) were added and the resulting mixture was heated at 45 °C overnight. The reaction mixture was concentrated under a stream of nitrogen and diluted in EtOAc. The organic layer was extracted with 1 M aqueous HCl solution (× 3) and the combined aqueous layers were basified. The aqueous layer was back-extracted with EtOAc (× 3), the combined organic layers were dried over $MgSO_4$ and concentrated under reduced pressure to afford the title compound as a colourless oil (19.6 mg, 0.13 mmol, 74%).

^{1}H NMR (700 MHz, $CDCl_3$): *δ* 7.35 (t, *J* = 7.8 Hz, 2H, H-2), 7.29 (d, *J* = 7.3 Hz, 2H, H-3), 7.26 (t, *J* = 7.6 Hz, 1H, H-1), 4.13 (m, 1H, H-4), 3.82 (m, 2H, H-6), 2.52 (br. s, 3H, O-H, N-H), 1.90 (m, 2H, H-5) ppm;

^{13}C NMR (176 MHz, $CDCl_3$): *δ* 146.2, 128.9, 127.3, 125.8, 62.4, 56.7, 39.6 ppm;

$[\alpha]_D^{25.0}$ = + 26.5 (c. 0.8, $CHCl_3$);

The absolute stereochemistry of **3**, *and all other C-H amination products by analogy, was assigned based on comparison of optical rotation data to the literature. The spectroscopic and optical rotation data of* **3** *was in agreement with the previous literature report ($[\alpha]_D^{20.0}$ = + 21.9 (c. 1.0, $CHCl_3$) for the same enantiomer).*[49] *Alternatively, $[\alpha]_D^{28.0}$ = – 20.4 (c. 0.44, $CHCl_3$) has been reported for the opposite enantiomer.*[50]

(R)-3-hydroxy-1-phenylpropan-1-aminium chloride (**3•HCl**)

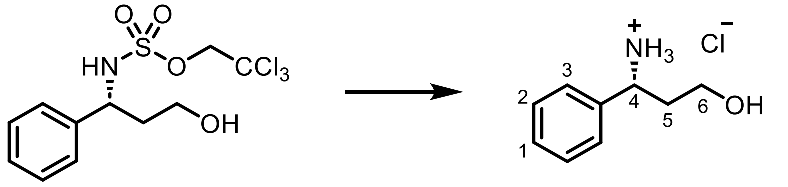

To enable another comparison point of optical rotation data, the free amine **3** can be converted to the HCl salt by the following procedure: The free amine was dissolved in 2 M HCl in Et_2O and MeOH and then concentrated under a stream of nitrogen over 1 hour. The crude was washed with 1:1 hexane/Et_2O then THF and dried to give the title compound as a white solid.

^{1}H NMR (400 MHz, CD_3OD): δ 7.50-7.40 (m, 5H, H-1, H-2, H-3), 4.47 (dd, *J* = 7.5, 7.0 Hz, 1H, H-4), 3.65 (m, 1H, H-6_A), 3.50 (m, 1H, H-6_B), 2.23 (m, 1H, H-5_A), 2.09 (m, 1H, H-5_B) ppm;

^{13}C NMR (176 MHz, CD_3OD): δ 138.1, 130.37, 130.36, 128.3, 59.2, 55.0, 37.6 ppm;

$[\alpha]_D^{25.0}$ = – 27.3 (c. 0.3, MeOH);

Note: the same enantiomers, **3** *and* **3•HCl**, *give opposite sign for optical rotation depending on whether the free amine in $CHCl_3$ or the HCl salt in MeOH is used for measurement.*

The antipode **2b**-**ent** was also taken through the deprotection and salt formation sequence to afford (*S*)-3-hydroxy-1-phenylpropan-1-aminium chloride (**3•HCl**-ent). In this case, $[\alpha]_D^{25.0}$ = + 25.3 (c. 0.3, MeOH).

The optical rotation data of **3•HCl**-ent *was in agreement with the previous literature report ($[\alpha]_D^{23.0}$ = + 23.8 (c. 1.0, MeOH) for the same enantiomer).*[51]

2,2,2-trichloroethyl (R)-(3-hydroxy-1-(thiophen-2-yl)propyl)sulfamate (**2t**)

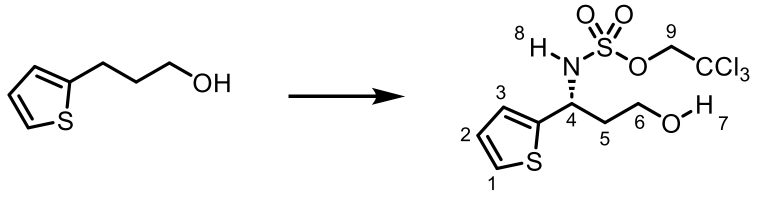

Prepared according to **GP10** using 3-(thiophen-2-yl)propan-1-ol as the starting material. In this case the following procedural modification was used: The reaction time was 48 hours. Purification by flash column chromatography (SiO_2, 0-25% v/v Acetone in $CHCl_3$) afforded the title compound as a white amorphous solid (34.4 mg, 0.093 mmol, 93%, 88% *ee*).

***Rf* value** = 0.51 (20% v/v Acetone in $CHCl_3$);

^{1}H NMR (400 MHz, $(CD_3)_2CO$) δ 7.40 (dd, *J* = 5.1 Hz, 0.8 Hz, 1H, H-1), 7.15 (d, *J* = 3.3 Hz, 1H, H-3), 7.00 (dd, *J* = 5.0 Hz, 3.6 Hz, 1H, H-2), 5.09 (t, *J* = 7.2 Hz, 1H, H-4), 4.55 (d, *J* = 11.0 Hz, 1H, H-9a), 4.44 (d, *J* = 11.0 Hz, 1H, H-9b), 3.71-3.77 (m, 1H, H-6a), 3.57-3.62 (m, 1H, H-6b), 2.20-2.28 (m, 1H, H-5a), 2.08-2.16 (m, 1H, H-5b) ppm;

^{13}C NMR (101 MHz, $(CD_3)_2CO$) δ 145.9, 127.7, 126.4, 125.8, 94.7, 78.7, 58.8, 52.7, 41.0 ppm;

HRMS (−ESI) *m/z* Found $[M-H]^-$ 365.9209, $[C_9H_{11}Cl_3NO_4S_2]^-$ requires 365.9201, (δ = − 2.2 ppm);

$[\alpha]_D^{25.0}$ = + 16.1 (c. 1.1, MeOH);

Chiral SFC Analysis: CHIRALPAK IG (CO_2: MeOH, 85:15, 2.5 mL min^{-1}, 40 °C) t_R = 6.1 (minor), 9.6 (major) minutes.

2,2,2-trichloroethyl (R)-(1-(benzo[b]thiophen-5-yl)-3-hydroxypropyl)sulfamate (**2u**)

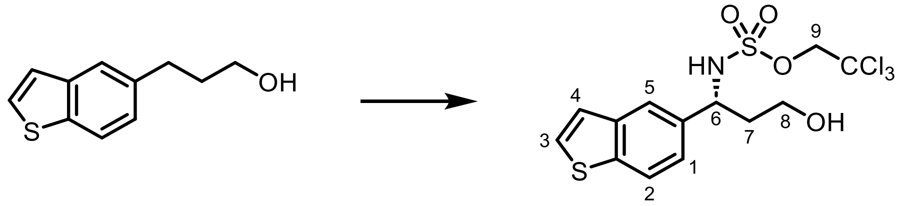

Prepared according to **GP10** using 3-(benzo[b]thiophen-5-yl)propan-1-ol as the starting material. Purification by flash column chromatography (SiO_2, 0-30% v/v acetone in $CHCl_3$) afforded the title compound as a pale yellow solid (20.5 mg, 0.049 mmol, 49%, 86% *ee*).

R_f value: 0.50 (20% v/v acetone in $CHCl_3$);

^{1}H NMR (700 MHz, d_6-DMSO): δ 9.89 (br. s, 1H, O-H/N-H), 7.99 (d, *J* = 8.4 Hz, 1H, H-2), 7.83 (d, *J* = 1.1 Hz, 1H, H-5), 7.76 (d, *J* = 5.4 Hz, 1H, H-3), 7.45 (d, *J* = 5.4 Hz, 1H, H-4), 7.37 (dd, *J* = 8.4, 1.5 Hz, 1H, H-1), 4.65 (t, *J* = 7.4 Hz, 1H, H-6), 4.60 (br. s, 1H, O-H/N-H), 4.43 (d, *J* = 11.1 Hz, 1H, H-9_A), 4.27 (d, *J* = 11.1 Hz, 1H, H-9_B), 3.43 (m, 1H, H-8_A), 3.28 (m, 1H, H-8_B), 2.07 (m, 1H, H-7_A), 1.86 (sext, *J* = 6.2 Hz, 1H, H-7_B) ppm;

^{13}C NMR (176 MHz, d_6-DMSO): δ 139.5, 138.3, 138.2, 128.0, 123.9, 123.0, 122.7, 121.9, 93.6 (d, *J* = 0.7 Hz), 77.0, 57.2, 55.5 ppm;

Note: there is an additional ^{13}C resonance underneath the solvent peak.

HRMS (−ESI) *m/z* Found $[M-H]^-$ 415.9355, $[C_{13}H_{13}Cl_3NO_4S_2]^-$ requires 415.9357, (δ = − 0.5 ppm);

$[\alpha]_D^{25.0}$ = + 10.7 (c. 0.2, MeOH);

Chiral SFC Analysis: CHIRALPAK IG (CO_2:MeOH, 80.0:20.0, 2.50 mL min^{-1}, 40 °C) t_R = 5.6 (minor), 7.5 (major) minutes.

2,2,2-trichloroethyl (R)-(1-(benzo[b]thiophen-2-yl)-3-hydroxypropyl)sulfamate (**2v**)

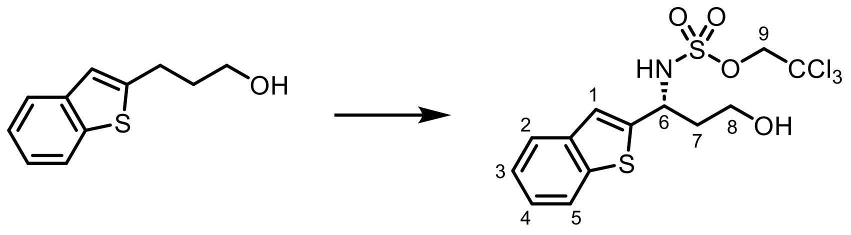

Prepared according to **GP10** using 3-(benzo[b]thiophen-2-yl)propan-1-ol as the starting material. Purification by flash column chromatography (SiO_2, 0-30% v/v acetone in $CHCl_3$) afforded the title compound as a white solid (23.0 mg, 0.055 mmol, 55%, 88% *ee*).

***R$_f$* value**: 0.53 (20% v/v acetone in $CHCl_3$);

^{1}H NMR (700 MHz, CD_3OD): δ 7.81 (d, *J* = 7.8 Hz, 1H, H-5), 7.73 (d, *J* = 7.7 Hz, 1H, H-2), 7.35-7.29 (m, 3H, H-1, H-3, H-4), 5.05 (t, *J* = 7.4 Hz, 1H, H-6), 4.46 (d, *J* = 10.9 Hz, 1H, H-9_A), 4.34 (d, *J* = 10.9 Hz, 1H, H-9_B), 3.71 (m, 1H, H-8_A), 3.58 (m, 1H, H-8_B), 2.21 (sext, *J* = 6.8 Hz, 1H, H-7_A), 2.11 (sext, *J* = 6.9 Hz, 1H, H-7_B), ppm;

^{13}C NMR (176 MHz, CD_3OD): δ 146.8, 140.9, 140.8, 125.59, 125.55, 124.7, 123.9, 123.4, 94.8, 79.1, 59.1, 53.3, 40.8 ppm;

HRMS (+ESI) *m/z* Found [M+H]$^-$ 417.9502, [$C_{13}H_{15}Cl_3NO_4S_2$]$^+$ requires 417.9503, (δ = – 0.2 ppm);

$[\alpha]_D^{25.0}$ = + 24.8 (c. 1.2, MeOH);

Chiral SFC Analysis: CHIRALPAK IG (CO_2:MeOH, 80.0:20.0, 2.50 mL min^{-1}, 40 °C) t_R = 6.7 (minor), 11.8 (major) minutes.

2,2,2-trichloroethyl (R)-(3-hydroxy-1-(1-(phenylsulfonyl)-1H-pyrrol-3-yl)propyl)sulfamate (**2w**)

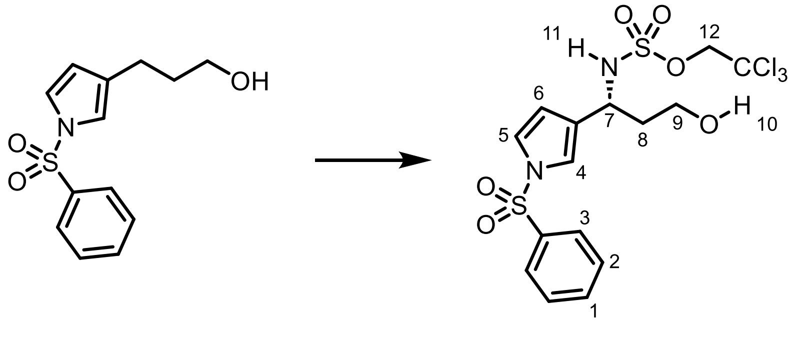

Prepared according to **GP10** using 3-(1-(phenylsulfonyl)-1*H*-pyrrol-3-yl)propan-1-ol as the starting material. In this case the following procedural modification was used: The reaction time was 48 hours. Purification by flash

column chromatography (SiO_2, 0-20% v/v Et_2O in CH_2Cl_2) afforded the title compound as a colourless oil (12.6 mg, 0.026 mmol, 26%, 89% *ee*).

***R_f* value** = 0.42 (20% v/v Et_2O in CH_2Cl_2);

^{1}H NMR (400 MHz, $CDCl_3$) δ 7.86 (d, *J* = 7.5 Hz, 2H, H-3), 7.62 (t, *J* = 7.4 Hz, 1H, H-1), 7.52 (t, *J* = 7.7 Hz, 2H, H-2), 7.19 (br s, 1H, H-4), 7.15 (t, *J* = 2.7 Hz, 1H, H-5), 6.30 (dd, *J* = 3.1 Hz, 1.6 Hz, 1H, H-6), 5.94 (d, *J* = 7.4 Hz, 1H, H-11), 4.76 (td, *J* = 7.6 Hz, 4.5 Hz, 1H, H-7), 4.52 (d, *J* = 10.8 Hz, 1H, H-12a), 4.47 (d, *J* = 10.8 Hz, 1H, H-12b), 3.72-3.85 (m, 2H, H-9a, H-9b), 2.12-2.20 (m, 1H, H-8a), 1.87-1.96 (m, 1H, H-8b) ppm;

^{13}C NMR (176 MHz, $CDCl_3$) δ 138.8, 134.3, 129.7, 128.6, 127.0, 121.9, 118.3, 112.3, 93.5, 78.1, 59.6, 51.3, 37.0 ppm;

HRMS (−ESI) *m/z* Found $[M-H]^-$ 488.9528, $[C_{15}H_{16}Cl_3N_2O_6S_2]^-$ requires 488.9521, (δ = + 1.4 ppm);

$[\alpha]_D^{25.0}$ = + 17.8 (c. 1.1, $CHCl_3$);

Chiral SFC Analysis: CHIRALPAK IG (CO_2: MeOH, 80:20, 2.5 mL min^{-1}, 40 °C) t_R = 5.6 (minor), 8.2 (major) minutes.

2,2,2-trichloroethyl (6-(phenylsulfonyl)-2,3,4,6,7,7a-hexahydropyrano[2,3-c]pyrrol-7-yl)sulfamate (**4a**)

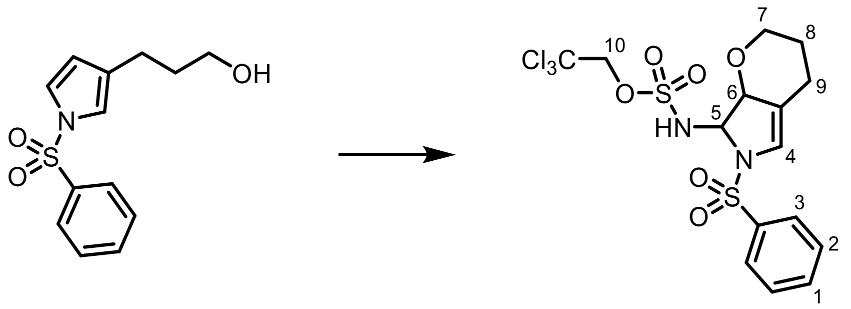

Prepared according to **GP10** using 3-(1-(phenylsulfonyl)-1*H*-pyrrol-3-yl)propan-1-ol as the starting material. In this case the following procedural modification was used: $Rh_2(esp)_2$ (2 mol %) was used instead of the chiral catalyst. Purification by flash column chromatography (SiO_2, 0-10% v/v Et_2O in CH_2Cl_2) afforded the title compound as a colourless oil (11.7 mg, 0.024 mmol, 24%).

***R_f* value**: 0.61 (10% v/v Et_2O in CH_2Cl_2);

^{1}H NMR (400 MHz, $CDCl_3$): δ 7.99 (d, *J* = 7.8 Hz, 2H, H-3), 7.62 (t, *J* = 7.2 Hz, 1H, H-1), 7.54 (t, *J* = 7.8 Hz, 2H, H-2), 5.86 (dt, *J* = 8.2, 1.9 Hz, 1H, H-5), 5.66 (t, *J* = 1.9 Hz, 1H, H-4), 5.36 (d, *J* = 8.4 Hz, 1H, N-H), 5.33 (s, 1H, H-6), 4.72 (d, *J* = 11.0 Hz, 1H, H-10_A), 4.68 (d, *J* = 11.0 Hz, 1H, H-10_B), 4.06 (m, 1H, H-7_A), 3.71 (td, *J* = 12.1, 3.3 Hz, 1H, H-7_B), 2.62 (m, 1H, H-9_A), 2.27 (m, 1H, H-9_B), 1.76 (m, 2H, H-8) ppm;

Note – there are traces of minor diastereomer visible in the ^{1}H spectrum but these peaks have not been assigned

^{13}C NMR (101 MHz, $CDCl_3$): δ 142.3, 139.4, 133.6, 129.5, 127.6, 119.4, 93.6, 91.2, 78.8, 74.6, 66.1, 27.3, 26.2 ppm;

HRMS (–ESI) *m/z* Found $[M-H]^-$ 488.9529, $[C_{15}H_{16}Cl_3N_2O_6S_2]^-$ requires 488.9521, (δ = + 1.6 ppm).

2,2,2-trichloroethyl (R)-(1-(benzofuran-2-yl)-3-hydroxypropyl)sulfamate (**2x**)

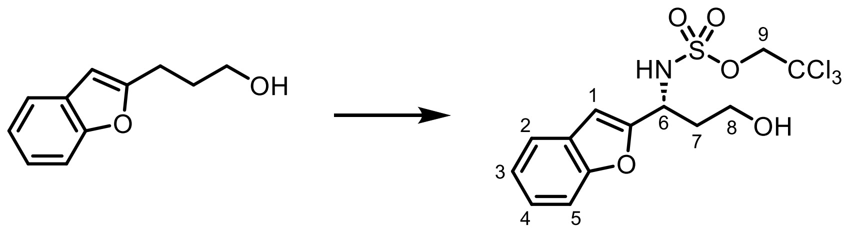

Prepared according to **GP10** using 3-(benzofuran-2-yl)propan-1-ol as the starting material. Purification by flash column chromatography (SiO_2, 0-20% v/v acetone in $CHCl_3$) afforded the title compound as a white solid (26.0 mg, 0.065 mmol, 65%, 87% *ee*).

***R_f* value**: 0.47 (20% v/v acetone in $CHCl_3$);

^{1}H NMR (700 MHz, CD_3OD): δ 7.54 (d, *J* = 7.7 Hz, 1H, H-5), 7.44 (d, *J* = 8.2 Hz, 1H, H-2), 7.27 (td, *J* = 7.3, 1.0 Hz, 1H, H-3), 7.20 (td, *J* = 7.3, 0.9 Hz, 1H, H-4), 6.78 (s, 1H, H-1), 4.89 (t, *J* = 7.5 Hz, 1H, H-6), 4.52 (d, *J* = 10.9 Hz, 1H, H-9_A), 4.37 (d, *J* = 10.9 Hz, 1H, H-9_B), 3.71 (m, 1H, H-8_A), 3.58 (m, 1H, H-8_B), 2.19 (m, 2H, H-7) ppm;

^{13}C NMR (176 MHz, CD_3OD): δ 157.2, 156.3, 129.5, 125.5, 124.1, 122.2, 112.0, 105.6, 94.8, 79.1, 59.0, 50.9, 37.7 ppm;

HRMS (–ESI) *m/z* Found $[M-(CH_2CCl_3)]^-$ 270.0439, $[C_{11}H_{12}NO_5S]^-$ requires 270.0442, (δ = – 1.1 ppm);

$[\alpha]_D^{25.0}$ = + 47.2 (c. 0.4, $CHCl_3$);

Chiral SFC Analysis: CHIRALPAK IG (CO_2:MeOH, 85.0:15.0, 2.50 mL min^{-1}, 40 °C) t_R = 5.7 (minor), 7.9 (major) minutes.

2,2,2-trichloroethyl (4',5'-dihydro-3H,3'H-spiro[benzofuran-2,2'-furan]-3-yl)sulfamate (**4b**)

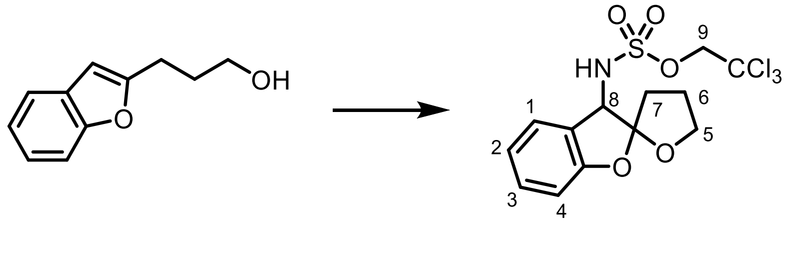

Prepared according to **GP10** using 3-(benzofuran-2-yl)propan-1-ol as the starting material. In this case the following procedural modification was used: $Rh_2(esp)_2$ (2 mol %) was used instead of the chiral catalyst. Purification by flash column chromatography (SiO_2, 0-10% v/v acetone in $CHCl_3$) afforded the title compound as a colourless oil (17.7 mg, 0.044 mmol, 44%, 6:1 mixture of diastereomers).

***R_f* value**: 0.31 (2% v/v acetone in $CHCl_3$);

HRMS (–ESI) *m/z* Found $[M-H]^-$ 399.9587, $[C_{13}H_{13}Cl_3NO_5S]^-$ requires 399.9586, (δ = + 0.3 ppm);

For major diastereomer:

^{1}H NMR (700 MHz, $CDCl_3$): δ 7.49 (d, *J* = 7.5 Hz, 1H, H-1), 7.29 (t, *J* = 7.8 Hz, 1H, H-3), 6.98 (t, *J* = 7.4 Hz, 1H, H-2), 6.84 (d, *J* = 8.1 Hz, 1H, H-4), 5.02 (d, *J* = 9.1 Hz, 1H, H-8), 4.89 (d, *J* = 9.1 Hz, 1H, N-H), 4.75 (d, *J* = 10.8 Hz, 1H, H-9_A), 4.70 (d, *J* = 10.7 Hz, 1H, H-9_B), 4.17 (m, 1H, H-5_A), 4.10 (m, 1H, H-5_B), 2.52 (m, 1H, H-7_A), 2.31-2.22 (m, 2H, H-7_B, H-6_A), 2.14 (m, 1H, H-6_B) ppm;

^{13}C NMR (176 MHz, $CDCl_3$): δ 158.3, 131.5, 125.9, 124.6, 122.0, 119.7, 111.0, 93.5 (d, *J* = 0.8 Hz), 78.3, 69.8, 62.0, 32.1, 23.9 ppm;

For minor diastereomer:

^{1}H NMR (700 MHz, $CDCl_3$): δ 7.43 (d, *J* = 7.2 Hz, 1H, H-1), 7.24 (t, *J* = 8.1 Hz, 1H, H-3), 6.99-6.95 (m, 1H, H-2, *underneath major diast.*), 6.79 (d, *J* = 8.1 Hz, 1H, H-4), 5.56 (d, *J* = 9.3 Hz, 1H, N-H), 5.24 (d, *J* = 9.3 Hz, 1H, H-8), 4.73 (m, 2H, H-9), 4.21 (m, 1H, H-5_A), 4.12-4.05 (m, 1H, H-5_B, *underneath major diast.*), 2.47 (m, 1H, H-7_A), 2.39 (m, 1H, H-7_B), 2.31-2.22 (m, 1H, H-6_A, *underneath major diast.*), 2.17-2.10 (m, 1H, H-6_B, *underneath major diast.*) ppm;

^{13}C NMR (176 MHz, $CDCl_3$): δ 156.9, 130.6, 125.1, 125.0, 121.9, 115.7, 110.5, 93.5 (d, *J* = 0.8 Hz), 78.5, 70.2, 60.1, 35.0, 24.3 ppm.

methyl (R)-4-(3-hydroxy-1-(((2,2,2-trichloroethoxy)sulfonyl)amino)propyl)furan-2-carboxylate (**2y**)

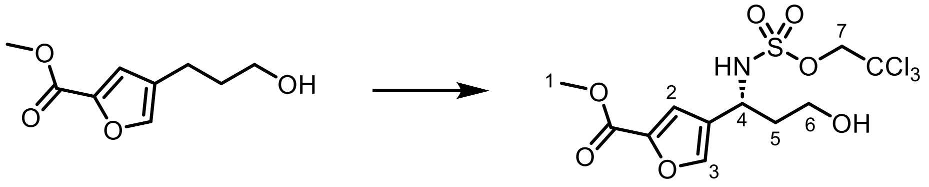

Prepared according to **GP10** using methyl 4-(3-hydroxypropyl)furan-2-carboxylate as the starting material. Purification by flash column chromatography (SiO_2, 0-20% v/v acetone in $CHCl_3$) afforded the title compound as a colourless oil (13.3 mg, 0.032 mmol, 32%, 92% *ee*).

***R_f* value**: 0.31 (20% v/v acetone in $CHCl_3$);

¹H NMR (700 MHz, $CDCl_3$): δ 7.61 (s, 1H, H-3), 7.21 (s, 1H, H-2), 6.32 (d, *J* = 7.2 Hz, 1H, N-H), 4.86 (m, 1H, H-4), 4.60 (d, *J* = 10.8 Hz, 1H, H-7_A), 4.56 (d, *J* = 10.7 Hz, 1H, H-7_B), 3.89 (s, 3H, H-1), 3.87 (m, 2H, H-6), 2.22 (m, 1H, H-5_A), 2.03 (br. s, 1H, O-H), 1.97 (m, 1H, H-5_B) ppm;

¹³C NMR (176 MHz, $CDCl_3$): δ 159.1, 145.5, 143.7, 127.5, 116.9, 93.5 (d, *J* = 0.8 Hz), 78.2, 59.6, 52.3, 50.0, 36.5 ppm;

HRMS (−ESI) *m/z* Found $[M-H]^-$ 407.9477, $[C_{11}H_{13}Cl_3NO_7S]^-$ requires 407.9484, (*δ* = − 1.7 ppm);

$[\alpha]_D^{25.0}$ = + 28.7 (c. 0.7, $CHCl_3$);

Chiral SFC Analysis: CHIRALPAK IG (CO_2:MeOH, 75.0:25.0, 2.50 mL min^{-1}, 40 °C) t_R = 2.8 (minor), 5.1 (major) minutes.

methyl 3a-(((2,2,2-trichloroethoxy)sulfonyl)amino)-3a,5,6,7a-tetrahydro-4H-furo[2,3-b]pyran-2-carboxylate (**4c**)

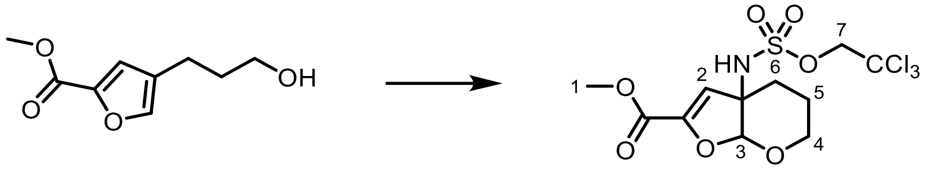

Prepared according to **GP10** using methyl 4-(3-hydroxypropyl)furan-2-carboxylate as the starting material. In this case the following procedural modification was used: $Rh_2(esp)_2$ (2 mol %) was used instead of the chiral catalyst. Purification by flash column chromatography (SiO_2, 0-10% v/v acetone in $CHCl_3$) afforded the title compound as a colourless oil (13.3 mg, 0.032 mmol, 32%, 7.5:1 mixture of diastereomers).

***R_f* value**: 0.73 (10% v/v acetone in $CHCl_3$);

HRMS (−ESI) *m/z* Found $[M-H]^-$ 407.9484, $[C_{11}H_{13}Cl_3NO_7S]^-$ requires 407.9484, (*δ* = + 0.0 ppm);

For major diastereomer:

¹H NMR (400 MHz, $CDCl_3$): δ 5.41 (d, *J* = 2.1 Hz, 1H, H-2), 5.38 (s, 1H, H-3), 4.84 (s, 1H, N-H), 4.73 (s, 2H, H-7), 4.08 (m, 1H, H-4_A), 3.86 (s, 3H, H-1), 3.71 (m, 1H, H-4_B), 2.65 (m, 1H, H-6_A), 2.36 (m, 1H, H-6_B), 1.85 (m, 2H, H-5) ppm;

¹³C NMR (101 MHz, $CDCl_3$): δ 170.9, 143.4, 121.1, 93.9, 93.5, 91.1, 79.0, 65.8, 54.6, 27.1, 26.0 ppm;

For minor diastereomer:

¹H NMR (400 MHz, $CDCl_3$): δ 5.53 (s, 1H, H-3), 5.49 (d, *J* = 2.1 Hz, H-2), 4.87 (s, 1H, N-H), 4.77 (d, *J* = 11.1 Hz, 1H, H-7_A), 4.66 (d, *J* = 11.1 Hz, 1H, H-7_B), 4.11-4.04 (m, 1H, H-4_A, *underneath major diast.*), 3.75-3.63 (m, 1H, H-4_B, *underneath major diast.*), 2.70-2.61 (m, 1H, H-6_A, *underneath major diast.*), 2.44-2.31 (m, 1H, H-6_B, *underneath major diast.*), 1.91-1.77 (m, 2H, H-5, *underneath major diast.*) ppm;

Note – [13]C resonances attributed to the minor diastereomer were not detected

2,2,2-trichloroethyl ((1R,3R)-3-hydroxy-1,5-diphenylpentyl)sulfamate (**2z**)

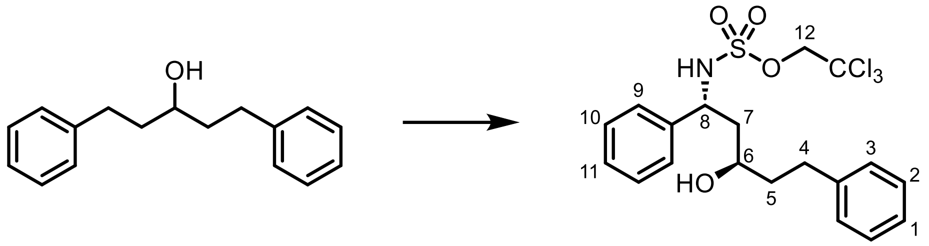

Prepared according to **GP10** using 1,5-diphenylpentan-3-ol as the starting material. Purification by flash column chromatography (SiO_2, 0-12% v/v acetone in $CHCl_3$) afforded the title compound as a white solid (single diastereomer, 37.6 mg, 0.081 mmol, 81%, 90% *ee*). *A >20:1 d.r. for this reaction was determined through analysis of the crude ^{1}H NMR before purification. The relative stereochemistry in* ***2z*** *was assigned from the relative stereochemistry in azetidine* ***5d****.*

***Rf* value**: 0.55 (10% v/v acetone in $CHCl_3$);

^{1}H NMR (700 MHz, d_6-acetone): δ 7.77 (br. s, 1H), 7.49 (d, *J* = 7.3 Hz, 2H, H-9), 7.38 (t, *J* = 7.6 Hz, 2H, H-10), 7.31 (tt, *J* = 7.4, 1.1 Hz, 1H, H-11), 7.23 (t, *J* = 7.4 Hz, 2H, H-2), 7.16-7.12 (m, 3H, H-1, H-3), 7.84 (m, 1H, H-8), 4.43 (d, *J* = 11.1 Hz, 1H, H-12_A), 4.30 (d, *J* = 11.1 Hz, 1H, H-12_B), 4.03 (br. s, 1H), 3.46-3.40 (m, 1H, H-6), 2.75-2.69 (m, 1H, H-4_A), 2.62-2.56 (m, 1H, H-4_B), 2.14 (m, 1H H-7_A), 2.05 (m, 1H, H-7_B), 1.76 (m, 2H, H-5) ppm;

^{13}C NMR (176 MHz, d_6-acetone): δ 143.2, 142.5, 129.4, 129.1, 129.1, 128.6, 128.1, 126.4, 94.6 (d, *J* = 0.8 Hz), 78.6, 68.5, 58.1, 45.1, 40.8, 32.5 ppm;

HRMS (−ESI) *m/z* Found $[M-H]^-$ 464.0261, $[C_{19}H_{21}Cl_3NO_4S]^-$ requires 464.0262, (*δ* = – 0.2 ppm);

$[\alpha]_D^{25.0}$ = + 3.9 (c. 1.3, MeOH);

Chiral SFC Analysis: CHIRALPAK IG (CO_2:MeOH, 80.0:20.0, 2.50 mL min^{-1}, 40 °C) t_R = 5.2 (major), 7.5 (minor) minutes.

2,2,2-trichloroethyl ((1R,3R)-3-hydroxy-1-phenylbutyl)sulfamate (**2zaa**)

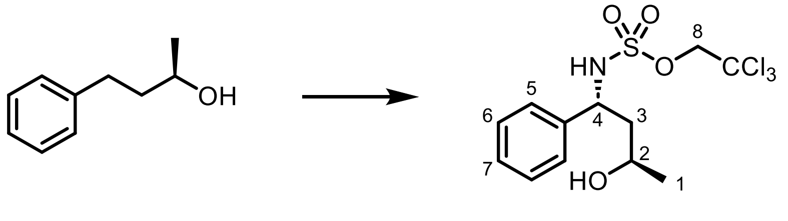

Prepared according to **GP10** using (*R*)-4-phenylbutan-2-ol as the starting material. Purification by flash column chromatography (SiO_2, 0-12% v/v Et_2O in CH_2Cl_2) afforded the title compound as a white solid (single diastereomer, 29.2 mg, 0.078 mmol, 78%). *A 20:1 d.r. for this reaction was determined through analysis of the crude ^{1}H NMR before purification. The relative stereochemistry in **2zaa** was assigned from the relative stereochemistry in azetidine **5a**.*

***R_f* value**: 0.36 (10% v/v Et_2O in CH_2Cl_2);

^{1}H NMR (500 MHz, CD_3OD): δ 7.40-7.34 (m, 4H, H-5, H-6), 7.29 (tt, *J* = 6.9, 1.8 Hz, 1H, H-7), 4.62 (dd, *J* = 9.1, 6.4 Hz, 1H, H-4), 4.28 (d, *J* = 10.9 Hz, 1H, H-8_A), 4.07 (d, *J* = 10.9 Hz, 1H, H-8_B), 3.48 (m, 1H, H-2), 2.06 (m, 1H, H-3_A), 1.82 (m, 1H, H-3_B), 1.15 (d, *J* = 6.3 Hz, 3H, H-1) ppm;

^{13}C NMR (126 MHz, CD_3OD): δ 142.9, 129.8, 128.9, 128.3, 94.9, 78.9, 65.3, 57.8, 47.1, 23.8 ppm;

HRMS (−ESI) *m/z* Found $[M-H]^-$ 373.9793, $[C_{12}H_{15}Cl_3NO_4S]^-$ requires 373.9793, (δ = + 0.0 ppm);

$[\alpha]_D^{25.0}$ = − 1.5 (c. 1.5, MeOH).

2,2,2-trichloroethyl ((1S,3R)-3-hydroxy-1-phenylbutyl)sulfamate (**2zab**)

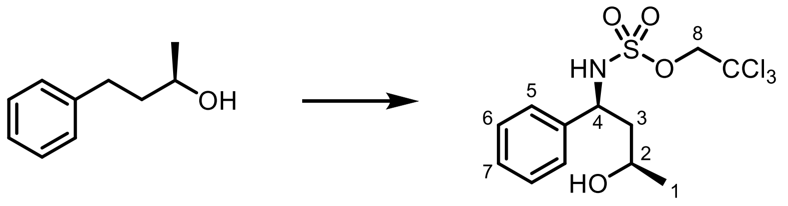

Prepared according to **GP10** using (*R*)-4-phenylbutan-2-ol as the starting material. Purification by flash column chromatography (SiO_2, 0-12% v/v Et_2O in CH_2Cl_2) afforded the title compound as a white solid (single diastereomer, 15.4 mg, 0.041 mmol, 41%). *A 7:1 d.r. for this reaction was determined through analysis of the crude ^{1}H NMR before purification. The relative stereochemistry in **2zab** was assigned as the opposite to **2zaa**.*

***R_f* value**: 0.39 (10% v/v Et_2O in CH_2Cl_2);

^{1}H NMR (700 MHz, CD_3OD): δ 7.39-7.33 (m, 4H, H-5, H-6), 7.27 (m, 1H, H-7), 4.67 (dd, *J* = 9.7, 4.6 Hz, 1H, H-4), 4.30 (d, *J* = 10.9 Hz, 1H, H-8_A), 4.12 (d, *J* = 10.9 Hz, 1H, H-8_B), 3.90 (m, 1H, H-2), 1.92 (ddd, *J* = 14.0, 9.9, 3.8 Hz, 1H, H-3_A), 1.77 (ddd, *J* = 13.7, 8.9, 4.6 Hz, 1H, H-3_B), 1.20 (d, *J* = 6.2 Hz, 3H, H-1) ppm;

^{13}C NMR (176 MHz, CD_3OD): δ 143.8, 129.8, 128.7, 127.8, 94.9 (d, *J* = 0.9 Hz), 78.9, 65.0, 57.4, 47.5, 23.9 ppm;

HRMS (−ESI) *m/z* Found $[M-H]^-$ 373.9796, $[C_{12}H_{15}Cl_3NO_4S]^-$ requires 373.9793, (δ = + 0.8 ppm);

$[\alpha]_D^{25.0}$ = − 28.9 (c. 1.0, MeOH).

ethyl (2R,4R)-2-hydroxy-4-phenyl-4-(((2,2,2-trichloroethoxy)sulfonyl)amino)butanoate (**2zba**)

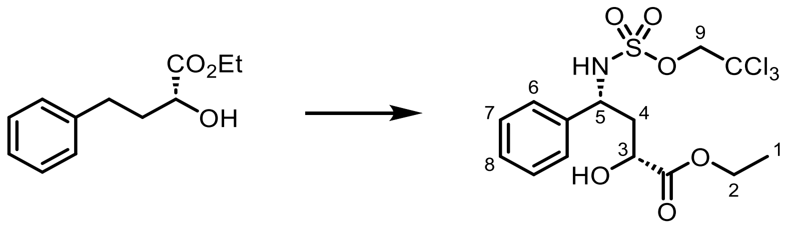

Prepared according to **GP10** using ethyl (*R*)-2-hydroxy-4-phenylbutanoate as the starting material. Purification by flash column chromatography (SiO_2, 0-10% v/v acetone in $CHCl_3$) afforded a 3.6:1 mixture of **2zbb** (below) to the title compound (20% calculated yield). The NMR data below is tentatively assigned based on this mixed sample. *This was used to assign 2:1 d.r. from crude ^{1}H NMR before purification. The relative stereochemistry in **2zba** was assigned as the opposite to **2zbb**.*

***R$_f$* value**: 0.55 (10% v/v acetone in $CHCl_3$);

^{1}H NMR (700 MHz, $CDCl_3$): δ 7.40-7.36 (m, 4H, H-6, H-7), 7.32 (m, 1H, H-8), 6.47 (br. s, 1H, N-H), 4.97 (m, 1H, H-5), 4.42 (d, *J* = 10.7 Hz, 1H, H-9$_A$), 4.40 (d, *J* = 10.7 Hz, 1H, H-9$_B$), 4.23-4.13 (m, 3H, H-2, H-3), 2.32 (m, 1H, H-4$_A$), 2.25 (m, 1H, H-4$_B$), 1.27 (t, *J* = 7.0 Hz, 3H, H-1) ppm;

^{13}C NMR (176 MHz, $CDCl_3$): δ 174.1, 139.2, 129.0, 128.3, 126.7, 93.5, 78.2, 67.9, 62.6, 56.8, 39.5, 14.3 ppm;

ethyl (2R,4S)-2-hydroxy-4-phenyl-4-(((2,2,2-trichloroethoxy)sulfonyl)amino)butanoate (**2zbb**)

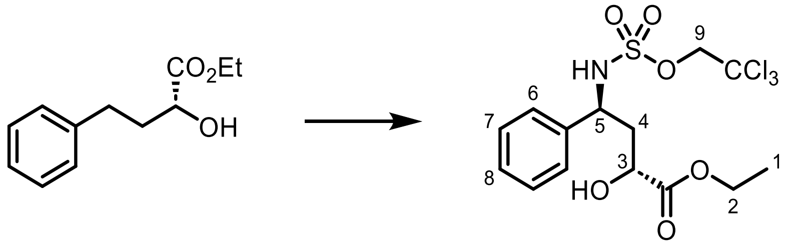

Prepared according to **GP10** using ethyl (*R*)-2-hydroxy-4-phenylbutanoate as the starting material. Purification by flash column chromatography (SiO_2, 0-8% v/v Et_2O in CH_2Cl_2) afforded the title compound as a colourless oil (single diastereomer, 23.9 mg, 0.055 mmol, 55%). *A 15:1 d.r. for this reaction was determined through analysis of the crude ^{1}H NMR before purification. The relative stereochemistry in **2zbb** was assigned from the relative stereochemistry in azetidine **5b**.*

***R$_f$* value**: 0.63 (10% v/v Et_2O in CH_2Cl_2);

^{1}H NMR (700 MHz, $CDCl_3$): δ 7.40-7.36 (m, 4H, H-6, H-7), 7.32 (m, 1H, H-8), 6.10 (br. s, 1H, N-H), 4.85 (m, 1H, H-5), 4.38 (d, *J* = 10.8 Hz, 1H, H-9$_A$), 4.34 (d, *J* = 10.8 Hz, 1H, H-9$_B$), 4.23-4.13 (m, 3H, H-2, H-3), 3.25 (br. s, 1H, O-H), 2.34 (ddd, *J* = 14.5, 5.8, 3.1 Hz, 1H, H-4$_A$), 2.21 (dt, *J* = 14.5, 8.8 Hz, 1H, H-4$_B$), 1.26 (t, *J* = 7.1 Hz, 3H, H-1) ppm;

^{13}C NMR (176 MHz, $CDCl_3$): δ 173.8, 139.9, 129.1, 128.6, 127.0, 93.4, 78.2, 69.2, 62.6, 58.0, 40.4, 14.2 ppm;

HRMS (−ESI) *m/z* Found [M−(CH_2CCl_3)]$^-$ 302.0711, [$C_{12}H_{16}NO_6S$]$^-$ requires 302.0704, (δ = + 2.3 ppm);

$[\alpha]_D^{25.0}$ = − 21.7 (c. 0.4, $CHCl_3$);

2,2,2-trichloroethyl (2S,4R)-2-methyl-4-phenylazetidine-1-sulfonate (**5a**)

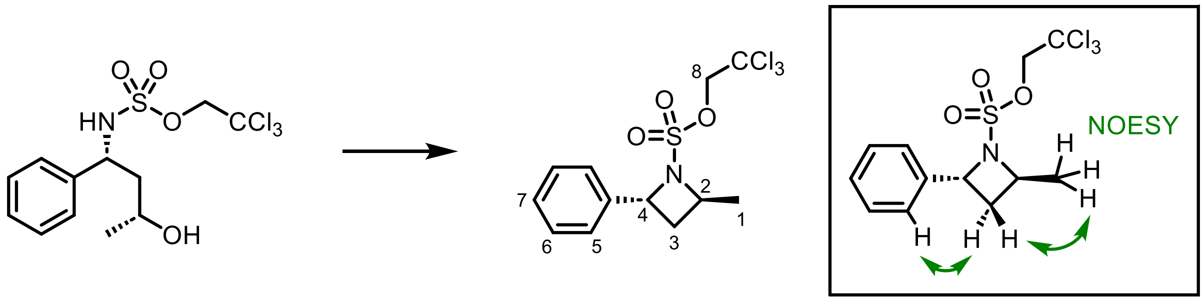

Prepared according to **GP11** using 2,2,2-trichloroethyl ((1*R*,3*R*)-3-hydroxy-1-phenylbutyl)sulfamate (**2zaa**) as the starting material on a 0.050 mmol scale. Purification by flash column chromatography (SiO_2, 0-10% v/v EtOAc in hexane) afforded the title compound as a colorless oil (13.3 mg, 0.037 mmol, 74%).

***R$_f$* value**: 0.33 (10% v/v EtOAc in hexane);

^{1}H NMR (700 MHz, $CDCl_3$): δ 7.48 (m, 2H, H-5), 7.39 (m, 2H, H-6), 7.33 (tt, *J* = 7.3, 1.9 Hz, 1H, H-7), 5.42 (dd, *J* = 8.8, 6.9 Hz, 1H, H-4), 4.65 (m, 1H, H-2), 4.37 (s, 2H, H-8), 2.58 (m, 1H, H-3$_A$), 2.34 (ddd, *J* = 11.4, 8.9, 4.6 Hz, 1H, H-3$_B$), 1.72 (d, *J* = 6.4 Hz, 3H, H-1) ppm;

^{13}C NMR (176 MHz, $CDCl_3$): δ 139.2, 129.0, 128.9, 127.2, 93.7 (d, *J* = 0.8 Hz), 77.9, 65.4, 60.5, 33.8, 20.0 ppm;

HRMS (−ESI): *m/z* found [M−H]$^-$ 355.9690, [$C_{12}H_{13}Cl_3NO_3S$]$^-$ requires 355.9687, (δ = + 0.8 ppm);

$[\alpha]_D^{25.0}$ = + 118.0 (c. 0.9, $CHCl_3$);

ethyl (2S,4S)-4-phenyl-1-((2,2,2-trichloroethoxy)sulfonyl)azetidine-2-carboxylate (**5b**)

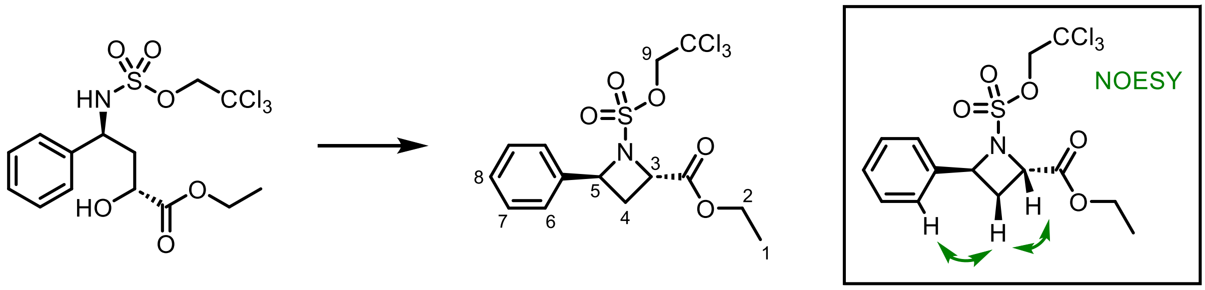

Prepared according to **GP11** using ethyl (2*R*,4*S*)-2-hydroxy-4-phenyl-4-(((2,2,2-trichloroethoxy)sulfonyl)amino)butanoate (**2zbb**) as the starting material on a 0.024 mmol scale. Purification by

flash column chromatography (SiO_2, 0-20% v/v EtOAc in hexane) afforded the title compound as a white solid (5.6 mg, 0.013 mmol, 55%).

***R**_f* **value**: 0.52 (20% v/v EtOAc in hexane);

^{1}H NMR (700 MHz, $CDCl_3$): δ 7.50 (m, 2H, H-6), 7.42 (m, 2H, H-7), 7.37 (tt, *J* = 7.3, 1.3 Hz, 1H, H-8), 5.67 (dd, *J* = 7.9, 7.8 Hz, 1H, H-5), 4.82 (dd, *J* = 9.1, 4.1 Hz, 1H, H-3), 4.37-4.33 (m, 3H, H-2, H-9_A), 4.24 (d, *J* = 10.8 Hz, 1H, H-9_B), 2.74 (ddd, *J* = 11.6, 9.1, 7.3 Hz, 1H, H-4_A), 2.67 (ddd, *J* = 11.6, 8.5, 4.1 Hz, 1H, H-4_B), 1.37 (t, *J* = 7.1 Hz, 3H, H-1) ppm;

^{13}C NMR (176 MHz, $CDCl_3$): δ 170.5, 138.3, 129.5, 129.1, 127.6, 93.3 (d, *J* = 0.9 Hz), 78.2, 67.9, 62.4, 62.3, 29.6, 14.2 ppm;

HRMS (+ESI): *m/z* found $[M+H]^+$ 415.9885, $[C_{14}H_{17}Cl_3NO_5S]^+$ requires 415.9888, (δ = – 0.7 ppm);

$[\alpha]_D^{25.0}$ = – 80.7 (c. 0.3, $CHCl_3$);

2,2,2-trichloroethyl (S)-1-phenyl-2-azaspiro[3.4]octane-2-sulfonate (**5c**)

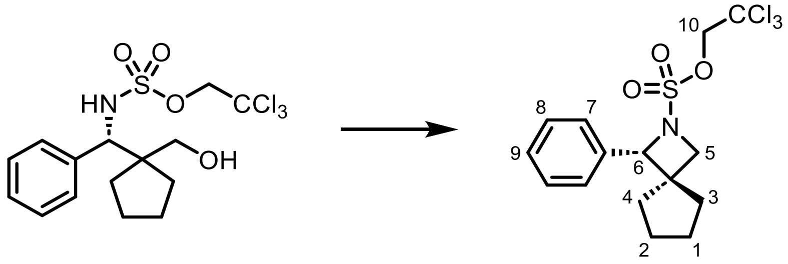

Prepared according to **GP11** using 2,2,2-trichloroethyl (S)-((1-(hydroxymethyl)cyclopentyl)(phenyl)methyl)sulfamate (**2q**) as the starting material on a 0.020 mmol scale. Purification by flash column chromatography (SiO_2, 0-6% v/v EtOAc in hexane) afforded the title compound as a white solid (5.4 mg, 0.014 mmol, 66%, 97% *ee*).

***R**_f* **value**: 0.46 (10% v/v EtOAc in hexane);

^{1}H NMR (700 MHz, $CDCl_3$): *δ* 7.40-7.34 (m, 4H, H-7, H-8), 7.31 (m, 1H, H-9), 5.33 (s, 1H, H-6), 4.67 (d, *J* = 10.9 Hz, 1H, H-10_A), 4.62 (d, *J* = 10.9 Hz, 1H, H-10_B), 4.13 (d, *J* = 7.6 Hz, 1H, H-5_A), 3.77 (d, *J* = 7.6 Hz, 1H, H-5_B), 1.96 (m, 1H, H-3_A), 1.89 (m, 1H, H-3_B), 1.58 (m, 2H, H-1), 1.51-1.40 (m, 2H, H-2_A, H-4_A), 1.35-1.25 (m, 2H, H-2_B, H-4_B) ppm;

^{13}C NMR (176 MHz, $CDCl_3$): *δ* 136.6, 128.6, 128.4, 126.7, 93.9, 77.9, 75.2, 62.7, 47.8, 38.5, 33.5, 23.63, 23.62 ppm;

HRMS (+ESI): *m/z* found $[M+H]^+$ 398.0149, $[C_{15}H_{19}Cl_3NO_3S]^+$ requires 398.0146, (δ = + 0.8 ppm);

$[\alpha]_D^{25.0}$ = + 81.2 (c. 0.2, $CHCl_3$);

Chiral SFC Analysis: CHIRALPAK IK (CO_2:MeOH, 96.0:4.0, 2.50 mL min^{-1}, 40 °C) t_R = 4.9 (minor), 5.3 (major) minutes.

2,2,2-trichloroethyl (2S,4R)-2-phenethyl-4-phenylazetidine-1-sulfonate (**5d**)

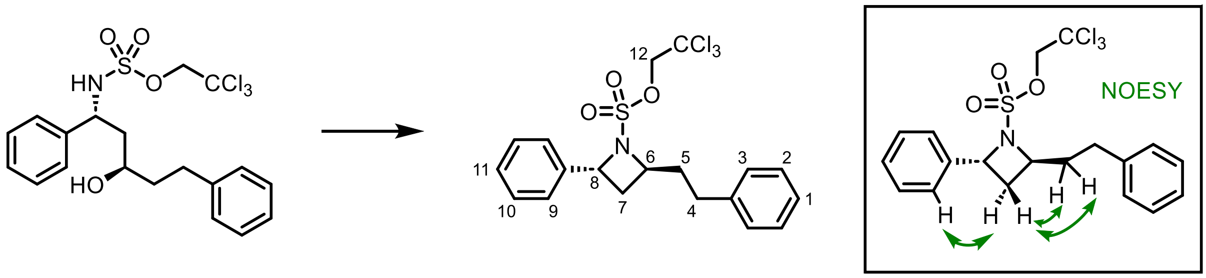

Prepared according to **GP11** using 2,2,2-trichloroethyl ((1*R*,3*R*)-3-hydroxy-1,5-diphenylpentyl)sulfamate (**2z**) as the starting material on a 0.048 mmol scale. Purification by flash column chromatography (SiO_2, 0-12% v/v EtOAc in hexane) afforded the title compound as a colorless oil (13.6 mg, 0.030 mmol, 64%, 88% *ee*).

***R_f* value**: 0.39 (10% v/v EtOAc in hexane);

^{1}H NMR (700 MHz, $CDCl_3$): δ 7.47 (m, 2H, H-9), 7.39 (m, 2H, H-10), 7.34 (tt, *J* = 7.4, 2.0 Hz, 1H, H-11), 7.30 (m, 2H, H-2), 7.23-7.20 (m, 3H, H-1, H-3), 5.38 (dd, *J* = 8.9, 6.5 Hz, 1H, H-8), 4.56 (m, 1H, H-6), 4.35 (d, *J* = 10.8 Hz, 1H, H-12_A), 4.29 (d, *J* = 10.9 Hz, 1H, H-12_B), 2.76 (m, 1H, H-4_A), 2.70-2.63 (m, 2H, H-4_B, H-5_A), 2.50 (m, 1H, H-7_A), 2.42 (ddd, *J* = 11.7, 9.1, 5.1 Hz, 1H, H-7_B), 2.30 (m, 1H, H-5_B) ppm;

^{13}C NMR (176 MHz, $CDCl_3$): δ 140.6, 139.0, 129.0, 128.9, 128.7, 128.5, 127.4, 126.4, 93.7, 77.9, 65.9, 64.0, 34.8, 31.9, 31.1 ppm;

HRMS (−ESI): *m/z* found [M−(CH_2CCl_3)]$^-$ 316.1009, [$C_{17}H_{18}NO_3S$]$^-$ requires 316.1013, (δ = − 1.3 ppm);

$[\alpha]_D^{25.0}$ = + 60.9 (c. 0.4, $CHCl_3$);

Chiral SFC Analysis: CHIRALPAK IG (CO_2:MeOH, 90.0:10.0, 2.50 mL min^{-1}, 40 °C) t_R = 7.7 (major), 12.7 (minor) minutes.

2,2,2-trichloroethyl (R)-2-phenyl-1-azaspiro[3.3]heptane-1-sulfonate (**5e**)

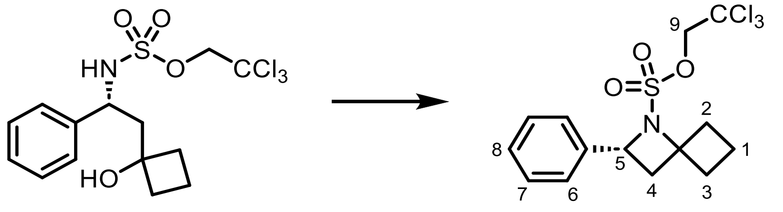

A vial was charged 2,2,2-trichloroethyl (*R*)-(2-(1-hydroxycyclobutyl)-1-phenylethyl)sulfamate (**2s**) (25.7 mg, 0.064 mmol, 83% *ee*) and triphenylphosphine (25.1 mg, 0.096 mmol) and placed under a nitrogen atmosphere. THF (2.0 mL) was added and the vial was cooled to 0 °C. After stirring at 0 °C for 10 minutes, diisopropyl azodicarboxylate (14 µL, 0.083 mmol) was added dropwise and the resulting mixture was gradually warmed to room temperature overnight. Evaporation of the solvent and purification of the crude material by flash column chromatography (SiO_2, 0-10% v/v EtOAc in hexane) afforded the title compound as a colorless oil (9.6 mg, 0.025 mmol, 39%, 82% *ee*).

***R_f* value**: 0.43 (10% v/v EtOAc in hexane);

^{1}H NMR (700 MHz, $CDCl_3$): δ 7.43 (m, 2H, H-6), 7.37 (m, 2H, H-7), 7.31 (m, 1H, H-8), 5.25 (dd, *J* = 8.2, 8.0 Hz, 1H, H-5), 4.51 (d, *J* = 10.8 Hz, 1H, H-9$_A$), 4.38 (d, *J* = 10.8 Hz, 1H, H-9$_B$), 3.08 (q, *J* = 10.5 Hz, 1H, H-2$_A$), 2.85 (dd, *J* = 11.2, 8.7 Hz, 1H, H-4$_A$), 2.70 (q, *J* = 10.5 Hz, 1H, H-3$_A$), 2.45 (dd, *J* = 11.1, 7.5 Hz, 1H, H-4$_B$), 2.28 (m, 1H, H-2$_B$), 2.20 (m, 1H, H-3$_B$), 1.84 (qt, *J* = 10.3, 2.6 Hz, 1H, H-1$_A$), 1.69 (m, 1H, H-1$_B$) ppm;

^{13}C NMR (176 MHz, $CDCl_3$): δ 139.5, 128.9, 128.7, 126.8, 93.6, 77.8, 69.1, 63.0, 41.3, 36.6, 34.1, 12.9 ppm;

HRMS (–ESI): *m/z* found $[M-(CH_2CCl_3)]^-$ 252.0698, $[C_{12}H_{14}NO_3S]^-$ requires 252.0700, (δ = – 0.8 ppm);

$[\alpha]_D^{25.0}$ = + 20.5 (c. 0.14, $CHCl_3$);

Chiral SFC Analysis: CHIRALPAK IG (CO_2:MeOH, 95.0:5.0, 2.50 mL min^{-1}, 40 °C) t_R = 5.5 (major), 8.6 (minor) minutes.

*Note: residual 'grease' was collected in the isolation of compound **5e**. This is most visible in the ^{1}H NMR spectrum below 1.5 ppm. The isolated yield reported above has been adjusted to account for this through calculation of wt% purity by comparison of ^{1}H integration relative to 1,3,5-trimethoxybenzene.*

2,2,2-trichloroethyl (R)-(4-hydroxy-1-phenylbutyl)sulfamate (**7a**)

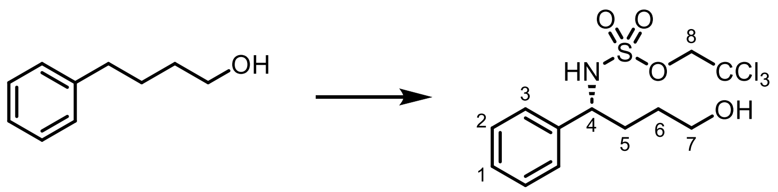

Prepared according to **GP10** using 4-phenylbutan-1-ol as the starting material. Purification by flash column chromatography (SiO_2, 0-24% v/v acetone in $CHCl_3$) afforded the title compound as a colorless oil (35.8 mg, 0.095 mmol, 95%, 92% *ee*).

***R_f* value**: 0.43 (20% v/v acetone in $CHCl_3$);

^{1}H NMR (400 MHz, $CDCl_3$): δ 7.39-7.27 (m, 5H, H-1, H-2, H-3), 6.18 (br. s, 1H, N-H), 4.55 (t, *J* = 6.4 Hz, 1H, H-4), 4.30 (q, *J* = 10.8 Hz, 2H, H-8), 3.69 (m, 2H, H-7), 2.10-1.90 (m, 3H, H-5, O-H), 1.62 (m, 2H, H-6) ppm;

^{13}C NMR (101 MHz, $CDCl_3$): δ 140.8, 129.1, 128.3, 126.8, 93.4, 78.1, 62.4, 59.3, 34.0, 28.6 ppm;

HRMS (−ESI): *m/z* found $[M-H]^-$ 373.9798, $[C_{12}H_{15}Cl_3NO_4S]^-$ requires 373.9793, (δ = + 1.3 ppm);

$[\alpha]_D^{25.0}$ = + 24.5 (c. 1.4, $CHCl_3$);

Chiral HPLC Analysis: CHIRAL ART SC (hexane:*i*PrOH, 90.0:10.0, 1.25 mL min^{-1}, 40 °C) t_R = 10.8 (major), 11.9 (minor) minutes.

ethyl (R)-3-(4-hydroxy-1-(((2,2,2-trichloroethoxy)sulfonyl)amino)butyl)benzoate (**7b**)

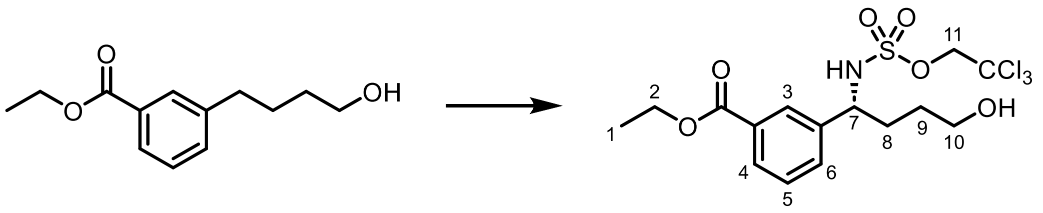

Prepared according to **GP10** using ethyl 3-(4-hydroxybutyl)benzoate as the starting material. Purification by flash column chromatography (SiO_2, 0-26% v/v acetone in $CHCl_3$) afforded the title compound as a colorless oil (33.1 mg, 0.074 mmol, 74%, 92% *ee*).

***Rf* value**: 0.37 (20% v/v acetone in $CHCl_3$);

^{1}H NMR (700 MHz, $CDCl_3$): δ 8.03 (t, *J* = 1.7 Hz, 1H, H-3), 7.96 (dt, *J* = 7.8, 1.2 Hz, 1H, H-4), 7.53 (dt, *J* = 7.7, 1.4 Hz, 1H, H-6), 7.43 (t, *J* = 7.7 Hz, 1H, H-5), 6.71 (br. s, 1H, N-H), 4.62 (dd, *J* = 8.0, 6.1 Hz, 1H, H-7), 4.42-4.34 (m, 4H, H-2, H-11), 3.73 (m, 1H, H-10_A), 3.68 (m, 1H, H-10_B), 2.26 (br. s, 1H, O-H), 2.05 (m, 1H, H-8_A), 1.98 (m, 1H, H-8_B), 1.70 (m, 1H, H-9_A), 1.60 (m, 1H, H-9_B), 1.39 (t, *J* = 7.2 Hz, 3H, H-1) ppm;

^{13}C NMR (176 MHz, $CDCl_3$): δ 166.7, 141.7, 131.6, 131.2, 129.2, 129.0, 127.6, 93.4 (d, *J* = 0.8 Hz), 78.0, 62.3, 61.5, 58.9, 34.2, 28.5, 14.5 ppm;

HRMS (−ESI): *m/z* found $[M-H]^-$ 446.0005, $[C_{15}H_{19}Cl_3NO_6S]^-$ requires 446.0004, (δ = + 0.2 ppm);

$[\alpha]_D^{25.0}$ = + 32.5 (c. 1.3, $CHCl_3$);

Chiral SFC Analysis: CHIRALPAK IG (CO_2:MeOH, 80.0:20.0, 1.25 mL min^{-1}, 40 °C) t_R = 7.0 (minor), 7.4 (major) minutes.

2,2,2-trichloroethyl (R)-(1-(3-chloro-2-methylphenyl)-4-hydroxybutyl)sulfamate (**7c**)

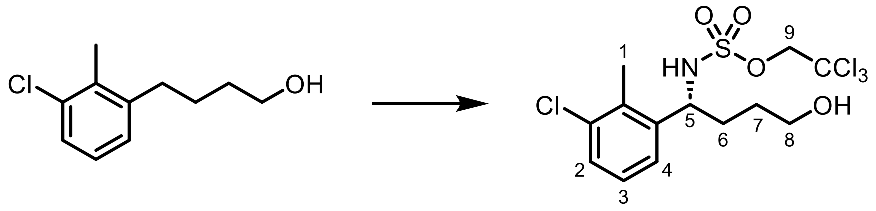

Prepared according to **GP10** using 4-(3-chloro-2-methylphenyl)butan-1-ol as the starting material. Purification by flash column chromatography (SiO_2, 0-25% v/v acetone in $CHCl_3$) afforded the title compound as a white solid (39.2 mg, 0.092 mmol, 92%, 93% *ee*).

***R_f* value**: 0.42 (20% v/v acetone in $CHCl_3$);

^{1}H NMR (700 MHz, CD_3OD): δ 7.35 (dd, J = 7.8, 0.9 Hz, 1H, H-4), 7.30 (dd, J = 8.0, 1.2 Hz, 1H, H-2), 7.21 (t, J = 7.9 Hz, 1H, H-3), 4.83 (dd, J = 8.6, 6.5 Hz, 1H, H-5), 4.36 (d, J = 10.8 Hz, 1H, H-9$_A$), 4.06 (d, J = 10.8 Hz, 1H, H-9$_B$), 3.58 (m, 2H, H-8), 2.46 (s, 3H, H-1), 1.89 (m, 1H, H-6$_A$), 1.82 (m, 1H, H-6$_B$), 1.70 (m, 1H, H-7$_A$), 1.53 (m, 1H, H-7$_B$) ppm;

^{13}C NMR (176 MHz, CD_3OD): δ 144.4, 136.2, 134.4, 129.4, 128.4, 125.8, 94.8 (d, J = 0.9 Hz), 78.8, 62.1, 56.0, 34.3, 30.2, 15.8 ppm;

HRMS (–ESI): *m/z* found $[M-H]^-$ 421.9564, $[C_{13}H_{16}Cl_4NO_4S]^-$ requires 421.9560, (δ = + 0.9 ppm);

$[\alpha]_D^{25.0}$ = – 4.1 (c. 2.5, MeOH);

Chiral SFC Analysis: CHIRALPAK IG (CO_2:MeOH, 80.0:20.0, 1.25 mL min^{-1}, 40 °C) t_R = 5.4 (minor), 5.7 (major) minutes.

2,2,2-trichloroethyl (R)-(5-hydroxy-1-phenylpentyl)sulfamate (**7d**)

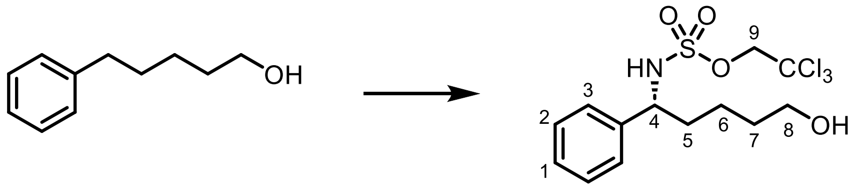

Prepared according to **GP10** using 5-phenylpentan-1-ol as the starting material. In this case the following procedural modification was used: Rh_2(**B-I**)$_2$●(**Cat1**)$_2$●(**pyr**)$_2$ was used as the chiral catalyst. Purification by flash column chromatography (SiO_2, 0-20% v/v acetone in $CHCl_3$) afforded the title compound as a colorless oil (30.4 mg, 0.078 mmol, 78%, 92% *ee*).

***R_f* value**: 0.45 (20% v/v acetone in $CHCl_3$);

^{1}H NMR (400 MHz, $CDCl_3$): δ 7.39-7.27 (m, 5H, H-1, H-2, H-3), 5.66 (br. s, 1H, N-H), 4.50 (m, 1H, H-4), 4.27 (q, J = 11.7 Hz, 2H, H-9), 3.63 (t, J = 6.3 Hz, 2H, H-8), 1.97 (m, 1H, H-5$_A$), 1.83 (m, 1H, H-5$_B$), 1.73 (br. s, 1H, O-H), 1.59 (m, 2H, H-7), 1.49 (m, 1H, H-6$_A$), 1.37 (m, 1H, H-6$_B$) ppm;

^{13}**C NMR** (101 MHz, $CDCl_3$): δ 140.8, 129.1, 128.4, 126.7, 93.4, 78.1, 62.5, 59.7, 36.7, 32.0, 22.5 ppm;

HRMS (−ESI): *m/z* found $[M-H]^-$ 387.9948, $[C_{13}H_{17}Cl_3NO_4S]^-$ requires 387.9949, (δ = − 0.3 ppm);

$[\alpha]_D^{25.0}$ = + 15.5 (c. 1.6, $CHCl_3$);

Chiral HPLC Analysis: CHIRAL ART SC (hexane:*i*PrOH, 90.0:10.0, 1.25 mL min^{-1}, 40 °C) t_R = 11.7 (major), 12.8 (minor) minutes.

2,2,2-trichloroethyl (R)-(1-(4-(tert-butyl)phenyl)-5-hydroxypentyl)sulfamate (**7e**)

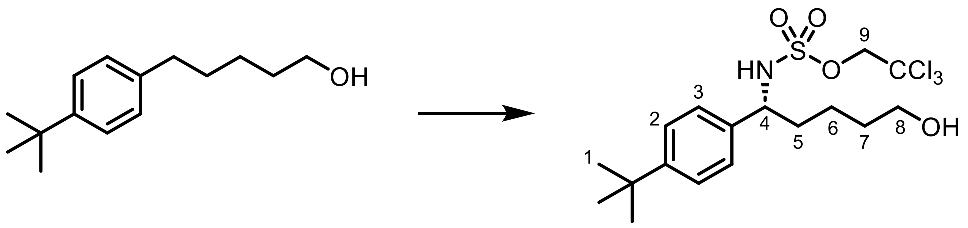

Prepared according to **GP10** using 5-(4-(tert-butyl)phenyl)pentan-1-ol as the starting material. In this case the following procedural modification was used: Rh_2(**B-I**)$_2$●(**Cat1**)$_2$●(**pyr**)$_2$ was used as the chiral catalyst. Purification by flash column chromatography (SiO_2, 0-20% v/v acetone in $CHCl_3$) afforded the title compound as a white solid (36.0 mg, 0.081 mmol, 81%, 86% *ee*).

***R$_f$* value**: 0.49 (20% v/v acetone in $CHCl_3$);

^{1}**H NMR** (700 MHz, $CDCl_3$): δ 7.37 (m, 2H, H-2), 7.23 (m, 2H, H-3), 5.63 (d, *J* = 7.6 Hz, 1H, N-H), 4.46 (q, *J* = 7.3 Hz, 1H, H-4), 4.18 (d, *J* = 10.8 Hz, 1H, H-9$_A$), 4.12 (d, *J* = 10.8 Hz, 1H, H-9$_B$), 3.64 (t, *J* = 6.3 Hz, 2H, H-8), 1.97 (m, 1H, H-5$_A$), 1.86-1.80 (m, 2H, H-5$_B$, O-H), 1.60 (m, 2H, H-7), 1.51 (m, 1H, H-6$_A$), 1.39 (m, 1H, H-6$_B$), 1.30 (s, 9H, H-1) ppm;

^{13}**C NMR** (176 MHz, $CDCl_3$): δ 151.4, 137.8, 126.5, 126.0, 93.4 (d, *J* = 0.8 Hz), 78.1, 62.6, 59.4, 36.6, 34.7, 32.1, 31.4, 22.6 ppm;

HRMS (−ESI): *m/z* found $[M-H]^-$ 444.0578, $[C_{17}H_{25}Cl_3NO_4S]^-$ requires 444.0575, (δ = + 0.7 ppm);

$[\alpha]_D^{25.0}$ = + 18.6 (c. 2.2, $CHCl_3$);

Chiral HPLC Analysis: CHIRAL ART SC (hexane:*i*PrOH, 90.0:10.0, 1.25 mL min^{-1}, 40 °C) t_R = 11.8 (major), 13.7 (minor) minutes.

2,2,2-trichloroethyl (R)-(1-(3-fluoro-2-methylphenyl)-5-hydroxypentyl)sulfamate (**7f**)

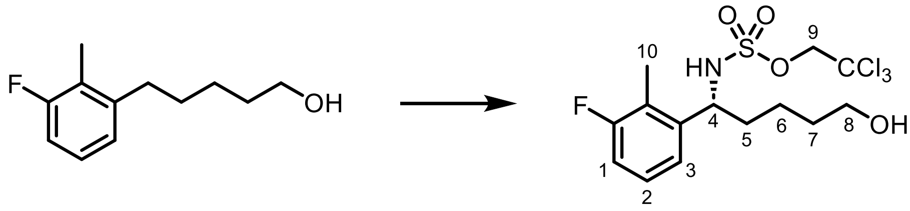

Prepared according to **GP10** using 5-(3-fluoro-2-methylphenyl)pentan-1-ol as the starting material. In this case the following procedural modification was used: Rh_2(**B-I**)$_2$●(**Cat1**)$_2$●(**pyr**)$_2$ was used as the chiral catalyst. Purification by flash column chromatography (SiO_2, 0-23% v/v acetone in $CHCl_3$) afforded the title compound as a colorless oil (33.9 mg, 0.080 mmol, 80%, 94% *ee*).

***R_f* value**: 0.42 (20% v/v acetone in $CHCl_3$);

^{1}H NMR (700 MHz, $CDCl_3$): δ 7.19 (m, 1H, H-2), 7.07 (d, *J* = 7.7 Hz, 1H, H-3), 6.96 (t, *J* = 8.7 Hz, 1H, H-1), 5.84 (d, *J* = 7.2 Hz, 1H, N-H), 4.81 (q, *J* = 7.1 Hz, 1H, H-4), 4.34 (d, *J* = 10.7 Hz, 1H, H-9_A), 4.21 (d, *J* = 10.7 Hz, 1H, H-9_B), 3.65 (t, *J* = 6.0 Hz, 2H, H-8), 2.30 (d, *J* = 1.8 Hz, 3H, H-10), 1.92 (m, 1H, H-5_A), 1.77 (m, 1H, H-5_B), 1.64 (m, 1H, H-7_A), 1.60-1.53 (m, 2H, H-6_A, H-7_B), 1.40 (m, 1H, H-6_B) ppm;

^{13}C NMR (176 MHz, $CDCl_3$): δ 161.3 (d, J_{C-F} = 245 Hz), 142.0, (d, J_{C-F} = 3.6 Hz), 127.5 (d, J_{C-F} = 9.0 Hz), 122.9 (d, J_{C-F} = 16.5 Hz), 121.0 (d, J_{C-F} = 3.3 Hz), 114.6 (d, J_{C-F} = 23.4 Hz), 93.3 (d, *J* = 0.8 Hz), 78.1, 62.5, 55.0 (d, J_{C-F} = 3.0 Hz), 36.5, 32.0, 22.5, 10.3 (d, J_{C-F} = 6.2 Hz) ppm;

^{19}F NMR (376 MHz, $CDCl_3$): δ – 114.7 ppm;

HRMS (–ESI): *m/z* found $[M-H]^-$ 420.0009, $[C_{14}H_{18}Cl_3FNO_4S]^-$ requires 420.0012, (δ = – 0.7 ppm);

$[\alpha]_D^{25.0}$ = + 12.3 (c. 2.1, $CHCl_3$);

Chiral HPLC Analysis: CHIRAL ART SC (hexane:*i*PrOH, 90.0:10.0, 1.25 mL min^{-1}, 40 °C) t_R = 8.0 (major), 9.1 (minor) minutes.

Enantioselective Intermolecular Aziridination Products

2,2,3,3,3-pentafluoropropyl (S)-3-(hydroxymethyl)-2,2-dimethylaziridine-1-sulfonate (**9a**)

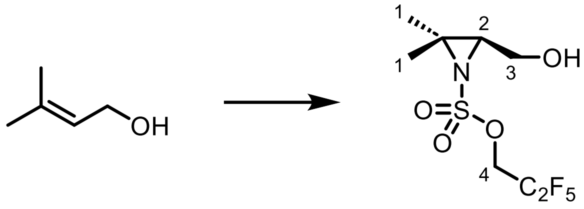

Prepared according to **GP12** using 3-methylbut-2-en-1-ol as the starting material. Purification by flash column chromatography (SiO_2, 0-10% v/v acetone in $CHCl_3$) afforded the title compound as a colourless oil (23.8 mg, 0.076 mmol, 76%).

***R_f* value**: 0.35 (10% v/v acetone in $CHCl_3$);

^{1}H NMR (500 MHz, $CDCl_3$): δ 4.66 (m, 2H, H-4), 3.82 (m, 1H, H-3_A), 3.74 (m, 1H, H-3_B), 3.09 (dd, J = 7.0, 5.5 Hz, 1H, H-2), 1.66 (s, 3H, H-1_A), 1.60 (s, 1H, O-H), 1.39 (s, 3H, H-1_B) ppm;

^{13}C NMR (126 MHz, $CDCl_3$): δ 118.3 (qt, $J_{C\text{-}F}$ = 286, 33.8 Hz), 111.4 (tq, $J_{C\text{-}F}$ = 257, 38.3 Hz), 65.5 (t, $J_{C\text{-}F}$ = 28.4 Hz), 60.2, 54.2, 51.8, 21.1, 20.8 ppm;

^{19}F NMR (376 MHz, $CDCl_3$): δ – 84.5, – 124.6 ppm;

HRMS (+ESI) *m/z* Found $[M+H]^+$ 314.0479, $[C_8H_{13}F_5NO_4S]^+$ requires 314.0480 (δ = – 0.3 ppm);

$[\alpha]_D^{25.0}$ = + 16.5 (c. 1.2, $CHCl_3$).

Conversion of **9a** *to the thiophenolate adduct for ee determination: 2,2,3,3,3-pentafluoropropyl (R)-(1-hydroxy-3-methyl-3-(phenylthio)butan-2-yl)sulfamate* (A) and *2,2,3,3,3-pentafluoropropyl (R)-(4-hydroxy-2-methyl-3-(phenylthio)butan-2-yl)sulfamate* (B)

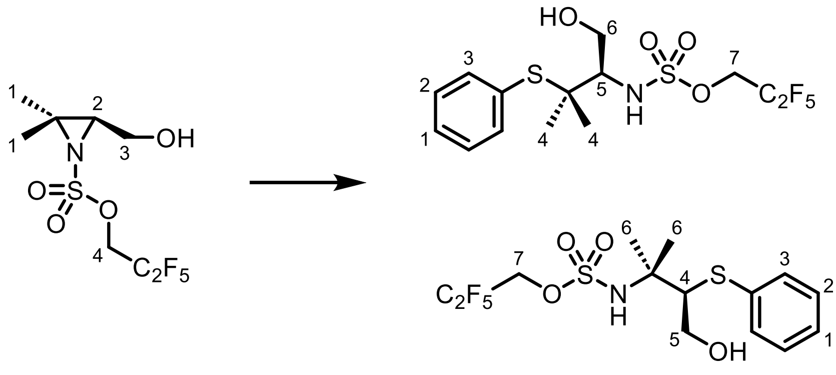

A

B

Prepared according to **GP13** on a 0.08 mmol scale with respect to 2,2,3,3,3-pentafluoropropyl (R)-3-(hydroxymethyl)-2,2-dimethylaziridine-1-sulfonate (**9a**). The 6.7:1 mixture of title compounds A:B was isolated as a white solid (24.2 mg, 0.057 mmol, 71%, 89% *ee*).

NMR data for major product **A**:

^{1}H NMR (700 MHz, $CDCl_3$): δ 7.52 (m, 2H, H-3), 7.42 (tt, *J* = 7.5, 2.1 Hz, 1H, H-1), 7.36 (m, 2H, H-2), 5.58 (d, *J* = 8.7 Hz, 1H, N-H), 4.55 (t, *J* = 12.5 Hz, 2H, H-7), 4.05 (dd, *J* = 11.9, 4.7 Hz, 1H, H-6A), 3.97 (dd, *J* = 11.9, 4.0 Hz, 1H, H-6B), 3.47 (m, 1H, H-5), 2.19 (br. s, 1H, O-H), 1.37 (s, 3H, H-4_A), 1.31 (s, 3H, H-4_B) ppm;

^{13}C NMR (176 MHz, $CDCl_3$): δ 137.6, 129.9, 129.8, 129.2, 118.3 (qt, J_{C-F} = 286, 34.3 Hz), 111.6 (tq, J_{C-F} = 257, 38.4 Hz), 64.2 (t, J_{C-F} = 28.2 Hz), 64.2, 61.5, 52.0, 27.5, 27.0 ppm;

^{19}F NMR (376 MHz, $CDCl_3$): δ − 83.5, − 123.5 ppm;

NMR data for minor product **B**:

^{1}H NMR (700 MHz, $CDCl_3$): δ 7.47 (m, 0.3H, H-3), 7.33 (m, 0.3H, H-2), 7.30 (m, 0.17H, H-1), 6.17 (s, 0.14H, N-H), 4.48 (m, 0.26H, H-7), 4.02 (dd, *J* = 12.0, 3.8 Hz, 0.17H, H-5A), 3.89 (dd, *J* = 12.0, 4.3 Hz, 0.15H, H-5B), 3.22 (t, *J* = 4.3 Hz, 0.15H, H-4), 2.41 (br. s, 0.15H, O-H), 1.62 (s, 0.57H, H-6_A), 1.57 (s, 0.47H, H-6_B) ppm;

^{13}C NMR (176 MHz, $CDCl_3$): δ 134.2, 132.5, 129.6, 128.2, 64.1 (t, J_{C-F} = 28.1 Hz), 63.0, 62.2, 60.8, 26.5, 25.0 ppm;

Note – the ^{13}C resonances for the fluorinated carbon atoms in the minor product are not observed

^{19}F NMR (376 MHz, $CDCl_3$): δ − 83.5, − 123.2 ppm;

HRMS (+ESI) *m/z* Found $[M+H]^+$ 424.0669, $[C_{14}H_{19}F_5NO_4S_2]^+$ requires 424.0670 (δ = − 0.2 ppm);

$[\alpha]_D^{25.0}$ = − 7.2 (c. 1.3, $CHCl_3$).

Chiral SFC Analysis: CHIRALPAK IG (CO_2:MeOH, 85.0:15.0, 2.50 mL min^{-1}, 40 °C) for major regioisomer A t_R = 2.1 (major), 3.6 (minor) minutes.

2,2,3,3,3-pentafluoropropyl (S)-2,2-dibutyl-3-(hydroxymethyl)aziridine-1-sulfonate (**9b**)

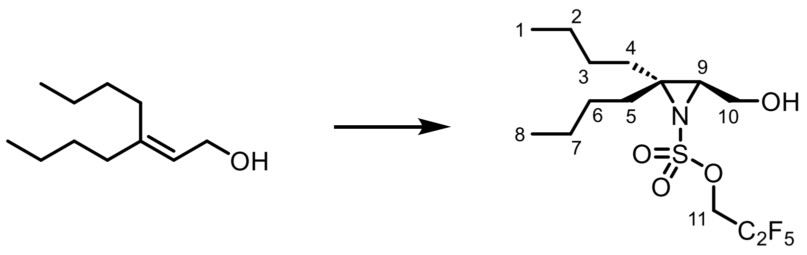

Prepared according to **GP12** using 3-butylhept-2-en-1-ol as the starting material. Purification by flash column chromatography (SiO_2, 0-3% v/v acetone in $CHCl_3$) afforded the title compound as a colourless oil (25.8 mg, 0.065 mmol, 65%).

***R*$_f$ value**: 0.58 (5% v/v acetone in $CHCl_3$);

^{1}H NMR (500 MHz, $CDCl_3$): δ 4.65 (t, J = 12.7 Hz, 2H, H-11), 3.78 (m, 2H, H-10), 3.05 (dd, J = 6.9, 5.7 Hz, 1H, H-9), 2.14 (m, 1H, C<u>H</u>), 1.58 (m, 1H, O-H), 1.74-1.31 (m, 11H, 11 × C<u>H</u>), 0.93 (m, 6H, H-1, H-8) ppm;

^{13}C NMR (126 MHz, $CDCl_3$): δ 118.4 (qt, $J_{C\text{-}F}$ = 287, 34.9 Hz), 111.5 (tq, $J_{C\text{-}F}$ = 256, 38.9 Hz), 65.4 (t, $J_{C\text{-}F}$ = 28.7 Hz), 59.9, 58.8, 54.5, 31.2, 30.1, 28.6, 27.3, 22.7, 22.6, 14.1, 14.0 ppm;

^{19}F NMR (376 MHz, $CDCl_3$): δ – 83.5, – 123.6 (m) ppm;

HRMS (+ESI) *m/z* Found $[M+(CH_3CN)+H]^+$ 439.1684, $[C_{16}H_{28}F_5N_2O_4S]^+$ requires 439.1684 (δ = + 0.0 ppm);

$[\alpha]_D^{25.0}$ = + 14.4 (c. 0.7, $CHCl_3$).

*Conversion of **9b** to the thiophenolate adduct for ee determination: 2,2,3,3,3-pentafluoropropyl (R)-(3-butyl-1-hydroxy-3-(phenylthio)heptan-2-yl)sulfamate.*

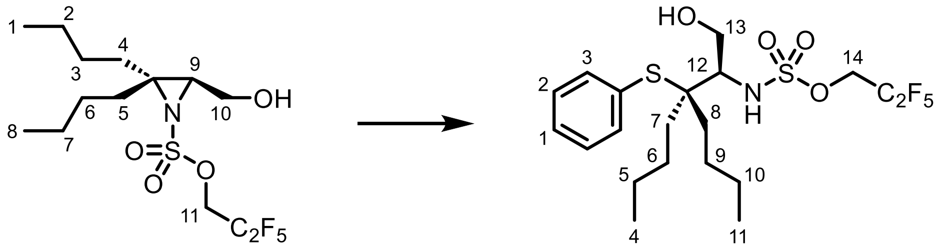

Prepared according to **GP13** on a 0.057 mmol scale with respect to 2,2,3,3,3-pentafluoropropyl (S)-2,2-dibutyl-3-(hydroxymethyl)aziridine-1-sulfonate (**9b**). Purification by flash column chromatography (SiO_2, 0-3% v/v acetone in $CHCl_3$) afforded the title compound as a colourless oil (24.6 mg, 0.048 mmol, 86%, 88% *ee*).

***R*$_f$ value**: 0.61 (5% v/v acetone in $CHCl_3$);

^{1}H NMR (700 MHz, $CDCl_3$): δ 7.53 (m, 2H, H-3), 7.41 (m, 1H, H-1), 7.36 (m, 2H, H-2), 5.37 (br. s, 1H, N-H), 4.44 (m, 2H, H-14), 3.90 (dd, J = 12.0, 5.3 Hz, 1H, H-13$_A$), 3.78 (m, 1H, H-13$_B$), 3.61 (dd, J = 5.6, 4.0 Hz, 1H, H-12), 2.37 (s, 1H, O-H), 1.63-1.20 (m, 12H, H-5, H-6, H-7, H-8, H-9, H-10), 0.93 (m, 6H, H-4, H-11) ppm;

^{13}C NMR (176 MHz, $CDCl_3$): δ 137.2, 130.6, 129.9, 129.4, 118.4 (qt, $J_{C\text{-}F}$ = 286, 34.2 Hz), 111.6 (tq, $J_{C\text{-}F}$ = 256, 38.2 Hz), 64.1 (t, $J_{C\text{-}F}$ = 27.7 Hz), 63.7, 62.1, 59.5, 34.7, 34.6, 25.8, 25.7, 23.2, 23.0, 14.2, 14.1 ppm;

^{19}F NMR (376 MHz, $CDCl_3$): δ – 83.6, – 123.5 ppm;

Note: there are traces of the thiophenol-opened minor regioisomer visible in the ^{1}H NMR spectrum but this has not been assigned.

HRMS (+ESI) *m/z* Found $[M+H]^+$ 508.1603, $[C_{20}H_{31}F_5NO_4S_2]^+$ requires 508.1609 (δ = – 1.2 ppm);

$[\alpha]_D^{25.0}$ = + 16.0 (c. 1.5, $CHCl_3$).

Chiral SFC Analysis: CHIRALPAK IJ (CO_2:MeOH, 98.0:2.0, 2.50 mL min^{-1}, 40 °C) t_R = 3.6 (major), 4.2 (minor) minutes

2,2,3,3,3-pentafluoropropyl (S)-2-(hydroxymethyl)-1-azaspiro[2.5]octane-1-sulfonate (**9c**)

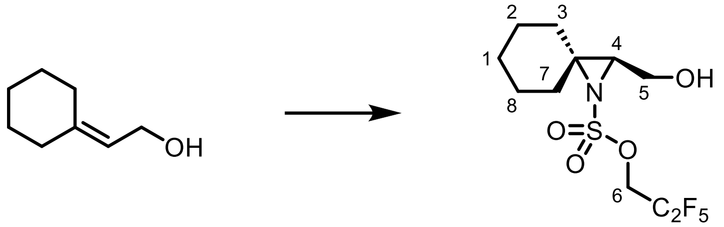

Prepared according to **GP12** using 2-cyclohexylideneethan-1-ol as the starting material. Purification by flash column chromatography (SiO_2, 0-8% v/v acetone in $CHCl_3$) afforded the title compound as a colourless oil (26.0 mg, 0.074 mmol, 74%).

***Rf* value**: 0.50 (10% v/v acetone in $CHCl_3$);

^{1}H NMR (700 MHz, $CDCl_3$): δ 4.66 (t, J = 12.3 Hz, 2H, H-6), 3.84 (dd, J = 11.8, 5.5 Hz, 1H, H-5$_A$), 3.74 (dd, J = 11.8, 7.1 Hz, 1H, H-5$_B$), 3.06 (dd, J = 7.1, 5.5 Hz, 1H, H-4), 2.06 (m, 1H, C<u>H</u>), 1.95-1.88 (m, 2H, 2 × C<u>H</u>), 1.78-1.72 (m, 2H, 2 × C<u>H</u>), 1.65-1.45 (m, 6H, 5 × C<u>H</u>, O-H) ppm;

^{13}C NMR (176 MHz, $CDCl_3$): δ 118.9 (qt, $J_{C\text{-}F}$ = 287, 34.6 Hz), 111.5 (tq, $J_{C\text{-}F}$ = 256, 38.3 Hz), 65.5 (t, $J_{C\text{-}F}$ = 28.4 Hz), 59.8, 57.7, 54.0, 31.6, 31.2, 25.4, 25.3, 25.2 ppm;

^{19}F NMR (376 MHz, $CDCl_3$): δ – 83.5, – 123.6 (m);

HRMS (+ESI) *m/z* Found [M+(CH_3CN)+H]$^+$ 391.1061, [$C_{13}H_{20}F_5N_2O_4S$]$^+$ requires 391.1058 (δ = + 0.8 ppm);

$[\alpha]_D^{25.0}$ = + 20.5 (c. 0.7, $CHCl_3$).

Conversion of **9c** *to the thiophenolate adduct for ee determination: 2,2,3,3,3-pentafluoropropyl (R)-(2-hydroxy-1-(1-(phenylthio)cyclohexyl)ethyl)sulfamate* (**A**) and *2,2,3,3,3-pentafluoropropyl (R)-(1-(2-hydroxy-1-(phenylthio)ethyl)cyclohexyl)sulfamate* (**B**)

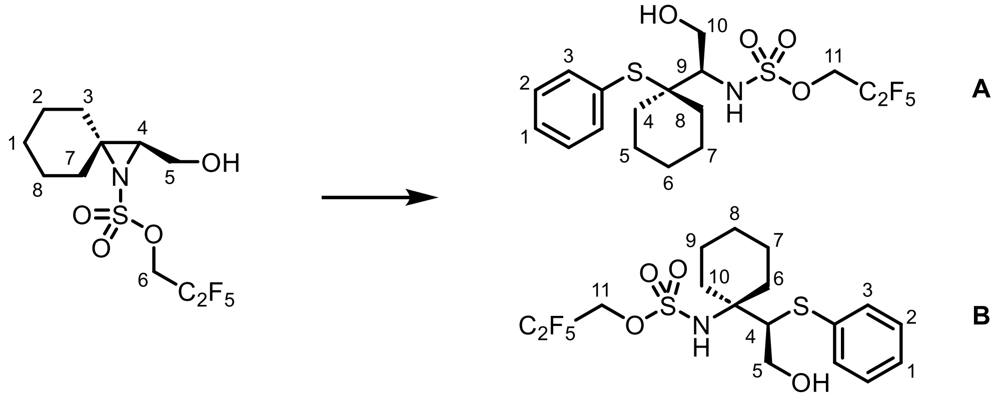

Prepared according to **GP13** on a 0.042 mmol scale with respect to 2,2,3,3,3-pentafluoropropyl (S)-2-(hydroxymethyl)-1-azaspiro[2.5]octane-1-sulfonate (**9c**). Purification by flash column chromatography (SiO_2, 0-6% v/v acetone in $CHCl_3$) afforded the 4:1 mixture of title compounds A:B as a colourless oil (22.0 mg, quant.%, 92% *ee*).

***R$_f$* value**: 0.54 (10% v/v acetone in $CHCl_3$);

NMR data for major product **A**:

^{1}H NMR (700 MHz, $CDCl_3$): δ 7.52 (m, 2H, H-3), 7.41 (m, 1H, H-1), 7.36 (m, 2H, H-2), 5.45 (d, *J* = 8.9 Hz, 1H, N-H), 4.48 (m, 2H, H-11), 4.03 (m, 1H, H-10$_A$), 3.88 (m, 1H, H-10$_B$), 3.64 (m, 1H, H-9), 1.84-1.25 (m, 14H, 10 × C<u>H</u>) ppm;

^{13}C NMR (176 MHz, $CDCl_3$): δ 137.2, 132.9, 129.8, 129.4, 118.4 (qt, J_{C-F} = 287, 33.8 Hz), 111.5 (tq, J_{C-F} = 256, 39.4 Hz), 64.1 (t, J_{C-F} = 27.8 Hz), 63.0, 61.4, 57.1, 34.3, 33.6, 25.4, 22.0, 21.8 ppm;

^{19}F NMR (376 MHz, $CDCl_3$): δ − 83.6, − 123.5 ppm;

NMR data for minor product **B**:

^{1}H NMR (700 MHz, $CDCl_3$): δ 7.48 (m, 0.50H, H-3), 7.35-7.29 (m, 0.70H, H-1, H-2), 6.02 (br. s, 0.22H, N-H), 4.62 (q, *J* = 12.2 Hz, 0.24H, H-11$_A$), 4.52 (q, *J* = 12.2 Hz, 0.28H, H-11$_B$), 4.03 (m, 0.24H, H-5$_A$), 3.88 (m, 0.24H, H-5$_B$), 3.62 (t, *J* = 3.7 Hz, 0.28H, H-4), 2.28 (m, 0.37H, C<u>H</u>), 2.17 (m, 0.37H, C<u>H</u>), 2.11-2.04 (m, 0.63H, 2 × C<u>H</u>), 1.84-1.25 (m, 14H, 6 × C<u>H</u>) ppm;

^{13}C NMR (176 MHz, $CDCl_3$): δ 134.2, 130.0, 129.6, 128.2, 64.4, 64.2 (t, J_{C-F} = 27.9 Hz), 61.4, 57.5, 33.7, 31.9, 24.9, 22.3, 22.2 ppm;

*Note: the resonances attributed to the fluorinated carbon atoms in the minor product **B** were not detected.*

^{19}F NMR (376 MHz, $CDCl_3$): δ − 83.5, − 123.2 ppm;

HRMS (+ESI) *m/z* Found [M+H]$^+$ 464.0981, $[C_{17}H_{23}F_5NO_4S_2]^+$ requires 464.0983 (δ = − 0.4 ppm);

$[\alpha]_D^{25.0}$ = + 2.9 (c. 0.8, $CHCl_3$).

Chiral SFC Analysis: CHIRALPAK IG (CO_2:MeOH, 92.0:8.0, 2.50 mL min^{-1}, 40 °C) for major regioisomer A t_R = 3.7 (major), 4.5 (minor) minutes.

2,2,3,3,3-pentafluoropropyl (S)-2-(hydroxymethyl)-1-azaspiro[2.11]tetradecane-1-sulfonate (**9d**)

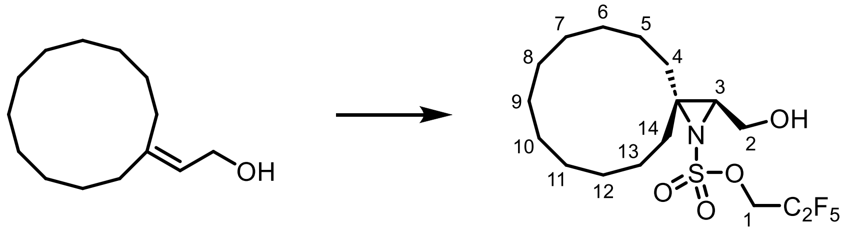

Prepared according to **GP12** using 2-cyclododecylideneethan-1-ol as the starting material. Purification by flash column chromatography (SiO_2, 0-4% v/v acetone in $CHCl_3$) afforded the title compound as a white solid (32.1 mg, 0.073 mmol, 73%).

***R_f* value**: 0.38 (5% v/v acetone in $CHCl_3$);

^{1}H NMR (500 MHz, $CDCl_3$): δ 4.65 (t, J = 12.3 Hz, 2H, H-1), 3.81 (dd, J = 11.9, 5.6 Hz, 1H, H-2$_A$), 3.74 (dd, J = 11.7, 7.5 Hz, 1H, H-2$_B$), 3.05 (dd, J = 7.1, 5.7 Hz, 1H, H-3), 2.11 (m, 1H, H-4$_A$), 1.74 (m, 1H, H-4$_B$), 1.70-1.48 (m, 7H, H-5, H-14, 2 × C<u>H</u>, O-H), 1.45-1.30 (m, 14H, 14 × C<u>H</u>) ppm;

^{13}C NMR (126 MHz, $CDCl_3$): δ 118.3 (qt, J_{C-F} = 286, 34.2 Hz), 111.5 (tq, J_{C-F} = 256, 38.4 Hz), 65.5 (t, J_{C-F} = 28.4 Hz), 60.1, 59.1, 54.1, 28.2, 27.0, 26.2, 26.1, 26.0, 22.34, 22.26, 22.2, 22.1, 21.4, 20.7 ppm;

^{19}F NMR (376 MHz, $CDCl_3$): δ – 83.5, – 123.5 (m) ppm;

HRMS (+ESI) *m/z* Found $[M+H]^+$ 438.1730, $[C_{17}H_{29}F_5NO_4S]^+$ requires 438.1732 (δ = – 0.5 ppm);

$[\alpha]_D^{25.0}$ = + 21.2 (c. 1.7, $CHCl_3$).

Conversion of **9d** *to the thiophenolate adduct for ee determination: 2,2,3,3,3-pentafluoropropyl (R)-(2-hydroxy-1-(1-(phenylthio)cyclododecyl)ethyl)sulfamate* (**A**) and *2,2,3,3,3-pentafluoropropyl (R)-(1-(2-hydroxy-1-(phenylthio)ethyl)cyclododecyl)sulfamate* (**B**)

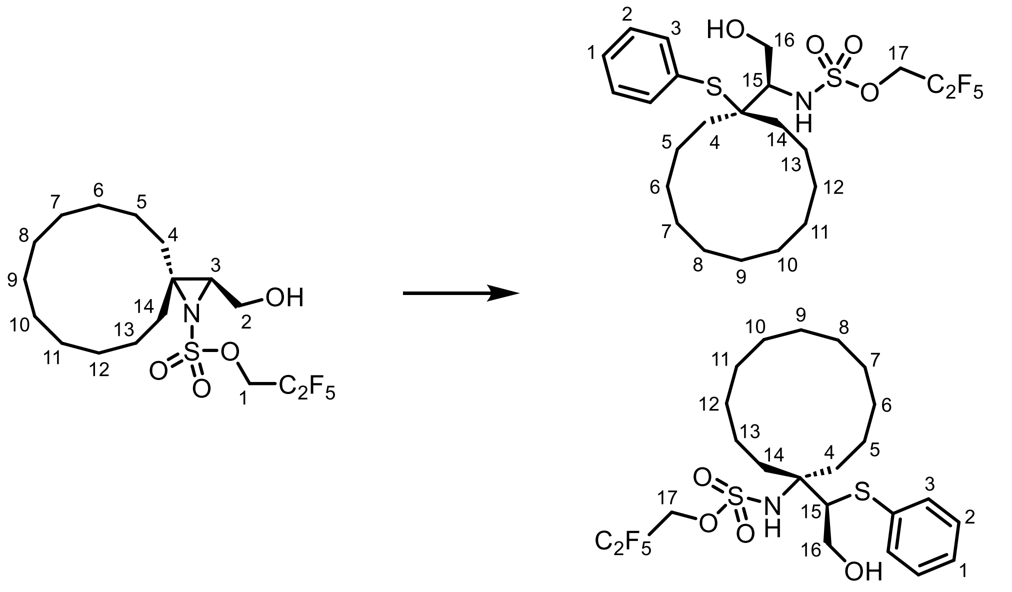

A

B

Prepared according to **GP13** on a 0.053 mmol scale with respect to 2,2,3,3,3-pentafluoropropyl (S)-2-(hydroxymethyl)-1-azaspiro[2.11]tetradecane-1-sulfonate (**9d**). Purification by flash column chromatography (SiO_2, 0-3% v/v acetone in $CHCl_3$) afforded the 2.4:1 mixture of title compounds A:B as a colourless oil (21.9 mg, 0.040 mmol, 75%, 90% *ee*).

*Note: there is approximately 5% of inseparable, unreacted aziridine (**9d**) present in this compound. This is most noticeable by the peak at 3.05 ppm in the ^{1}H NMR spectrum. The yield has been adjusted to take this into account.*

***R_f* value**: 0.58 (4% v/v acetone in $CHCl_3$);

<u>NMR data for major product **A**:</u>

^{1}H NMR (500 MHz, $CDCl_3$): δ 7.55 (m, 2H, H-3), 7.43-7.28 (m, 4.3H, H-1, H-2), 5.31 (br. s, 1H, N-H), 4.44 (m, 2H, H-17), 3.90 (m, 1H, H-16_A), 3.78 (m, 1H, H-16_B), 3.53 (t, *J* = 3.9 Hz, 1H, H-15), 2.33 (dd, *J* = 8.3, 4.3 Hz, 1H, O-H), 1.82-1.71 (m, 2.49H, 2 × C<u>H</u>), 1.76-1.22 (m, 27.2H, 20 × C<u>H</u>) ppm;

^{19}F NMR (376 MHz, $CDCl_3$): δ – 83.6, – 123.5 ppm;

<u>NMR data for minor product **B**:</u>

^{1}H NMR (500 MHz, $CDCl_3$): δ 7.45 (m, 0.87H, H-3), 7.43-7.28 (m, 4.3H, H-1, H-2), 5.86 (br. s, 0.39H, N-H), 4.61 (m, 0.57H, H-17_A), 4.52 (m, 0.52H, H-17_B), 4.05 (m, 0.42H, H-16_A), 3.86 (m, 0.43H, H-16_B), 3.22 (dd, *J* = 4.8, 3.5 Hz, 0.41H, H-15), 2.41 (t, *J* = 6.1 Hz, 0.39H, O-H), 2.20 (m, 0.45H, C<u>H</u>), 2.00-1.91 (m, 0.83H, 2 × C<u>H</u>), 1.82-1.71 (m, 2.49H, C<u>H</u>), 1.76-1.22 (m, 27.2H, 18 × C<u>H</u>) ppm;

^{19}F NMR (376 MHz, $CDCl_3$): δ – 83.5, – 123.3 ppm;

<u>Combined ^{13}C NMR data for the mixture of **A** and **B**:</u>

^{13}C NMR (126 MHz, $CDCl_3$): δ 136.9, 134.4, 132.2, 130.7, 129.8, 129.6, 129.5, 128.0, 118.4 (qt, J_{C-F} = 285, 33.6 Hz), 118.3 (qt, J_{C-F} = 287, 34.0 Hz), 111.7 (tq, J_{C-F} = 259, 37.9 Hz), 111.5 (tq, J_{C-F} = 257, 38.3 Hz), 67.4, 64.3, 64.0 (m), 63.8, 62.3, 61.3, 60.6, 59.7, 32.32, 32.25, 30.8, 26.82, 26.75, 26.6, 26.2, 26.1, 23.2, 23.2, 23.04, 22.99, 22.8, 22.7, 22.6, 19.9, 19.6 ppm;

HRMS (+ESI) *m/z* Found $[M+H]^+$ 548.1929, $[C_{23}H_{35}F_5NO_4S_2]^+$ requires 548.1922 (δ = + 1.3 ppm);

$[\alpha]_D^{25.0}$ = – 2.8 (c. 1.3, $CHCl_3$).

Chiral SFC Analysis: CHIRALPAK IJ (CO_2:MeOH, 95.0:5.0, 2.50 mL min^{-1}, 40 °C) for major regioisomer t_R = 4.6 (major), 5.2 (minor) minutes.

2,2,3,3,3-pentafluoropropyl (S)-6,6-difluoro-2-(hydroxymethyl)-1-azaspiro[2.5]octane-1-sulfonate (**9e**)

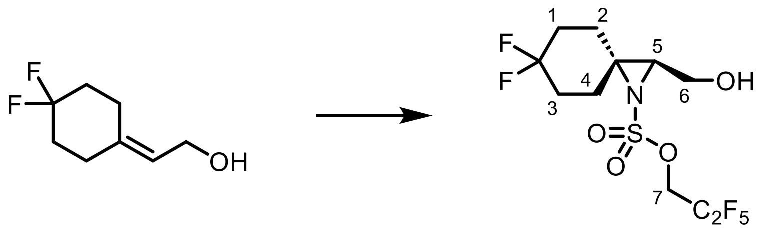

Prepared according to **GP12** using 2-(4,4-difluorocyclohexylidene)ethan-1-ol as the starting material. Purification by flash column chromatography (SiO_2, 0-8% v/v acetone in $CHCl_3$) afforded the title compound as a colourless oil (26.9 mg, 0.069 mmol, 69%).

R —

$\boldsymbol{R_f}$ **value**: 0.44 (10% v/v acetone in $CHCl_3$);

^{1}H NMR (500 MHz, $CDCl_3$): δ 4.68 (m, 2H, H-7), 3.82 (m, 2H, H-6), 3.15 (t, J = 6.3 Hz, 1H, H-5), 2.38-2.19 (m, 3H, 2 × C<u>H</u>, 1 × C<u>H</u>CF$_2$), 2.15-2.03 (m, 3H, 3 × C<u>H</u>CF$_2$), 1.93-1.80 (m, 2H, 2 × C<u>H</u>), 1.67 (br. t, J = 5.2 Hz, 1H, O-H) ppm;

^{13}C NMR (126 MHz, $CDCl_3$): δ 122.1 (t, $J_{C\text{-}F}$ = 242 Hz), 118.3 (qt, $J_{C\text{-}F}$ = 286, 34.0 Hz), 111.4 (tq, $J_{C\text{-}F}$ = 257, 38.8 Hz), 65.8 (t, $J_{C\text{-}F}$ = 28.6 Hz), 59.6, 54.3, 52.7, 32.1 (m), 27.2 (m) ppm;

Note: the peaks at 32.1 (m) and 27.2 (m) each account for 2 × ^{13}C resonances;

^{19}F NMR (376 MHz, $CDCl_3$): δ – 83.5, – 96.3 (d, J = 239 Hz), – 100.4 (d, J = 240 Hz), – 123.5 ppm;

HRMS (+ESI) *m/z* Found $[M+(CH_3CN)+H]^+$ 431.0881, $[C_{13}H_{18}F_7N_2O_4S]^+$ requires 431.0870 (δ = + 2.6 ppm);

$[\alpha]_D^{25.0}$ = + 20.3 (c. 0.7, $CHCl_3$).

Conversion of **9e** *to the thiophenolate adduct for ee determination: 2,2,3,3,3-pentafluoropropyl (R)-(4,4-difluoro-1-(2-hydroxy-1-(phenylthio)ethyl)cyclohexyl)sulfamate* (**A**) and *2,2,3,3,3-pentafluoropropyl (R)-(1-(4,4-difluoro-1-(phenylthio)cyclohexyl)-2-hydroxyethyl)sulfamate* (**B**)

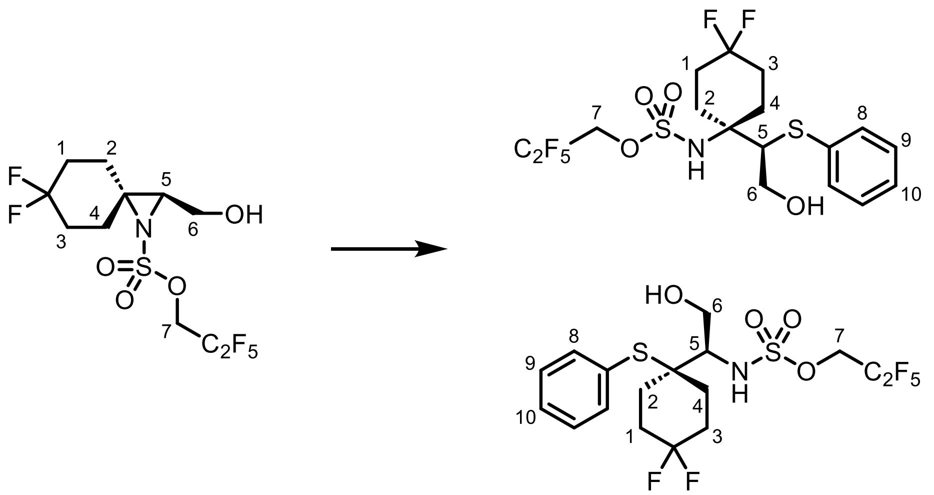

Prepared according to **GP13** on a 0.059 mmol scale with respect to 2,2,3,3,3-pentafluoropropyl (S)-6,6-difluoro-2-(hydroxymethyl)-1-azaspiro[2.5]octane-1-sulfonate (**9e**). Purification by flash column chromatography (SiO_2, 0-35% v/v EtOAc in hexane) afforded the 4.3:1 mixture of title compounds A:B as a colourless oil (21.9 mg, 0.044 mmol, 75%, 87% *ee*).

$\boldsymbol{R_f}$ **value**: 0.50 and 0.35 (30% v/v EtOAc in hexane);

<u>NMR data for major product **A**:</u>

^{1}H NMR (700 MHz, $CDCl_3$): δ 7.50-7.43 (m, 2.76H, H-8), 7.36-7.30 (m, 3H, H-9, H-10), 4.53 (t, J = 12.6 Hz, 2H, H-7), 4.04 (dd, J = 12.2, 4.4 Hz, 1H, H-6_A), 3.93 (dd, J = 12.3, 4.9 Hz, 1H, H-6_B), 3.63 (t, J = 4.5 Hz, 1H, H-5), 2.33-1.70 (m, 11.28H, 8 × C<u>H</u>, O-H) ppm;

^{19}F NMR (376 MHz, $CDCl_3$): δ – 84.5, – 96.5 (d, J = 244 Hz), – 101.1 (d, J = 234 Hz), – 124.3 ppm;

<u>NMR data for minor product **B**:</u>

^{1}H NMR (700 MHz, $CDCl_3$): δ 7.50-7.43 (m, 2.76H, H-8, H-10), 7.39 (m, 0.50H, H-9), 4.48 (t, J = 12.4 Hz, 0.50H, H-7), 4.26 (dd, J = 11.7, 4.0 Hz, 0.23H, H-6_A), 3.97 (dd, J = 11.5, 4.4 Hz, 0.23H, H-6_B), 3.49 (t, J = 4.2 Hz, 0.23H, H-5), 2.33-1.70 (m, 11.28H, 8 × C<u>H</u>, O-H) ppm;

^{19}F NMR (376 MHz, $CDCl_3$): δ – 84.5, – 94.9 (d, J = 237 Hz), – 101.5 (d, J = 234 Hz), – 124.5 ppm;

<u>Combined ^{13}C NMR data for the mixture of **A** and **B**:</u>

^{13}C NMR (176 MHz, $CDCl_3$): δ 136.9, 133.8, 132.6, 130.3, 129.7, 129.2, 128.4, 122.6 (t, J_{C-F} = 241 Hz), 121.9 (t, J_{C-F} = 242 Hz), 118.3 (qt, J_{C-F} = 286, 34.4 Hz), 111.5 (tq, J_{C-F} = 258, 39.0 Hz), 64.13 (t, J_{C-F} = 28.3 Hz), 64.09 (t, J_{C-F} = 27.9 Hz), 62.9, 62.2, 61.9, 61.0, 58.4, 55.4, 30.1-29.6 (m), 29.5 (t, J_{C-F} = 4.6 Hz), 29.2 (m), 28.8 (t, J_{C-F} = 4.3 Hz) ppm;

HRMS (+ESI) *m/z* Found [M–(OH)]$^+$ 482.0692, $[C_{17}H_{19}F_7NO_3S_2]^+$ requires 482.0689 (δ = + 0.6 ppm);

$[\alpha]_D^{25.0}$ = + 2.4 (c. 1.0, $CHCl_3$).

Chiral SFC Analysis: CHIRALPAK IG (CO_2:MeOH, 95.0:5.0, 2.50 mL min^{-1}, 40 °C) for major regioisomer A t_R = 6.1 (minor), 10.1 (major) minutes.

2,2,3,3,3-pentafluoropropyl (S)-2-(hydroxymethyl)-6-oxa-1-azaspiro[2.5]octane-1-sulfonate (**9f**)

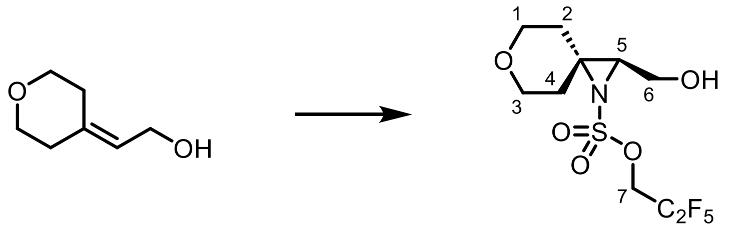

Prepared according to **GP12** using 2-(tetrahydro-4H-pyran-4-ylidene)ethan-1-ol as the starting material. Purification by flash column chromatography (SiO_2, 0-26% v/v Et_2O in CH_2Cl_2) afforded the title compound as a colourless oil (19.1 mg, 0.054 mmol, 54%).

***R*$_f$ value**: 0.18 (15% v/v Et_2O in CH_2Cl_2);

^{1}**H NMR** (500 MHz, $CDCl_3$): δ 4.69 (t, J = 12.4 Hz, 2H, H-7), 4.01 (m, 1H, C<u>H</u>O), 3.87 (m, 1H, C<u>H</u>O), 3.81 (m, 2H, H-6), 3.79-3.72 (m, 2H, 2 × C<u>H</u>O), 3.13 (t, J = 6.2 Hz, 1H, H-5), 2.24 (m, 1H, C<u>H</u>), 2.08 (m, 1H, C<u>H</u>), 1.87-1.75 (m, 2H, 2 × C<u>H</u>), 1.71 (br. t, J = 4.9 Hz, 1H, O-H) ppm;

^{13}**C NMR** (126 MHz, $CDCl_3$): δ 118.3 (qt, J_{C-F} = 286, 34.1 Hz), 111.4 (tq, J_{C-F} = 257, 38.9 Hz), 66.6, 66.5, 65.7 (t, J_{C-F} = 28.6 Hz), 59.5, 54.5, 53.1, 31.6, 31.5 ppm;

^{19}**F NMR** (376 MHz, $CDCl_3$): δ – 83.5, – 123.6 (m) ppm;

HRMS (+ESI) *m/z* Found $[M+(CH_3CN)+H]^+$ 397.0852, $[C_{12}H_{18}F_5N_2O_5S]^+$ requires 397.0851 (δ = + 0.3 ppm);

$[\alpha]_D^{25.0}$ = + 19.3 (c. 0.8, $CHCl_3$).

Conversion of **9f** *to the thiophenolate adduct for ee determination: 2,2,3,3,3-pentafluoropropyl (R)-(2-hydroxy-1-(4-(phenylthio)tetrahydro-2H-pyran-4-yl)ethyl)sulfamate* (**A**) and *2,2,3,3,3-pentafluoropropyl (R)-(4-(2-hydroxy-1-(phenylthio)ethyl)tetrahydro-2H-pyran-4-yl)sulfamate* (**B**)

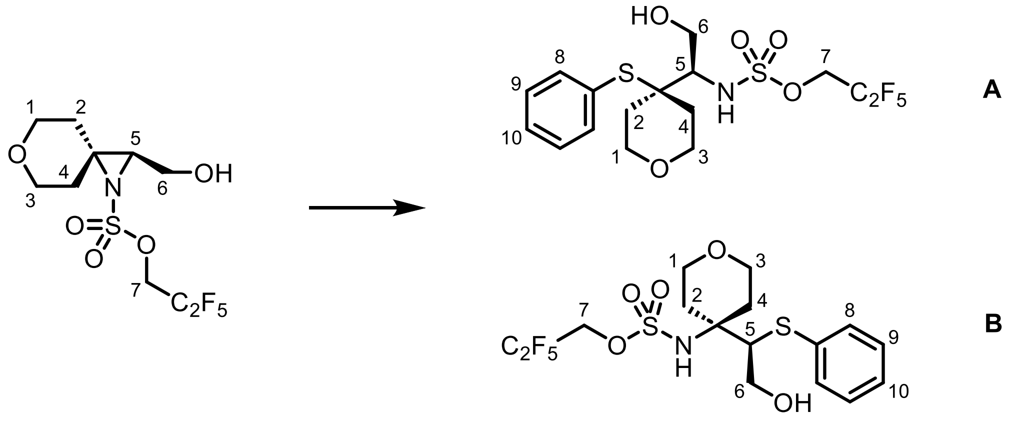

Prepared according to **GP13** on a 0.039 mmol scale with respect 2,2,3,3,3-pentafluoropropyl (S)-2-(hydroxymethyl)-6-oxa-1-azaspiro[2.5]octane-1-sulfonate (**9f**). Purification by flash column chromatography (SiO_2, 0-50% v/v EtOAc in hexane) afforded the 1.6:1 mixture of title compounds A:B as a colourless oil (13.9 mg, 0.030 mmol, 77%, 90% *ee*).

R_f **value**: 0.46 and 0.34 (50% v/v EtOAc in hexane);

<u>NMR data for major product **A**:</u>

^{1}**H NMR** (700 MHz, $CDCl_3$): δ 7.50 (m, 2H, H-8), 7.43 (m, 1H, H-10), 7.38 (m, 2H, H-9), 5.50 (br. s, 1H, N-H), 4.49 (t, J = 12.4 Hz, 2H, H-7), 4.17 (m, 1H, H-6$_A$), 4.04-3.99 (m, 1.62H, C<u>H</u>O), 3.98-3.91 (m, 2.68H, H-6$_B$, C<u>H</u>O), 3.84-3.76 (m, 3.28H, 2 × C<u>H</u>O), 3.57 (t, J = 4.2 Hz, 1H, H-5), 2.01 (m, 1H, C<u>H</u>), 1.91 (m, 1H, C<u>H</u>), 1.69 (m, 1H, C<u>H</u>), 1.62 (m, 1H, C<u>H</u>) ppm;

^{19}F NMR (376 MHz, $CDCl_3$): δ – 83.6, – 123.5 (m) ppm;

NMR data for minor product **B**:

^{1}H NMR (700 MHz, $CDCl_3$): δ 7.47 (m, 1.29H, H-8), 7.34 (m, 1.23H, H-9), 7.30 (m, 0.64H, H-10), 5.79 (br. s, 0.60H, N-H), 4.54 (m, 1.26H, H-7), 4.04-3.99 (m, 1.62H, H-6_A), 3.98-3.91 (m, 2.68H, H-6_B), 3.84-3.76 (m, 3.28H, 2 × C<u>H</u>O), 3.74 (t, *J* = 4.6 Hz, 0.64H, H-5), 3.73-3.66 (m, 1.29H, 2 × C<u>H</u>O), 2.28 (m, 0.69H, C<u>H</u>), 2.22-2.14 (m, 1.30H, 2 × C<u>H</u>), 2.10 (m, 0.67H, C<u>H</u>) ppm;

^{19}F NMR (376 MHz, $CDCl_3$): δ – 83.5, – 123.3 (m) ppm;

Combined ^{13}C NMR data for the mixture of **A** and **B**:

^{13}C NMR (176 MHz, $CDCl_3$): δ 137.3, 134.0, 132.4, 130.1, 129.7, 129.4, 128.2, 118.4 (qt, J_{C-F} = 286, 34.4 Hz), 111.5 (tq, J_{C-F} = 256, 38.8 Hz), 64.1 (t, J_{C-F} = 28.3 Hz), 64.0 (t, J_{C-F} = 27.8 Hz), 63.64, 63.62, 63.5, 63.44, 63.43, 62.1, 61.6, 61.0, 59.1, 54.4, 33.1, 33.0, 32.8, 32.6 ppm;

Note: there is an additional ^{13}C resonance underneath the peak at 129.7 ppm.

HRMS (+ESI) *m/z* Found $[M+H]^+$ 466.0781, $[C_{16}H_{21}F_5NO_5S_2]^+$ requires 466.0776 (δ = + 1.1 ppm);

$[\alpha]_D^{25.0}$ = + 2.1 (c. 0.9, $CHCl_3$).

Chiral SFC Analysis: CHIRALPAK IG (CO_2:MeOH, 94.0:6.0, 2.50 mL min^{-1}, 40 °C) for major regioisomer A t_R = 5.7 (major), 6.2 (minor) minutes.

2,2,3,3,3-pentafluoropropyl (S)-2-(hydroxymethyl)-7,10-dioxa-1-azadispiro[2.2.4^6.2^3]dodecane-1-sulfonate (**9g**)

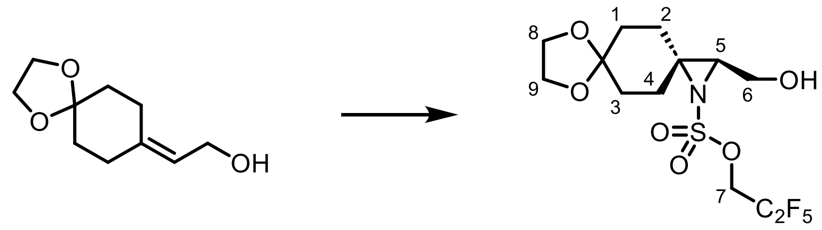

Prepared according to **GP12** using 2-(1,4-dioxaspiro[4.5]decan-8-ylidene)ethan-1-ol as the starting material. Purification by flash column chromatography (SiO_2, 0-15% v/v Et_2O in CH_2Cl_2) afforded the title compound as a colourless oil (29.5 mg, 0.072 mmol, 72%).

***R_f* value**: 0.24 (10% v/v Et_2O in CH_2Cl_2);

^{1}H NMR (500 MHz, $CDCl_3$): δ 4.67 (t, *J* = 12.5 Hz, 2H, H-7), 4.00-3.94 (m, 4H, H-8, H-9), 3.79 (m, 2H, H-6), 3.11 (dd, *J* = 6.9, 5.7 Hz, 1H, H-5), 2.25 (m, 1H, H-2_A), 2.11 (m, 1H, H-2_B), 2.02 (m, 1H, C<u>H</u>), 1.92-1.71 (m, 5H, H-4, 3 × C<u>H</u>), 1.63 (t, *J* = 5.6 Hz, 1H, O-H) ppm;

^{13}C NMR (176 MHz, $CDCl_3$): δ 118.3 (qt, J_{C-F} = 286, 34.5 Hz), 111.4 (tq, J_{C-F} = 257, 38.6 Hz), 107.5, 65.6 (t, J_{C-F} = 28.5 Hz), 64.7, 64.6, 59.8, 56.0, 53.2, 33.4, 33.3, 28.3, 28.2 ppm;

^{19}F NMR (376 MHz, $CDCl_3$): δ – 83.5, – 123.6 (m) ppm;

HRMS (–ESI) *m/z* Found $[M+(HCO_2)]^-$ 456.0750, $[C_{14}H_{19}F_5NO_8S]^-$ requires 456.0757 (δ = – 1.5 ppm);

$[\alpha]_D^{25.0}$ = + 22.1 (c. 0.8, $CHCl_3$).

Conversion of **9g** *to the thiophenolate adduct for ee determination: 2,2,3,3,3-pentafluoropropyl (R)-(2-hydroxy-1-(8-(phenylthio)-1,4-dioxaspiro[4.5]decan-8-yl)ethyl)sulfamate* (**A**) and *2,2,3,3,3-pentafluoropropyl (R)-(8-(2-hydroxy-1-(phenylthio)ethyl)-1,4-dioxaspiro[4.5]decan-8-yl)sulfamate* (**B**)

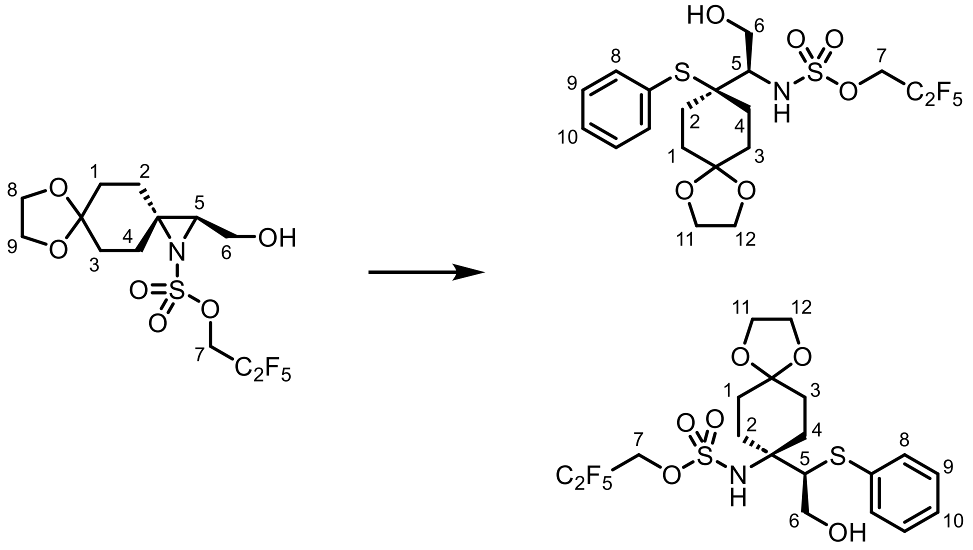

A

B

Prepared according to **GP13** on a 0.043 mmol scale with respect to 2,2,3,3,3-pentafluoropropyl (S)-2-(hydroxymethyl)-7,10-dioxa-1-azadispiro[2.2.46.23]dodecane-1-sulfonate (**9g**). Purification by flash column chromatography (SiO_2, 0-20% v/v acetone in $CHCl_3$) afforded the 1:1 mixture of title compounds A:B as a colourless oil (17.7 mg, 0.034 mmol, 79%, 94% *ee*).

***R_f* value**: 0.46 (20% v/v acetone in $CHCl_3$);

NMR data for product **A**:

^{1}H NMR (700 MHz, $CDCl_3$): δ 7.51 (m, 2H, H-8), 7.41 (m, 1H, H-10), 7.36 (m, 2H, H-9), 5.48 (br. s, 1H, N-H), 4.60-4.44 (m, 4H, H-7), 4.12 (dt, *J* = 11.6, 4.6 Hz, 1H, H-6_A), 3.97-3.88 (m, 10H, H-6_B, H-11, H-12), 3.52 (br. t, *J* = 3.9 Hz, 1H, H-5), 2.23-2.17 (m, 2H, O-H), 2.11 (td, *J* = 12.2, 3.9 Hz, 1H, C<u>H</u>), 2.04 (td, *J* = 12.7, 3.6 Hz, 1H, C<u>H</u>), 1.99 (m, 1H, C<u>H</u>), 1.88 (m, 1H, C<u>H</u>), 1.83-1.77 (m, 3H, C<u>H</u>), 1.73 (m, 1H, C<u>H</u>), 1.70-1.63 (m, 2H, 2 × C<u>H</u>) ppm;

NMR data for product **B**:

^{1}H NMR (700 MHz, $CDCl_3$): δ 7.48 (m, 2H, H-8), 7.34-7.28 (m, 3H, H-9, H-10), 5.85 (br. s, 1H, N-H), 4.60-4.44 (m, 4H, H-7), 4.02 (m, 1H, H-6_A), 3.97-3.88 (m, 10H, H-6_B, H-11, H-12), 3.65 (t, *J* = 4.1 Hz, 1H, H-5), 2.42 (m, 1H, O-H), 2.35 (m, 1H, C$\underline{H}$), 2.31-2.24 (m, 2H, 2 × C$\underline{H}$), 2.23-2.17 (m, 2H, C$\underline{H}$), 1.83-1.77 (m, 3H, 2 × C$\underline{H}$), 1.60-1.53 (m, 2H, 2 × C$\underline{H}$) ppm;

Combined ^{19}F and ^{13}C NMR data for the mixture of **A** and **B**:

^{13}C NMR (176 MHz, $CDCl_3$): δ 136.9, 134.1, 132.7, 129.9, 129.7, 129.64, 129.55, 128.2, 118.3 (qtd, J_{C-F} = 286, 33.6, 6.8 Hz), 111.6 (tqd, J_{C-F} = 256, 38.9, 10.0 Hz), 108.0, 107.3, 64.7, 64.62, 64.57, 64.4, 64.1 (t, J_{C-F} = 28.1 Hz), 63.4, 63.3, 61.7, 61.5, 58.1, 56.0, 31.01, 30.94, 30.90, 30.82, 30.78, 30.64, 30.58, 29.7 ppm;

^{19}F NMR (376 MHz, $CDCl_3$): δ – 83.5, – 83.6, – 123.2, – 123.5 ppm;

HRMS (+ESI) *m/z* Found $[M+H]^+$ 522.1041, $[C_{19}H_{25}F_5NO_6S_2]^+$ requires 522.1038 (δ = + 0.6 ppm);

$[\alpha]_D^{25.0}$ = – 15.3 (c. 1.2, $CHCl_3$).

Chiral SFC Analysis: CHIRALPAK IG (CO_2:MeOH, 90.0:10.0, 1.25 mL min^{-1}, 40 °C) for one regioisomer t_R = 9.1 (minor), 12.9 (major) minutes.

2,2,3,3,3-pentafluoropropyl (2S,3R)-2-(hydroxymethyl)-3-propylaziridine-1-sulfonate (**9h**)

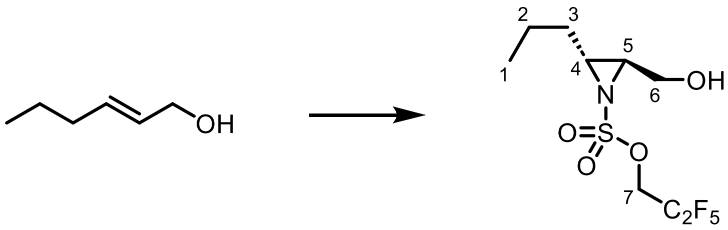

Prepared according to **GP12** using (*E*)-hex-2-en-1-ol as the starting material. Purification by flash column chromatography (SiO_2, 0-5% v/v acetone in $CHCl_3$) afforded the title compound as a colourless oil (5.9 mg, 0.018 mmol, 18%).

***R$_f$* value**: 0.34 (5% v/v acetone in $CHCl_3$);

^{1}H NMR (400 MHz, $CDCl_3$): δ 4.68 (m, 2H, H-7), 4.06 (m, 1H, H-6_A), 3.83 (m, 1H, H-6_B), 3.01-2.91 (m, 2H, H-4, H-5), 2.07 (t, *J* = 6.7 Hz, 1H, O-H), 1.78 (m, 1H, H-3_A), 1.65 (m, 1H, H-3_B), 1.51 (m, 2H, H-2), 0.98 (t, *J* = 7.3 Hz, 3H, H-1) ppm;

^{13}C NMR (176 MHz, $CDCl_3$): δ 118.3 (qt, J_{C-F} = 286, 34.2 Hz), 111.3 (tq, J_{C-F} = 257, 38.8 Hz), 65.7 (t, J_{C-F} = 28.5 Hz), 60.6, 51.1, 47.3, 31.5, 20.2, 13.7 ppm;

^{19}F NMR (376 MHz, $CDCl_3$): δ – 83.5, – 123.6 ppm;

HRMS (–ESI) *m/z* Found $[M+(HCO_2)]^-$ 372.0531, $[C_{10}H_{15}F_5NO_6S]^-$ requires 372.0546 (δ = – 4.0 ppm);

$[\alpha]_D^{25.0}$ = + 15.8 (c. 0.3, $CHCl_3$).

Conversion of **9h** *to the thiophenolate adduct for ee determination: 2,2,3,3,3-pentafluoropropyl ((2R,3S)-1-hydroxy-3-(phenylthio)hexan-2-yl)sulfamate*

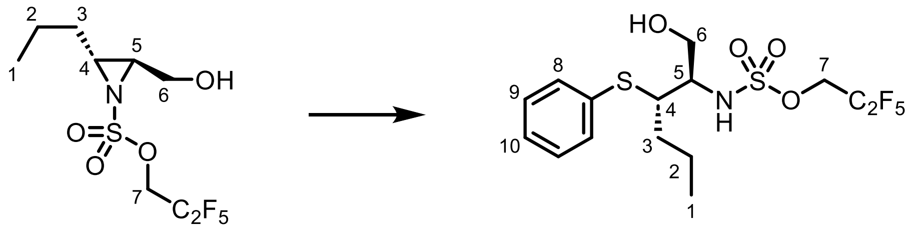

Prepared according to **GP13** on a 0.012 mmol scale with respect to 2,2,3,3,3-pentafluoropropyl (2S,3R)-2-(hydroxymethyl)-3-propylaziridine-1-sulfonate (**9h**). Purification by flash column chromatography (SiO_2, 0-8% v/v acetone in $CHCl_3$) afforded the title compound as a colourless oil (4.7 mg, 0.011 mmol, 90%, 83% *ee*).

***R_f* value**: 0.28 (5% v/v acetone in $CHCl_3$);

^{1}H NMR (700 MHz, $CDCl_3$): δ 7.45 (m, 2H, H-8), 7.33 (m, 2H, H-9), 7.28 (m, 1H, H-10), 5.41 (br. s, 1H, N-H), 4.50 (q, *J* = 12.4 Hz, 1H, H-7_A), 4.43 (q, *J* = 12.2 Hz, 1H, H-7_B), 3.94 (m, 1H, H-6_A), 3.81 (m, 1H, H-6_B), 3.69 (q, *J* = 4.6 Hz, 1H, H-5), 3.38 (m, 1H, H-4), 1.78-1.72 (m, 2H, H-3_A, O-H), 1.69-1.58 (m, 2H, H-2_A, H-3_B), 1.46 (m, 1H, H-2_B), 0.93 (t, *J* = 7.3 Hz, 3H, H-1) ppm;

^{13}C NMR (176 MHz, $CDCl_3$): δ 134.8, 132.0, 129.6, 127.9, 118.4 (qt, J_{C-F} = 285, 34.5 Hz), 111.5 (tq, J_{C-F} = 257, 38.1 Hz), 64.2 (t, J_{C-F} = 28.0 Hz), 61.6, 59.5, 53.3, 35.0, 20.7, 13.8 ppm;

^{19}F NMR (376 MHz, $CDCl_3$): δ – 83.5, – 123.5 (t, *J* = 12.5 Hz) ppm;

HRMS (+ESI) *m/z* Found $[M+H]^+$ 438.0812, $[C_{15}H_{21}F_5NO_4S_2]^+$ requires 438.0827 (δ = – 3.4 ppm);

$[\alpha]_D^{25.0}$ = + 3.5 (c. 0.4, $CHCl_3$).

Chiral SFC Analysis: CHIRALPAK IG (CO_2:MeOH, 95.0:5.0, 2.50 mL min^{-1}, 40 °C) t_R = 3.8 (minor), 4.6 (major) minutes.

2,2,3,3,3-pentafluoropropyl (R)-(2-hydroxy-1-(1-(phenylthio)cyclopentyl)ethyl)sulfamate (**10a**)

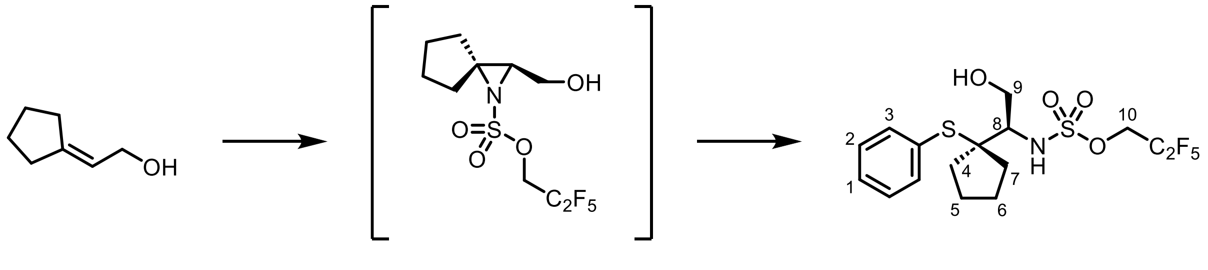

Prepared according to **GP12** using 2-cyclopentylideneethan-1-ol as the starting material. Purification by flash column chromatography (SiO_2, 0-8% v/v acetone in $CHCl_3$) afforded the intermediate aziridine. In this case, additional degradation side-products were observed after purification. The semi-purified aziridine was then subjected to thiophenol ring-opening.

The thiophenol adduct was prepared according to **GP13**. Purification by flash column chromatography (SiO_2, 0-5% v/v Et_2O in CH_2Cl_2) afforded the title compound as a colourless oil (15.3 mg, 0.034 mmol, 34% over 2 steps, 92% *ee*).

***Rf* value**: 0.50 (5% v/v Et_2O in CH_2Cl_2);

^{1}H NMR (500 MHz, $CDCl_3$): δ 7.54 (m, 2H, H-3), 7.43-7.35 (m, 3H, H-1, H-2), 5.41 (br. s, 1H, N-H), 4.45 (m, 2H, H-10), 4.03 (dt, *J* = 12.0, 4.8 Hz, 1H, H-9$_A$), 3.83 (m, 1H, H-9$_B$), 3.54 (br. s, 1H, H-8), 2.27 (dd, *J* = 7.9, 4.6 Hz, 1H, O-H), 2.00-1.72 (m, 8H, H-4, H-5, H-6, H-7) ppm;

^{13}C NMR (126 MHz, $CDCl_3$): δ 136.8, 131.5, 129.8, 129.5, 118.4 (qt, J_{C-F} = 287, 34.4 Hz), 111.5 (tq, J_{C-F} = 256, 38.9 Hz), 65.0, 64.6, 64.1 (t, J_{C-F} = 28.4 Hz), 37.0, 36.9, 24.0, 23.5 ppm;

^{19}F NMR (376 MHz, $CDCl_3$): δ – 84.5, – 124.5 ppm;

HRMS (+ESI) *m/z* Found $[M+H]^+$ 450.0822, $[C_{16}H_{21}F_5NO_4S_2]^+$ requires 450.0827 (δ = – 1.1 ppm);

$[\alpha]_D^{25.0}$ = + 7.2 (c. 0.4, $CHCl_3$).

Chiral SFC Analysis: CHIRALPAK IG (CO_2:MeOH, 96.0:4.0, 2.50 mL min^{-1}, 40 °C) t_R = 12.6 (major), 13.3 (minor) minutes.

2,2,3,3,3-pentafluoropropyl (R)-(2-hydroxy-1-(1-(phenylthio)cycloheptyl)ethyl)sulfamate (**10b**)

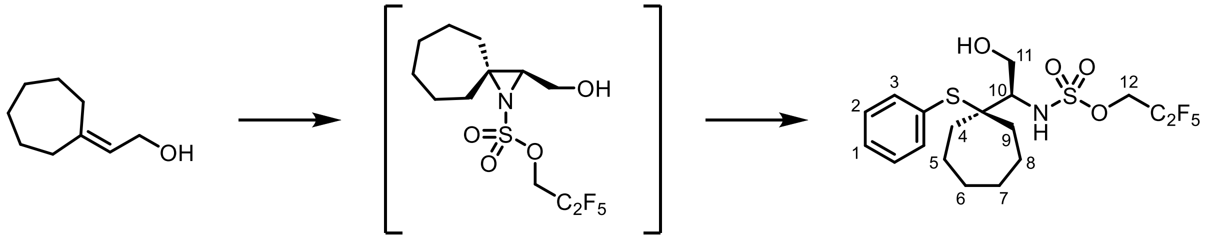

Prepared according to **GP12** using 2-cycloheptylideneethan-1-ol as the starting material. Purification by flash column chromatography (SiO_2, 0-6% v/v acetone in $CHCl_3$) afforded the intermediate aziridine. In this case, additional degradation side-products were observed after purification. The semi-purified aziridine was then subjected to thiophenol ring-opening.

The thiophenol adduct was prepared according to **GP13**. Purification by flash column chromatography (SiO_2, 0-5% v/v acetone in $CHCl_3$) afforded the title compound as a colourless oil (26.4 mg, 0.055 mmol, 55% over 2 steps, 91% *ee*).

***Rf* value**: 0.44 (5% v/v acetone in $CHCl_3$);

^{1}**H NMR** (700 MHz, $CDCl_3$): δ 7.54 (m, 2H, H-3), 7.41 (m, 1H, H-1), 7.36 (m, 2H, H-2), 5.54 (br. s, 1H, N-H), 4.47 (m, 2H, H-12), 3.97 (dt, J = 12.2, 4.6 Hz, 1H, H-11$_A$), 3.84 (ddd, J = 12.1, 8.1, 4.0 Hz, H-11$_B$), 3.55 (br. t, J = 4.4 Hz, 1H, H-10), 2.37 (dd, J = 8.2, 4.4 Hz, 1H, O-H), 1.97 (dd, J = 15.0, 8.8 Hz, 1H, C$\underline{H}$), 1.92 (dd, J = 15.0, 9.2 Hz, 1H, C$\underline{H}$), 1.78-1.58 (m, 7H, 7 × C$\underline{H}$), 1.48-1.39 (m, 3H, 3 × C$\underline{H}$) ppm;^{13}**C NMR** (176 MHz, $CDCl_3$): δ 137.3, 130.5, 129.9, 129.4, 118.3 (qt, J_{C-F} = 286, 34.2 Hz), 111.5 (tq, J_{C-F} = 256, 38.4 Hz), 64.1 (t, J_{C-F} = 28.0 Hz), 63.8, 61.8, 60.2, 37.8, 37.0, 30.5, 30.4, 23.0 ppm;

Note: there is an additional ^{13}C resonance underneath the peak at 23.0 ppm.

^{19}**F NMR** (376 MHz, $CDCl_3$): δ – 84.5, – 124.5 ppm;

HRMS (+ESI) *m/z* Found $[M+H]^+$ 478.1142, $[C_{18}H_{25}F_5NO_4S_2]^+$ requires 478.1140 (δ = + 0.4 ppm);

$[\alpha]_D^{25.0}$ = + 11.5 (c. 0.6, $CHCl_3$).

Chiral SFC Analysis: CHIRALPAK IG (CO_2:MeOH, 95.0:5.0, 2.50 mL min^{-1}, 40 °C) t_R = 7.5 (major), 8.3 (minor) minutes.

2,2,3,3,3-pentafluoropropyl (2R,3S)-3-(hydroxymethyl)-2-methyl-2-(4-methylpent-3-en-1-yl)aziridine-1-sulfonate (**9i**)

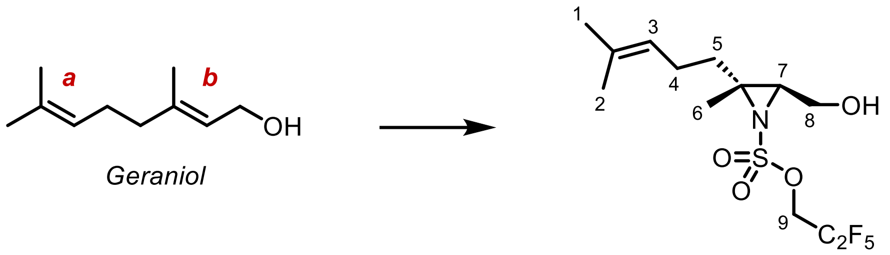

Prepared according to **GP12** using geraniol as the starting material. In this case the following procedural modification was employed: 1.05 equivalents of sulfamate ester were used. Purification by flash column chromatography (SiO_2, 0-1.5% v/v Et_2O in CH_2Cl_2) afforded the title compound as a colourless oil (14.2 mg, 0.037 mmol, 37%).

***R$_f$* value**: 0.51 (5% v/v Et_2O in CH_2Cl_2);

^{1}**H NMR** (500 MHz, $CDCl_3$): δ 5.09 (m, 1H, H-3), 4.65 (t, J = 12.4 Hz, 2H, H-9), 3.76 (m, 2H, H-8), 3.07 (dd, J = 6.9, 5.7 Hz, 1H, H-7), 2.26 (m, 1H, H-4$_A$), 2.20-2.04 (m, 2H, H-4$_B$, H-5$_A$), 1.81 (m, 1H, H-5$_B$), 1.70 (s, 3H, H-1), 1.62 (s, 3H, H-2), 1.38 (s, 3H, H-6) ppm;

^{13}**C NMR** (126 MHz, $CDCl_3$): δ 133.4, 122.4, 118.4 (qt, J_{C-F} = 286, 34.8 Hz), 111.5 (tq, J_{C-F} = 257, 39.1 Hz), 65.5 (t, J_{C-F} = 28.2 Hz), 60.2, 55.0, 54.0, 34.8, 25.8, 25.1, 17.9, 17.8 ppm;

^{19}**F NMR** (376 MHz, $CDCl_3$): δ – 83.5, – 123.6 (t, J = 12.4 Hz) ppm;

HRMS (–ESI) *m/z* Found $[M+OH]^-$ 398.1059, $[C_{13}H_{21}F_5NO_5S]^-$ requires 398.1066 (δ = – 1.8 ppm);

$[\alpha]_D^{25.0}$ = + 6.6 (c. 0.4, $CHCl_3$).

Site-selectivity analysis for the aziridination of geraniol:

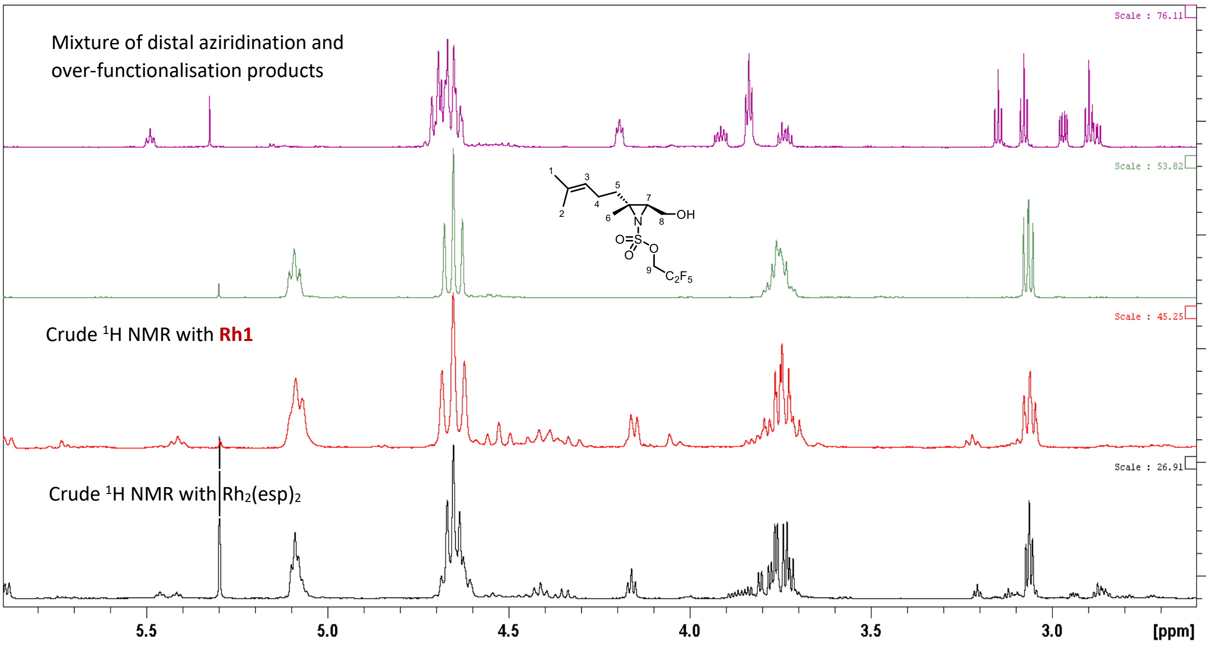

Supplementary Figure 2. ^{1}H NMR spectra comparison.

- In the crude ^{1}H NMR spectrum for the reaction with $Rh_2(esp)_2$ as a catalyst (black spectrum), a mixture of mono-aziridination isomers and over-functionalisation products is observed
- In contrast, the crude ^{1}H NMR spectrum for the reaction with **Rh1** as a catalyst (red spectrum) shows much greater selectivity for the desired aziridine **9i** (a purified ^{1}H NMR is shown in the green spectrum)
- The ^{1}H NMR for a semi-purified sample of a mixture of mono-aziridination at the distal site and over-functionalisation products is shown in the purple spectrum. The peak at 5.49 ppm was assigned to the alkene CH in the mono-aziridination minor product
- The alkene peak at 5.49 ppm for distal aziridination and aziridine CH peak at 3.06 ppm for proximal aziridination are used to provide a site-selectivity ratio from the crude ^{1}H NMR spectra. **This ratio was 16:1 in favour of 9i for Rh1 and 8.5:1 for $Rh_2(esp)_2$**
- The analysis is complicated by the formation of over-functionalisation products. As well as a higher site-selectivity ratio with **Rh1** (red spectrum), it is also clear that lower amounts of over-functionalised products are observed with **Rh1** (compare the region of 3.2-2.8 ppm in black and red spectra)

Conversion of **9i** *to the thiophenolate adduct for ee determination: 2,2,3,3,3-pentafluoropropyl ((2R,3S)-1-hydroxy-3,7-dimethyl-3-(phenylthio)oct-6-en-2-yl)sulfamate*

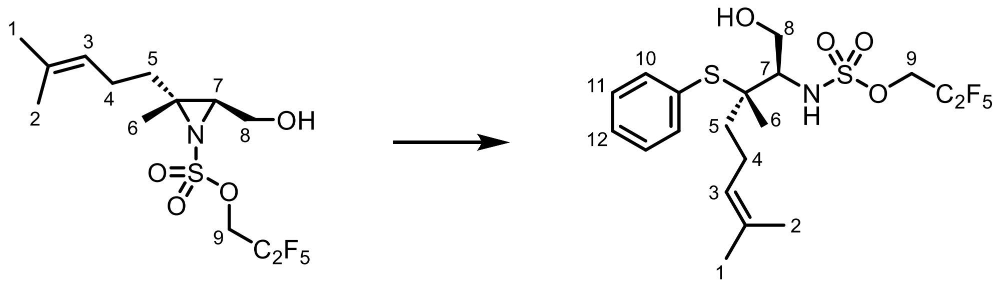

Prepared according to **GP13** on a 0.021 mmol scale with respect to 2,2,3,3,3-pentafluoropropyl (2R,3S)-3-(hydroxymethyl)-2-methyl-2-(4-methylpent-3-en-1-yl)aziridine-1-sulfonate (**9i**). Purification by flash column chromatography (SiO_2, 0-2% v/v Et_2O in CH_2Cl_2) afforded the title compound as a colourless oil (8.9 mg, 0.018 mmol, 85%, 83% *ee*).

***R_f* value**: 0.58 (5% v/v Et_2O in CH_2Cl_2);

^{1}H NMR (700 MHz, $CDCl_3$): δ 7.52 (m, 2H, H-10), 7.41 (m, 1H, H-12), 7.36 (m, 2H, H-11), 5.55 (d, J = 5.1 Hz, 1H, N-H), 5.05 (m, 1H, H-3), 4.53 (m, 2H, H-9), 4.00 (m, 1H, H-8$_A$), 3.90 (m, 1H, H-8$_B$), 3.56 (br. s, 1H, H-7), 2.20 (m, 1H, H-4$_A$), 2.15 (br. t, J = 5.5 Hz, 1H, O-H), 2.07 (m, 1H, H-4$_B$), 1.76 (m, 1H, H-5$_A$), 1.69 (s, 3H, H-1), 1.65 (m, 1H, H-5$_B$), 1.62 (s, 3H, H-2), 1.27 (s, 3H, H-6) ppm;

^{13}C NMR (176 MHz, $CDCl_3$): δ 137.5, 132.8, 129.9, 129.8, 129.3, 123.2, 118.4 (qt, J_{C-F} = 286, 34.5 Hz), 111.6 (tq, J_{C-F} = 256, 38.5 Hz), 64.2 (t, J_{C-F} = 27.7 Hz), 63.3, 61.5, 55.9, 39.2, 25.8, 24.0, 23.1, 17.9 ppm;

^{19}F NMR (376 MHz, $CDCl_3$): δ – 83.6, – 123.5 (t, J = 12.5 Hz) ppm;

HRMS (+ESI) *m/z* Found $[M+H]^+$ 492.1274, $[C_{19}H_{27}F_5NO_4S_2]^+$ requires 492.1296 (δ = – 4.5 ppm);

$[\alpha]_D^{25.0}$ = + 22.0 (c. 0.3, $CHCl_3$).

Chiral SFC Analysis: CHIRALPAK IG (CO_2:MeOH, 95.0:5.0, 2.50 mL min^{-1}, 40 °C) t_R = 4.3 (major), 6.1 (minor) minutes.

2,2,3,3,3-pentafluoropropyl (2S,3S)-3-(hydroxymethyl)-2-methyl-2-(4-methylpent-3-en-1-yl)aziridine-1-sulfonate (**9j**)

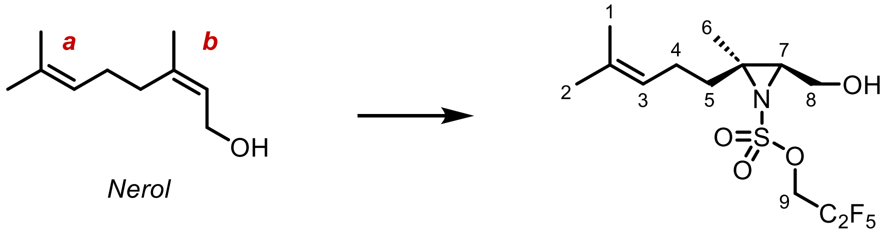

Prepared according to **GP12** using nerol as the starting material. In this case the following procedural modification was employed: 1.05 equivalents of sulfamate ester were used. Purification by flash column chromatography (SiO_2, 0-5% v/v acetone in $CHCl_3$) afforded the title compound as a pale-yellow oil (20.0 mg, 0.052 mmol, 52%).

***R**$_f$* **value**: 0.54 (10% v/v acetone in $CHCl_3$);

^{1}H NMR (700 MHz, $CDCl_3$): δ 5.09 (m, 1H, H-3), 4.66 (t, J = 12.4 Hz, 2H, H-9), 3.81 (m, 1H, H-8$_A$), 3.71 (m, 1H, H-8$_B$), 3.08 (dd, J = 7.1, 5.5 Hz, 1H, H-7), 2.15 (q, J = 7.8 Hz, 2H, H-4), 1.69 (s, 3H, C<u>H</u>$_3$), 1.68-1.63 (m, 5H, H-5$_A$, H-6, O-H), 1.62 (s, 3H, H-C<u>H</u>$_3$), 1.55 (m, 1H, H-5$_B$) ppm;

^{13}C NMR (176 MHz, $CDCl_3$): δ 133.3, 122.6, 118.4 (qt, J_{C-F} = 287, 34.6 Hz), 111.4 (tq, J_{C-F} = 257, 38.7 Hz), 65.4 (t, J_{C-F} = 28.4 Hz), 60.0, 54.9, 54.8, 34.5, 25.8, 24.2, 18.5, 17.8 ppm;

^{19}F NMR (376 MHz, $CDCl_3$): δ – 84.5, – 124.6 ppm;

HRMS (+ESI) *m/z* Found $[M+H]^+$ 382.1108, $[C_{13}H_{21}F_5NO_4S]^+$ requires 382.1106 (δ = + 0.5 ppm);

$[\alpha]_D^{25.0}$ = + 32.1 (c. 1.1, $CHCl_3$).

Site-selectivity analysis for the aziridination of nerol:

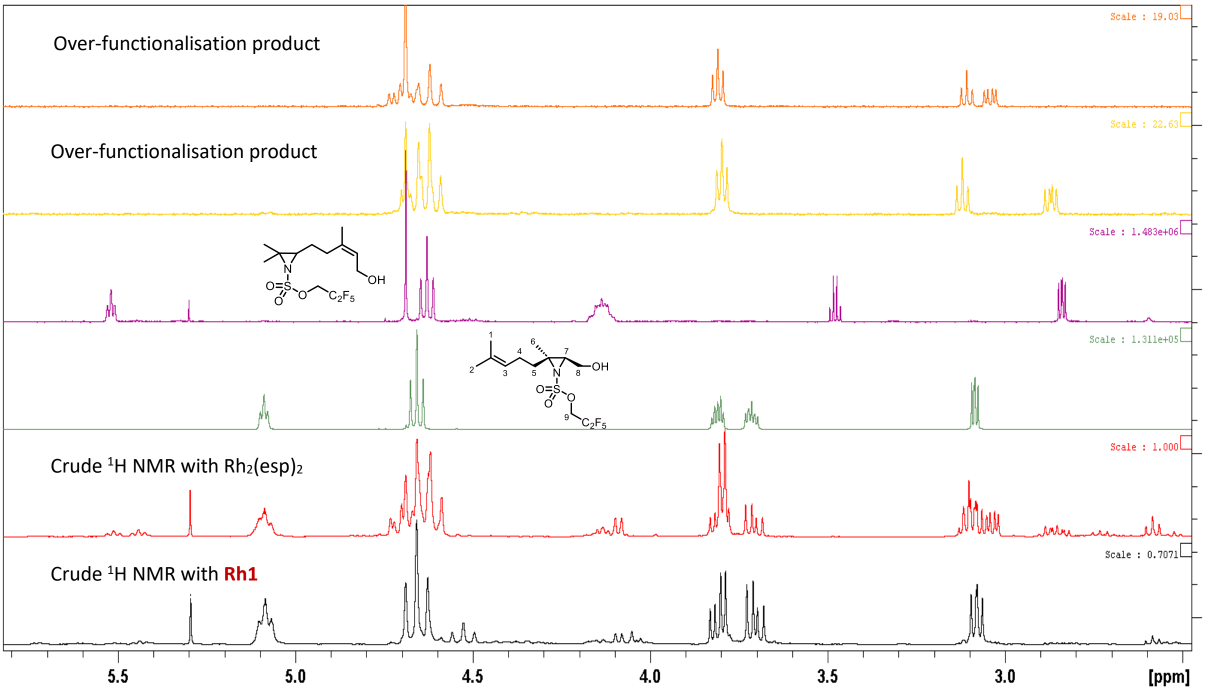

Supplementary Figure 3. ^{1}H NMR spectra comparison.

- In the crude ^{1}H NMR spectrum for the reaction with $Rh_2(esp)_2$ as a catalyst (red spectrum), a mixture of mono-aziridination isomers and over-functionalisation products is observed
- In contrast, the crude ^{1}H NMR spectrum for the reaction with **Rh1** as a catalyst (black spectrum) shows much greater selectivity for the desired aziridine **9j** (a purified ^{1}H NMR is shown in the green spectrum)

- The ¹H NMR for a semi-purified sample of mono-aziridination at the distal site is shown in the purple spectrum. *NMR data for this compound is presented below*
- The alkene peak at 5.52 ppm for distal aziridination and C<u>H</u>$_2$OH peak at 3.70 ppm for proximal aziridination are used to provide a site-selectivity ratio from the crude ¹H NMR spectra. **This ratio was 18:1 in favour of 9j for Rh1 and 3.6:1 for $Rh_2(esp)_2$**
- The analysis is complicated by the formation of over-functionalisation products (tentatively assigned as double-aziridination diastereomers, orange and yellow spectra). As well as a higher site-selectivity ratio with **Rh1** (black spectrum), it is also clear that lower amounts of over-functionalised products are observed with **Rh1** (compare the region of 3.0-3.1 ppm in black and red spectra)

*NMR data for a semi-purified sample of minor aziridine product, arising from the aziridination of nerol at alkene **a** with $Rh_2(esp)_2$: 2,2,3,3,3-pentafluoropropyl (Z)-3-(5-hydroxy-3-methylpent-3-en-1-yl)-2,2-dimethylaziridine-1-sulfonate*

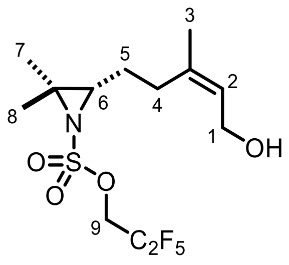

(racemate)

This compound was isolated alongside **9j** when running the racemic reaction with $Rh_2(esp)_2$. This compound could not be fully purified, and as such, impurities are clearly visible in the ¹H and ¹³C NMR spectra. The NMR data presented below is based on an assignment from the semi-purified sample.

¹H NMR (700 MHz, $CDCl_3$): δ 5.52 (t, *J* = 7.1 Hz, 1H, H-2), 4.63 (t, *J* = 12.4 Hz, 2H, H-9), 4.14 (m, 2H, H-1), 2.84 (dd, *J* = 7.8, 5.5 Hz, 1H, H-6), 2.26 (m, 1H, H-4$_A$), 2.21 (m, 1H, H-4$_B$), 1.76 (m, 3H, H-3), 1.67 (m, 2H, H-5), 1.62 (s, 3H, C<u>H</u>$_3$), 1.39 (t, *J* = 5.0 Hz, 1H, O-H), 1.34 (s, 3H, C<u>H</u>$_3$) ppm;

¹³C NMR (176 MHz, $CDCl_3$): δ 137.6, 126.1, 63.4 (t, $J_{C\text{-}F}$ = 28.3 Hz), 58.9, 54.6, 52.0, 29.1, 26.4, 23.3, 21.3, 20.6 ppm;

Note: the ¹³C resonances attributed to the fluorinated carbon atoms in this product were not detected.

¹⁹F NMR (376 MHz, $CDCl_3$): δ – 84.5, – 124.5 ppm.

*Conversion of **9j** to the thiophenolate adduct for ee determination: 2,2,3,3,3-pentafluoropropyl ((2R,3R)-1-hydroxy-3,7-dimethyl-3-(phenylthio)oct-6-en-2-yl)sulfamate* (**A**) and *2,2,3,3,3-pentafluoropropyl ((2R,3S)-1-hydroxy-3,7-dimethyl-2-(phenylthio)oct-6-en-3-yl)sulfamate* (**B**)

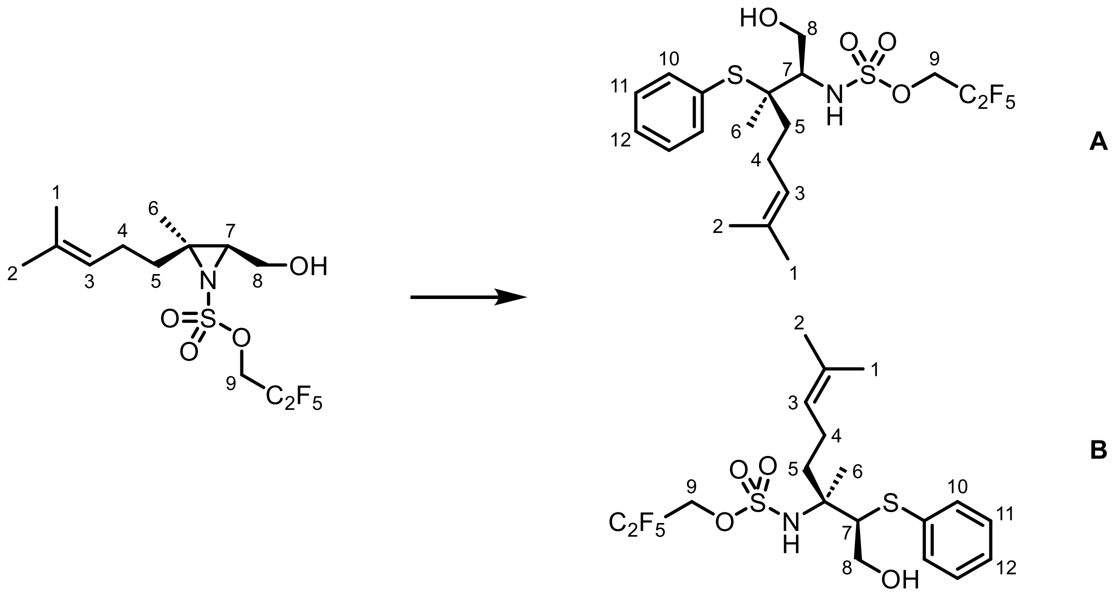

Prepared according to **GP13** on a 0.037 mmol scale with respect to 2,2,3,3,3-pentafluoropropyl (2S,3S)-3-(hydroxymethyl)-2-methyl-2-(4-methylpent-3-en-1-yl)aziridine-1-sulfonate (**9j**). Purification by flash column chromatography (SiO_2, 0-4% v/v acetone in $CHCl_3$) afforded the 6:1 mixture of title compounds A:B as a colourless oil (14.8 mg, 0.030 mmol, 81%, 84% *ee*).

***R_f* value**: 0.57 (5% v/v acetone in $CHCl_3$);

NMR data for product **A**:

^{1}H NMR (700 MHz, $CDCl_3$): δ 7.51 (m, 2H, H-10), 7.41 (m, 1H, H-12), 7.35 (m, 2H, H-11), 5.47 (br. s, 1H, N-H), 5.03 (m, 1.17H, H-3), 4.51 (m, 2.35H, H-9), 4.06 (m, 1H, H-8$_A$), 3.96 (m, 1H, H-8$_B$), 3.54 (t, J = 4.2 Hz, 1H, H-7), 2.21 (br. s, 1H, O-H), 2.18 (m, 2H, H-4), 1.71 (m, 1H, H-5$_A$), 1.68 (m, 3.52H, C$\underline{H}_3$), 1.62 (s, 3H, C$\underline{H}_3$), 1.51 (m, 1H, H-5$_B$), 1.31 (s, 3H, H-6) ppm;

^{13}C NMR (176 MHz, $CDCl_3$): δ 137.4, 132.7, 129.9, 129.3, 123.3, 118.4 (qt, $J_{C\text{-}F}$ = 287, 33.8 Hz), 111.5 (tq, $J_{C\text{-}F}$ = 257, 38.3 Hz), 64.2 (t, $J_{C\text{-}F}$ = 27.9 Hz), 63.0, 61.5, 55.7, 38.0, 25.8, 23.8, 23.0, 17.9 ppm;

Note: there is an additional ^{13}C resonance underneath the peak at 129.9 ppm;

^{19}F NMR (376 MHz, $CDCl_3$): δ – 84.5, – 124.5 ppm;

NMR data for product **B**:

^{1}H NMR (700 MHz, $CDCl_3$): δ 7.46 (m, 0.35H, H-10), 7.35-7.28 (m, 0.56H, H-11, H-12), 6.08 (br. s, 0.15H, N-H), 5.03 (m, 1.17H, H-3), 4.51 (m, 2.35H, H-9), 4.00 (dd, J = 12.2, 4.5 Hz, 0.17H, H-8$_A$), 3.90 (dd, J = 12.1, 4.6 Hz, 0.18H,

H-8B), 3.38 (t, J = 4.6 Hz, 0.16H, H-7), 2.07 (m, 0.20H, H-5A), 1.99 (m, 0.35H, H-4), 1.89 (m, 0.17H, H-5B), 1.68 (m, 3.52H, C$\underline{H}_3$), 1.60 (s, 0.63H, H-6), 1.57 (s, 0.72H, C$\underline{H}_3$) ppm;

^{13}C NMR (176 MHz, $CDCl_3$): δ 134.0, 132.9, 132.4, 129.7, 128.2, 122.9, 63.9 (t, $J_{C\text{-}F}$ = 27.9 Hz), 63.8, 61.8, 59.8, 37.0, 25.8, 23.0, 22.6, 17.8 ppm;

*Note: the resonances attributed to the fluorinated carbon atoms in the minor product **B** were not detected.*

^{19}F NMR (376 MHz, $CDCl_3$): δ – 84.4, – 124.2 ppm;

HRMS (+ESI) *m/z* Found $[M+H]^+$ 492.1304, $[C_{19}H_{27}F_5NO_4S_2]^+$ requires 492.1296 (δ = + 1.6 ppm);

$[\alpha]_D^{25.0}$ = – 7.3 (c. 0.1, $CHCl_3$).

Chiral SFC Analysis: CHIRALPAK IG (CO_2:MeOH, 95.0:5.0, 2.50 mL min^{-1}, 40 °C) for major regioisomer A t_R = 4.0 (major), 4.4 (minor) minutes.

*Conversion of **S2** to the thiophenolate adduct for ee determination: 2,2,2-trichloroethyl (R)-(1-hydroxy-3-methyl-3-(phenylthio)butan-2-yl)sulfamate* (**A**) and *2,2,2-trichloroethyl (R)-(4-hydroxy-2-methyl-3-(phenylthio)butan-2-yl)sulfamate* (**B**)

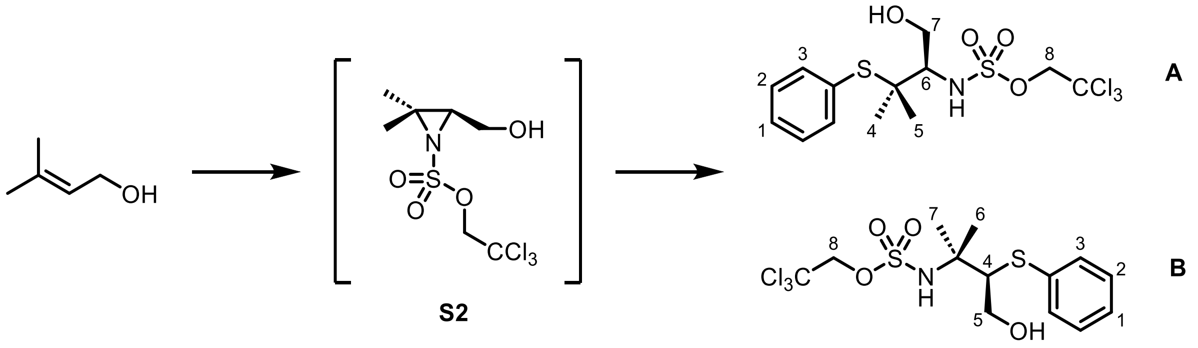

Prepared according to **GP12** using 3-methylbut-2-en-1-ol as the starting material. In this case, the following procedural modifications were employed: the reaction was conducted at – 35 °C, 1.0 mol % of Rh_2(**A-III**)$_2$●(**Cat1**)$_2$●(**pyr**)$_2$ was used, the solvent was 1,3-difluorobenzene and 1.2 equivalents of **NH_2Tces** were used.

Crude ^{1}H NMR analysis revealed a 69% NMR yield of the intermediate aziridine **S2** through comparison of integration relative to 1,3,5-trimethoxybenzene. Purification by flash column chromatography (SiO_2, 0-10% v/v acetone in $CHCl_3$) afforded the intermediate aziridine **S2** which was subjected directly to thiophenol ring-opening.

The thiophenol adduct was prepared according to **GP13**. Purification by flash column chromatography (SiO_2, 0-10% v/v acetone in $CHCl_3$) afforded the 5:1 mixture of title compounds A:B as a white solid (15.7 mg, 0.037 mmol, 37% over 2 steps, 62% *ee*).

***R_f* value**: 0.53 (10% v/v acetone in $CHCl_3$);

NMR data for product **A**:

^{1}H NMR (700 MHz, $CDCl_3$): δ 7.54 (m, 2H, H-3), 7.41 (m, 1H, H-1), 7.36 (m, 2H, H-2), 5.57 (d, J = 8.6 Hz, 1H, N-H), 4.70 (d, J = 10.7 Hz, 1H, H-8$_A$), 4.68 (d, J = 10.7 Hz, 1H, H-8$_B$), 4.09 (dd, J = 12.0, 4.4 Hz, 1H, H-7$_A$), 4.03 (dd, J = 12.0, 4.1 Hz, 1H, H-7$_B$), 3.51 (m, 1H, H-6), 1.40 (s, 3H, C$\underline{H}_3$), 1.34 (s, 3H, C$\underline{H}_3$) ppm;

^{13}C NMR (176 MHz, $CDCl_3$): δ 137.6, 130.0, 129.8, 129.2, 93.6, 78.3, 64.0, 61.7, 52.1, 27.6, 27.0 ppm;

NMR data for product **B**:

^{1}H NMR (700 MHz, $CDCl_3$): δ 7.48 (m, 0.40H, H-3), 7.33 (m, 0.44H, H-2), 7.29 (m, 0.21H, H-1), 6.08 (s, 0.20H, N-H), 4.67 (d, J = 10.7 Hz, 0.35H, H-8$_A$), 4.63 (d, J = 10.7 Hz, 0.19H, H-8$_B$), 4.02 (m, 0.20H, H-5$_A$), 3.90 (dd, J = 12.1, 4.6 Hz, 0.20H, H-5$_B$), 3.27 (t, J = 4.5 Hz, 0.19H, H-4), 1.67 (s, 0.69H, C$\underline{H}_3$), 1.61 (s, 0.66H, C$\underline{H}_3$) ppm;

^{13}C NMR (176 MHz, $CDCl_3$): δ 134.3, 132.6, 129.6, 128.2, 93.5, 78.4, 63.2, 62.2, 60.8, 26.7, 25.3 ppm;

HRMS (+ESI) *m/z* Found $[M+H]^+$ 421.9828, $[C_{13}H_{19}Cl_3NO_4S_2]^+$ requires 421.9816 (δ = + 2.8 ppm);

$[\alpha]_D^{25.0}$ = – 6.7 (c. 1.0, $CHCl_3$).

Chiral SFC Analysis: CHIRALPAK IE (CO_2:MeOH, 90.0:10.0, 2.50 mL min^{-1}, 40 °C) for major regioisomer A t_R = 7.6 (major), 8.8 (minor) minutes.

Examining a cis-alkyl allylic alcohol substrate:

2,2,3,3,3-pentafluoropropyl ((2R,3R)-1-hydroxy-3-(phenylthio)hexan-2-yl)sulfamate (**A**) and *2,2,3,3,3-pentafluoropropyl ((2R,3S)-1-hydroxy-2-(phenylthio)hexan-3-yl)sulfamate* (**B**)

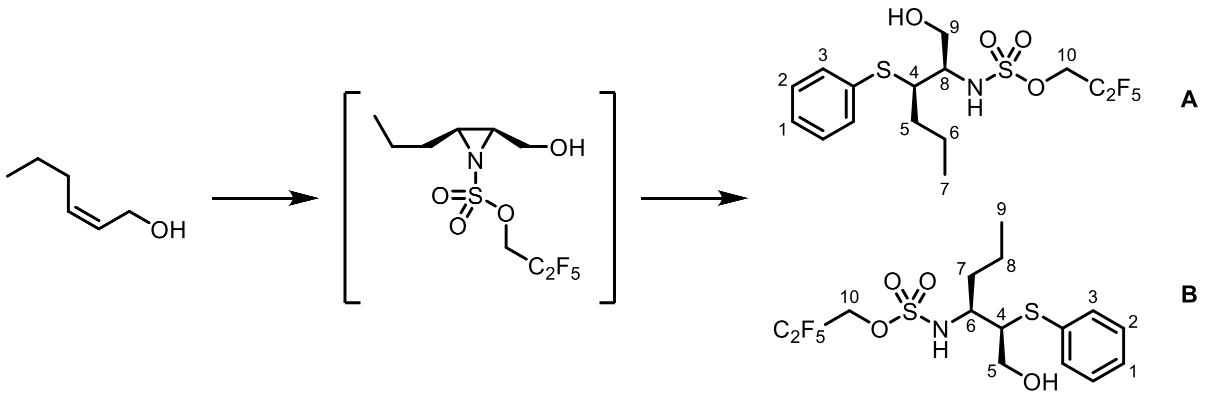

Prepared according to **GP12** using *cis*-2-hexen-1-ol as the starting material.

Crude ^{1}H NMR analysis revealed a 9% NMR yield of the intermediate aziridine through comparison of integration relative to 1,3,5-trimethoxybenzene. Purification by flash column chromatography (SiO_2, 0-10% v/v acetone in $CHCl_3$) afforded the intermediate aziridine which was subjected directly to thiophenol ring-opening.

The thiophenol adduct was prepared according to **GP13**. Purification by flash column chromatography (SiO_2, 0-7% v/v acetone in $CHCl_3$) afforded the 5:1 mixture of title compounds A:B as a colourless oil (1.4 mg, 0.003 mmol, 3% over 2 steps, 55% *ee*).

***R_f* value**: 0.38 (6% v/v acetone in $CHCl_3$);

<u>NMR data for product **A**:</u>

^{1}H NMR (700 MHz, $CDCl_3$): δ 7.44 (m, 2H, H-3), 7.32 (m, 2H, H-2), 7.27 (m, 1H, H-1), 5.33 (br. s, 1H, N-H), 4.50 (m, 2H, H-10), 3.78-3.71 (m, 3H, H-8, H-9), 3.47 (m, 1H, H-4), 1.73 (m, 1H, H-5_A), 1.68 (m, 1H, H-6_A), 1.63-1.50 (m, 2H, H-5_B, H-6_B), 0.96 (t, J = 7.3 Hz, 3H, H-7) ppm;

^{13}C NMR (176 MHz, $CDCl_3$): δ 134.4, 131.7, 129.5, 127.8, 118.3 (qt, $J_{C\text{-}F}$ = 286.1, 34.8 Hz), 111.5 (tq, $J_{C\text{-}F}$ = 256.5, 38.7 Hz), 64.2 (t, $J_{C\text{-}F}$ = 27.9 Hz), 62.4, 59.6, 51.1, 34.2, 20.8, 13.8 ppm;

^{19}F NMR (376 MHz, $CDCl_3$): δ – 84.51, – 124.5 ppm;

<u>NMR data for product **B**:</u>

^{1}H NMR (700 MHz, $CDCl_3$): δ 7.44 (m, 0.33H, H-3), 7.32 (m, 0.30H, H-2), 7.27 (m, 0.17H, H-1), 4.48 (m, 0.36H, H-10), 3.91-3.83 (m, 0.49H, H-5, H-6), 3.44 (m, 0.16H, H-4), 1.73 (m, 0.17H, H-7_A), 1.68 (m, 0.17H, H-7_B), 1.43 (m, 0.17H, H-8_A), 1.30 (m, 0.17H, H-8_B), 0.89 (t, J = 7.3 Hz, 0.59H, H-9) ppm;

^{13}C NMR (176 MHz, $CDCl_3$): δ 133.6, 132.3, 129.5, 128.1, 64.2 (t, $J_{C\text{-}F}$ = 27.9 Hz), 62.8, 56.4, 54.1, 34.4, 19.3, 13.7 ppm;

*Note: the ^{13}C resonances attributed to the fluorinated carbon atoms in the minor product **B** were not detected.*

^{19}F NMR (376 MHz, $CDCl_3$): δ – 84.49, – 124.4 ppm;

HRMS (–ESI) *m/z* Found $[M{-}H]^-$ 436.0675, $[C_{15}H_{19}F_5NO_4S_2]^-$ requires 436.0681 (δ = – 1.4 ppm);

$[\alpha]_D^{25.0}$ = + 19.8 (c. 0.05, $CHCl_3$);

Chiral SFC Analysis: CHIRALPAK IK (CO_2:MeOH, 96.0:4.0, 2.50 mL min^{-1}, 40 °C) for major regioisomer A t_R = 3.8 (minor), 4.2 (major) minutes.

Determination of the Absolute Stereochemistry of Allylic Alcohol-Derived Aziridines

In order to determine the absolute stereochemistry of our aziridine products, we targeted the comparison of optical rotation and chiral GC analysis of enantioenriched samples of **S5** accessed through (a) post-functionalisation of **9a** and (b) protection of commercially available single enantiomers of valinol (**Supplementary Scheme 2**).

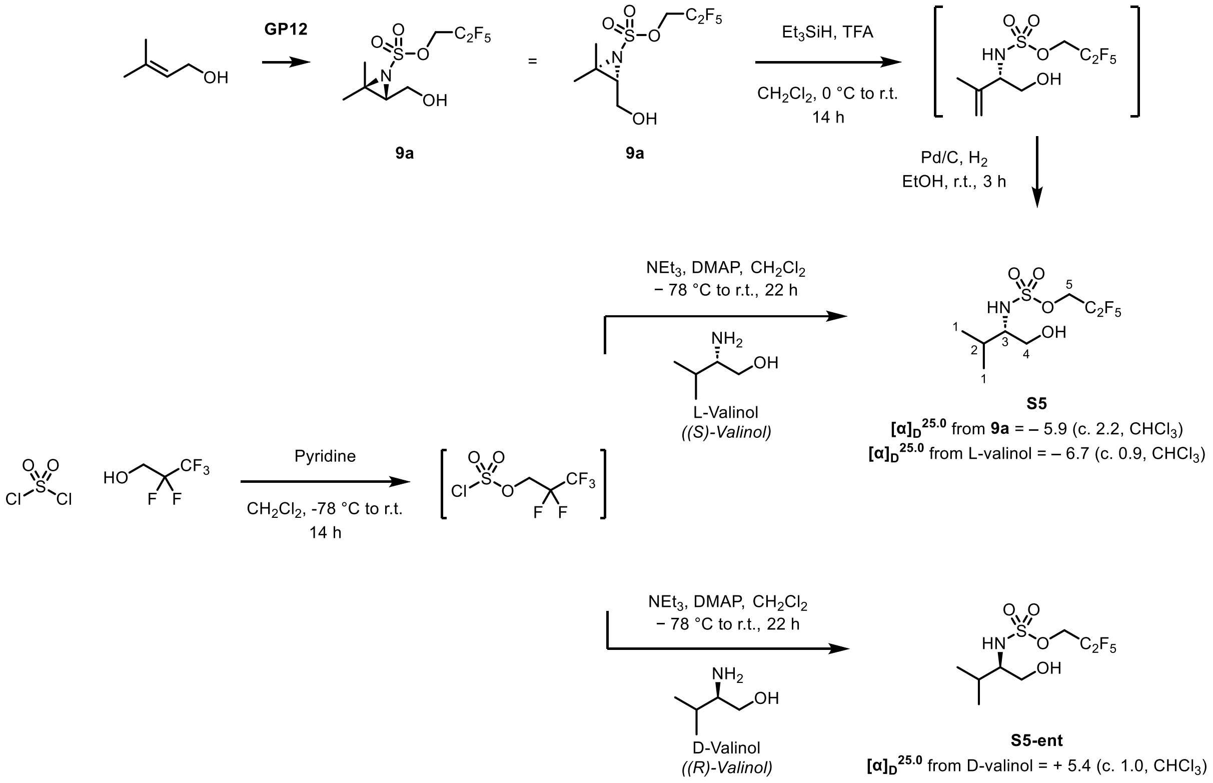

Supplementary Scheme 2: Synthetic routes to access **S5/S5-ent** starting from **9a** or (*R*)- or (*S*)-valinol.

Derivatisation of **9a** *to access 2,2,3,3,3-pentafluoropropyl (S)-(1-hydroxy-3-methylbutan-2-yl)sulfamate* (**S5**):

To a solution of the compound **9a** (86.1 mg, 0.275 mmol, 1 equiv.) in dry CH_2Cl_2 (6.0 mL), Et_3SiH (0.132 mL, 0.825 mmol, 3 equiv.) and TFA (0.211 mL, 2.75 mmol, 10 equiv.) were added dropwise at 0 °C under an inert atmosphere of nitrogen. The reaction mixture was allowed to warm to room temperature and stirred for 14 hours. After the reaction mixture was diluted with CH_2Cl_2 (10 mL), saturated aqueous solution of $NaHCO_3$ (20 mL) was added to the mixture at 0 °C and then the resulting mixture was diluted with water and CH_2Cl_2. The aqueous layer was thrice extracted with CH_2Cl_2 the combined organic layer was dried over Na_2SO_4, filtered and concentrated. Purification by flash column chromatography (SiO_2, 30% v/v ethyl acetate in hexane) was conducted roughly and the alkene intermediate (with impurities) was obtained and used for next step without any further purification. To a solution of the obtained intermediate in EtOH (5.0 mL) was added Pd/C (10 wt %

loading Pd, 14.6 mg, 0.0138 mmol, 5 mol %). The microwave vial was then capped with a fresh suba seal following which it was evacuated and backfilled three times with hydrogen gas. The reaction mixture was stirred at room temperature for 3 hours under a balloon of hydrogen. The reaction was then filtered through Celite®, eluting with ethyl acetate. The filtrate was collected and concentrated under reduced pressure. Purification by flash column chromatography (SiO_2, 30% v/v ethyl acetate in hexane) afforded the title compound as an off-white solid (30.4 mg, 0.0964 mmol, 35% yield over 2 steps, 94% *ee*).

***R_f* value**: 0.26 (30% v/v ethyl acetate in hexane);

^{1}H NMR (700 MHz, $CDCl_3$): δ 5.08 (br s, 1 H, N*H*), 4.57-4.49 (m, 2 H, H-5), 3.81 (dd, *J* = 11.4, 4.0 Hz, 1 H, H-4a), 3.74 (dd, *J* = 11.4, 4.7 Hz, 1 H, H-4b), 3.29-3.27 (m, 1 H, H-3), 1.95 (m, 1 H, H-2), 1.01 (d, *J* = 6.8 Hz, 3 H, H-1a), 0.99 (d, *J* = 6.8 Hz, 3 H, H-1b) ppm;

^{13}C NMR (176 MHz, $CDCl_3$): δ 118.4 (qt, $J_{C\text{-}F}$ = 286.6, 34.2 Hz), 111.6 (tq, $J_{C\text{-}F}$ = 256.9, 38.8 Hz), 64.0 (t, $J_{C\text{-}F}$ = 28.3 Hz), 62.4, 62.1, 29.6, 19.2, 18.9 ppm;

^{19}F NMR (376 MHz, $CDCl_3$): δ -87.6, -123.5 ppm;

HRMS (–ESI) *m/z* Found $[M\text{-}H]^-$ 314.0506, $[C_8H_{13}F_5NO_4S]^-$ requires 314.0491 (*δ* = + 4.8 ppm);

$[\alpha]_D^{25.0}$ = – 5.9 (c. 2.2, $CHCl_3$);

Chiral GC analysis: CycloSil-B (25 m × 0.25 mm × 0.25 µm, 160 °C isothermal, 1.47 mL min $^{-1}$) t_R = 20.2 (major) and 22.0 (minor) minutes.

D-Valinol and L-Valinol were converted to **S5/S5-ent** according to the scheme above. The protocol below is provided for the reaction with D-Valinol to access *2,2,3,3,3-pentafluoropropyl (R)-(1-hydroxy-3-methylbutan-2-yl)sulfamate* (**S5-ent**):

To a solution of 2,2,3,3,3-pentafluoro-1-propanol (0.448 mL, 5.0 mmol, 1 equiv.) and pyridine (0.403 mL, 5.0 mL, 1 equiv.) in dry CH_2Cl_2 (20 mL), sulfuryl chloride (0.496 mL, 5.5 mmol, 1.1 equiv.) was added dropwise at -78 °C under an inert atmosphere of nitrogen. The reaction mixture was allowed to warm to room temperature and stirred for 14 hours. After confirming generation of the corresponding sulfurochloridate intermediate by ^{1}H NMR analysis, the solution was used in the next step directly. To a solution of D-Valinol ((*R*)-(−)-2-Amino-3-methyl-1-butanol, 515 mg, 5.0 mmol, 1 equiv.), Et_3N (2.09 mL, 15.0 mmol, 3 equiv.) and DMAP (611 mg, 5.0 mmol, 1 equiv.) in dry CH_2Cl_2 (20 mL), the solution from the first step was added slowly at -78 °C under an inert atmosphere of nitrogen. The reaction mixture was allowed to warm to room temperature and stirred for 22 hours. After that, saturated aqueous solution of $NaHCO_3$ (40 mL) was added to the mixture at 0 °C. The aqueous layer was thrice extracted with CH_2Cl_2 and the combined organic layer was dried over Na_2SO_4, filtered and concentrated. The residue was purified by flash column chromatography (SiO_2, 15% v/v ethyl acetate in hexane) and the obtained pale-yellow oil was dissolved in ethyl acetate again and washed with saturated aqueous solution of NH_4Cl, $NaHCO_3$ and brine to remove pyridine. The organic layer was dried over Na_2SO_4, filtered and

concentrated. The compound **S5-ent** was obtained as an off-white solid (0.452 g, 1.43 mmol, 29% yield over 2 steps).

From D-Valinol, **S5-ent**:

$[\alpha]_D^{25.0}$ = + 5.4 (c. 0.99, $CHCl_3$);

Chiral GC analysis: CycloSil-B (25 m × 0.25 mm × 0.25 µm, 160 °C isothermal, 1.47 mL min $^{-1}$) t_R = 20.2 minutes.

From L-Valinol, **S5**:

$[\alpha]_D^{25.0}$ = – 6.7 (c. 0.93, $CHCl_3$);

Chiral GC analysis: CycloSil-B (25 m × 0.25 mm × 0.25 µm, 160 °C isothermal, 1.47 mL min $^{-1}$) t_R = 22.0 minutes.

We are very grateful to Michel Sigrist for assistance in acquiring chiral GC traces.

In conclusion, the optical rotation data for **S5** when obtained from **9a** matched the sign as when obtained from L-Valinol. Additionally, the single enantiomeric peak observed in the chiral GC analysis of **S5** derived from L-valinol was also the major enantiomeric peak in the sample of **S5** obtained from **9a**.

Therefore, the aziridination of prenol with **Rh1** afforded **9a** with (*S*)-configuration. The remaining aziridines in this study were assigned the same stereochemistry by analogy.

References

(1) Gottlieb, H. E.; Kotlyar, V.; Nudelman, A. NMR Chemical Shifts of Common Laboratory Solvents as Trace Impurities. *J. Org. Chem.* **1997**, *62*, 7512-7515.
(2) Piotto, M.; Bourdonneau, M.; Elbayed, K.; Wieruszeski, J.-M.; Lippens, G. New DEFT sequences for the acquisition of one-dimensional carbon NMR spectra of small unlabelled molecules. *Magn. Reson. Chem.* **2006**, *44*, 943-947.
(3) Genov, G. R.; Douthwaite, J. L.; Lahdenperä, A. S. K.; Gibson, D. C.; Phipps, R. J. Enantioselective remote C–H activation directed by a chiral cation. *Science* **2020**, *367*, 1246-1251.
(4) Chiappini, N. D.; Mack, J. B. C.; Du Bois, J. Intermolecular C(sp^3)–H Amination of Complex Molecules. *Angew. Chem. Int. Ed.* **2018**, *57*, 4956-4959.
(5) Fanourakis, A.; Hodson, N. J.; Lit, A. R.; Phipps, R. J. Substrate-Directed Enantioselective Aziridination of Alkenyl Alcohols Controlled by a Chiral Cation. *J. Am. Chem. Soc.* **2023**, *145*, 7516-7527.
(6) Fanourakis, A.; Williams, B. D.; Paterson, K. J.; Phipps, R. J. Enantioselective Intermolecular C-H Amination Directed by a Chiral Cation. *J. Am. Chem. Soc.* **2021**, *143*, 10070-10076.
(7) Dominguez-Molano, P.; Bru, G.; Salvado, O.; Maza, R. J.; Carbó, J. J.; Fernández, E. Transborylation of alkenylboranes with diboranes. *Chem. Commun.* **2021**, *57*, 13361-13364.
(8) Raheem, I. T.; Goodman, S. N.; Jacobsen, E. N. Catalytic Asymmetric Total Syntheses of Quinine and Quinidine. *J. Am. Chem. Soc.* **2004**, *126*, 706-707.
(9) Lygo, B.; Crosby, J.; Lowdon, T.; Wainwright, P. G. Asymmetric approaches to 2-hydroxymethylquinuclidine derivatives. *Tetrahedron* **1999**, *55*, 2795-2810.
(10) Jin, J.; Morales-Ramos, Á.; Eidam, P.; Mecom, J.; Li, Y.; Brooks, C.; Hilfiker, M.; Zhang, D.; Wang, N.; Shi, D.; Tseng, P.-S.; Wheless, K.; Budzik, B.; Evans, K.; Jaworski, J.-P.; Jugus, J.; Leon, L.; Wu, C.; Pullen, M.; Karamshi, B.; Rao, P.; Ward, E.; Laping, N.; Evans, C.; Leach, C.; Holt, D.; Su, X.; Morrow, D.; Fries, H.; Thorneloe, K.; Edwards, R. Novel 3-Oxazolidinedione-6-aryl-pyridinones as Potent, Selective, and Orally Active EP3 Receptor Antagonists. *ACS Med. Chem. Lett.* **2010**, *1*, 316-320.
(11) Yousuf, Z.; Richards, A. K.; Dwyer, A. N.; Linclau, B.; Harrowven, D. C. The development of a short route to the API ropinirole hydrochloride. *Org. Biomol. Chem.* **2015**, *13*, 10532-10539.
(12) Kandagatla, B.; Raju, V. V. N. K. V. P.; Reddy, G. M.; Rao, S. C.; Iqbal, J.; Bandichhor, R.; Oruganti, S. A facile synthesis of melatonergic antidepressant agomelatine. *Tetrahedron Lett.* **2012**, *53*, 7125-7127.
(13) Bruno, N. C.; Tudge, M. T.; Buchwald, S. L. Design and preparation of new palladium precatalysts for C–C and C–N cross-coupling reactions. *Chem. Sci.* **2013**, *4*, 916-920.
(14) Sarkar, S. M.; Taira, Y.; Nakano, A.; Takahashi, K.; Ishihara, J.; Hatakeyama, S. Organocatalytic asymmetric synthesis of quinine and quinidine. *Tetrahedron Lett.* **2011**, *52*, 923-927.
(15) Yu, W.; Mei, Y.; Kang, Y.; Hua, Z.; Jin, Z. Improved Procedure for the Oxidative Cleavage of Olefins by OsO_4–$NaIO_4$. *Org. Lett.* **2004**, *6*, 3217-3219.
(16) Olsen, E. P. K.; Madsen, R. Iridium-Catalyzed Dehydrogenative Decarbonylation of Primary Alcohols with the Liberation of Syngas. *Chem. Eur. J.* **2012**, *18*, 16023-16029.
(17) Hintermann, L.; Schmitz, M.; Englert, U. Nucleophilic Addition of Organometallic Reagents to Cinchona Alkaloids: Simple Access to Diverse Architectures. *Angew. Chem. Int. Ed.* **2007**, *46*, 5164-5167.
(18) Paterson, K. J.; Dahiya, A.; Williams, B. D.; Phipps, R. J. Tertiary Amides as Directing Groups for Enantioselective C–H Amination using Ion-Paired Rhodium Complexes. *Angew. Chem. Int. Ed.* **2024**, *63*, e202317489.
(19) Zhao, Q.; Hanson, J. E. Direct Synthesis of Poly(arylmethyl Sulfone) Monodendrons. *Synthesis* **2006**, *2006*, 397-399.
(20) Bheeter, C. B.; Bera, J. K.; Doucet, H. Palladium-Catalysed Intramolecular Direct Arylation of 2-Bromobenzenesulfonic Acid Derivatives. *Adv. Synth. Catal.* **2012**, *354*, 3533-3538.

(21) Szostak, M.; Spain, M.; Procter, D. J. Electron Transfer Reduction of Carboxylic Acids Using SmI_2-H_2O-Et_3N. *Org. Lett.* **2012**, *14*, 840-843.
(22) Gaiser, B. I.; Danielsen, M.; Marcher-Rorsted, E.; Ropke Jorgensen, K.; Wrobel, T. M.; Frykman, M.; Johansson, H.; Brauner-Osborne, H.; Gloriam, D. E.; Mathiesen, J. M.; Sejer Pedersen, D. Probing the Existence of a Metastable Binding Site at the beta(2)-Adrenergic Receptor with Homobivalent Bitopic Ligands. *J. Med. Chem.* **2019**, *62*, 7806-7839.
(23) Falk, E.; Gasser, V. C. M.; Morandi, B. Synthesis of N-Alkyl Anilines from Arenes via Iron-Promoted Aromatic C-H Amination. *Org. Lett.* **2021**, *23*, 1422-1426.
(24) Konopacki, D. B.; Shortsleeves, K. C.; Turnbull, M. M.; Wikaira, J. L.; Hobson, A. D. Lewis Acid Catalyzed Cyclizations of Epoxidized Baylis–Hillman Products: A Straightforward Synthesis of Octahydro-benzo[e]azulenes. *Eur. J. Org. Chem.* **2015**, *2015*, 5453-5463.
(25) Ambler, B. R.; Peddi, S.; Altman, R. A. Ligand-Controlled Regioselective Copper-Catalyzed Trifluoromethylation To Generate (Trifluoromethyl)allenes. *Org. Lett.* **2015**, *17*, 2506-2509.
(26) Lunic, D.; Sanosa, N.; Funes-Ardoiz, I.; Teskey, C. J. Mild and Chemoselective Carboxylic Acid Reduction Promoted by Borane Catalysis. *Angew. Chem. Int. Ed.* **2022**, *61*, e202207647.
(27) Huang, C.; Ma, W.; Zheng, X.; Xu, M.; Qi, X.; Lu, Q. Epoxide Electroreduction. *J. Am. Chem. Soc.* **2022**, *144*, 1389-1395.
(28) Kumari, A.; Gholap, S. P.; Fernandes, R. A. Tandem IBX-Promoted Primary Alcohol Oxidation/Opening of Intermediate beta,gamma-Diolcarbonate Aldehydes to (*E*)-gamma-Hydroxy-alpha,beta-enals. *Chem. Asian J.* **2019**, *14*, 2278-2290.
(29) Kobayashi, M.; Itoh, S.; Yoshimura, K.; Tsukamoto, Y.; Obora, Y. Iridium Complex-Catalyzed C2-Extension of Primary Alcohols with Ethanol via a Hydrogen Autotransfer Reaction. *J. Org. Chem.* **2020**, *85*, 11952-11958.
(30) Zimmermann, B. M.; Ngoc, T. T.; Tzaras, D. I.; Kaicharla, T.; Teichert, J. F. A Bifunctional Copper Catalyst Enables Ester Reduction with H_2: Expanding the Reactivity Space of Nucleophilic Copper Hydrides. *J. Am. Chem. Soc.* **2021**, *143*, 16865-16873.
(31) Venditto, N. J.; Liang, Y. S.; El Mokadem, R. K.; Nicewicz, D. A. Ketone-Olefin Coupling of Aliphatic and Aromatic Carbonyls Catalyzed by Excited-State Acridine Radicals. *J. Am. Chem. Soc.* **2022**, *144*, 11888-11896.
(32) Chen, D. D.; Zhang, B. Y.; Liu, X. X.; Li, X. Q.; Yang, X. J.; Zhou, L. Bioactivity and structure-activity relationship of cinnamic acid derivatives and its heteroaromatic ring analogues as potential high-efficient acaricides against Psoroptes cuniculi. *Bioorg. Med. Chem. Lett.* **2018**, *28*, 1149-1153.
(33) Sandford, C.; Rasappan, R.; Aggarwal, V. K. Synthesis of Enantioenriched Alkylfluorides by the Fluorination of Boronate Complexes. *J. Am. Chem. Soc.* **2015**, *137*, 10100-10103.
(34) Zhou, R.; Wang, W.; Jiang, Z.-j.; Wang, K.; Zheng, X.-l.; Fu, H.-y.; Chen, H.; Li, R.-x. One-pot synthesis of 2-substituted benzo[b]furans via Pd–tetraphosphine catalyzed coupling of 2-halophenols with alkynes. *Chem. Commun.* **2014**, *50*, 6023-6026.
(35) Zhang, Q. C.; Wu, F. T.; Hao, H. M.; Xu, H.; Zhao, H. X.; Long, L. S.; Huang, R. B.; Zheng, L. S. Modulating the rotation of a molecular rotor through hydrogen-bonding interactions between the rotator and stator. *Angew. Chem. Int. Ed.* **2013**, *52*, 12602-12605.
(36) Tahara, A.; Sunada, Y.; Takeshita, T.; Inoue, R.; Nagashima, H. Remarkably high catalyst efficiency of a disilaruthenacyclic complex for hydrosilane reduction of carbonyl compounds. *Chem. Commun.* **2018**, *54*, 11192-11195.
(37) Pandit, S.; Adhikari, A. S.; Majumdar, N. Iridium-Catalyzed Enantioselective Ring Opening of Alkenyl Oxiranes by Unactivated Carboxylic Acids. *Org. Lett.* **2022**, *24*, 7388-7393.
(38) Szcześniak, P.; Pieczykolan, M.; Stecko, S. The Synthesis of α,α-Disubstituted α-Amino Acids via Ichikawa Rearrangement. *J. Org. Chem.* **2016**, *81*, 1057-1074.
(39) Zhang, C.; Wang, D.-S.; Lee, W.-C. C.; McKillop, A. M.; Zhang, X. P. Controlling Enantioselectivity and Diastereoselectivity in Radical Cascade Cyclization for Construction of Bicyclic Structures. *J. Am. Chem. Soc.* **2021**, *143*, 11130-11140.

(40) Wang, X.; Chen, Y.; Liang, P.; Chen, J.-Q.; Wu, J. Synthesis of β-fluorocarboxylic esters via organophotoredox-catalyzed fluoroalkoxycarbonylation of alkenes in EtOH. *Green Chem.* **2022**, *24*, 5077-5082.
(41) Zhao, X.; Jia, J.; Li, Z.; Li, H.; Wang, Y.; Wang, G. Stereoselective $ZnCl_2$-Catalyzed B-H Bond Insertion of Vinyl Carbenes Generated from Cyclopropenes for the Synthesis of Allylboranes. *J. Org. Chem.* **2022**, *87*, 13053-13061.
(42) Bamborough, P.; Chung, C. W.; Furze, R. C.; Grandi, P.; Michon, A. M.; Watson, R. J.; Mitchell, D. J.; Barnett, H.; Prinjha, R. K.; Rau, C.; Sheppard, R. J.; Werner, T.; Demont, E. H. Aiming to Miss a Moving Target: Bromo and Extra Terminal Domain (BET) Selectivity in Constrained ATAD2 Inhibitors. *J. Med. Chem.* **2018**, *61*, 8321-8336.
(43) Szczesniak, P.; Pieczykolan, M.; Stecko, S. The Synthesis of α,α-Disubstituted α-Amino Acids via Ichikawa Rearrangement. *J. Org. Chem.* **2016**, *81*, 1057-1074.
(44) Reed, J. H.; Donets, P. A.; Miaskiewicz, S.; Cramer, N. A 1,3,2-Diazaphospholene-Catalyzed Reductive Claisen Rearrangement. *Angew. Chem. Int. Ed.* **2019**, *58*, 8893-8897.
(45) Stocks, P. A.; Bray, P. G.; Barton, V. E.; Al-Helal, M.; Jones, M.; Araujo, N. C.; Gibbons, P.; Ward, S. A.; Hughes, R. H.; Biagini, G. A.; Davies, J.; Amewu, R.; Mercer, A. E.; Ellis, G.; O'Neill, P. M. Evidence for a common non-heme chelatable-iron-dependent activation mechanism for semisynthetic and synthetic endoperoxide antimalarial drugs. *Angew. Chem. Int. Ed.* **2007**, *46*, 6278-6283.
(46) Srikrishna, A.; Kumar, P. P. Claisen rearrangement based methodology for the spiroannulation of a cyclopentane ring.: Formal total synthesis of (±)-acorone and isoacorones. *Tetrahedron* **2000**, *56*, 8189-8195.
(47) Comito, R. J.; Finelli, F. G.; MacMillan, D. W. Enantioselective intramolecular aldehyde alpha-alkylation with simple olefins: direct access to homo-ene products. *J. Am. Chem. Soc.* **2013**, *135*, 9358-9361.
(48) Guthikonda, K.; Du Bois, J. A Unique and Highly Efficient Method for Catalytic Olefin Aziridination. *J. Am. Chem. Soc.* **2002**, *124*, 13672-13673.
(49) You, P.; Qiu, J.; Su, E.; Wei, D. Carica papaya Lipase Catalysed Resolution of β-Amino Esters for the Highly Enantioselective Synthesis of (*S*)-Dapoxetine. *Eur. J. Org. Chem.* **2012**, *2013*, 557-565.
(50) Kang, S.; Lee, H.-K. Highly Efficient, Enantioselective Syntheses of (*S*)-(+)- and (*R*)-(−)-Dapoxetine Starting with 3-Phenyl-1-propanol. *J. Org. Chem.* **2010**, *75*, 237-240.
(51) Guo, S.; Yang, J. C.; Buchwald, S. L. A Practical Electrophilic Nitrogen Source for the Synthesis of Chiral Primary Amines by Copper-Catalyzed Hydroamination. *J. Am. Chem. Soc.* **2018**, *140*, 15976-15984.

Chiral SFC, HPLC and GC Traces

(4-((2S,3S)-2,3-dihydroxy-3-(naphthalen-1-yl)propyl)piperidin-1-yl)(phenyl)methanone (**11d'**)

Chiral SFC Analysis: CHIRALPAK IG (CO_2:MeOH, 60:40, 2.5 mL min^{-1}, 40 °C, 221 nm) indicated >99% *ee*, t_R = 8.99 (major), 10.27 (minor) minutes.

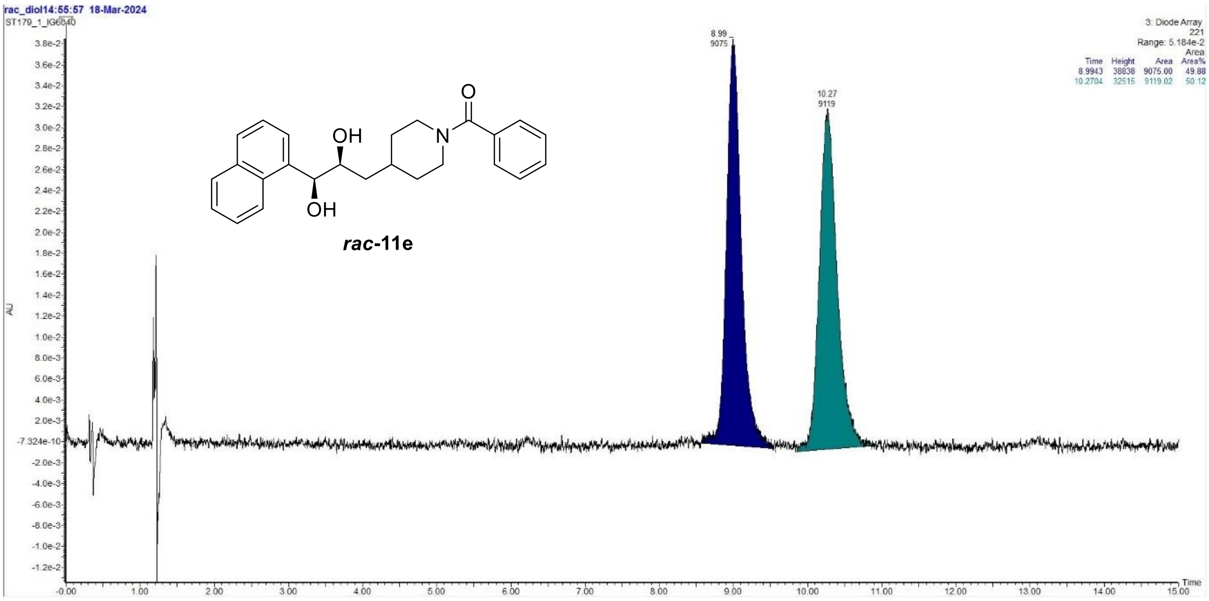

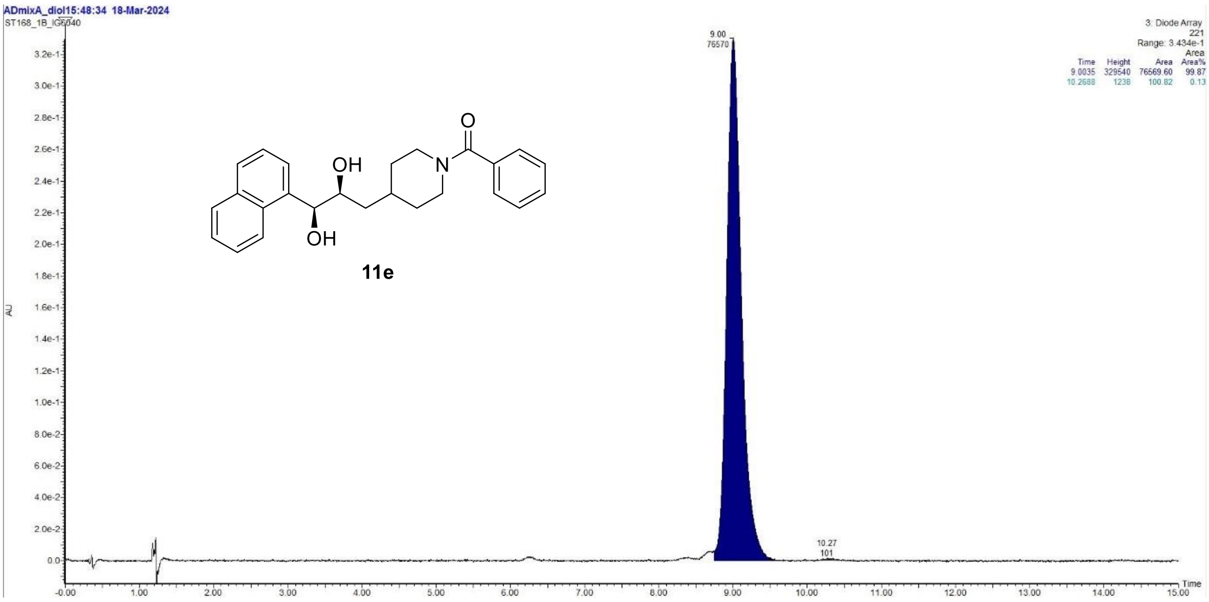

(4-(((2S,3S)-3-(naphthalen-1-yl)oxiran-2-yl)methyl)piperidin-1-yl)(phenyl)methanone (**11e**)

Chiral SFC Analysis: CHIRALPAK IA (CO_2:MeOH, 65:35, 2.5 mL min^{-1}, 40 °C, 220 nm) indicated 98% *ee*, t_R = 4.94 (major), 5.58 (minor) minutes.

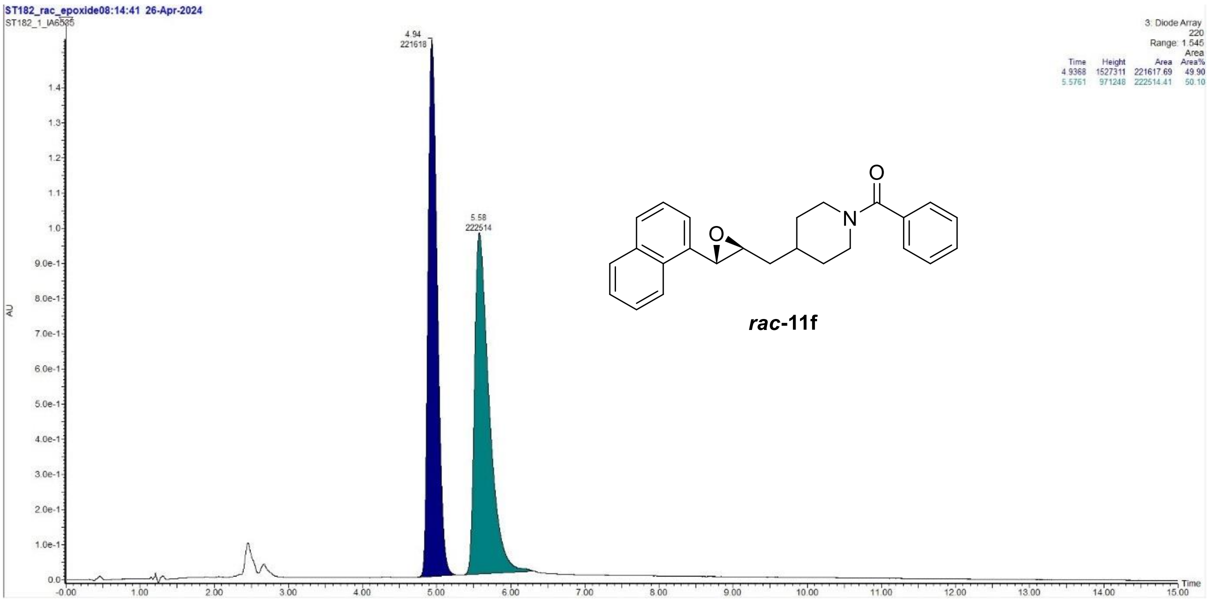

***rac*-11f**

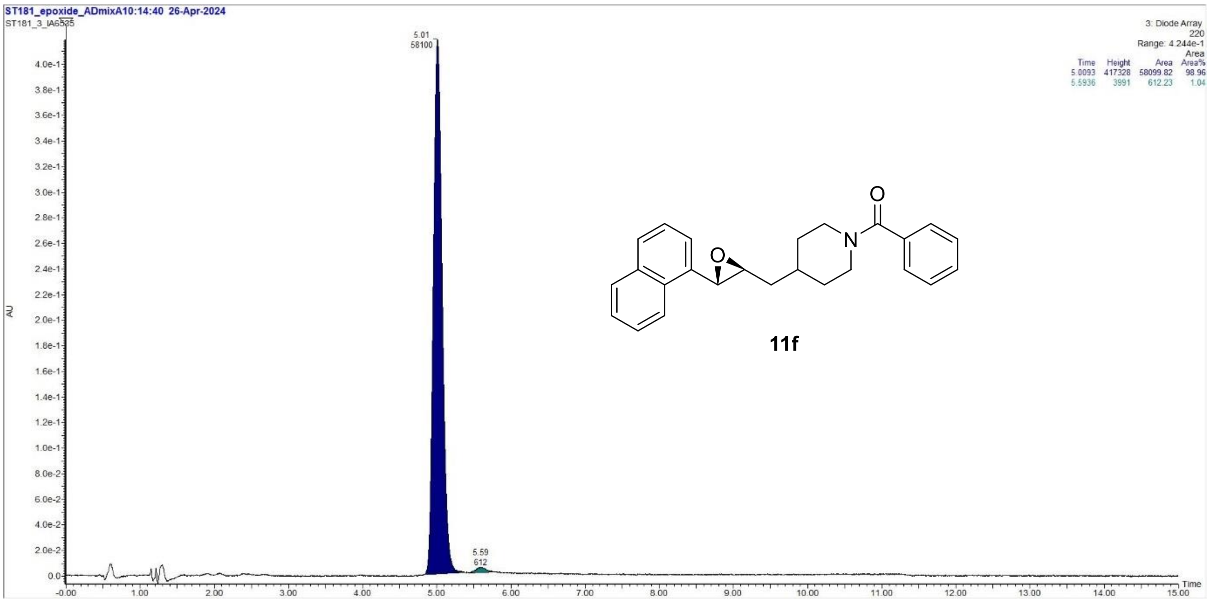

11f

(4-((2S,3S)-2,3-dihydroxy-3-(7-methoxynaphthalen-1-yl)propyl)piperidin-1-yl)(phenyl)methanone

Chiral SFC Analysis: CHIRALPAK IG (CO_2:MeOH, 60:40, 2.5 mL min^{-1}, 40 °C, 227 nm) indicated >99% *ee*, t_R = 8.23 (minor), 10.46 (major) minutes.

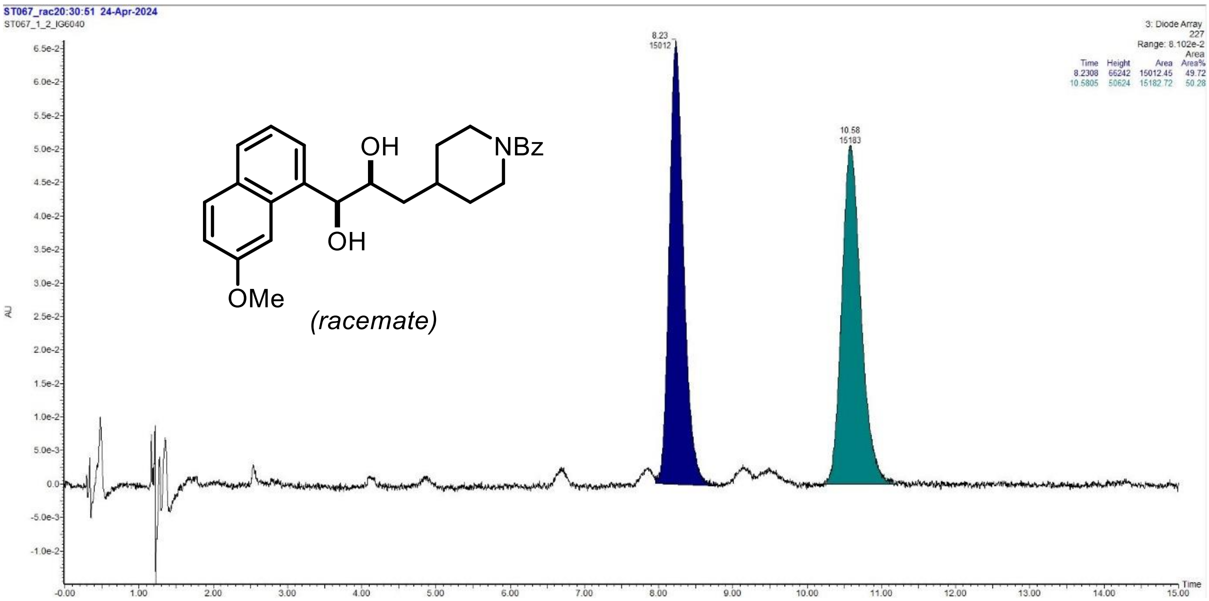

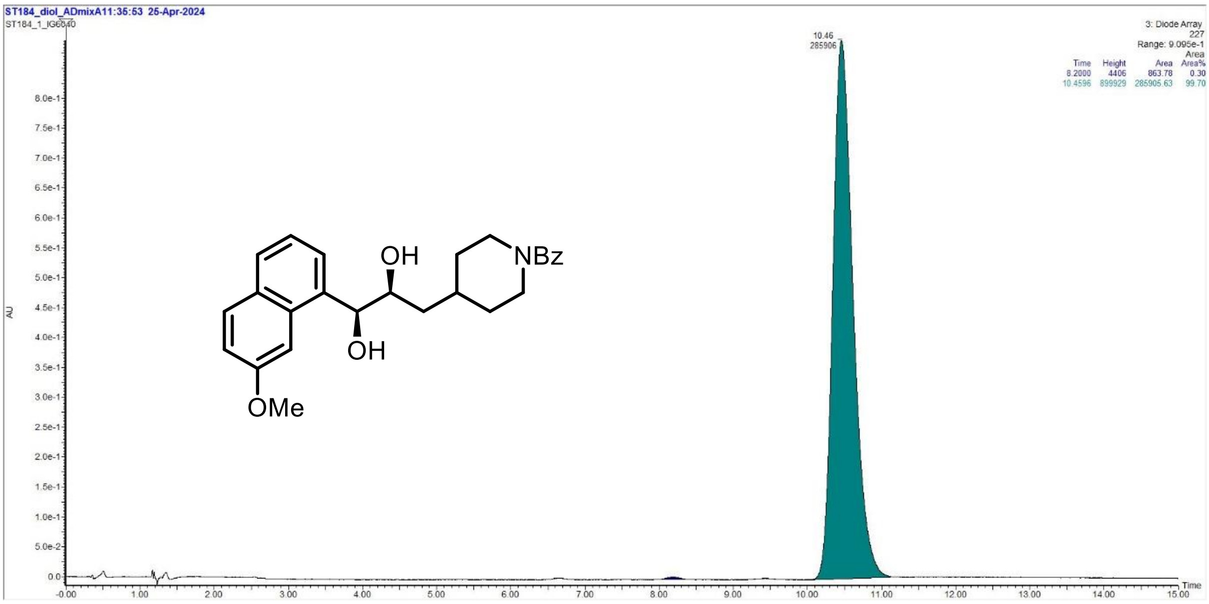

(4-(((2S,3S)-3-(7-methoxynaphthalen-1-yl)oxiran-2-yl)methyl)piperidin-1-yl)(phenyl)methanone

Chiral SFC Analysis: CHIRALPAK IG (CO_2:MeOH, 60:40, 2.5 mL min^{-1}, 40 °C, 227 nm) indicated >99% *ee*, t_R = 23.83 (major), 31.25 (minor) minutes.

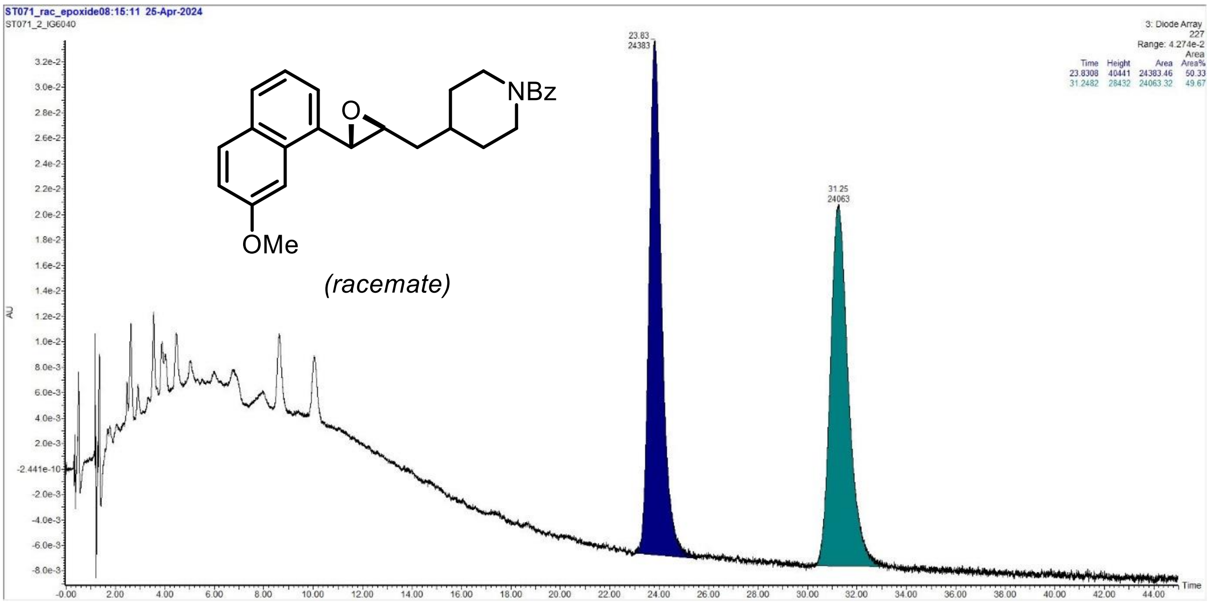

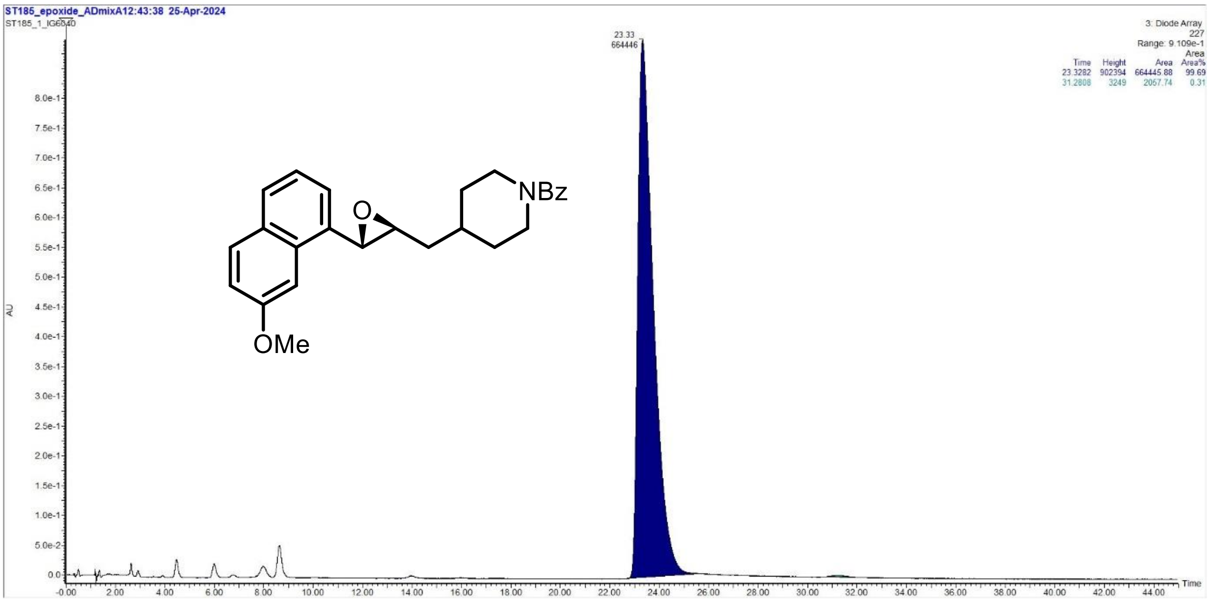

(4-((2S,3S)-2,3-dihydroxy-3-(quinolin-5-yl)propyl)piperidin-1-yl)(phenyl)methanone

Chiral SFC Analysis: CHIRALPAK IG (CO_2:MeOH, 60:40, 2.5 mL min^{-1}, 40 °C, 227 nm) indicated >99% *ee*, t_R = 6.73 (minor), 7.72 (major) minutes.

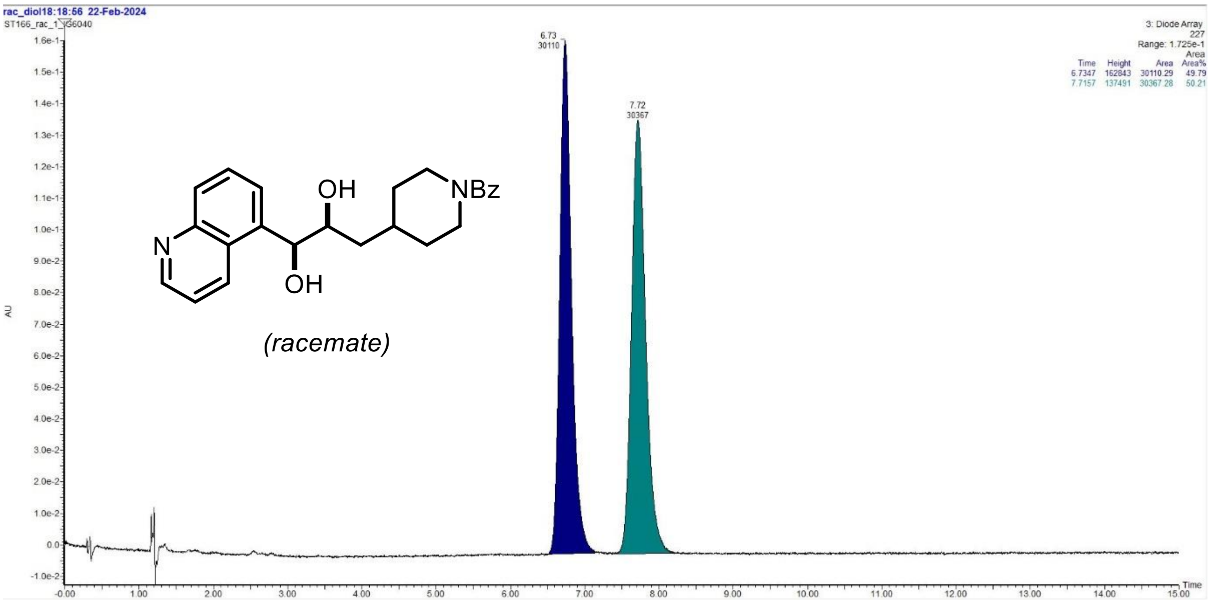

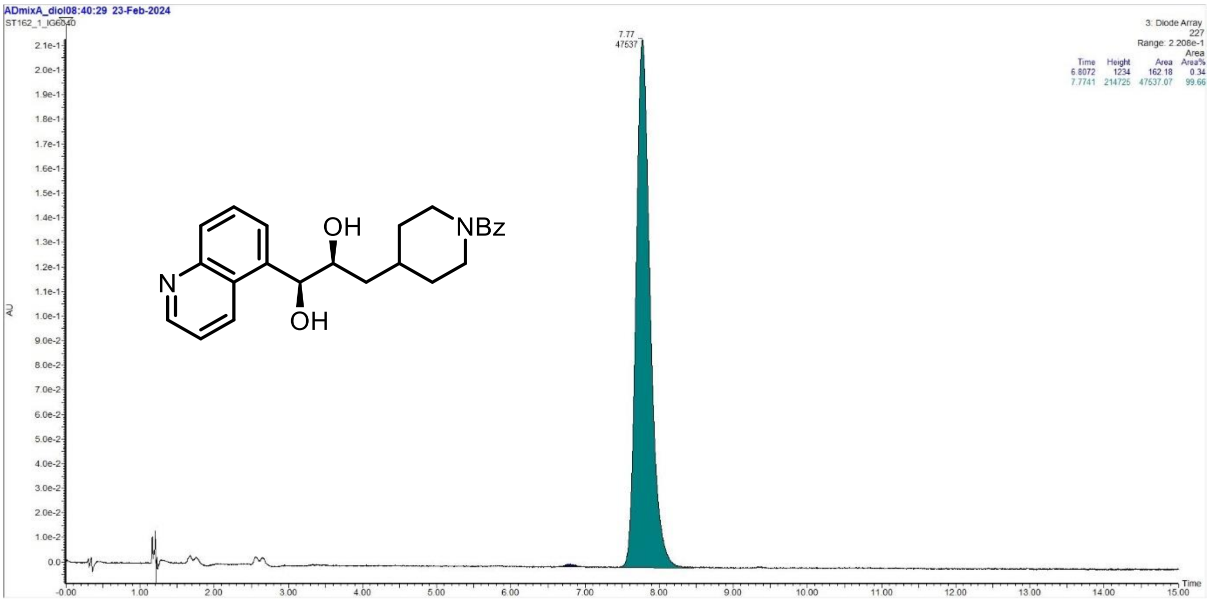

phenyl(4-(((2S,3S)-3-(quinolin-5-yl)oxiran-2-yl)methyl)piperidin-1-yl)methanone

Chiral SFC Analysis: CHIRALPAK IG (CO_2:MeOH, 60:40, 2.5 mL min^{-1}, 40 °C, 227 nm) indicated >99% *ee*, t_R = 13.26 (major), 20.65 (minor) minutes.

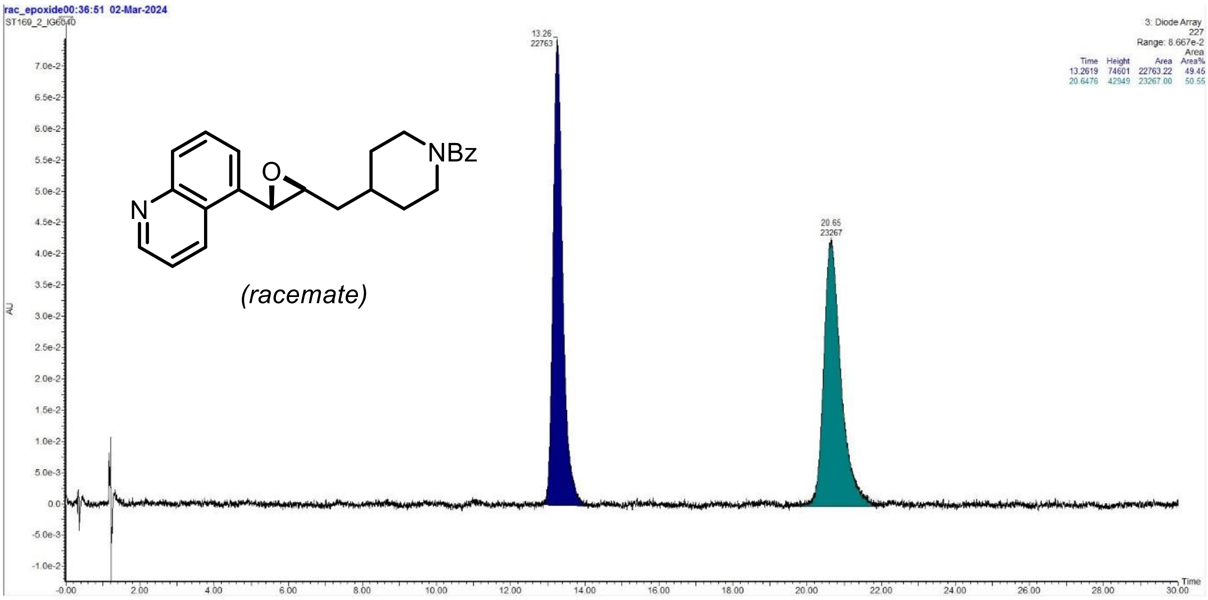

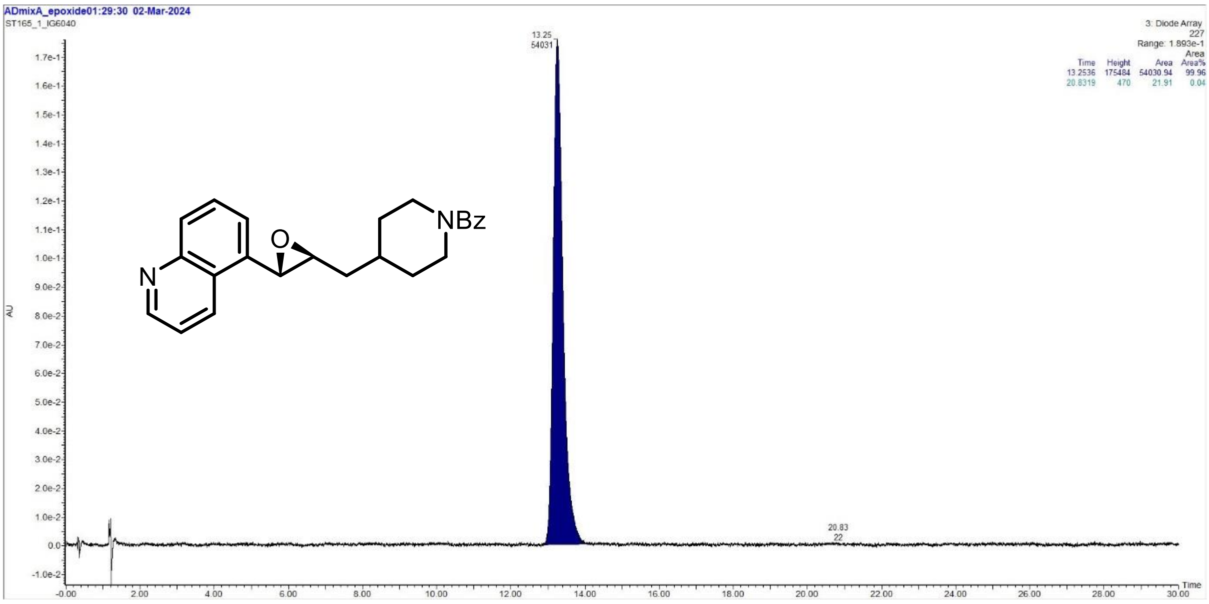

2,2,3,3,4,4,4-heptafluorobutyl (R)-(3-hydroxy-1-phenylpropyl)sulfamate (**2a**)

Obtained using Rh_2(**A**-**III**)$_2$●(**Cat8**)$_2$●(**pyr**)$_2$

Chiral SFC Analysis CHIRALPAK IG (CO_2:MeOH, 97.5:2.5, 2.50 mL min^{-1}, 40 °C, 205 nm) indicated 95% *ee*, t_R = 7.6 (minor), 8.2 (major) minutes.

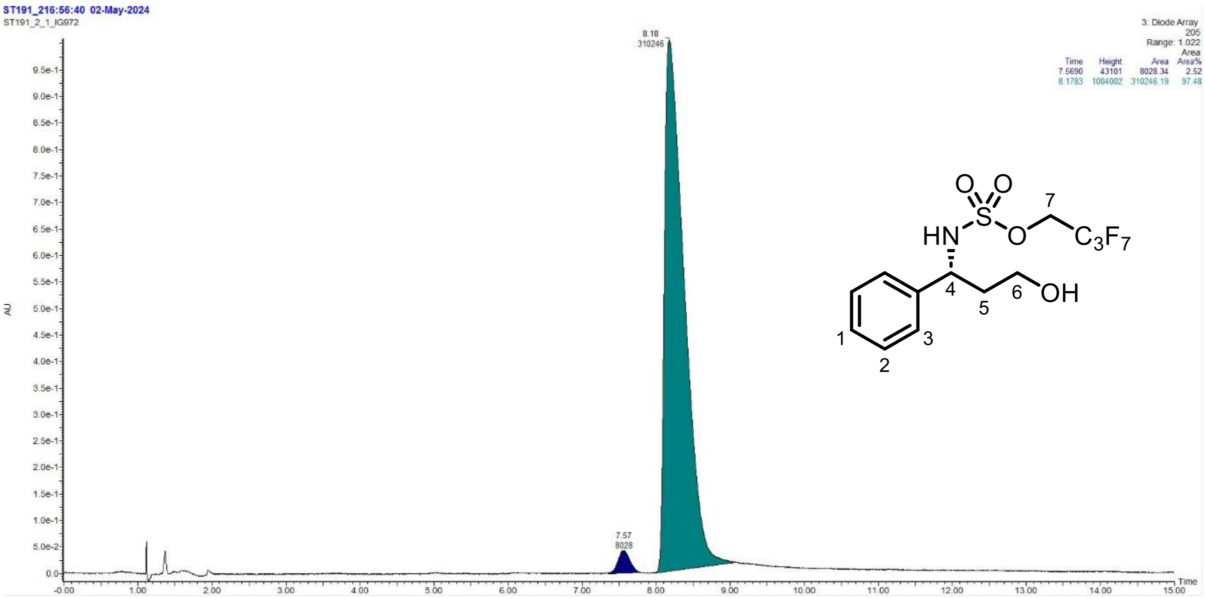

Note: see J. Am. Chem. Soc. **2021**, *143*, 10070-10076*, supporting information, page S181 for a racemic trace for compound* **2a**

2,2,2-trichloroethyl (R)-(3-hydroxy-1-phenylpropyl)sulfamate (**2b**)

Chiral SFC Analysis CHIRALPAK IG (CO_2:MeOH, 85.0:15.0, 2.50 mL min^{-1}, 40 °C, 210 nm) indicated 93% *ee*, t_R = 4.5 (minor), 6.1 (major) minutes.

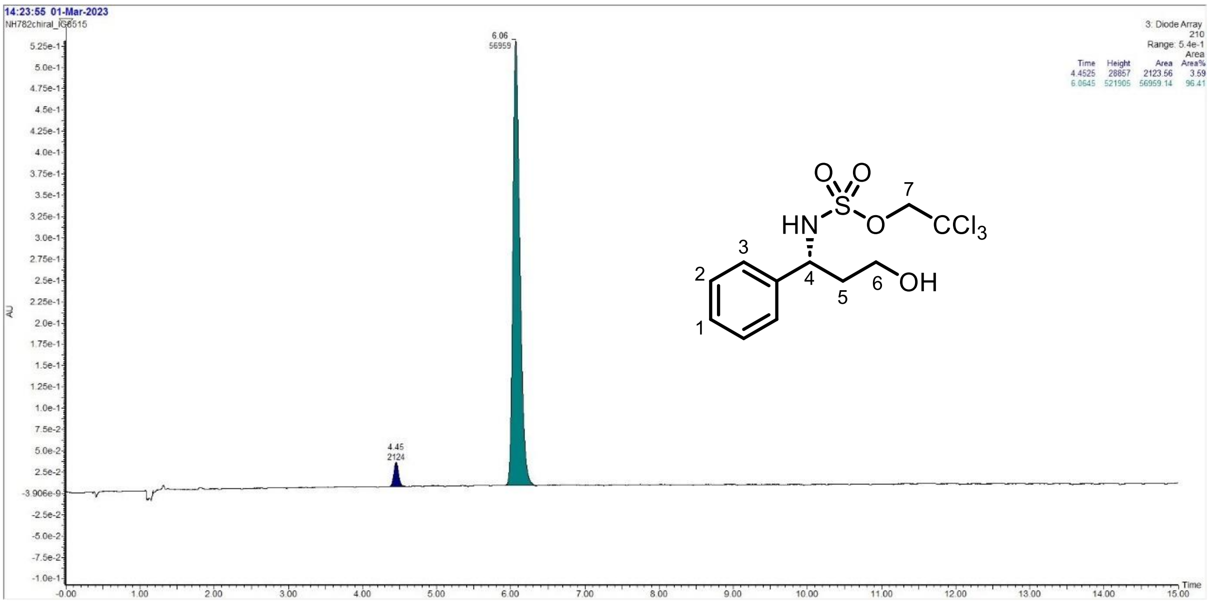

Chiral SFC Analysis CHIRALPAK IG (CO_2:MeOH, 85.0:15.0, 2.50 mL min^{-1}, 40 °C, 210 nm) indicated >99% *ee*, t_R = 4.6 (minor), 6.2 (major) minutes.

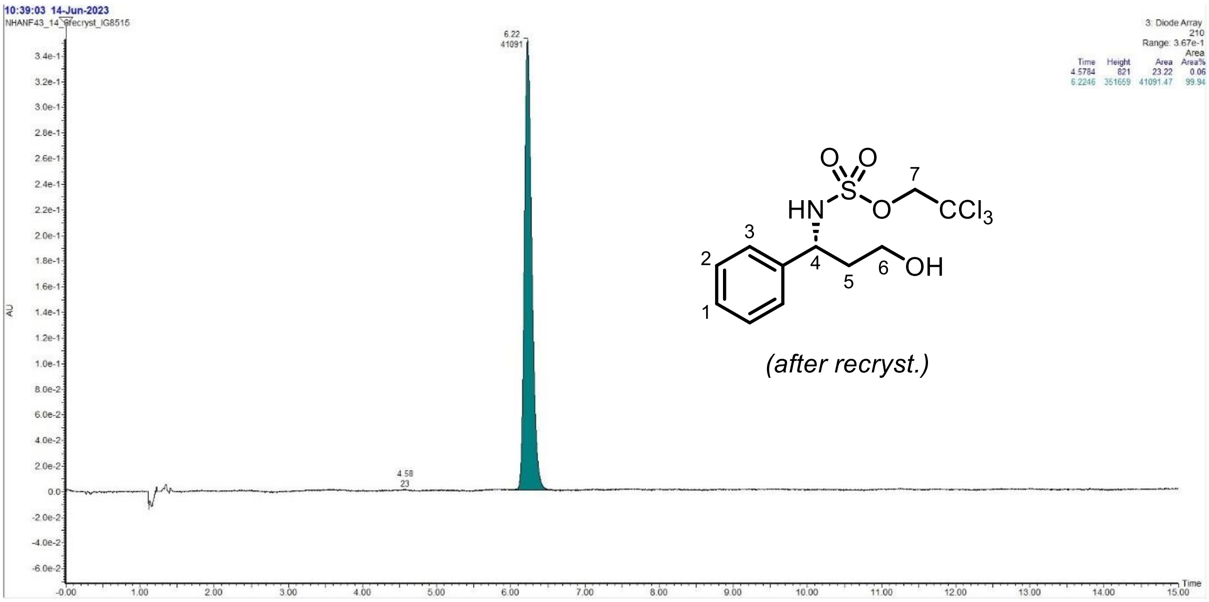

2,2,2-trichloroethyl (S)-(3-hydroxy-1-phenylpropyl)sulfamate (**2b-ent**)

Chiral SFC Analysis CHIRALPAK IG (CO_2:MeOH, 85.0:15.0, 2.50 mL min^{-1}, 40 °C, 210 nm) indicated 89% *ee*, t_R = 4.6 (major), 6.3 (minor) minutes.

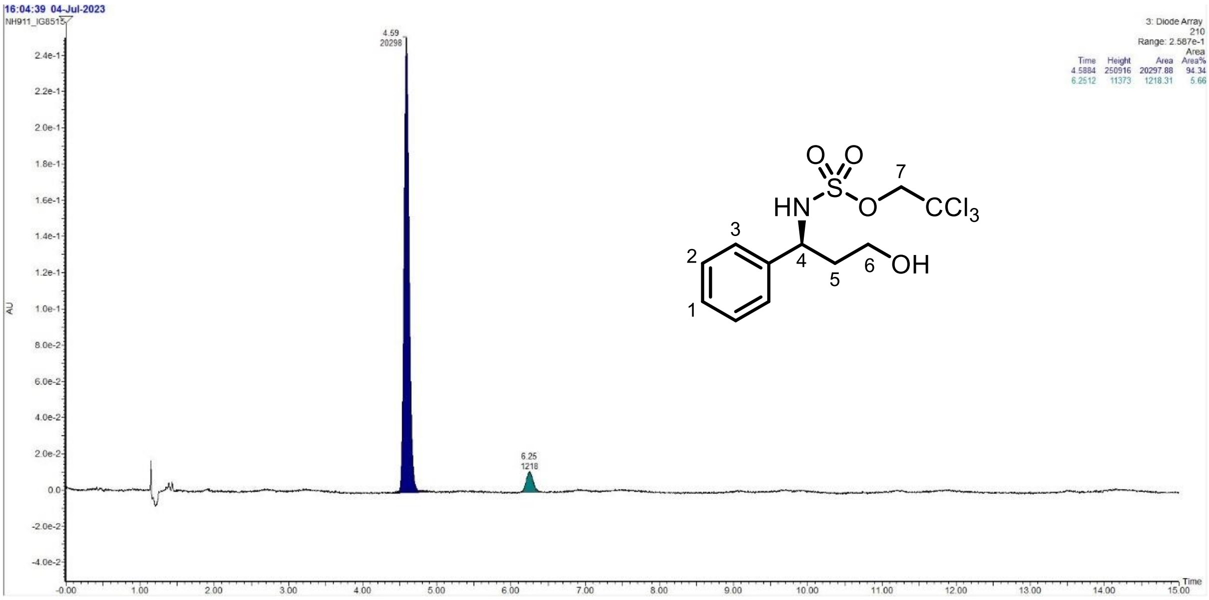

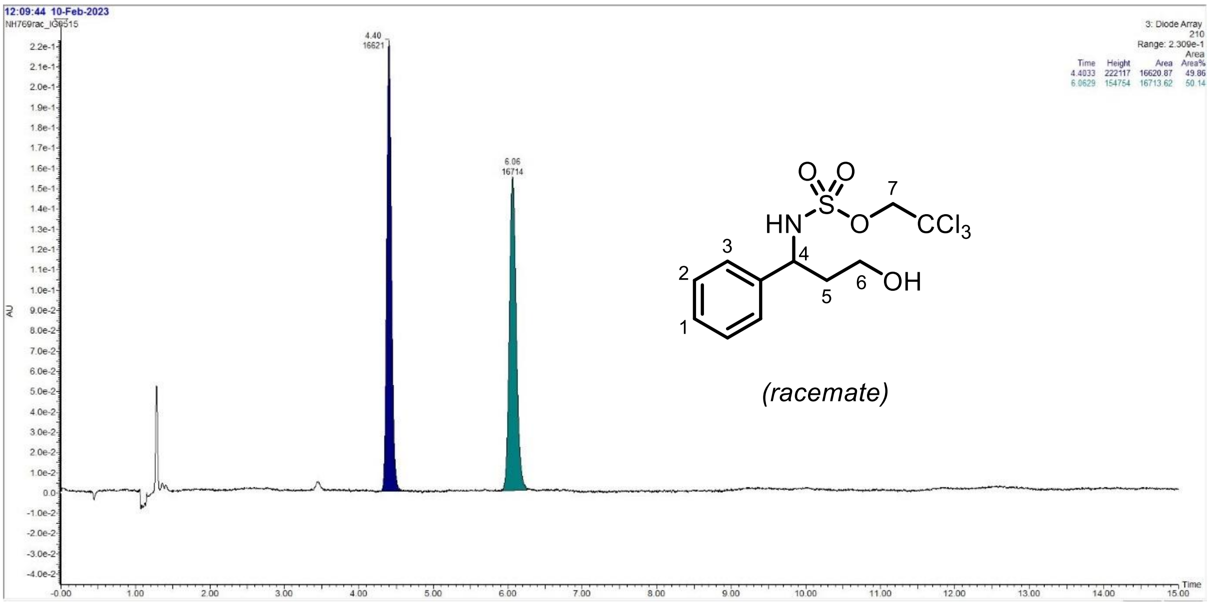

2,2,2-trichloroethyl (R)-(3-hydroxy-1-(o-tolyl)propyl)sulfamate (**2c**)

Chiral SFC Analysis CHIRALPAK IG (CO_2:MeOH, 90.0:10.0, 2.50 mL min^{-1}, 40 °C, 210 nm) indicated 94% *ee*, t_R = 6.2 (minor), 6.9 (major) minutes.

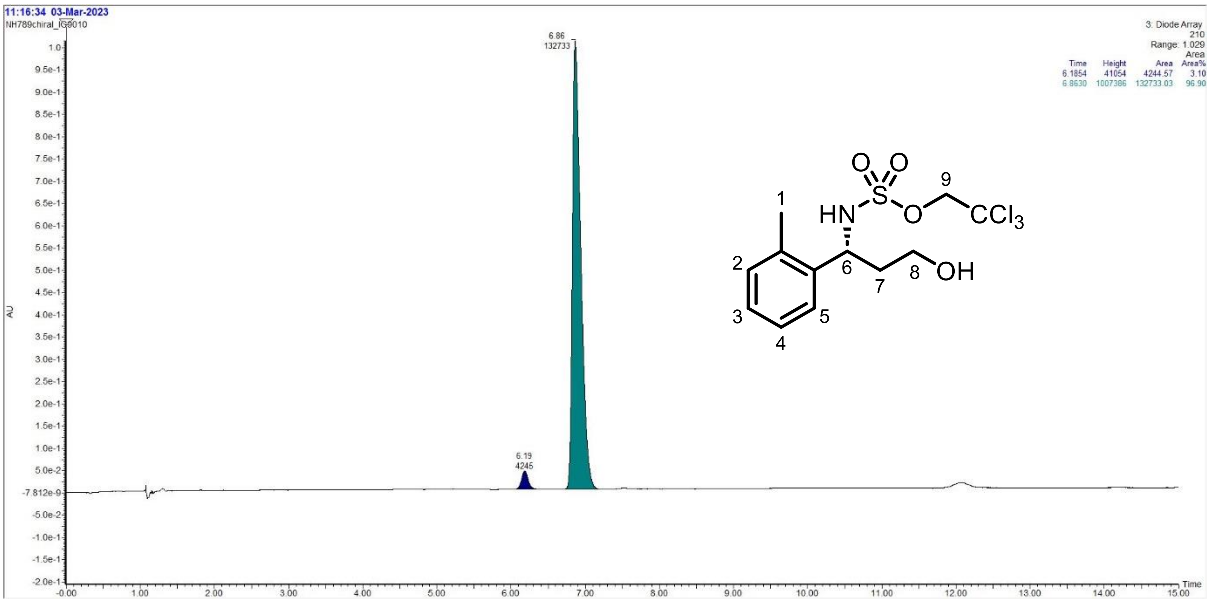

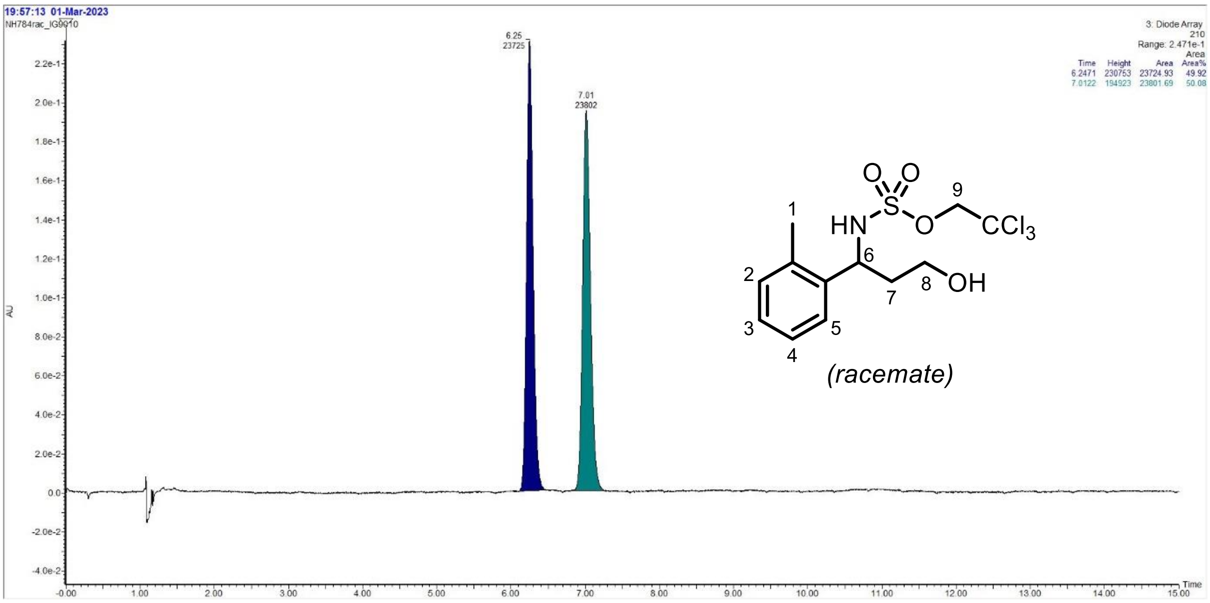

2,2,2-trichloroethyl (R)-(3-hydroxy-1-(2-methoxyphenyl)propyl)sulfamate (**2d**)

Chiral HPLC Analysis CHIRAL ART SC (hexane:*i*PrOH, 88.0:12.0, 1.25 mL min^{-1}, 40 °C, 216 nm) indicated 95% *ee*, t_R = 10.8 (minor), 11.6 (major) minutes.

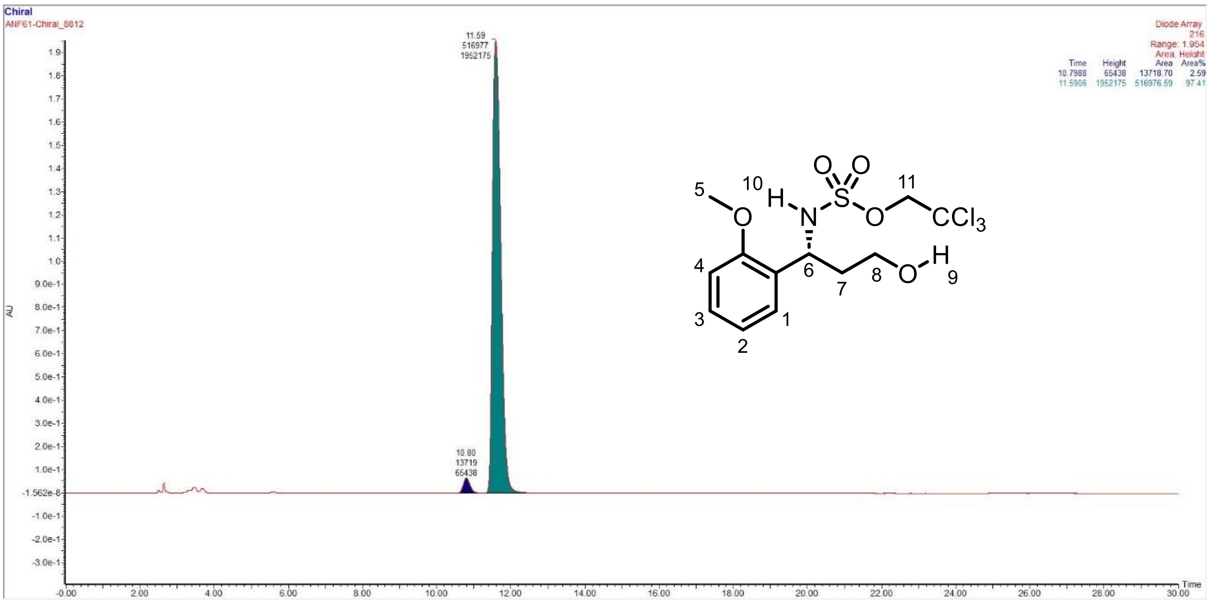

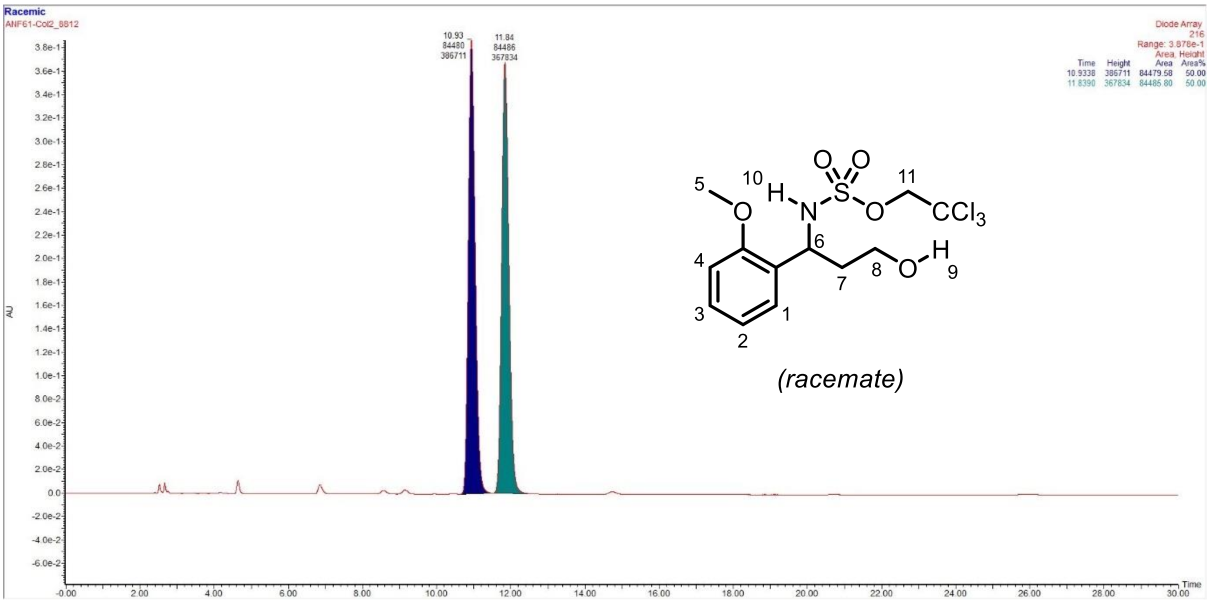

2,2,2-trichloroethyl (R)-(1-(2-chlorophenyl)-3-hydroxypropyl)sulfamate (**2e**)

Chiral SFC Analysis CHIRALPAK IG (CO_2:MeOH, 88.0:12.0, 2.50 mL min^{-1}, 40 °C, 210 nm) indicated 92% *ee*, t_R = 5.4 (minor), 5.7 (major) minutes.

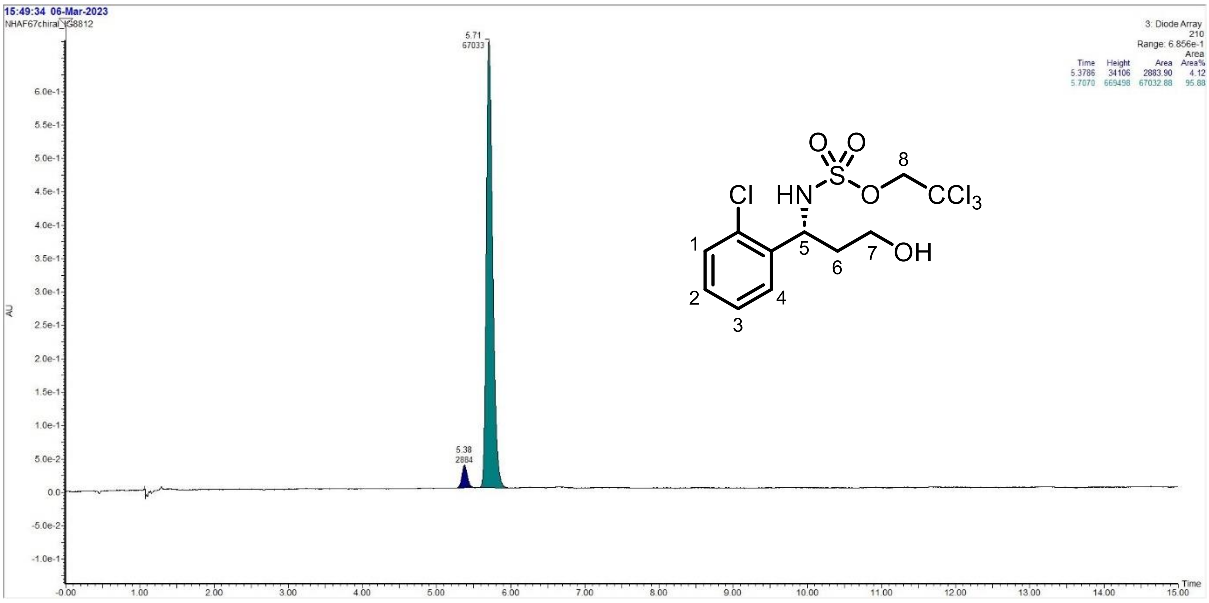

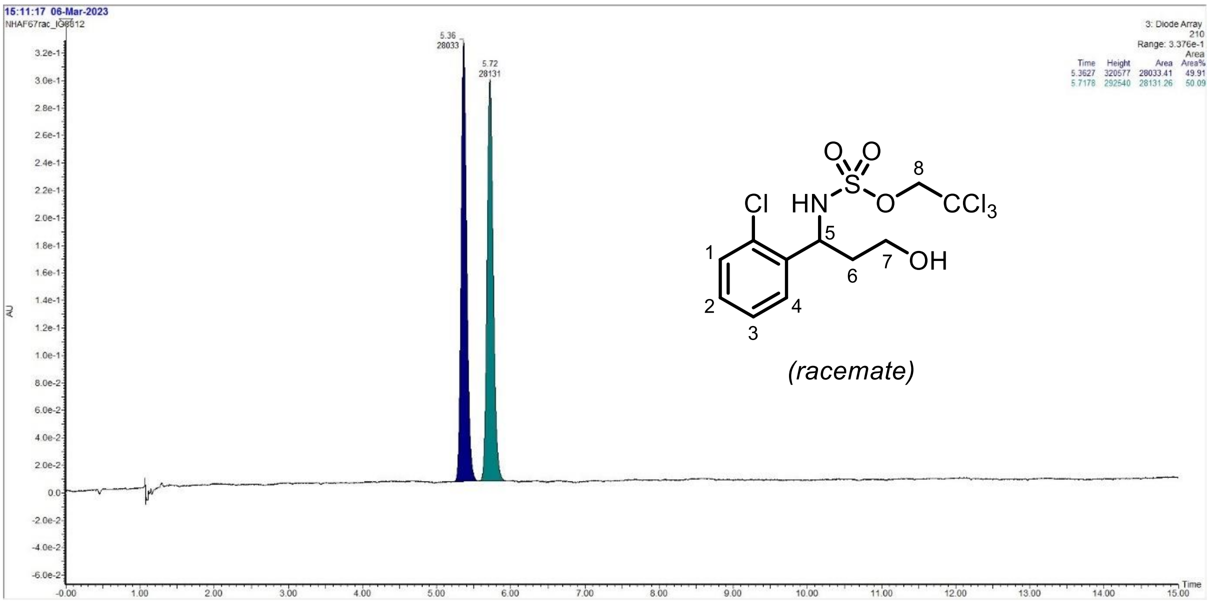

2,2,2-trichloroethyl (R)-(1-(2-bromophenyl)-3-hydroxypropyl)sulfamate (**2f**)

Chiral SFC Analysis CHIRALPAK IG (CO_2:*i*PrOH, 83.0:17.0, 2.50 mL min^{-1}, 40 °C, 206 nm) indicated 94% *ee*, t_R = 4.9 (major), 5.9 (minor) minutes.

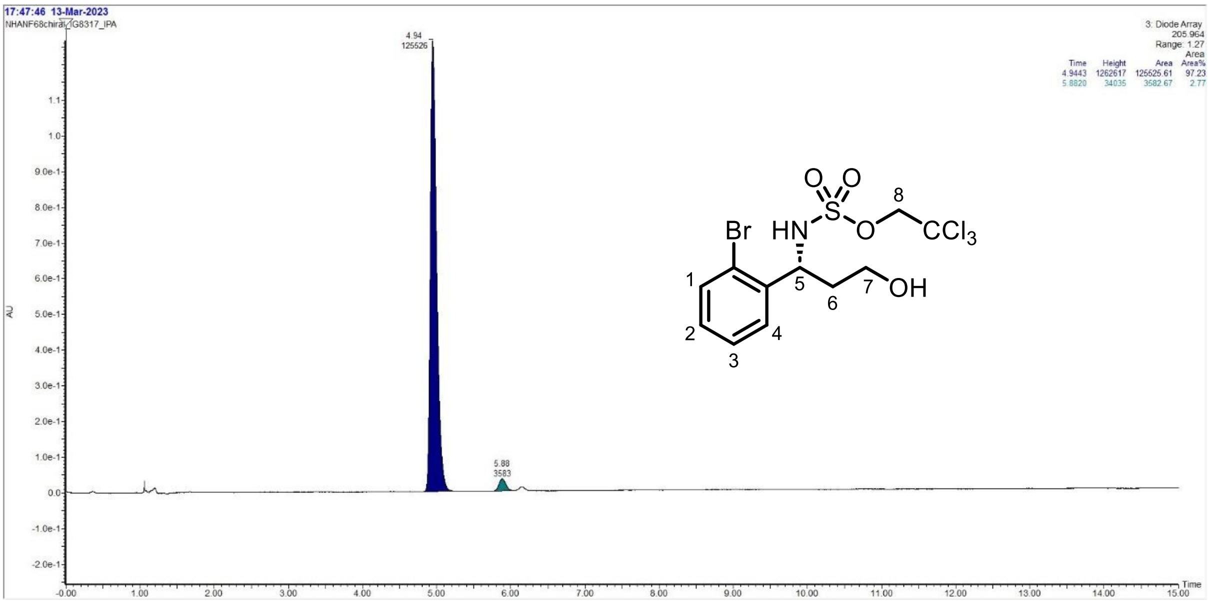

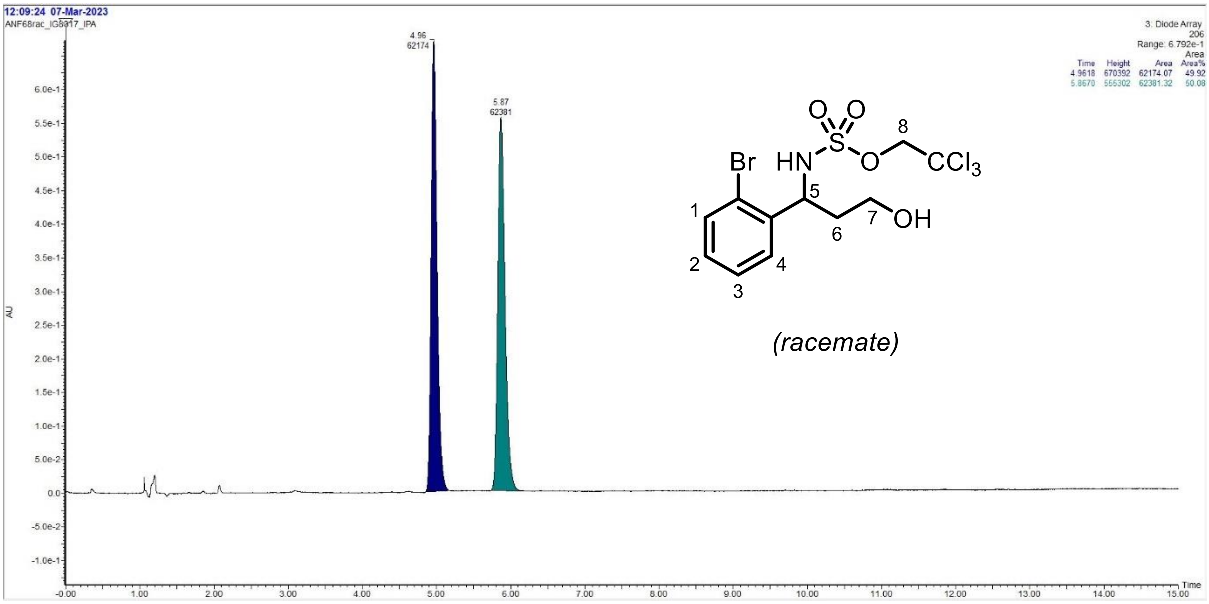

2,2,2-trichloroethyl (R)-(3-hydroxy-1-(m-tolyl)propyl)sulfamate (**2g**)

Chiral SFC Analysis CHIRALPAK IG (CO_2:MeOH, 88.0:12.0, 2.50 mL min^{-1}, 40 °C, 210 nm) indicated 93% *ee*, t_R = 5.0 (minor), 5.8 (major) minutes.

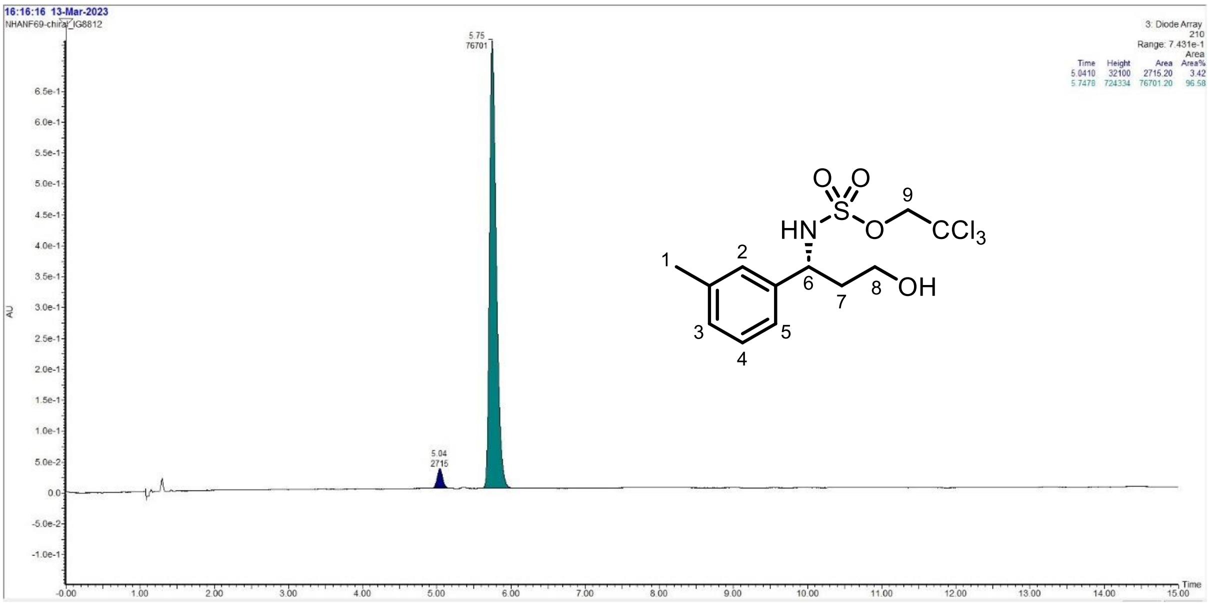

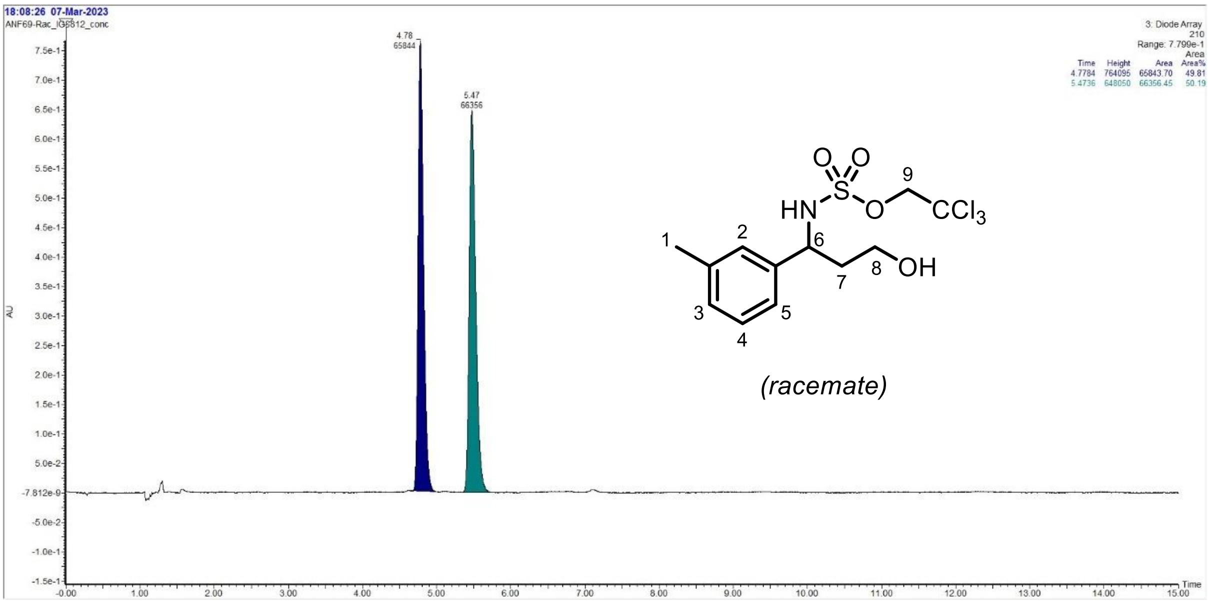

2,2,2-trichloroethyl (R)-(1-(3-(tert-butyl)phenyl)-3-hydroxypropyl)sulfamate (**2h**)

Chiral HPLC Analysis CHIRAL ART SC (hexane:*i*PrOH, 92.0:8.0, 1.25 mL min^{-1}, 40 °C, 210 nm) indicated 90% *ee*, t_R = 9.6 (minor), 10.6 (major) minutes.

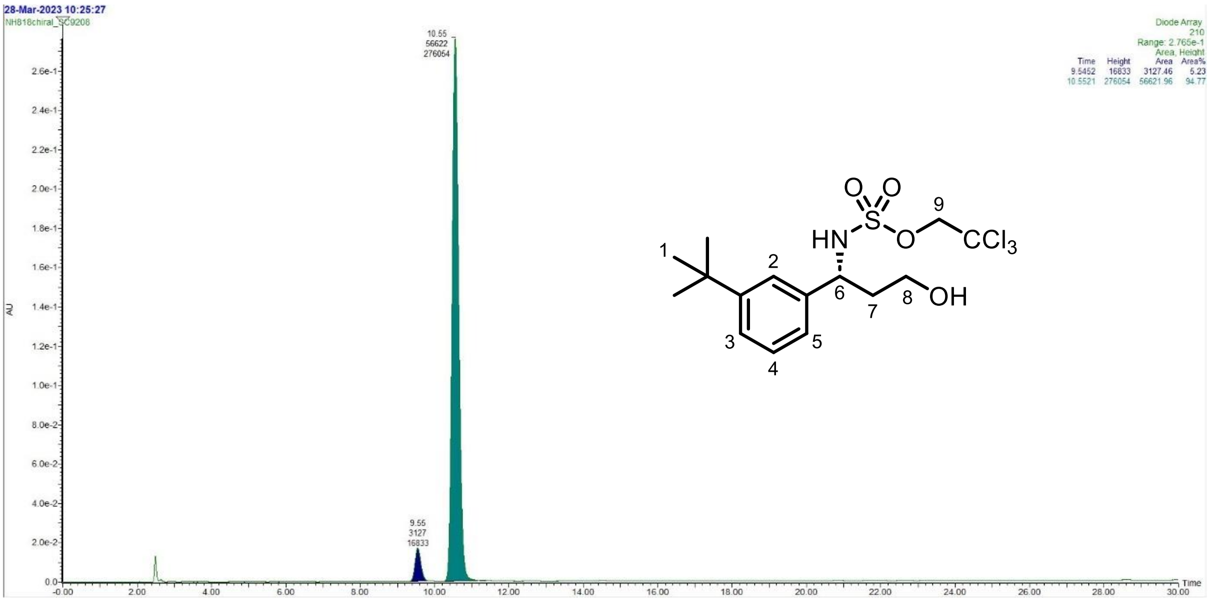

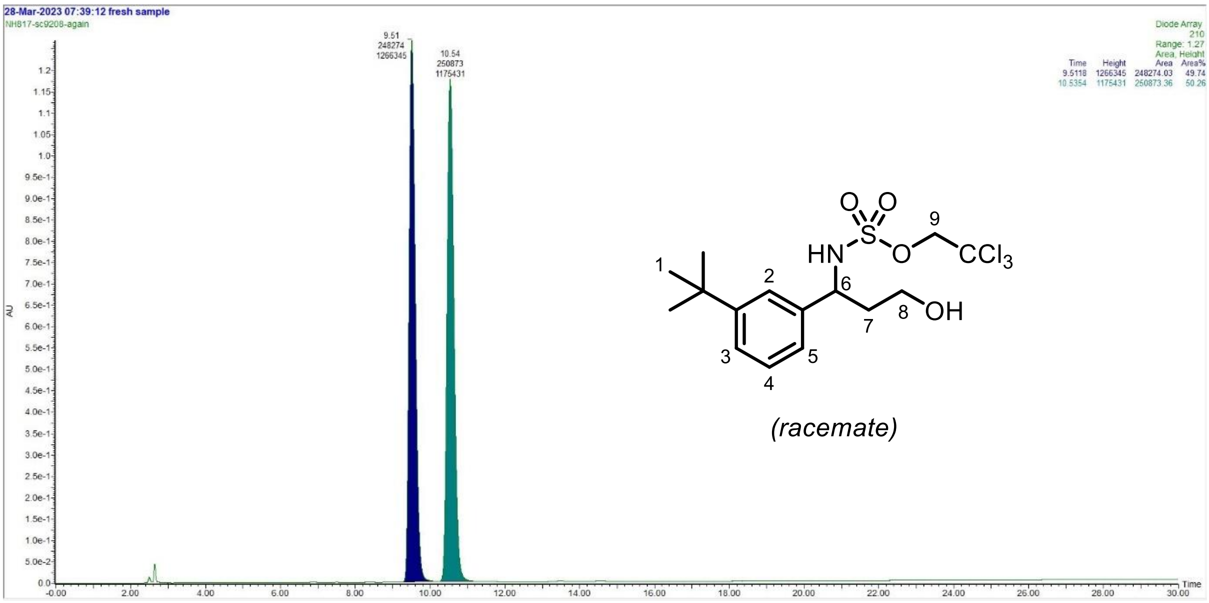

2,2,2-trichloroethyl (R)-(3-hydroxy-1-(3-(trifluoromethyl)phenyl)propyl)sulfamate (**2i**)

Chiral SFC Analysis CHIRALPAK IG (CO_2:MeOH, 93.0:7.0, 2.50 mL min^{-1}, 40 °C, 210 nm) indicated 93% *ee*, t_R = 4.5 (minor), 4.9 (major) minutes.

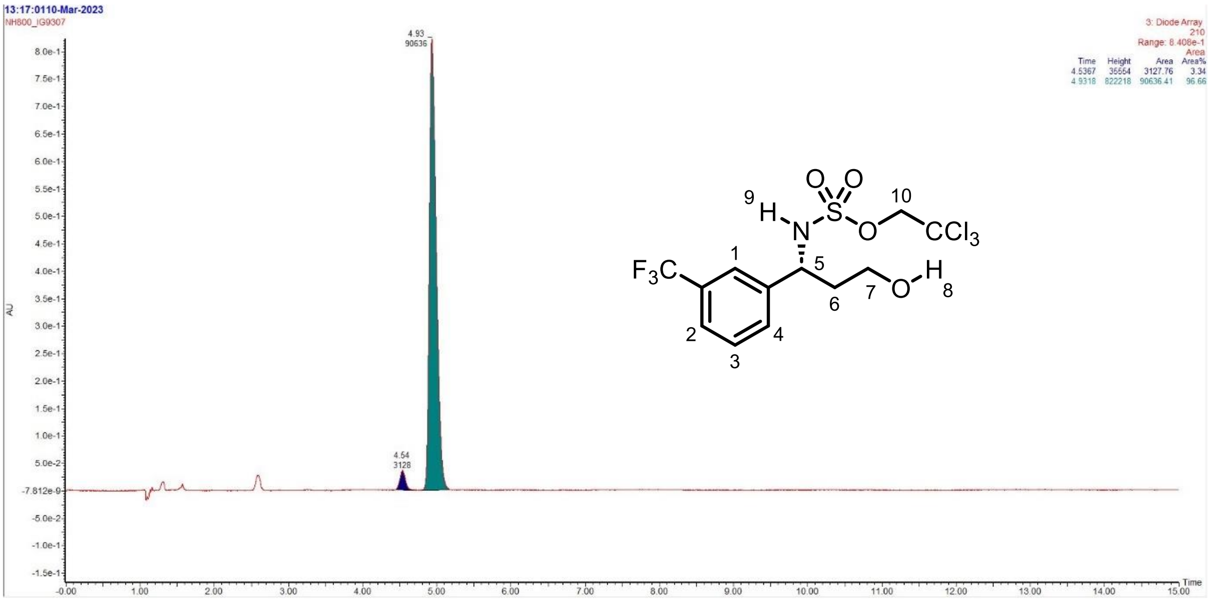

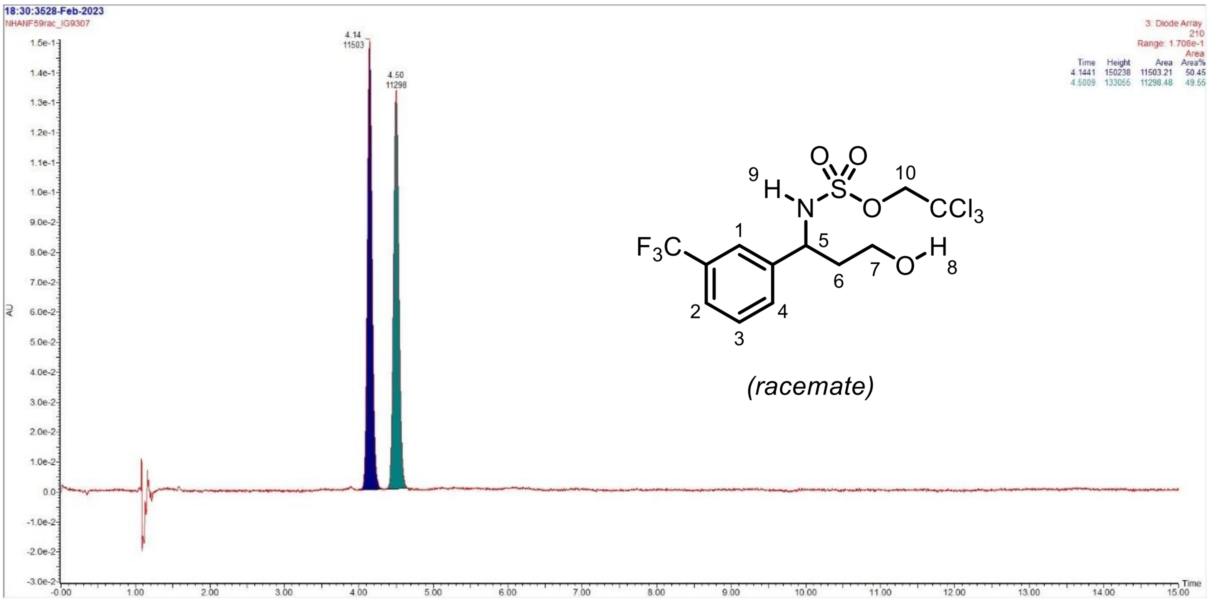

ethyl (R)-3-(3-hydroxy-1-(((2,2,2-trichloroethoxy)sulfonyl)amino)propyl)benzoate (**2j**)

Chiral SFC Analysis CHIRALPAK IG (CO_2:MeOH, 85.0:15.0, 2.50 mL min^{-1}, 40 °C, 229 nm) indicated 91% *ee*, t_R = 6.4 (minor), 6.8 (major) minutes.

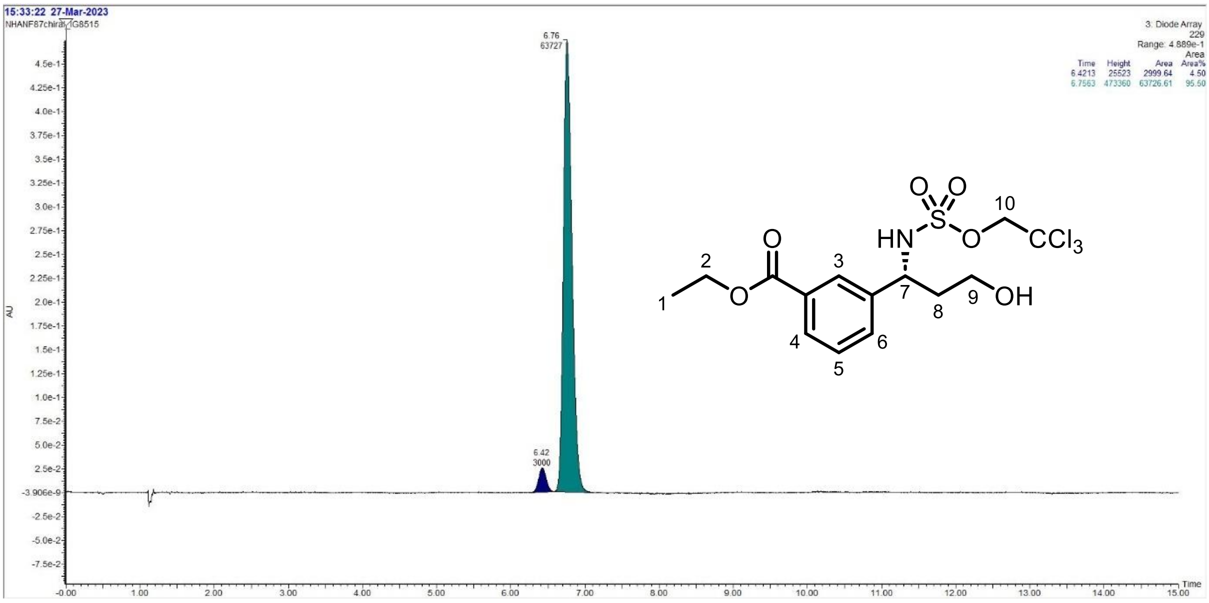

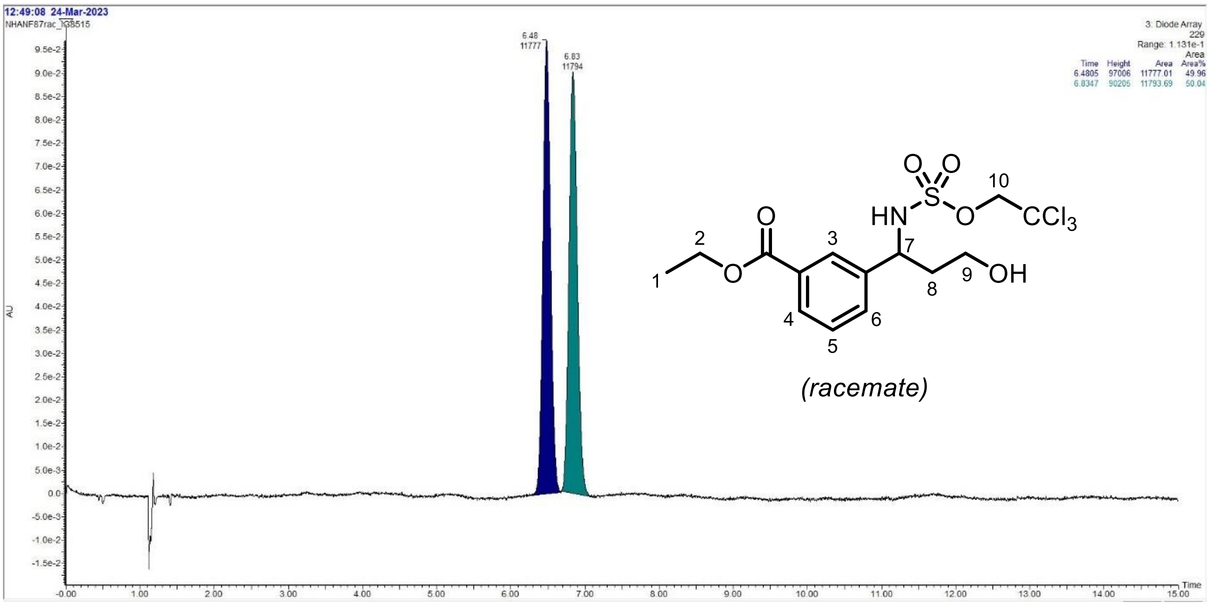

2,2,2-trichloroethyl (R)-(3-hydroxy-1-(naphthalen-1-yl)propyl)sulfamate (**2k**)

Chiral SFC Analysis CHIRALPAK IK (CO_2:MeOH, 85.0:15.0, 2.50 mL min^{-1}, 40 °C, 210 nm) indicated 89% *ee*, t_R = 5.6 (minor), 6.1 (major) minutes.

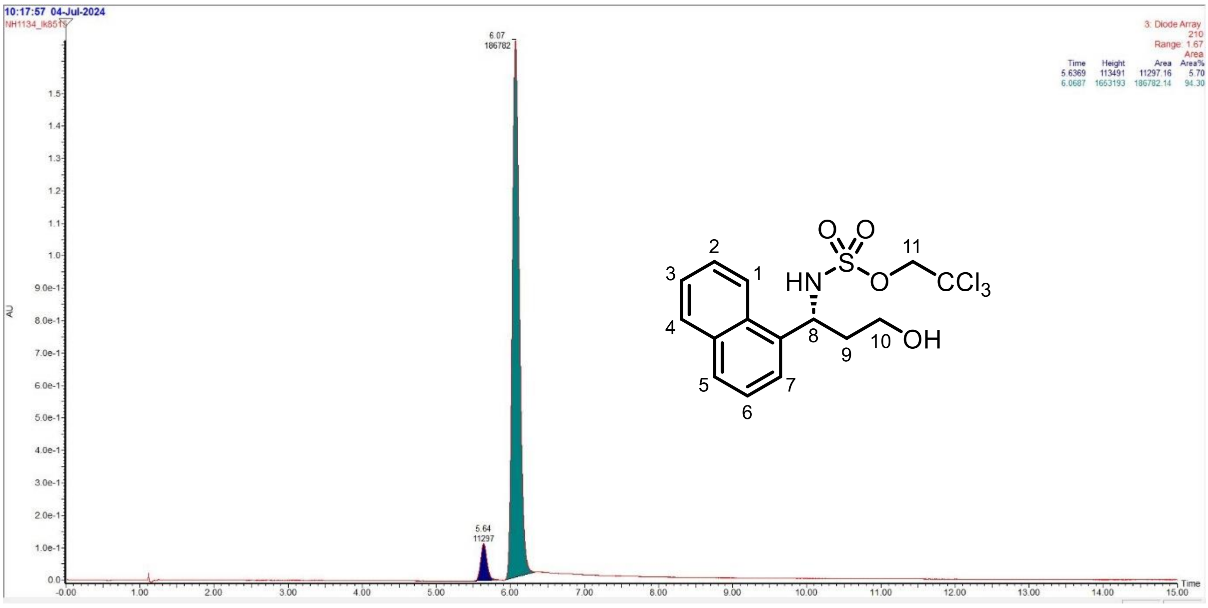

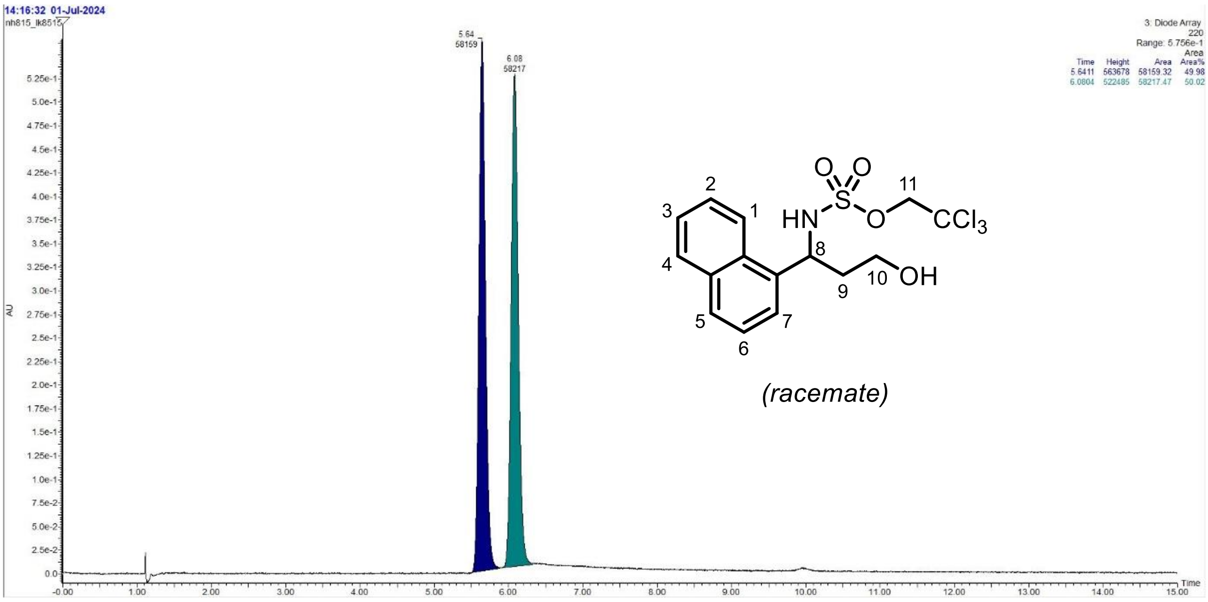

2,2,2-trichloroethyl (R)-(1-(4-chlorophenyl)-3-hydroxypropyl)sulfamate (**2l**)

Chiral SFC Analysis CHIRALPAK IG (CO_2:MeOH, 85.0:15.0, 2.50 mL min^{-1}, 40 °C, 219 nm) indicated 87% *ee*, t_R = 5.1 (minor), 7.3 (major) minutes.

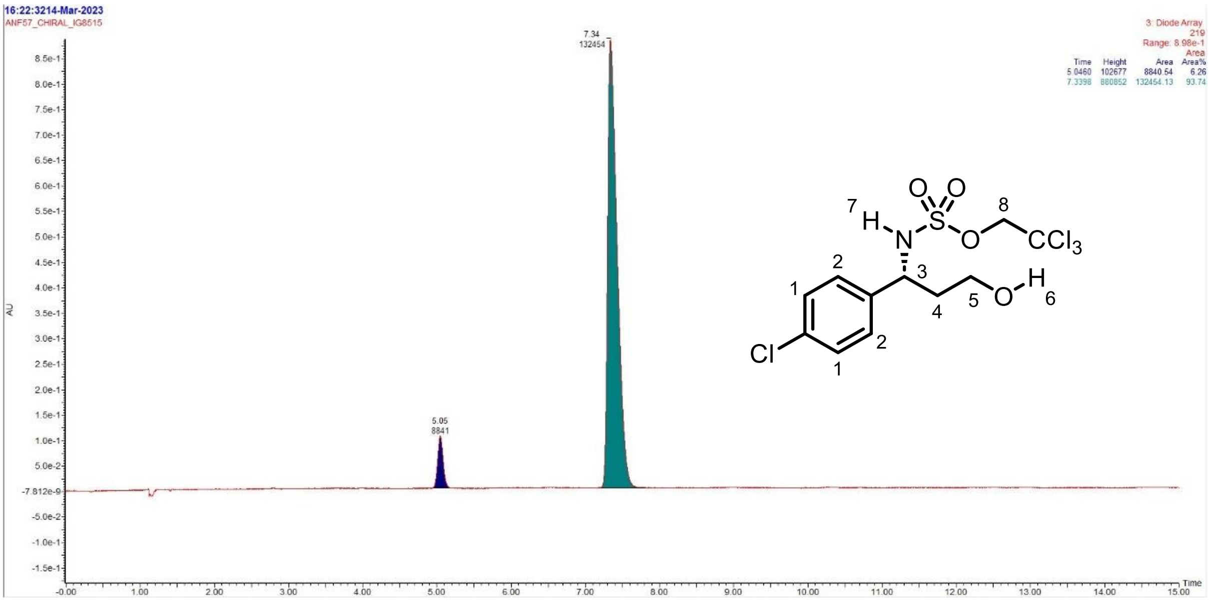

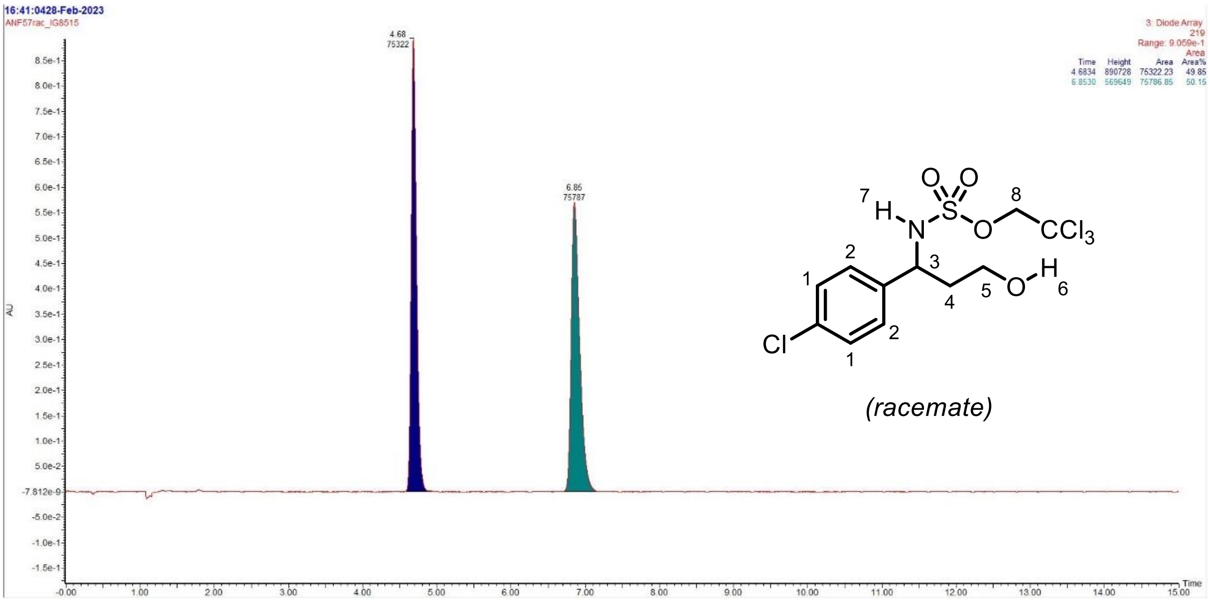

2,2,2-trichloroethyl (R)-(3-hydroxy-1-(4-methoxyphenyl)propyl)sulfamate (**2m**)

Chiral SFC Analysis CHIRALPAK IG (CO_2:MeOH, 83.0:17.0, 2.50 mL min^{-1}, 40 °C, 224 nm) indicated 88% *ee*, t_R = 4.8 (minor), 7.6 (major) minutes.

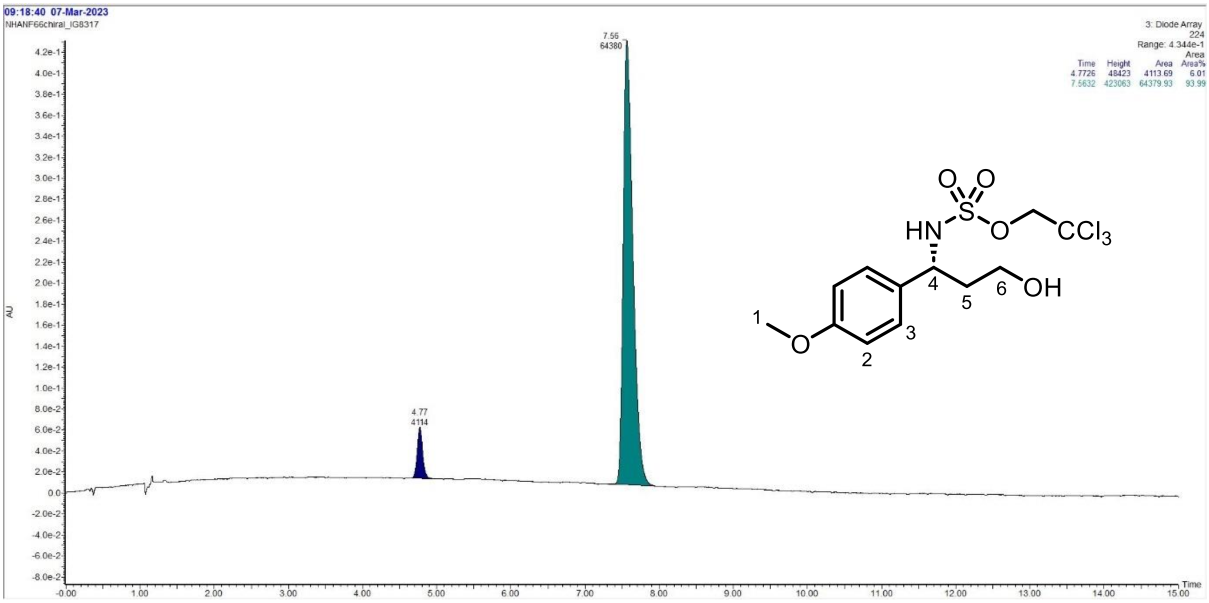

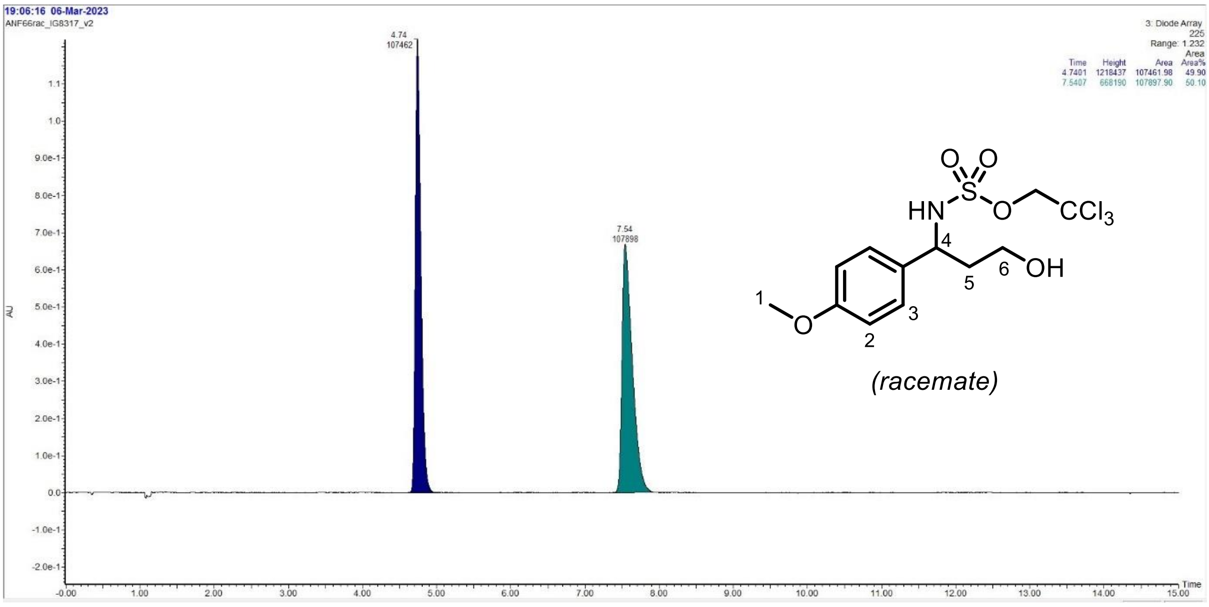

2,2,2-trichloroethyl (R)-(1-(4-bromophenyl)-3-hydroxypropyl)sulfamate (**2n**)

Chiral SFC Analysis CHIRALPAK IG (CO_2:MeOH, 85.0:15.0, 2.50 mL min^{-1}, 40 °C, 220 nm) indicated 92% *ee*, t_R = 5.7 (minor), 9.1 (major) minutes.

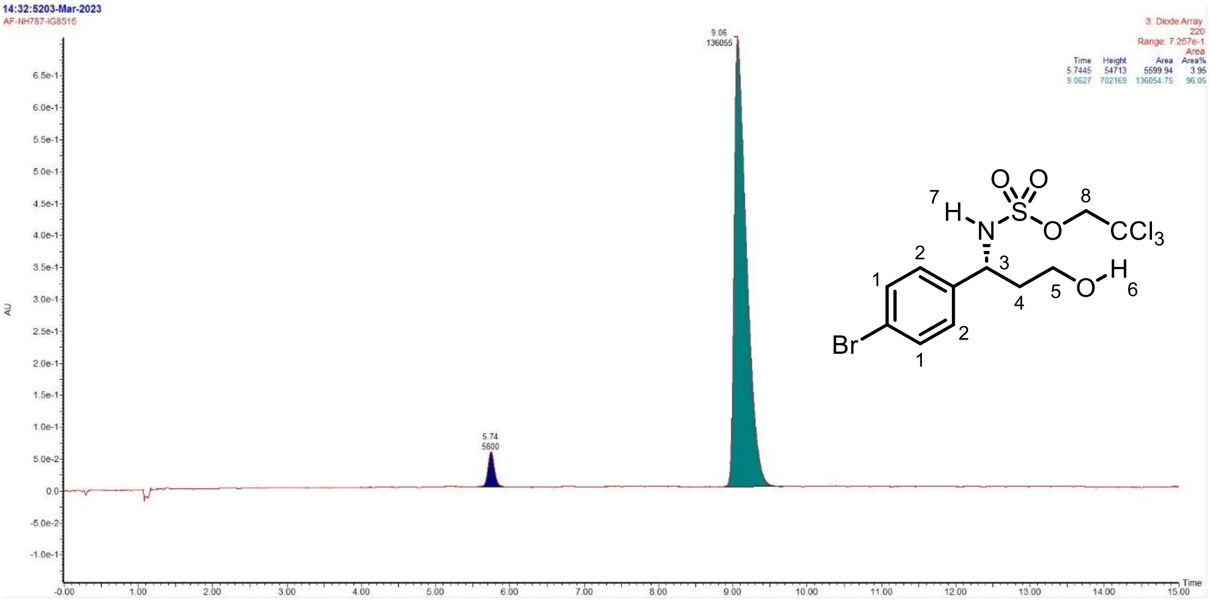

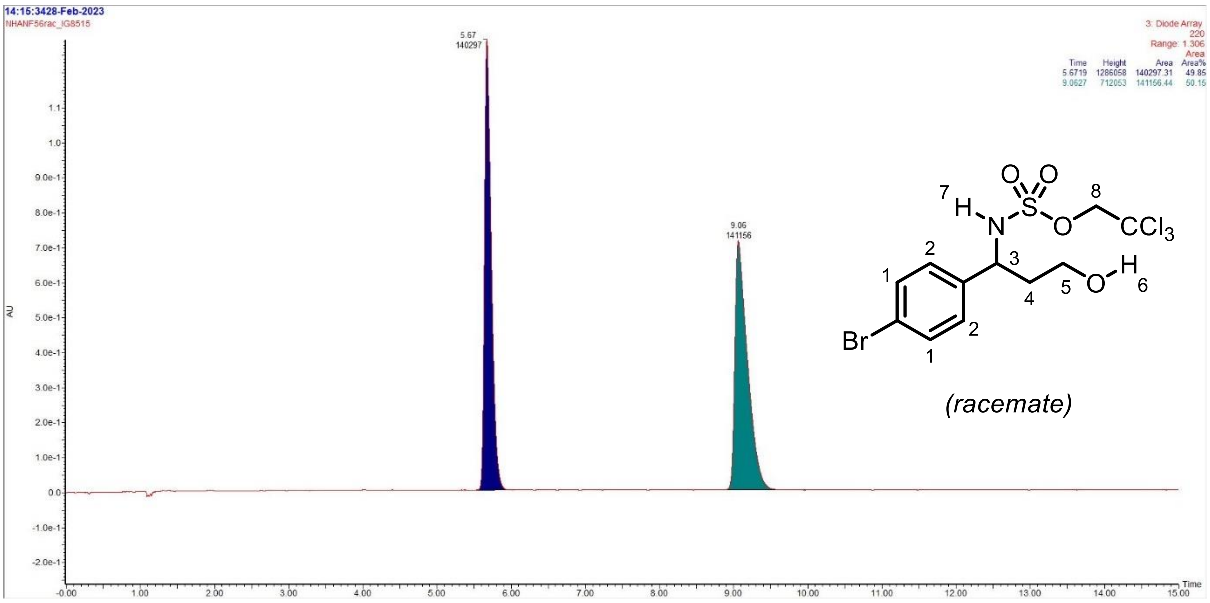

2,2,2-trichloroethyl (R)-(3-hydroxy-1-(4-(trifluoromethyl)phenyl)propyl)sulfamate (**2o**)

Chiral SFC Analysis CHIRALPAK IG (CO_2:MeOH, 93.0:7.0, 2.50 mL min^{-1}, 40 °C, 213 nm) indicated 85% *ee*, t_R = 6.6 (minor), 11.3 (major) minutes.

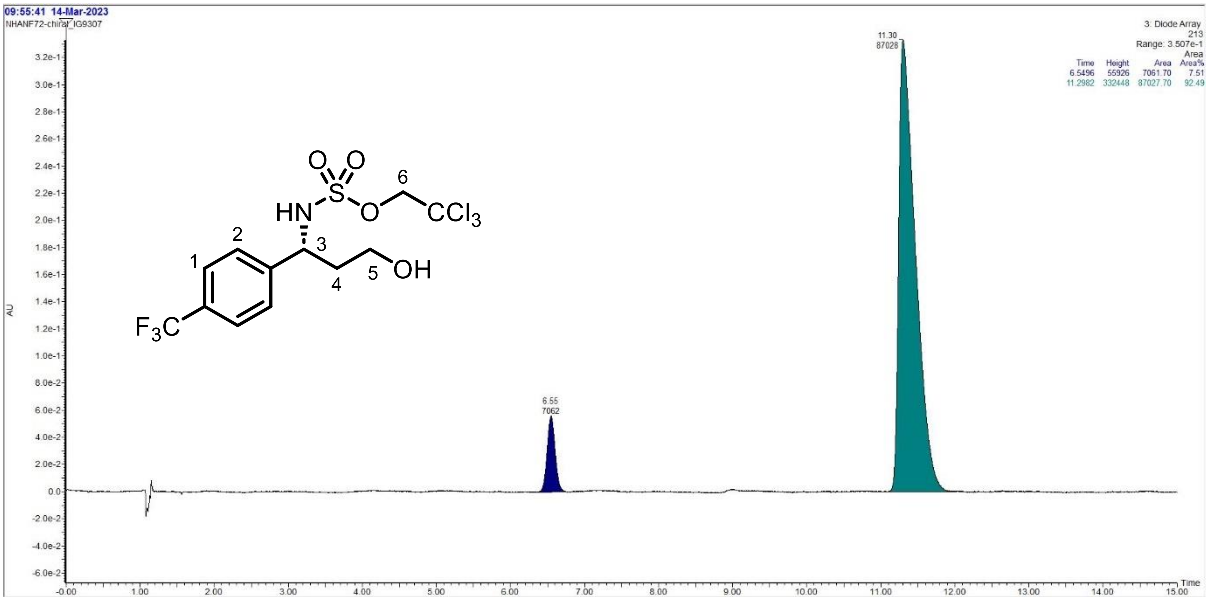

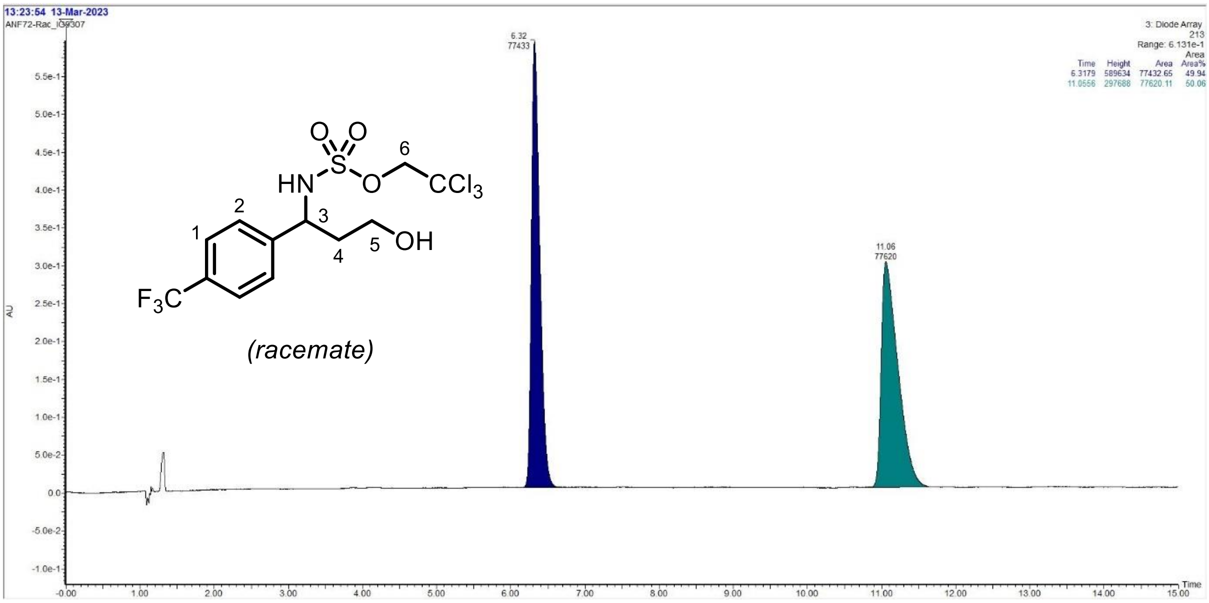

2,2,2-trichloroethyl (S)-(3-hydroxy-2,2-dimethyl-1-phenylpropyl)sulfamate (**2p**)

Chiral SFC Analysis CHIRALPAK IG (CO_2:MeOH, 90.0:10.0, 2.50 mL min^{-1}, 40 °C, 210 nm) indicated 98% *ee*, t_R = 6.6 (minor), 8.8 (major) minutes.

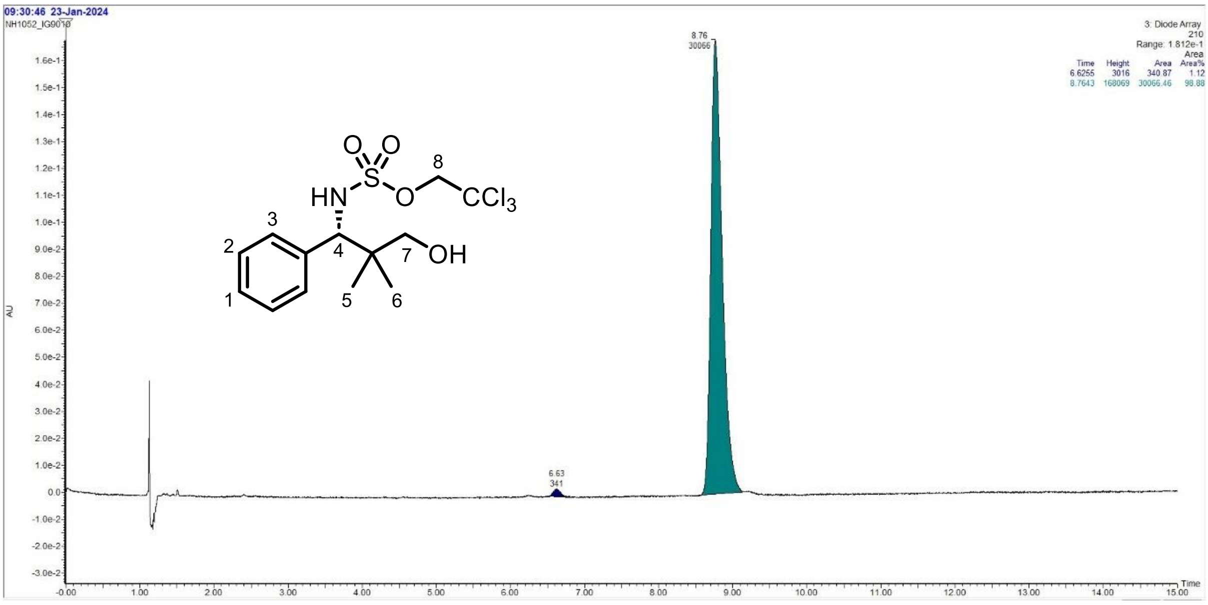

Note: only traces of product were detected when $Rh_2(esp)_2$ was used as an achiral catalyst. Due to this weaker intensity racemic trace (lower trace, below), an ES+ mass spectrometry trace for $[M+Na]^+$ = 412 m/z is shown (higher trace, below).

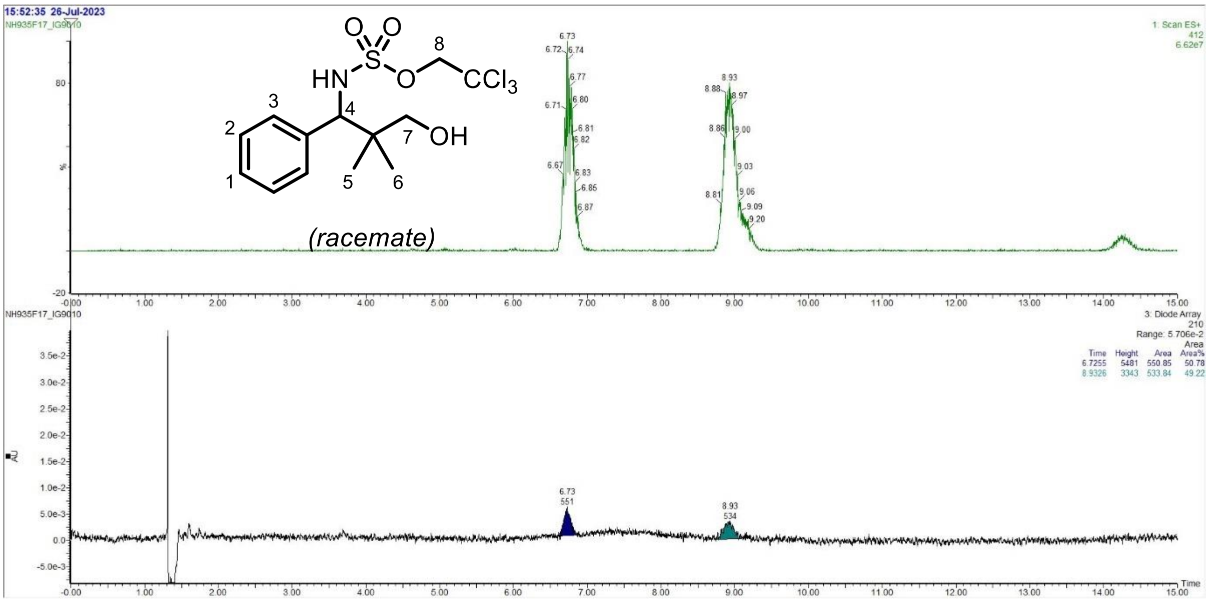

2,2,2-trichloroethyl (S)-((1-(hydroxymethyl)cyclopentyl)(phenyl)methyl)sulfamate (**2q**)

Chiral SFC Analysis CHIRALPAK IG (CO_2:MeOH, 85.0:15.0, 1.25 mL min^{-1}, 40 °C, 210 nm) indicated 97% *ee*, t_R = 12.8 (minor), 13.9 (major) minutes.

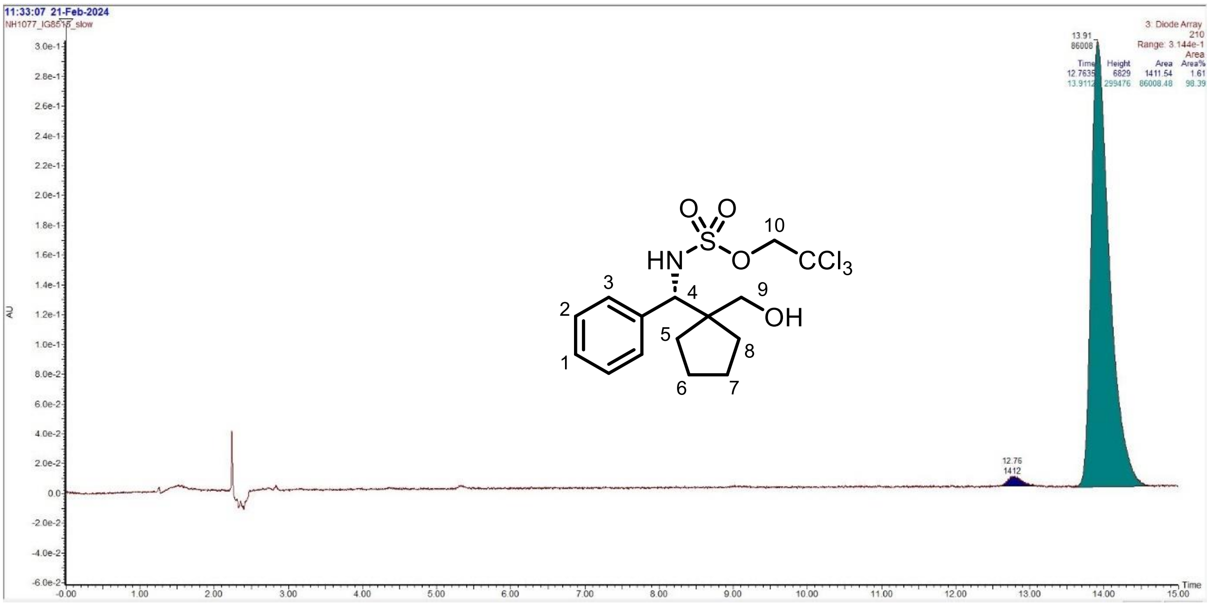

Note: only traces of product were detected when $Rh_2(esp)_2$ was used as an achiral catalyst. Due to this weaker intensity racemic trace (lower trace, below), an ES+ mass spectrometry trace for $[M+Na]^+$ = 438 m/z is shown (higher trace, below).

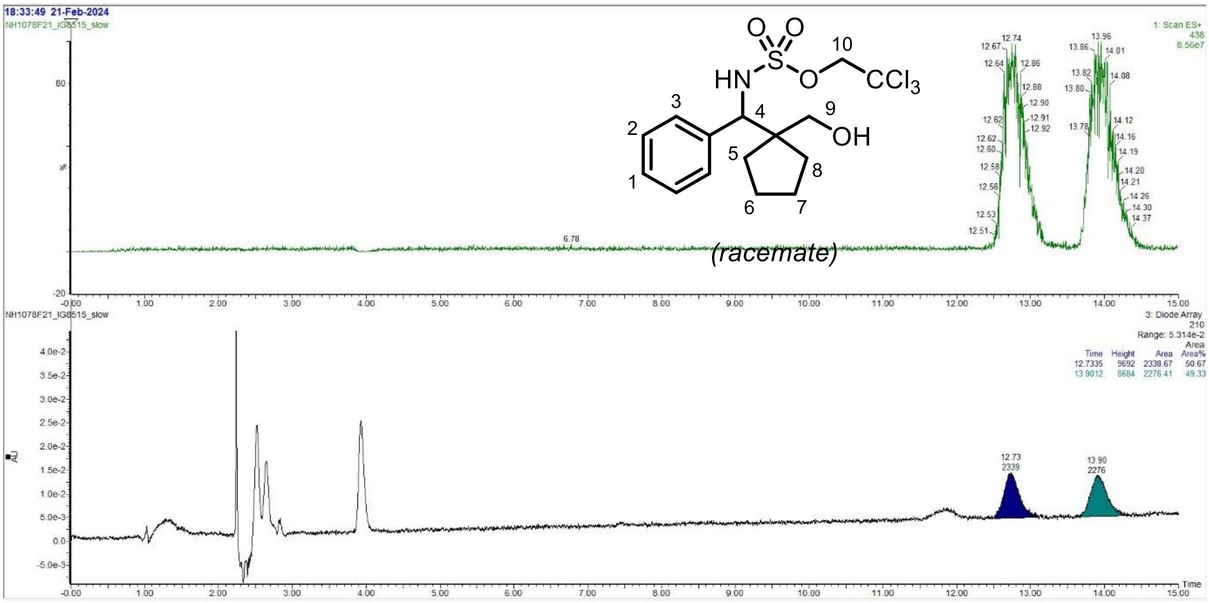

2,2,2-trichloroethyl (R)-(3-hydroxy-3-methyl-1-phenylbutyl)sulfamate (**2r**)

Chiral SFC Analysis CHIRALPAK IG (CO_2:MeOH, 90.0:10.0, 2.50 mL min^{-1}, 40 °C, 210 nm) indicated 85% *ee*, t_R = 5.8 (minor), 6.5 (major) minutes.

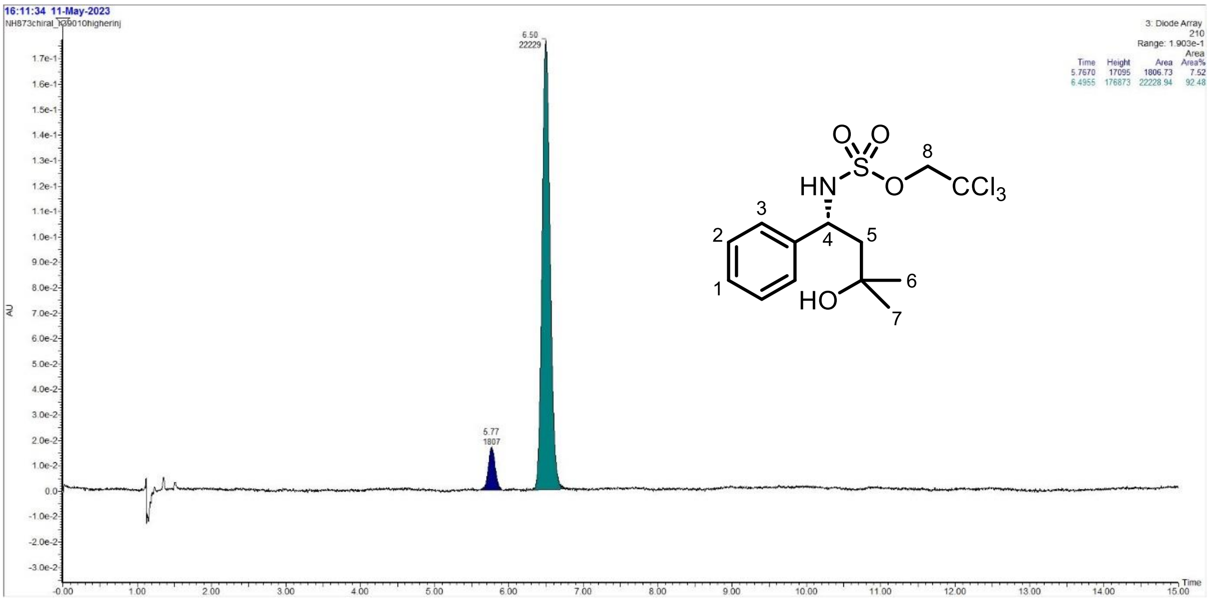

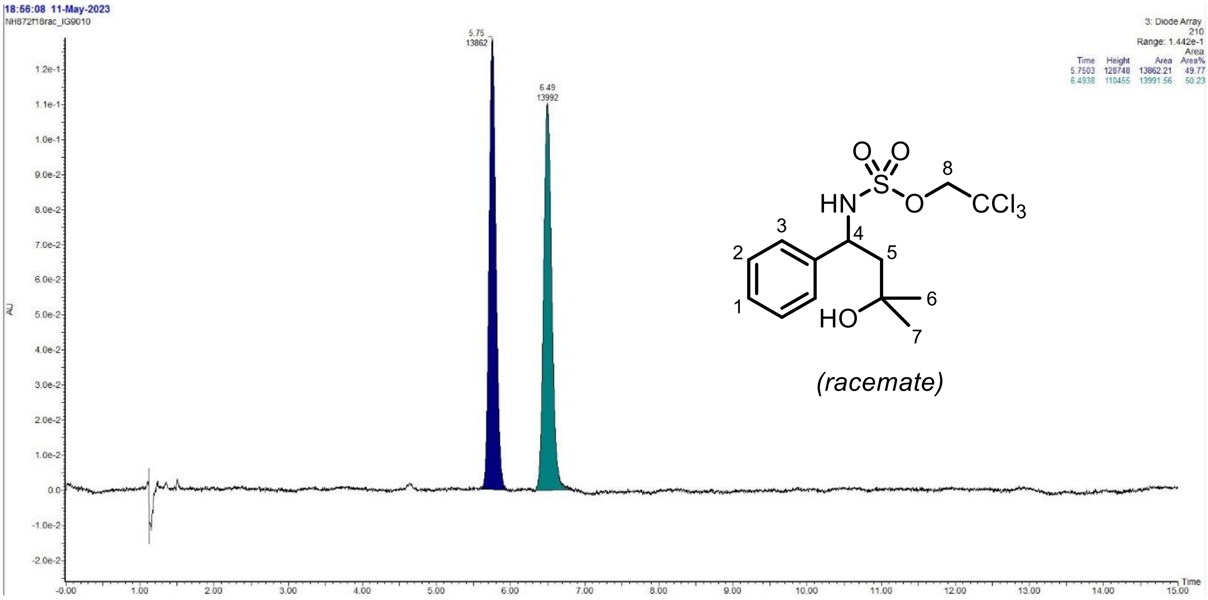

2,2,2-trichloroethyl (R)-(2-(1-hydroxycyclobutyl)-1-phenylethyl)sulfamate (**2s**)

Chiral SFC Analysis CHIRALPAK IC (CO_2:MeOH, 90.0:10.0, 2.50 mL min^{-1}, 40 °C, 210 nm) indicated 93% *ee*, t_R = 4.4 (minor), 4.8 (major) minutes.

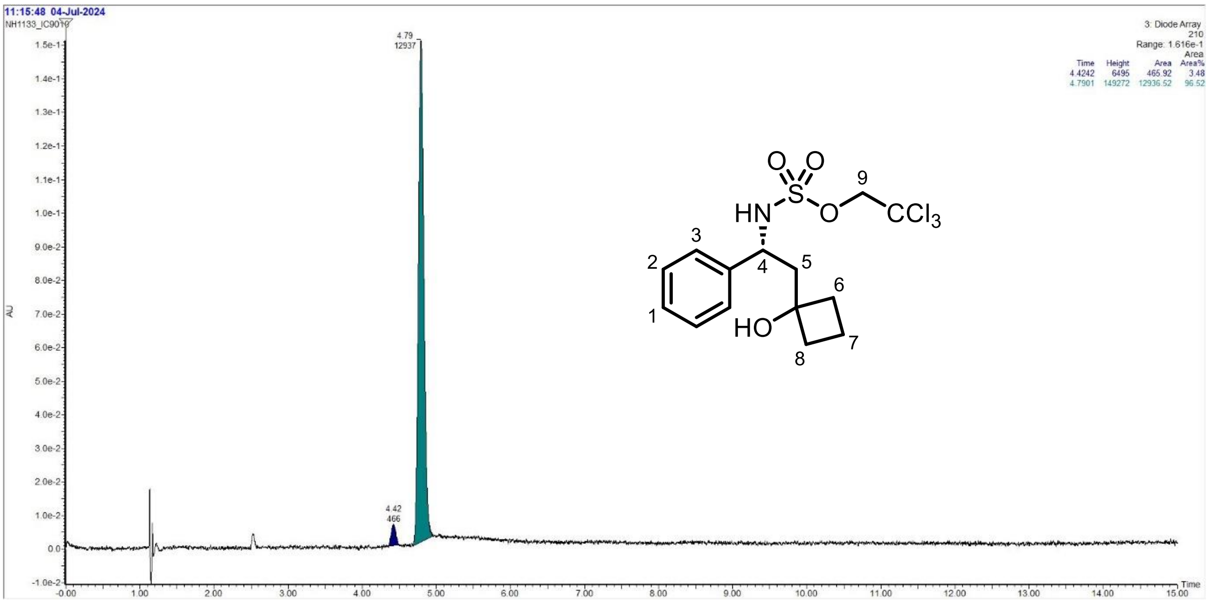

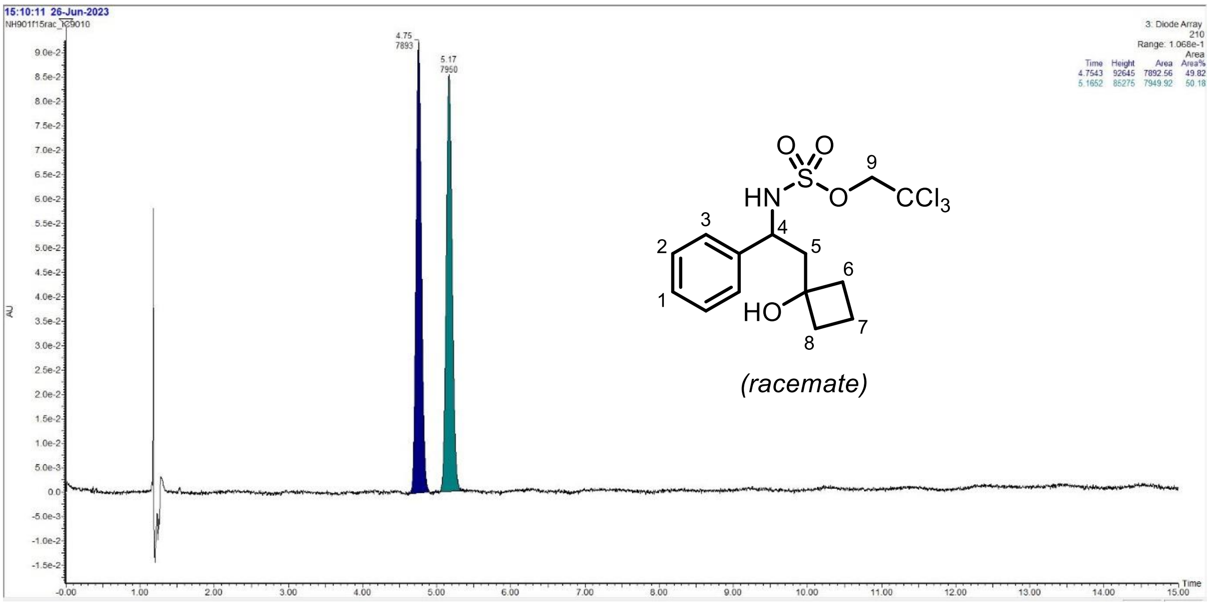

2,2,2-trichloroethyl (R)-(3-hydroxy-1-(thiophen-2-yl)propyl)sulfamate (**2t**)

Chiral SFC Analysis CHIRALPAK IG (CO_2:MeOH, 85.0:15.0, 2.50 mL min^{-1}, 40 °C, 232 nm) indicated 88% *ee*, t_R = 6.1 (minor), 9.6 (major) minutes.

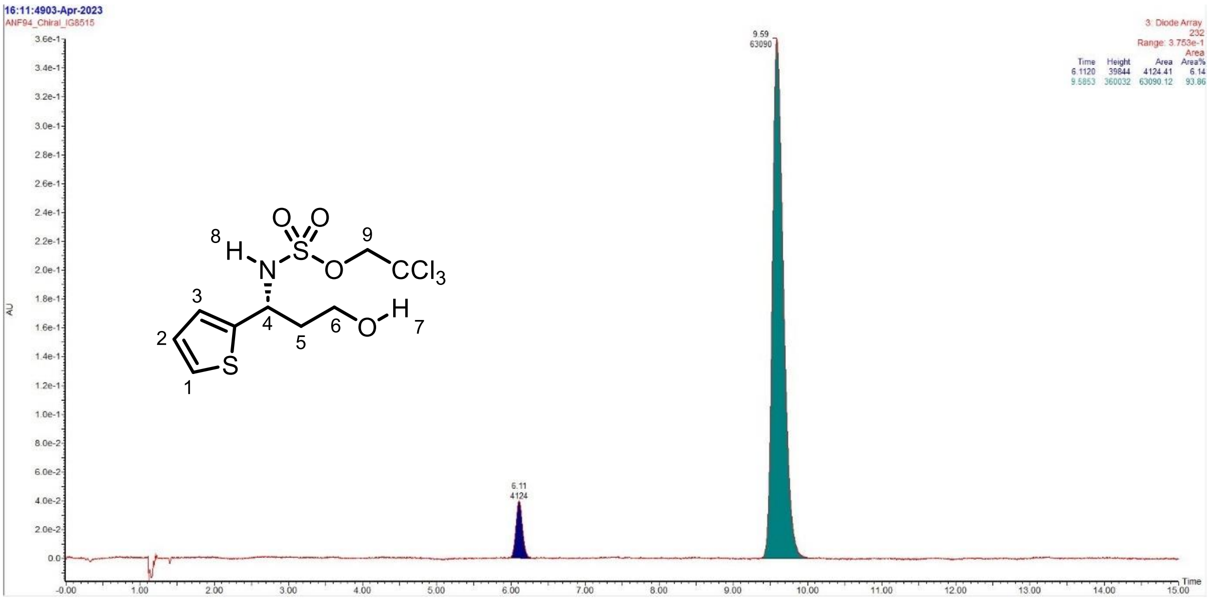

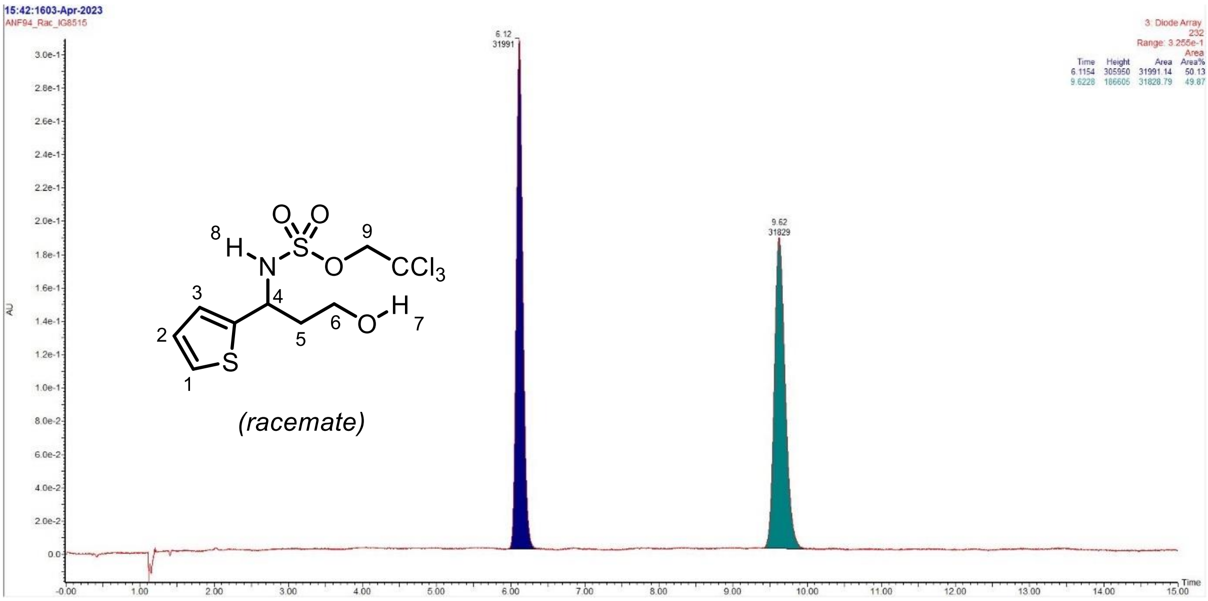

2,2,2-trichloroethyl (R)-(1-(benzo[b]thiophen-5-yl)-3-hydroxypropyl)sulfamate (**2u**)

Chiral SFC Analysis CHIRALPAK IG (CO_2:MeOH, 80.0:20.0, 2.50 mL min^{-1}, 40 °C, 229 nm) indicated 86% *ee*, t_R = 5.6 (minor), 7.5 (major) minutes.

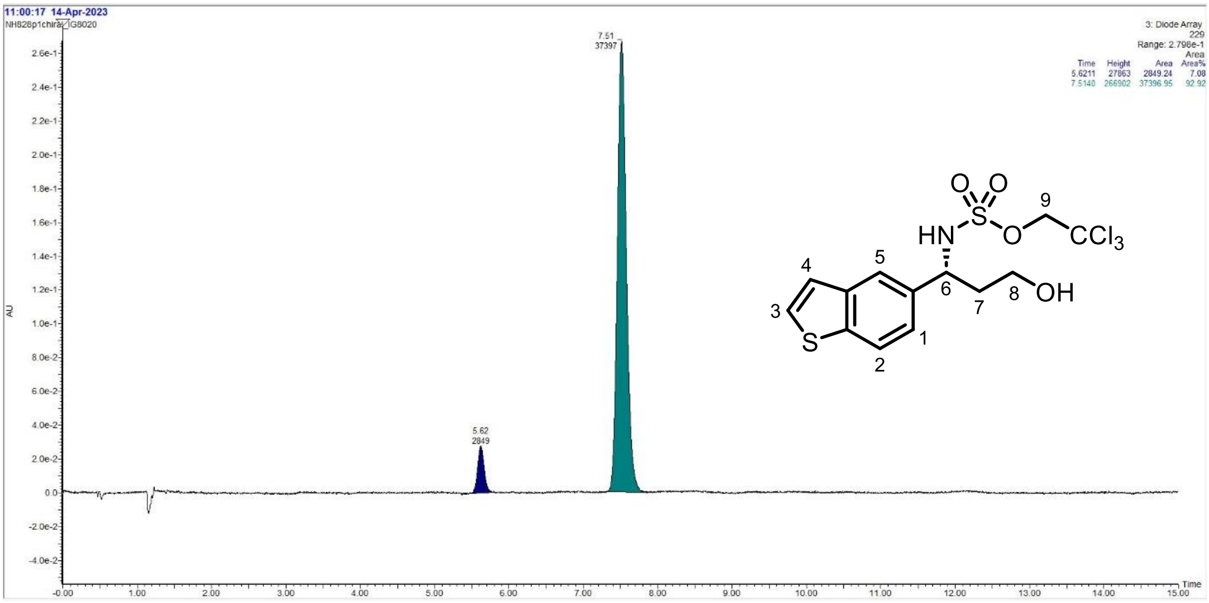

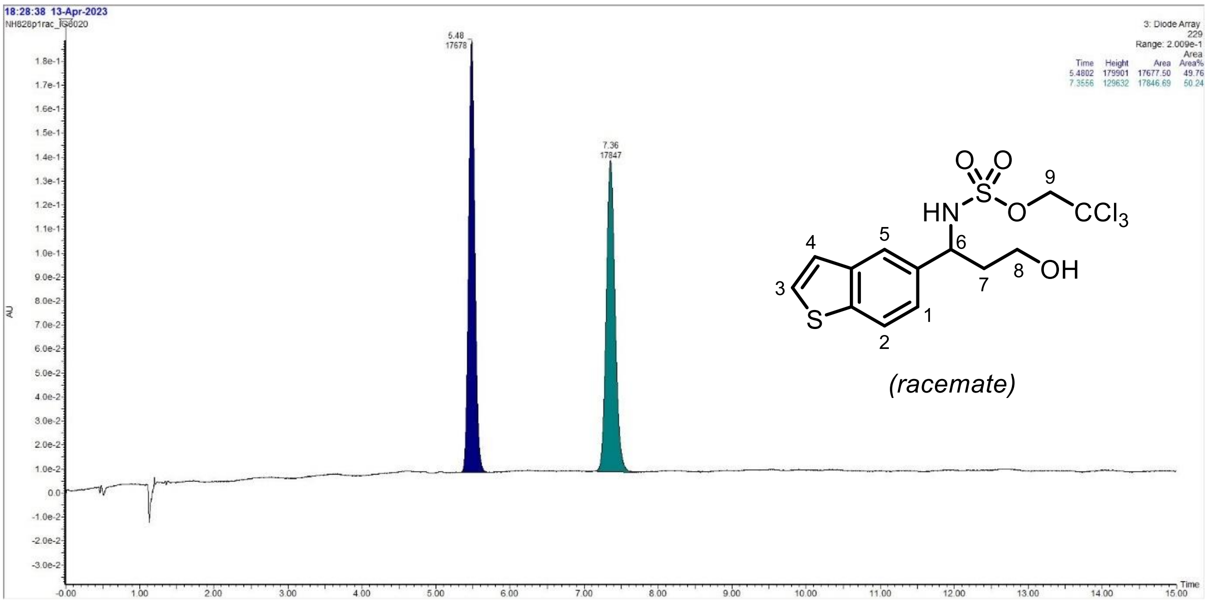

2,2,2-trichloroethyl (R)-(1-(benzo[b]thiophen-2-yl)-3-hydroxypropyl)sulfamate (**2v**)

Chiral SFC Analysis CHIRALPAK IG (CO_2:MeOH, 80.0:20.0, 2.50 mL min^{-1}, 40 °C, 227 nm) indicated 88% *ee*, t_R = 6.7 (minor), 11.8 (major) minutes.

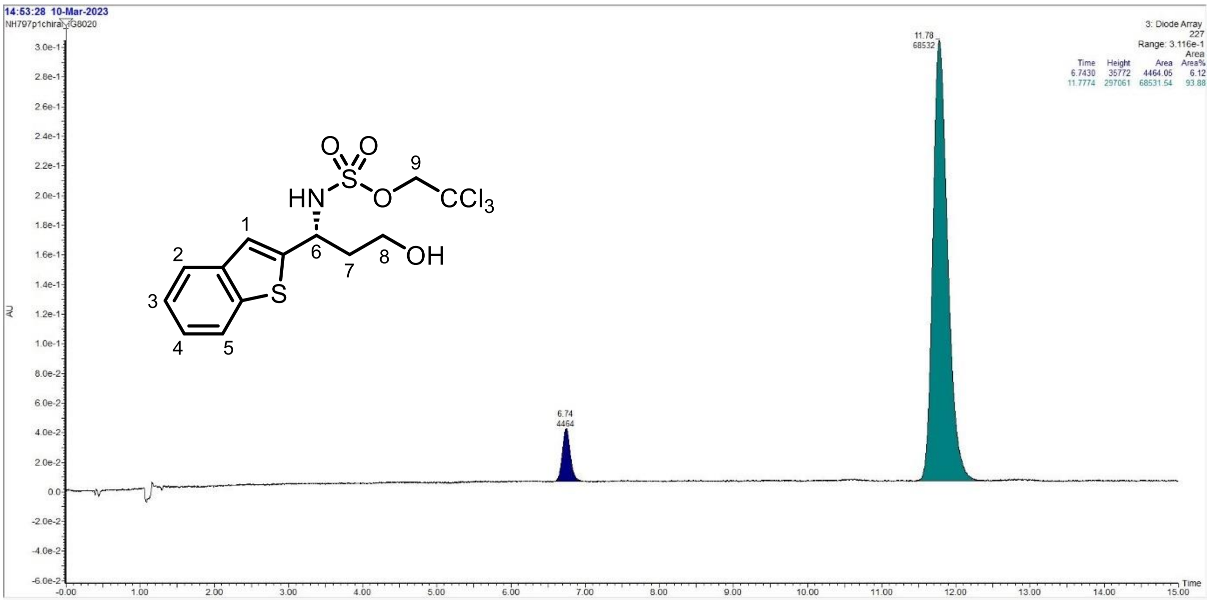

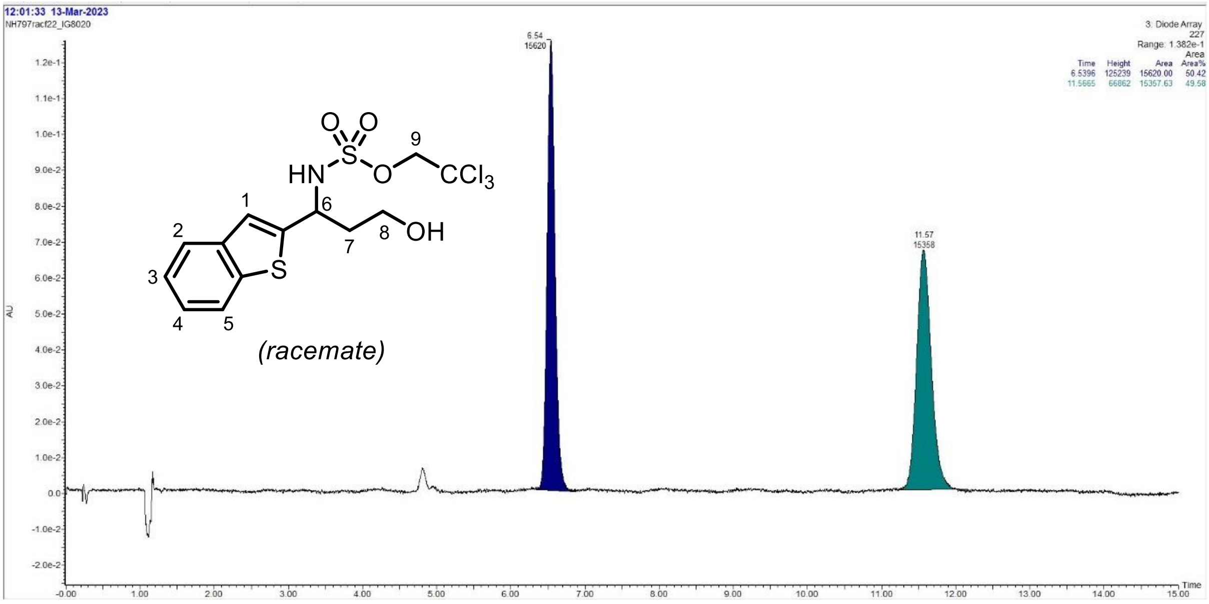

2,2,2-trichloroethyl (R)-(3-hydroxy-1-(1-(phenylsulfonyl)-1H-pyrrol-3-yl)propyl)sulfamate (**2w**)

Chiral SFC Analysis CHIRALPAK IG (CO_2:MeOH, 80.0:20.0, 2.50 mL min^{-1}, 40 °C, 232 nm) indicated 89% *ee*, t_R = 5.6 (minor), 8.2 (major) minutes.

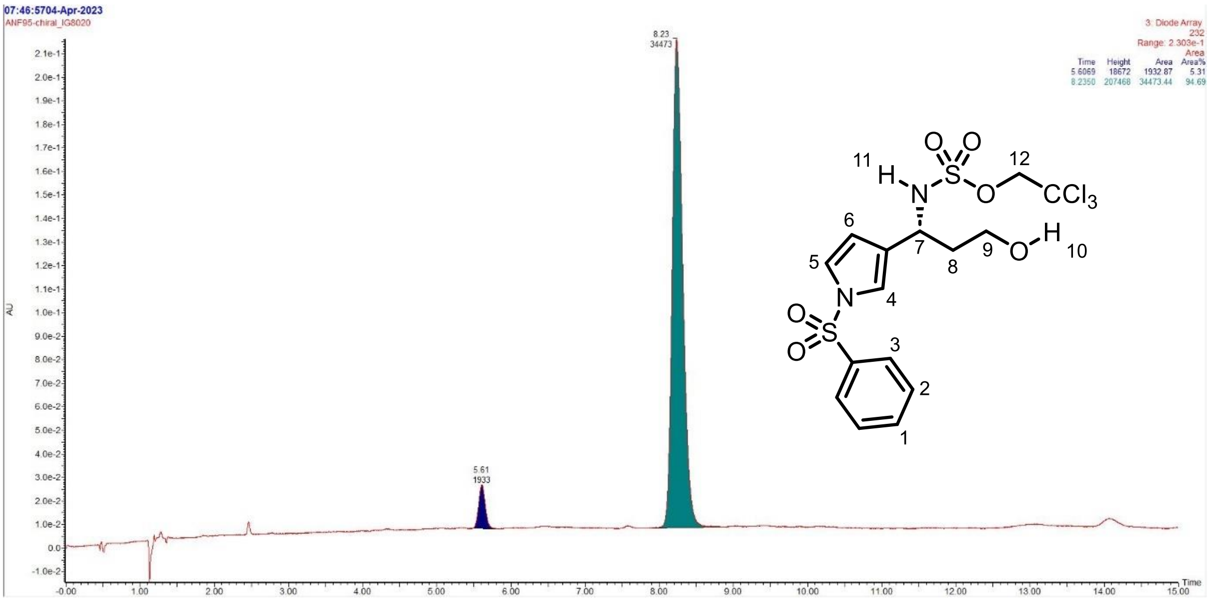

*Note: only traces of **rac-2w** were detected when $Rh_2(esp)_2$ was used as an achiral catalyst. Since a racemic trace could not be obtained, below is a trace for the opposite enantiomer (2,2,2-trichloroethyl (S)-(3-hydroxy-1-(1-(phenylsulfonyl)-1H-pyrrol-3-yl)propyl)sulfamate) obtained using catalyst* Rh_2(**A-III**)$_2$•(**Cat5**)$_2$•(**pyr**)$_2$ (**Rh2**)*, in (−)82% ee.*

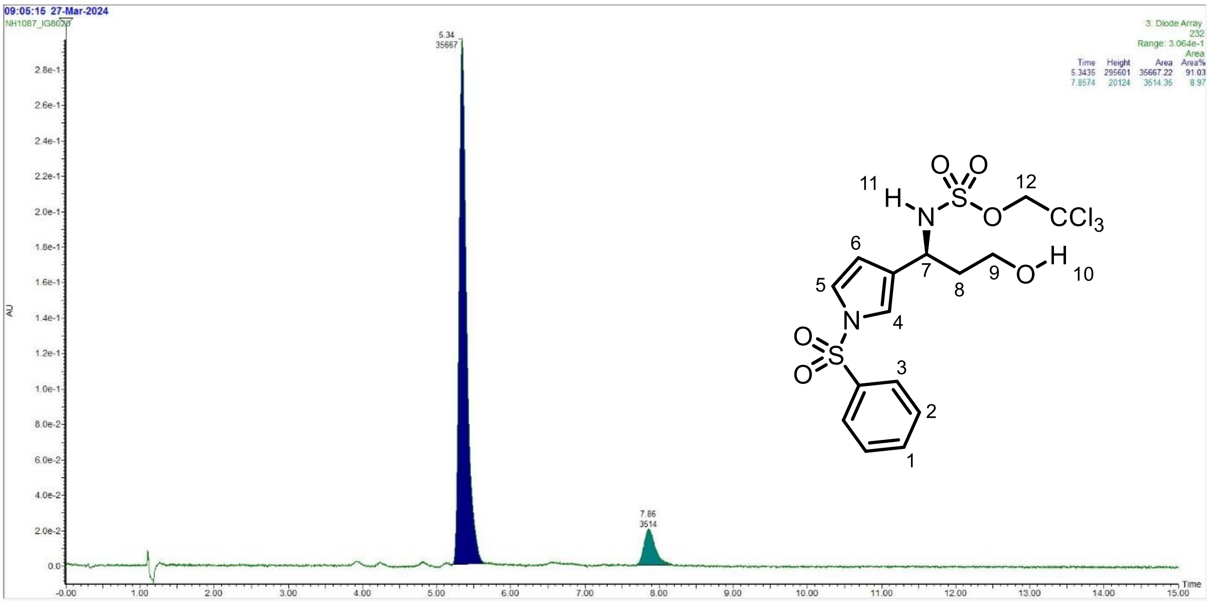

2,2,2-trichloroethyl (R)-(1-(benzofuran-2-yl)-3-hydroxypropyl)sulfamate (**2x**)

Chiral SFC Analysis CHIRALPAK IG (CO_2:MeOH, 85.0:15.0, 2.50 mL min^{-1}, 40 °C, 244 nm) indicated 87% *ee*, t_R = 5.7 (minor), 7.9 (major) minutes.

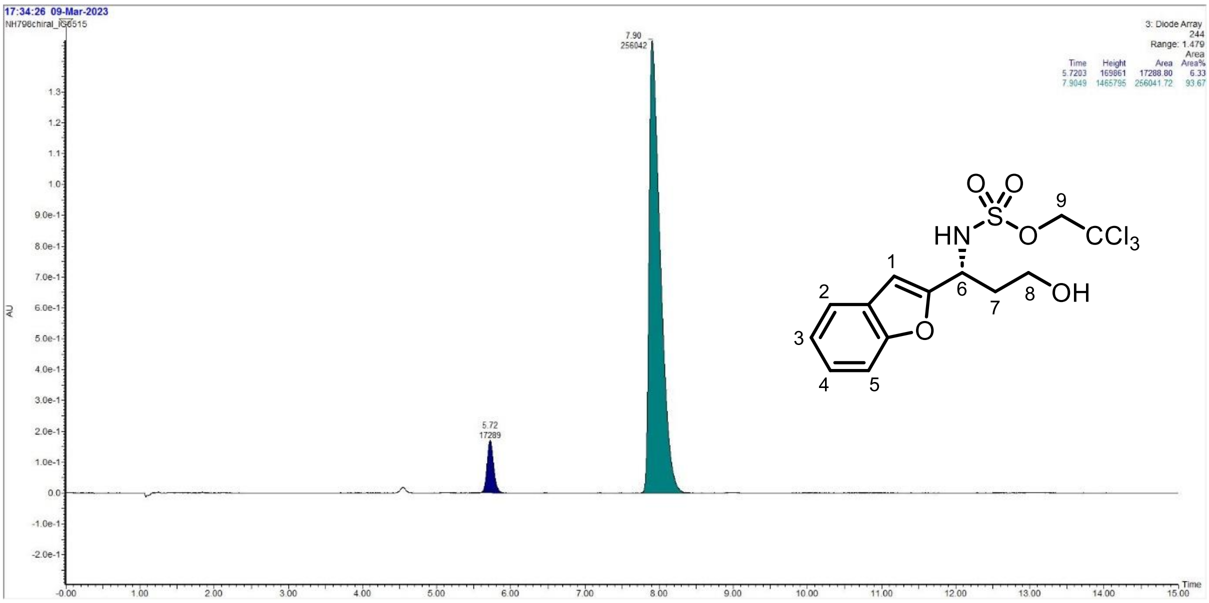

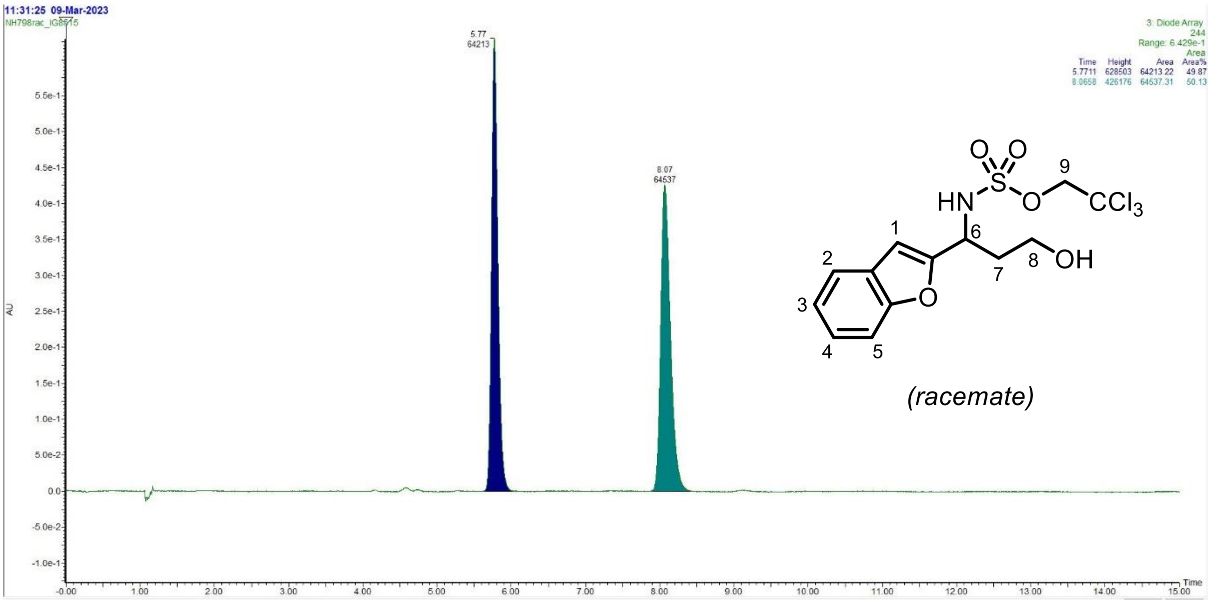

methyl (R)-4-(3-hydroxy-1-(((2,2,2-trichloroethoxy)sulfonyl)amino)propyl)furan-2-carboxylate (**2y**)

Chiral SFC Analysis CHIRALPAK IG (CO_2:MeOH, 75.0:25.0, 2.50 mL min^{-1}, 40 °C, 252 nm) indicated 92% *ee*, t_R = 2.8 (minor), 5.1 (major) minutes.

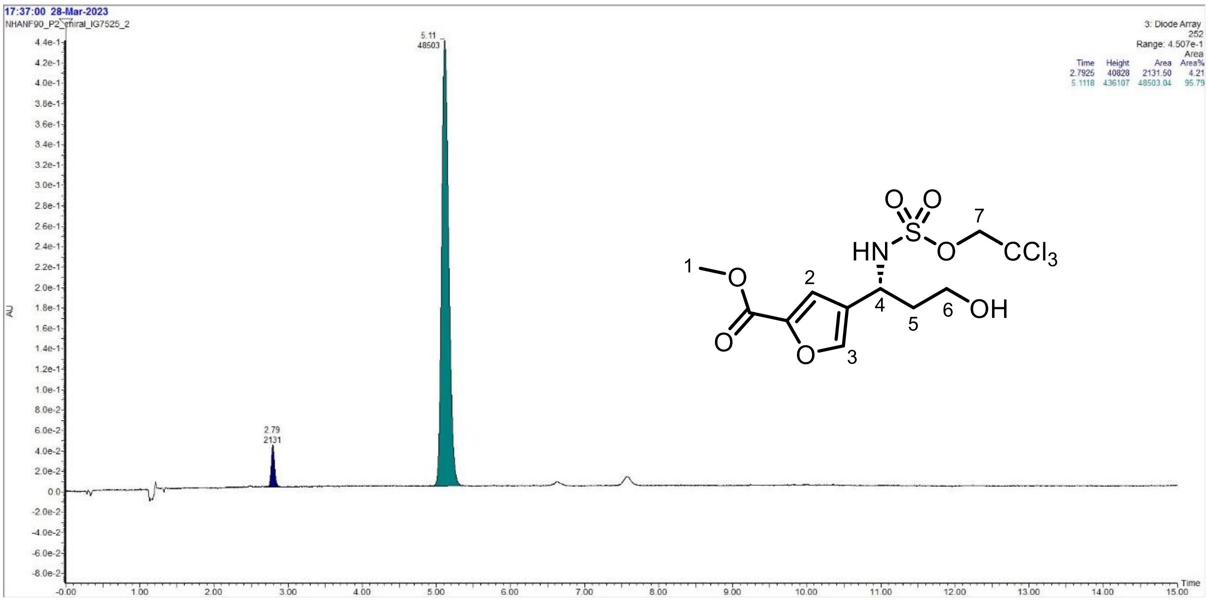

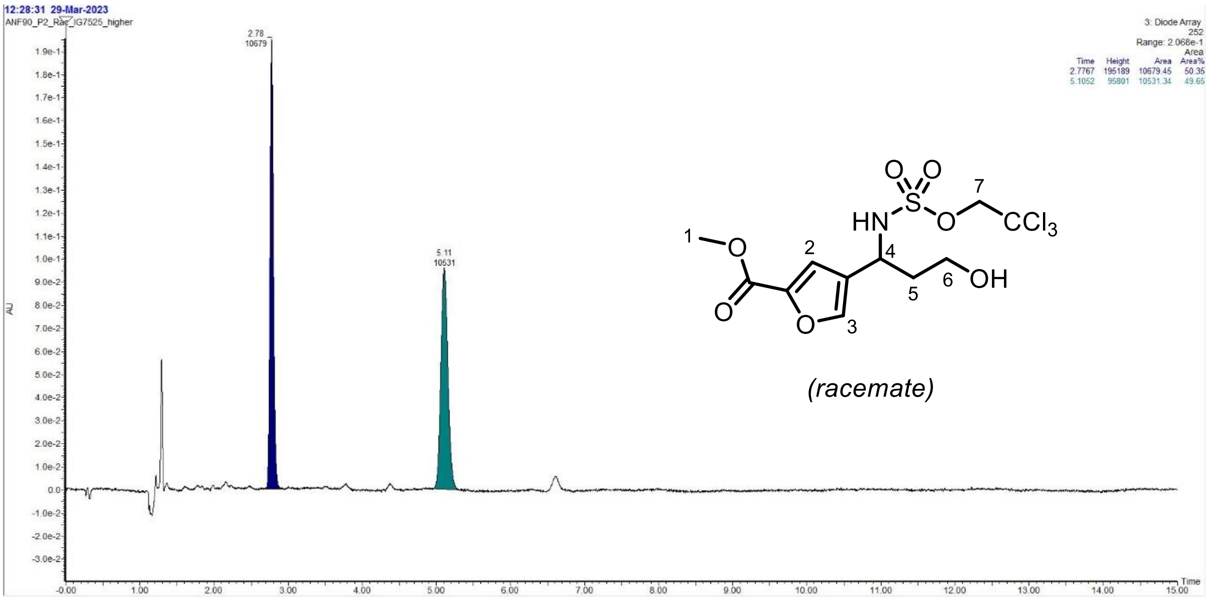

2,2,2-trichloroethyl ((1R,3R)-3-hydroxy-1,5-diphenylpentyl)sulfamate (**2z**)

Chiral SFC Analysis CHIRALPAK IG (CO_2:MeOH, 80.0:20.0, 2.50 mL min^{-1}, 40 °C, 210 nm) indicated 90% *ee*, t_R = 5.2 (major), 7.5 (minor) minutes.

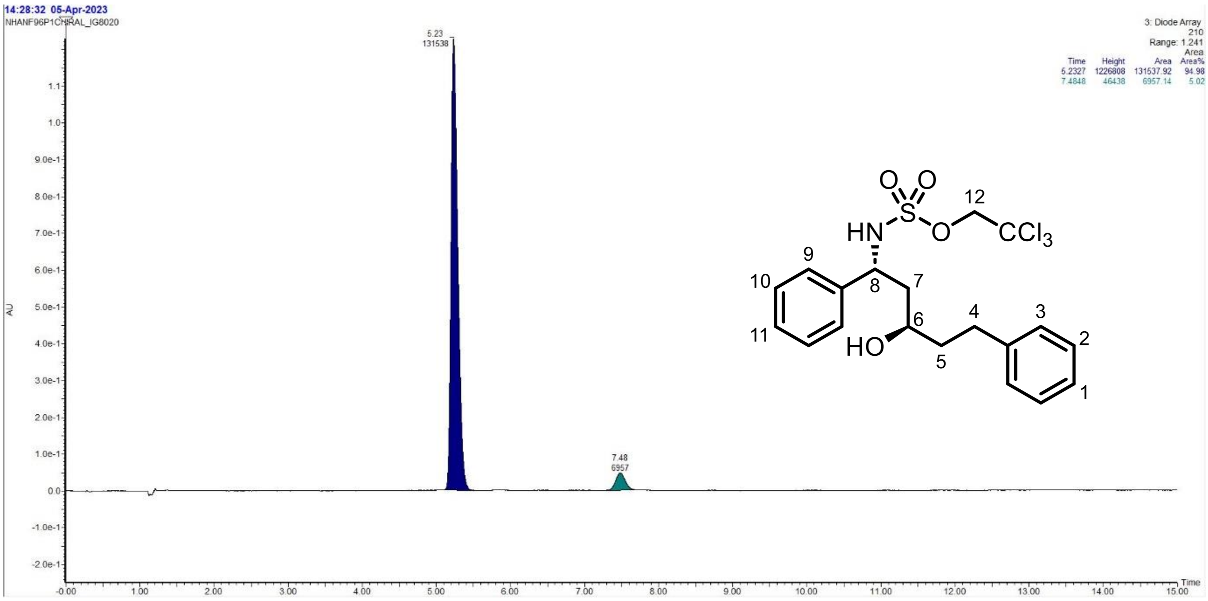

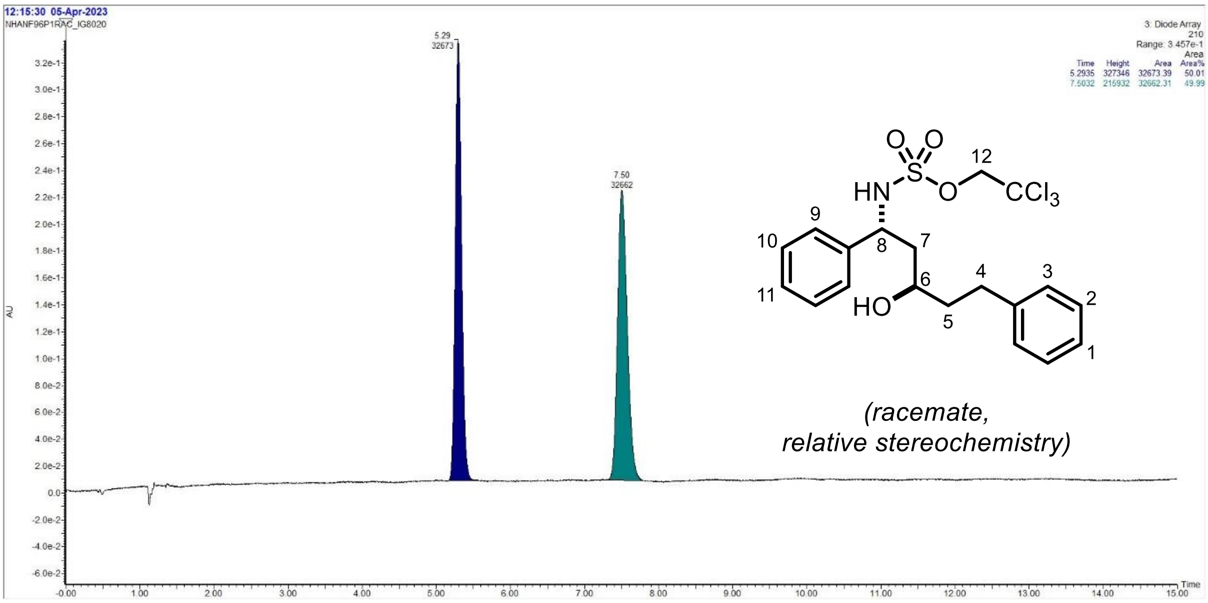

2,2,2-trichloroethyl (S)-1-phenyl-2-azaspiro[3.4]octane-2-sulfonate (**5c**)

Chiral SFC Analysis CHIRALPAK IK (CO_2:MeOH, 96.0:4.0, 2.50 mL min^{-1}, 40 °C, 210 nm) indicated 97% *ee*, t_R = 4.9 (minor), 5.3 (major) minutes.

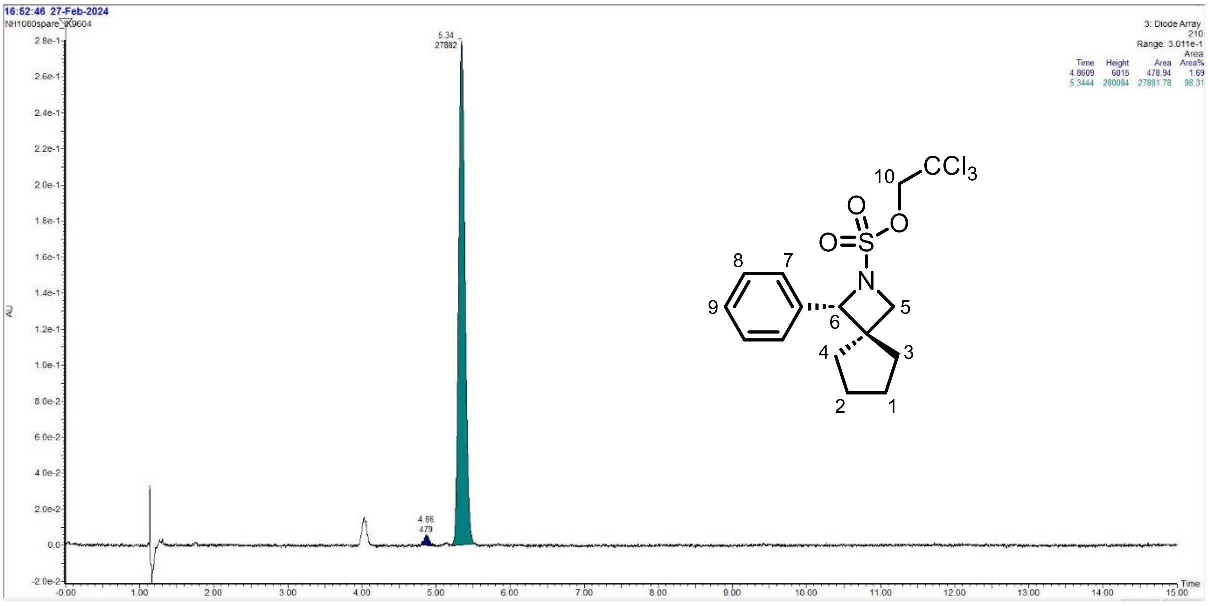

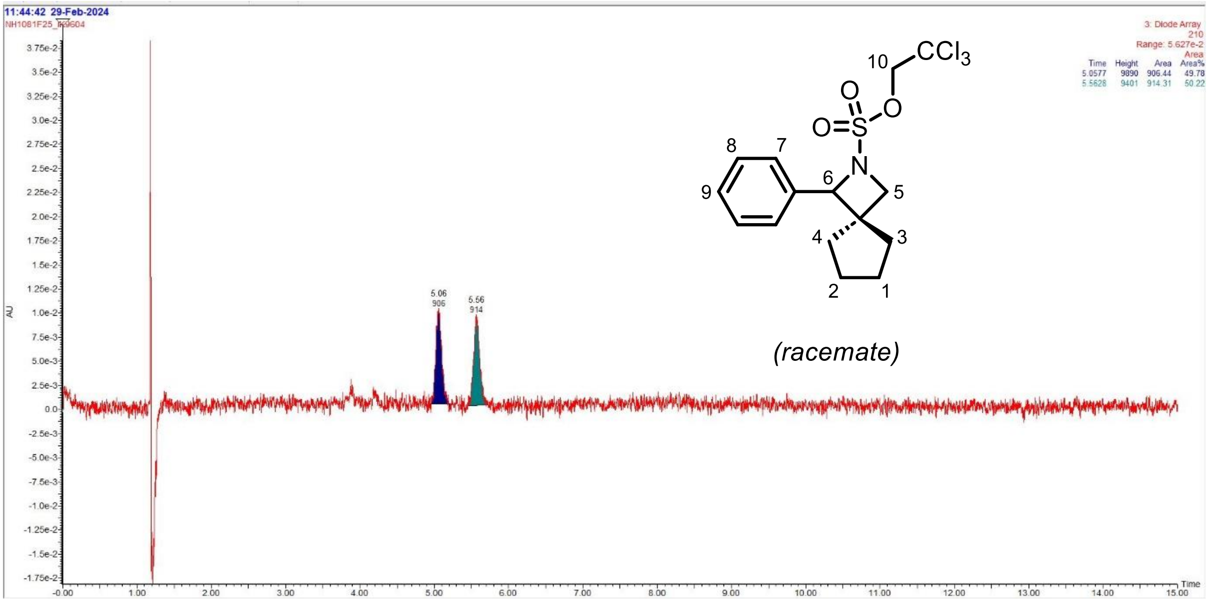

2,2,2-trichloroethyl (2S,4R)-2-phenethyl-4-phenylazetidine-1-sulfonate (**5d**)

Chiral SFC Analysis CHIRALPAK IG (CO_2:MeOH, 90.0:10.0, 2.50 mL min^{-1}, 40 °C, 210 nm) indicated 88% *ee*, t_R = 7.7 (major), 12.7 (minor) minutes.

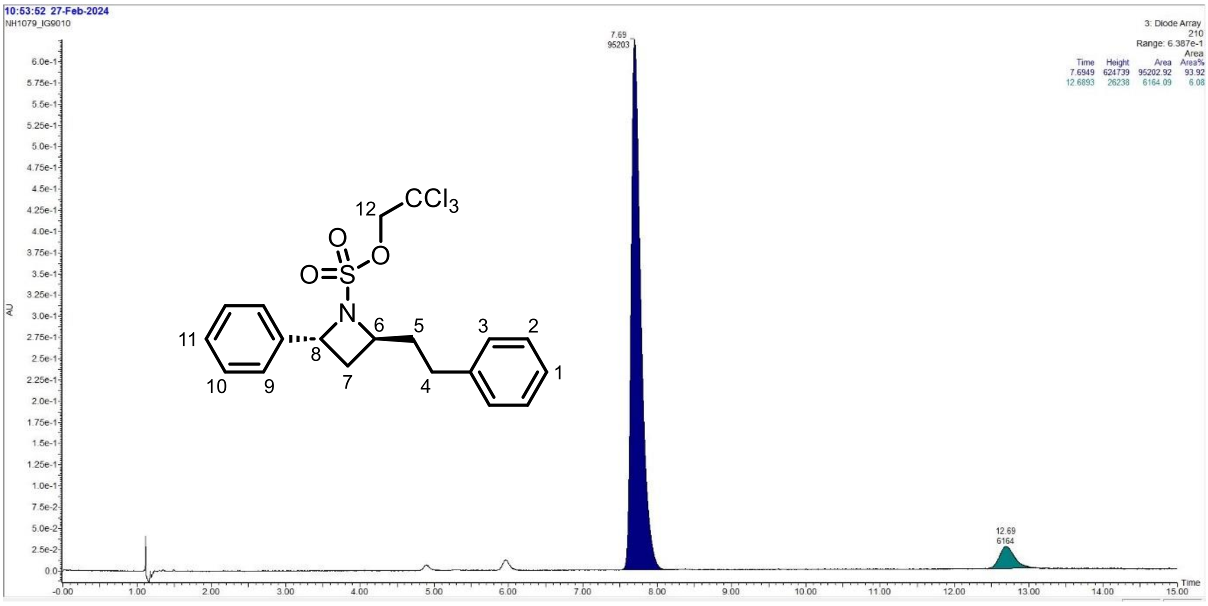

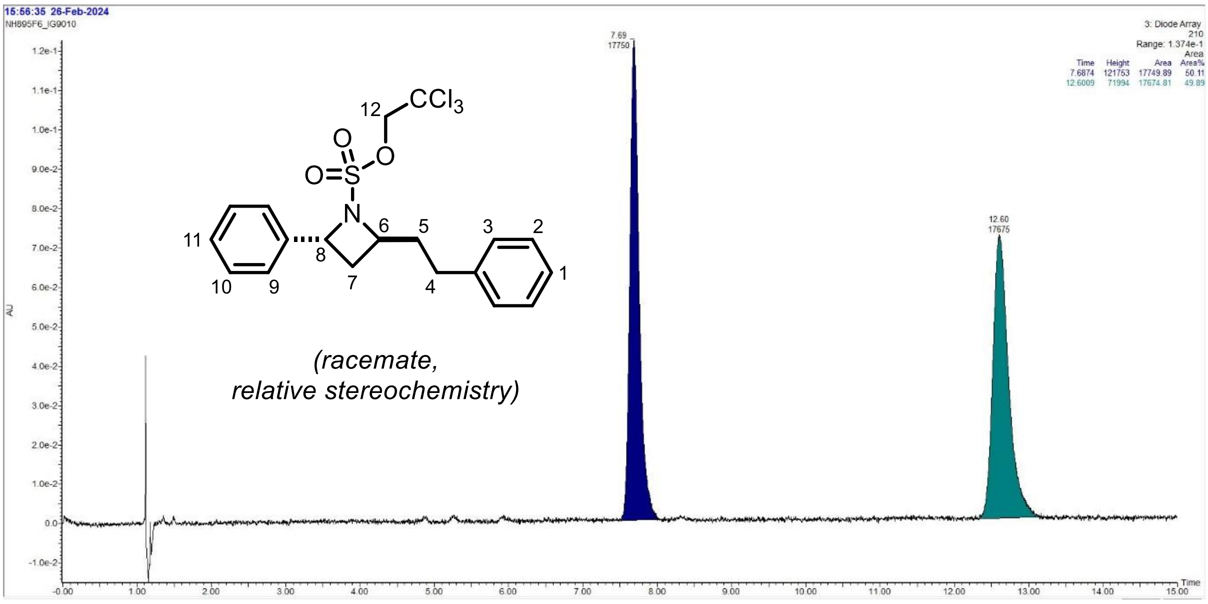

2,2,2-trichloroethyl (R)-2-phenyl-1-azaspiro[3.3]heptane-1-sulfonate (**5e**)

Chiral SFC Analysis CHIRALPAK IG (CO_2:MeOH, 95.0:5.0, 2.50 mL min^{-1}, 40 °C, 210 nm) indicated 82% *ee*, t_R = 5.5 (major), 8.6 (minor) minutes.

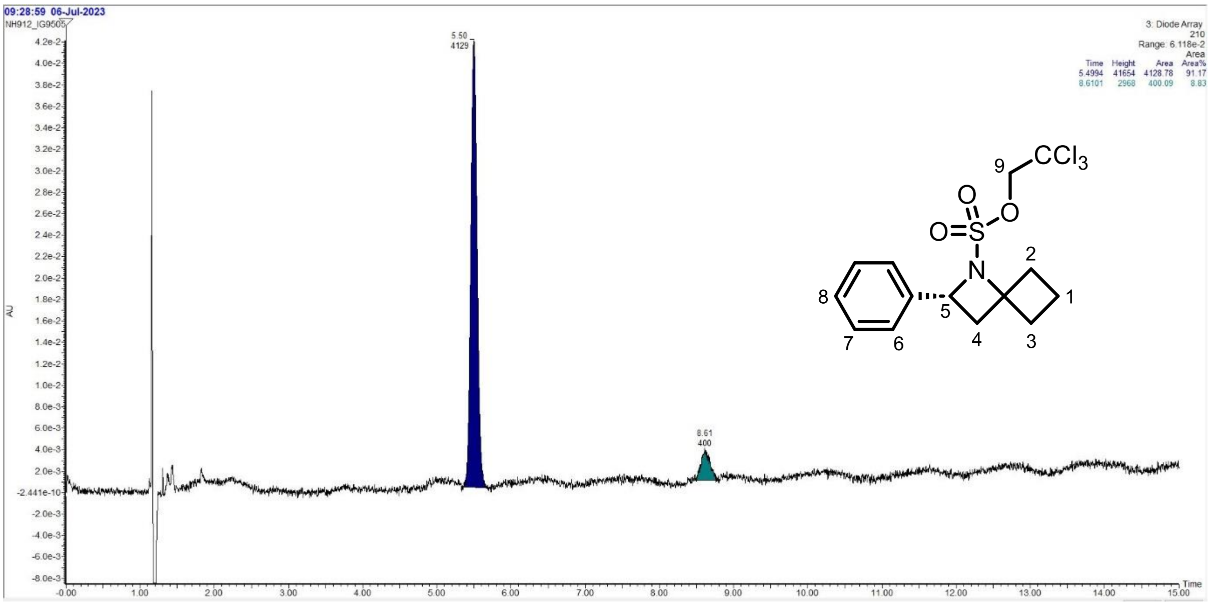

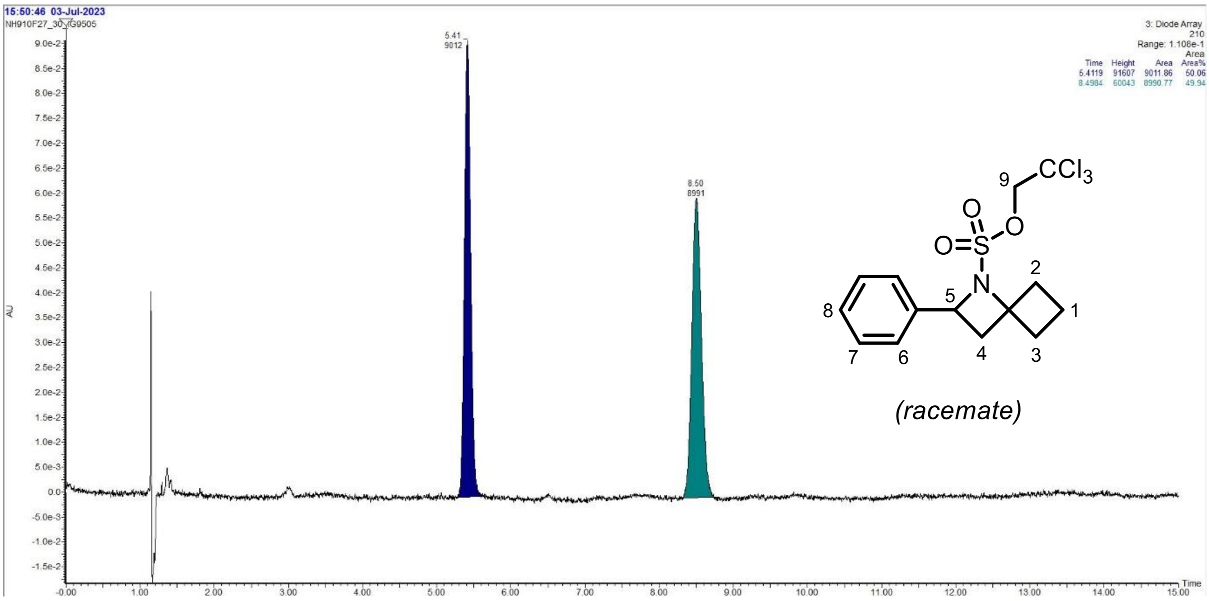

2,2,2-trichloroethyl (R)-(4-hydroxy-1-phenylbutyl)sulfamate (**7a**)

Chiral HPLC Analysis CHIRAL ART SC (hexane:*i*PrOH, 90.0:10.0, 1.25 mL min^{-1}, 40 °C, 210 nm) indicated 92% *ee*, t_R = 10.8 (major), 11.9 (minor) minutes.

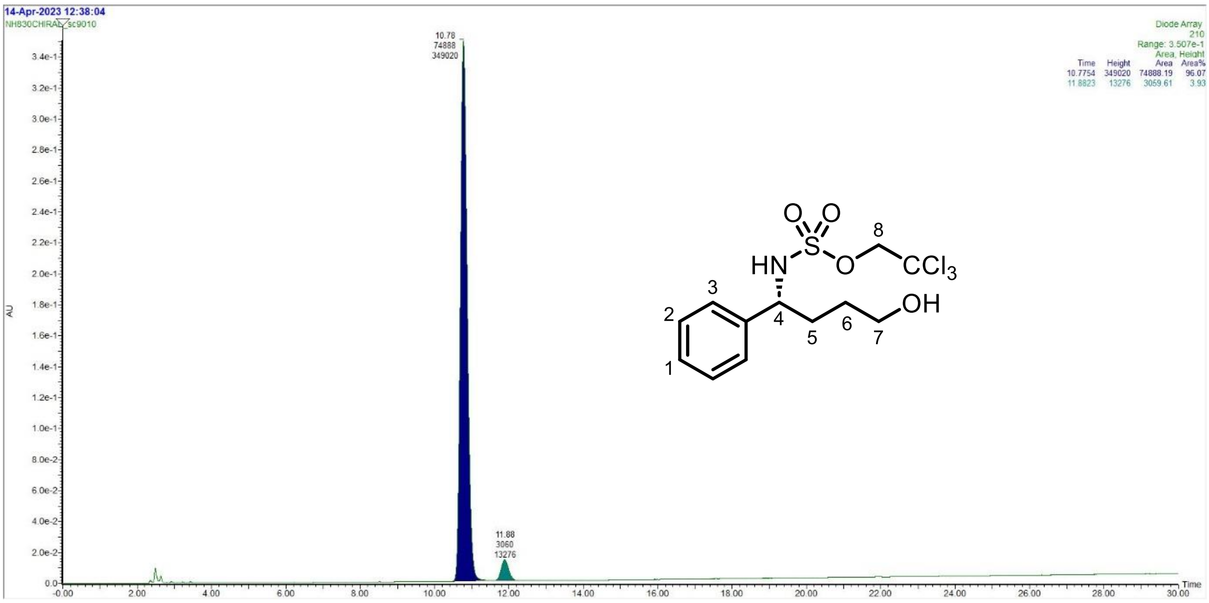

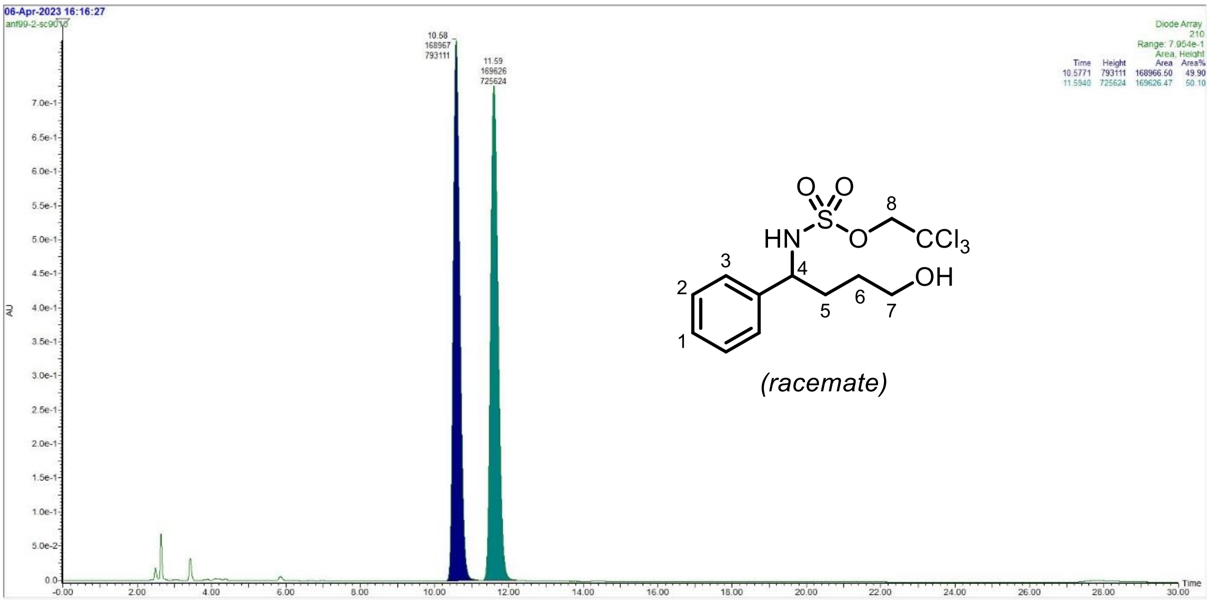

ethyl (R)-3-(4-hydroxy-1-(((2,2,2-trichloroethoxy)sulfonyl)amino)butyl)benzoate (**7b**)

Chiral SFC Analysis CHIRALPAK IG (CO_2:MeOH, 80.0:20.0, 1.25 mL min^{-1}, 40 °C, 229 nm) indicated 92% *ee*, t_R = 7.0 (minor), 7.4 (major) minutes.

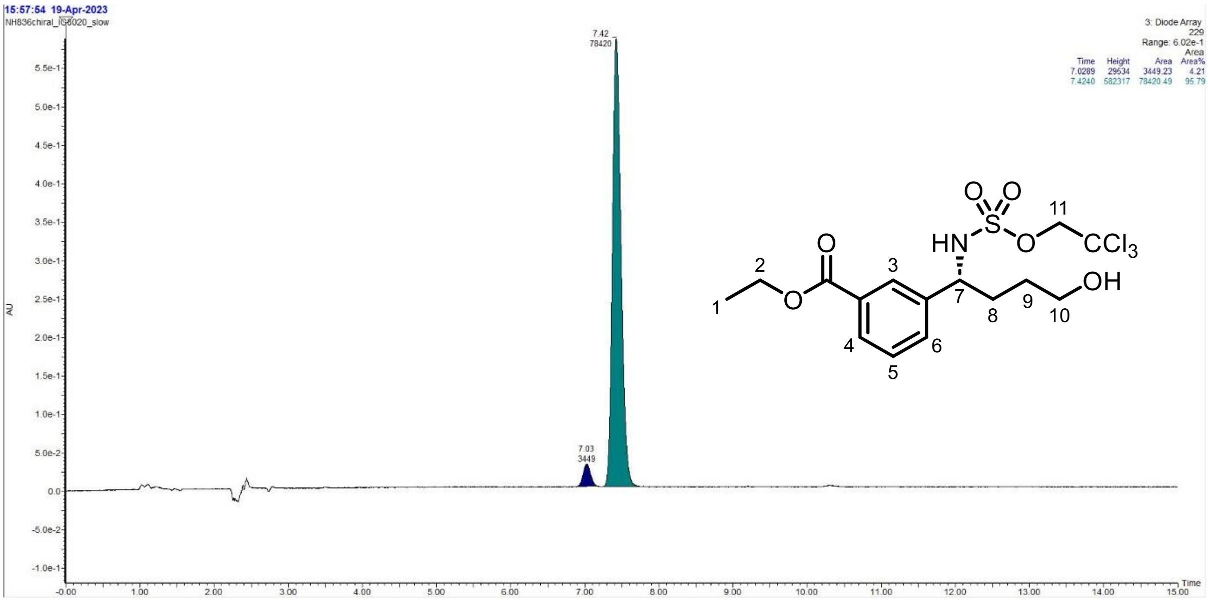

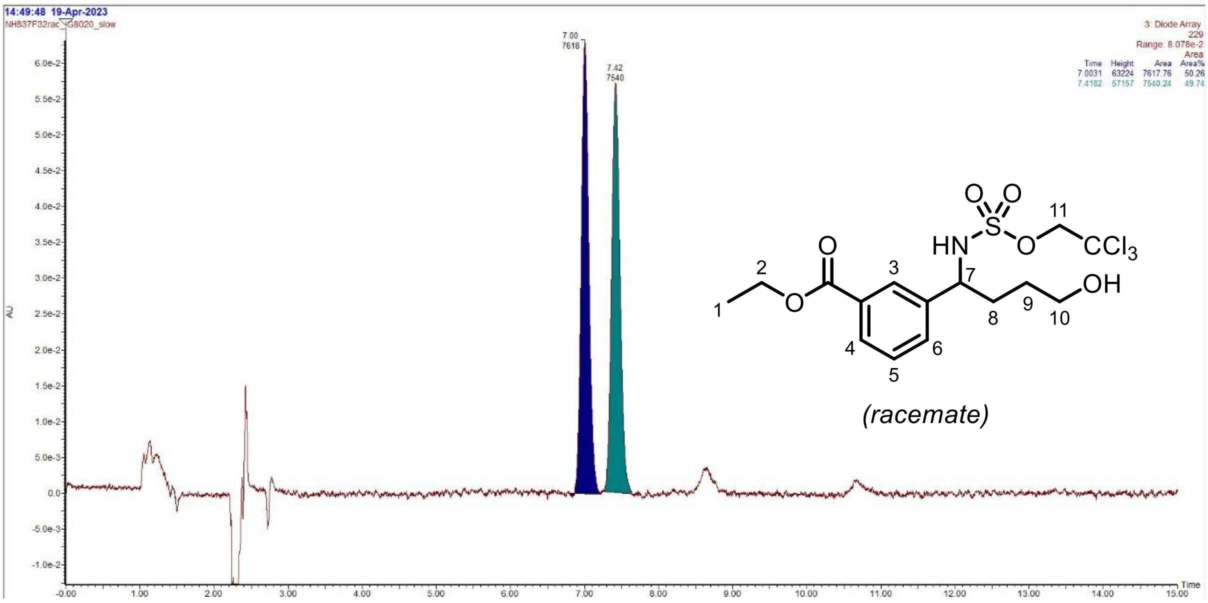

2,2,2-trichloroethyl (R)-(1-(3-chloro-2-methylphenyl)-4-hydroxybutyl)sulfamate (**7c**)

Chiral SFC Analysis CHIRALPAK IG (CO_2:MeOH, 80.0:20.0, 1.25 mL min^{-1}, 40 °C, 229 nm) indicated 93% *ee*, t_R = 5.4 (minor), 5.7 (major) minutes.

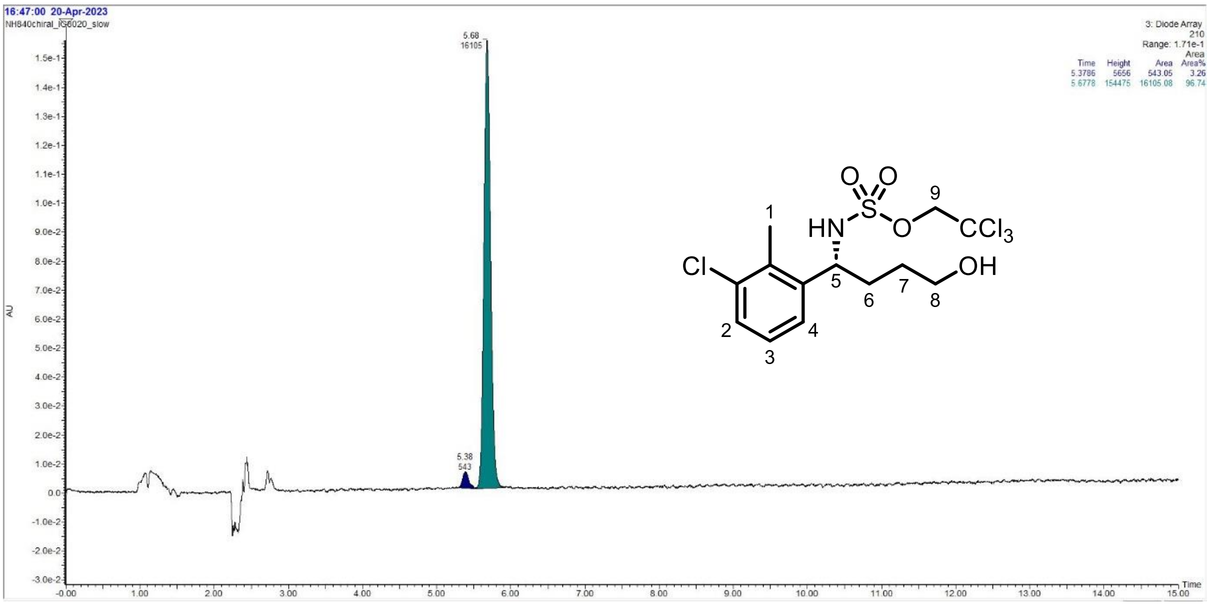

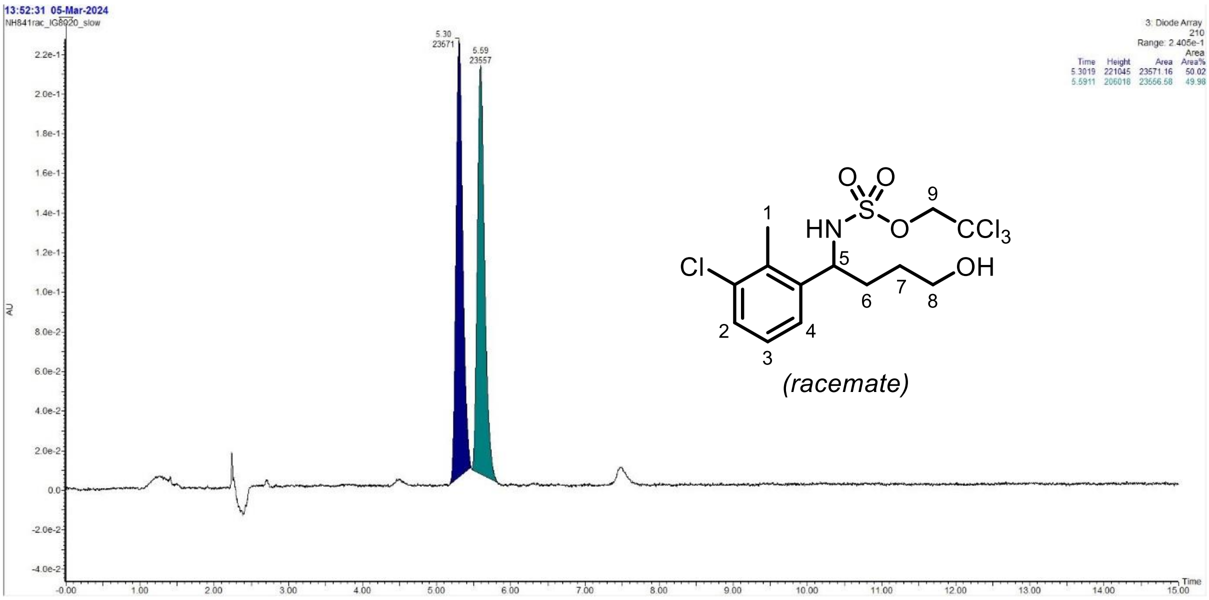

2,2,2-trichloroethyl (R)-(5-hydroxy-1-phenylpentyl)sulfamate (**7d**)

Chiral HPLC Analysis CHIRAL ART SC (hexane:*i*PrOH, 90.0:10.0, 1.25 mL min^{-1}, 40 °C, 210 nm) indicated 92% *ee*, t_R = 11.7 (major), 12.8 (minor) minutes.

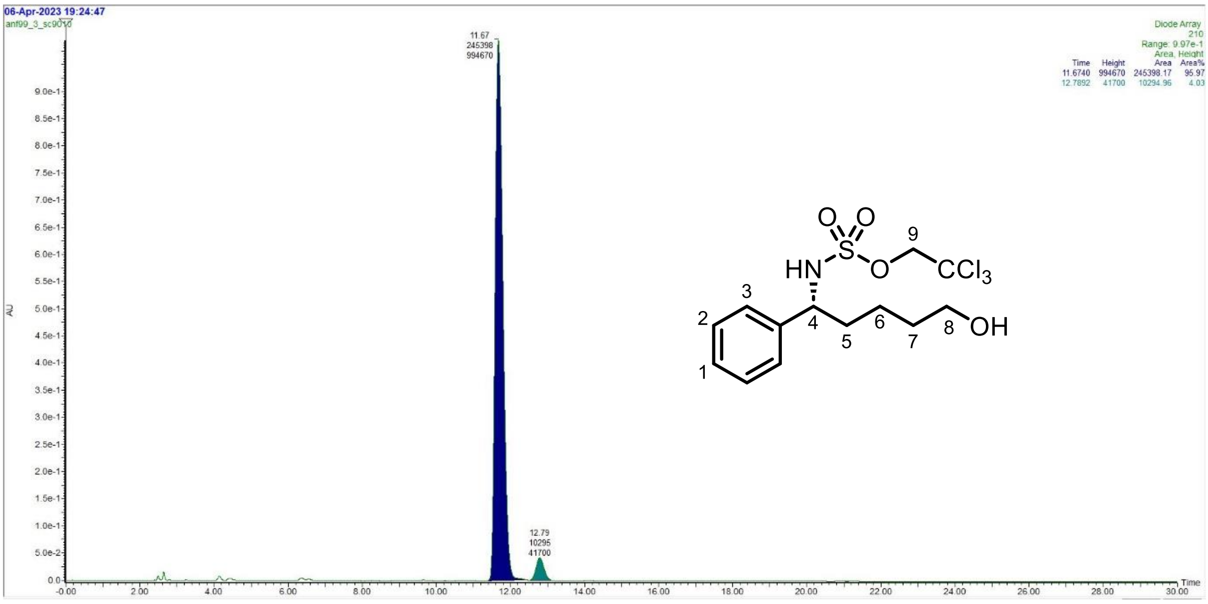

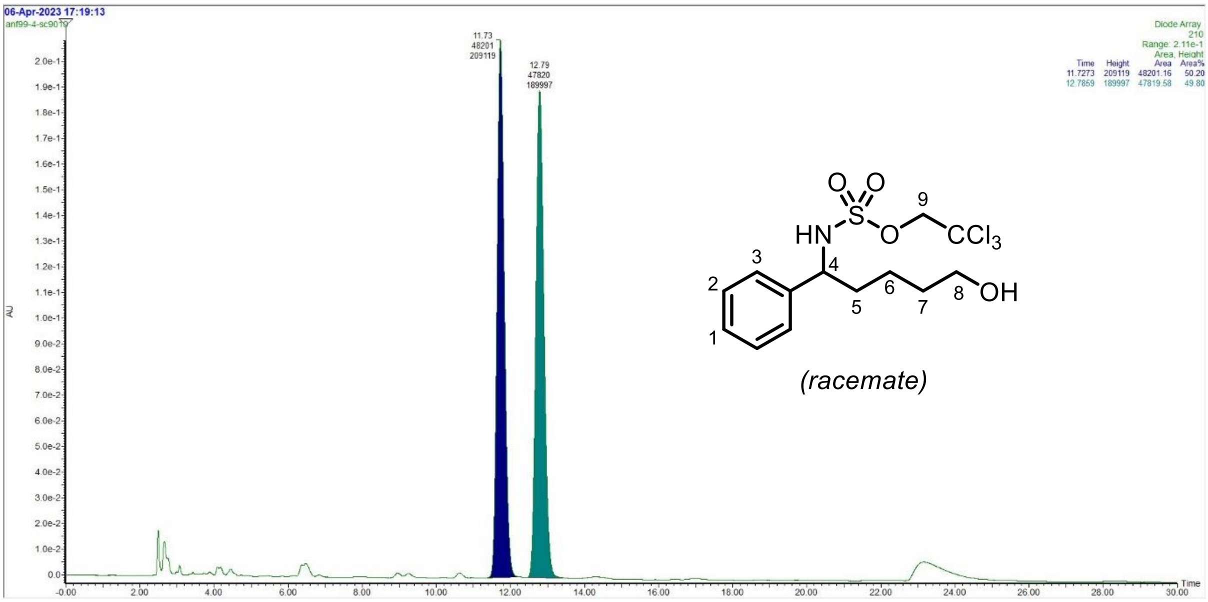

2,2,2-trichloroethyl (R)-(1-(4-(tert-butyl)phenyl)-5-hydroxypentyl)sulfamate (**7e**)

Chiral HPLC Analysis CHIRAL ART SC (hexane:*i*PrOH, 90.0:10.0, 1.25 mL min^{-1}, 40 °C, 210 nm) indicated 86% *ee*, t_R = 11.8 (major), 13.7 (minor) minutes.

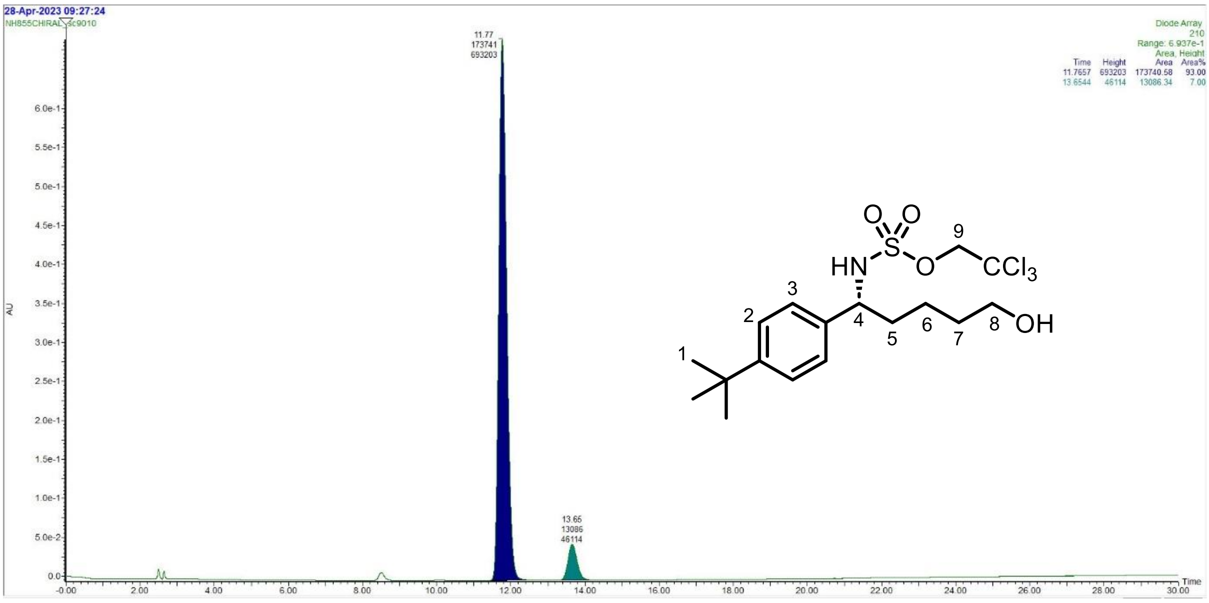

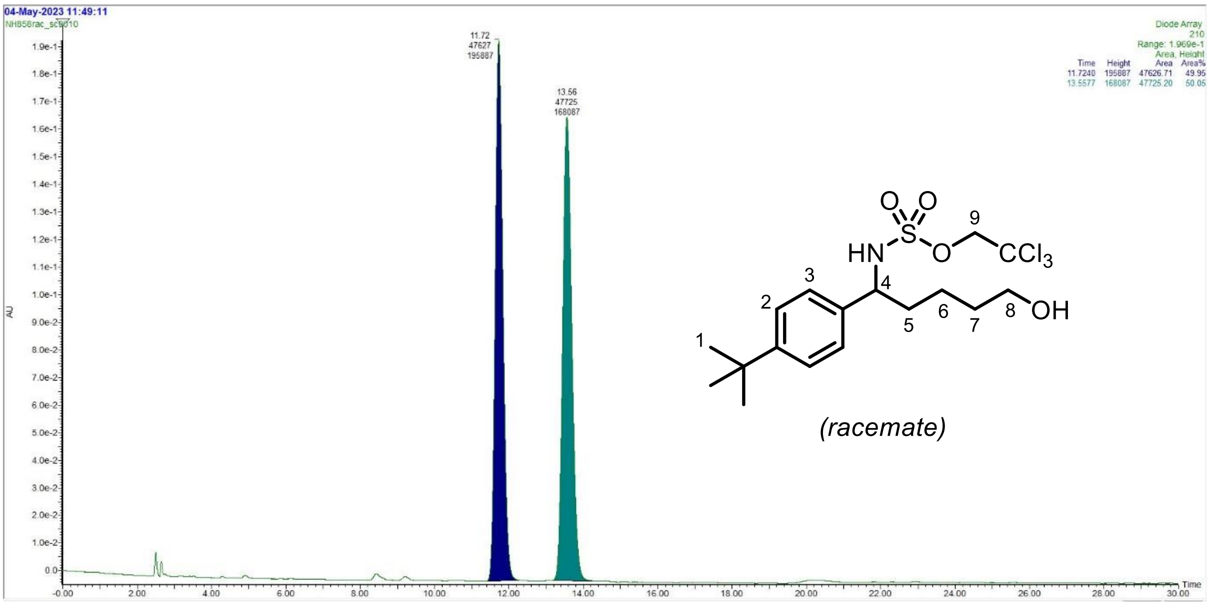

2,2,2-trichloroethyl (R)-(1-(3-fluoro-2-methylphenyl)-5-hydroxypentyl)sulfamate (**7f**)

Chiral HPLC Analysis CHIRAL ART SC (hexane:*i*PrOH, 90.0:10.0, 1.25 mL min^{-1}, 40 °C, 210 nm) indicated 94% *ee*, t_R = 8.0 (major), 9.1 (minor) minutes.

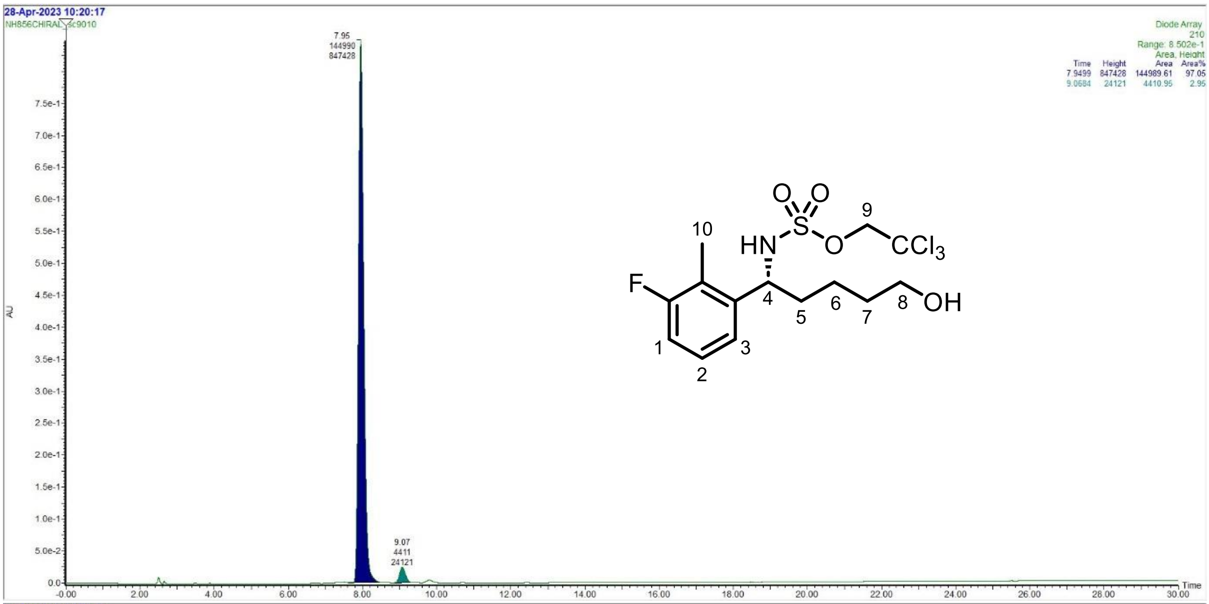

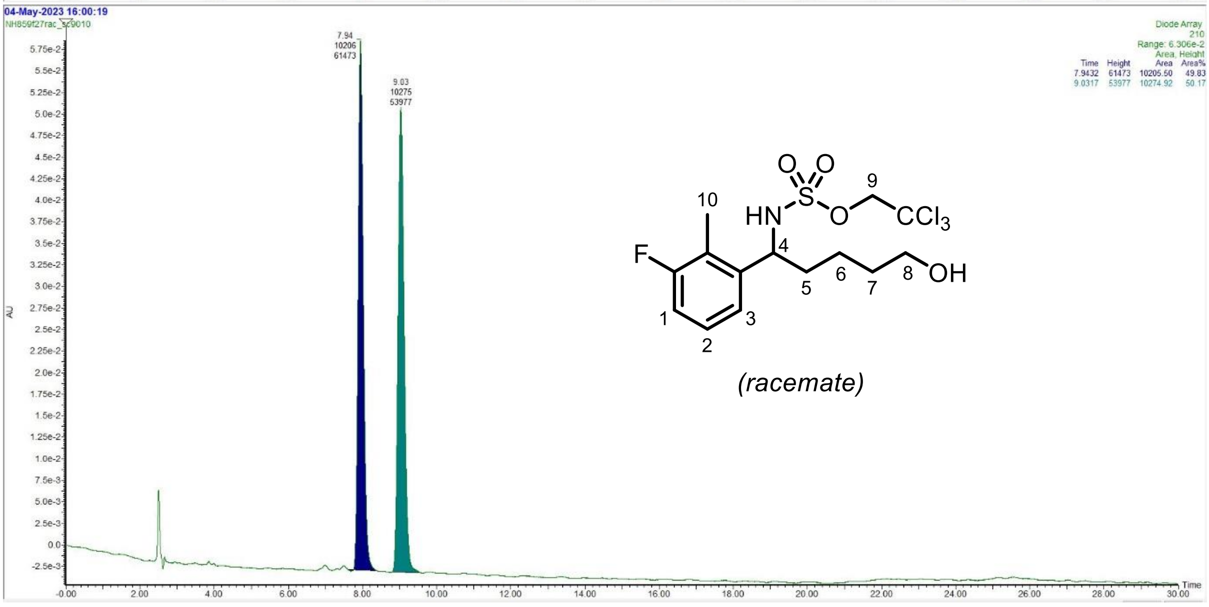

2,2,3,3,3-pentafluoropropyl (R)-(1-hydroxy-3-methyl-3-(phenylthio)butan-2-yl)sulfamate (A) and *2,2,3,3,3-pentafluoropropyl (R)-(4-hydroxy-2-methyl-3-(phenylthio)butan-2-yl)sulfamate* (B). **Obtained following derivatisation of 9a**.

Chiral SFC Analysis CHIRALPAK IG (CO_2:MeOH, 85.0:15.0, 2.50 mL min^{-1}, 40 °C, 217 nm) for major regioisomer A indicated 89% *ee*, t_R = 2.1 (major), 3.6 (minor) minutes.

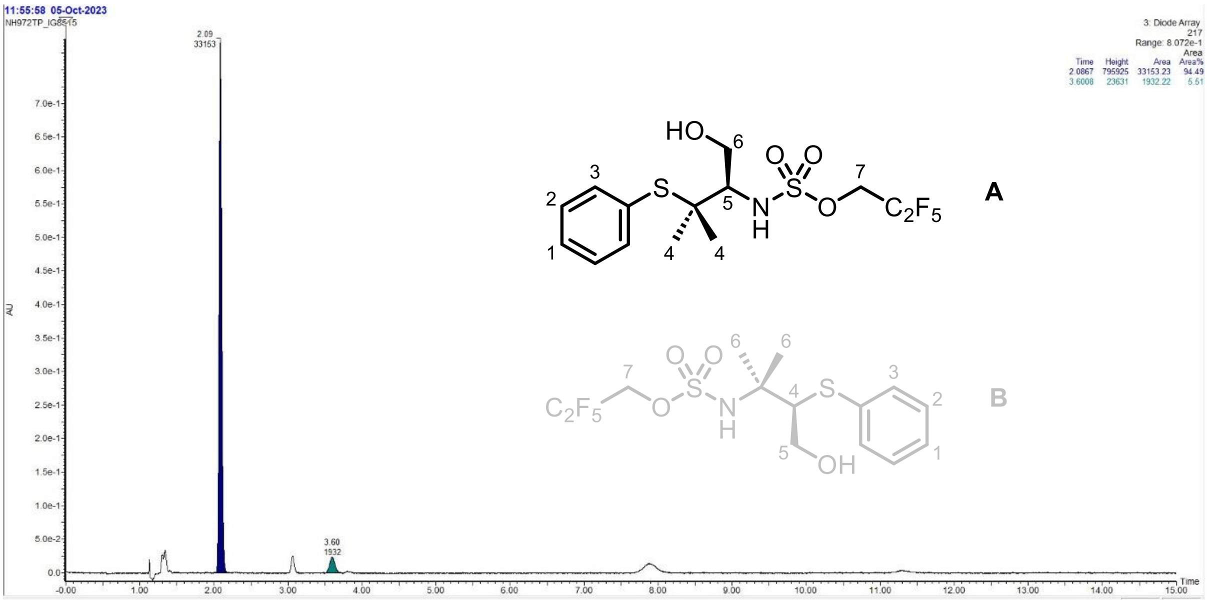

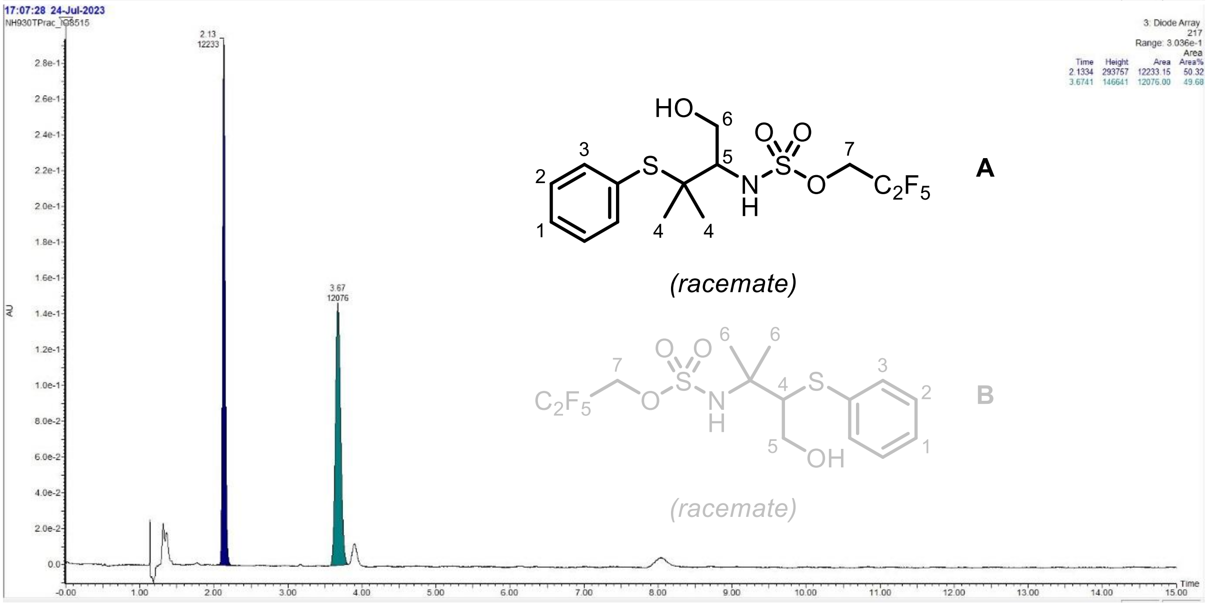

2,2,3,3,3-pentafluoropropyl (R)-(3-butyl-1-hydroxy-3-(phenylthio)heptan-2-yl)sulfamate. **Obtained following derivatisation of 9b**.

Chiral SFC Analysis CHIRALPAK IJ (CO_2:MeOH, 98.0:2.0, 2.50 mL min^{-1}, 40 °C, 218 nm) indicated 88% *ee*, t_R = 3.6 (major), 4.2 (minor) minutes. *Note: additional peaks visible in the traces below were attributed to trace amounts of minor regioisomer which was not assigned.*

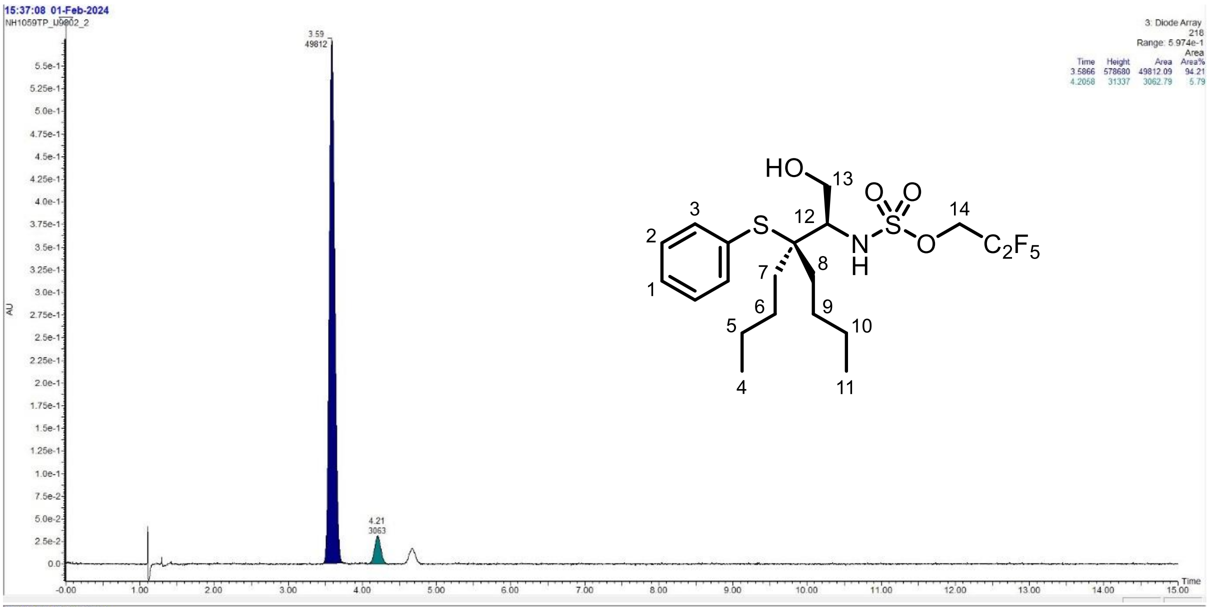

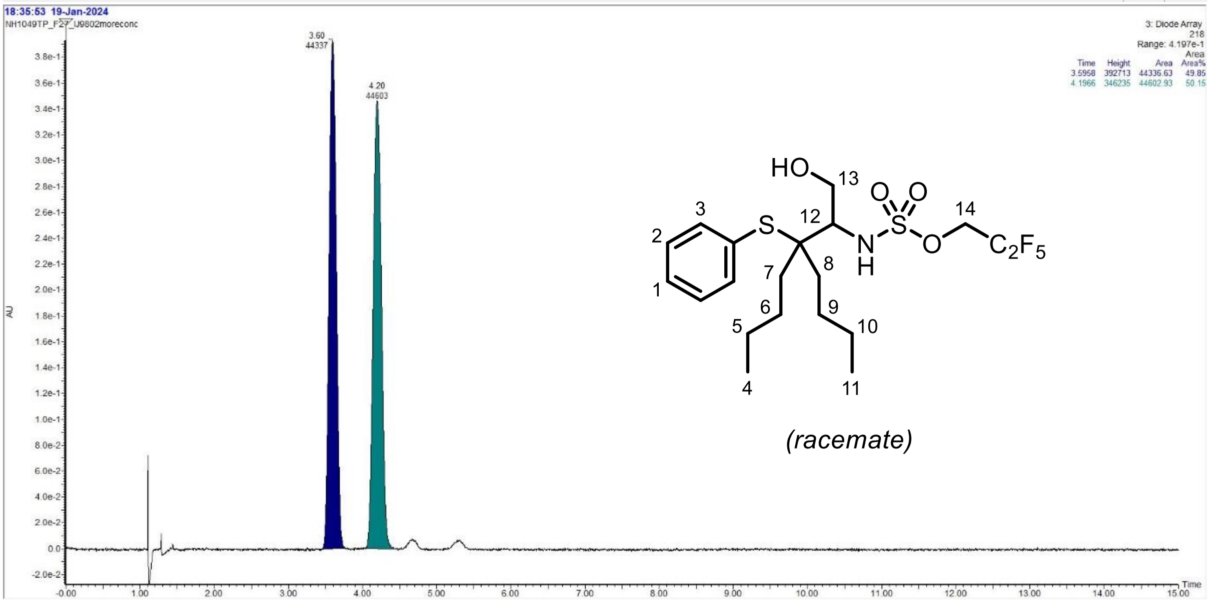

2,2,3,3,3-pentafluoropropyl (R)-(2-hydroxy-1-(1-(phenylthio)cyclohexyl)ethyl)sulfamate (A) and *2,2,3,3,3-pentafluoropropyl (R)-(1-(2-hydroxy-1-(phenylthio)ethyl)cyclohexyl)sulfamate* (B). **Obtained following derivatisation of 9c**.

Chiral SFC Analysis CHIRALPAK IG (CO_2:MeOH, 92.0:8.0, 2.50 mL min^{-1}, 40 °C, 218 nm) for major regioisomer A indicated 92% *ee*, t_R = 3.7 (major), 4.5 (minor) minutes.

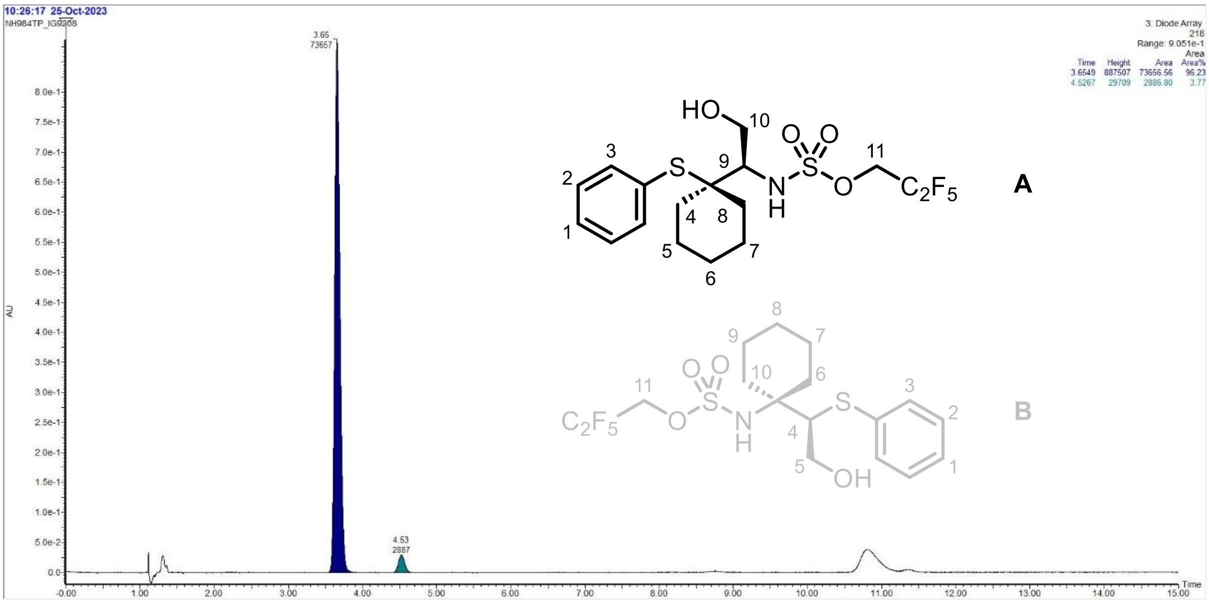

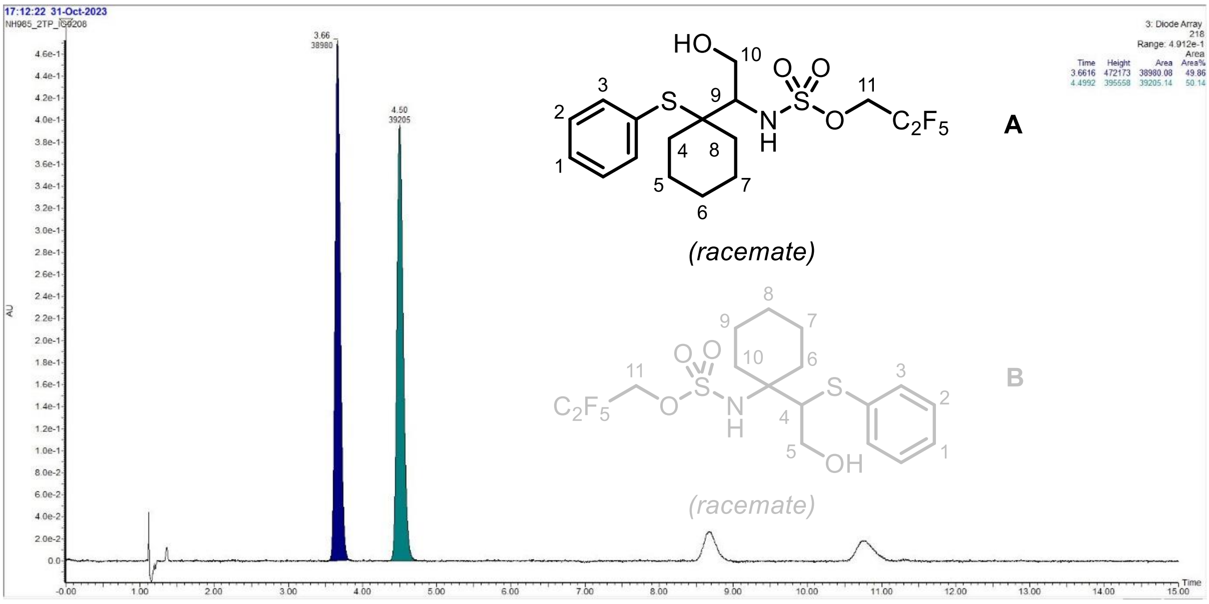

2,2,3,3,3-pentafluoropropyl (R)-(2-hydroxy-1-(1-(phenylthio)cyclododecyl)ethyl)sulfamate (**A**) and *2,2,3,3,3-pentafluoropropyl (R)-(1-(2-hydroxy-1-(phenylthio)ethyl)cyclododecyl)sulfamate* (**B**). **Obtained following derivatisation of 9d**.

Chiral SFC Analysis CHIRALPAK IJ (CO_2:MeOH, 95.0:5.0, 2.50 mL min^{-1}, 40 °C, 218 nm) for major regioisomer A indicated 90% *ee*, t_R = 4.6 (major), 5.2 (minor) minutes.

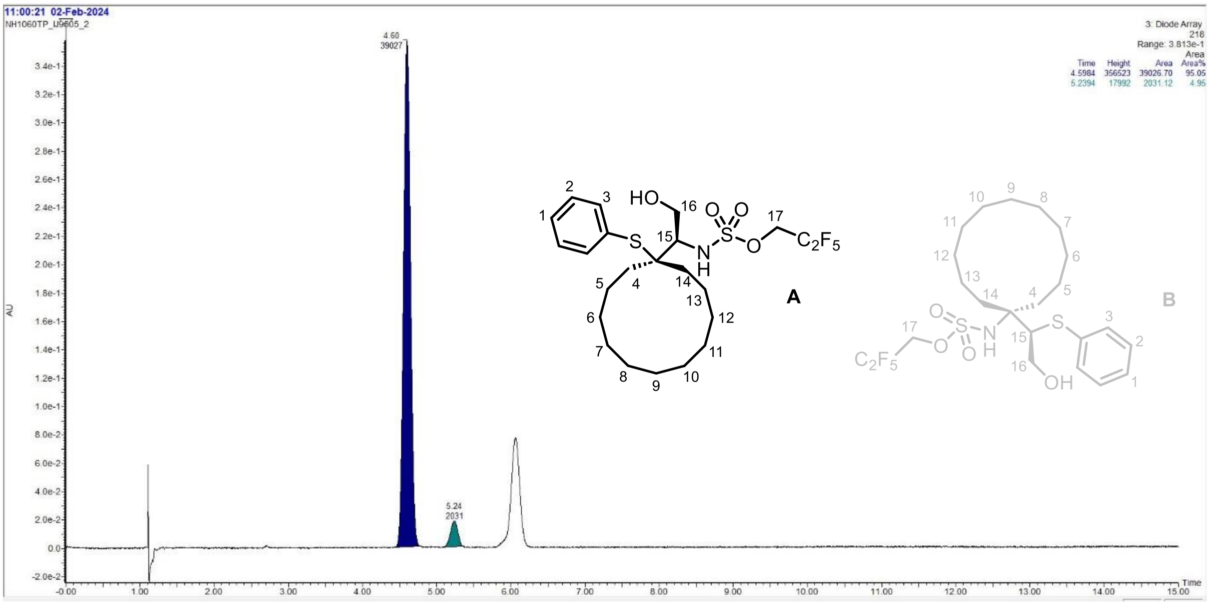

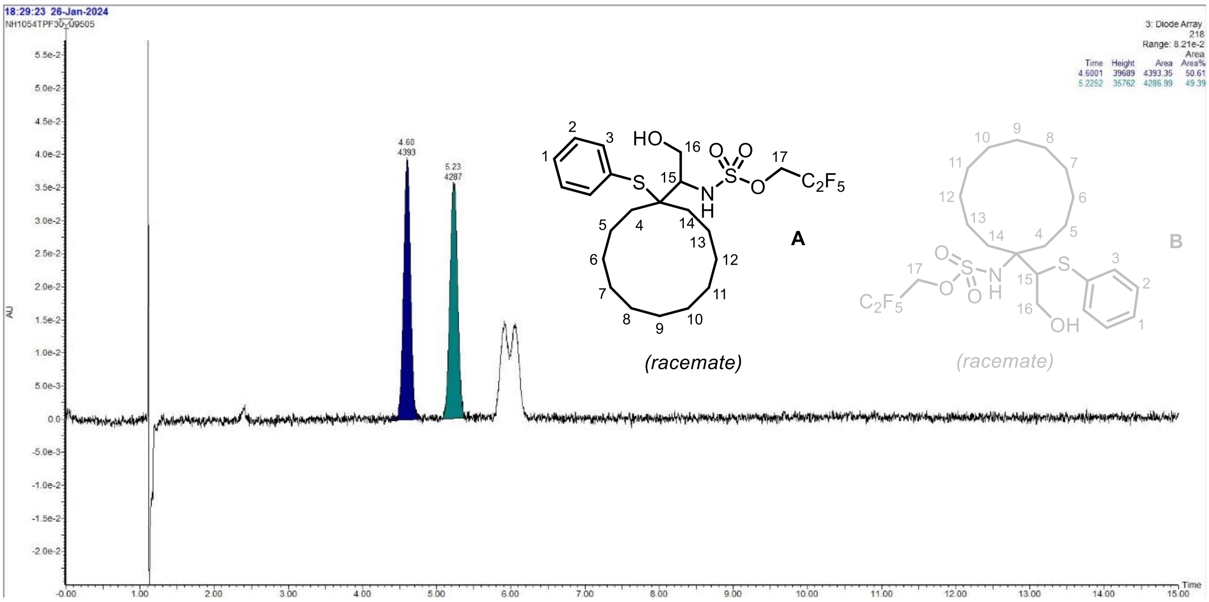

2,2,3,3,3-pentafluoropropyl (R)-(4,4-difluoro-1-(2-hydroxy-1-(phenylthio)ethyl)cyclohexyl)sulfamate (**A**) and *2,2,3,3,3-pentafluoropropyl (R)-(1-(4,4-difluoro-1-(phenylthio)cyclohexyl)-2-hydroxyethyl)sulfamate* (**B**). **Obtained following derivatisation of 9e**.

Chiral SFC Analysis CHIRALPAK IG (CO_2:MeOH, 95.0:5.0, 2.50 mL min^{-1}, 40 °C, 251 nm) for major regioisomer A indicated 87% *ee*, t_R = 6.1 (minor), 10.1 (major) minutes.

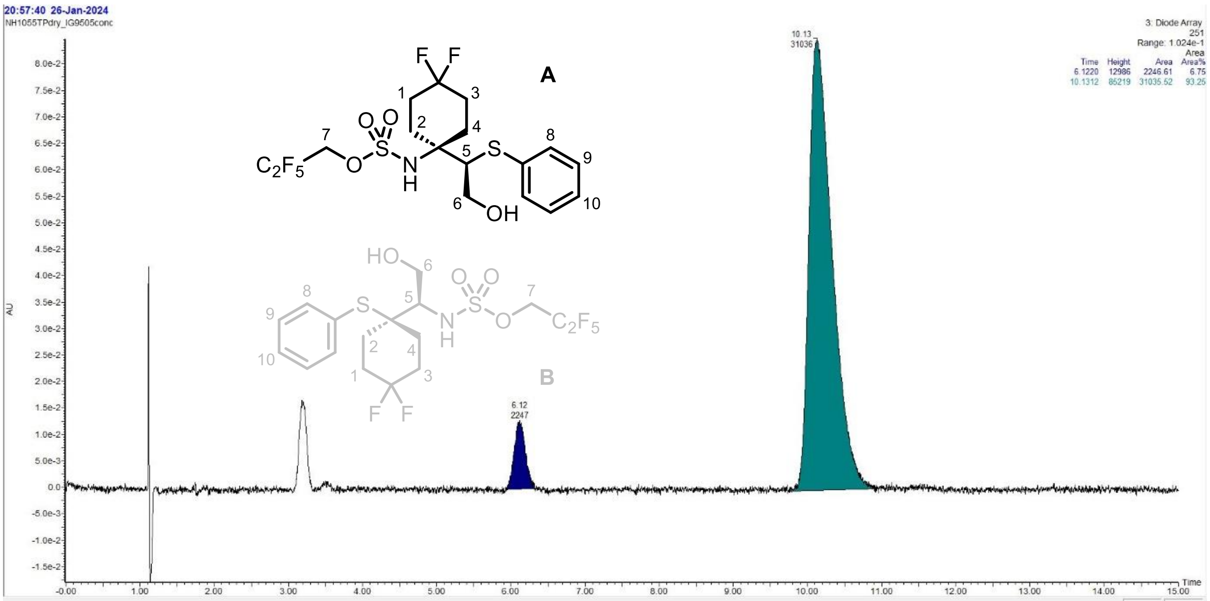

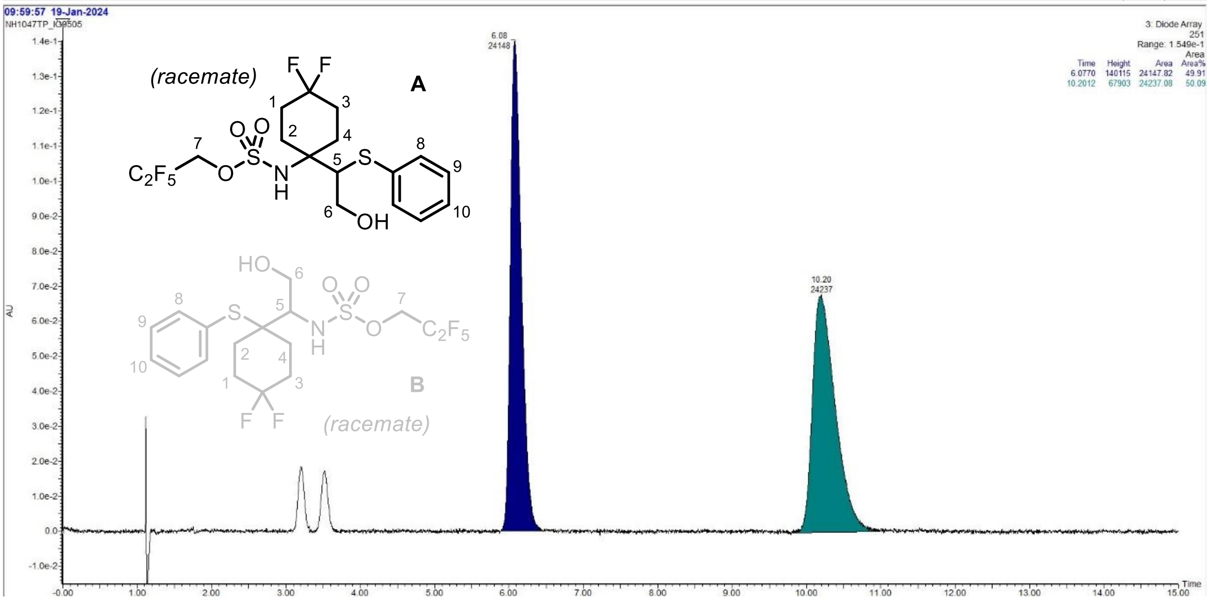

2,2,3,3,3-pentafluoropropyl (R)-(2-hydroxy-1-(4-(phenylthio)tetrahydro-2H-pyran-4-yl)ethyl)sulfamate (**A**) and *2,2,3,3,3-pentafluoropropyl (R)-(4-(2-hydroxy-1-(phenylthio)ethyl)tetrahydro-2H-pyran-4-yl)sulfamate* (**B**). **Obtained following derivatisation of 9f**.

Chiral SFC Analysis CHIRALPAK IG (CO_2:MeOH, 94.0:6.0, 2.50 mL min^{-1}, 40 °C, 217 nm) for major regioisomer A indicated 90% *ee*, t_R = 5.7 (major), 6.2 (minor) minutes.

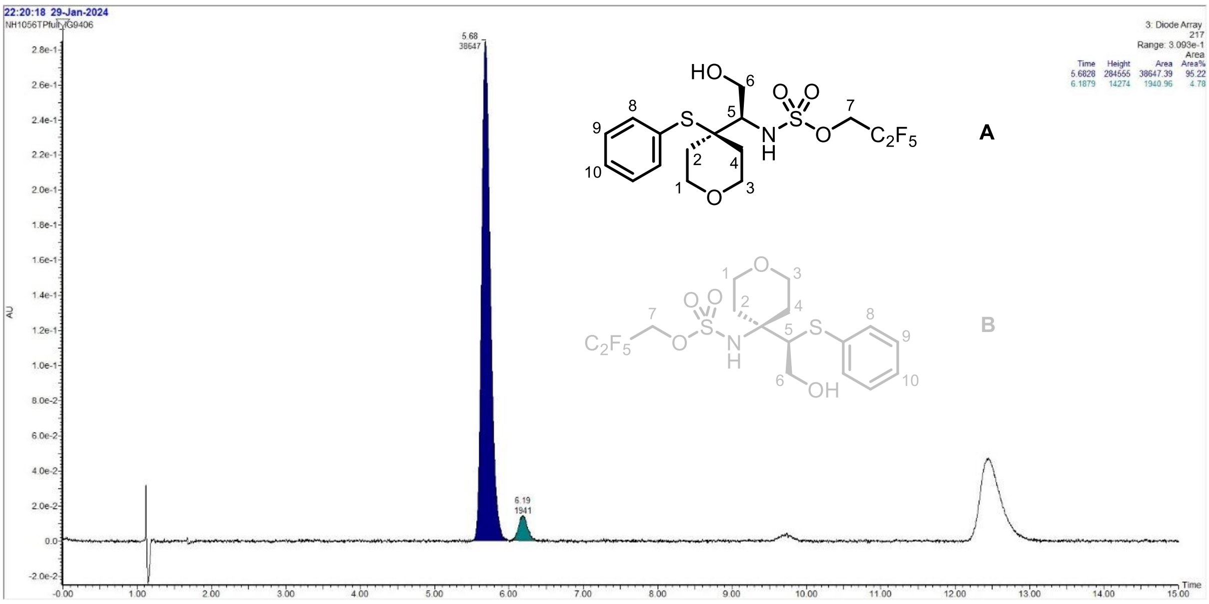

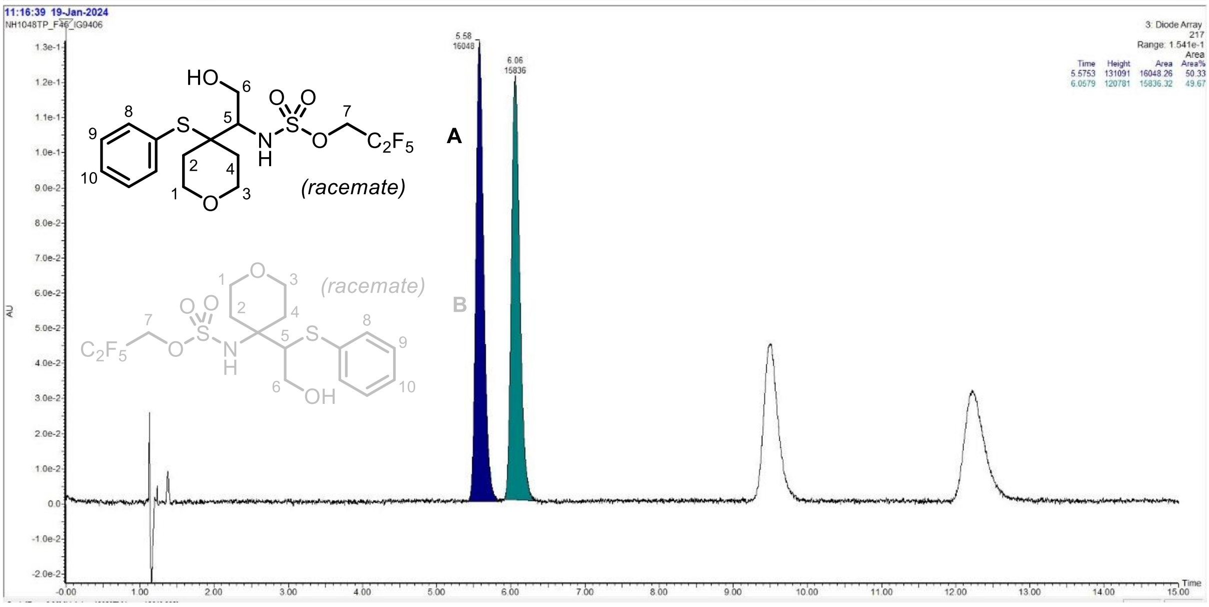

2,2,3,3,3-pentafluoropropyl (R)-(2-hydroxy-1-(8-(phenylthio)-1,4-dioxaspiro[4.5]decan-8-yl)ethyl)sulfamate (**A**) and *2,2,3,3,3-pentafluoropropyl (R)-(8-(2-hydroxy-1-(phenylthio)ethyl)-1,4-dioxaspiro[4.5]decan-8-yl)sulfamate* (**B**). **Obtained following derivatisation of 9g**.

Chiral SFC Analysis CHIRALPAK IG (CO_2:MeOH, 90.0:10.0, 1.25 mL min^{-1}, 40 °C, 217 nm) for one regioisomer indicated 94% *ee*, t_R = 9.1 (minor), 12.9 (major) minutes.

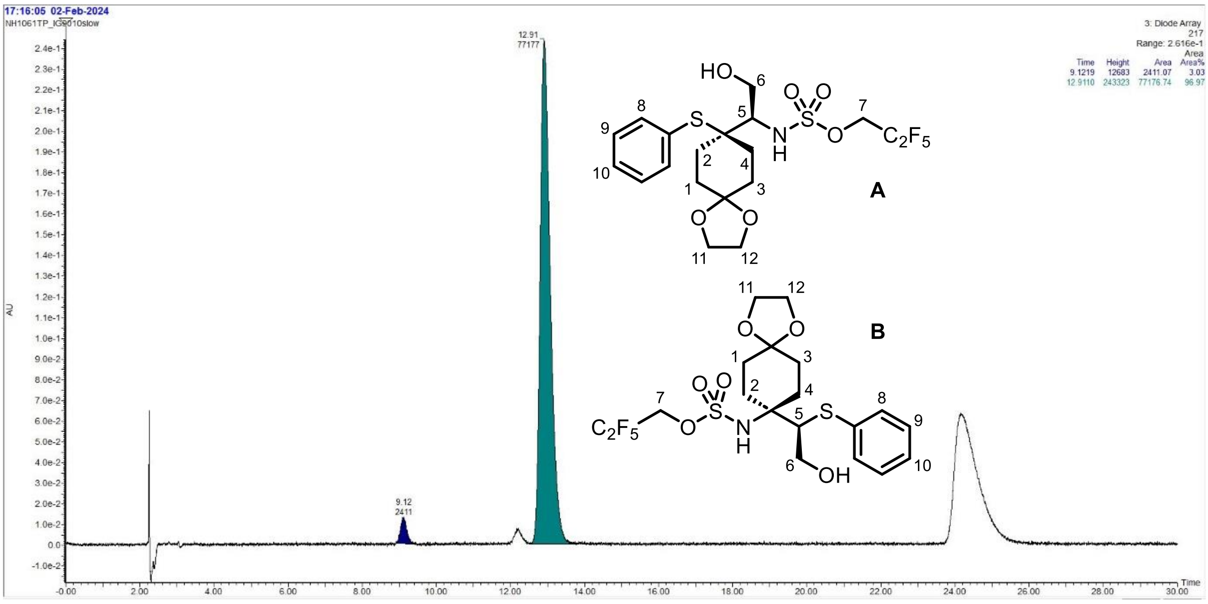

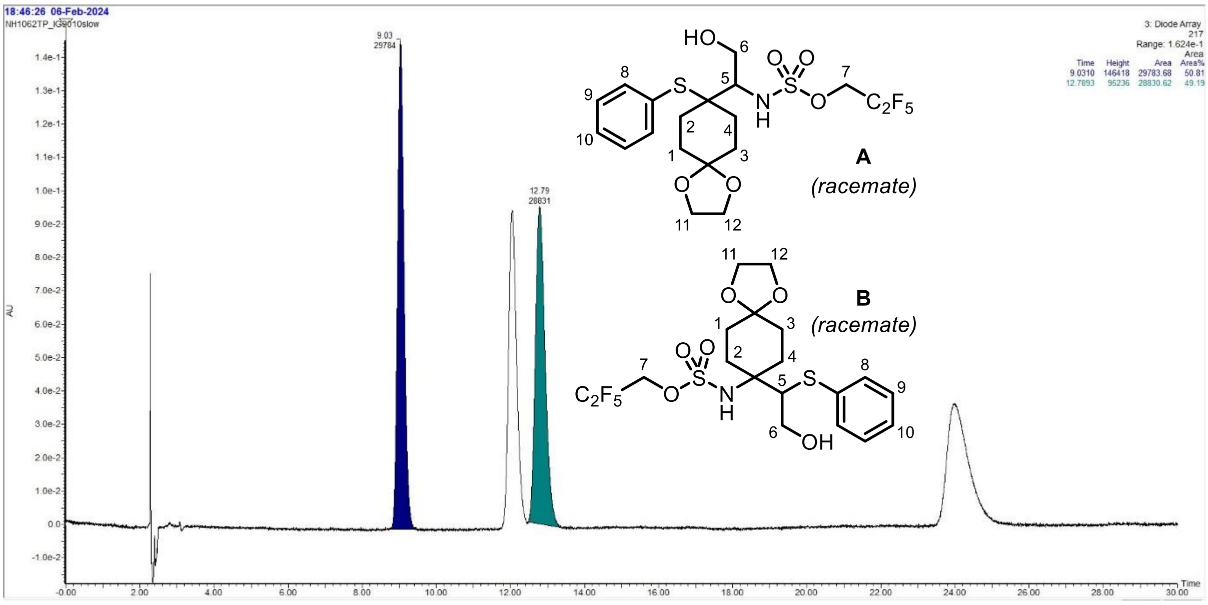

Note: in the racemic trace, a slight difference in absorption maxima for each pair of regioisomer enantiomers enables identification of the correct enantiomeric pairs for integration.

2,2,3,3,3-pentafluoropropyl ((2R,3S)-1-hydroxy-3-(phenylthio)hexan-2-yl)sulfamate. **Obtained following derivatisation of 9h**.

Chiral SFC Analysis CHIRALPAK IG (CO_2:MeOH, 95.0:5.0, 2.50 mL min^{-1}, 40 °C, 253 nm) indicated 83% *ee*, t_R = 3.8 (minor), 4.6 (major) minutes. *Note: additional peaks visible in the traces below are attributed to a minor regioisomer which was not detected in NMR analysis.*

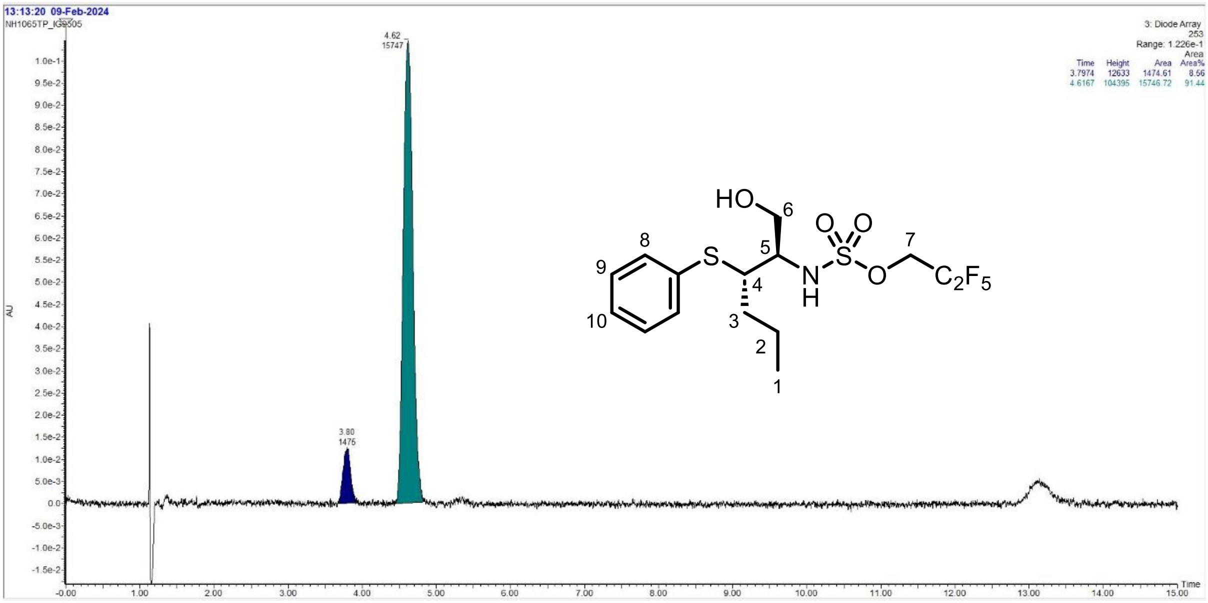

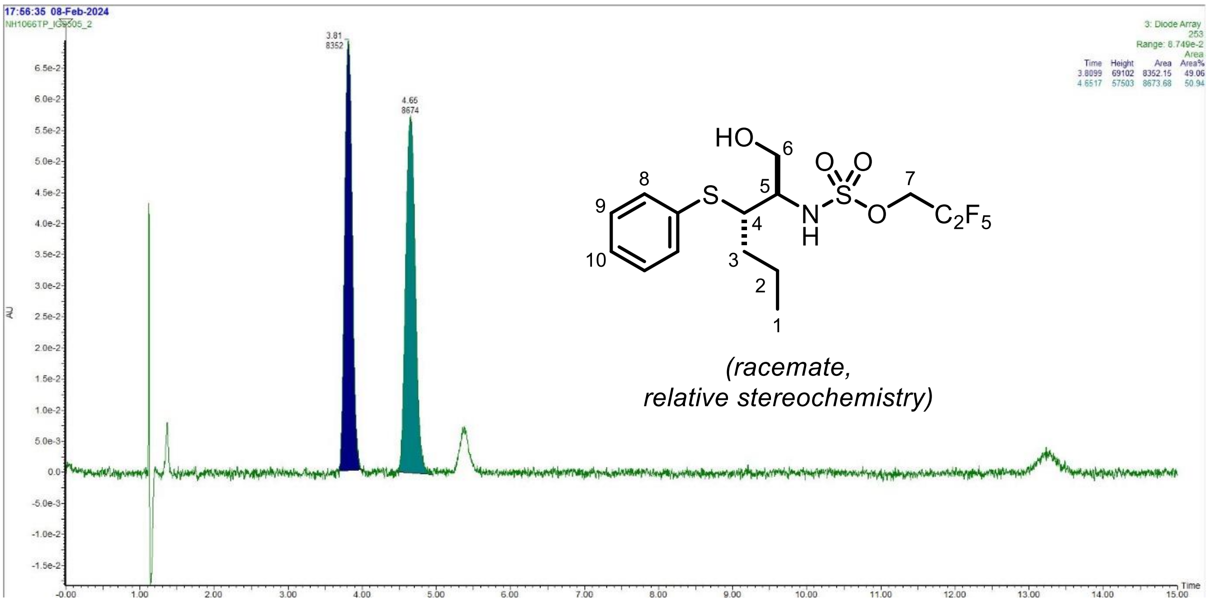

2,2,3,3,3-pentafluoropropyl (R)-(2-hydroxy-1-(1-(phenylthio)cyclopentyl)ethyl)sulfamate (**10a**).

Chiral SFC Analysis CHIRALPAK IG (CO_2:MeOH, 96.0:4.0, 2.50 mL min^{-1}, 40 °C, 217 nm) indicated 92% *ee*, t_R = 12.6 (major), 13.3 (minor) minutes.

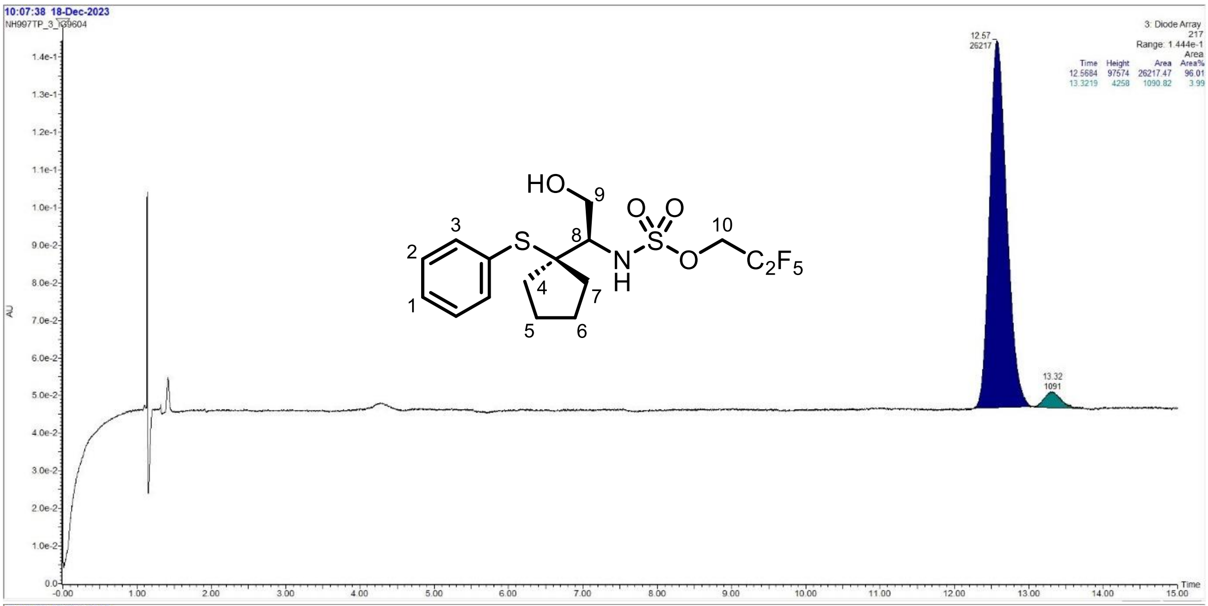

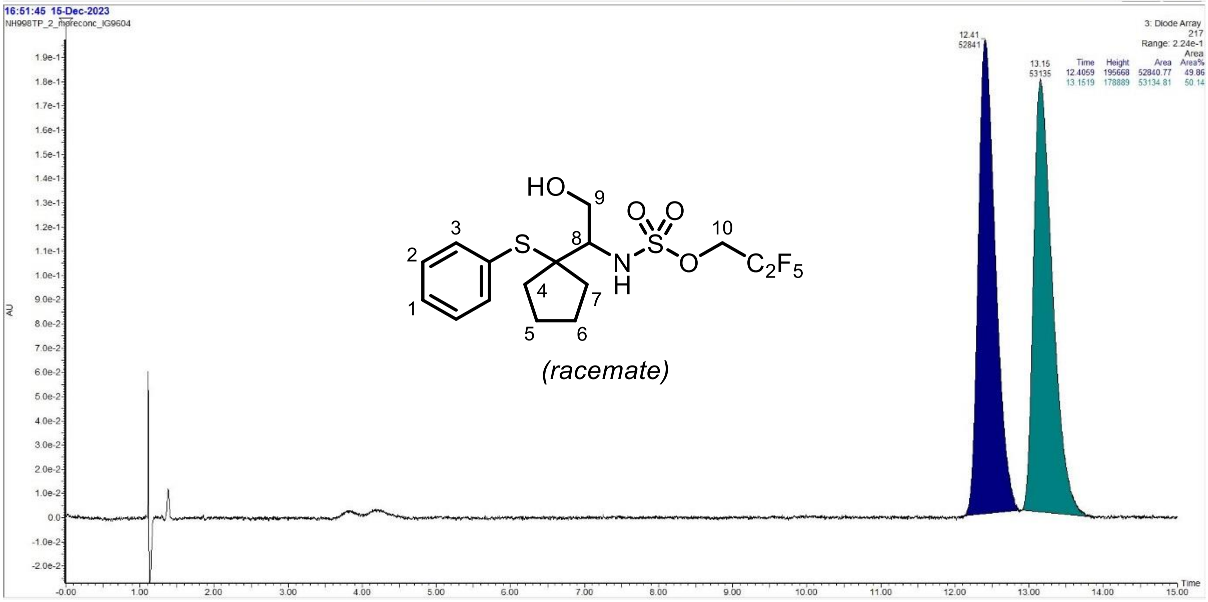

2,2,3,3,3-pentafluoropropyl (R)-(2-hydroxy-1-(1-(phenylthio)cycloheptyl)ethyl)sulfamate (**10b**).

Chiral SFC Analysis CHIRALPAK IG (CO_2:MeOH, 95.0:5.0, 2.50 mL min^{-1}, 40 °C, 218 nm) indicated 91% *ee*, t_R = 7.5 (major), 8.3 (minor) minutes.

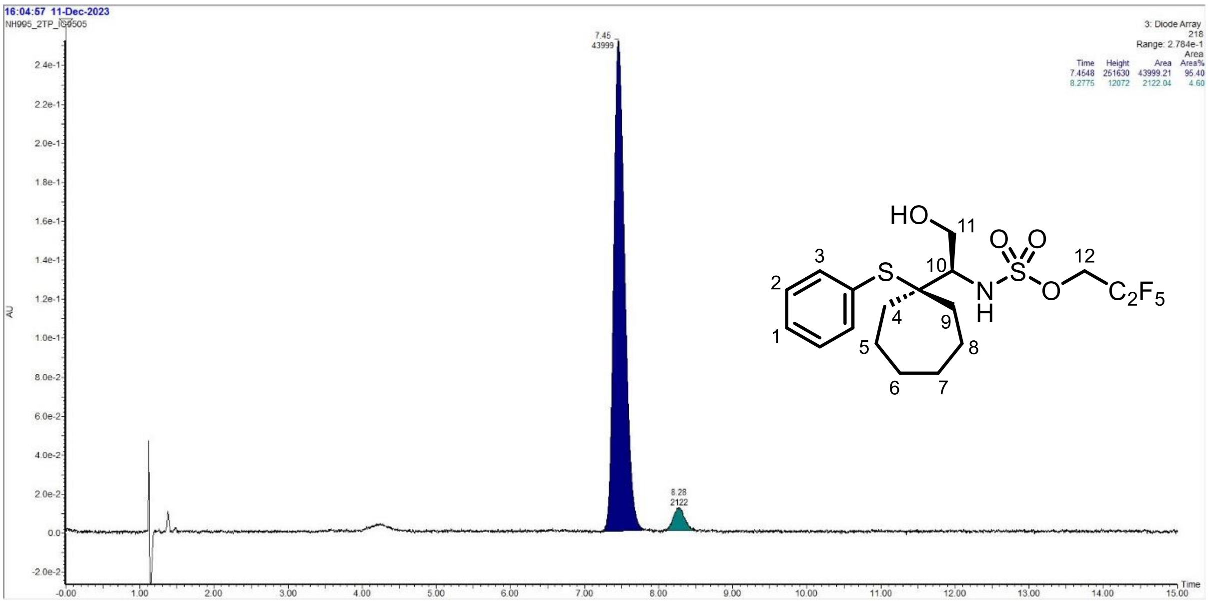

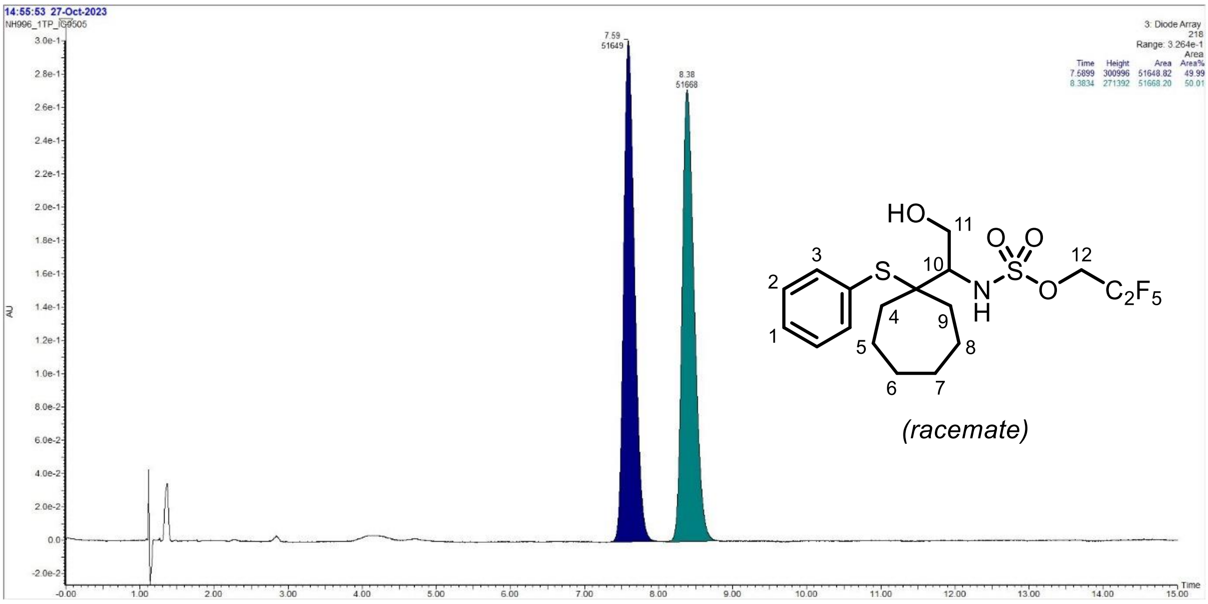

2,2,3,3,3-pentafluoropropyl ((2R,3S)-1-hydroxy-3,7-dimethyl-3-(phenylthio)oct-6-en-2-yl)sulfamate. **Obtained following derivatisation of 9i**.

Chiral SFC Analysis CHIRALPAK IG (CO_2:MeOH, 95.0:5.0, 2.50 mL min^{-1}, 40 °C, 217 nm) indicated 83% *ee*, t_R = 4.3 (major), 6.1 (minor) minutes.

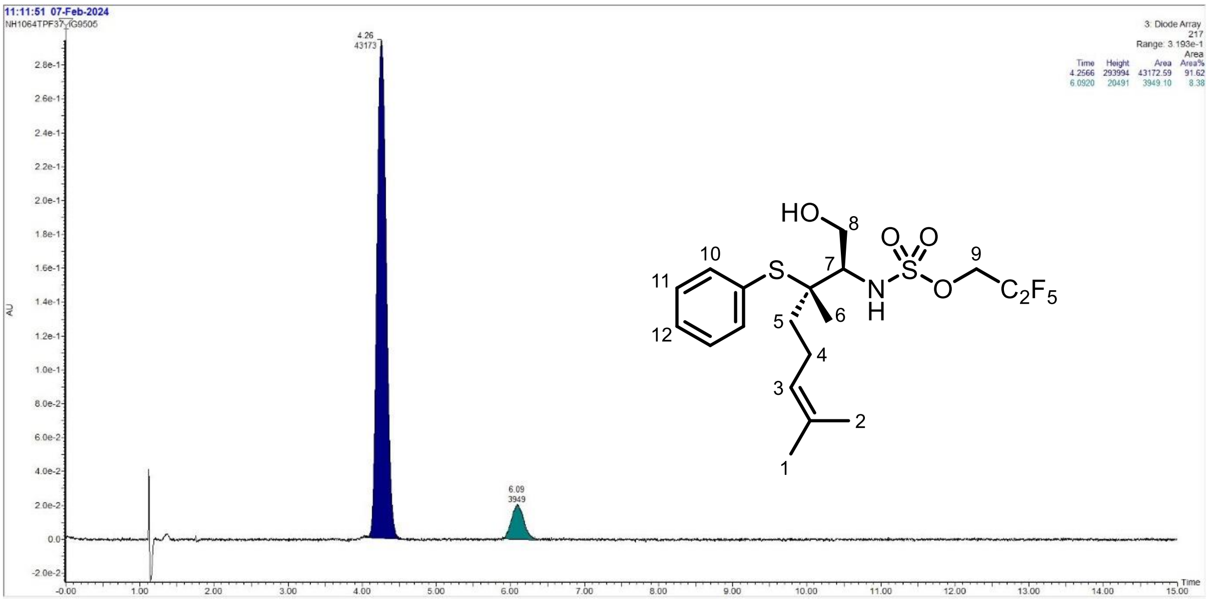

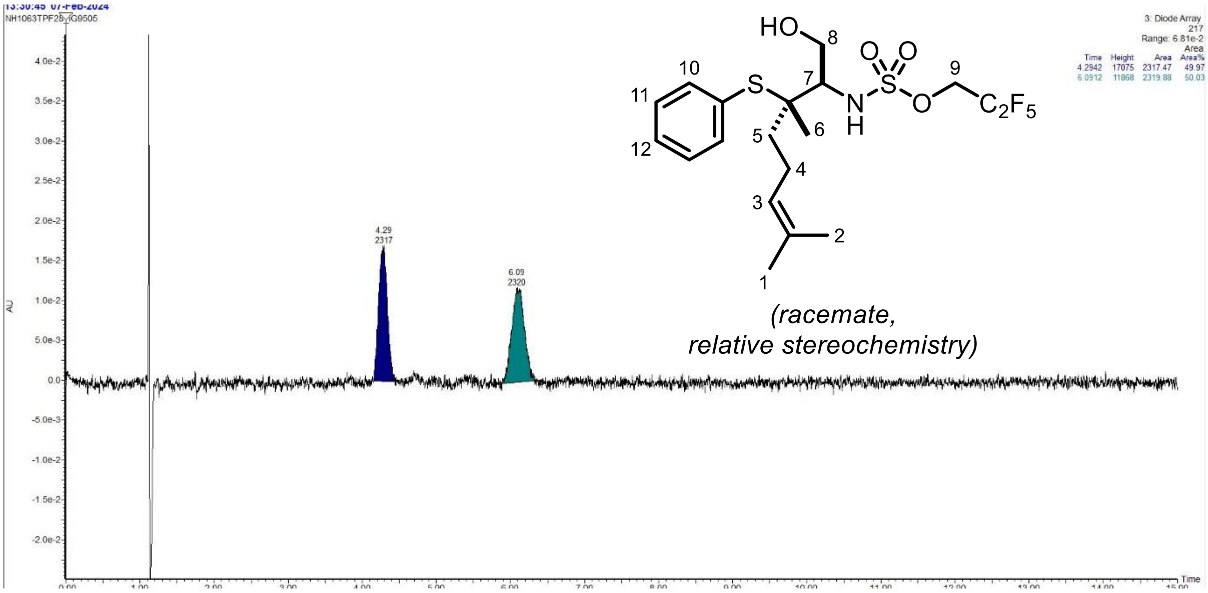

2,2,3,3,3-pentafluoropropyl ((2R,3R)-1-hydroxy-3,7-dimethyl-3-(phenylthio)oct-6-en-2-yl)sulfamate (**A**) and *2,2,3,3,3-pentafluoropropyl ((2R,3S)-1-hydroxy-3,7-dimethyl-2-(phenylthio)oct-6-en-3-yl)sulfamate* (**B**). **Obtained following derivatisation of 9j**.

Chiral SFC Analysis CHIRALPAK IG (CO_2:MeOH, 95.0:5.0, 2.50 mL min^{-1}, 40 °C, 217 nm) for major regioisomer A indicated 84% *ee*, t_R = 4.0 (major), 4.4 (minor) minutes.

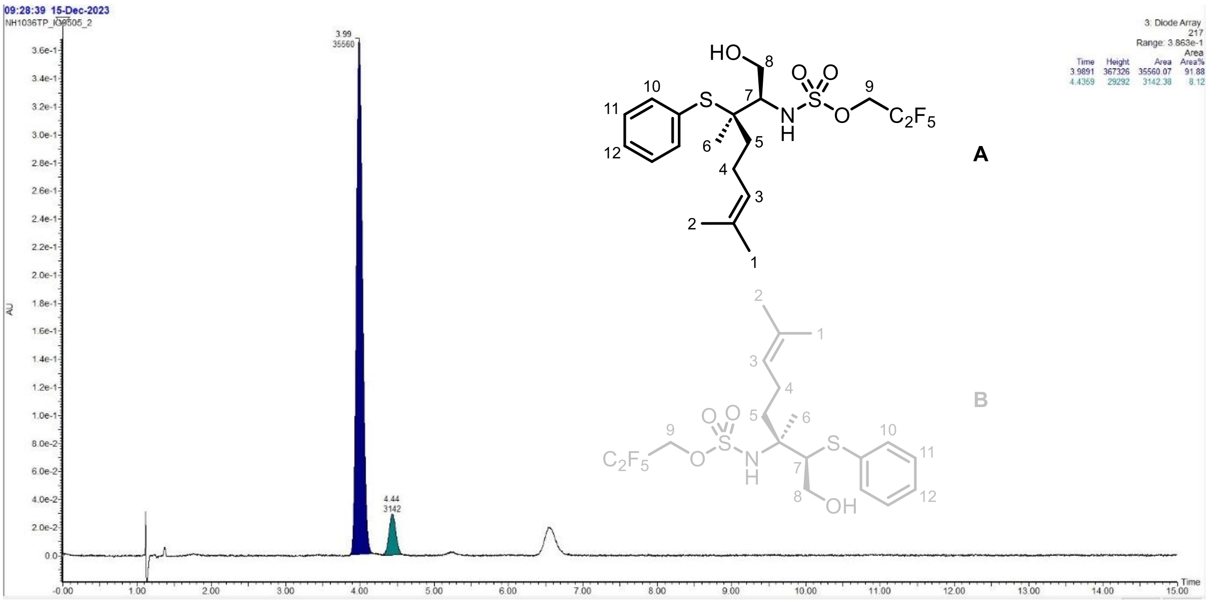

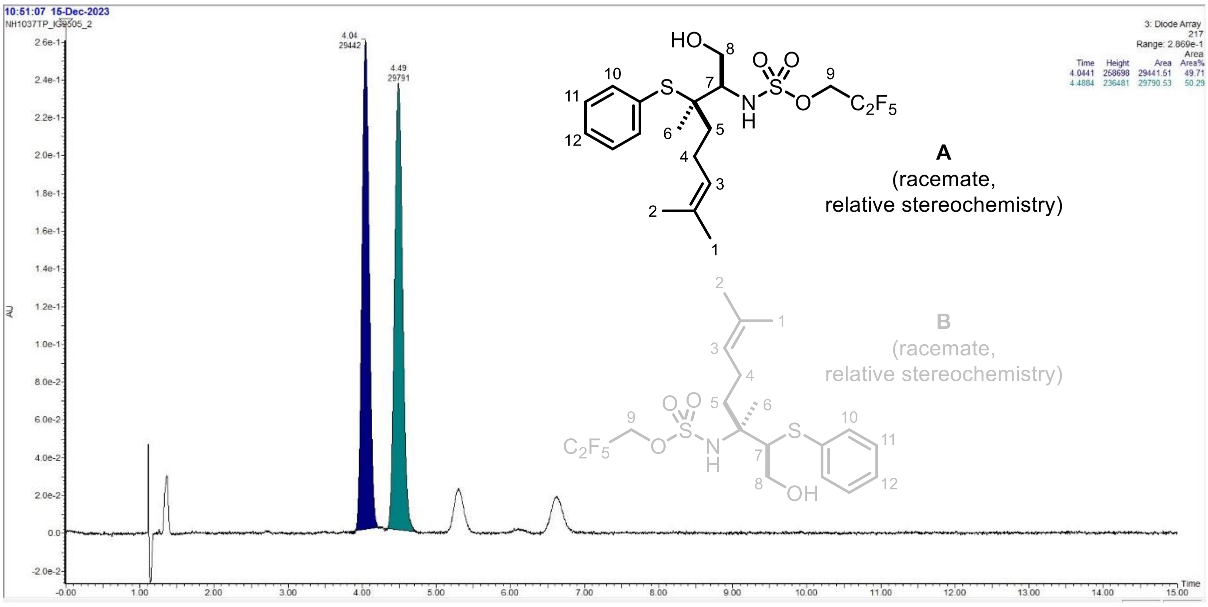

2,2,2-trichloroethyl (R)-(1-hydroxy-3-methyl-3-(phenylthio)butan-2-yl)sulfamate. **Obtained following derivatisation of S2**.

Chiral SFC Analysis CHIRALPAK IE (CO_2:MeOH, 90.0:10.0, 2.50 mL min^{-1}, 40 °C, 217 nm) for major regioisomer A indicated 62% *ee*, t_R = 7.6 (major), 8.8 (minor) minutes.

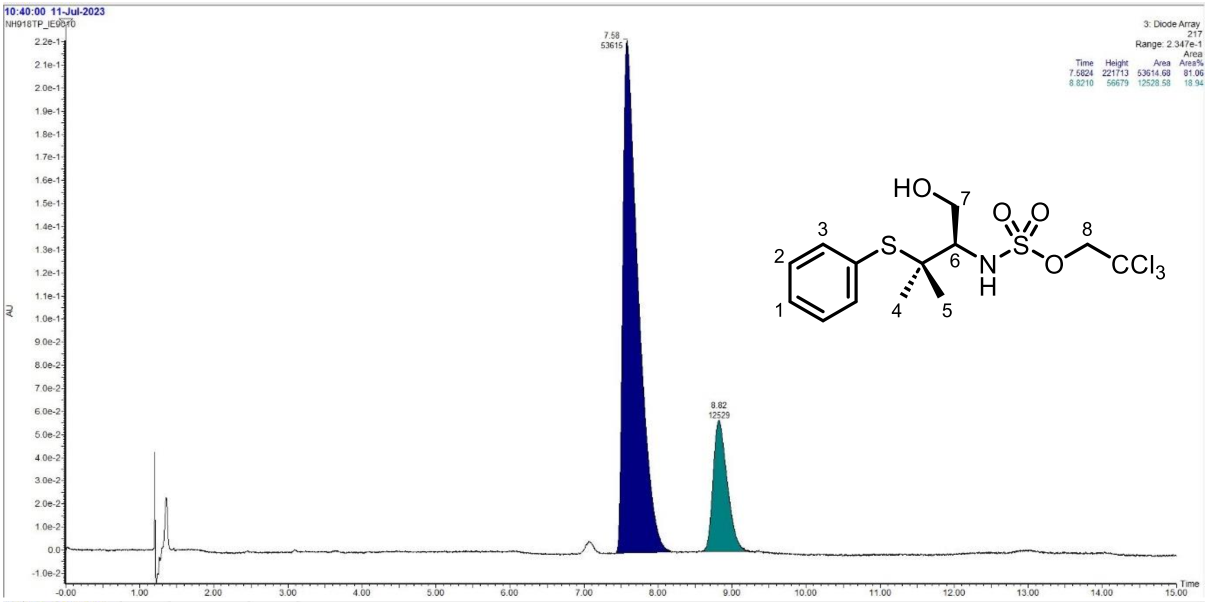

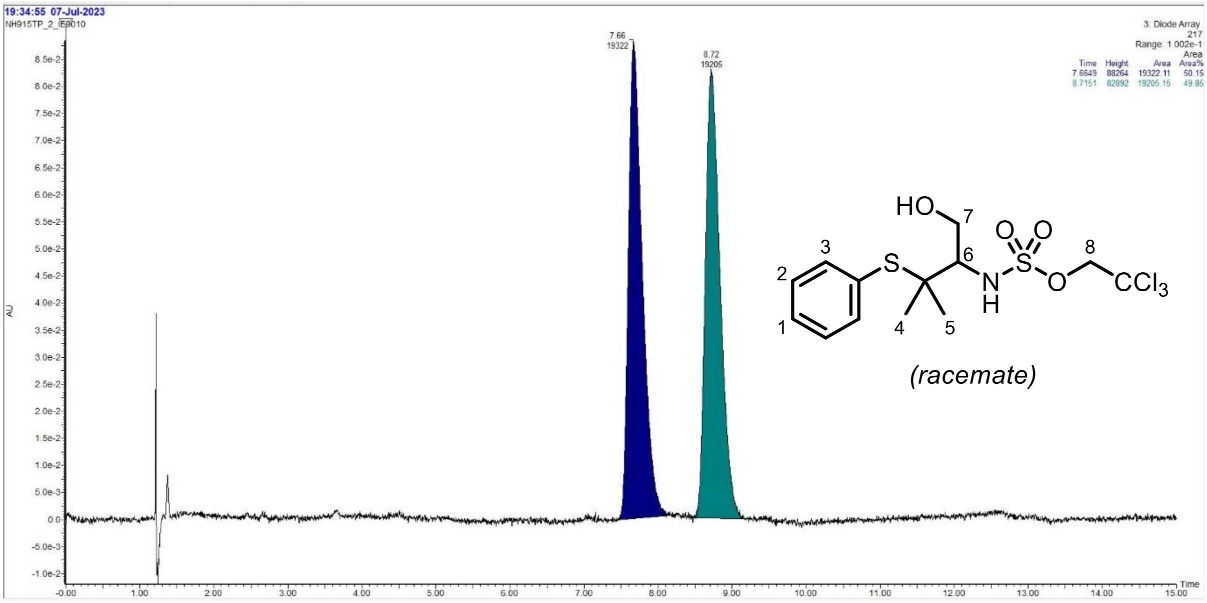

2,2,3,3,3-pentafluoropropyl ((2R,3R)-1-hydroxy-3-(phenylthio)hexan-2-yl)sulfamate (**A**) and *2,2,3,3,3-pentafluoropropyl ((2R,3S)-1-hydroxy-2-(phenylthio)hexan-3-yl)sulfamate* (**B**). **Obtained following aziridination and derivatisation of a *cis*-alkyl allylic alcohol substrate.**

Chiral SFC Analysis CHIRALPAK IK (CO_2:MeOH, 96.0:4.0, 2.50 mL min^{-1}, 40 °C, 253 nm) for major regioisomer A indicated 55% *ee*, t_R = 3.8 (minor), 4.2 (major) minutes.

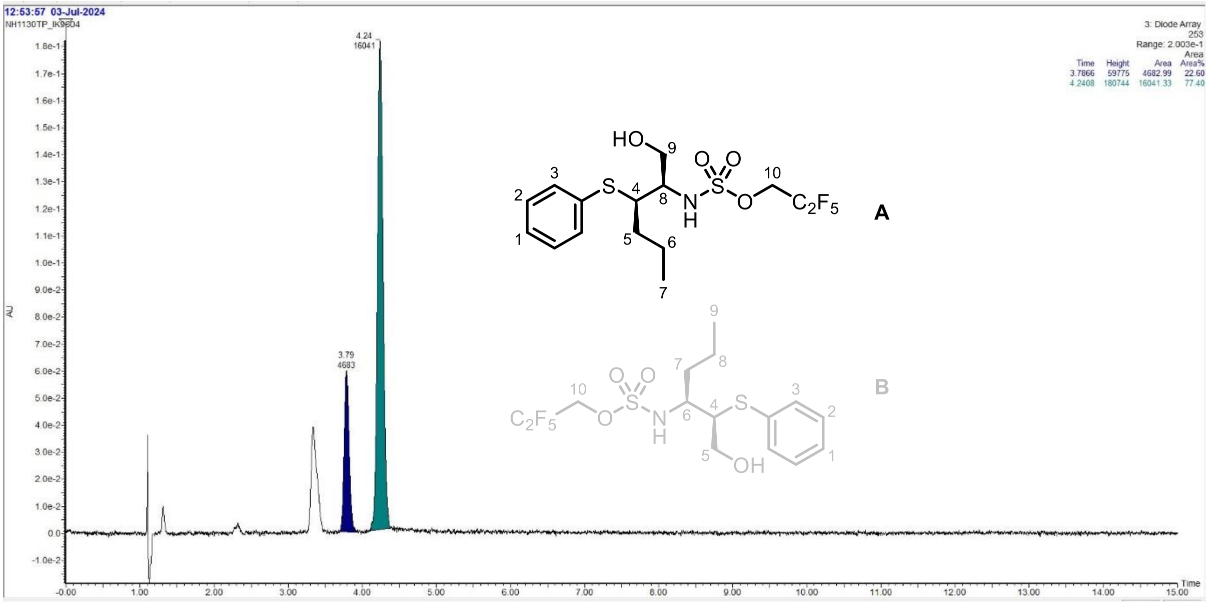

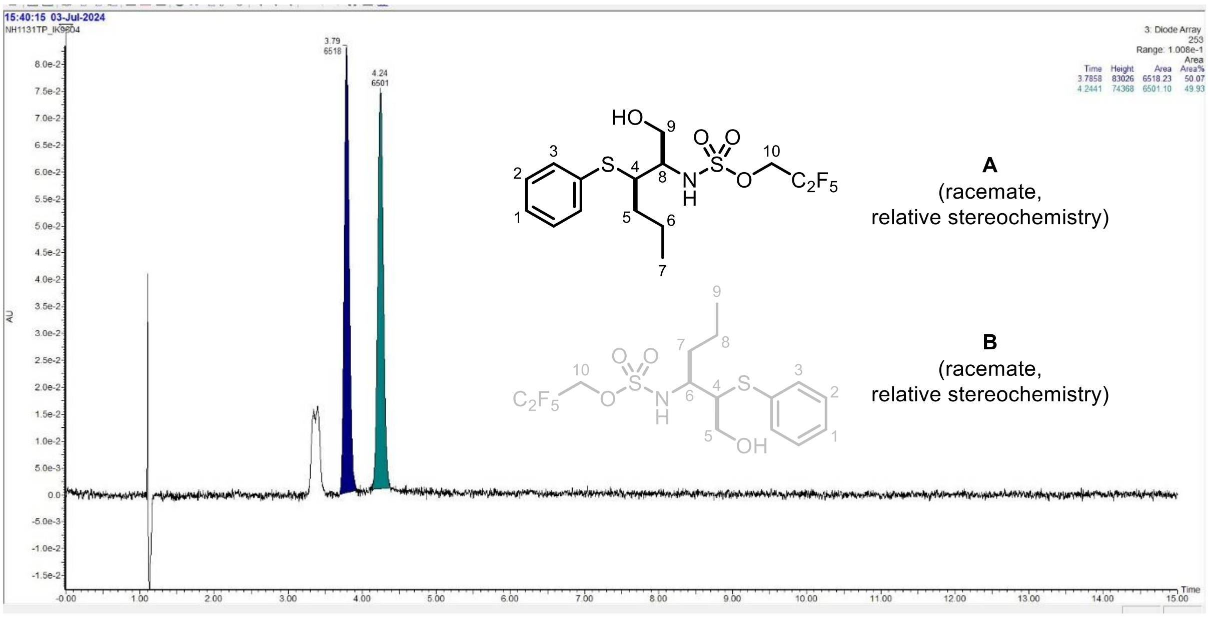

2,2,3,3,3-pentafluoropropyl (S)-(1-hydroxy-3-methylbutan-2-yl)sulfamate (**S5**). **Obtained following derivatisation of 9a**.

Chiral GC Analysis CycloSil-B (25 m × 0.25 mm × 0.25 µm, 160 °C isothermal, 1.47 mL min $^{-1}$) indicated 94% *ee*, t_R = 20.2 (major), 22.0 (minor) minutes.

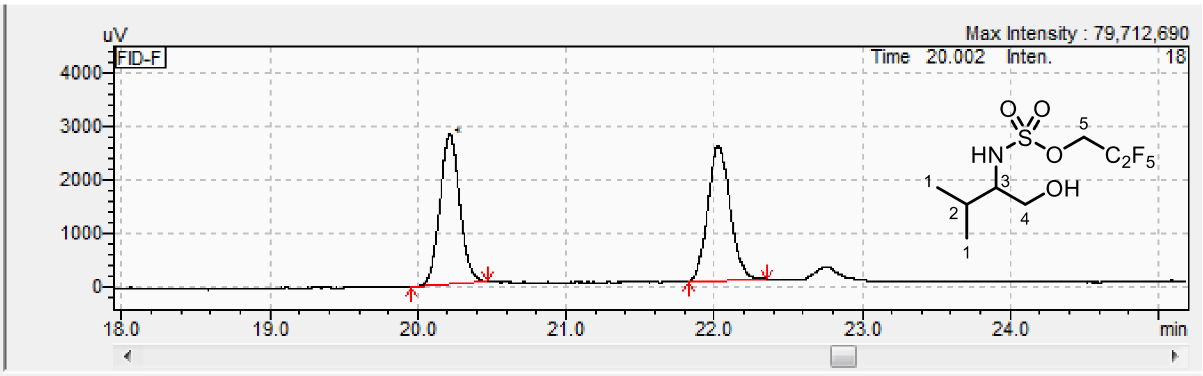

Results View - Peak Table

Peak Table | Compound | Group | Calibration Curve

Peak#	Ret. Time	Area	Conc.	Area%
1	20.216	26049	49.693	49.693
2	22.027	26370	50.307	50.307
Total		52419	100.000	100.000

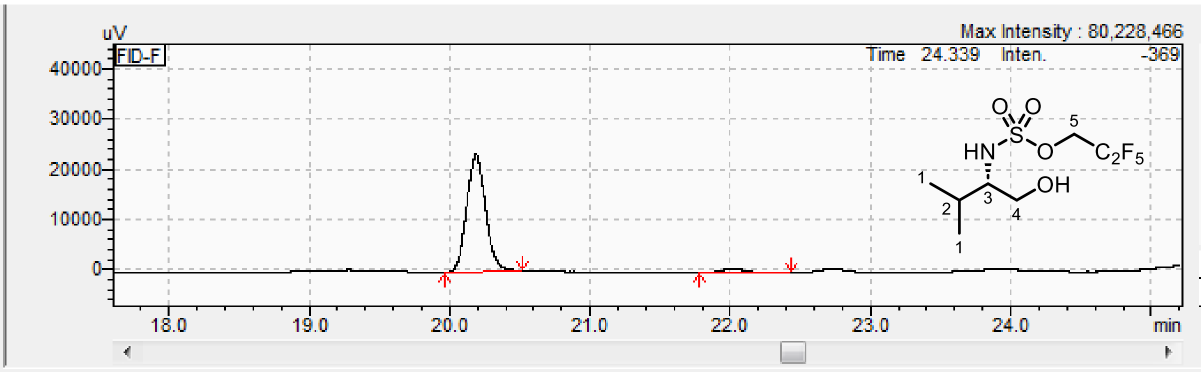

Results View - Peak Table

Peak Table | Compound | Group | Calibration Curve

Peak#	Ret. Time	Area	Conc.	Area%
1	20.191	218450	96.827	96.827
2	22.023	7160	3.173	3.173
Total		225609	100.000	100.000

2,2,3,3,3-pentafluoropropyl (S)-(1-hydroxy-3-methylbutan-2-yl)sulfamate (**S5**). **Obtained following derivatisation of L-valinol**.

Chiral GC Analysis CycloSil-B (25 m × 0.25 mm × 0.25 µm, 160 °C isothermal, 1.47 mL min $^{-1}$) indicated >99% *ee*, t_R = 20.2 minutes.

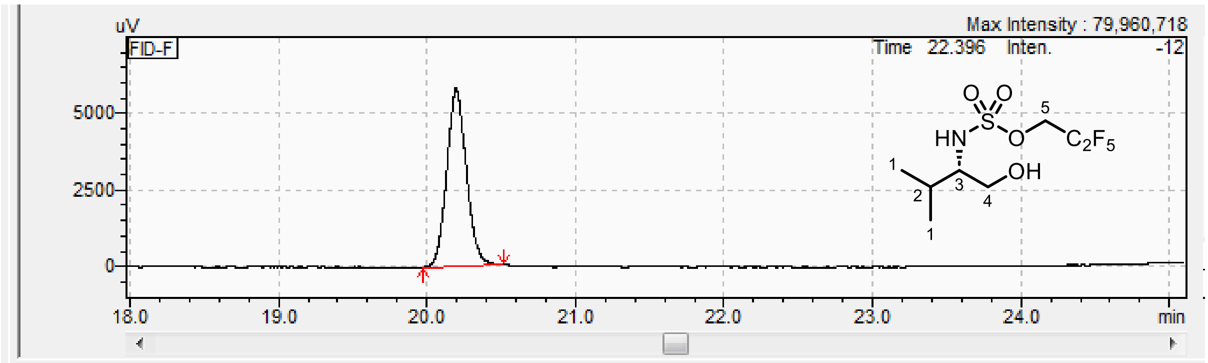

Results View - Peak Table

Peak Table | Compound | Group | Calibration Curve

Peak#	Ret. Time	Area	Conc.	Area%
1	20.194	53256	100.000	100.000
Total		53256	100.000	100.000

2,2,3,3,3-pentafluoropropyl (R)-(1-hydroxy-3-methylbutan-2-yl)sulfamate (**S5-ent**). **Obtained following derivatisation of D-valinol**.

Chiral GC Analysis CycloSil-B (25 m × 0.25 mm × 0.25 µm, 160 °C isothermal, 1.47 mL min $^{-1}$) indicated >99% *ee*, t_R = 22.0 minutes.

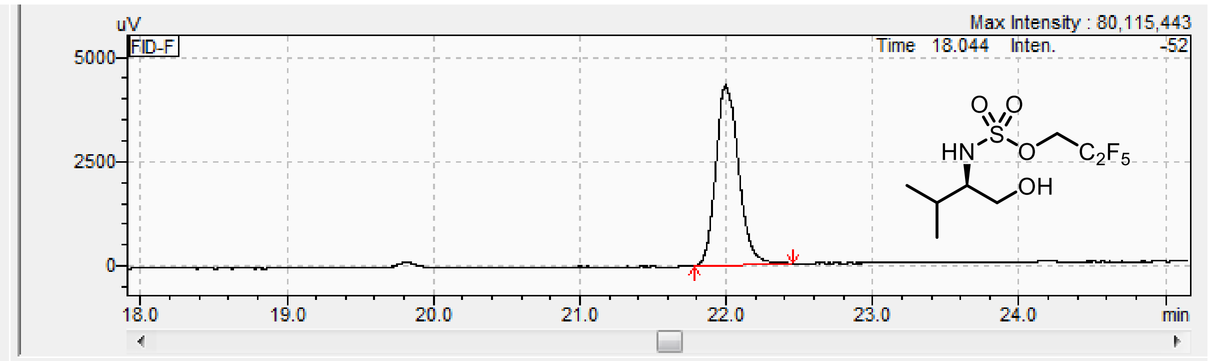

Results View - Peak Table

Peak Table | Compound | Group | Calibration Curve

Peak#	Ret. Time	Area	Conc.	Area%
1	21.997	46676	100.000	100.000
Total		46676	100.000	100.000

NMR Spectra

¹H NMR (400 MHz, $CDCl_3$) for *(4-(2-hydroxyethyl)piperidin-1-yl)(phenyl)methanone* (**11a'**)

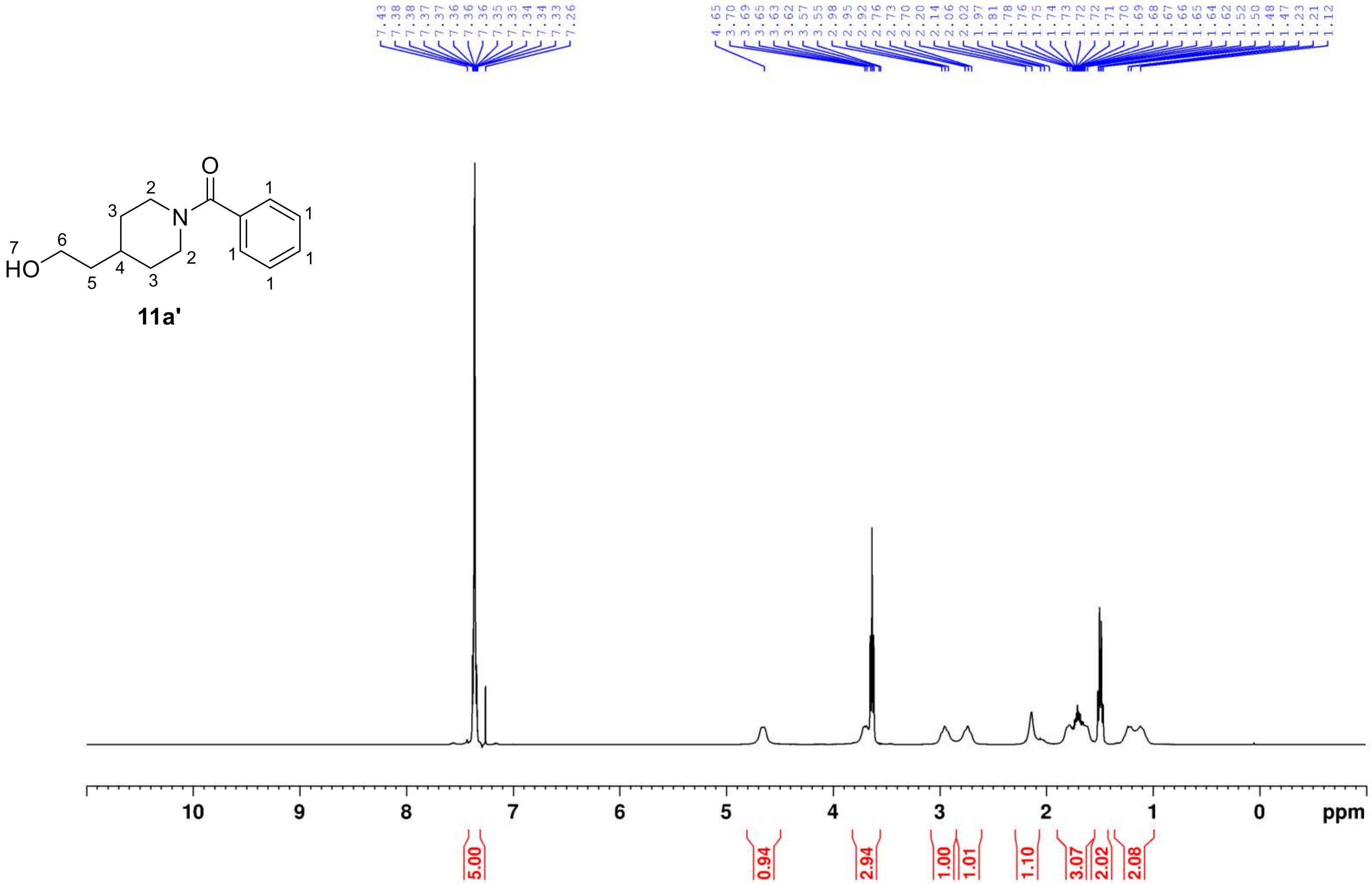

^{13}C NMR (101 MHz, $CDCl_3$) for *(4-(2-hydroxyethyl)piperidin-1-yl)(phenyl)methanone* (**11a'**)

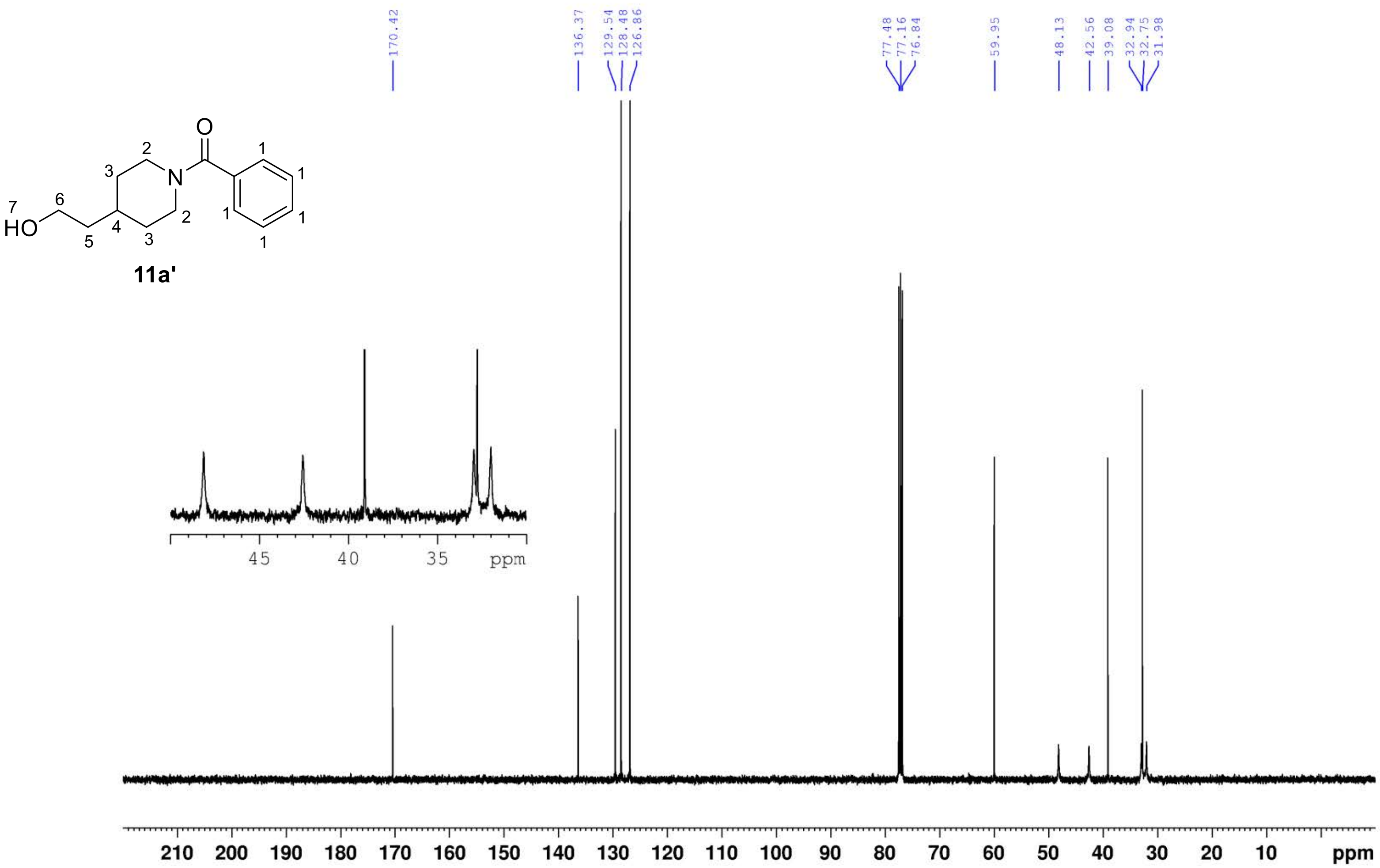

¹H NMR (400 MHz, $CDCl_3$) for *2-(1-benzoylpiperidin-4-yl)acetaldehyde* (**11b**)

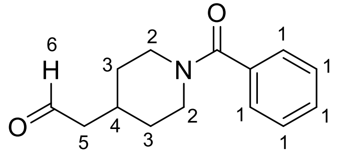

11b

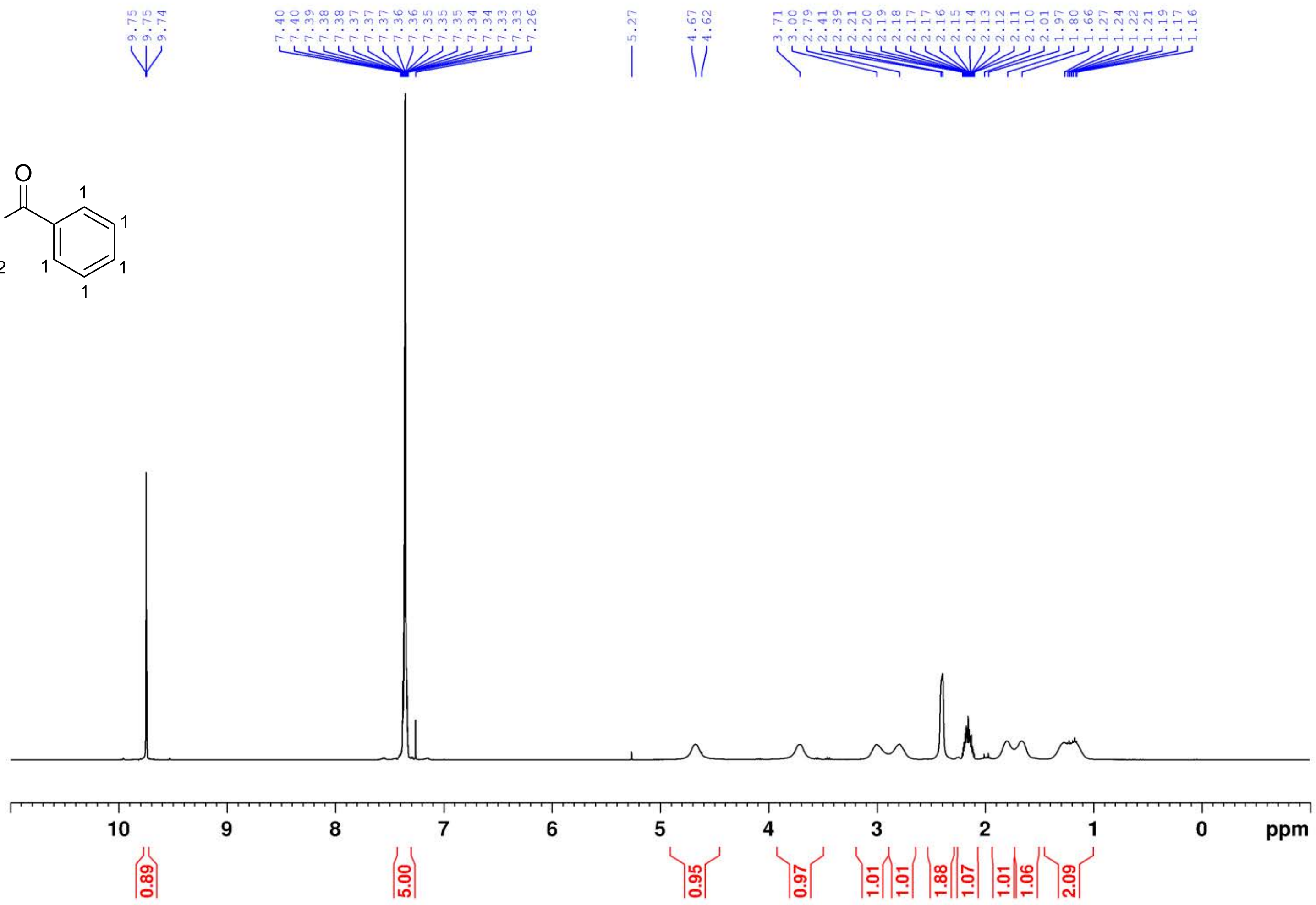

^{13}C NMR (101 MHz, $CDCl_3$) for *2-(1-benzoylpiperidin-4-yl)acetaldehyde* (**11b**)

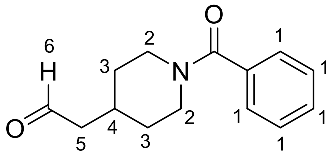

11b

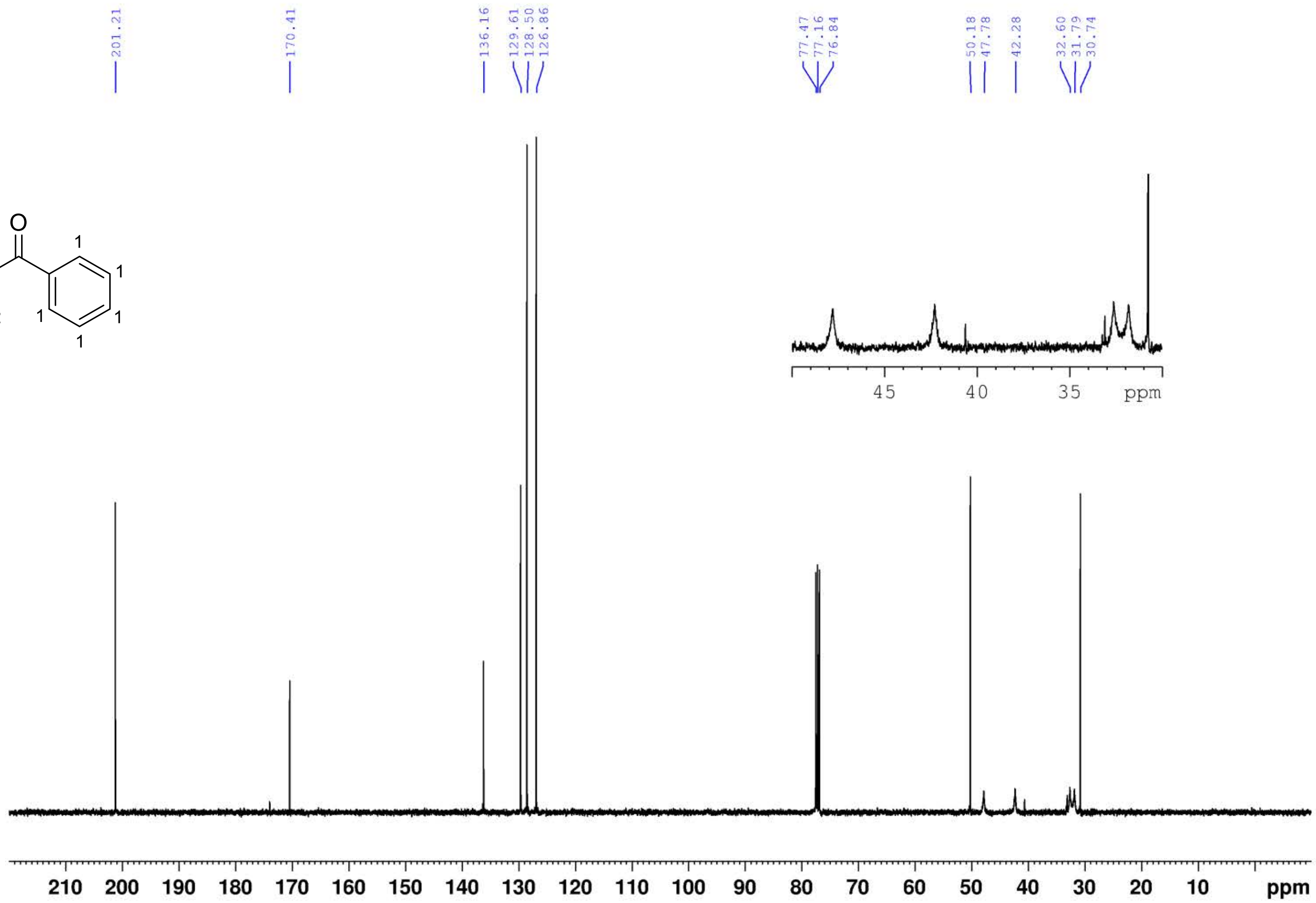

^{1}H NMR (400 MHz, $CDCl_3$) for *(E)-phenyl(4-(3-(4,4,5,5-tetramethyl-1,3,2-dioxaborolan-2-yl)allyl)piperidin-1-yl)methanone* (**11c**)

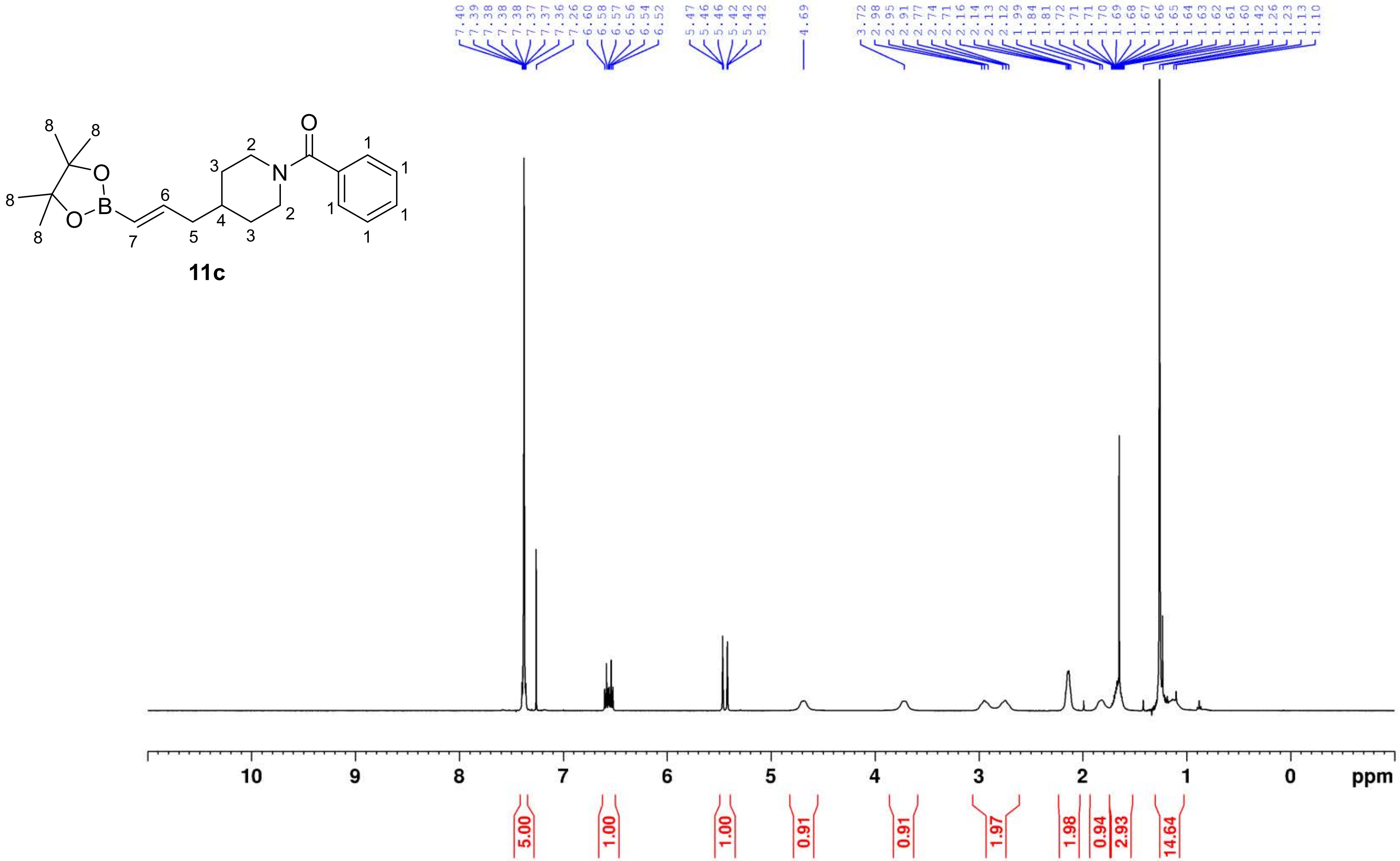

11c

^{13}C NMR (101 MHz, $CDCl_3$) for *(E)-phenyl(4-(3-(4,4,5,5-tetramethyl-1,3,2-dioxaborolan-2-yl)allyl)piperidin-1-yl)methanone* (**11c**)

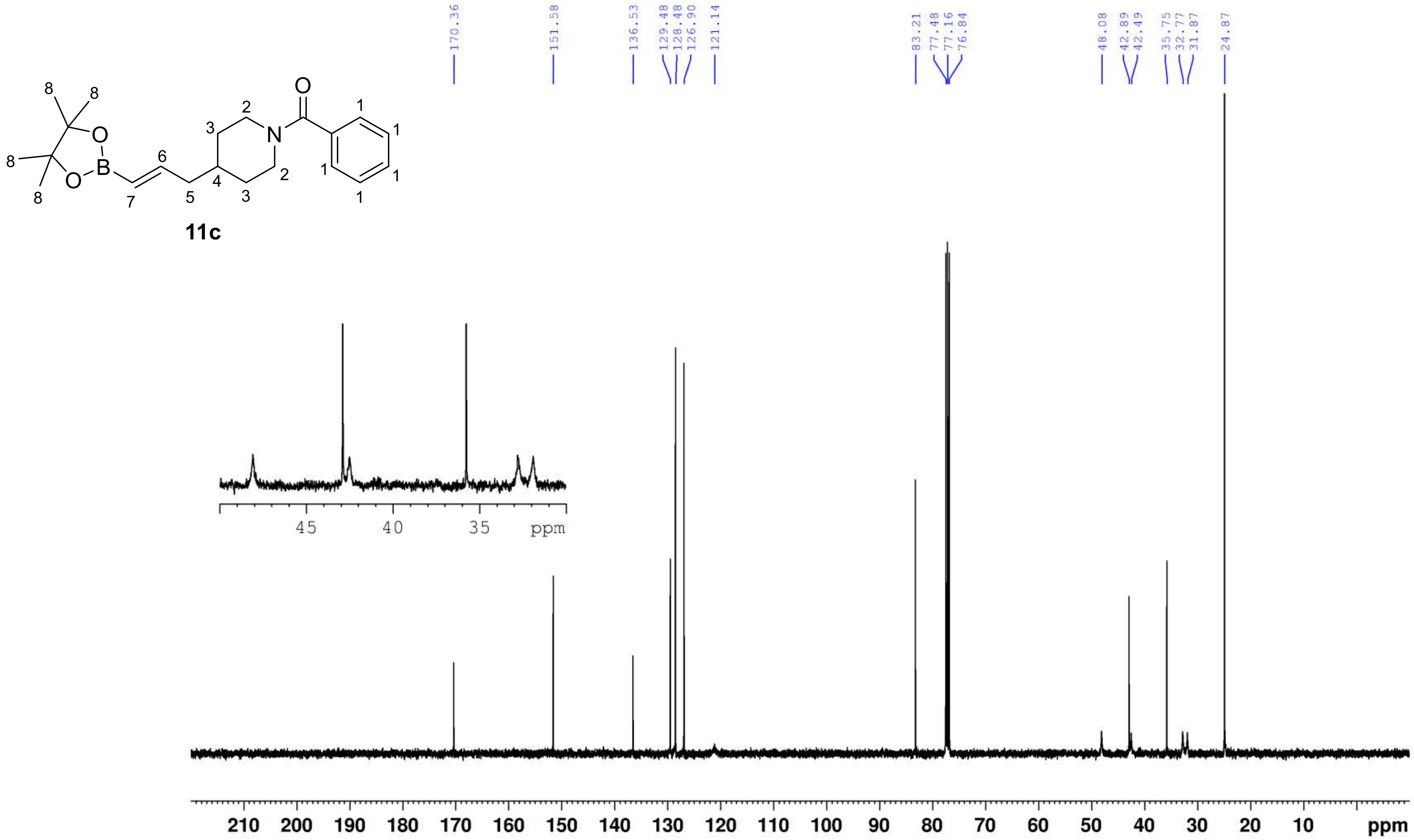

^{1}H NMR (400 MHz, $CDCl_3$) for *(E)-(4-(3-(naphthalen-1-yl)allyl)piperidin-1-yl)(phenyl)methanone* (**11d**)

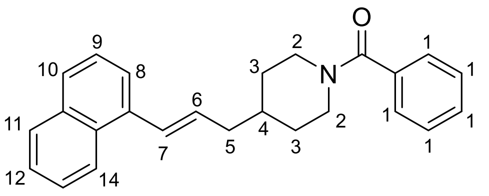

11d

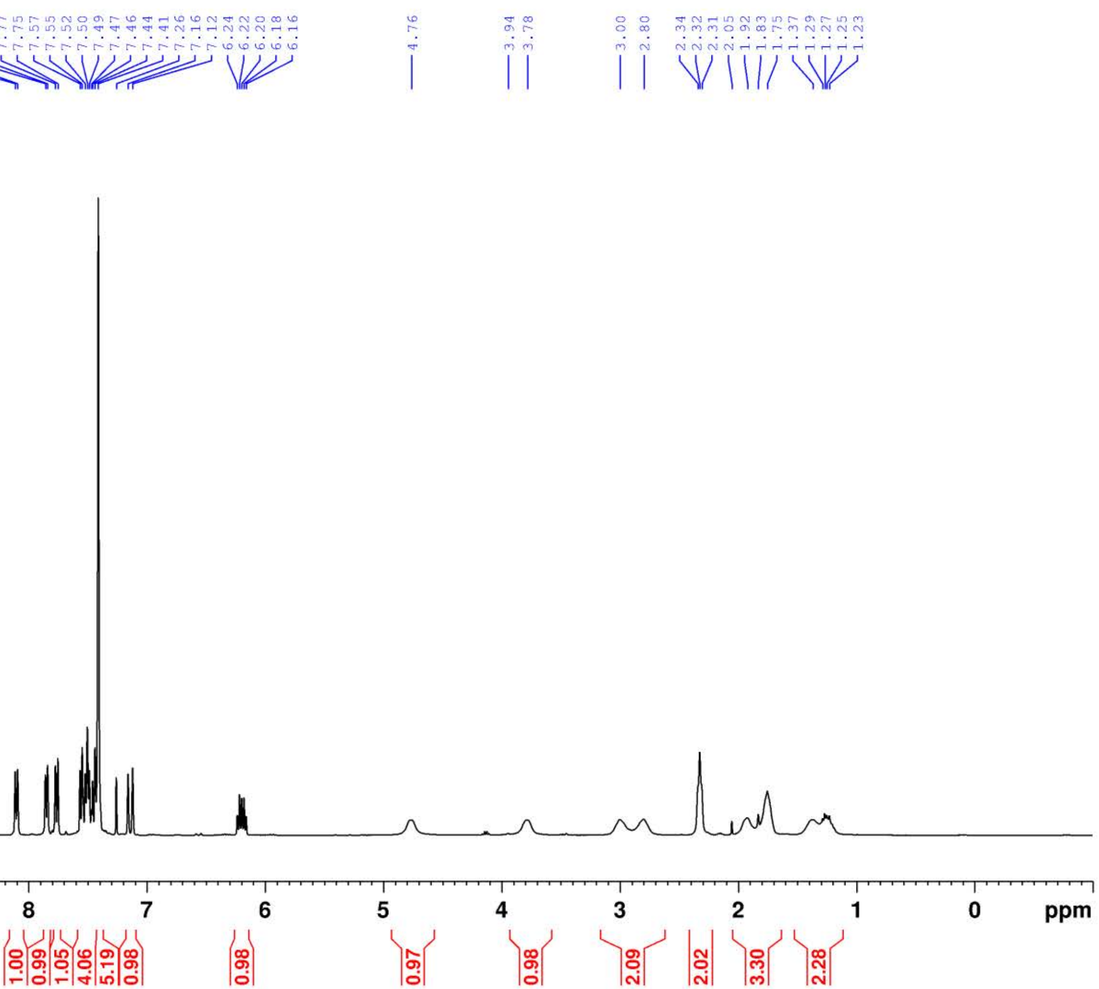

^{13}C NMR (101 MHz, $CDCl_3$) for *(E)-(4-(3-(naphthalen-1-yl)allyl)piperidin-1-yl)(phenyl)methanone* (**11d**)

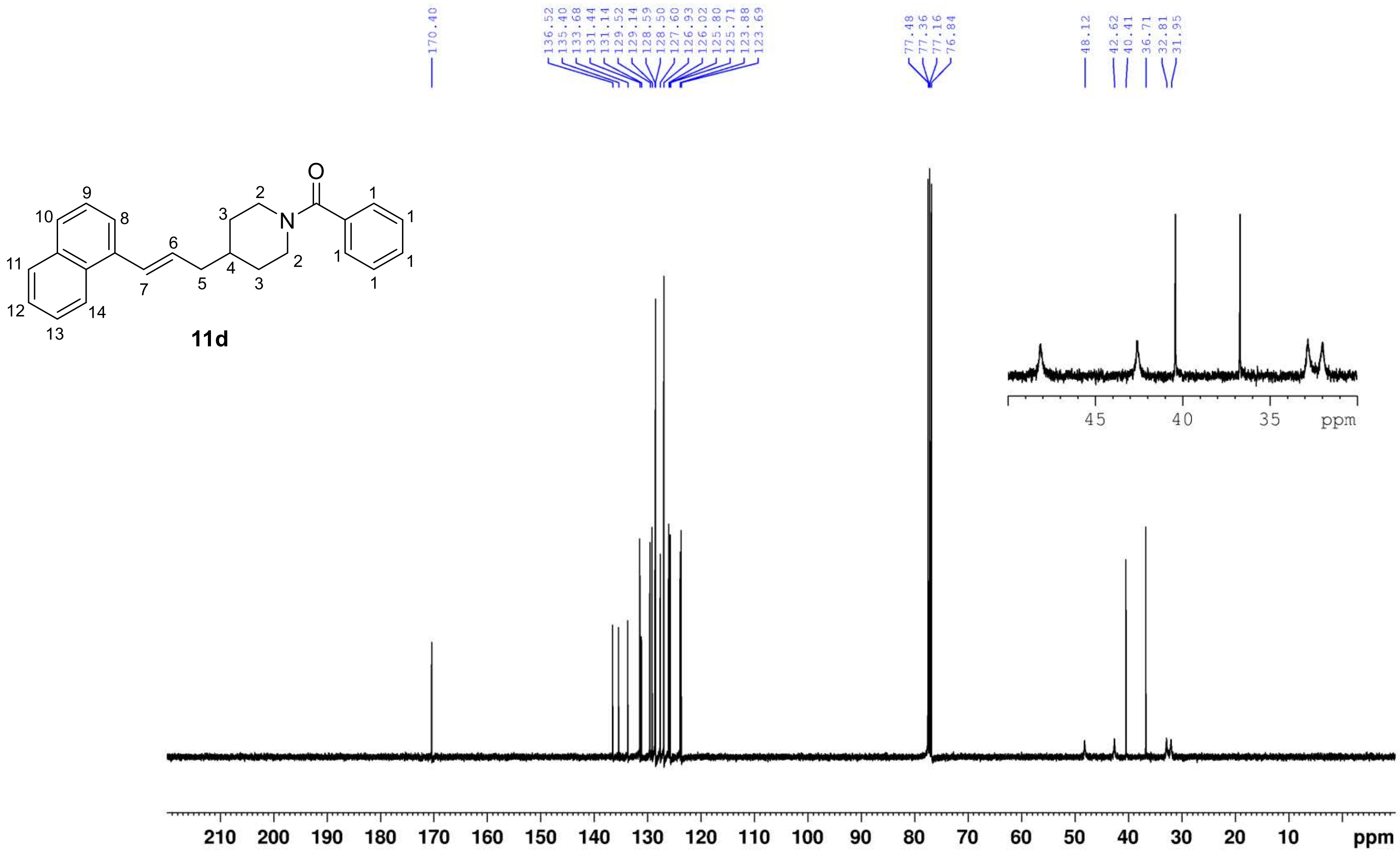

^{1}H NMR (400 MHz, $CDCl_3$) for *(4-((2S,3S)-2,3-dihydroxy-3-(naphthalen-1-yl)propyl)piperidin-1-yl)(phenyl)methanone* (**11d'**)

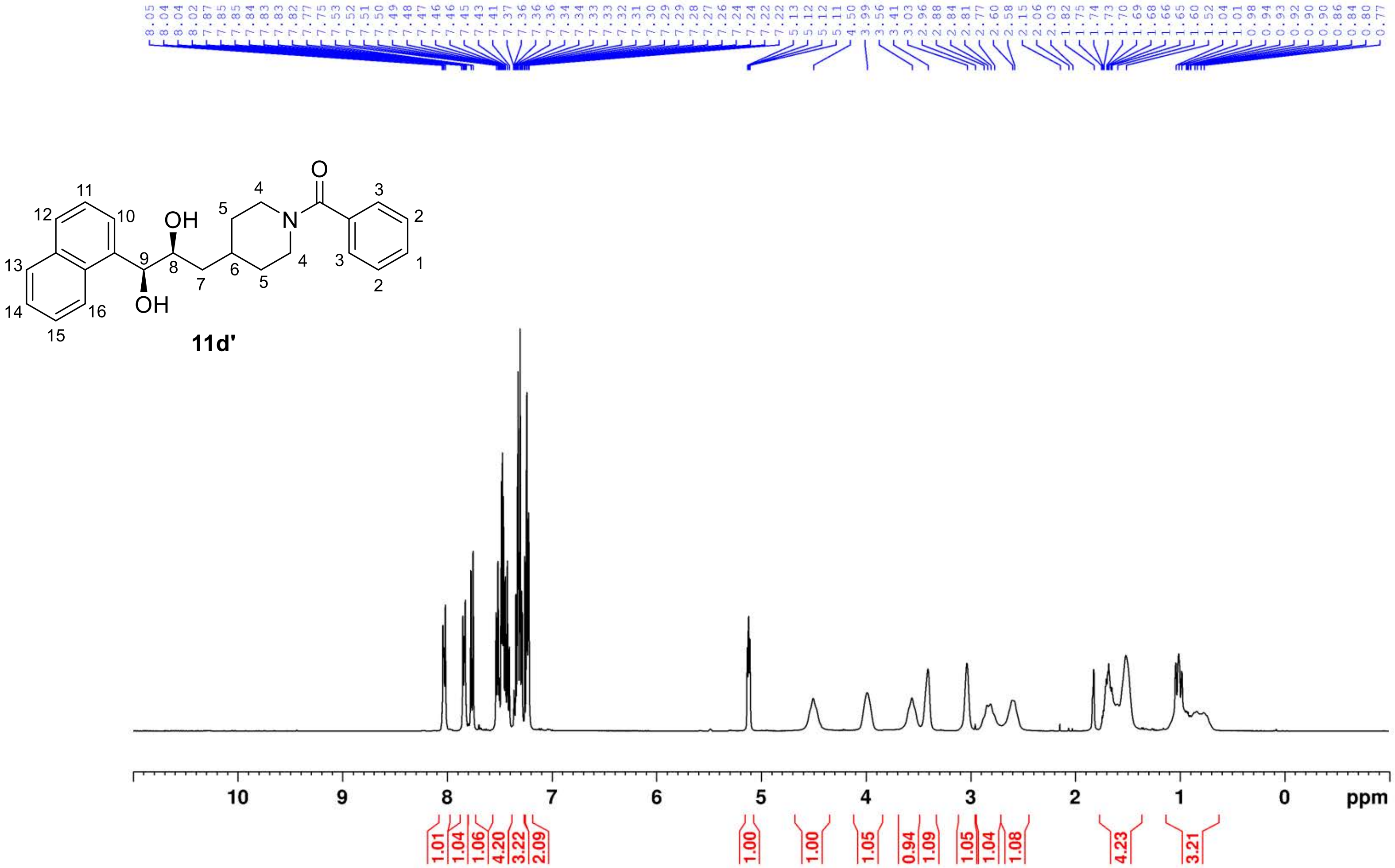

11d'

^{13}C NMR (101 MHz, $CDCl_3$) for *(4-((2S,3S)-2,3-dihydroxy-3-(naphthalen-1-yl)propyl)piperidin-1-yl)(phenyl)methanone* (**11d'**)

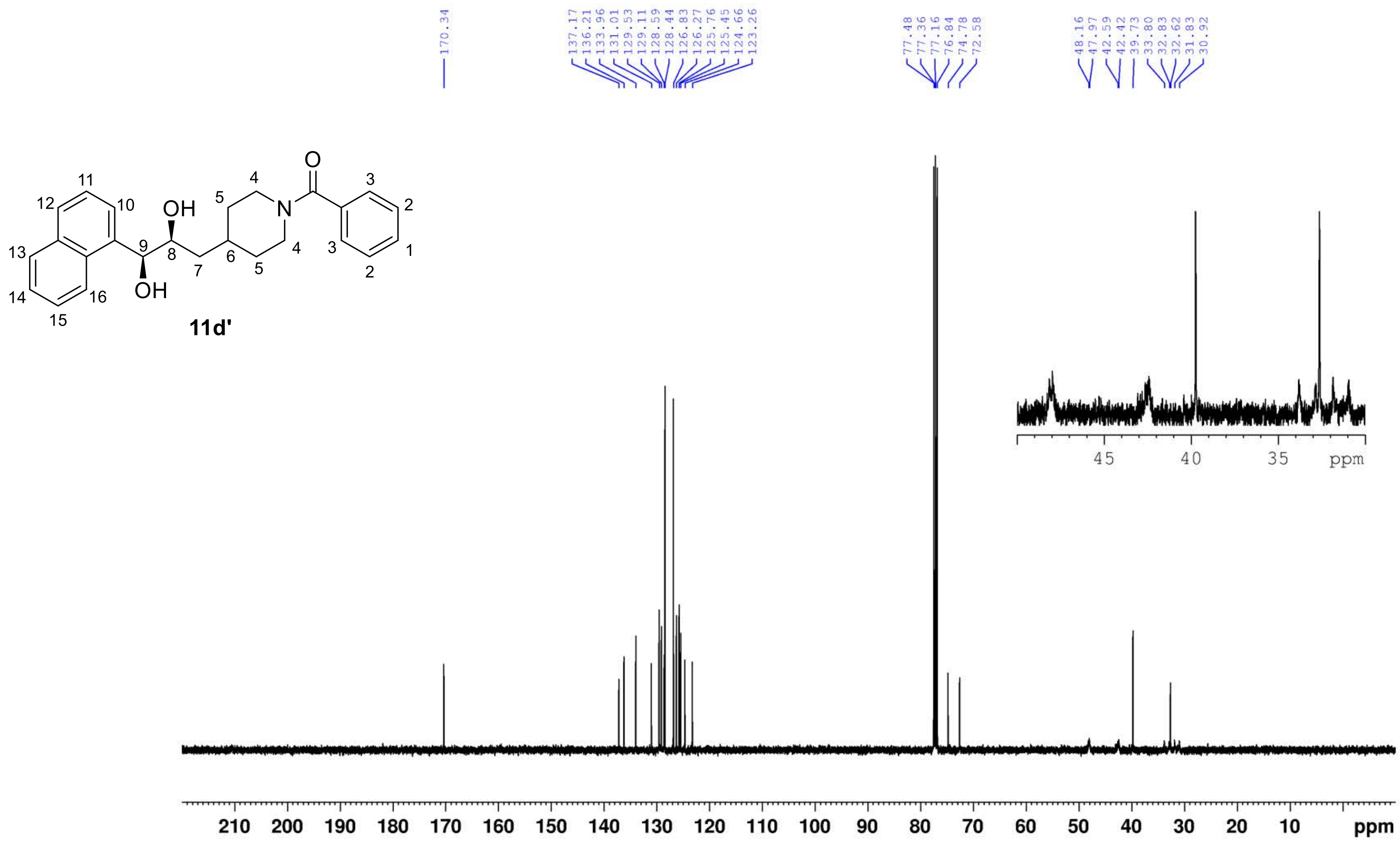

¹H NMR (700 MHz, $CDCl_3$) for *(4-(((2S,3S)-3-(naphthalen-1-yl)oxiran-2-yl)methyl)piperidin-1-yl)(phenyl)methanone* (**11e**)

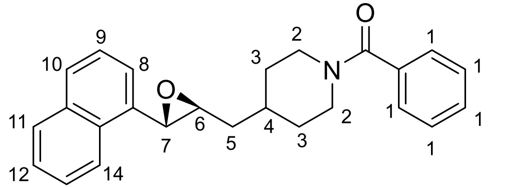

11e

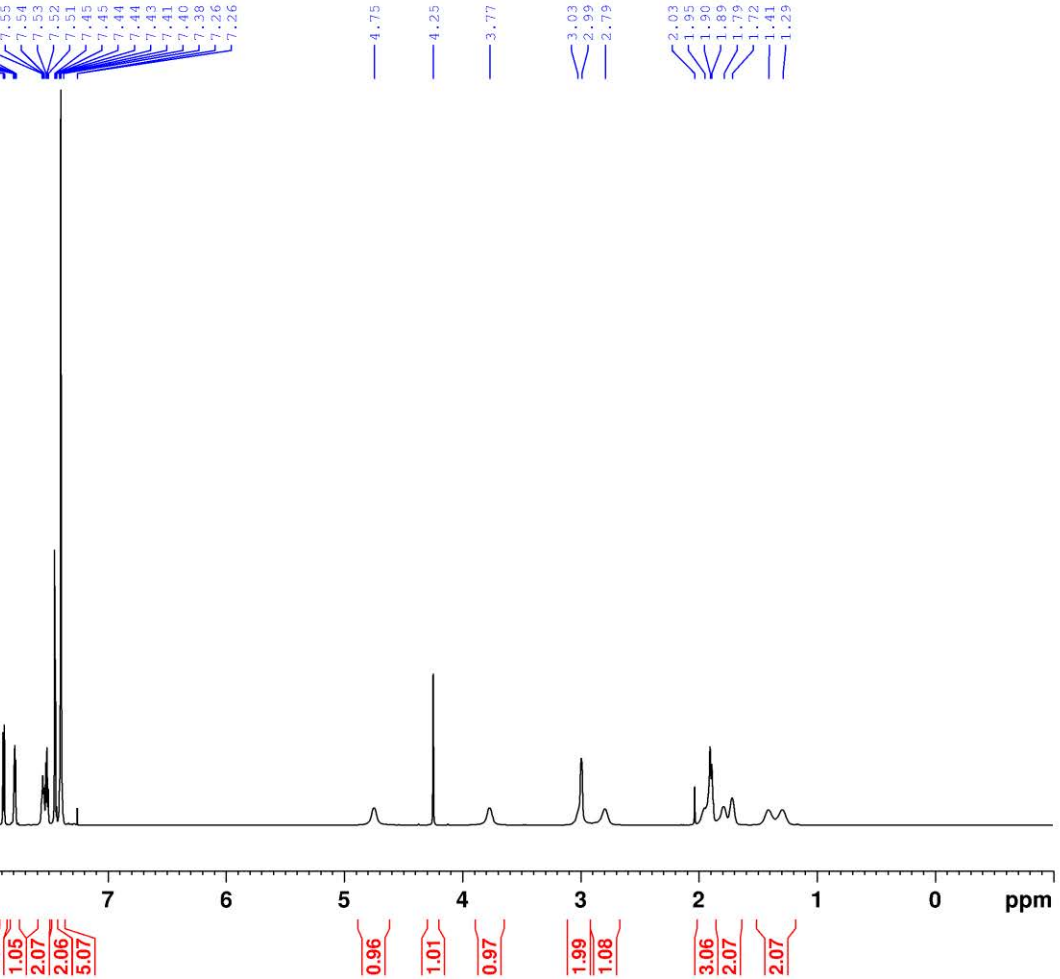

^{13}C NMR (176 MHz, $CDCl_3$) for *(4-(((2S,3S)-3-(naphthalen-1-yl)oxiran-2-yl)methyl)piperidin-1-yl)(phenyl)methanone* (**11e**)

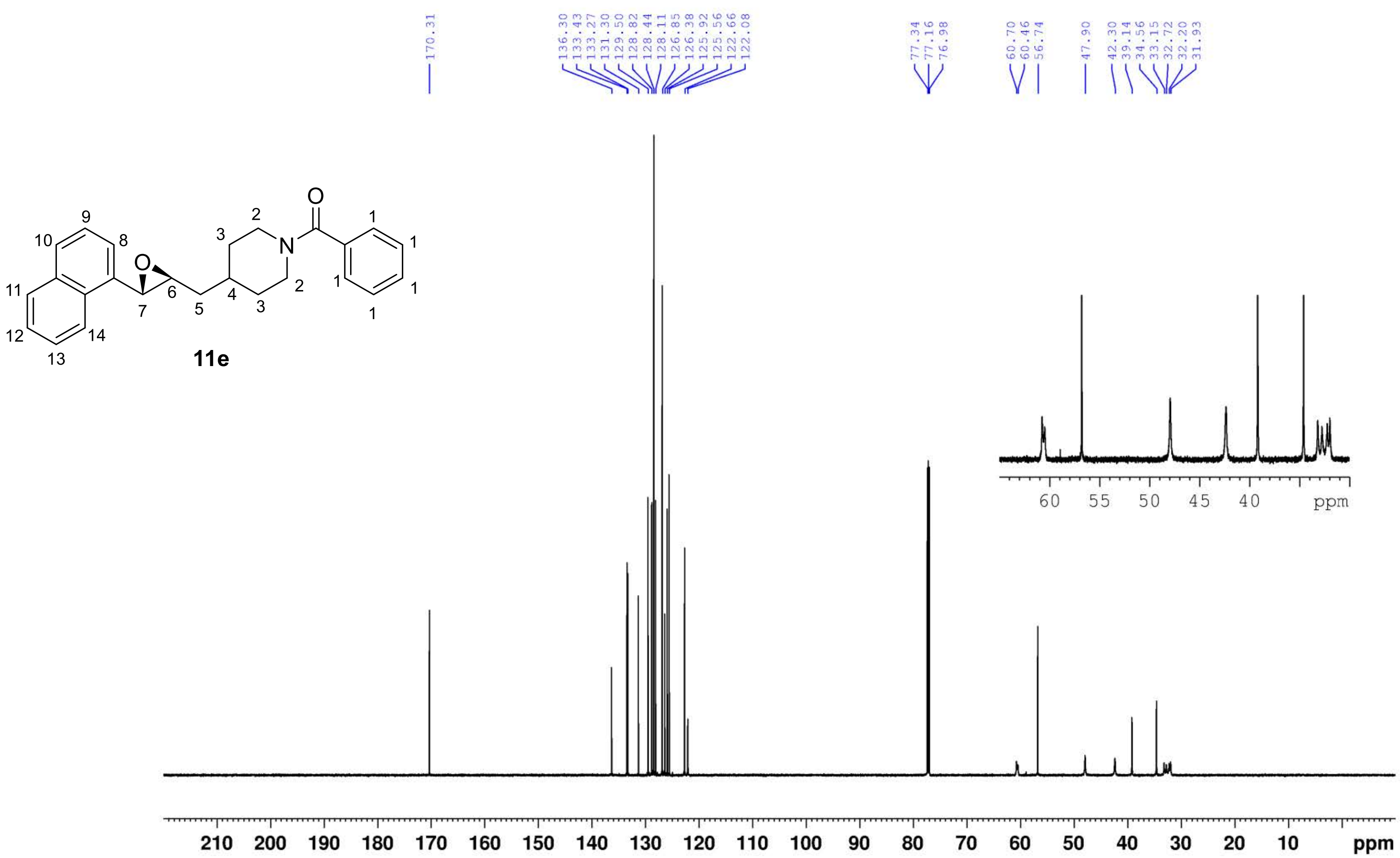

¹H NMR (400 MHz, $CDCl_3$) for *(S)-naphthalen-1-yl((1S,2R,4S)-quinuclidin-2-yl)methanol* (**11f**)

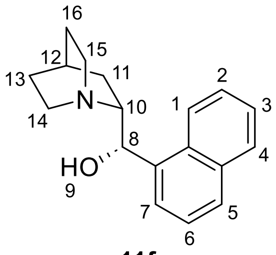

11f

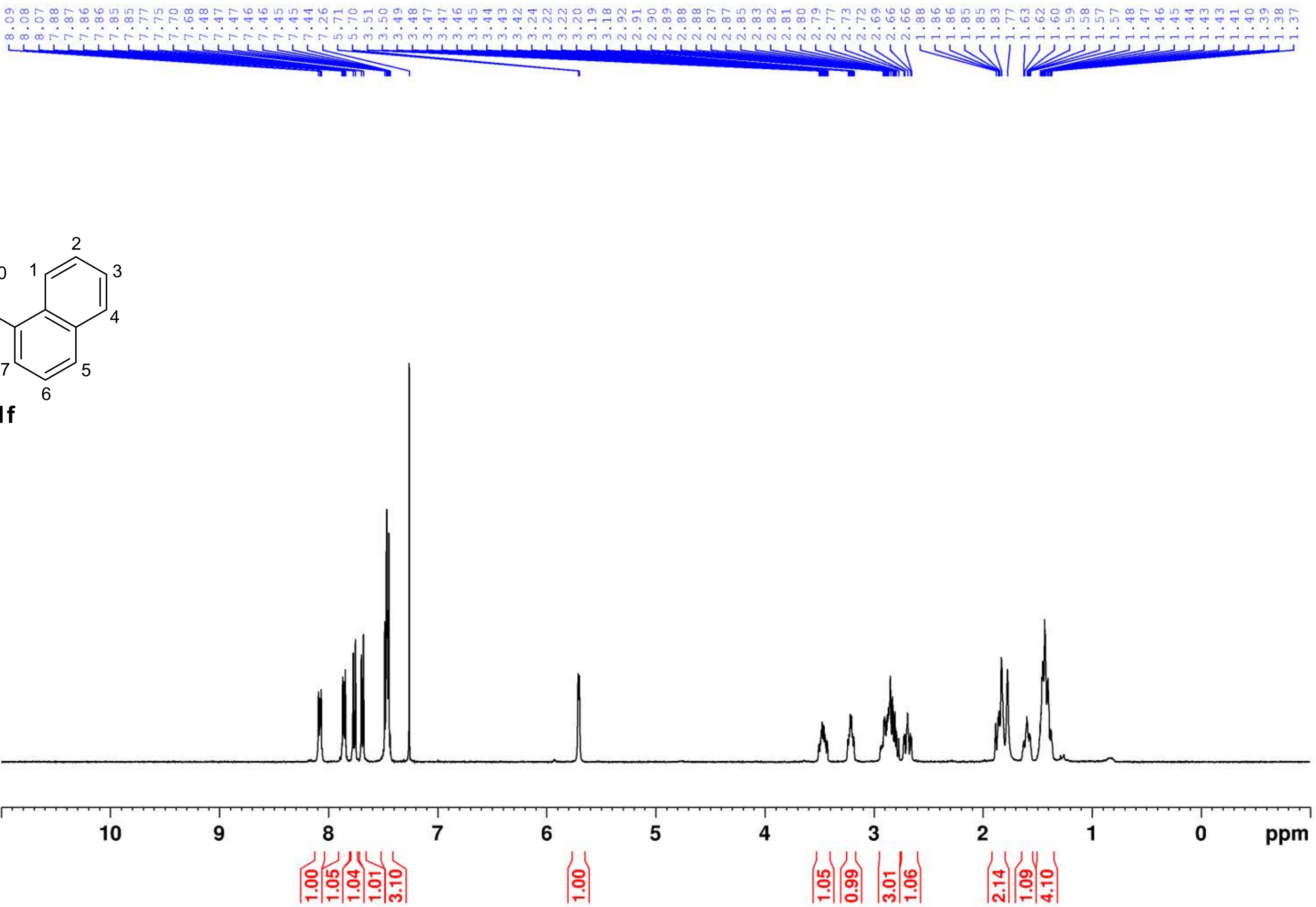

^{13}**C NMR** (101 MHz, $CDCl_3$) for *(S)-naphthalen-1-yl((1S,2R,4S)-quinuclidin-2-yl)methanol* (**11f**)

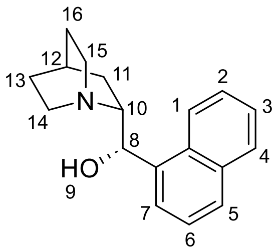

11f

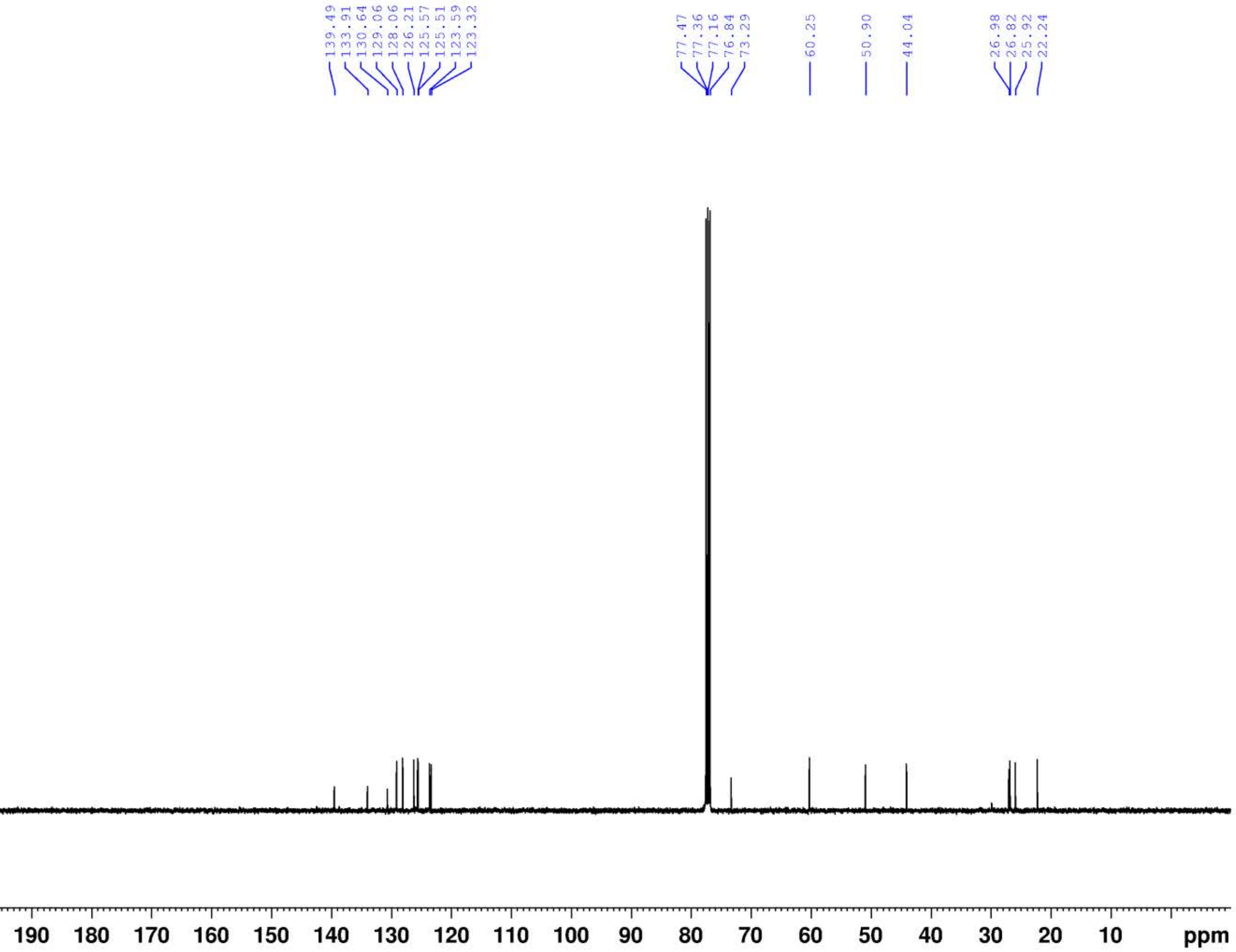

^{1}H NMR (700 MHz, $CDCl_3$) for *(1S,2R,4S)-2-((S)-hydroxy(naphthalen-1-yl)methyl)-1-((3,3'',5,5''-tetra-tert-butyl-[1,1':3',1''-terphenyl]-5'-yl)methyl)quinuclidin-1-ium bromide* (**Cat6•Br**)

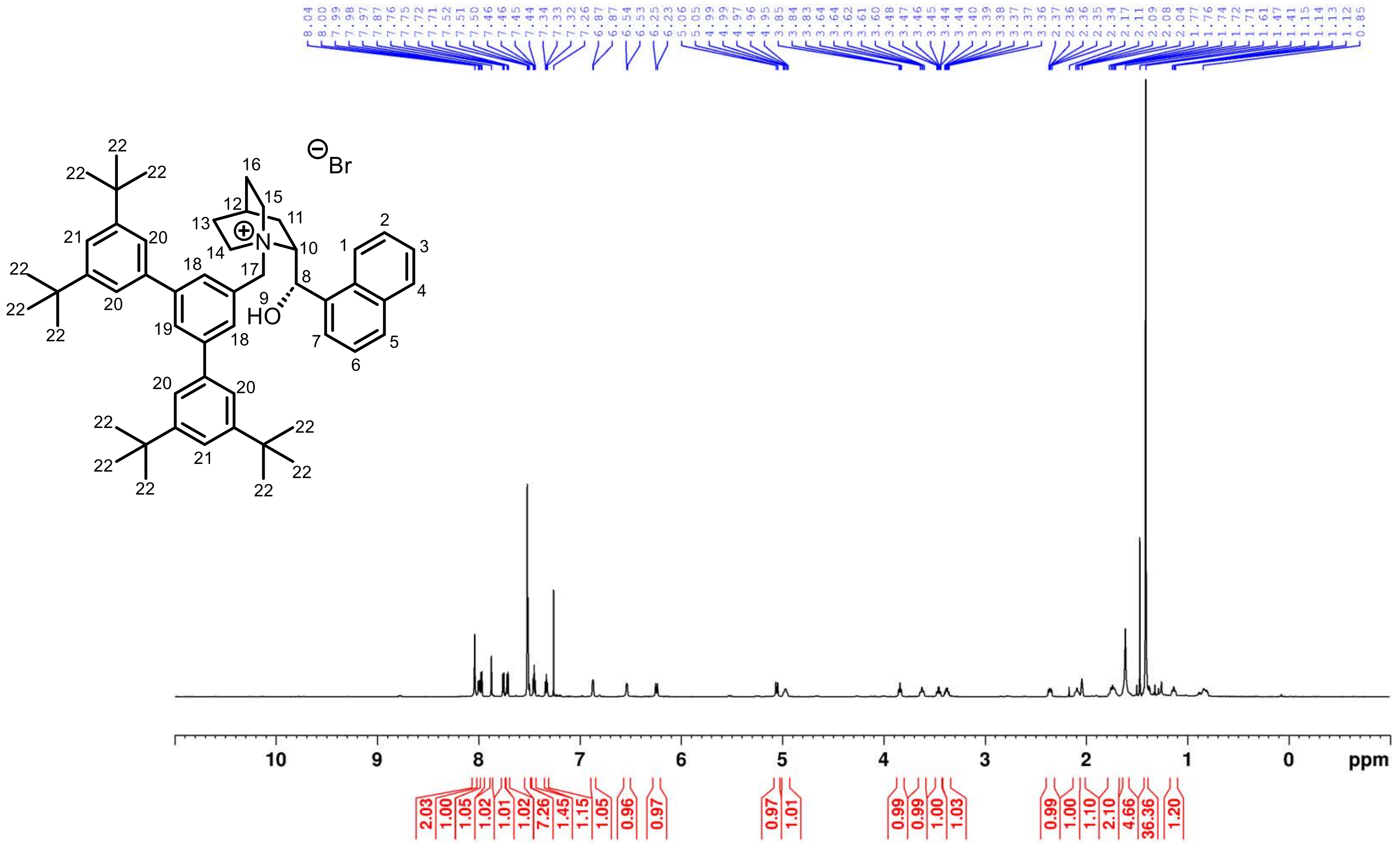

^{13}C NMR (176 MHz, $CDCl_3$) for *(1S,2R,4S)-2-((S)-hydroxy(naphthalen-1-yl)methyl)-1-((3,3'',5,5''-tetra-tert-butyl-[1,1':3',1''-terphenyl]-5'-yl)methyl)quinuclidin-1-ium bromide* (**Cat6•Br**)

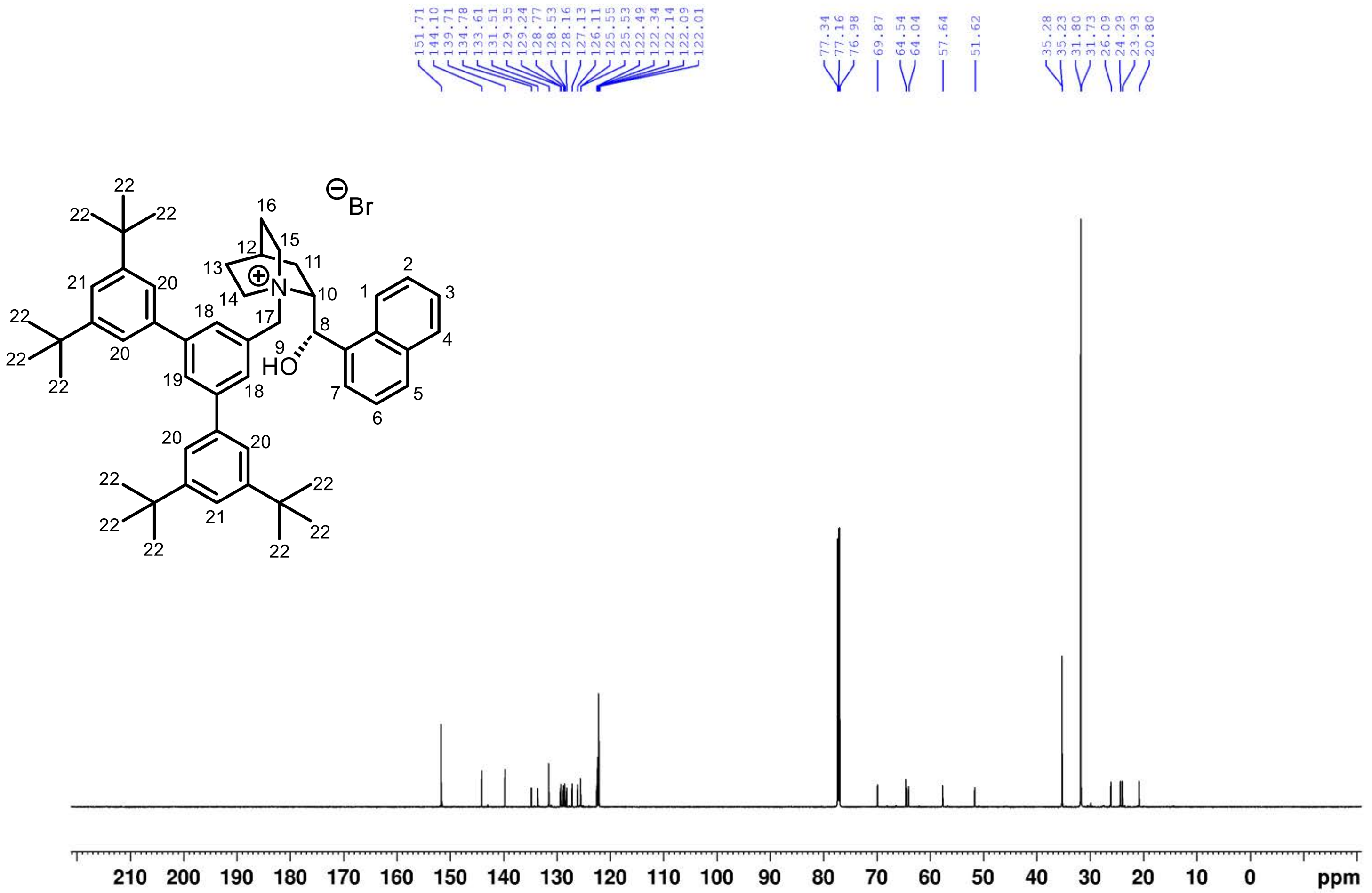

^{1}H NMR (400 MHz, $CDCl_3$) for *(E)-(4-(3-(7-methoxynaphthalen-1-yl)allyl)piperidin-1-yl)(phenyl)methanone*

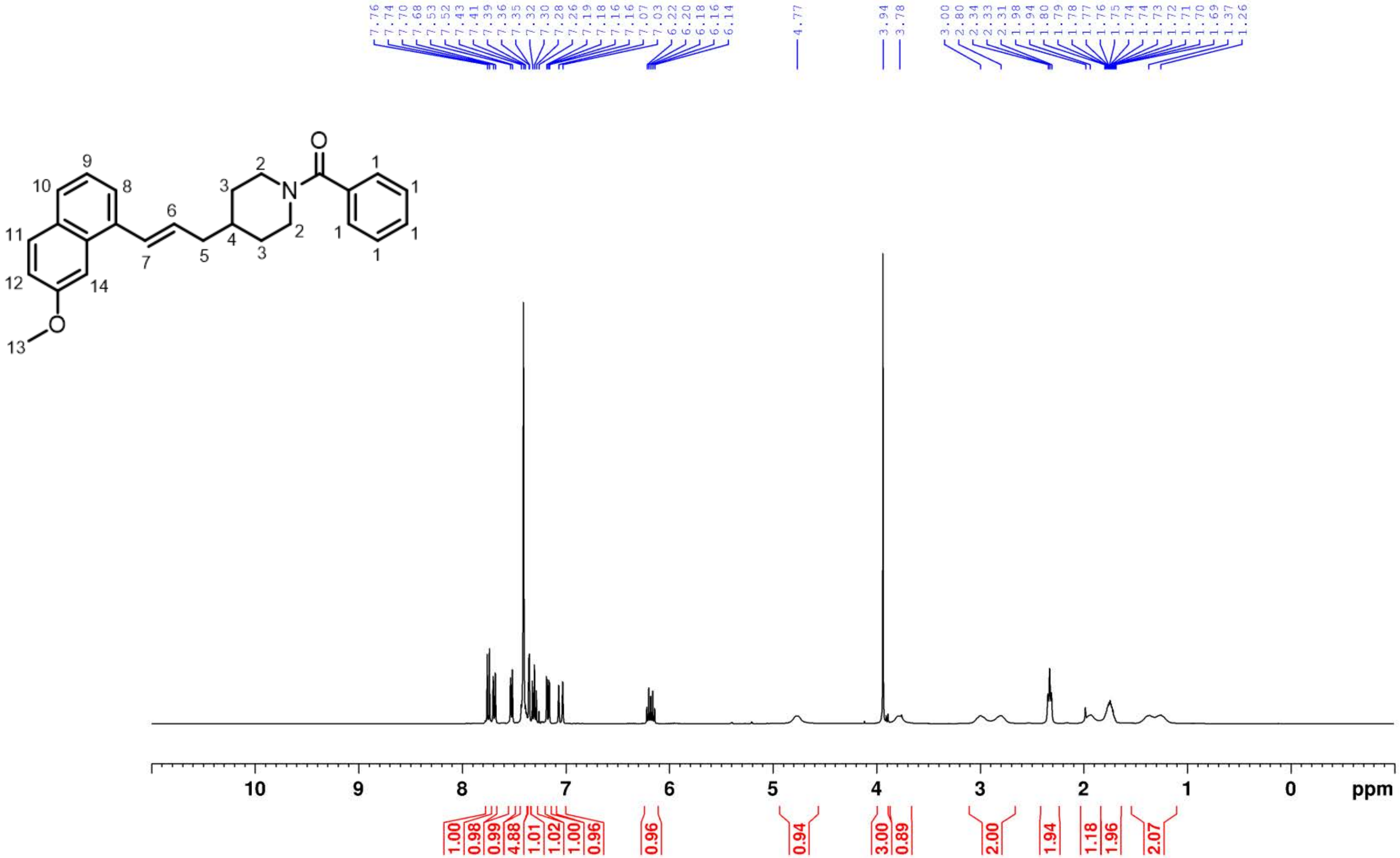

^{13}C NMR (101 MHz, $CDCl_3$) for *(E)-(4-(3-(7-methoxynaphthalen-1-yl)allyl)piperidin-1-yl)(phenyl)methanone*

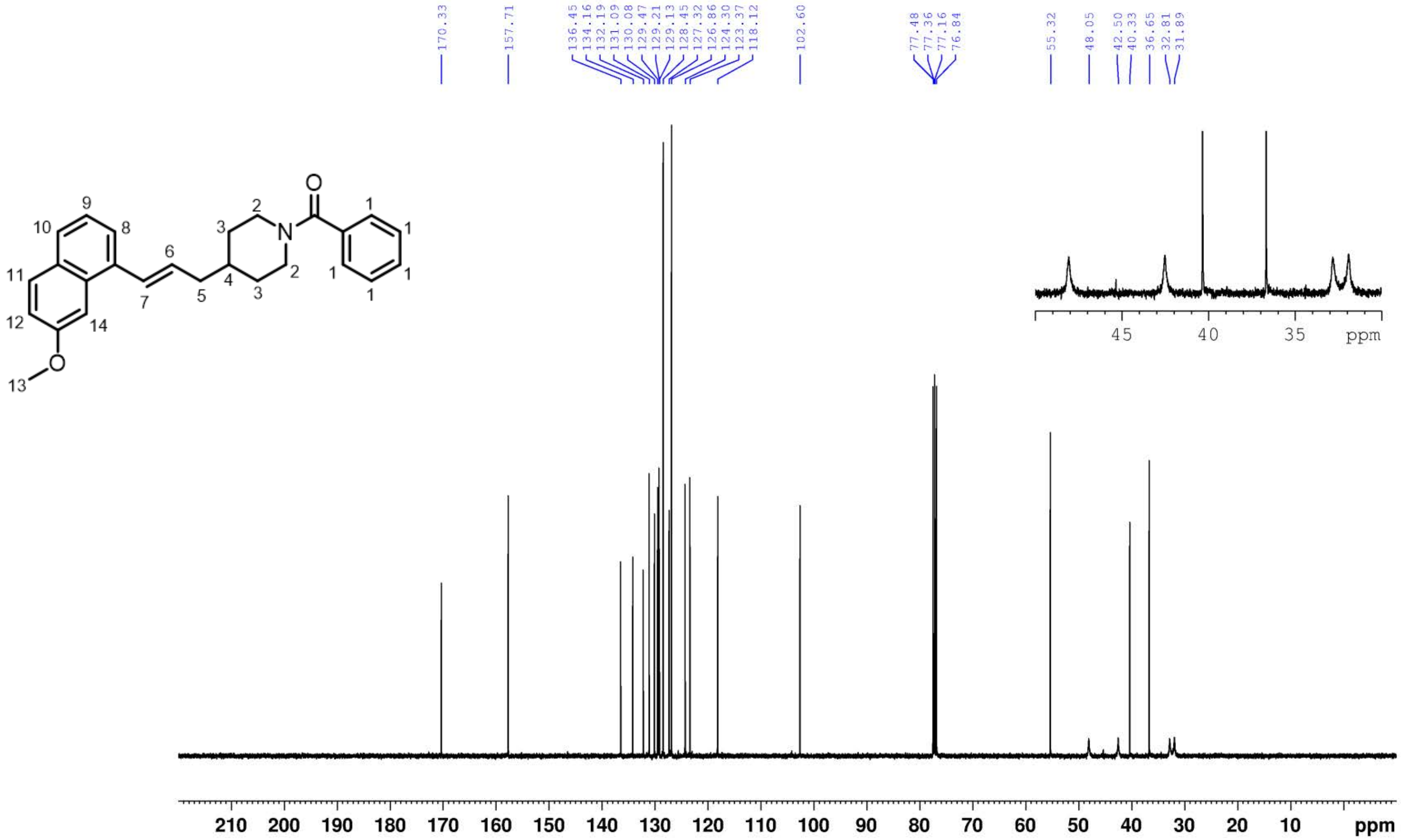

^{1}H NMR (700 MHz, $CDCl_3$) for *(4-((2S,3S)-2,3-dihydroxy-3-(7-methoxynaphthalen-1-yl)propyl)piperidin-1-yl)(phenyl)methanone*

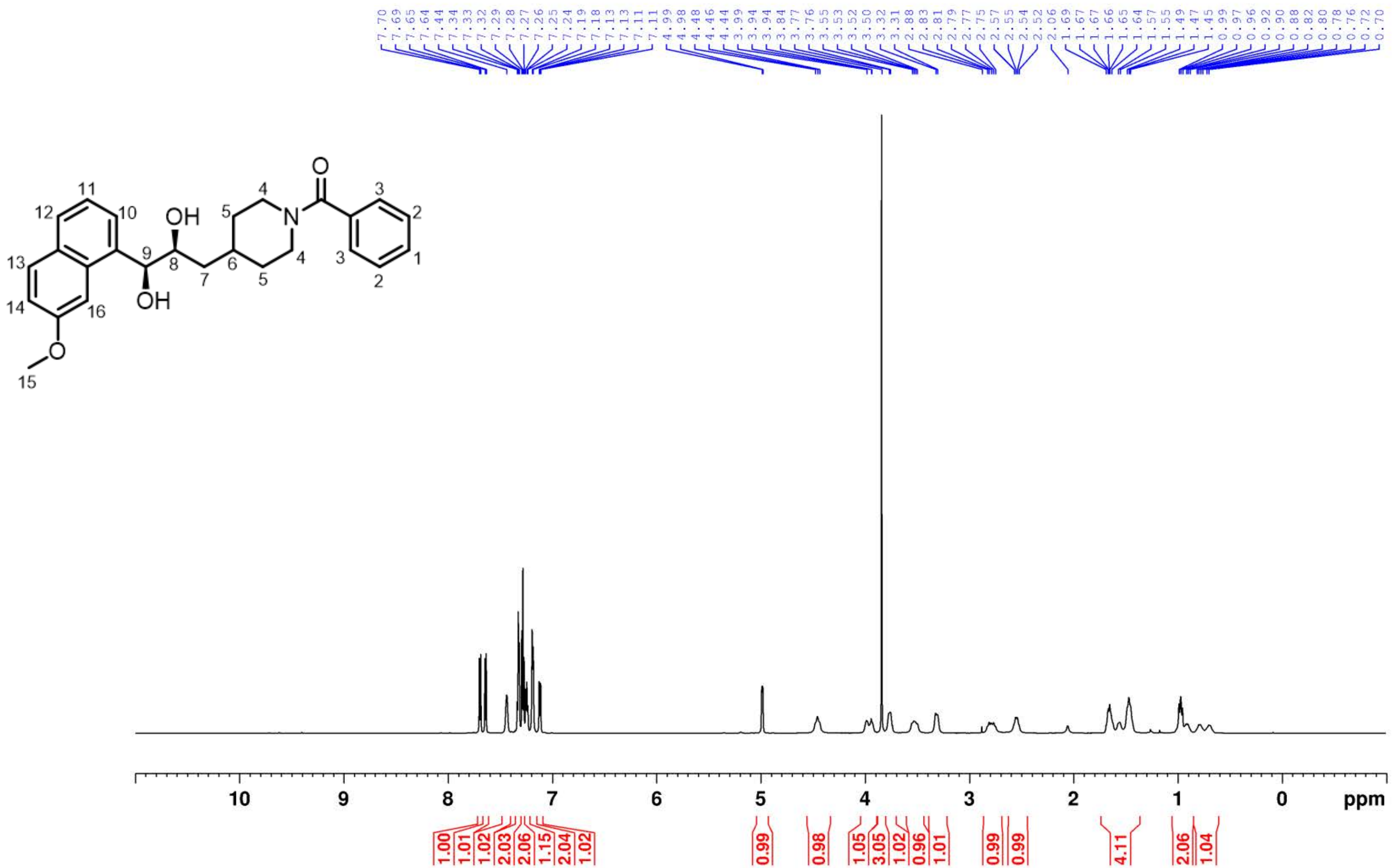

^{13}C NMR (176 MHz, CDCl₃) for *(4-((2S,3S)-2,3-dihydroxy-3-(7-methoxynaphthalen-1-yl)propyl)piperidin-1-yl)(phenyl)methanone*

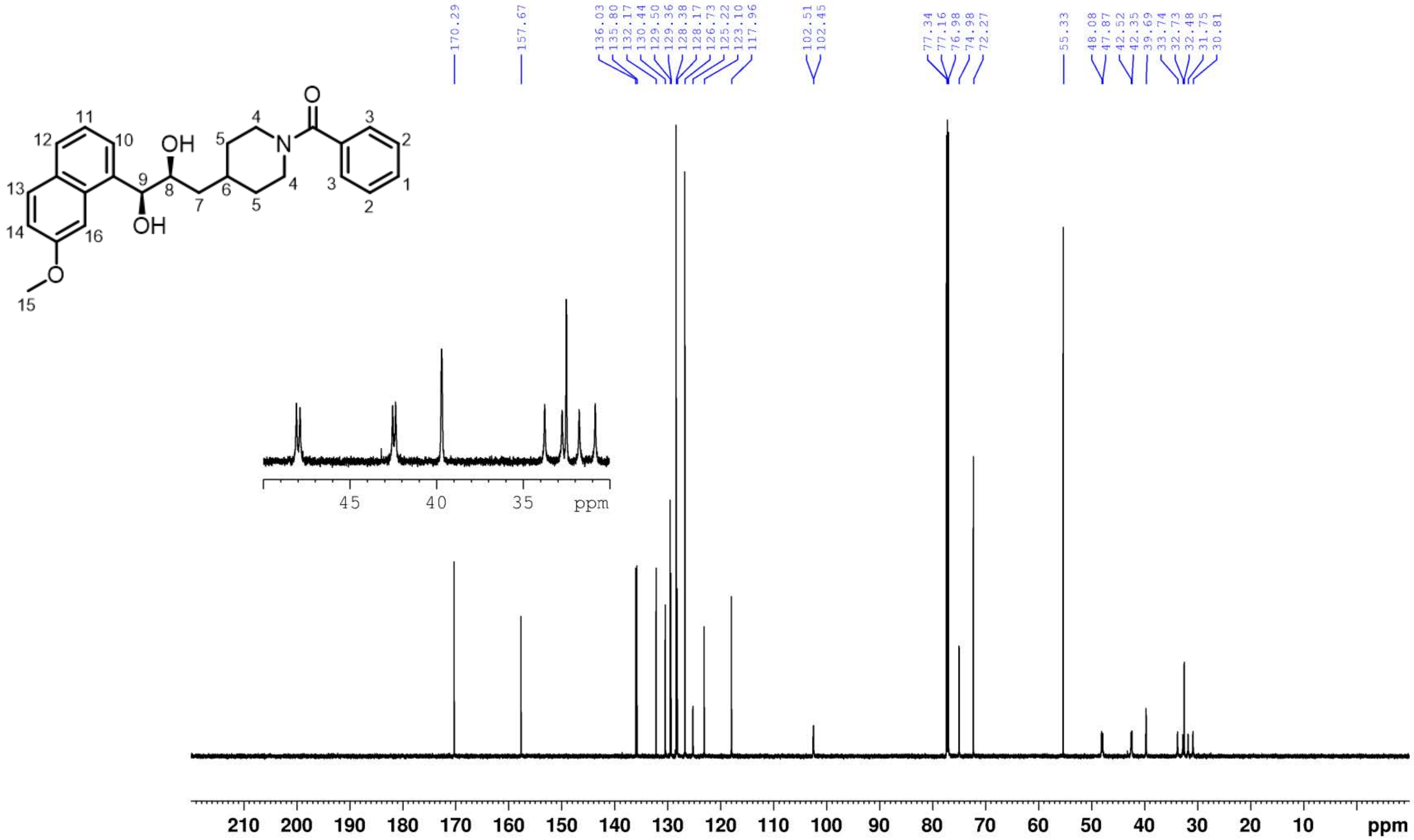

^{1}H NMR (700 MHz, $CDCl_3$) for *(4-(((2S,3S)-3-(7-methoxynaphthalen-1-yl)oxiran-2-yl)methyl)piperidin-1-yl)(phenyl)methanone*

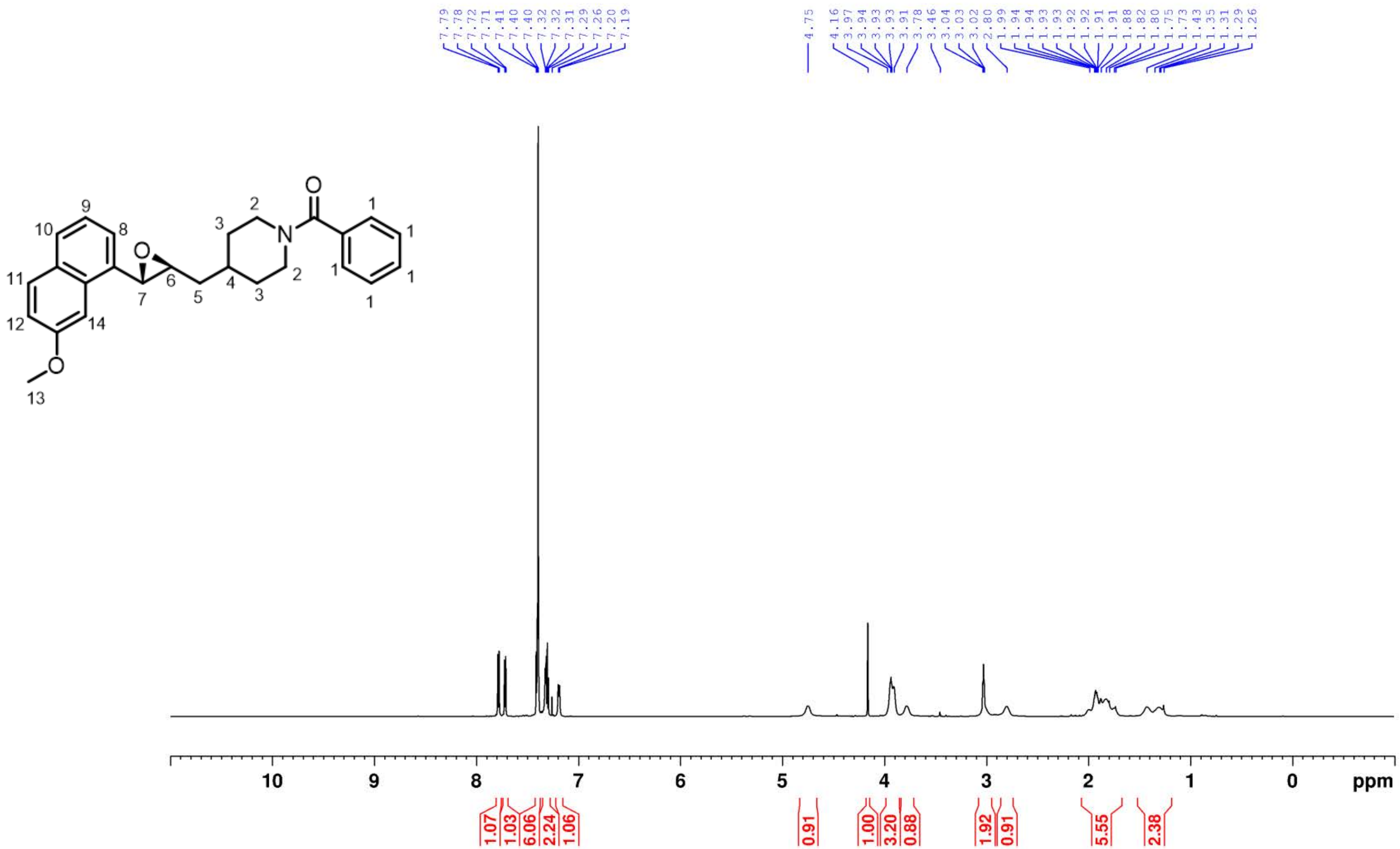

^{13}C NMR (176 MHz, $CDCl_3$) for *(4-(((2S,3S)-3-(7-methoxynaphthalen-1-yl)oxiran-2-yl)methyl)piperidin-1-yl)(phenyl)methanone*

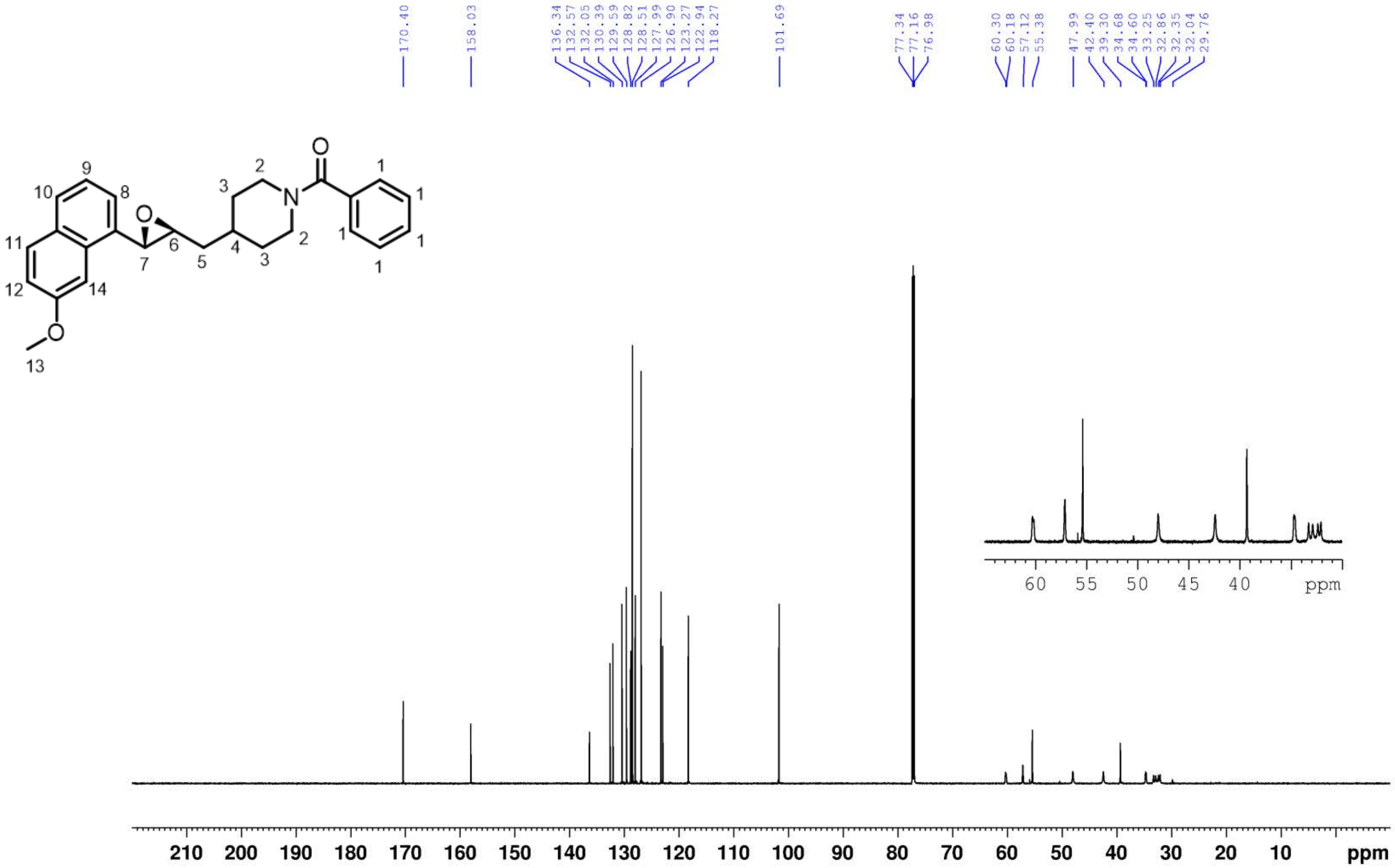

^{1}H NMR (400 MHz, $CDCl_3$) for *(S)-(7-methoxynaphthalen-1-yl)((1S,2R,4S)-quinuclidin-2-yl)methanol*

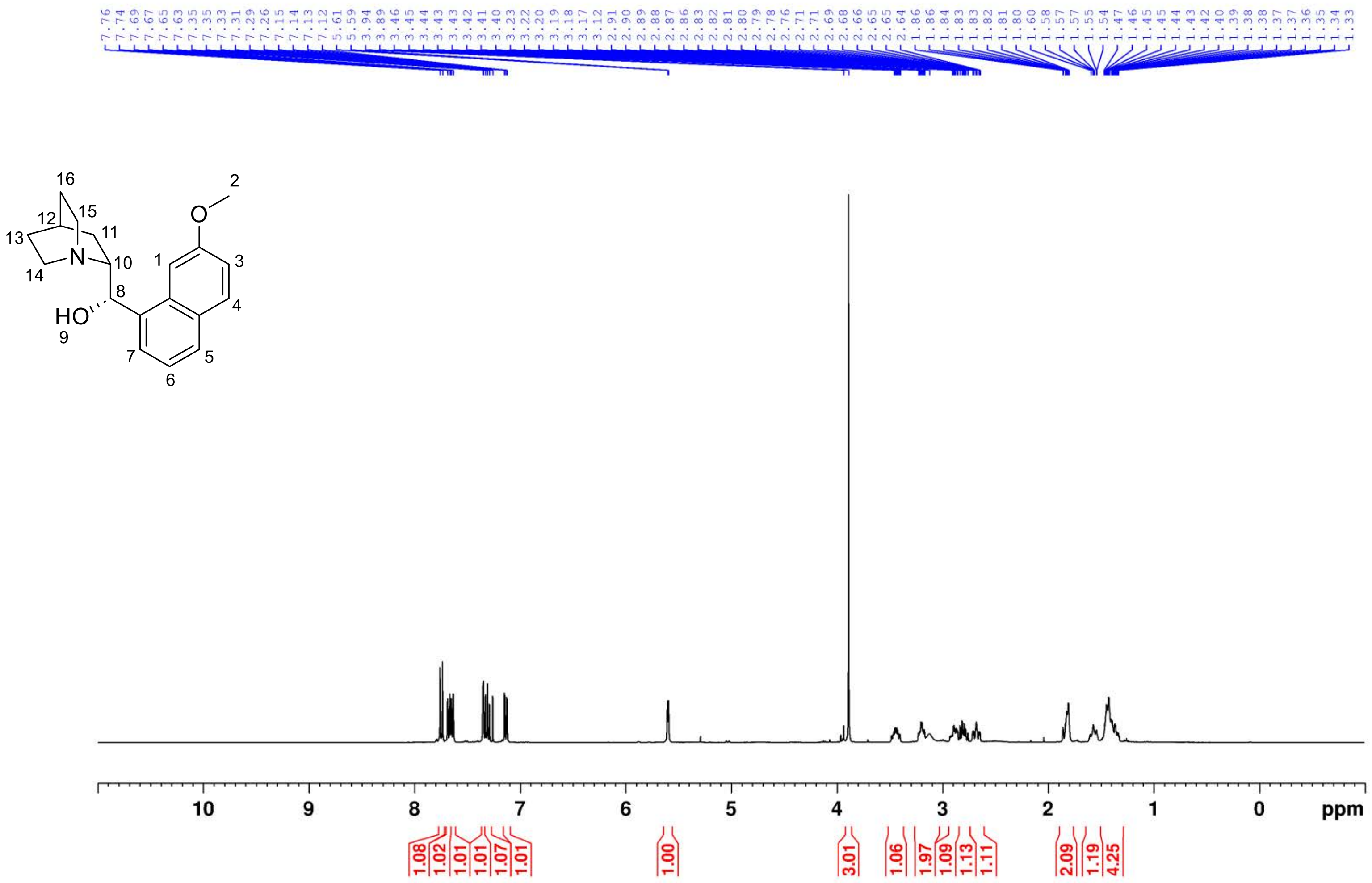

^{13}C NMR (101 MHz, $CDCl_3$) for *(S)-(7-methoxynaphthalen-1-yl)((1S,2R,4S)-quinuclidin-2-yl)methanol*

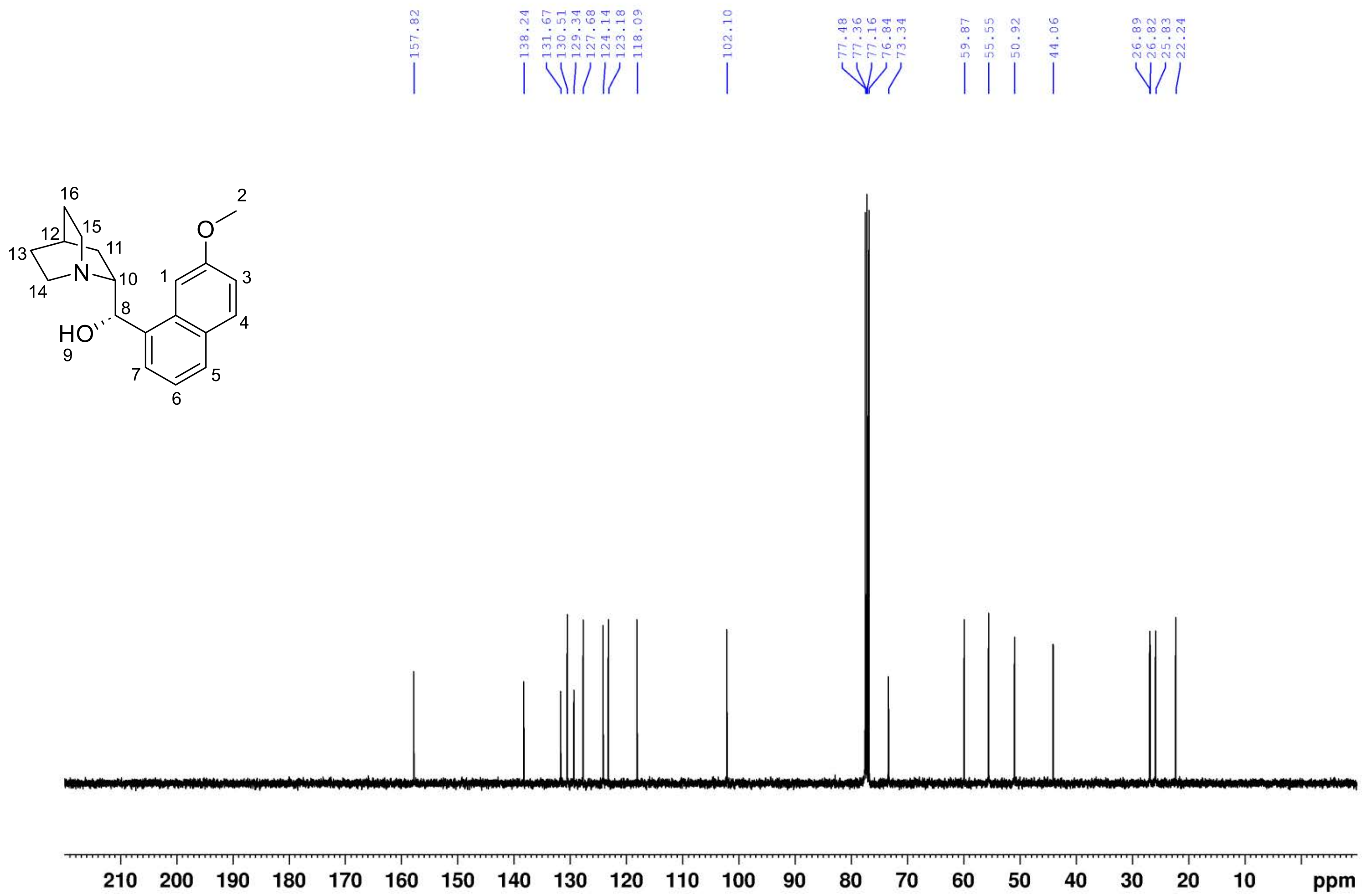

¹H NMR (400 MHz, $CDCl_3$) for *(1S,2R,4S)-2-((S)-hydroxy(7-methoxynaphthalen-1-yl)methyl)-1-((3,3'',5,5''-tetra-tert-butyl-[1,1':3',1''-terphenyl]-5'-yl)methyl)quinuclidin-1-ium bromide* (**Cat7•Br**)

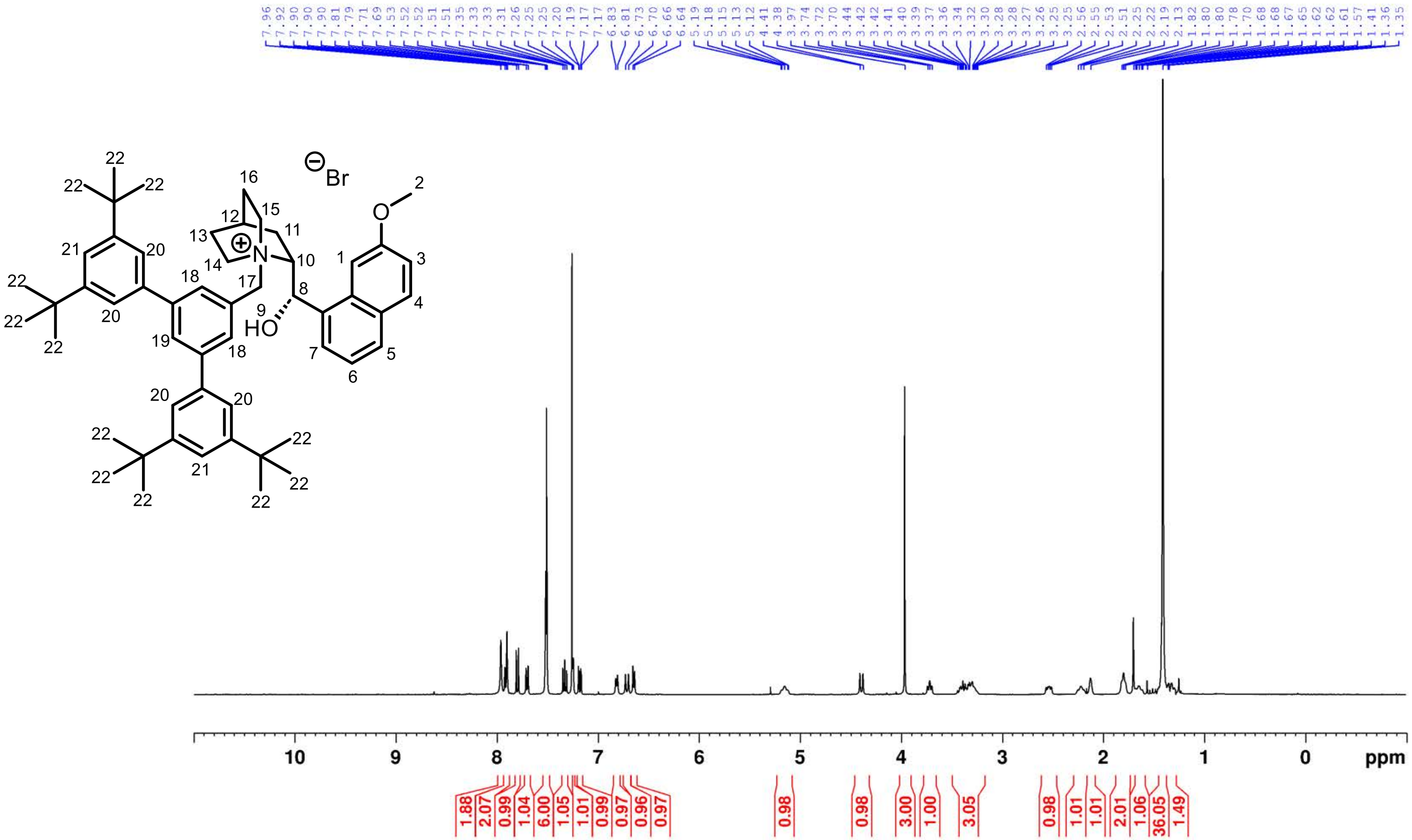

^{13}C NMR (101 MHz, $CDCl_3$) for *(1S,2R,4S)-2-((S)-hydroxy(7-methoxynaphthalen-1-yl)methyl)-1-((3,3'',5,5''-tetra-tert-butyl-[1,1':3',1''-terphenyl]-5'-yl)methyl)quinuclidin-1-ium bromide* (**Cat7•Br**)

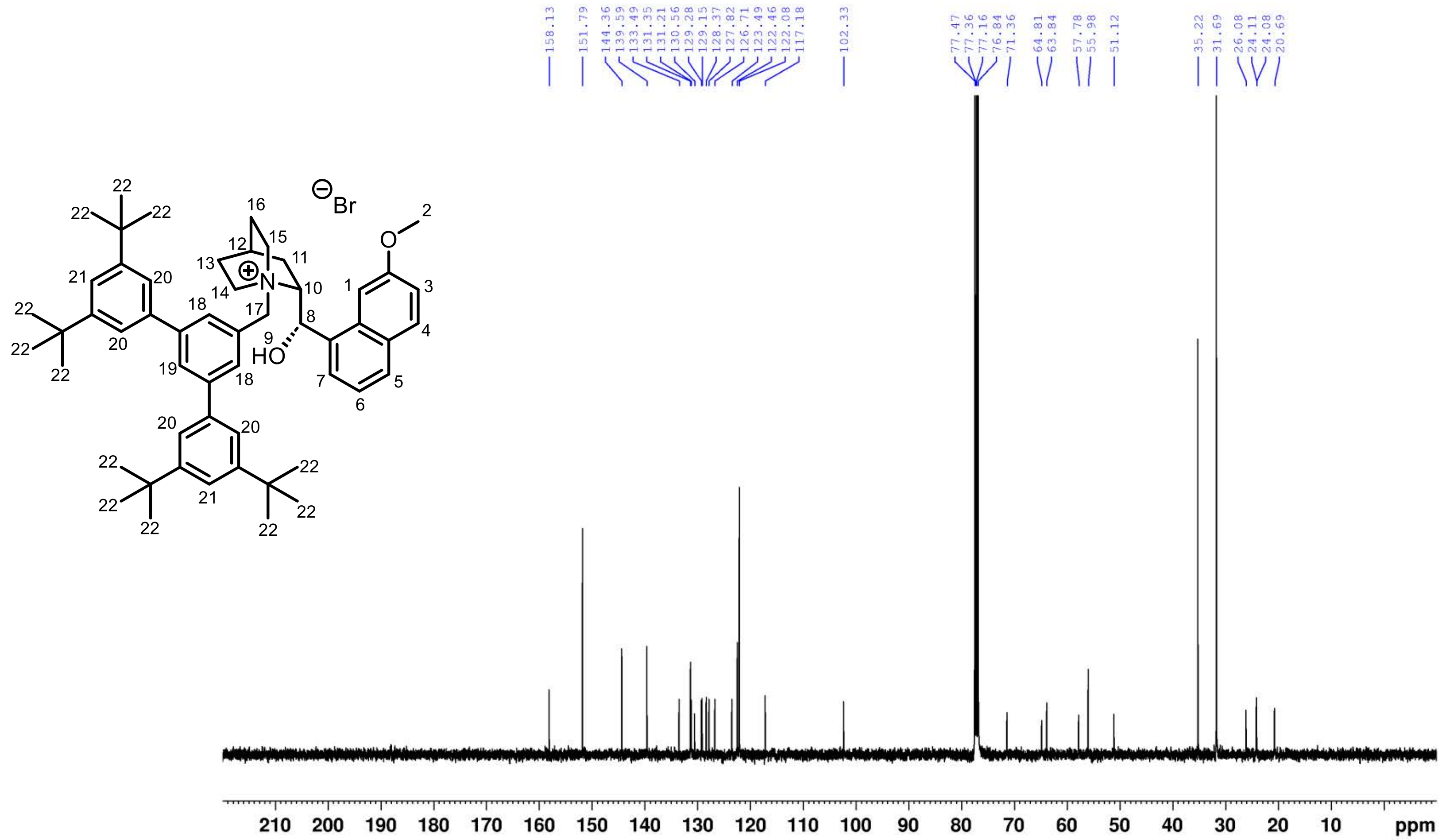

^{1}H NMR (400 MHz, $CDCl_3$) for *(E)-phenyl(4-(3-(quinolin-5-yl)allyl)piperidin-1-yl)methanone*

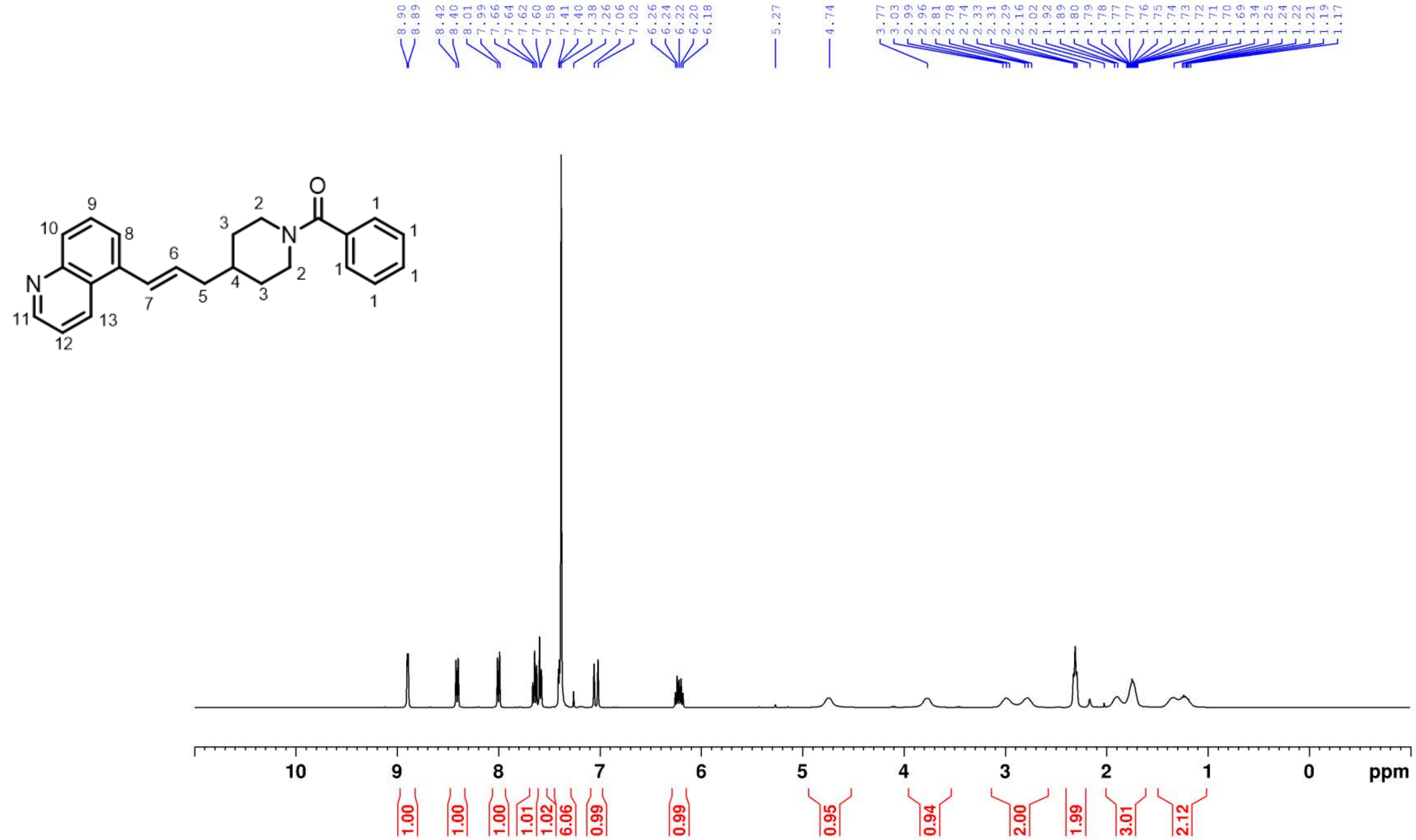

^{13}C NMR (101 MHz, $CDCl_3$) for *(E)-phenyl(4-(3-(quinolin-5-yl)allyl)piperidin-1-yl)methanone*

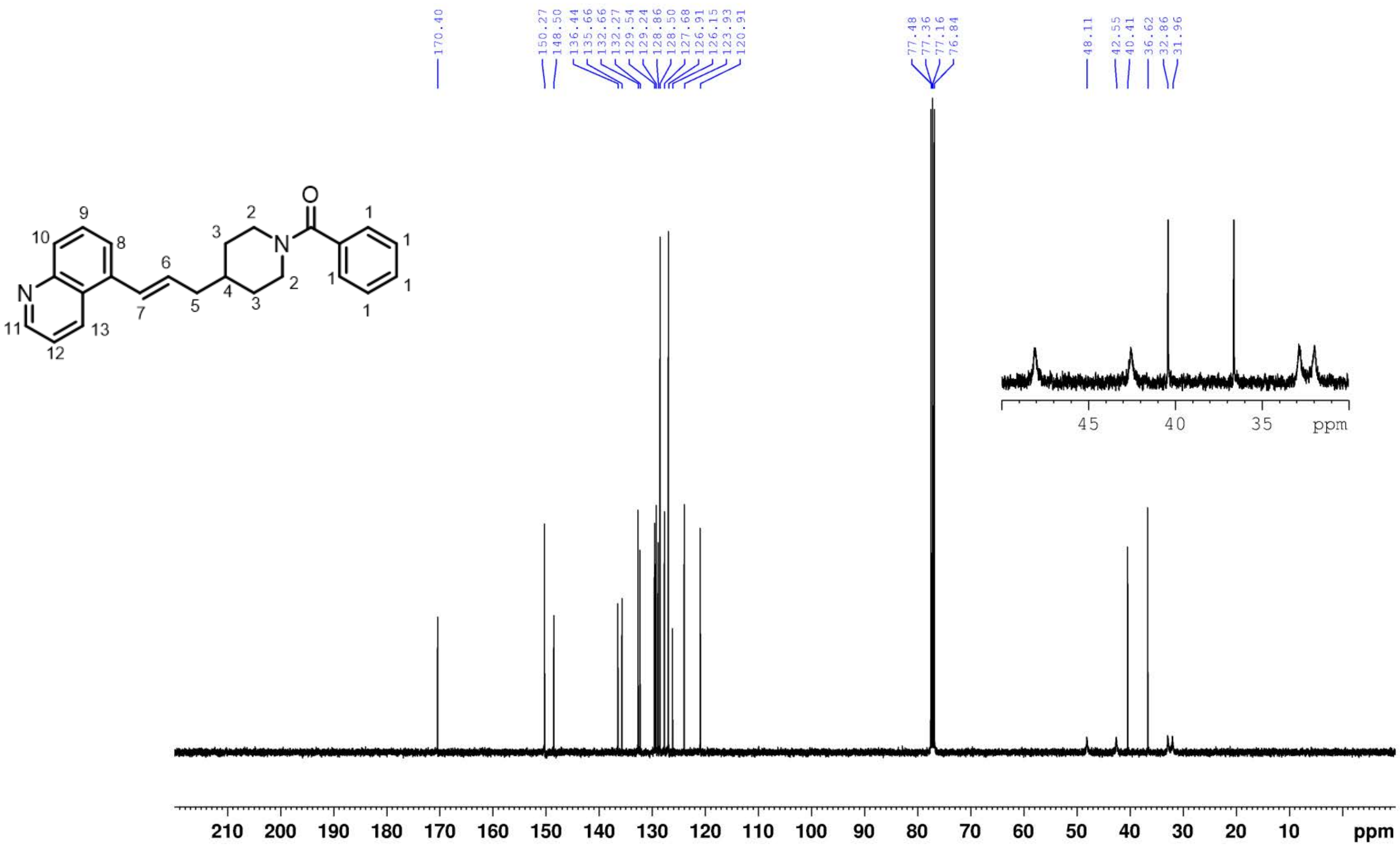

¹H NMR (700 MHz, $CDCl_3$) for *(4-((2S,3S)-2,3-dihydroxy-3-(quinolin-5-yl)propyl)piperidin-1-yl)(phenyl)methanone*

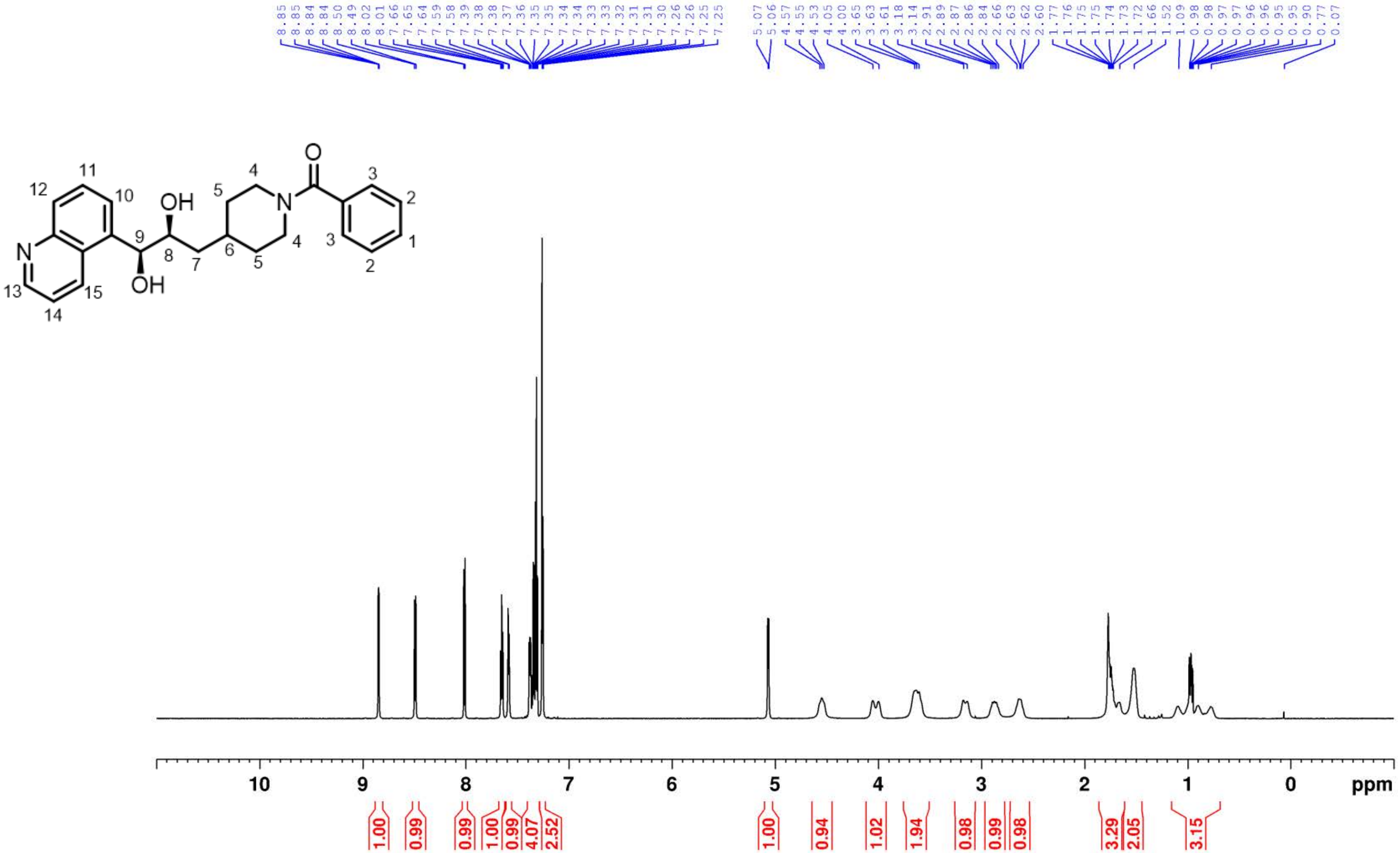

^{13}C NMR (176 MHz, $CDCl_3$) for *(4-((2S,3S)-2,3-dihydroxy-3-(quinolin-5-yl)propyl)piperidin-1-yl)(phenyl)methanone*

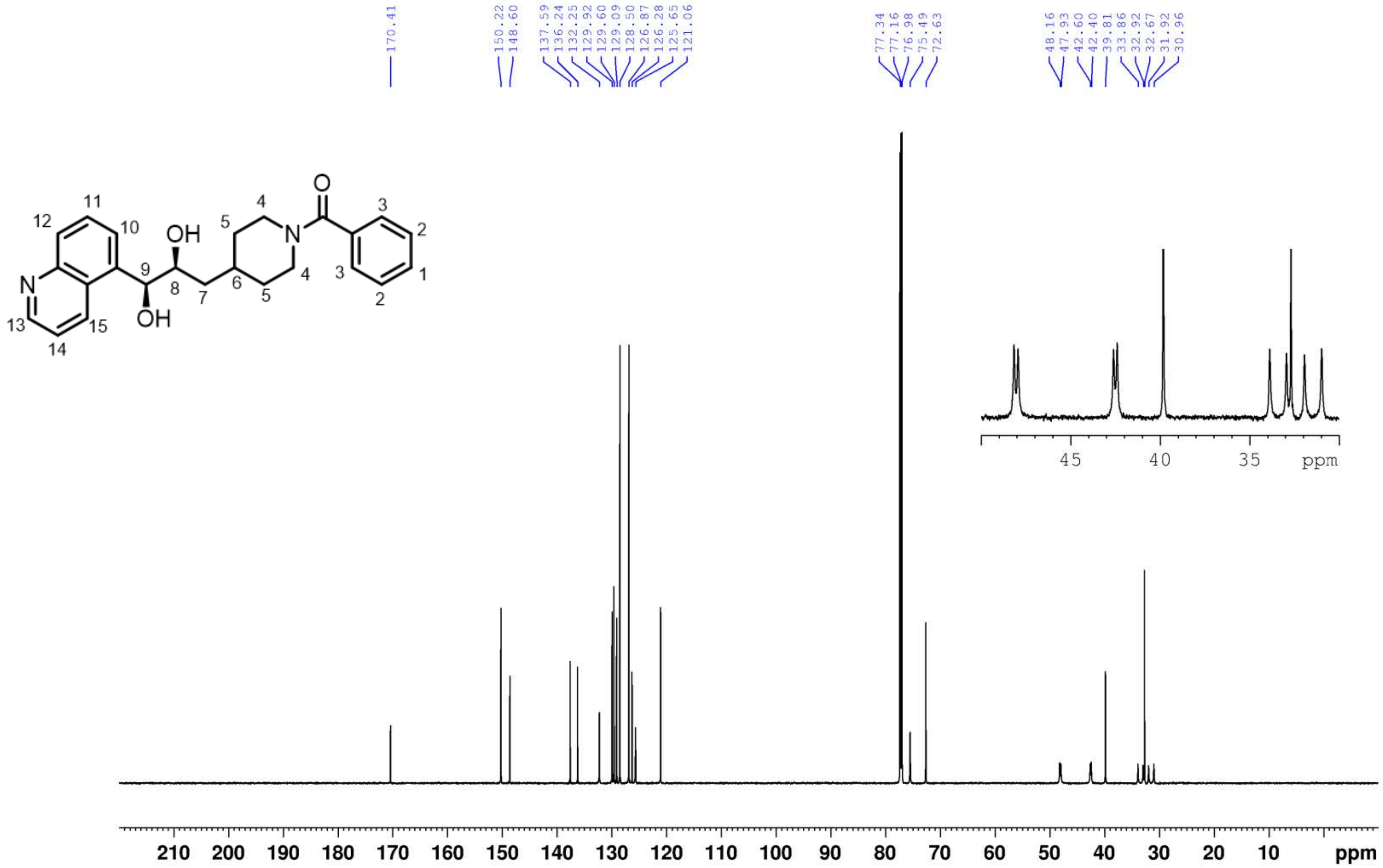

^{1}H NMR (700 MHz, $CDCl_3$) for *phenyl(4-(((2S,3S)-3-(quinolin-5-yl)oxiran-2-yl)methyl)piperidin-1-yl)methanone*

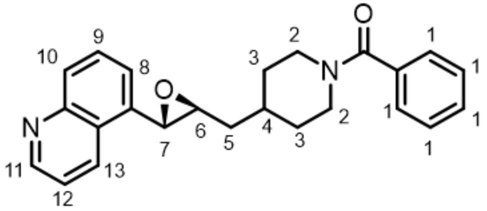

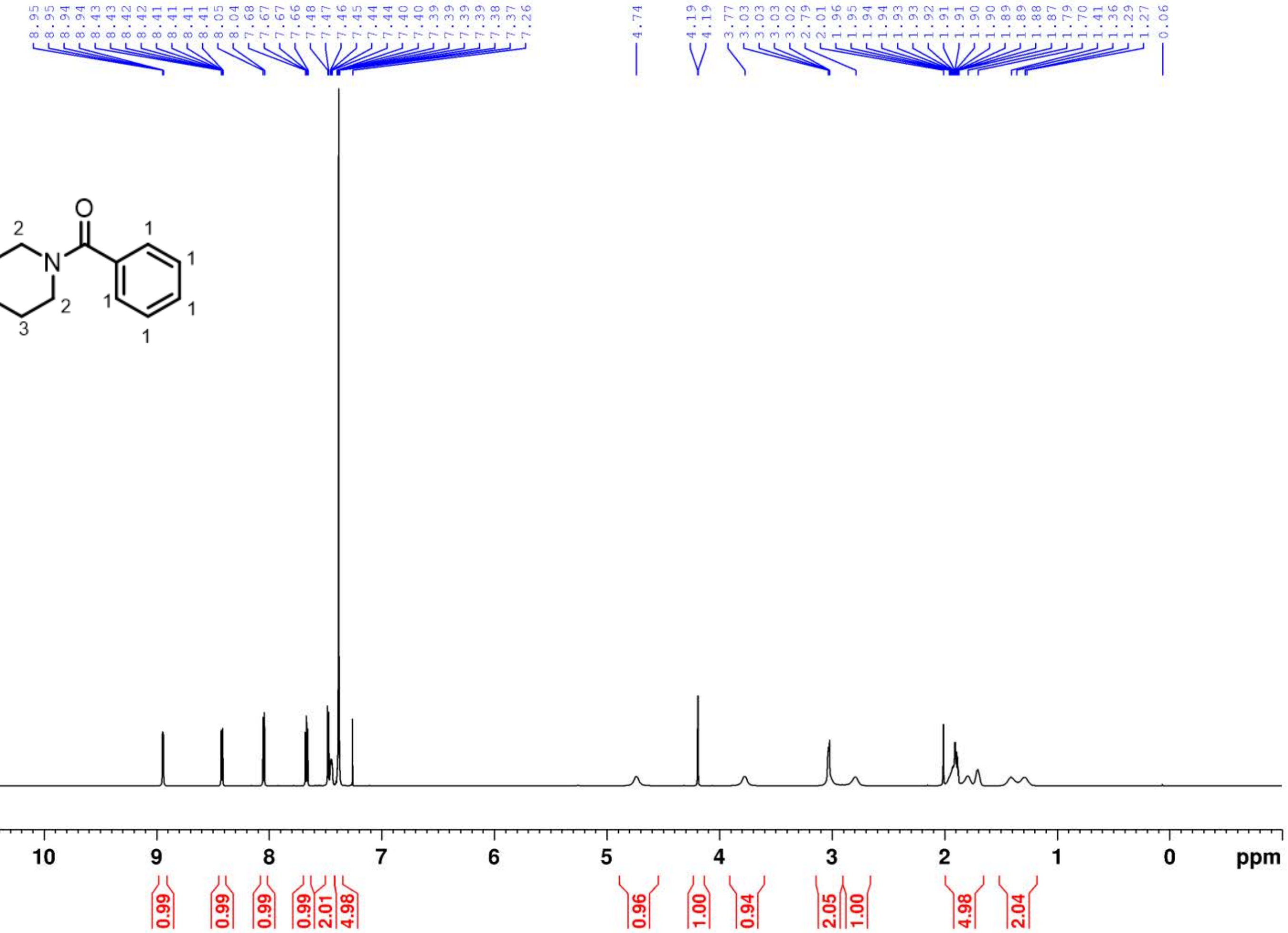

^{13}C NMR (176 MHz, $CDCl_3$) for *phenyl(4-(((2S,3S)-3-(quinolin-5-yl)oxiran-2-yl)methyl)piperidin-1-yl)methanone*

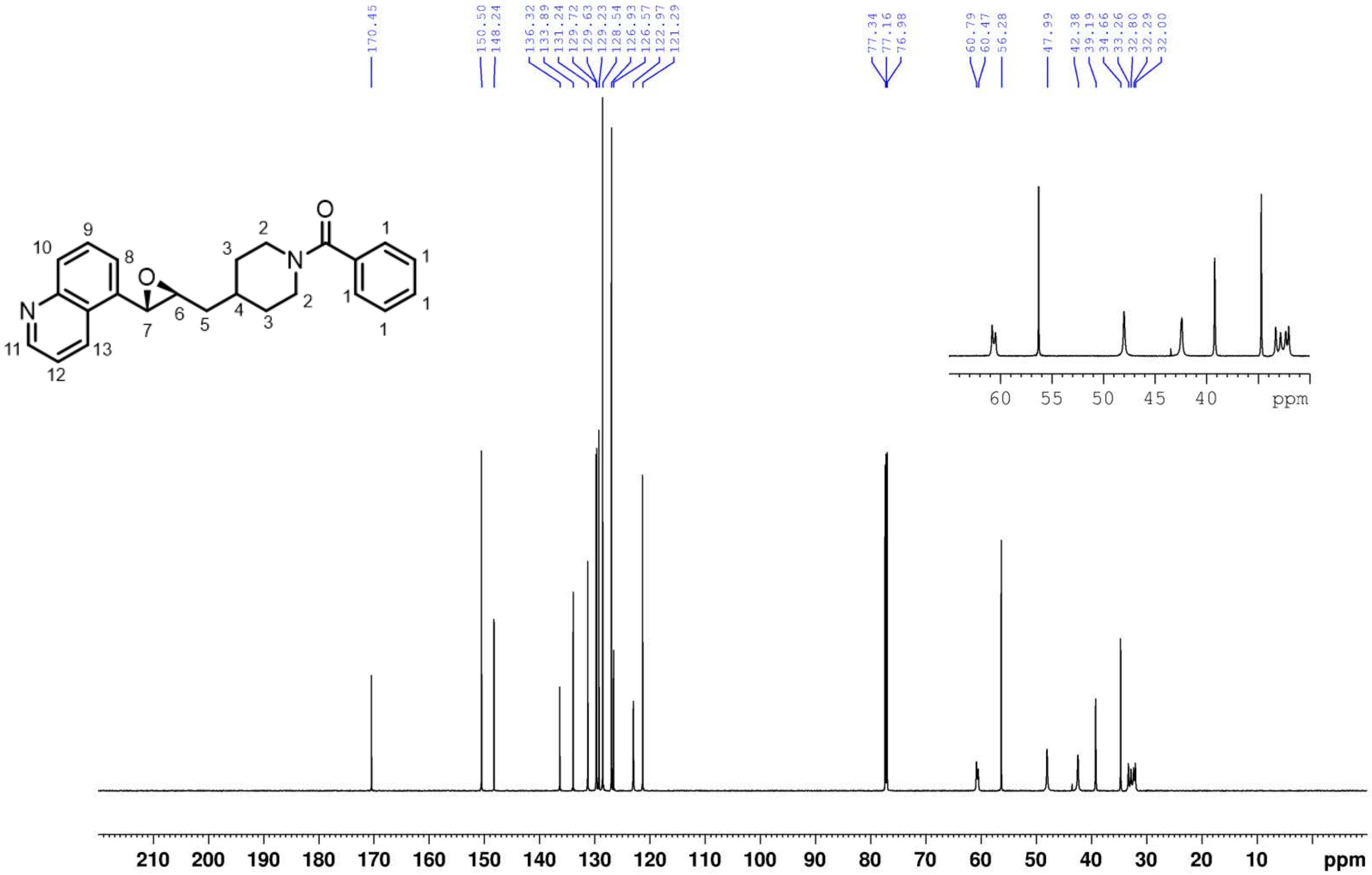

^{1}H NMR (700 MHz, $CDCl_3$) for *(S)-quinolin-5-yl((1S,2R,4S)-quinuclidin-2-yl)methanol*

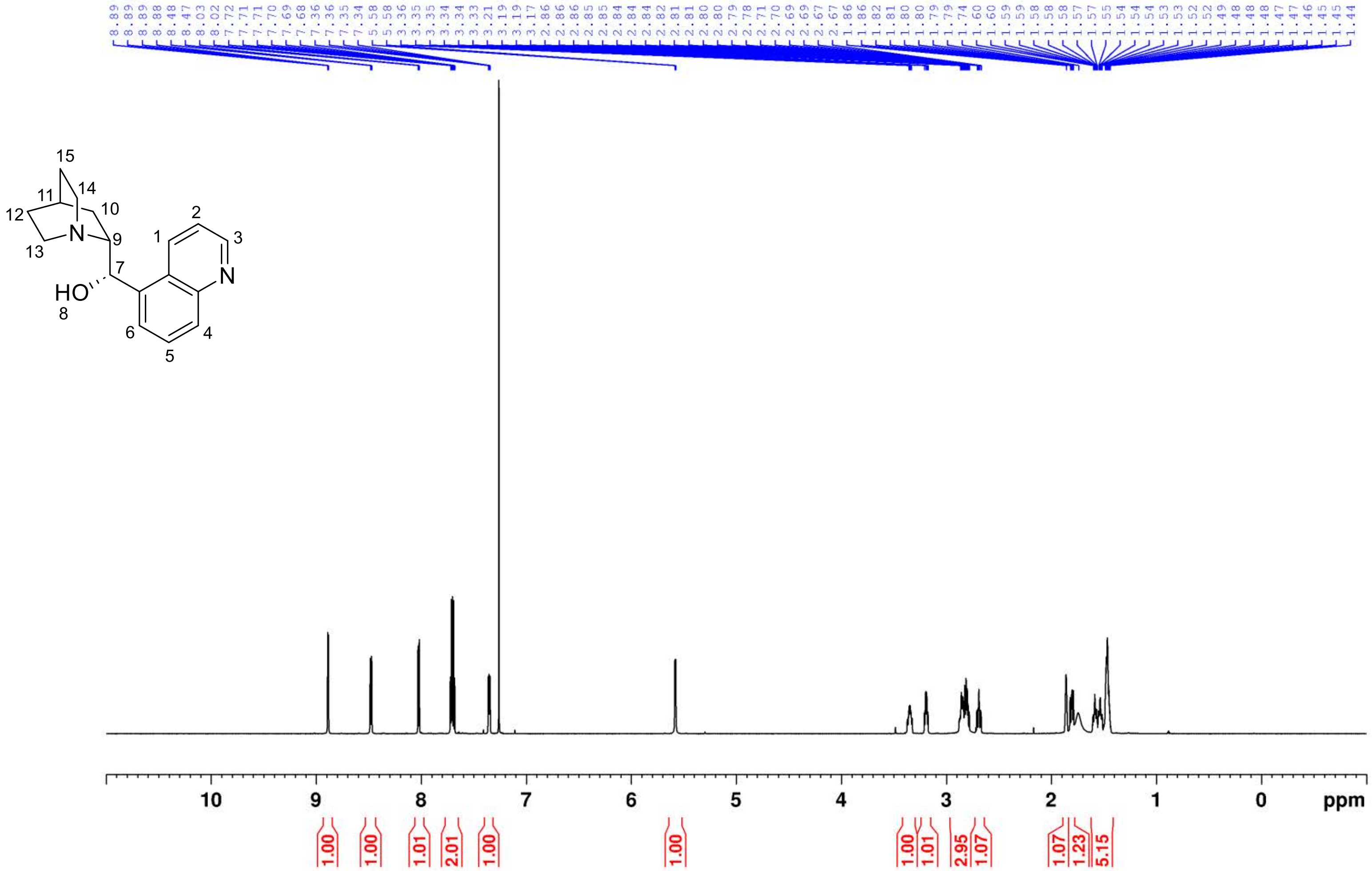

^{13}C NMR (176 MHz, $CDCl_3$) for *(S)-quinolin-5-yl((1S,2R,4S)-quinuclidin-2-yl)methanol*

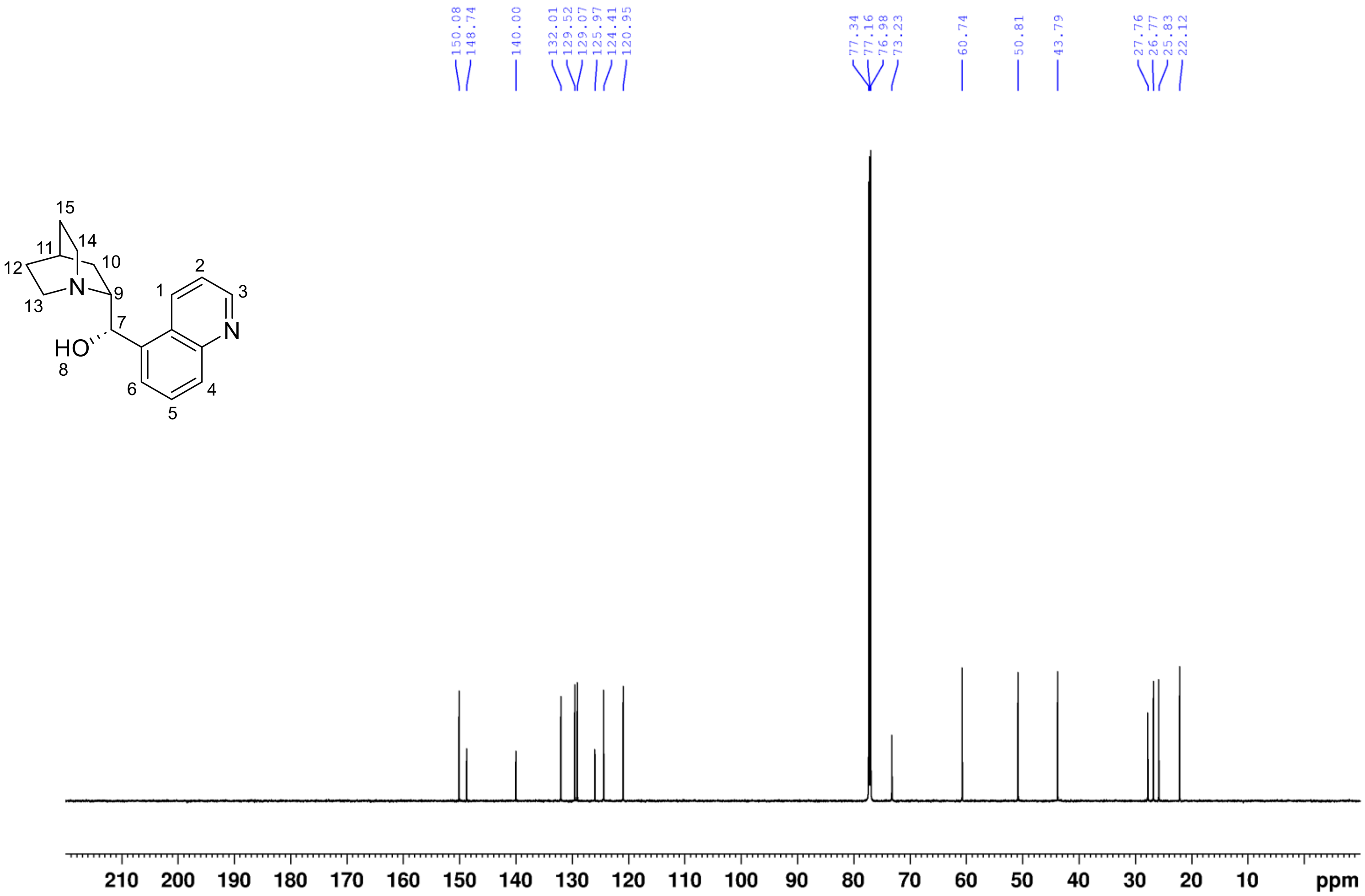

^{1}H NMR (700 MHz, $CDCl_3$) for *(1S,2R,4S)-2-((S)-hydroxy(quinolin-5-yl)methyl)-1-((3,3'',5,5''-tetra-tert-butyl-[1,1':3',1''-terphenyl]-5'-yl)methyl)quinuclidin-1-ium bromide* (**Cat8•Br**)

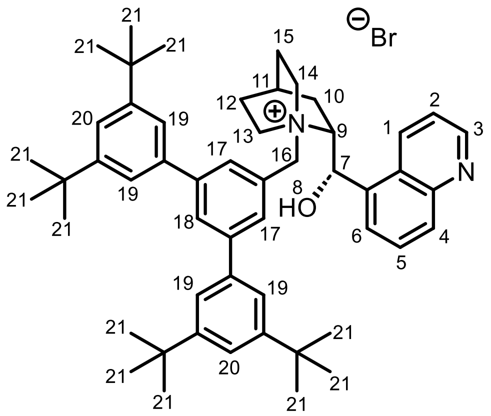

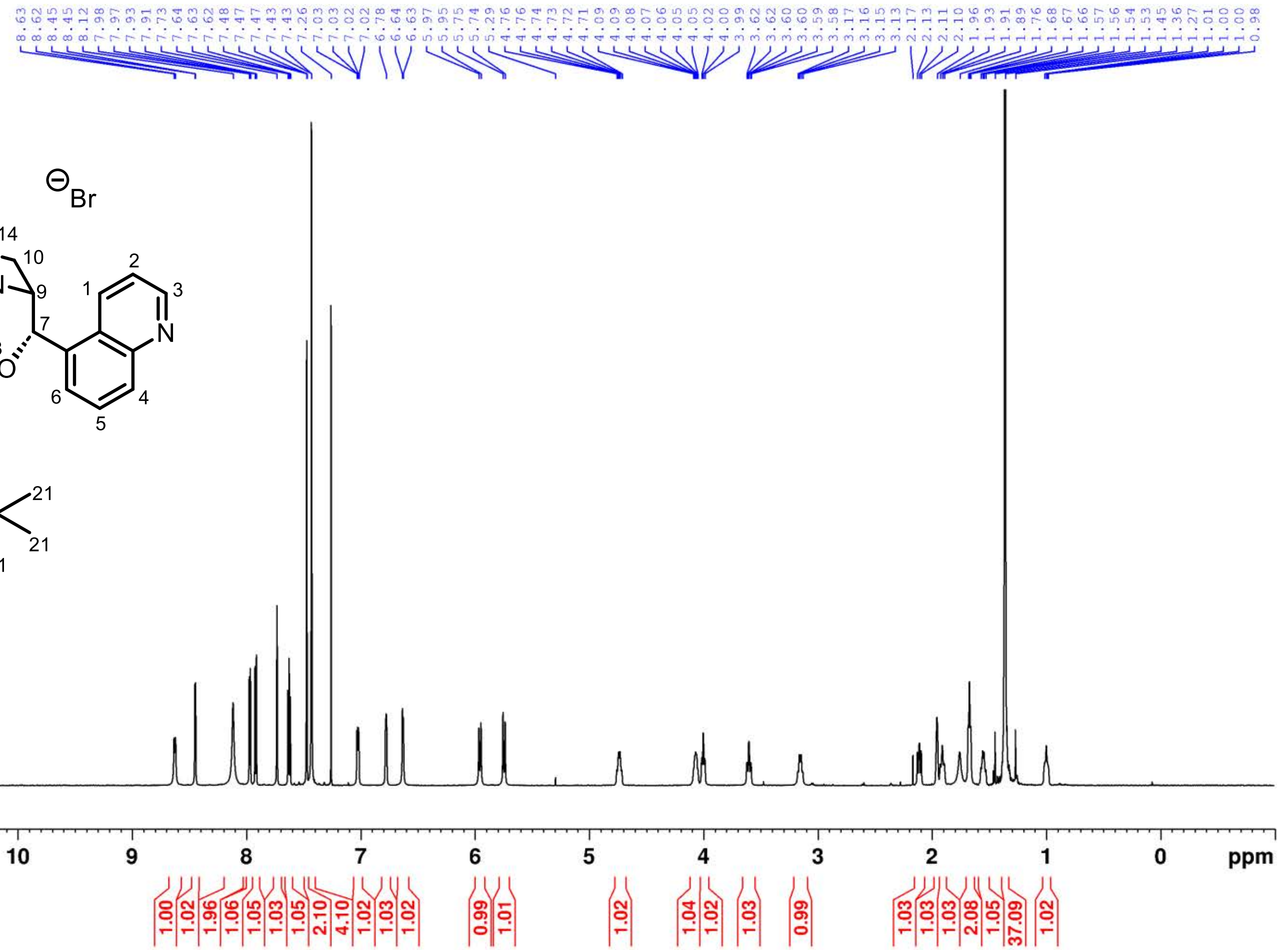

^{13}**C NMR** (176 MHz, $CDCl_3$) for *(1S,2R,4S)-2-((S)-hydroxy(quinolin-5-yl)methyl)-1-((3,3'',5,5''-tetra-tert-butyl-[1,1':3',1''-terphenyl]-5'-yl)methyl)quinuclidin-1-ium bromide* (**Cat8•Br**)

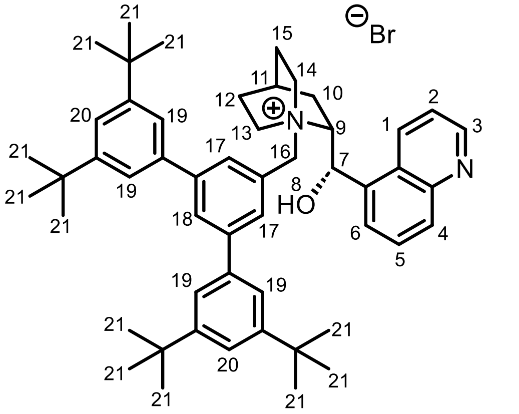

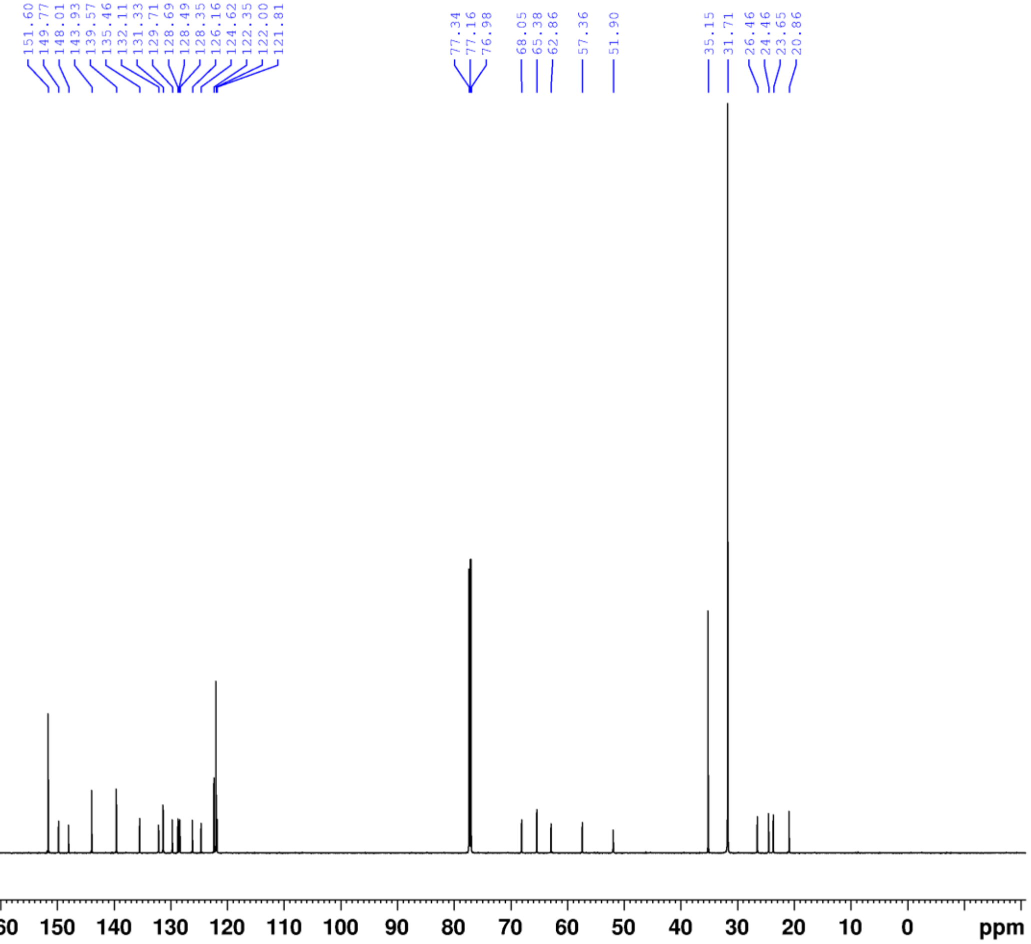

^{1}H NMR (700 MHz, $CDCl_3$) for *(S)-((1S,2R,4S)-5-formylquinuclidin-2-yl)(quinolin-4-yl)methyl acetate*

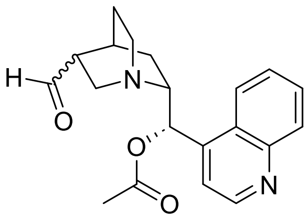

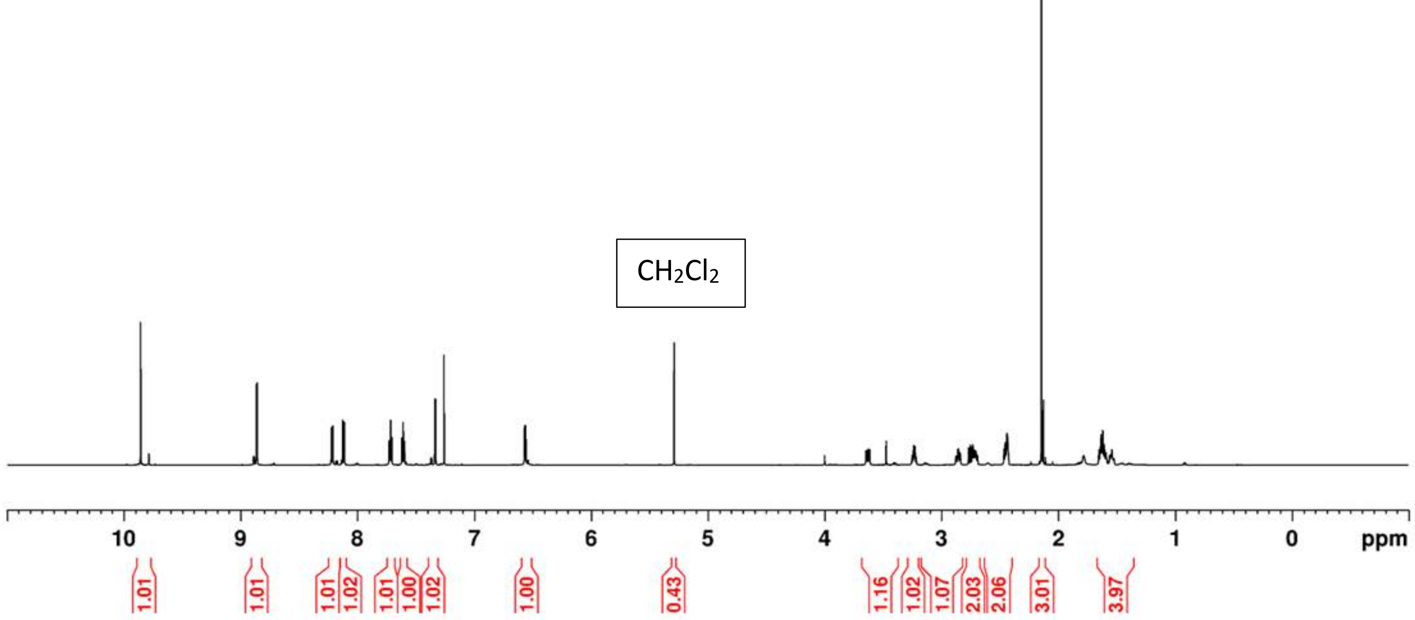

^{13}C NMR (176 MHz, $CDCl_3$) for *(S)-((1S,2R,4S)-5-formylquinuclidin-2-yl)(quinolin-4-yl)methyl acetate*

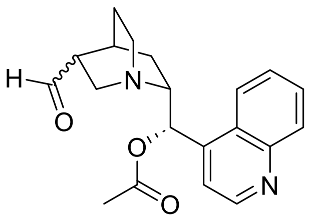

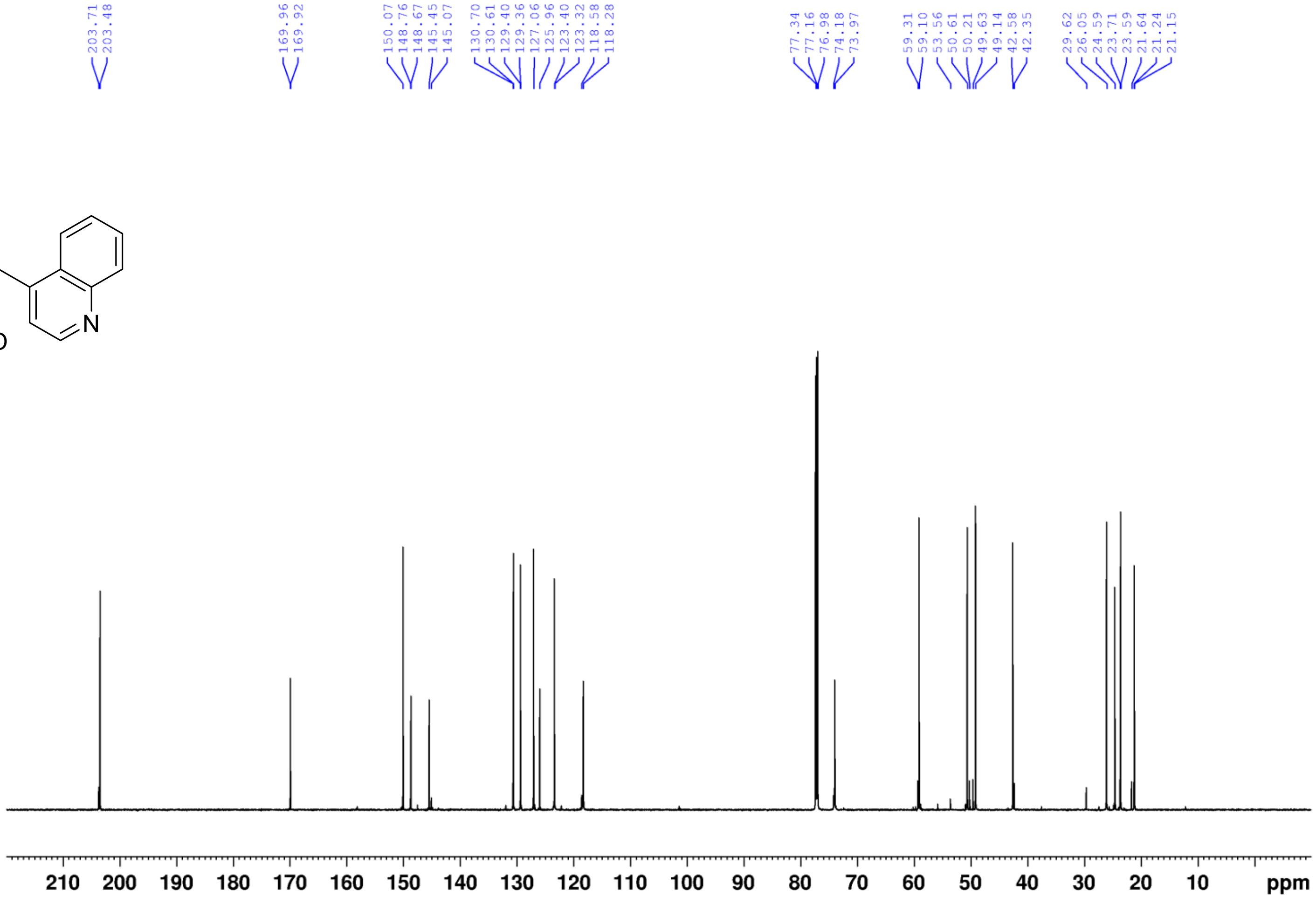

^{1}H NMR (400 MHz, $CDCl_3$) for *(S)-quinolin-4-yl((1S,2R,4S)-quinuclidin-2-yl)methanol*

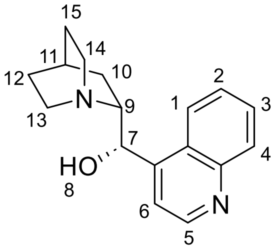

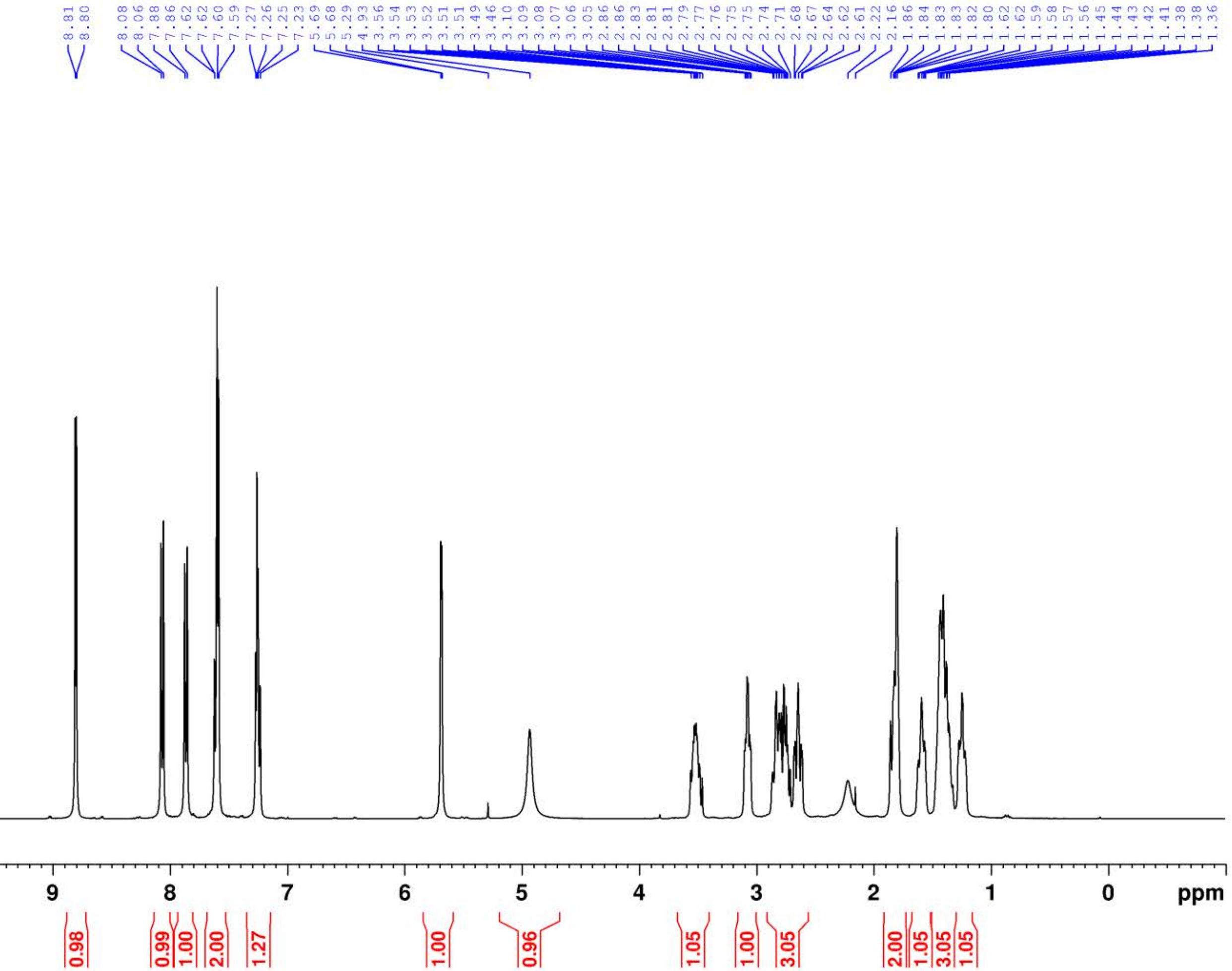

^{13}C NMR (101 MHz, $CDCl_3$) for *(S)-quinolin-4-yl((1S,2R,4S)-quinuclidin-2-yl)methanol*

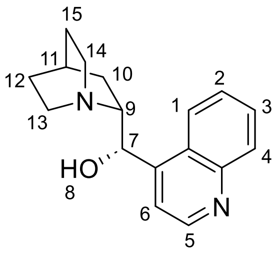

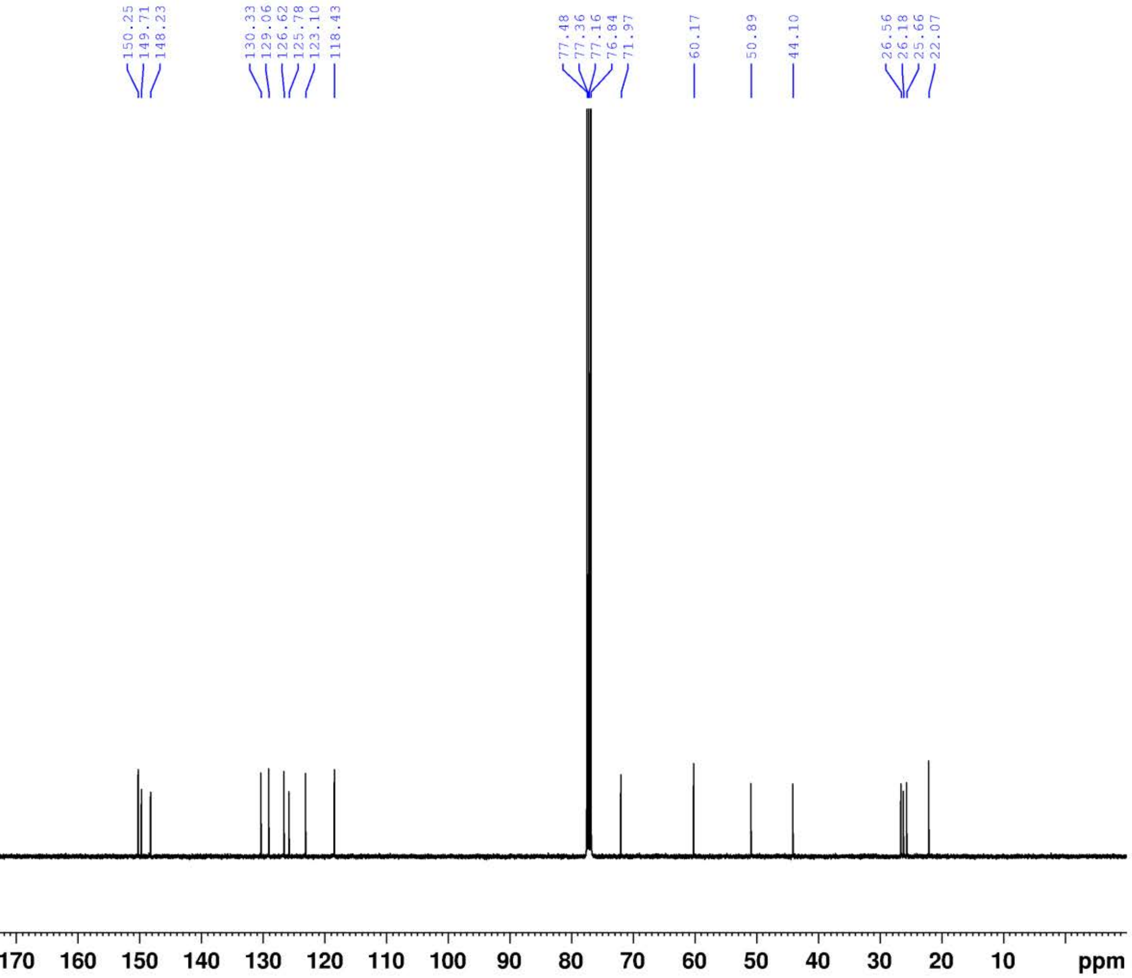

¹H NMR (700 MHz, $CDCl_3$) for *(1S,2R,4S)-2-((S)-hydroxy(quinolin-4-yl)methyl)-1-((3,3'',5,5''-tetra-tert-butyl-[1,1':3',1''-terphenyl]-5'-yl)methyl)quinuclidin-1-ium bromide* (**Cat9•Br**)

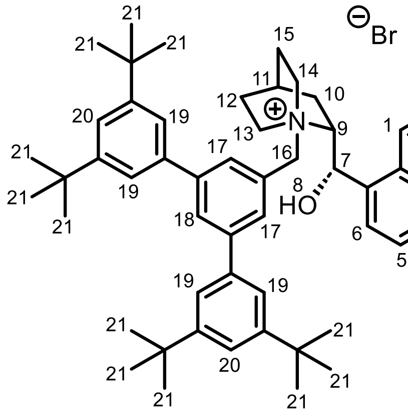

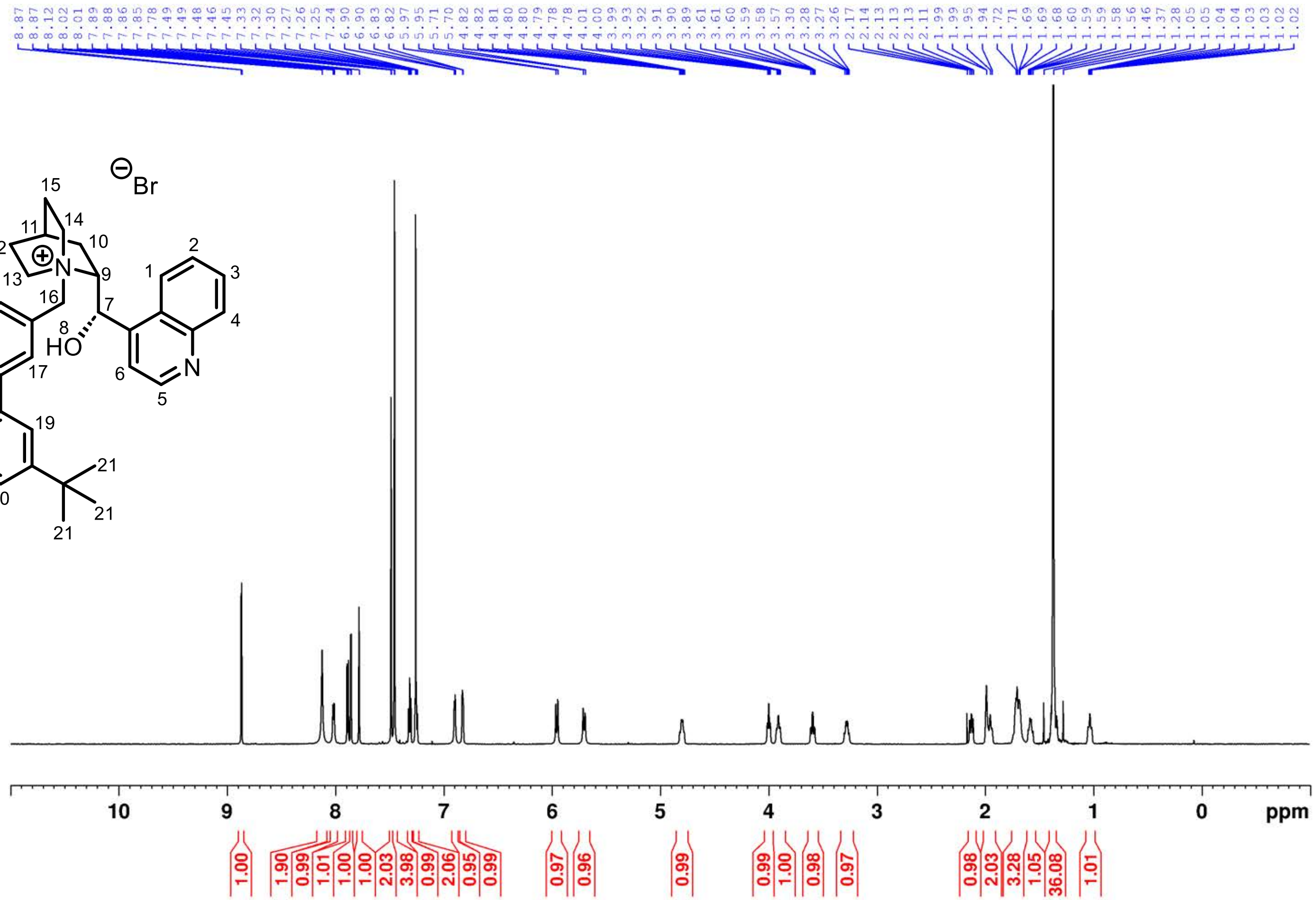

^{13}C NMR (176 MHz, $CDCl_3$) for *(1S,2R,4S)-2-((S)-hydroxy(quinolin-4-yl)methyl)-1-((3,3'',5,5''-tetra-tert-butyl-[1,1':3',1''-terphenyl]-5'-yl)methyl)quinuclidin-1-ium bromide* (**Cat9•Br**)

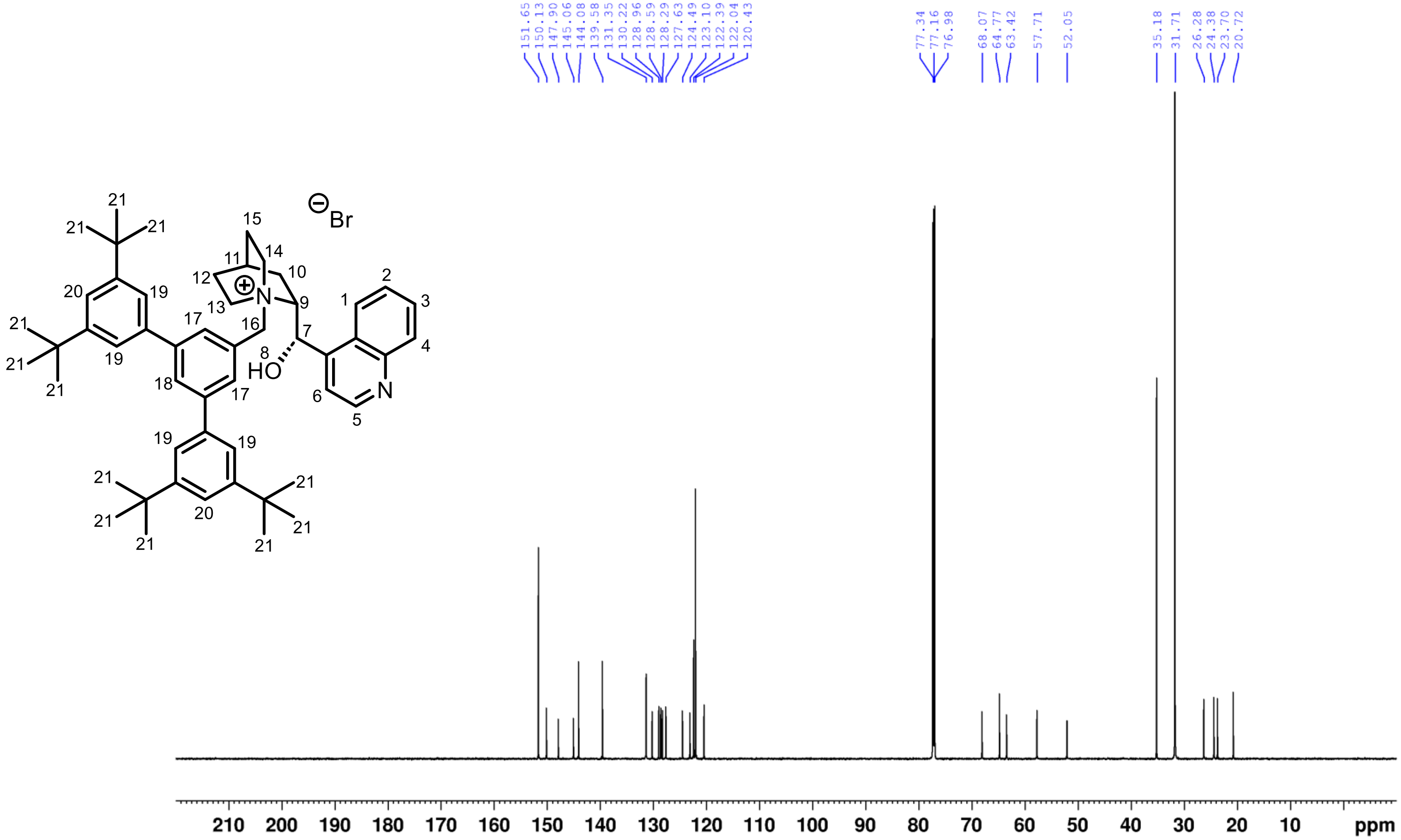

^{1}H NMR (400 MHz, $CDCl_3$) for *(S)-(2-butyl-6-methoxyquinolin-4-yl)((1S,2R,4S,5R)-5-ethylquinuclidin-2-yl)methanol*

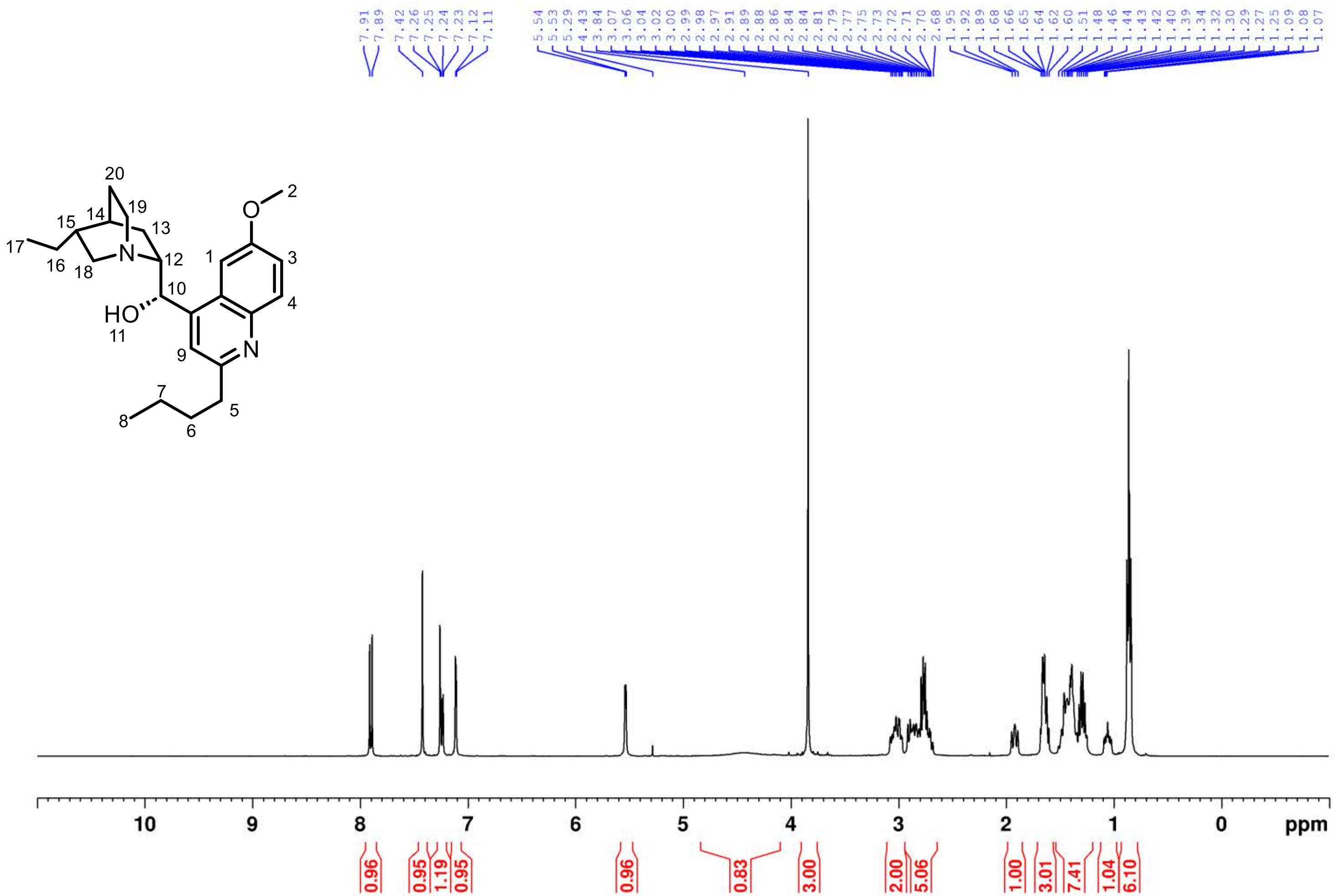

^{13}C NMR (101 MHz, $CDCl_3$) for *(S)-(2-butyl-6-methoxyquinolin-4-yl)((1S,2R,4S,5R)-5-ethylquinuclidin-2-yl)methanol*

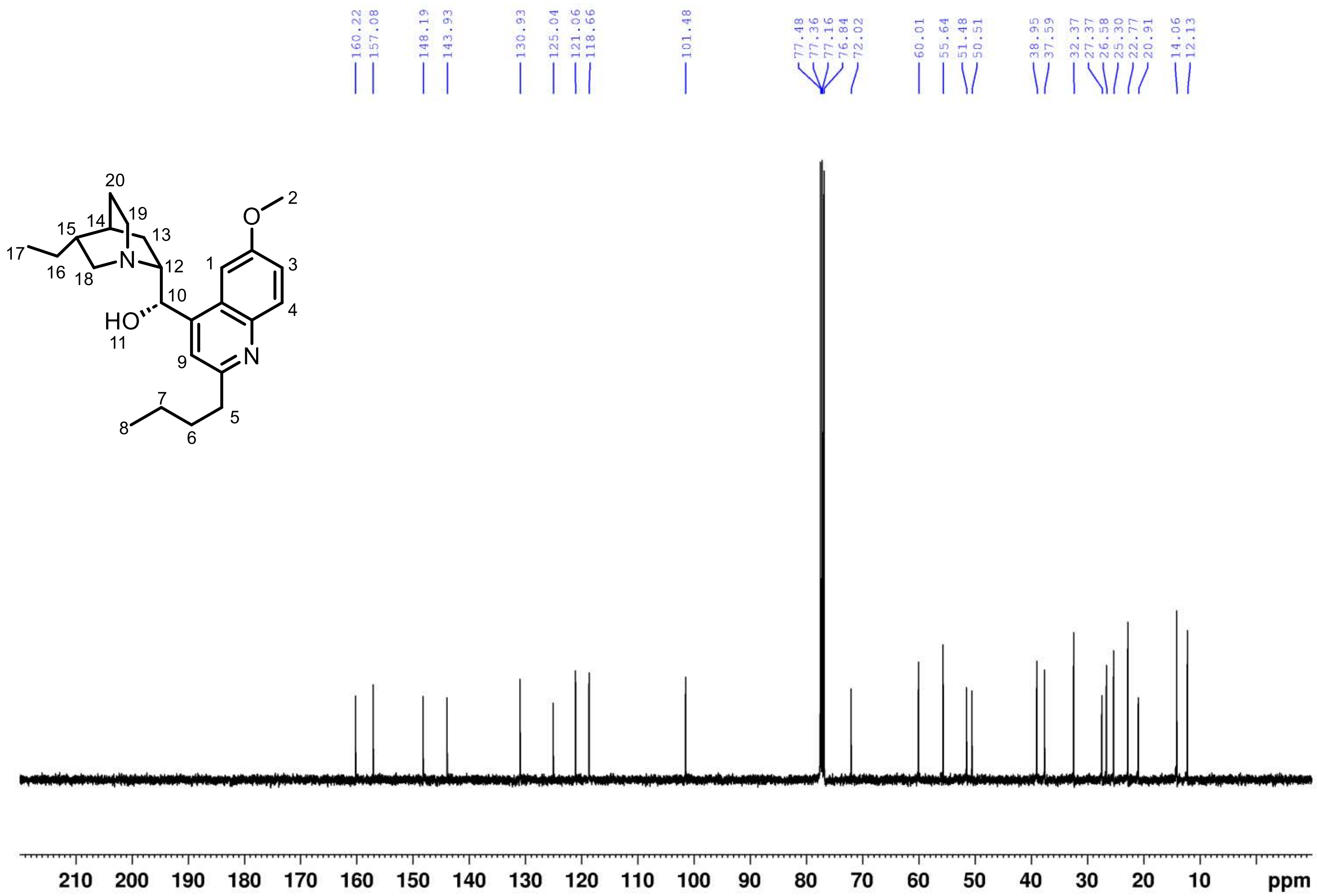

^{1}H NMR (700 MHz, $CDCl_3$) for *(1S,2R,4S,5R)-2-((S)-(2-butyl-6-methoxyquinolin-4-yl)(hydroxy)methyl)-5-ethyl-1-((3,3'',5,5''-tetra-tert-butyl-[1,1':3',1''-terphenyl]-5'-yl)methyl)quinuclidin-1-ium bromide* (**Cat11•Br**)

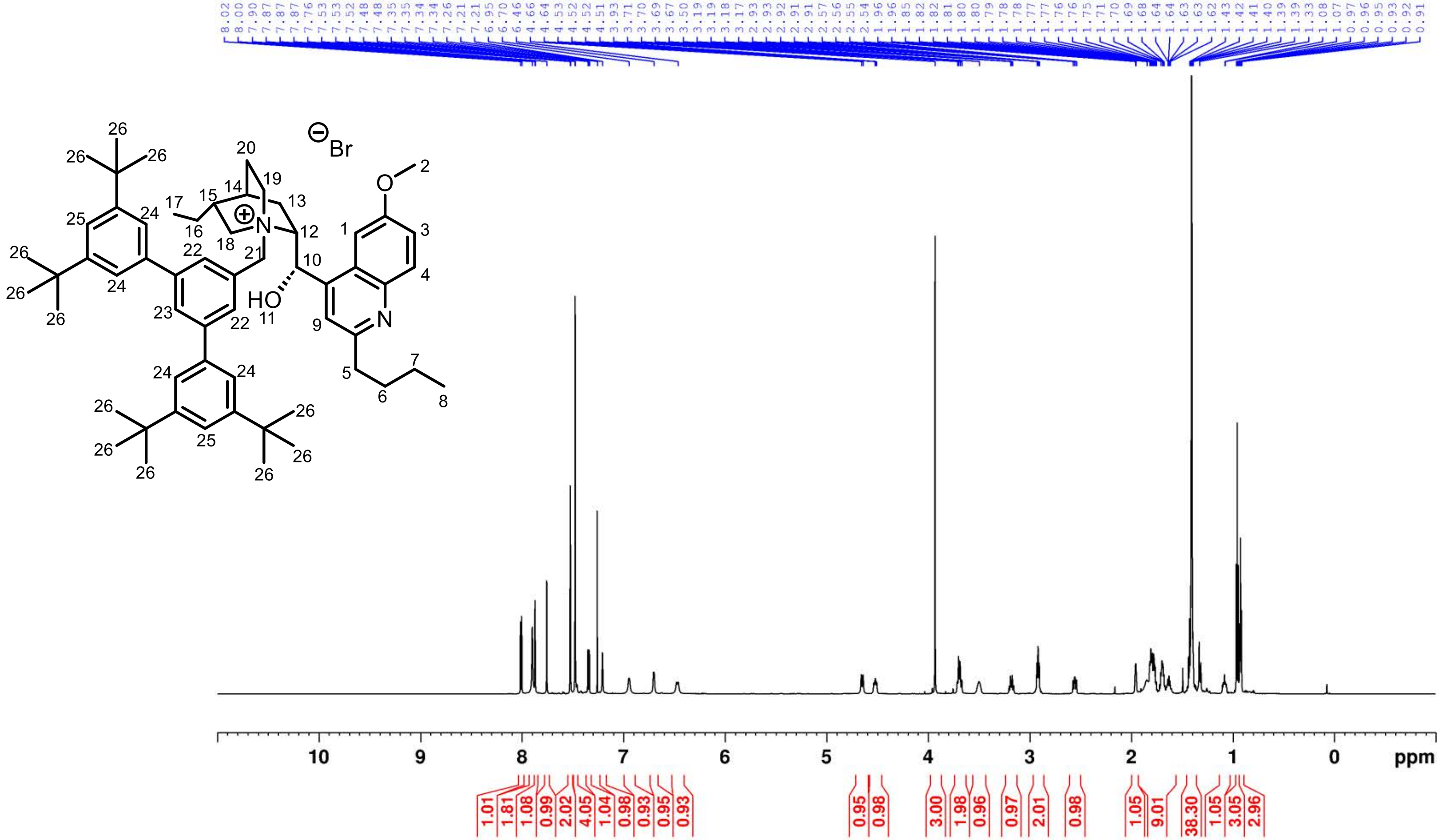

^{13}C NMR (176 MHz, $CDCl_3$) for *(1S,2R,4S,5R)-2-((S)-(2-butyl-6-methoxyquinolin-4-yl)(hydroxy)methyl)-5-ethyl-1-((3,3'',5,5''-tetra-tert-butyl-[1,1':3',1''-terphenyl]-5'-yl)methyl)quinuclidin-1-ium bromide* (**Cat11•Br**)

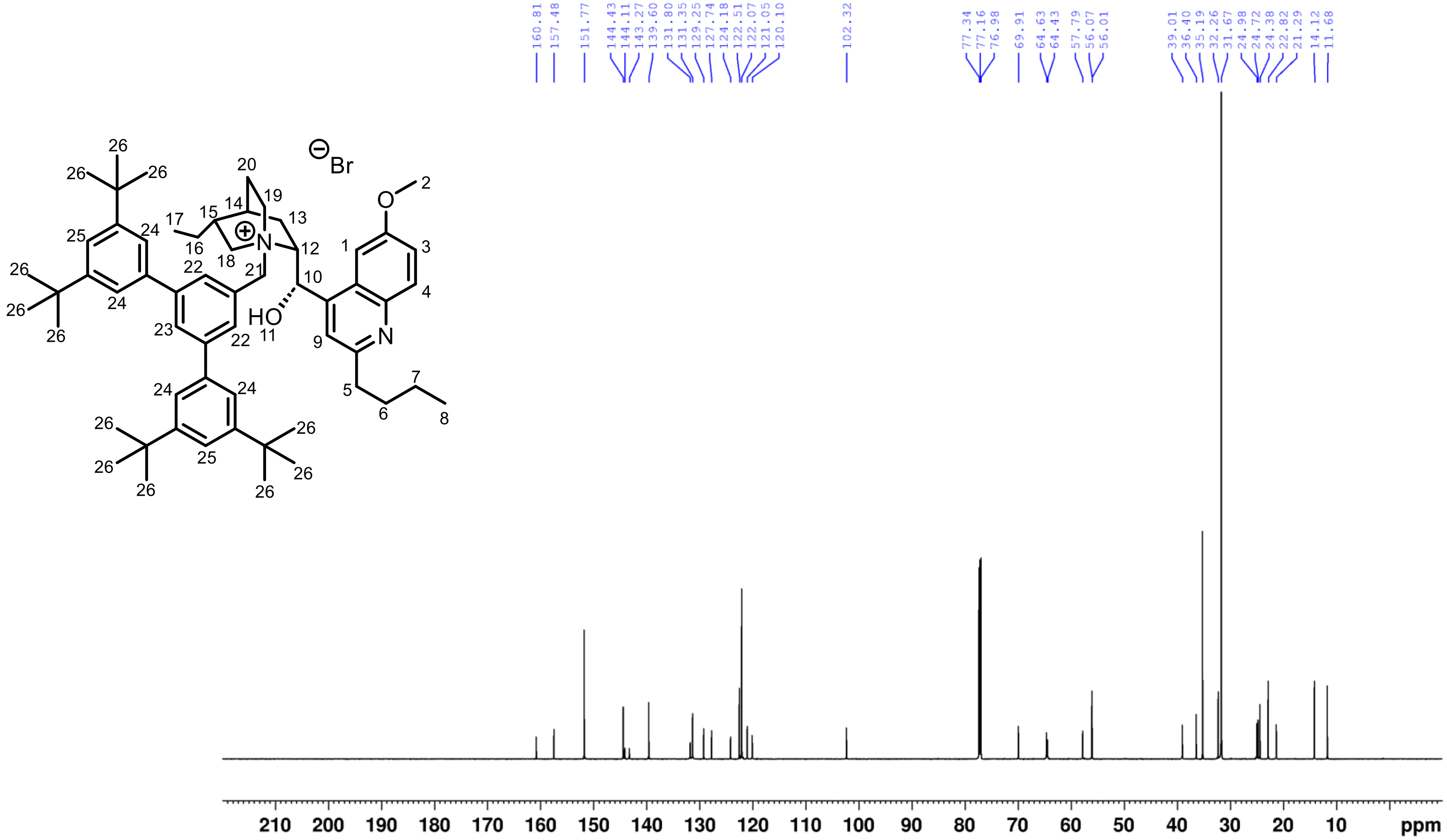

¹H NMR (700 MHz, DMSO-d_6) for *(1S,2R,4S,5R)-5-ethyl-2-((S)-methoxy(6-methoxyquinolin-4-yl)methyl)-1-((3,3'',5,5''-tetra-tert-butyl-[1,1':3',1''-terphenyl]-5'-yl)methyl)quinuclidin-1-ium bromide* (**Cat12•Br**)

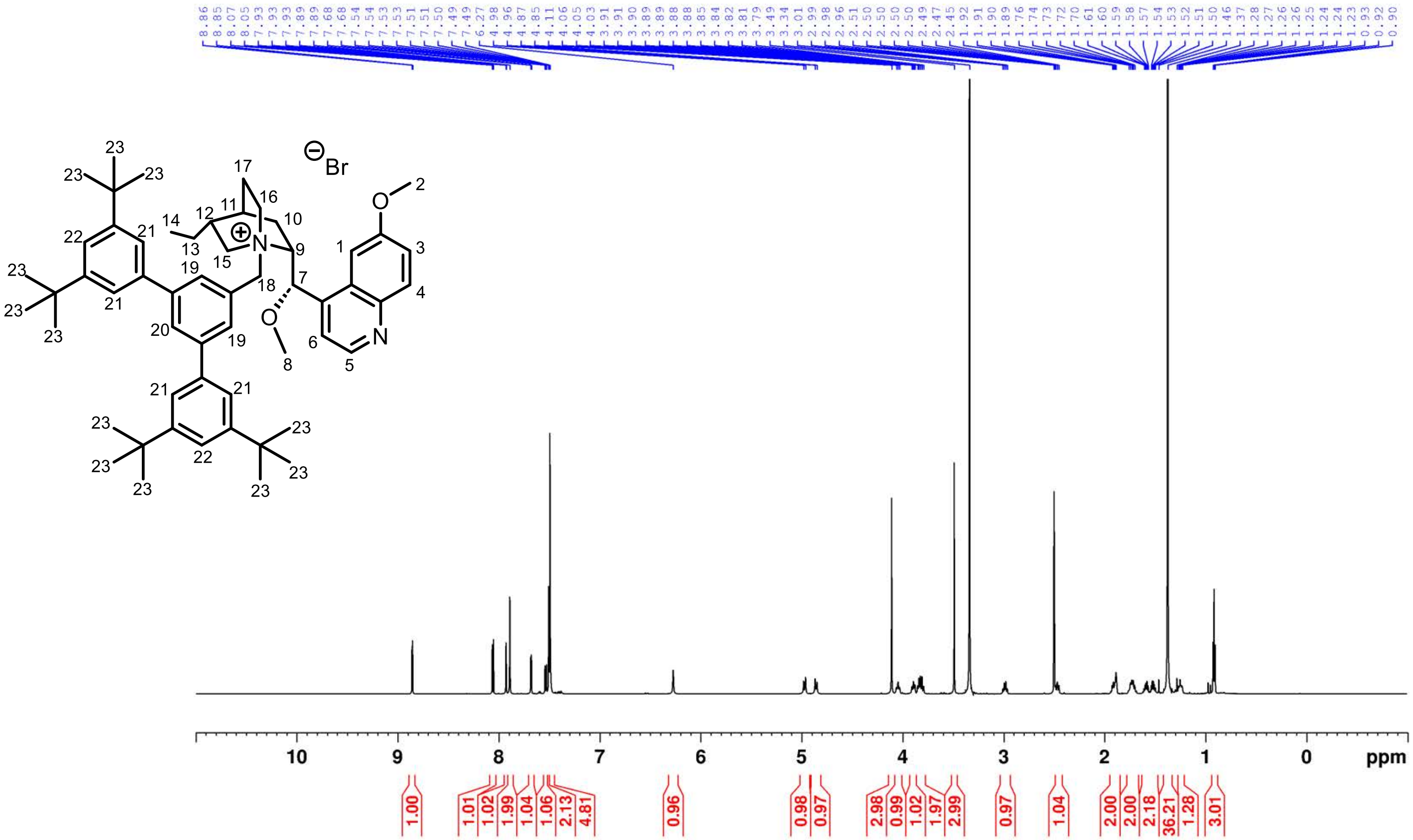

^{13}C NMR (176 MHz, DMSO-d_6) for *(1S,2R,4S,5R)-5-ethyl-2-((S)-methoxy(6-methoxyquinolin-4-yl)methyl)-1-((3,3'',5,5''-tetra-tert-butyl-[1,1':3',1''-terphenyl]-5'-yl)methyl)quinuclidin-1-ium bromide* (**Cat12•Br**)

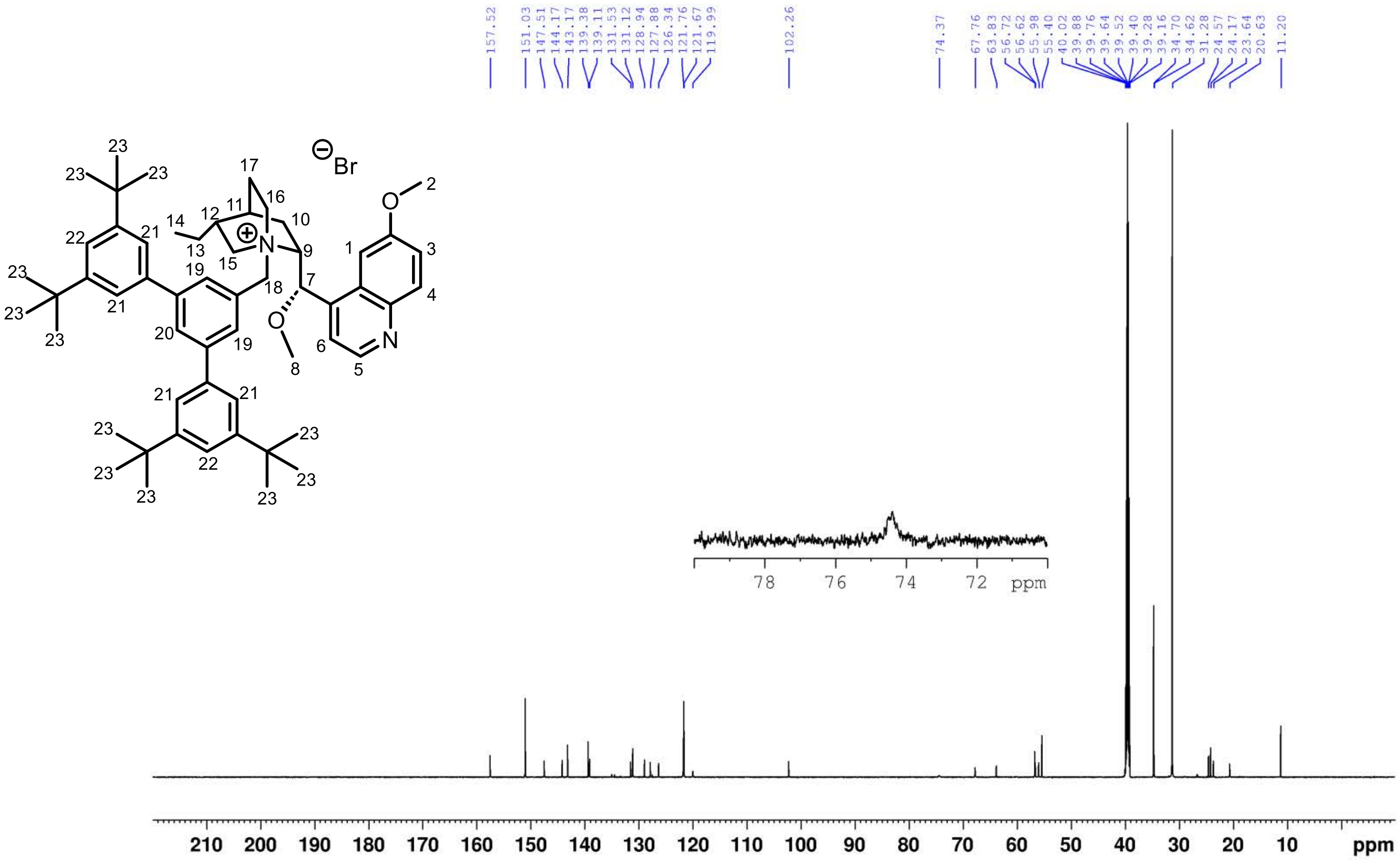

^{1}H NMR (400 MHz, CDCl$_3$) for *dimethyl 5-(phenoxysulfonyl)isophthalate*

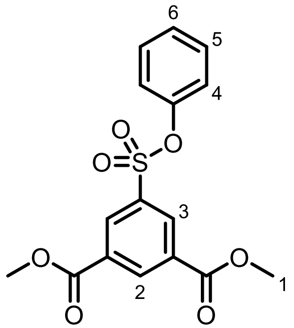

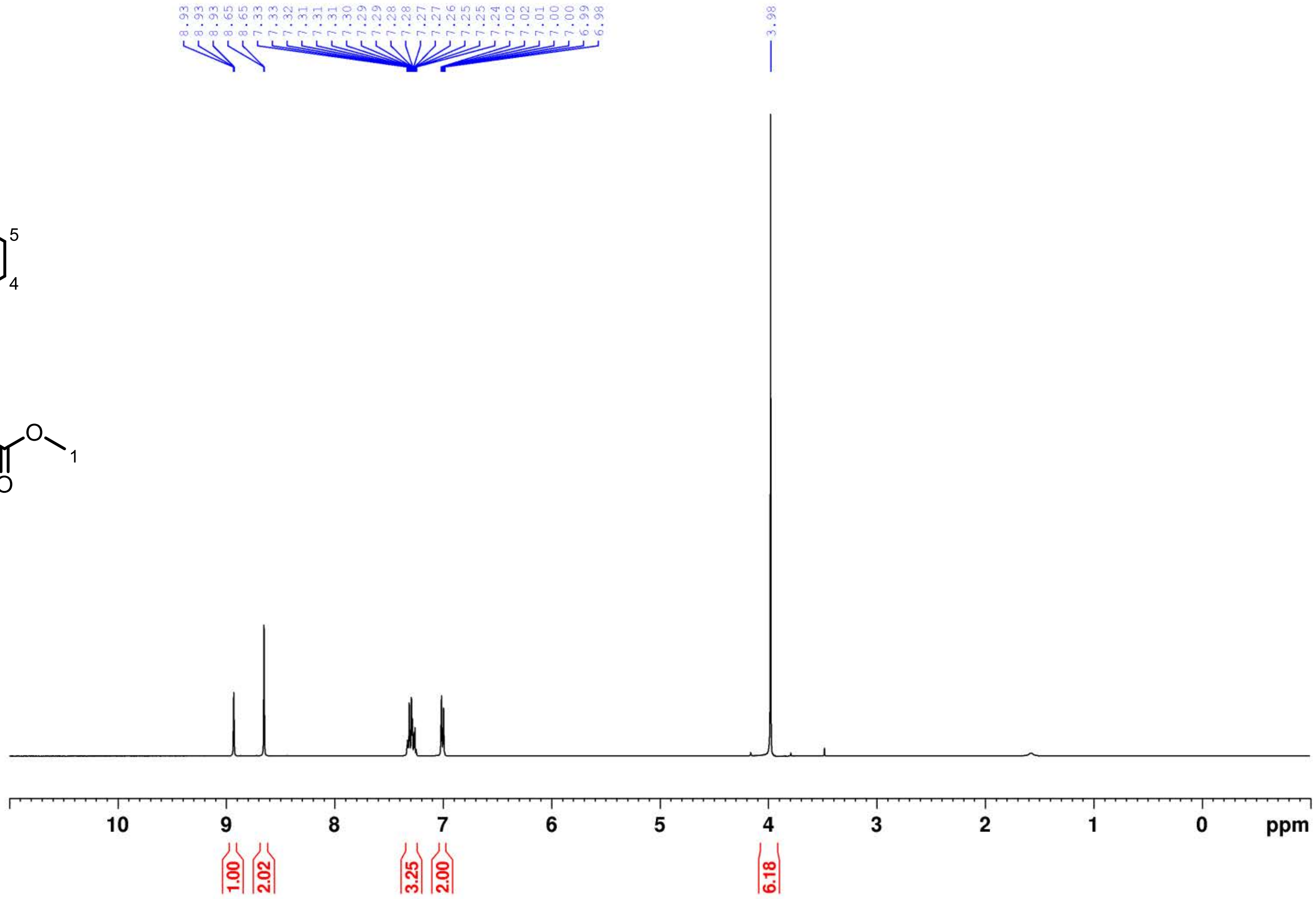

^{13}C NMR (101 MHz, $CDCl_3$) for *dimethyl 5-(phenoxysulfonyl)isophthalate*

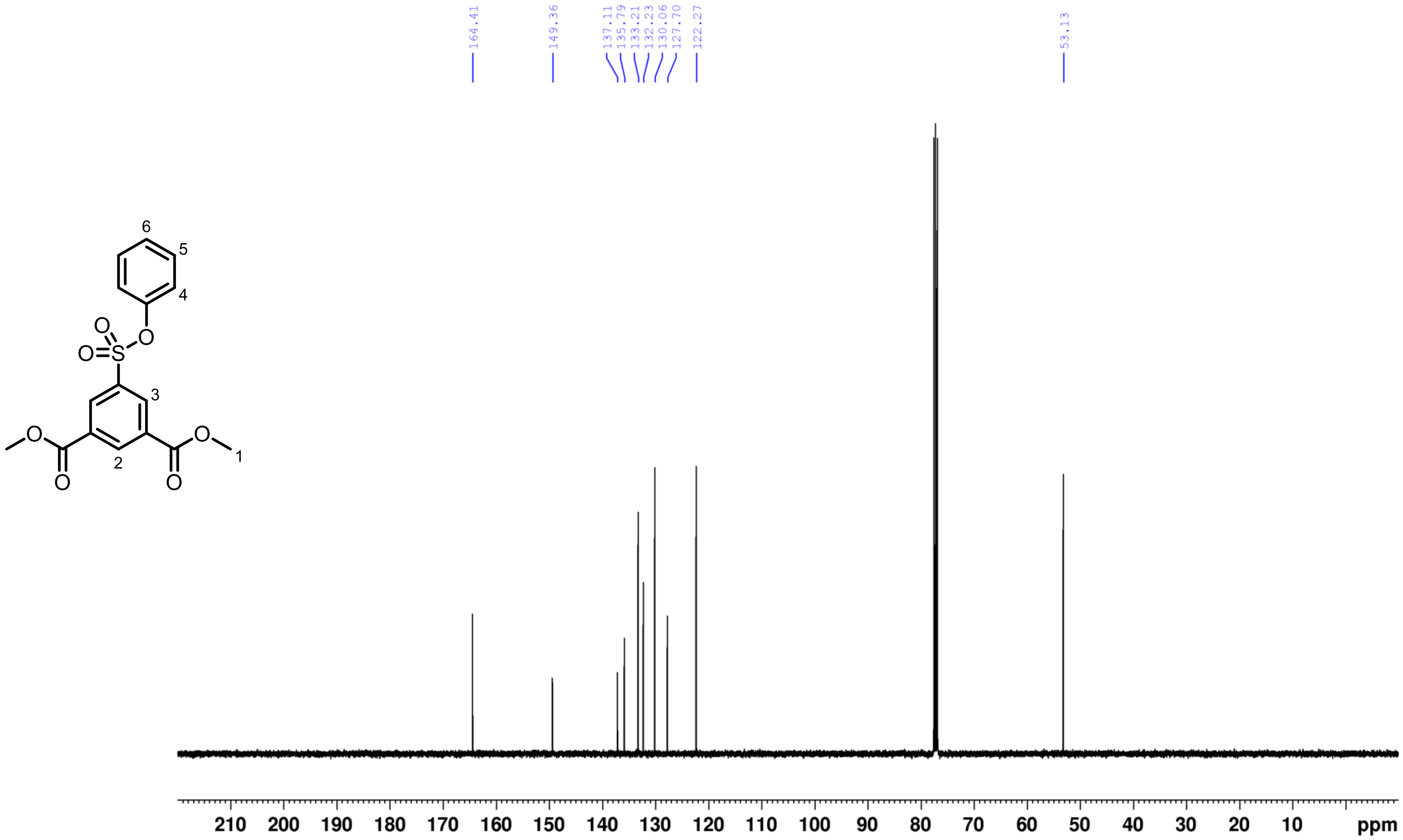

^{1}H NMR (400 MHz, $CDCl_3$) for *phenyl 3,5-bis(hydroxymethyl)benzenesulfonate*

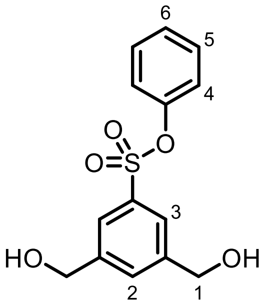

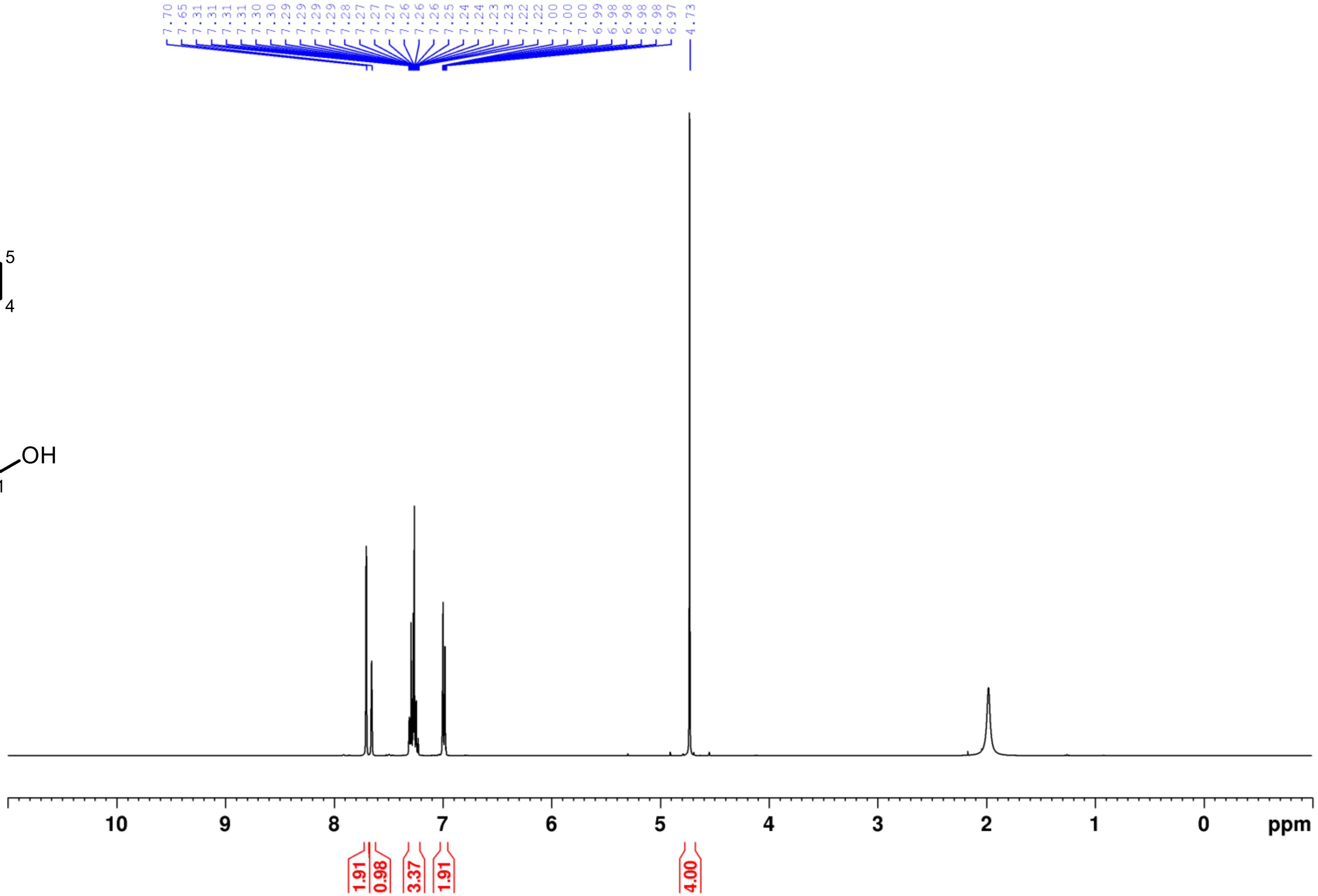

^{13}C NMR (101 MHz, CDCl$_3$) for *phenyl 3,5-bis(hydroxymethyl)benzenesulfonate*

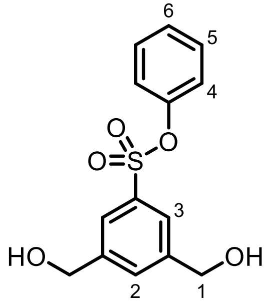

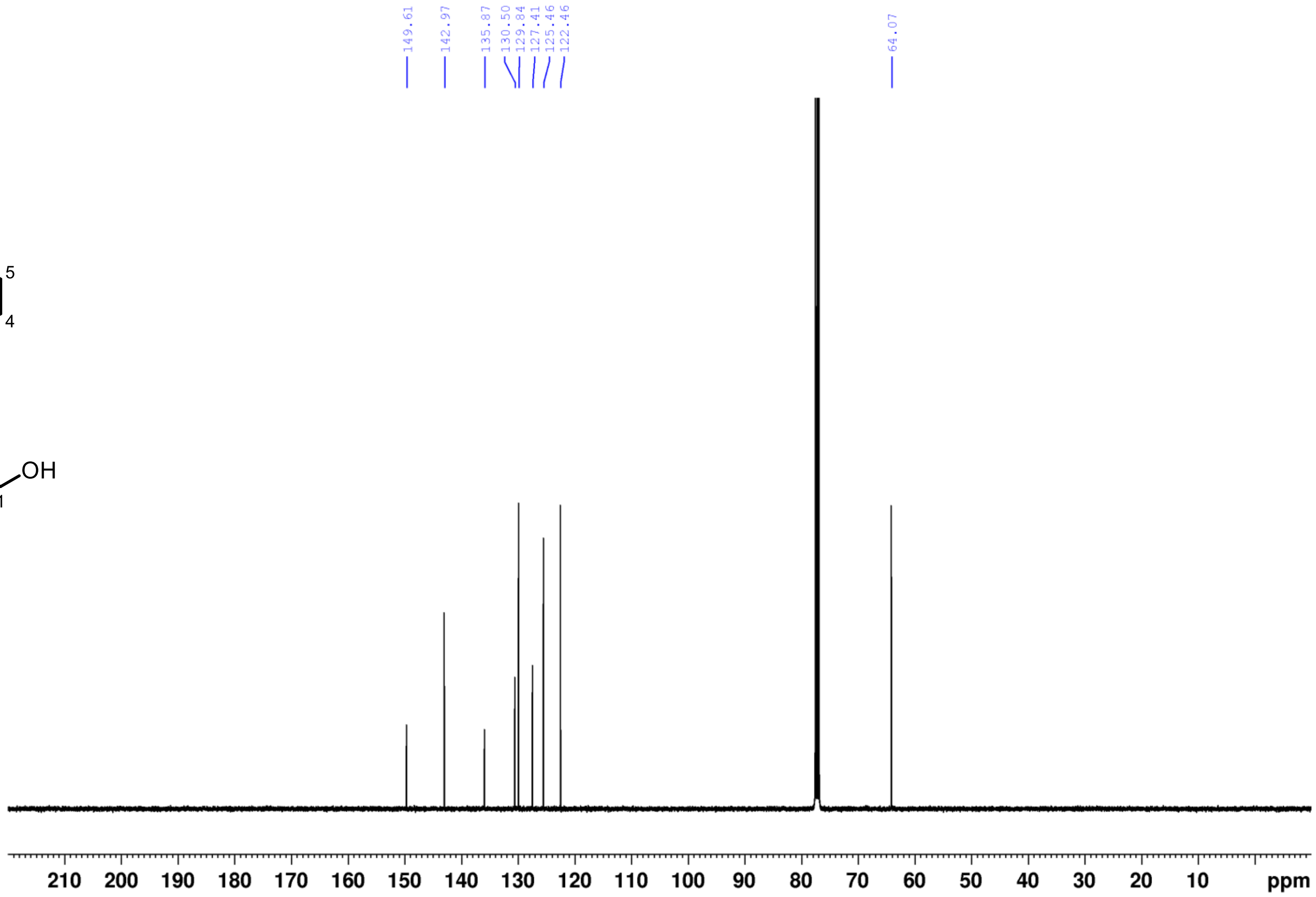

^{1}H NMR (400 MHz, $CDCl_3$) for *phenyl 3,5-bis(bromomethyl)benzenesulfonate*

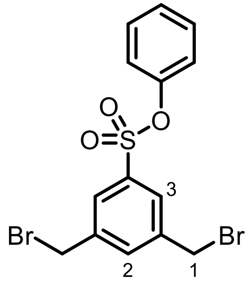

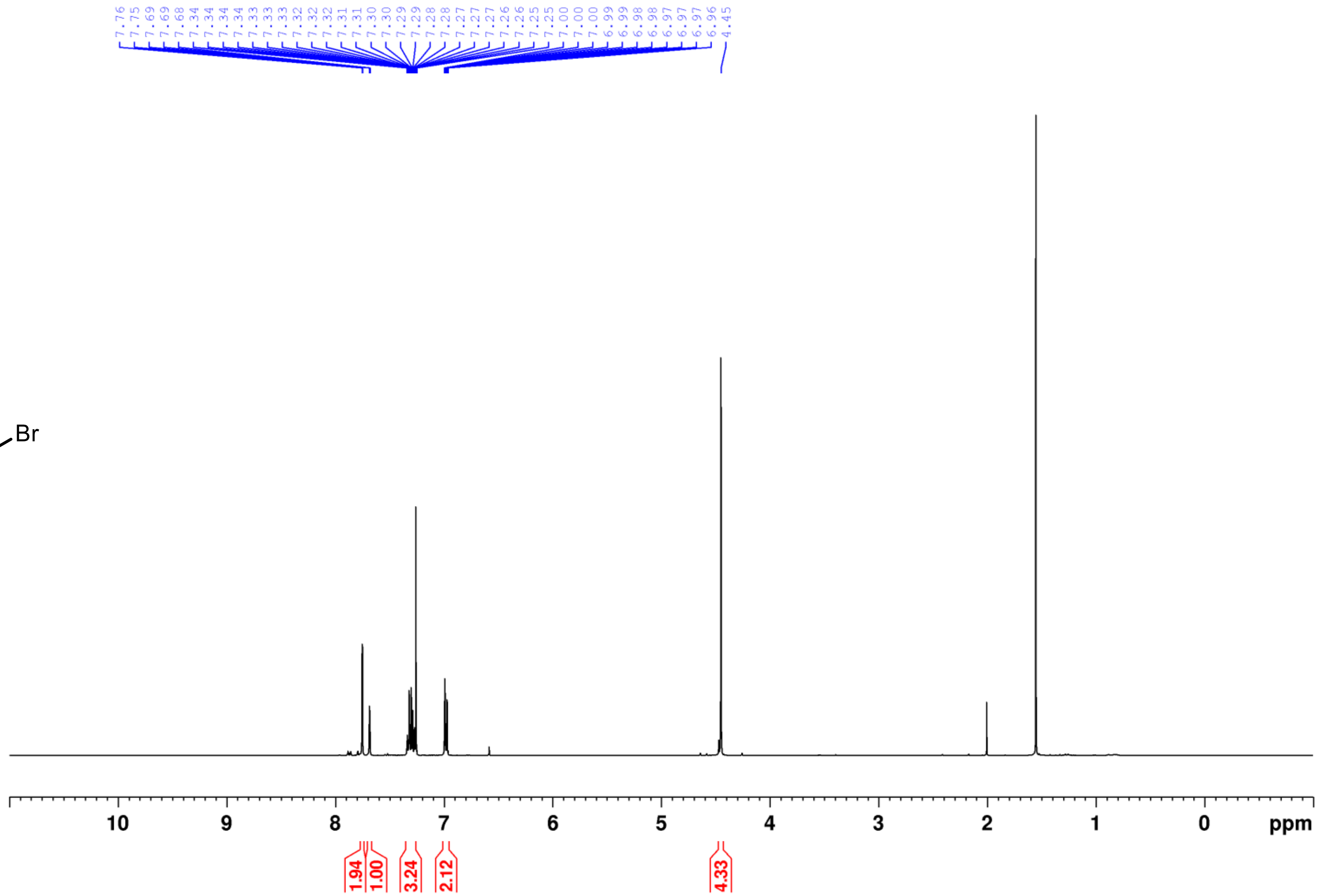

^{13}C NMR (101 MHz, CDCl$_3$) for *phenyl 3,5-bis(bromomethyl)benzenesulfonate*

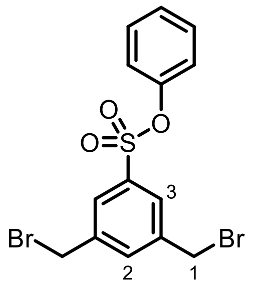

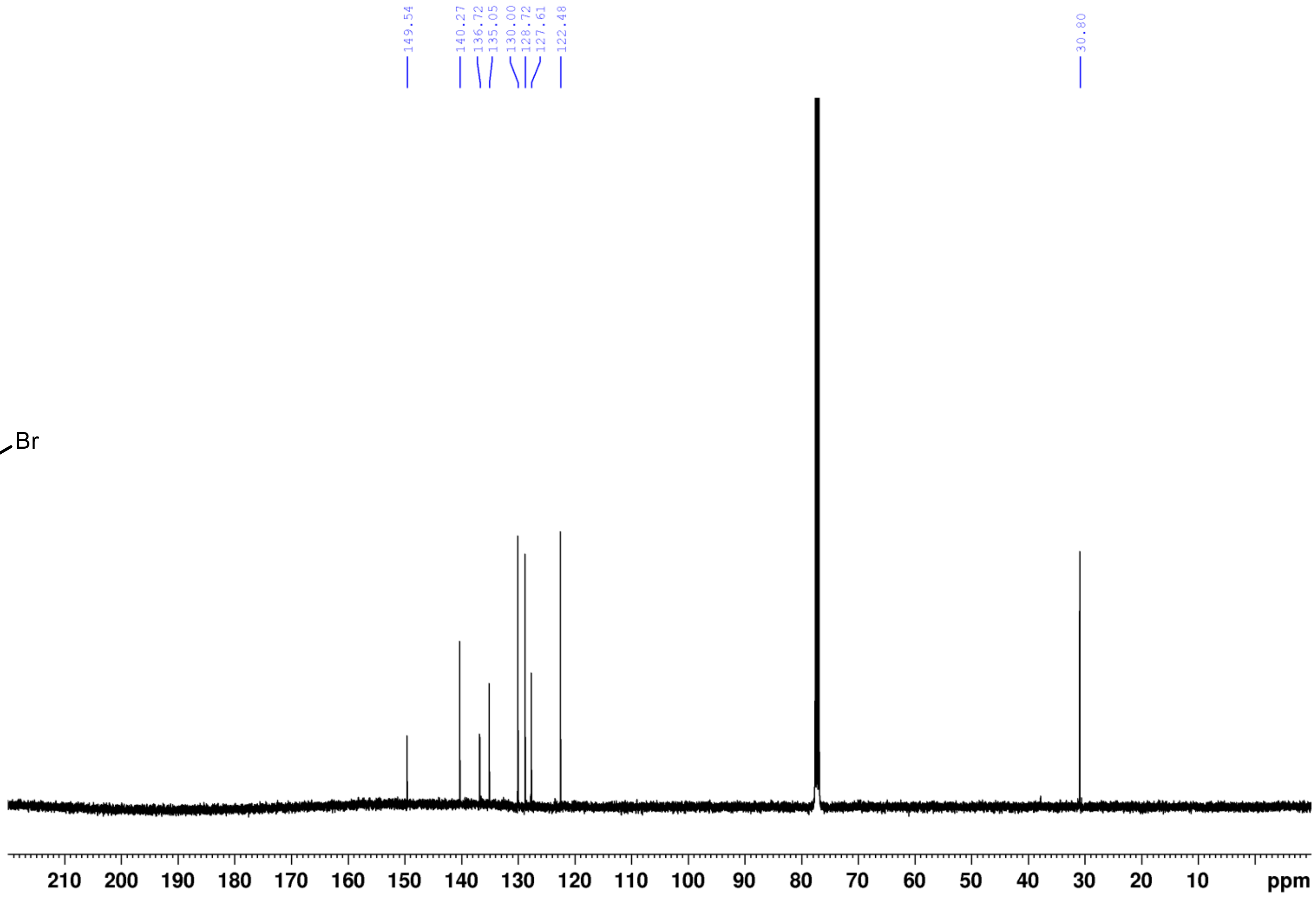

^{1}H NMR (400 MHz, CD_3OD) for *dimethyl 3,3'-(5-(phenoxysulfonyl)-1,3-phenylene)bis(2,2-dimethylpropanoate)*

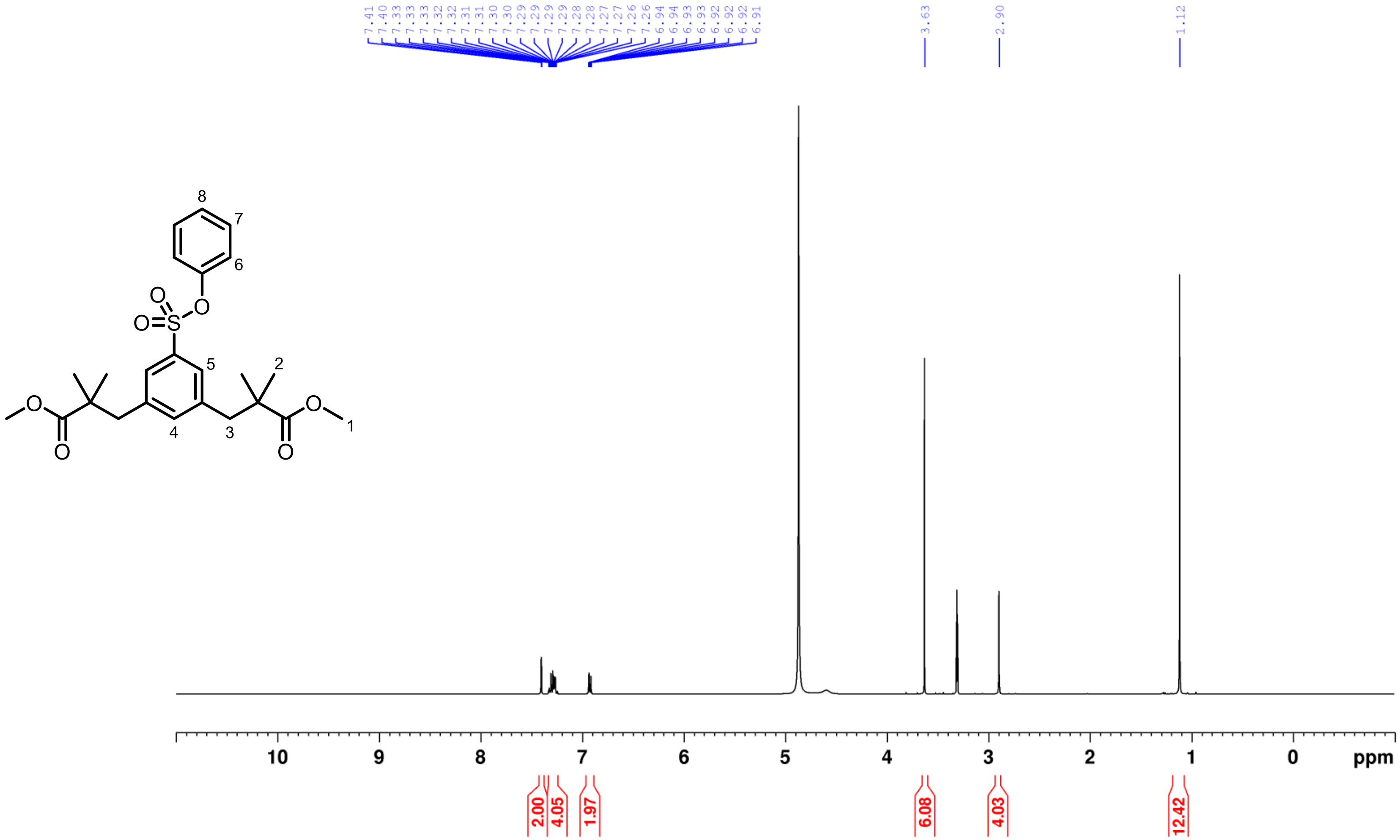

^{13}**C NMR (101 MHz, CD$_3$OD)** for *dimethyl 3,3'-(5-(phenoxysulfonyl)-1,3-phenylene)bis(2,2-dimethylpropanoate)*

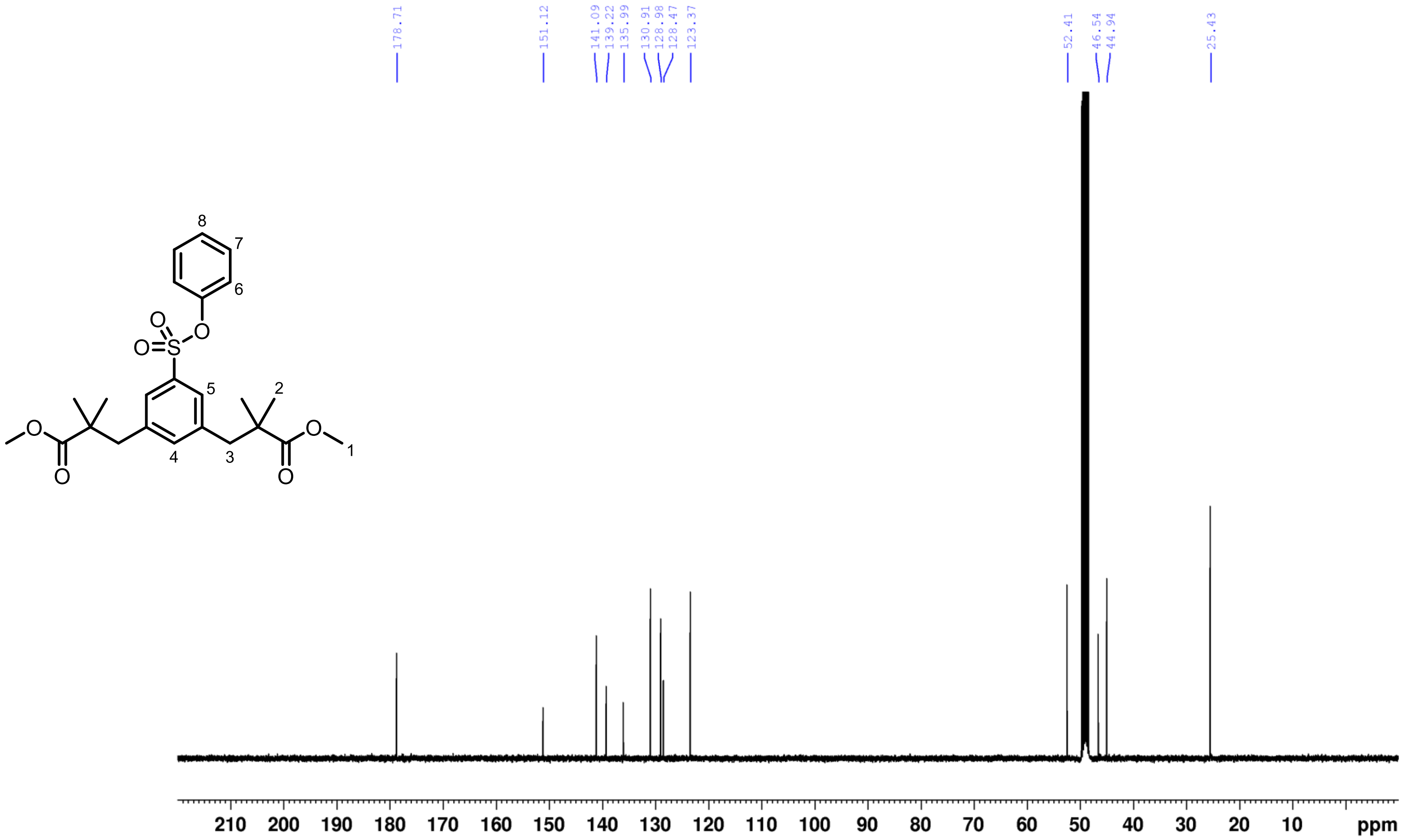

^{1}H NMR (400 MHz, CD_3OD) for *tetrabutylammonium 3,5-bis(2-carboxy-2-methylpropyl)benzenesulfonate*

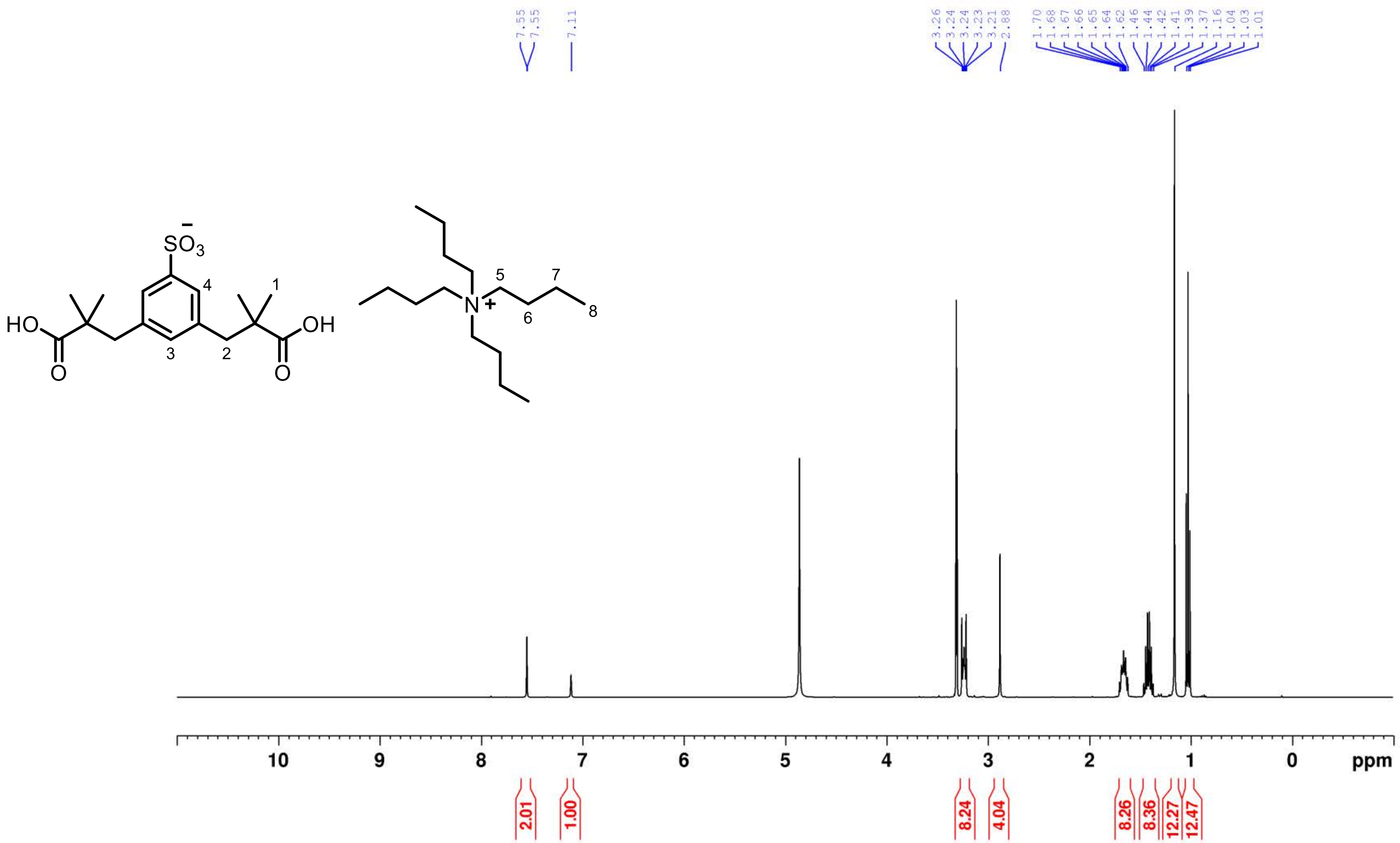

^{13}C NMR (101 MHz, CD_3OD) for *tetrabutylammonium 3,5-bis(2-carboxy-2-methylpropyl)benzenesulfonate*

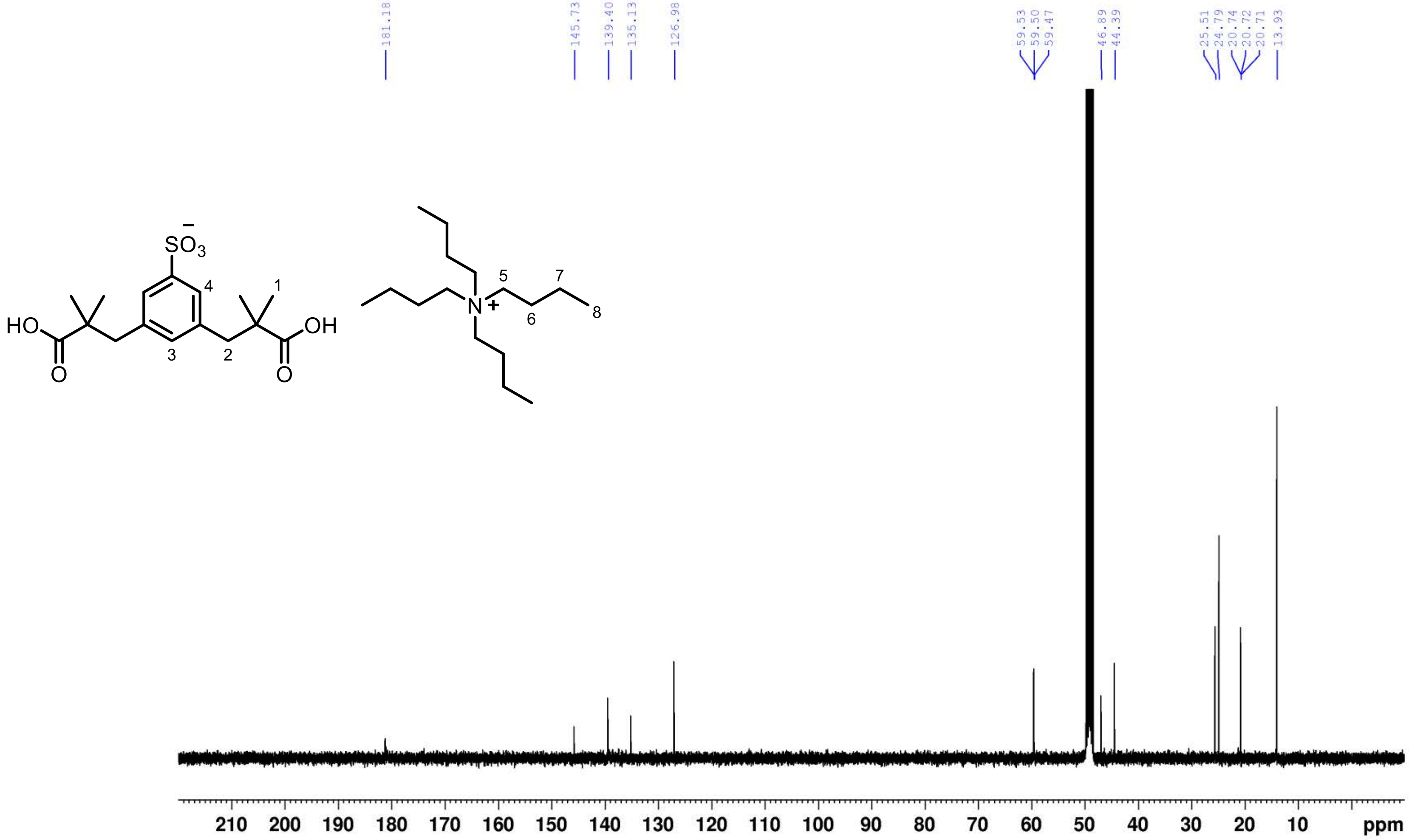

^{1}H NMR (400 MHz, CD_3OD) for *bis[rhodium tetrabutylammonium 3,5-bis(2-carboxy-2-methylpropyl)benzenesulfonate]* (Rh_2(**A-II**)$_2$●(**NBu_4**)$_2$)

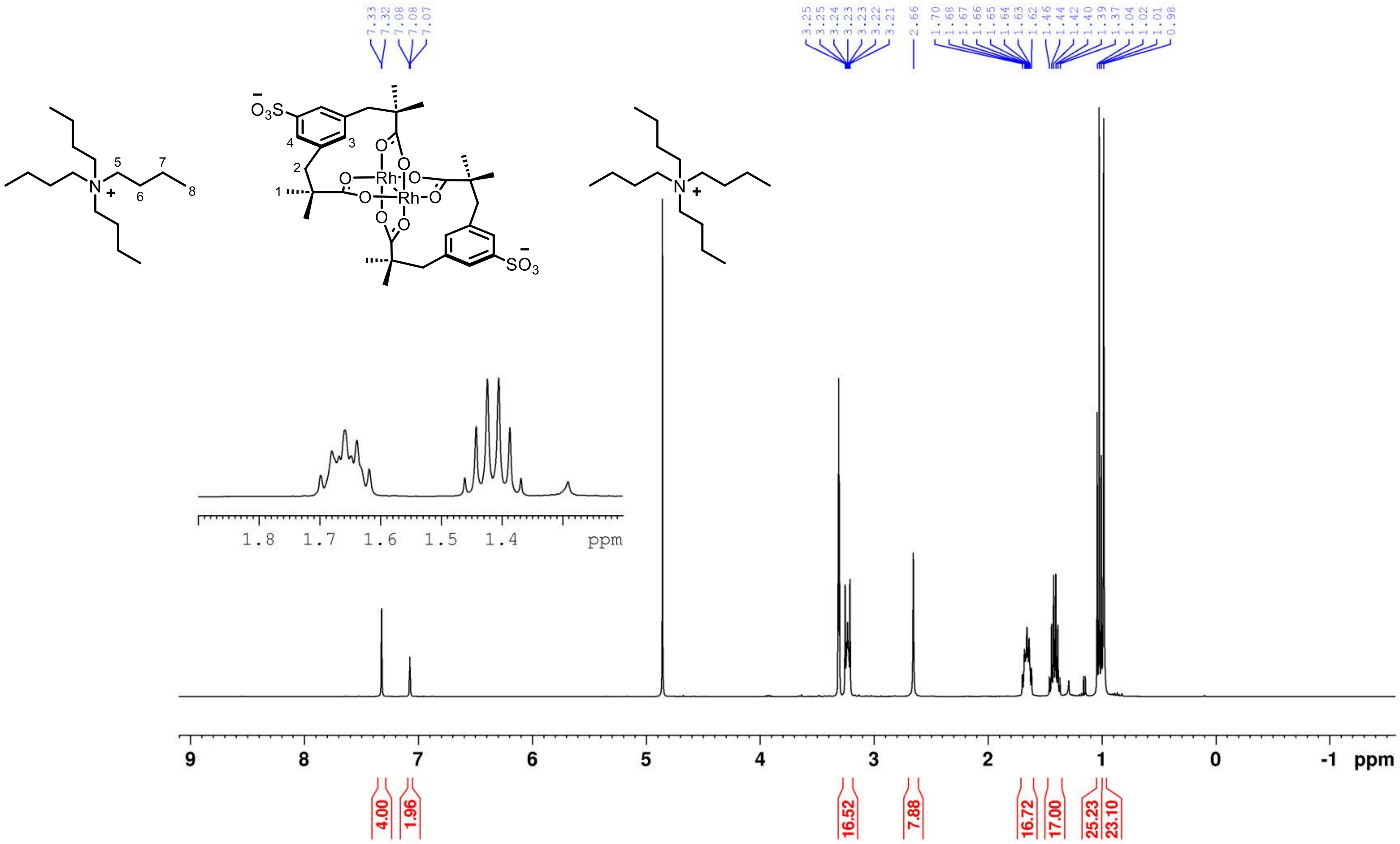

^{13}C NMR (101 MHz, CD_3OD) for *bis[rhodium tetrabutylammonium 3,5-bis(2-carboxy-2-methylpropyl)benzenesulfonate]* (Rh_2(**A-II**)$_2$●(**NBu_4**)$_2$)

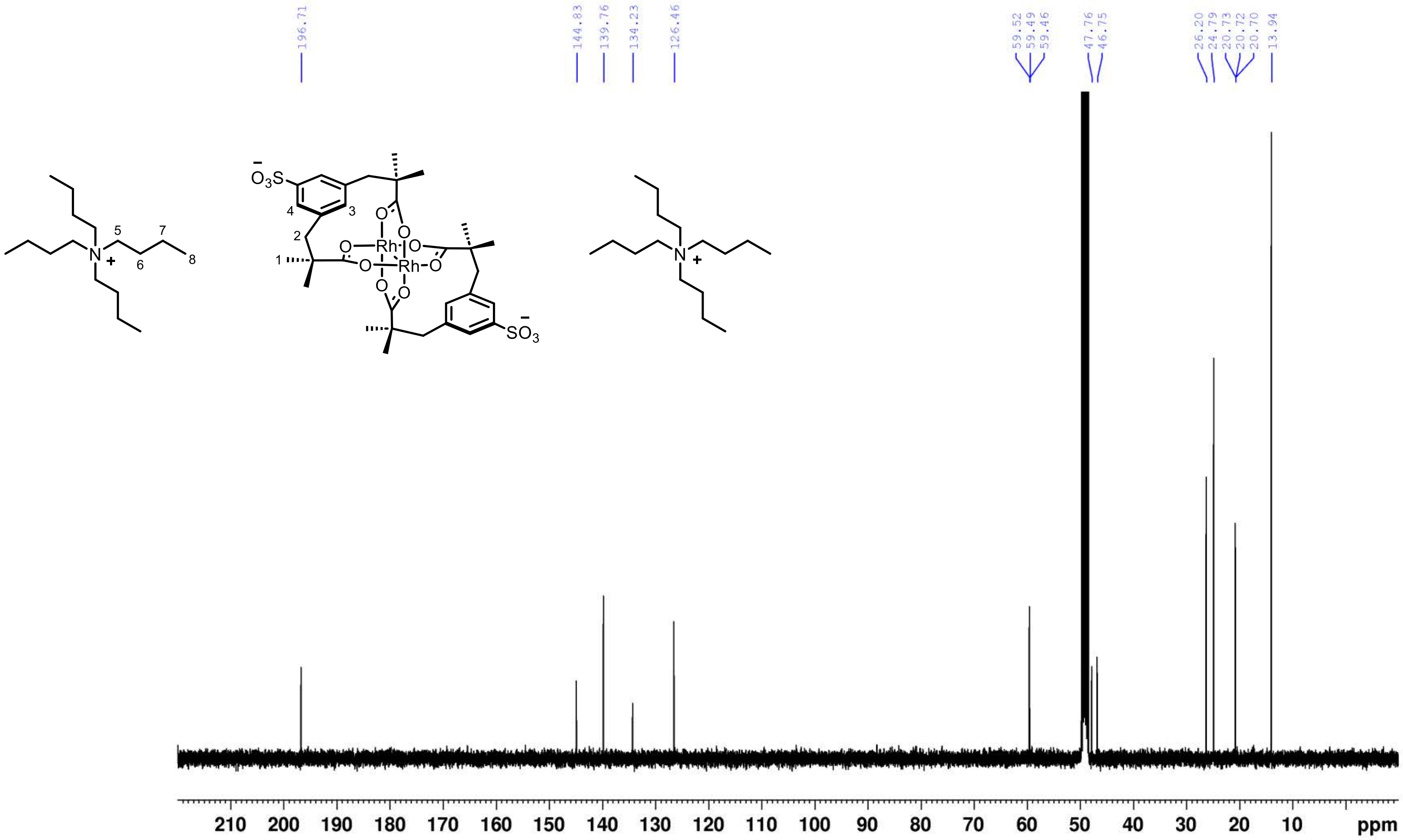

^{1}H NMR (400 MHz, $CDCl_3$) for *1-bromo-3,5-bis(bromomethyl)benzene*

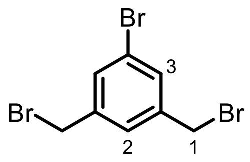

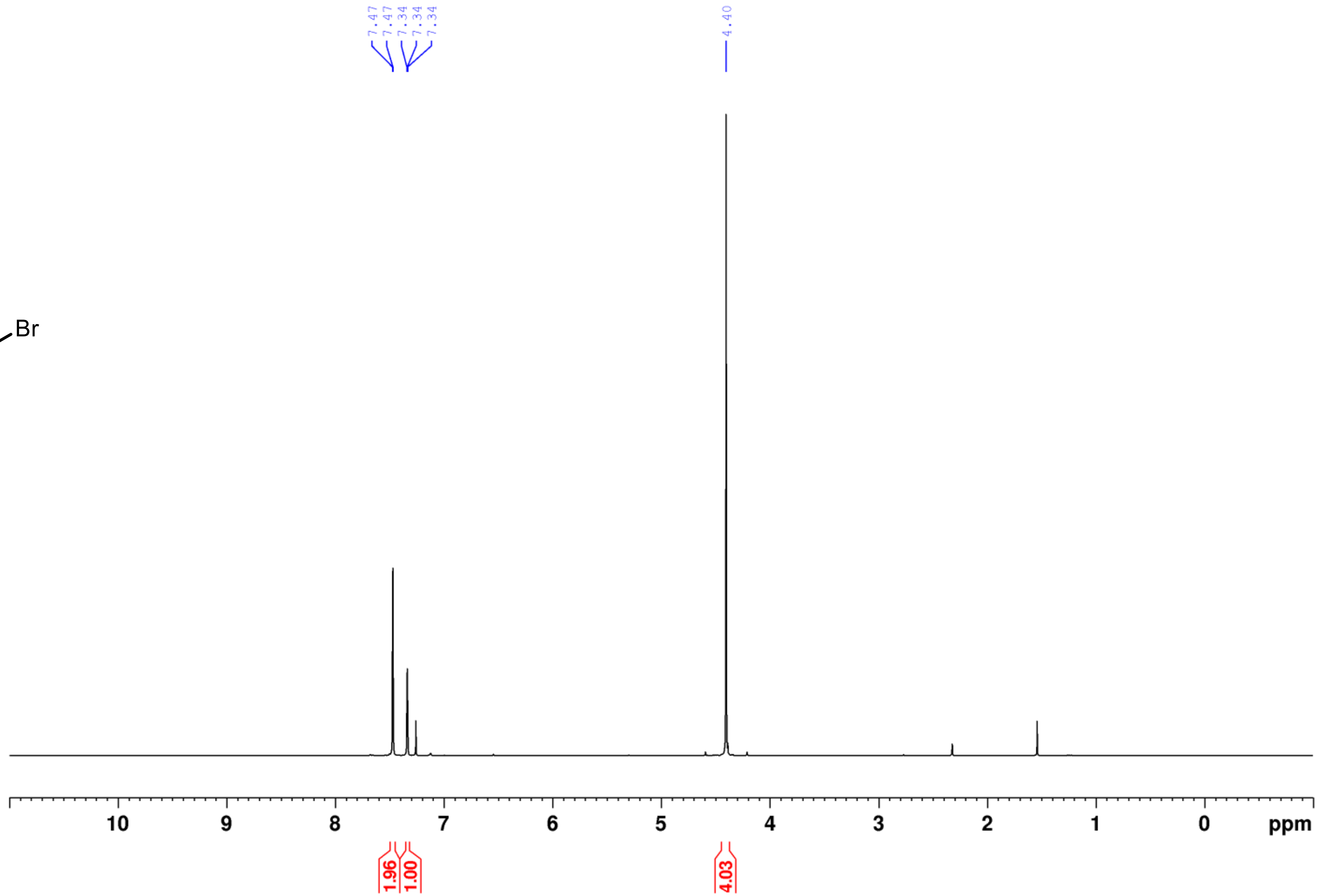

^{13}C NMR (101 MHz, $CDCl_3$) for *1-bromo-3,5-bis(bromomethyl)benzene*

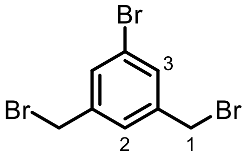

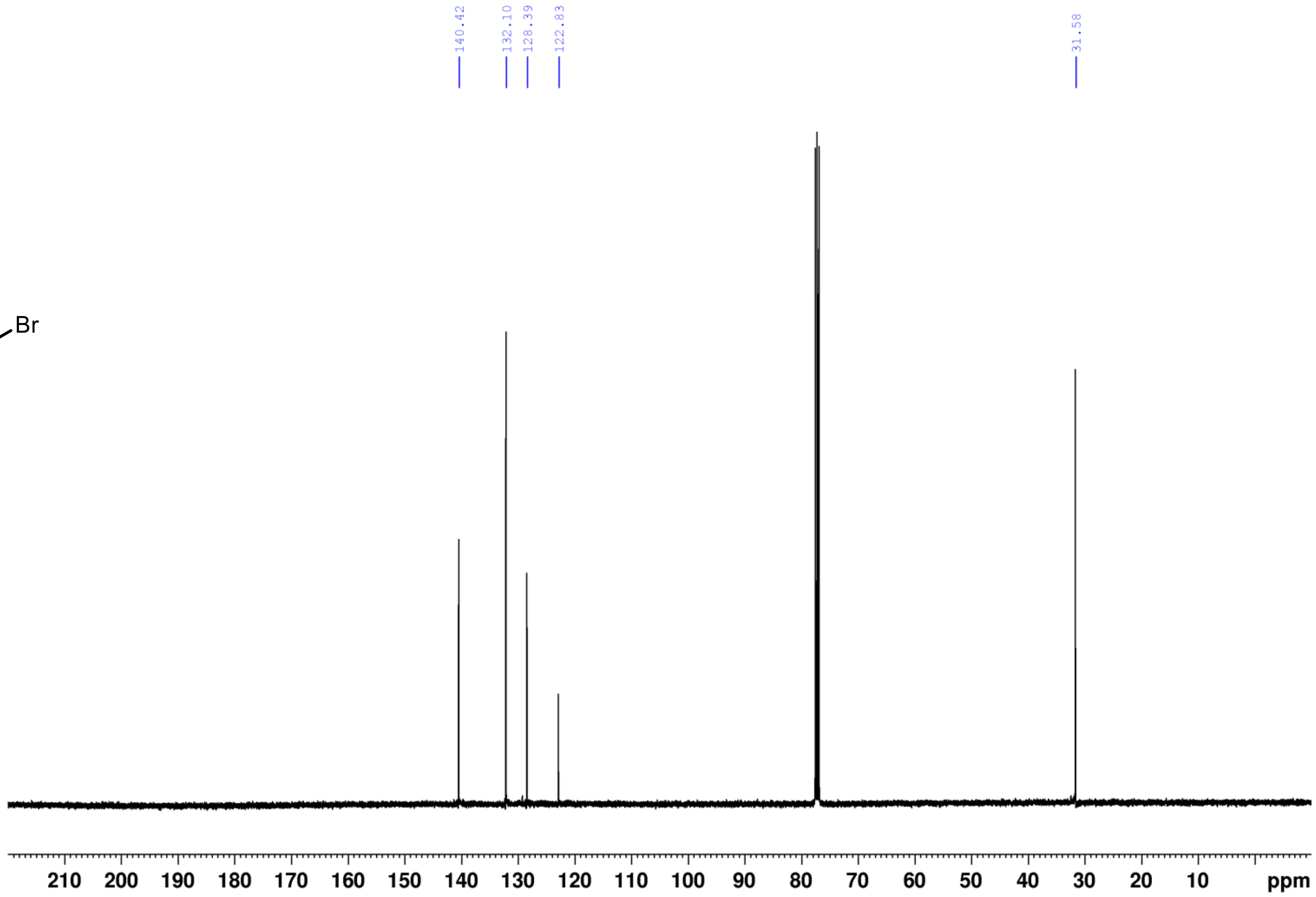

^{1}H NMR (400 MHz, $CDCl_3$) for *dimethyl 3,3'-(5-bromo-1,3-phenylene)bis(2,2-dimethylpropanoate)*

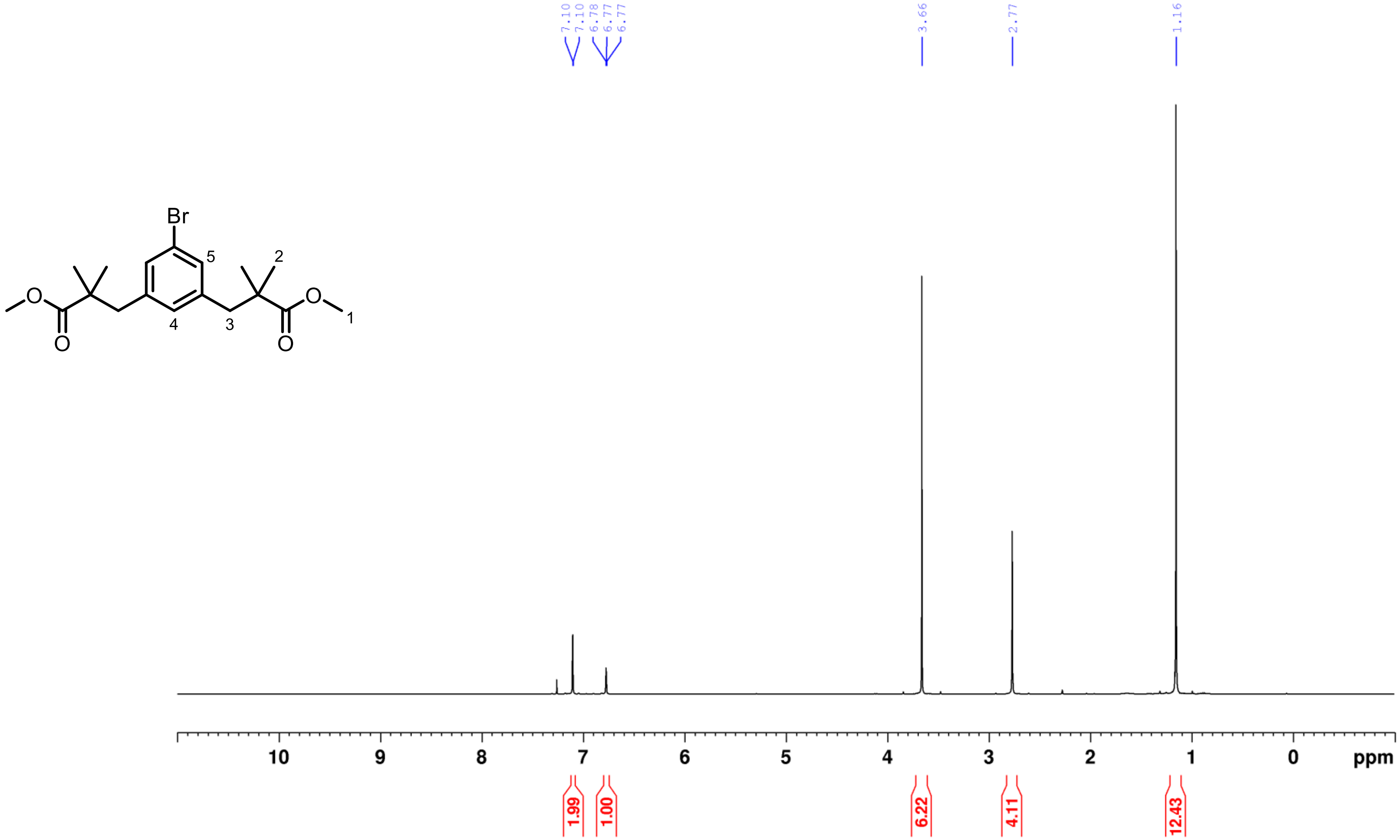

^{13}C NMR (101 MHz, $CDCl_3$) for *dimethyl 3,3'-(5-bromo-1,3-phenylene)bis(2,2-dimethylpropanoate)*

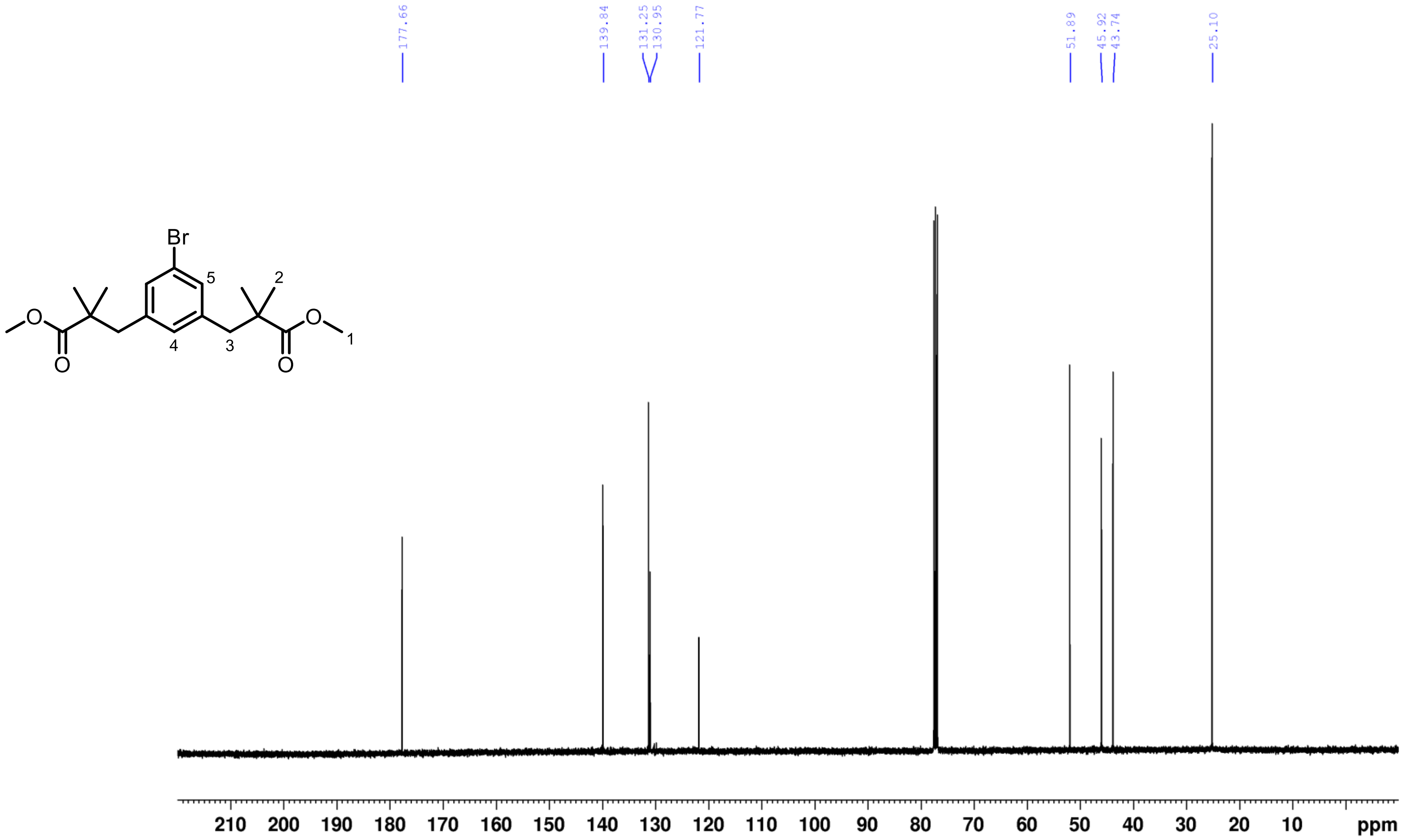

^{1}H NMR (500 MHz, CDCl$_3$) for *dimethyl 3,3'-(5-(4,4,5,5-tetramethyl-1,3,2-dioxaborolan-2-yl)-1,3-phenylene)bis(2,2-dimethylpropanoate)*

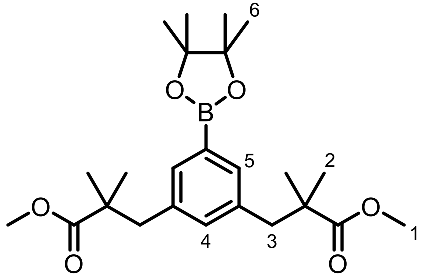

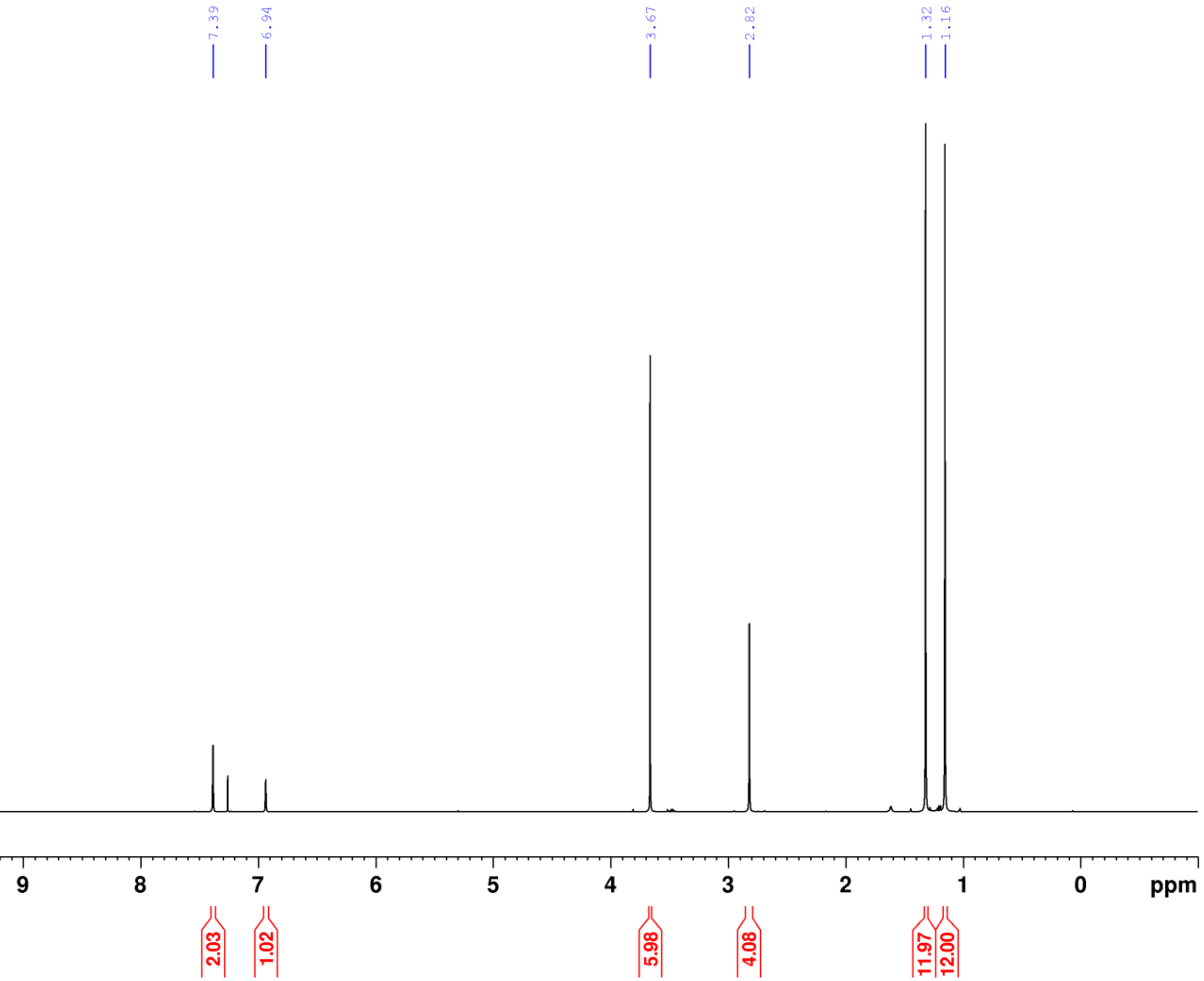

^{13}C NMR (126 MHz, $CDCl_3$) for *dimethyl 3,3'-(5-(4,4,5,5-tetramethyl-1,3,2-dioxaborolan-2-yl)-1,3-phenylene)bis(2,2-dimethylpropanoate)*

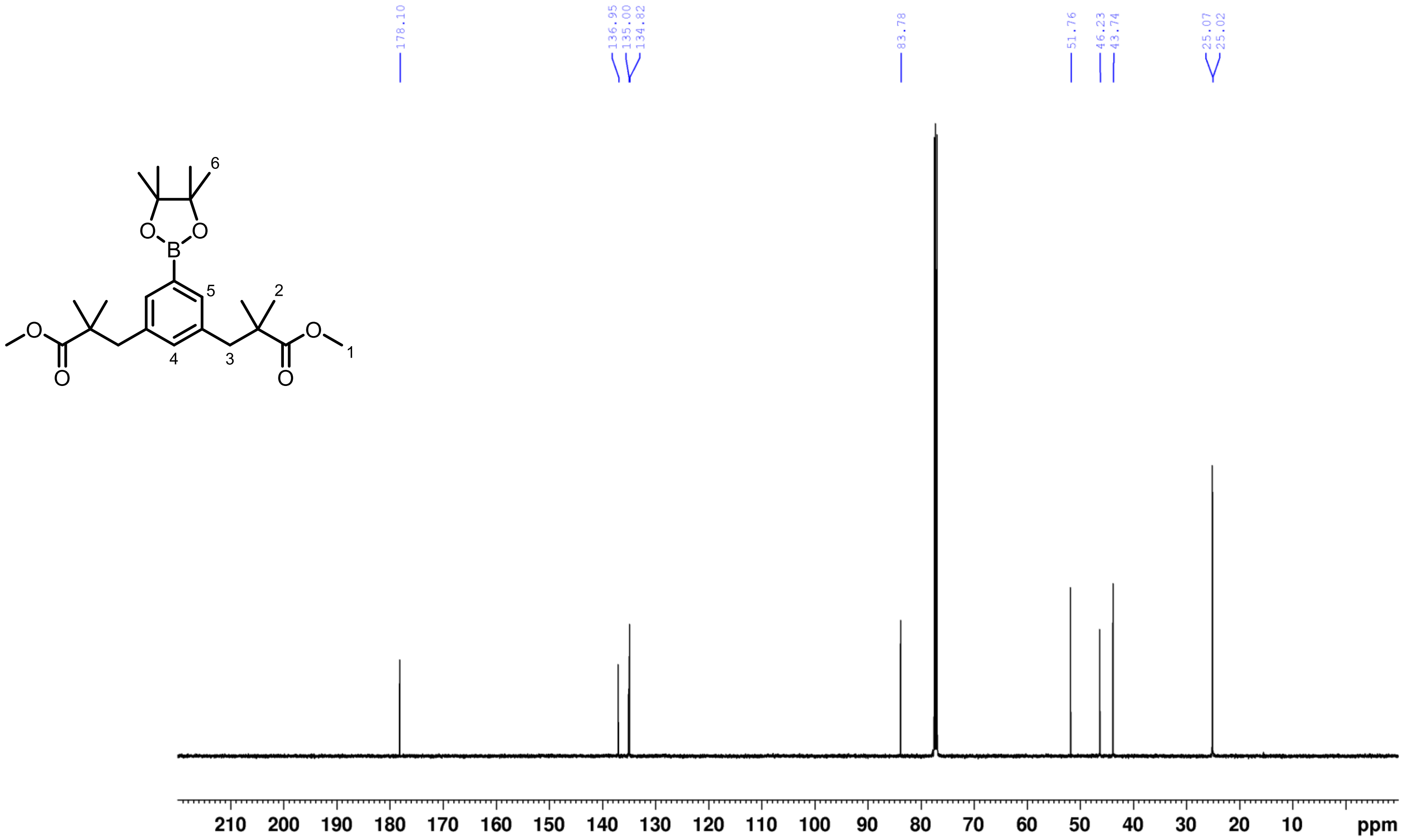

^{1}H NMR (400 MHz, $(CD_3)_2SO$) for *phenyl 2-bromobenzenesulfonate*

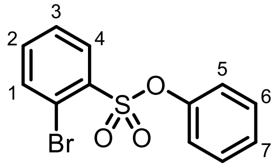

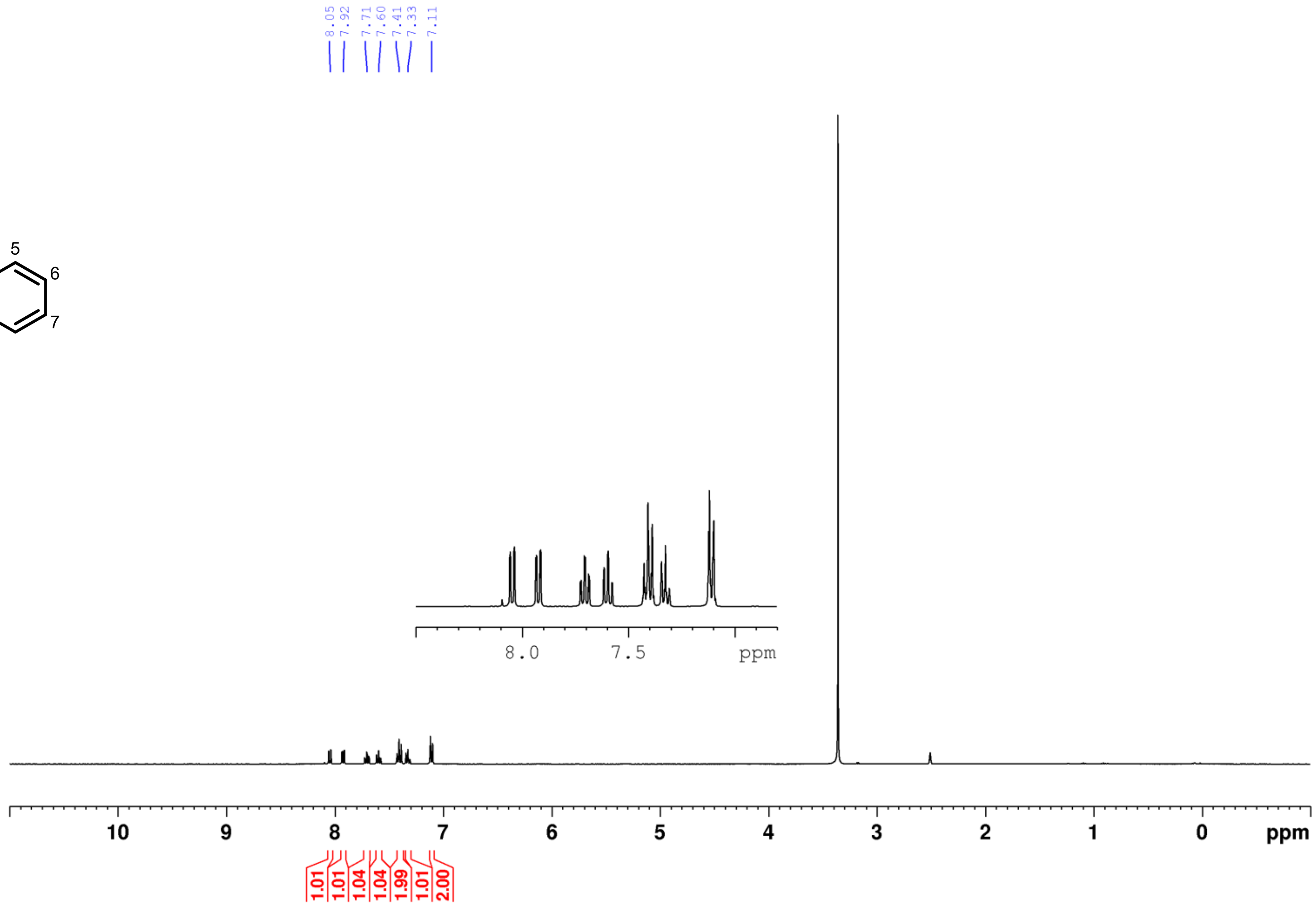

^{13}C NMR (101 MHz, $(CD_3)_2SO$) for *phenyl 2-bromobenzenesulfonate*

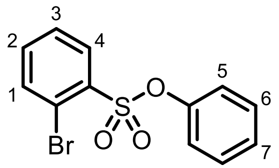

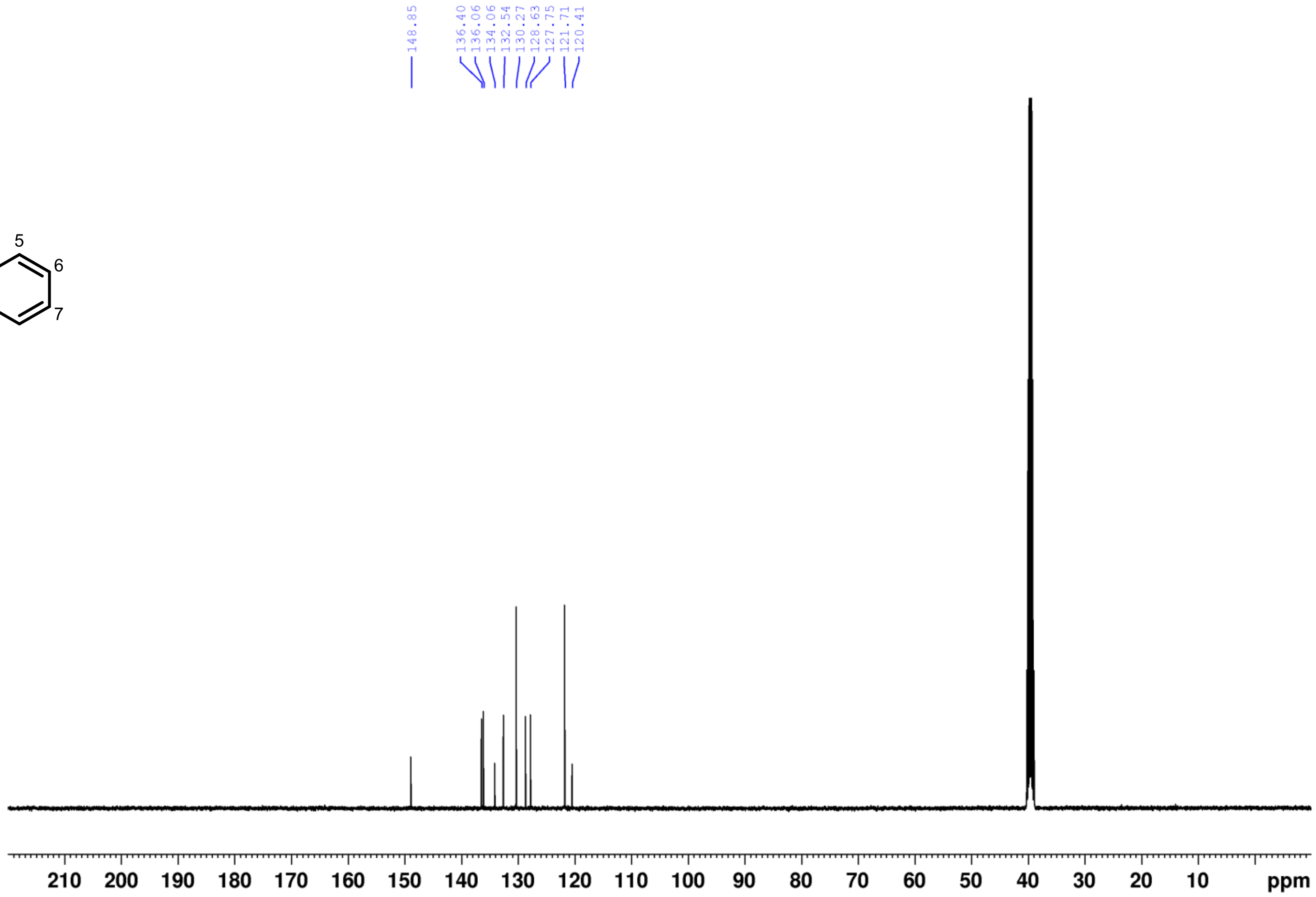

^{1}H NMR (400 MHz, $CDCl_3$) for *dimethyl 3,3'-(2'-(phenoxysulfonyl)-[1,1'-biphenyl]-3,5-diyl)bis(2,2-dimethylpropanoate)*

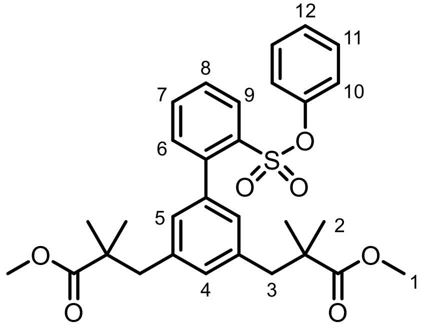

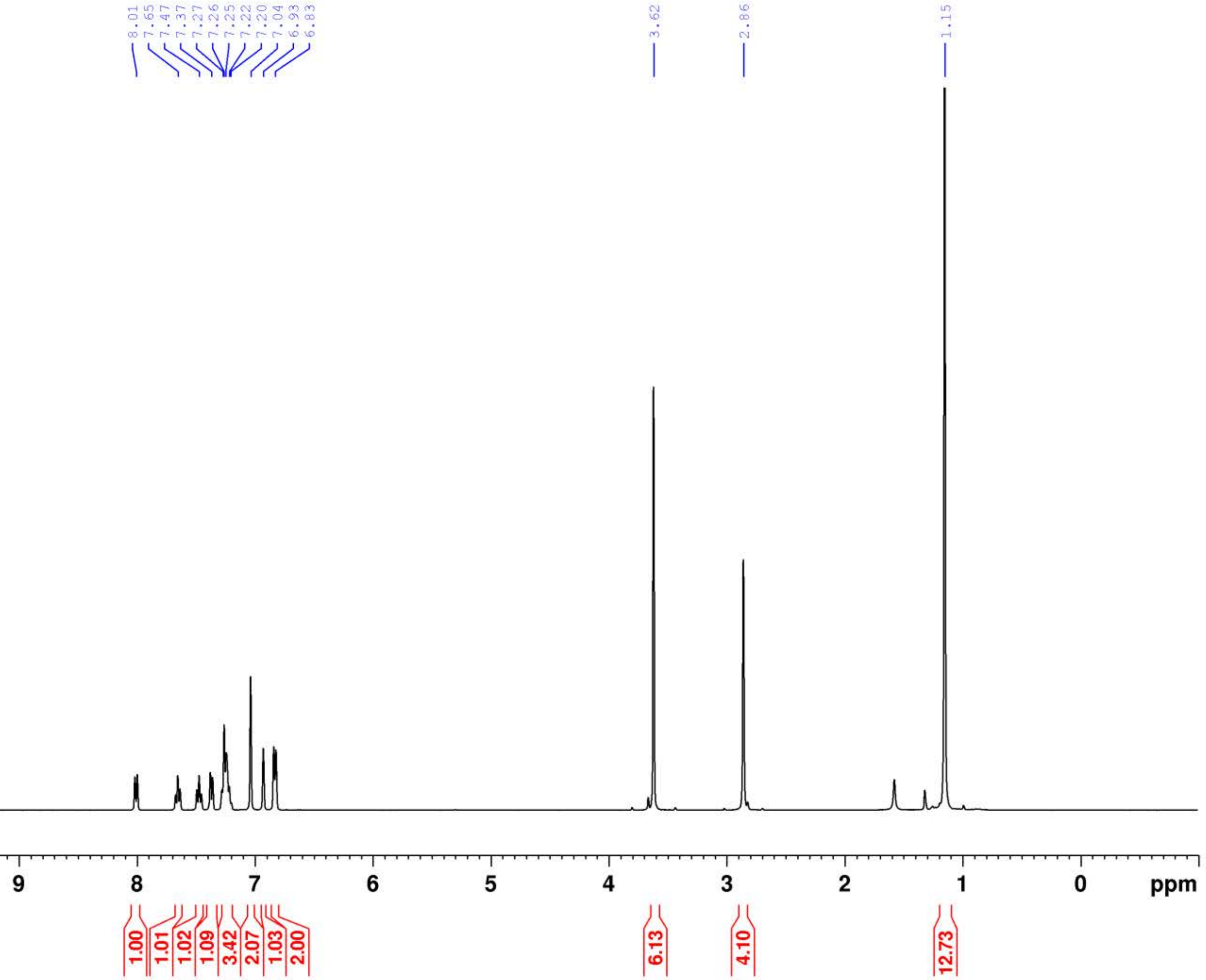

^{13}C NMR (101 MHz, $CDCl_3$) for *dimethyl 3,3'-(2'-(phenoxysulfonyl)-[1,1'-biphenyl]-3,5-diyl)bis(2,2-dimethylpropanoate)*

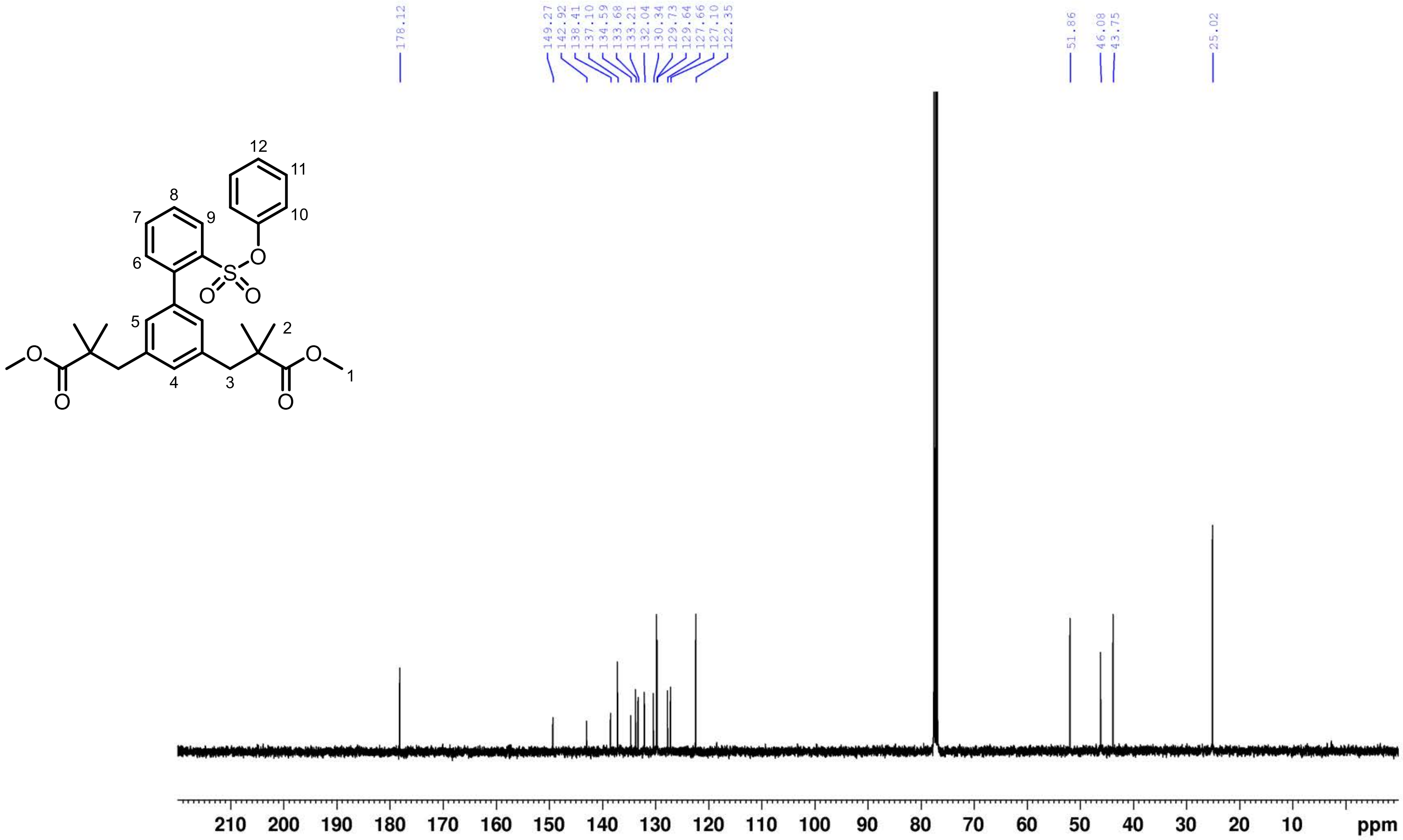

^{1}H NMR (400 MHz, CD_3OD) for *tetrabutylammonium 3',5'-bis(2-carboxy-2-methylpropyl)-[1,1'-biphenyl]- 2-sulfonate*

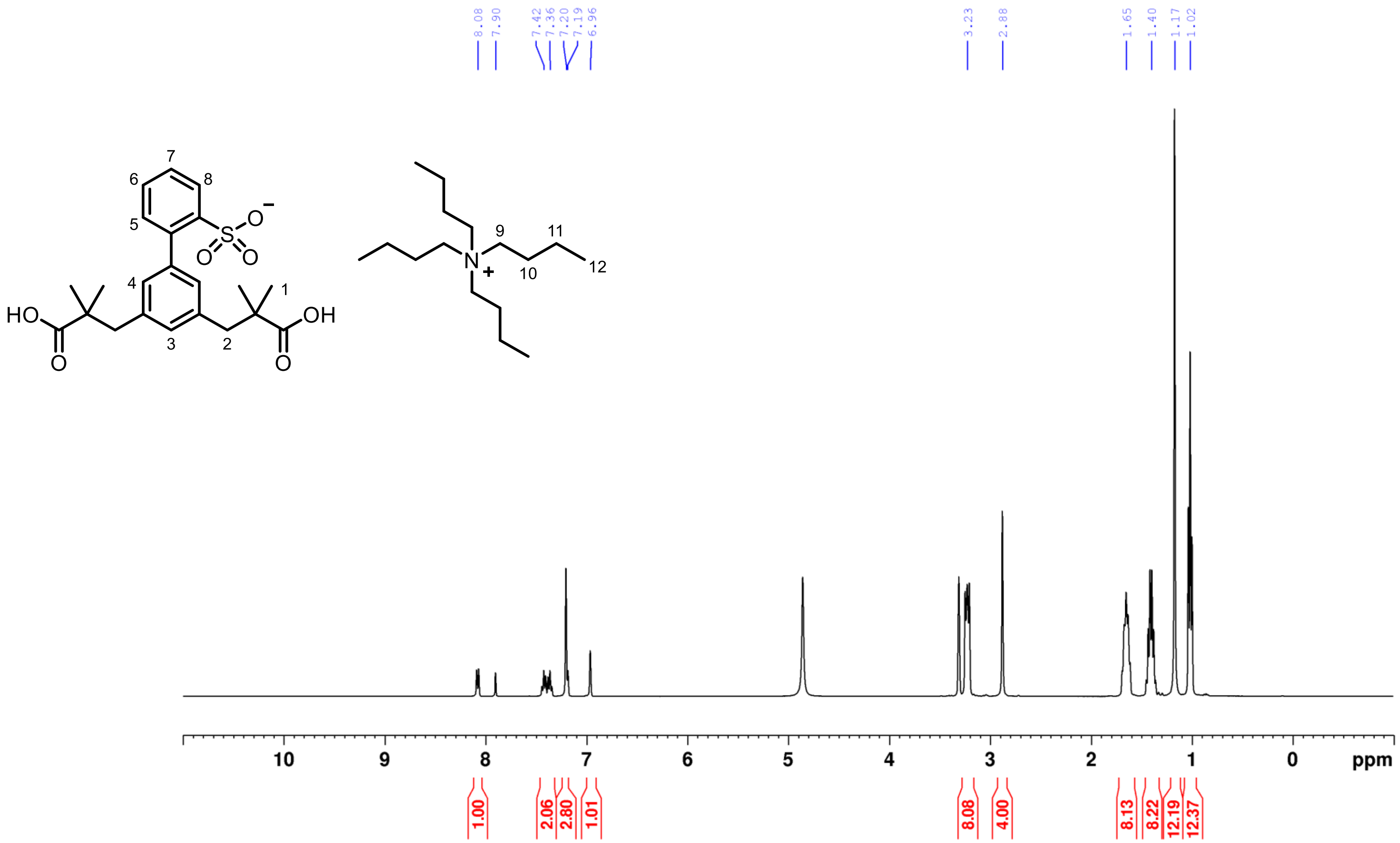

^{13}C NMR (101 MHz, CD_3OD) for *tetrabutylammonium 3',5'-bis(2-carboxy-2-methylpropyl)-[1,1'-biphenyl]-2-sulfonate*

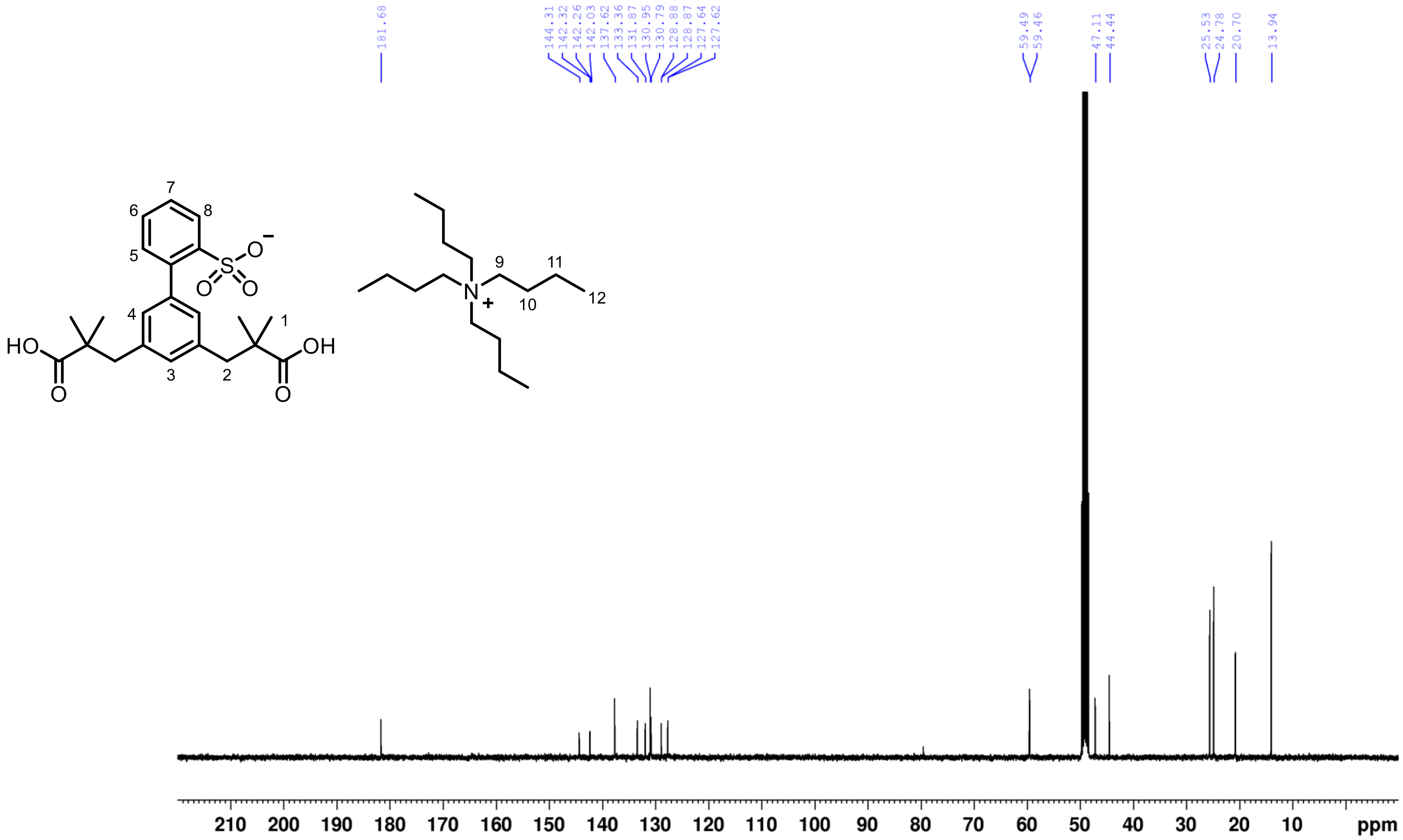

^{1}H NMR (500 MHz, CD_3OD) for *bis[rhodium tetrabutylammonium (3',5'-bis(2-carboxy-2-methylpropyl)-[1,1'-biphenyl]-2-sulfonate)]* (Rh_2(**A-III**)$_2$●(NBu_4)$_2$)

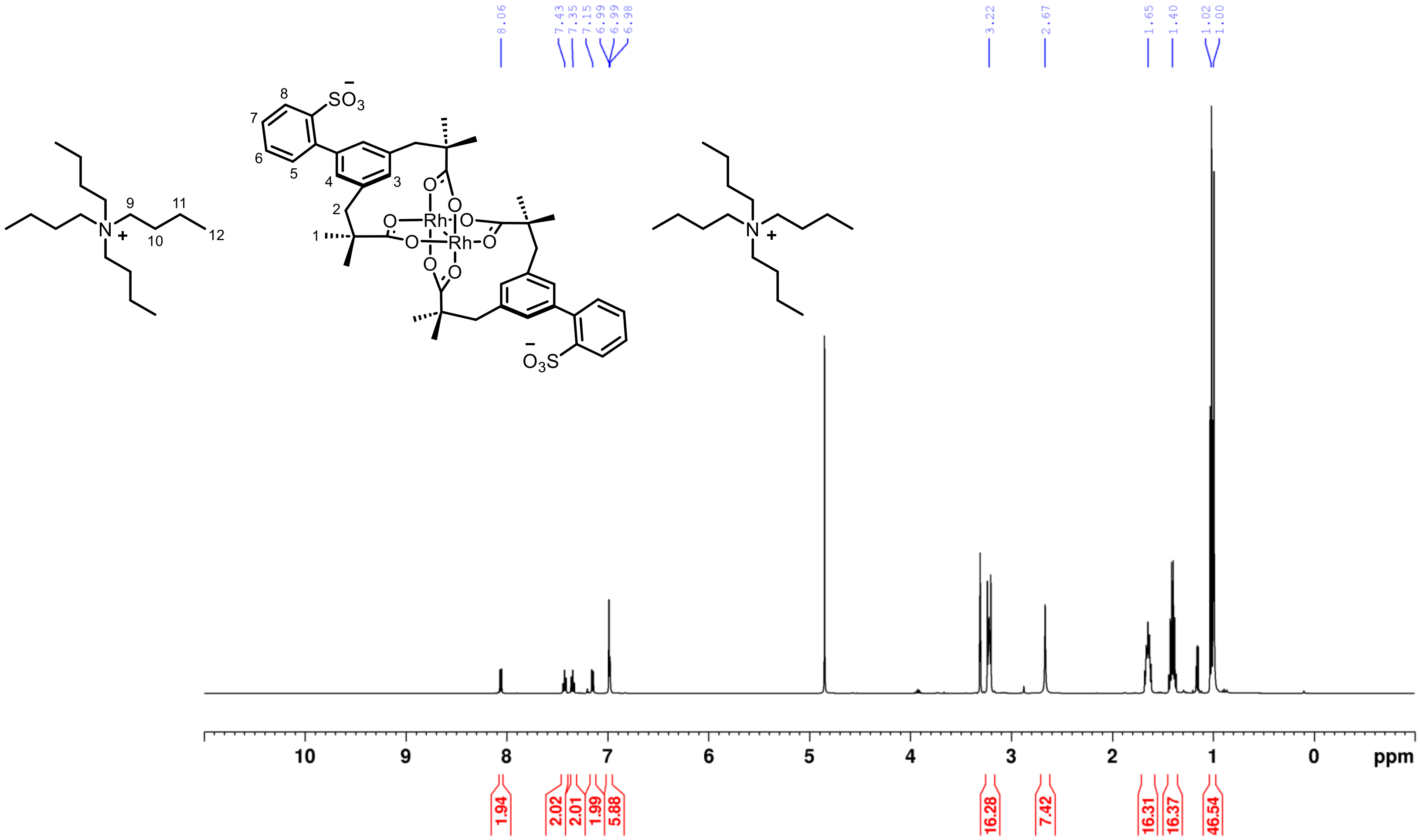

^{13}C NMR (126 MHz, CD_3OD) for *bis[rhodium tetrabutylammonium (3',5'-bis(2-carboxy-2-methylpropyl)-[1,1'-biphenyl]-2-sulfonate)]* (Rh_2(**A-III**)$_2$●(**NBu$_4$**)$_2$)

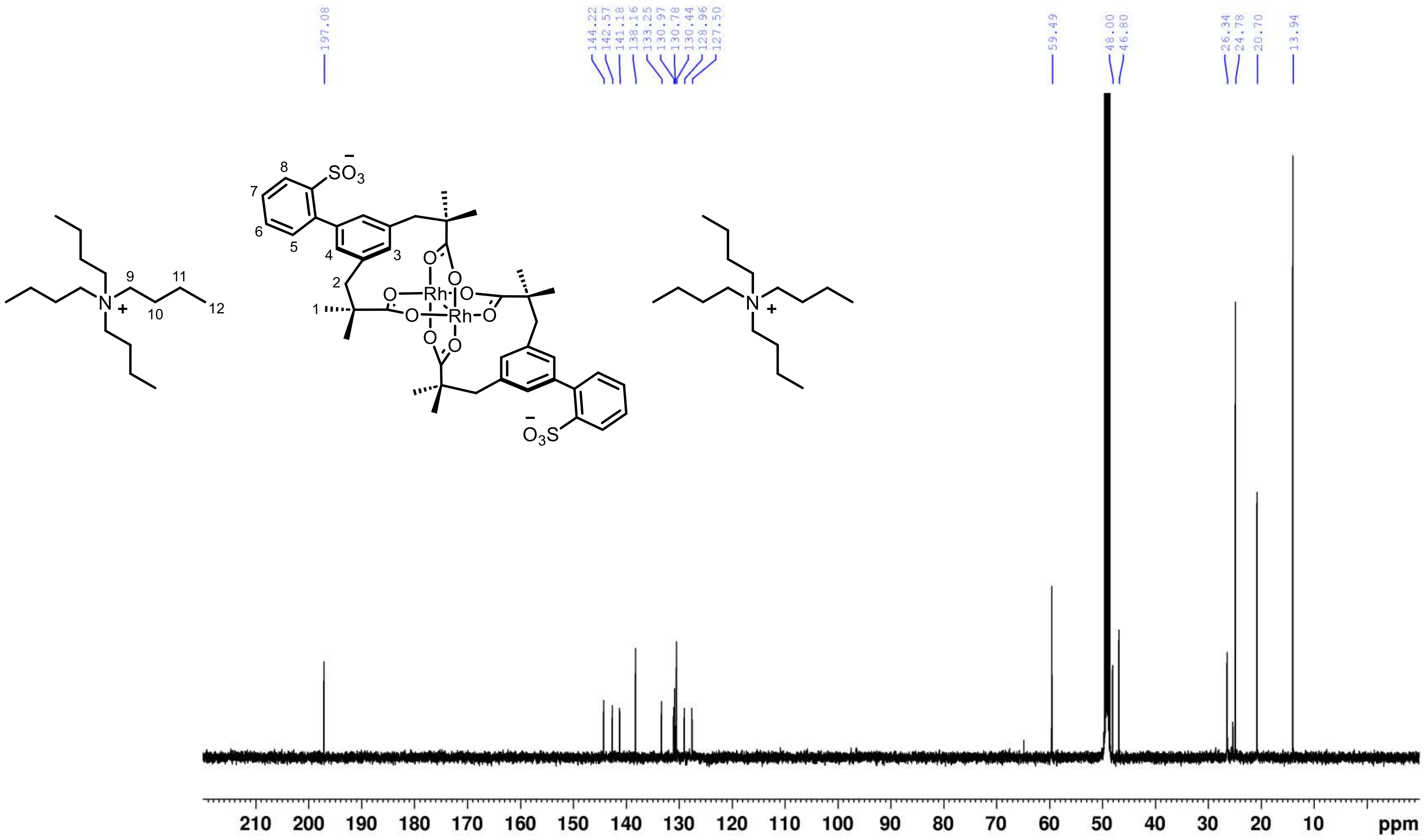

^{1}H NMR (400 MHz, $CDCl_3$) for *diethyl 1,1'-((5-bromo-1,3-phenylene)bis(methylene))bis(cyclobutane-1-carboxylate)*

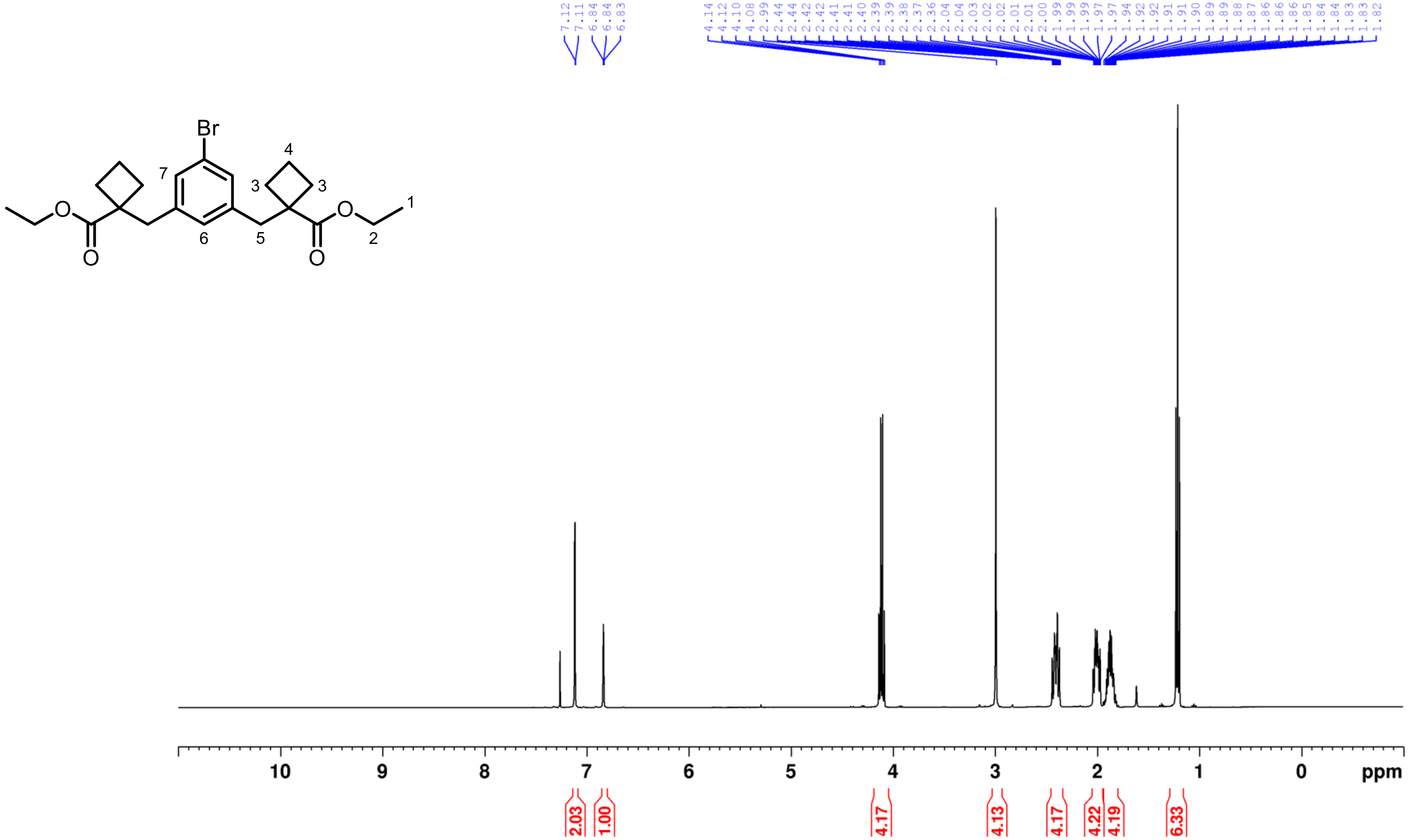

^{13}C NMR (101 MHz, CDCl$_3$) for *diethyl 1,1'-((5-bromo-1,3-phenylene)bis(methylene))bis(cyclobutane-1-carboxylate)*

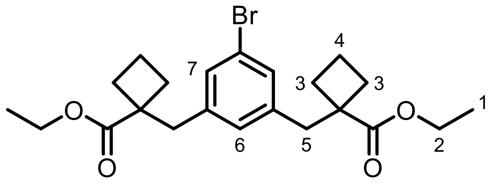

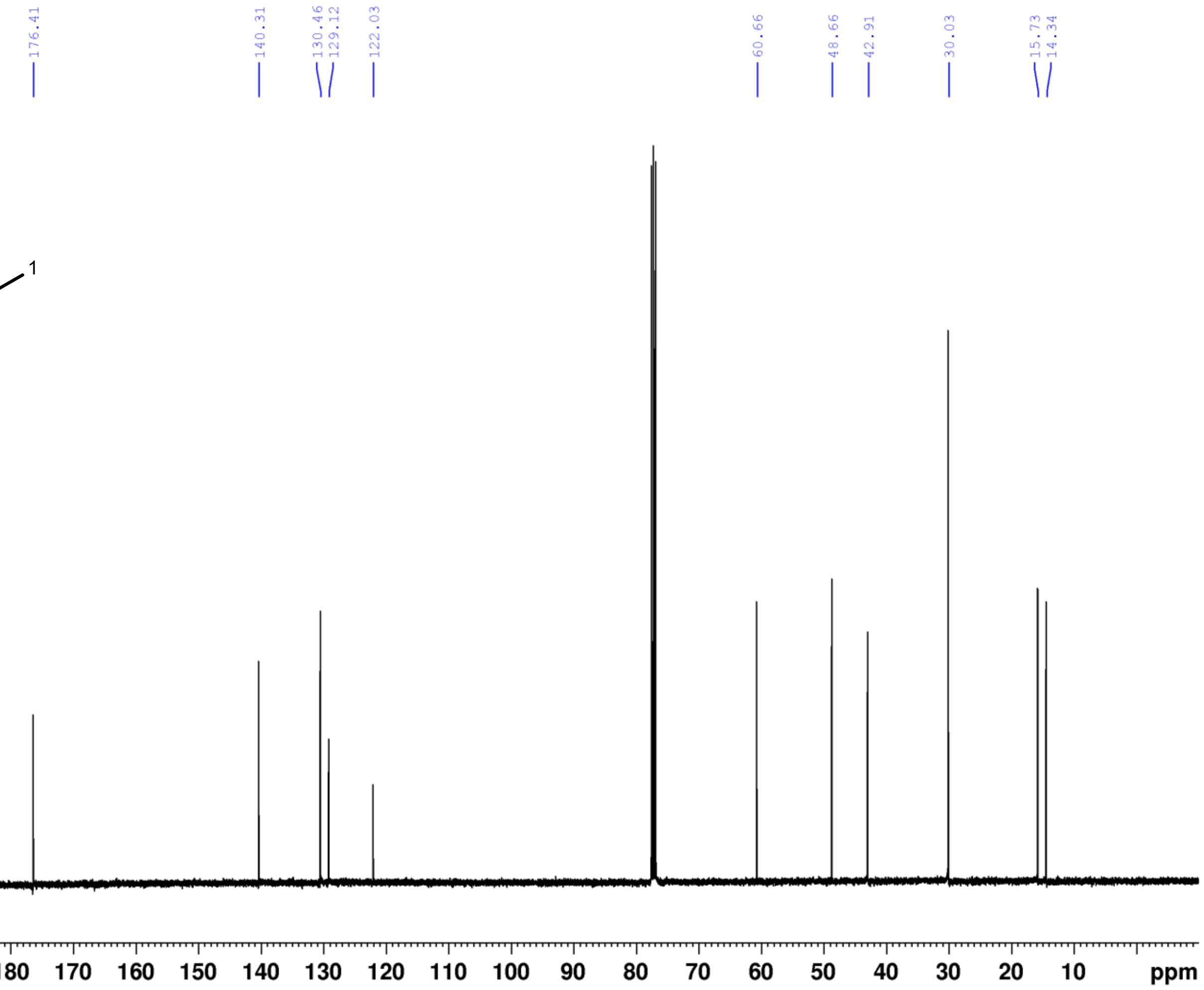

^{1}H NMR (400 MHz, CDCl$_3$) for *diethyl 1,1'-((5-(4,4,5,5-tetramethyl-1,3,2-dioxaborolan-2-yl)-1,3-phenylene)bis(methylene))bis(cyclobutane-1-carboxylate)*

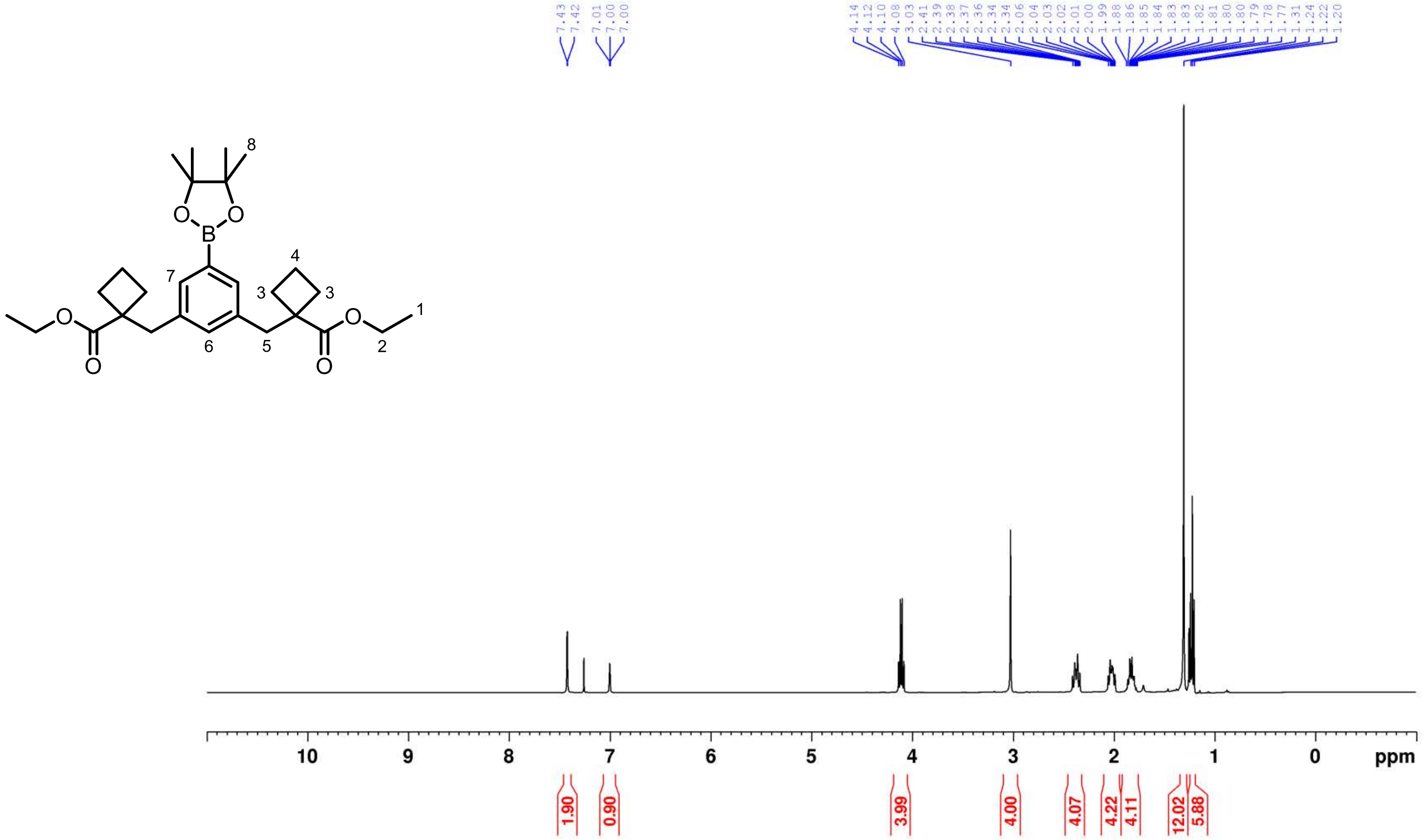

^{13}C NMR (101 MHz, CDCl$_3$) for *diethyl 1,1'-((5-(4,4,5,5-tetramethyl-1,3,2-dioxaborolan-2-yl)-1,3-phenylene)bis(methylene))bis(cyclobutane-1-carboxylate)*

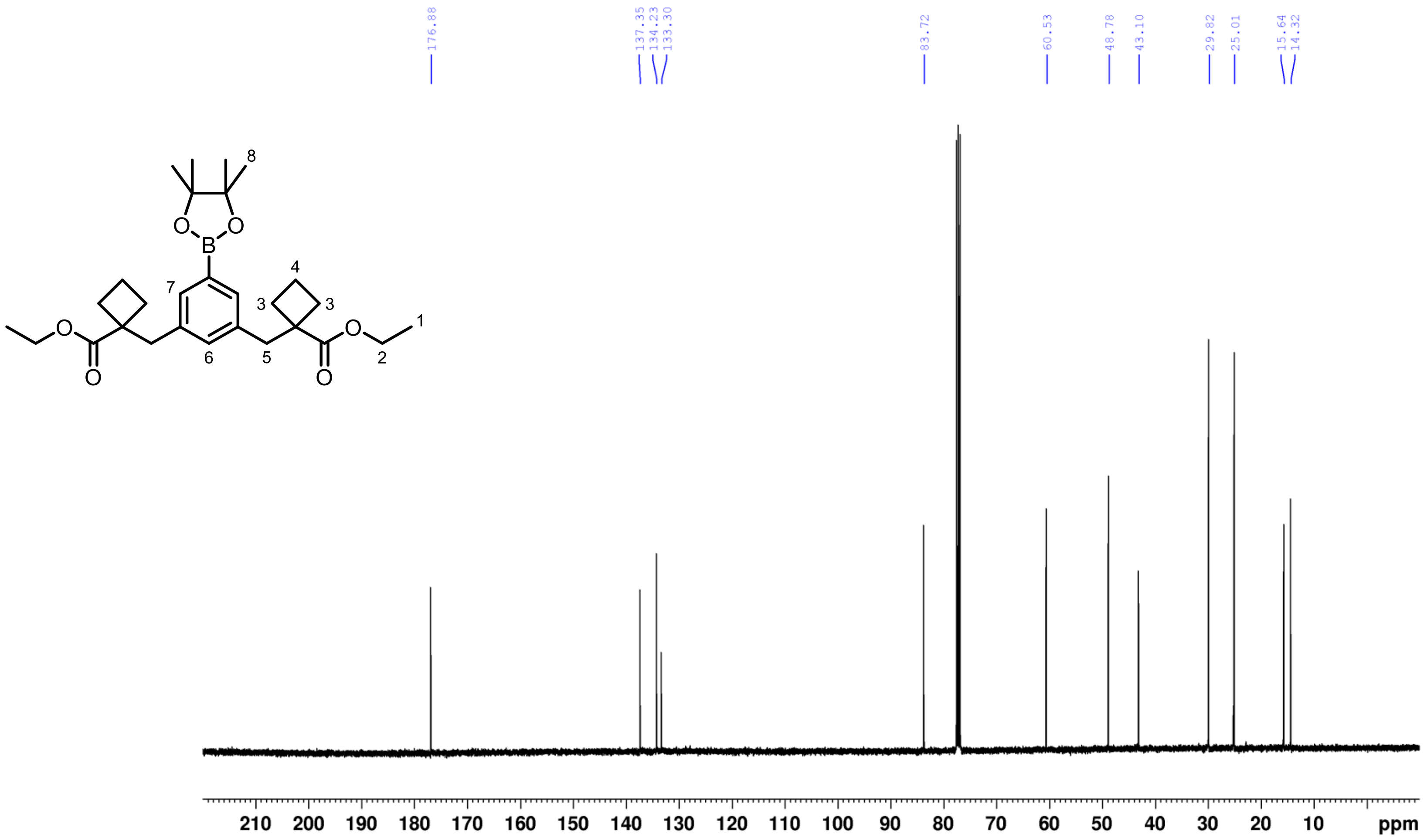

^{1}H NMR (500 MHz, $CDCl_3$) for *diethyl 1,1'-((2'-(phenoxysulfonyl)-[1,1'-biphenyl]-3,5-diyl)bis(methylene))bis(cyclobutane-1-carboxylate). Note: this compound contained traces of an aromatic impurity which is fully separated in the remaining steps in the synthesis of* Rh_2(**B-III**)$_2$●(**NBu_4**)$_2$.

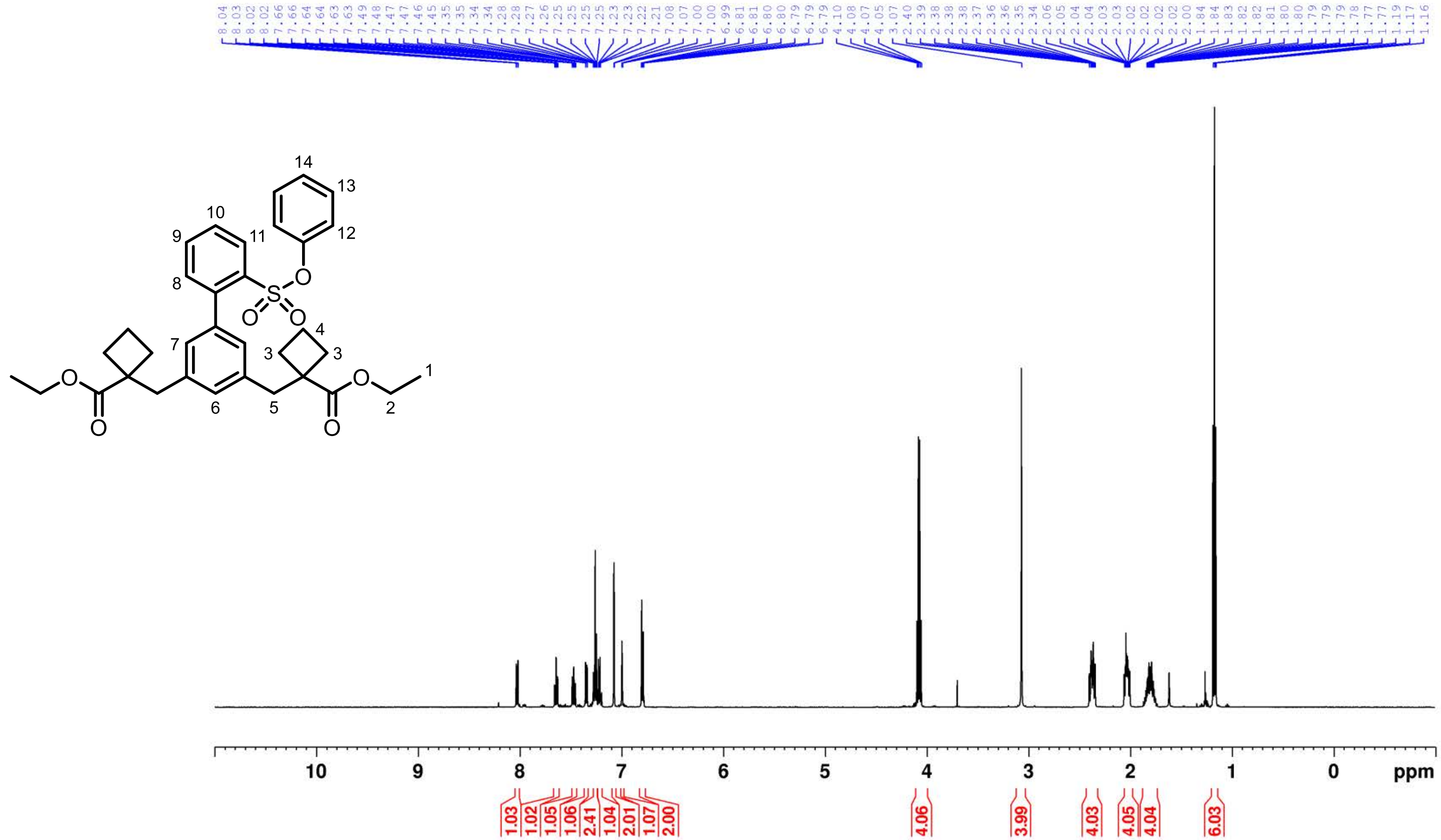

^{13}C NMR (126 MHz, $CDCl_3$) for *diethyl 1,1'-((2'-(phenoxysulfonyl)-[1,1'-biphenyl]-3,5-diyl)bis(methylene))bis(cyclobutane-1-carboxylate). Note: this compound contained traces of an aromatic impurity which is fully separated in the remaining steps in the synthesis of* Rh_2(**B-III**)$_2$●(**NBu_4**)$_2$.

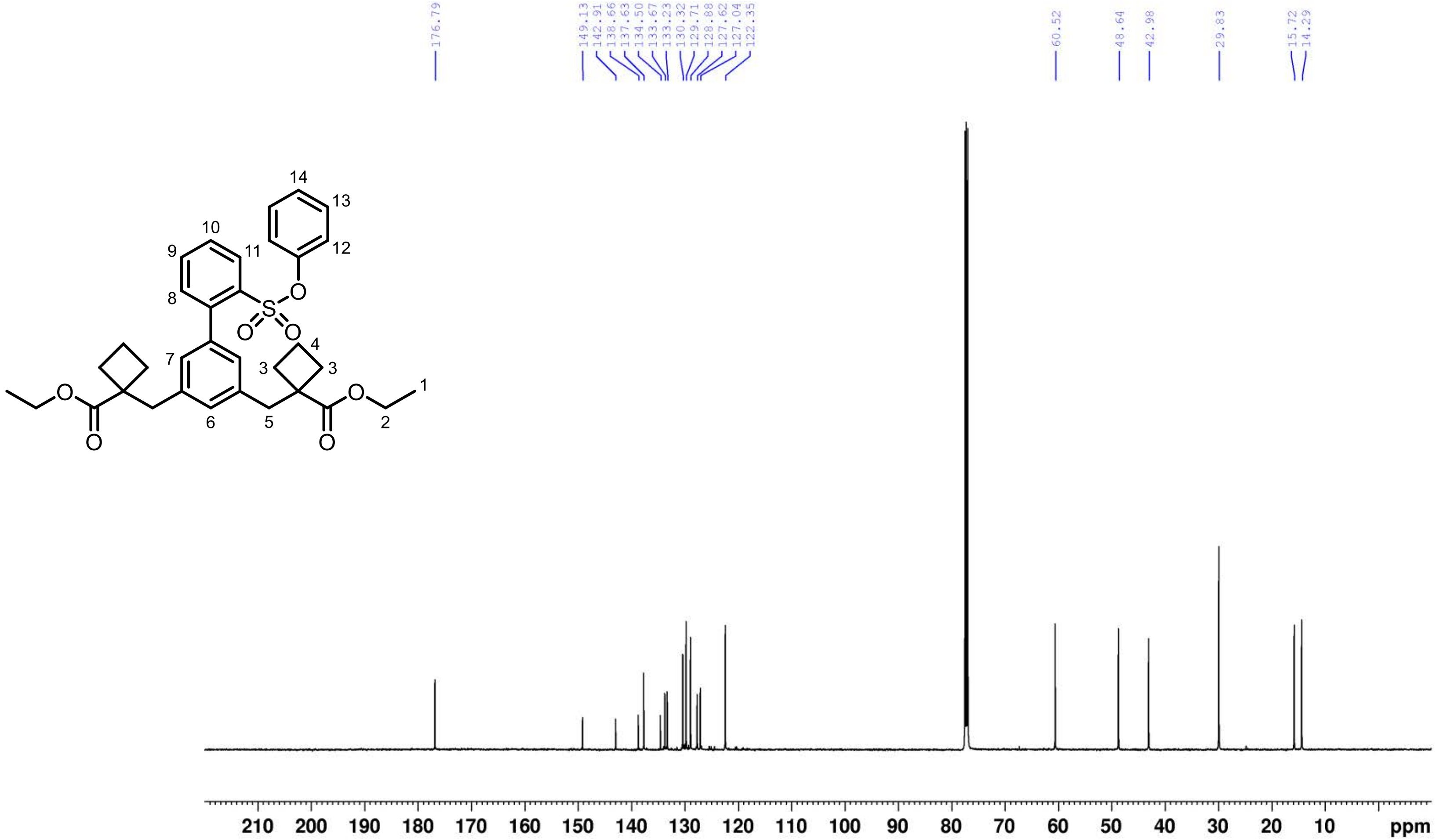

^{1}H NMR (400 MHz, CD_3OD) for *tetrabutylammonium 3',5'-bis((1-carboxycyclobutyl)methyl)-[1,1'-biphenyl]-2-sulfonate*

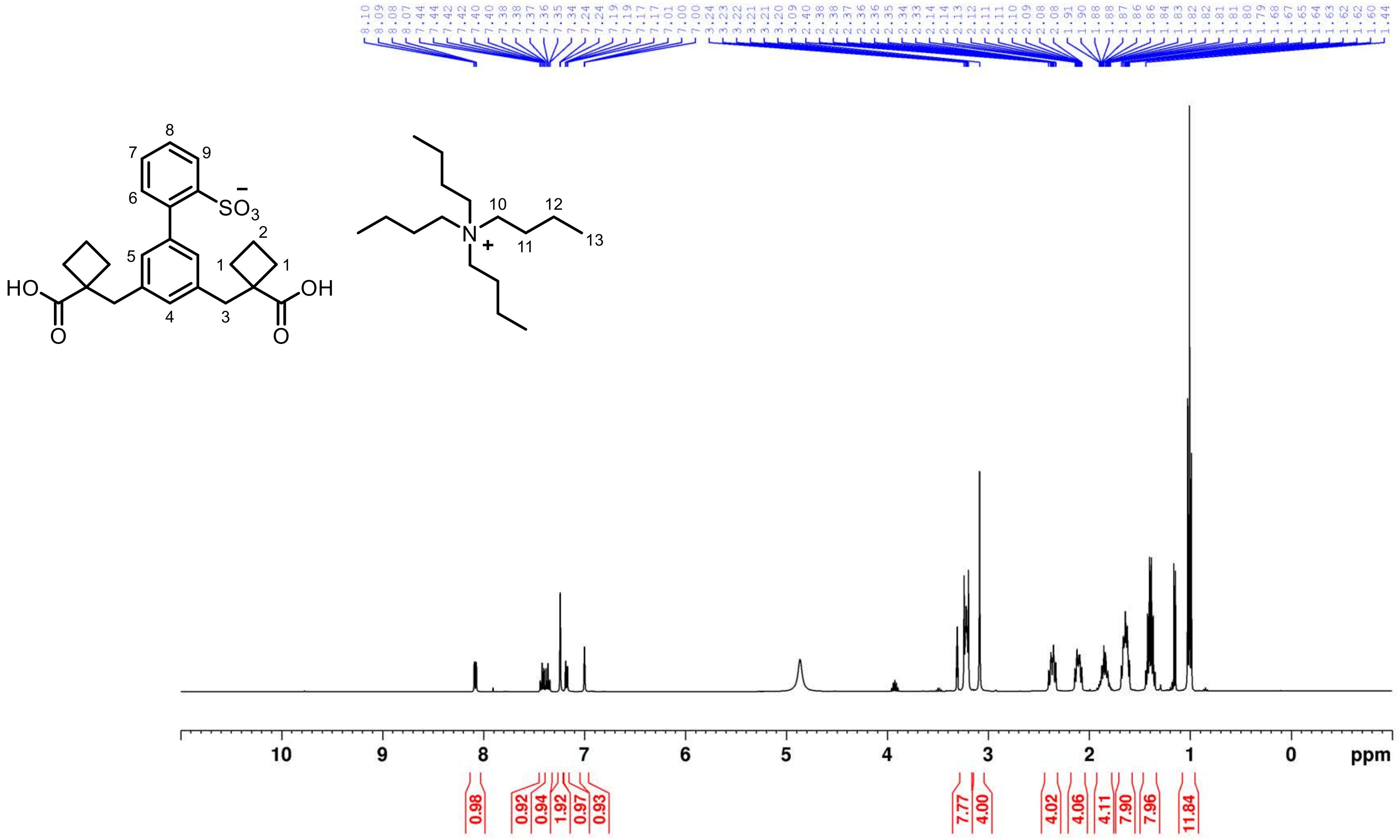

^{13}C NMR (101 MHz, CD_3OD) for *tetrabutylammonium 3',5'-bis((1-carboxycyclobutyl)methyl)-[1,1'-biphenyl]-2-sulfonate*

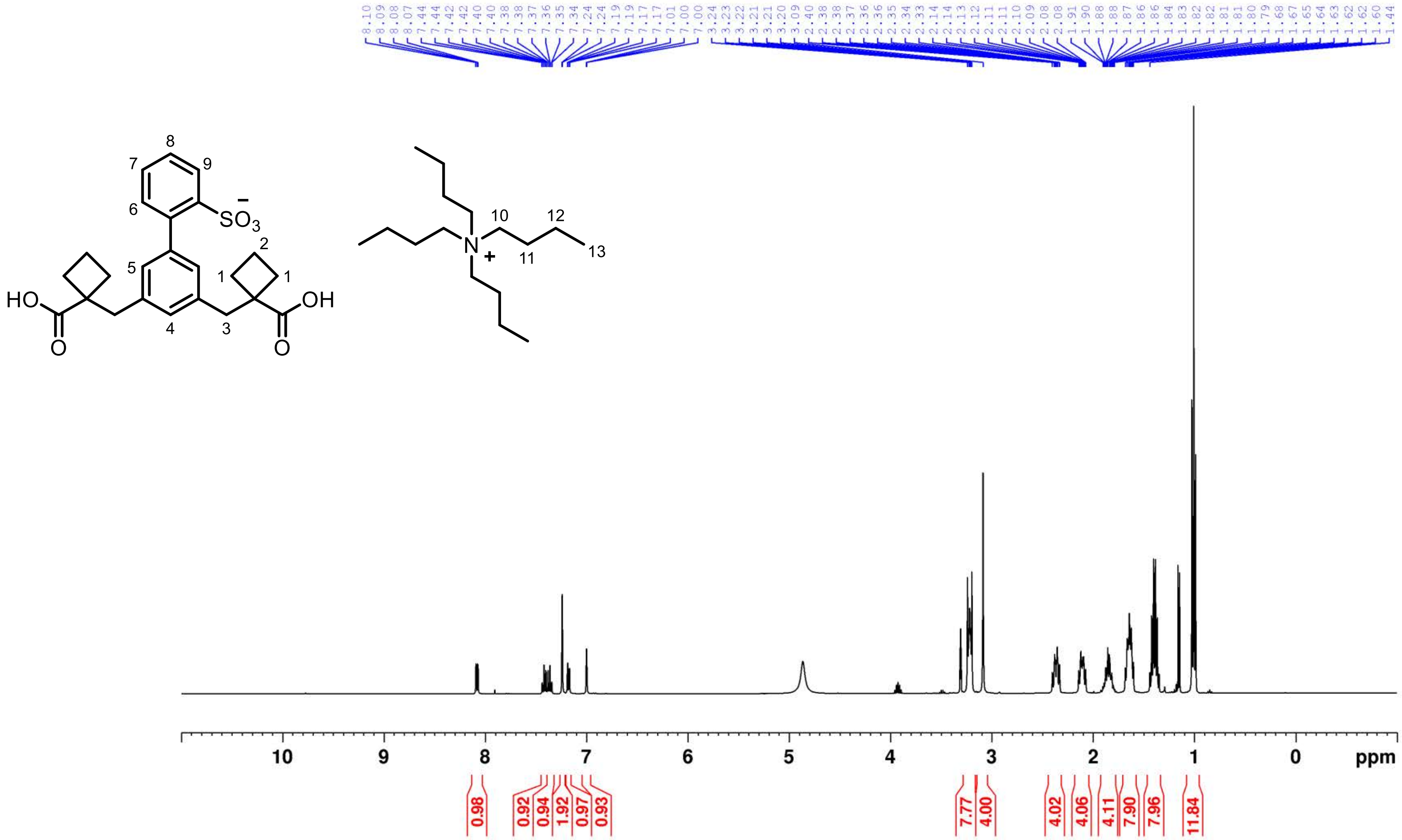

^{1}H NMR (500 MHz, CD_3OD) for *bis[rhodium tetrabutylammonium 3',5'-bis((1-carboxycyclobutyl)methyl)-[1,1'-biphenyl]-2-sulfonate]* (Rh_2(**B-III**)$_2$●(**NBu_4**)$_2$)

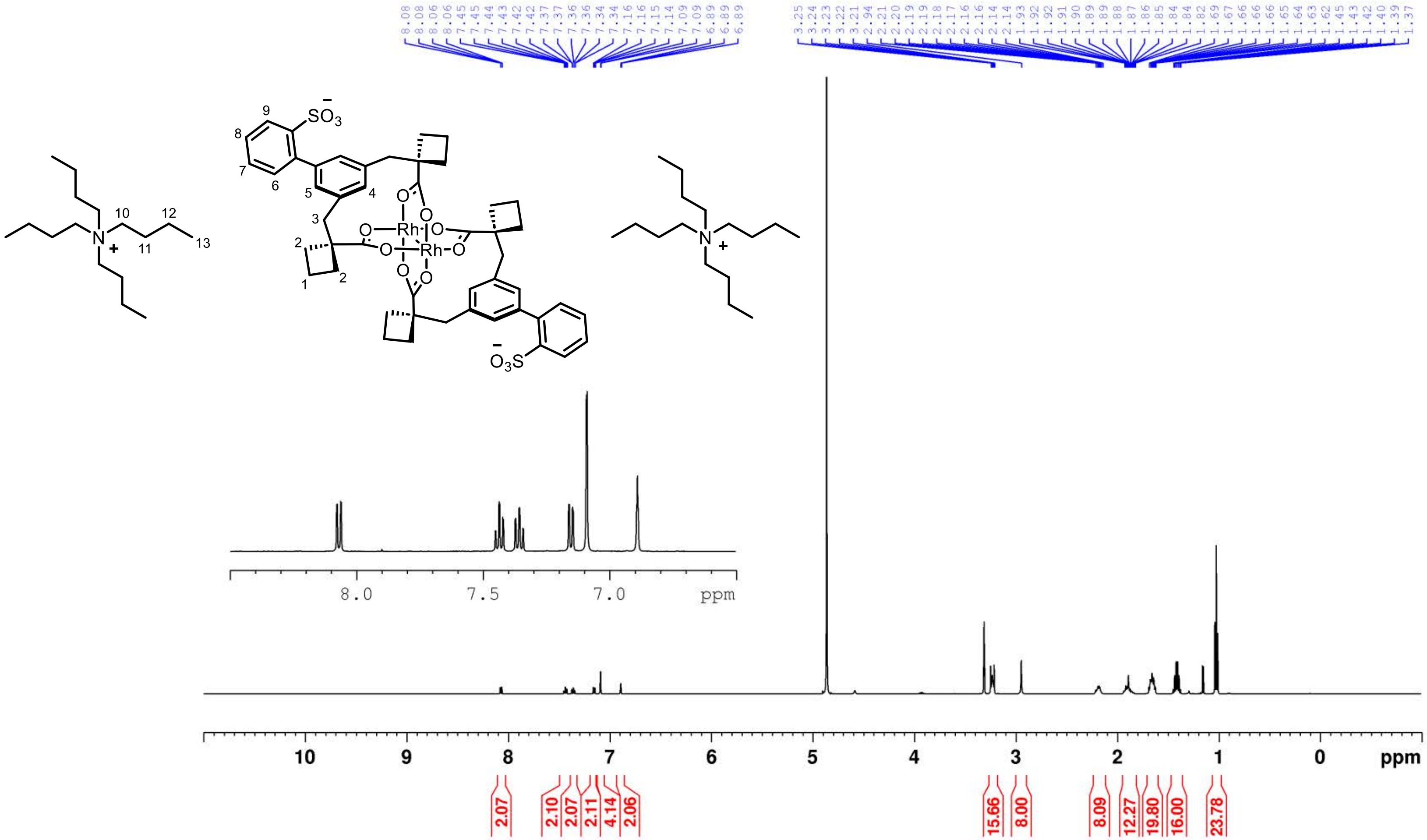

^{13}C NMR (126 MHz, CD_3OD) for *bis[rhodium tetrabutylammonium 3',5'-bis((1-carboxycyclobutyl)methyl)-[1,1'-biphenyl]-2-sulfonate]* (Rh_2(**B-III**)$_2$●(NBu_4)$_2$)

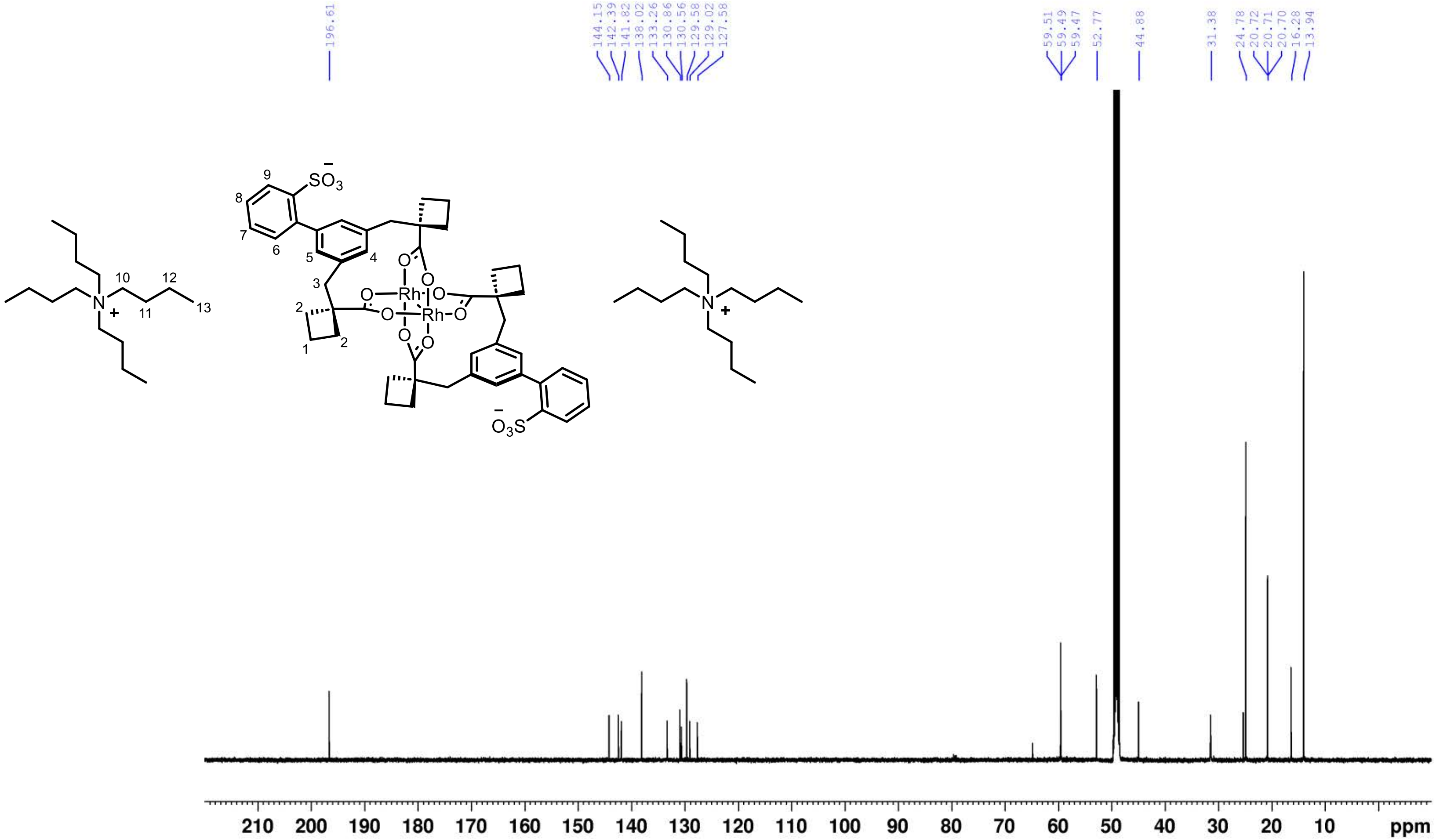

^{1}H NMR (400 MHz, CDCl$_3$) for *dimethyl 1,1'-((5-bromo-1,3-phenylene)bis(methylene))bis(cyclopentane-1-carboxylate)*

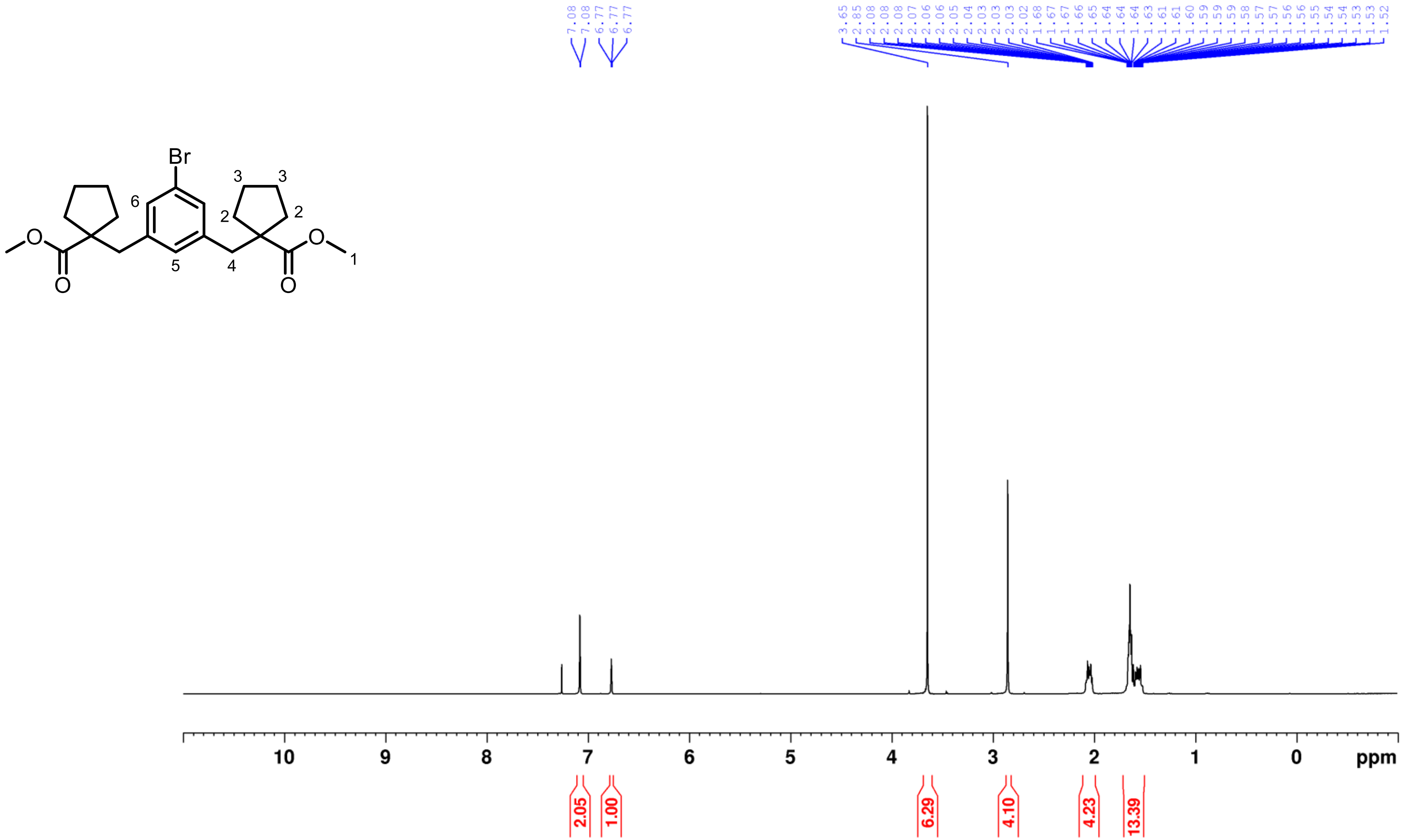

^{13}C NMR (101 MHz, $CDCl_3$) for *dimethyl 1,1'-((5-bromo-1,3-phenylene)bis(methylene))bis(cyclopentane-1-carboxylate)*

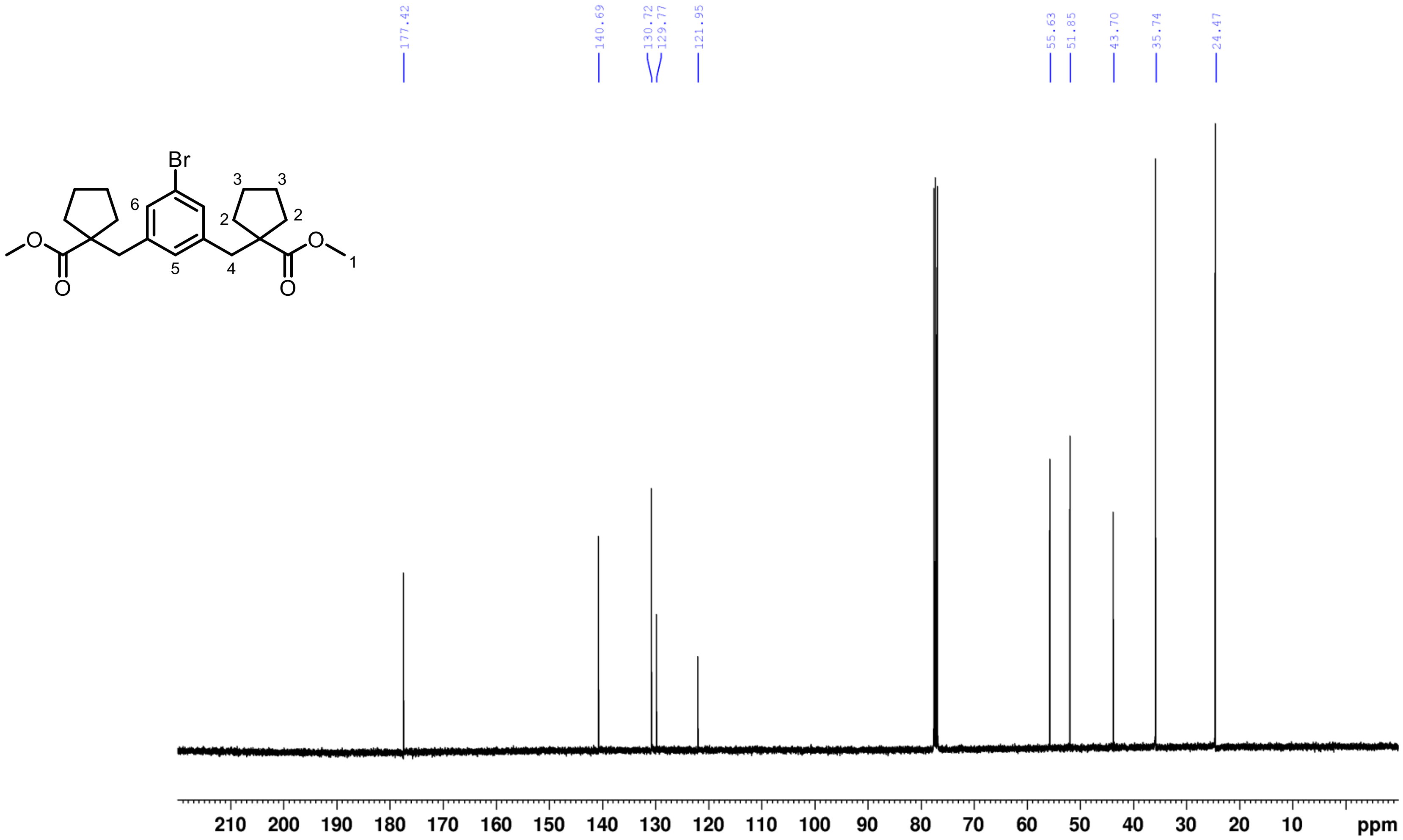

^{1}H NMR (400 MHz, $CDCl_3$) for *dimethyl 1,1'-((5-(4,4,5,5-tetramethyl-1,3,2-dioxaborolan-2-yl)-1,3-phenylene)bis(methylene))bis(cyclopentane-1-carboxylate)*

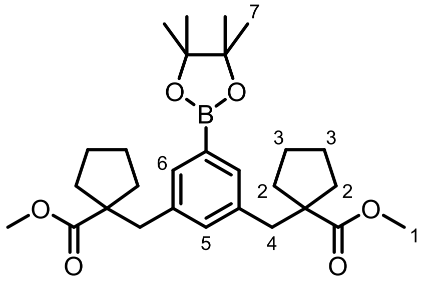

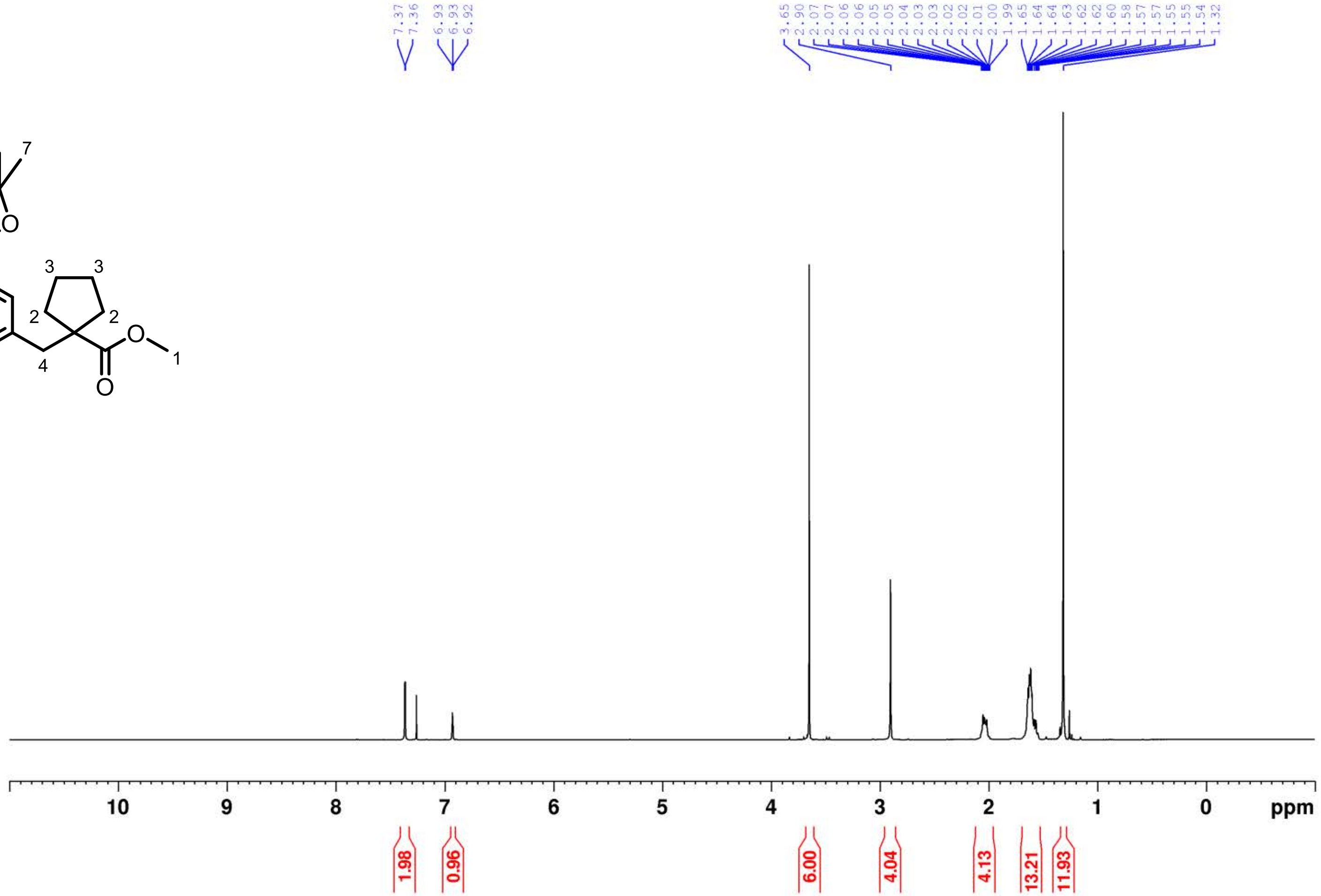

^{13}C NMR (101 MHz, CDCl$_3$) for *dimethyl 1,1'-((5-(4,4,5,5-tetramethyl-1,3,2-dioxaborolan-2-yl)-1,3-phenylene)bis(methylene))bis(cyclopentane-1-carboxylate)*

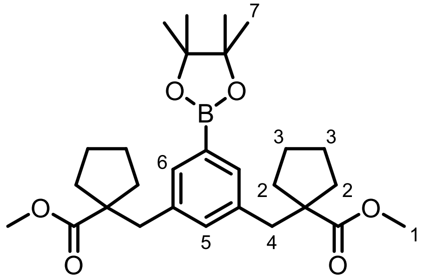

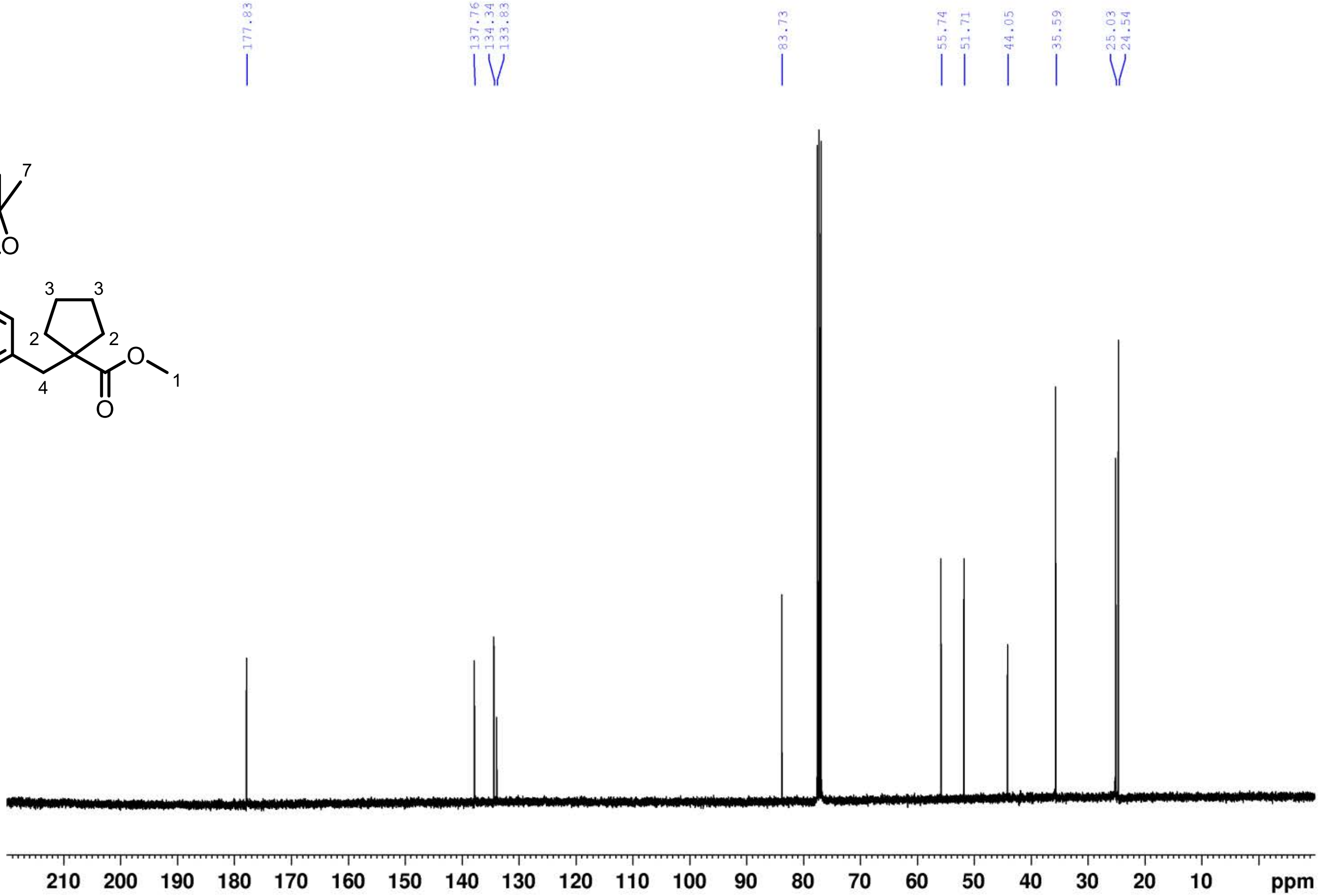

^{1}H NMR (400 MHz, CD_3OD) for *tetrabutylammonium 3',5'-bis((1-carboxycyclopentyl)methyl)-[1,1'-biphenyl]-2-sulfonate*

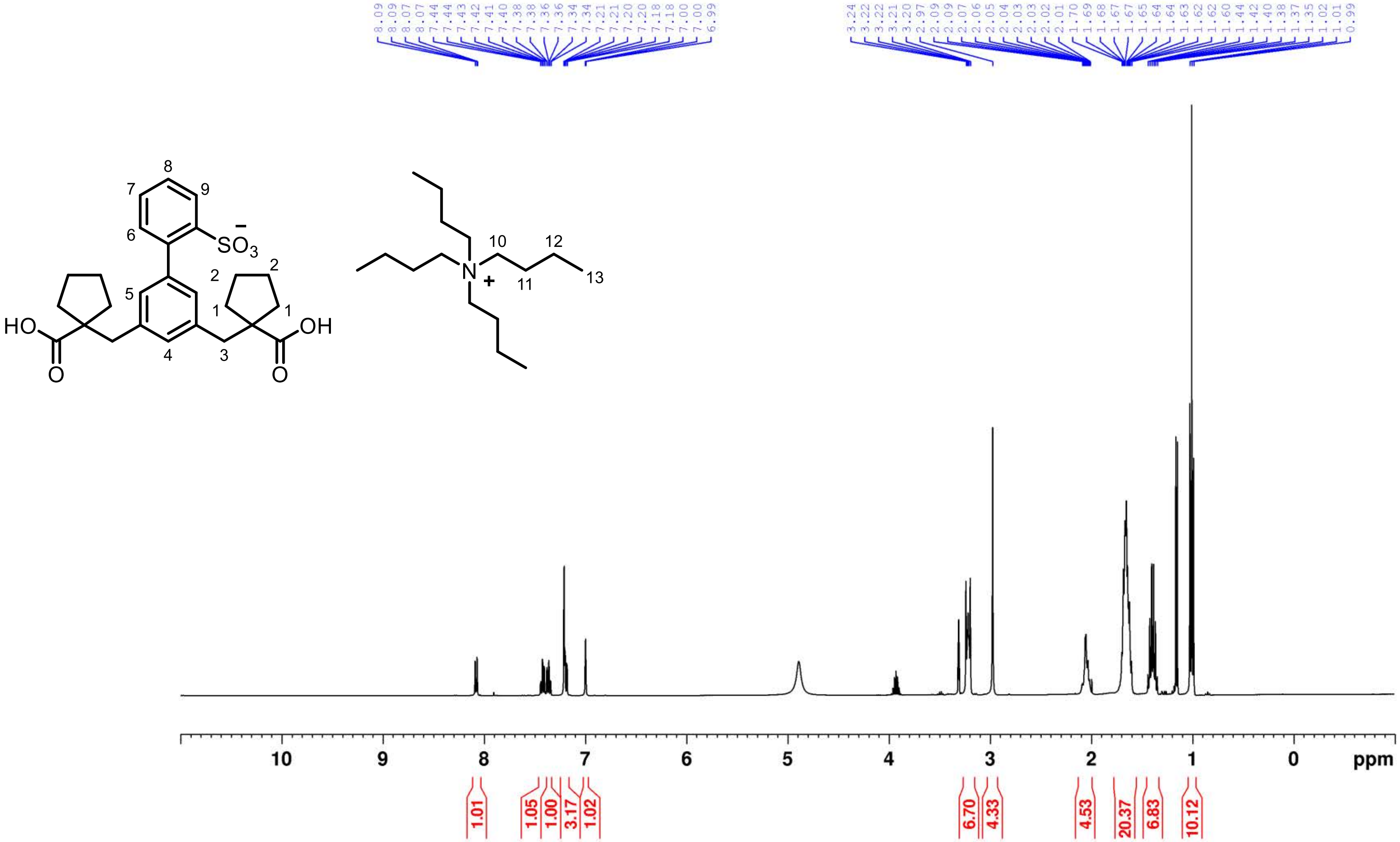

^{13}C NMR (101 MHz, CD_3OD) for *tetrabutylammonium 3',5'-bis((1-carboxycyclopentyl)methyl)-[1,1'-biphenyl]-2-sulfonate*

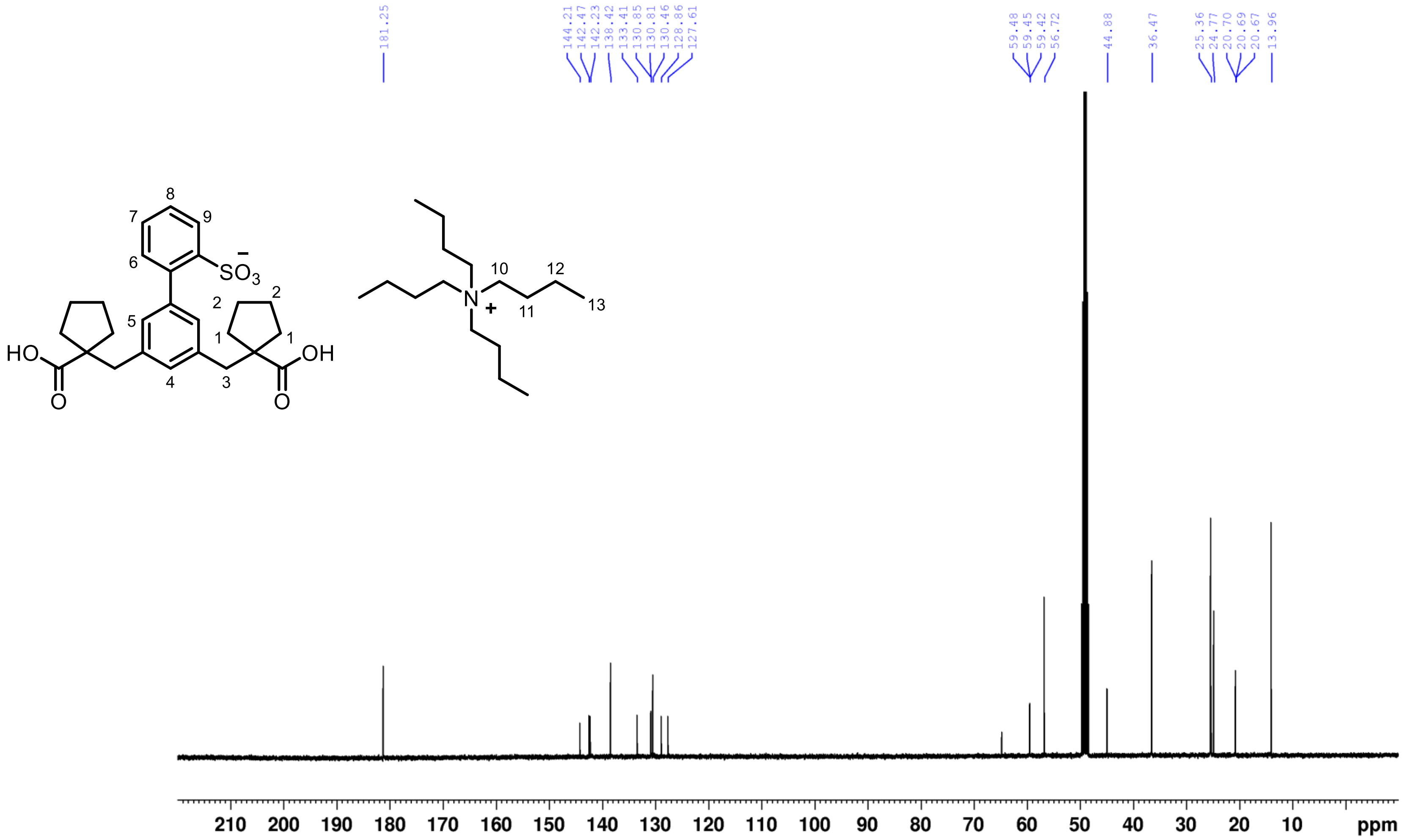

^{1}H NMR (500 MHz, $(CD_3)_2SO$) for *bis[rhodium tetrabutylammonium 3',5'-bis((1-carboxycyclopentyl)methyl)-[1,1'-biphenyl]-2-sulfonate]* (Rh_2(**C-III**)$_2$●(**NBu_4**)$_2$)

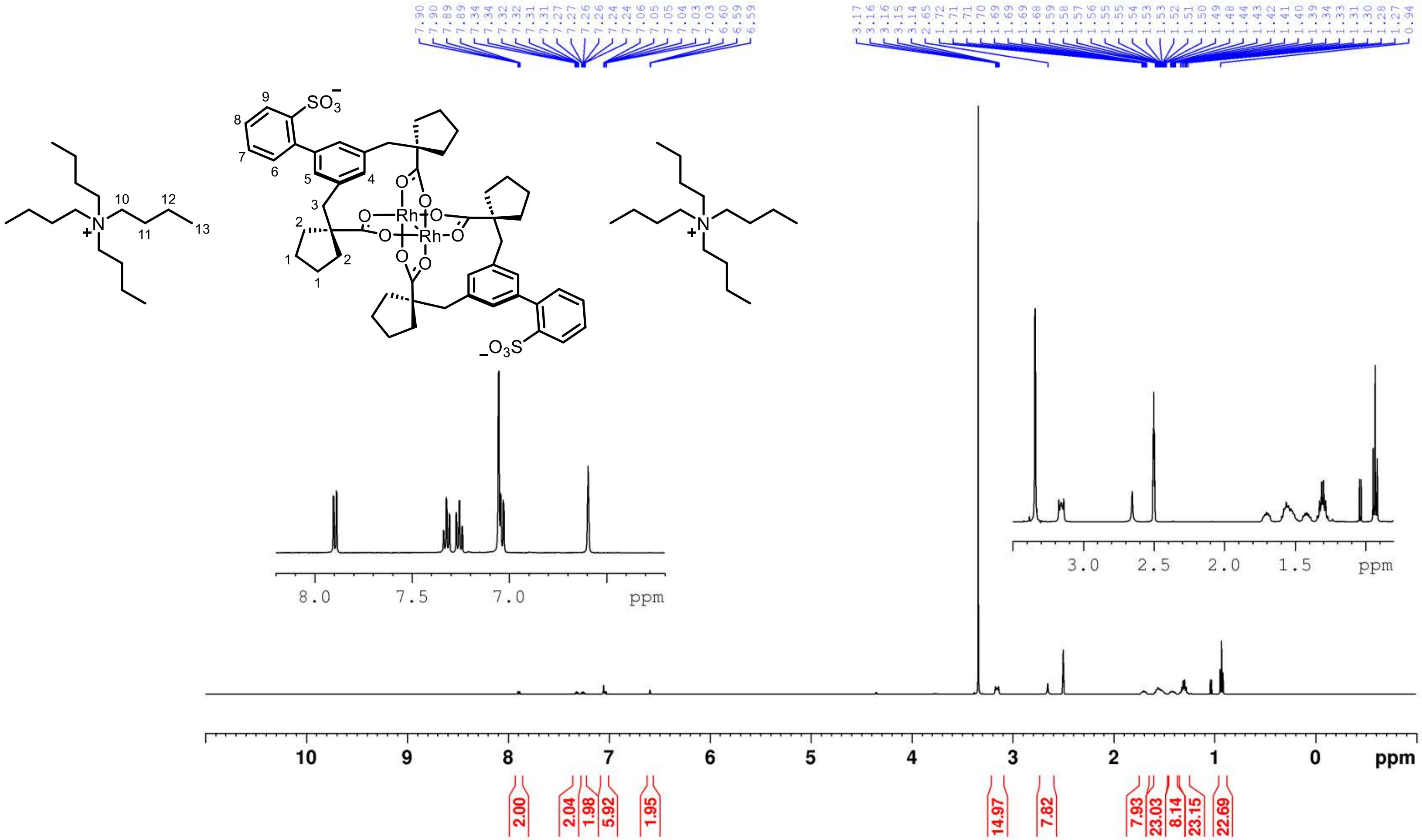

^{13}C NMR (126 MHz, $(CD_3)_2SO$) for *bis[rhodium tetrabutylammonium 3',5'-bis((1-carboxycyclopentyl)methyl)-[1,1'-biphenyl]-2-sulfonate]* (Rh_2(**C-III**)$_2$●(**NBu_4**)$_2$)

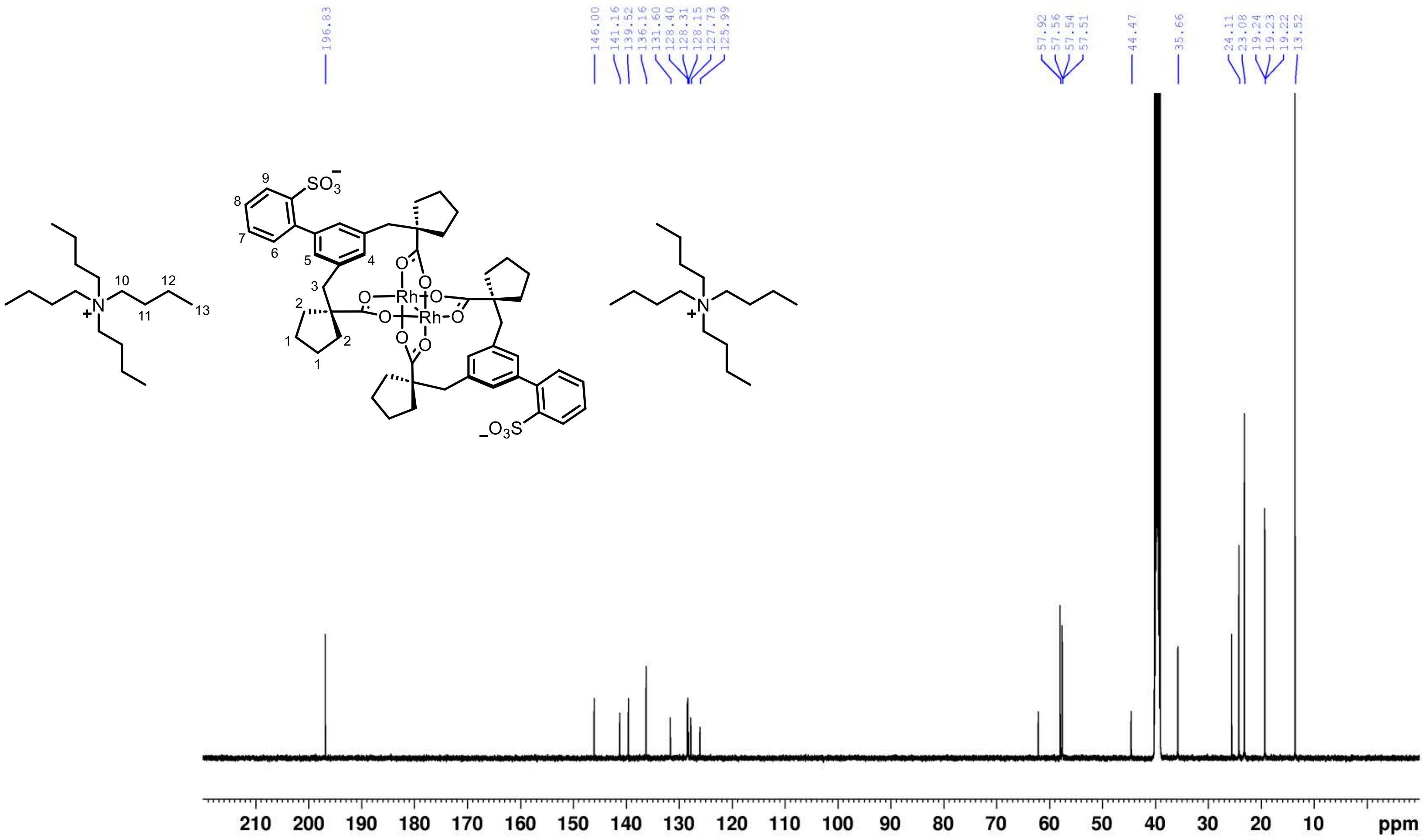

¹H NMR (500 MHz, CDCl₃) for *dimethyl 1,1'-(1,3-phenylenebis(methylene))bis(cycloheptane-1-carboxylate)*

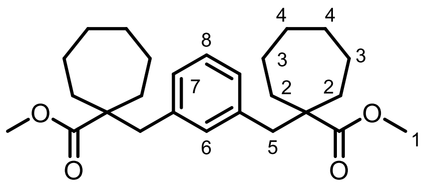

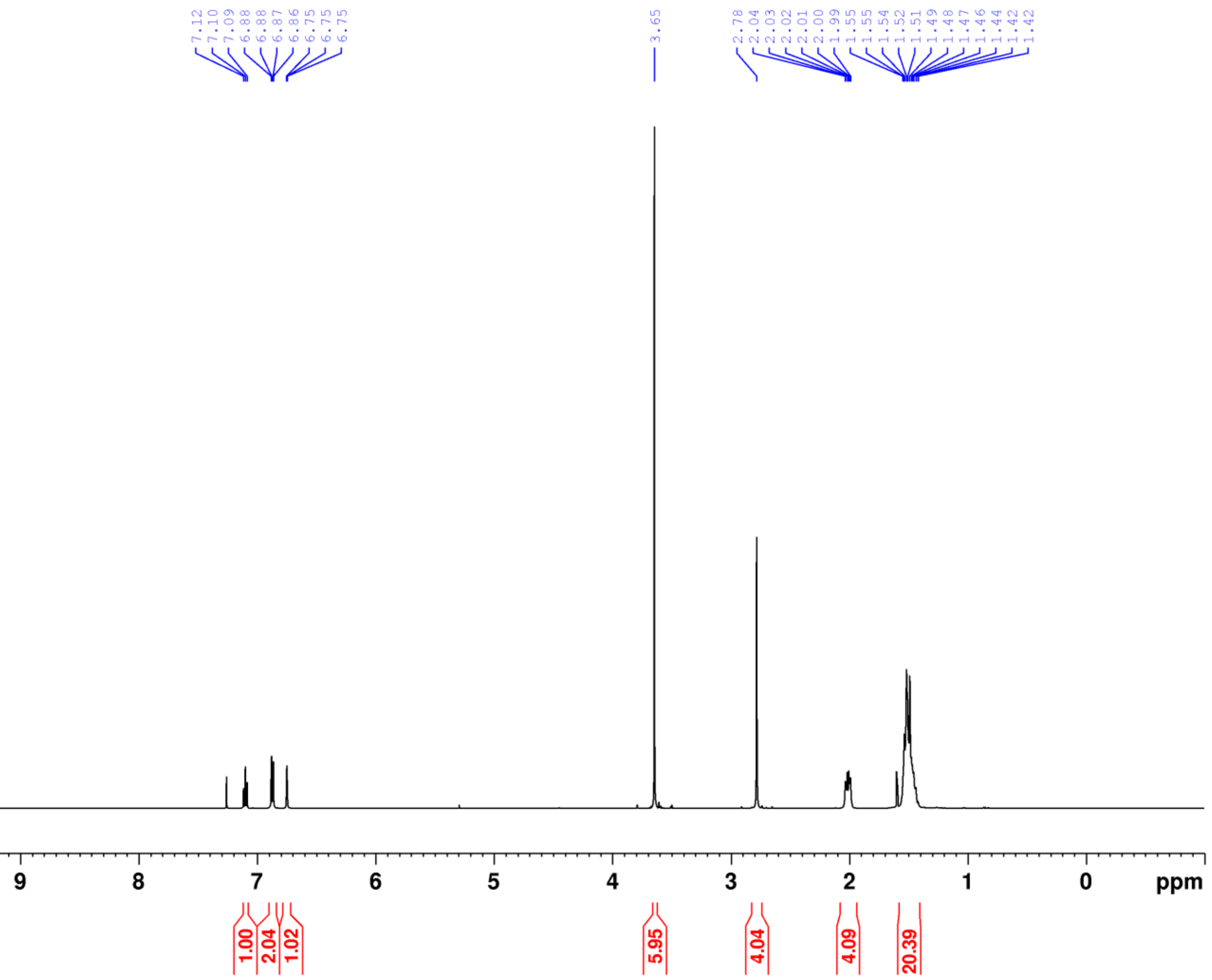

^{13}C NMR (126 MHz, CDCl$_3$) for *dimethyl 1,1'-(1,3-phenylenebis(methylene))bis(cycloheptane-1-carboxylate)*

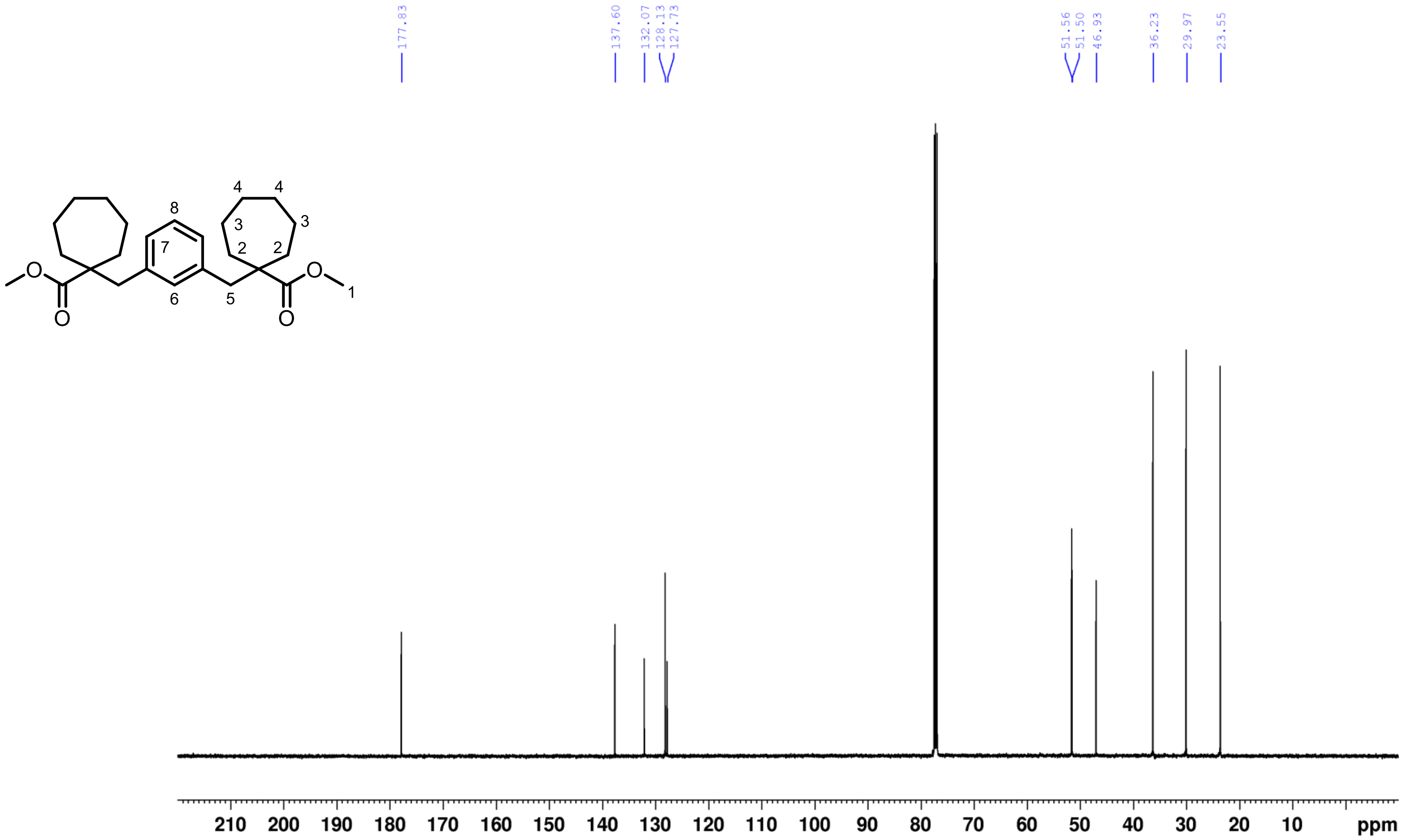

¹H NMR (500 MHz, CDCl$_3$) for *dimethyl 1,1'-((5-(4,4,5,5-tetramethyl-1,3,2-dioxaborolan-2-yl)-1,3-phenylene)bis(methylene))bis(cycloheptane-1-carboxylate)*

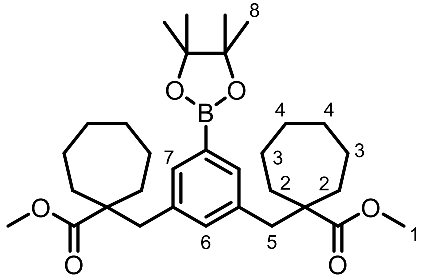

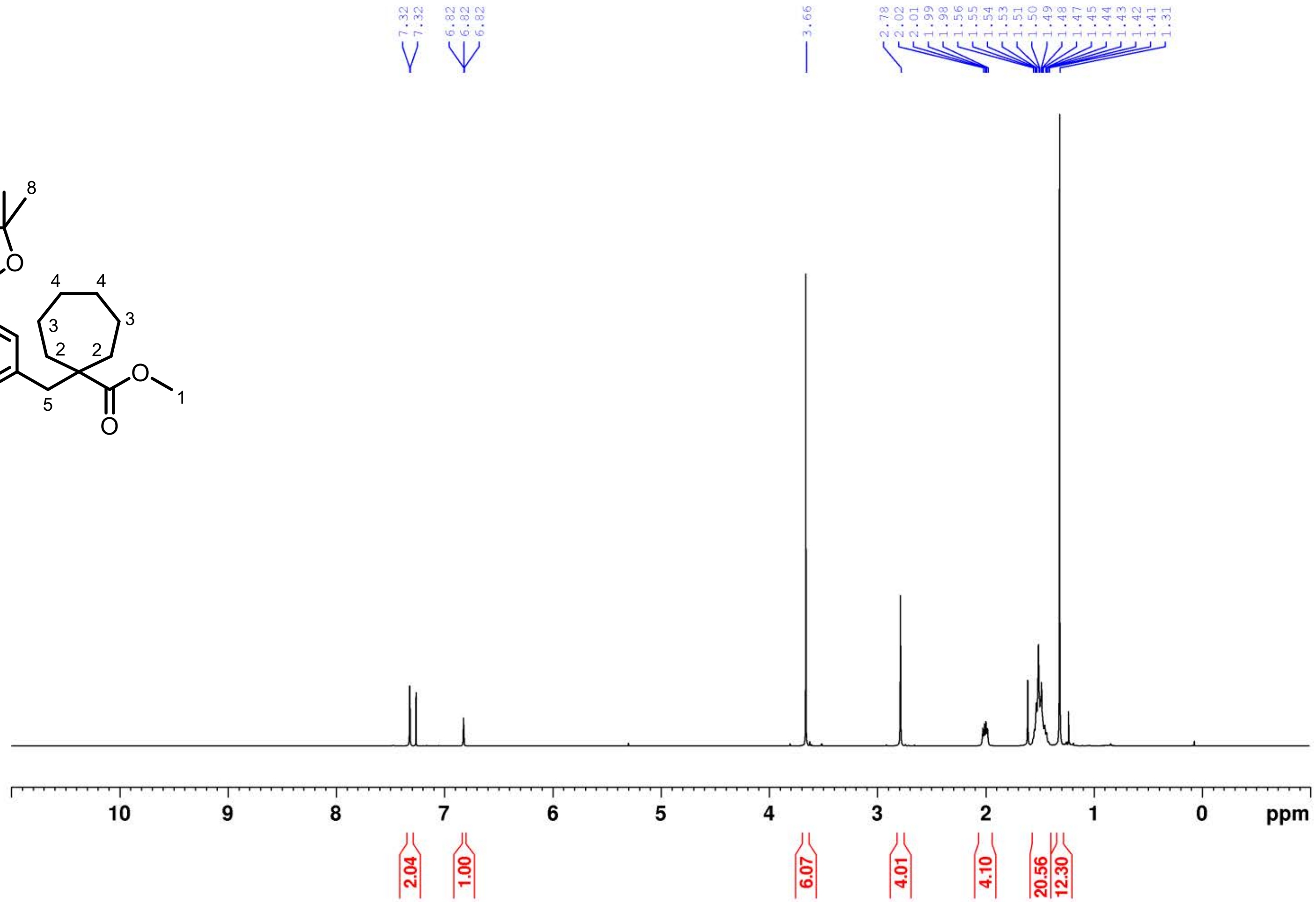

^{13}C NMR (126 MHz, CDCl$_3$) for *dimethyl 1,1'-((5-(4,4,5,5-tetramethyl-1,3,2-dioxaborolan-2-yl)-1,3-phenylene)bis(methylene))bis(cycloheptane-1-carboxylate)*

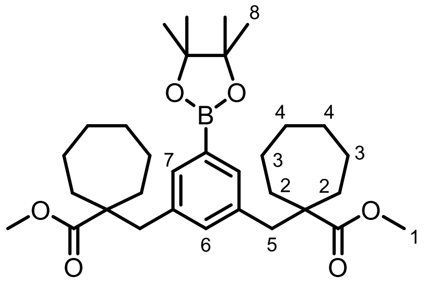

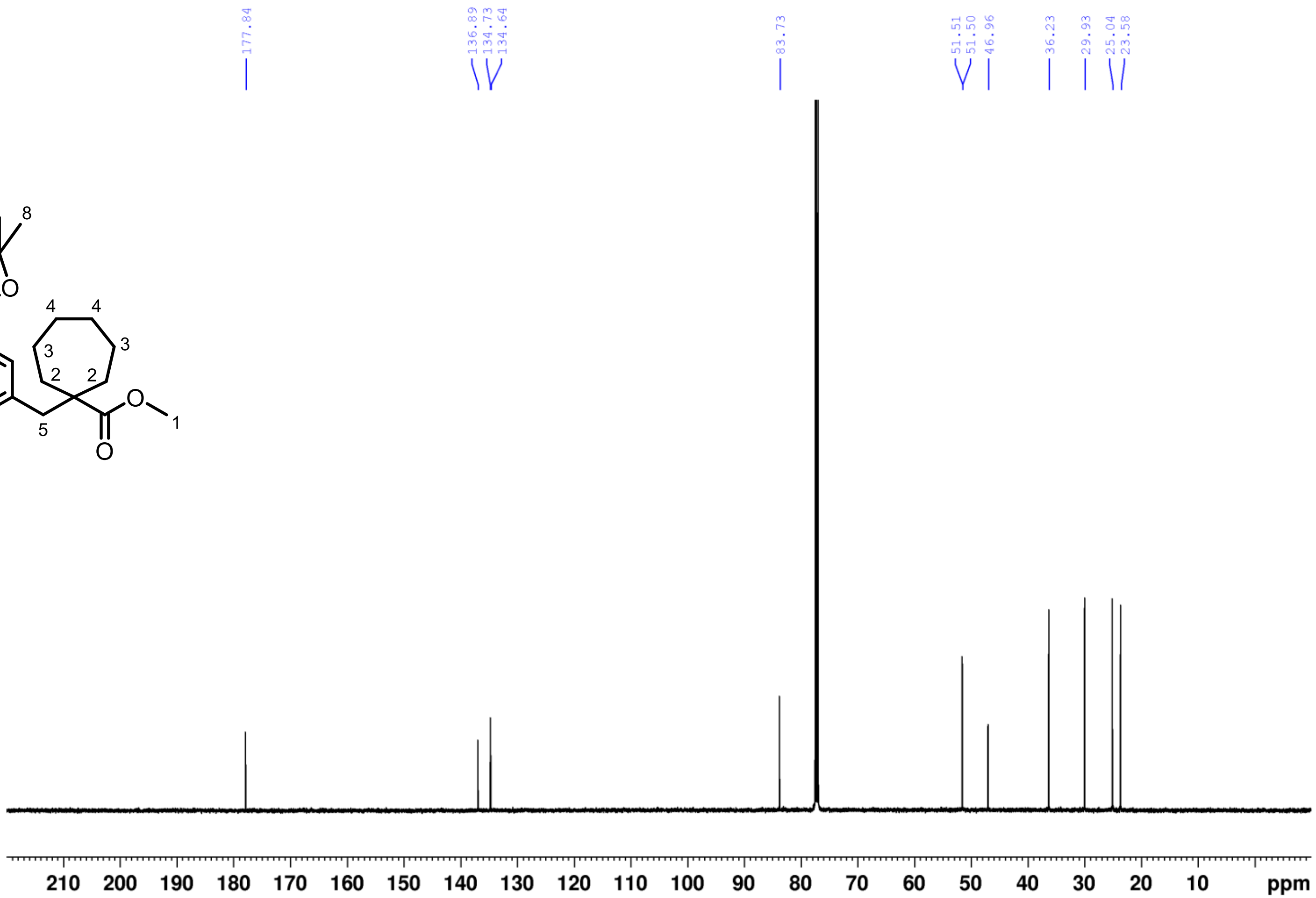

^{1}H NMR (400 MHz, $CDCl_3$) for *dimethyl 1,1'-((2'-(phenoxysulfonyl)-[1,1'-biphenyl]-3,5-diyl)bis(methylene))bis(cycloheptane-1-carboxylate)*

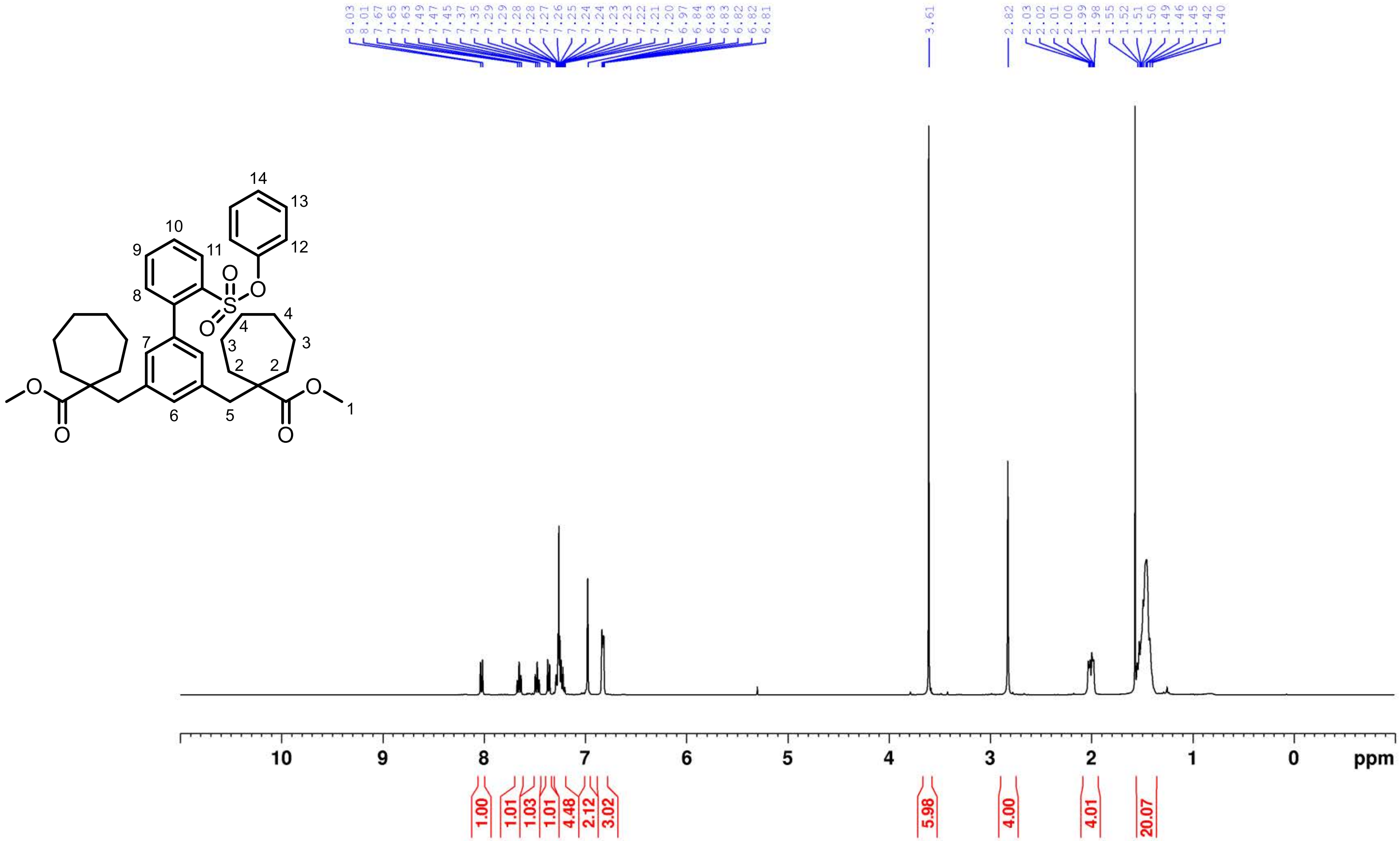

^{13}C NMR (101 MHz, $CDCl_3$) for *dimethyl 1,1'-((2'-(phenoxysulfonyl)-[1,1'-biphenyl]-3,5-diyl)bis(methylene))bis(cycloheptane-1-carboxylate)*

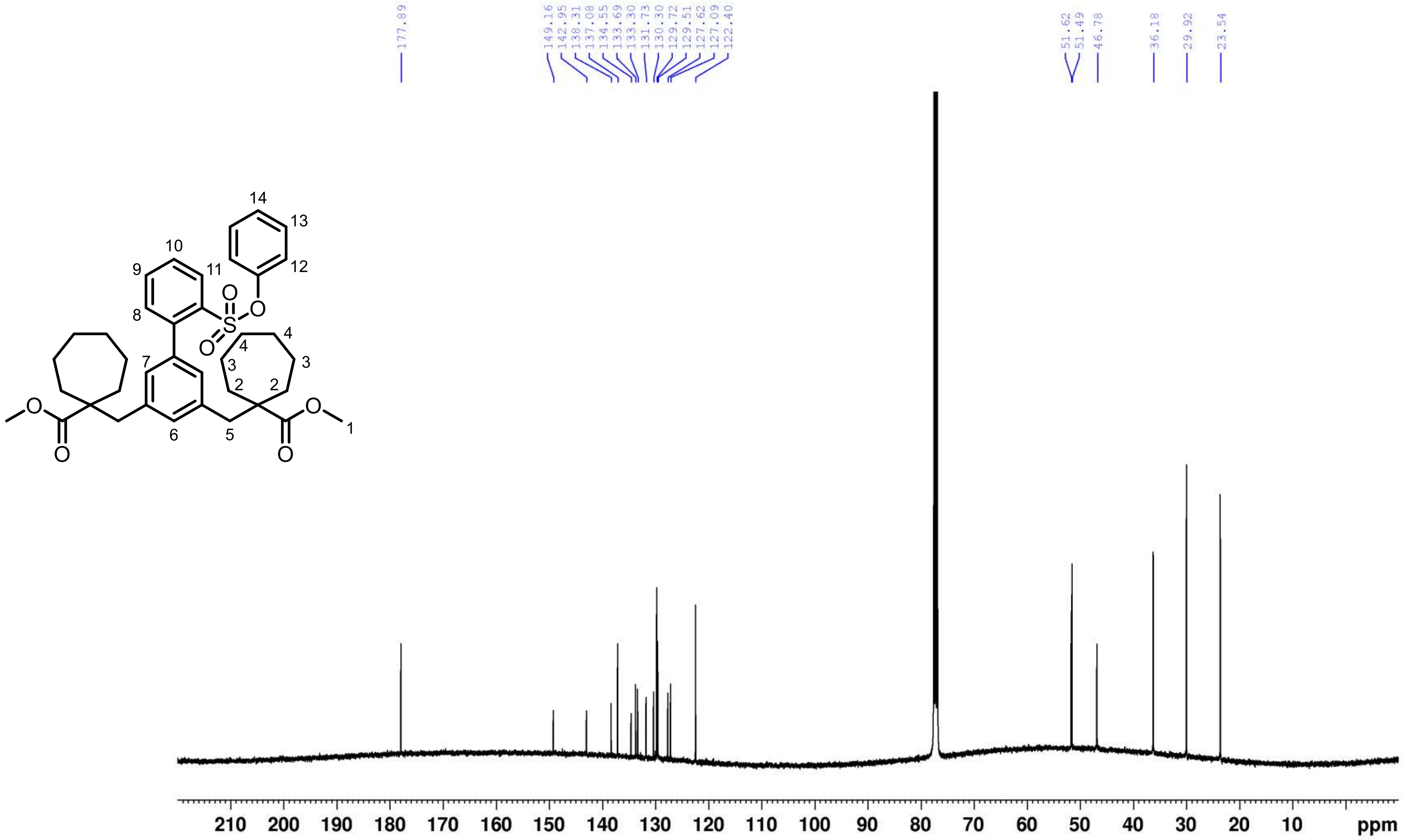

^{1}H NMR (500 MHz, CD_3OD) for *tetrabutylammonium 3',5'-bis((1-carboxycycloheptyl)methyl)-[1,1'-biphenyl]-2-sulfonate*

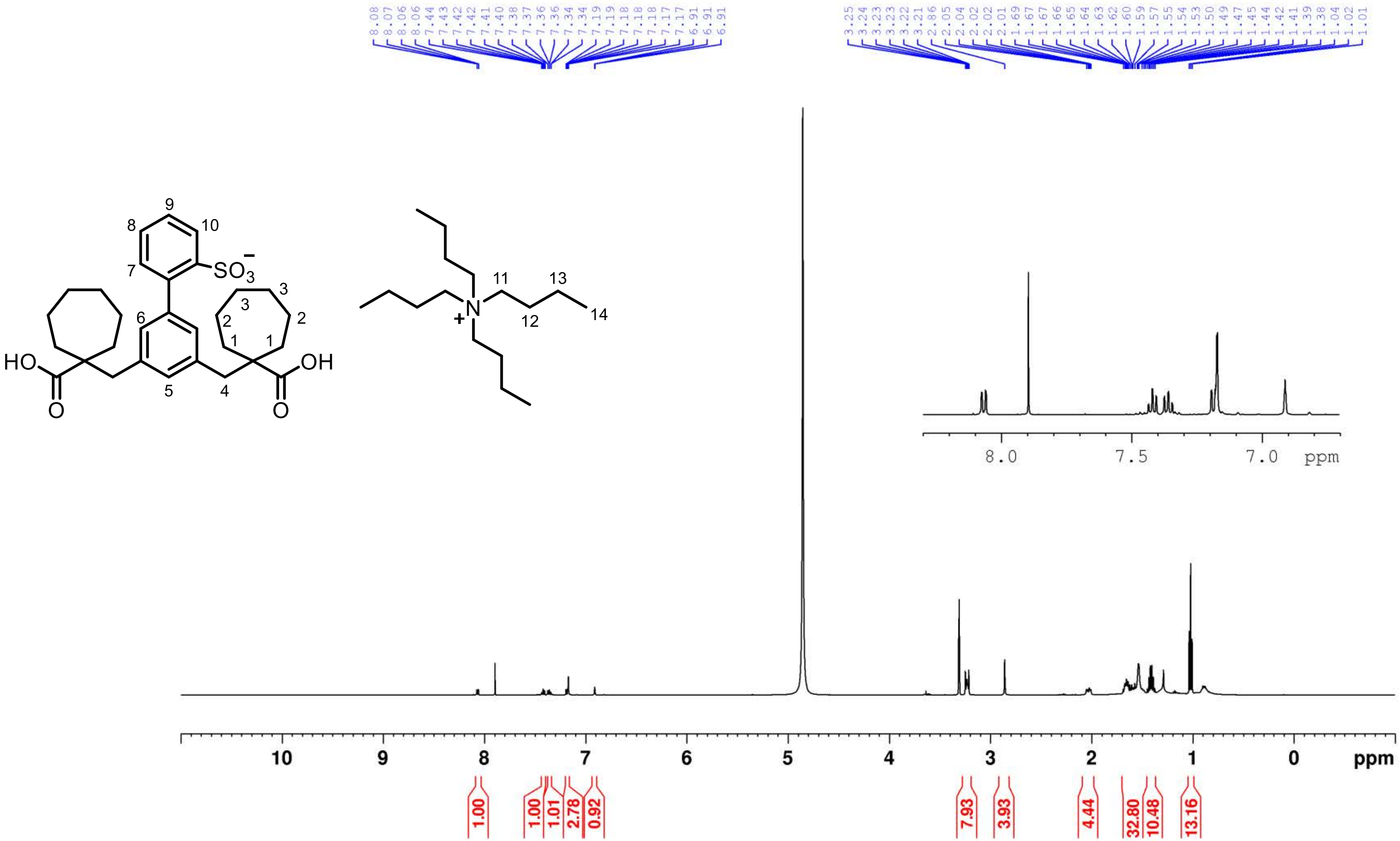

^{13}C NMR (126 MHz, CD_3OD) for *tetrabutylammonium 3',5'-bis((1-carboxycycloheptyl)methyl)-[1,1'-biphenyl]-2-sulfonate*

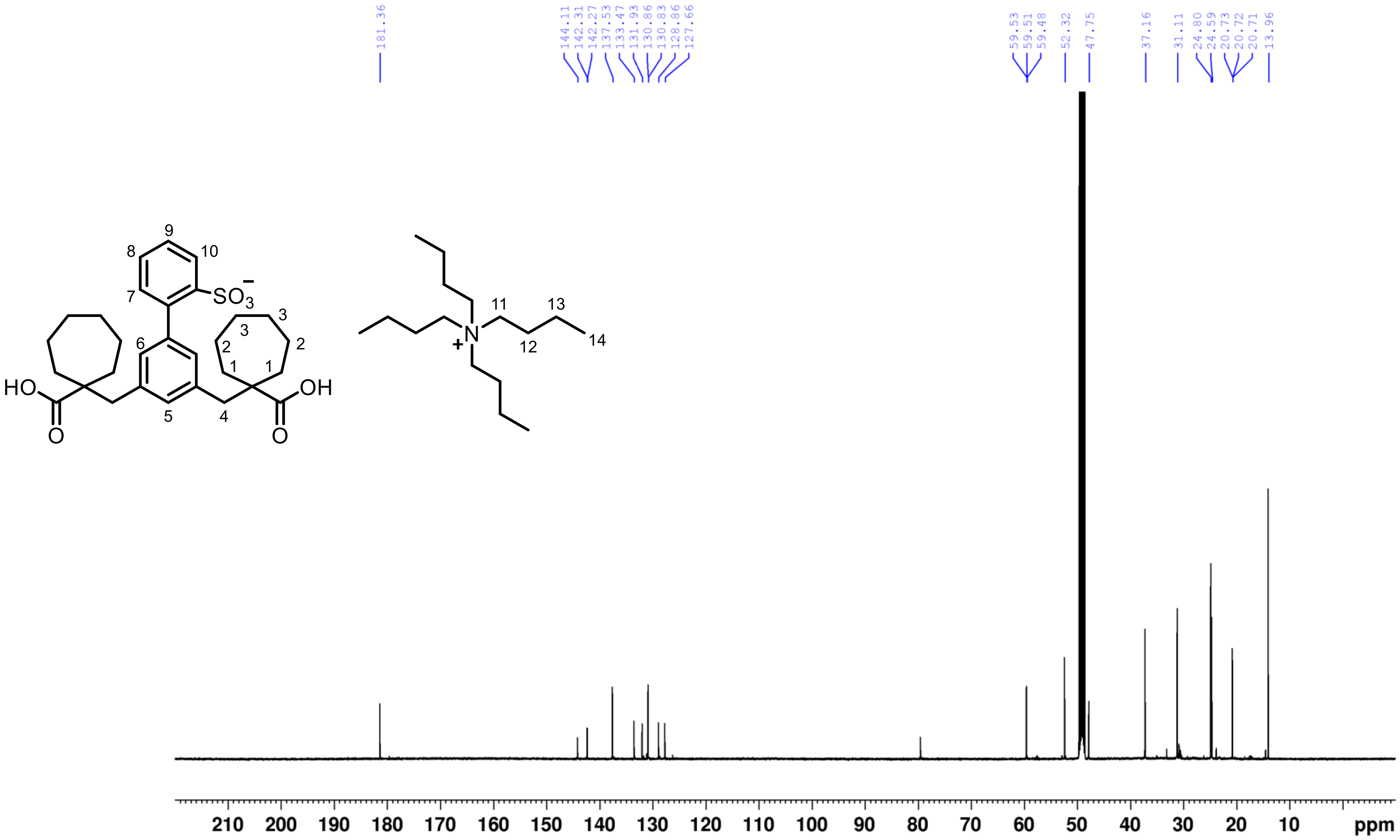

^{1}H NMR (500 MHz, CD_3OD) for *bis[rhodium tetrabutylammonium 3',5'-bis((1-carboxycycloheptyl)methyl)-[1,1'-biphenyl]-2-sulfonate]* (Rh_2(**D-III**)$_2$●(NBu_4)$_2$)

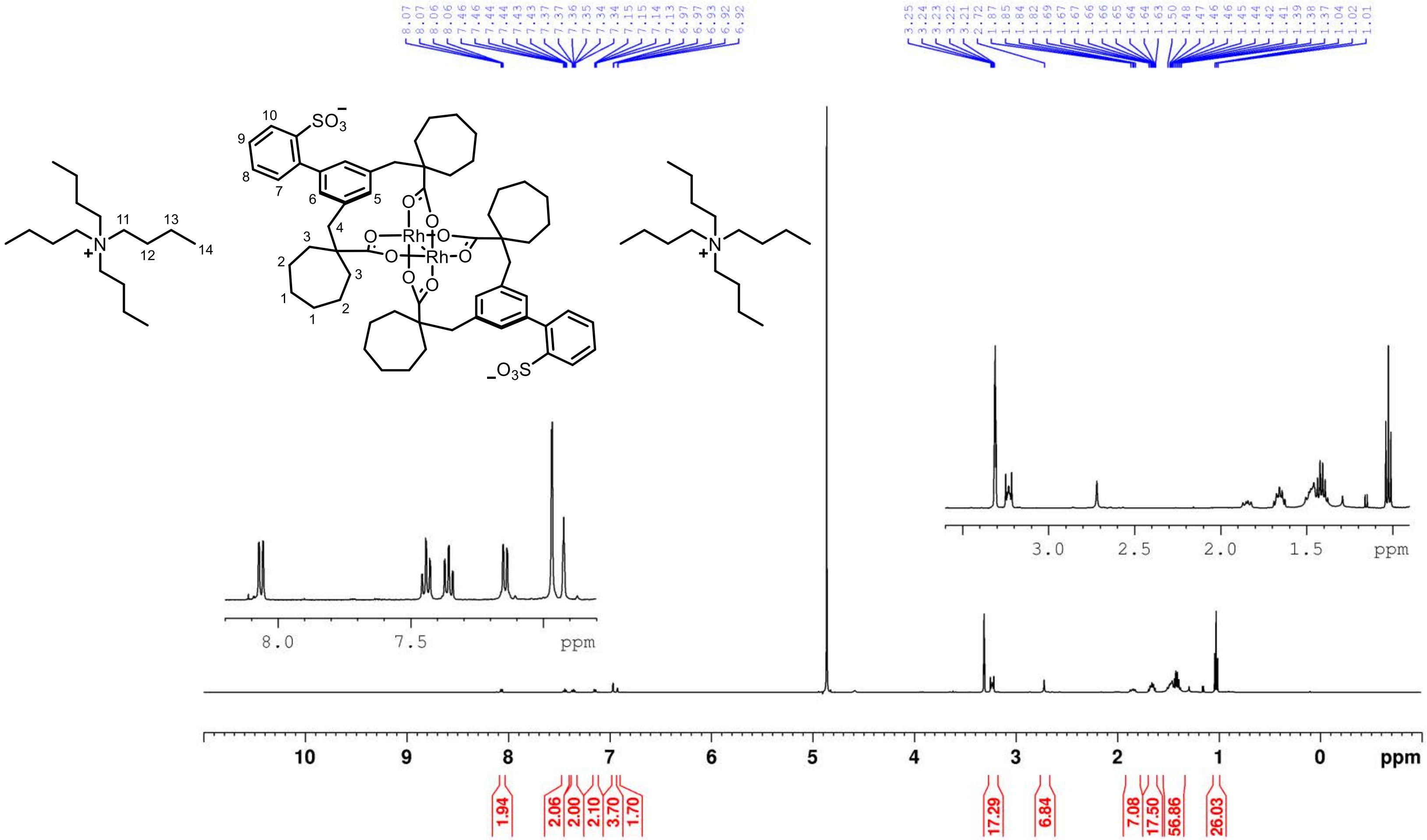

^{13}C NMR (126 MHz, CD_3OD) for *bis[rhodium tetrabutylammonium 3',5'-bis((1-carboxycycloheptyl)methyl)-[1,1'-biphenyl]-2-sulfonate]* (Rh_2(**D-III**)$_2$●(**NBu_4**)$_2$)

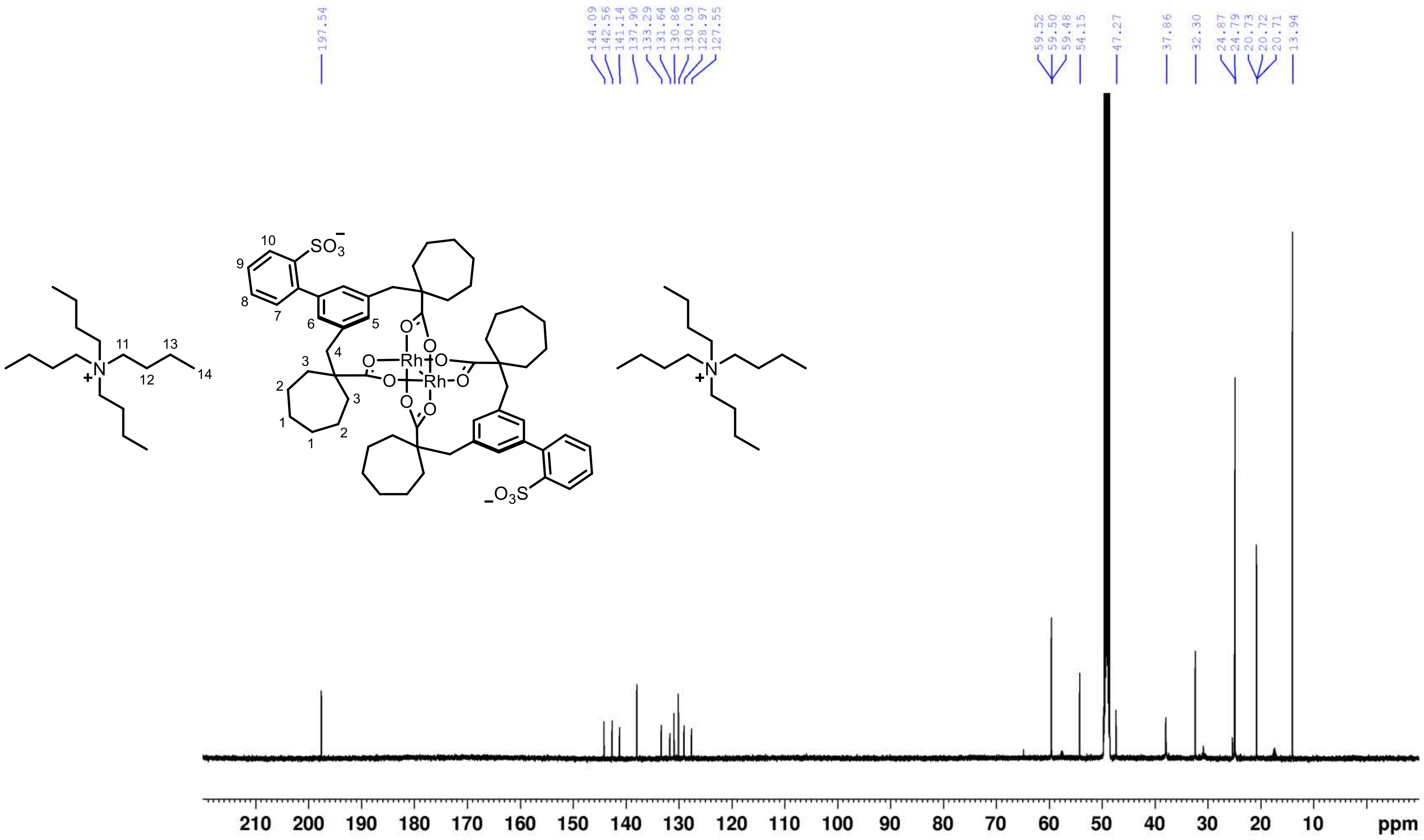

^{1}H NMR (400 MHz, $CDCl_3$) for *dimethyl 3,3'-(2'-(hydroxymethyl)-[1,1'-biphenyl]-3,5-diyl)bis(2,2-dimethylpropanoate)*

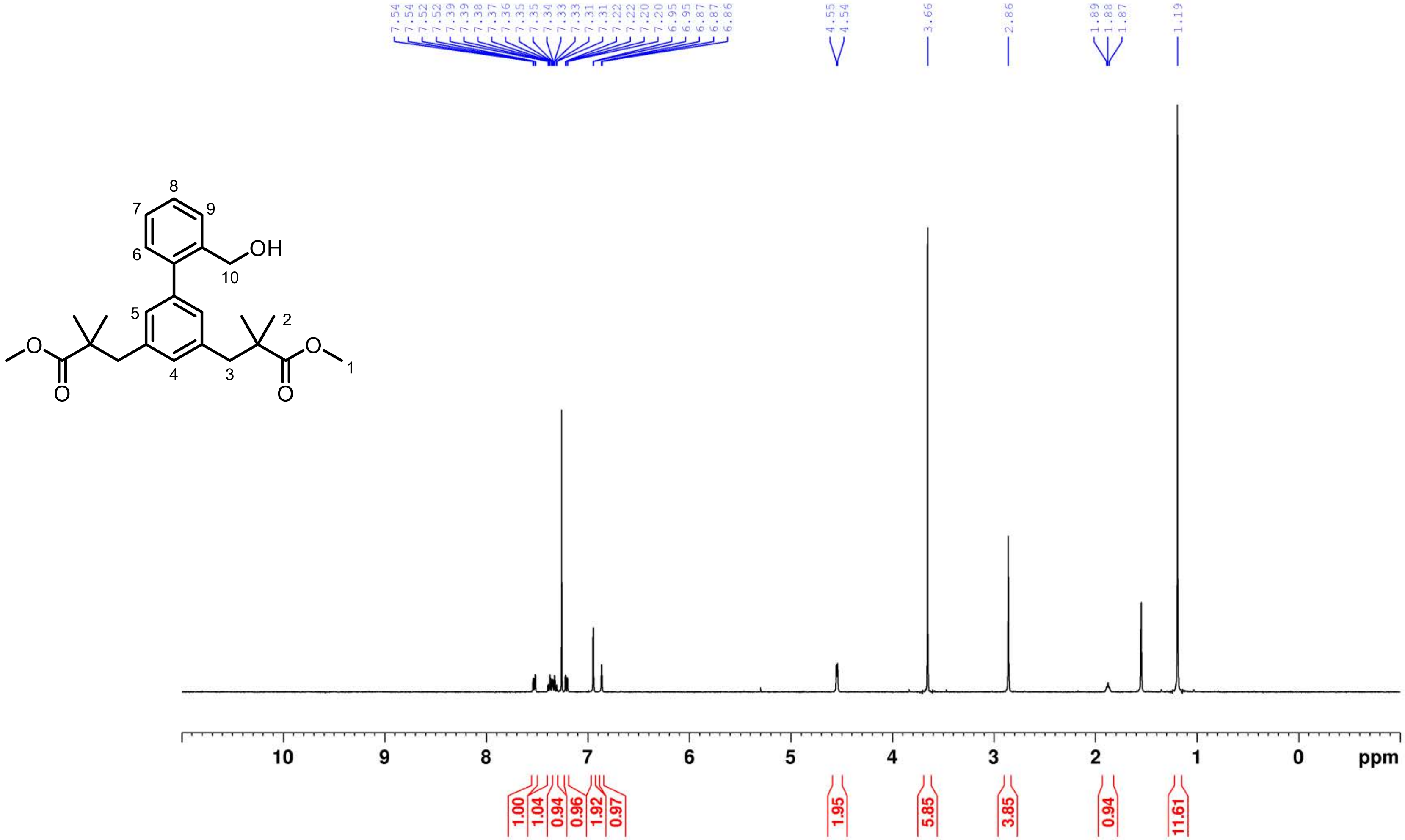

^{13}C NMR (101 MHz, $CDCl_3$) for *dimethyl 3,3'-(2'-(hydroxymethyl)-[1,1'-biphenyl]-3,5-diyl)bis(2,2-dimethylpropanoate)*

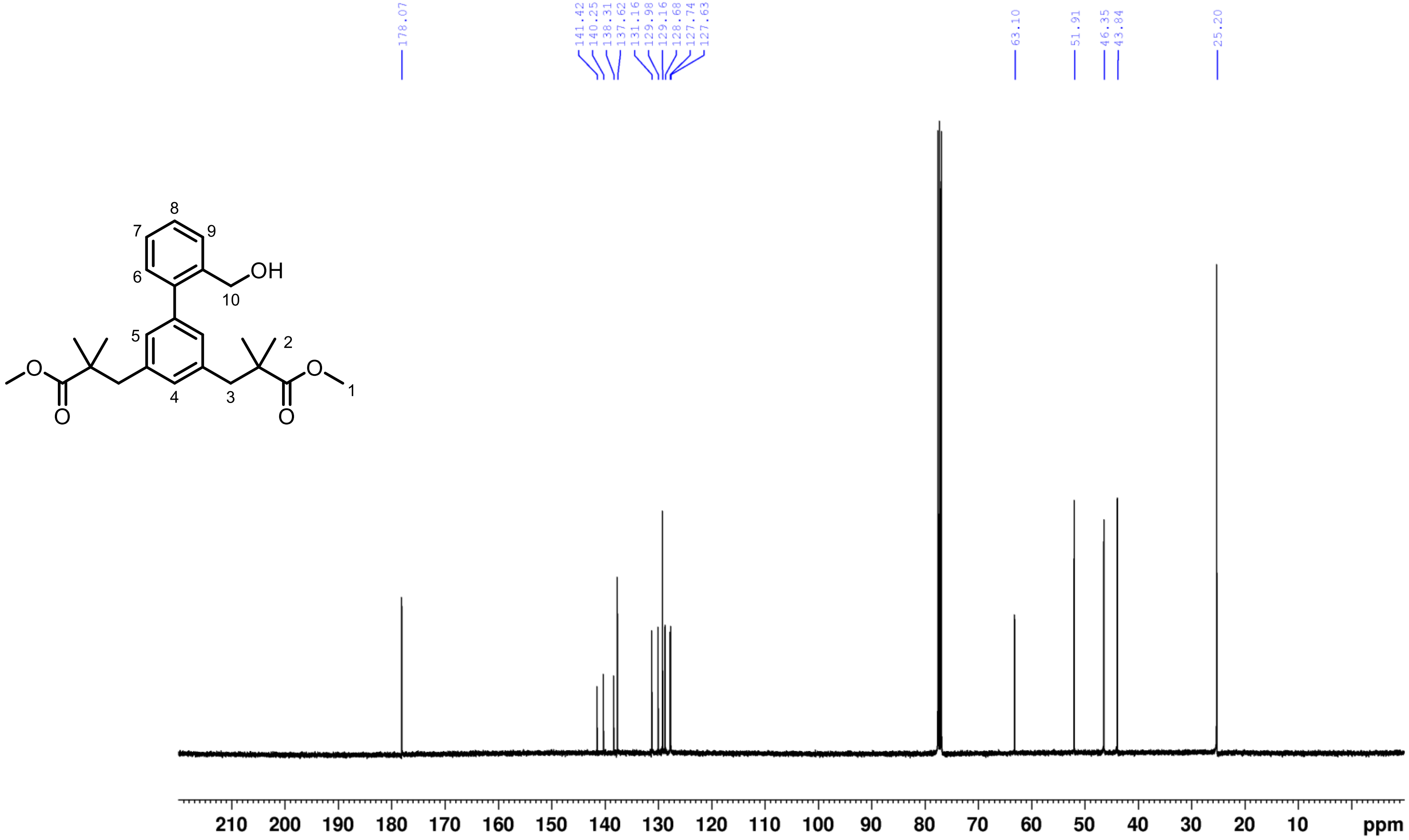

^{1}H NMR (400 MHz, $CDCl_3$) for *dimethyl 3,3'-(2'-(bromomethyl)-[1,1'-biphenyl]-3,5-diyl)bis(2,2-dimethylpropanoate)*

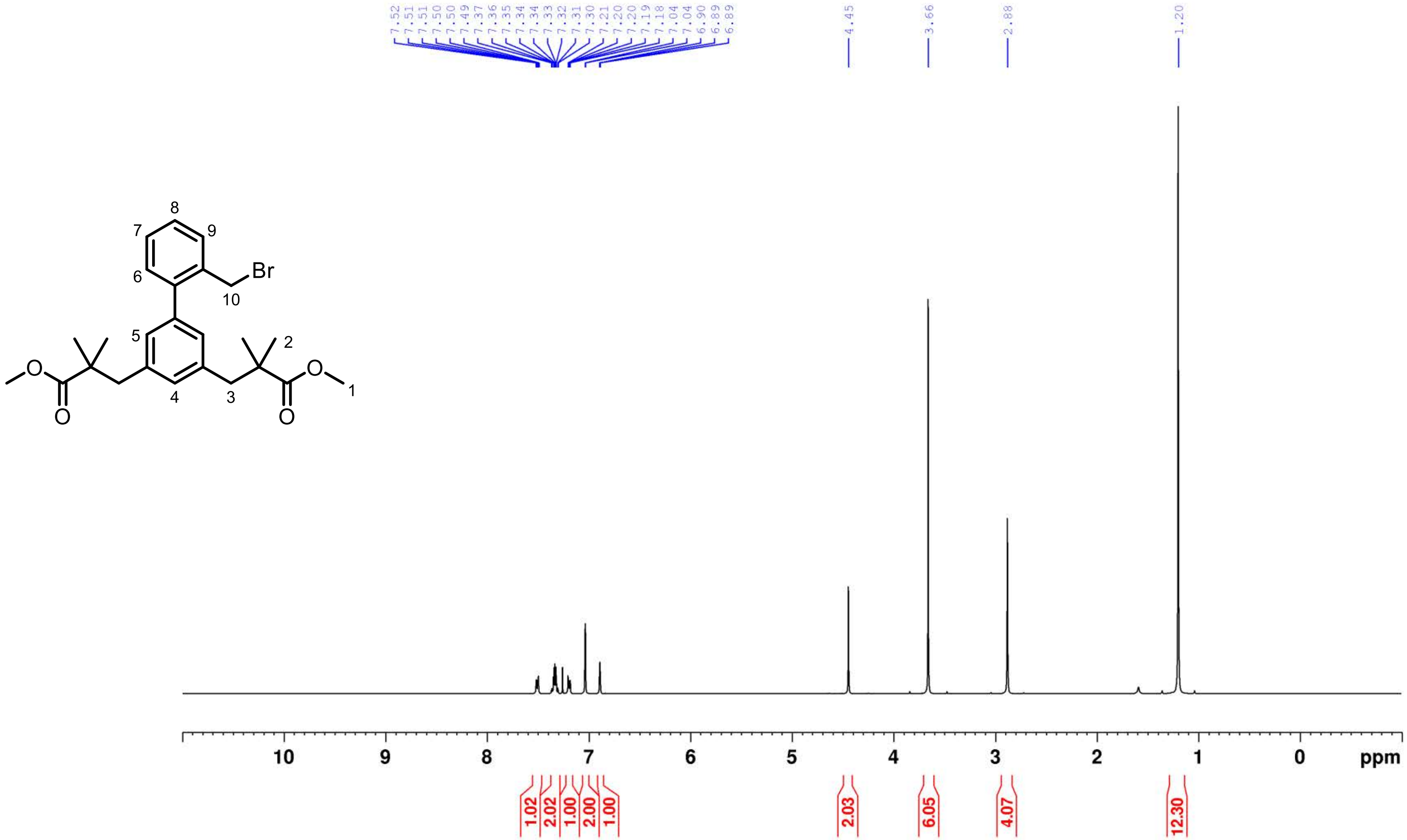

^{13}C NMR (101 MHz, $CDCl_3$) for *dimethyl 3,3'-(2'-(bromomethyl)-[1,1'-biphenyl]-3,5-diyl)bis(2,2-dimethylpropanoate)*

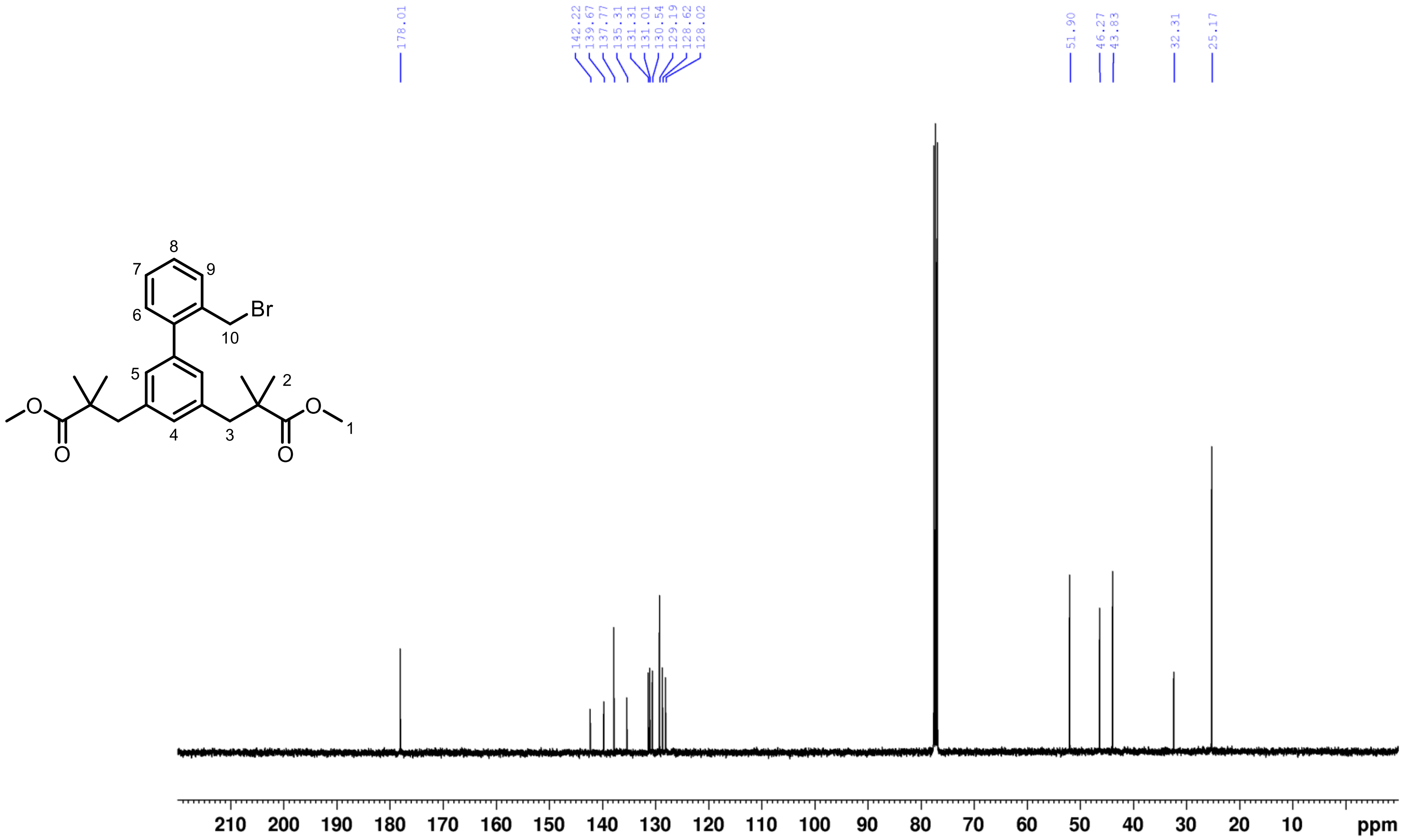

^{1}H NMR (400 MHz, CDCl$_3$) for *tetrabutylammonium (3',5'-bis(3-methoxy-2,2-dimethyl-3-oxopropyl)-[1,1'-biphenyl]-2-yl)methanesulfonate*

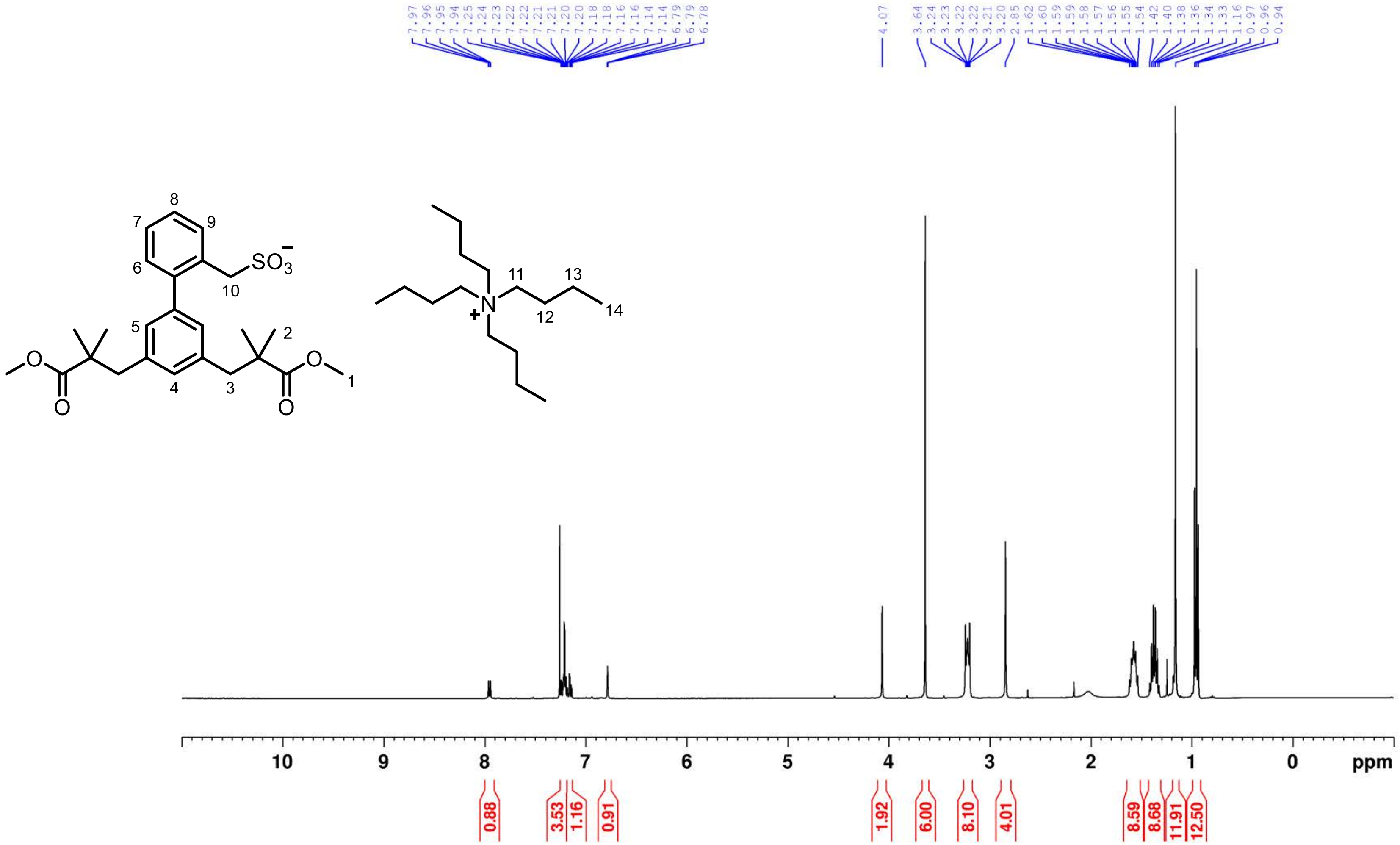

^{13}C NMR (101 MHz, $CDCl_3$) for *tetrabutylammonium (3',5'-bis(3-methoxy-2,2-dimethyl-3-oxopropyl)-[1,1'-biphenyl]-2-yl)methanesulfonate*

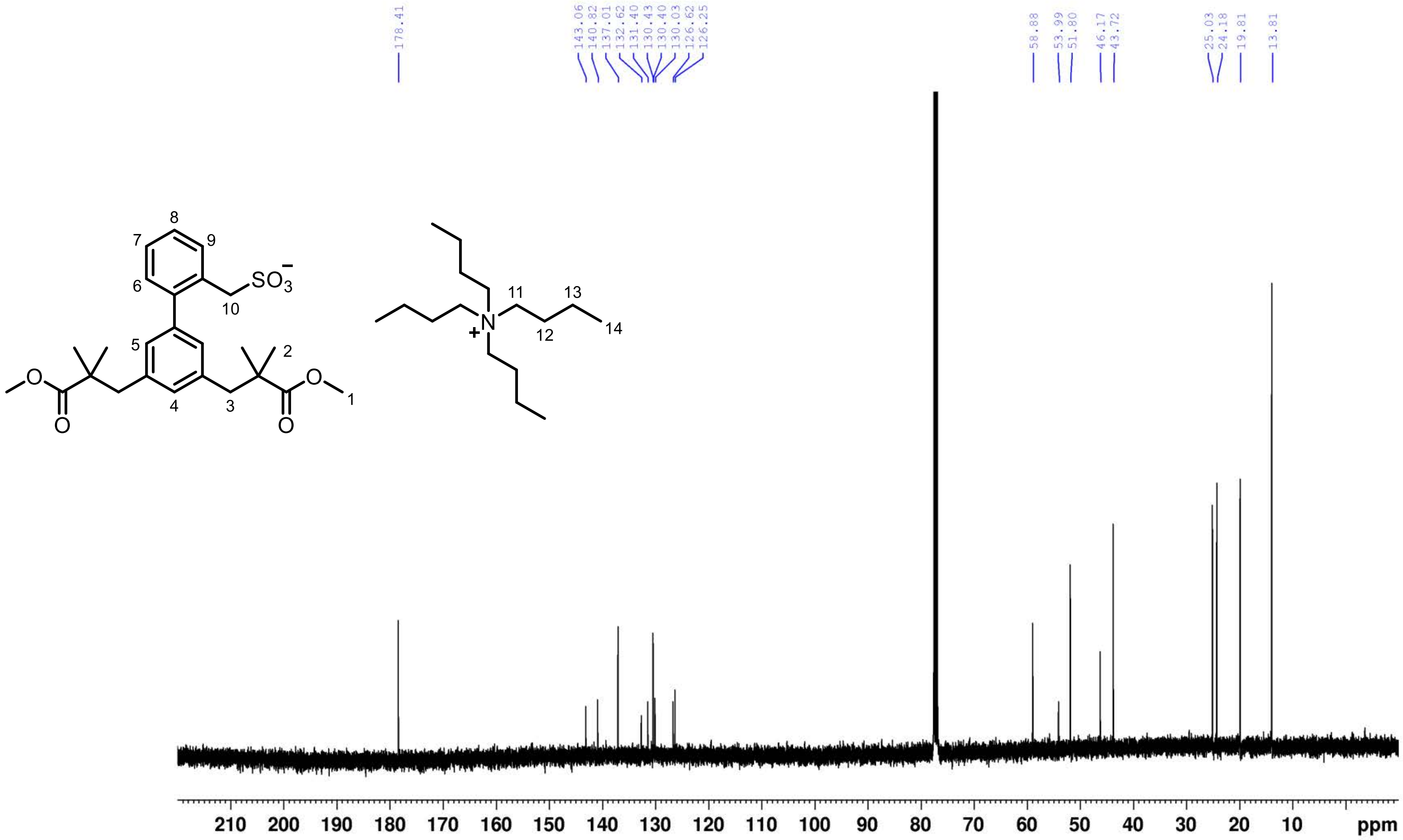

^{1}H NMR (400 MHz, CD_3OD) for *tetrabutylammonium (3',5'-bis(2-carboxy-2-methylpropyl)-[1,1'-biphenyl]-2-yl)methanesulfonate*

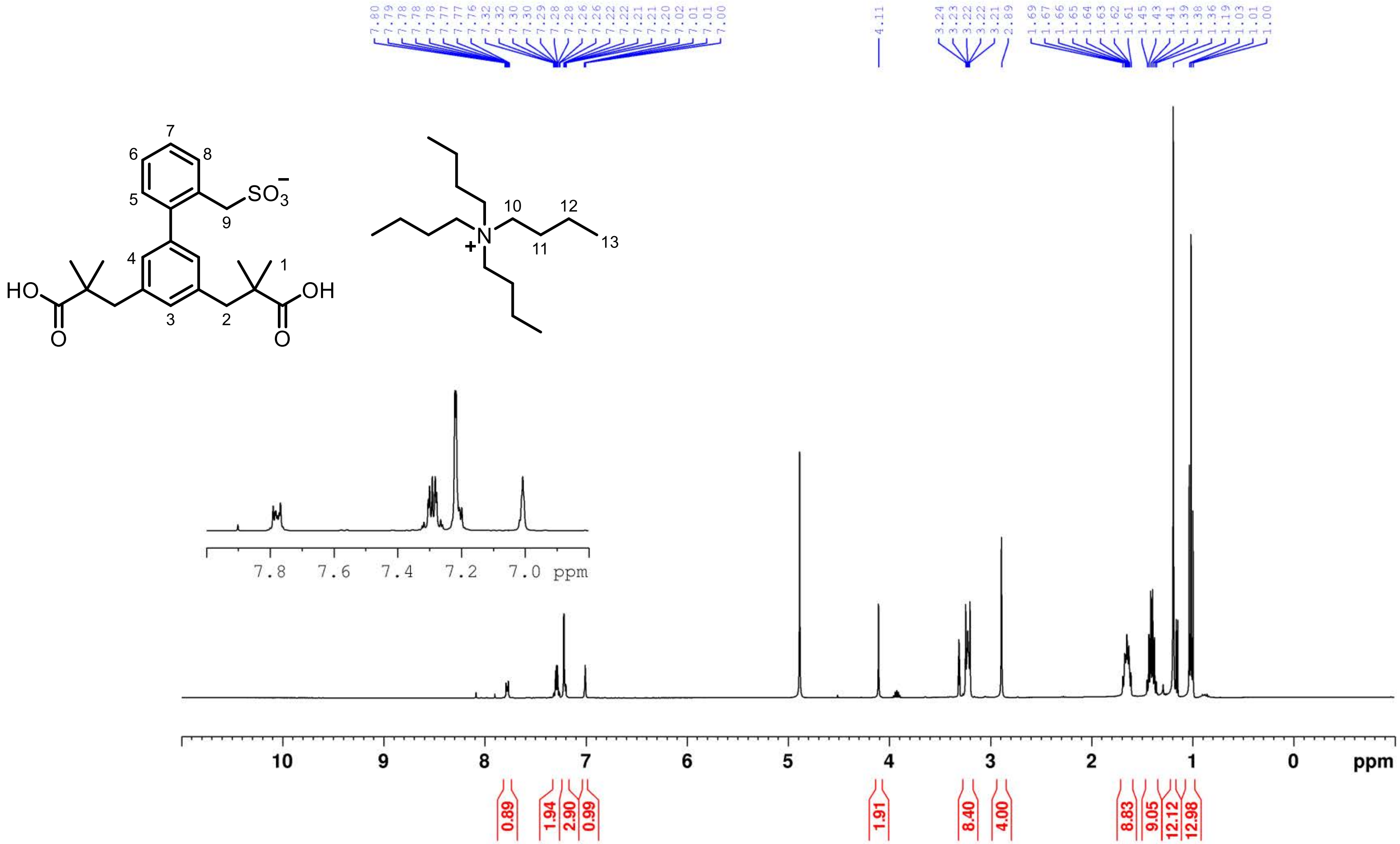

^{13}C NMR (101 MHz, CD_3OD) for *tetrabutylammonium (3',5'-bis(2-carboxy-2-methylpropyl)-[1,1'-biphenyl]-2-yl)methanesulfonate*

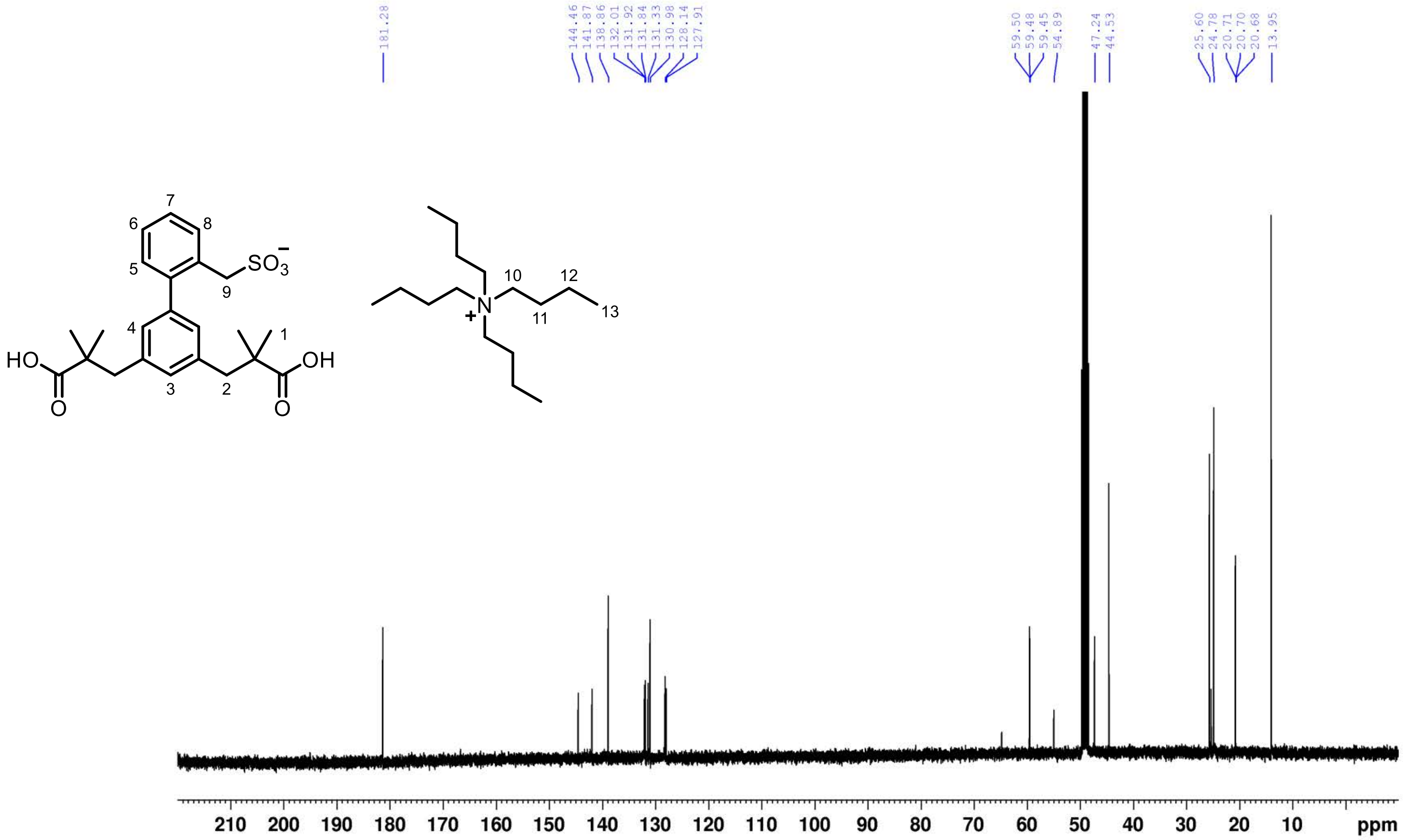

^{1}H NMR (700 MHz, CD_3OD) for *bis[rhodium tetrabutylammonium (3',5'-bis(2-carboxy-2-methylpropyl)-[1,1'-biphenyl]-2-yl)methanesulfonate]* (Rh_2(**A-IV**)$_2$●(**NBu_4**)$_2$)

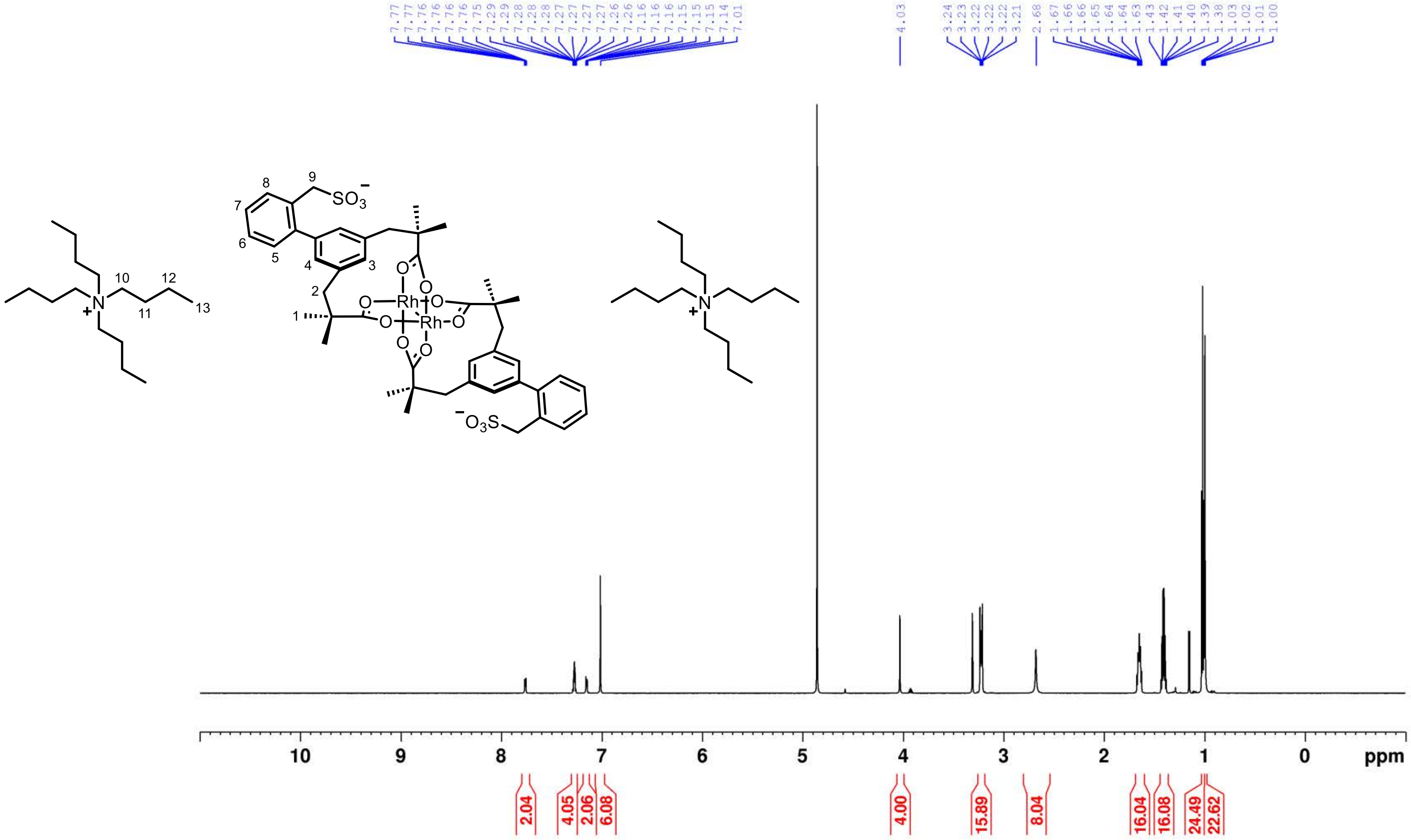

^{13}C NMR (176 MHz, CD_3OD) for *bis[rhodium tetrabutylammonium (3',5'-bis(2-carboxy-2-methylpropyl)-[1,1'-biphenyl]-2-yl)methanesulfonate]* (Rh_2(**A-IV**)$_2$●(**NBu$_4$**)$_2$)

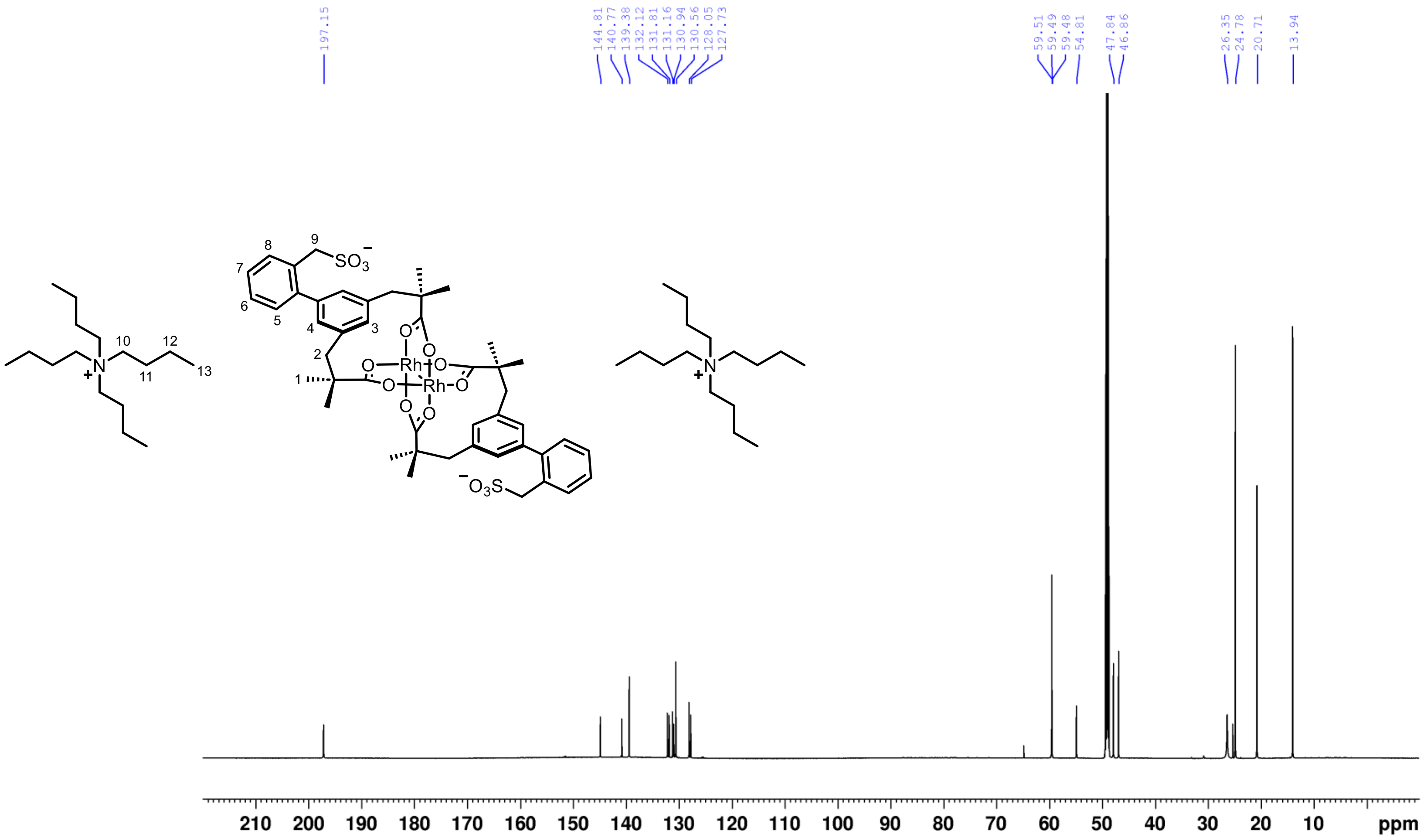

^{1}H NMR (500 MHz, $CDCl_3$) for *phenyl 8-iodonaphthalene-2-sulfonate*

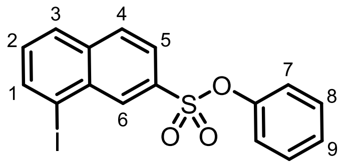

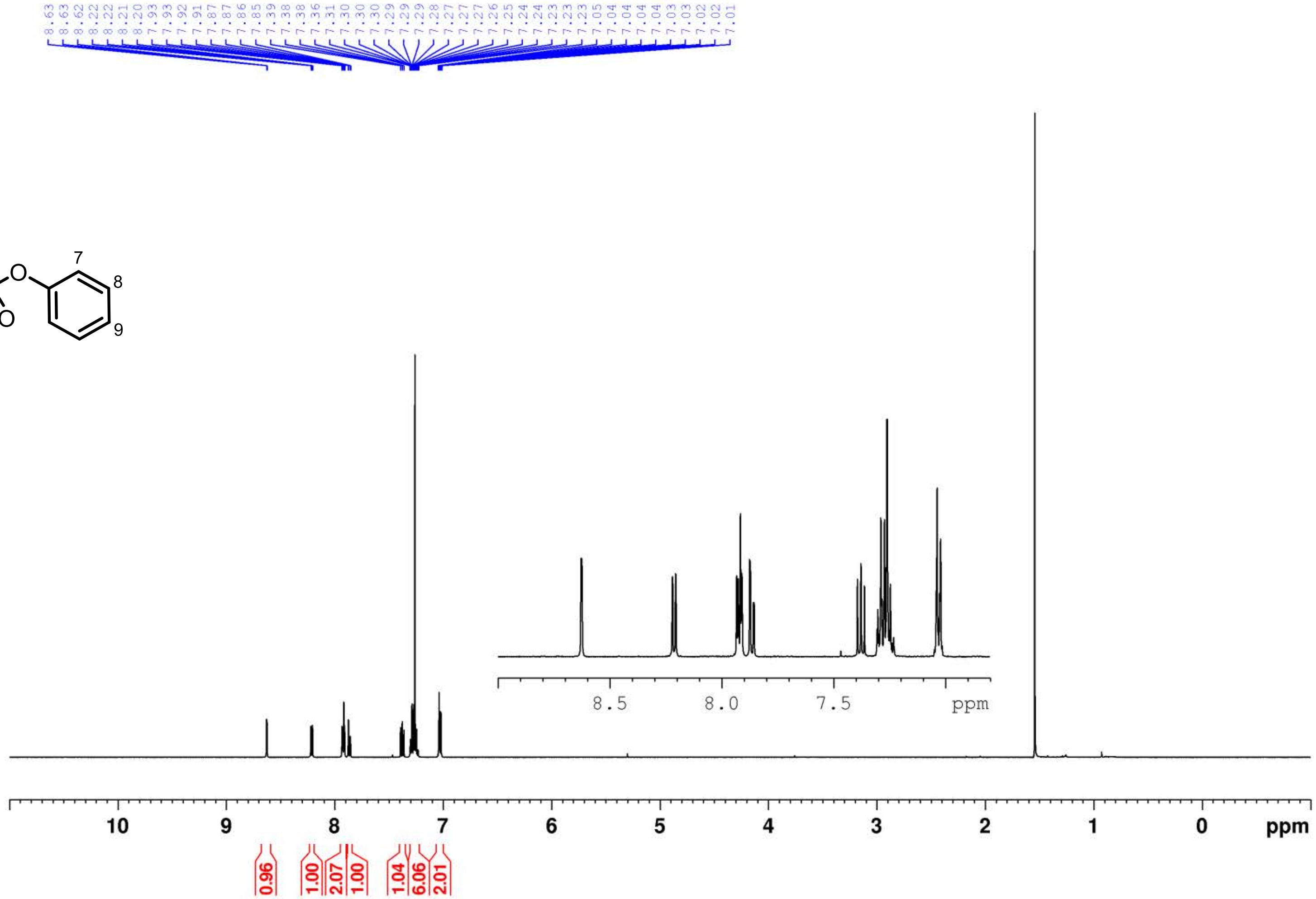

^{13}C NMR (101 MHz, $CDCl_3$) for *phenyl 8-iodonaphthalene-2-sulfonate*

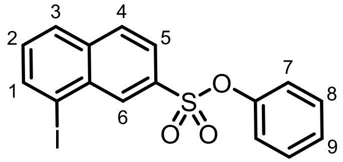

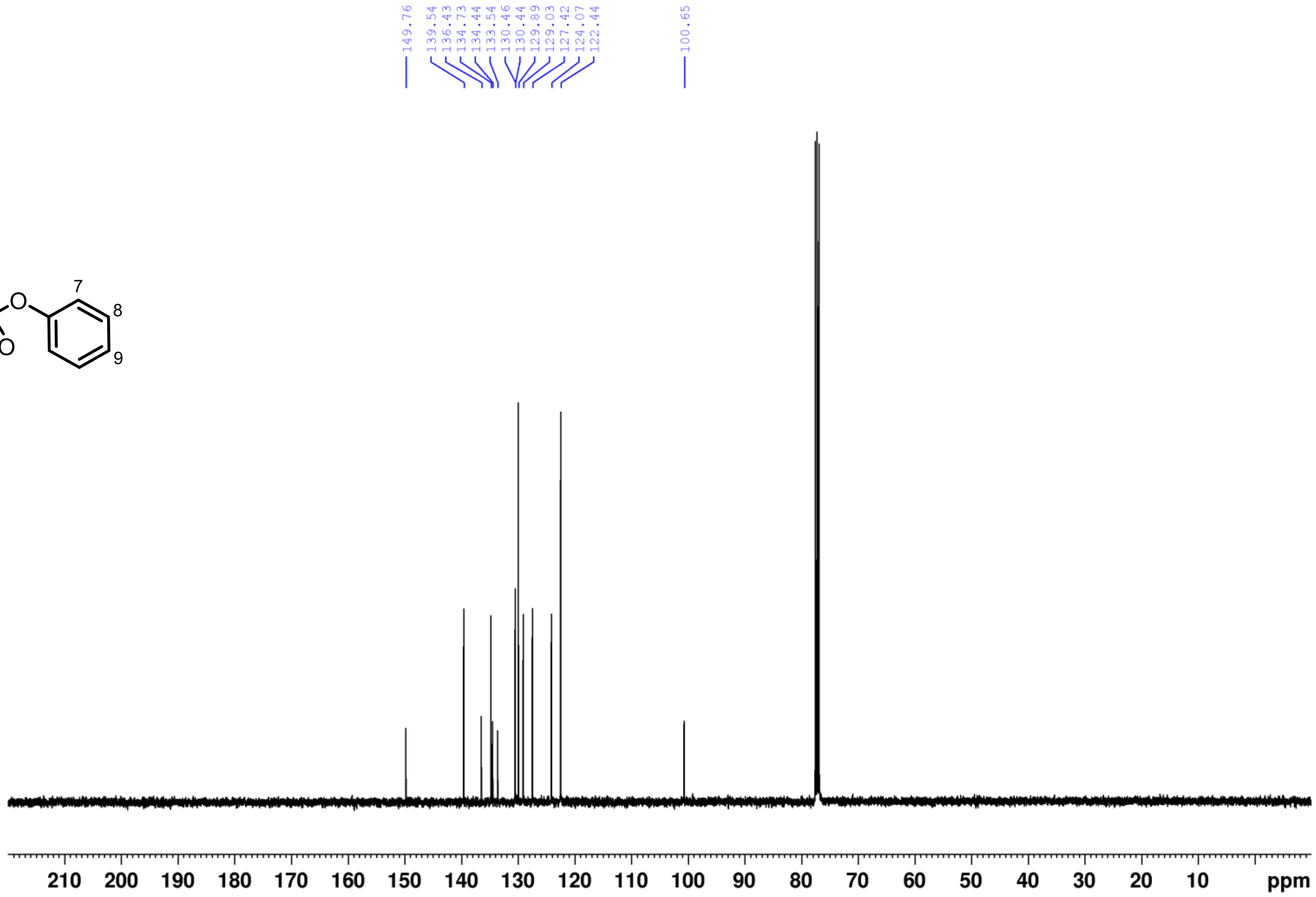

^{1}H NMR (400 MHz, CDCl$_3$) for *dimethyl 3,3'-(5-(7-(phenoxysulfonyl)naphthalen-1-yl)-1,3-phenylene)bis(2,2-dimethylpropanoate)*

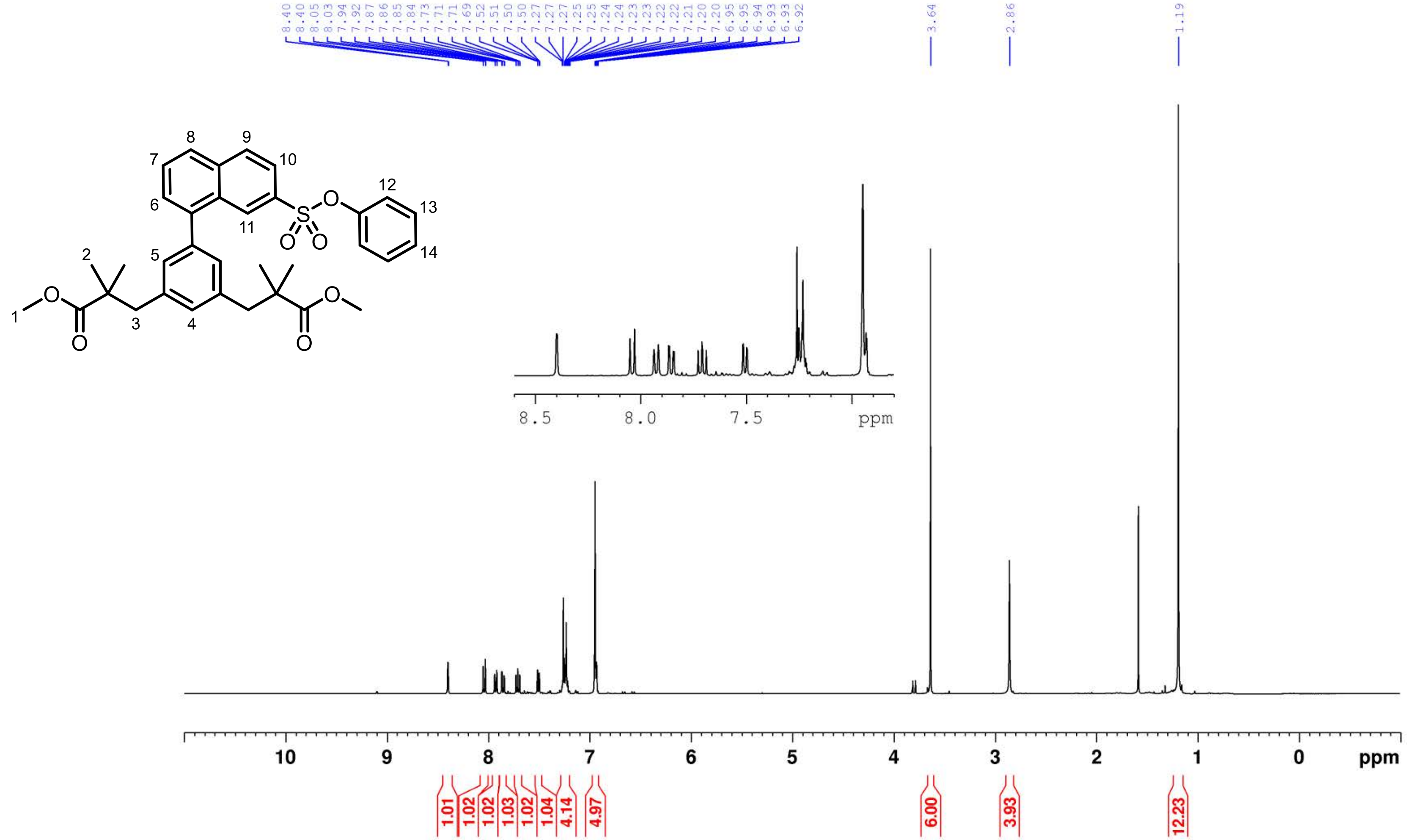

^{13}C NMR (101 MHz, CDCl$_3$) for *dimethyl 3,3'-(5-(7-(phenoxysulfonyl)naphthalen-1-yl)-1,3-phenylene)bis(2,2-dimethylpropanoate)*

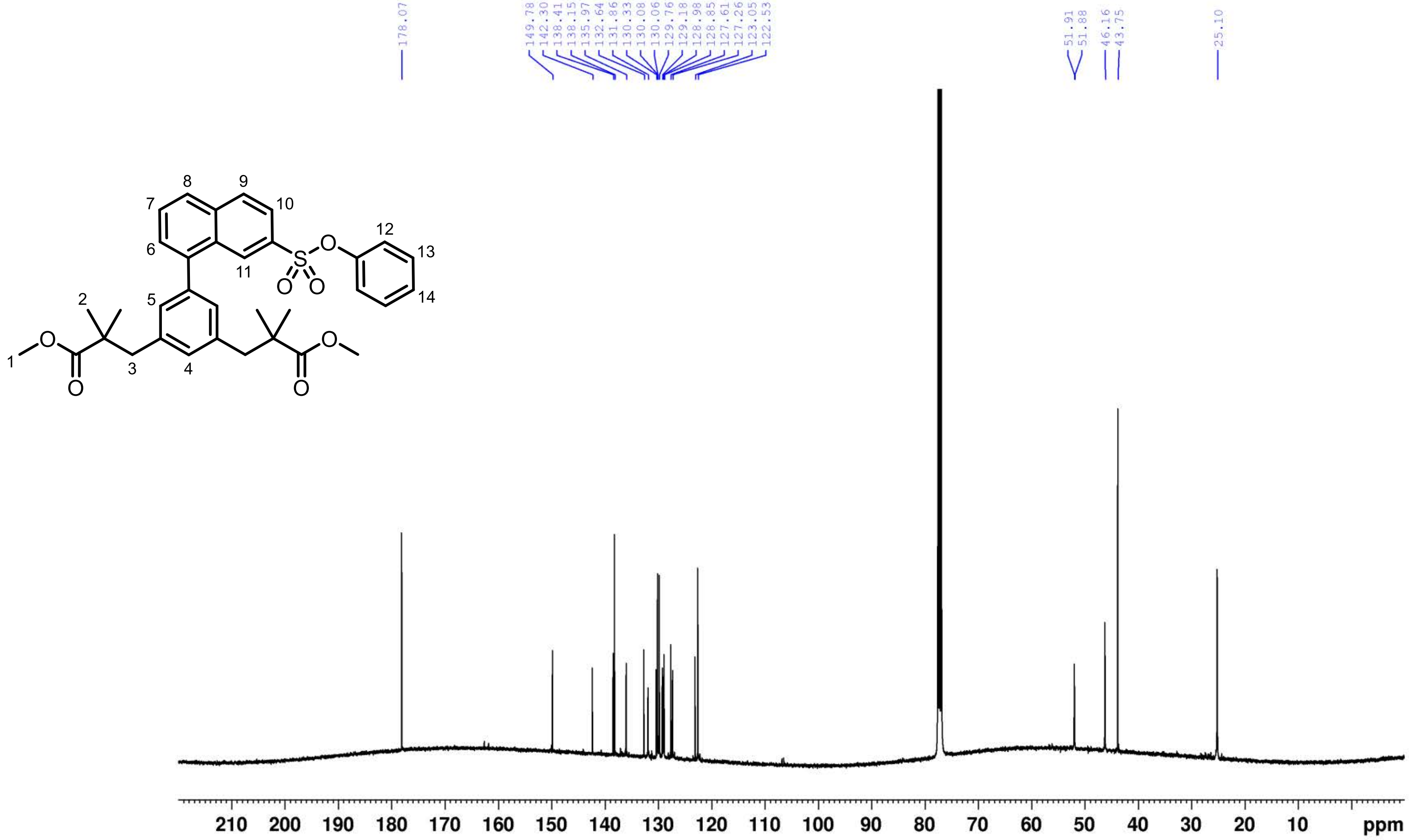

^{1}H NMR (400 MHz, CD_3OD) for *tetrabutylammonium 8-(3,5-bis(2-carboxy-2-methylpropyl)phenyl)naphthalene-2-sulfonate*

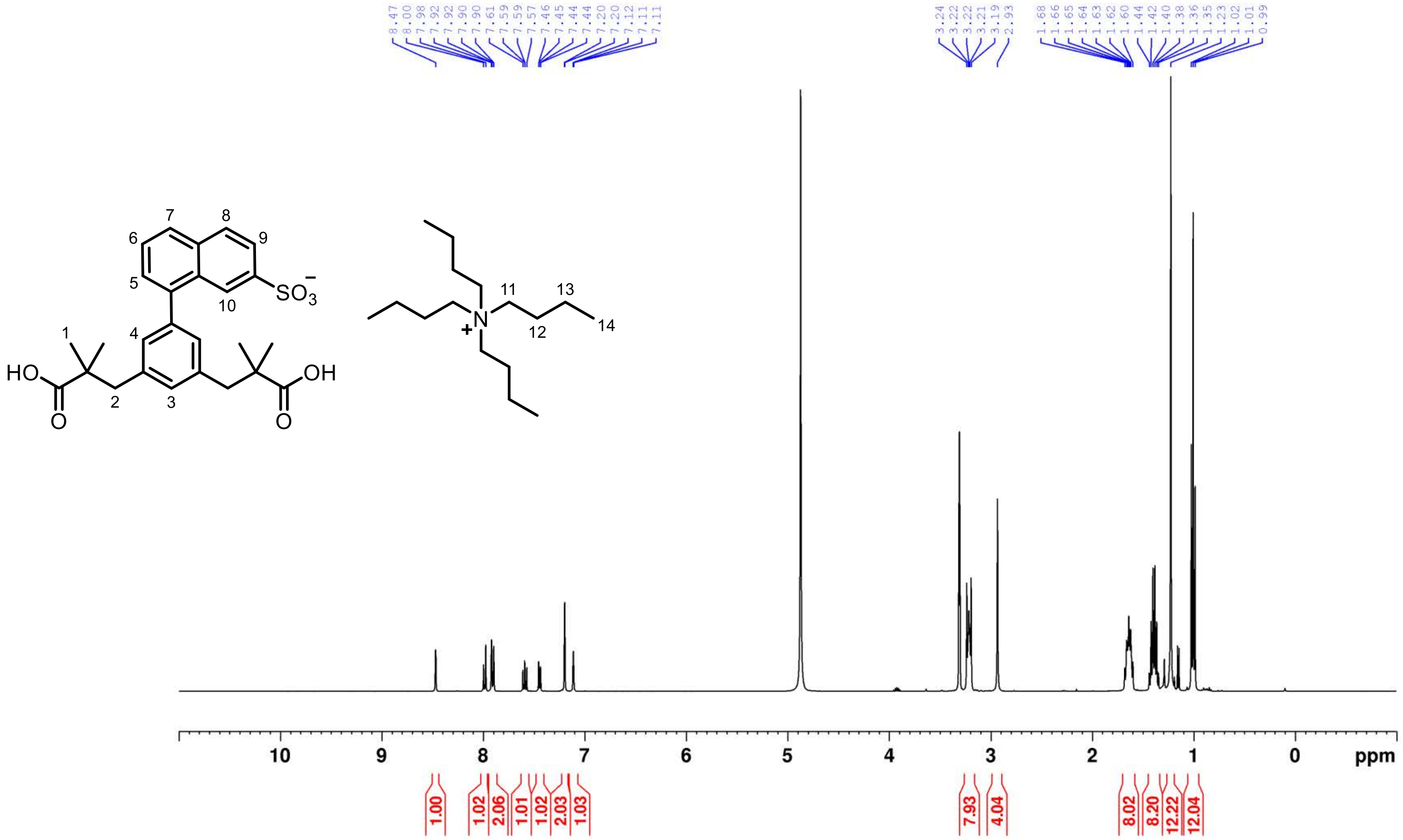

^{13}C NMR (101 MHz, CD_3OD) for *tetrabutylammonium 8-(3,5-bis(2-carboxy-2-methylpropyl)phenyl)naphthalene-2-sulfonate*

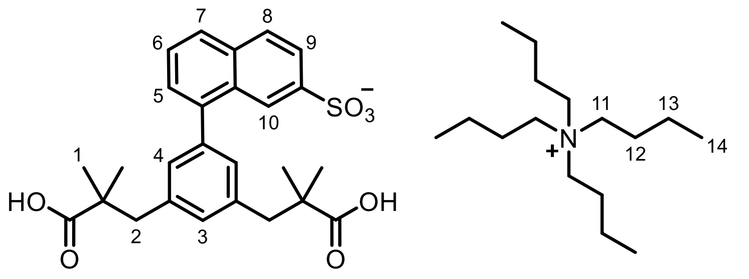

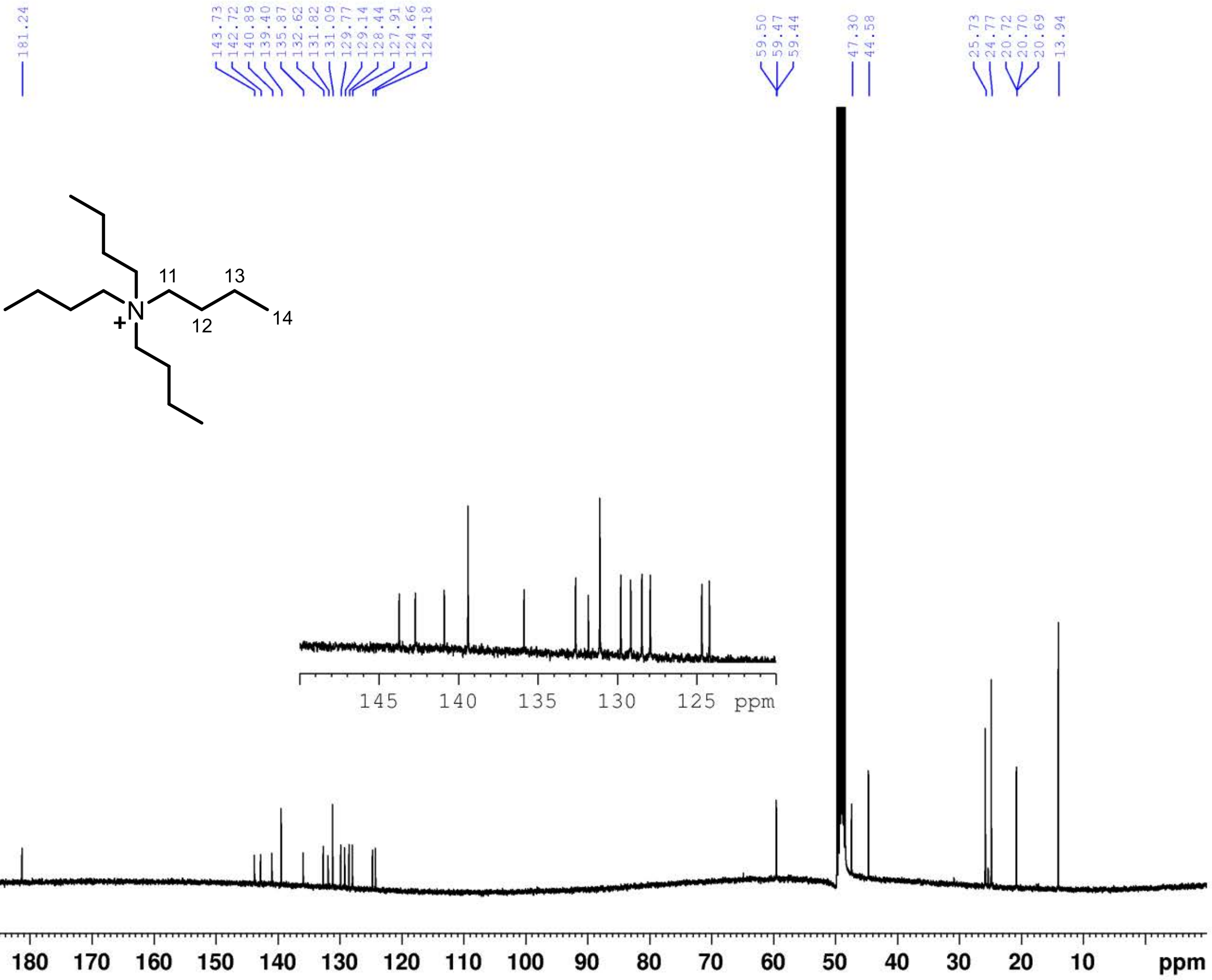

^{1}H NMR (700 MHz, CD$_3$OD) for *bis[rhodium tetrabutylammonium 8-(3,5-bis(2-carboxy-2-methylpropyl)phenyl)naphthalene-2-sulfonate]* (Rh$_2$(**A-V**)$_2$●(**NBu$_4$**)$_2$)

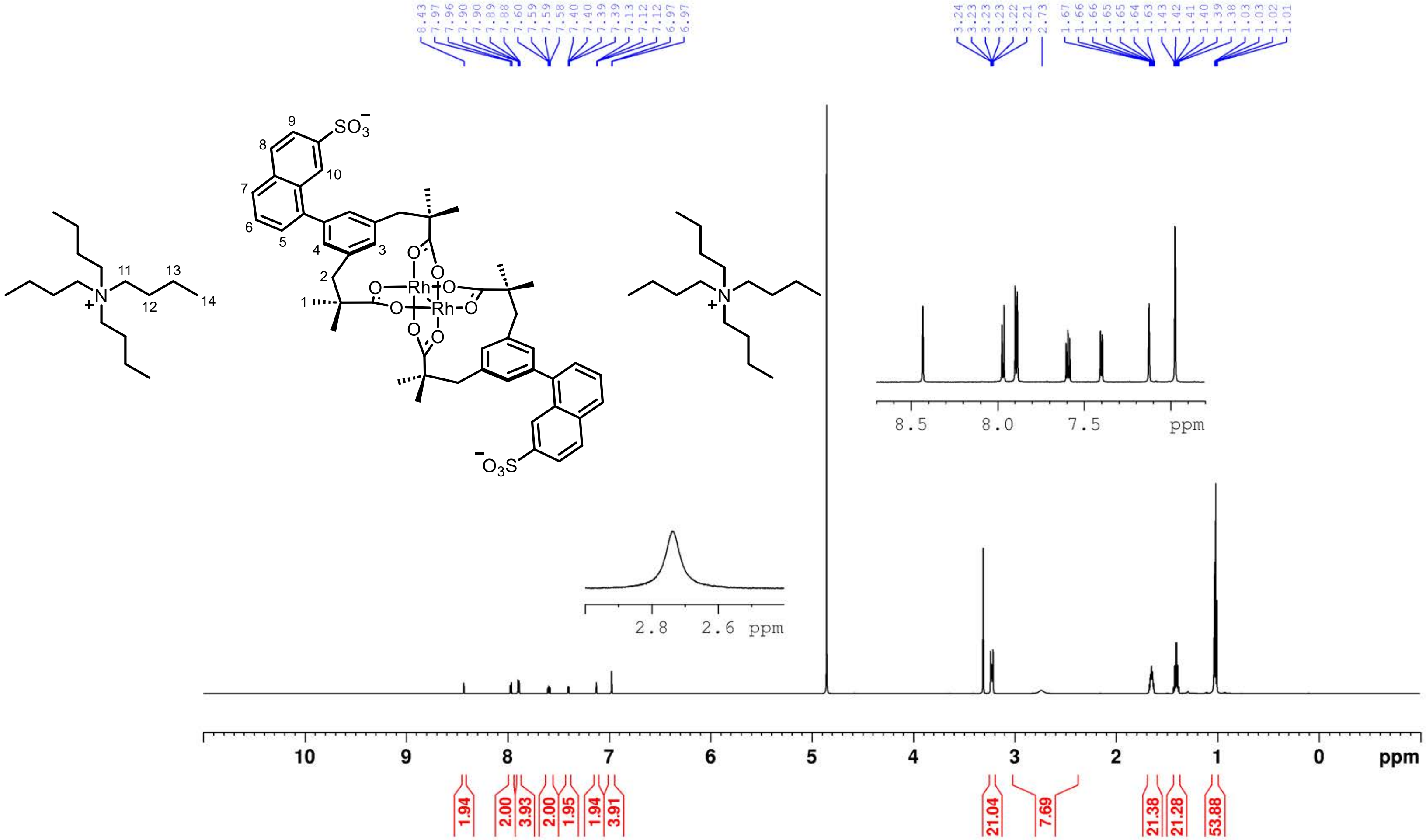

^{13}C NMR (176 MHz, CD_3OD) for *bis[rhodium tetrabutylammonium 8-(3,5-bis(2-carboxy-2-methylpropyl)phenyl)naphthalene-2-sulfonate]* (Rh_2(**A-V**)$_2$●(**NBu_4**)$_2$)

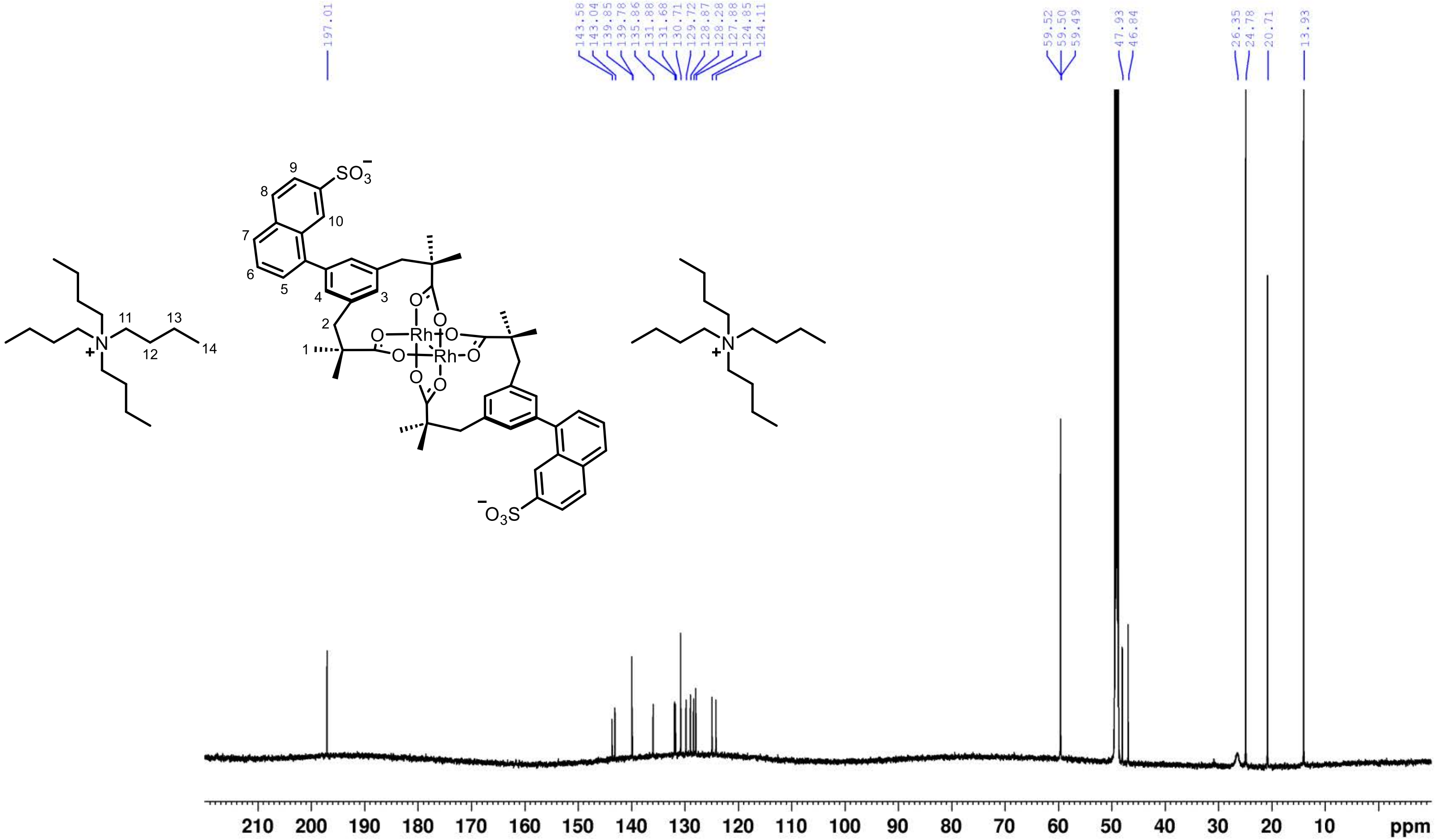

^{1}H NMR (500 MHz, C_5D_5N) for *bis[rhodium (1S,2R,4S,5R)-5-ethyl-2-((S)-hydroxy(6-methoxyquinolin-4-yl)methyl)-1-((3,3'',5,5''-tetra-tert-butyl-[1,1':3',1''-terphenyl]-5'-yl)methyl)quinuclidin-1-ium (3,5-bis(2-carboxy-2-methylpropyl)benzenesulfonate)]* (Rh_2(**A**-**II**)$_2$●(**Cat1**)$_2$)

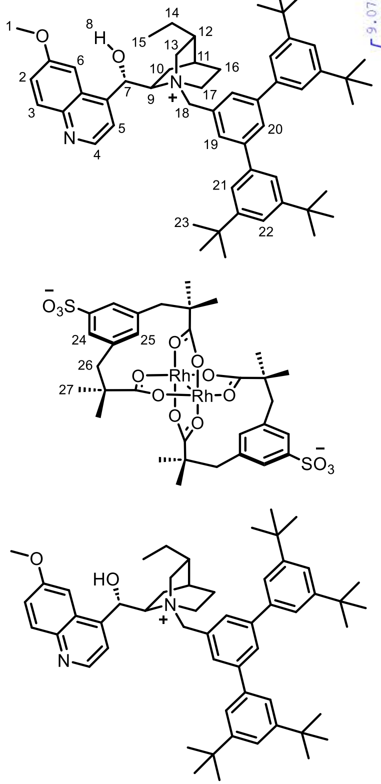

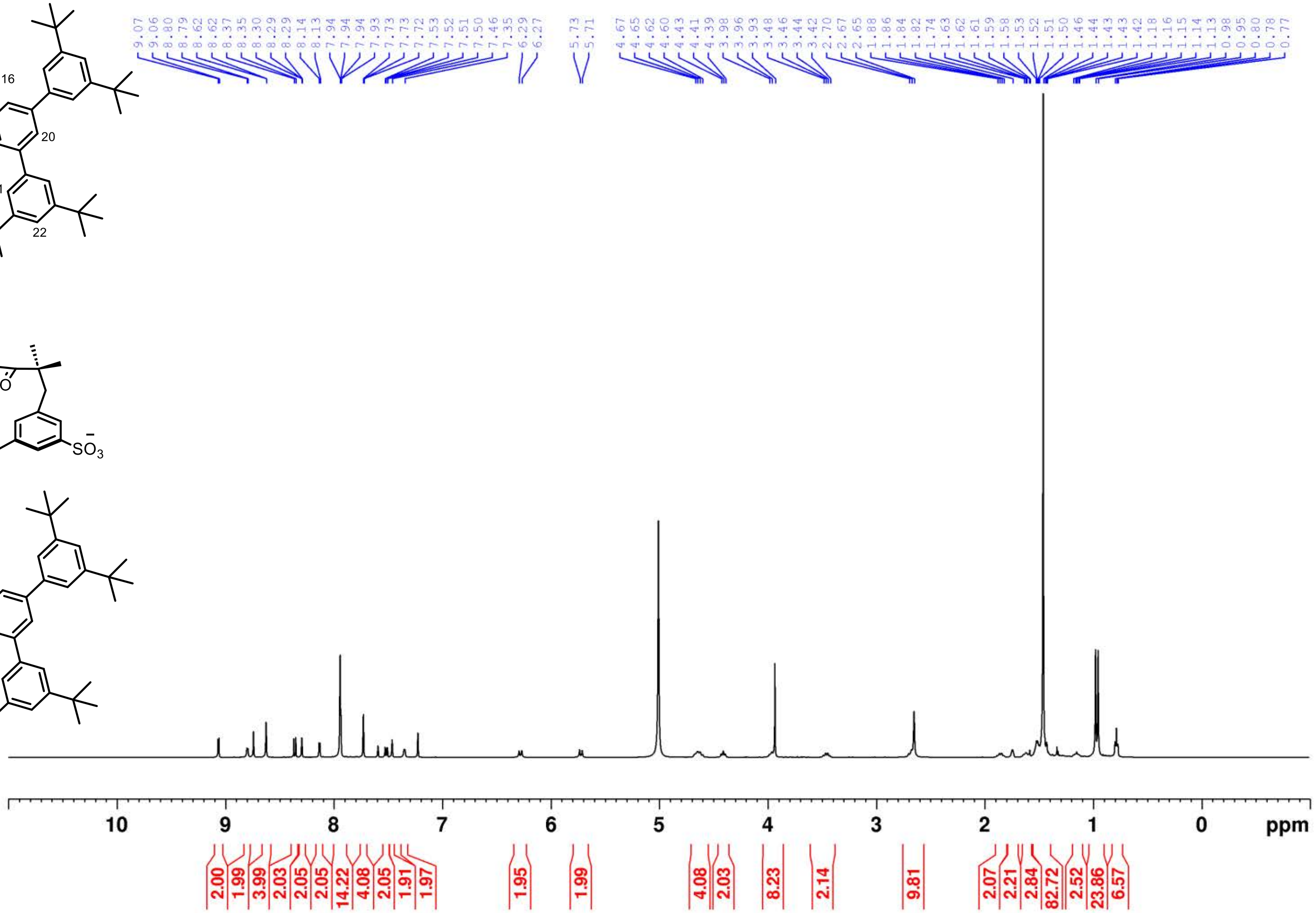

^{13}C NMR (126 MHz, C_5D_5N) for *bis[rhodium (1S,2R,4S,5R)-5-ethyl-2-((S)-hydroxy(6-methoxyquinolin-4-yl)methyl)-1-((3,3'',5,5''-tetra-tert-butyl-[1,1':3',1''-terphenyl]-5'-yl)methyl)quinuclidin-1-ium (3,5-bis(2-carboxy-2-methylpropyl)benzenesulfonate)]* (Rh_2(**A-II**)$_2$●(**Cat1**)$_2$)

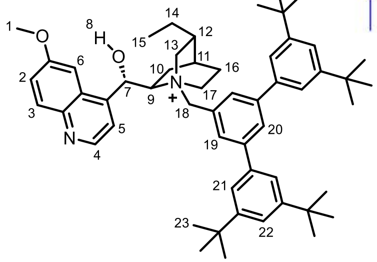

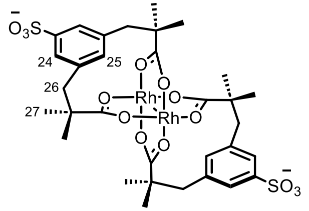

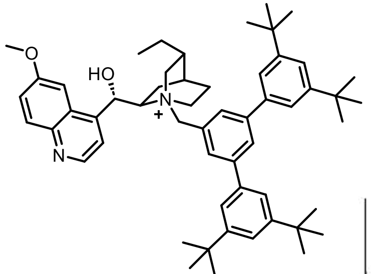

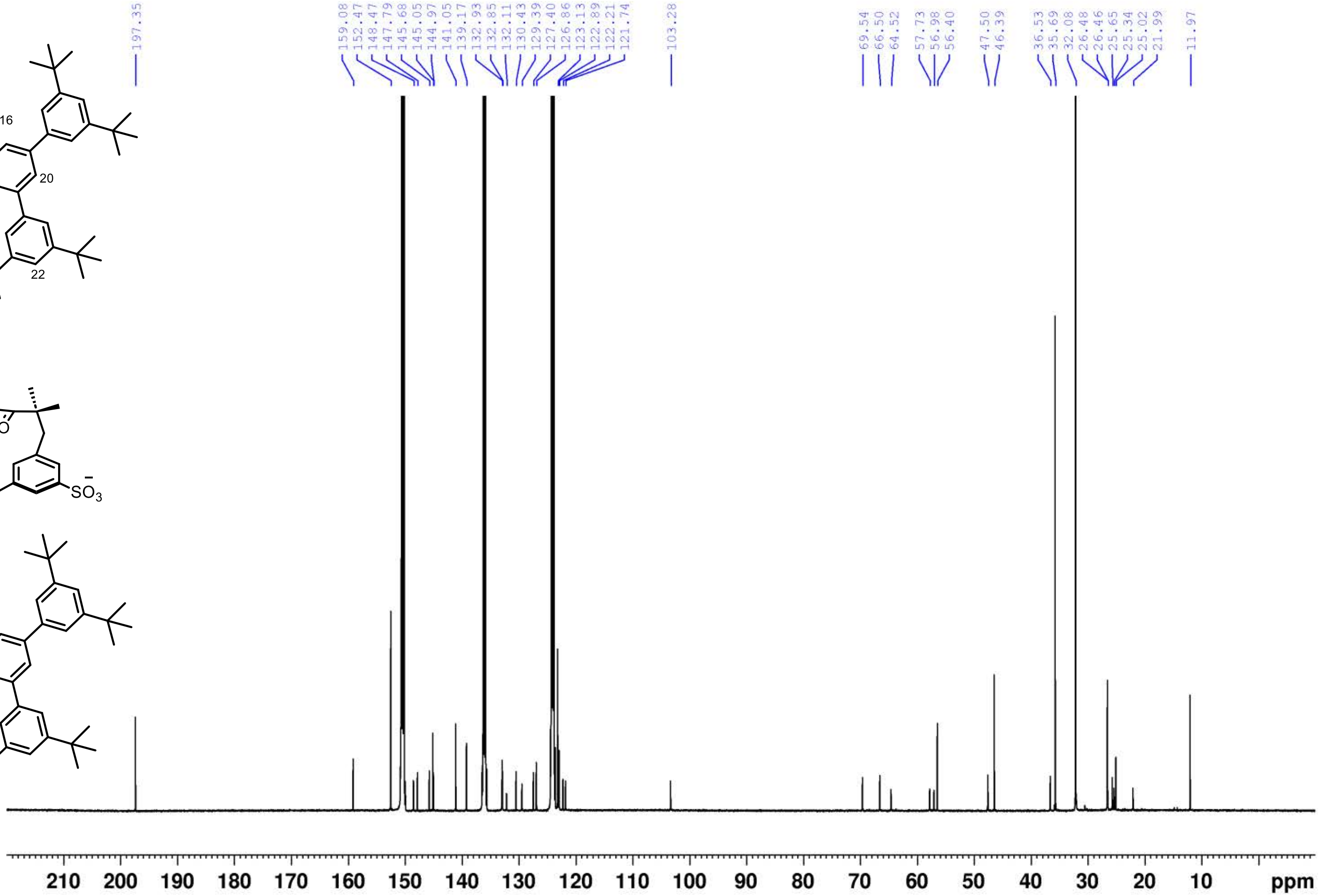

^{1}H NMR (700 MHz, C_5D_5N) for *bis[rhodium (1S,2S,4S,5R)-5-ethyl-2-((R)-hydroxy(6-methoxyquinolin-4-yl)methyl)-1-((3,3'',5,5''-tetra-tert-butyl-[1,1':3',1''-terphenyl]-5'-yl)methyl)quinuclidin-1-ium (3,5-bis(2-carboxy-2-methylpropyl)benzenesulfonate)]* (Rh_2(**A**-**II**)$_2$●(**Cat2**)$_2$)

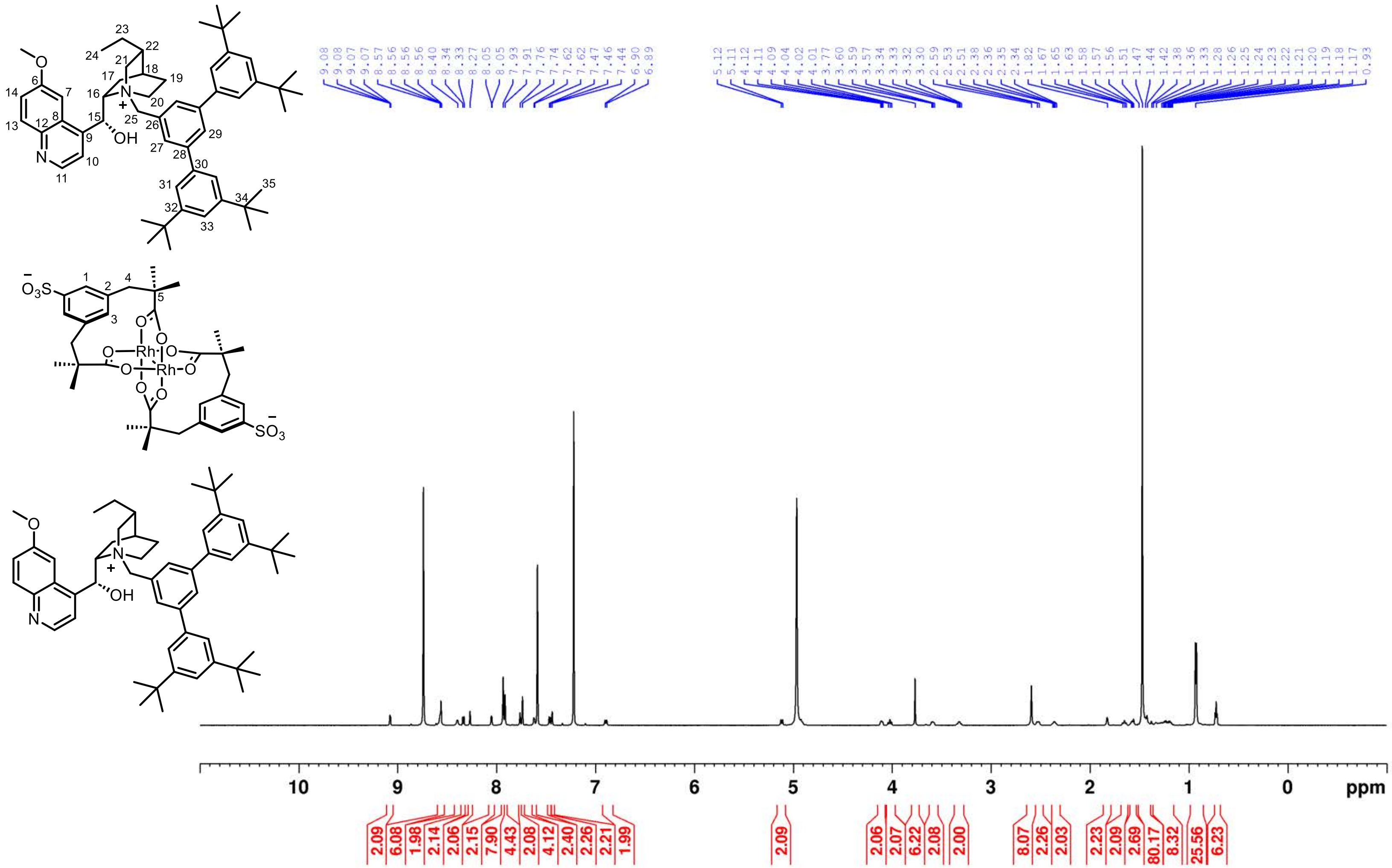

^{13}C NMR (176 MHz, C_5D_5N) for *bis[rhodium (1S,2S,4S,5R)-5-ethyl-2-((R)-hydroxy(6-methoxyquinolin-4-yl)methyl)-1-((3,3'',5,5''-tetra-tert-butyl-[1,1':3',1''-terphenyl]-5'-yl)methyl)quinuclidin-1-ium (3,5-bis(2-carboxy-2-methylpropyl)benzenesulfonate)]* (Rh_2(**A-II**)$_2$●(**Cat2**)$_2$)

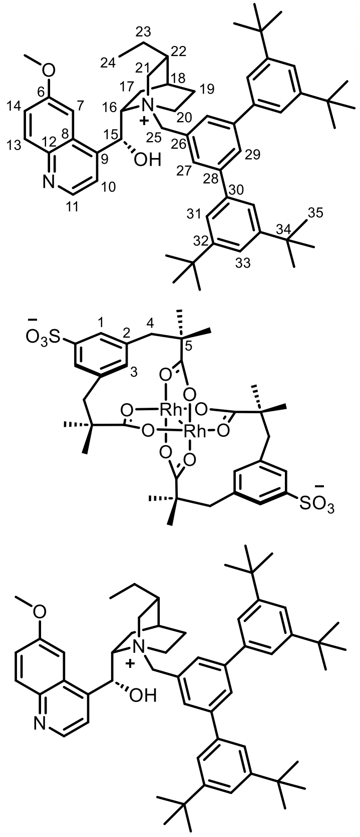

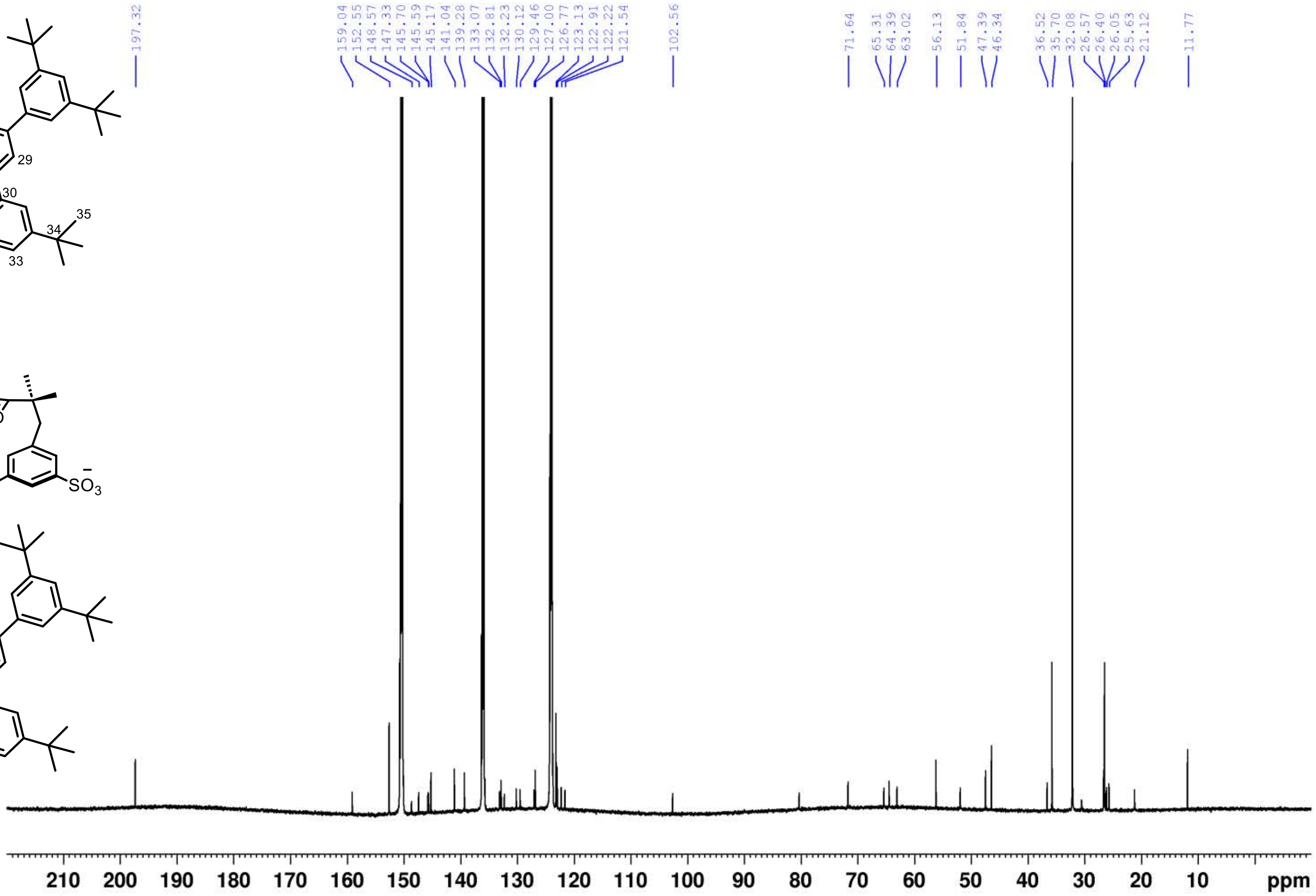

^{1}H NMR (400 MHz, C_5D_5N) for *bis[rhodium (1S,2R,4S,5R)-5-ethyl-2-((S)-hydroxy(6-methoxyquinolin-4-yl)methyl)-1-((3,3'',5,5''-tetra-tert-butyl-[1,1':3',1''-terphenyl]-5'-yl)methyl)quinuclidin-1-ium (3',5'-bis(2-carboxy-2-methylpropyl)-[1,1'-biphenyl]-2-sulfonate)]* (Rh_2(**A-III**)$_2$●(**Cat1**)$_2$)

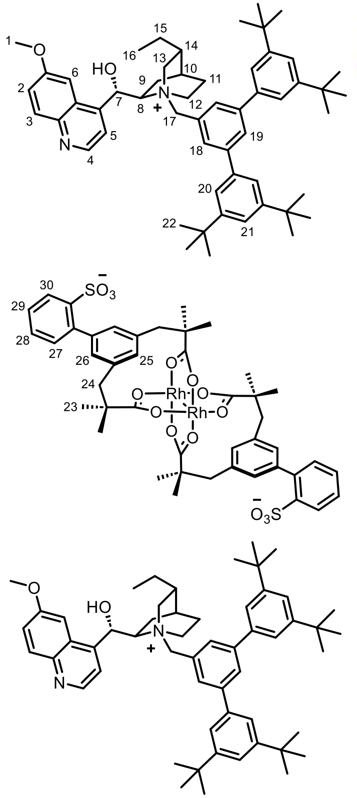

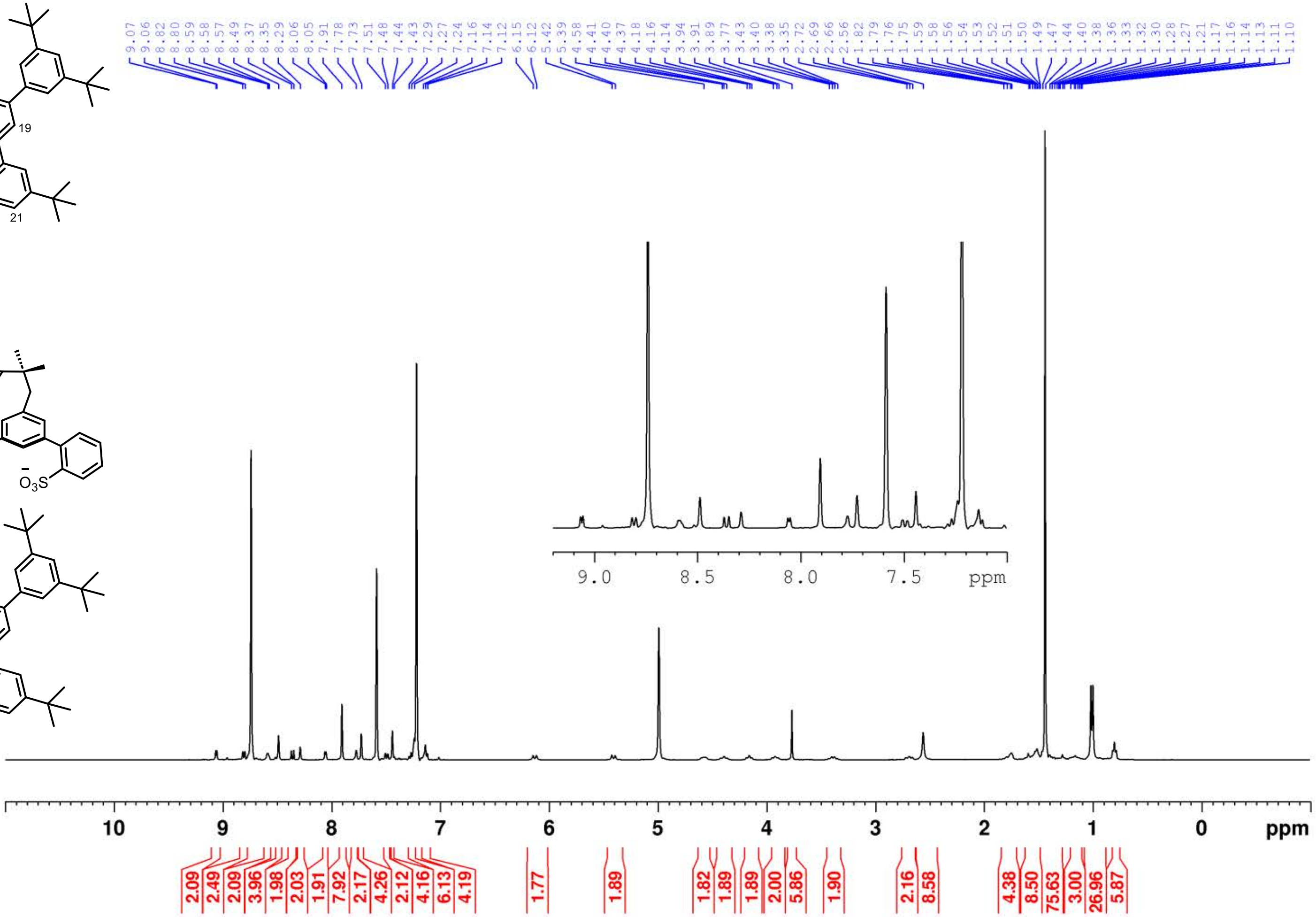

^{13}C NMR (101 MHz, C_5D_5N) for *bis[rhodium (1S,2R,4S,5R)-5-ethyl-2-((S)-hydroxy(6-methoxyquinolin-4-yl)methyl)-1-((3,3'',5,5''-tetra-tert-butyl-[1,1':3',1''-terphenyl]-5'-yl)methyl)quinuclidin-1-ium (3',5'-bis(2-carboxy-2-methylpropyl)-[1,1'-biphenyl]-2-sulfonate)]* (Rh_2(**A-III**)$_2$●(**Cat1**)$_2$)

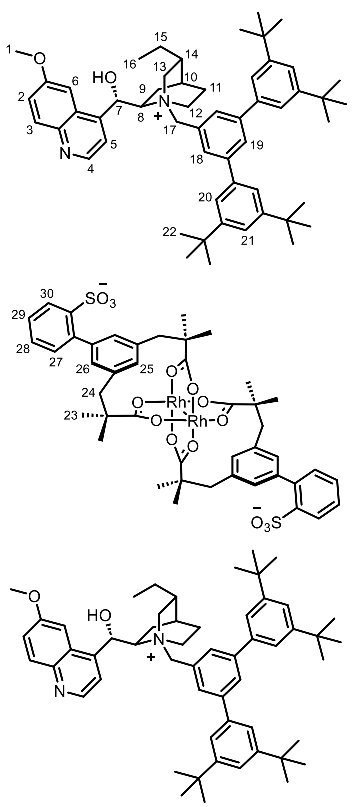

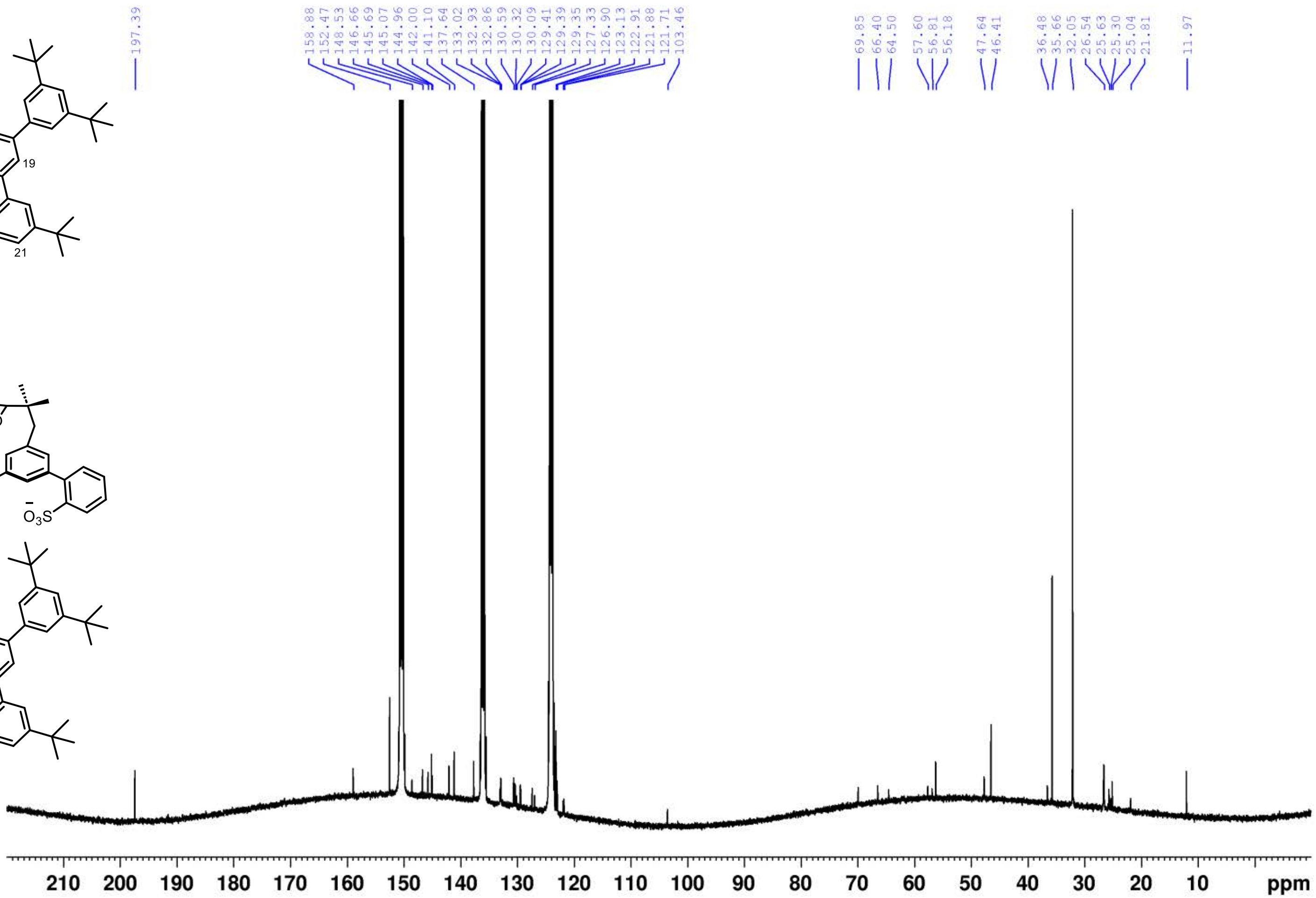

^{1}H NMR (500 MHz, C_5D_5N) for *bis[rhodium (1S,2S,4S,5R)-5-ethyl-2-((R)-hydroxy(6-methoxyquinolin-4-yl)methyl)-1-((3,3'',5,5''-tetra-tert-butyl-[1,1':3',1''-terphenyl]-5'-yl)methyl)quinuclidin-1-ium (3',5'-bis(2-carboxy-2-methylpropyl)-[1,1'-biphenyl]-2-sulfonate)]* (Rh_2(**A-III**)$_2$●(**Cat2**)$_2$)

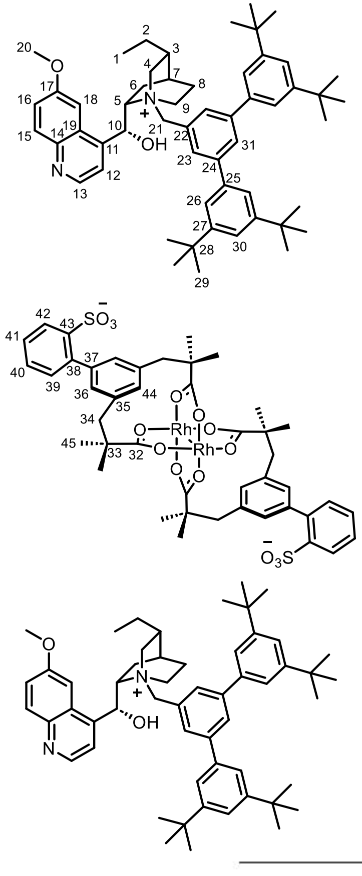

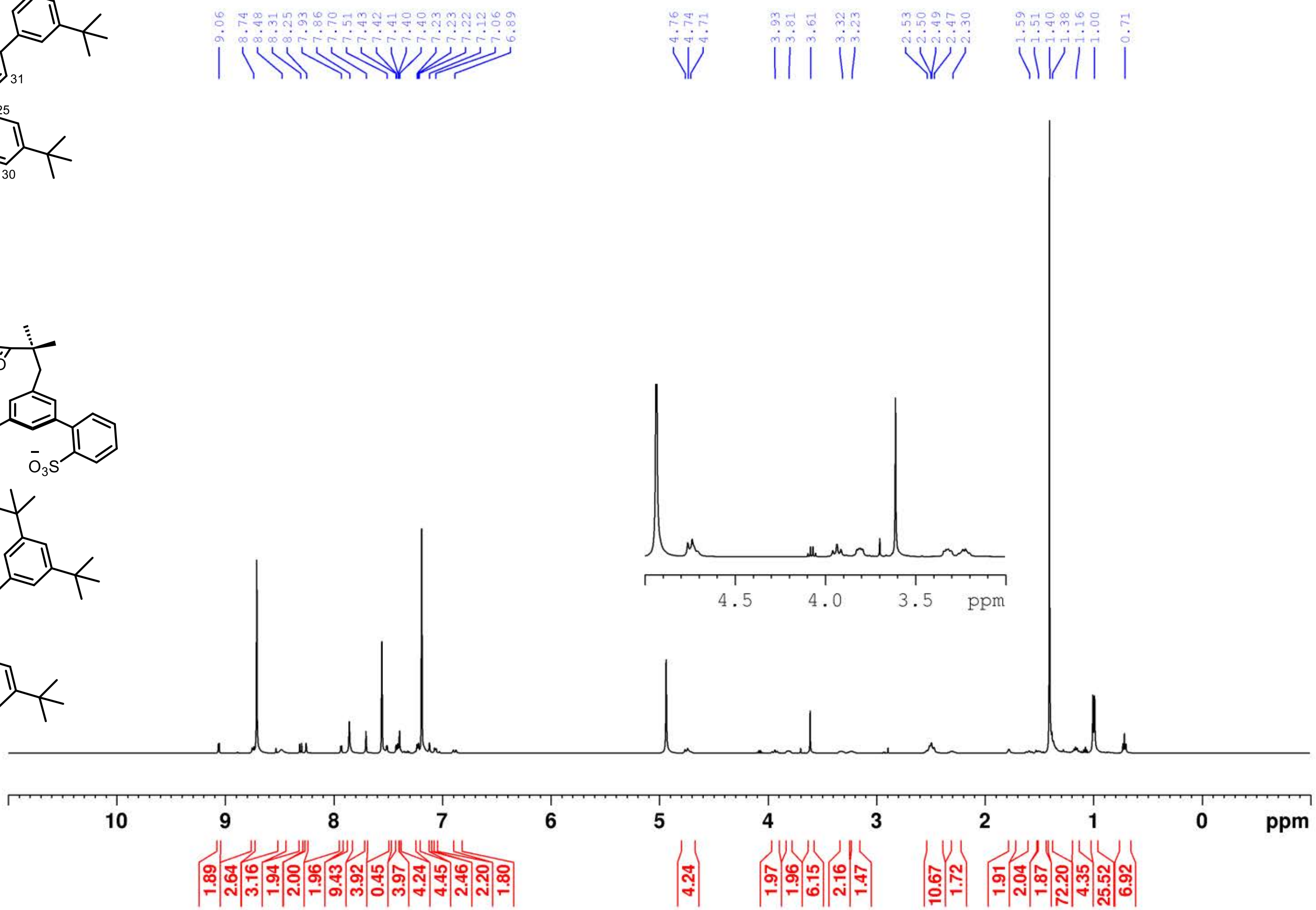

^{13}C NMR (126 MHz, C_5D_5N) for *bis[rhodium (1S,2S,4S,5R)-5-ethyl-2-((R)-hydroxy(6-methoxyquinolin-4-yl)methyl)-1-((3,3'',5,5''-tetra-tert-butyl-[1,1':3',1''-terphenyl]-5'-yl)methyl)quinuclidin-1-ium (3',5'-bis(2-carboxy-2-methylpropyl)-[1,1'-biphenyl]-2-sulfonate)]* (Rh_2(**A**-**III**)$_2$●(**Cat2**)$_2$)

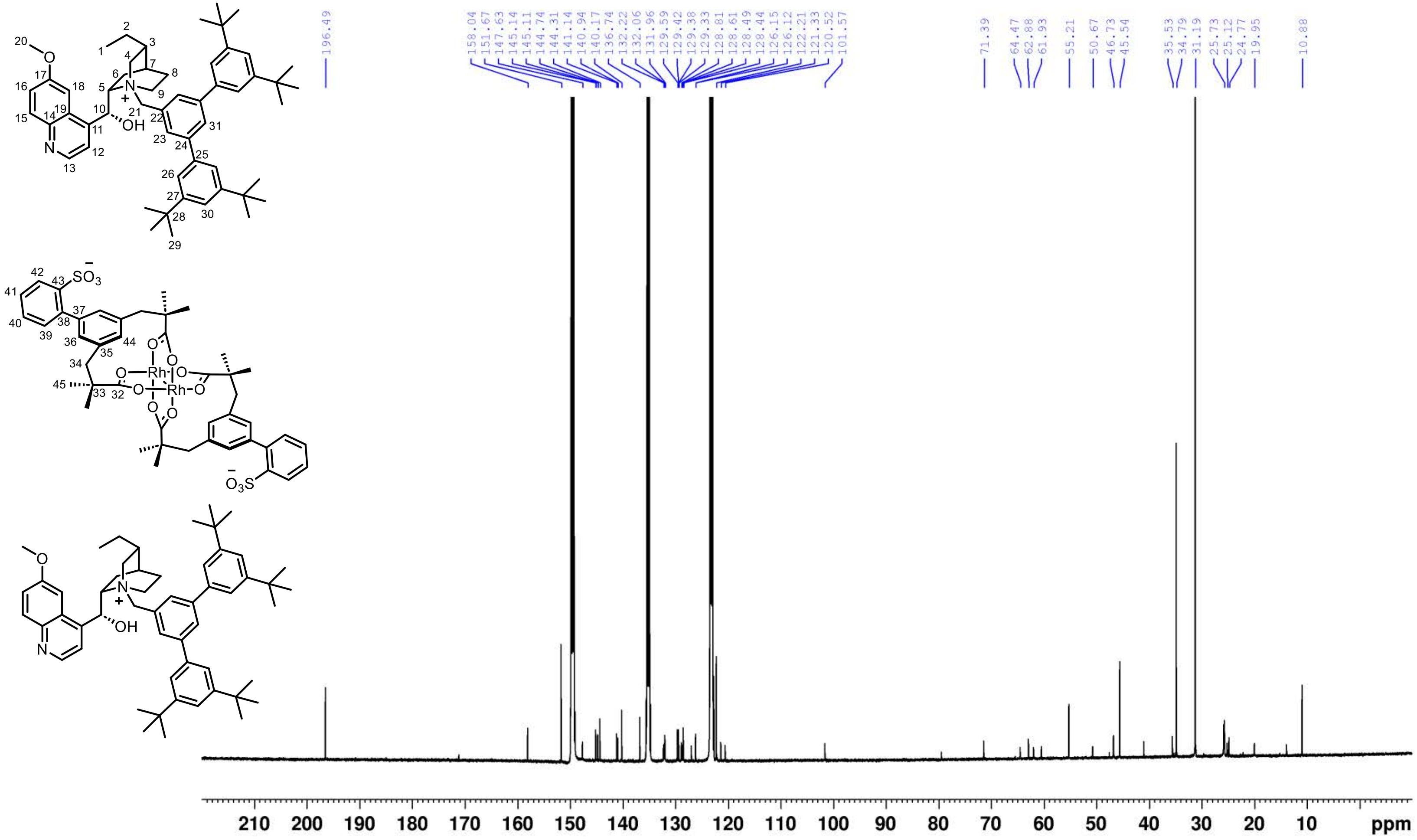

^{1}H NMR (500 MHz, C_5D_5N) for *bis[rhodium (1S,2R,4S,5R)-5-ethyl-2-((S)-hydroxy(6-methoxyquinolin-4-yl)methyl)-1-((3,3'',5,5''-tetra-tert-butyl-[1,1':3',1''-terphenyl]-5'-yl)methyl)quinuclidin-1-ium (3',5'-bis((1-carboxycyclobutyl)methyl)-[1,1'-biphenyl]-2-sulfonate)]* (Rh_2(**B**-**III**)$_2$●(**Cat1**)$_2$)

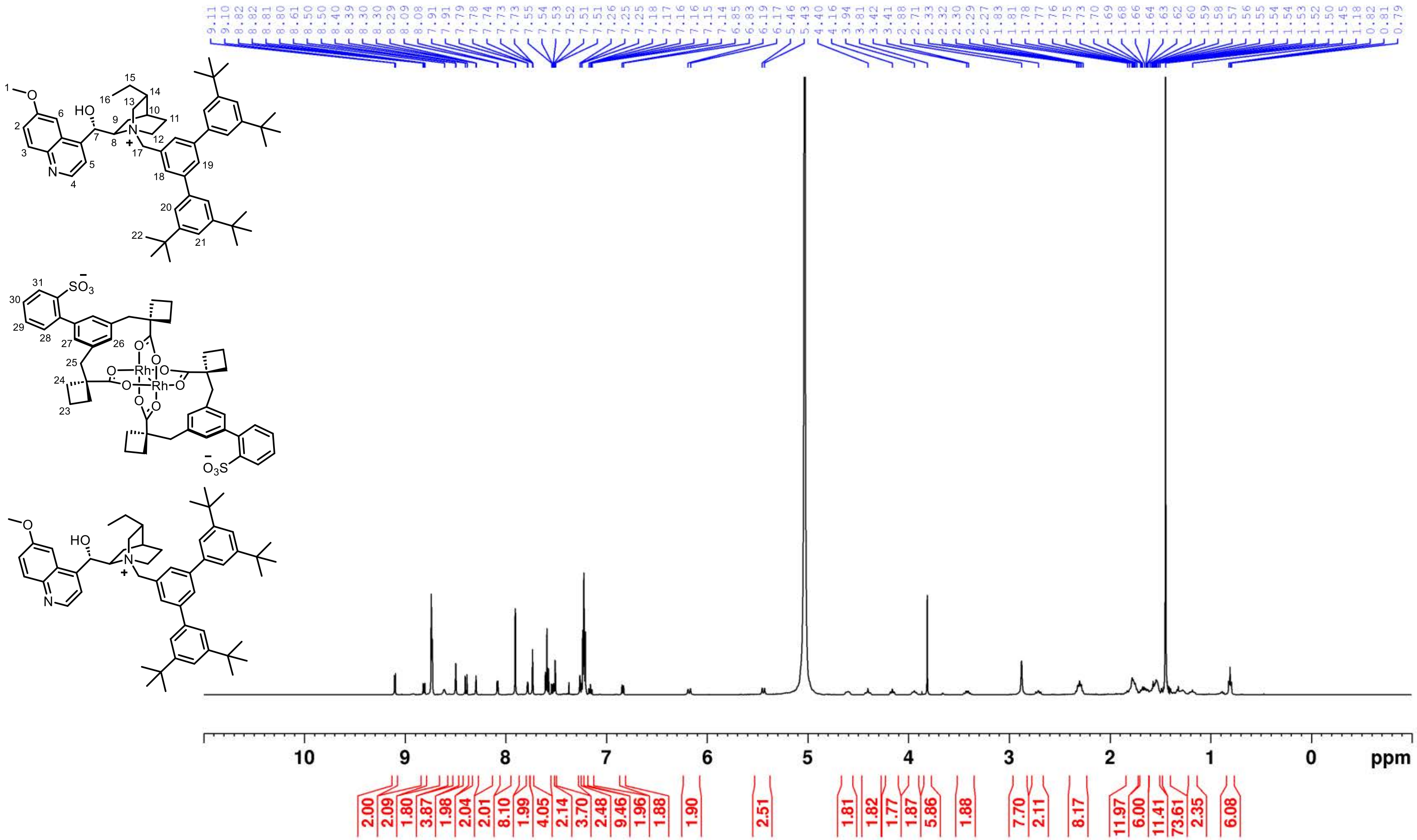

^{13}C NMR (126 MHz, C_5D_5N) for *bis[rhodium (1S,2R,4S,5R)-5-ethyl-2-((S)-hydroxy(6-methoxyquinolin-4-yl)methyl)-1-((3,3'',5,5''-tetra-tert-butyl-[1,1':3',1''-terphenyl]-5'-yl)methyl)quinuclidin-1-ium (3',5'-bis((1-carboxycyclobutyl)methyl)-[1,1'-biphenyl]-2-sulfonate)]* (Rh_2(**B-III**)$_2$●(**Cat1**)$_2$)

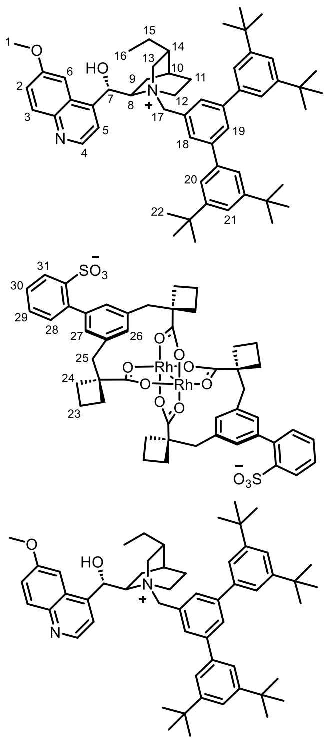

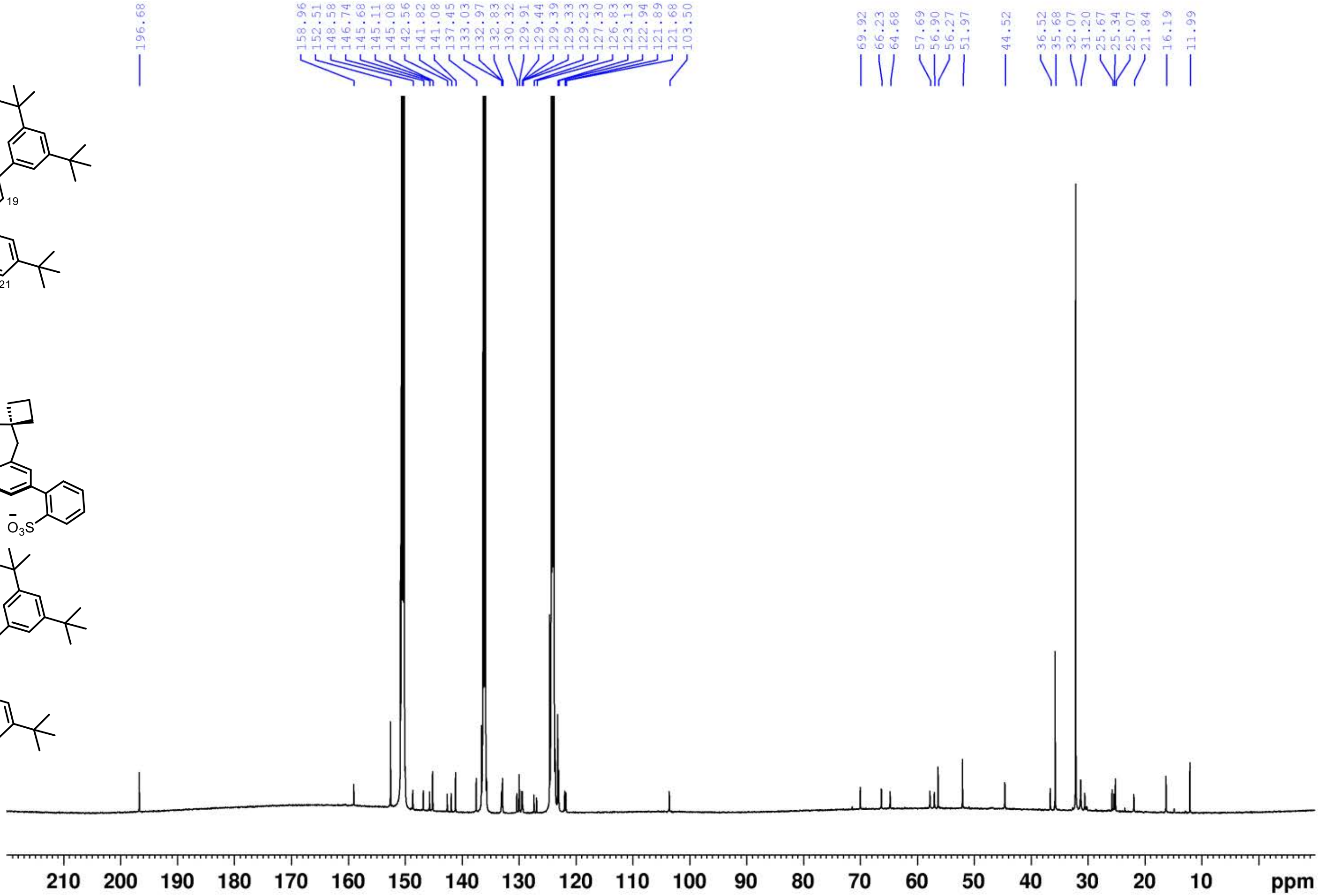

^{1}H NMR (500 MHz, C_5D_5N) for *bis[rhodium (1S,2R,4S,5R)-5-ethyl-2-((S)-hydroxy(6-methoxyquinolin-4-yl)methyl)-1-((3,3'',5,5''-tetra-tert-butyl-[1,1':3',1''-terphenyl]-5'-yl)methyl)quinuclidin-1-ium (3',5'-bis((1-carboxycyclopentyl)methyl)-[1,1'-biphenyl]-2-sulfonate)]* (Rh_2(**C-III**)$_2$●(**Cat1**)$_2$)

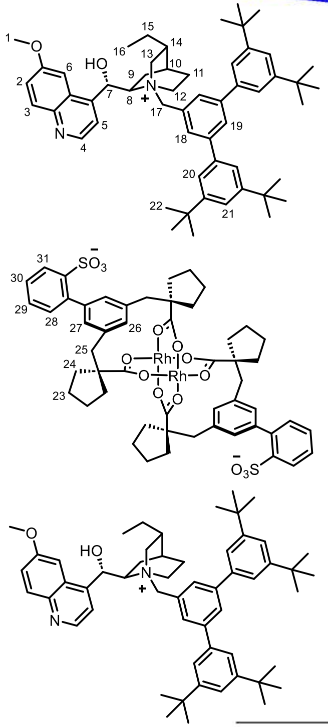

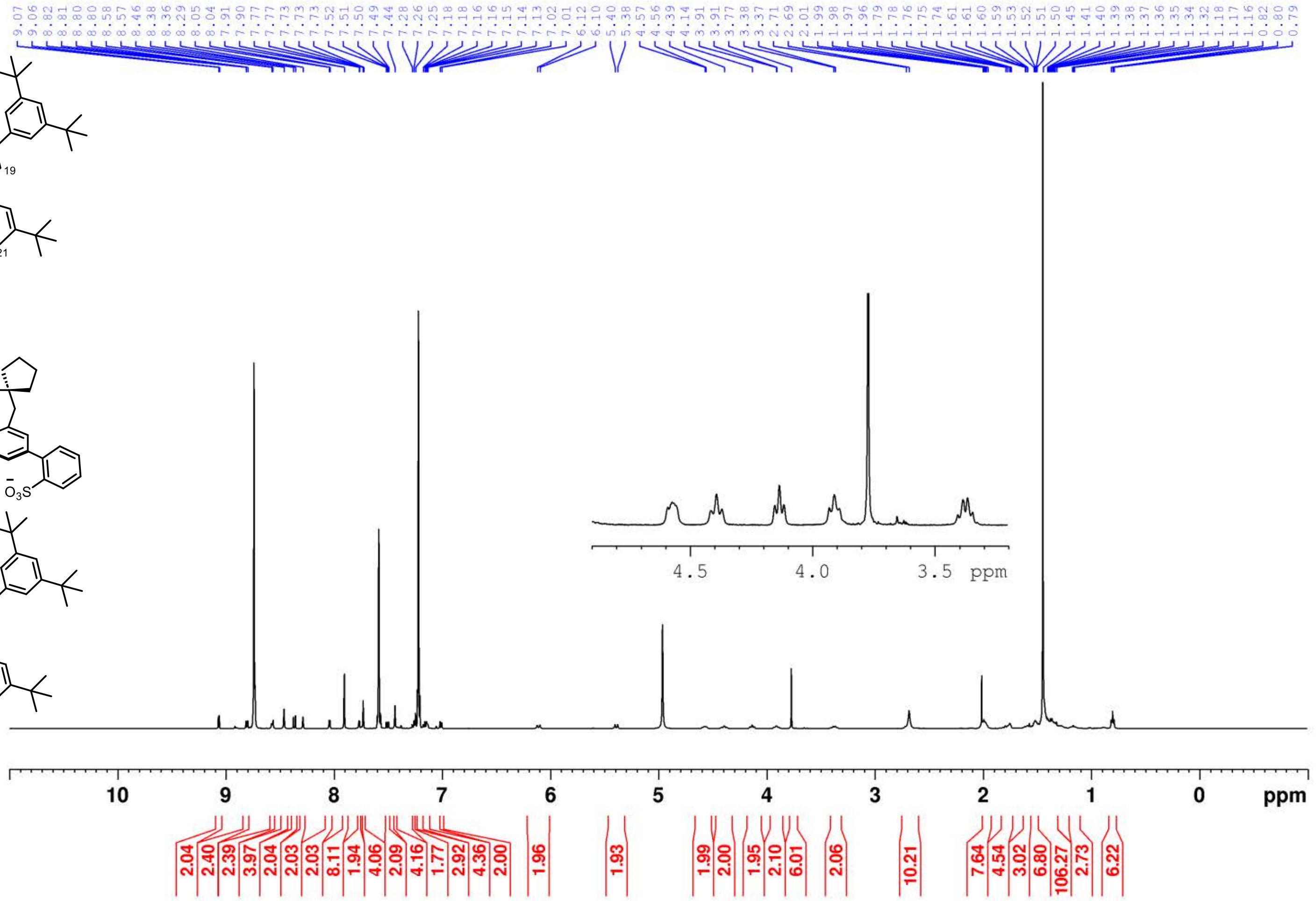

^{13}C NMR (126 MHz, C_5D_5N) for *bis[rhodium (1S,2R,4S,5R)-5-ethyl-2-((S)-hydroxy(6-methoxyquinolin-4-yl)methyl)-1-((3,3'',5,5''-tetra-tert-butyl-[1,1':3',1''-terphenyl]-5'-yl)methyl)quinuclidin-1-ium (3',5'-bis((1-carboxycyclopentyl)methyl)-[1,1'-biphenyl]-2-sulfonate)]* (Rh_2(**C-III**)$_2$●(**Cat1**)$_2$)

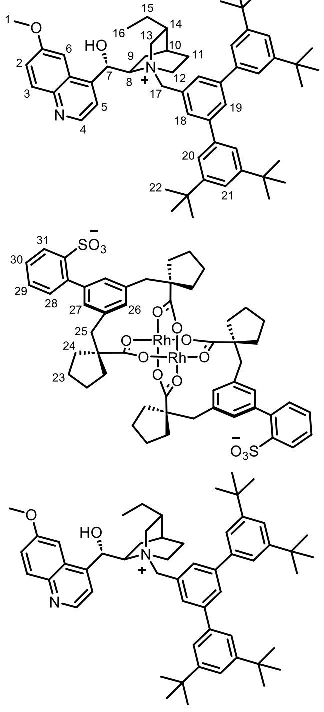

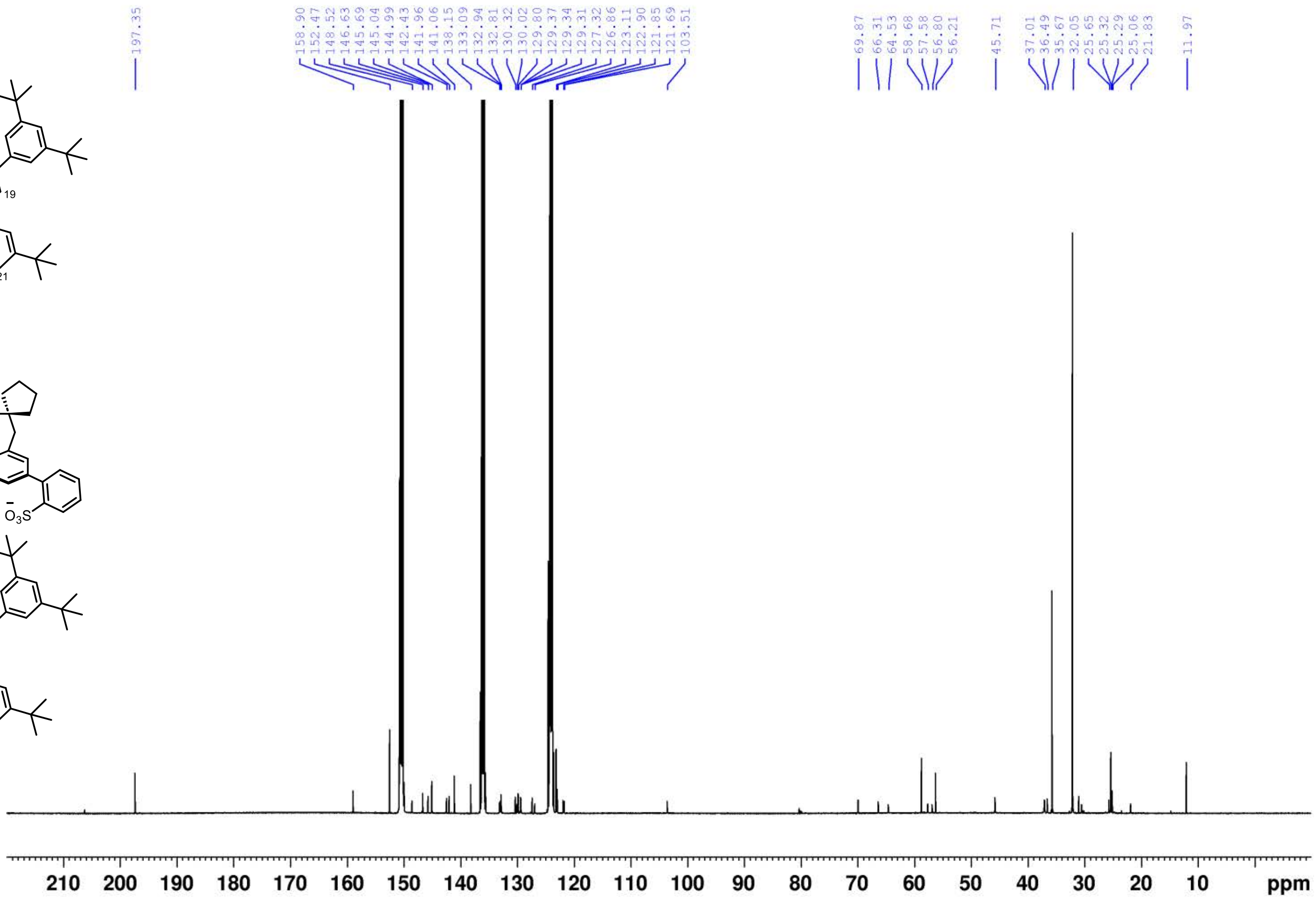

^{1}H NMR (500 MHz, C_5D_5N) for *bis[rhodium (1S,2R,4S,5R)-5-ethyl-2-((S)-hydroxy(6-methoxyquinolin-4-yl)methyl)-1-((3,3'',5,5''-tetra-tert-butyl-[1,1':3',1''-terphenyl]-5'-yl)methyl)quinuclidin-1-ium (3',5'-bis((1-carboxycycloheptyl)methyl)-[1,1'-biphenyl]-2-sulfonate)]* (Rh_2(**D-III**)$_2$●(**Cat1**)$_2$)

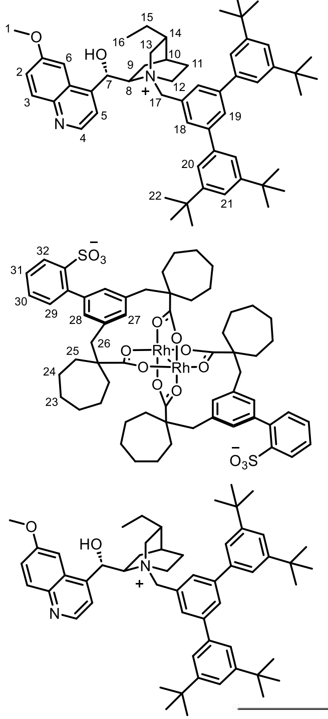

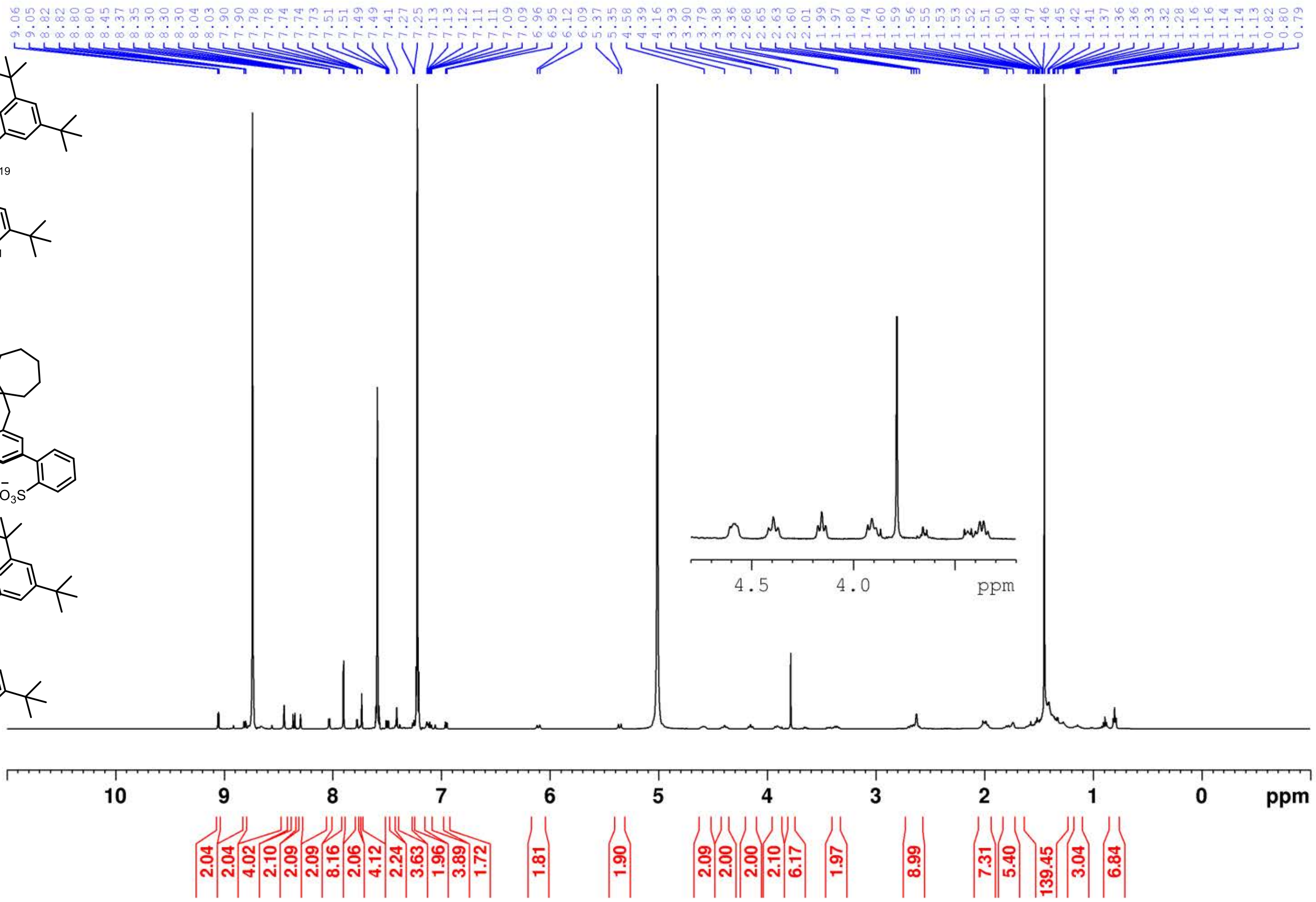

^{13}C NMR (126 MHz, C_5D_5N) for *bis[rhodium (1S,2R,4S,5R)-5-ethyl-2-((S)-hydroxy(6-methoxyquinolin-4-yl)methyl)-1-((3,3'',5,5''-tetra-tert-butyl-[1,1':3',1''-terphenyl]-5'-yl)methyl)quinuclidin-1-ium (3',5'-bis((1-carboxycycloheptyl)methyl)-[1,1'-biphenyl]-2-sulfonate)]* (Rh_2(**D-III**)$_2$●(**Cat1**)$_2$)

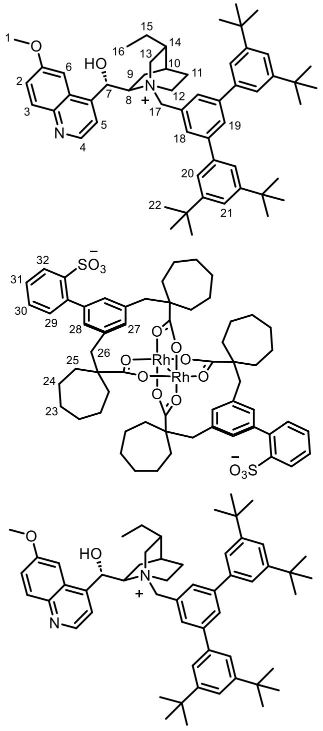

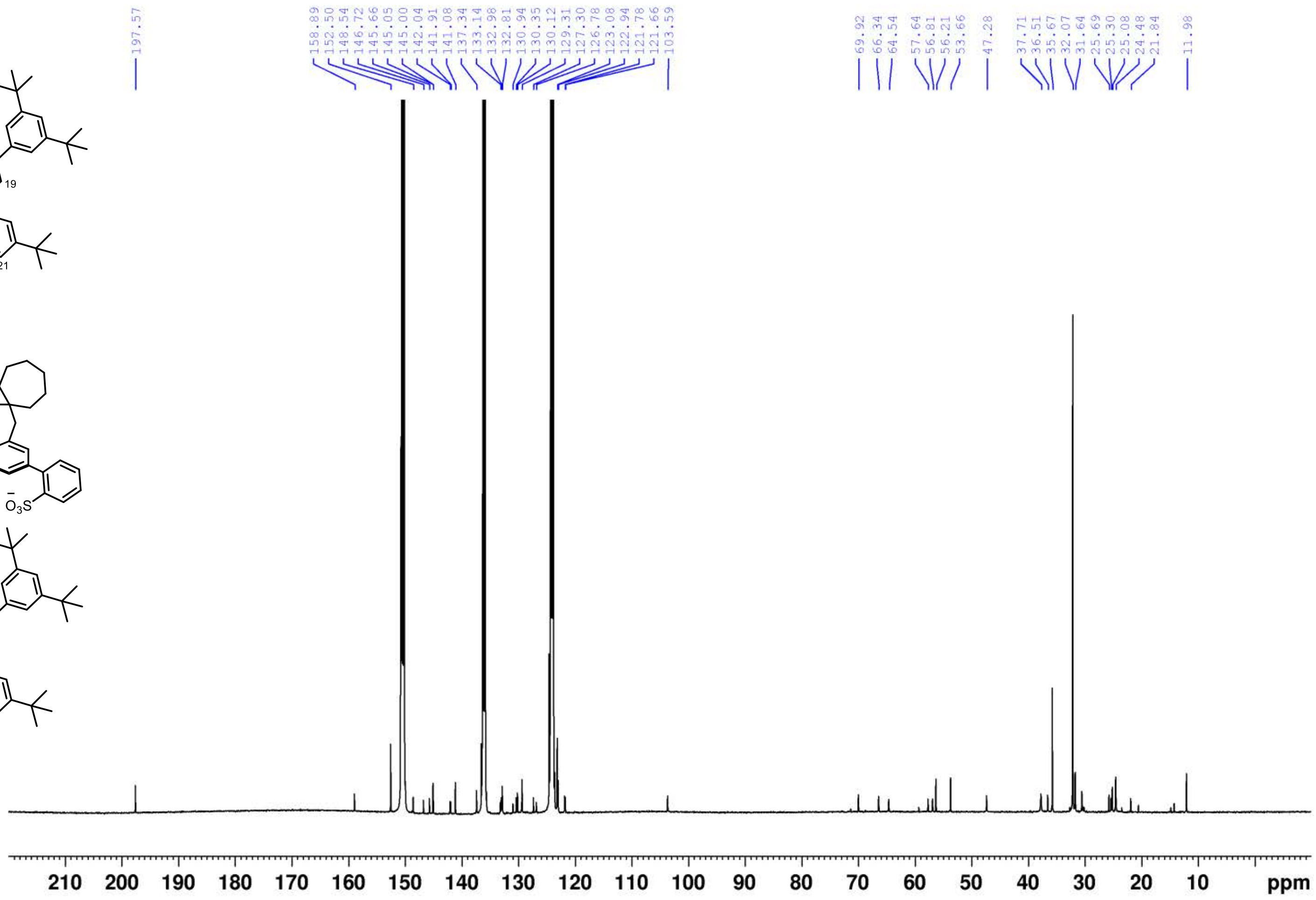

^{1}H NMR (700 MHz, C_5D_5N) for *bis[rhodium (1S,2R,4S,5R)-1-([1,1':3',1''-terphenyl]-5'-ylmethyl)-5-ethyl-2-((S)-hydroxy(6-methoxyquinolin-4-yl)methyl)quinuclidin-1-ium (3',5'-bis(2-carboxy-2-methylpropyl)-[1,1'-biphenyl]-2-sulfonate)]* (Rh_2(**A-III**)$_2$●(**Cat3**)$_2$)

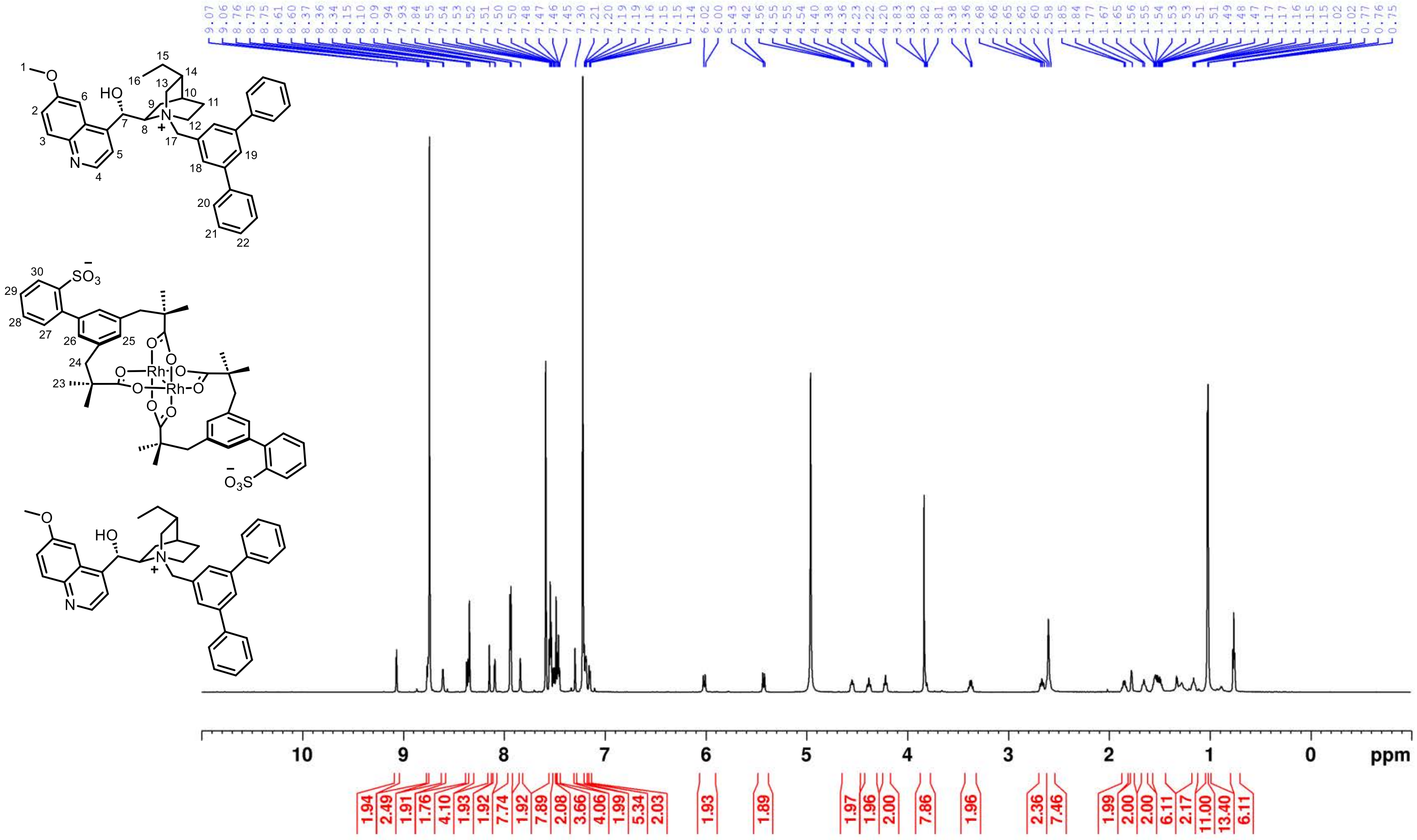

^{13}C NMR (176 MHz, C_5D_5N) for *bis[rhodium (1S,2R,4S,5R)-1-([1,1':3',1''-terphenyl]-5'-ylmethyl)-5-ethyl-2-((S)-hydroxy(6-methoxyquinolin-4-yl)methyl)quinuclidin-1-ium (3',5'-bis(2-carboxy-2-methylpropyl)-[1,1'-biphenyl]-2-sulfonate)]* (Rh_2(**A-III**)$_2$●(**Cat3**)$_2$)

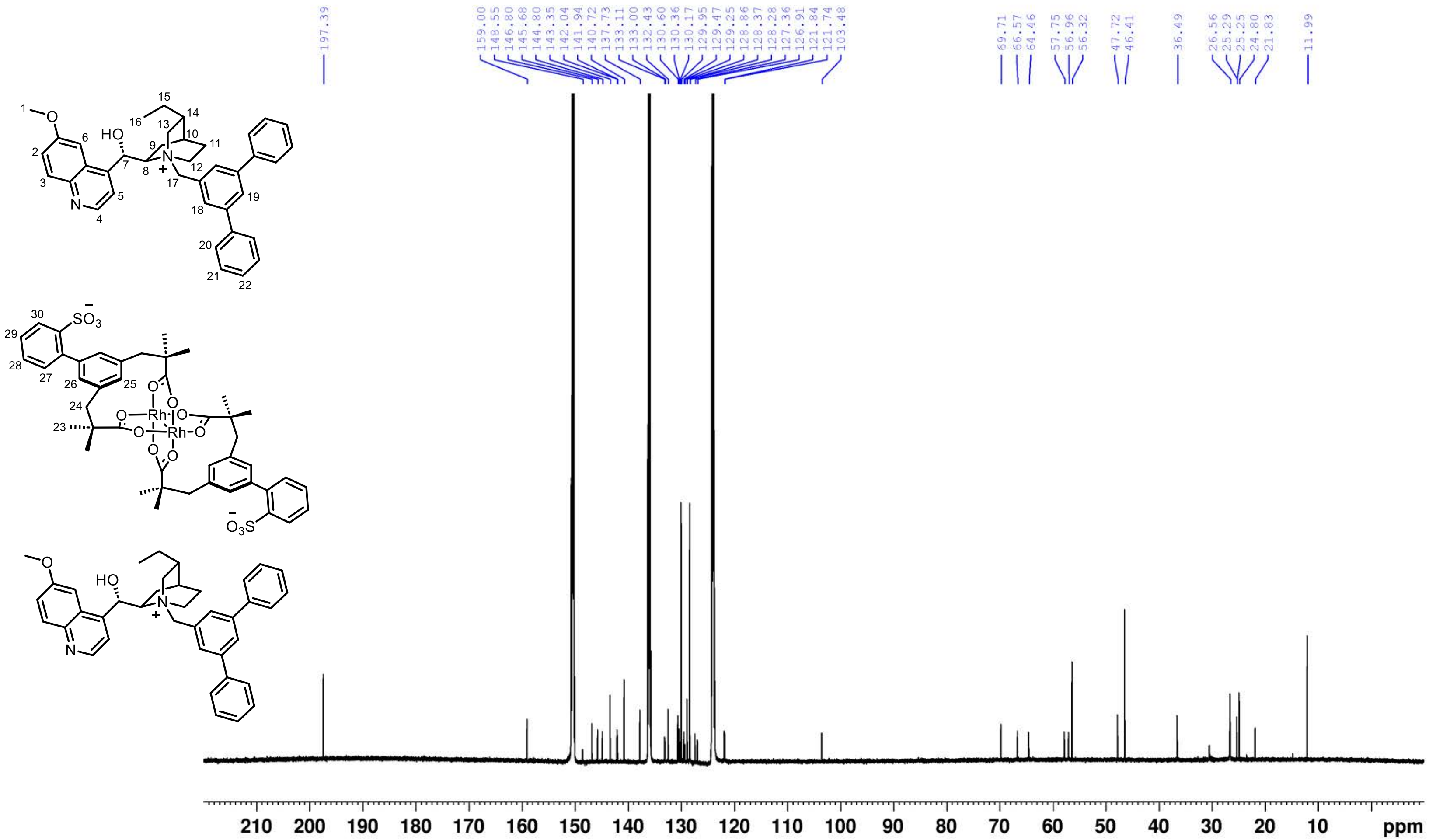

¹H NMR (700 MHz, C_5D_5N) for *bis[rhodium (1S,2R,4S,5R)-5-ethyl-2-((S)-hydroxy(6-methoxyquinolin-4-yl)methyl)-1-((3,3'',5,5''-tetrakis(triethylsilyl)-[1,1':3',1''-terphenyl]-5'-yl)methyl)quinuclidin-1-ium (3',5'-bis(2-carboxy-2-methylpropyl)-[1,1'-biphenyl]-2-sulfonate)]* (Rh_2(**A-III**)$_2$●(**Cat4**)$_2$) (**Rh1**)

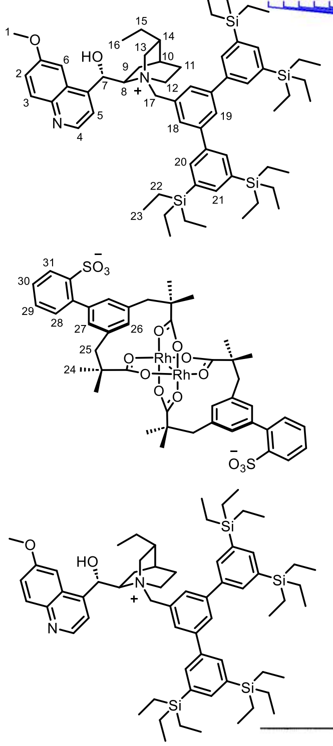

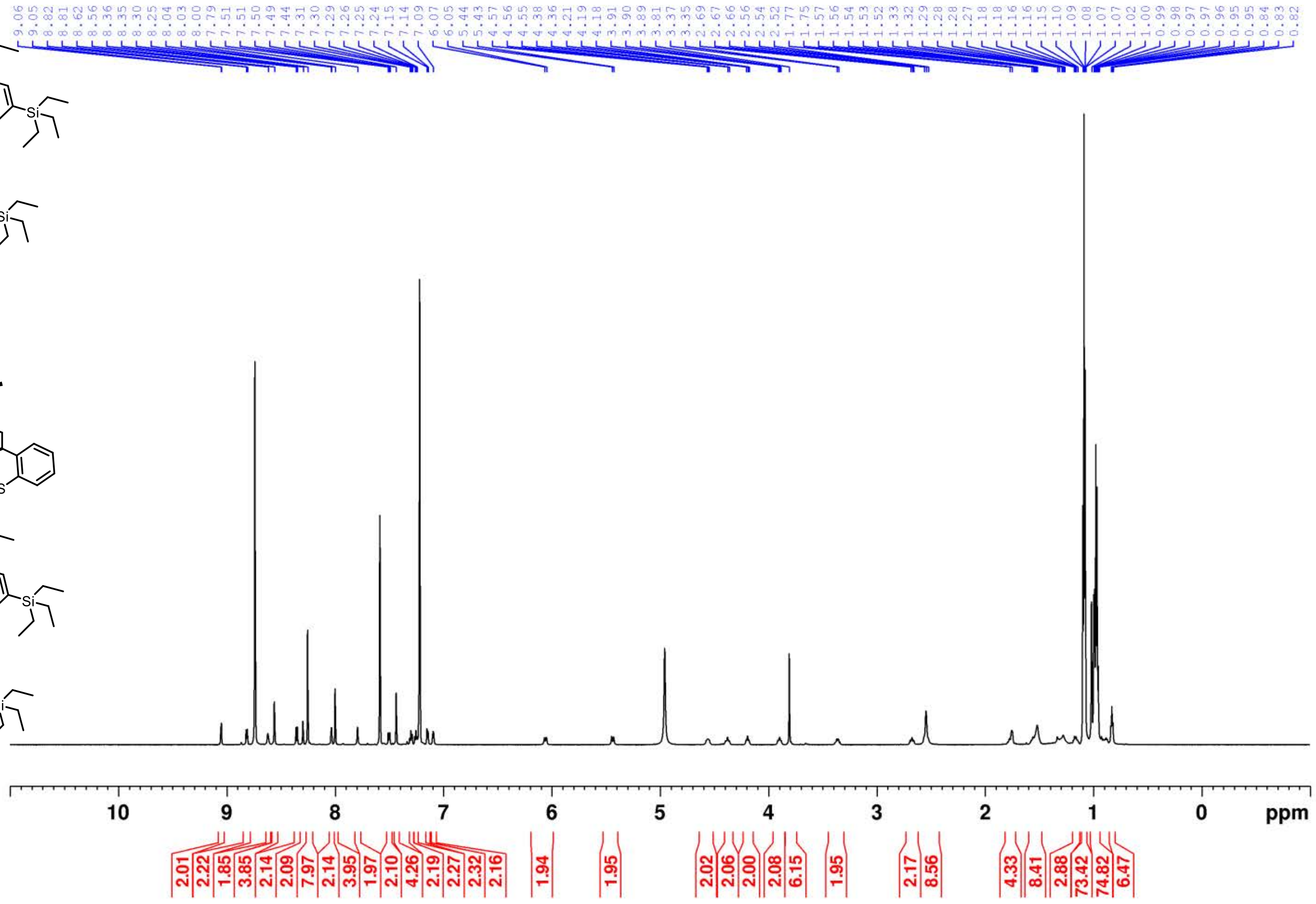

^{13}C NMR (176 MHz, C_5D_5N) for *bis[rhodium (1S,2R,4S,5R)-5-ethyl-2-((S)-hydroxy(6-methoxyquinolin-4-yl)methyl)-1-((3,3'',5,5''-tetrakis(triethylsilyl)-[1,1':3',1''-terphenyl]-5'-yl)methyl)quinuclidin-1-ium (3',5'-bis(2-carboxy-2-methylpropyl)-[1,1'-biphenyl]-2-sulfonate)]* (Rh_2(**A-III**)$_2$●(**Cat4**)$_2$) (**Rh1**)

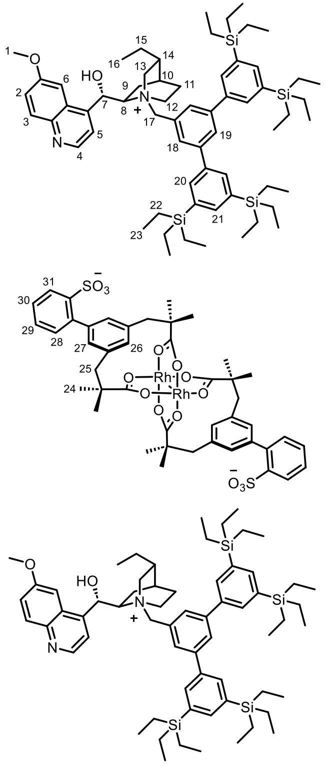

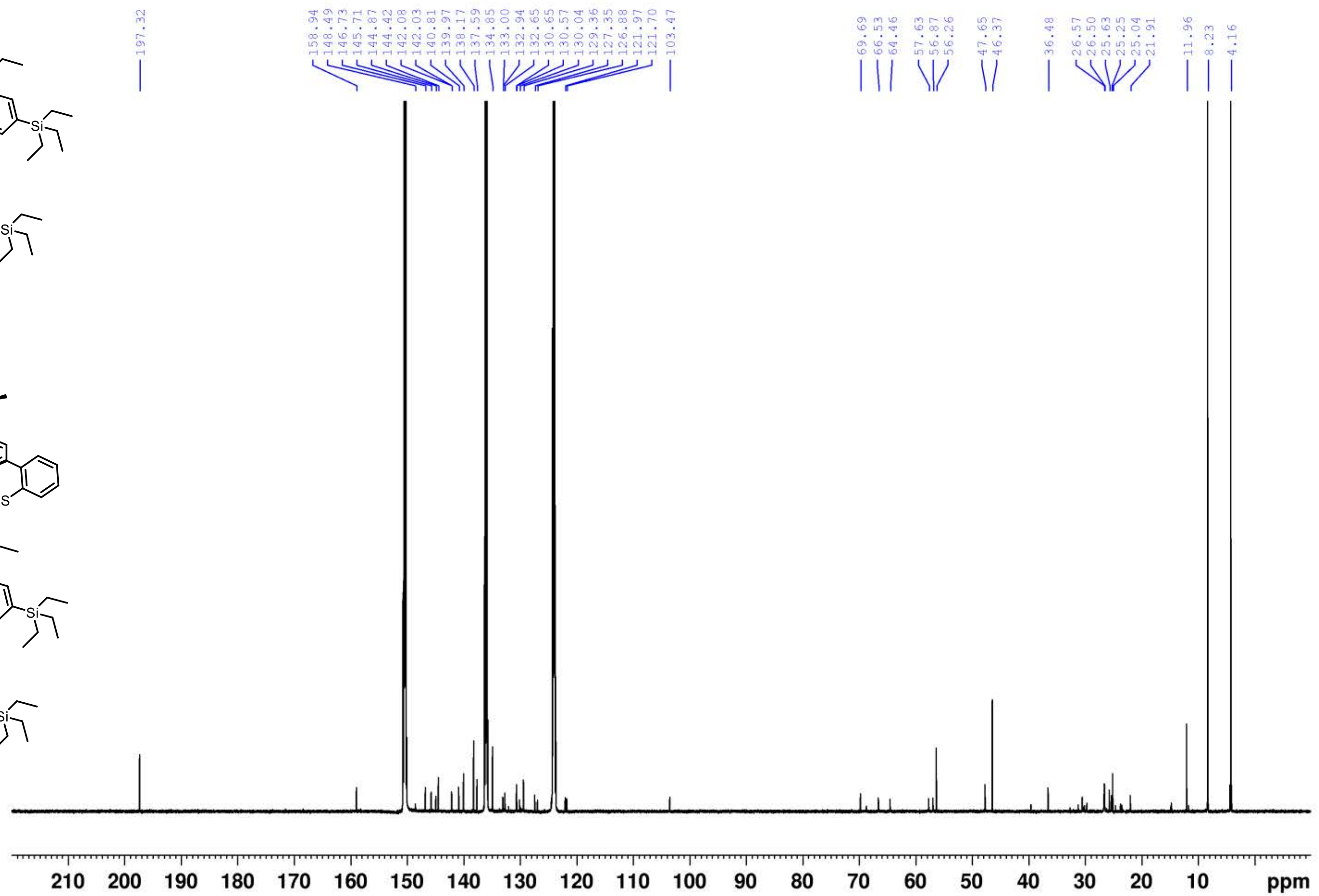

^{1}H NMR (700 MHz, C_5D_5N) for *bis[rhodium (1S,2S,4S,5R)-5-ethyl-2-((R)-hydroxy(6-methoxyquinolin-4-yl)methyl)-1-((3,3'',5,5''-tetrakis(triethylsilyl)-[1,1':3',1''-terphenyl]-5'-yl)methyl)quinuclidin-1-ium (3',5'-bis(2-carboxy-2-methylpropyl)-[1,1'-biphenyl]-2-sulfonate)]* (Rh_2(**A-III**)$_2$●(**Cat5**)$_2$) (**Rh2**)

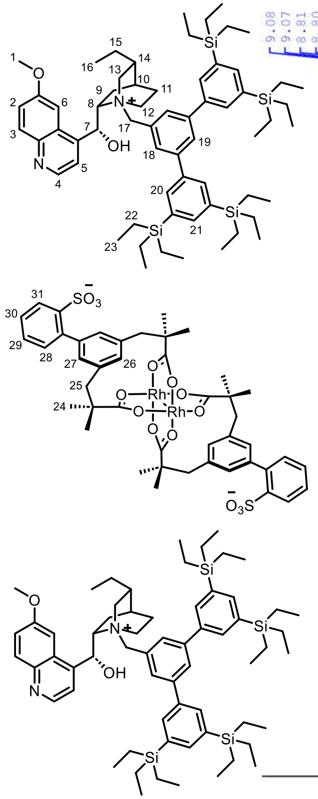

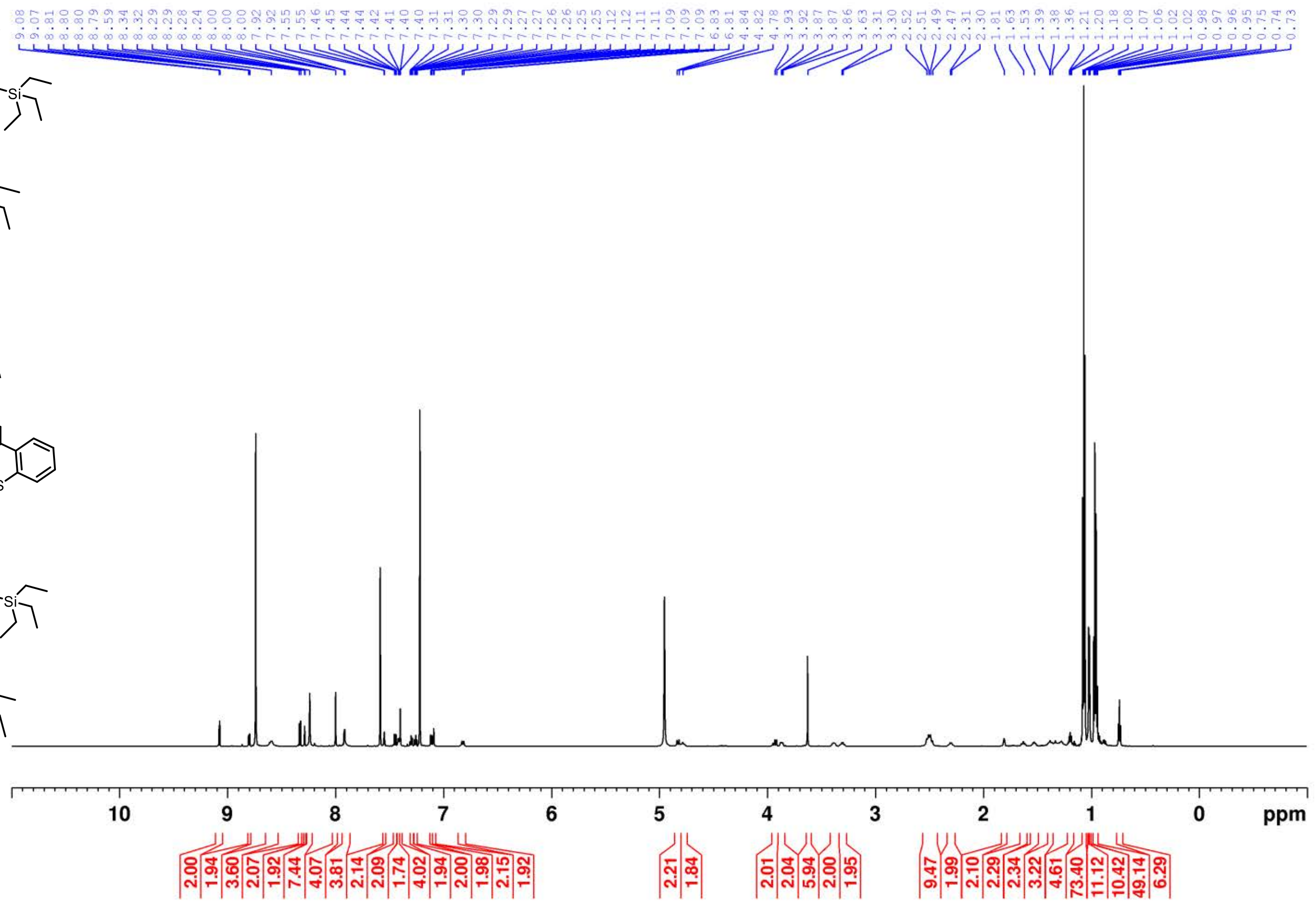

^{13}C NMR (176 MHz, C_5D_5N) for *bis[rhodium (1S,2S,4S,5R)-5-ethyl-2-((R)-hydroxy(6-methoxyquinolin-4-yl)methyl)-1-((3,3'',5,5''-tetrakis(triethylsilyl)-[1,1':3',1''-terphenyl]-5'-yl)methyl)quinuclidin-1-ium (3',5'-bis(2-carboxy-2-methylpropyl)-[1,1'-biphenyl]-2-sulfonate)]* (Rh_2(**A-III**)$_2$●(**Cat5**)$_2$) (**Rh2**)

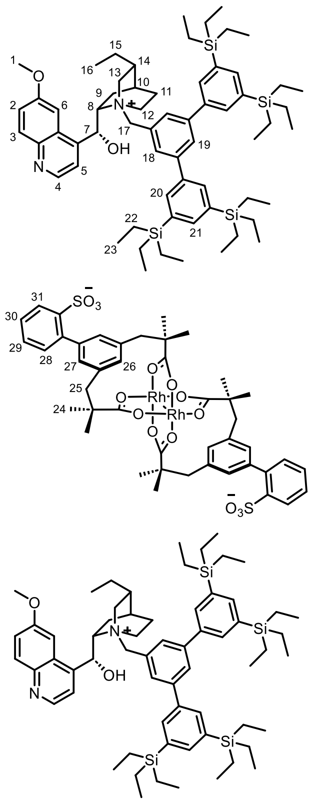

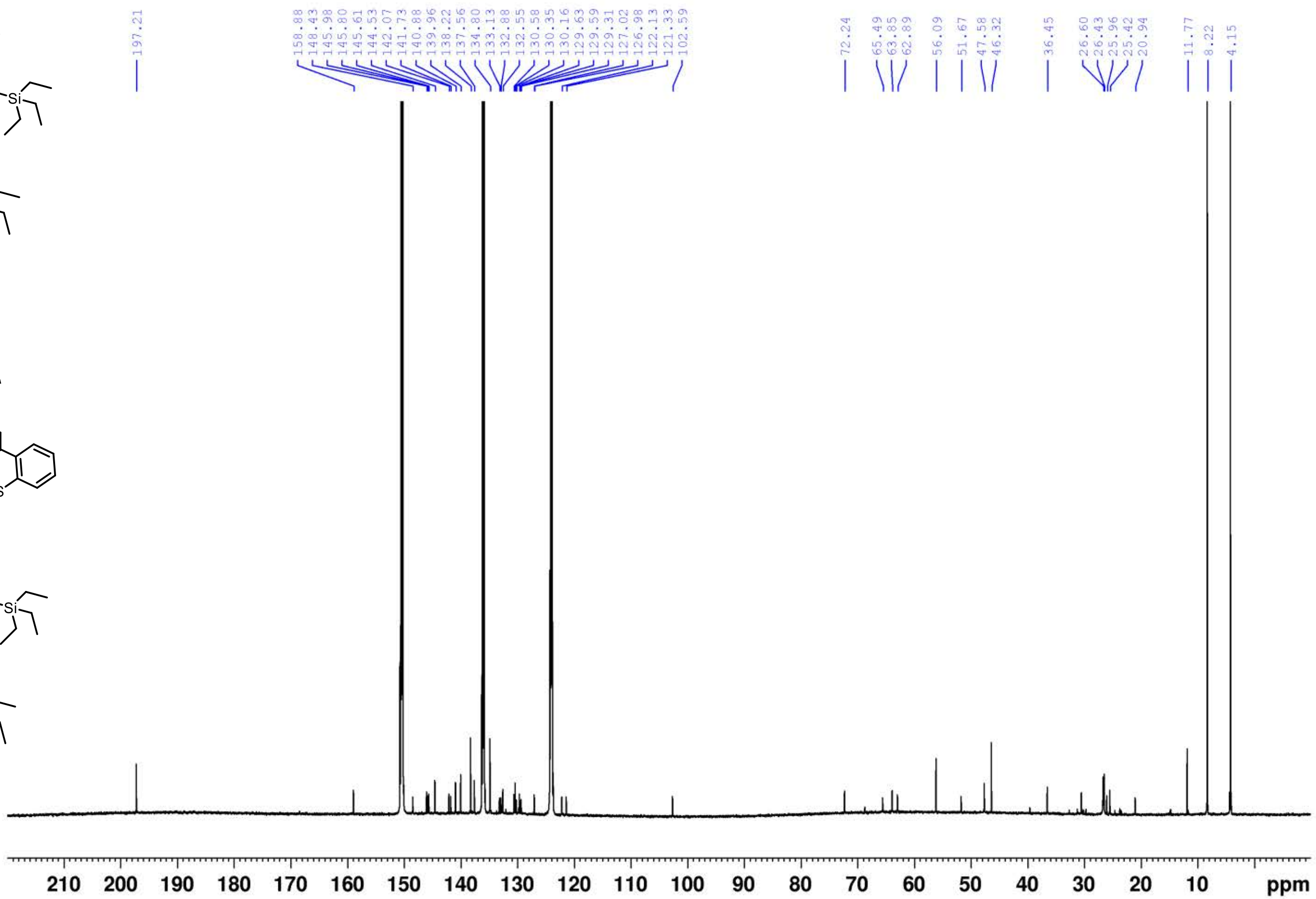

^{1}H NMR (700 MHz, C_5D_5N) for *bis[rhodium (1S,2R,4S,5R)-5-ethyl-2-((S)-hydroxy(6-methoxyquinolin-4-yl)methyl)-1-((3,3'',5,5''-tetra-tert-butyl-[1,1':3',1''-terphenyl]-5'-yl)methyl)quinuclidin-1-ium ((3',5'-bis(2-carboxy-2-methylpropyl)-[1,1'-biphenyl]-2-yl)methanesulfonate)]* (Rh_2(**A-IV**)$_2$●(**Cat1**)$_2$)

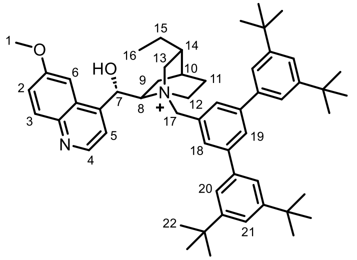

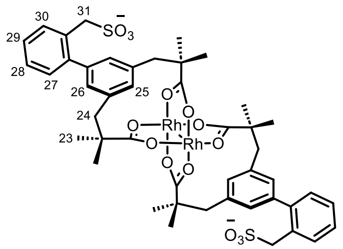

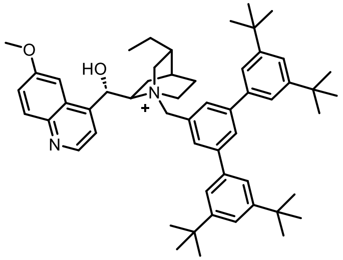

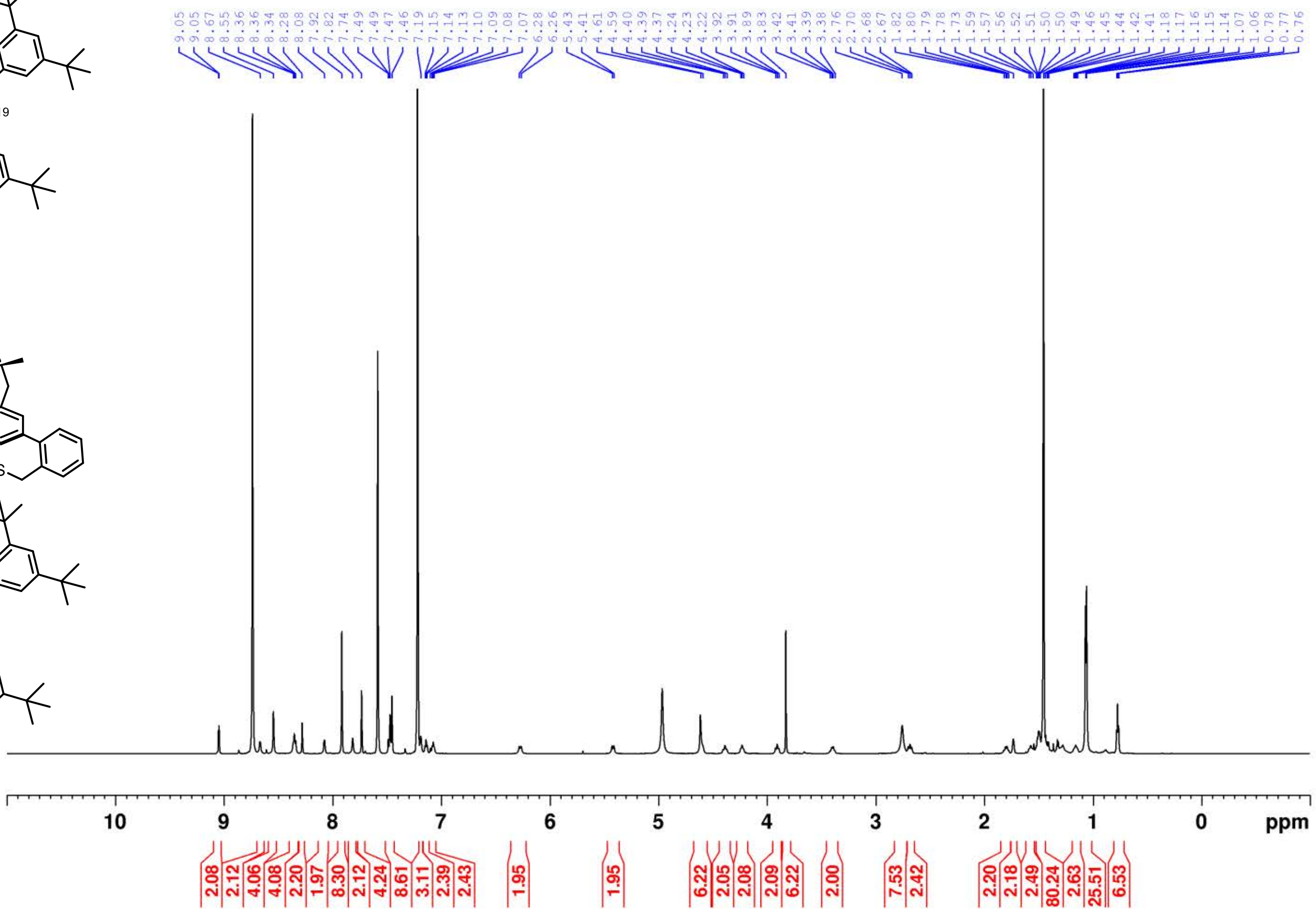

^{13}C NMR (176 MHz, C_5D_5N) for *bis[rhodium (1S,2R,4S,5R)-5-ethyl-2-((S)-hydroxy(6-methoxyquinolin-4-yl)methyl)-1-((3,3'',5,5''-tetra-tert-butyl-[1,1':3',1''-terphenyl]-5'-yl)methyl)quinuclidin-1-ium ((3',5'-bis(2-carboxy-2-methylpropyl)-[1,1'-biphenyl]-2-yl)methanesulfonate)]* (Rh_2(**A-IV**)$_2$●(**Cat1**)$_2$)

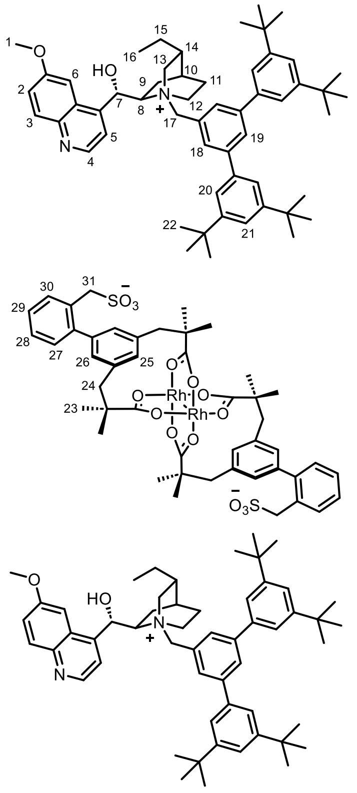

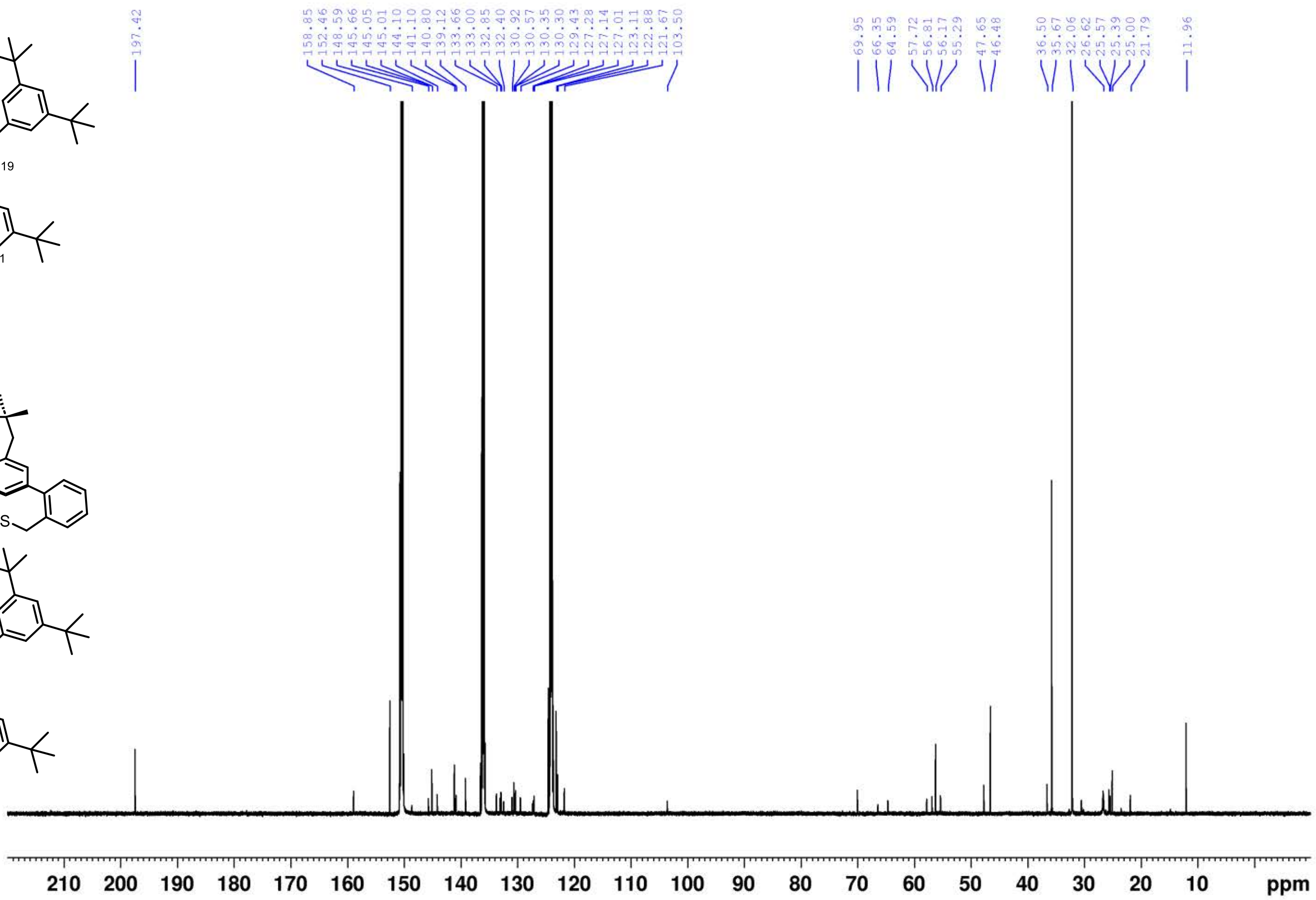

^{1}H NMR (700 MHz, C_5D_5N) for *bis[rhodium (1S,2S,4S,5R)-5-ethyl-2-((R)-hydroxy(6-methoxyquinolin-4-yl)methyl)-1-((3,3'',5,5''-tetra-tert-butyl-[1,1':3',1''-terphenyl]-5'-yl)methyl)quinuclidin-1-ium ((3',5'-bis(2-carboxy-2-methylpropyl)-[1,1'-biphenyl]-2-yl)methanesulfonate)]* (Rh_2(**A-IV**)$_2$●(**Cat2**)$_2$)

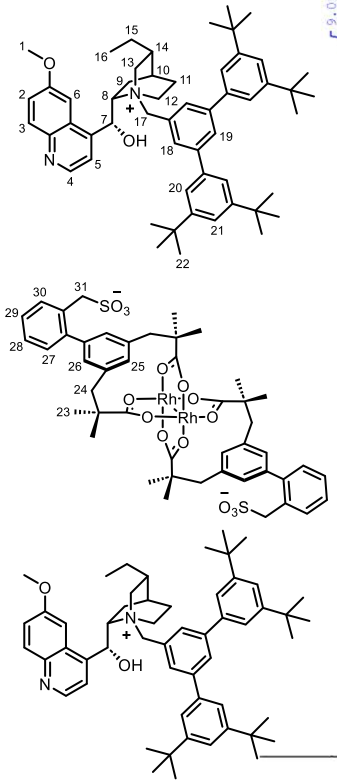

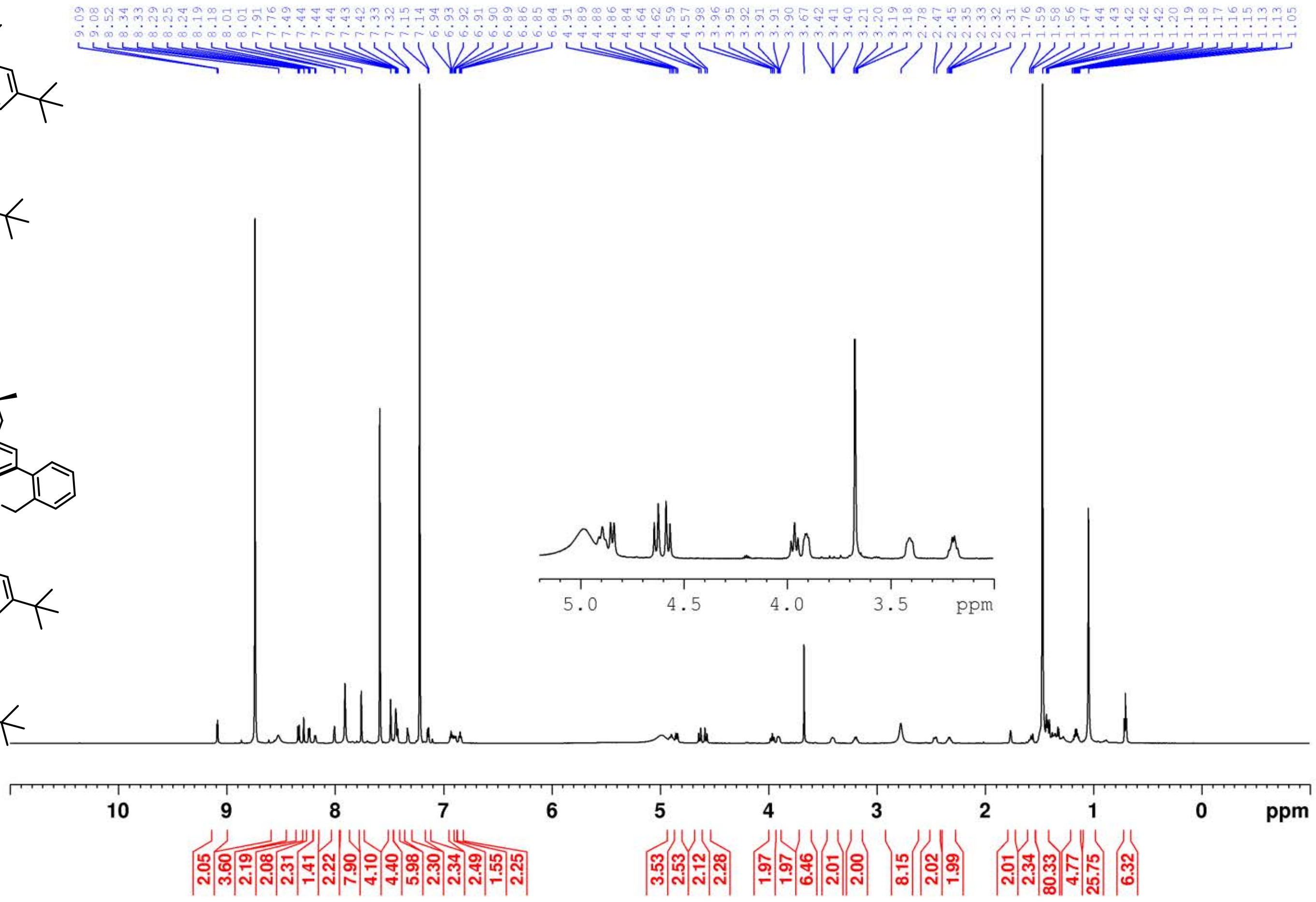

^{13}C NMR (176 MHz, C_5D_5N) for *bis[rhodium (1S,2S,4S,5R)-5-ethyl-2-((R)-hydroxy(6-methoxyquinolin-4-yl)methyl)-1-((3,3'',5,5''-tetra-tert-butyl-[1,1':3',1''-terphenyl]-5'-yl)methyl)quinuclidin-1-ium ((3',5'-bis(2-carboxy-2-methylpropyl)-[1,1'-biphenyl]-2-yl)methanesulfonate)]* (Rh$_2$(**A-IV**)$_2$●(**Cat2**)$_2$)

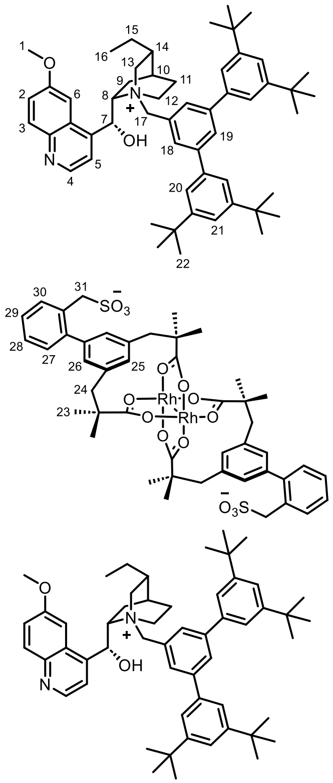

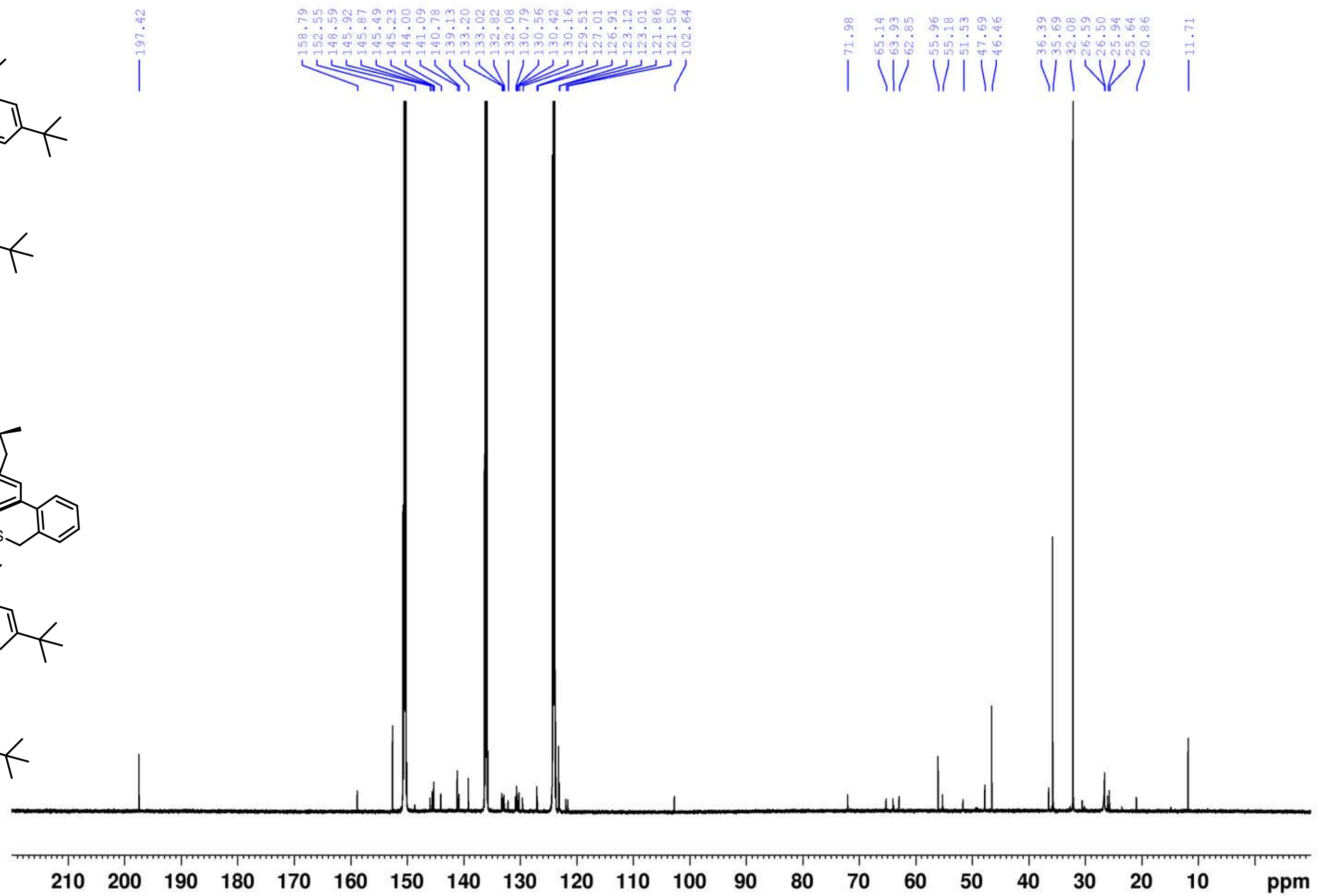

^{1}H NMR (700 MHz, C_5D_5N) for *bis[rhodium (1S,2R,4S,5R)-5-ethyl-2-((S)-hydroxy(6-methoxyquinolin-4-yl)methyl)-1-((3,3'',5,5''-tetra-tert-butyl-[1,1':3',1''-terphenyl]-5'-yl)methyl)quinuclidin-1-ium (8-(3,5-bis(2-carboxy-2-methylpropyl)phenyl)naphthalene-2-sulfonate)]* (Rh_2(**A-V**)$_2$●(**Cat1**)$_2$)

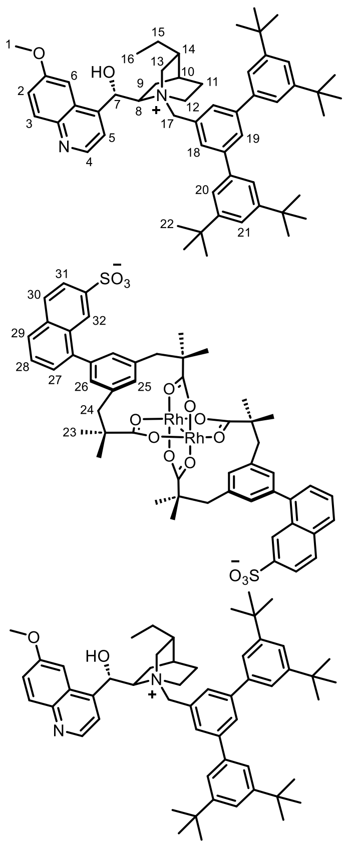

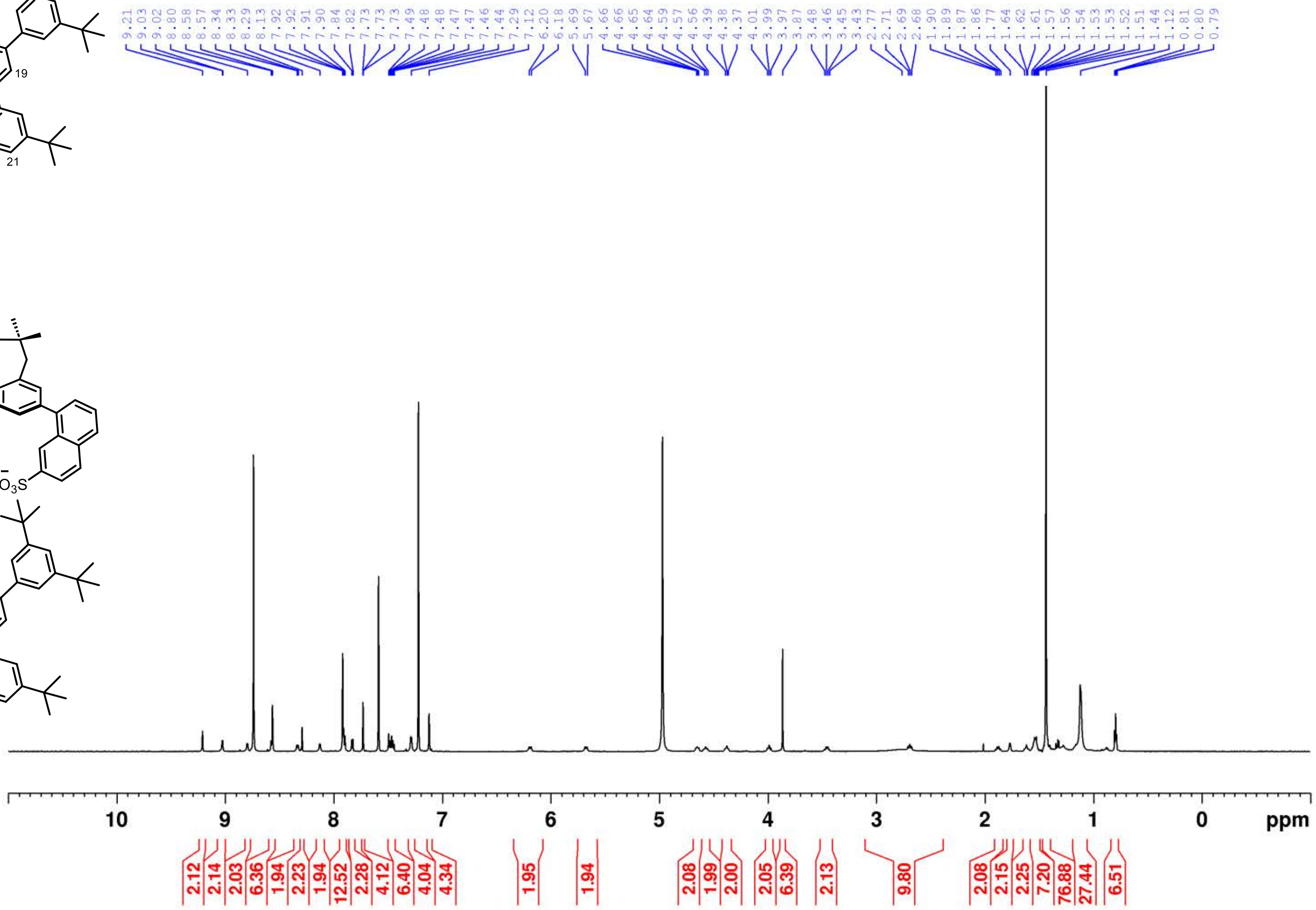

^{13}C NMR (176 MHz, C_5D_5N) for *bis[rhodium (1S,2R,4S,5R)-5-ethyl-2-((S)-hydroxy(6-methoxyquinolin-4-yl)methyl)-1-((3,3'',5,5''-tetra-tert-butyl-[1,1':3',1''-terphenyl]-5'-yl)methyl)quinuclidin-1-ium (8-(3,5-bis(2-carboxy-2-methylpropyl)phenyl)naphthalene-2-sulfonate)]* (Rh_2(**A-V**)$_2$●(**Cat1**)$_2$)

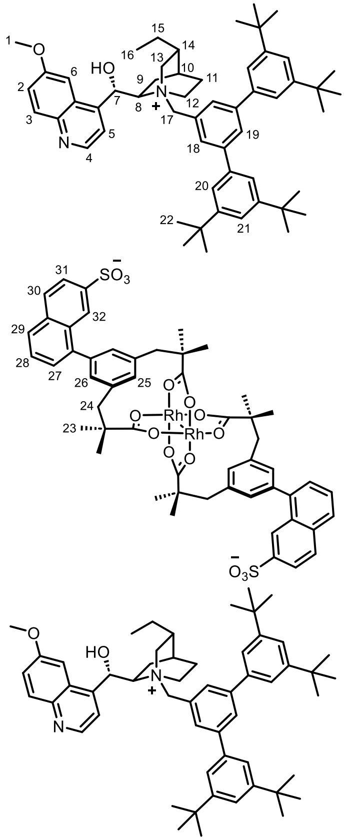

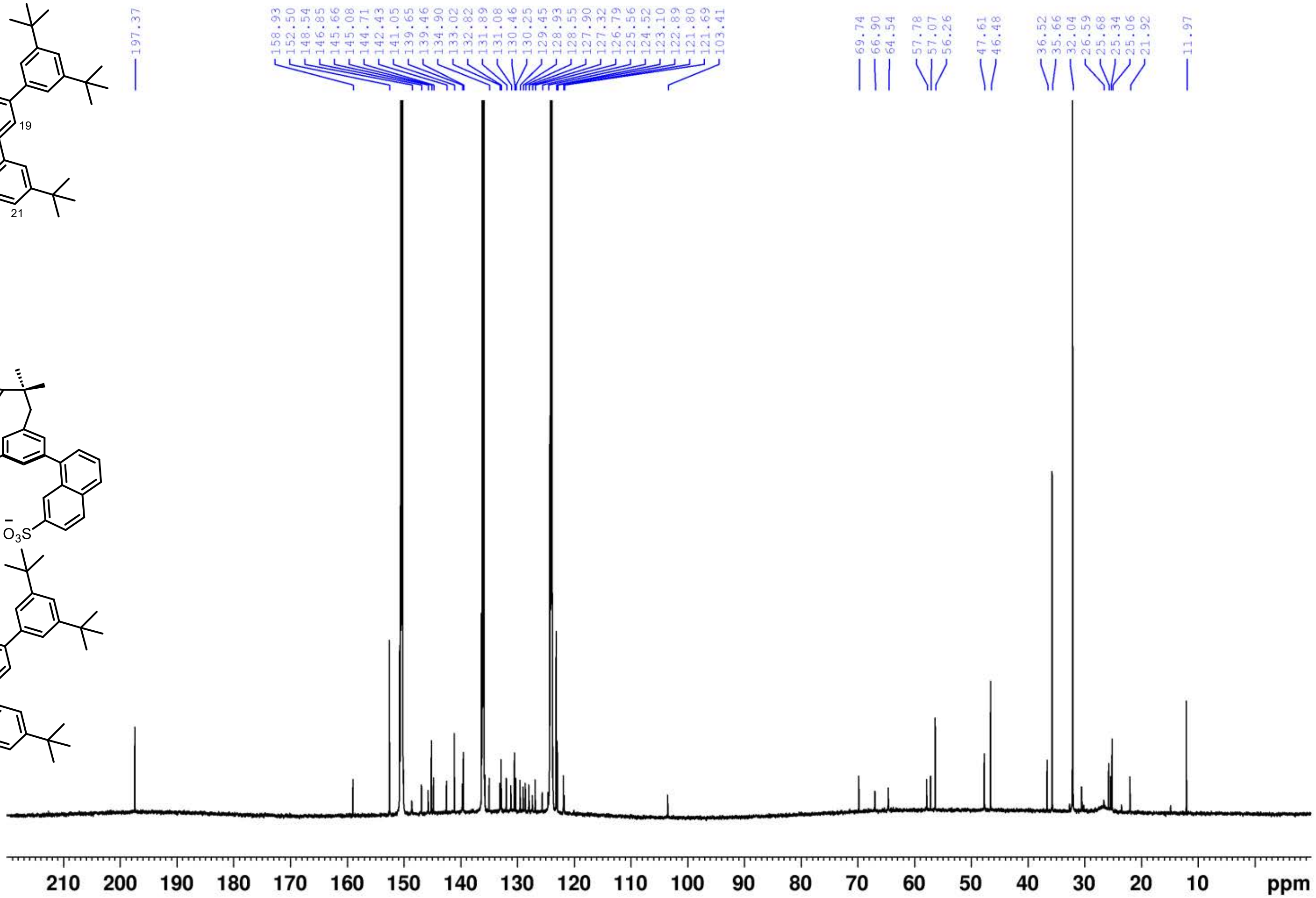

^{1}H NMR (500 MHz, C_5D_5N) for *bis[rhodium (1S,2S,4S,5R)-5-ethyl-2-((R)-hydroxy(6-methoxyquinolin-4-yl)methyl)-1-((3,3'',5,5''-tetra-tert-butyl-[1,1':3',1''-terphenyl]-5'-yl)methyl)quinuclidin-1-ium (8-(3,5-bis(2-carboxy-2-methylpropyl)phenyl)naphthalene-2-sulfonate)]* (Rh_2(**A**-**V**)$_2$●(**Cat2**)$_2$)

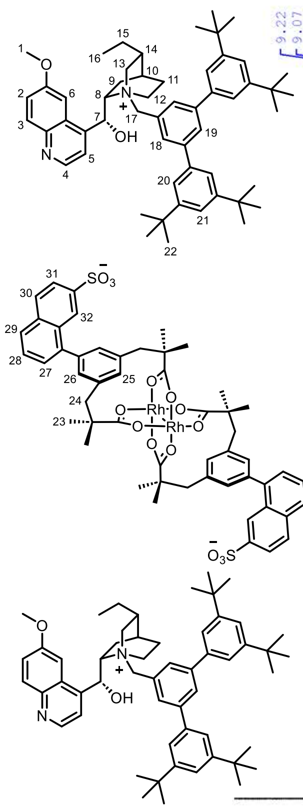

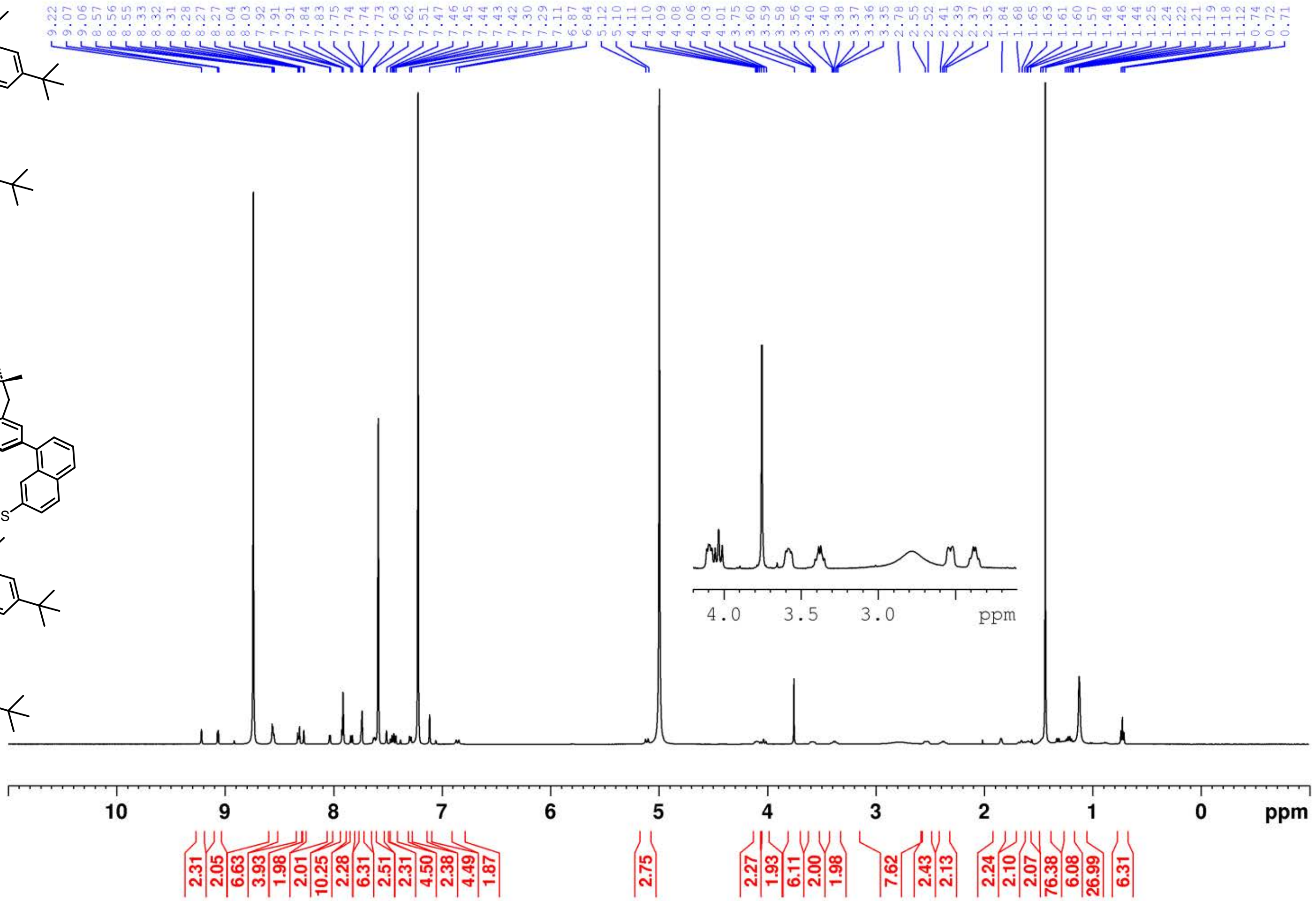

^{13}C NMR (126 MHz, C_5D_5N) for *bis[rhodium (1S,2S,4S,5R)-5-ethyl-2-((R)-hydroxy(6-methoxyquinolin-4-yl)methyl)-1-((3,3'',5,5''-tetra-tert-butyl-[1,1':3',1''-terphenyl]-5'-yl)methyl)quinuclidin-1-ium (8-(3,5-bis(2-carboxy-2-methylpropyl)phenyl)naphthalene-2-sulfonate)]* (Rh_2(**A-V**)$_2$●(**Cat2**)$_2$)

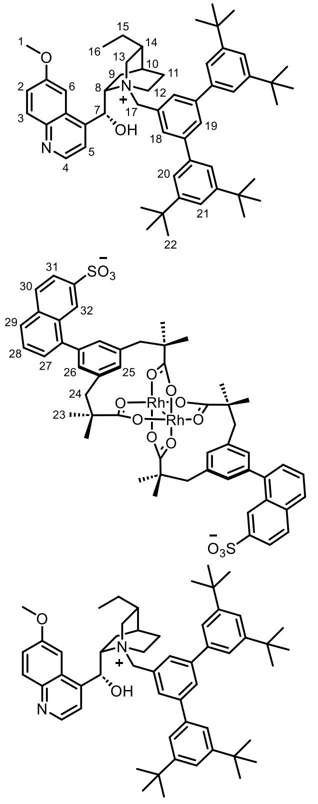

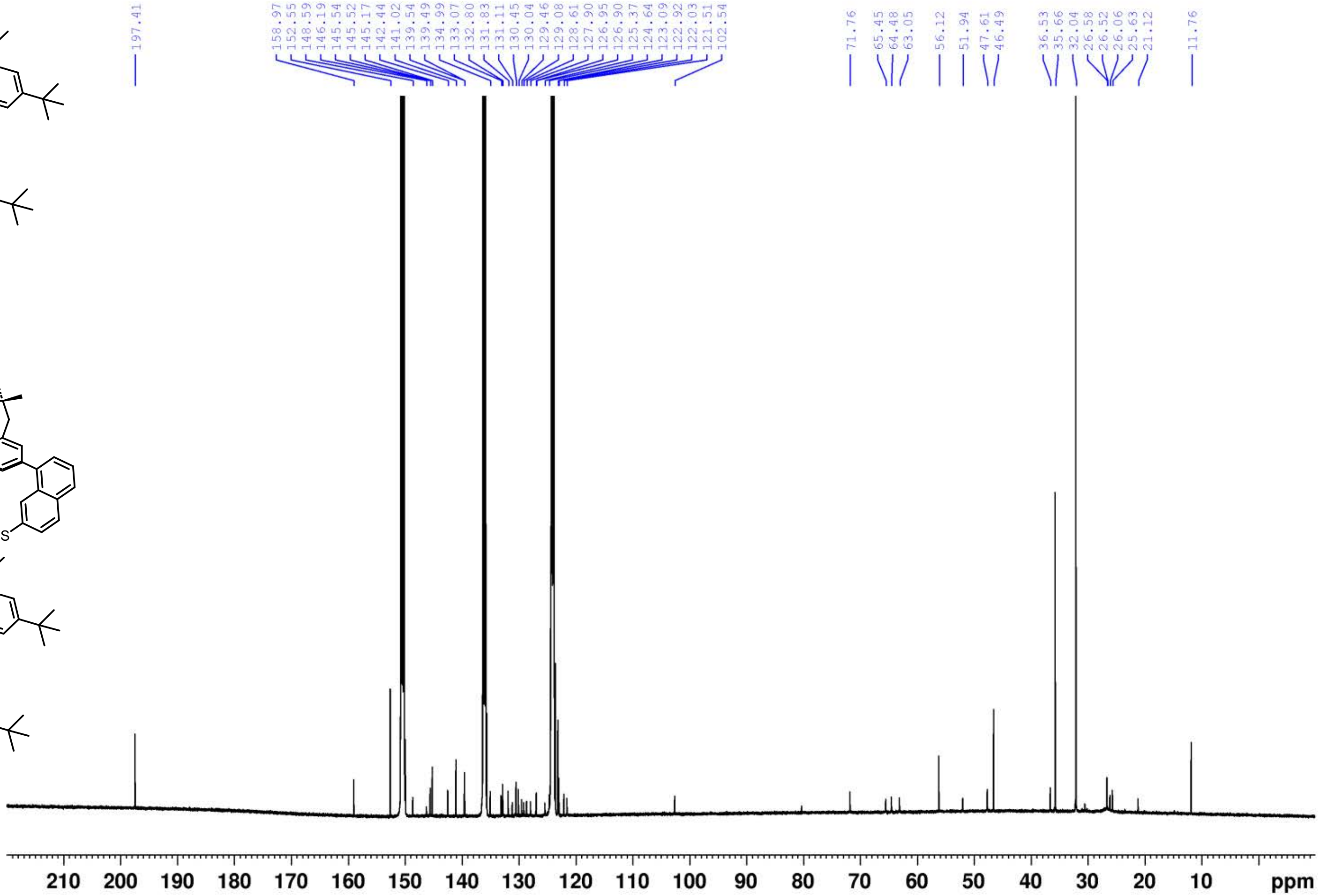

^{1}H NMR (700 MHz, C_5D_5N) for *Bis[rhodium (1S,2R,4S)-2-((S)-hydroxy(naphthalen-1-yl)methyl)-1-((3,3'',5,5''-tetra-tert-butyl-[1,1':3',1''-terphenyl]-5'-yl)methyl)quinuclidin-1-ium (3,5-bis(2-carboxy-2-methylpropyl)benzenesulfonate)]* (Rh_2(**A-I**)$_2$•(**Cat6**)$_2$)

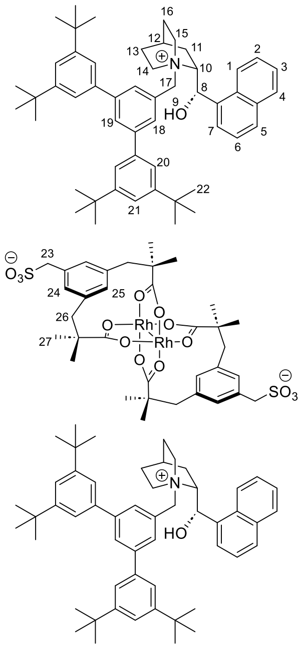

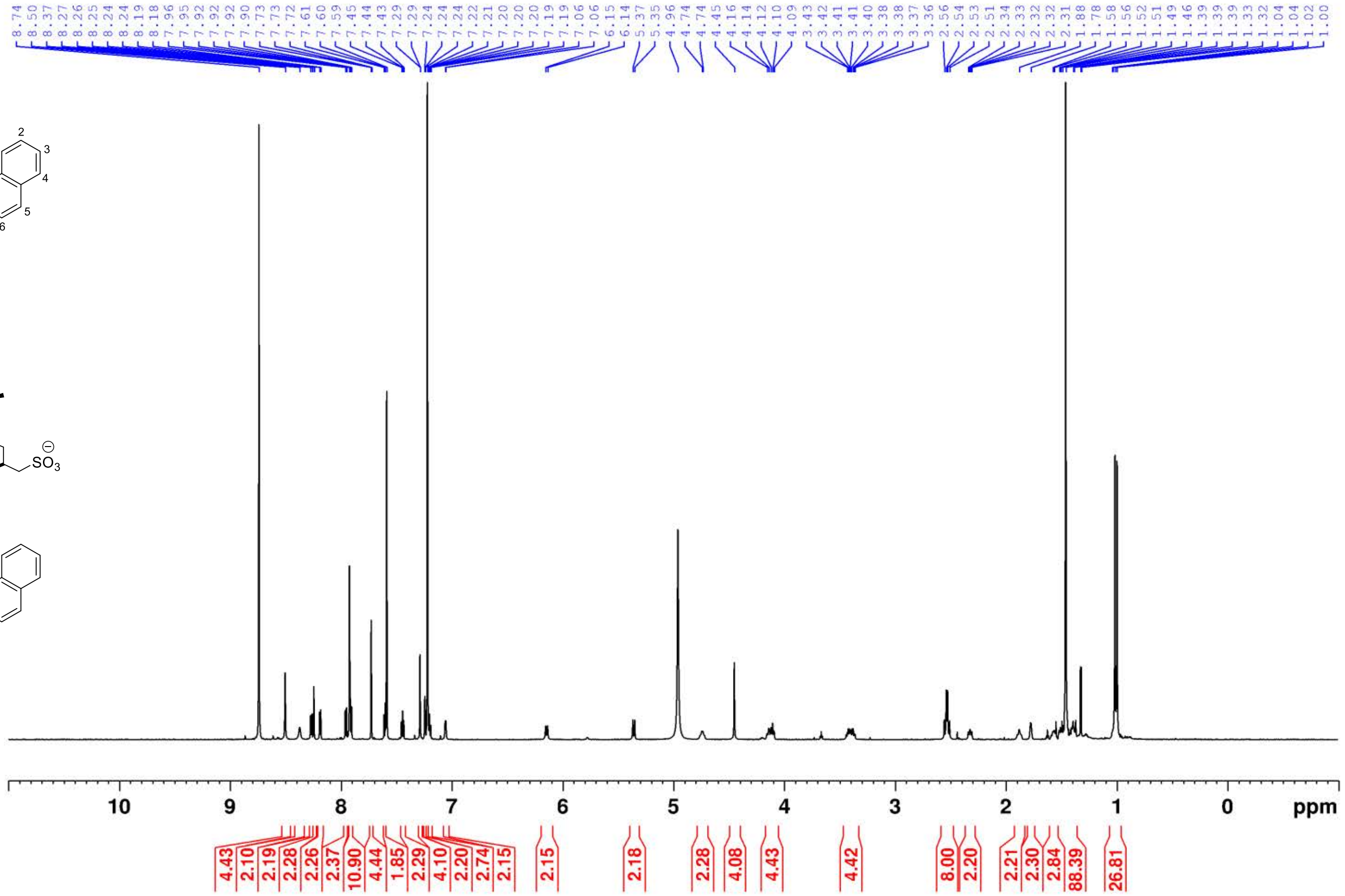

^{13}C NMR (176 MHz, C_5D_5N) for *Bis[rhodium (1S,2R,4S)-2-((S)-hydroxy(naphthalen-1-yl)methyl)-1-((3,3'',5,5''-tetra-tert-butyl-[1,1':3',1''-terphenyl]-5'-yl)methyl)quinuclidin-1-ium (3,5-bis(2-carboxy-2-methylpropyl)benzenesulfonate)]* (Rh_2(**A-I**)$_2$•(**Cat6**)$_2$)

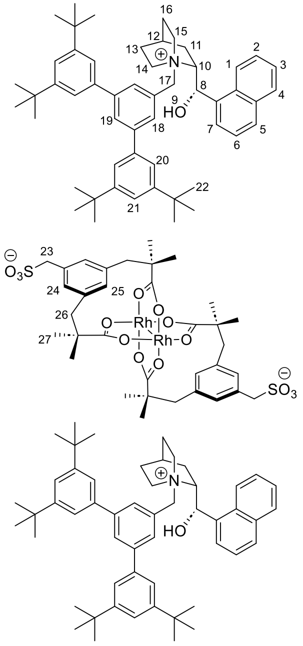

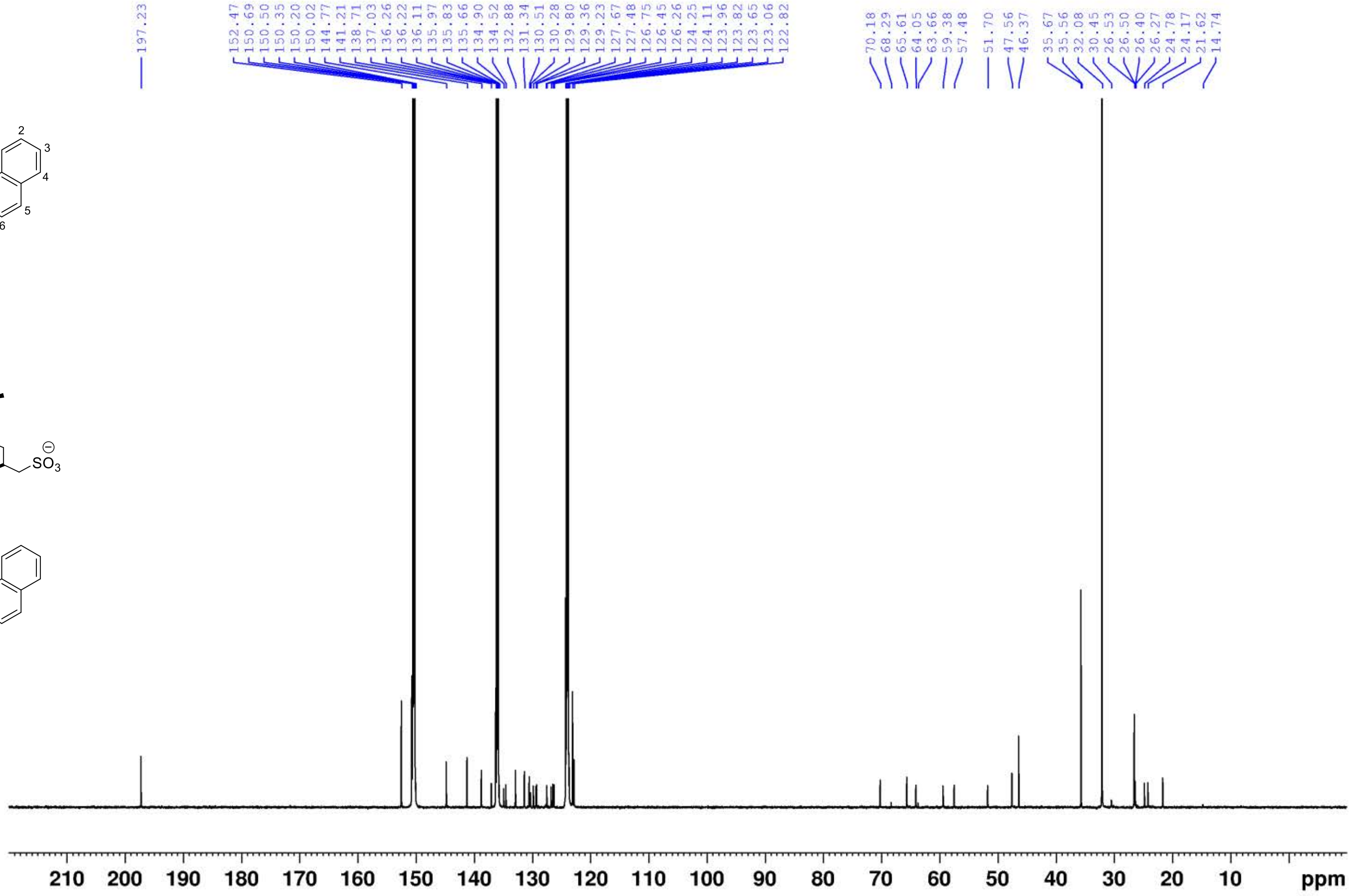

^{1}**H NMR** (700 MHz, C_5D_5N) for *Bis[rhodium (1S,2R,4S)-2-((S)-hydroxy(7-methoxynaphthalen-1-yl)methyl)-1-((3,3'',5,5''-tetra-tert-butyl-[1,1':3',1''-terphenyl]-5'-yl)methyl)quinuclidin-1-ium (3,5-bis(2-carboxy-2-methylpropyl)benzenesulfonate)]*(Rh_2(**A-I**)$_2$•(**Cat7**)$_2$)

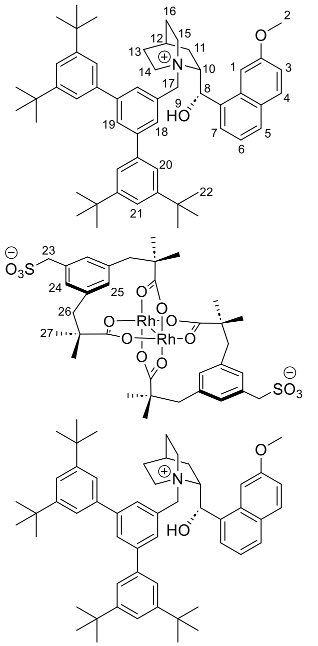

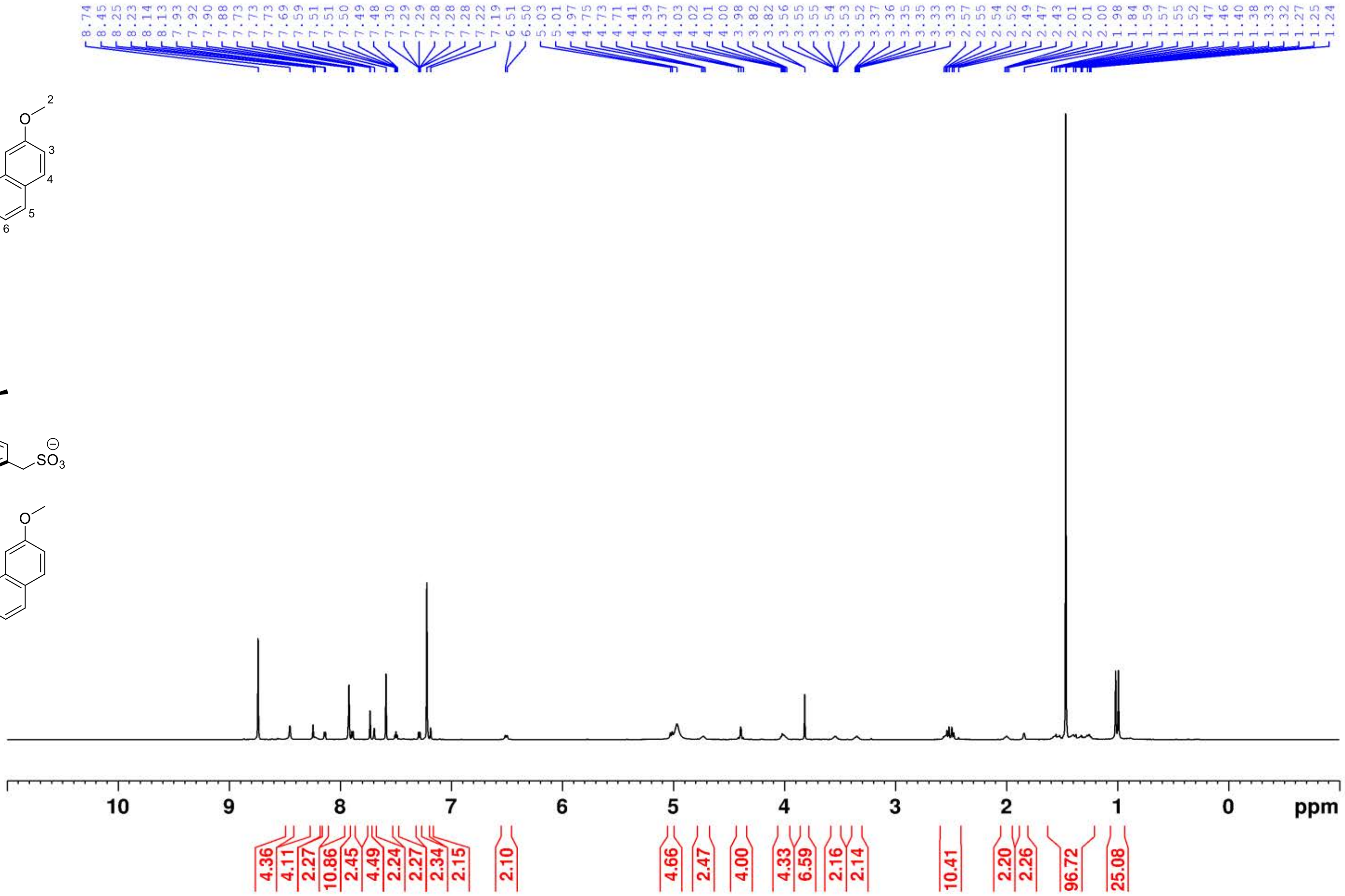

13C NMR (176 MHz, C_5D_5N) for *Bis[rhodium (1S,2R,4S)-2-((S)-hydroxy(7-methoxynaphthalen-1-yl)methyl)-1-((3,3'',5,5''-tetra-tert-butyl-[1,1':3',1''-terphenyl]-5'-yl)methyl)quinuclidin-1-ium (3,5-bis(2-carboxy-2-methylpropyl)benzenesulfonate)]*(Rh_2(**A-I**)$_2$•(**Cat7**)$_2$)

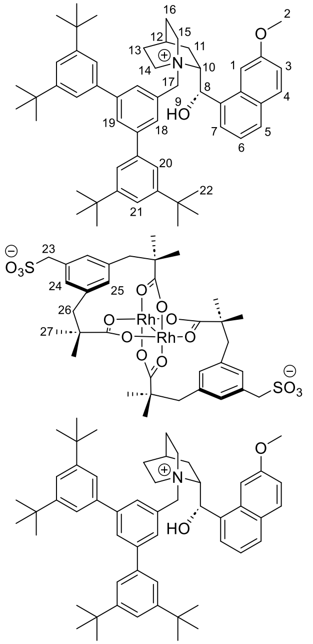

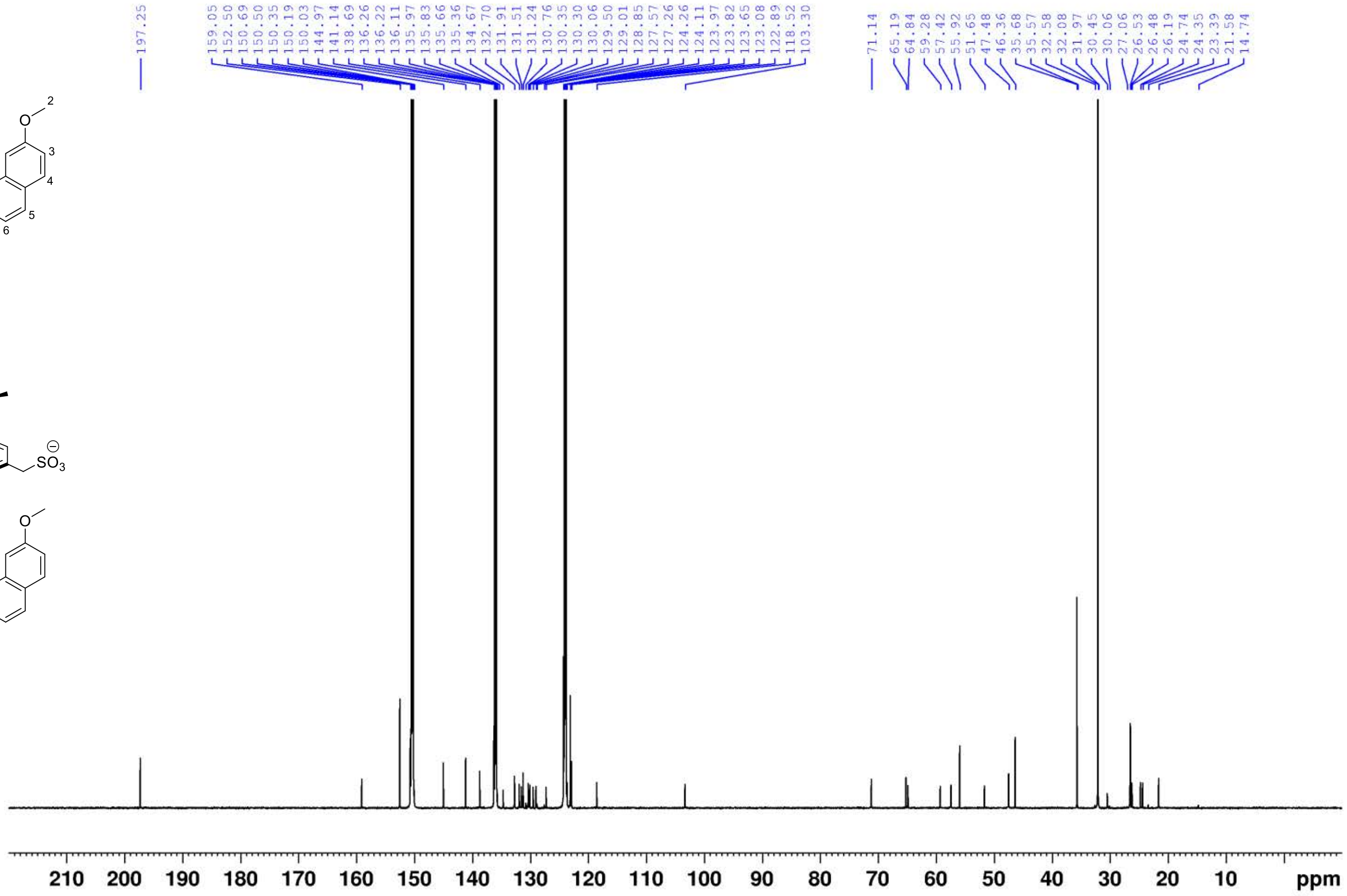

^{1}H NMR (700 MHz, C_5D_5N) for *Bis[rhodium (1S,2R,4S)-2-((S)-hydroxy(quinolin-5-yl)methyl)-1-((3,3'',5,5''-tetra-tert-butyl-[1,1':3',1''-terphenyl]-5'-yl)methyl)quinuclidin-1-ium (3,5-bis(2-carboxy-2-methylpropyl)benzenesulfonate)]* (Rh_2(**A-I**)$_2$•(**Cat8**)$_2$)

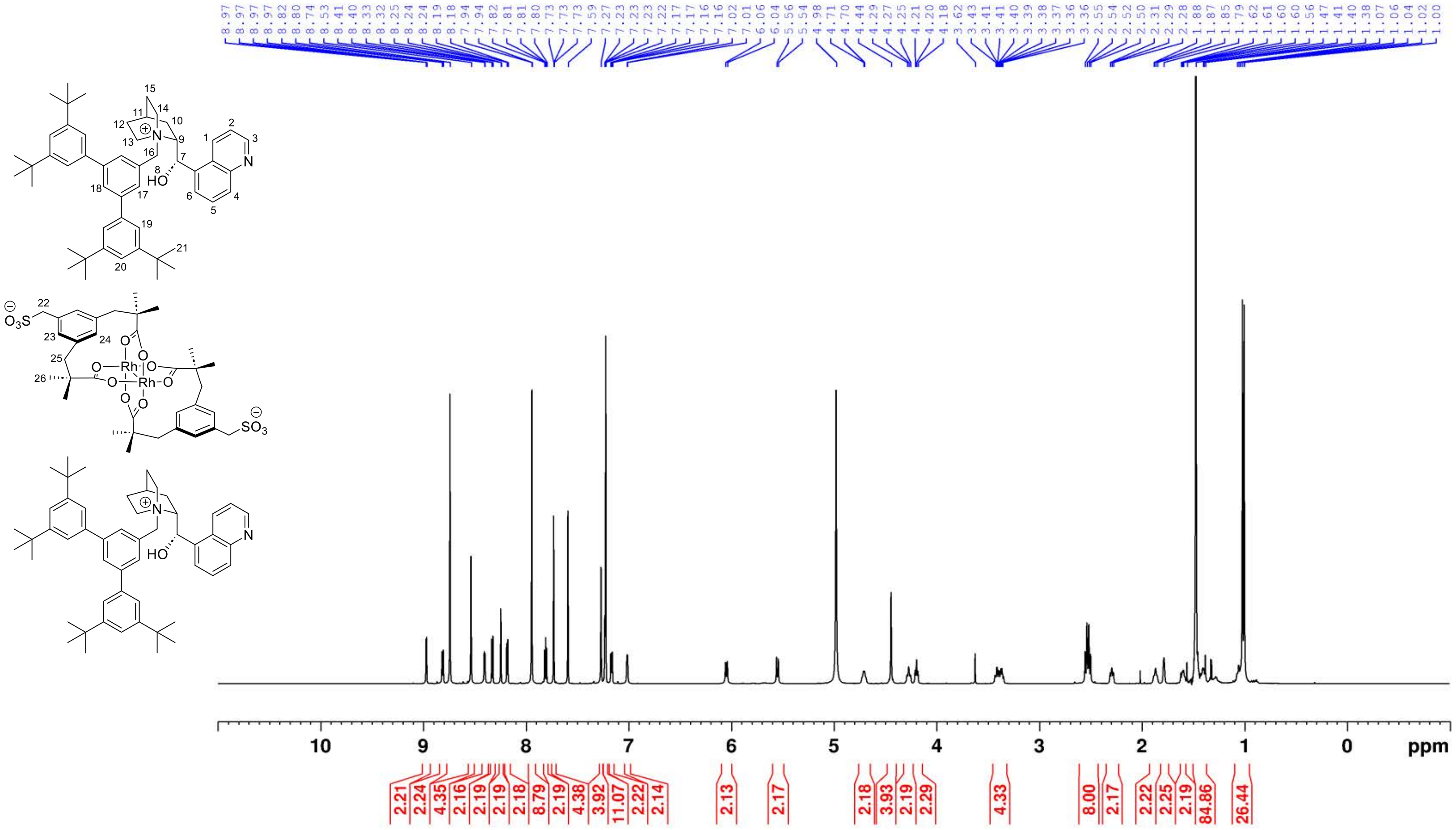

^{13}C NMR (176 MHz, C_5D_5N) for *Bis[rhodium (1S,2R,4S)-2-((S)-hydroxy(quinolin-5-yl)methyl)-1-((3,3'',5,5''-tetra-tert-butyl-[1,1':3',1''-terphenyl]-5'-yl)methyl)quinuclidin-1-ium (3,5-bis(2-carboxy-2-methylpropyl)benzenesulfonate)]* (Rh_2(**A-I**)$_2$•(**Cat8**)$_2$)

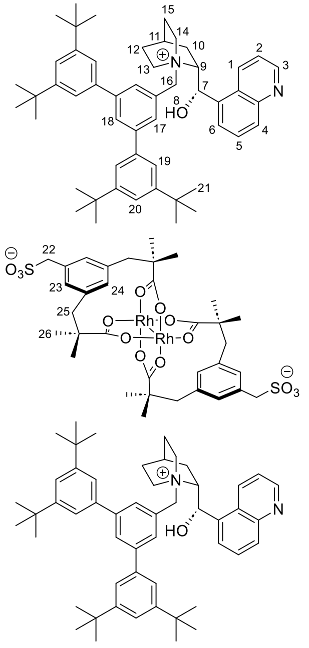

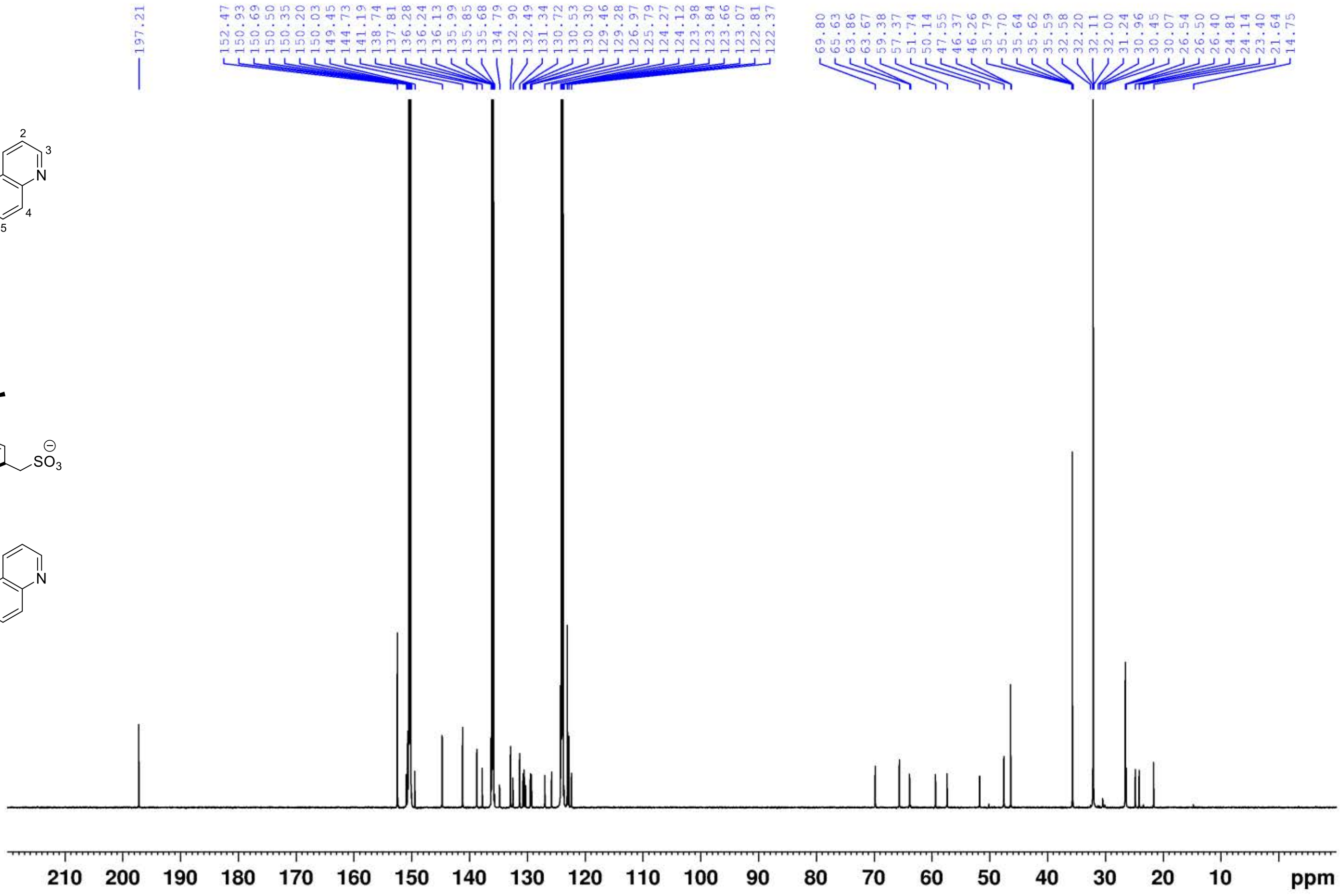

^{1}H NMR (700 MHz, C_5D_5N) for *Bis[rhodium (1S,2R,4S)-2-((S)-hydroxy(quinolin-4-yl)methyl)-1-((3,3'',5,5''-tetra-tert-butyl-[1,1':3',1''-terphenyl]-5'-yl)methyl)quinuclidin-1-ium (3,5-bis(2-carboxy-2-methylpropyl)benzenesulfonate)]* (Rh_2(**A-I**)$_2$•(**Cat9**)$_2$)

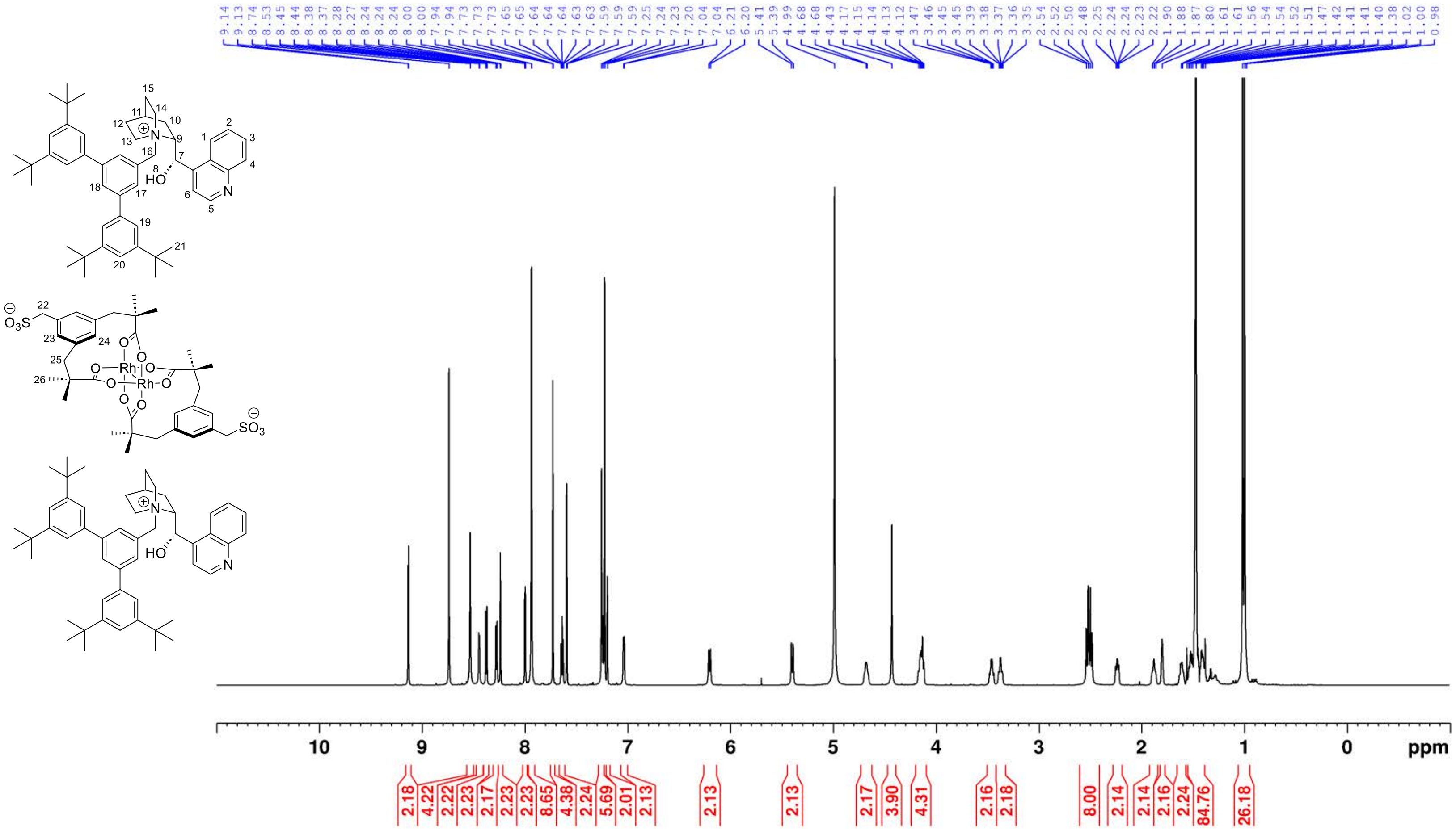

^{13}C NMR (176 MHz, C_5D_5N) for *Bis[rhodium (1S,2R,4S)-2-((S)-hydroxy(quinolin-4-yl)methyl)-1-((3,3'',5,5''-tetra-tert-butyl-[1,1':3',1''-terphenyl]-5'-yl)methyl)quinuclidin-1-ium (3,5-bis(2-carboxy-2-methylpropyl)benzenesulfonate)]* (Rh_2(**A-I**)$_2$•(**Cat9**)$_2$)

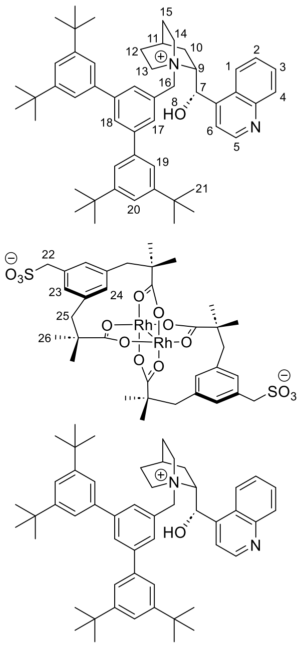

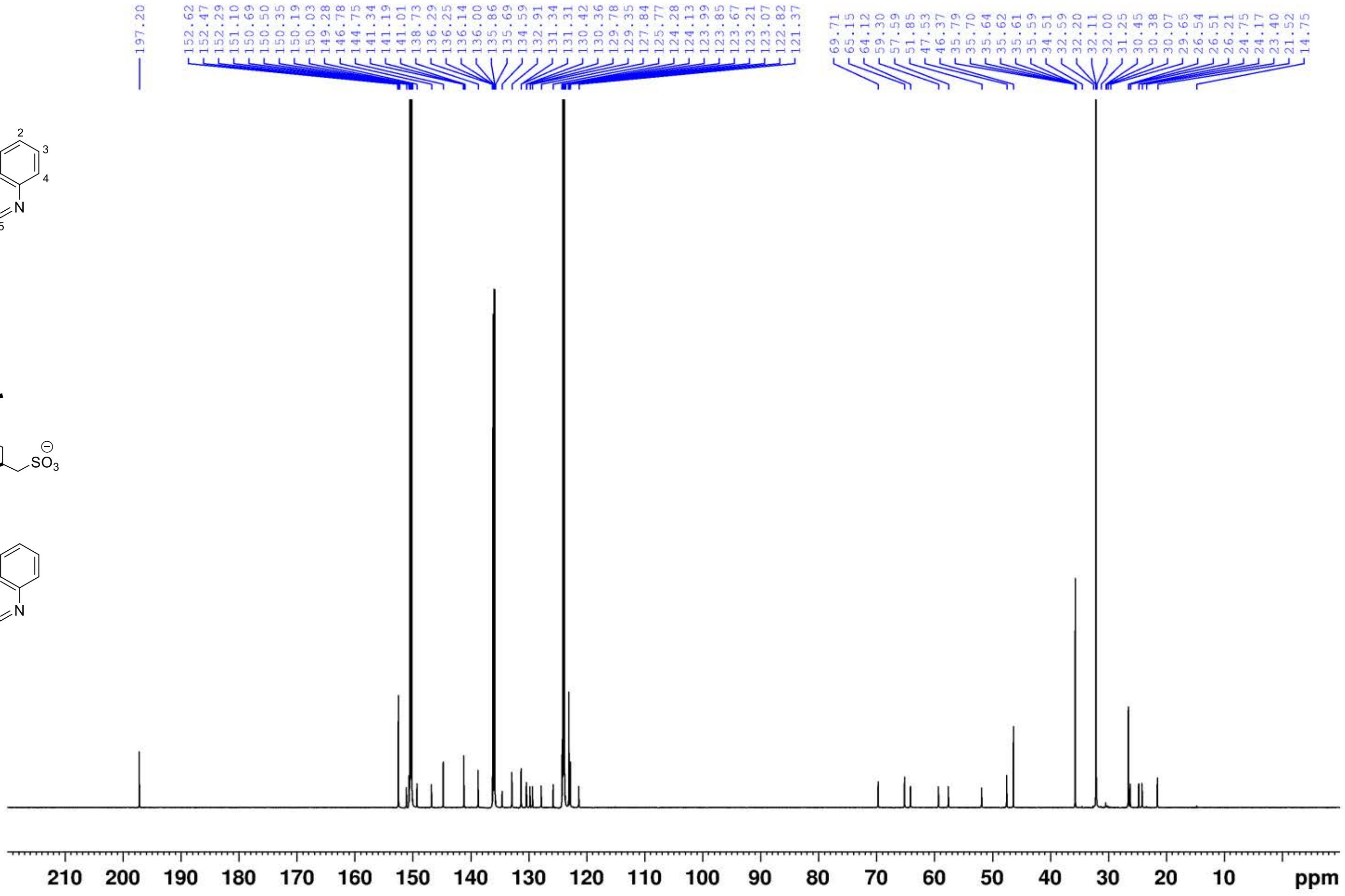

^{1}H NMR (700 MHz, C_5D_5N) for *Bis[rhodium (1R,2S,4R)-2-((R)-hydroxy(6-methoxyquinolin-4-yl)methyl)-1-((3,3'',5,5''-tetra-tert-butyl-[1,1':3',1''-terphenyl]-5'-yl)methyl)quinuclidin-1-ium (3,5-bis(2-carboxy-2-methylpropyl)benzenesulfonate)]* (Rh_2(**A-I**)$_2$•(**Cat10**)$_2$)

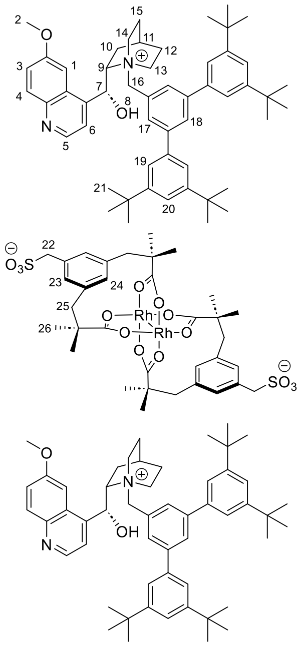

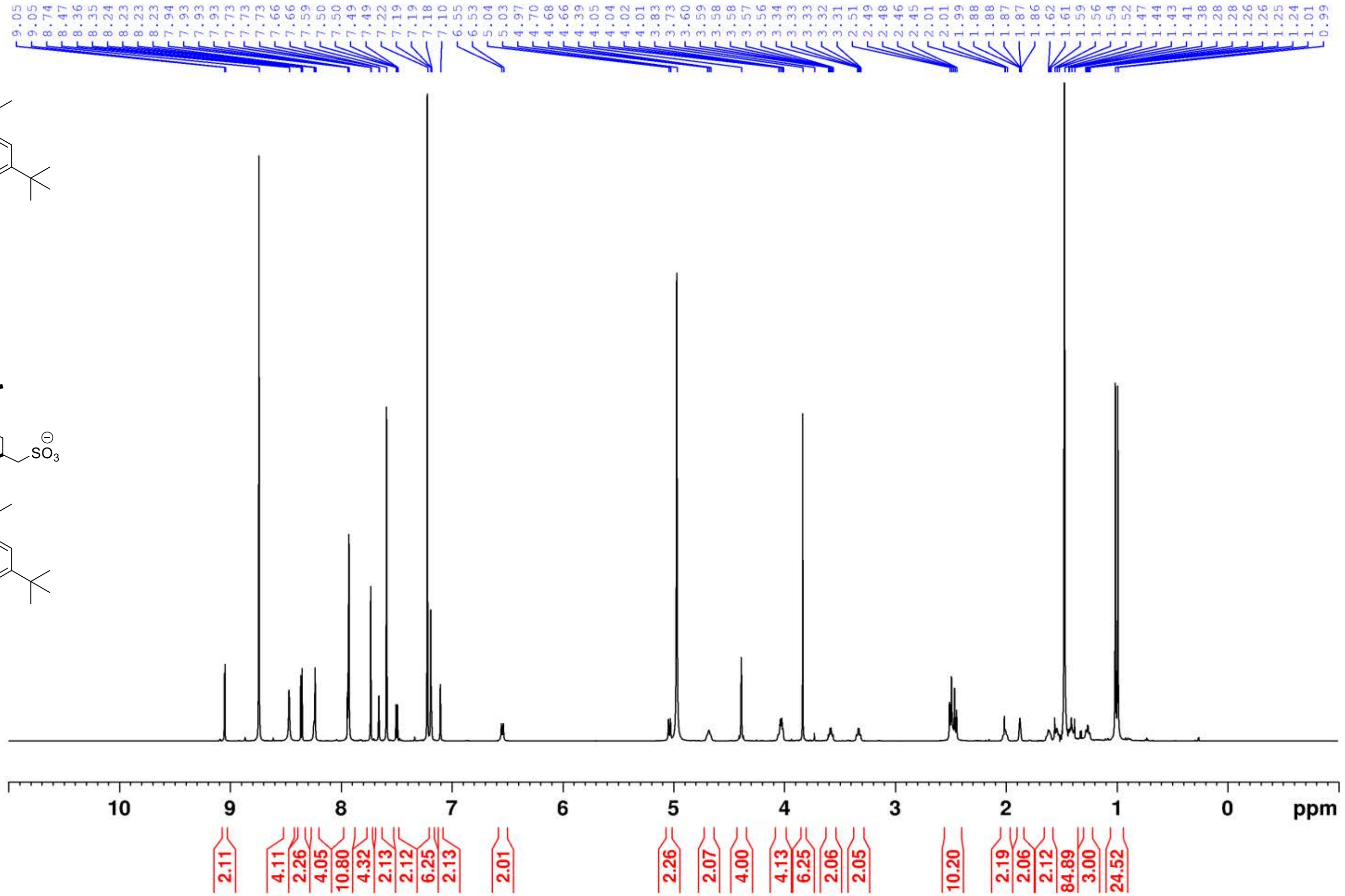

^{13}C NMR (176 MHz, C_5D_5N) for *Bis[rhodium (1R,2S,4R)-2-((R)-hydroxy(6-methoxyquinolin-4-yl)methyl)-1-((3,3'',5,5''-tetra-tert-butyl-[1,1':3',1''-terphenyl]-5'-yl)methyl)quinuclidin-1-ium (3,5-bis(2-carboxy-2-methylpropyl)benzenesulfonate)]* (Rh_2(**A-I**)$_2$•(**Cat10**)$_2$)

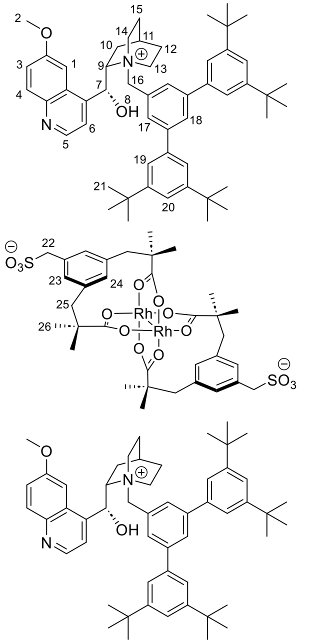

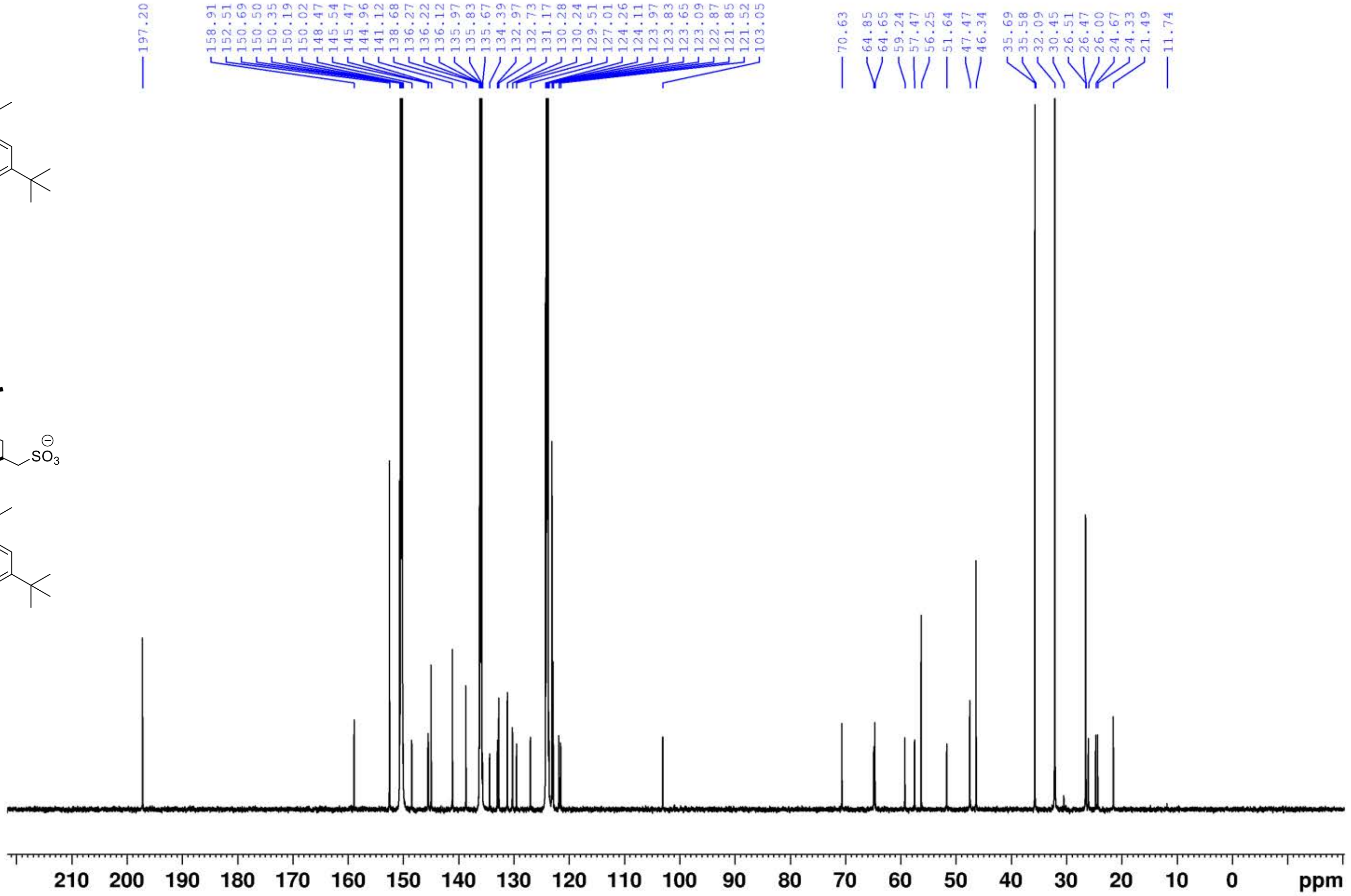

¹H NMR (700 MHz, C_5D_5N) for *Bis[rhodium (1S,2R,4S,5R)-2-((S)-(2-butyl-6-methoxyquinolin-4-yl)(hydroxy)methyl)-5-ethyl-1-((3,3'',5,5''-tetra-tert-butyl-[1,1':3',1''-terphenyl]-5'-yl)methyl)quinuclidin-1-ium (3,5-bis(2-carboxy-2-methylpropyl)benzenesulfonate)]* (Rh_2(**A-I**)$_2$•(**Cat11**)$_2$)

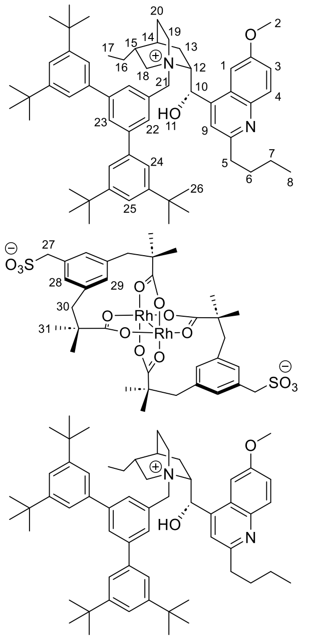

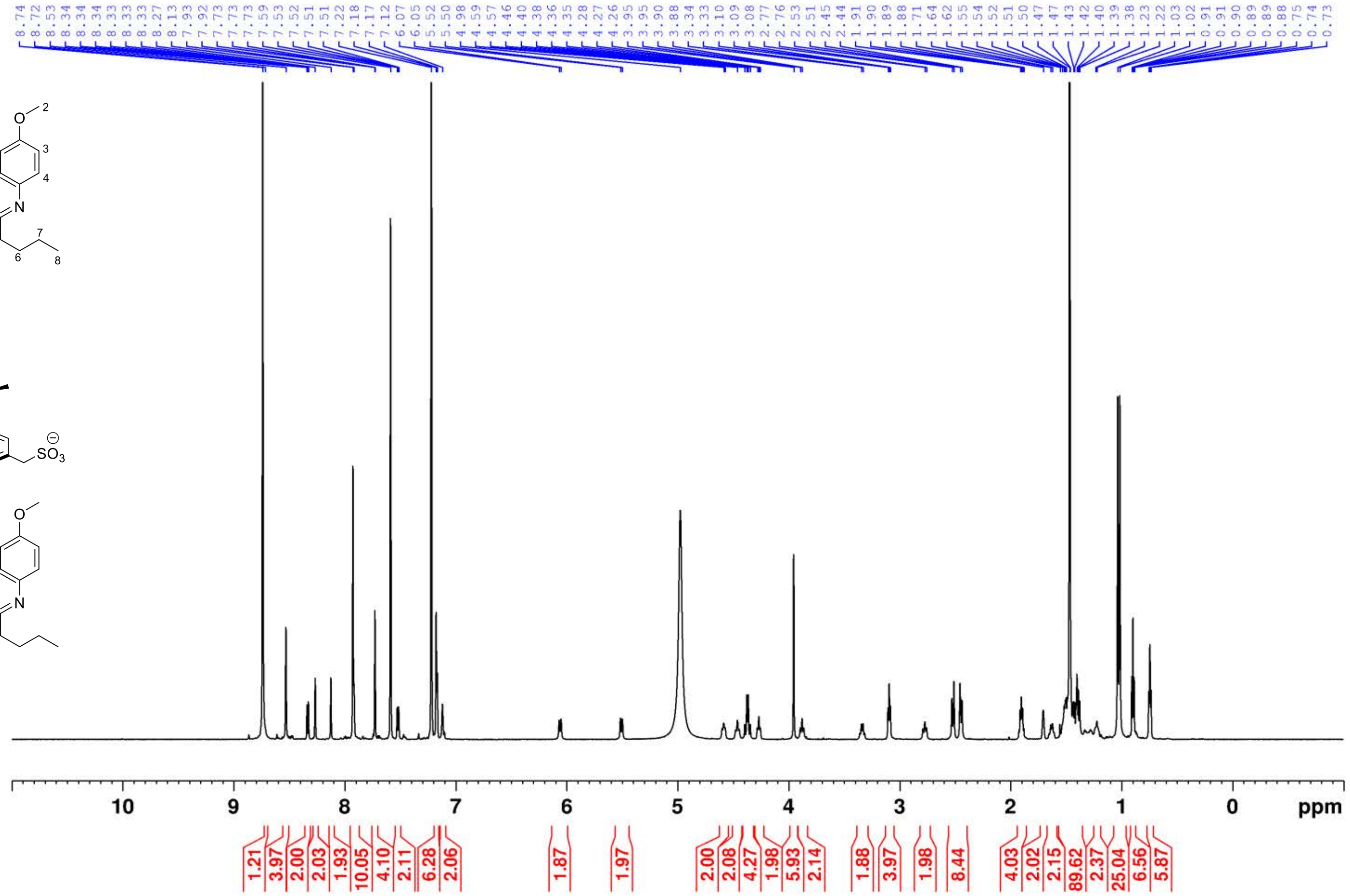

^{13}C NMR (176 MHz, C_5D_5N) for *Bis[rhodium (1S,2R,4S,5R)-2-((S)-(2-butyl-6-methoxyquinolin-4-yl)(hydroxy)methyl)-5-ethyl-1-((3,3'',5,5''-tetra-tert-butyl-[1,1':3',1''-terphenyl]-5'-yl)methyl)quinuclidin-1-ium (3,5-bis(2-carboxy-2-methylpropyl)benzenesulfonate)]* (Rh_2(**A-I**)$_2$•(**Cat11**)$_2$)

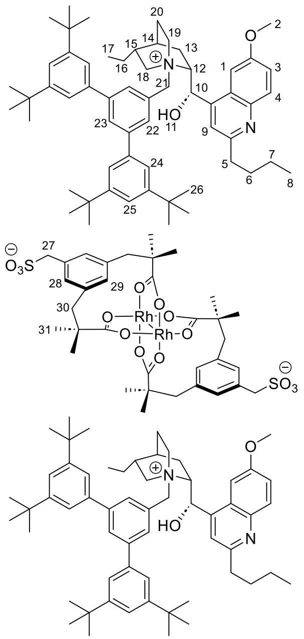

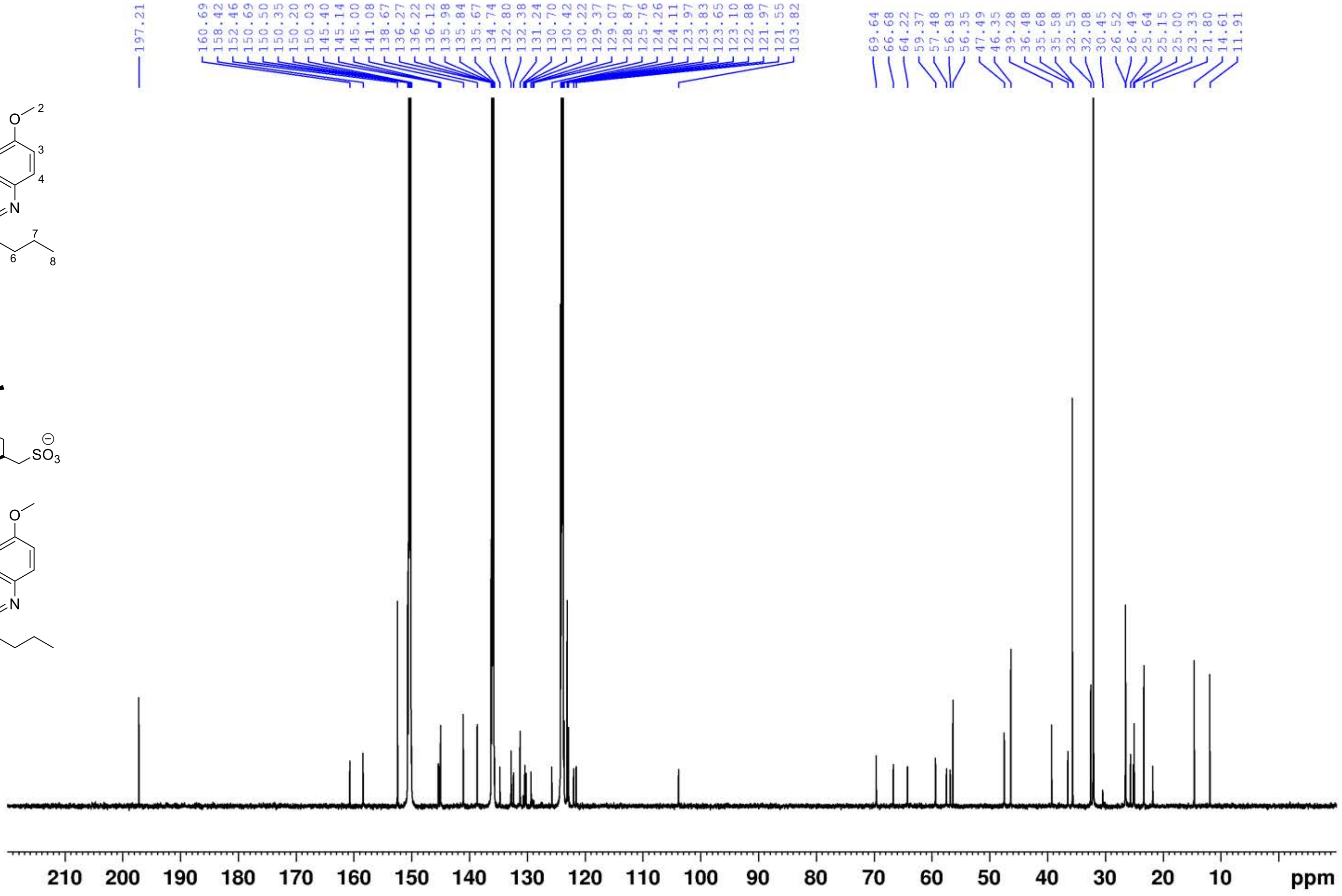

¹H NMR (700 MHz, C_5D_5N) for *Bis[rhodium (1S,2R,4S,5R)-5-ethyl-2-((S)-methoxy(6-methoxyquinolin-4-yl)methyl)-1-((3,3'',5,5''-tetra-tert-butyl-[1,1':3',1''-terphenyl]-5'-yl)methyl)quinuclidin-1-ium (3,5-bis(2-carboxy-2-methylpropyl)benzenesulfonate)]* (Rh$_2$(**A-I**)$_2$•(**Cat12**)$_2$)

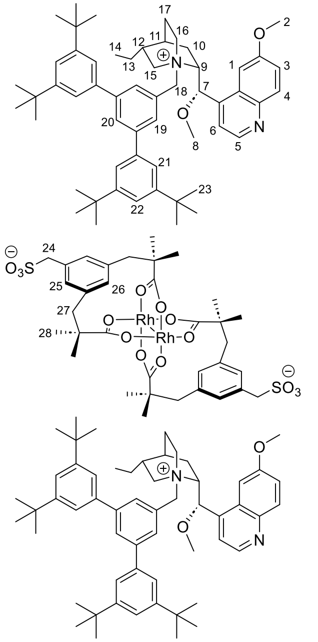

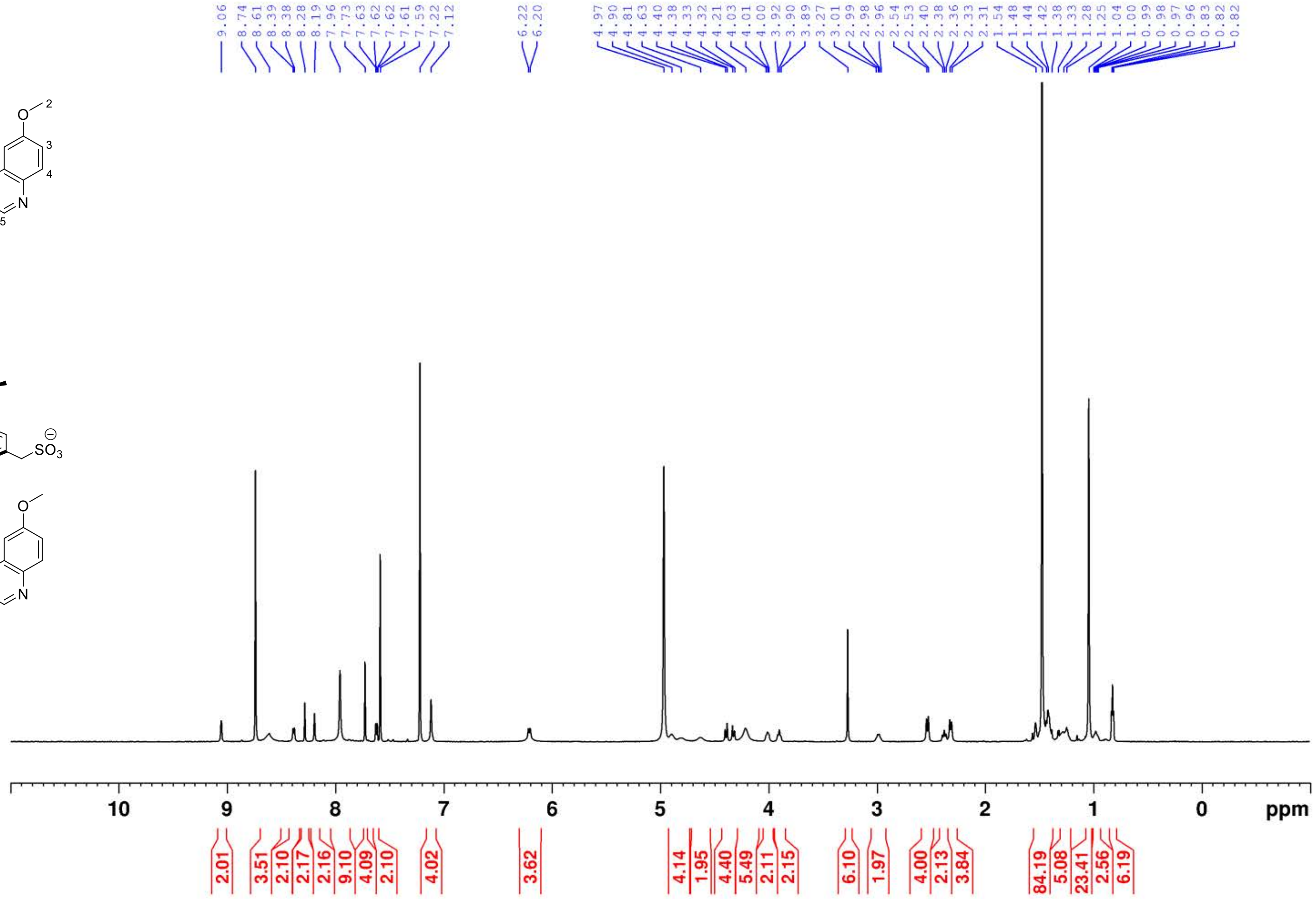

^{13}C NMR (176 MHz, C_5D_5N) for *Bis[rhodium (1S,2R,4S,5R)-5-ethyl-2-((S)-methoxy(6-methoxyquinolin-4-yl)methyl)-1-((3,3'',5,5''-tetra-tert-butyl-[1,1':3',1''-terphenyl]-5'-yl)methyl)quinuclidin-1-ium (3,5-bis(2-carboxy-2-methylpropyl)benzenesulfonate)]* (Rh_2(**A-I**)$_2$•(**Cat12**)$_2$)

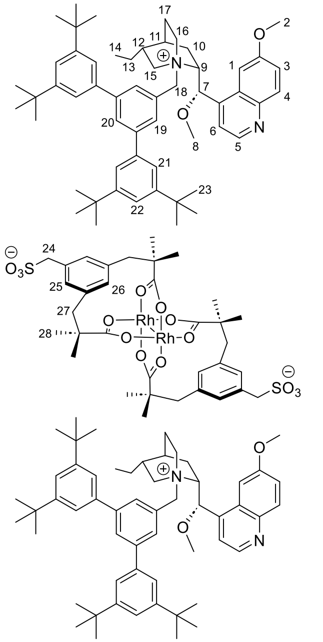

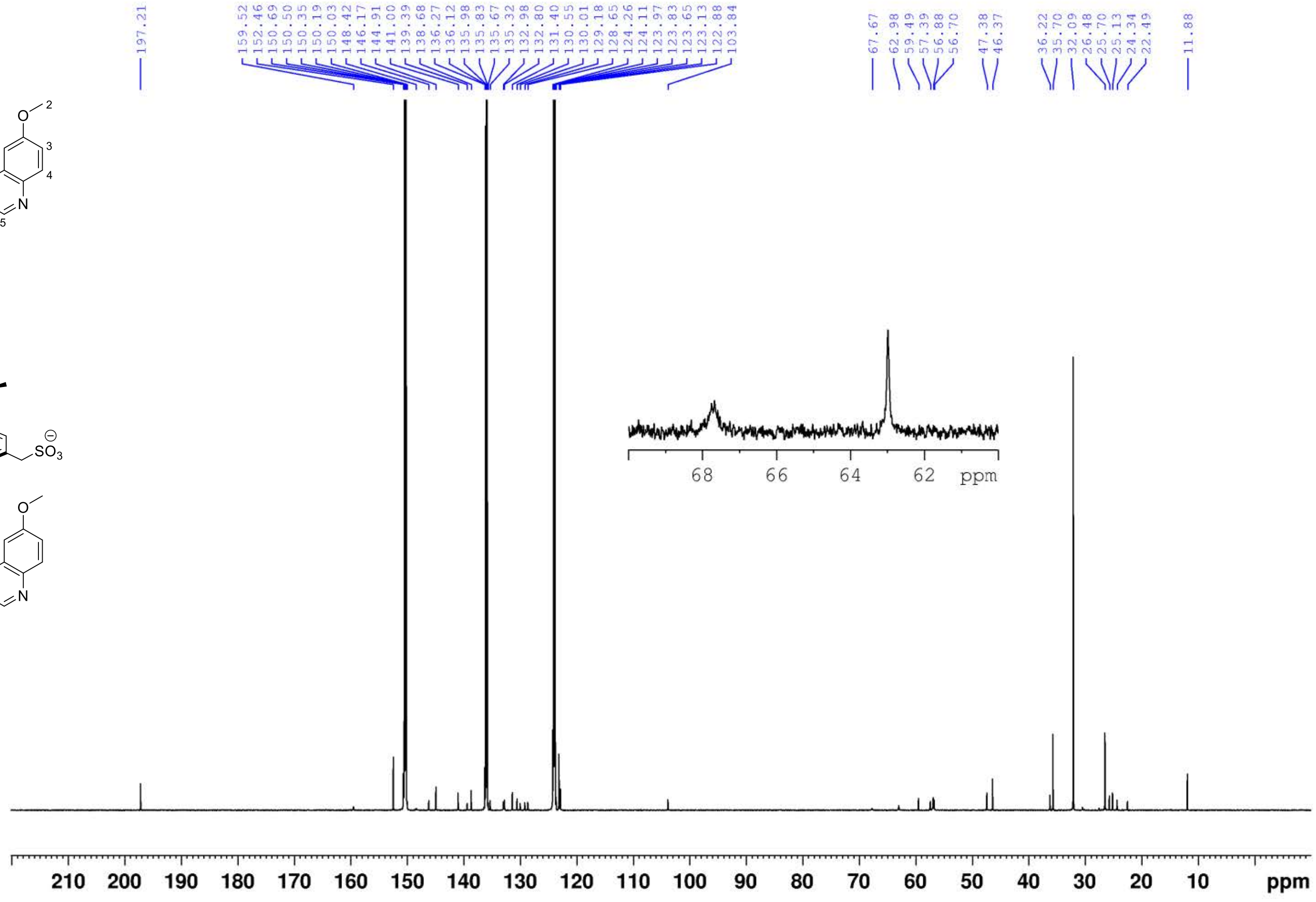

^{1}H NMR (700 MHz, C_5D_5N) for *Bis[rhodium (1S,2R,4S)-2-((S)-hydroxy(quinolin-5-yl)methyl)-1-((3,3'',5,5''-tetra-tert-butyl-[1,1':3',1''-terphenyl]-5'-yl)methyl)quinuclidin-1-ium (3',5'-bis(2-carboxy-2-methylpropyl)-[1,1'-biphenyl]-2-sulfonate)]* (Rh_2(**A-III**)$_2$•(**Cat8**)$_2$)

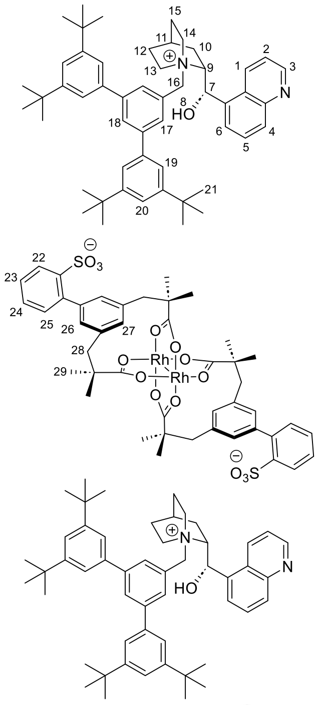

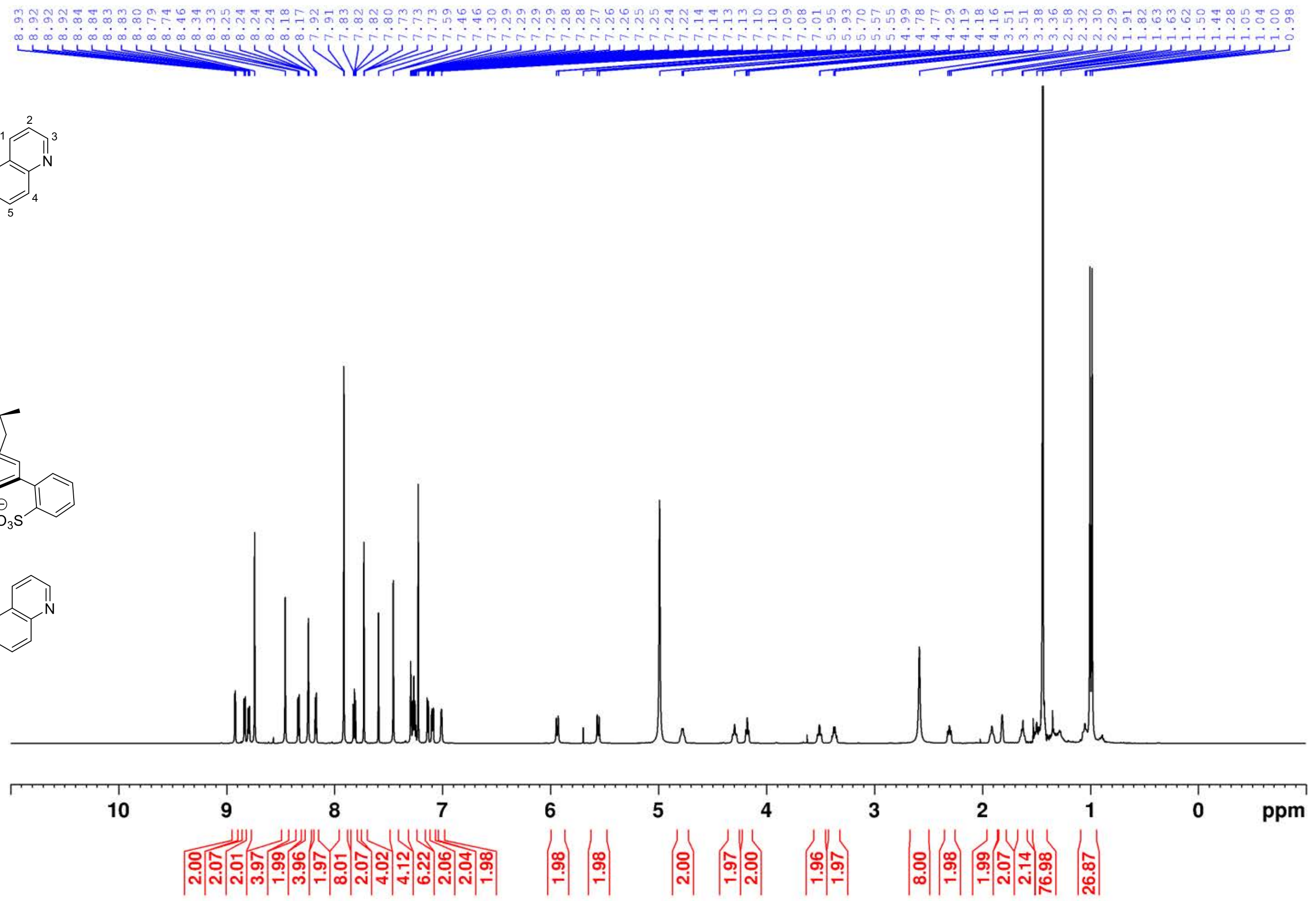

^{13}C NMR (176 MHz, C_5D_5N) for *Bis[rhodium (1S,2R,4S)-2-((S)-hydroxy(quinolin-5-yl)methyl)-1-((3,3'',5,5''-tetra-tert-butyl-[1,1':3',1''-terphenyl]-5'-yl)methyl)quinuclidin-1-ium (3',5'-bis(2-carboxy-2-methylpropyl)-[1,1'-biphenyl]-2-sulfonate)]* (Rh_2(**A-III**)$_2$•(**Cat8**)$_2$)

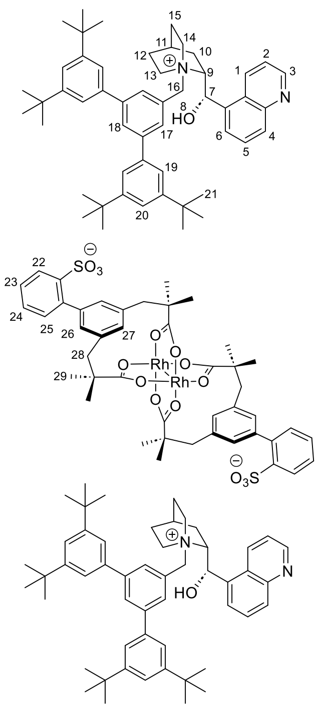

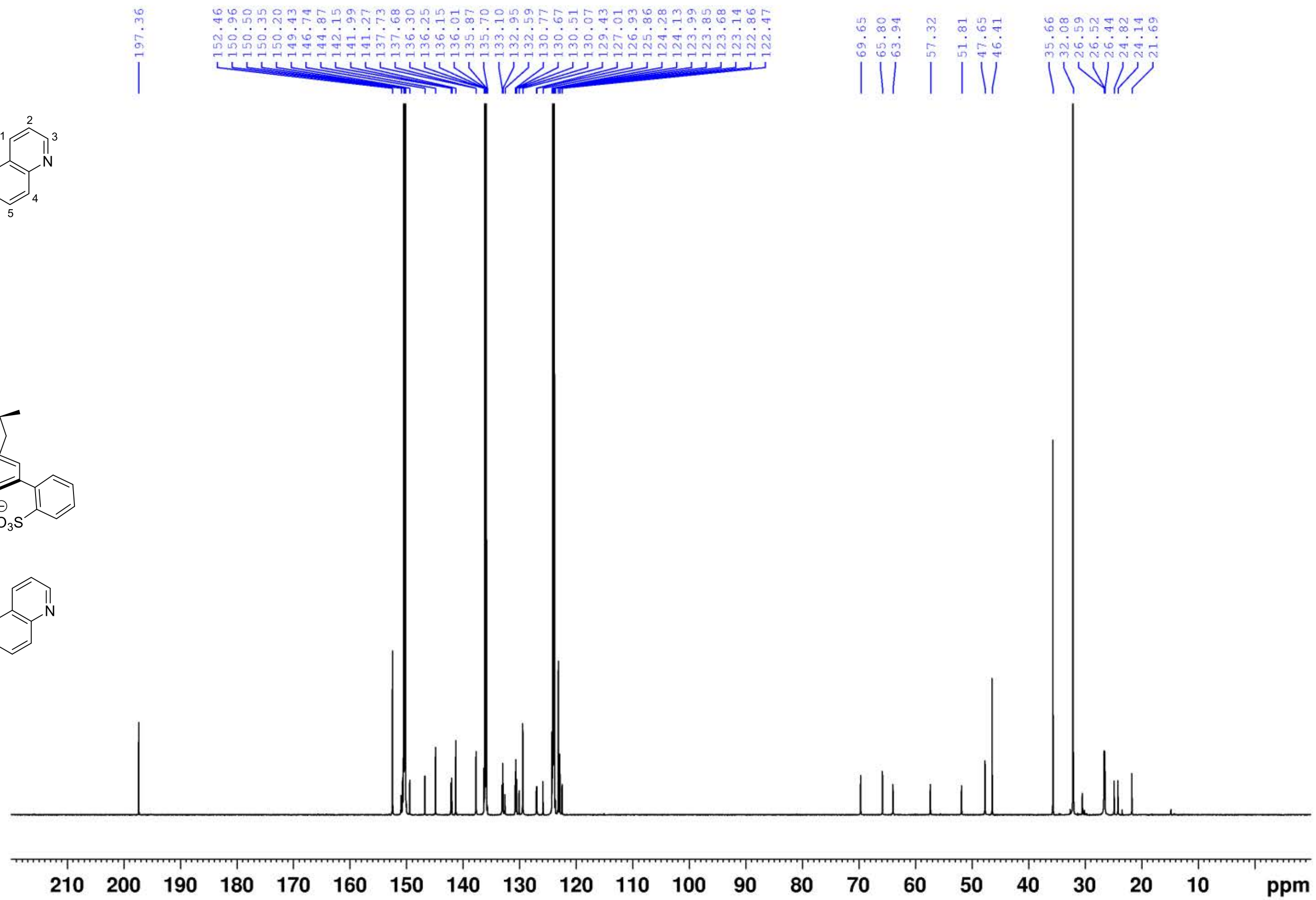

^{1}H NMR (400 MHz, $CDCl_3$) for *3-(o-tolyl)propan-1-ol* (**1c**)

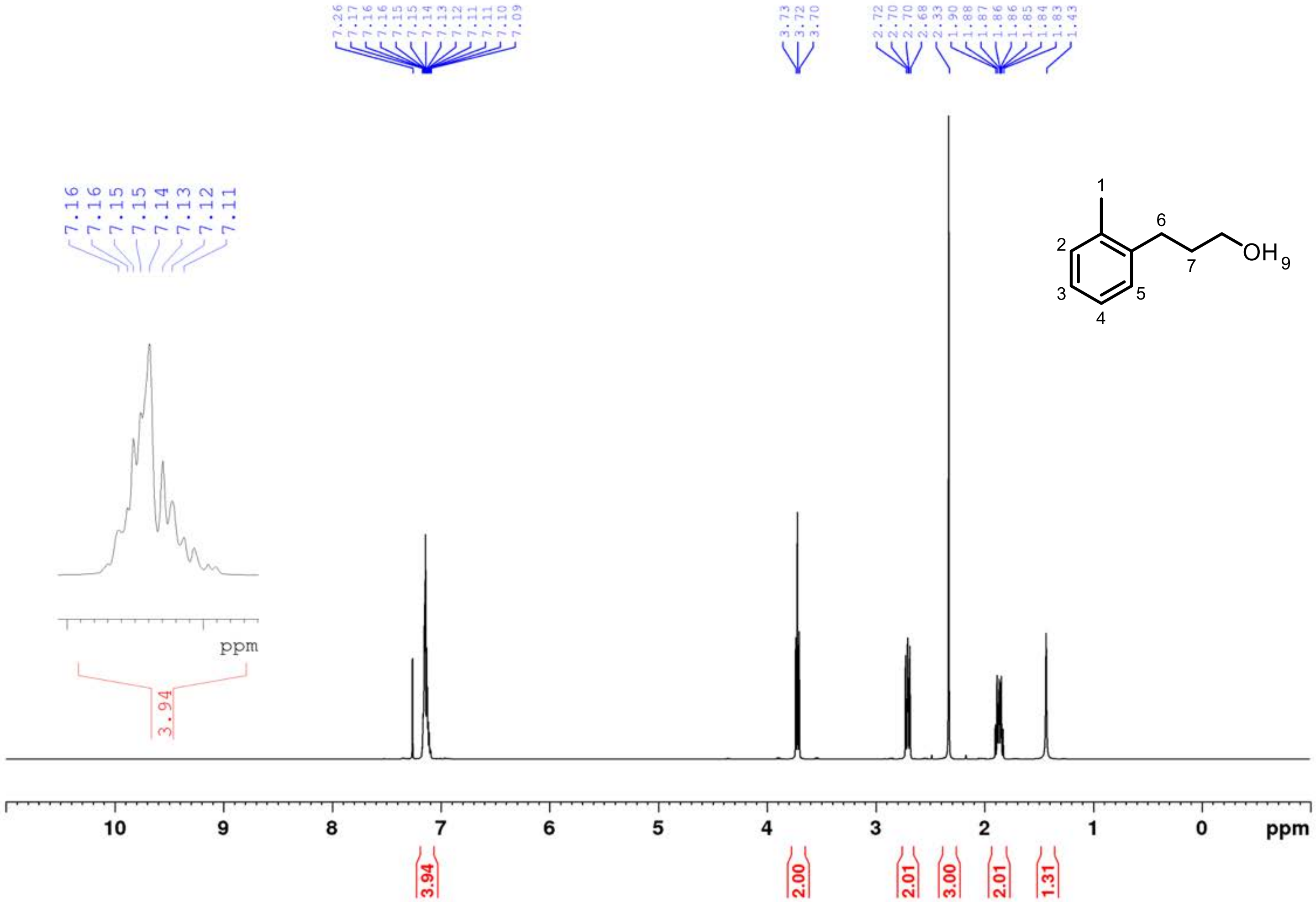

^{13}C NMR (101 MHz, CDCl$_3$) for *3-(o-tolyl)propan-1-ol* (**1c**)

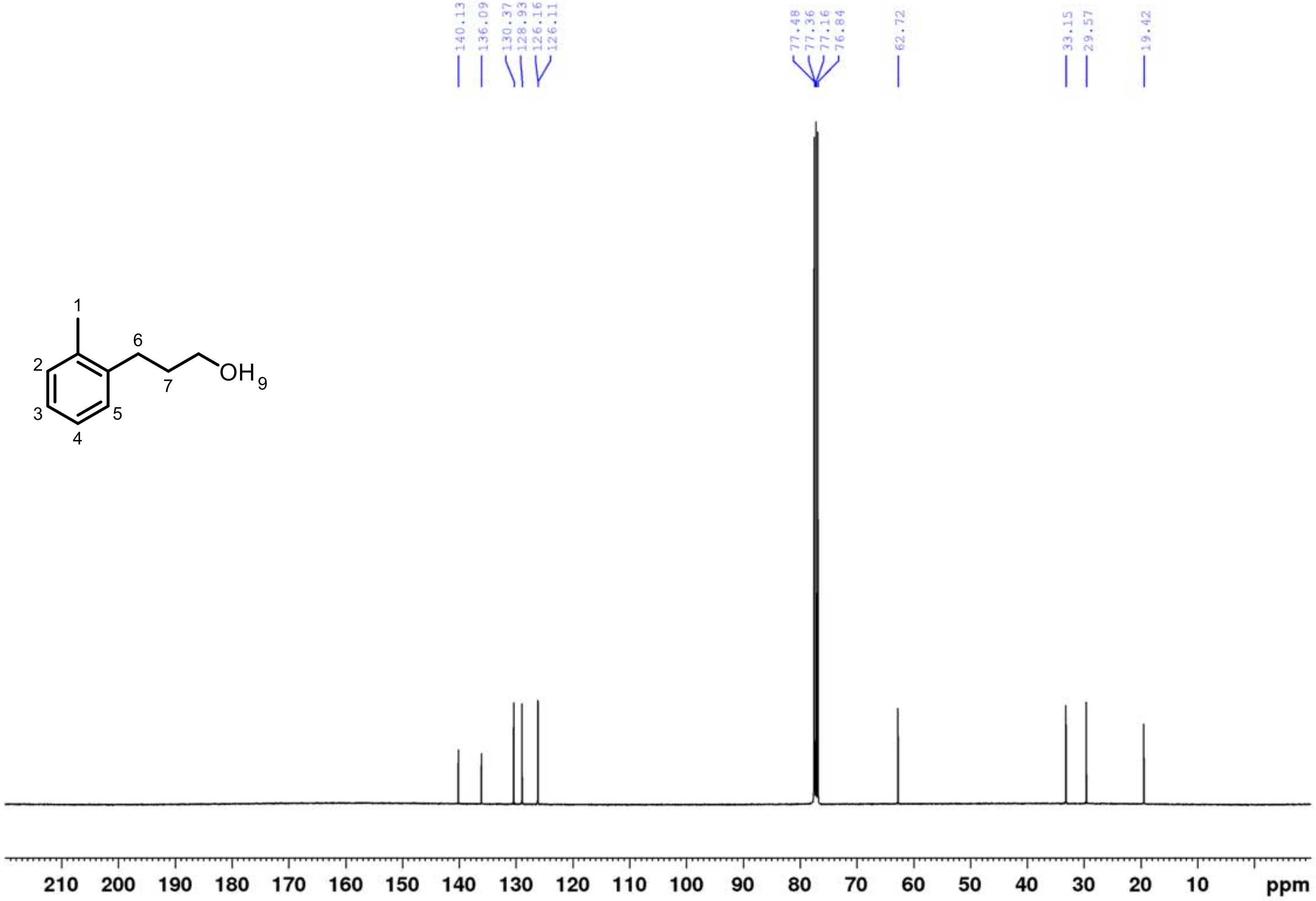

^{1}H NMR (400 MHz, $CDCl_3$) for *3-(2-methoxyphenyl)propan-1-ol* (**1d**)

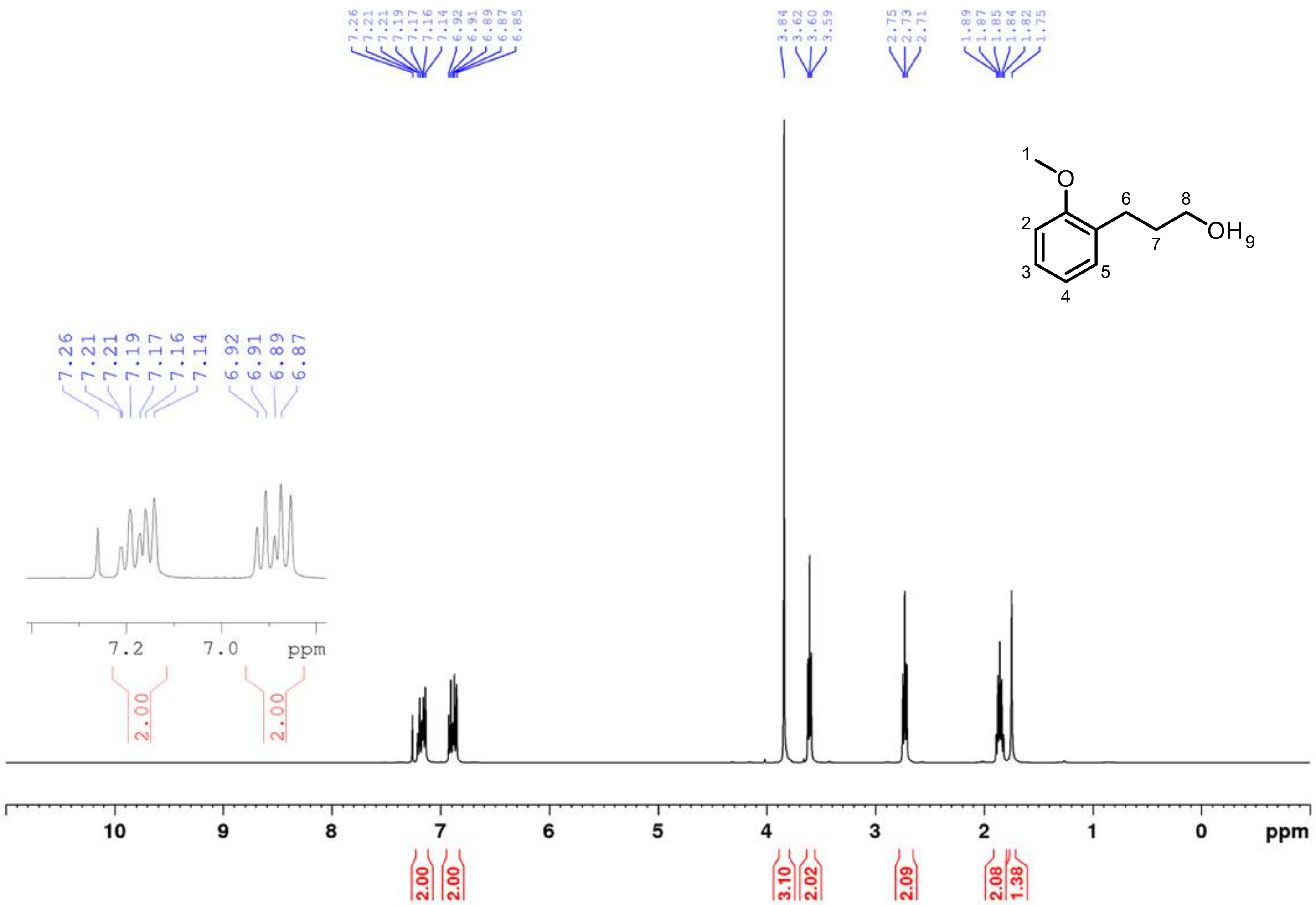

^{13}C NMR (101 MHz, $CDCl_3$) for *3-(2-methoxyphenyl)propan-1-ol* (**1d**)

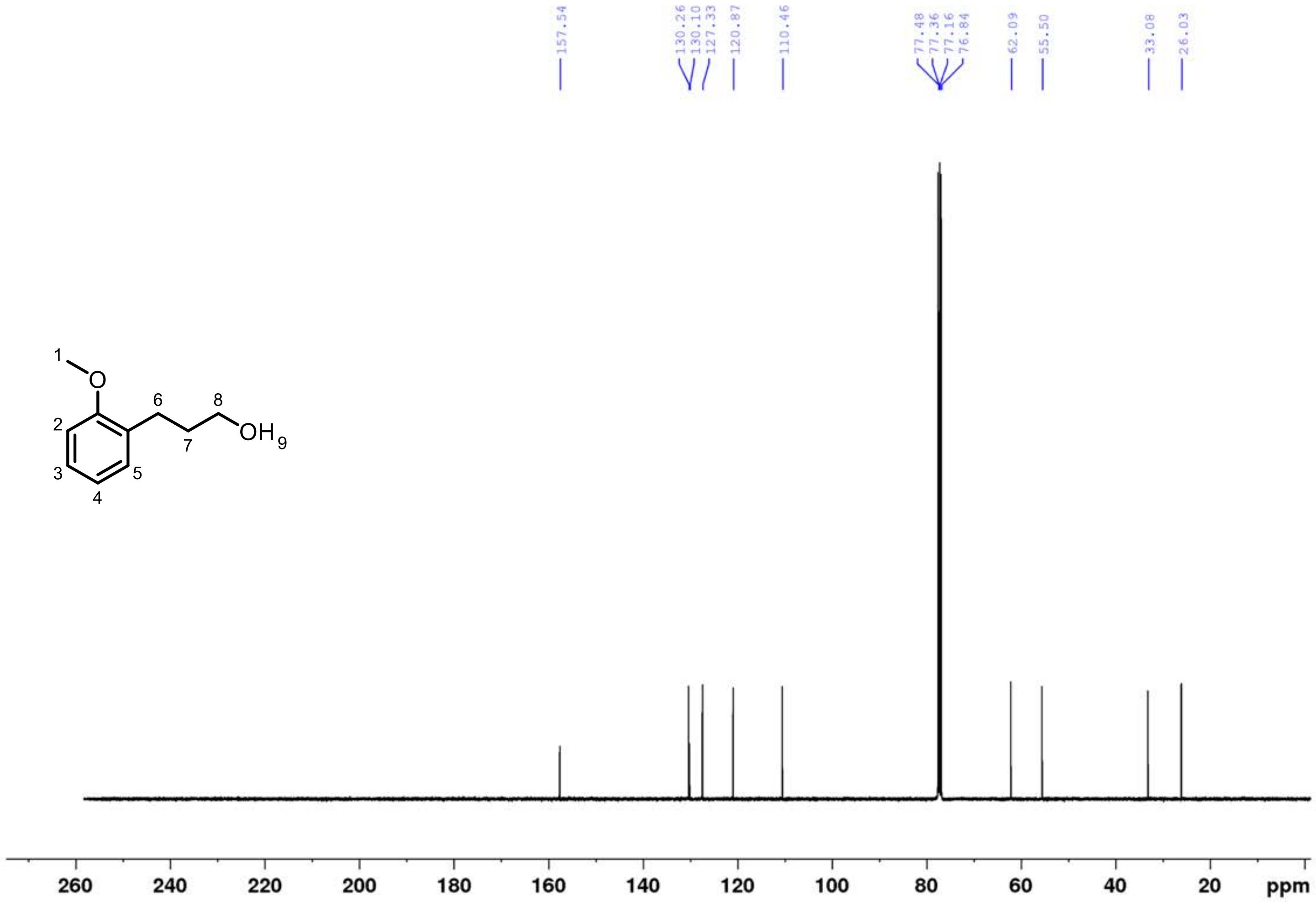

^{1}H NMR (400 MHz, $CDCl_3$) for *3-(2-chlorophenyl)propan-1-ol* (**1e**)

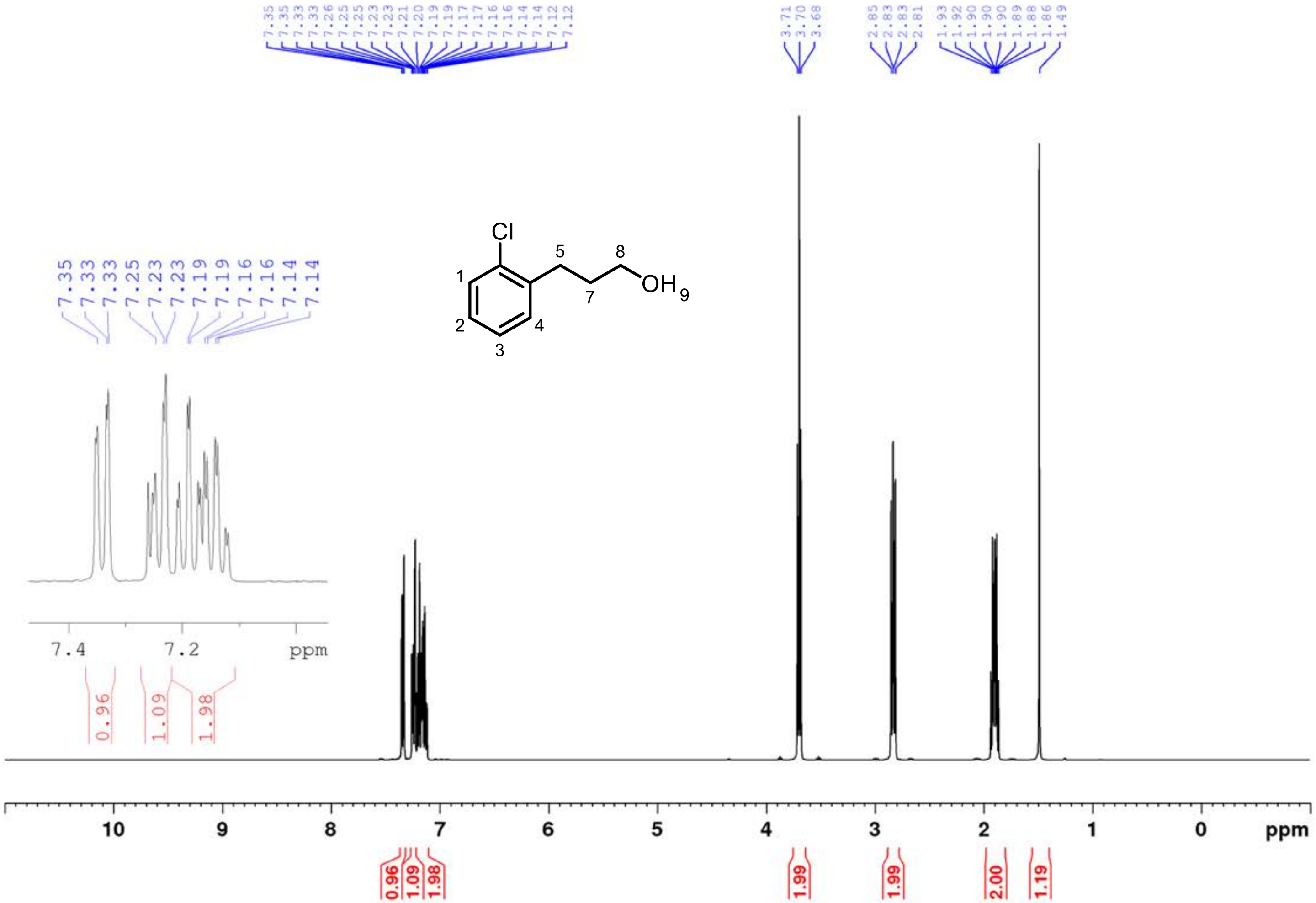

^{13}C NMR (101 MHz, $CDCl_3$) for *3-(2-chlorophenyl)propan-1-ol* (**1e**)

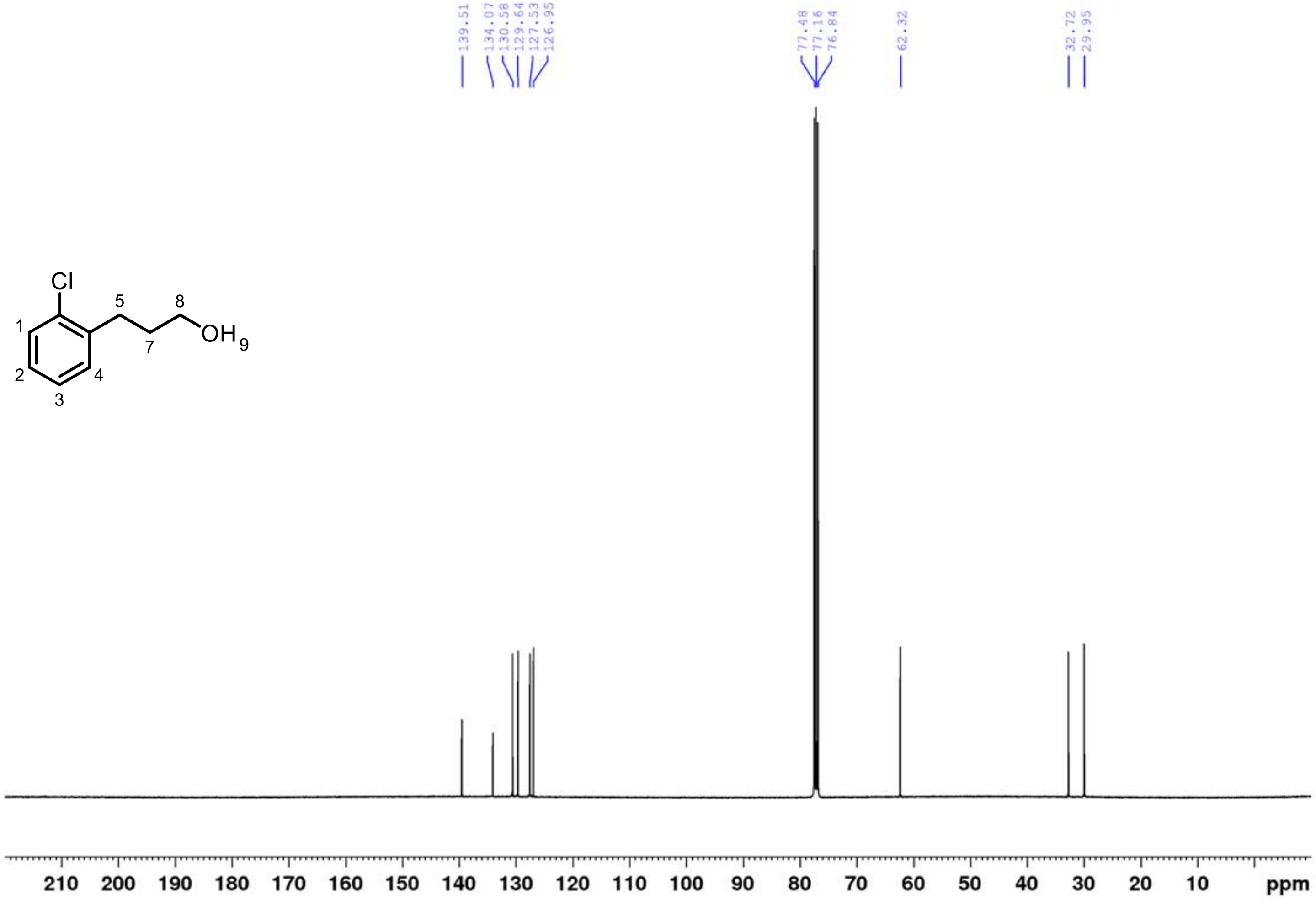

^{1}H NMR (400 MHz, $CDCl_3$) for *3-(m-tolyl)propan-1-ol* (**1g**)

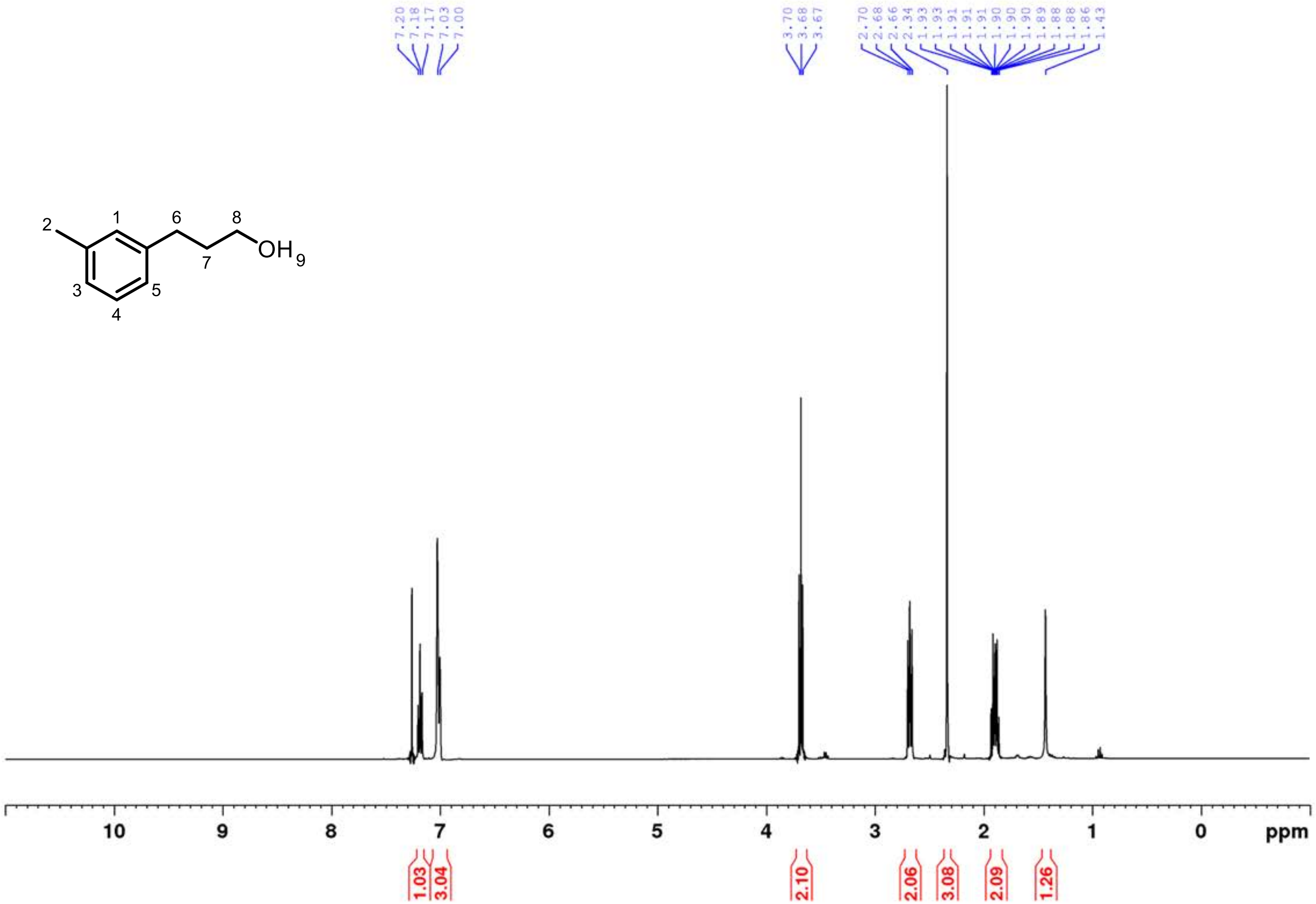

^{13}C NMR (101 MHz, CDCl$_3$) for *3-(m-tolyl)propan-1-ol* (**1g**)

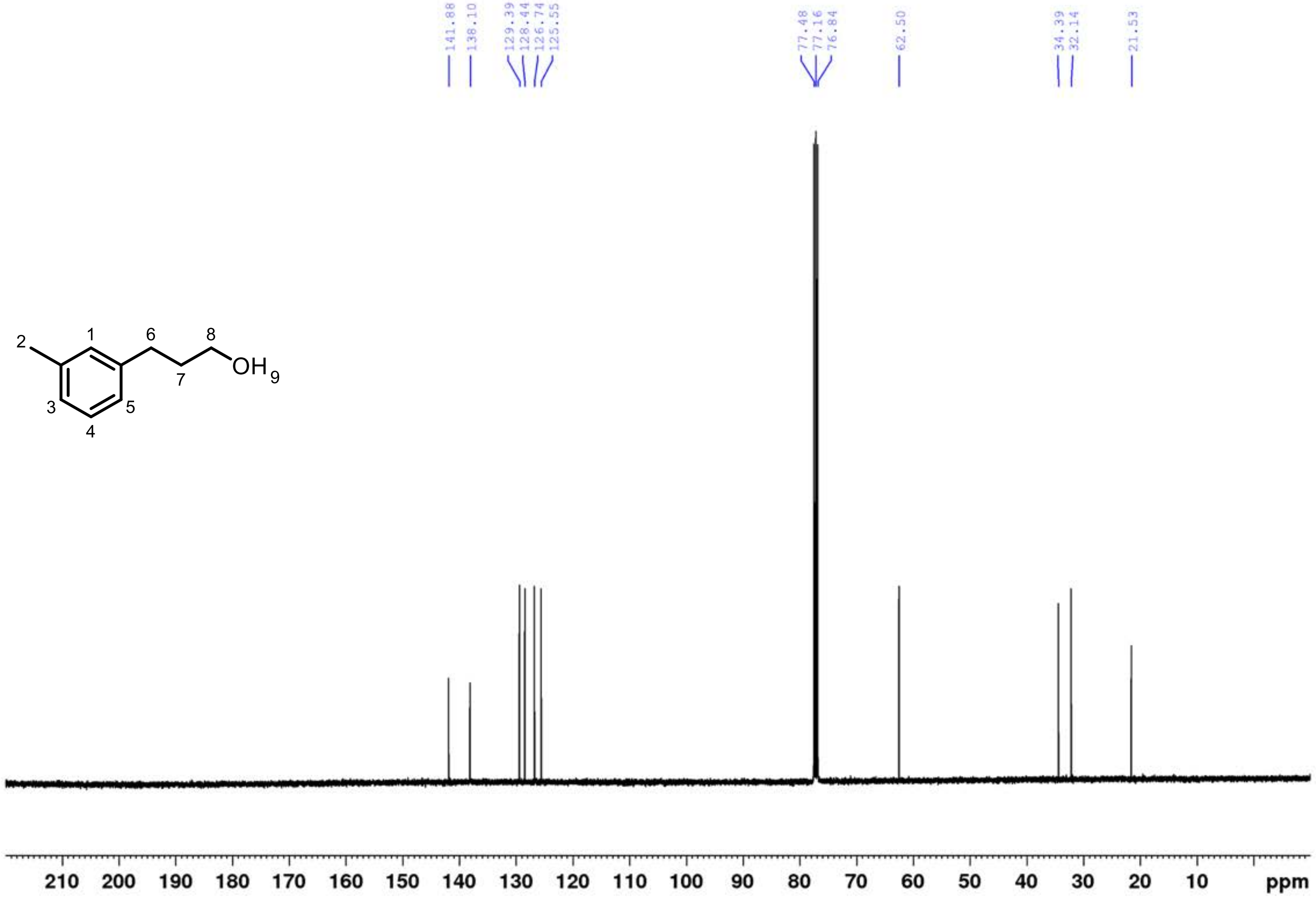

¹H NMR (400 MHz, CDCl₃) for *3-(3-(tert-butyl)phenyl)prop-2-yn-1-ol*

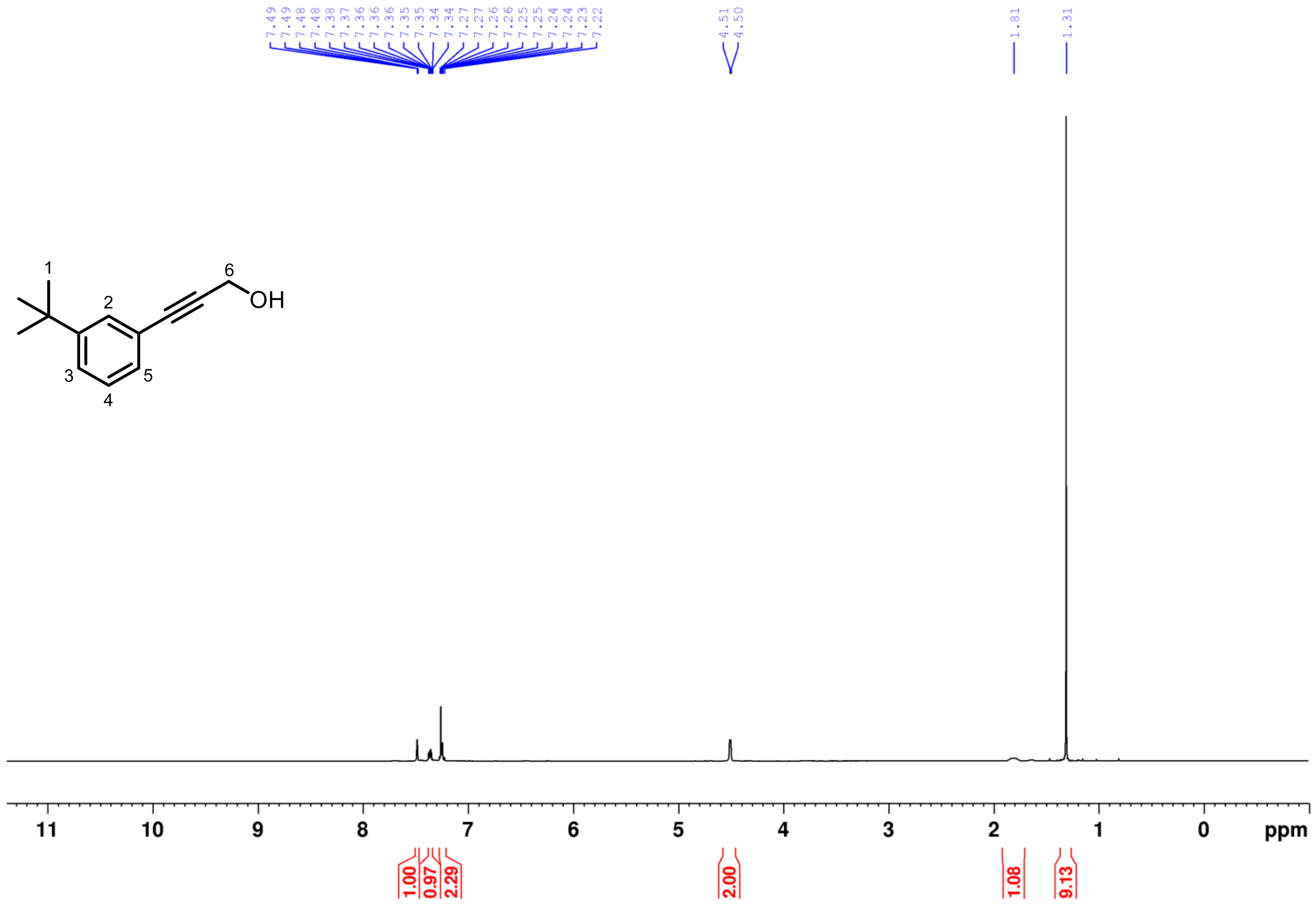

^{13}C NMR (101 MHz, CDCl$_3$) for *3-(3-(tert-butyl)phenyl)prop-2-yn-1-ol*

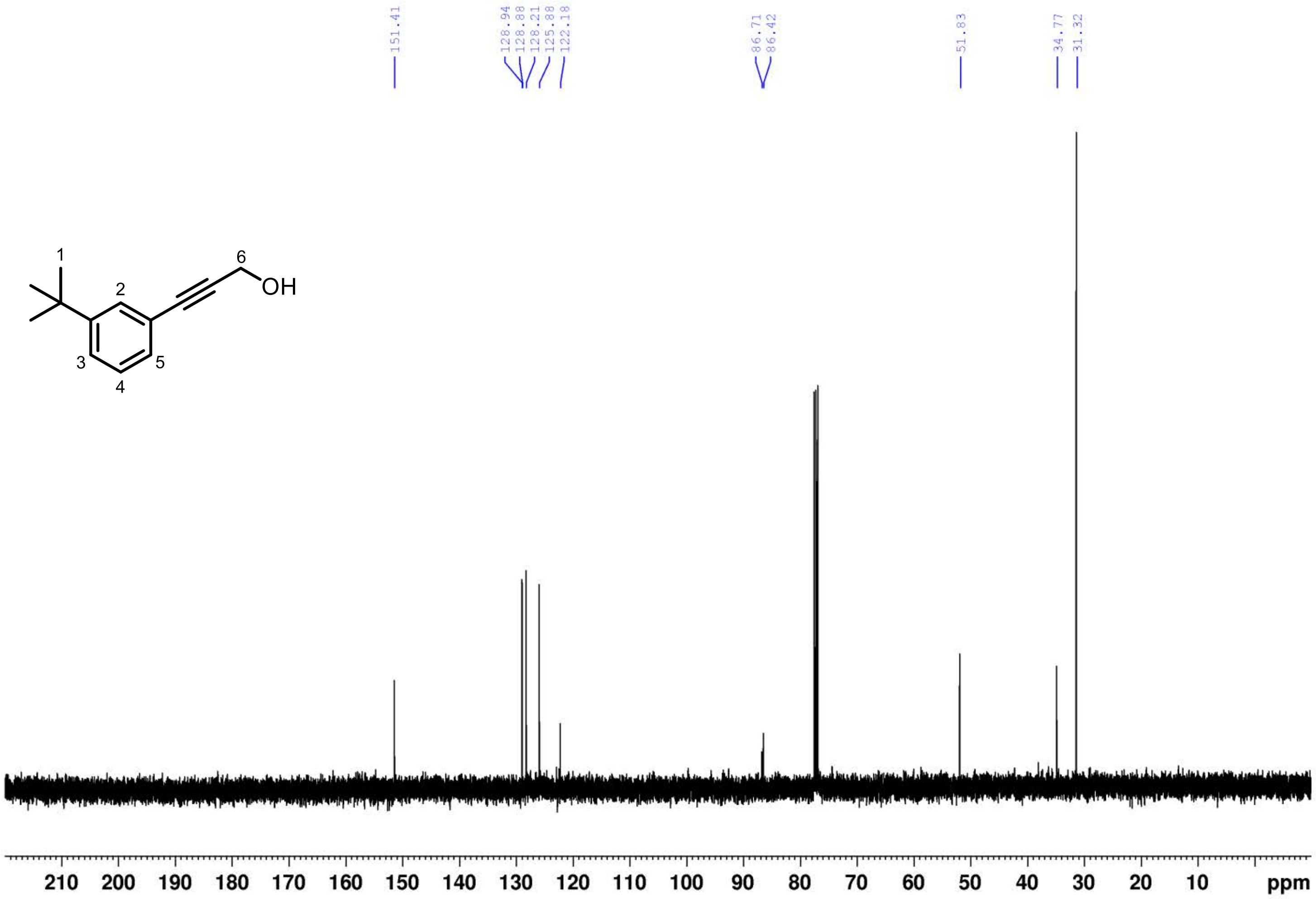

^{1}H NMR (400 MHz, $CDCl_3$) for *3-(3-(tert-butyl)phenyl)propan-1-ol* (**1h**)

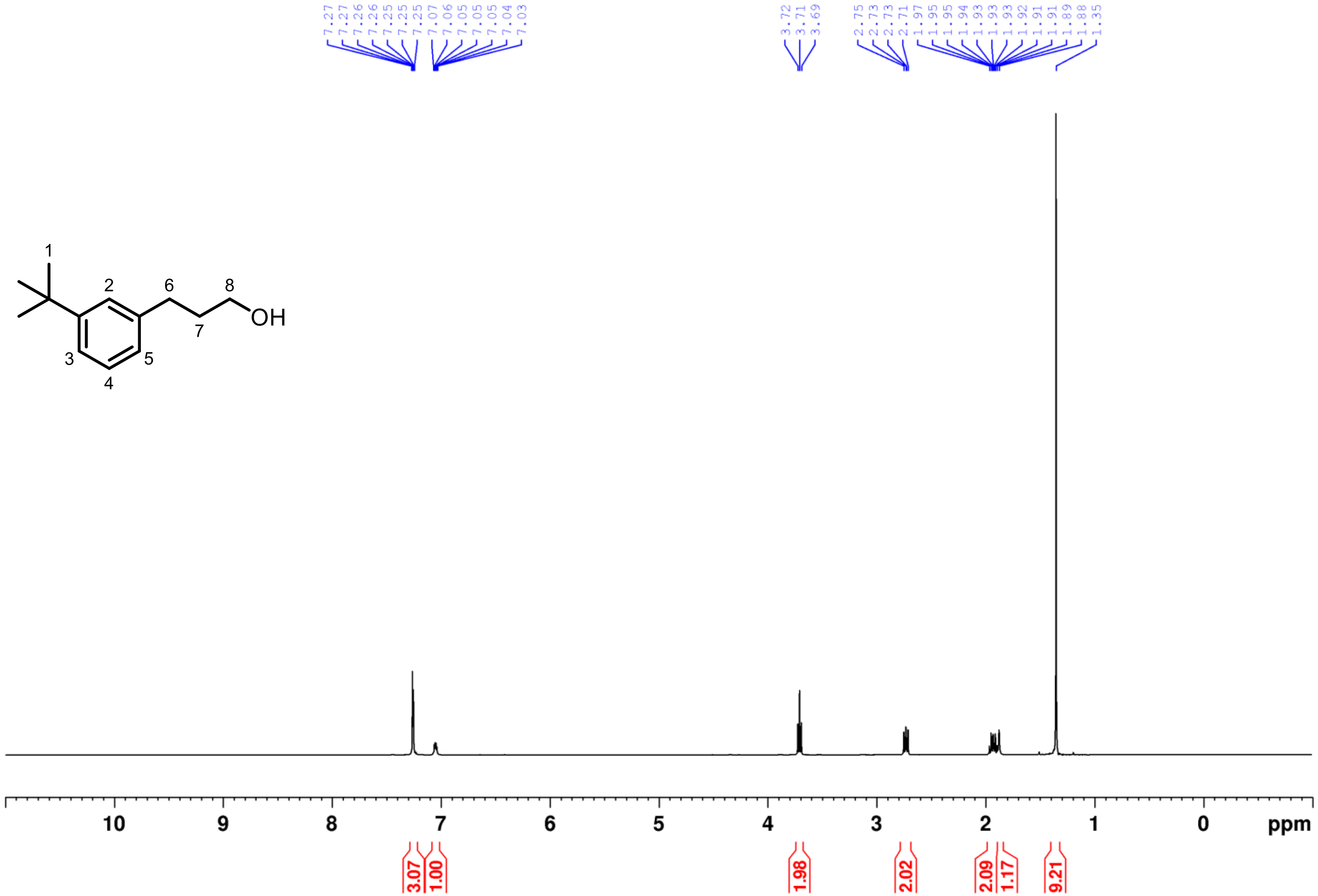

^{13}C NMR (101 MHz, $CDCl_3$) for *3-(3-(tert-butyl)phenyl)propan-1-ol* (**1h**)

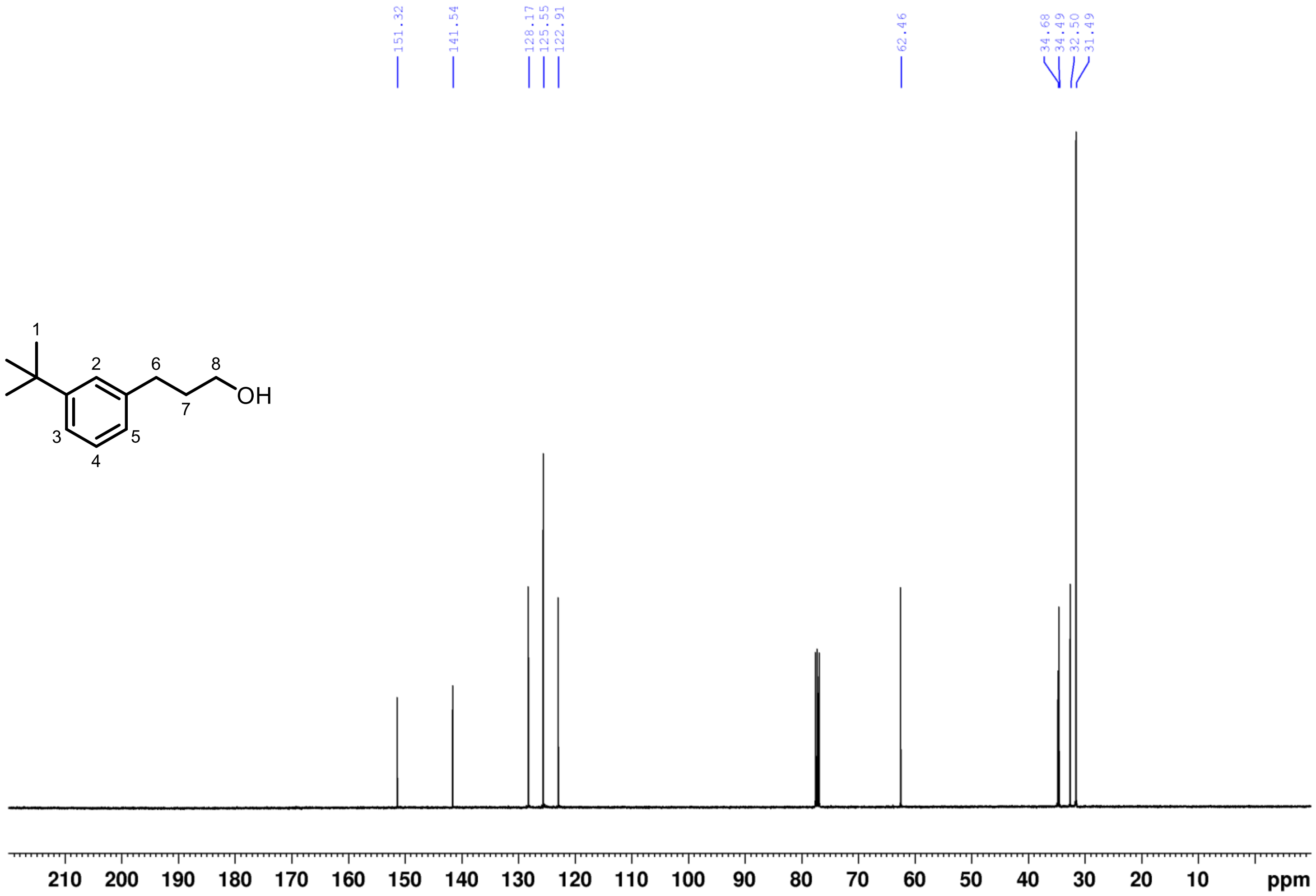

^{1}H NMR (400 MHz, $CDCl_3$) for *ethyl 3-(3-hydroxyprop-1-yn-1-yl)benzoate*

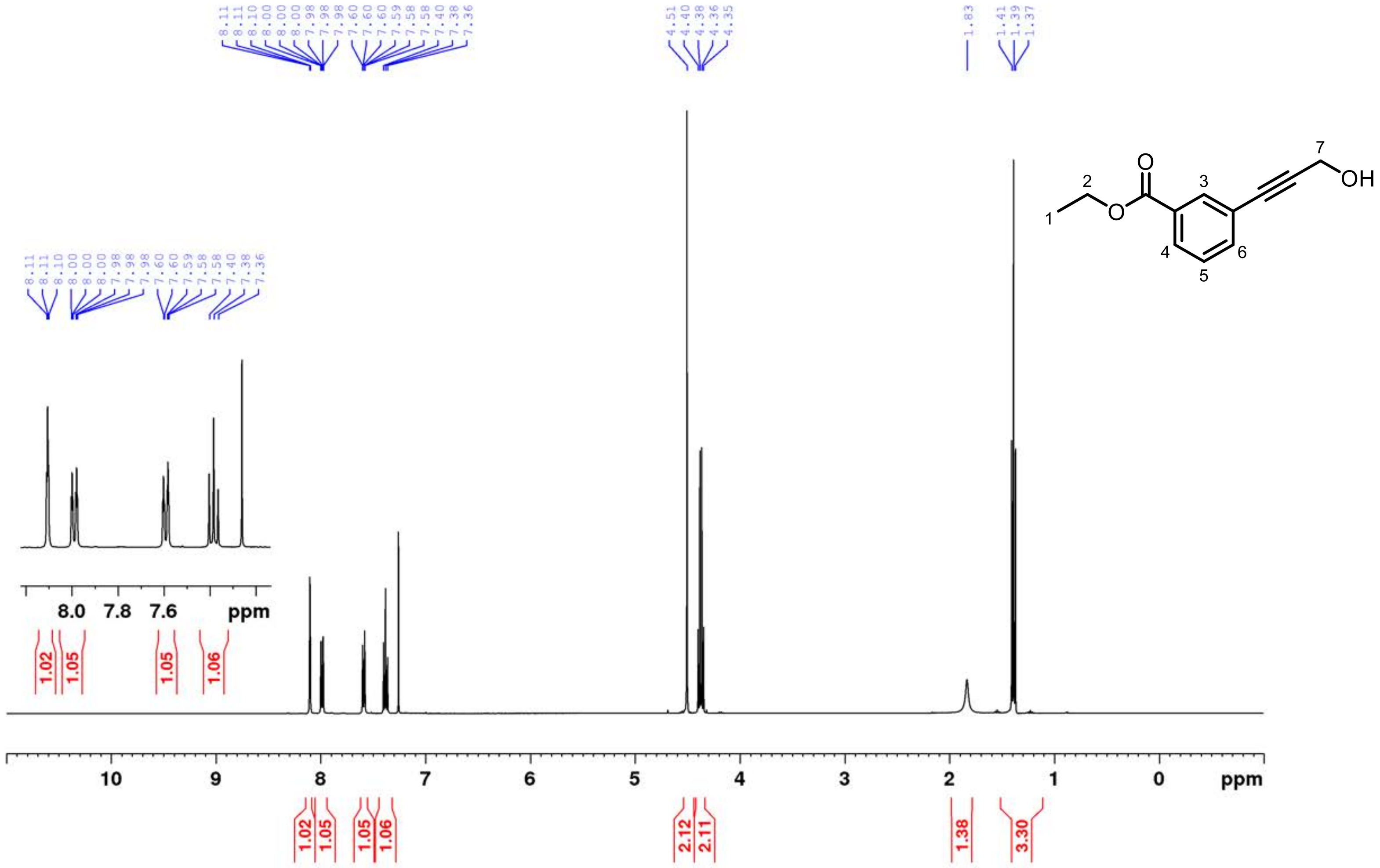

^{13}C NMR (101 MHz, $CDCl_3$) for *ethyl 3-(3-hydroxyprop-1-yn-1-yl)benzoate*

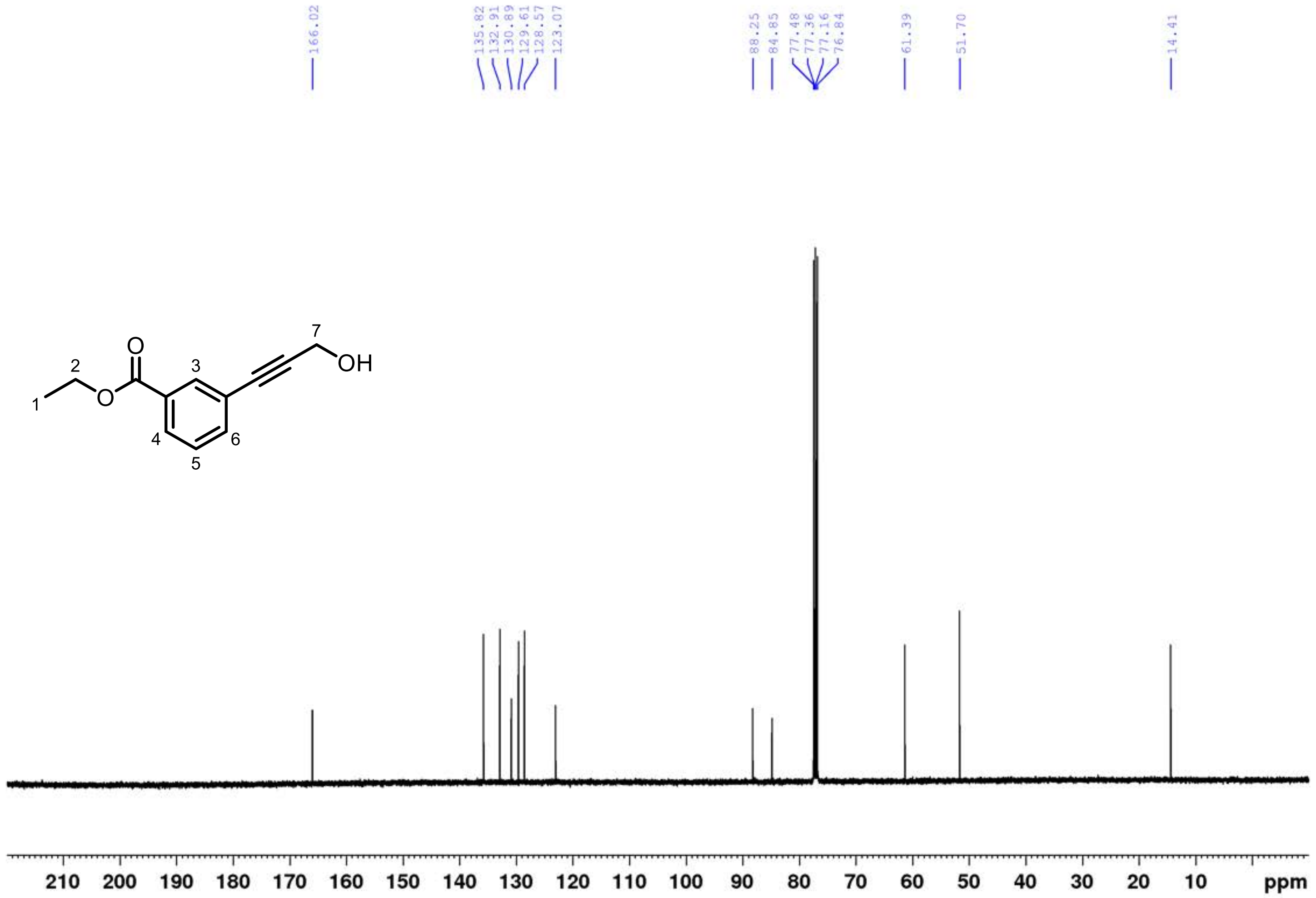

^{1}H NMR (400 MHz, CDCl$_3$) for *ethyl 3-(3-hydroxypropyl)benzoate* (**1j**)

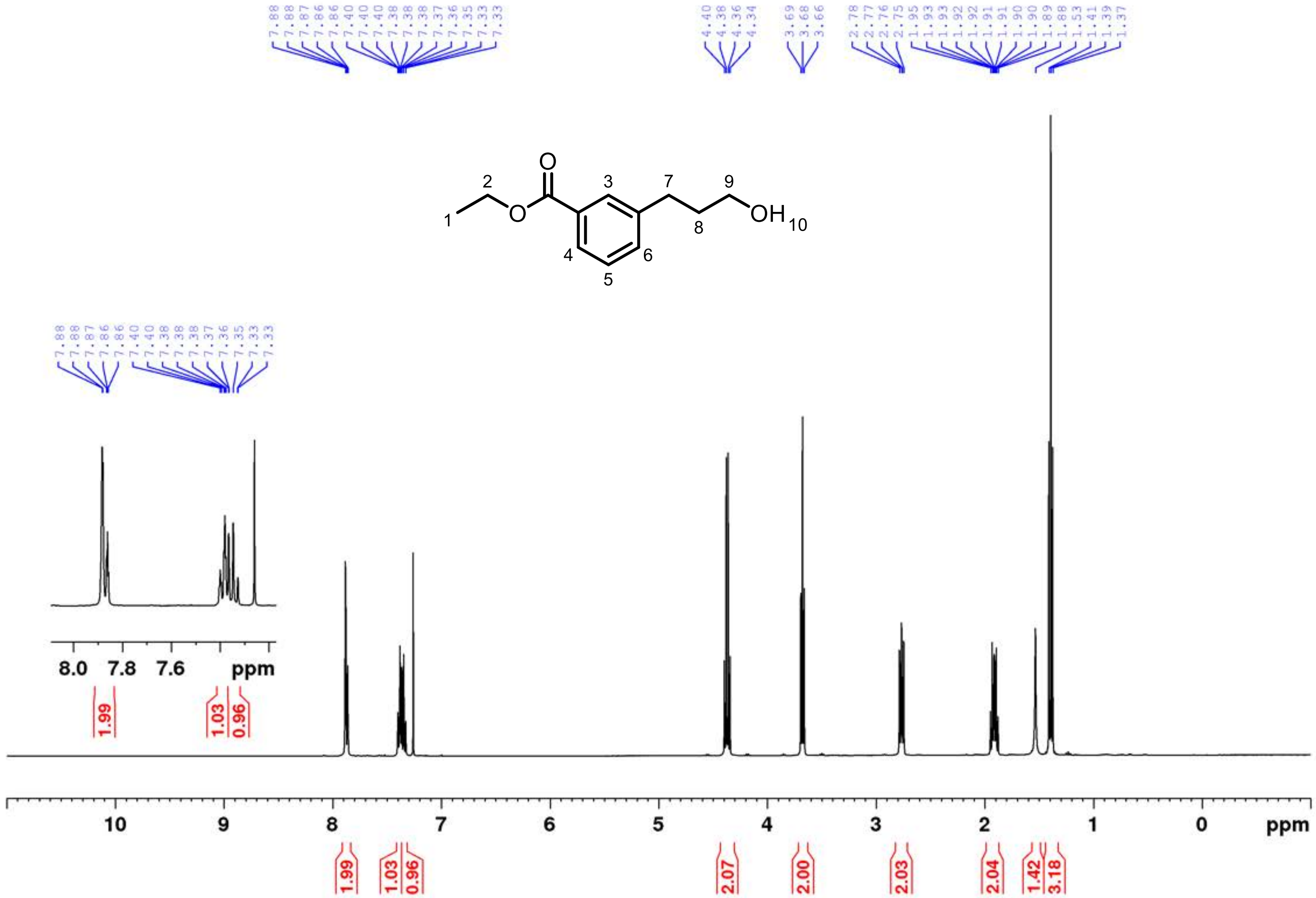

^{13}C NMR (101 MHz, CDCl$_3$) for *ethyl 3-(3-hydroxypropyl)benzoate* (**1j**)

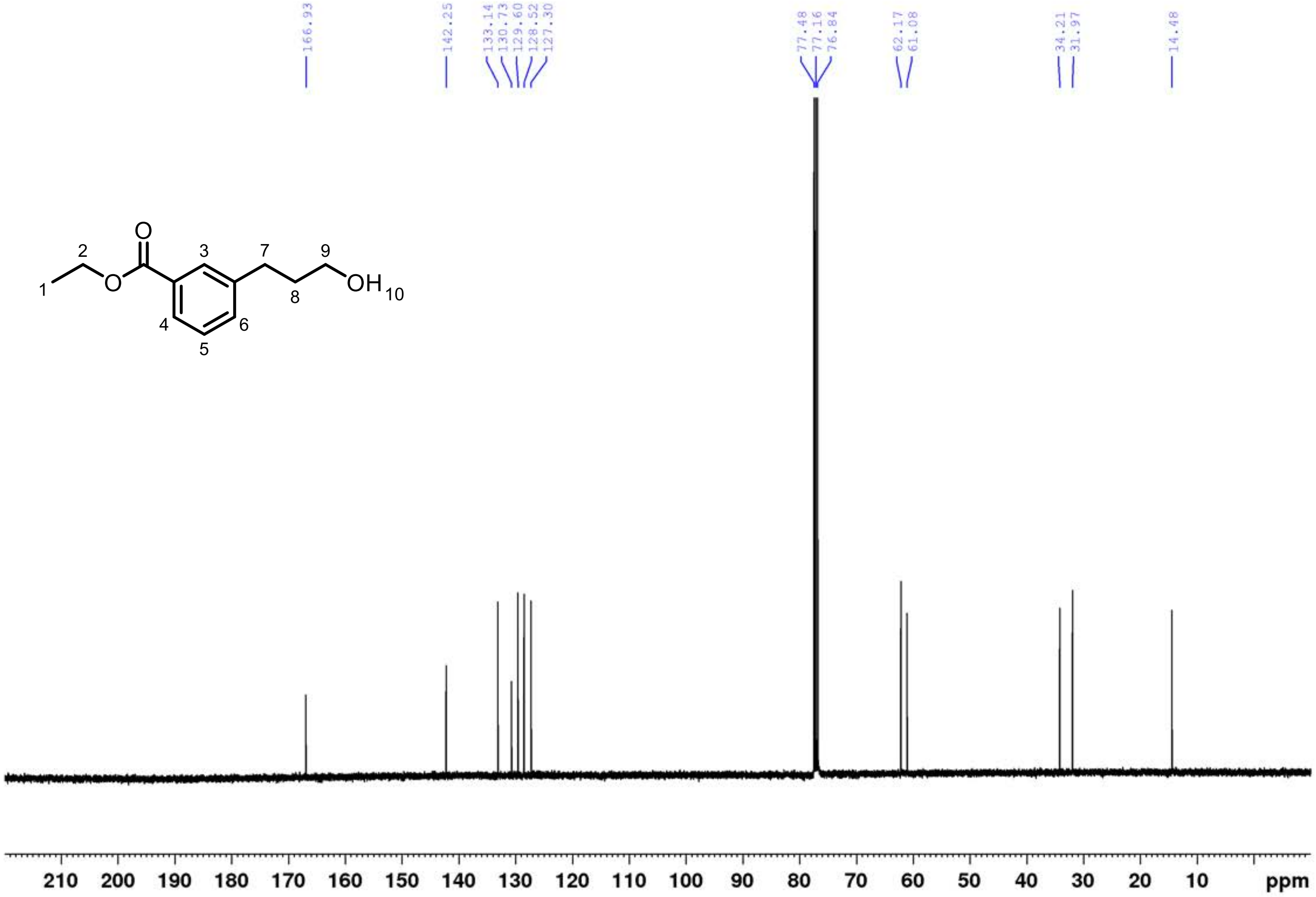

^{1}H NMR (400 MHz, $CDCl_3$) for *3-(naphthalen-1-yl)propan-1-ol* (**1k**)

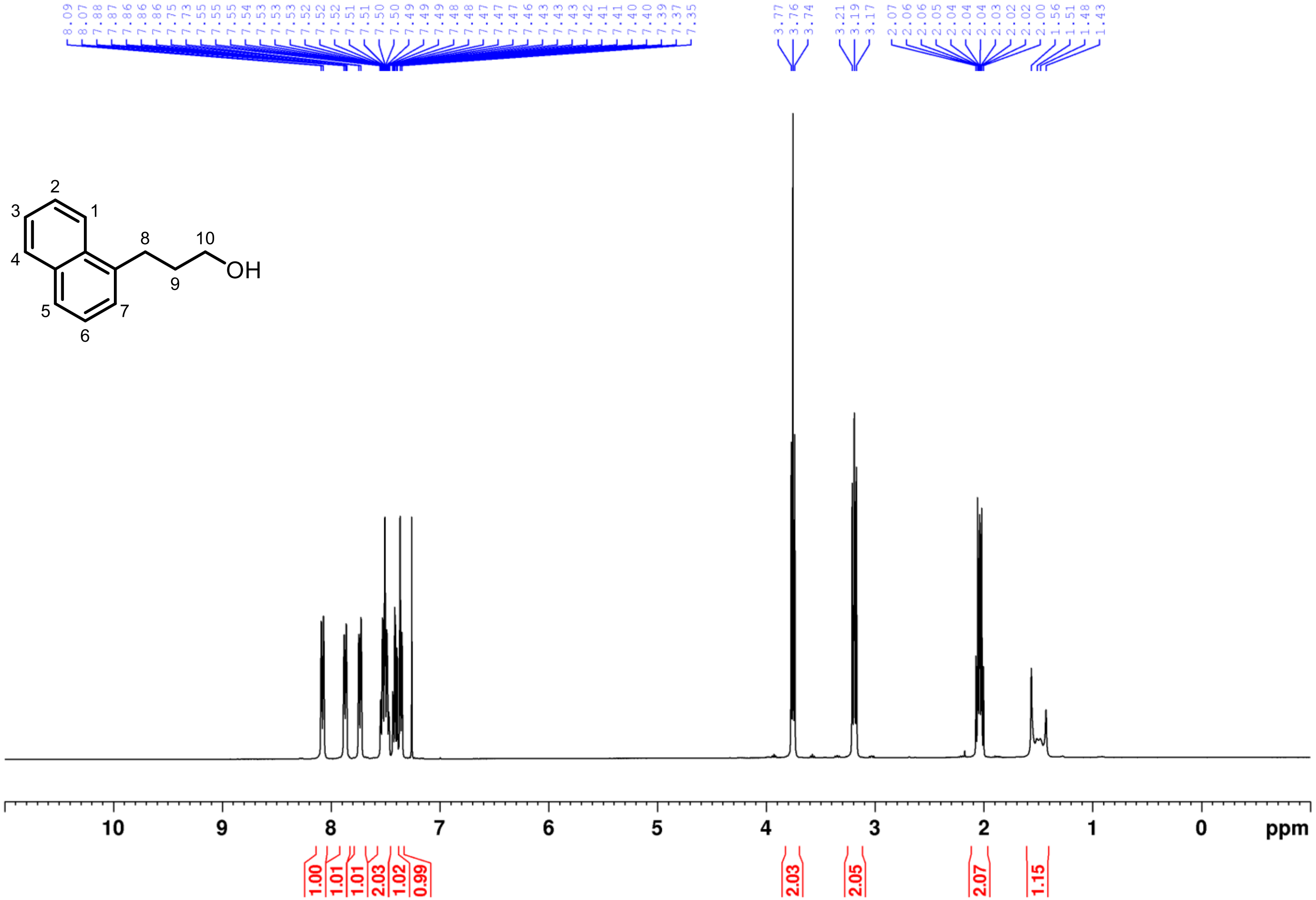

^{13}C NMR (101 MHz, $CDCl_3$) for *3-(naphthalen-1-yl)propan-1-ol* (**1k**)

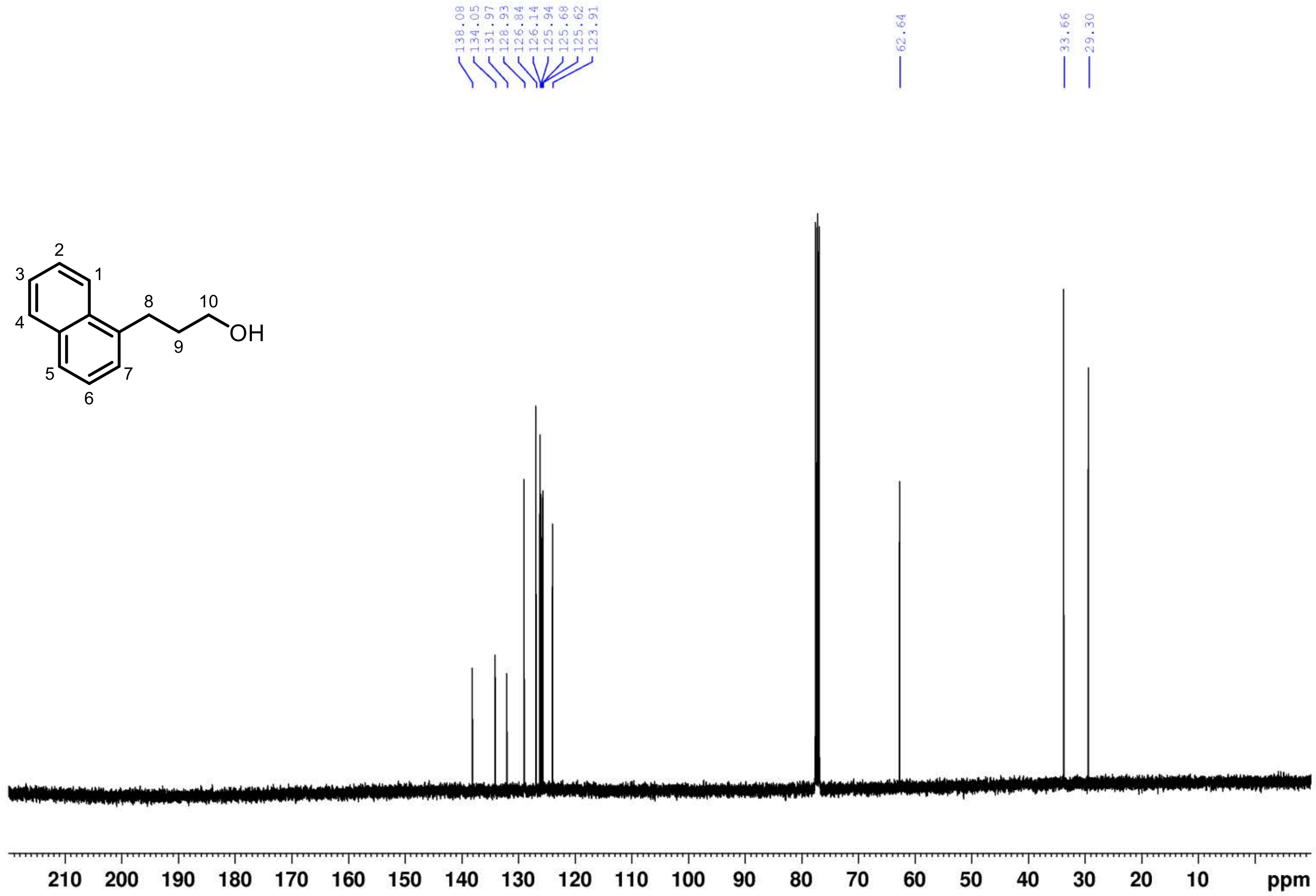

^{1}H NMR (400 MHz, $CDCl_3$) for *3-(4-methoxyphenyl)propan-1-ol* (**1m**)

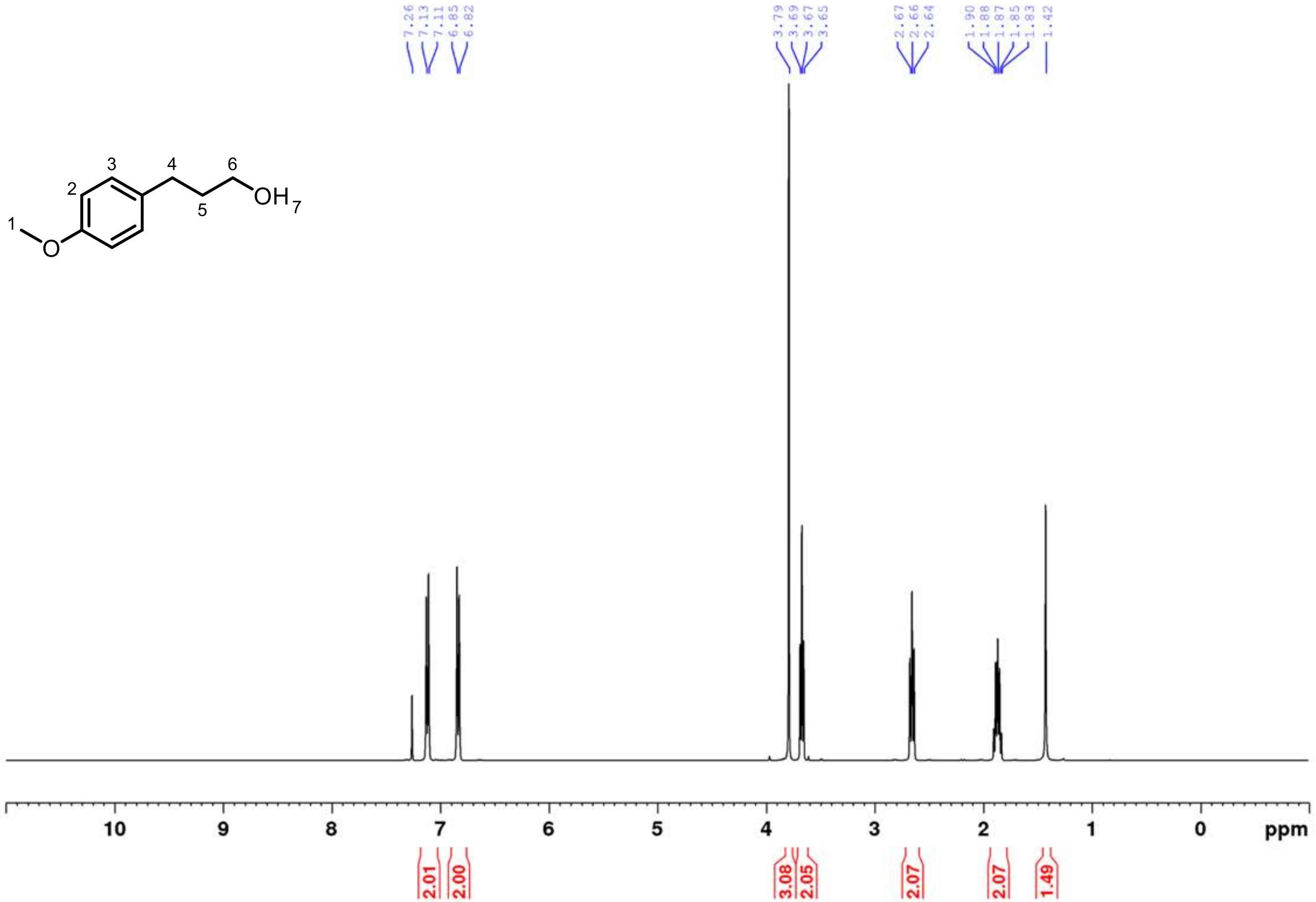

^{13}C NMR (101 MHz, CDCl$_3$) for *3-(4-methoxyphenyl)propan-1-ol* (**1m**)

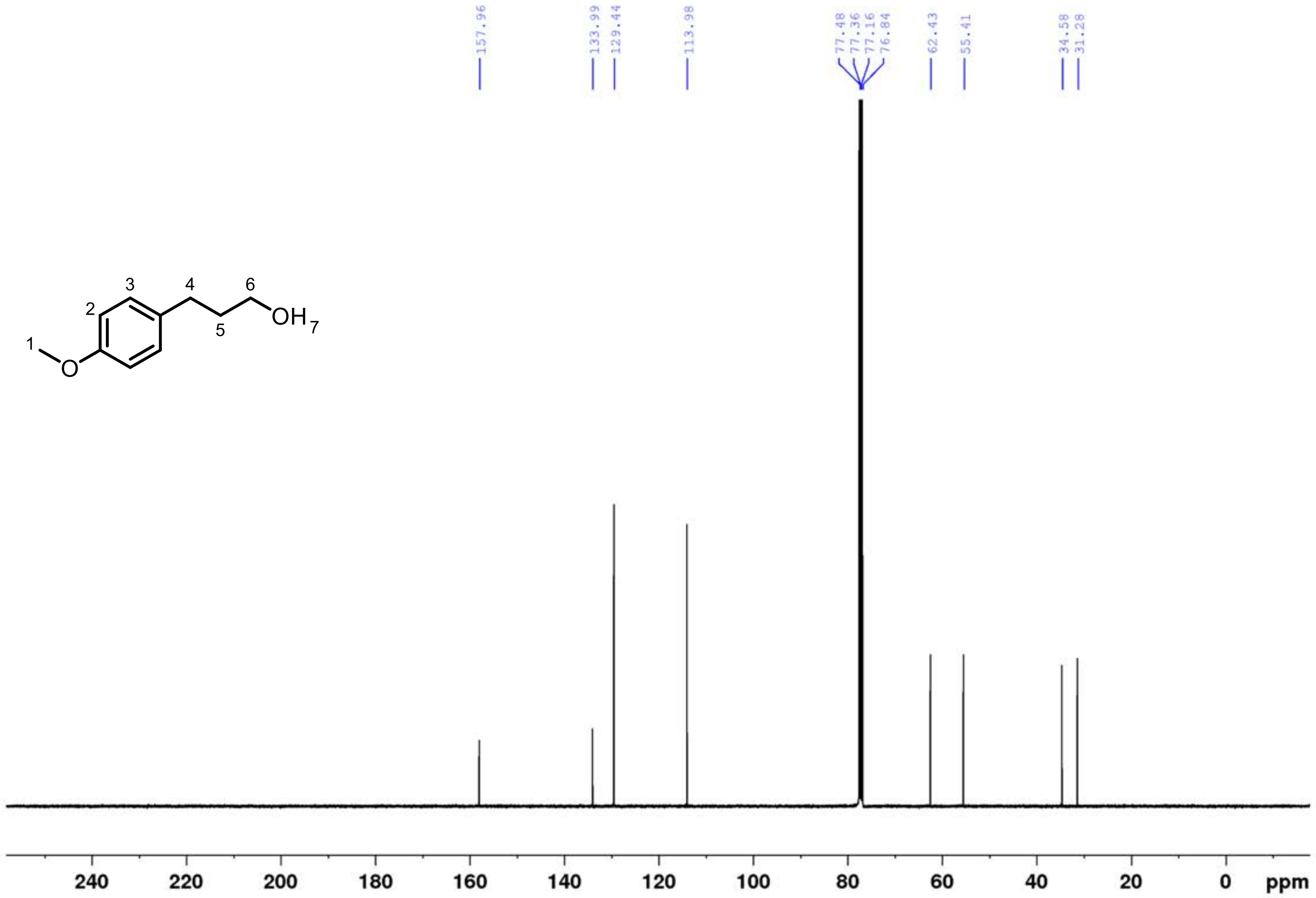

^{1}H NMR (400 MHz, CDCl$_3$) for *3-(4-(trifluoromethyl)phenyl)propan-1-ol* (**1o**)

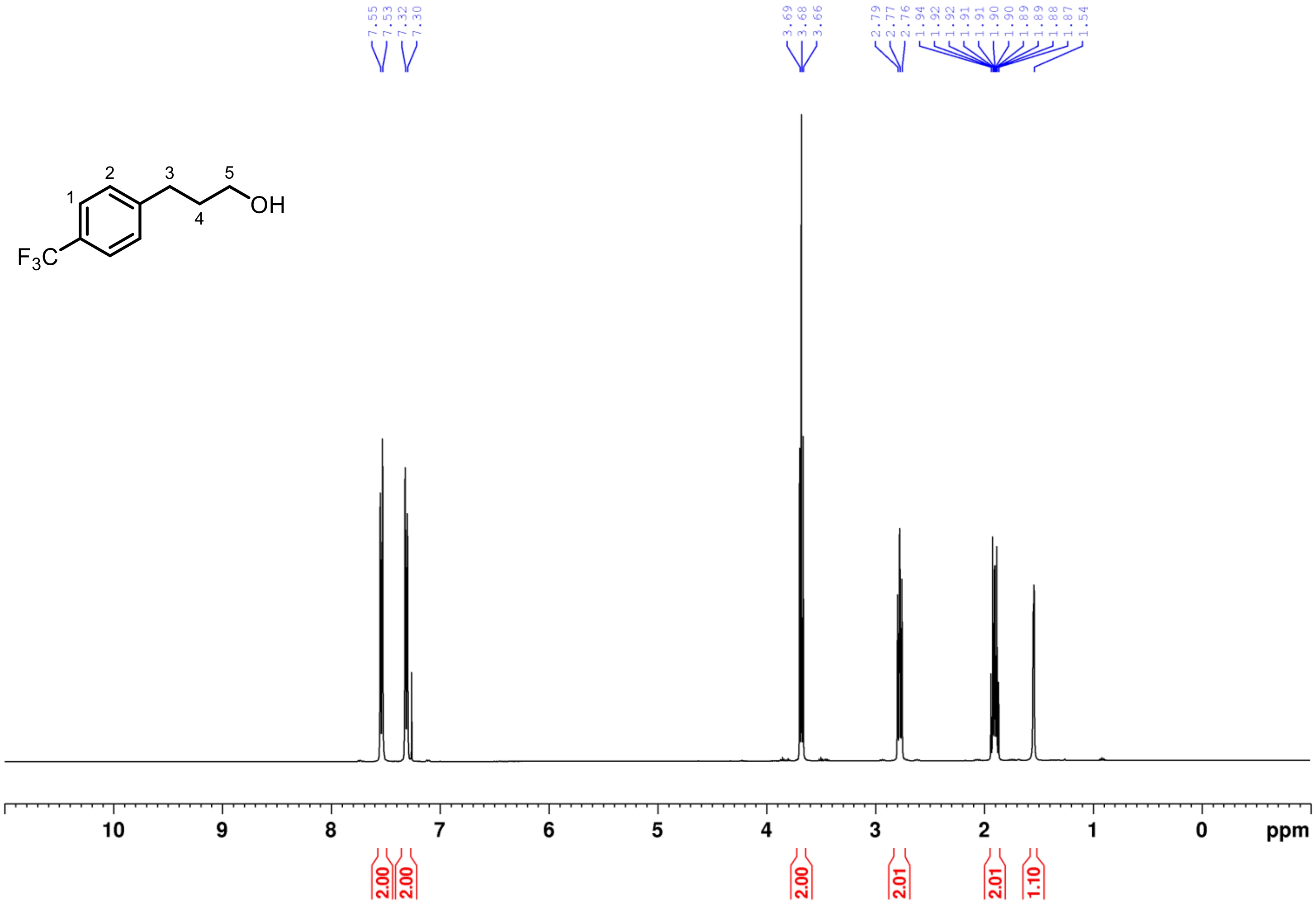

^{13}C NMR (101 MHz, CDCl$_3$) for *3-(4-(trifluoromethyl)phenyl)propan-1-ol* (**1o**)

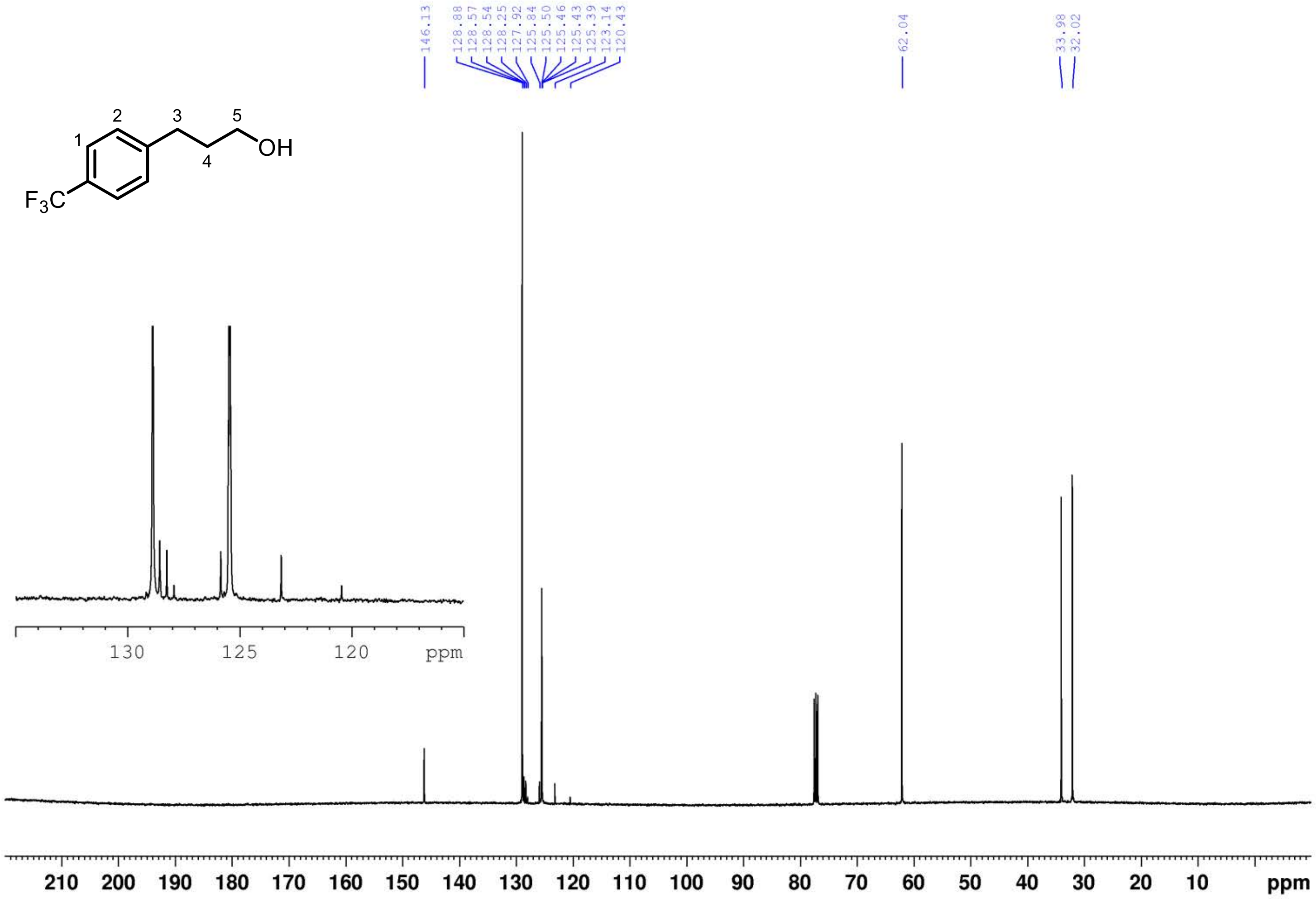

^{19}F NMR (376 MHz, CDCl$_3$) for *3-(4-(trifluoromethyl)phenyl)propan-1-ol* (**1o**)

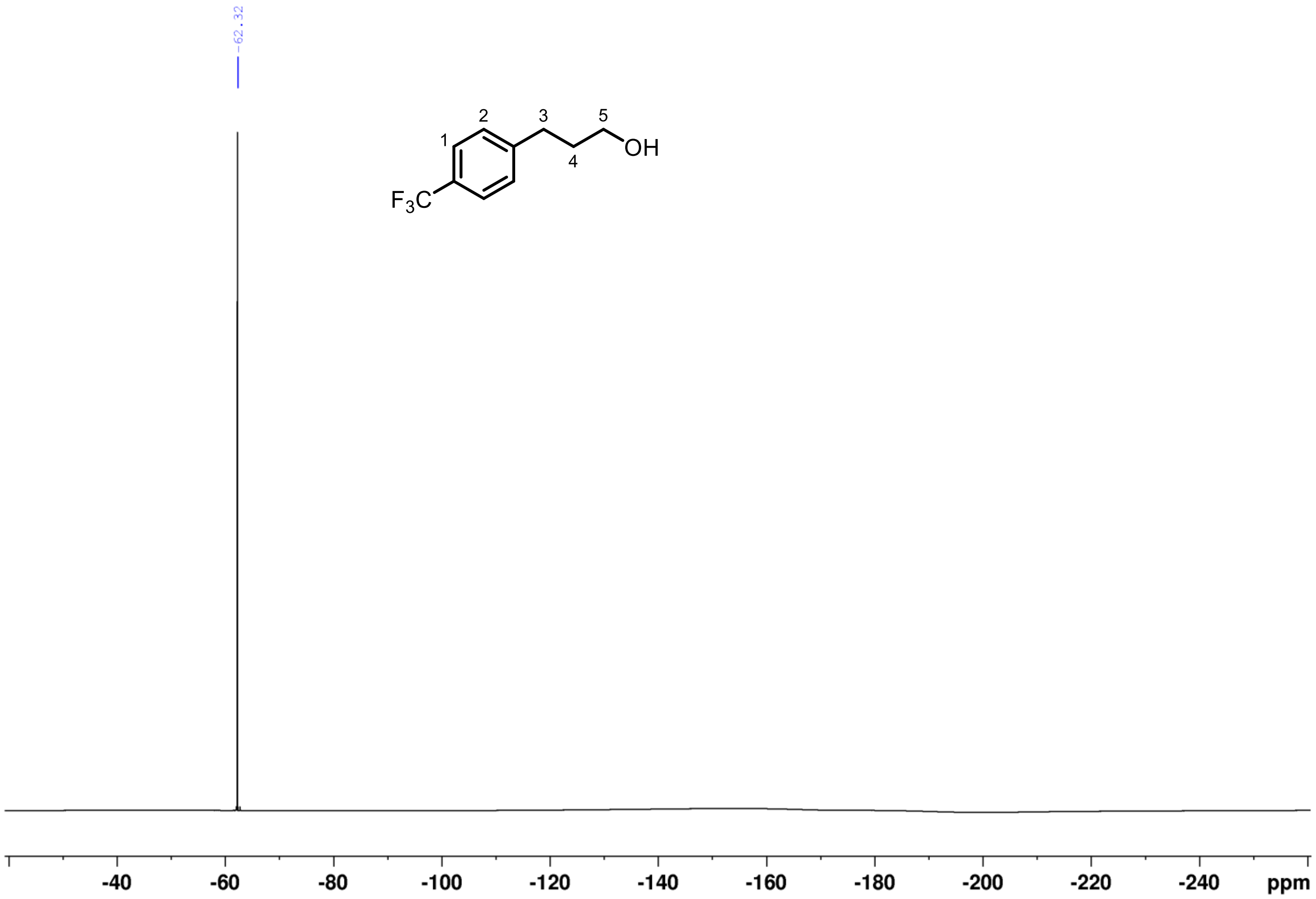

^{1}H NMR (400 MHz, $CDCl_3$) for *2,2-dimethyl-3-phenylpropan-1-ol* (**1p**)

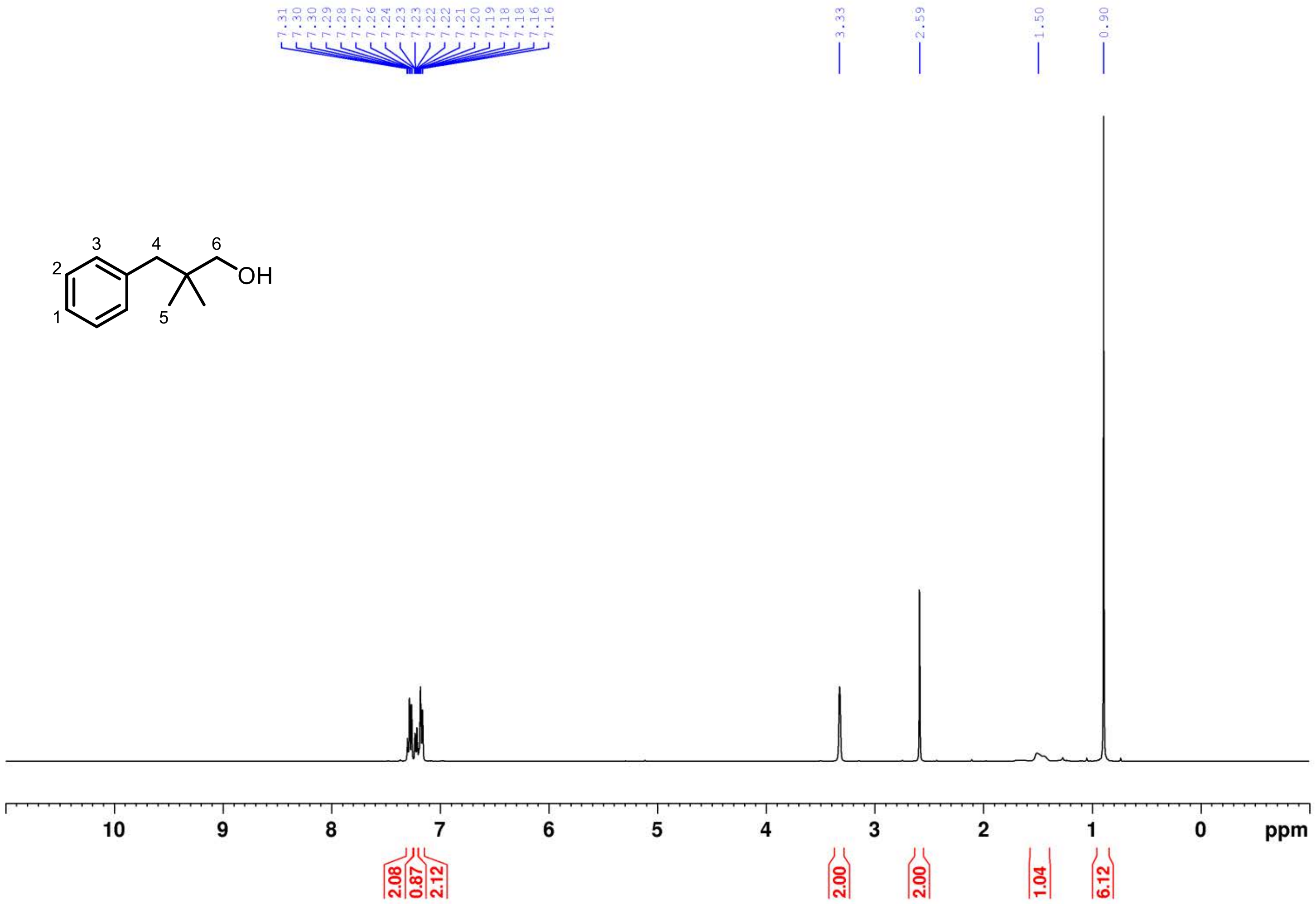

^{13}C NMR (101 MHz, $CDCl_3$) for *2,2-dimethyl-3-phenylpropan-1-ol* (**1p**)

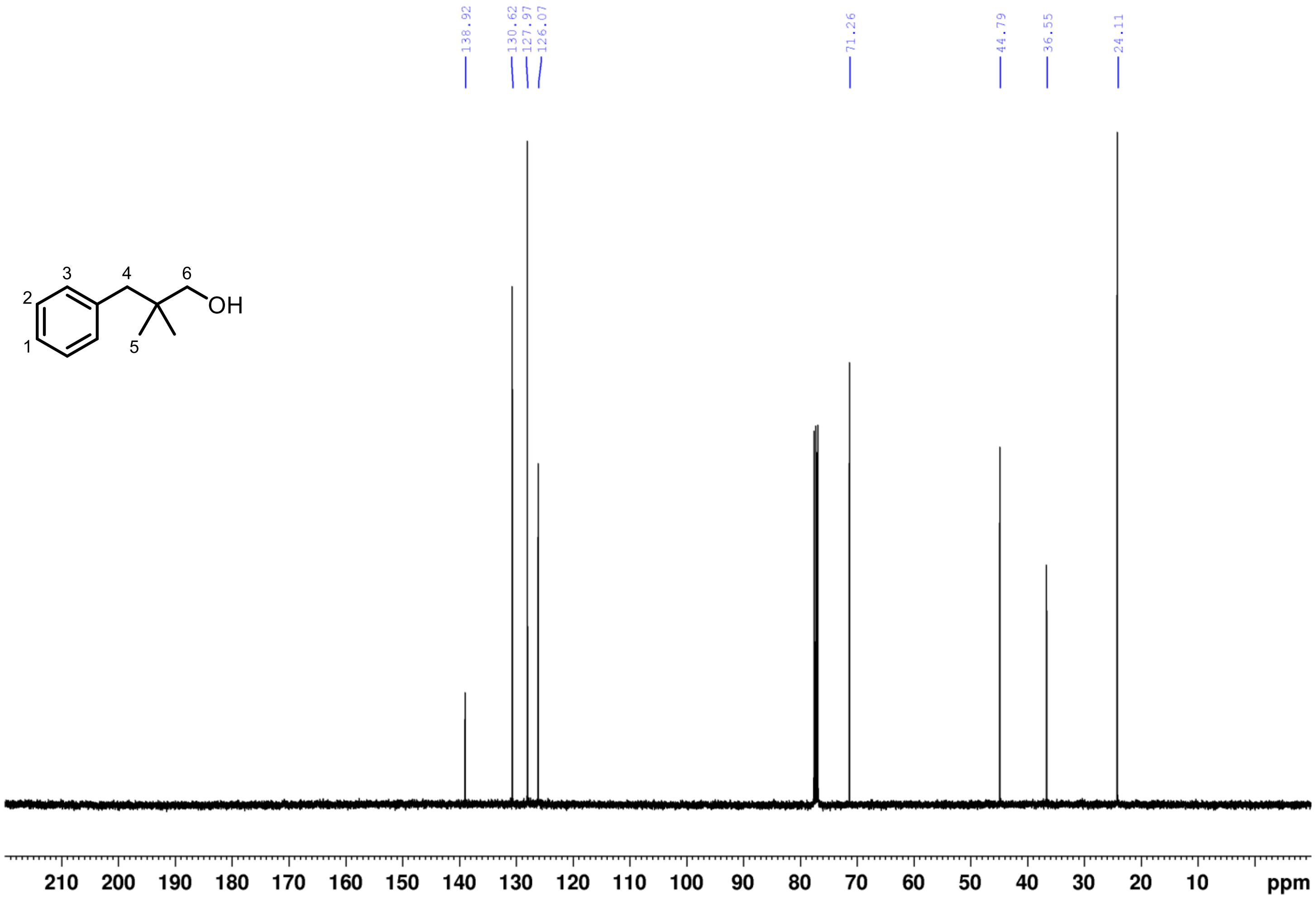

^{1}H NMR (700 MHz, $CDCl_3$) for *(1-benzylcyclopentyl)methanol* (**1q**)

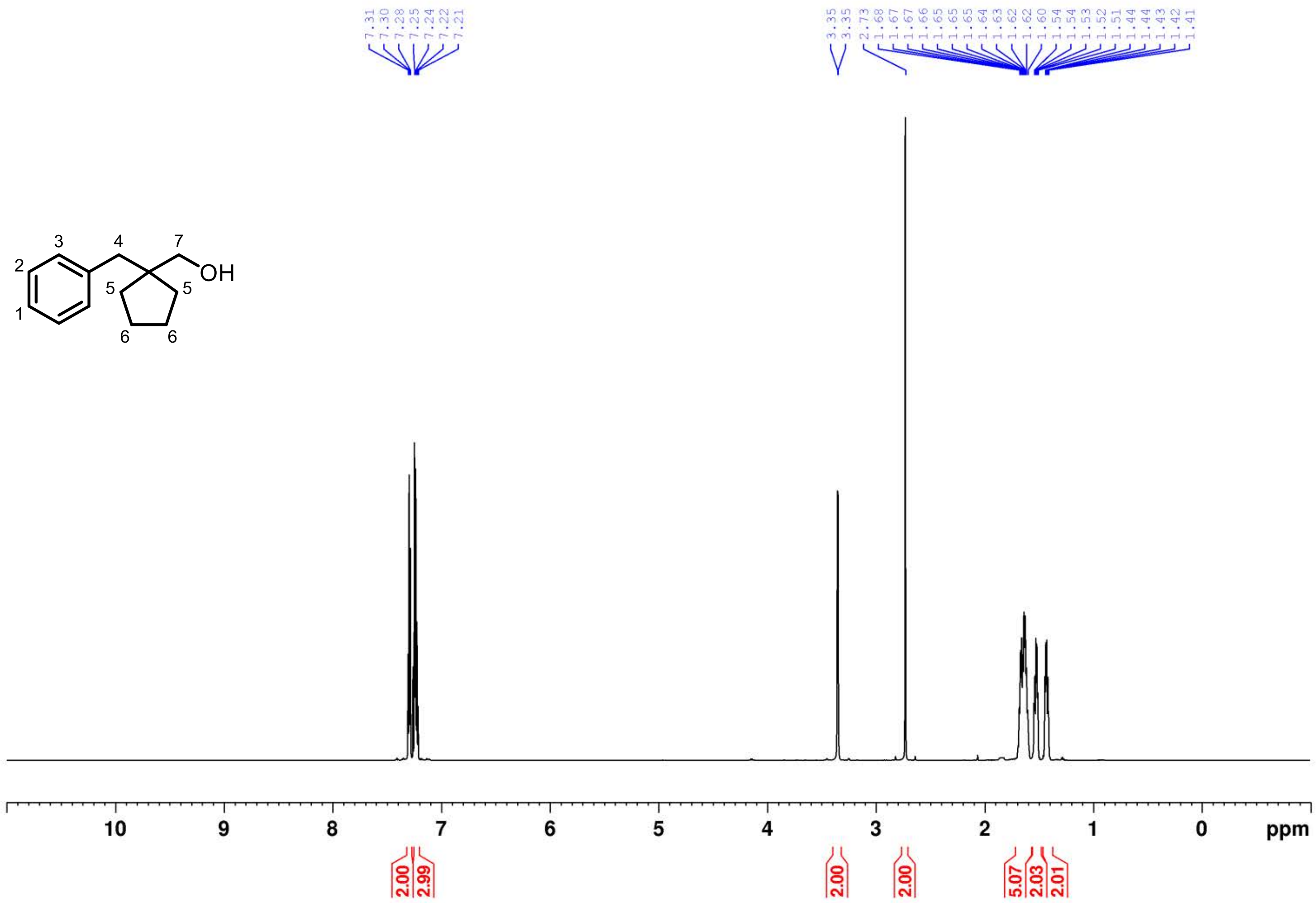

^{13}C NMR (176 MHz, $CDCl_3$) for *(1-benzylcyclopentyl)methanol* (**1q**)

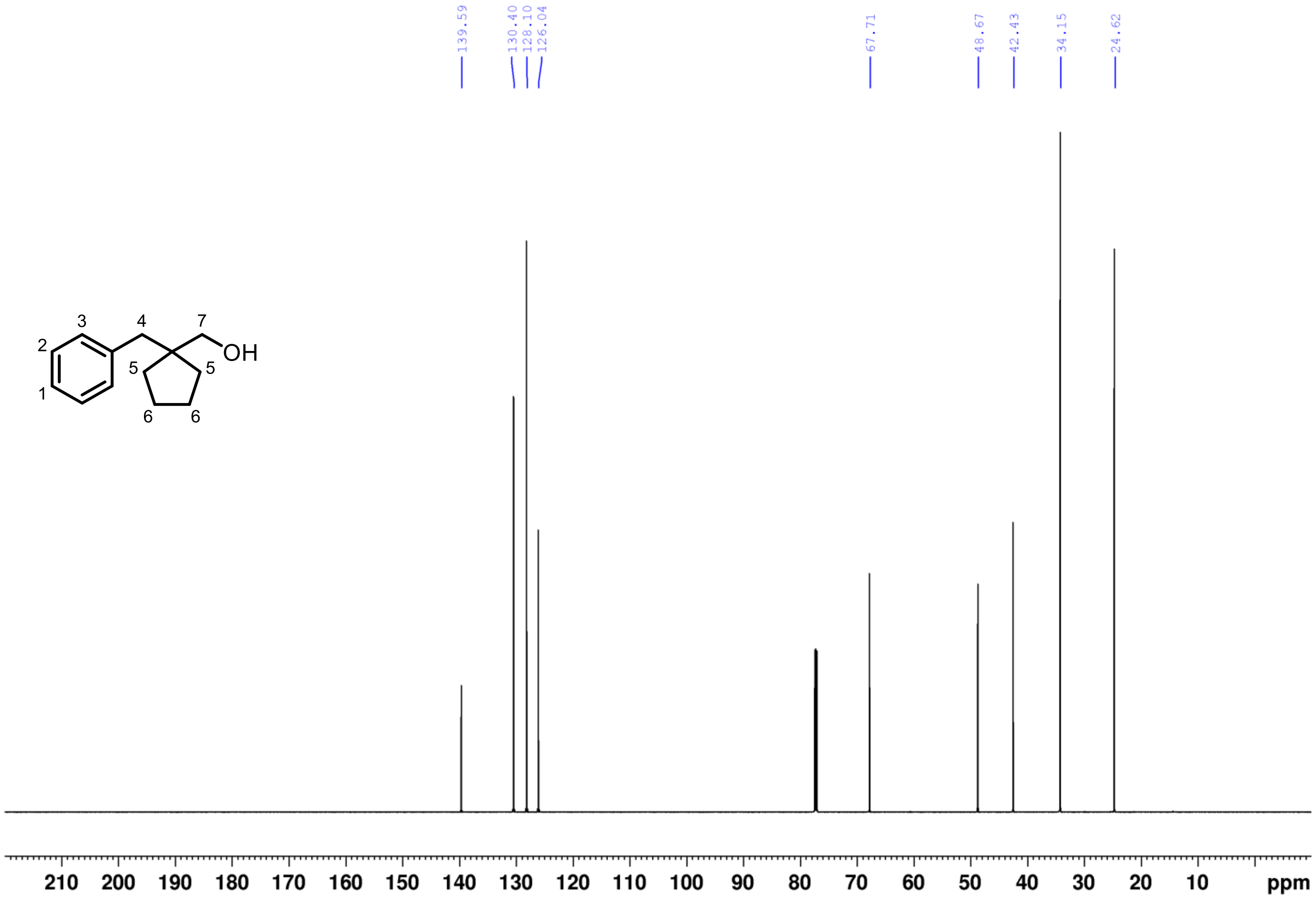

^{1}H NMR (400 MHz, CDCl$_3$) for *1-phenethylcyclobutan-1-ol* (**1s**)

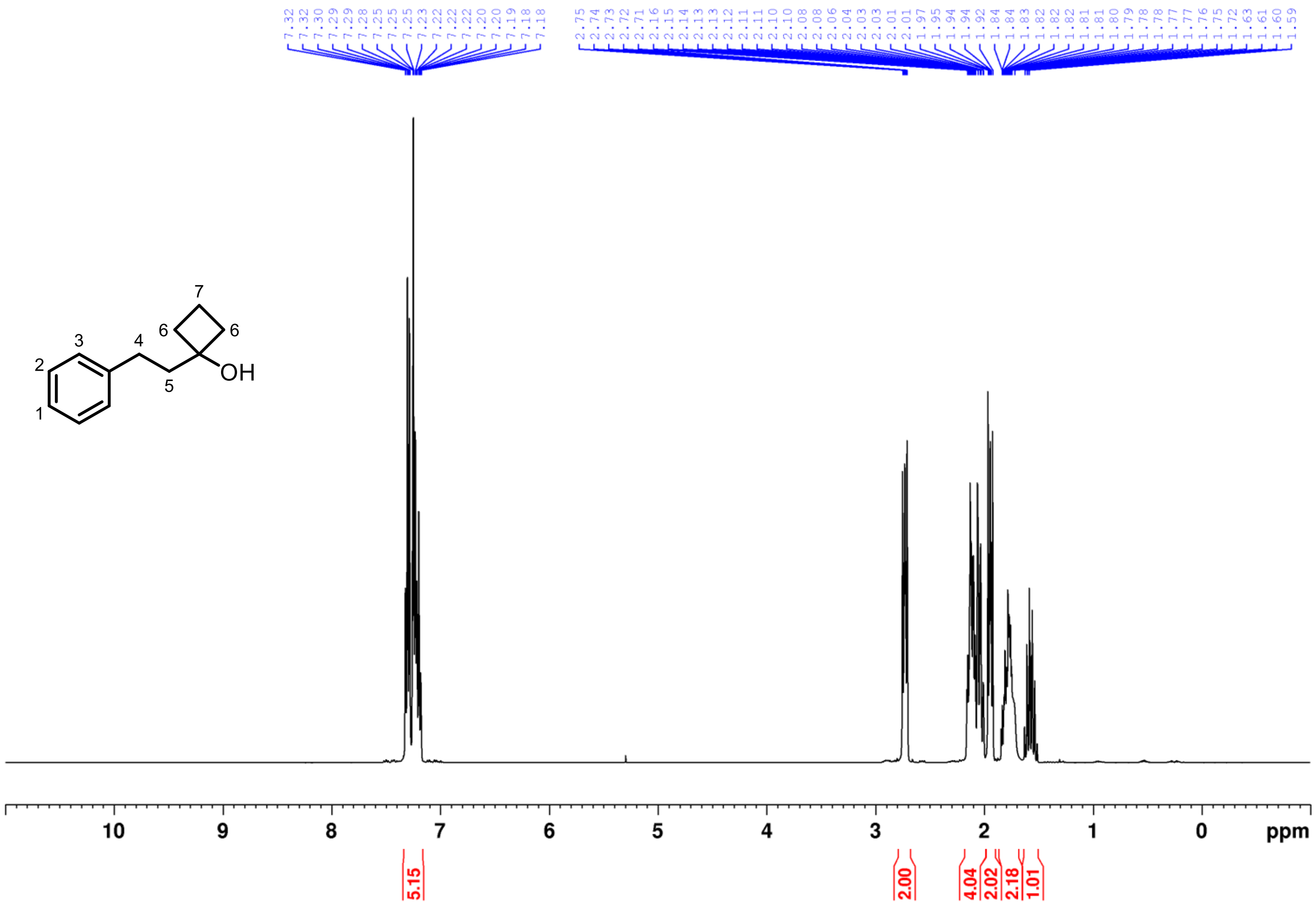

^{13}C NMR (101 MHz, $CDCl_3$) for *1-phenethylcyclobutan-1-ol* (**1s**)

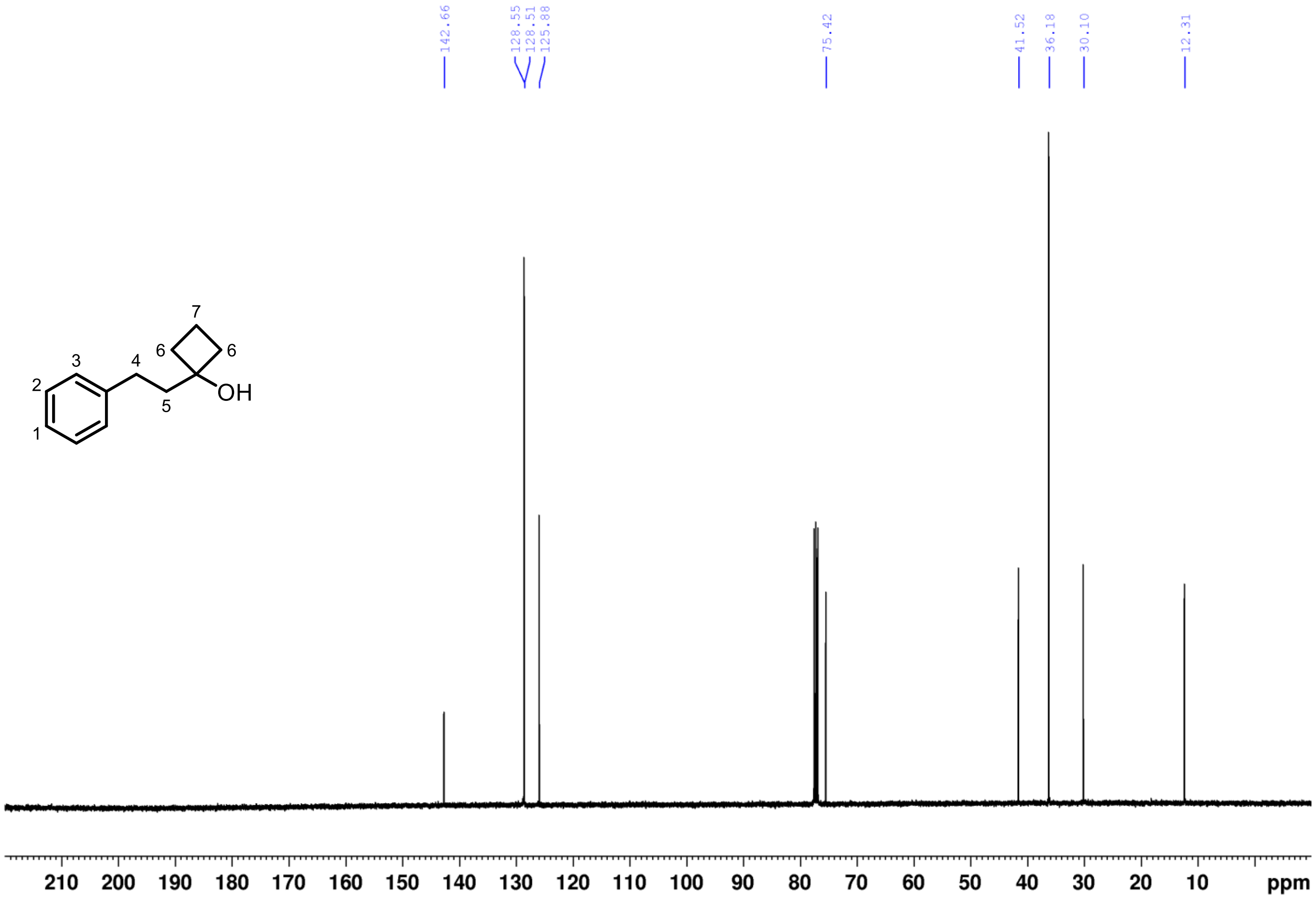

^{1}H NMR (400 MHz, CDCl$_3$) for *ethyl (E)-2-(thiophen-3-yl)acrylate*

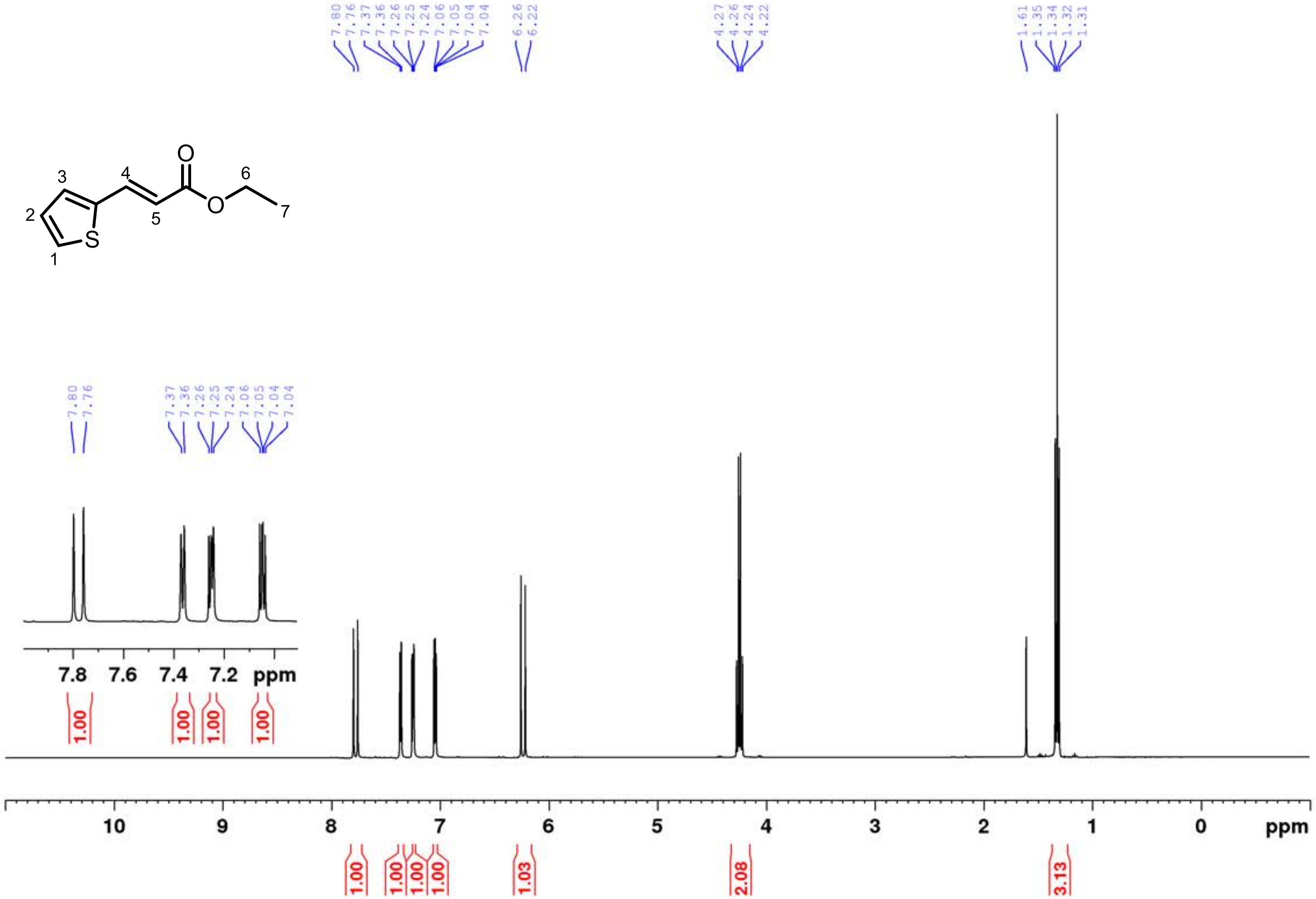

^{13}C NMR (101 MHz, CDCl$_3$) for *ethyl (E)-2-(thiophen-3-yl)acrylate*

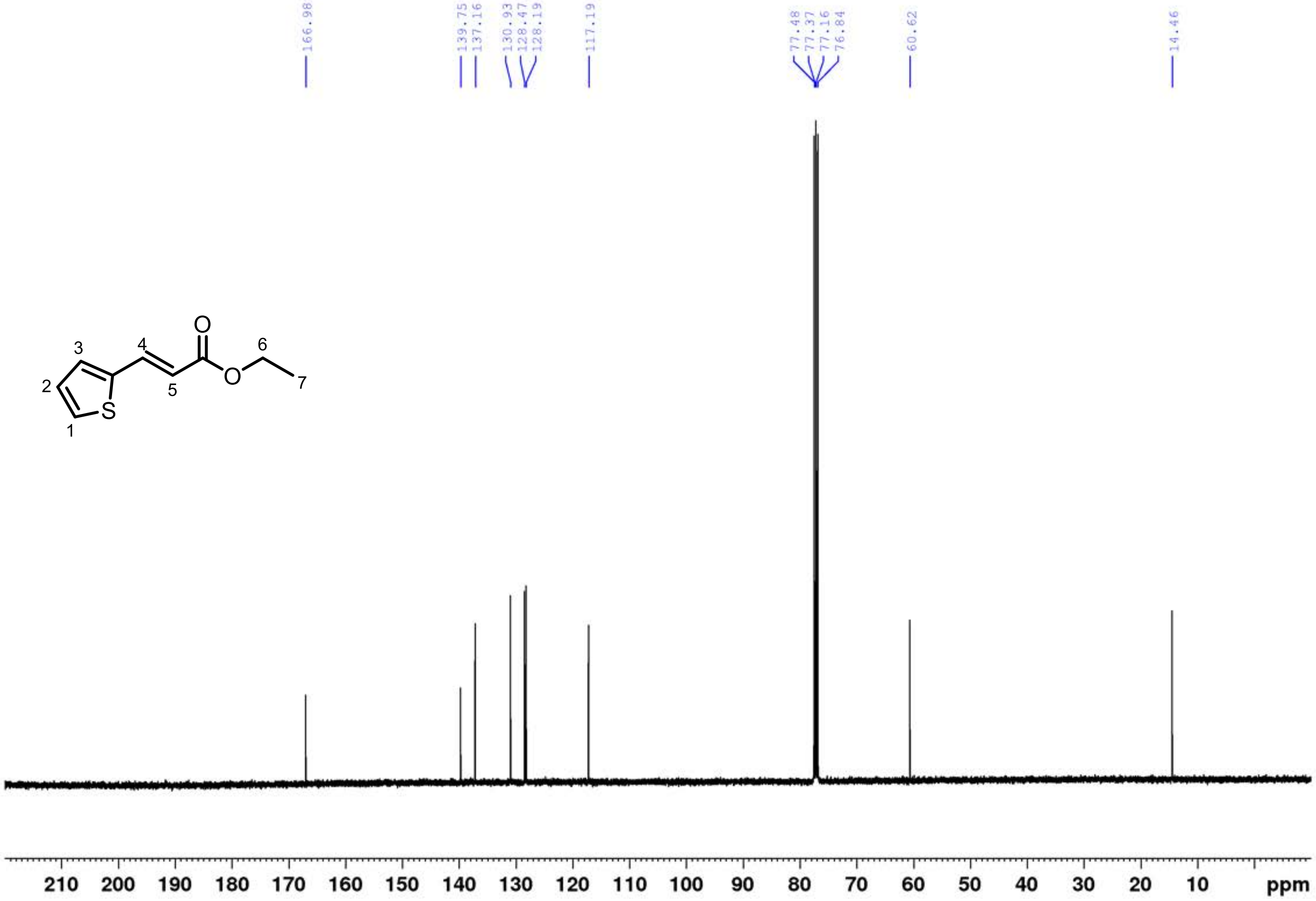

¹H NMR (400 MHz, CDCl$_3$) for *3-(thiophen-2-yl)propan-1-ol* (**1t**)

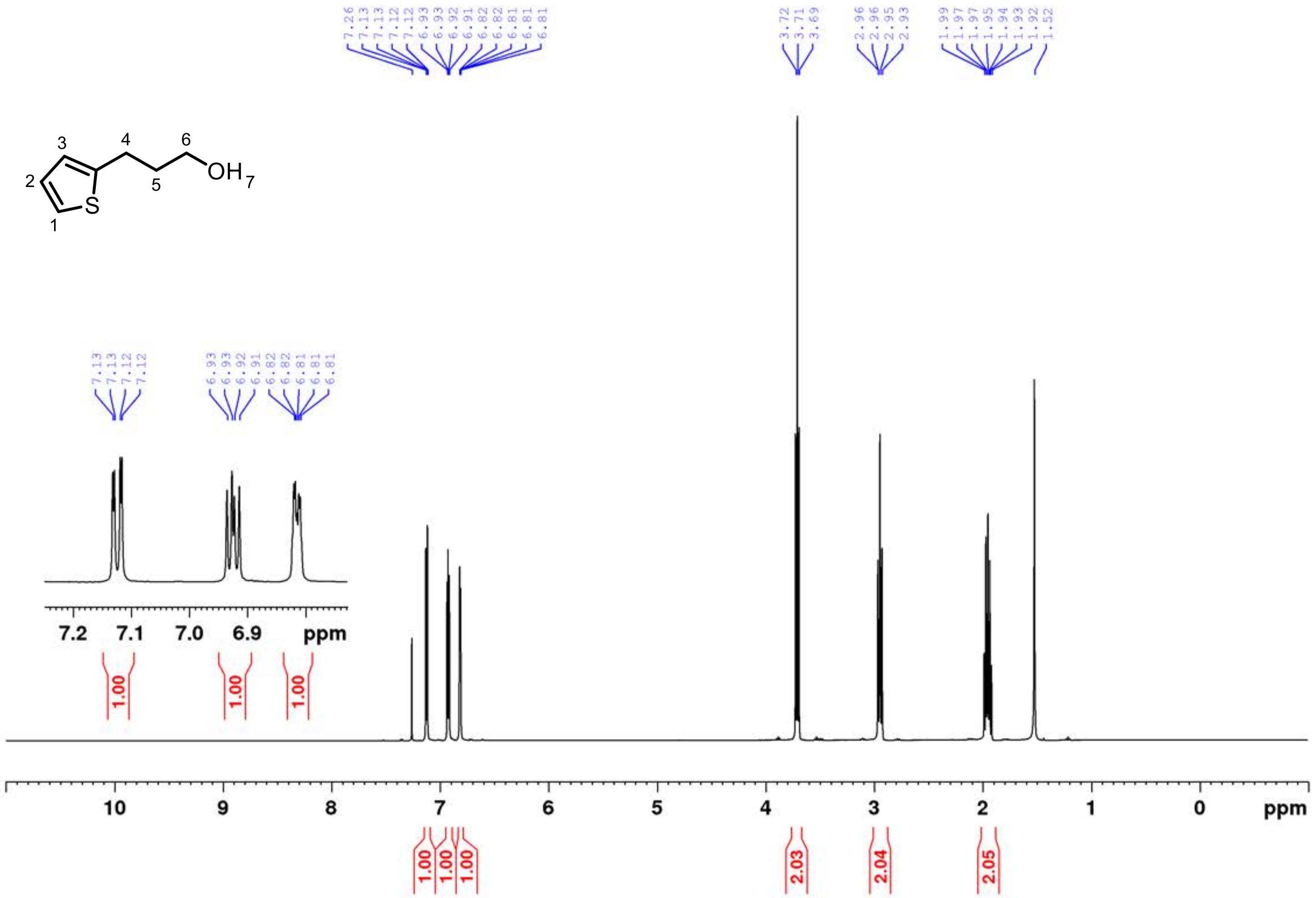

^{13}C NMR (101 MHz, $CDCl_3$) for *3-(thiophen-2-yl)propan-1-ol* (**1t**)

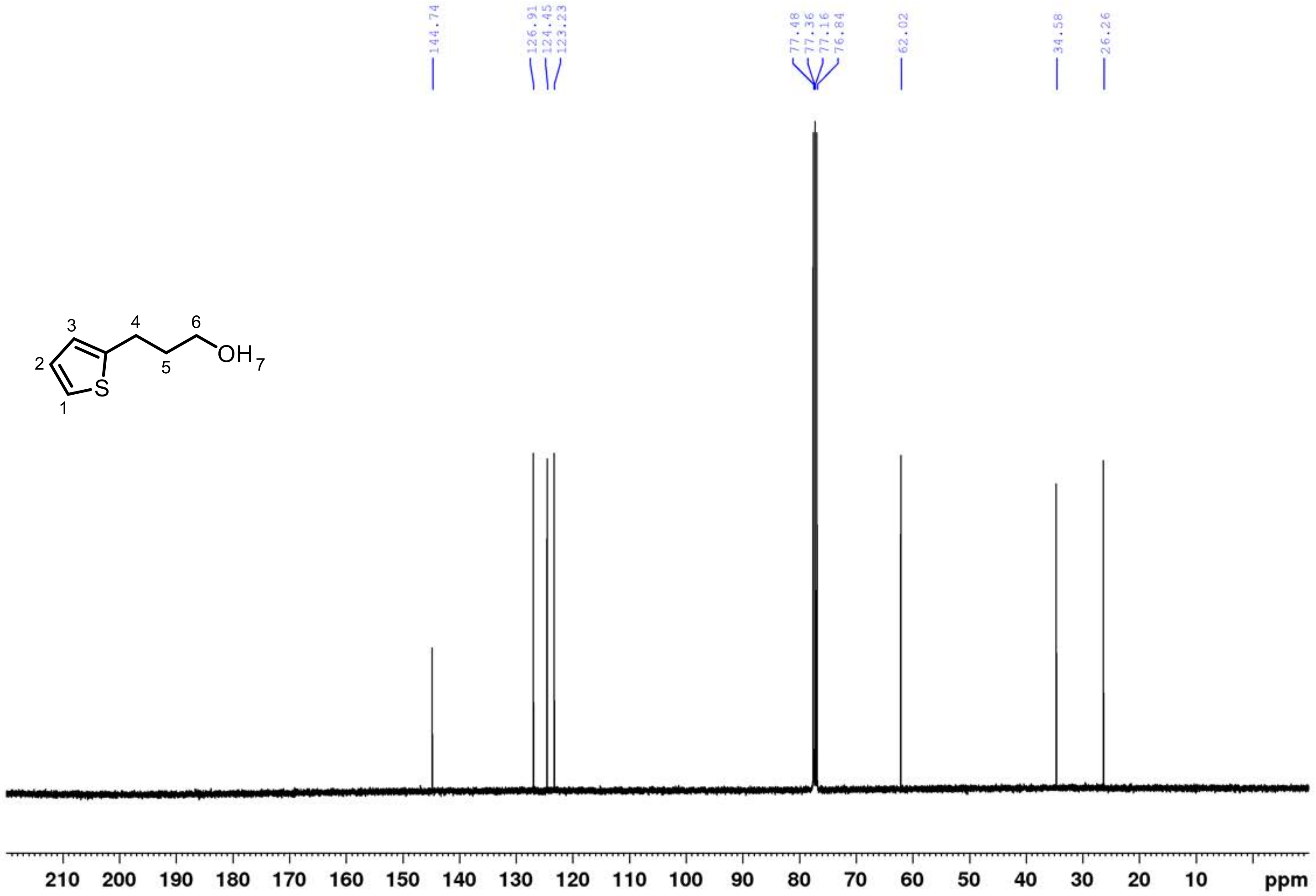

^{1}H NMR (700 MHz, CDCl$_3$) for *ethyl (E)-3-(benzo[b]thiophen-5-yl)acrylate*

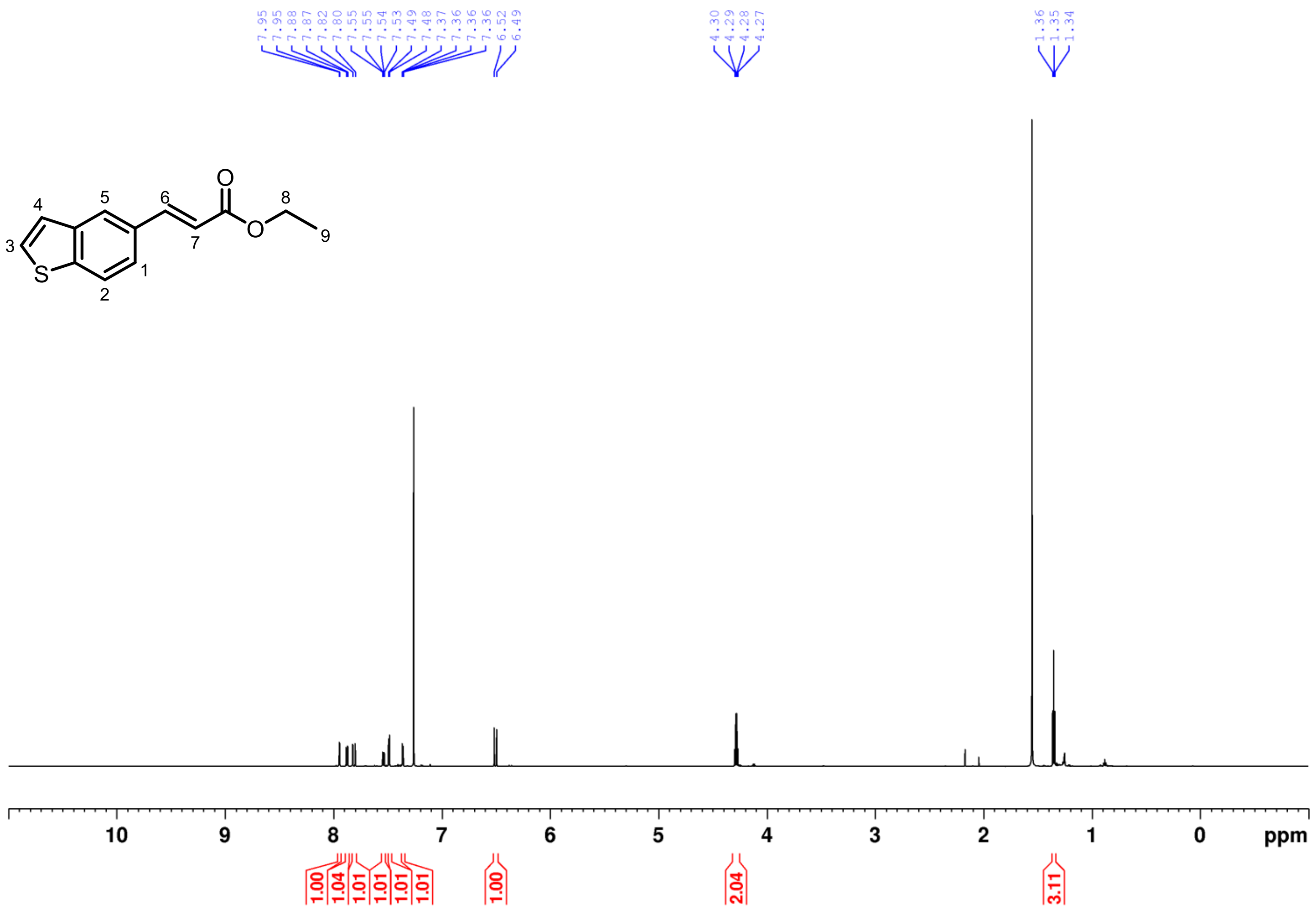

^{13}C NMR (176 MHz, $CDCl_3$) for *ethyl (E)-3-(benzo[b]thiophen-5-yl)acrylate*

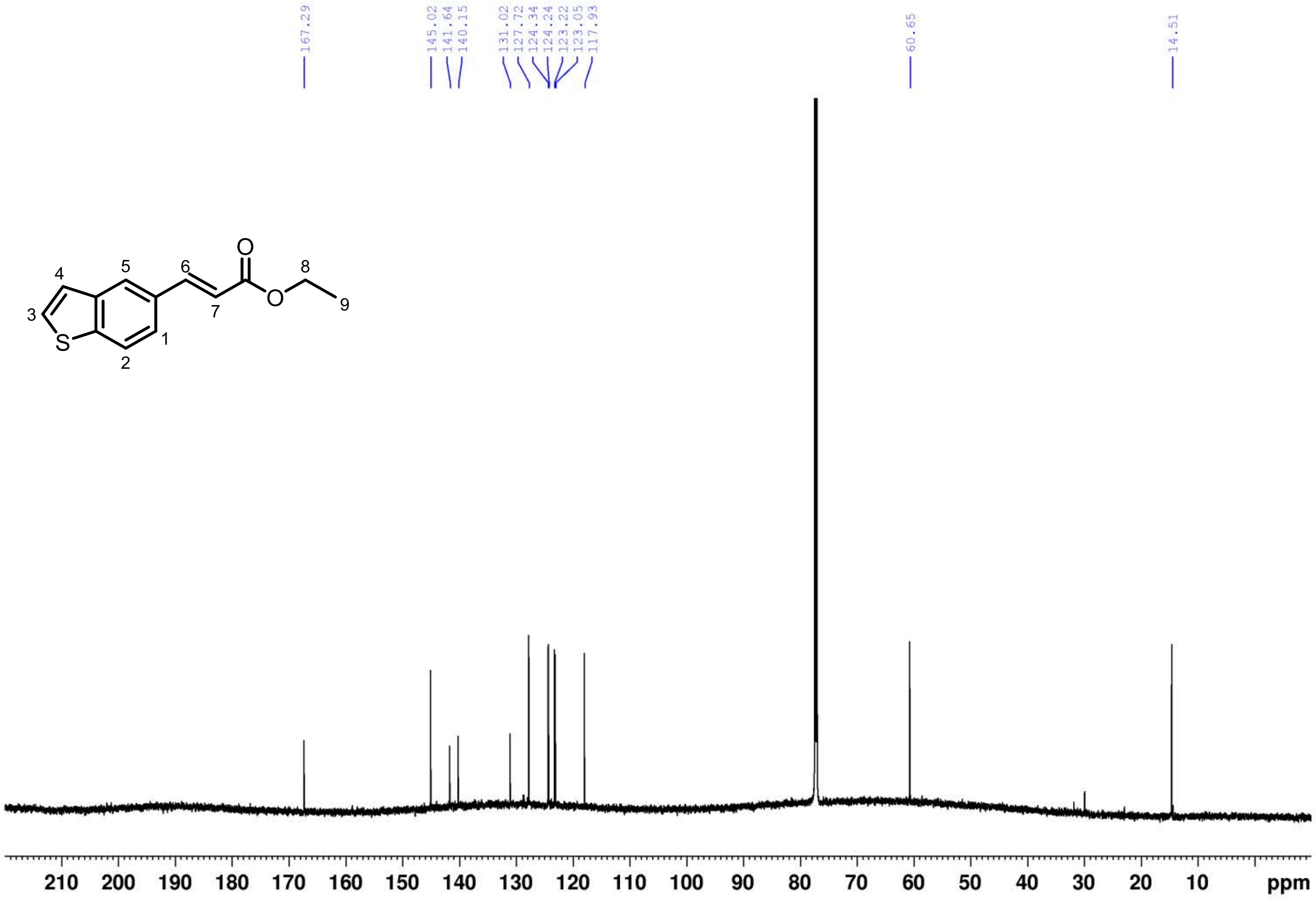

^{1}H NMR (700 MHz, $CDCl_3$) for *3-(benzo[b]thiophen-5-yl)propan-1-ol* (**1u**)

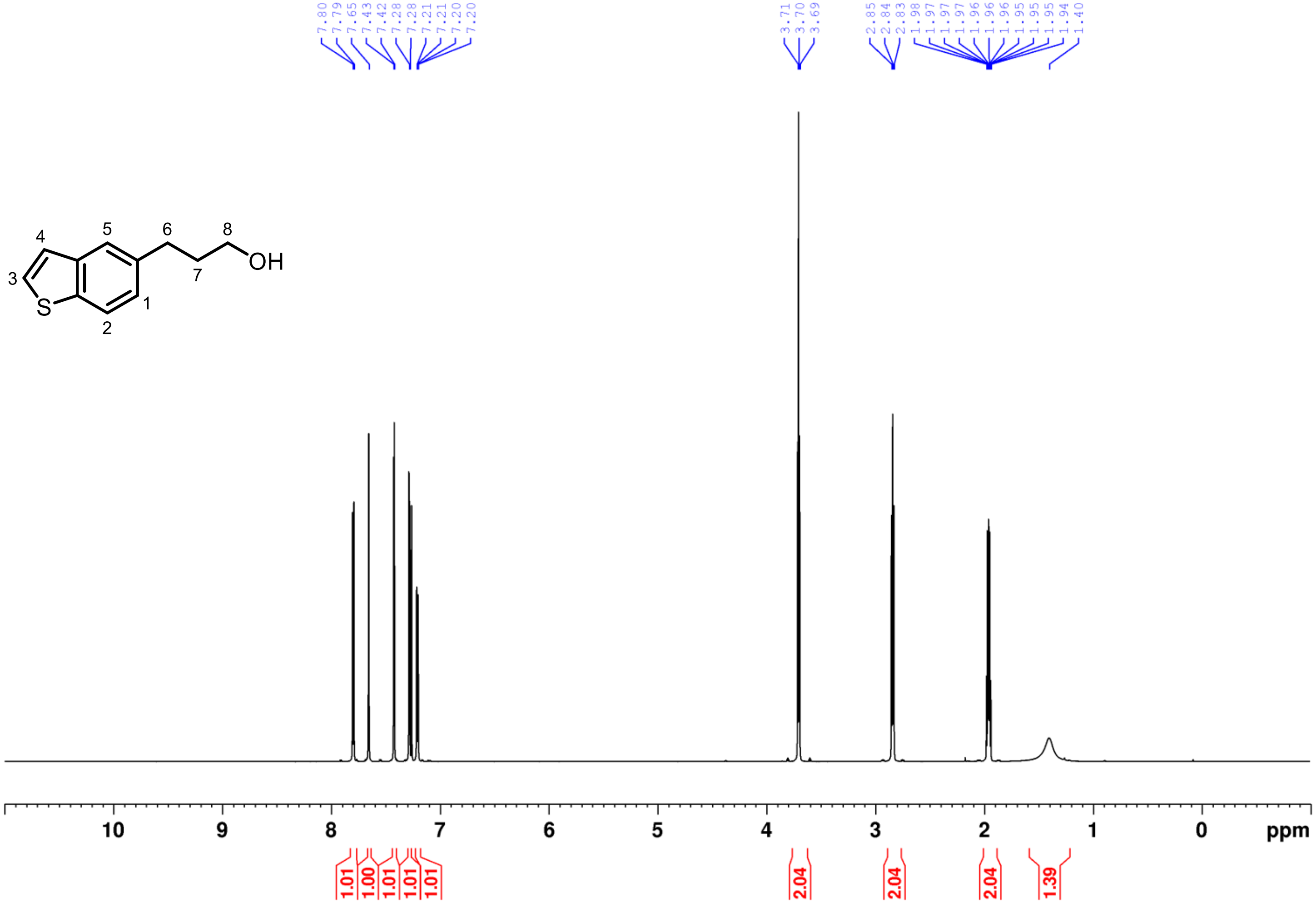

^{13}C NMR (176 MHz, CDCl₃) for *3-(benzo[b]thiophen-5-yl)propan-1-ol* (**1u**)

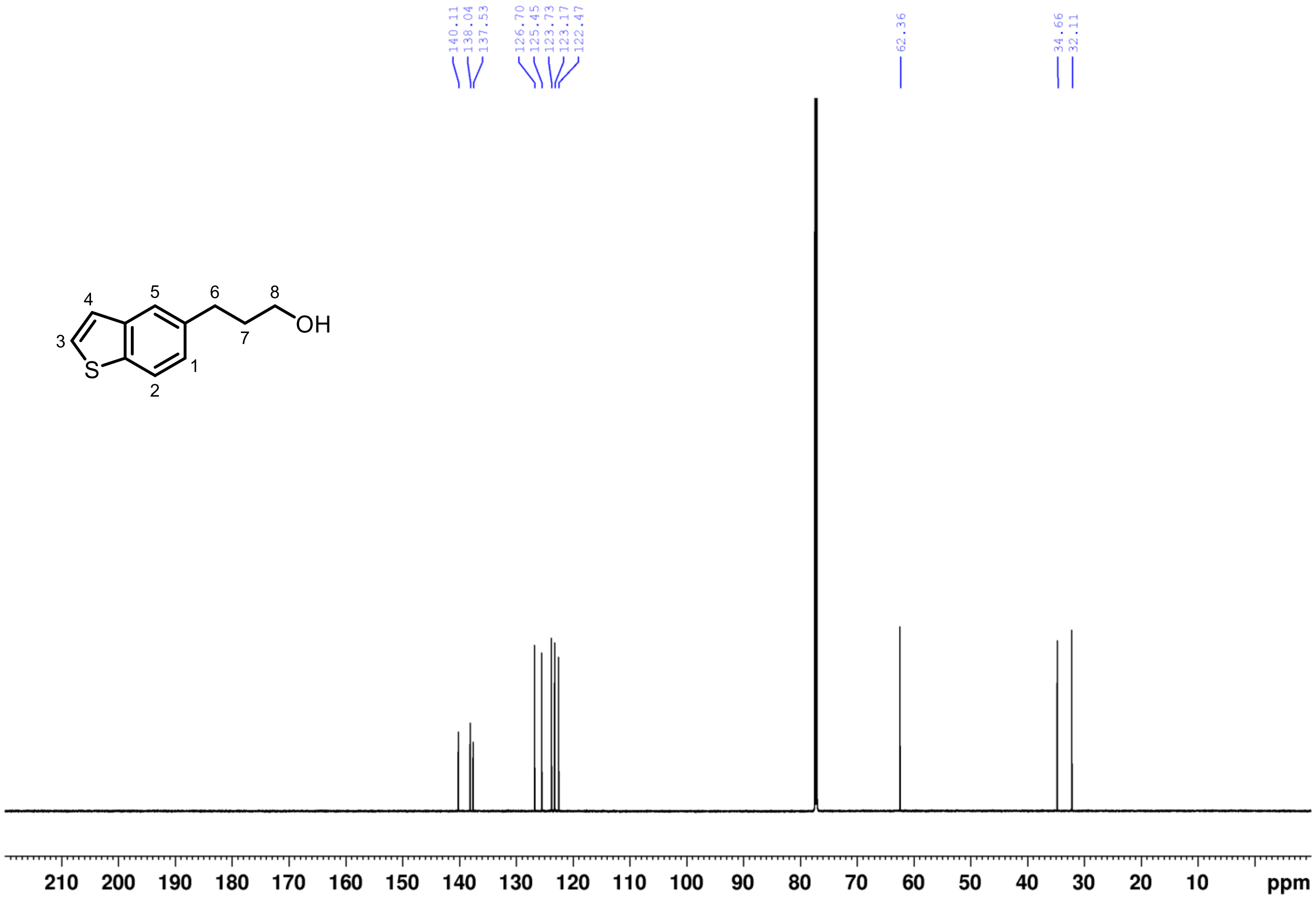

^{1}H NMR (400 MHz, $CDCl_3$) for *3-(benzo[b]thiophen-2-yl)propan-1-ol* (**1v**)

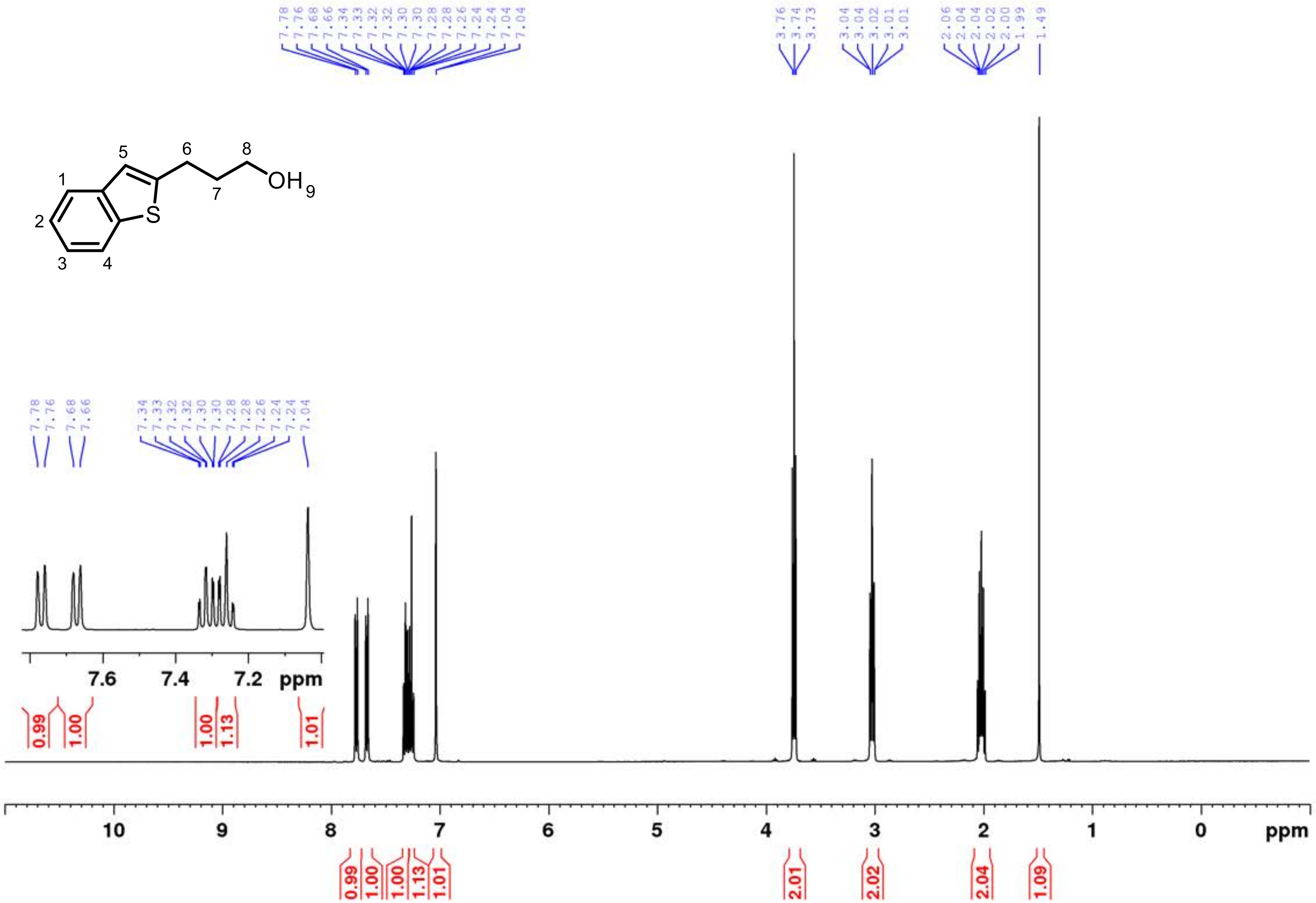

^{13}C NMR (101 MHz, $CDCl_3$) for *3-(benzo[b]thiophen-2-yl)propan-1-ol* (**1v**)

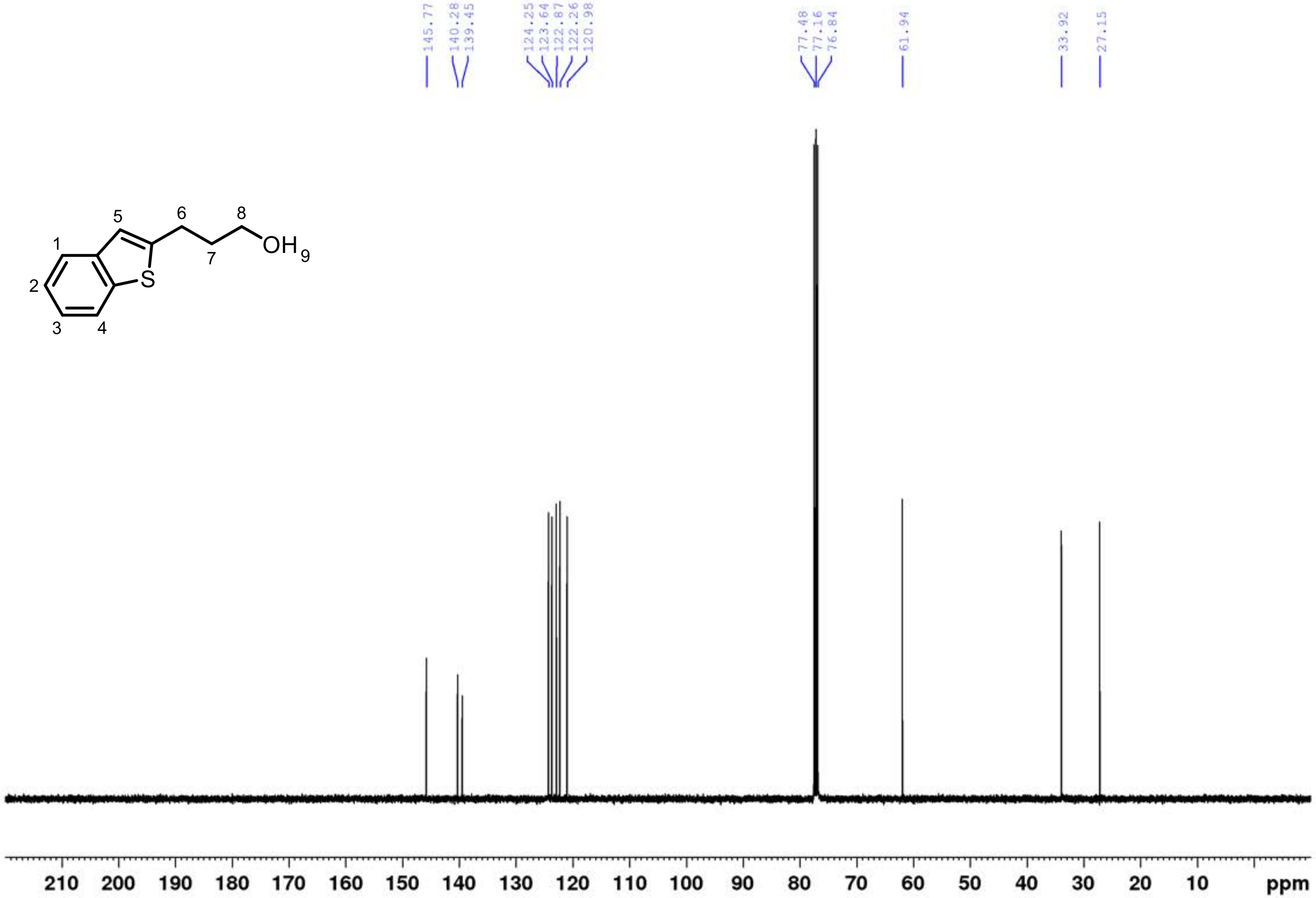

¹H NMR (400 MHz, $CDCl_3$) for *3-(1-(phenylsulfonyl)-1H-pyrrol-3-yl)prop-2-yn-1-ol*

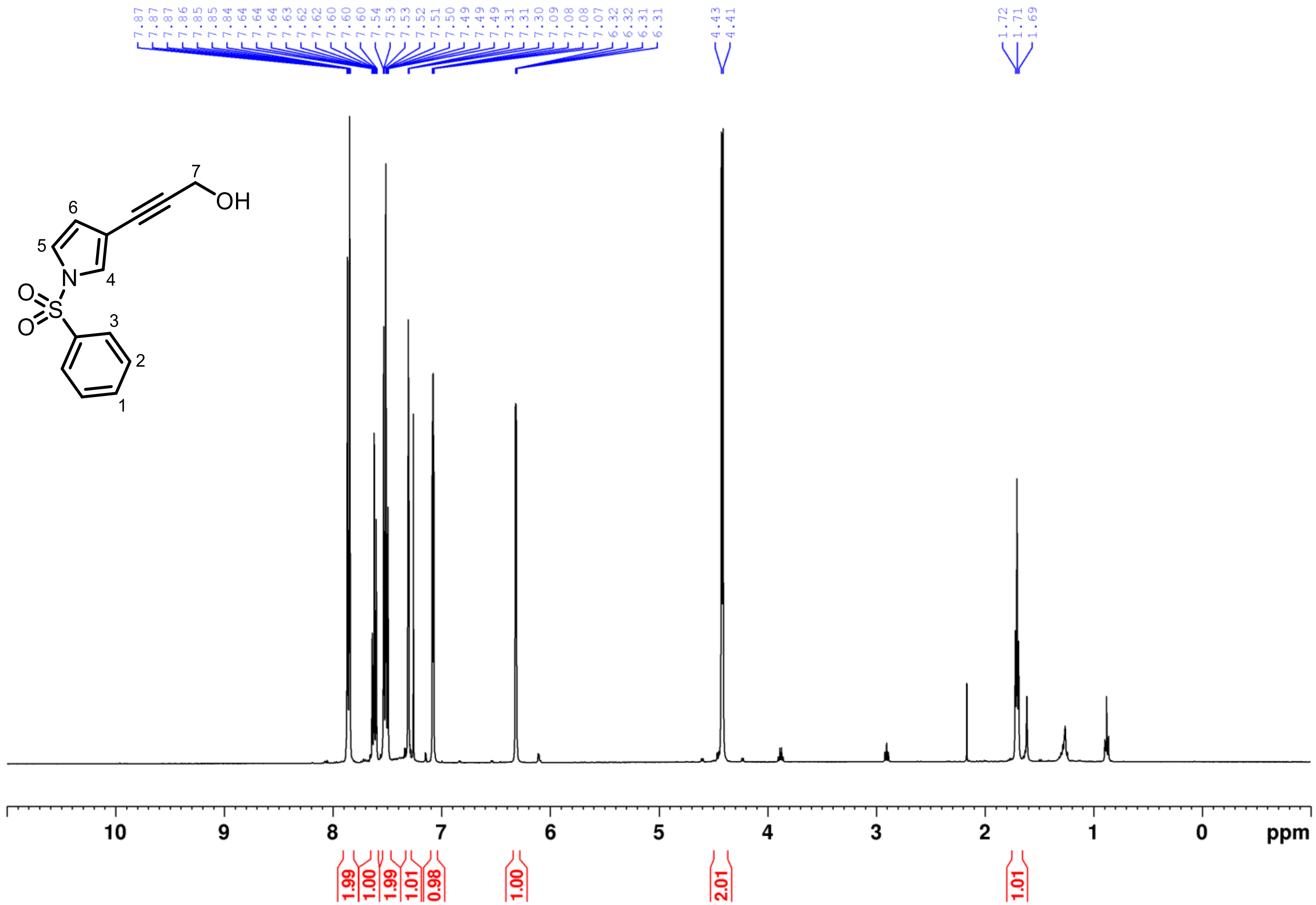

^{13}C NMR (101 MHz, CDCl$_3$) for *3-(1-(phenylsulfonyl)-1H-pyrrol-3-yl)prop-2-yn-1-ol*

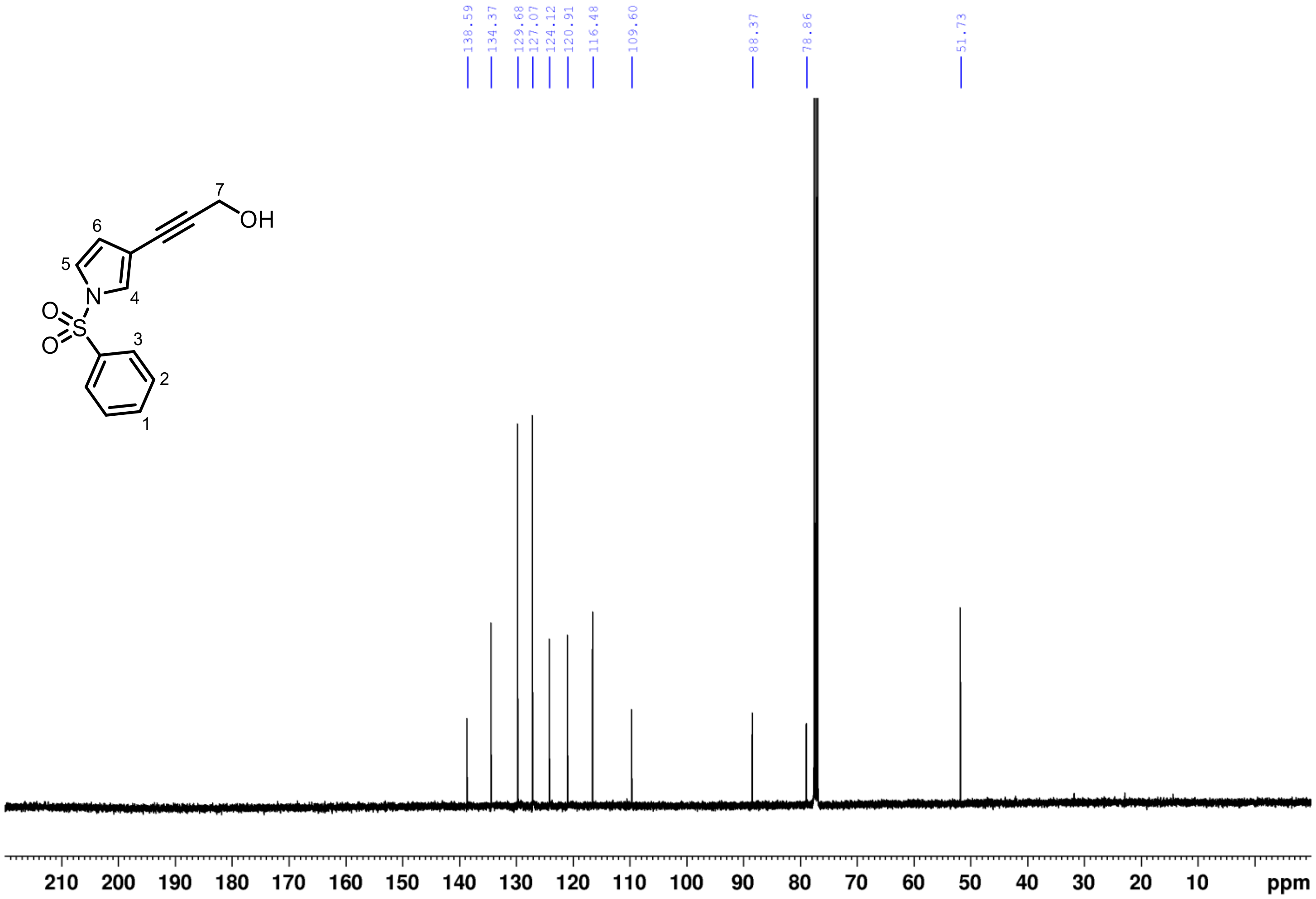

^{1}H NMR (400 MHz, CDCl$_3$) for *3-(1-(phenylsulfonyl)-1H-pyrrol-3-yl)propan-1-ol* (**1w**)

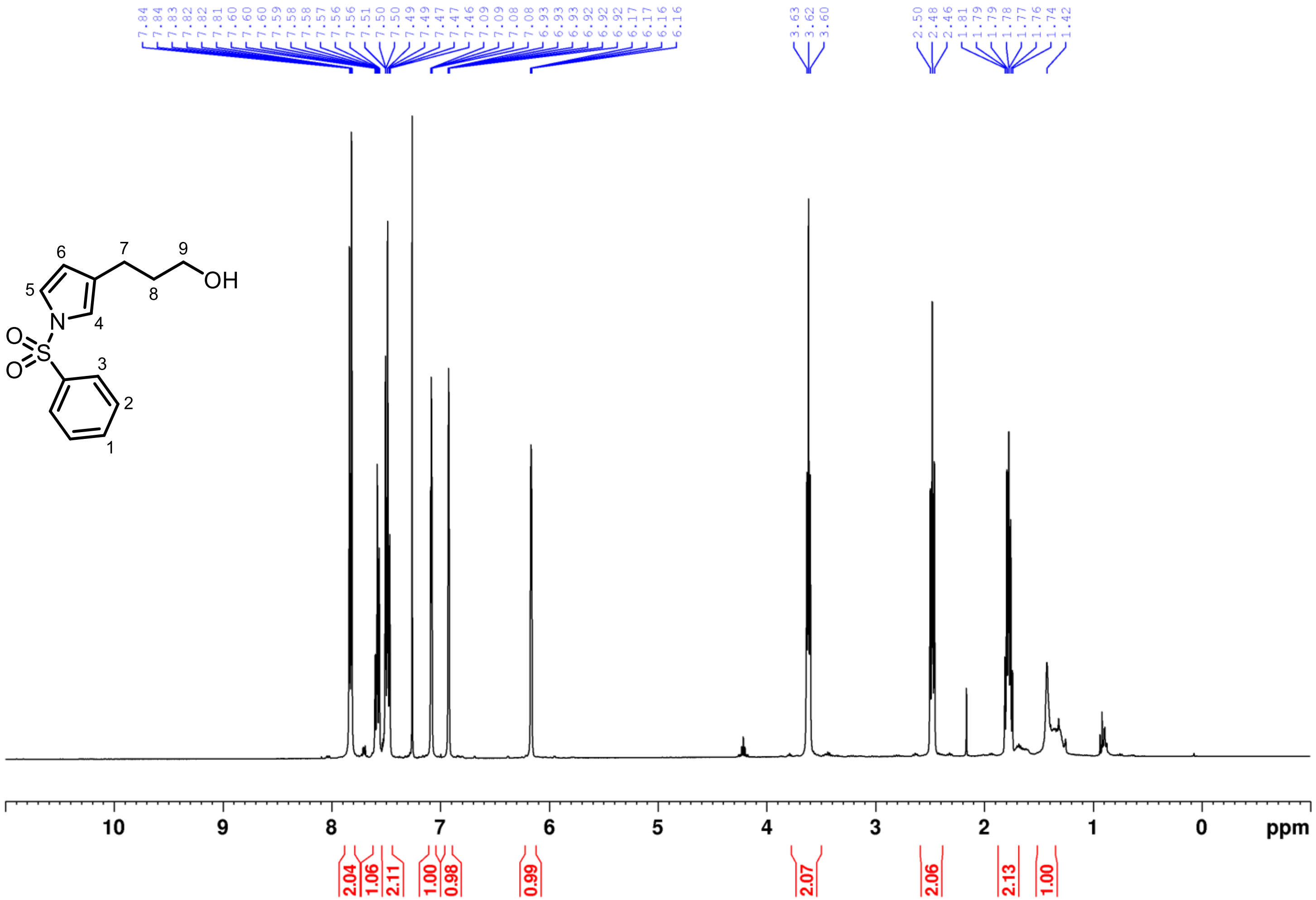

^{13}C NMR (101 MHz, $CDCl_3$) for *3-(1-(phenylsulfonyl)-1H-pyrrol-3-yl)propan-1-ol* (**1w**)

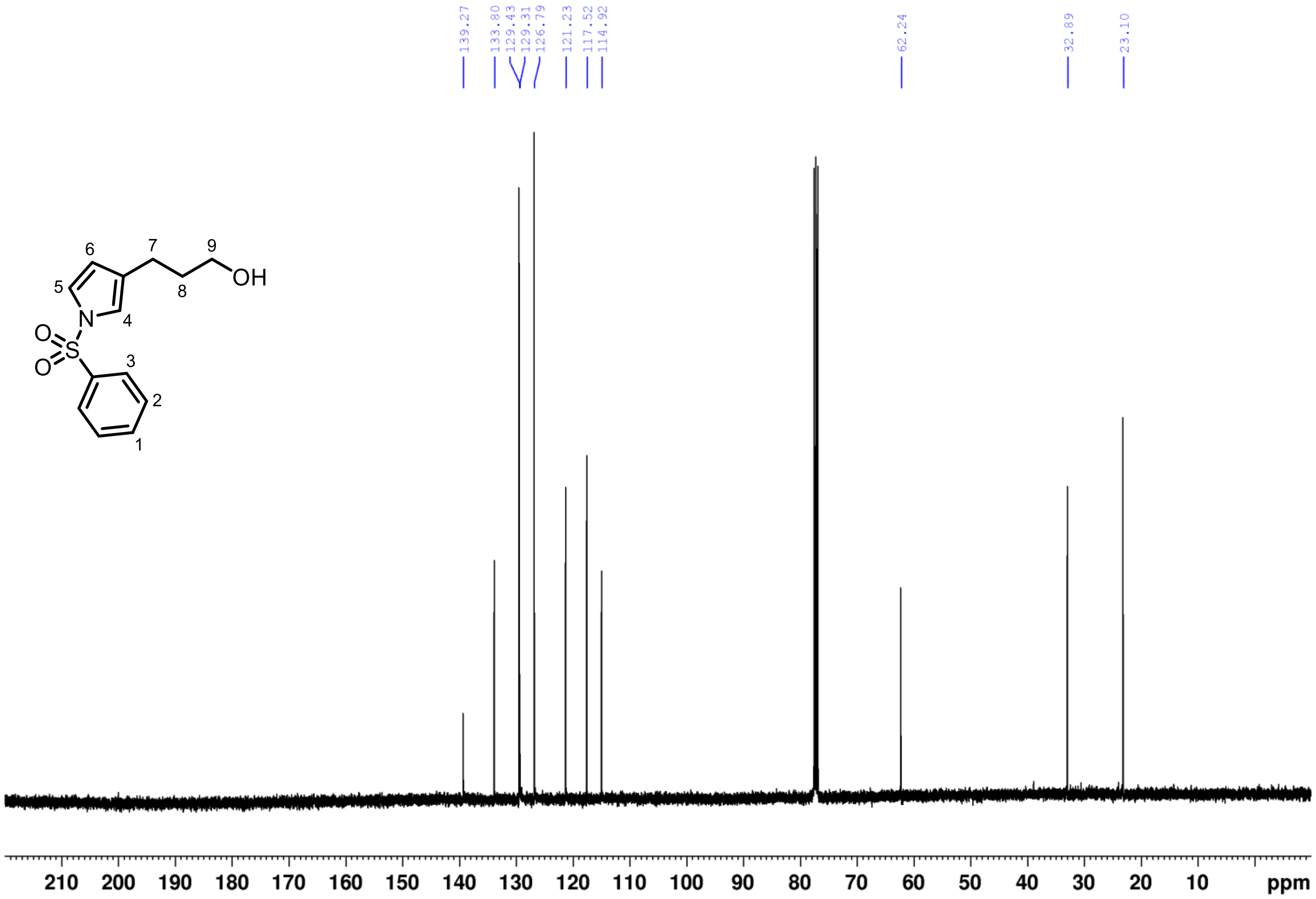

^{1}H NMR (400 MHz, CDCl$_3$) for *3-(benzofuran-2-yl)propan-1-ol* (**1x**)

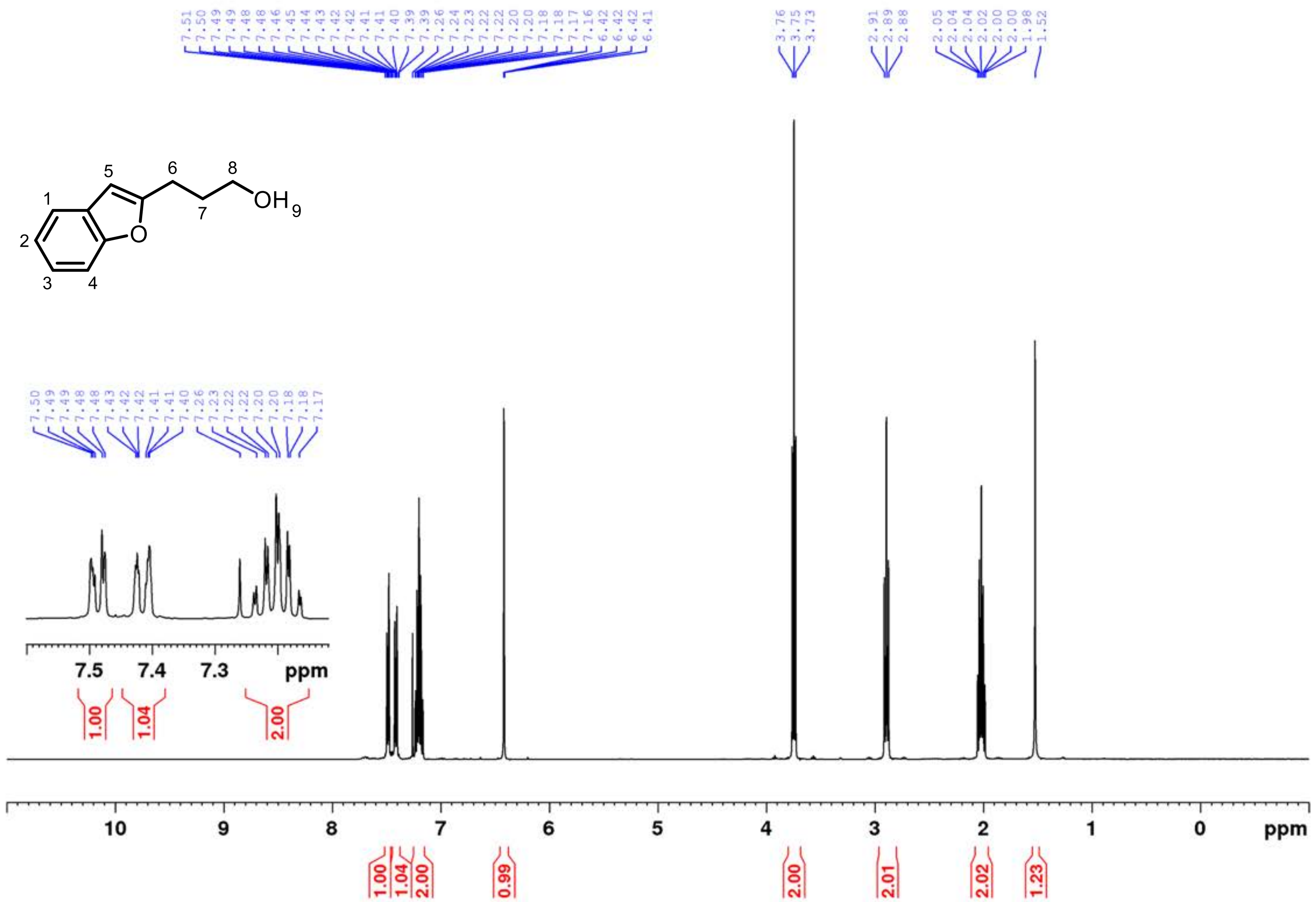

^{13}C NMR (101 MHz, CDCl$_3$) for *3-(benzofuran-2-yl)propan-1-ol* (**1x**)

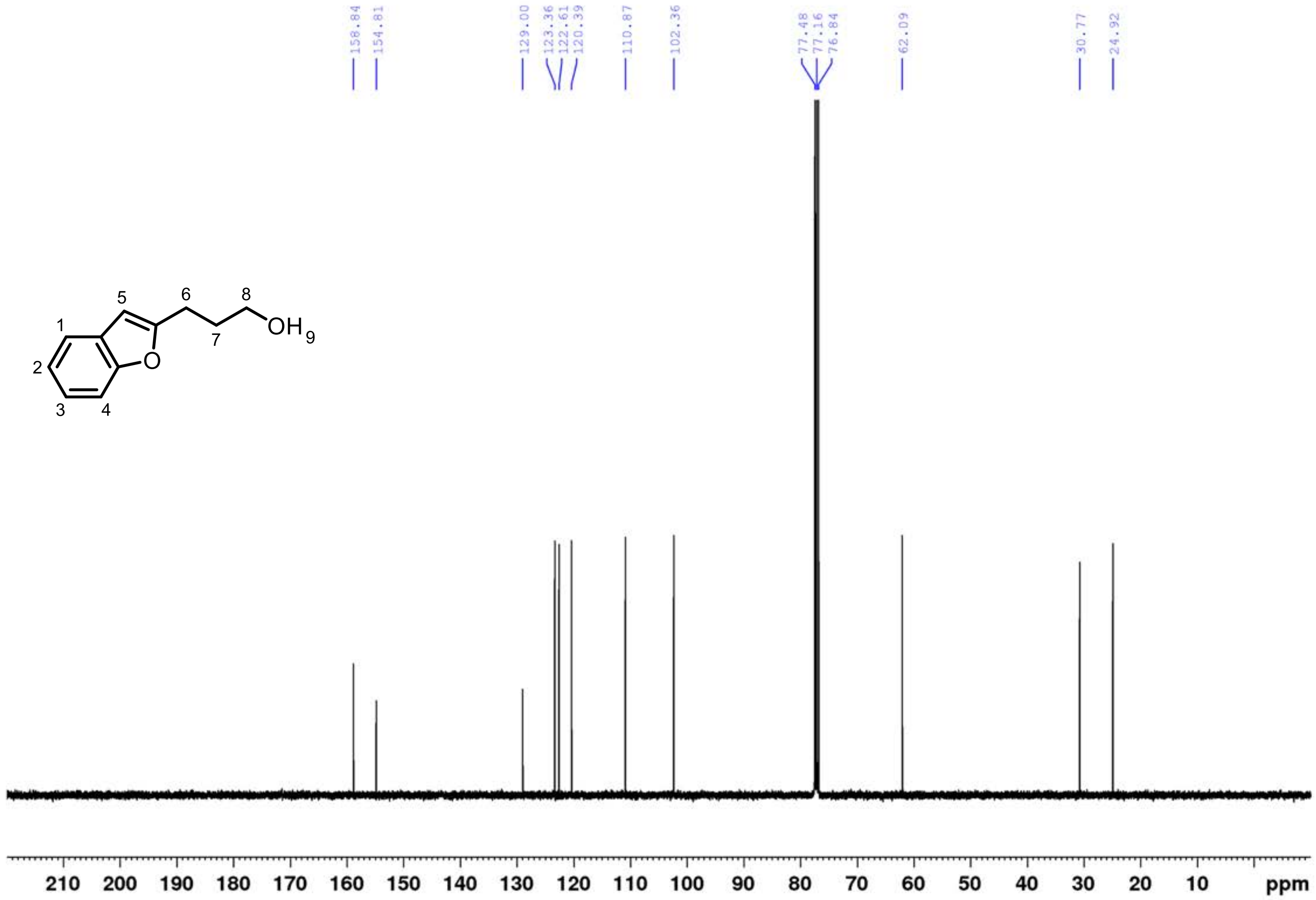

^{1}H NMR (700 MHz, CDCl$_3$) for *methyl 4-(3-hydroxyprop-1-yn-1-yl)furan-2-carboxylate*

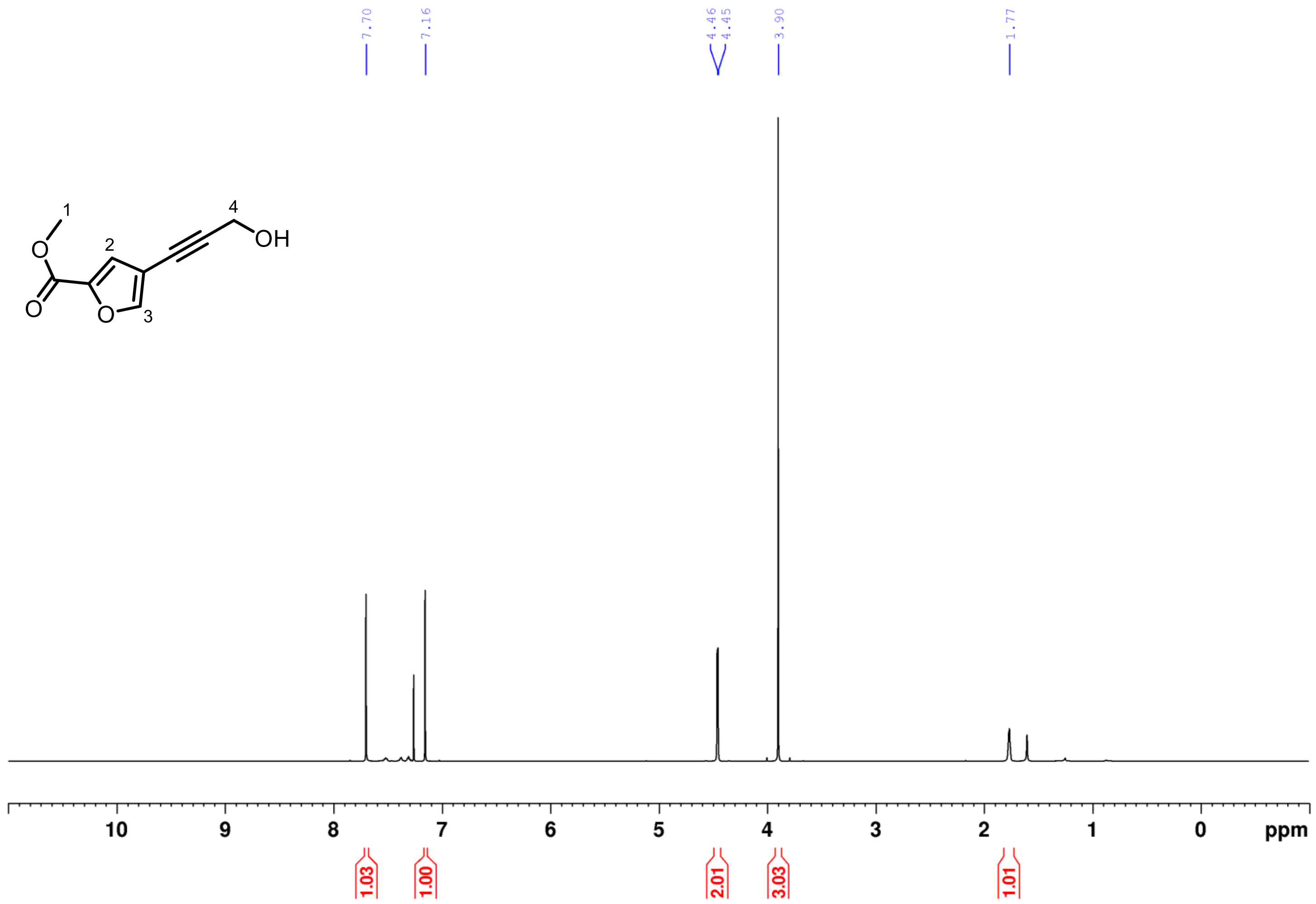

^{13}C NMR (176 MHz, $CDCl_3$) for *methyl 4-(3-hydroxyprop-1-yn-1-yl)furan-2-carboxylate*

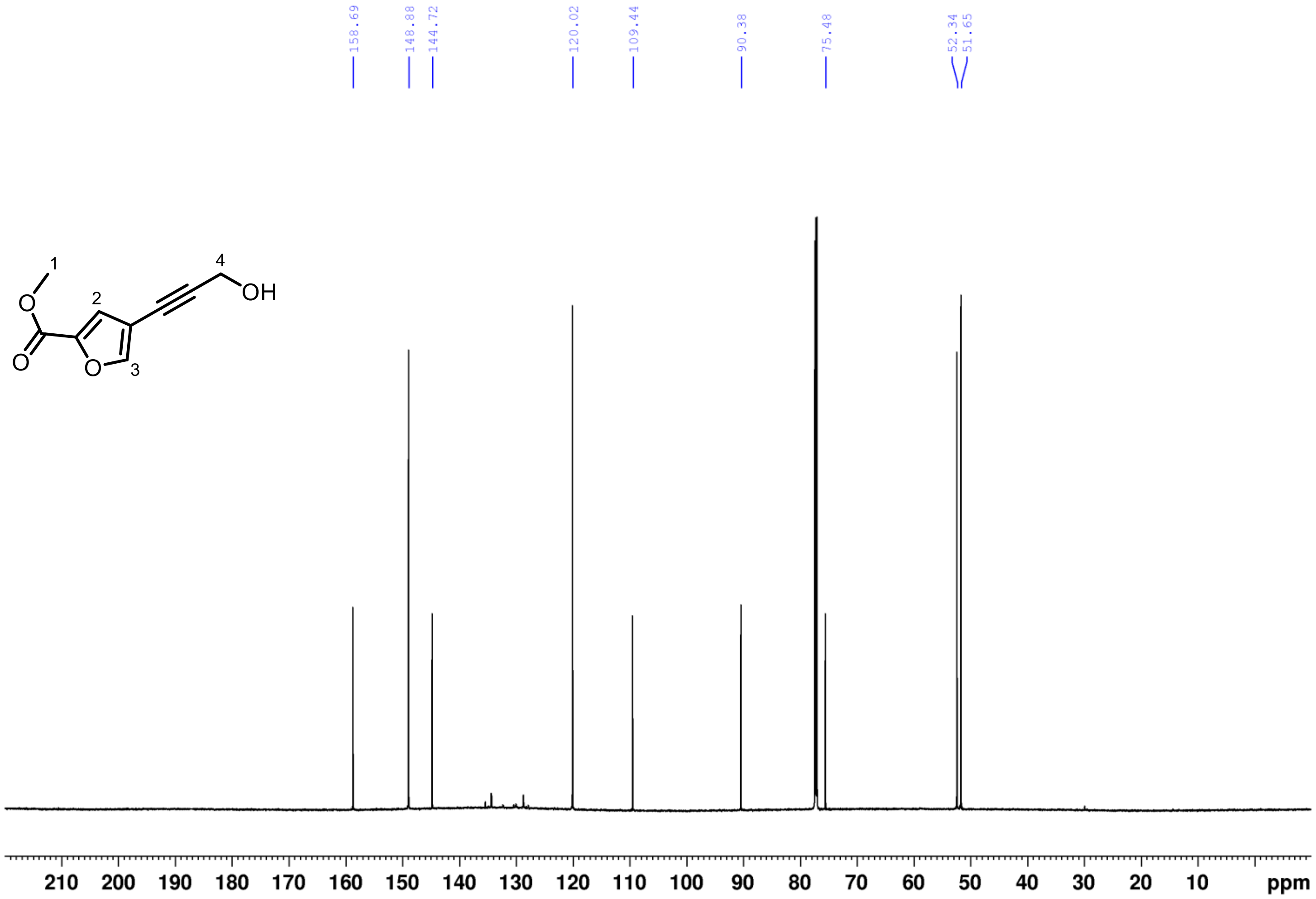

^{1}H NMR (400 MHz, $CDCl_3$) for *methyl 4-(3-hydroxypropyl)furan-2-carboxylate* (**1y**)

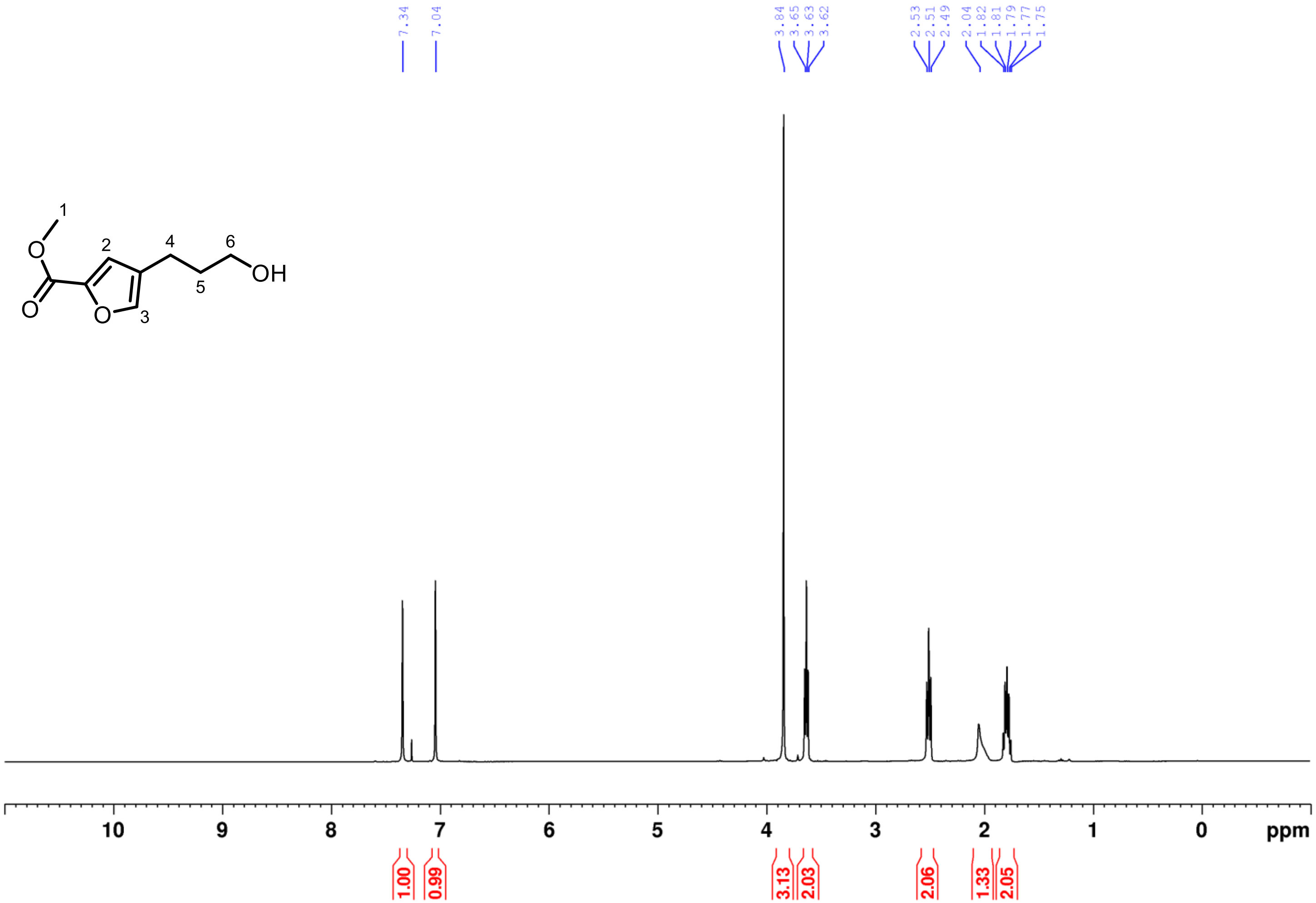

^{13}C NMR (101 MHz, CDCl$_3$) for *methyl 4-(3-hydroxypropyl)furan-2-carboxylate* (**1y**)

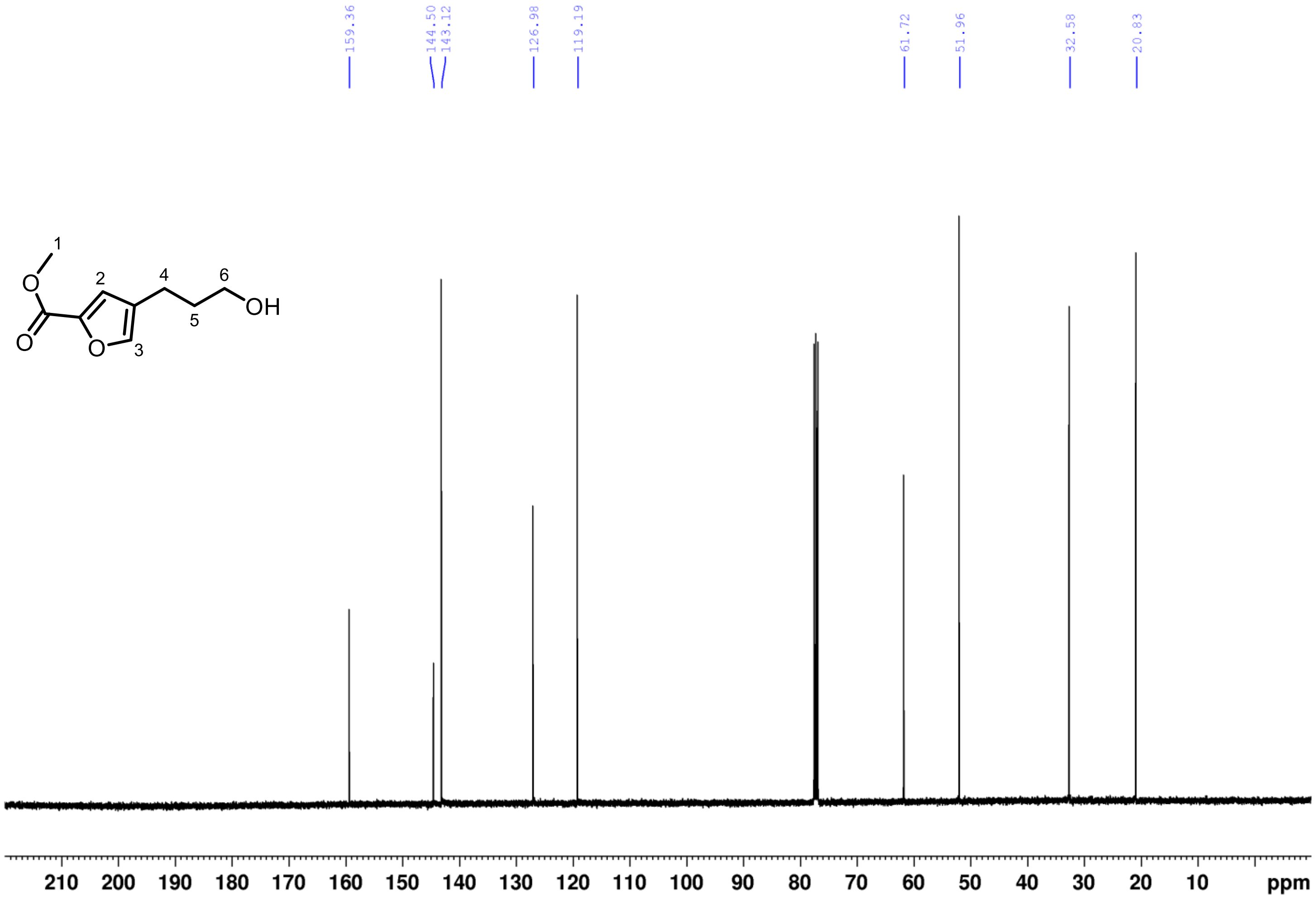

^{1}H NMR (400 MHz, CDCl$_3$) for *1,5-diphenylpentan-3-ol* (**1z**)

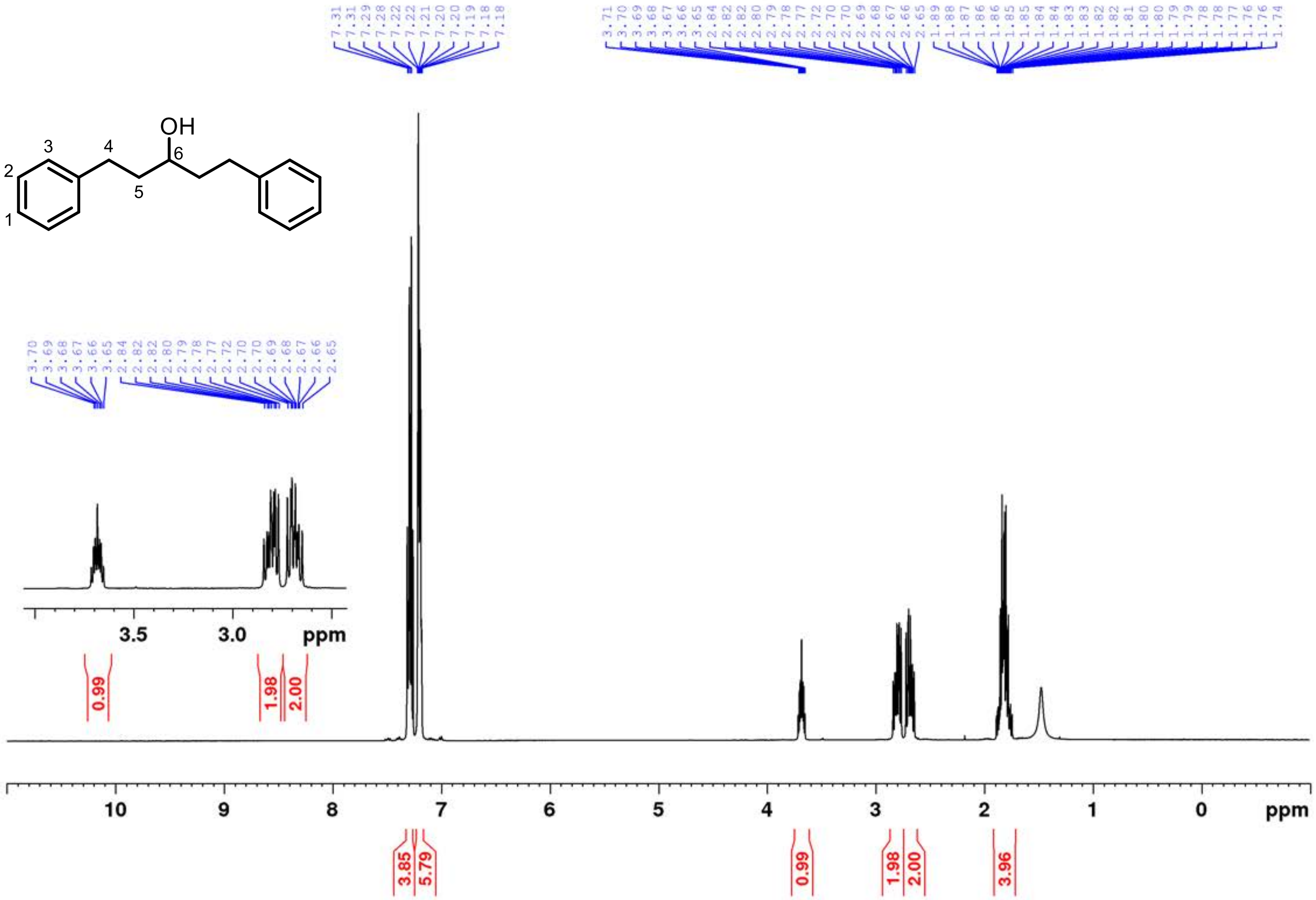

^{13}C NMR (101 MHz, CDCl$_3$) for *1,5-diphenylpentan-3-ol* (**1z**)

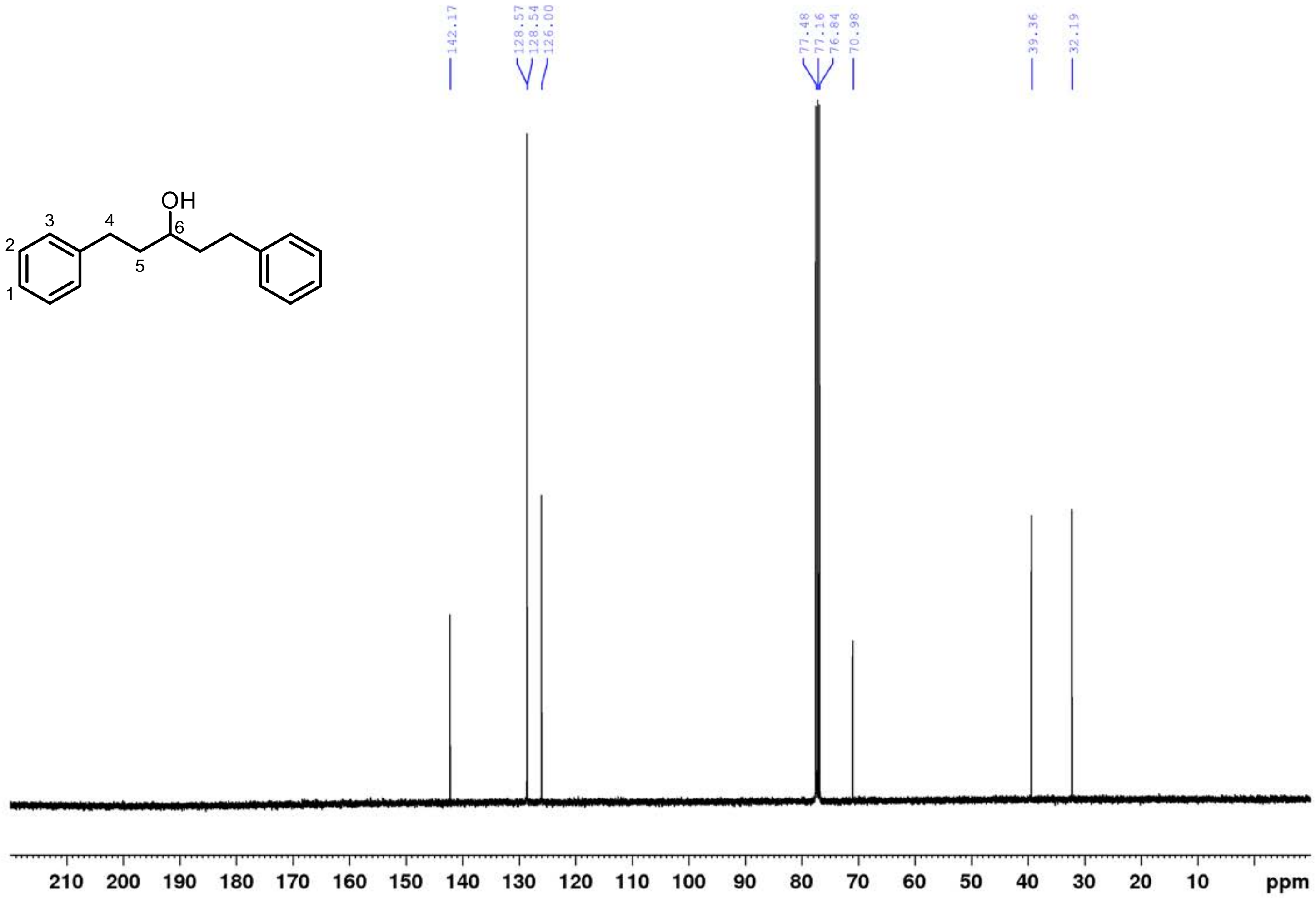

^{1}H NMR (400 MHz, $CDCl_3$) for *5-(4-(tert-butyl)phenyl)pentan-1-ol* (**6e**)

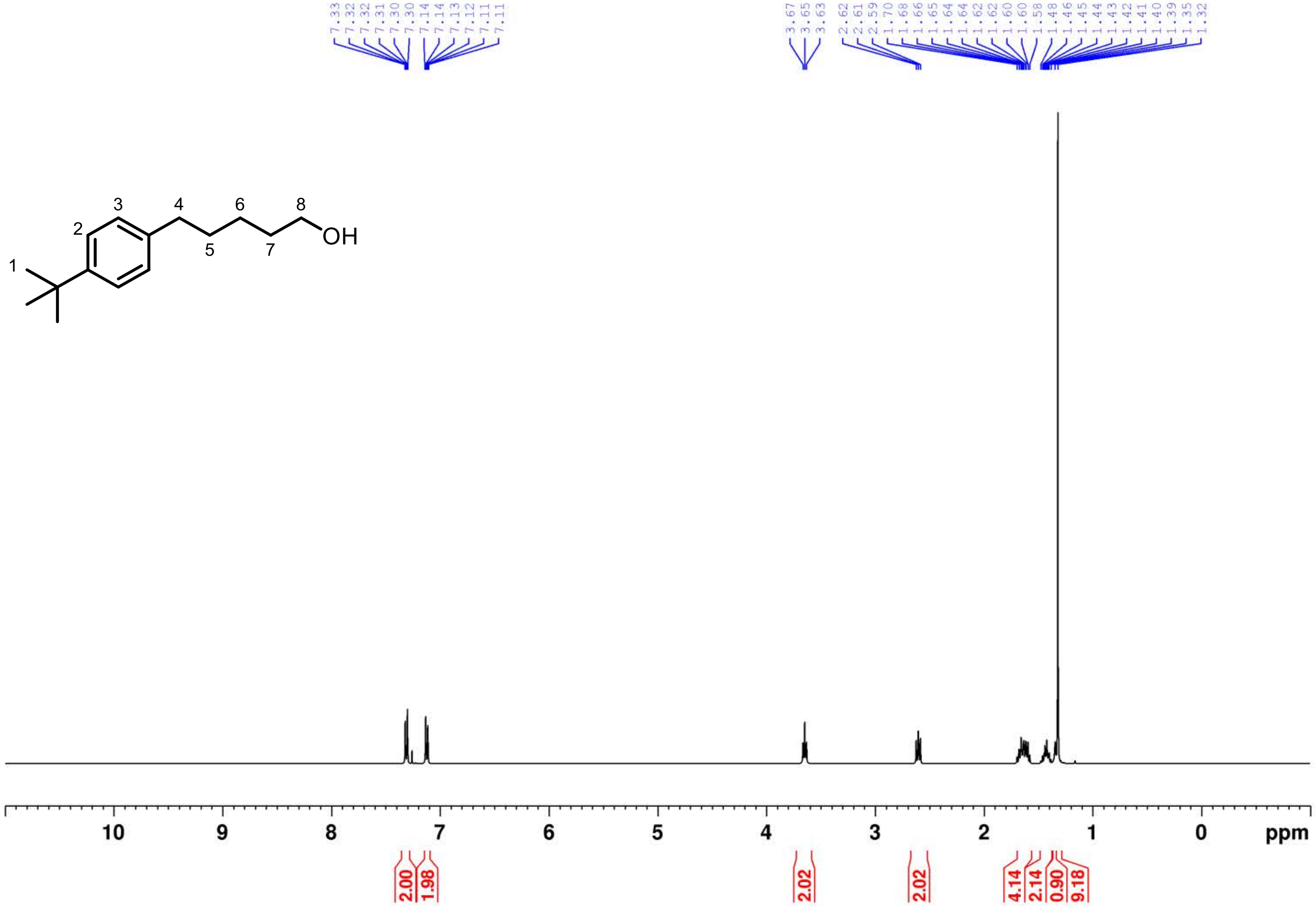

^{13}C NMR (101 MHz, $CDCl_3$) for *5-(4-(tert-butyl)phenyl)pentan-1-ol* (**6e**)

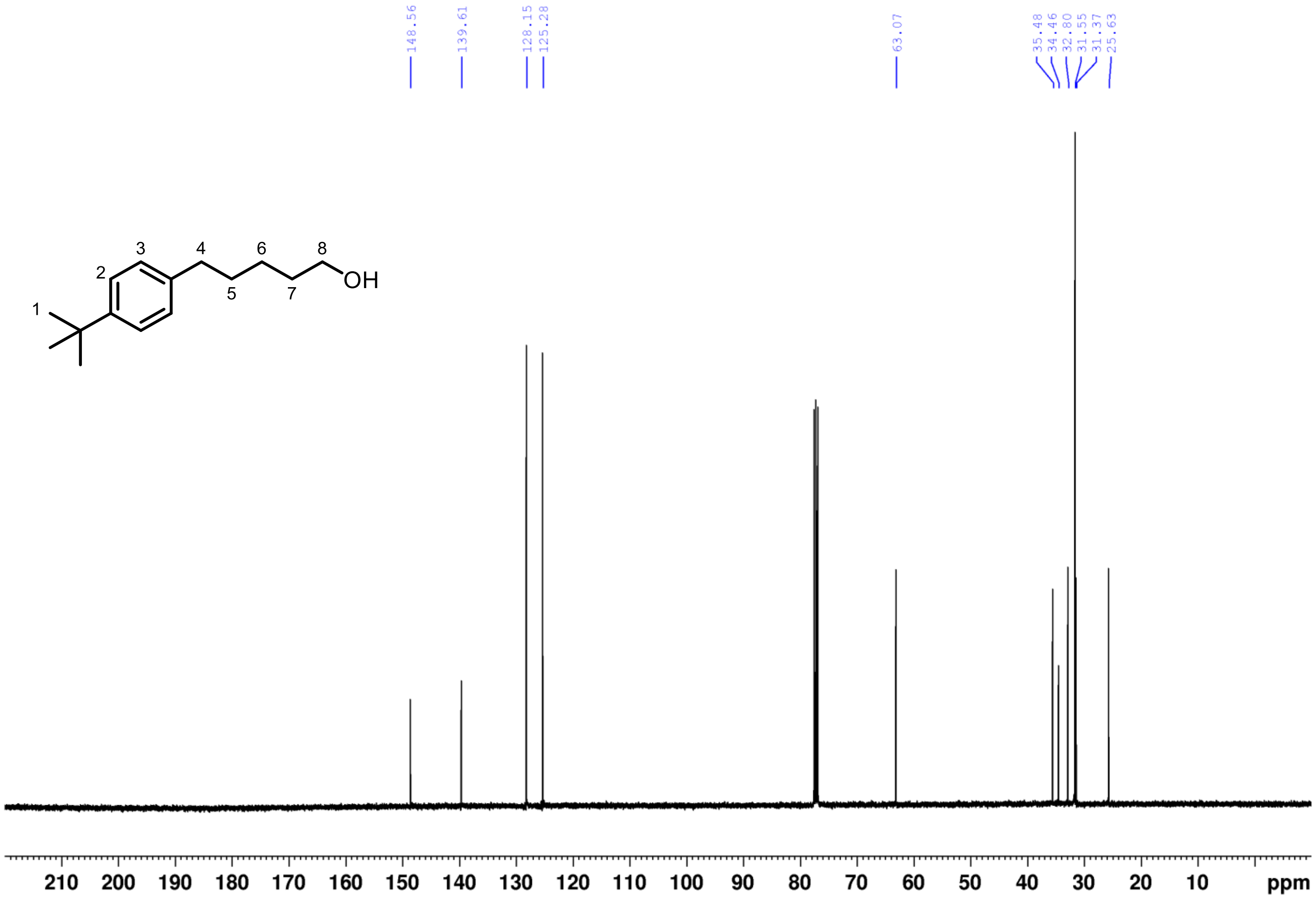

^{1}H NMR (400 MHz, CDCl$_3$) for *5-(3-fluoro-2-methylphenyl)pentan-1-ol* (**6f**)

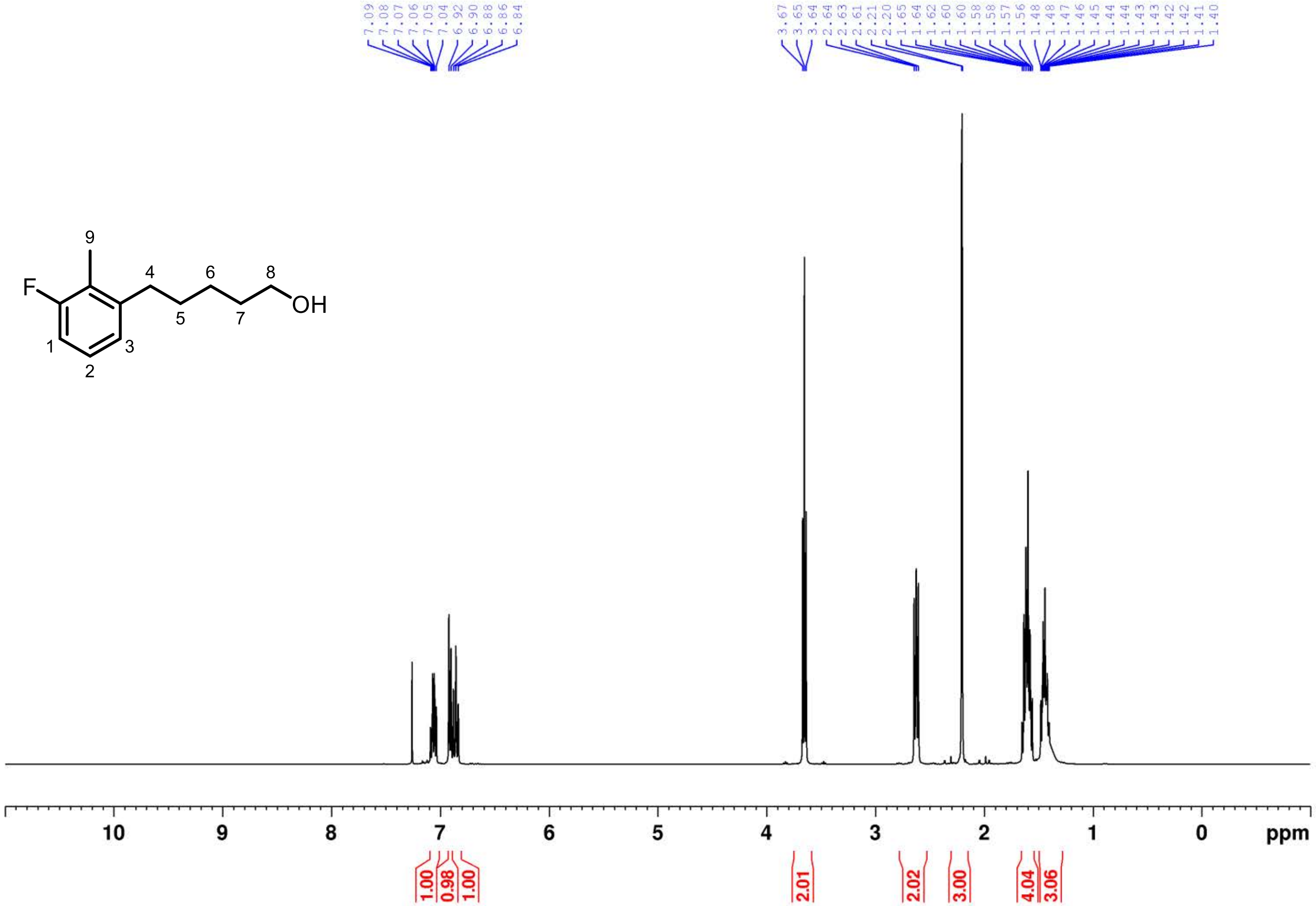

^{13}C NMR (101 MHz, $CDCl_3$) for *5-(3-fluoro-2-methylphenyl)pentan-1-ol* (**6f**)

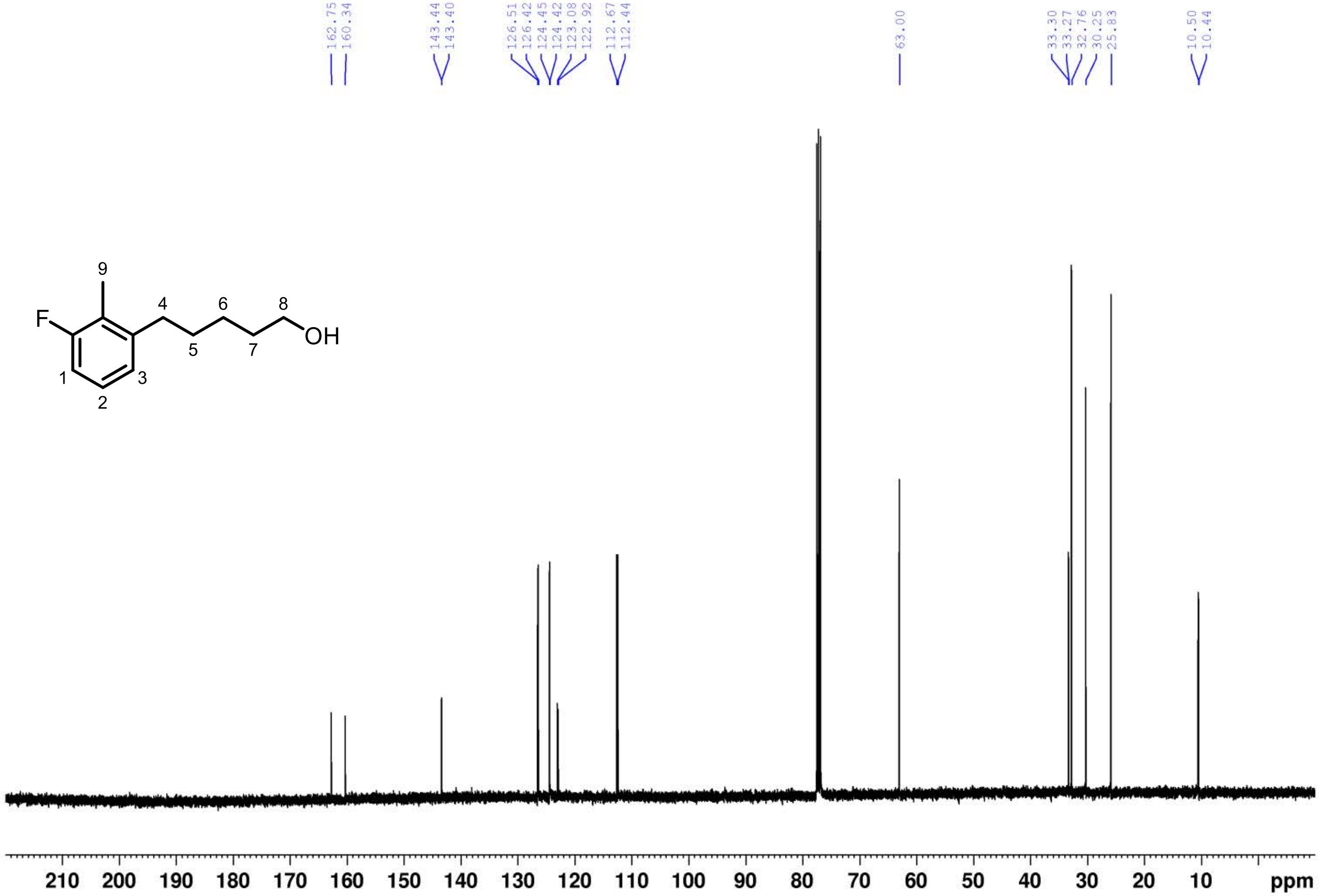

^{19}F NMR (376 MHz, CDCl$_3$) for *5-(3-fluoro-2-methylphenyl)pentan-1-ol* (**6f**)

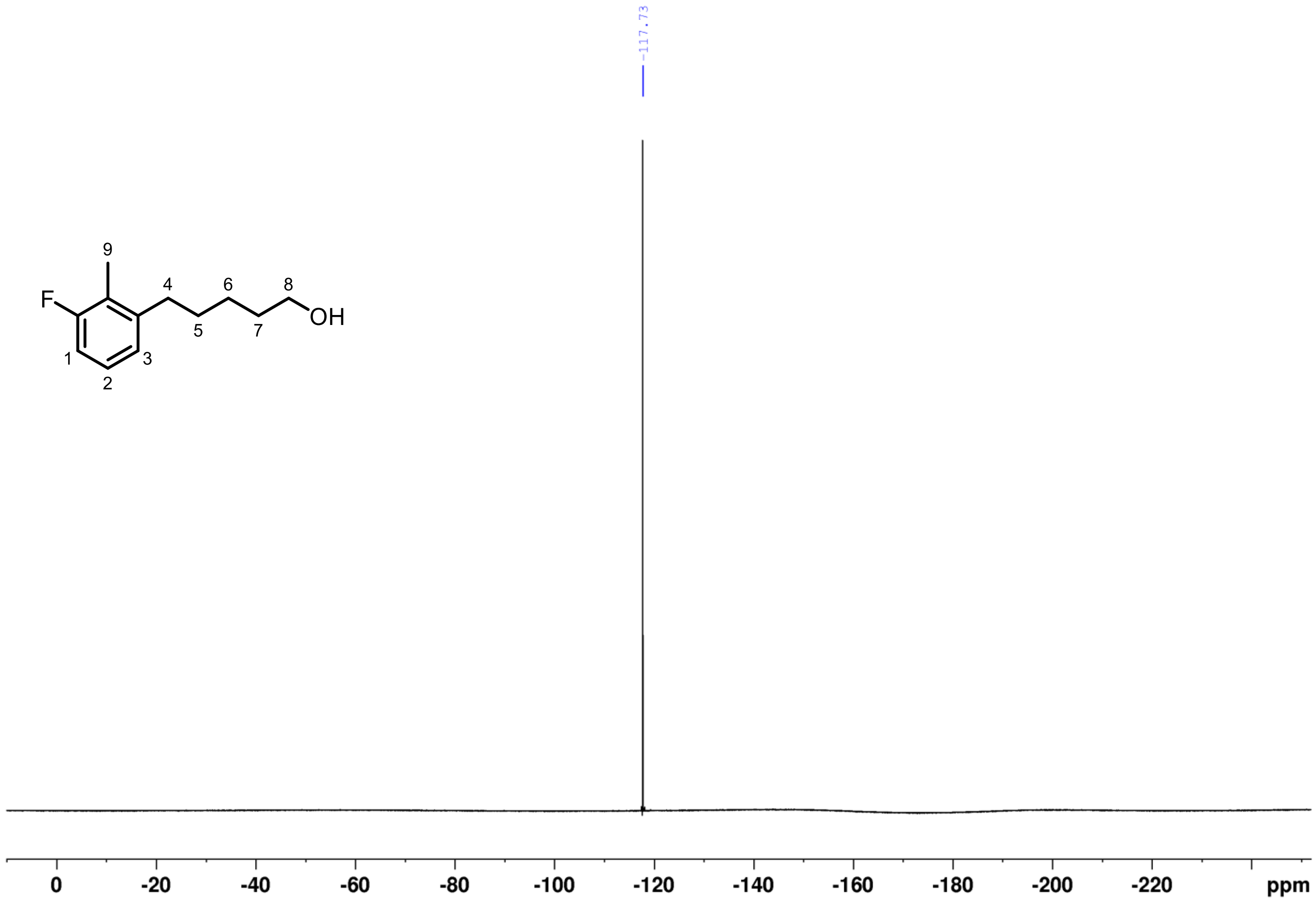

^{1}H NMR (400 MHz, $CDCl_3$) for *(3-methoxypropyl)benzene* (**S1**)

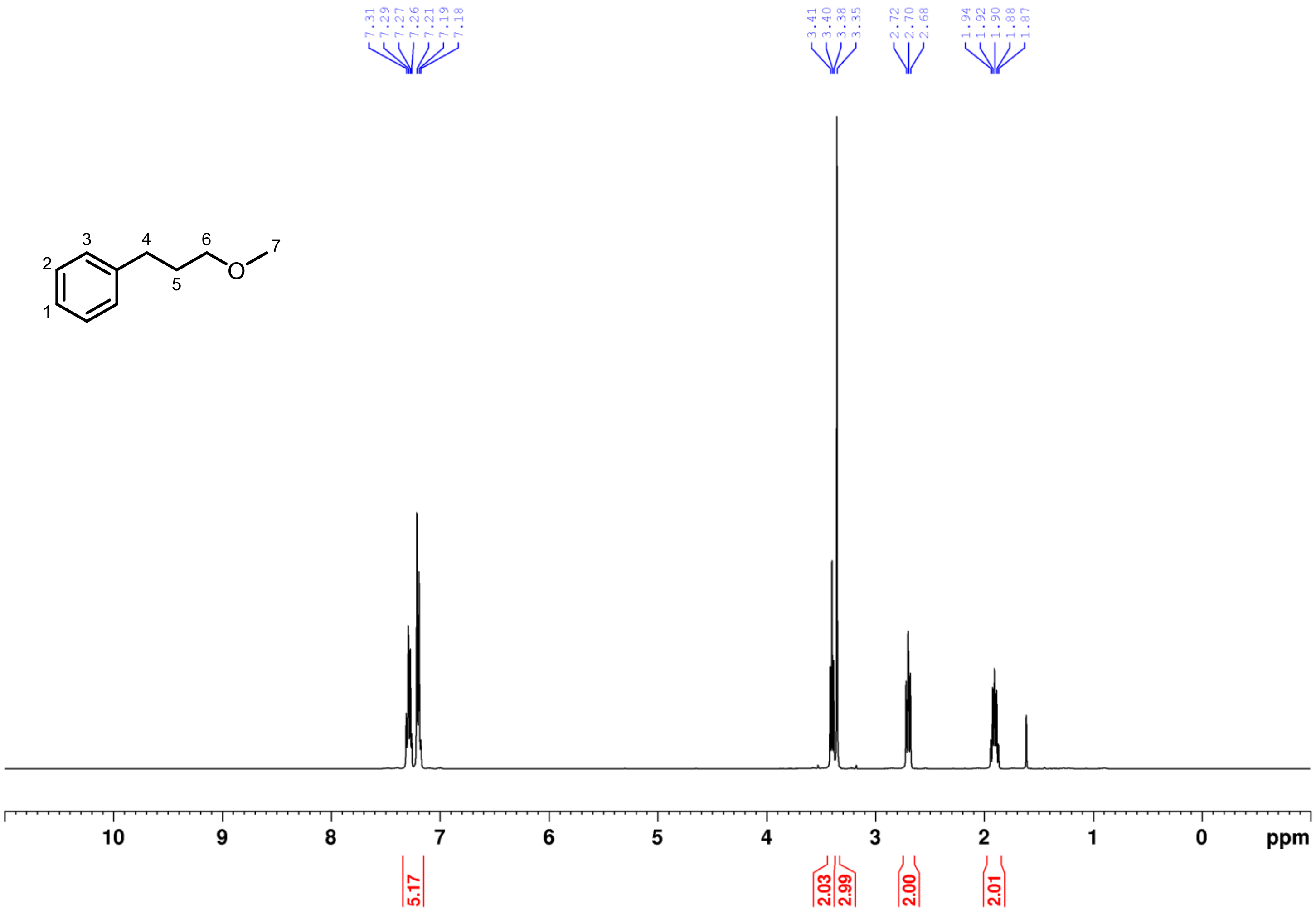

^{13}C NMR (101 MHz, CDCl$_3$) for *(3-methoxypropyl)benzene* (**S1**)

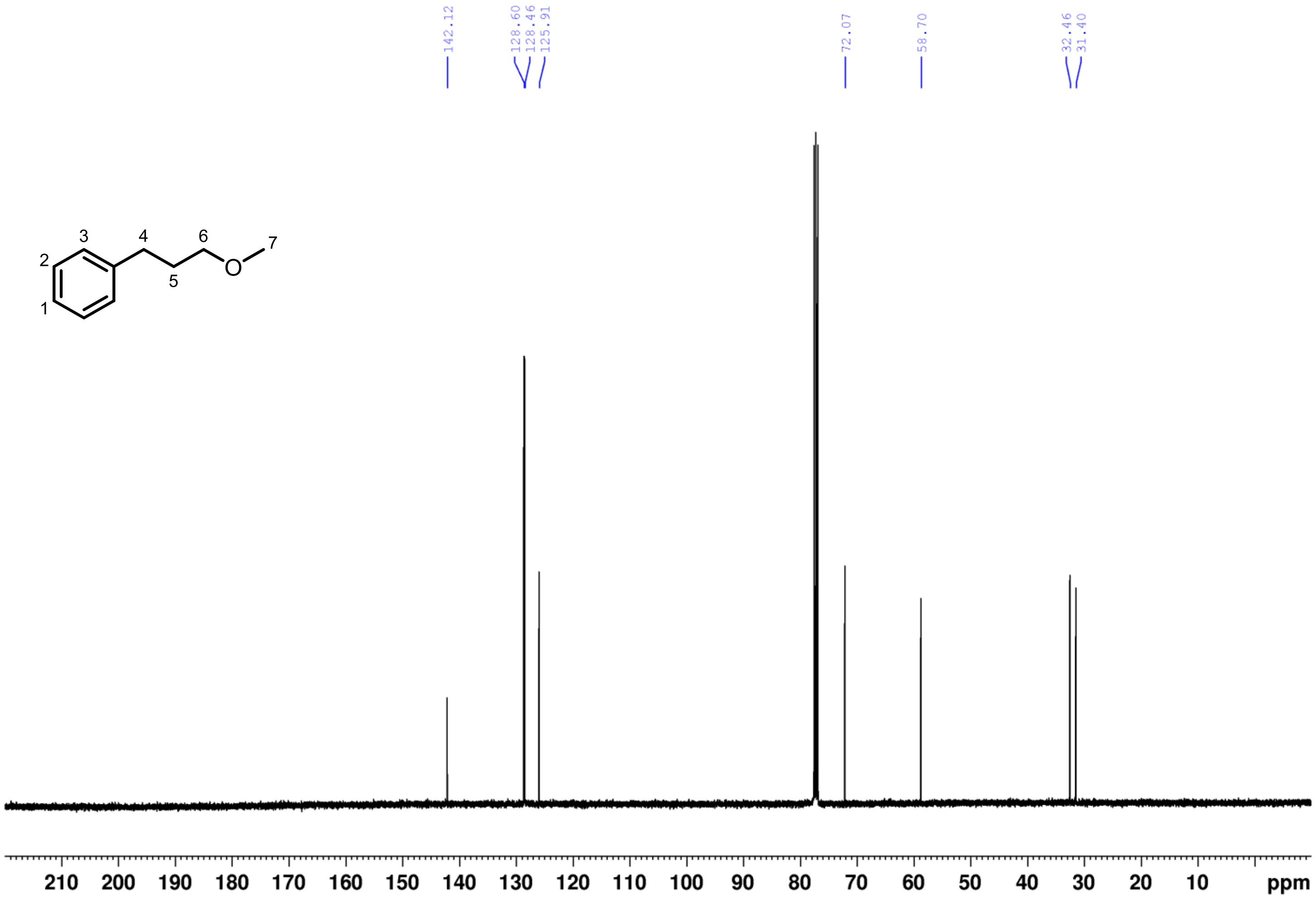

^{1}H NMR (400 MHz, CDCl$_3$) for *ethyl 3-butylhept-2-enoate*

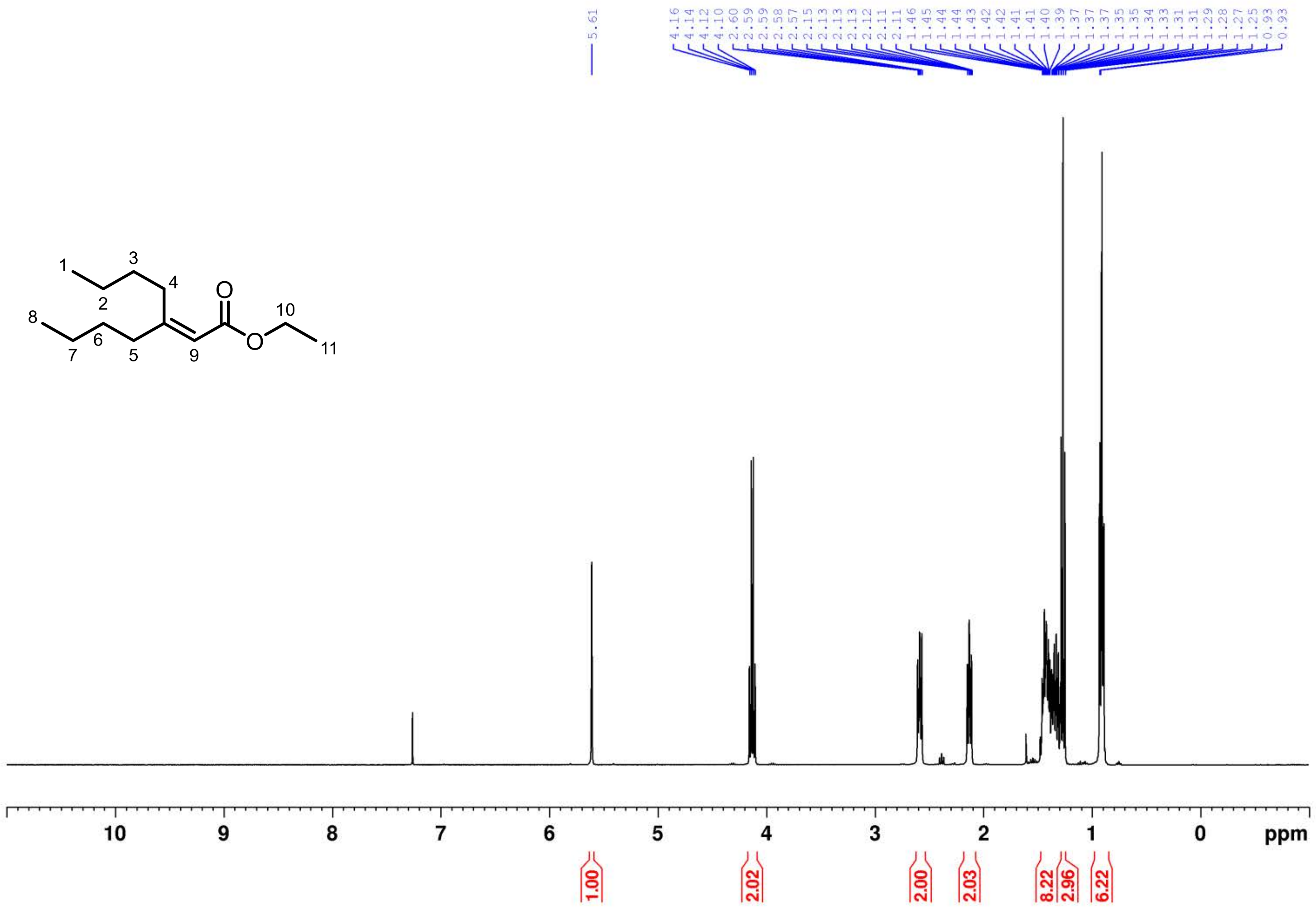

^{13}C NMR (101 MHz, CDCl$_3$) for *ethyl 3-butylhept-2-enoate*

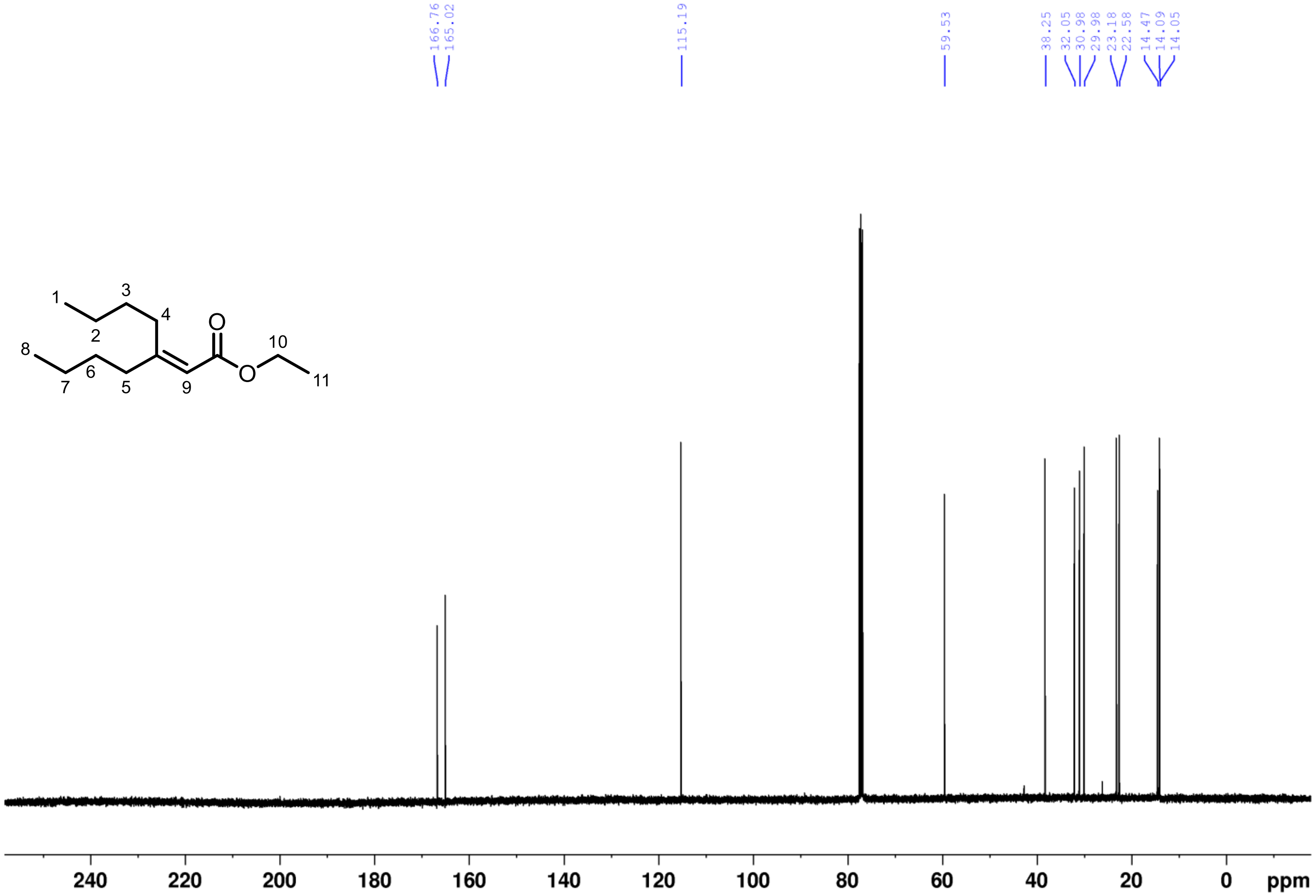

^{1}H NMR (400 MHz, CDCl$_3$) for *3-butylhept-2-en-1-ol* (**8b**)

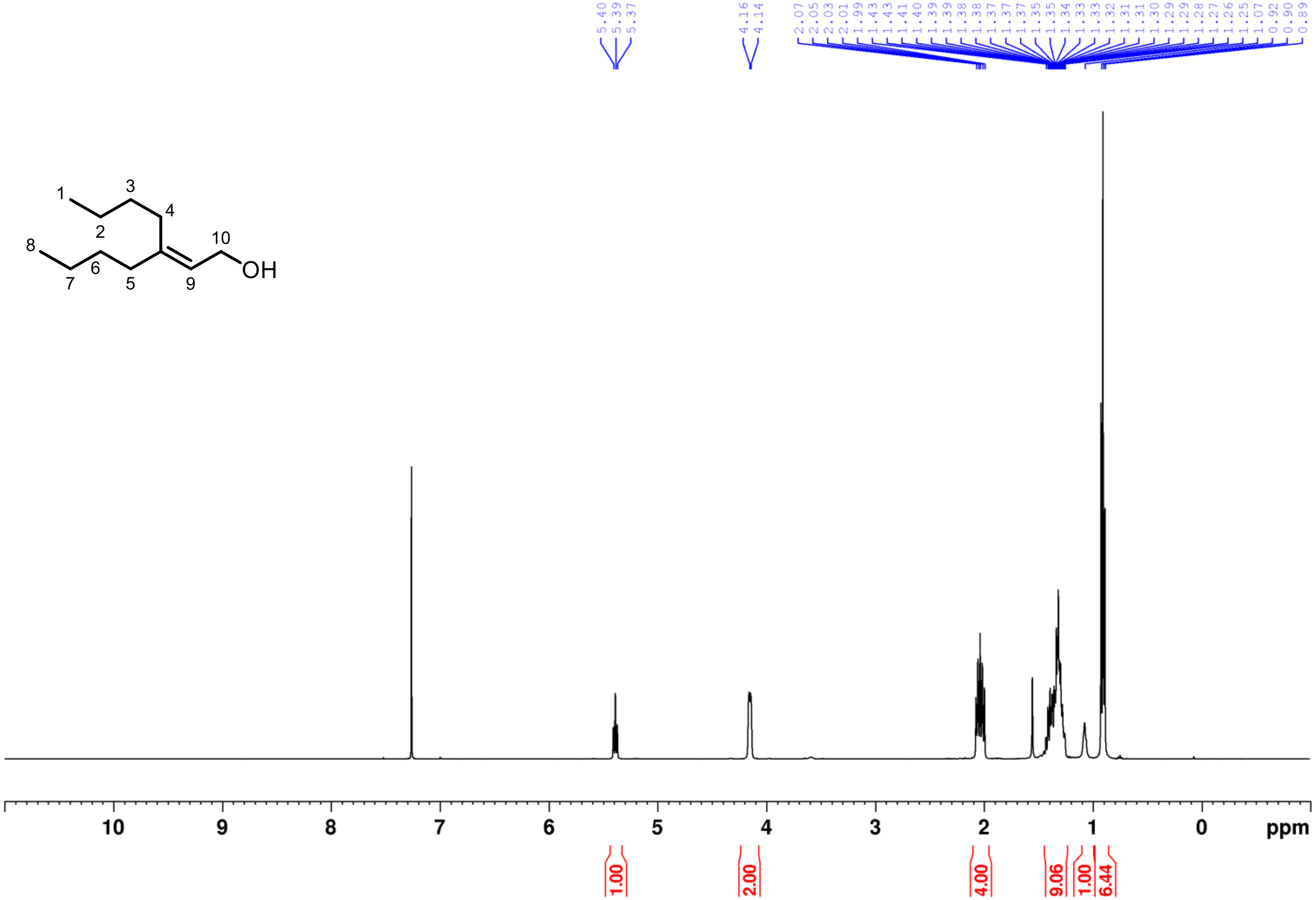

^{13}C NMR (101 MHz, CDCl$_3$) for *3-butylhept-2-en-1-ol* (**8b**)

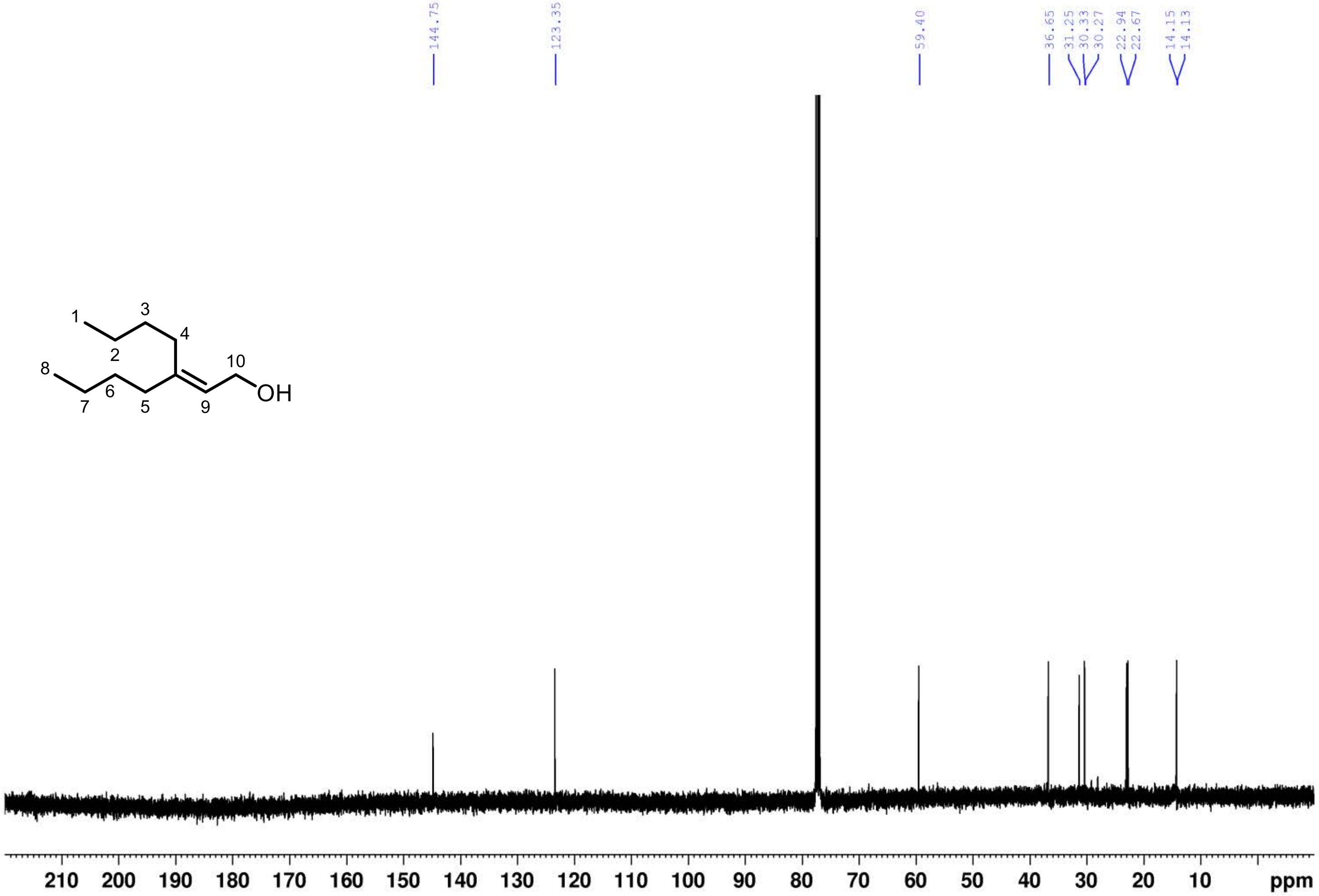

^{1}H NMR (700 MHz, $CDCl_3$) for *ethyl cyclohexylideneacetate*

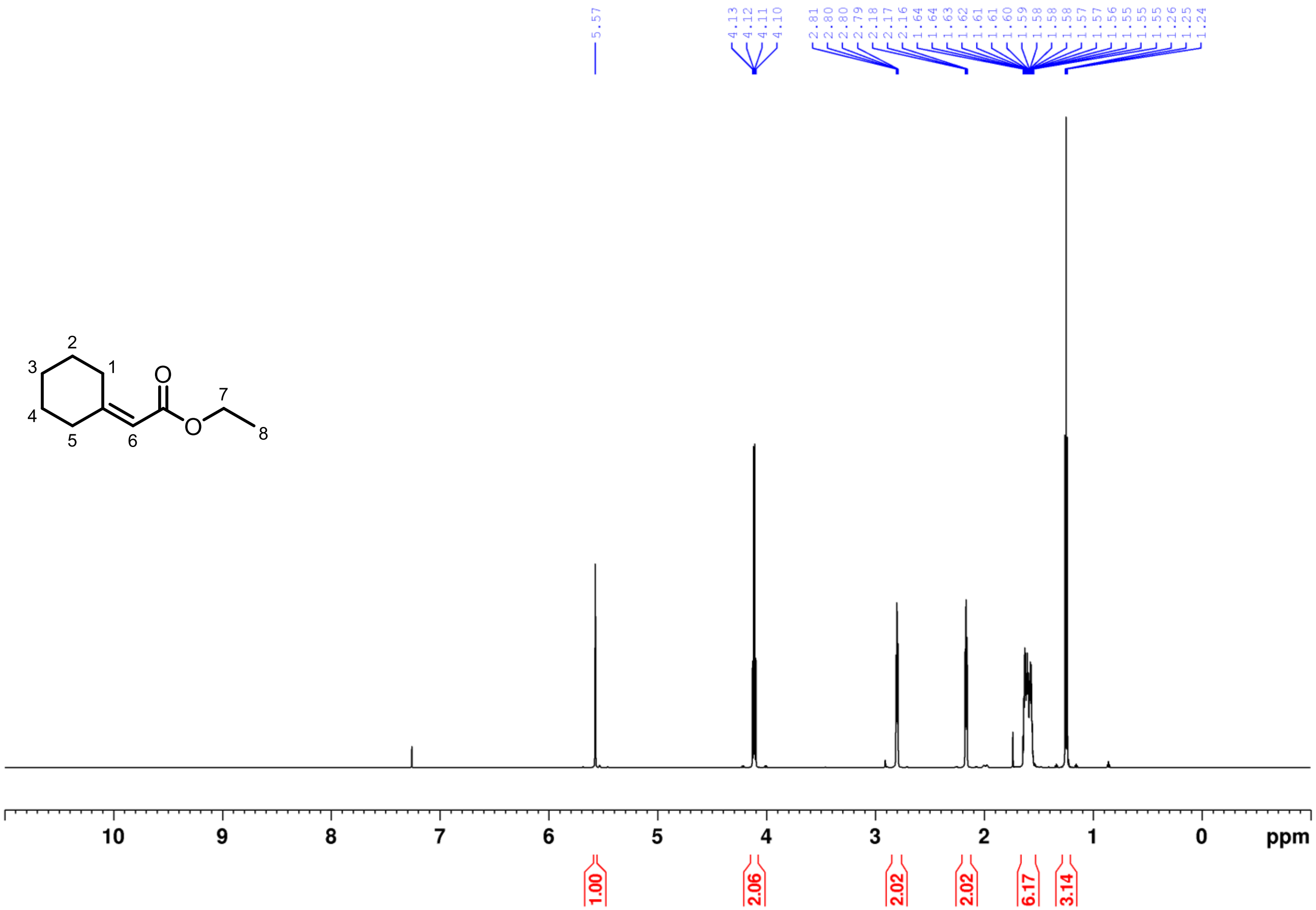

^{13}C NMR (176 MHz, CDCl$_3$) for *ethyl cyclohexylideneacetate*

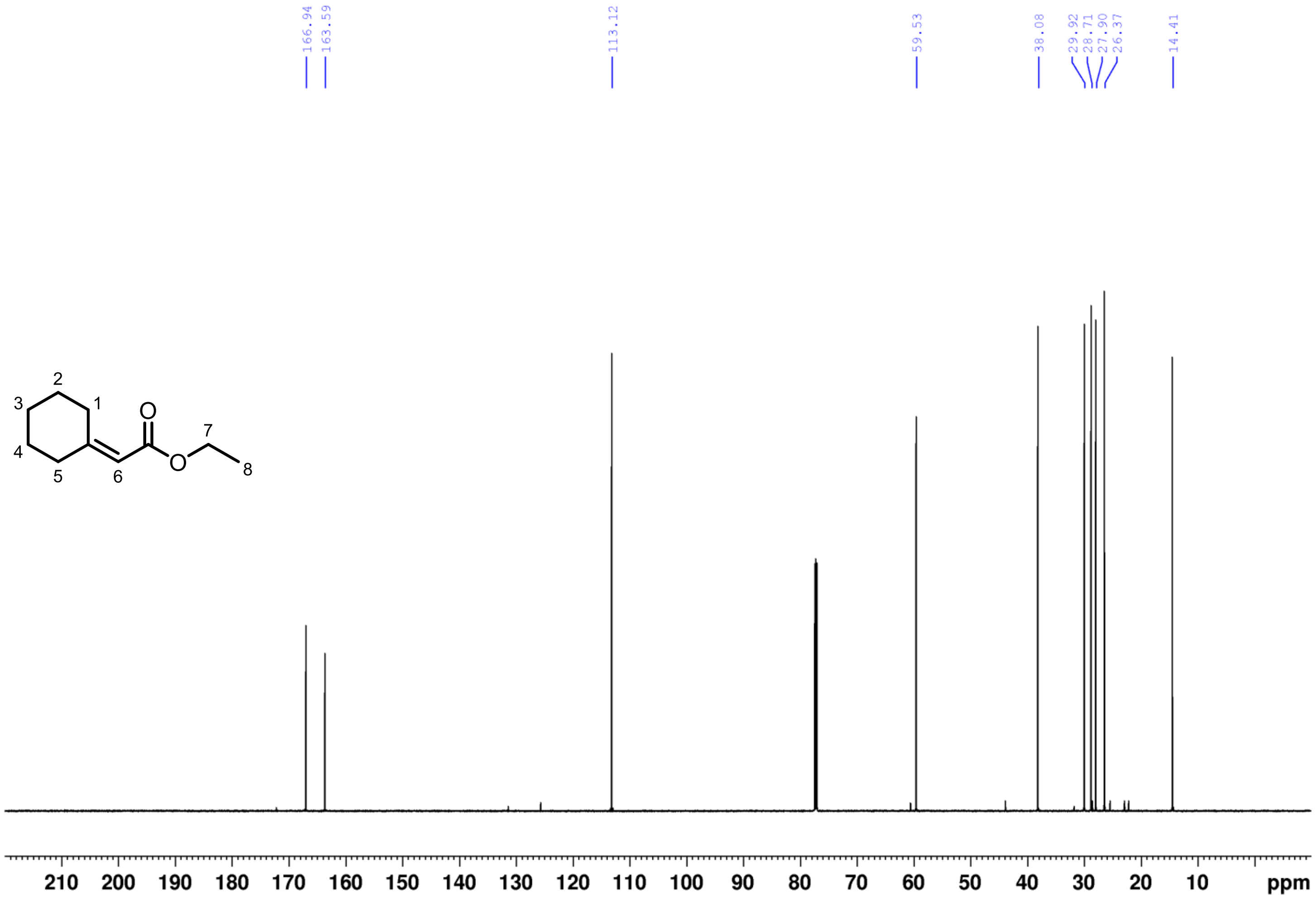

^{1}H NMR (700 MHz, $CDCl_3$) for *2-cyclohexylideneethan-1-ol* (**8c**)

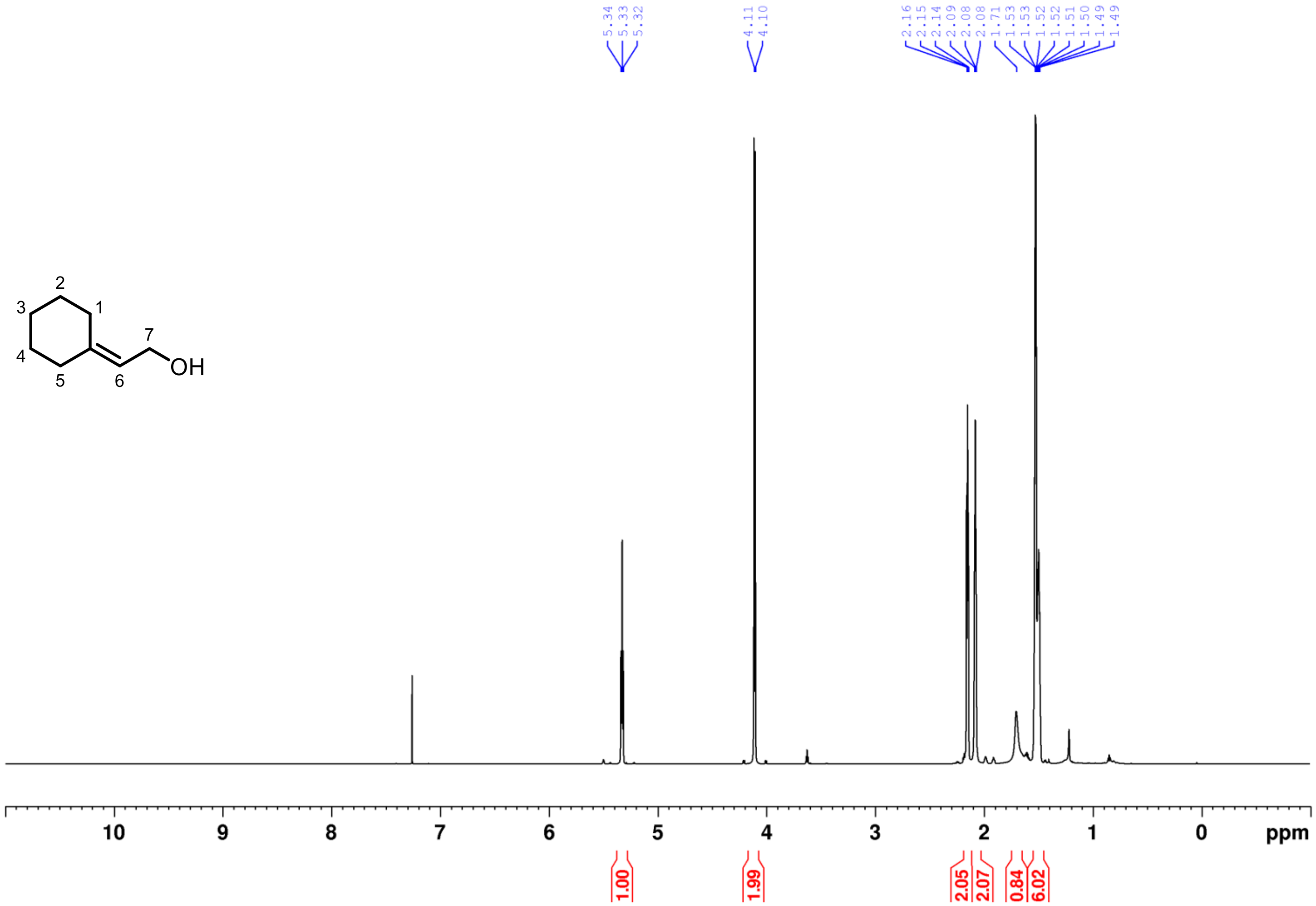

^{13}C NMR (176 MHz, $CDCl_3$) for *2-cyclohexylideneethan-1-ol* (**8c**)

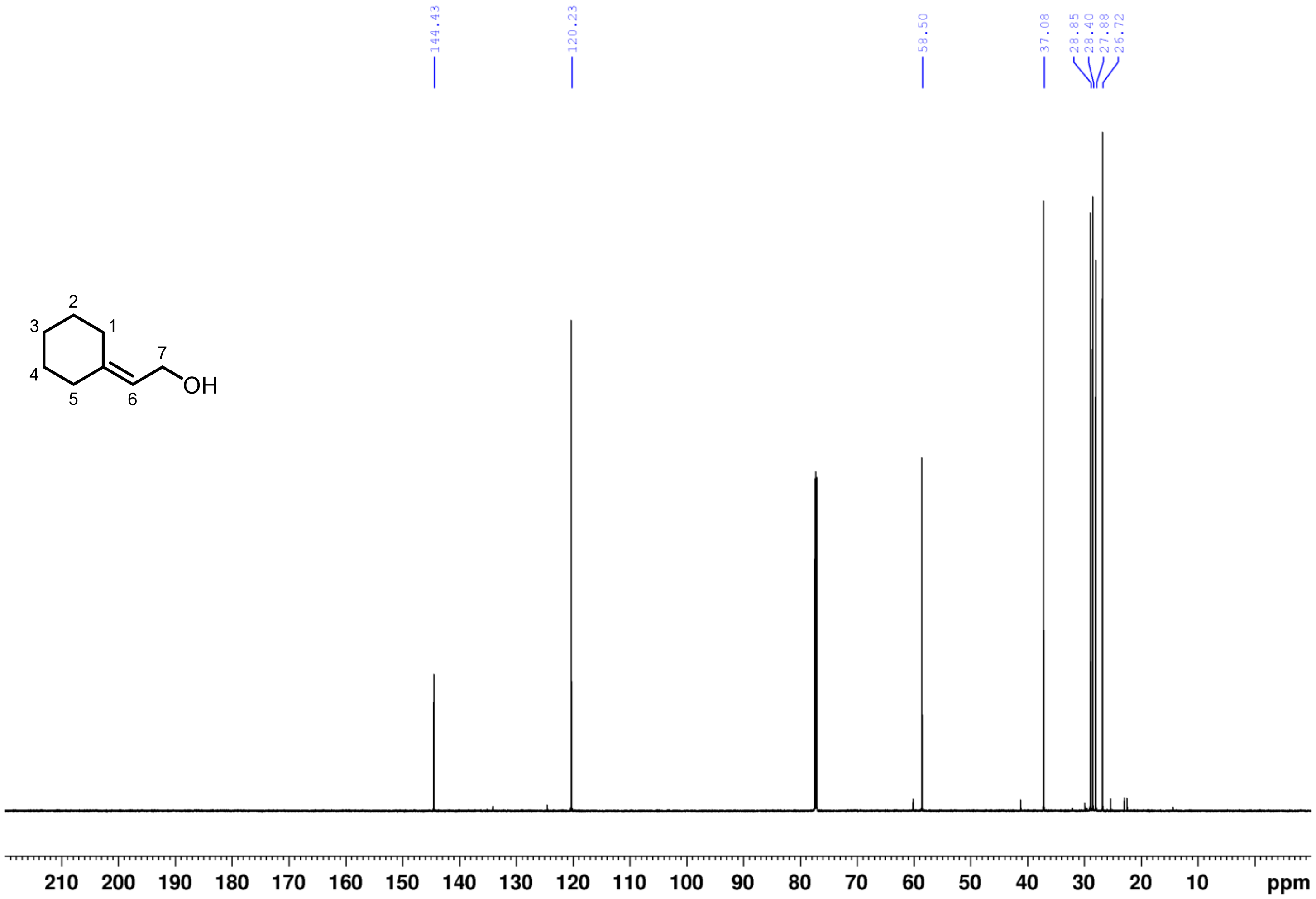

^{1}H NMR (700 MHz, $CDCl_3$) for *ethyl 2-cyclododecylideneacetate*

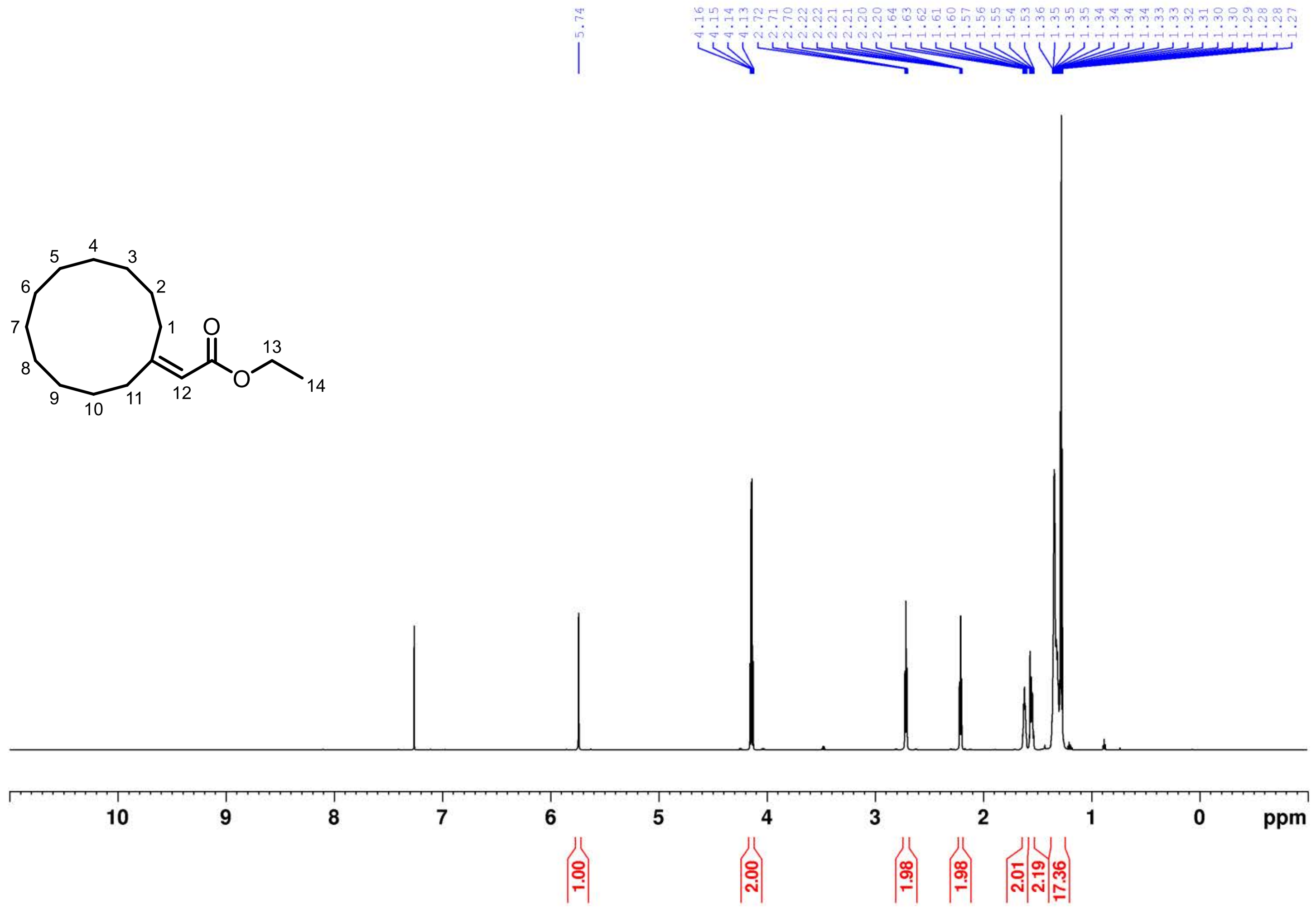

^{13}C NMR (176 MHz, CDCl$_3$) for *ethyl 2-cyclododecylideneacetate*

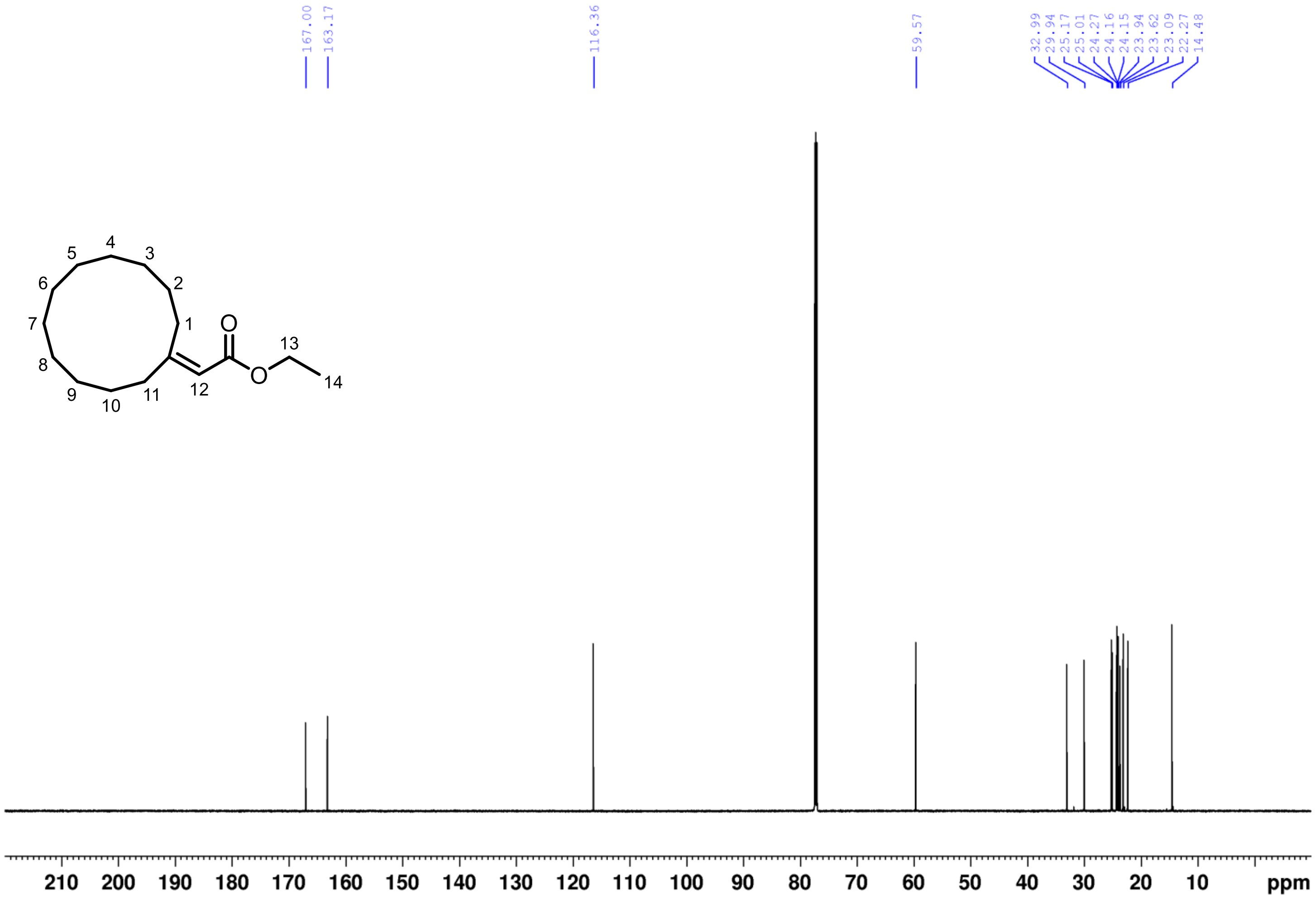

^{1}H NMR (400 MHz, CDCl$_3$) for *2-cyclododecylideneethan-1-ol* (**8d**)

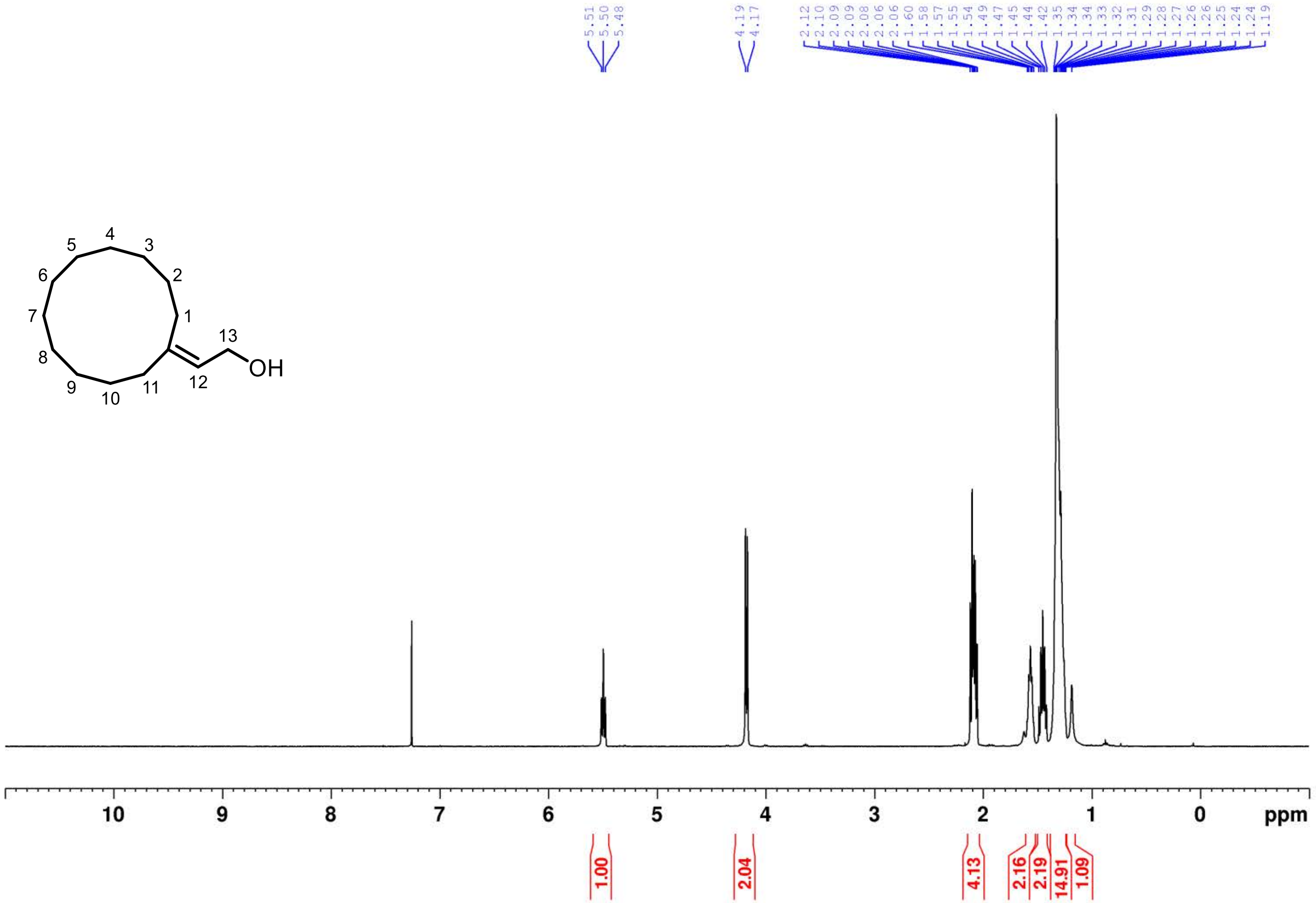

^{13}C NMR (101 MHz, CDCl₃) for *2-cyclododecylideneethan-1-ol* (**8d**)

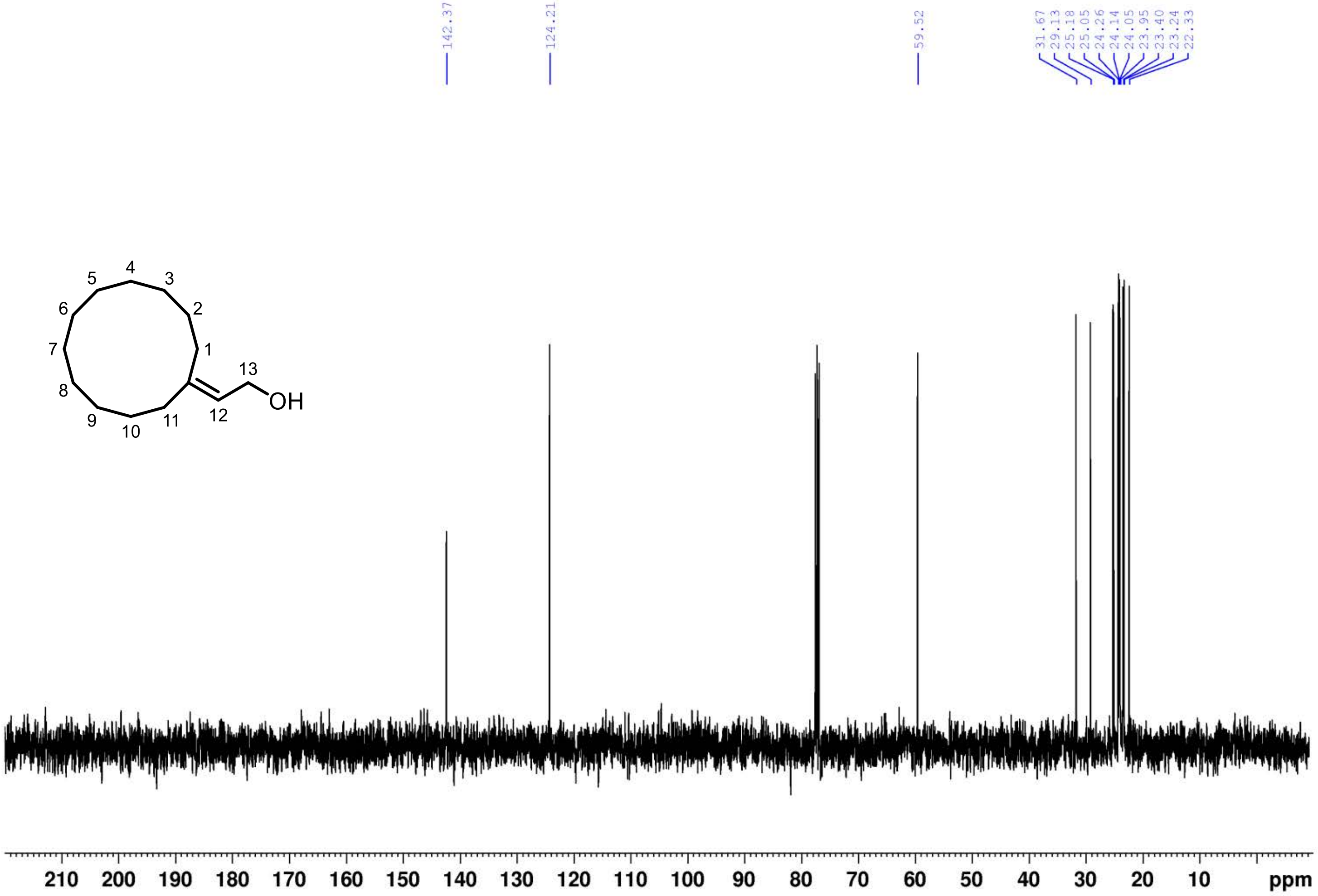

¹H NMR (400 MHz, $CDCl_3$) for *ethyl 2-(4,4-difluorocyclohexylidene)acetate*

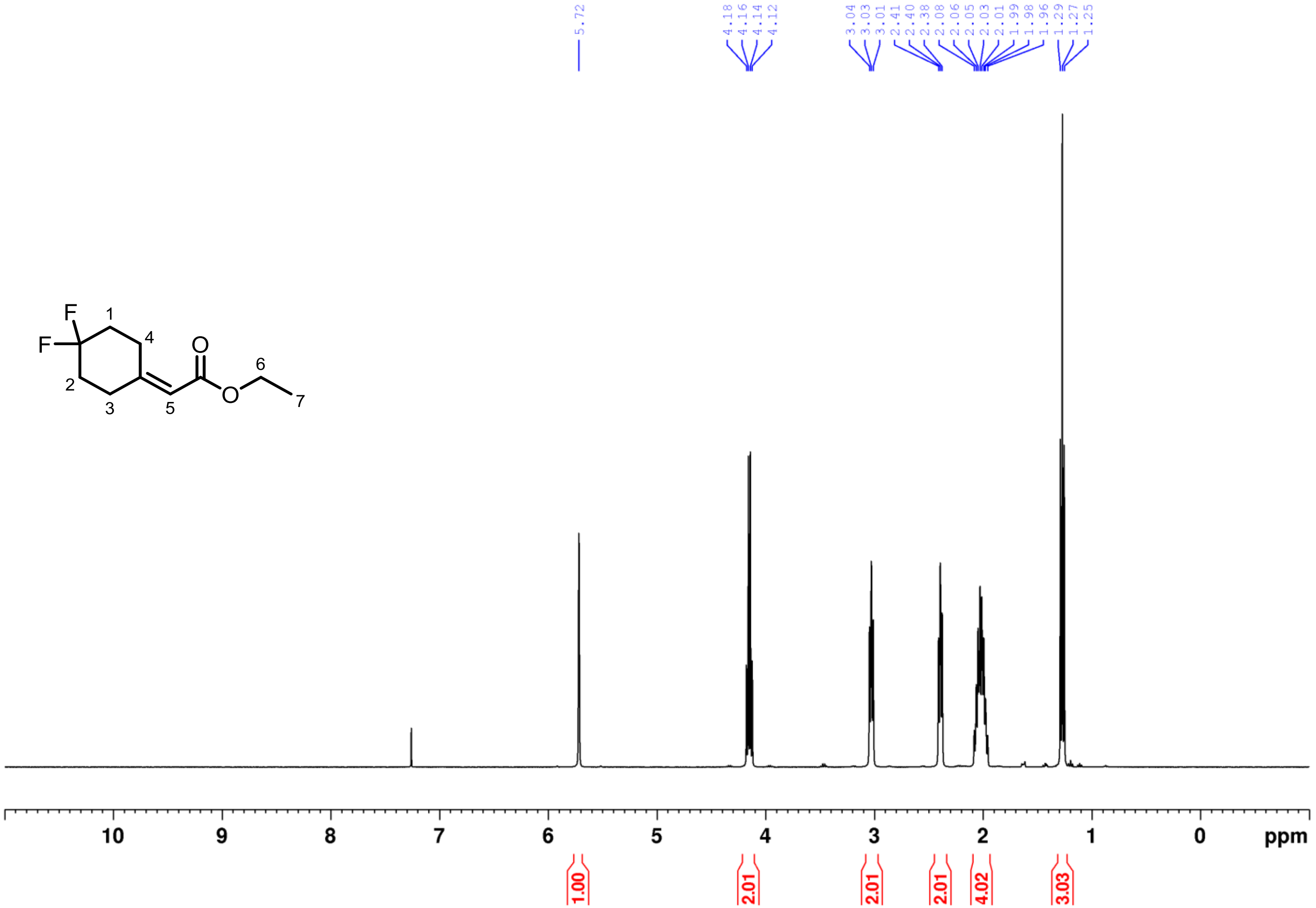

^{13}C NMR (101 MHz, CDCl$_3$) for *ethyl 2-(4,4-difluorocyclohexylidene)acetate*

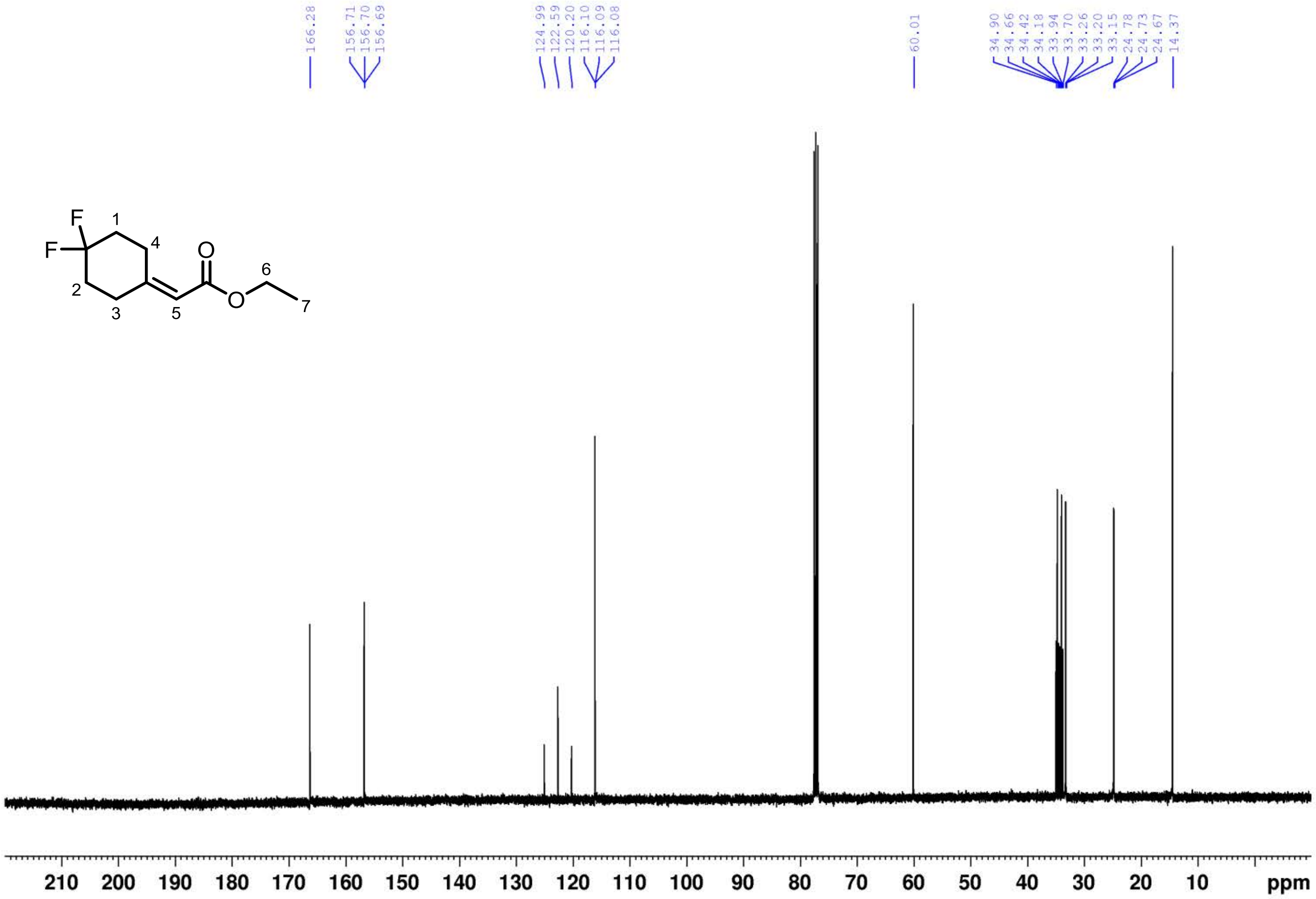

^{19}F NMR (376 MHz, CDCl$_3$) for *ethyl 2-(4,4-difluorocyclohexylidene)acetate*

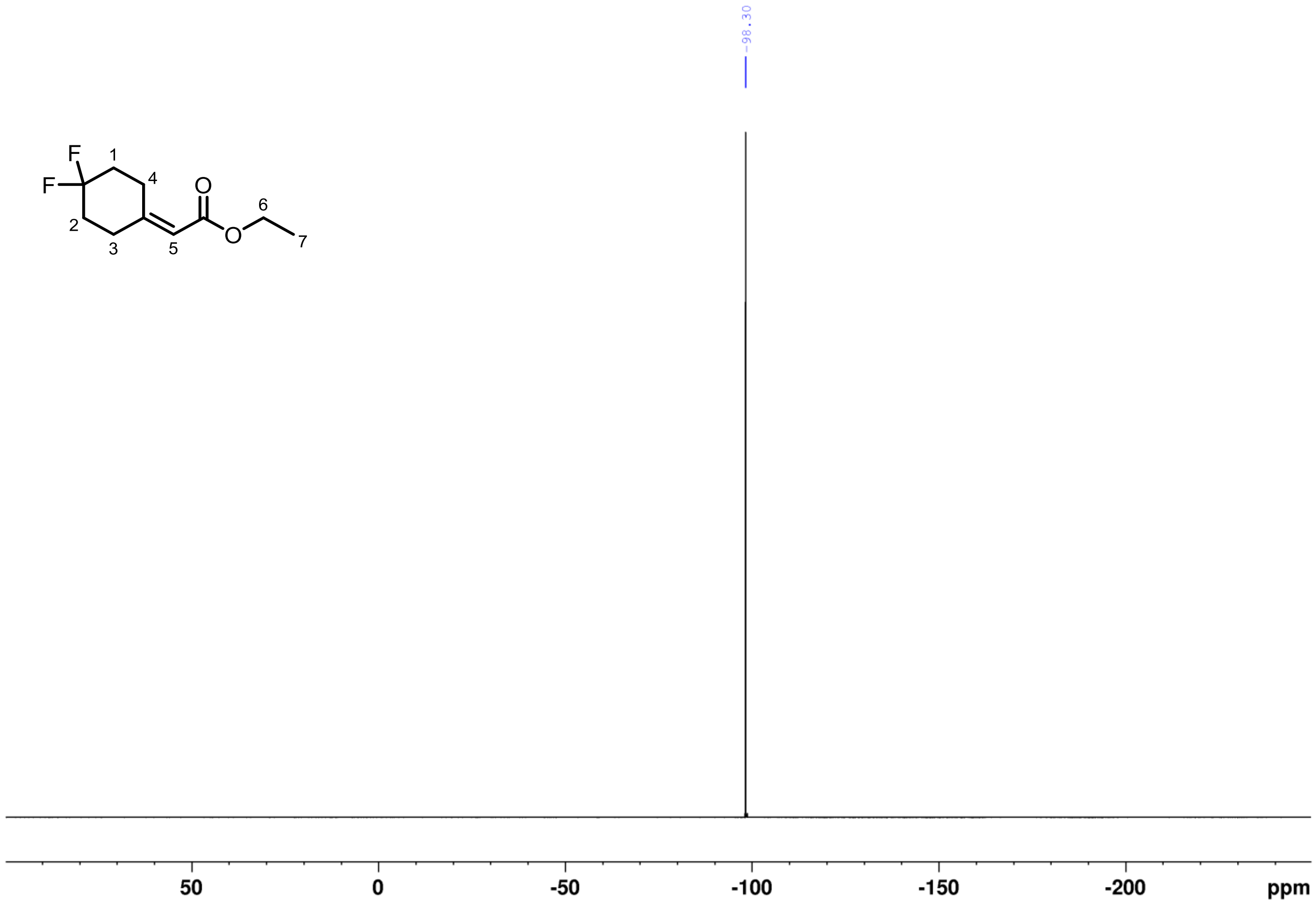

^{1}H NMR (400 MHz, CDCl$_3$) for *2-(4,4-difluorocyclohexylidene)ethan-1-ol* (**8e**)

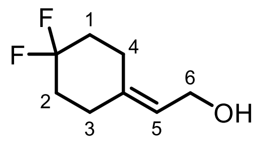

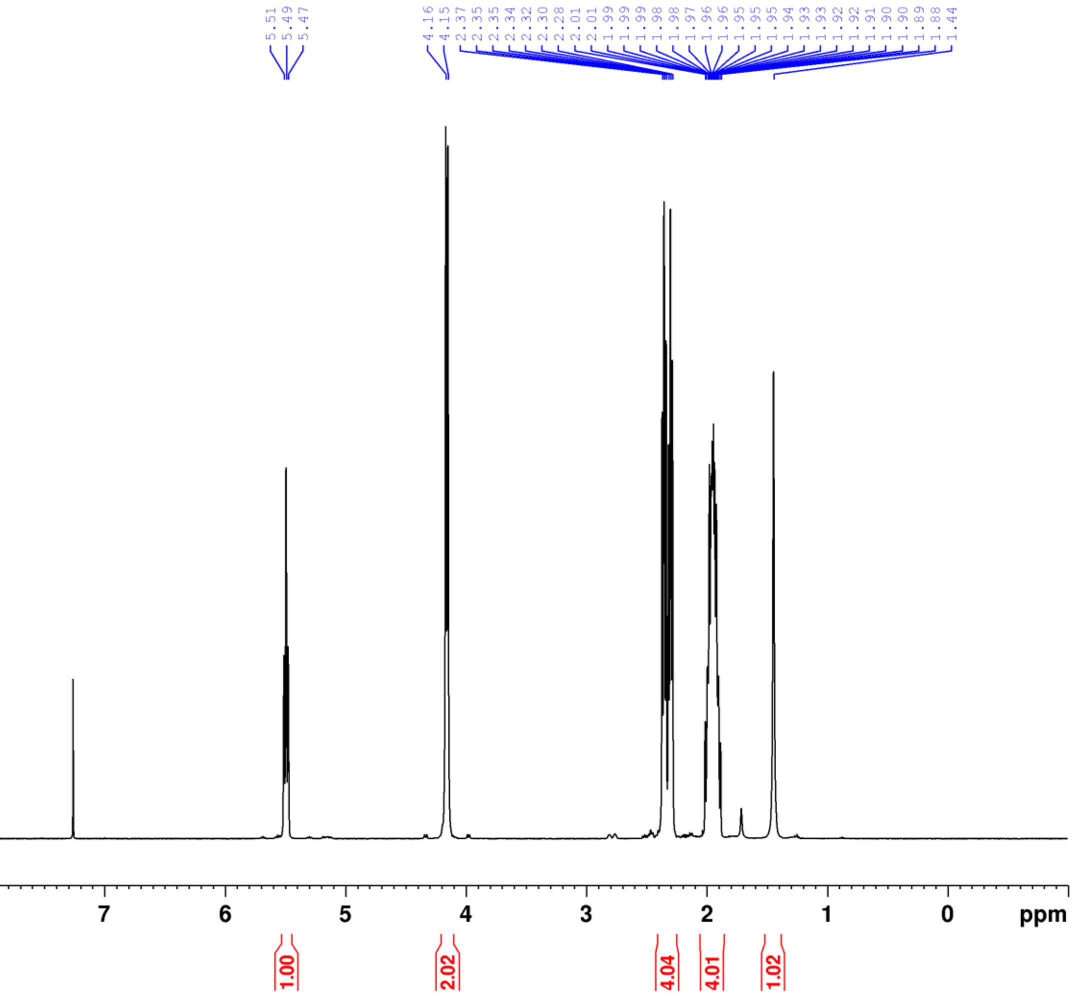

^{13}C NMR (101 MHz, $CDCl_3$) for *2-(4,4-difluorocyclohexylidene)ethan-1-ol* (**8e**)

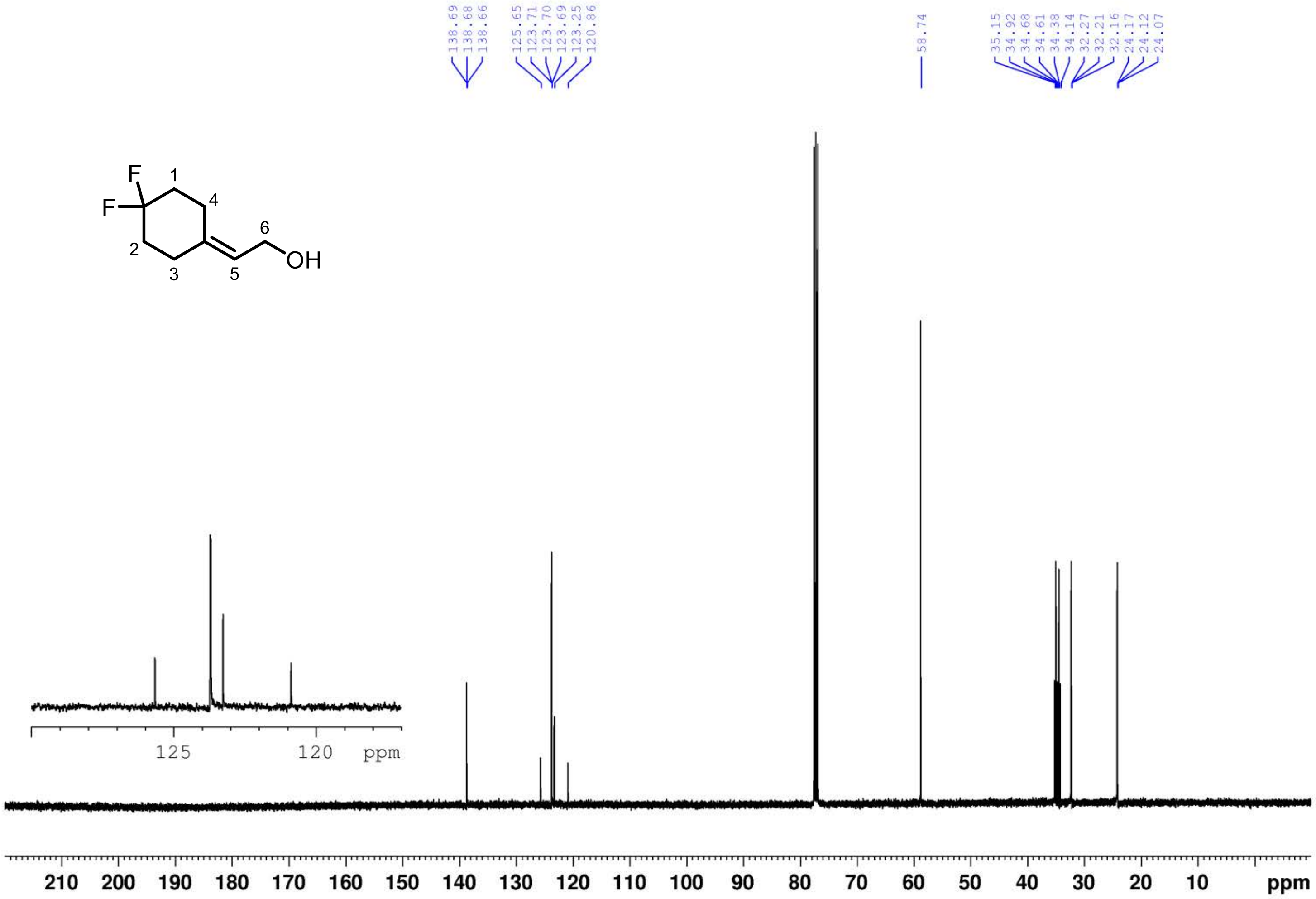

^{19}**F NMR (376 MHz, CDCl$_3$)** for *2-(4,4-difluorocyclohexylidene)ethan-1-ol* (**8e**)

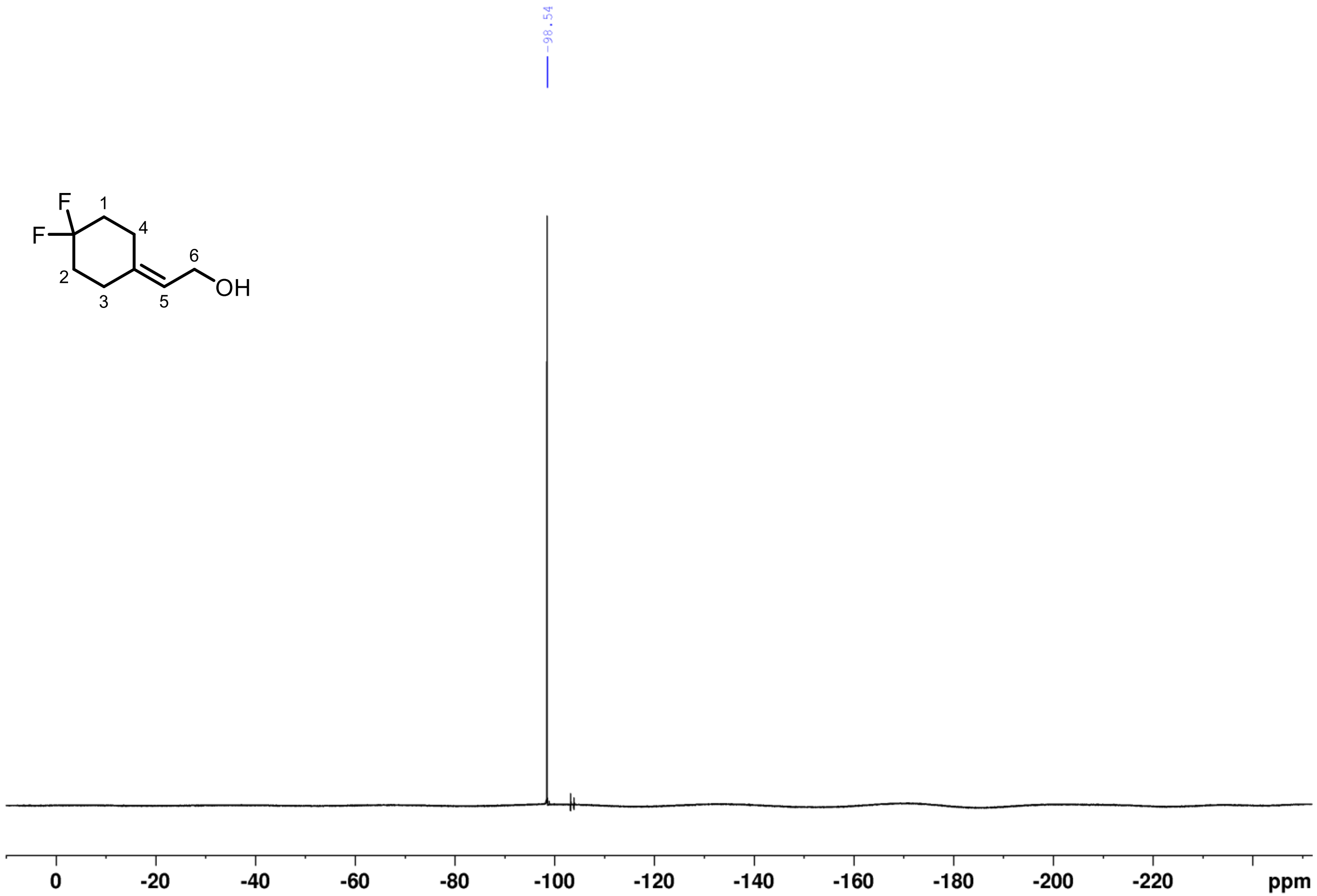

^{1}H NMR (400 MHz, CDCl$_3$) for *ethyl 2-(tetrahydro-4H-pyran-4-ylidene)acetate*

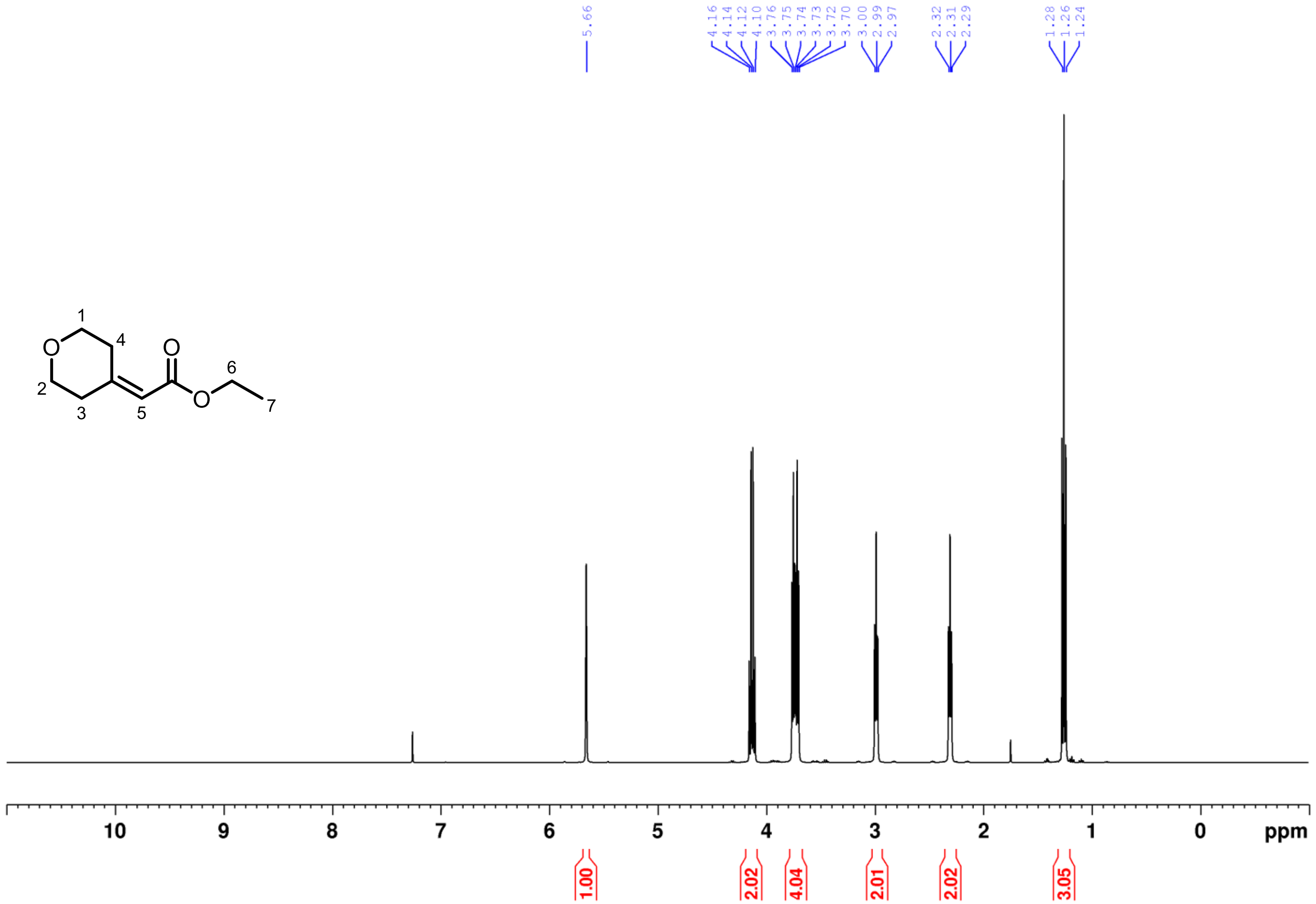

^{13}C NMR (101 MHz, CDCl$_3$) for *ethyl 2-(tetrahydro-4H-pyran-4-ylidene)acetate*

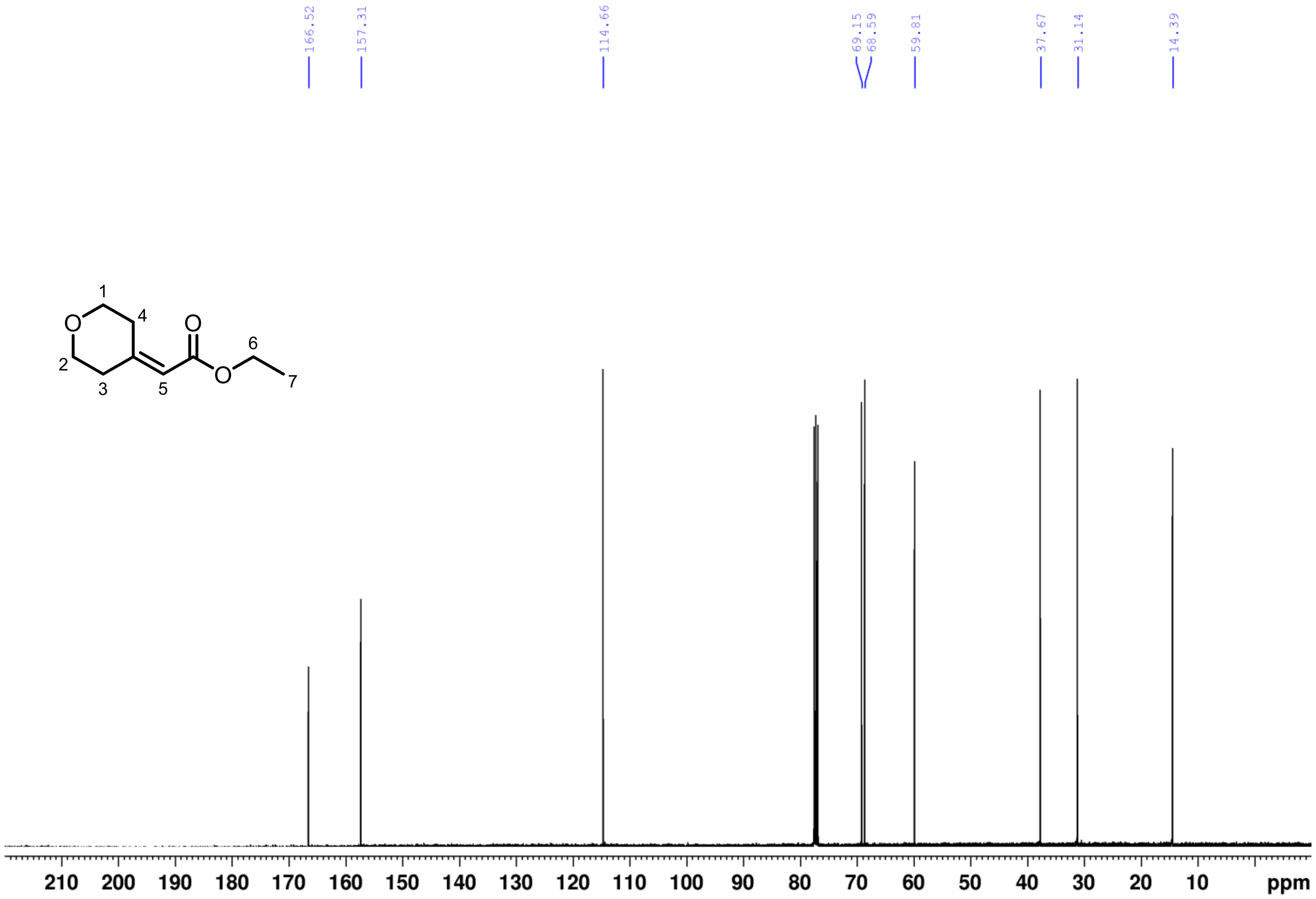

^{1}H NMR (700 MHz, CDCl$_3$) for *2-(tetrahydro-4H-pyran-4-ylidene)ethan-1-ol* (**8f**)

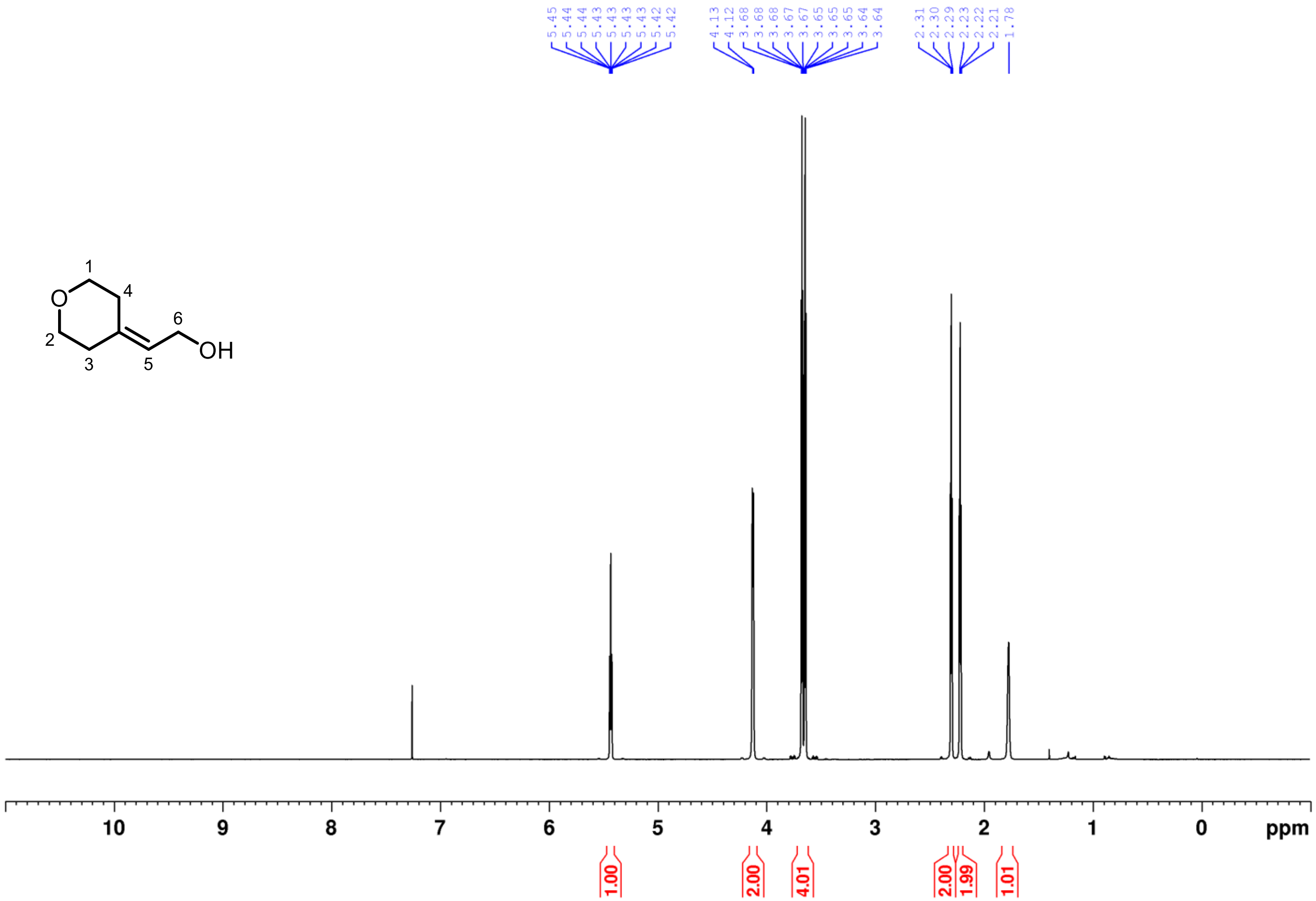

^{13}C NMR (176 MHz, $CDCl_3$) for *2-(tetrahydro-4H-pyran-4-ylidene)ethan-1-ol* (**8f**)

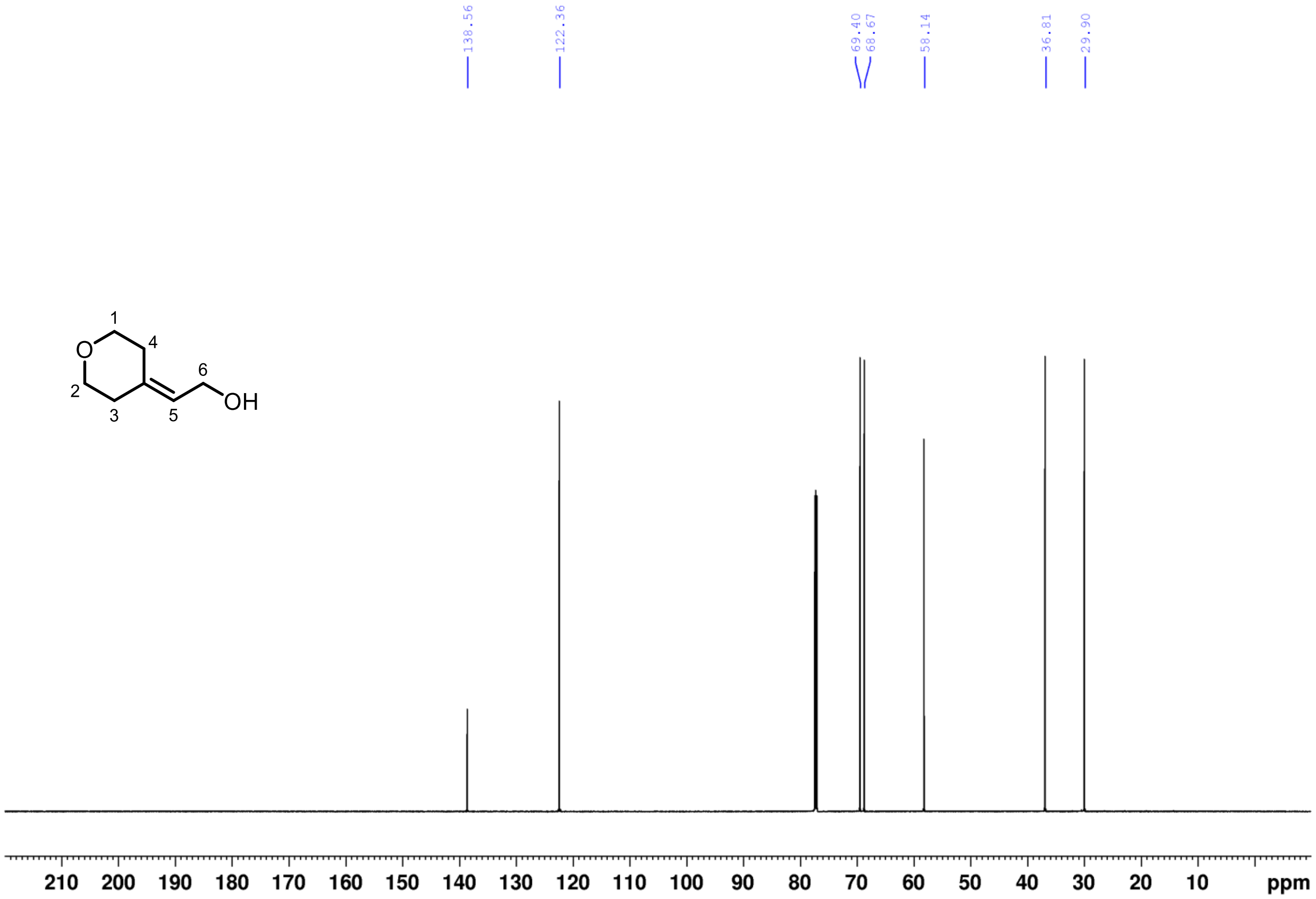

^{1}H NMR (400 MHz, $CDCl_3$) for *ethyl 2-(1,4-dioxaspiro[4.5]decan-8-ylidene)acetate*

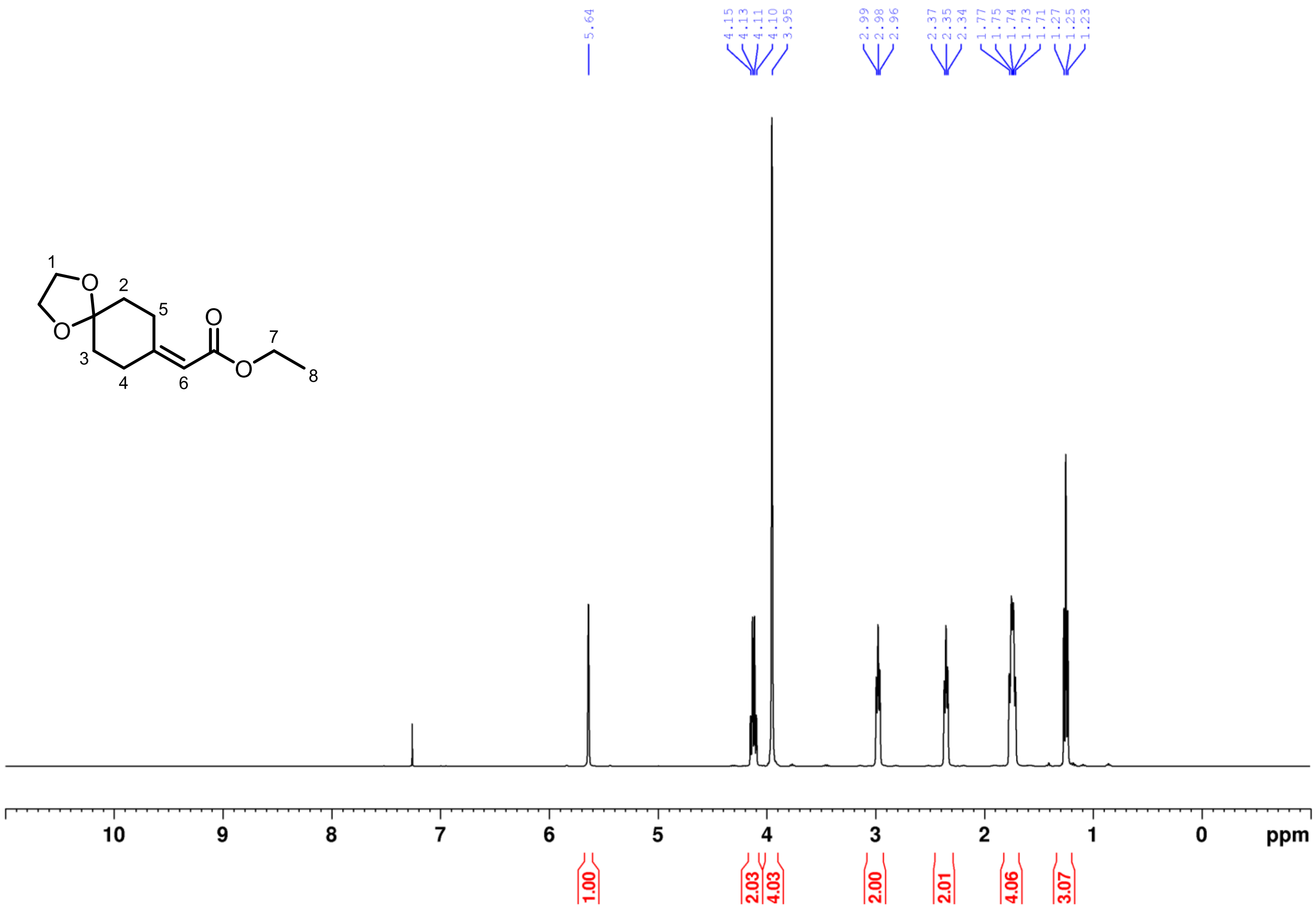

^{13}C NMR (101 MHz, CDCl$_3$) for *ethyl 2-(1,4-dioxaspiro[4.5]decan-8-ylidene)acetate*

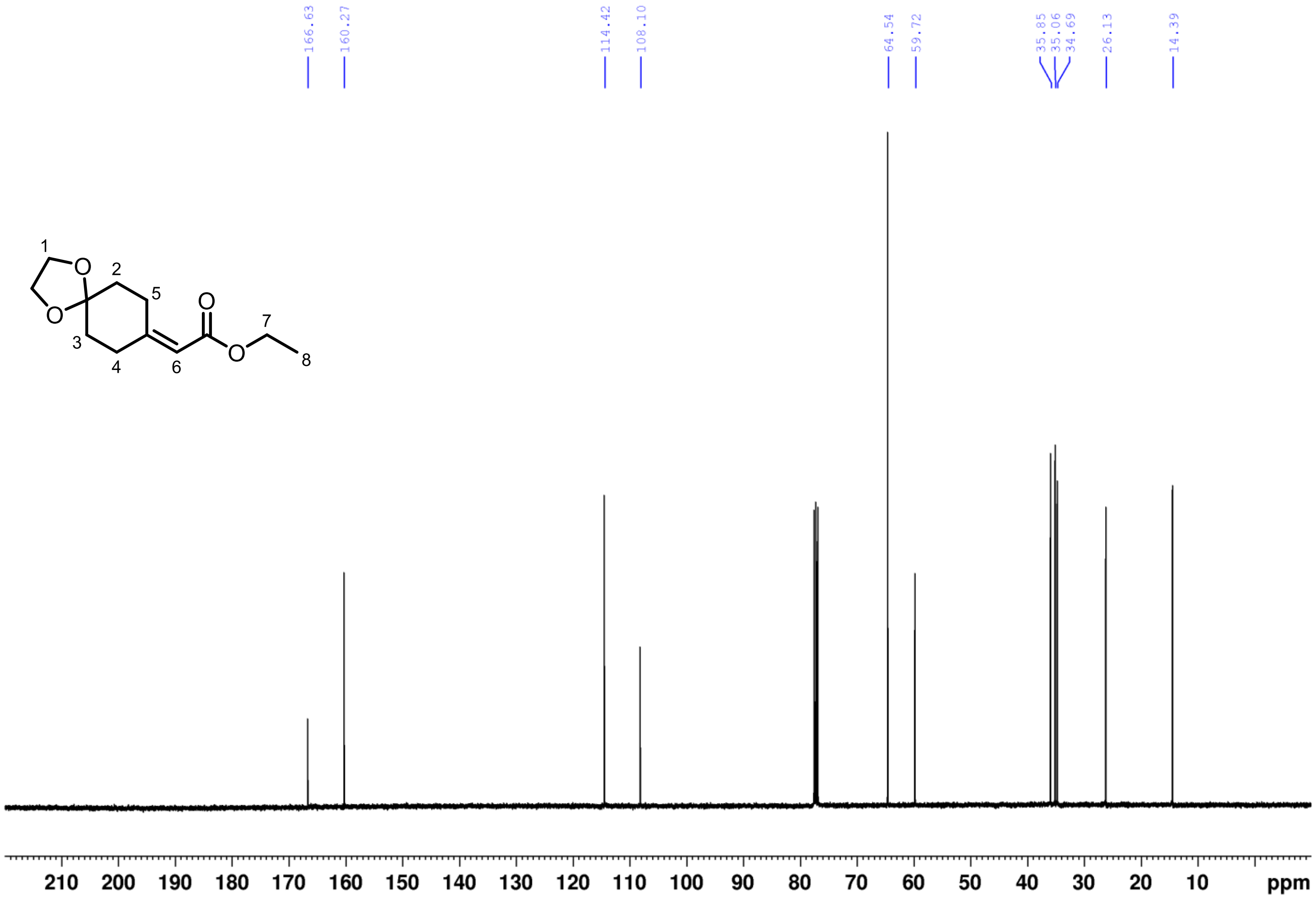

^{1}H NMR (400 MHz, $CDCl_3$) for *2-(1,4-dioxaspiro[4.5]decan-8-ylidene)ethan-1-ol* (**8g**)

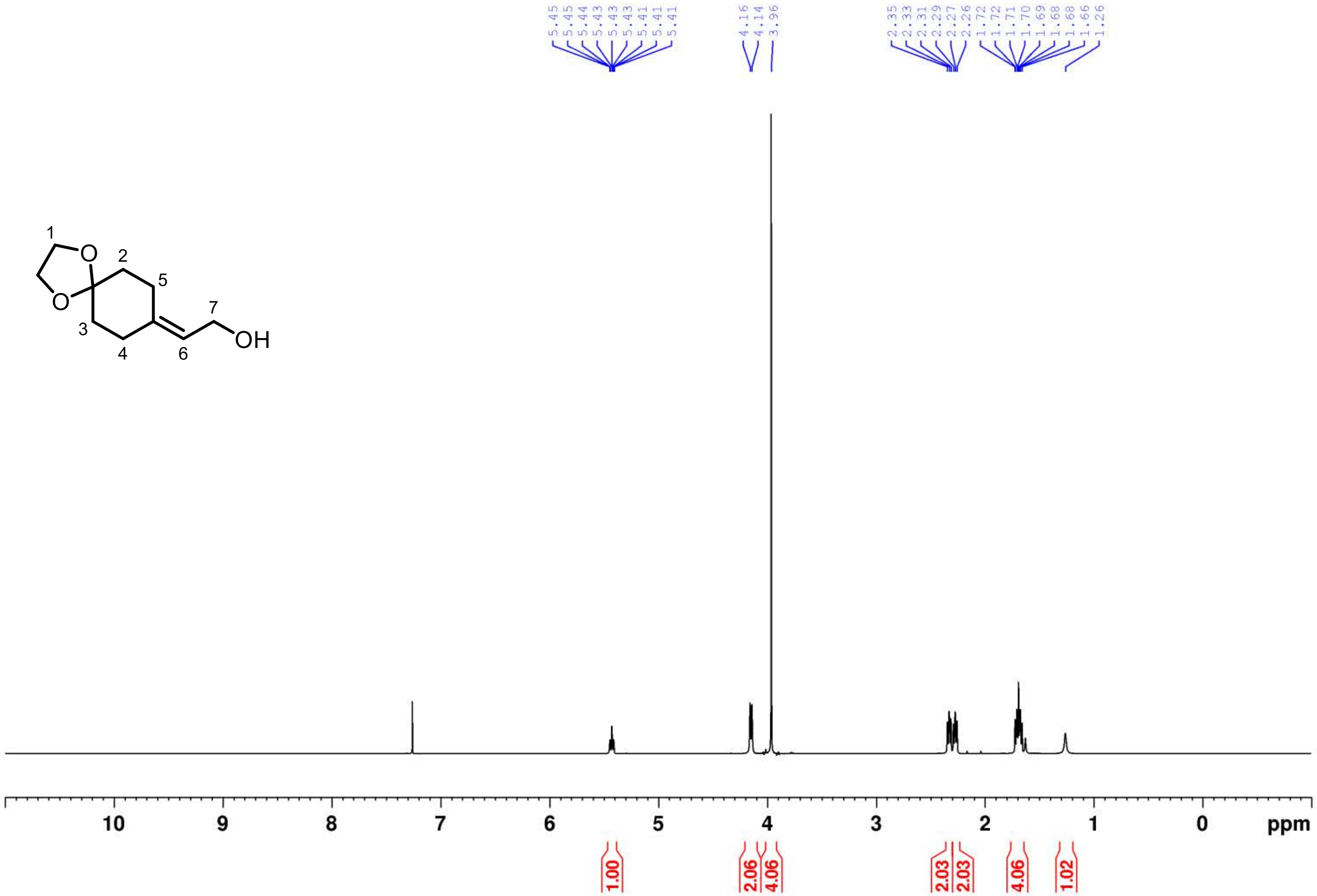

^{13}C NMR (101 MHz, CDCl$_3$) for *2-(1,4-dioxaspiro[4.5]decan-8-ylidene)ethan-1-ol* (**8g**)

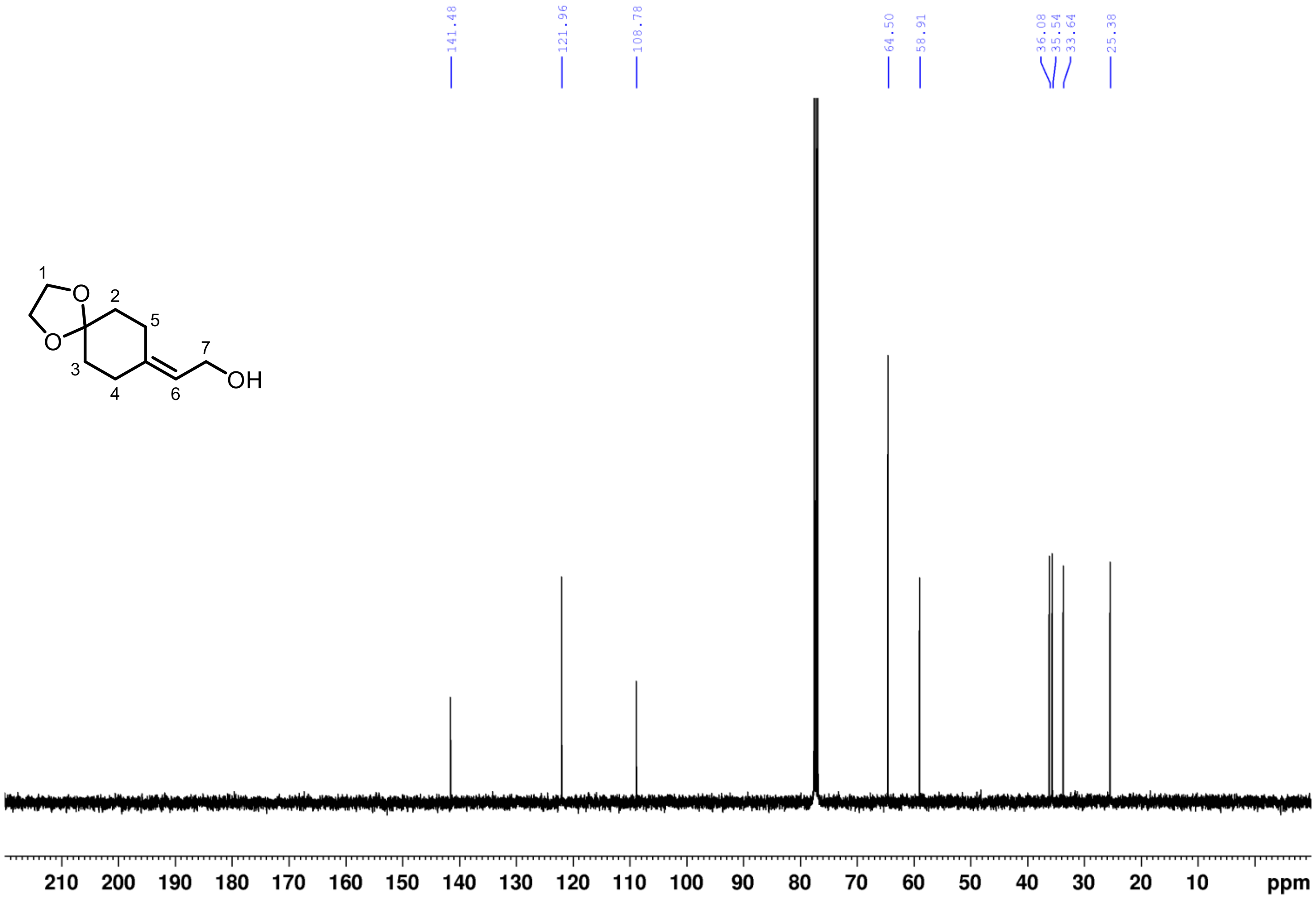

¹H NMR (400 MHz, CDCl₃) for *ethyl 2-cyclopentylideneacetate*. *Note: this compound contained 10% of the internal alkene isomer, presumably also formed in the Horner-Wadsworth-Emmons reaction. This isomer was partially separable at this stage but is fully separable in compound* **8i**.

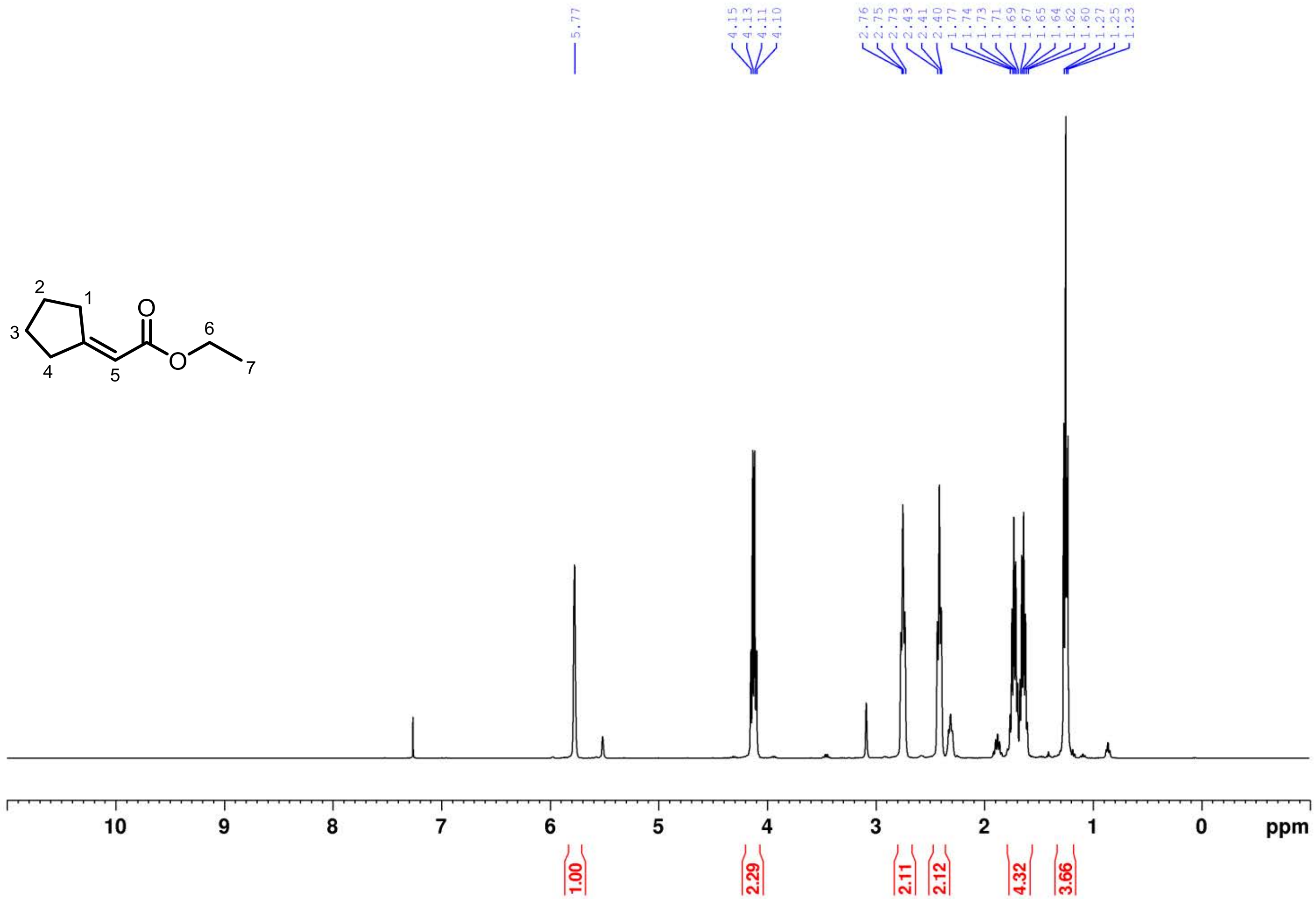

^{13}C NMR (101 MHz, CDCl$_3$) for *ethyl 2-cyclopentylideneacetate*. *Note: this compound contained 10% of the internal alkene isomer, presumably also formed in the Horner-Wadsworth-Emmons reaction. This isomer was partially separable at this stage but is fully separable in compound* **8i**.

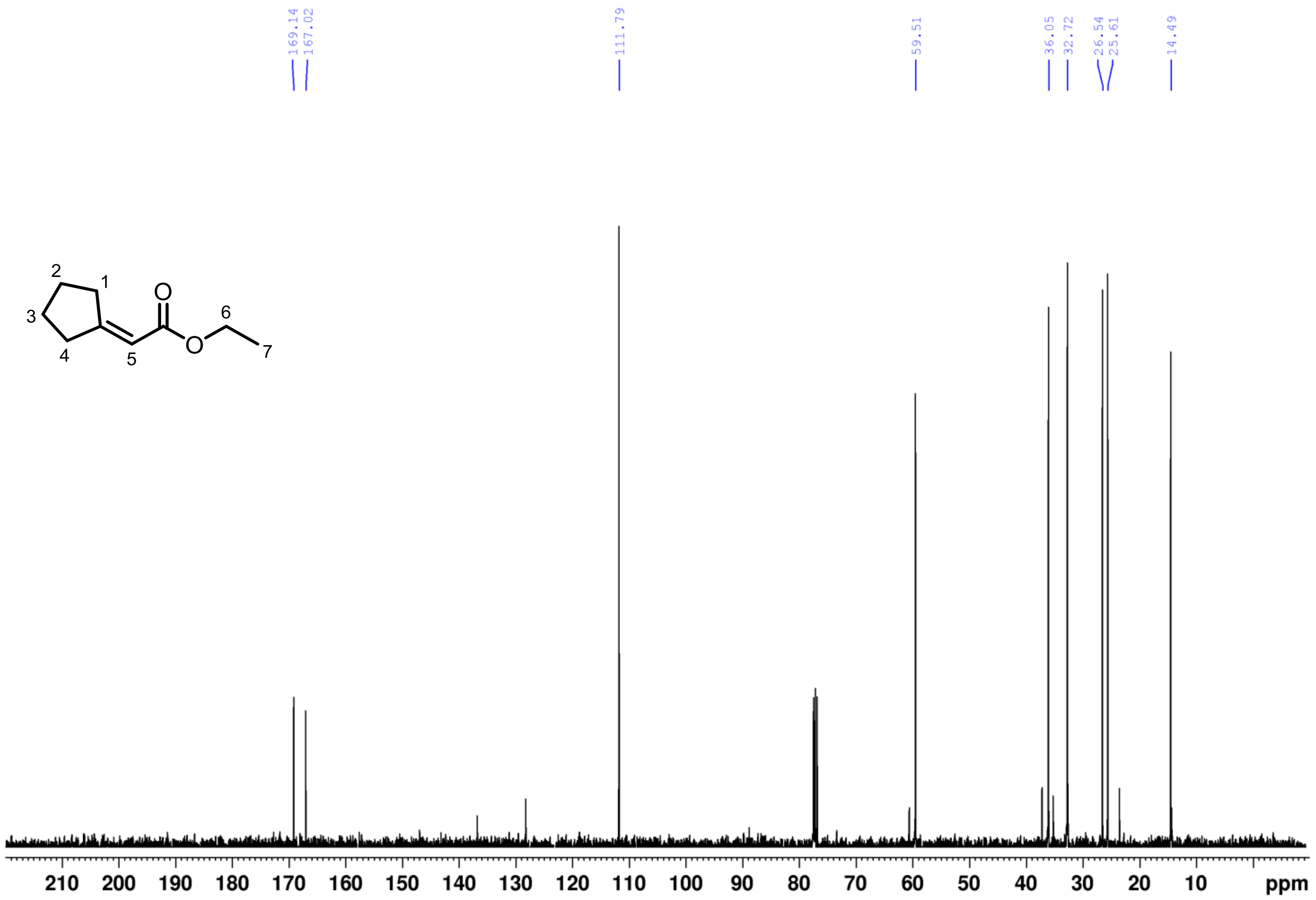

^{1}H NMR (700 MHz, CDCl$_3$) for *2-cyclopentylideneethan-1-ol* (**8i**)

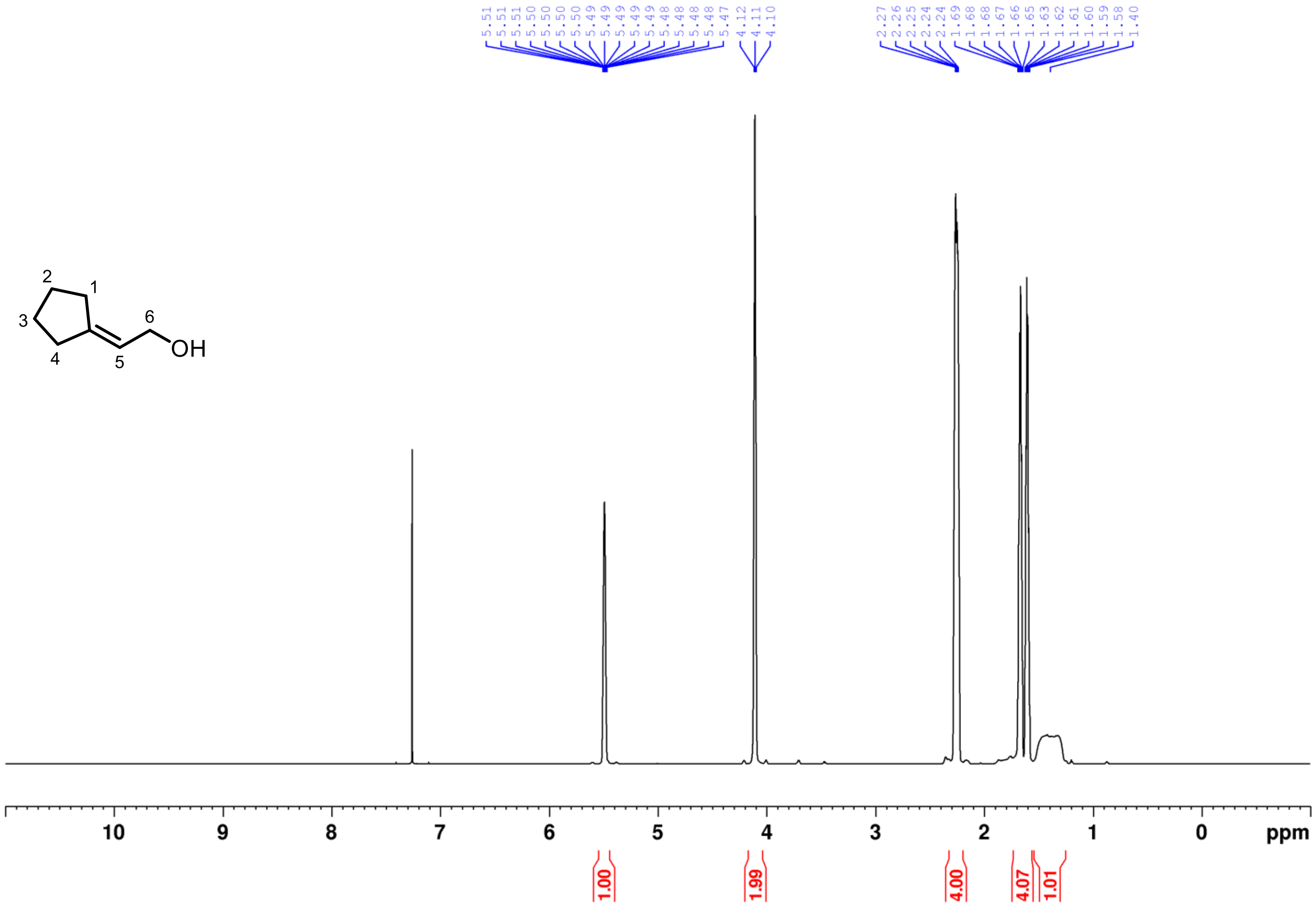

^{13}C NMR (176 MHz, $CDCl_3$) for *2-cyclopentylideneethan-1-ol* (**8i**)

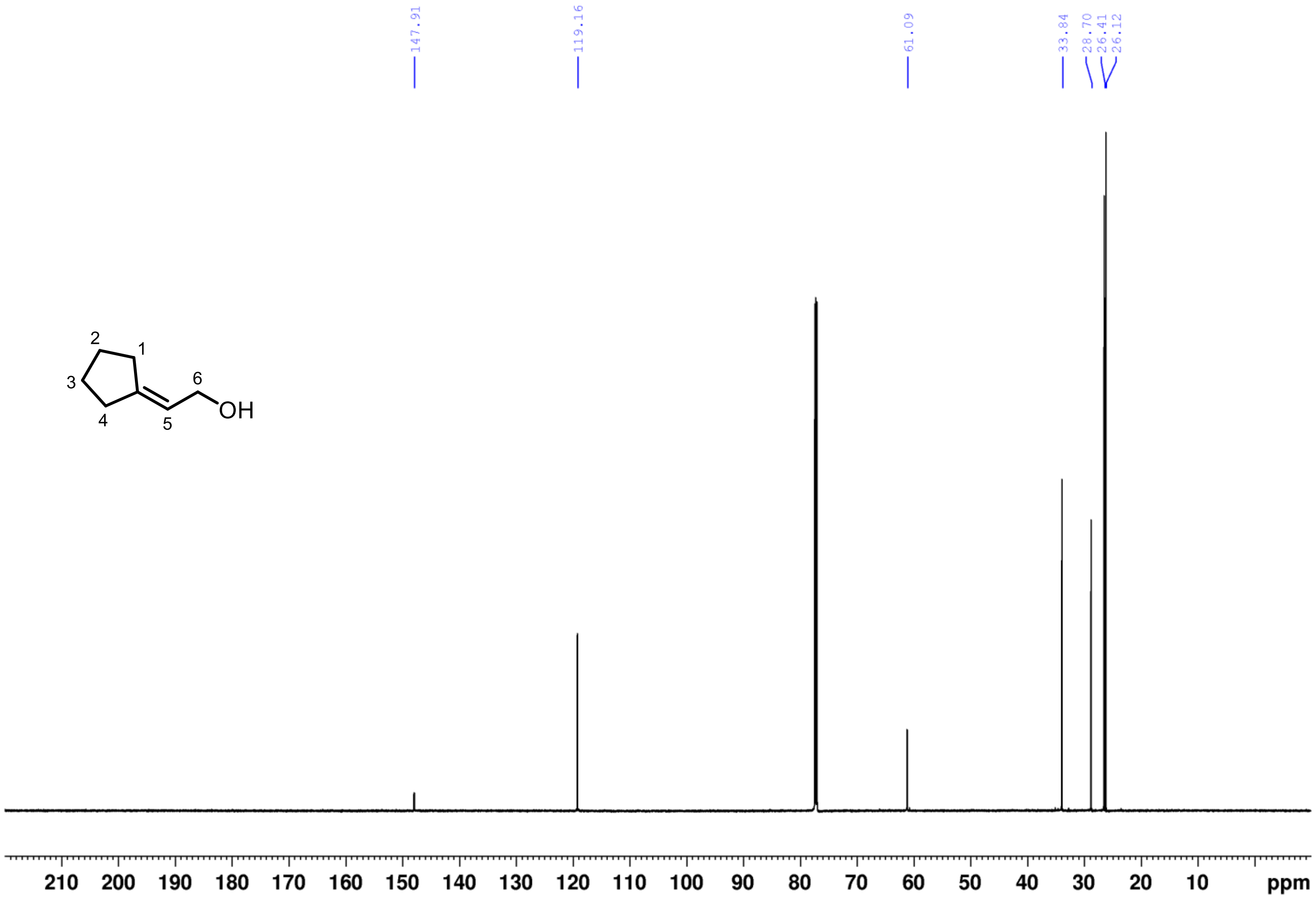

^{1}H NMR (400 MHz, CDCl$_3$) for *ethyl 2-cycloheptylideneacetate*

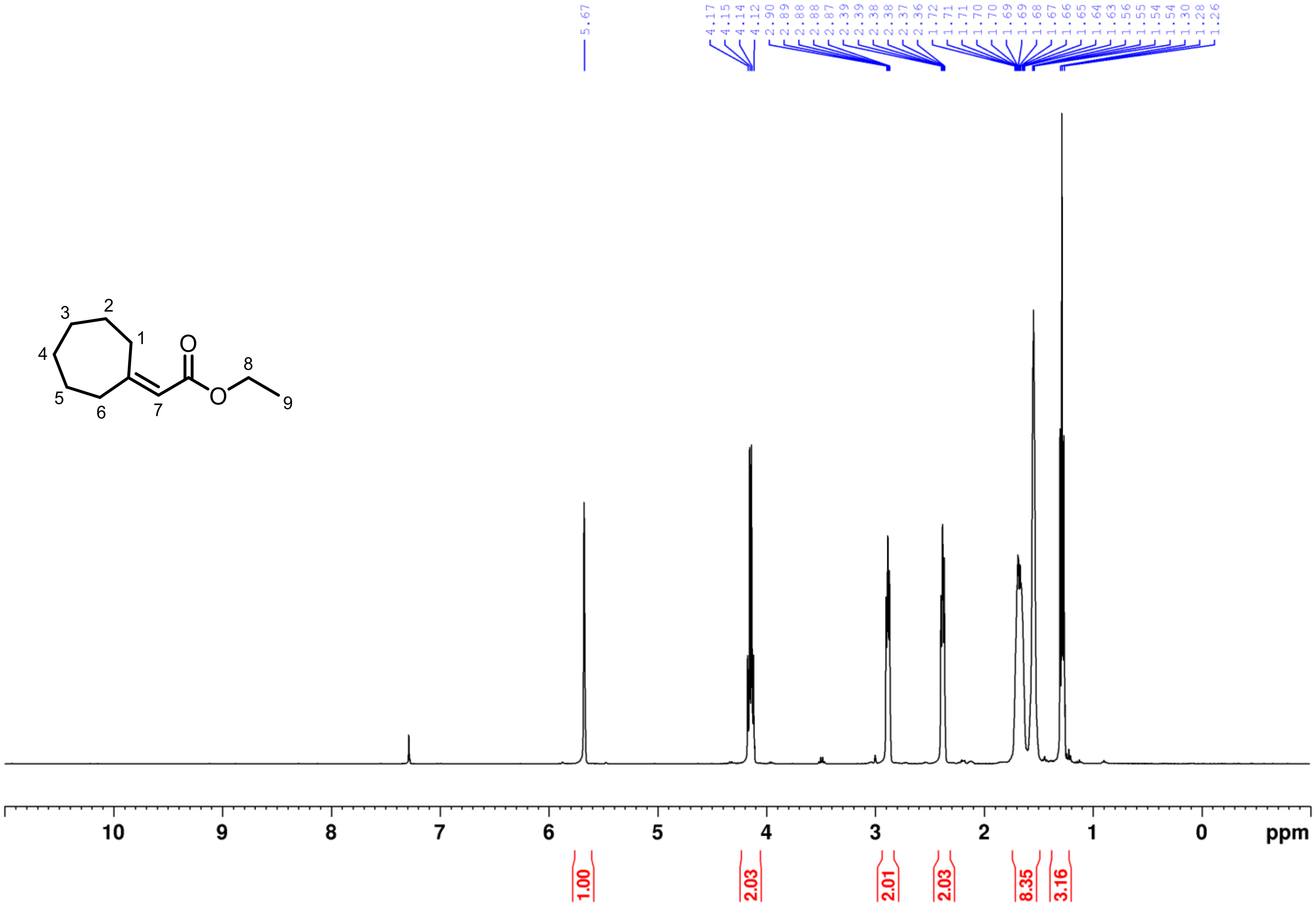

^{13}C NMR (101 MHz, CDCl$_3$) for *ethyl 2-cycloheptylideneacetate*

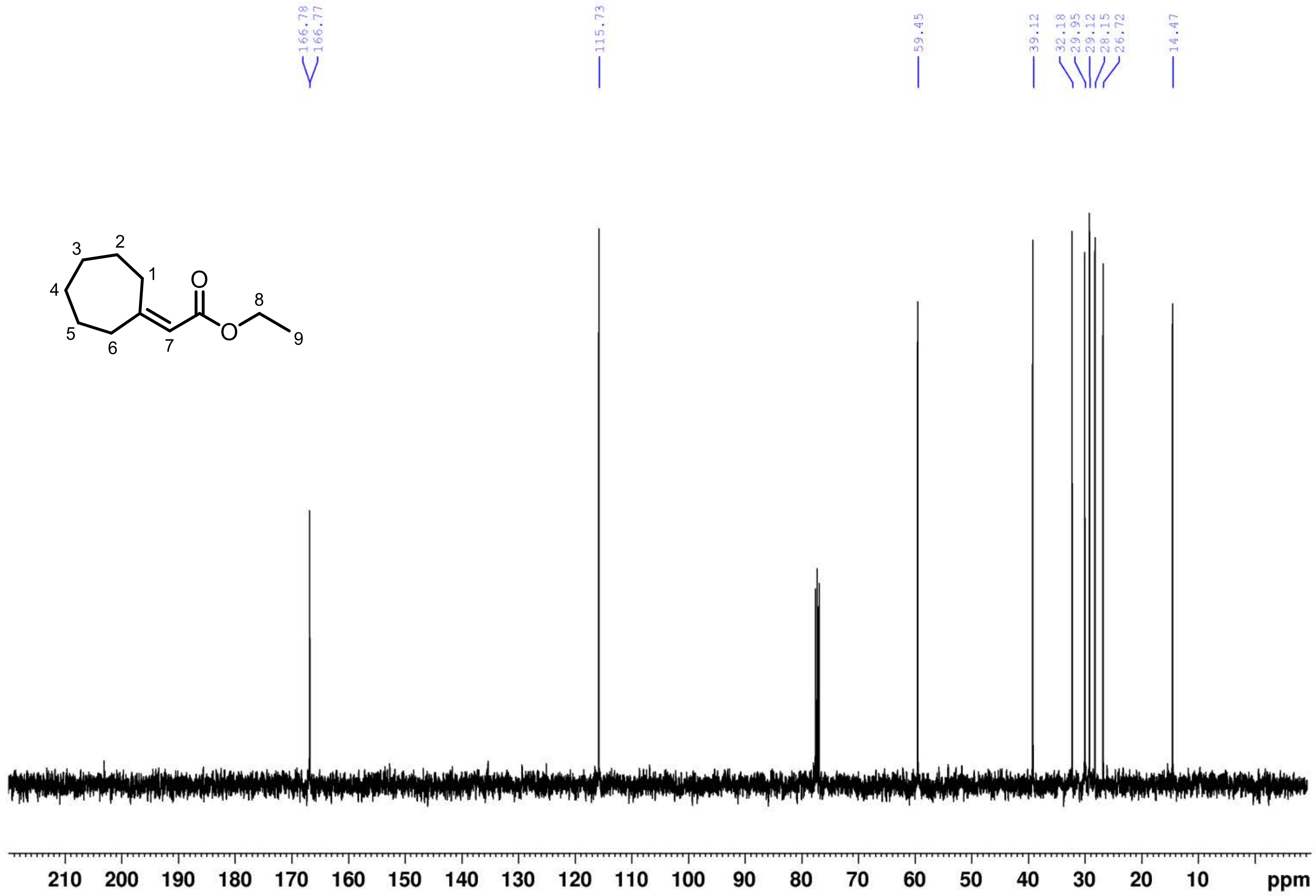

^{1}H NMR (400 MHz, CDCl$_3$) for *2-cycloheptylideneethan-1-ol* (**8j**)

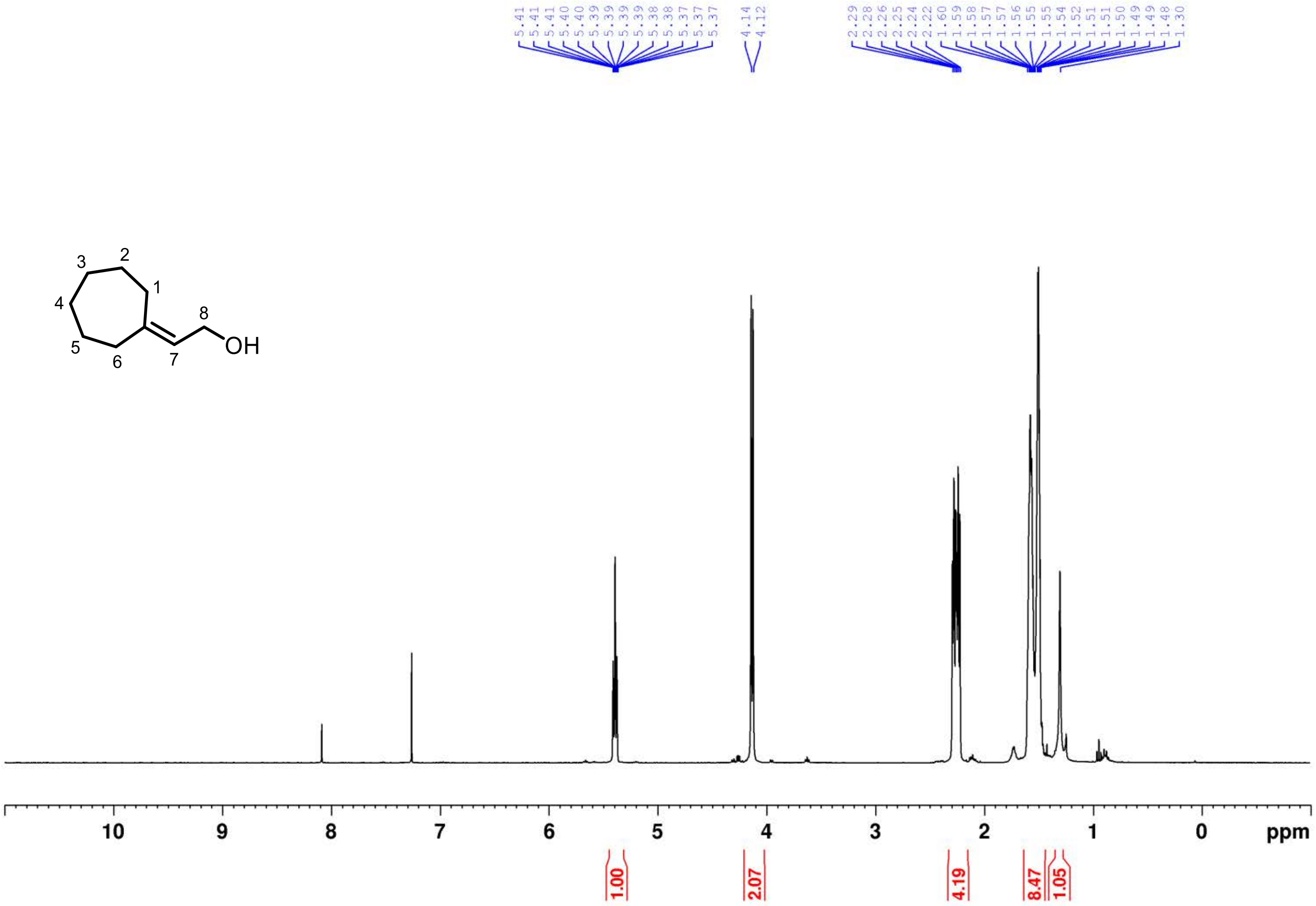

^{13}C NMR (101 MHz, $CDCl_3$) for *2-cycloheptylideneethan-1-ol* (**8j**)

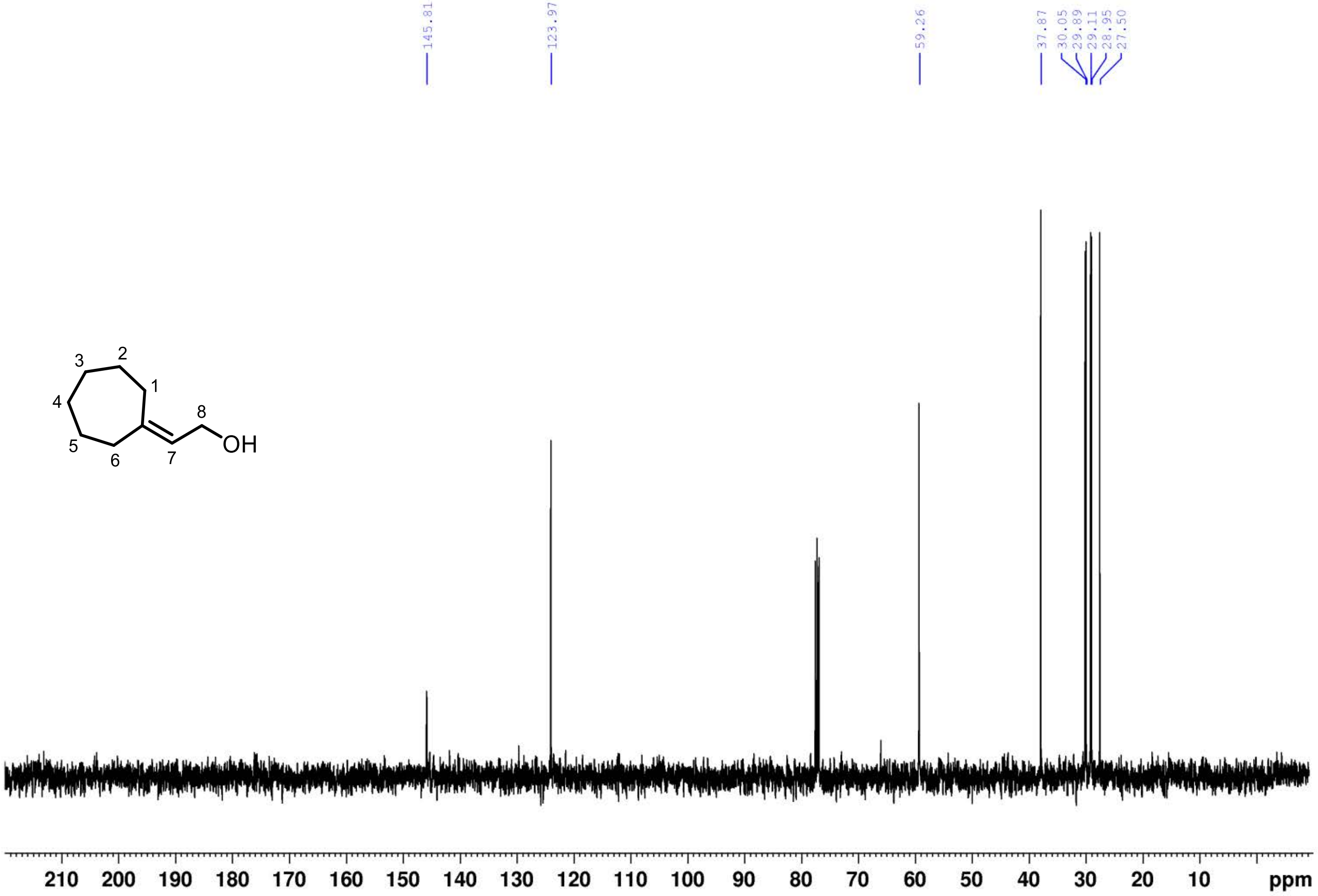

¹H NMR (700 MHz, $CDCl_3$) for *2,2,3,3,4,4,4-heptafluorobutyl (R)-(3-hydroxy-1-phenylpropyl)sulfamate* (**2a**)

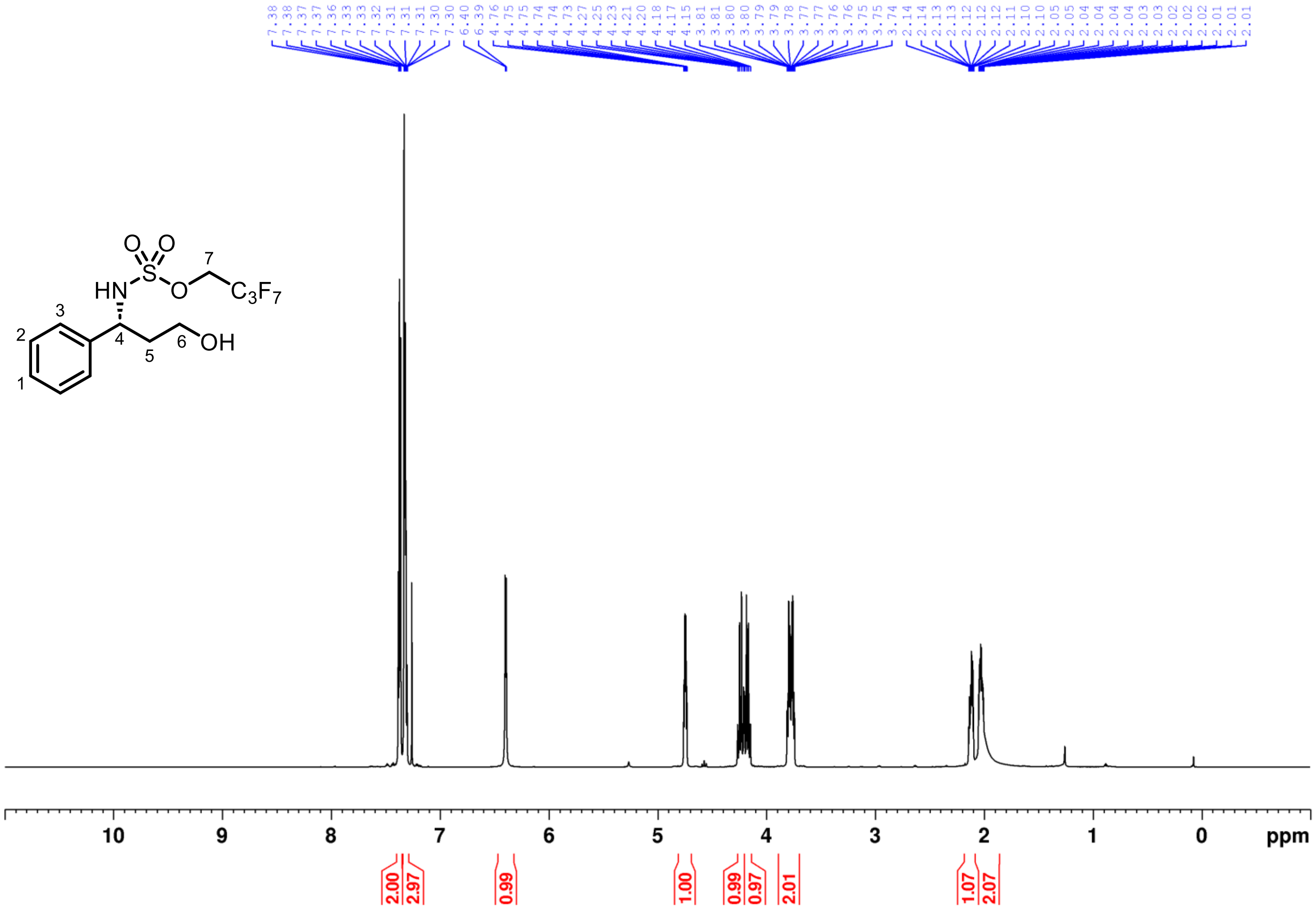

^{13}C NMR (176 MHz, CDCl$_3$) for *2,2,3,3,4,4,4-heptafluorobutyl (R)-(3-hydroxy-1-phenylpropyl)sulfamate* (**2a**)

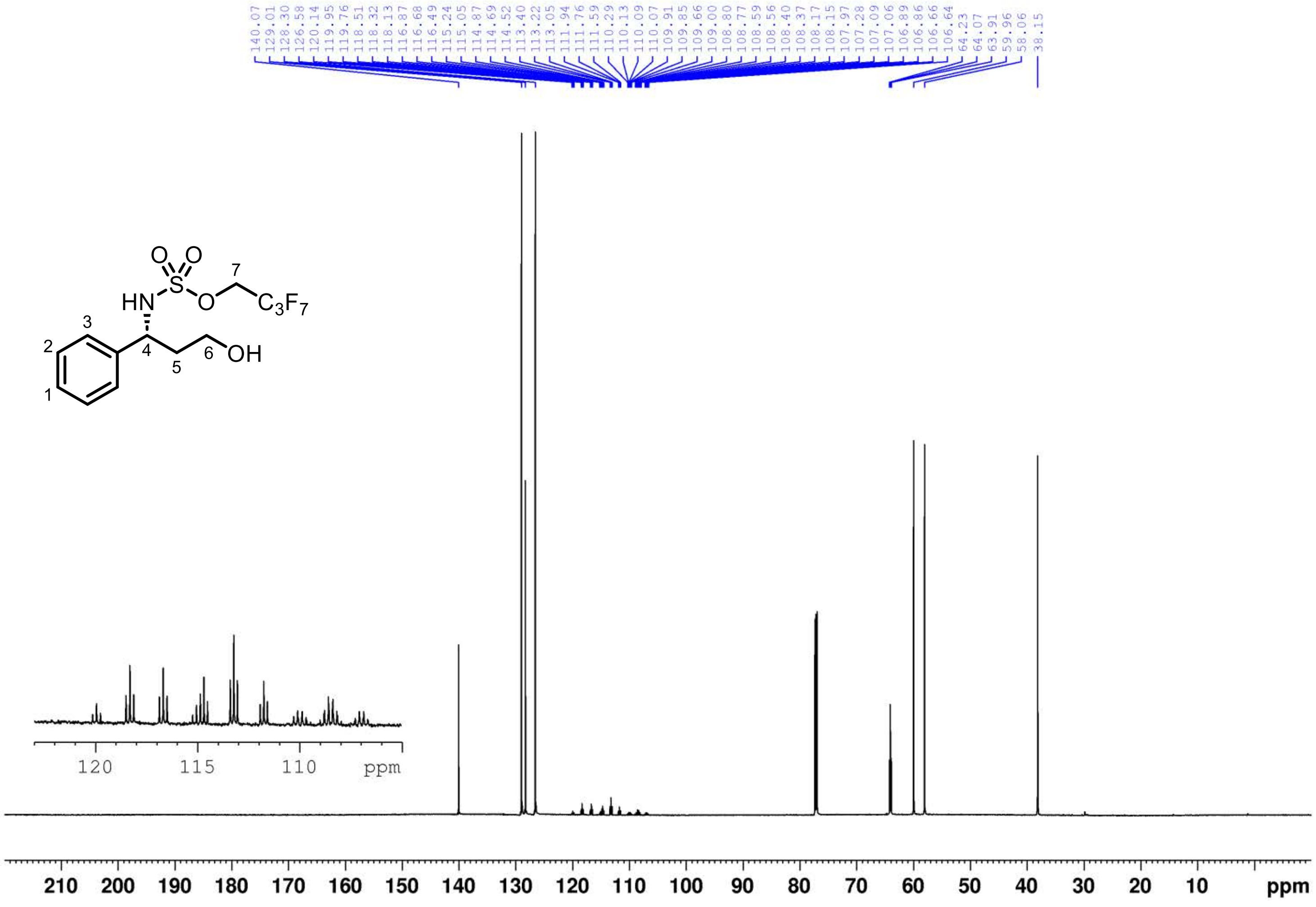

^{19}F NMR (376 MHz, CDCl$_3$) for *2,2,3,3,4,4,4-heptafluorobutyl (R)-(3-hydroxy-1-phenylpropyl)sulfamate* (**2a**)

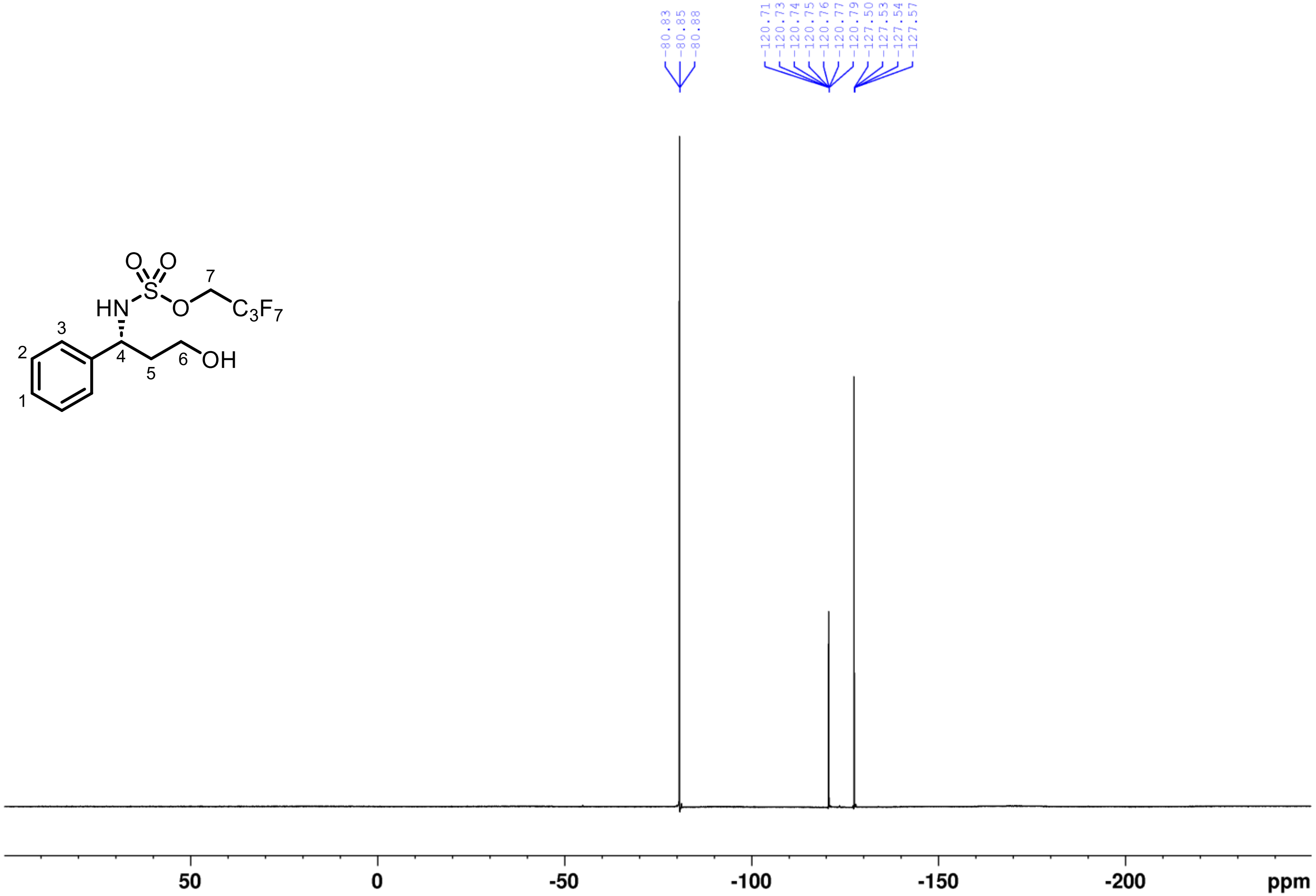

^{1}H NMR (700 MHz, CD_3OD) for *2,2,2-trichloroethyl (R)-(3-hydroxy-1-phenylpropyl)sulfamate* (**2b**)

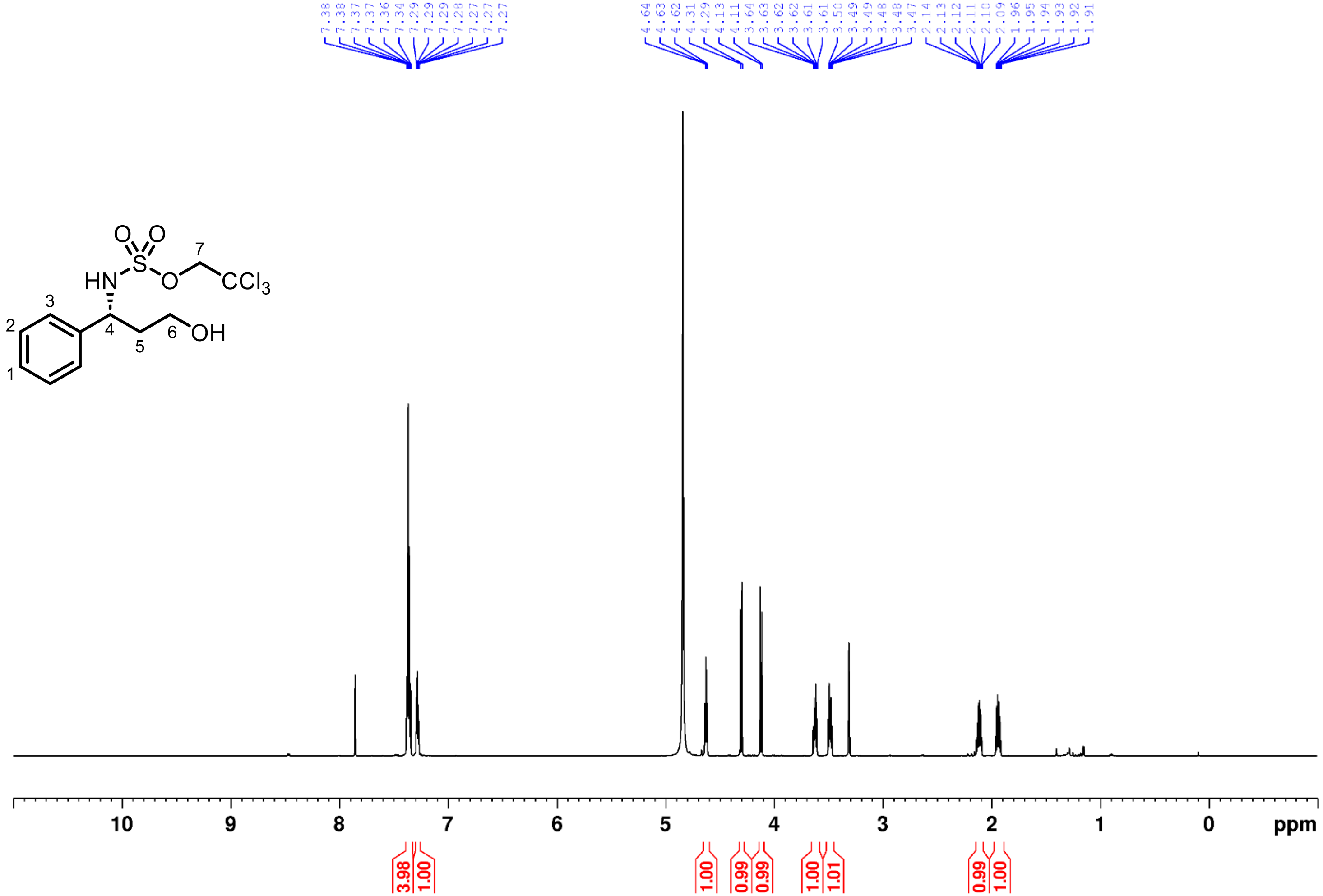

^{13}C NMR (176 MHz, CD$_3$OD) for *2,2,2-trichloroethyl (R)-(3-hydroxy-1-phenylpropyl)sulfamate* (**2b**)

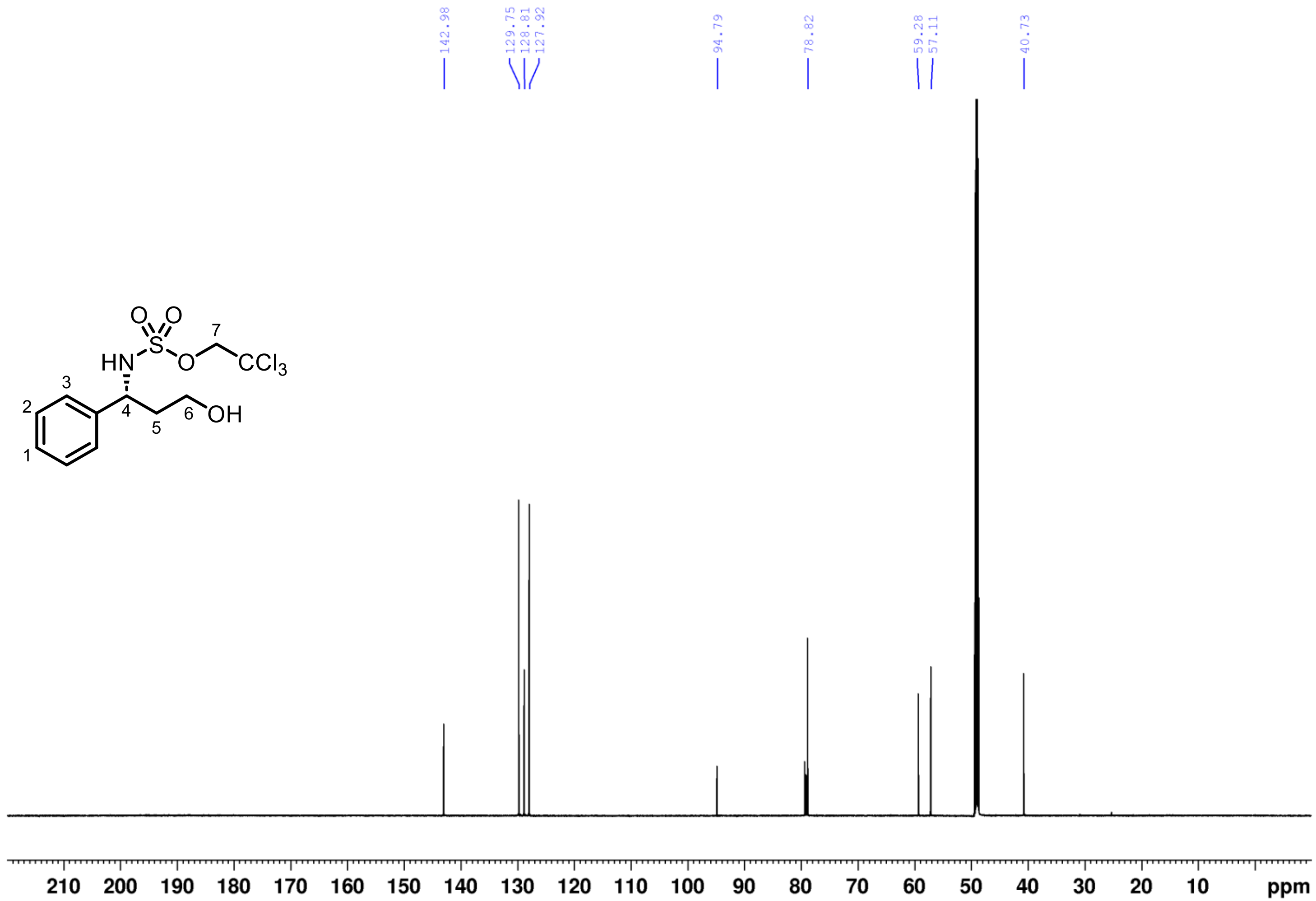

^{1}H NMR (700 MHz, $CDCl_3$) for *2,2,2-trichloroethyl (R)-(3-hydroxy-1-(o-tolyl)propyl)sulfamate* (**2c**)

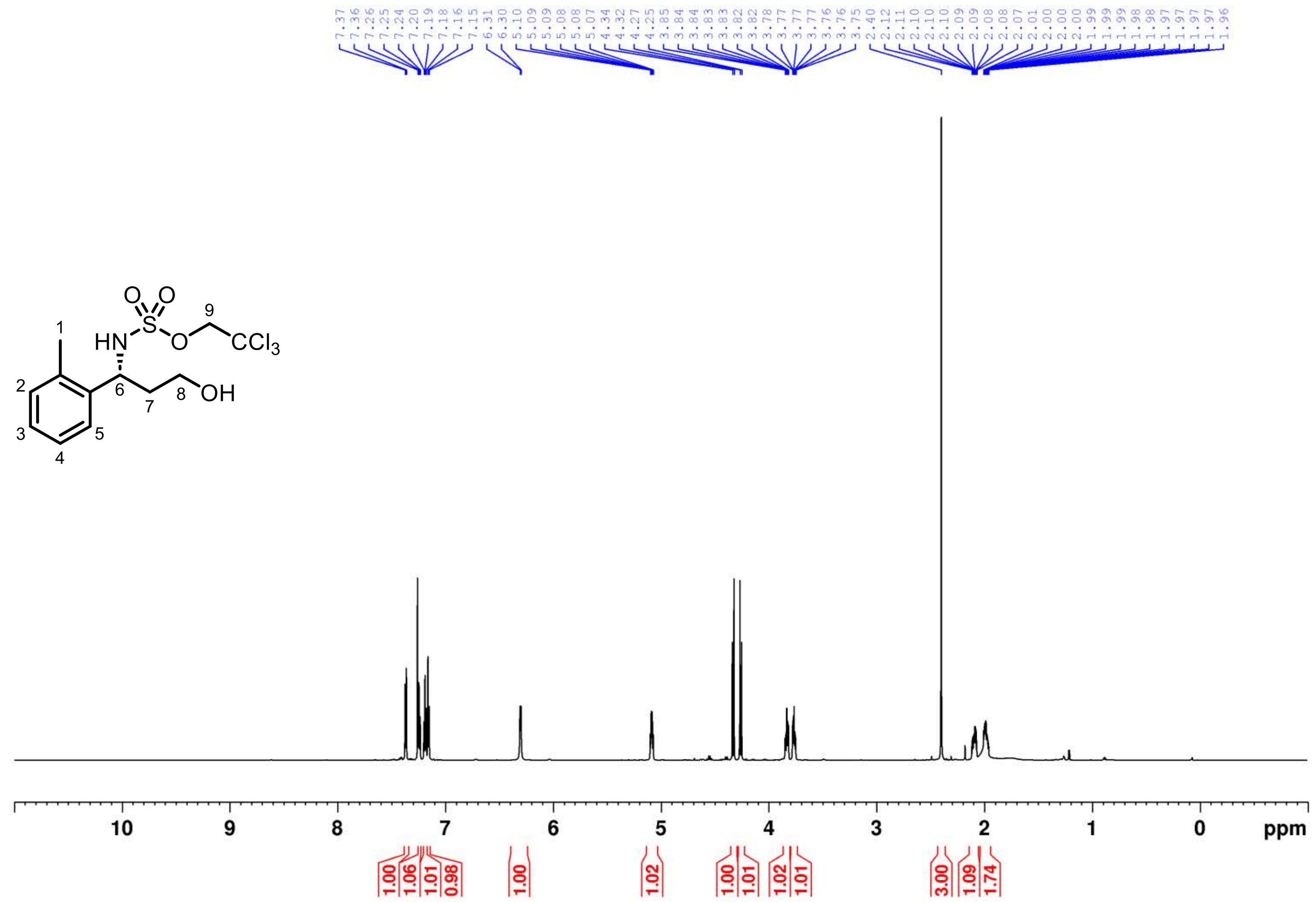

^{13}C NMR (176 MHz, $CDCl_3$) for *2,2,2-trichloroethyl (R)-(3-hydroxy-1-(o-tolyl)propyl)sulfamate* (**2c**)

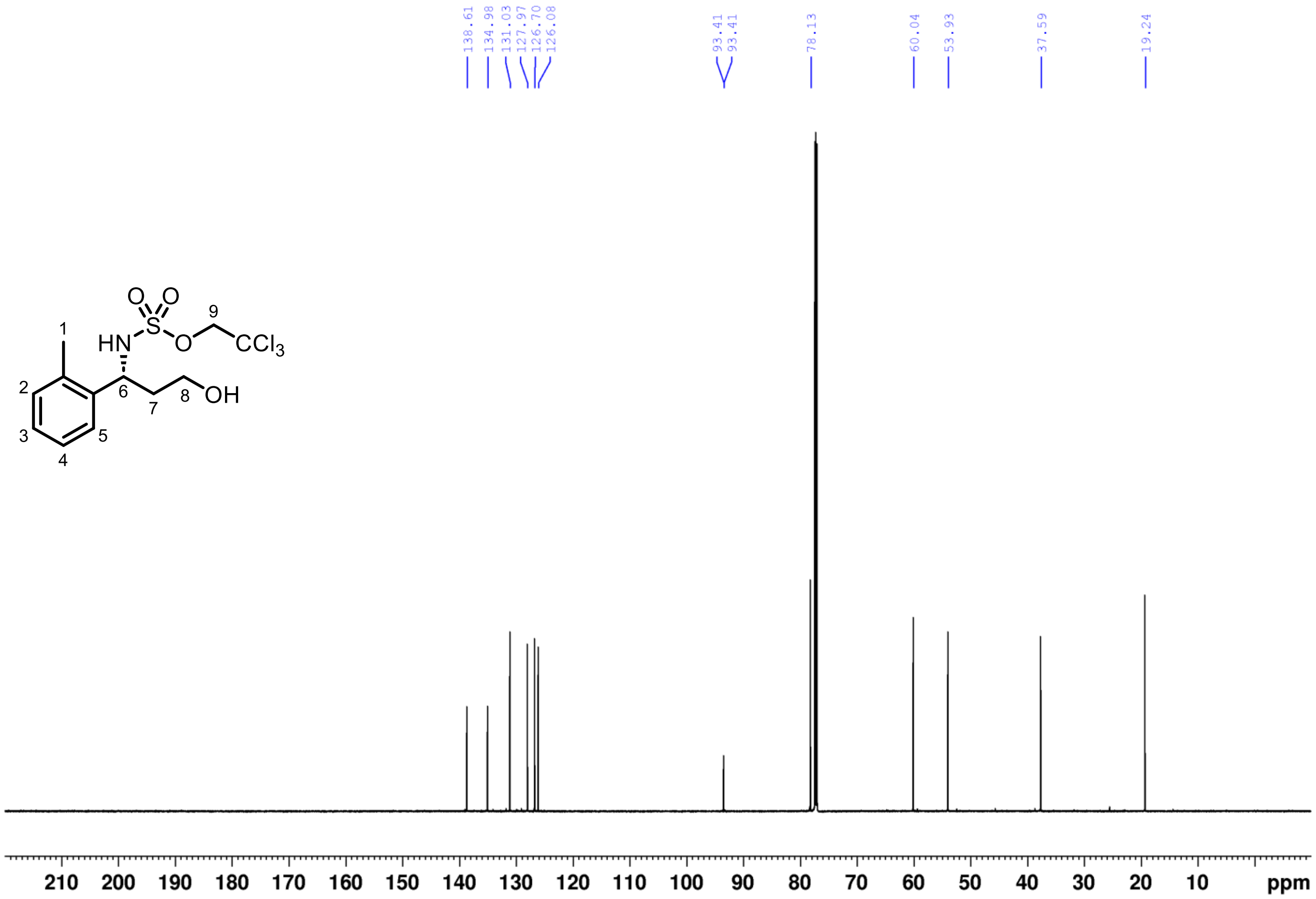

^{1}H NMR (400 MHz, $CDCl_3$) for *2,2,2-trichloroethyl (R)-(3-hydroxy-1-(2-methoxyphenyl)propyl)sulfamate* (**2d**)

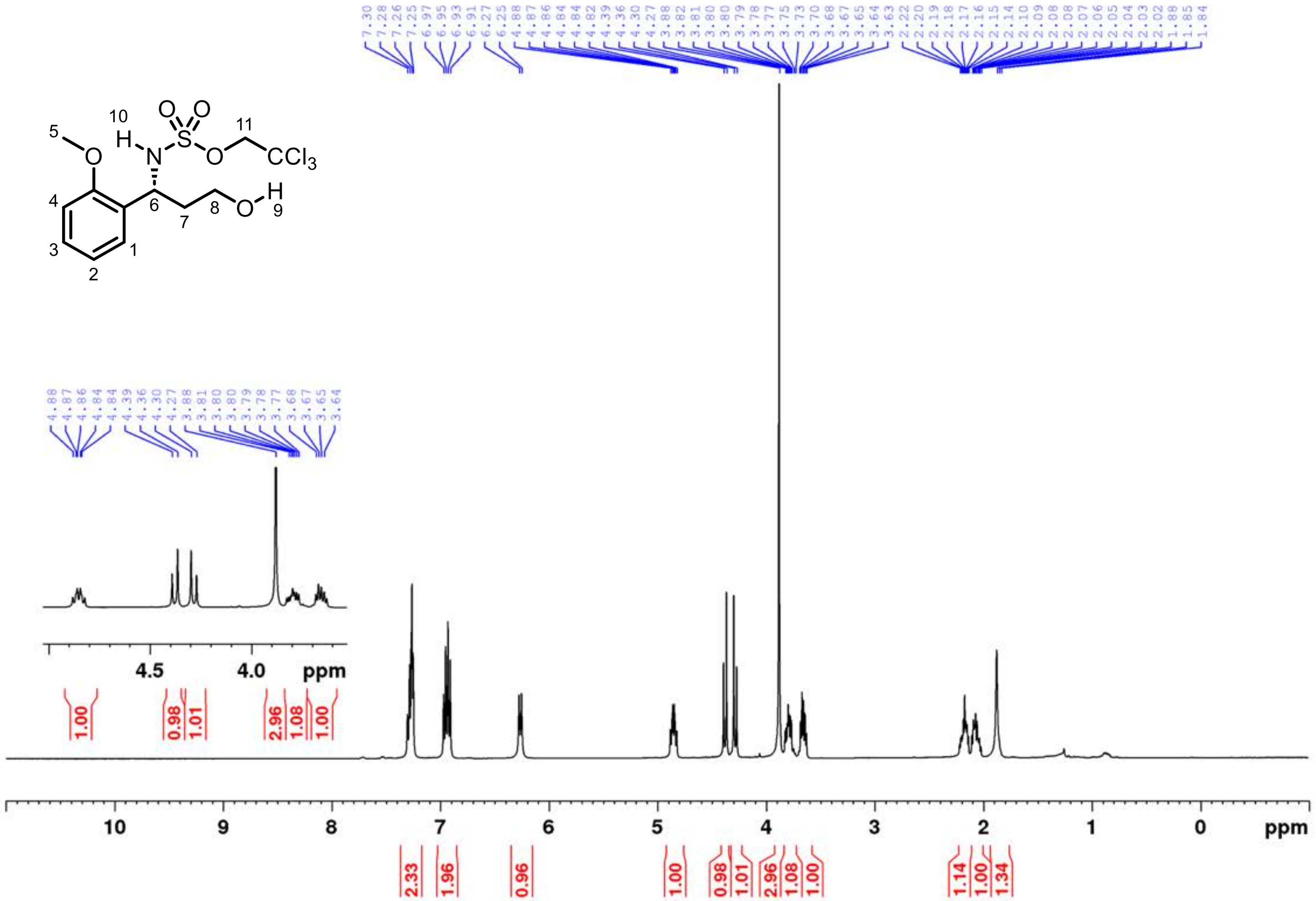

^{13}C NMR (101 MHz, CDCl$_3$) for *2,2,2-trichloroethyl (R)-(3-hydroxy-1-(2-methoxyphenyl)propyl)sulfamate* (**2d**)

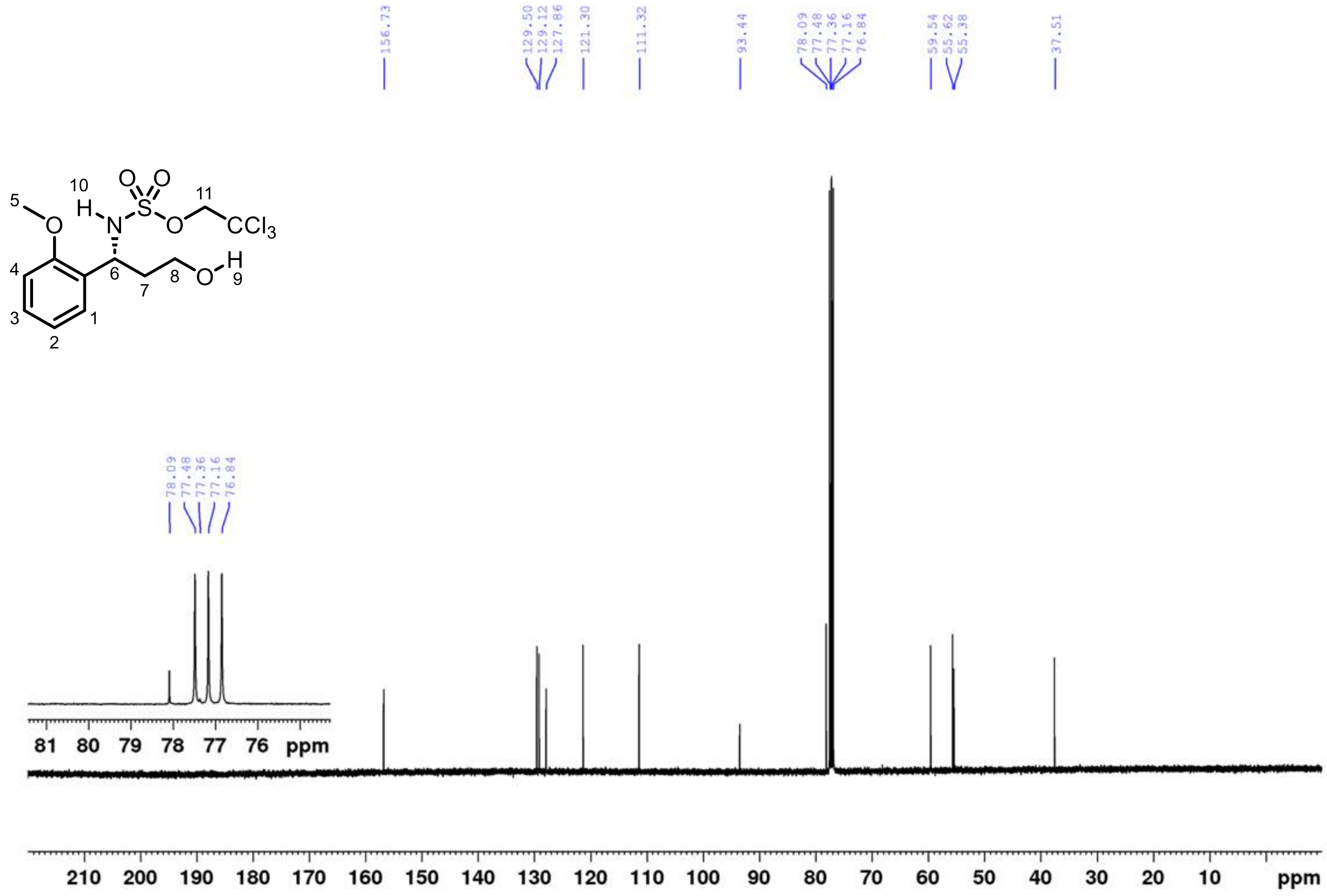

^{1}H NMR (700 MHz, $CDCl_3$) for *2,2,2-trichloroethyl (R)-(1-(2-chlorophenyl)-3-hydroxypropyl)sulfamate* (**2e**)

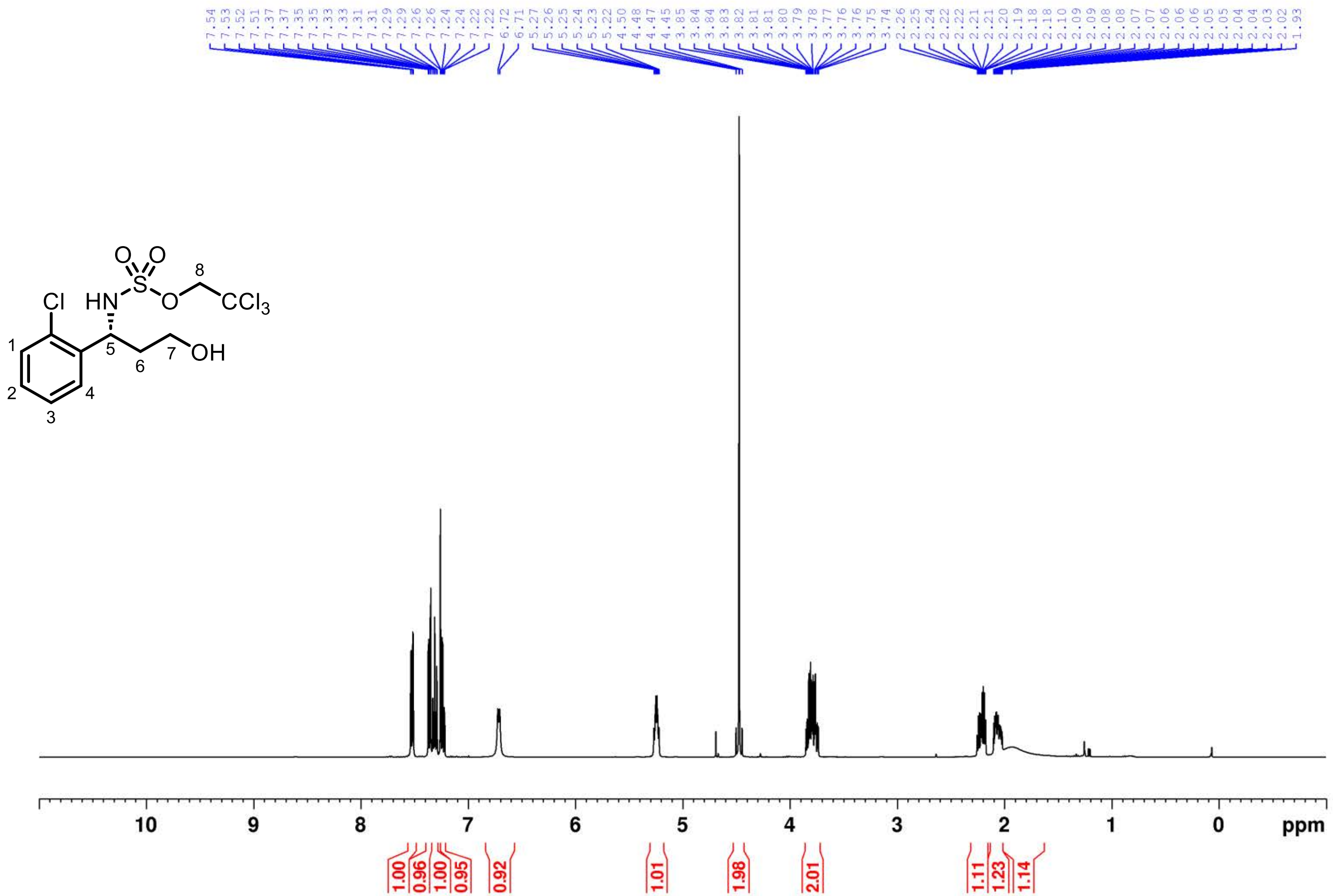

^{13}C NMR (176 MHz, $CDCl_3$) for *2,2,2-trichloroethyl (R)-(1-(2-chlorophenyl)-3-hydroxypropyl)sulfamate* (**2e**)

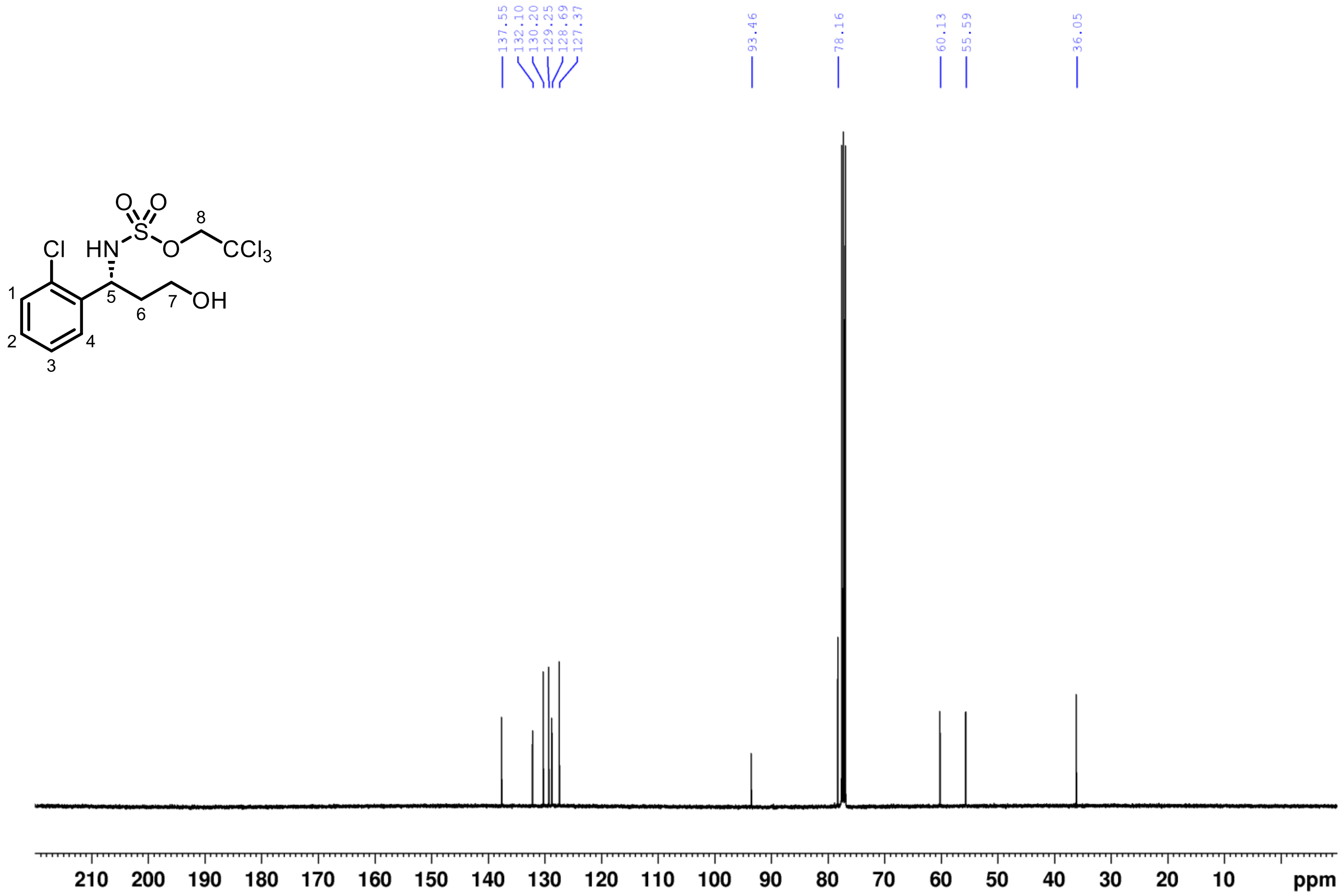

^{1}H NMR (700 MHz, $CDCl_3$) for *2,2,2-trichloroethyl (R)-(1-(2-bromophenyl)-3-hydroxypropyl)sulfamate* (**2f**)

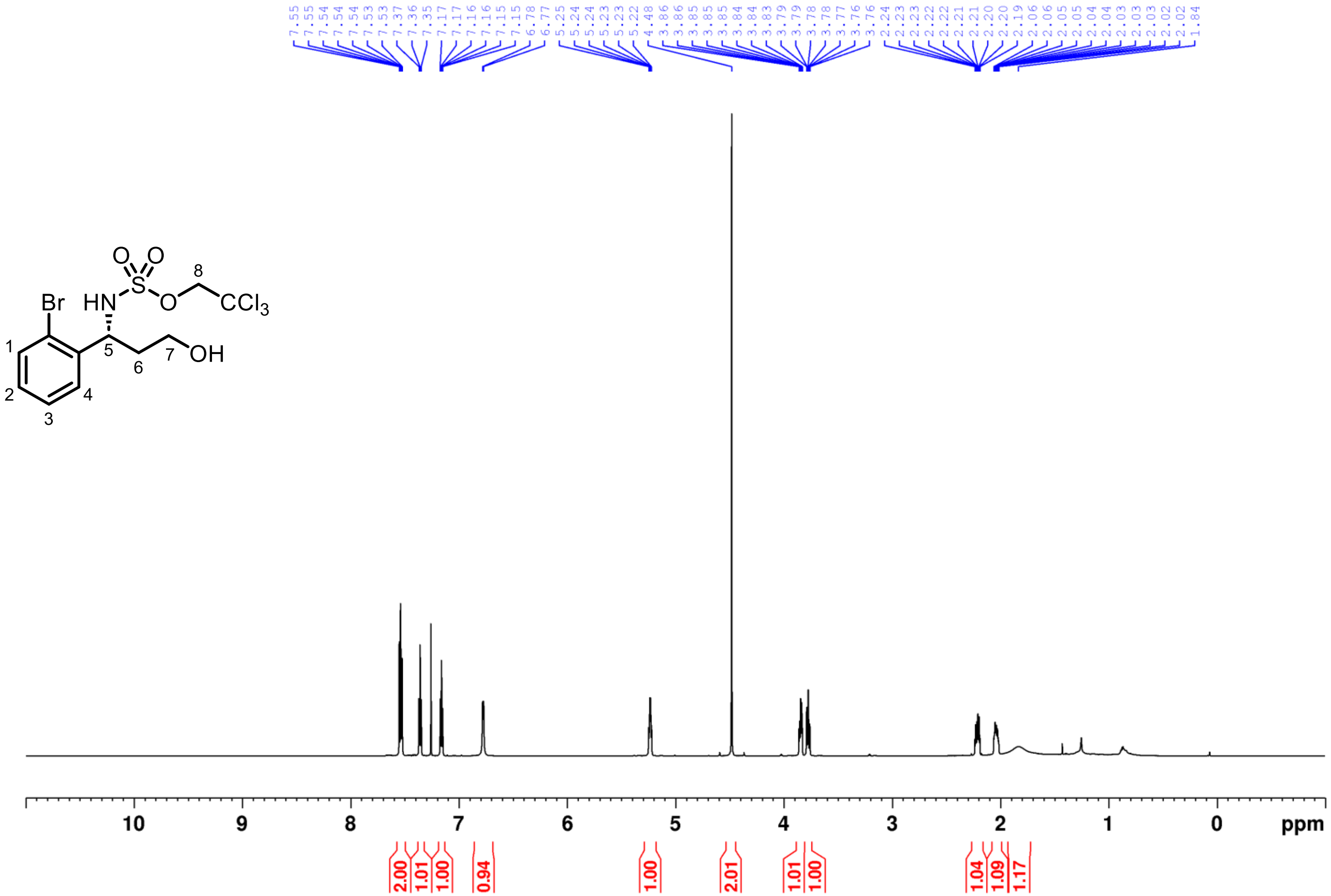

^{13}C NMR (176 MHz, CDCl$_3$) for *2,2,2-trichloroethyl (R)-(1-(2-bromophenyl)-3-hydroxypropyl)sulfamate* (**2f**)

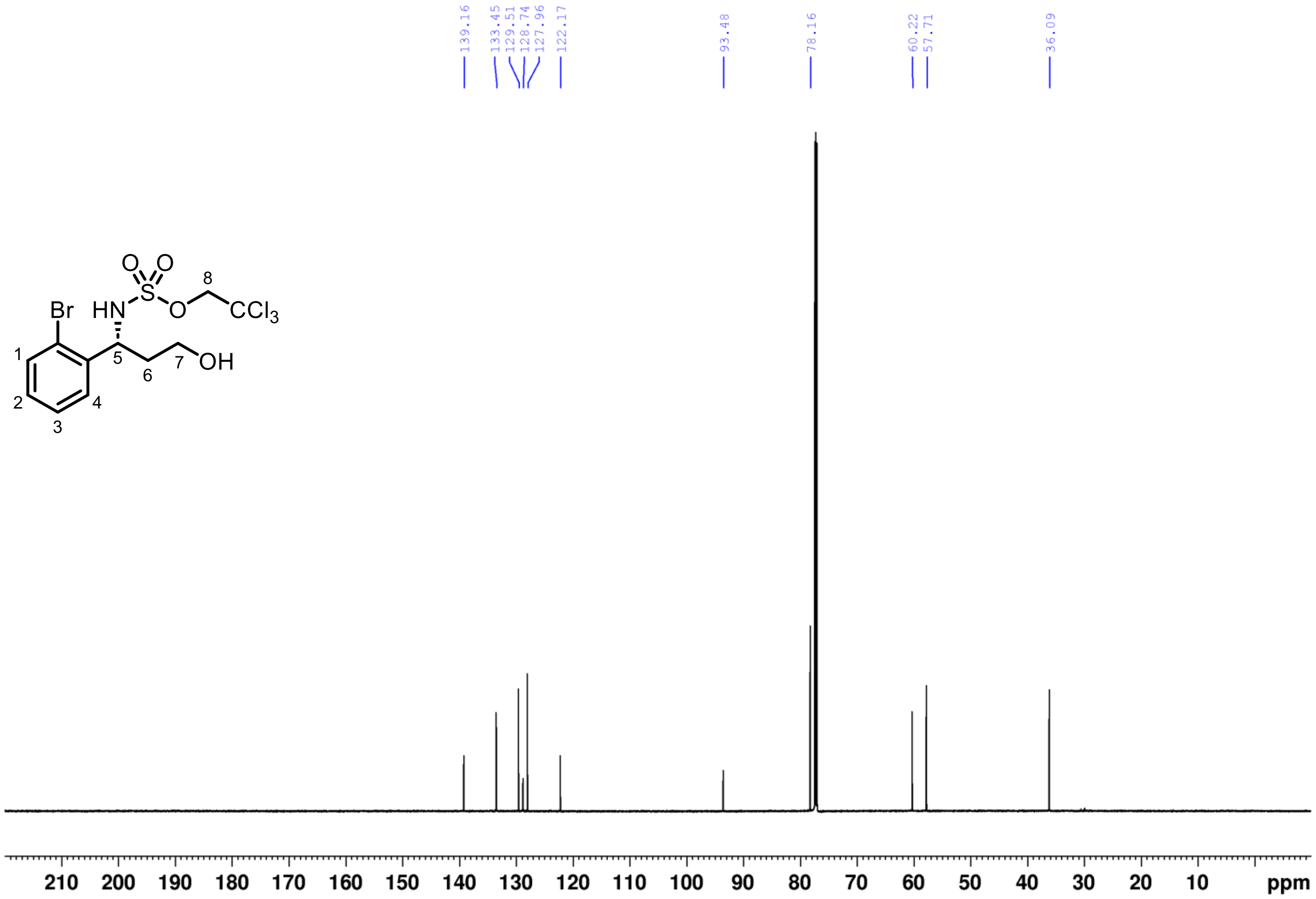

¹H NMR (700 MHz, CD_3OD) for *2,2,2-trichloroethyl (R)-(3-hydroxy-1-(m-tolyl)propyl)sulfamate* (**2g**)

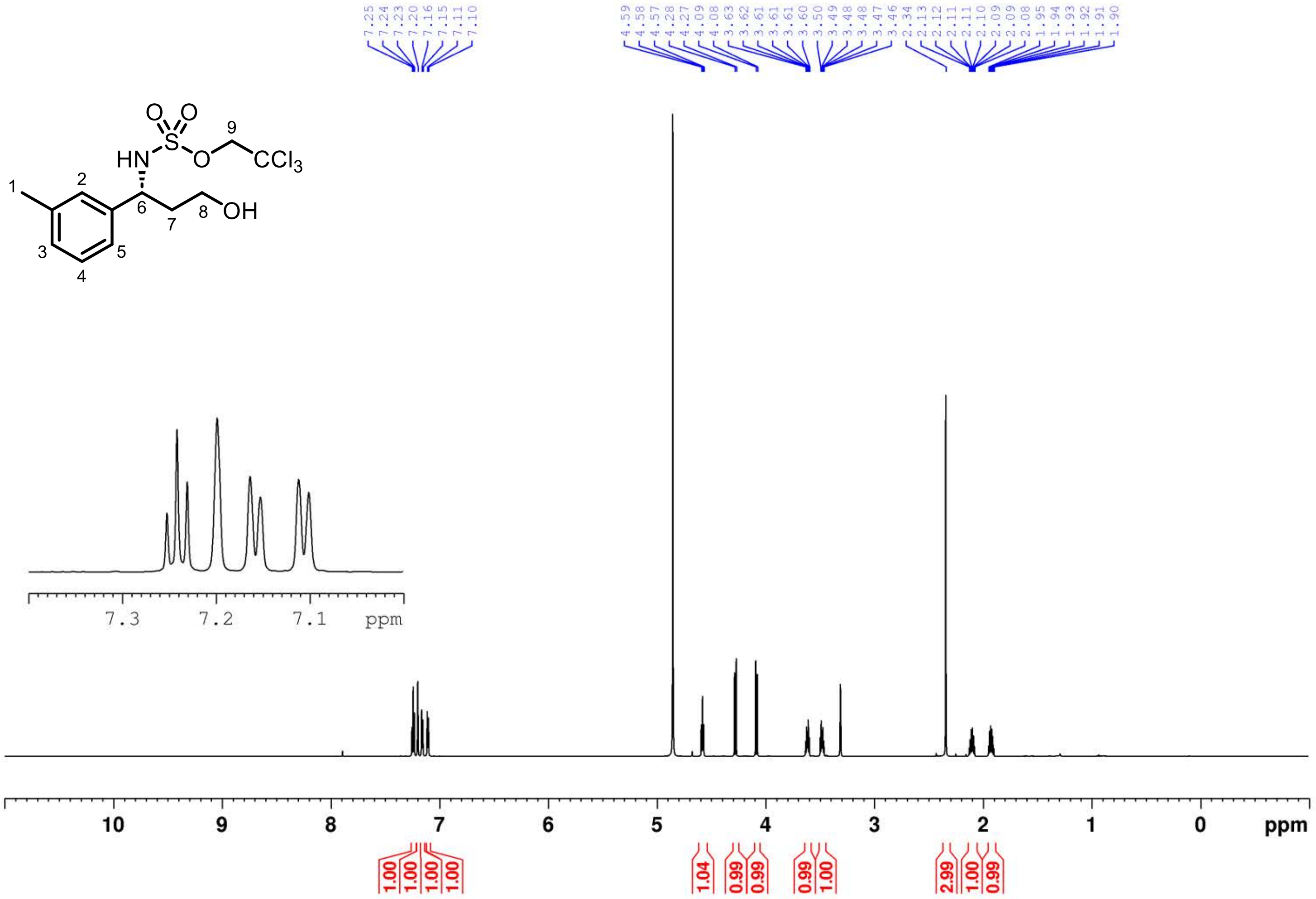

^{13}C NMR (176 MHz, CD_3OD) for *2,2,2-trichloroethyl (R)-(3-hydroxy-1-(m-tolyl)propyl)sulfamate* (**2g**)

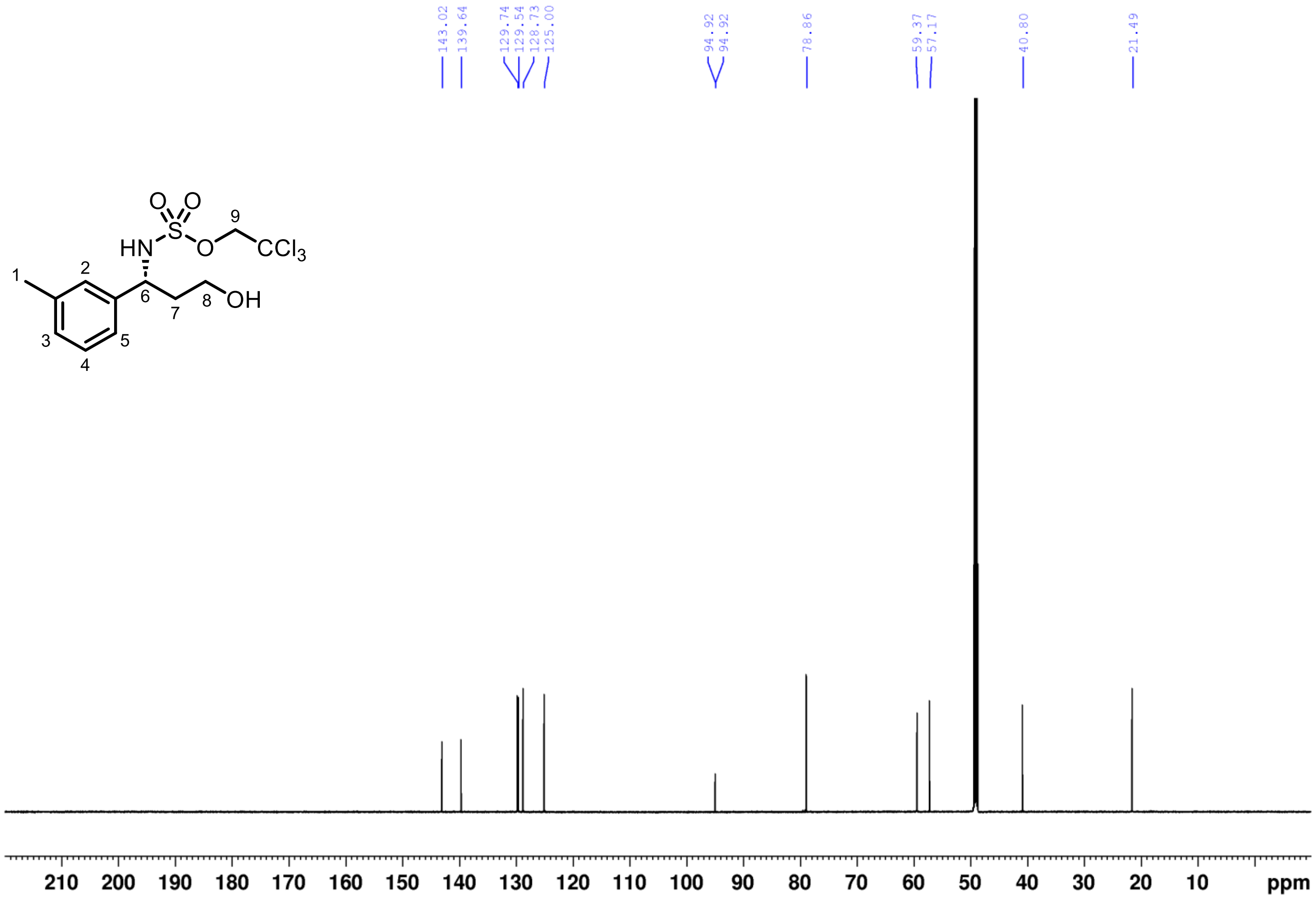

^{1}H NMR (700 MHz, CD_3OD) for *2,2,2-trichloroethyl (R)-(1-(3-(tert-butyl)phenyl)-3-hydroxypropyl)sulfamate* (**2h**)

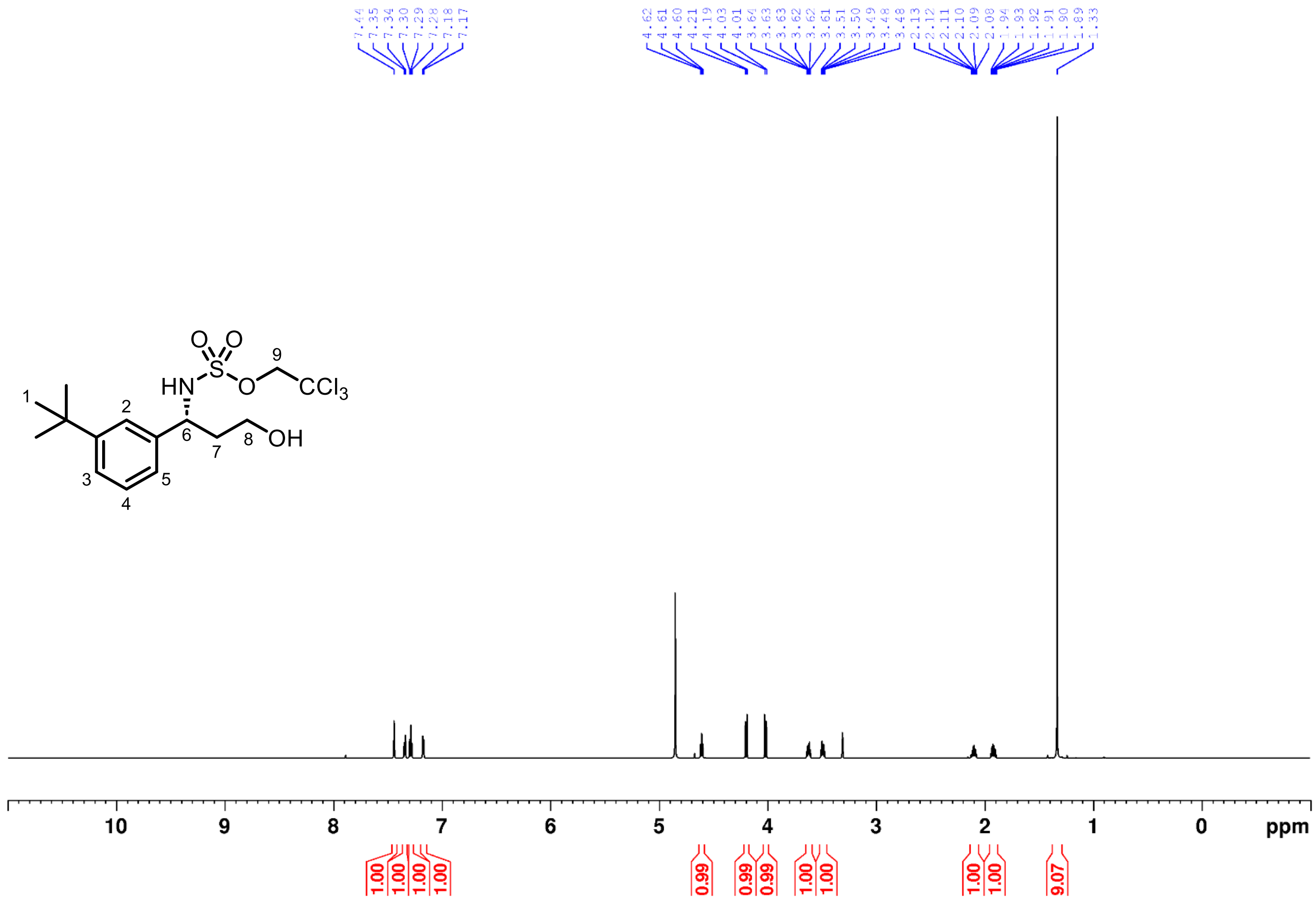

^{13}C NMR (176 MHz, CD_3OD) for *2,2,2-trichloroethyl (R)-(1-(3-(tert-butyl)phenyl)-3-hydroxypropyl)sulfamate* (**2h**)

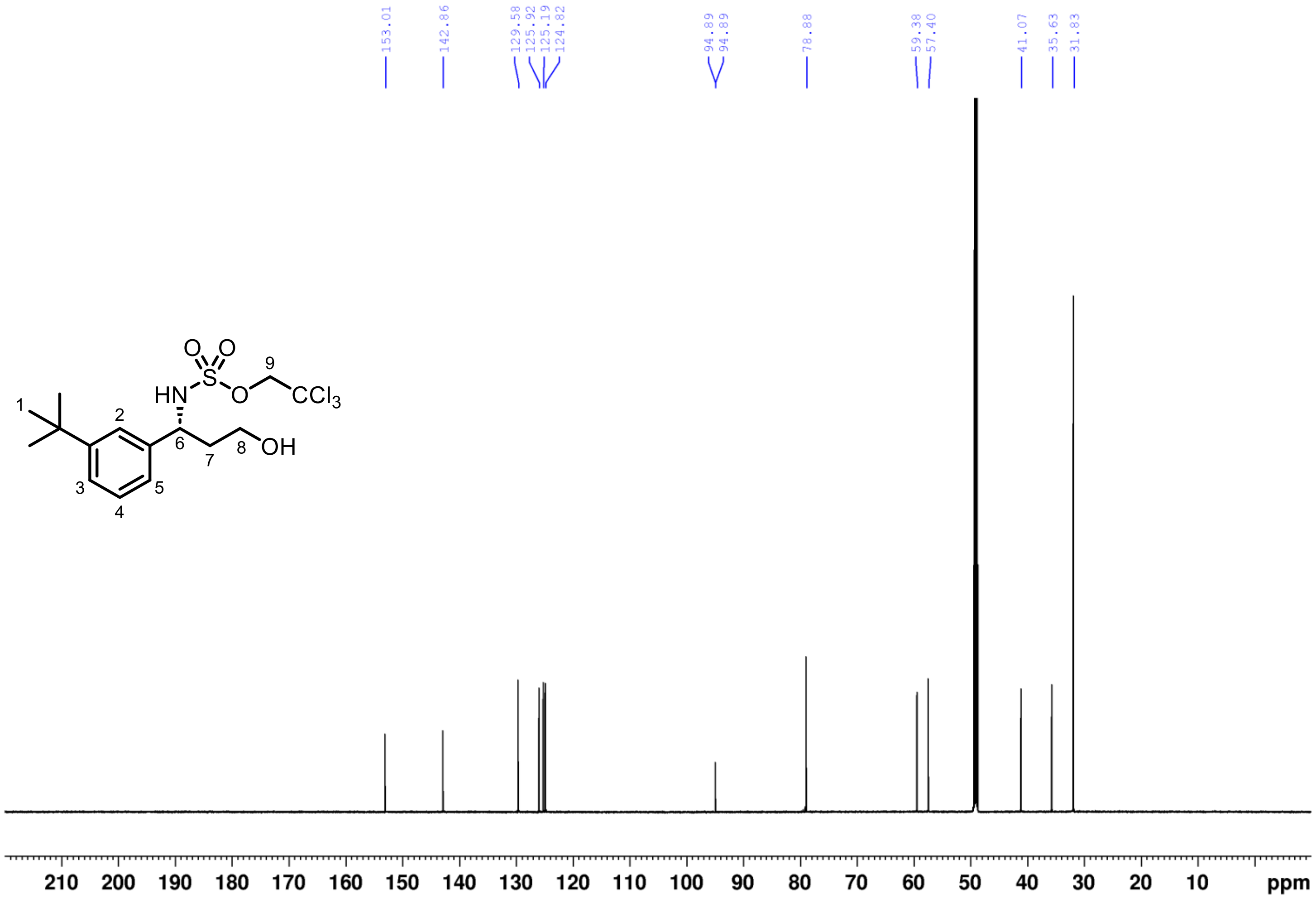

^{1}H NMR (700 MHz, $CDCl_3$) for *2,2,2-trichloroethyl (R)-(3-hydroxy-1-(3-(trifluoromethyl)phenyl)propyl)sulfamate* (**2i**)

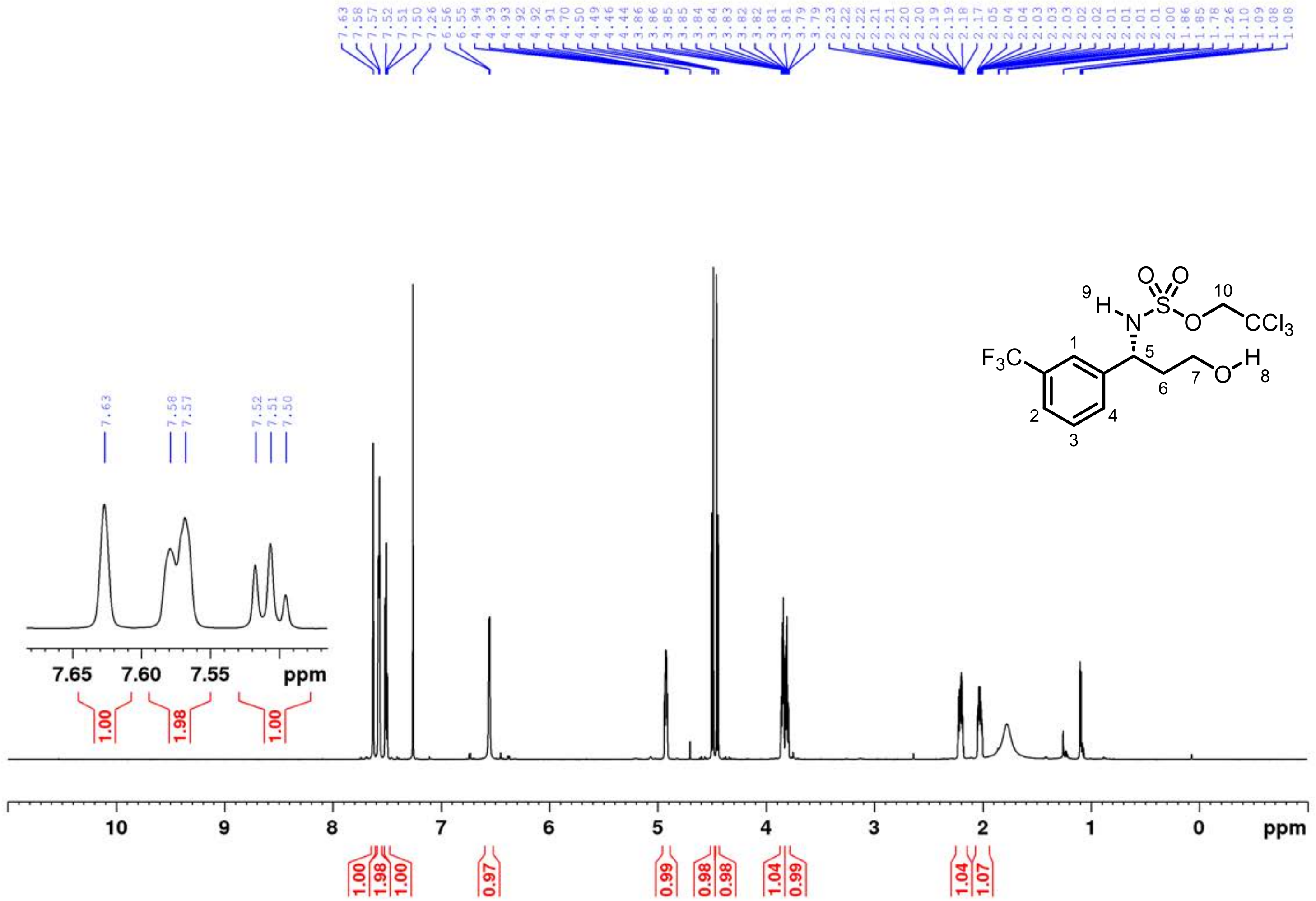

^{13}C NMR (176 MHz, $CDCl_3$) for *2,2,2-trichloroethyl (R)-(3-hydroxy-1-(3-(trifluoromethyl)phenyl)propyl)sulfamate* (**2i**)

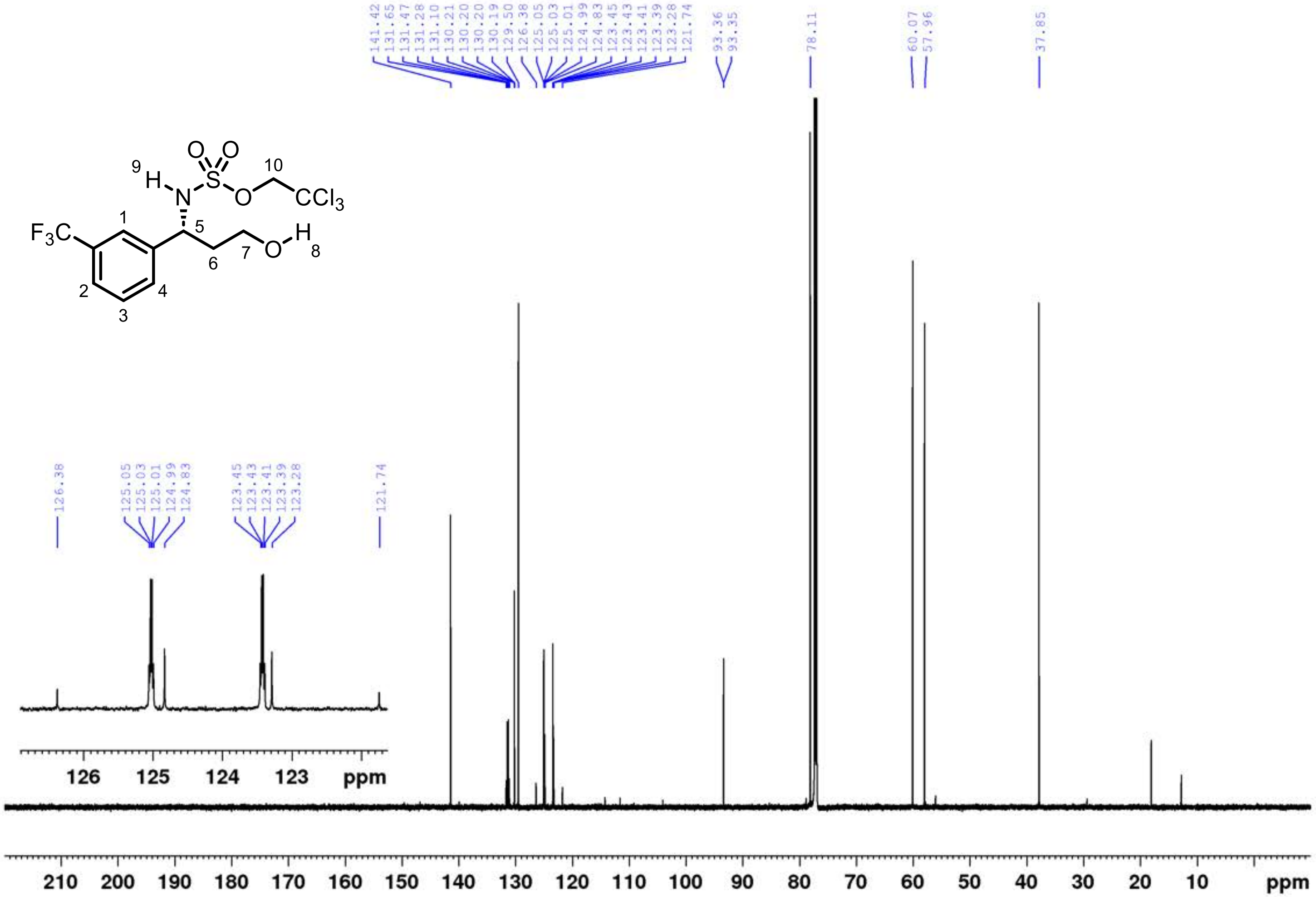

^{19}F NMR (376 MHz, $CDCl_3$) for *2,2,2-trichloroethyl (R)-(3-hydroxy-1-(3-(trifluoromethyl)phenyl)propyl)sulfamate* (**2i**)

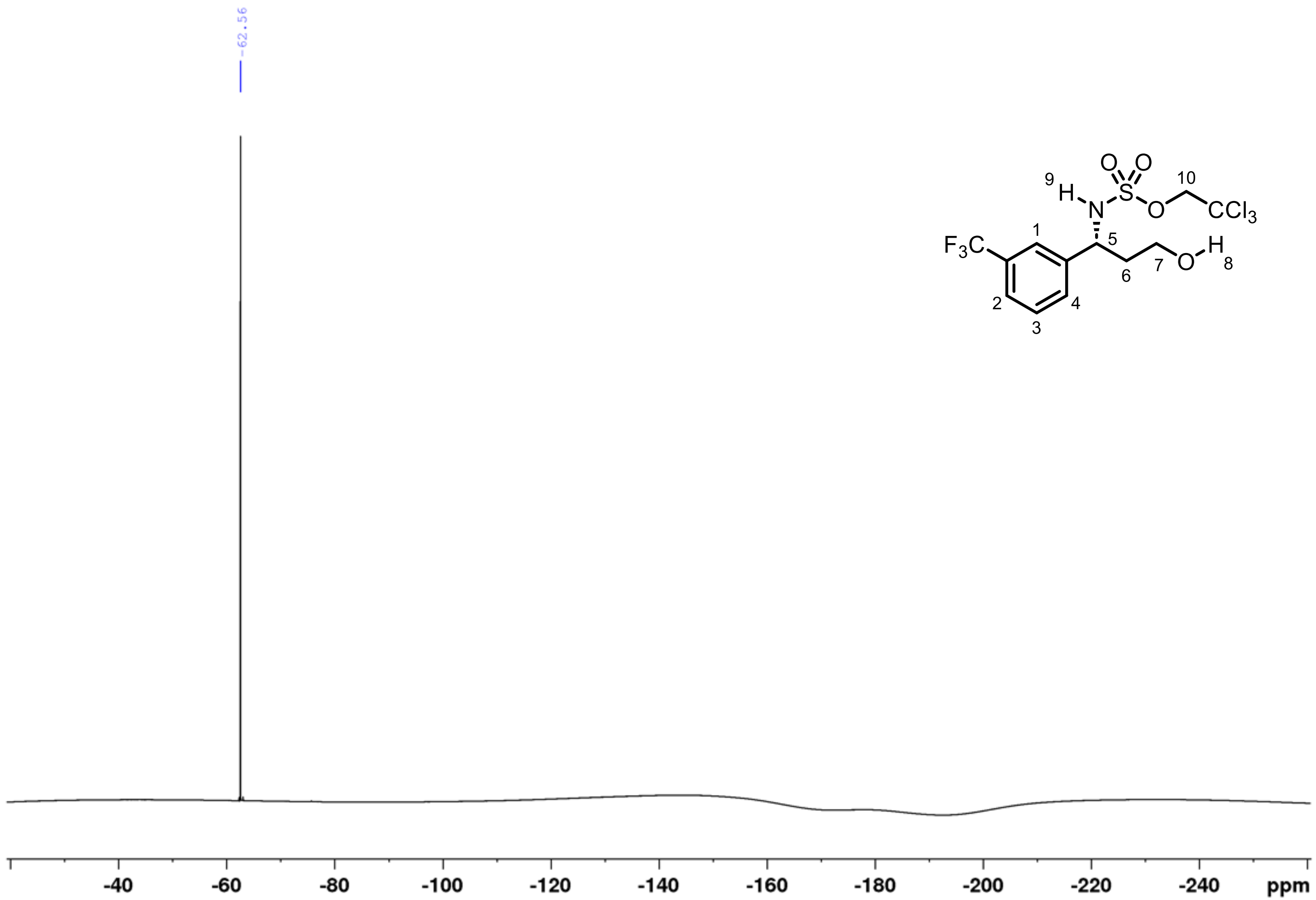

¹H NMR (700 MHz, $CDCl_3$) for *ethyl (R)-3-(3-hydroxy-1-(((2,2,2-trichloroethoxy)sulfonyl)amino)propyl)benzoate* (**2j**)

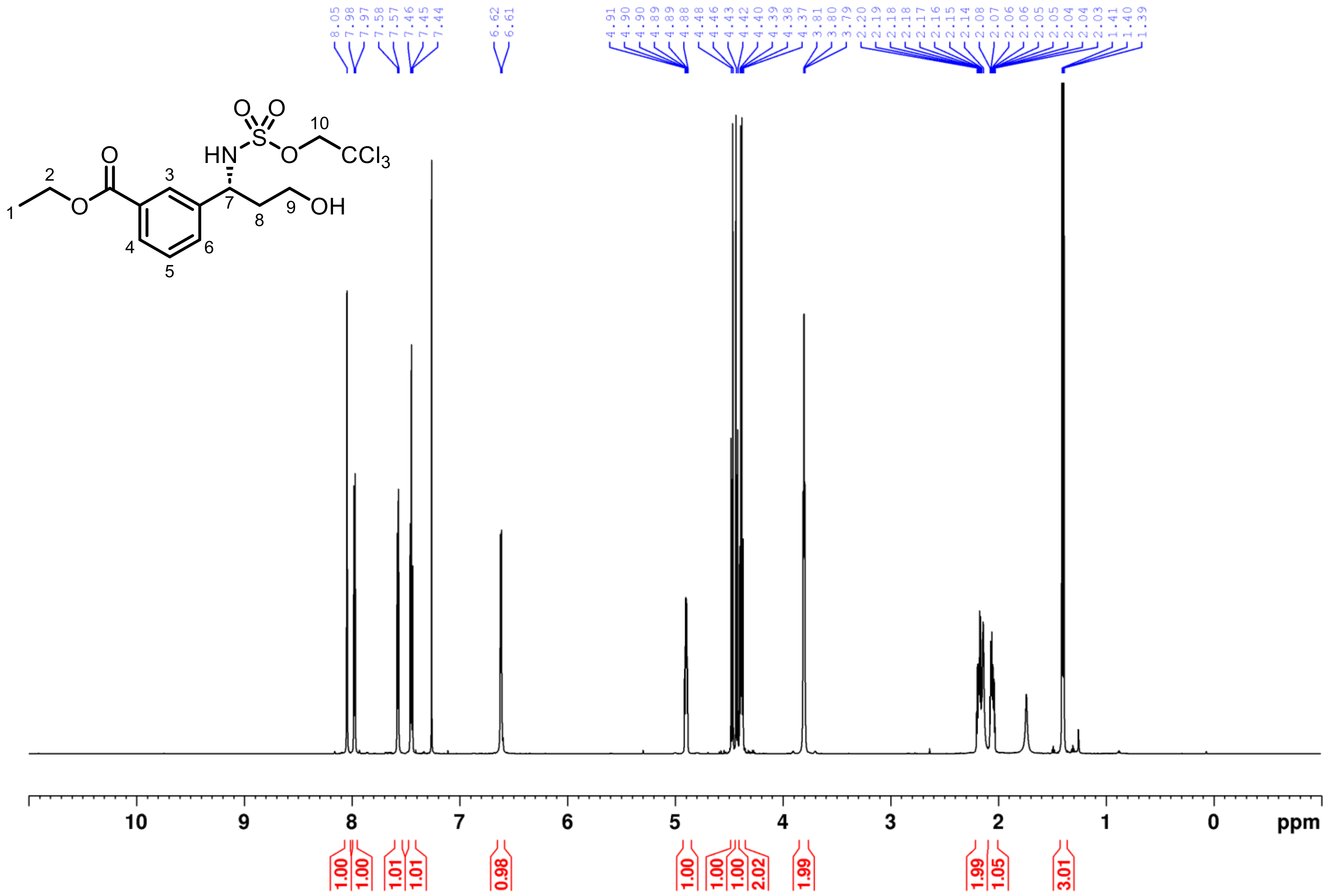

^{13}C NMR (176 MHz, CDCl$_3$) for *ethyl (R)-3-(3-hydroxy-1-(((2,2,2-trichloroethoxy)sulfonyl)amino)propyl)benzoate* (**2j**)

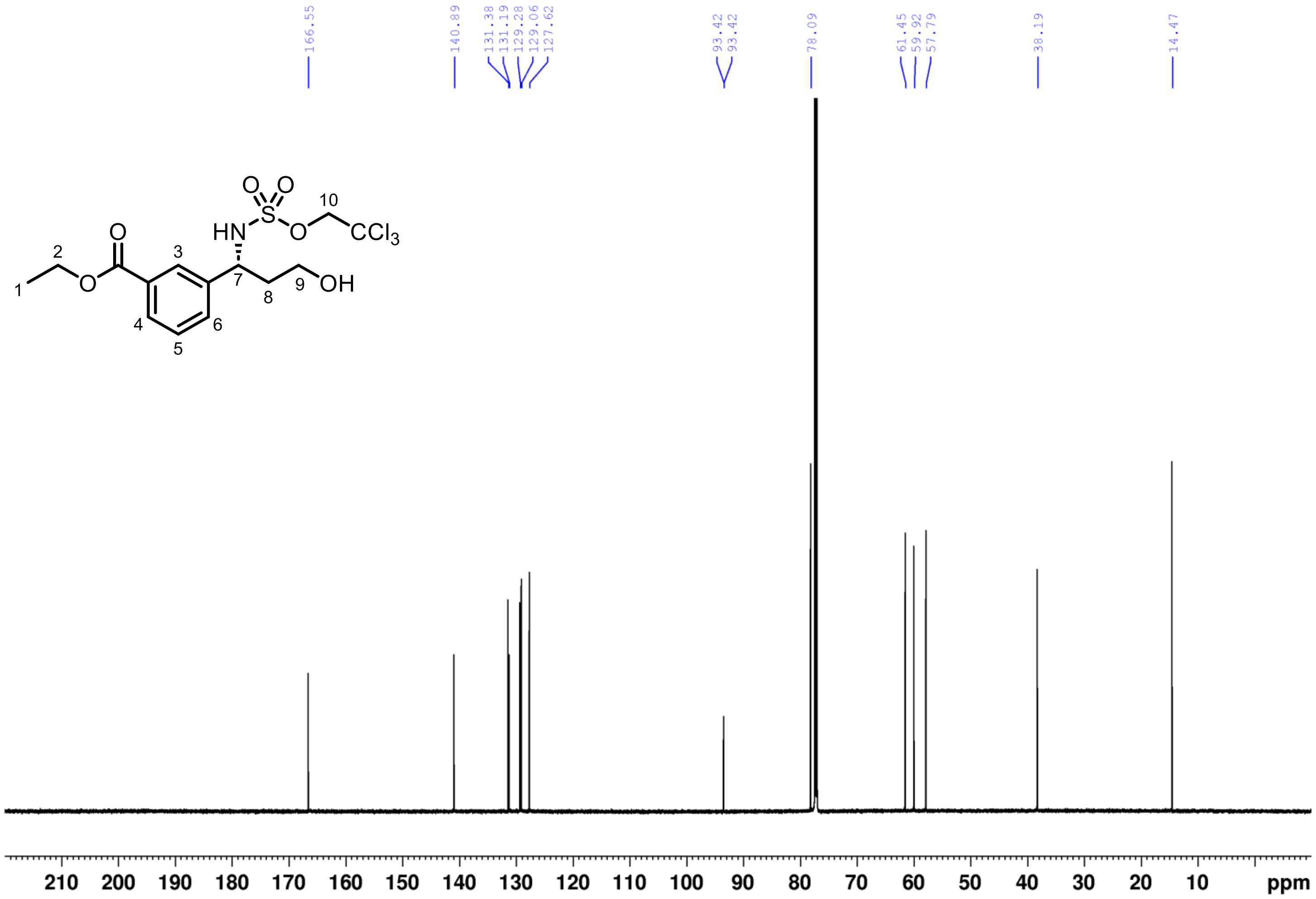

¹H NMR (400 MHz, $CDCl_3$) for *2,2,2-trichloroethyl (R)-(3-hydroxy-1-(naphthalen-1-yl)propyl)sulfamate* (**2k**)

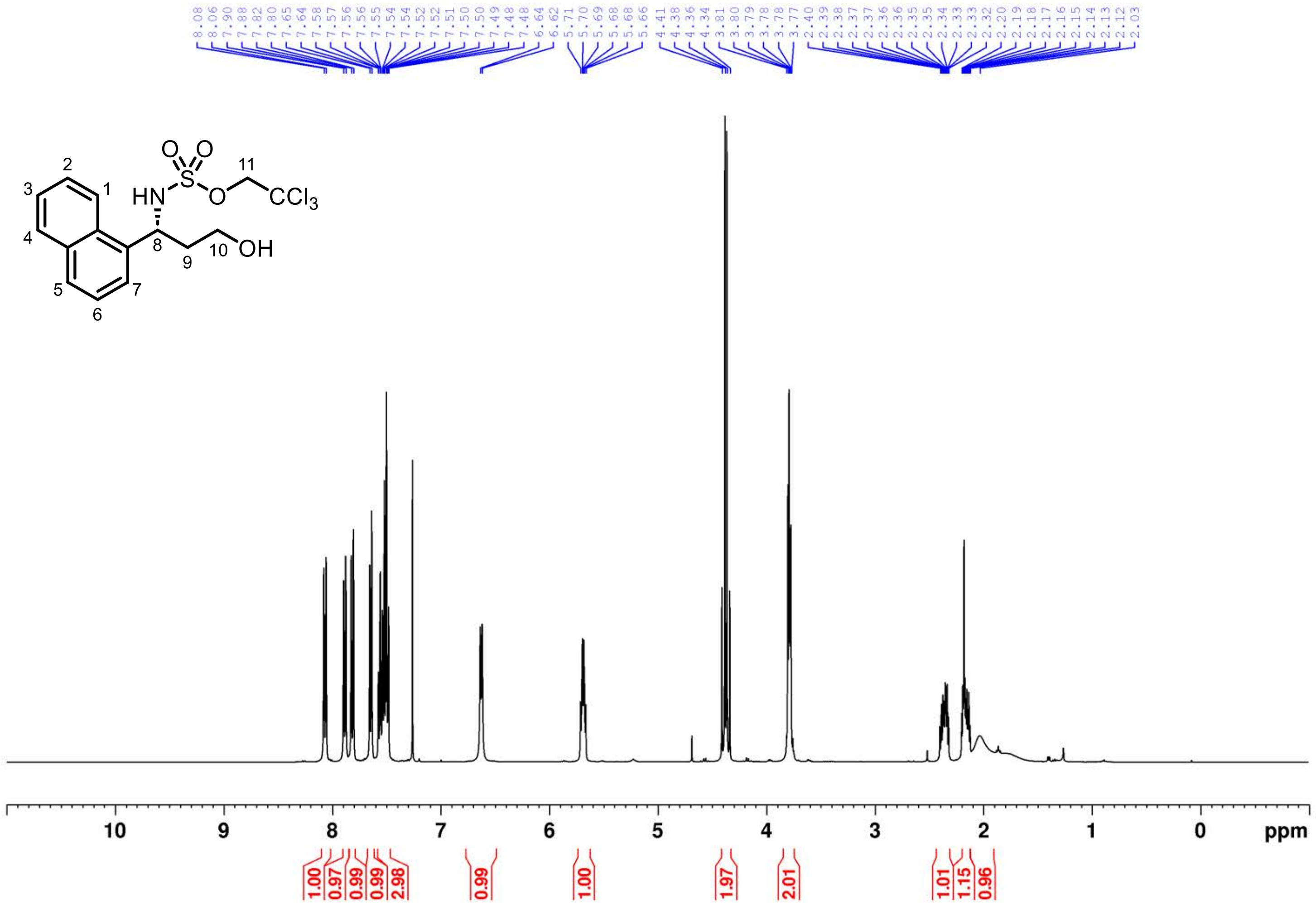

^{13}C NMR (101 MHz, $CDCl_3$) for *2,2,2-trichloroethyl (R)-(3-hydroxy-1-(naphthalen-1-yl)propyl)sulfamate* (**2k**)

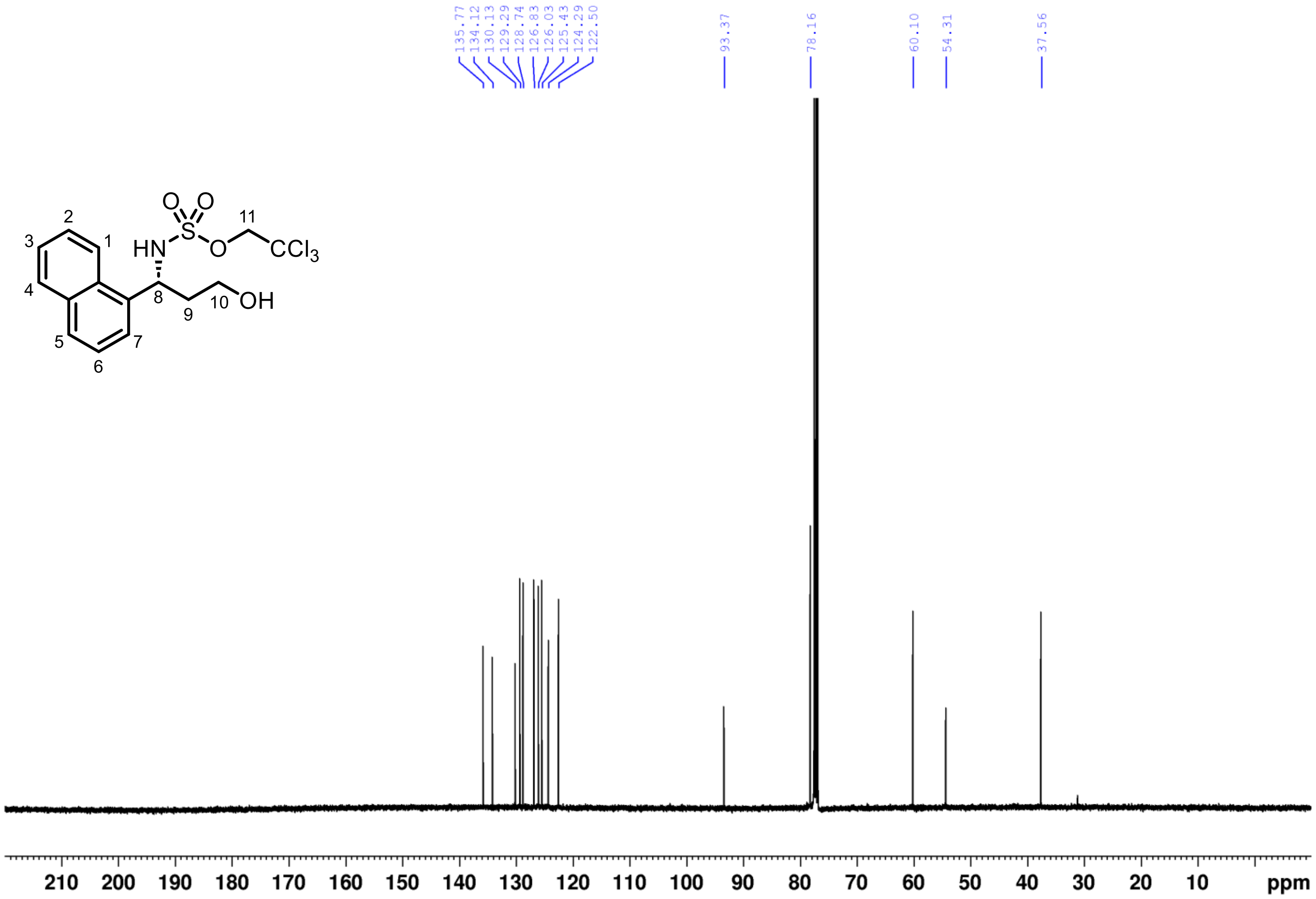

^{1}H NMR (700 MHz, $(CD_3)_2CO$) for *2,2,2-trichloroethyl (R)-(1-(4-chlorophenyl)-3-hydroxypropyl)sulfamate* (**2l**)

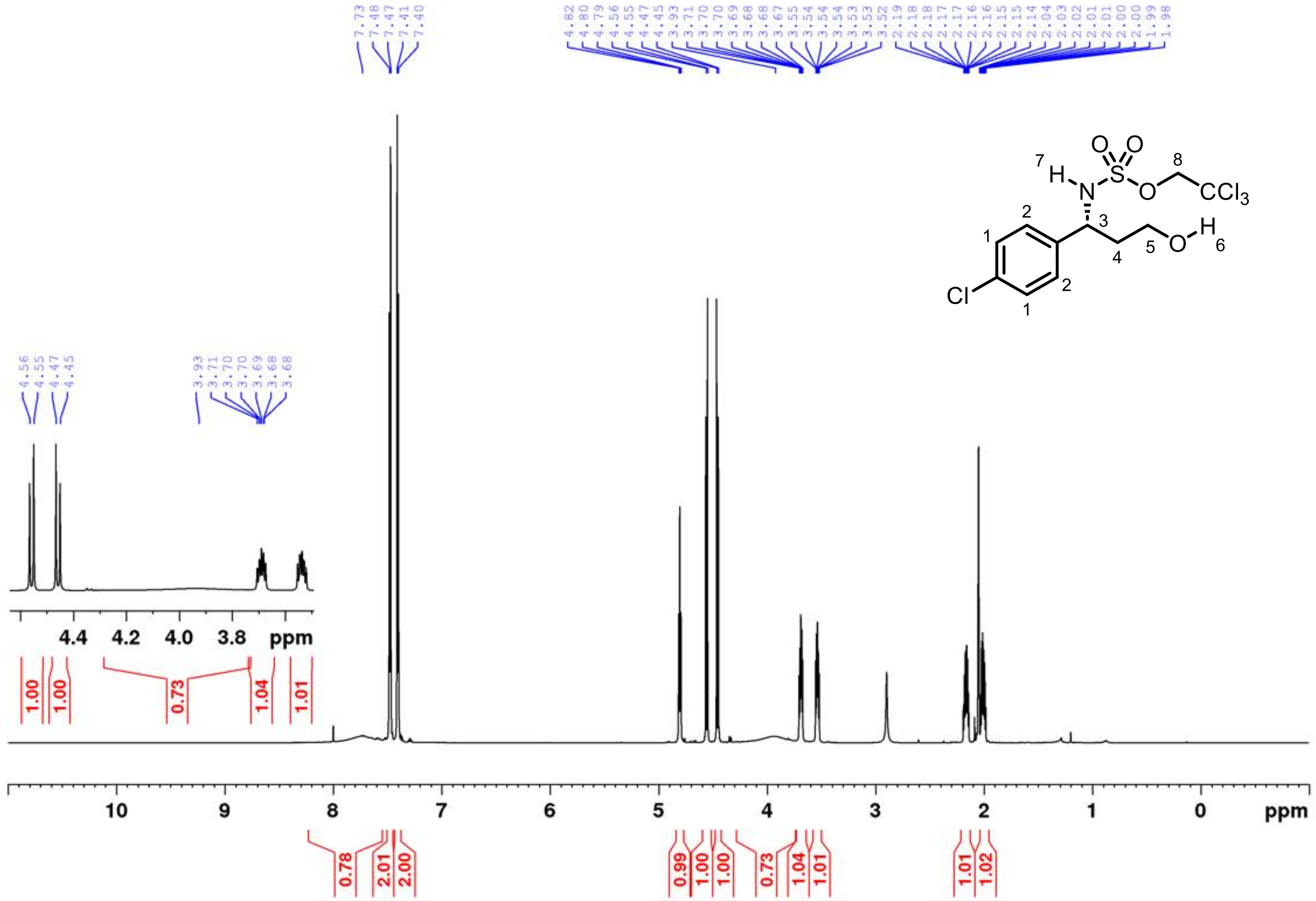

^{13}C NMR (176 MHz, $(CD_3)_2CO$) for *2,2,2-trichloroethyl (R)-(1-(4-chlorophenyl)-3-hydroxypropyl)sulfamate* (**2l**)

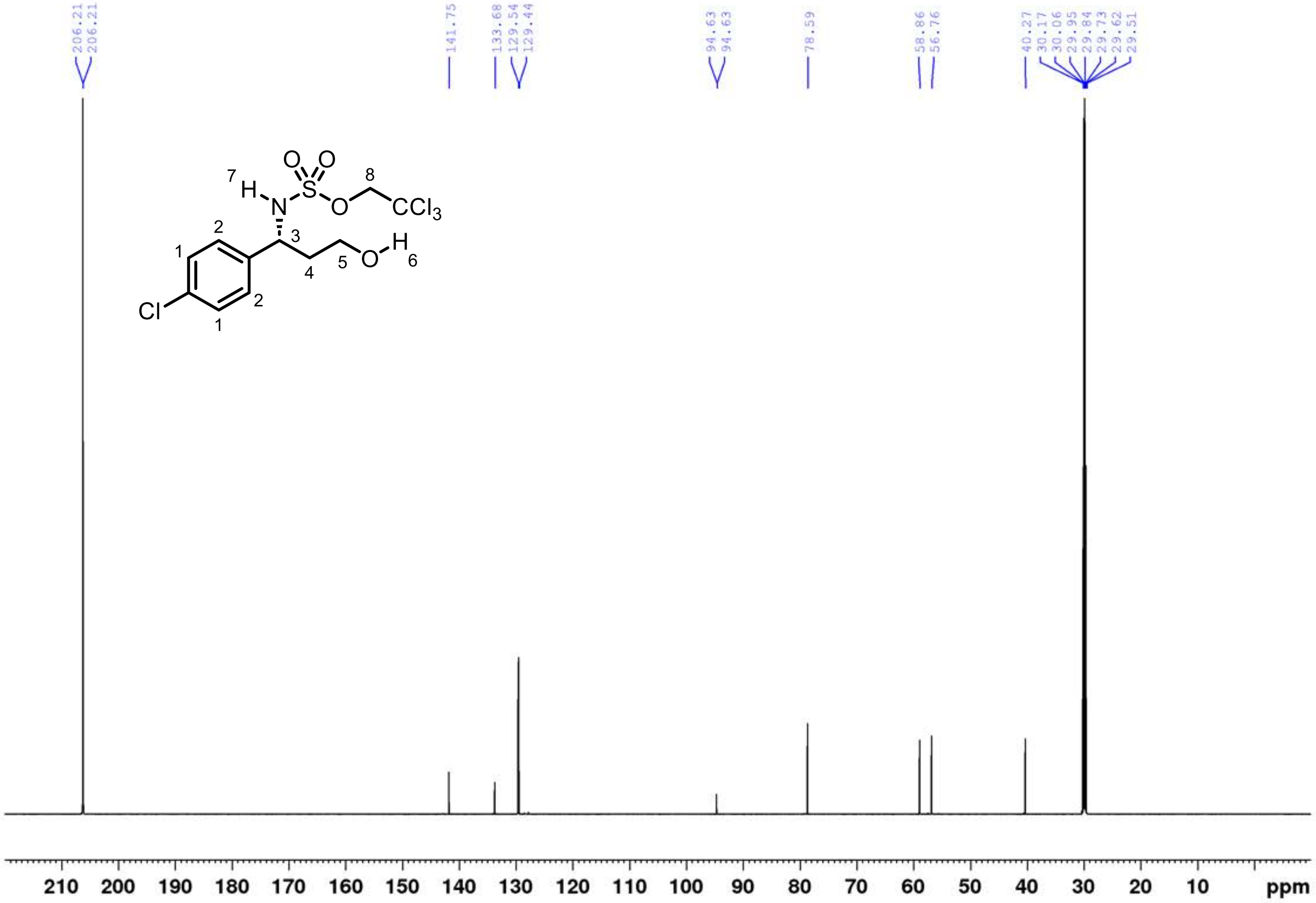

^{1}H NMR (400 MHz, CD_3OD) for *2,2,2-trichloroethyl (R)-(3-hydroxy-1-(4-methoxyphenyl)propyl)sulfamate* (**2m**)

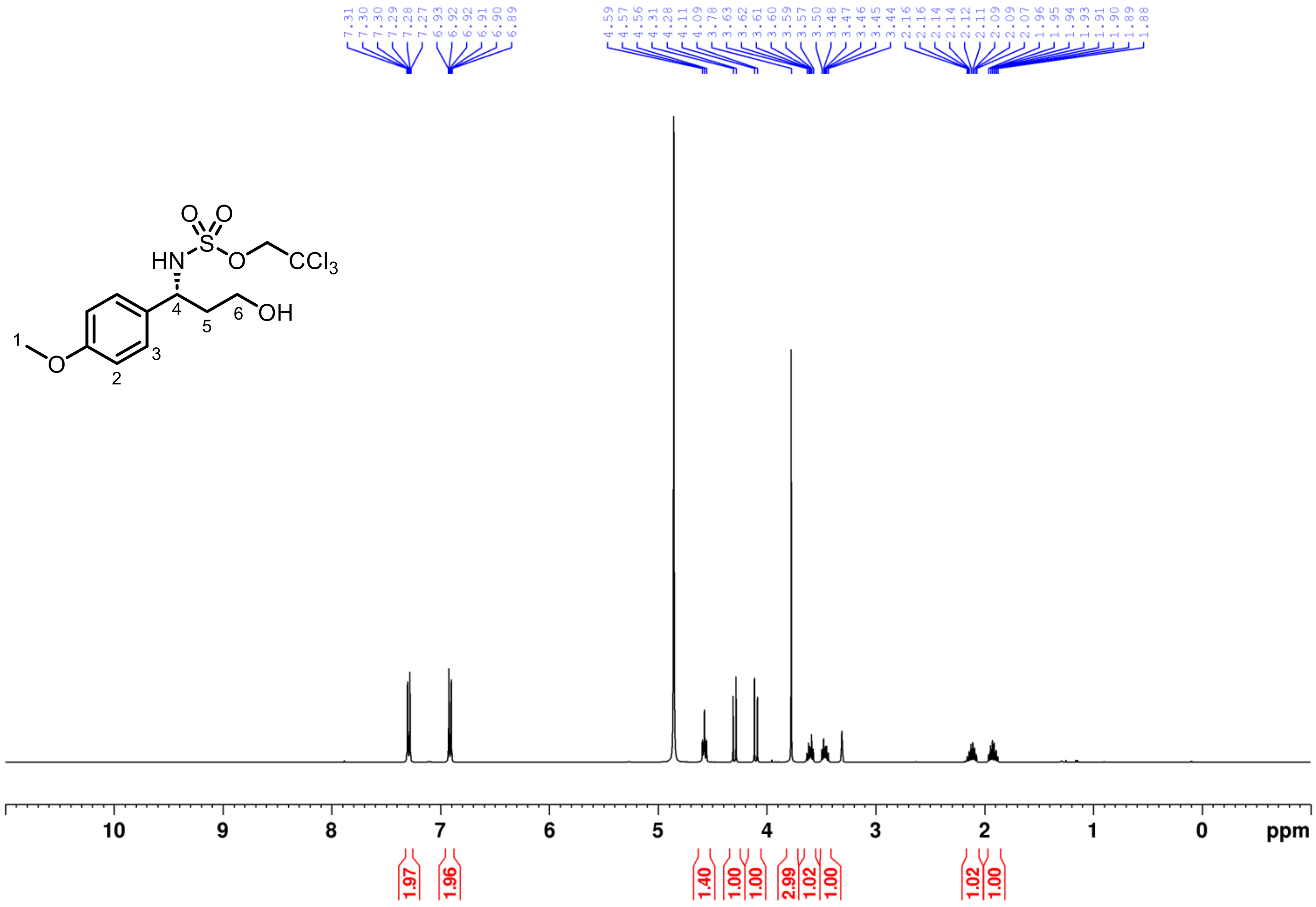

^{13}C NMR (101 MHz, CD_3OD) for *2,2,2-trichloroethyl (R)-(3-hydroxy-1-(4-methoxyphenyl)propyl)sulfamate* (**2m**)

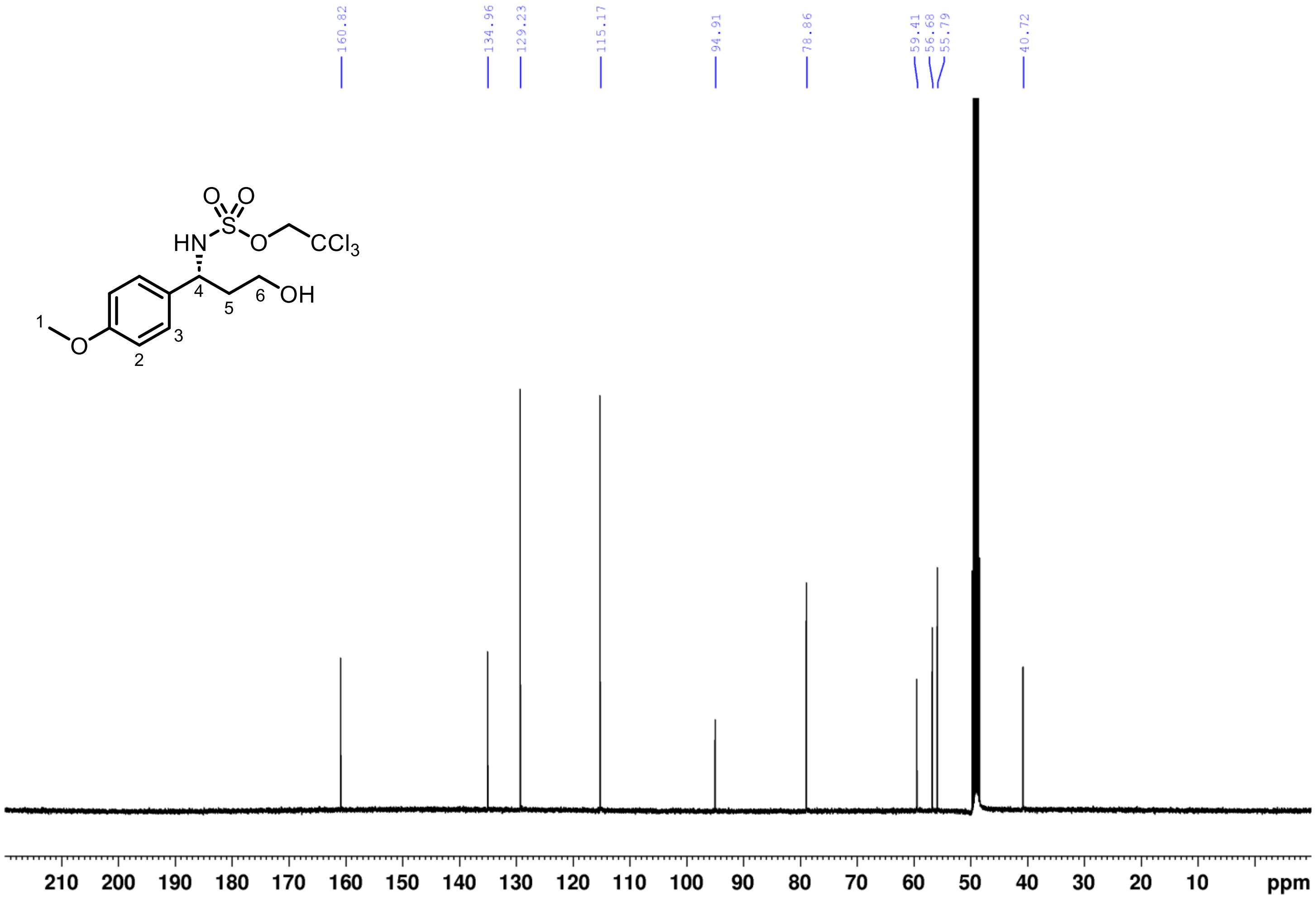

^{1}H NMR (400 MHz, $(CD_3)_2CO$) for *2,2,2-trichloroethyl (R)-(1-(4-bromophenyl)-3-hydroxypropyl)sulfamate* (**2n**)

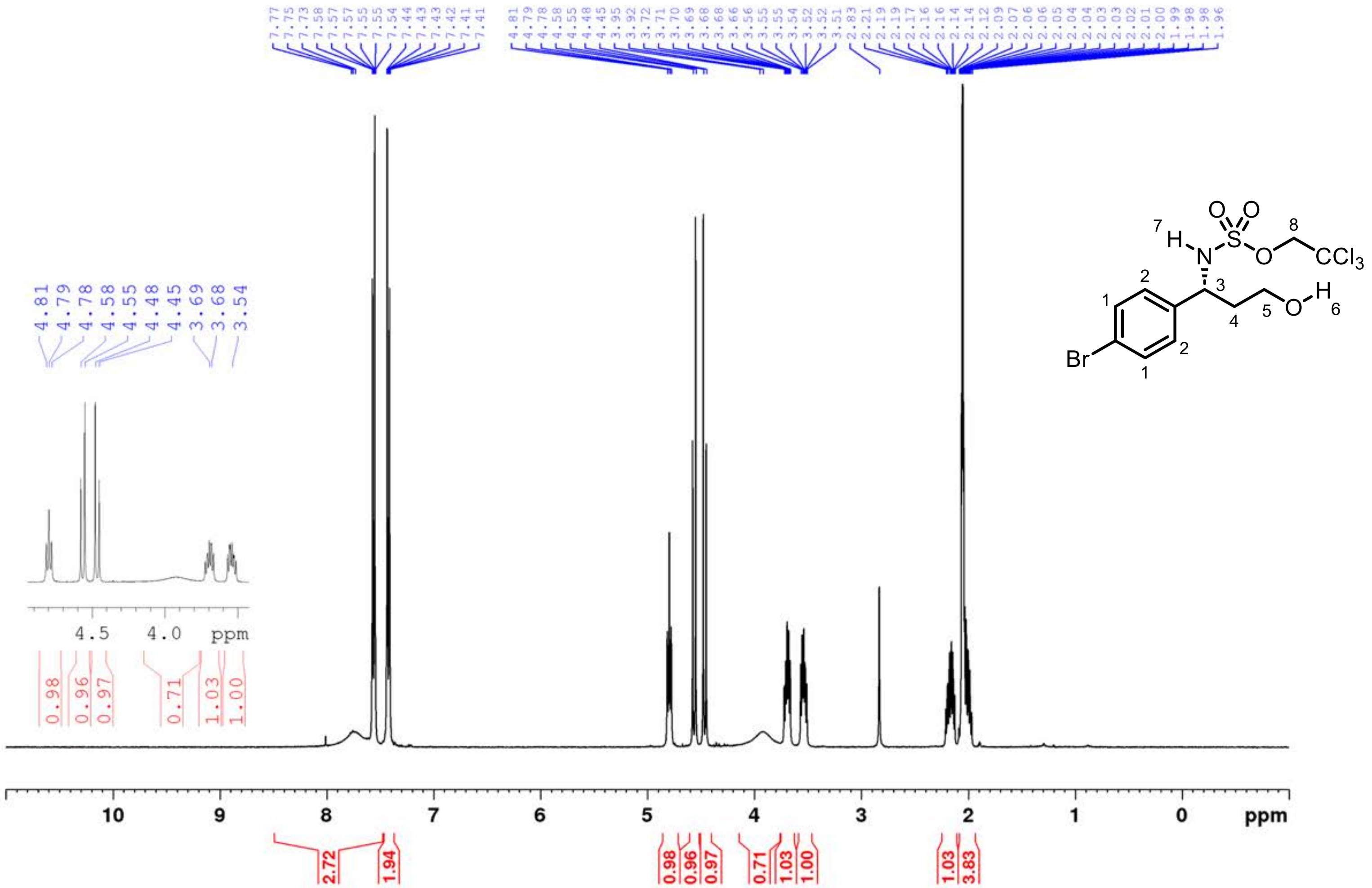

^{13}C NMR (101 MHz, $(CD_3)_2CO$) for *2,2,2-trichloroethyl (R)-(1-(4-bromophenyl)-3-hydroxypropyl)sulfamate* (**2n**)

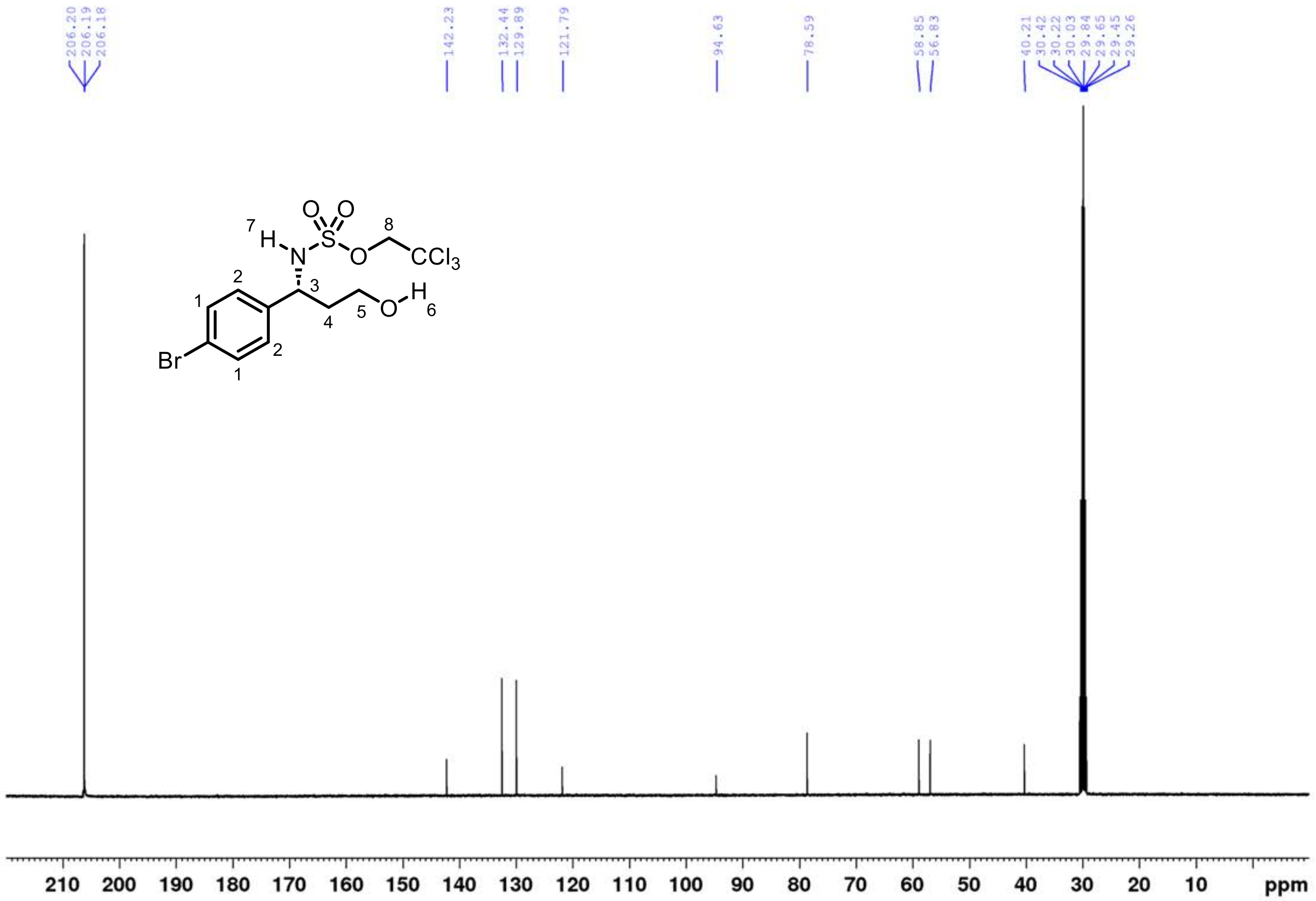

^{1}H NMR (700 MHz, CD_3OD) for *2,2,2-trichloroethyl (R)-(3-hydroxy-1-(4-(trifluoromethyl)phenyl)propyl)sulfamate* (**2o**)

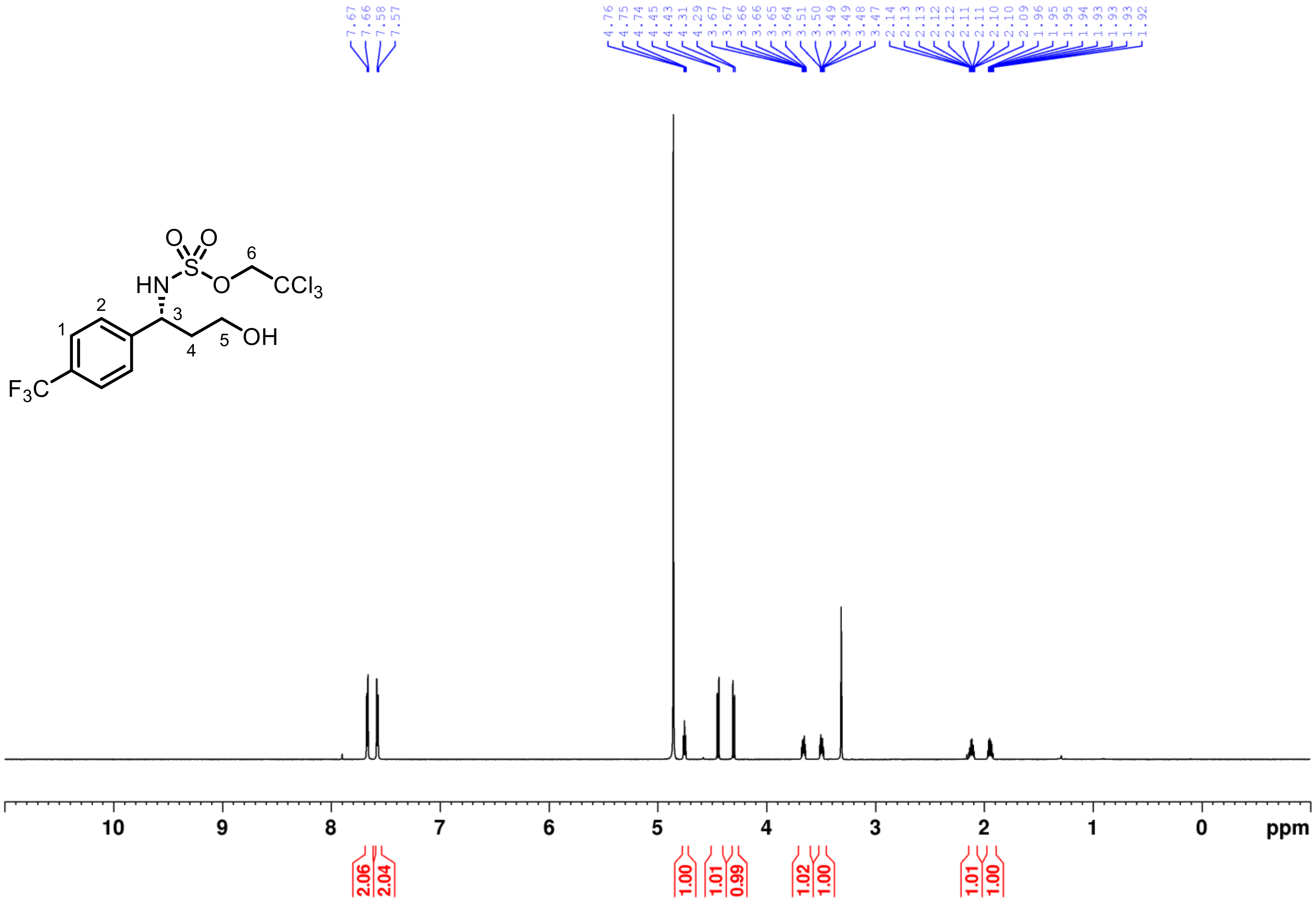

^{13}C NMR (176 MHz, CD_3OD) for *2,2,2-trichloroethyl (R)-(3-hydroxy-1-(4-(trifluoromethyl)phenyl)propyl)sulfamate* (**2o**)

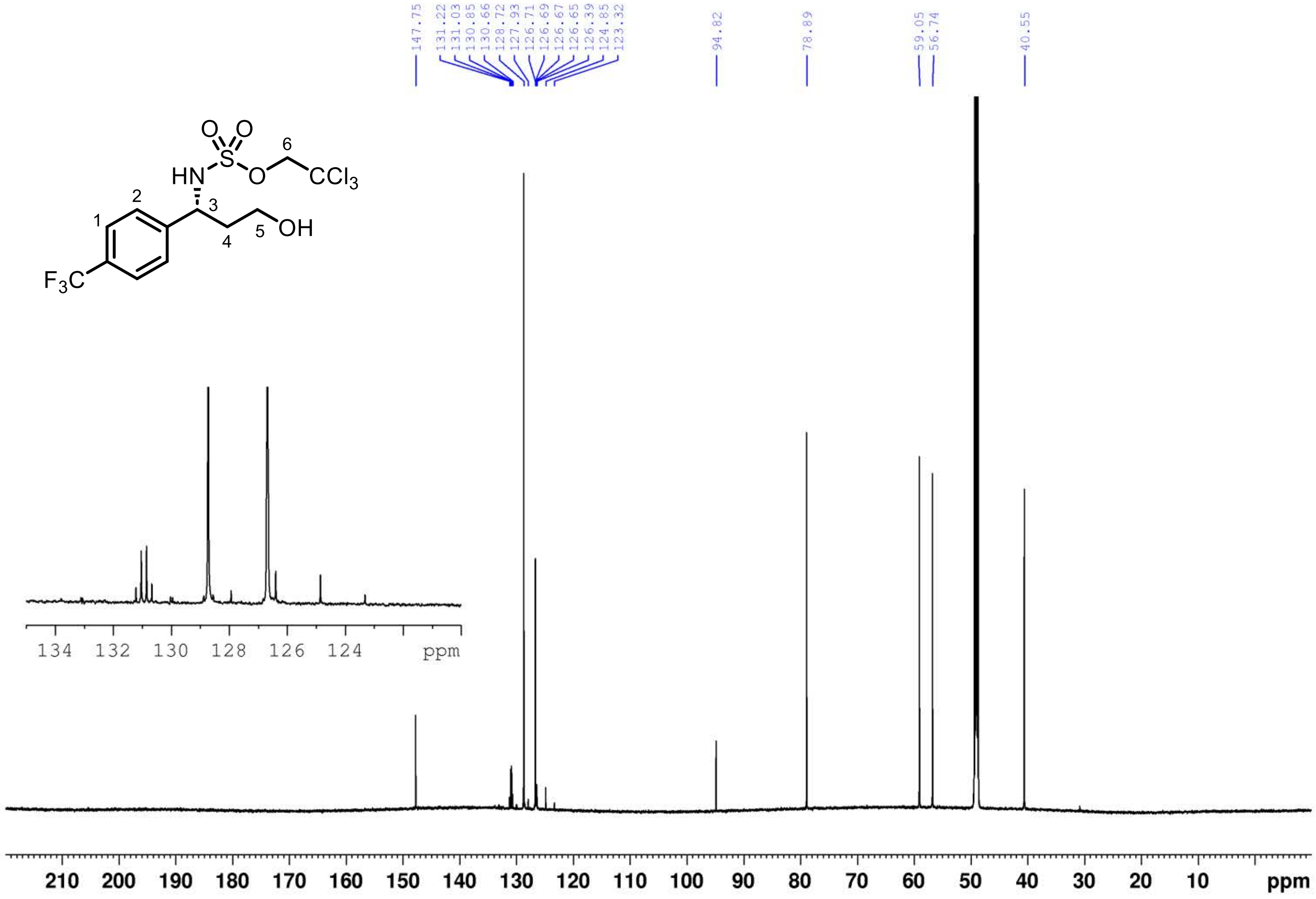

^{19}F NMR (376 MHz, CD_3OD) for *2,2,2-trichloroethyl (R)-(3-hydroxy-1-(4-(trifluoromethyl)phenyl)propyl)sulfamate* (**2o**)

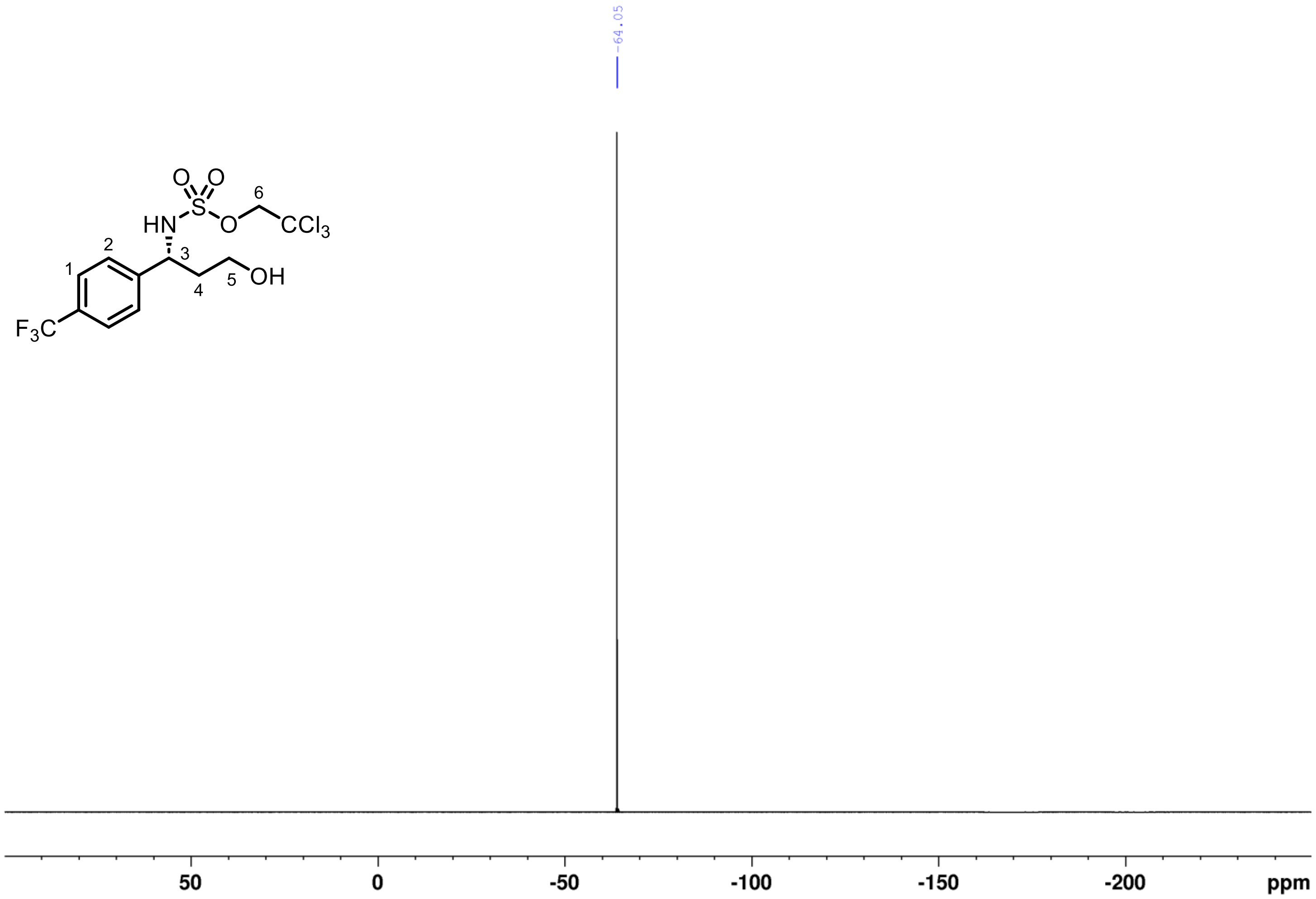

¹H NMR (400 MHz, CDCl₃) for *2,2,2-trichloroethyl (S)-(3-hydroxy-2,2-dimethyl-1-phenylpropyl)sulfamate* (**2p**)

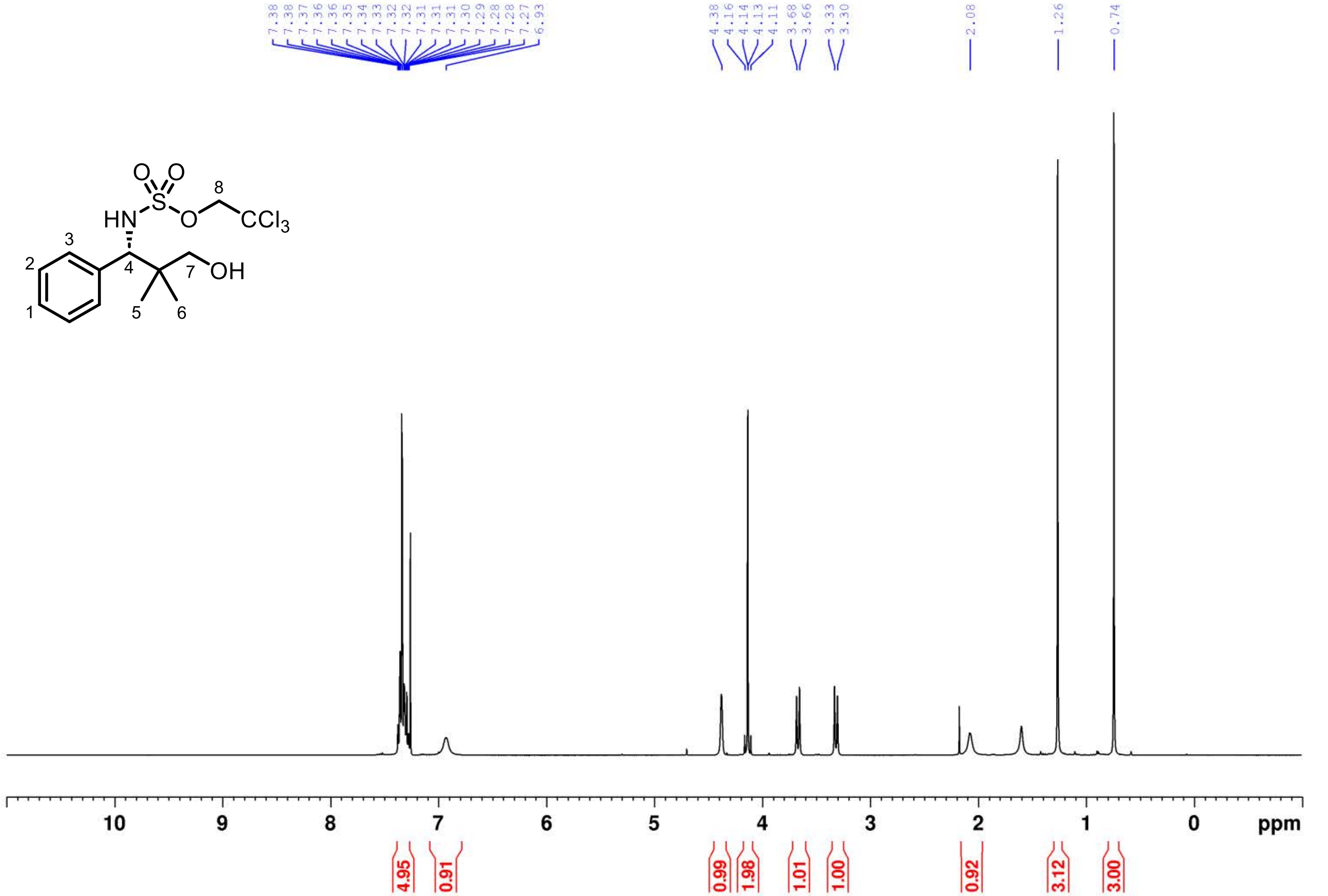

^{13}C NMR (101 MHz, $CDCl_3$) for *2,2,2-trichloroethyl (S)-(3-hydroxy-2,2-dimethyl-1-phenylpropyl)sulfamate* (**2p**)

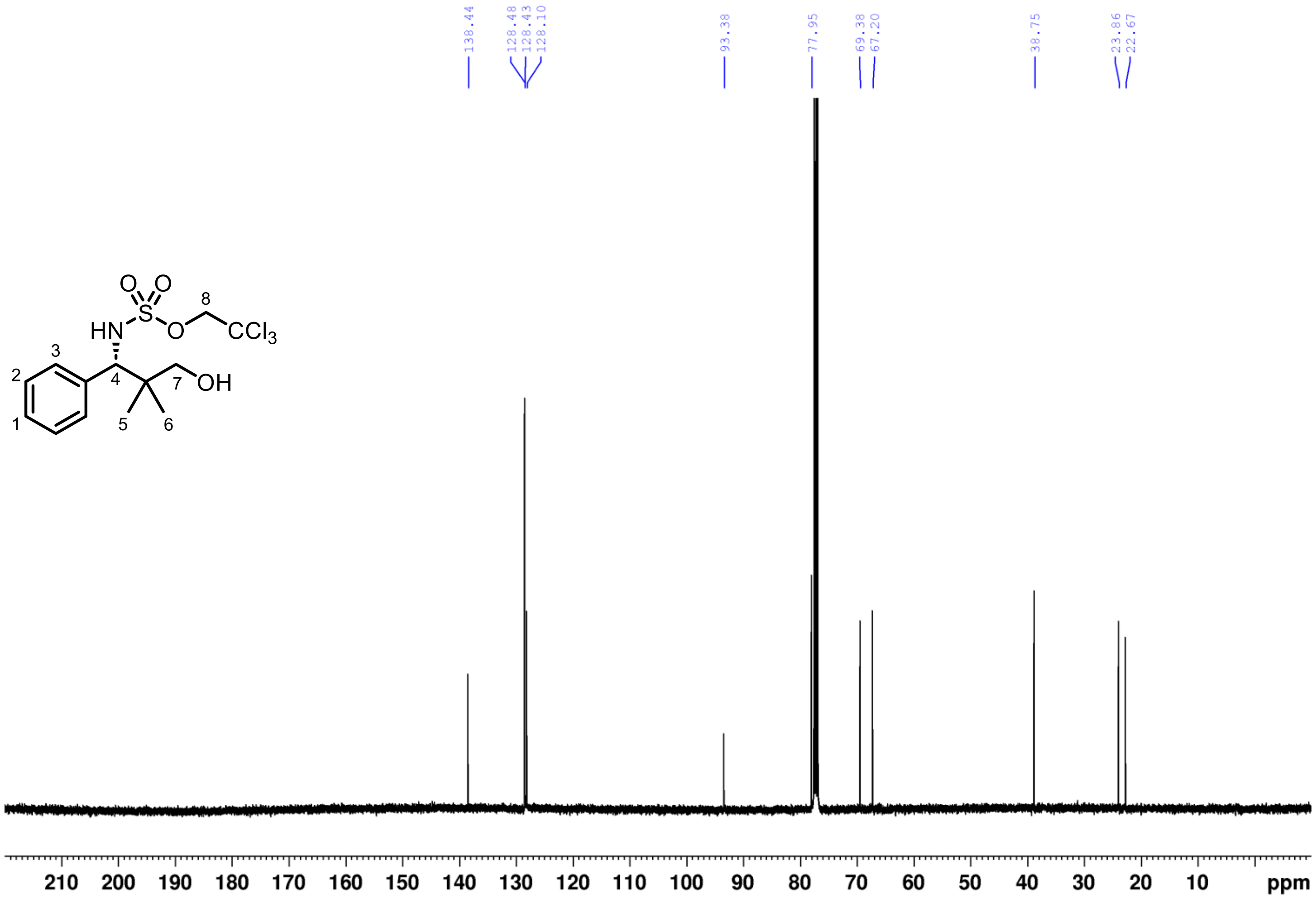

¹H NMR (400 MHz, CDCl₃) for *2,2,2-trichloroethyl (S)-((1-(hydroxymethyl)cyclopentyl)(phenyl)methyl)sulfamate* (**2q**)

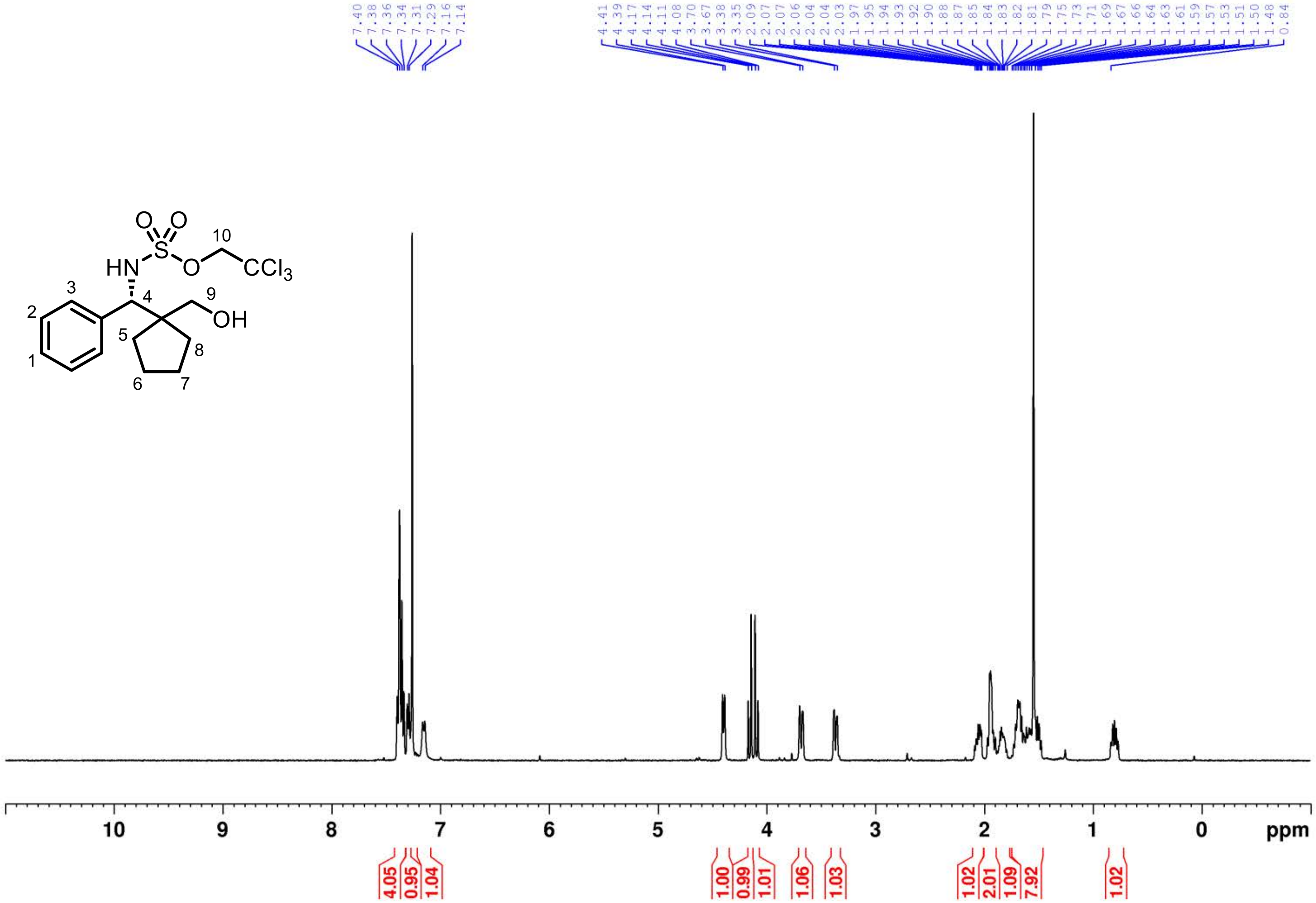

^{13}C NMR (101 MHz, $CDCl_3$) for *2,2,2-trichloroethyl (S)-((1-(hydroxymethyl)cyclopentyl)(phenyl)methyl)sulfamate* (**2q**)

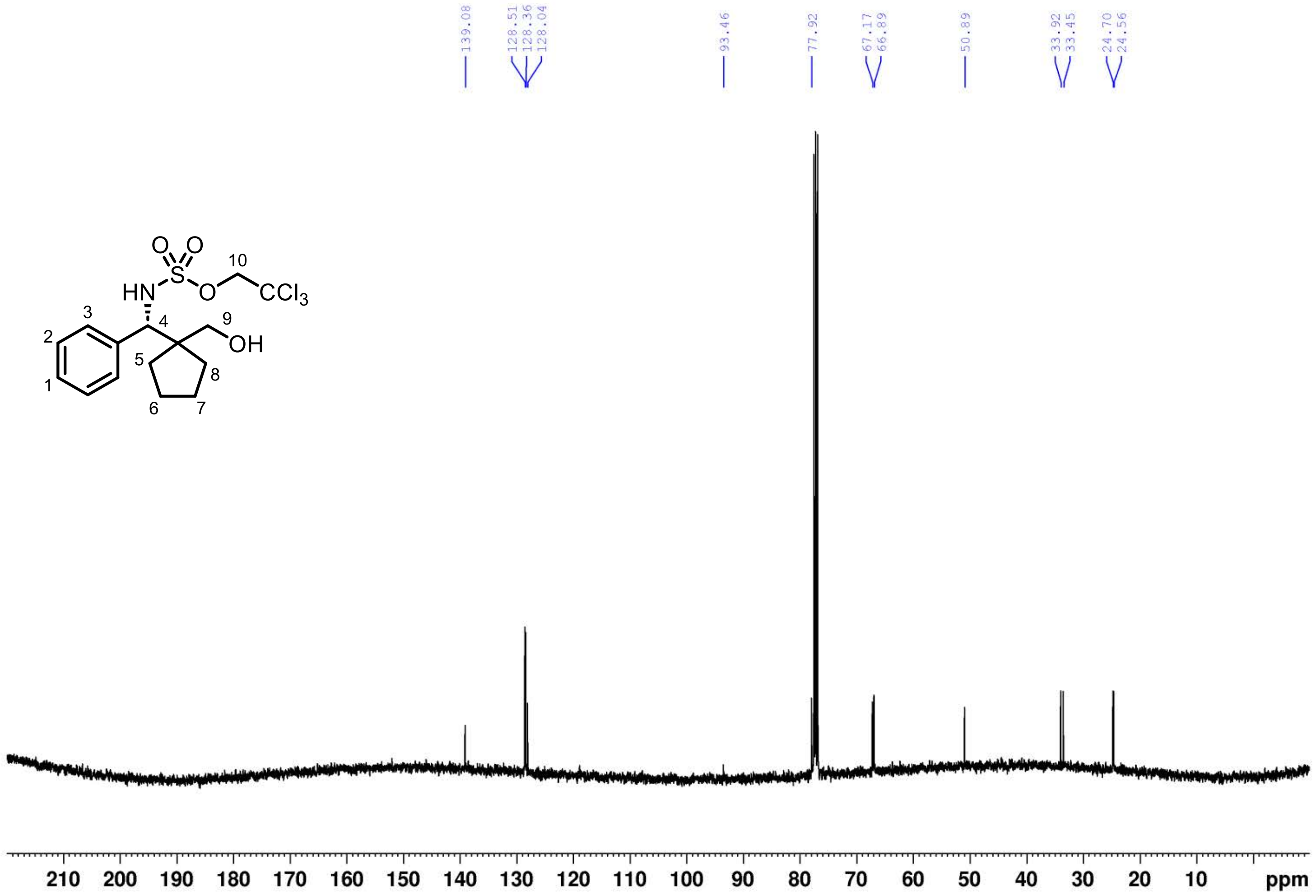

^{1}H NMR (700 MHz, $CDCl_3$) for *2,2,2-trichloroethyl (R)-(3-hydroxy-3-methyl-1-phenylbutyl)sulfamate* (**2r**)

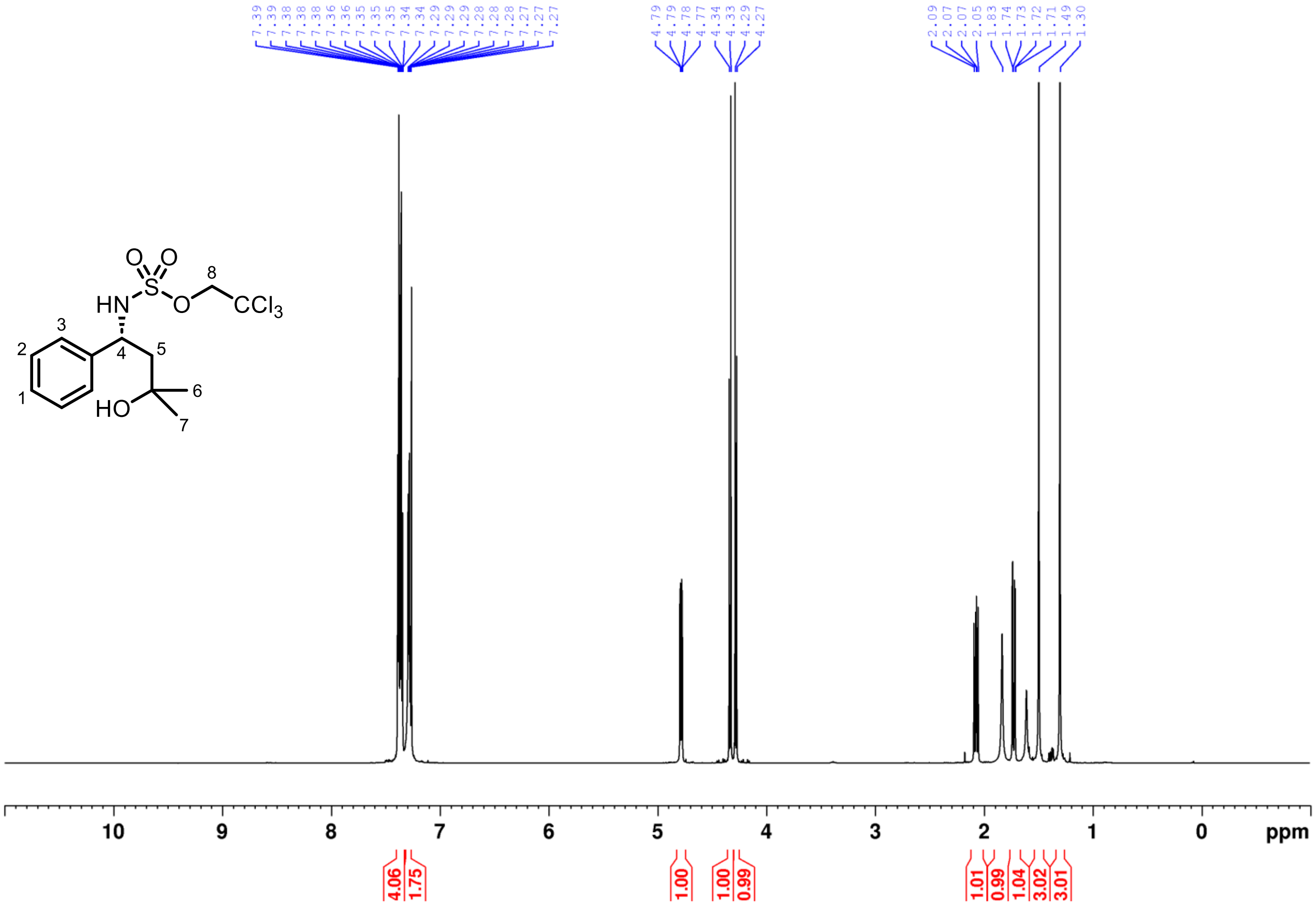

^{13}C NMR (176 MHz, $CDCl_3$) for *2,2,2-trichloroethyl (R)-(3-hydroxy-3-methyl-1-phenylbutyl)sulfamate* (**2r**)

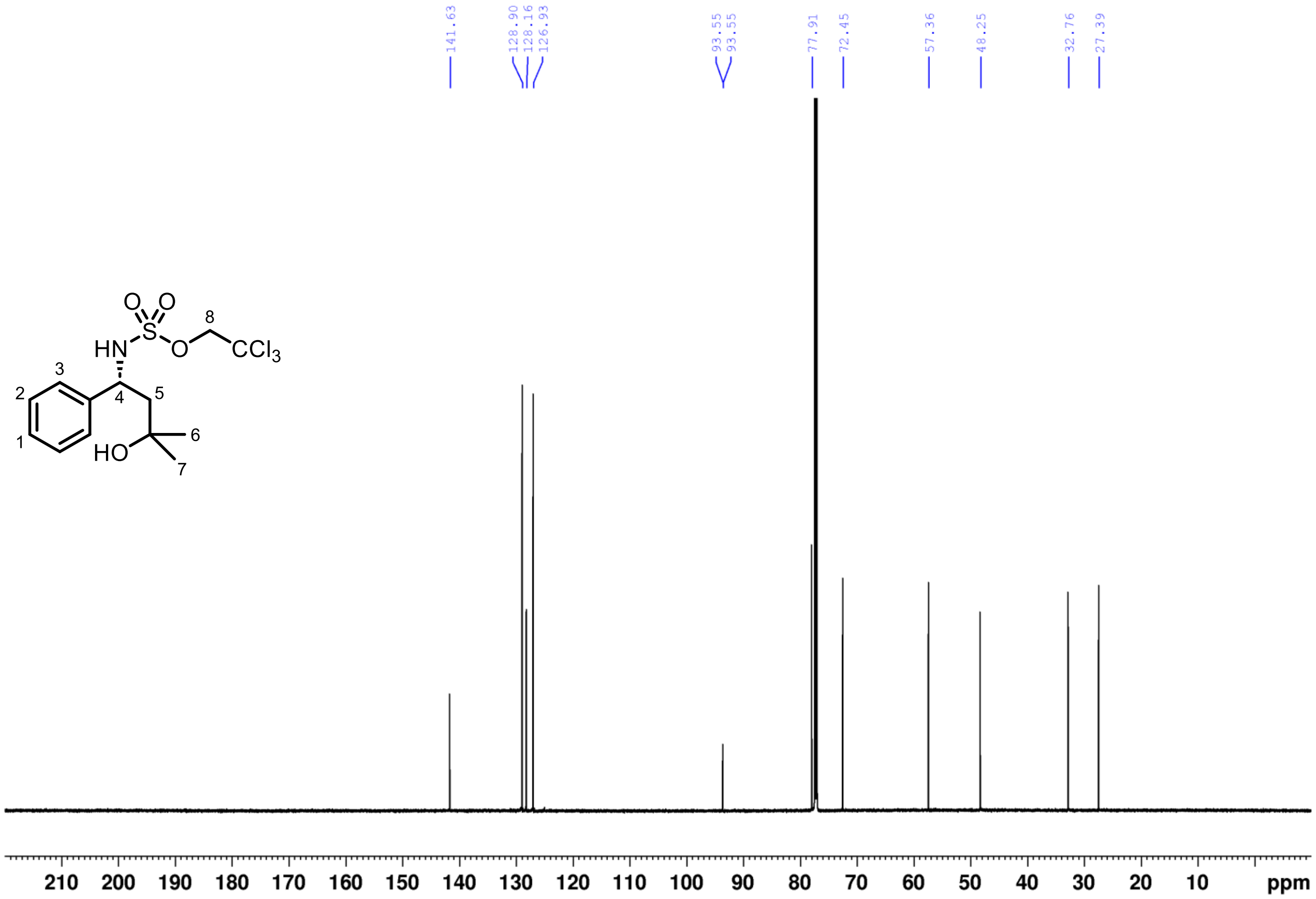

^{1}H NMR (400 MHz, $CDCl_3$) for *2,2,2-trichloroethyl (R)-(2-(1-hydroxycyclobutyl)-1-phenylethyl)sulfamate* (**2s**)

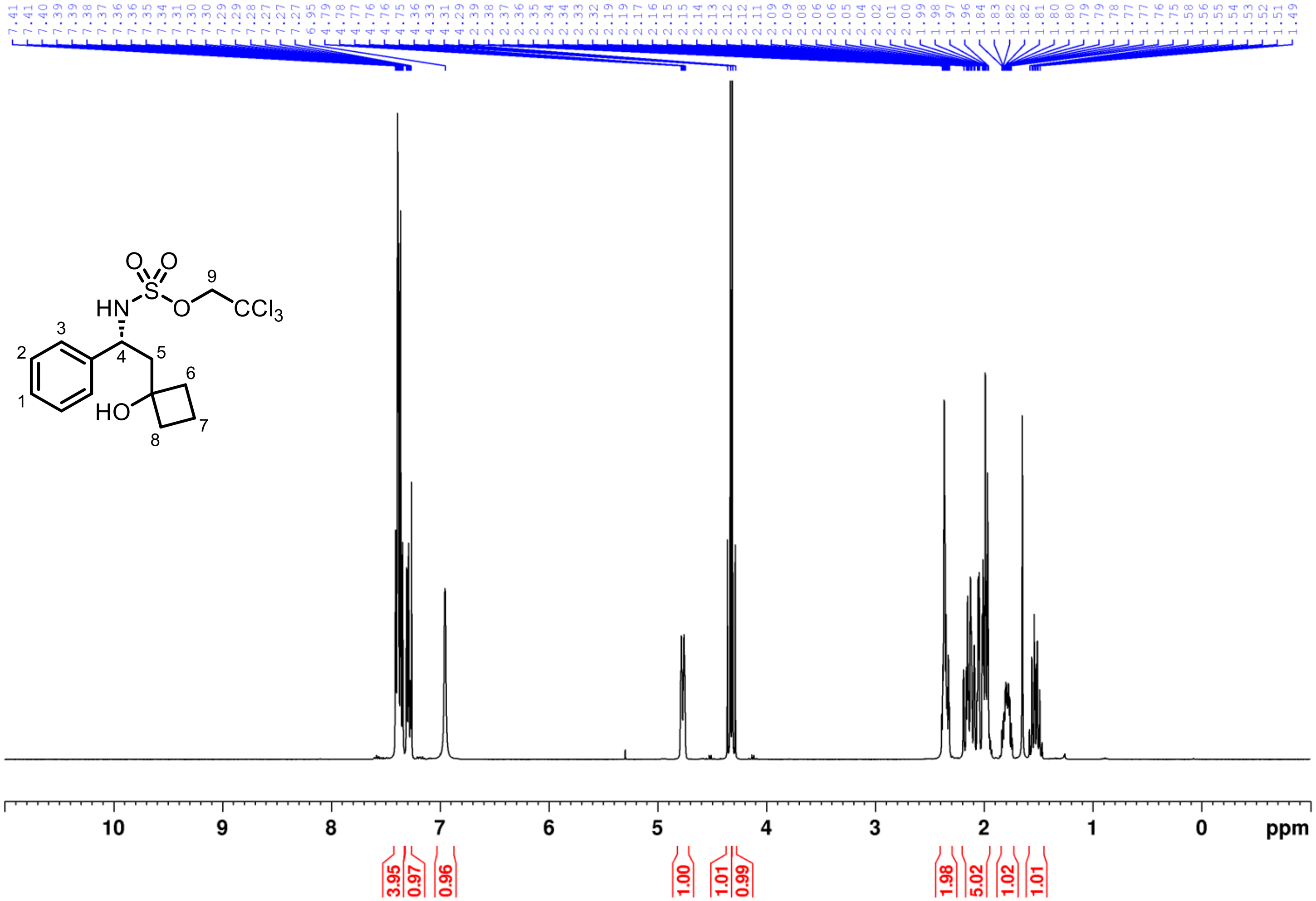

^{13}C NMR (101 MHz, CDCl$_3$) for *2,2,2-trichloroethyl (R)-(2-(1-hydroxycyclobutyl)-1-phenylethyl)sulfamate* (**2s**)

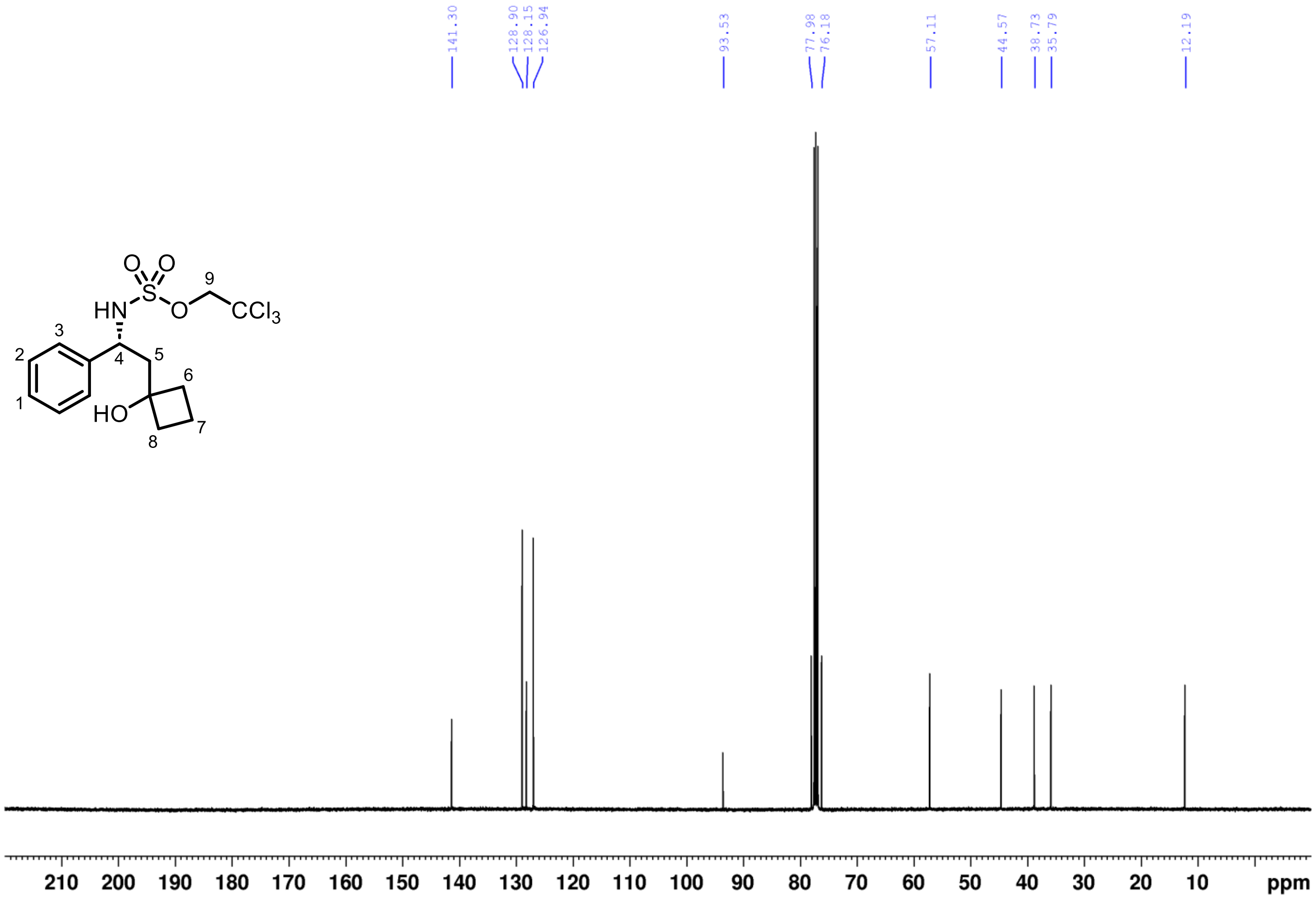

^{1}H NMR (700 MHz, CDCl$_3$) for *(R)-3-amino-3-phenylpropan-1-ol* (**3**)

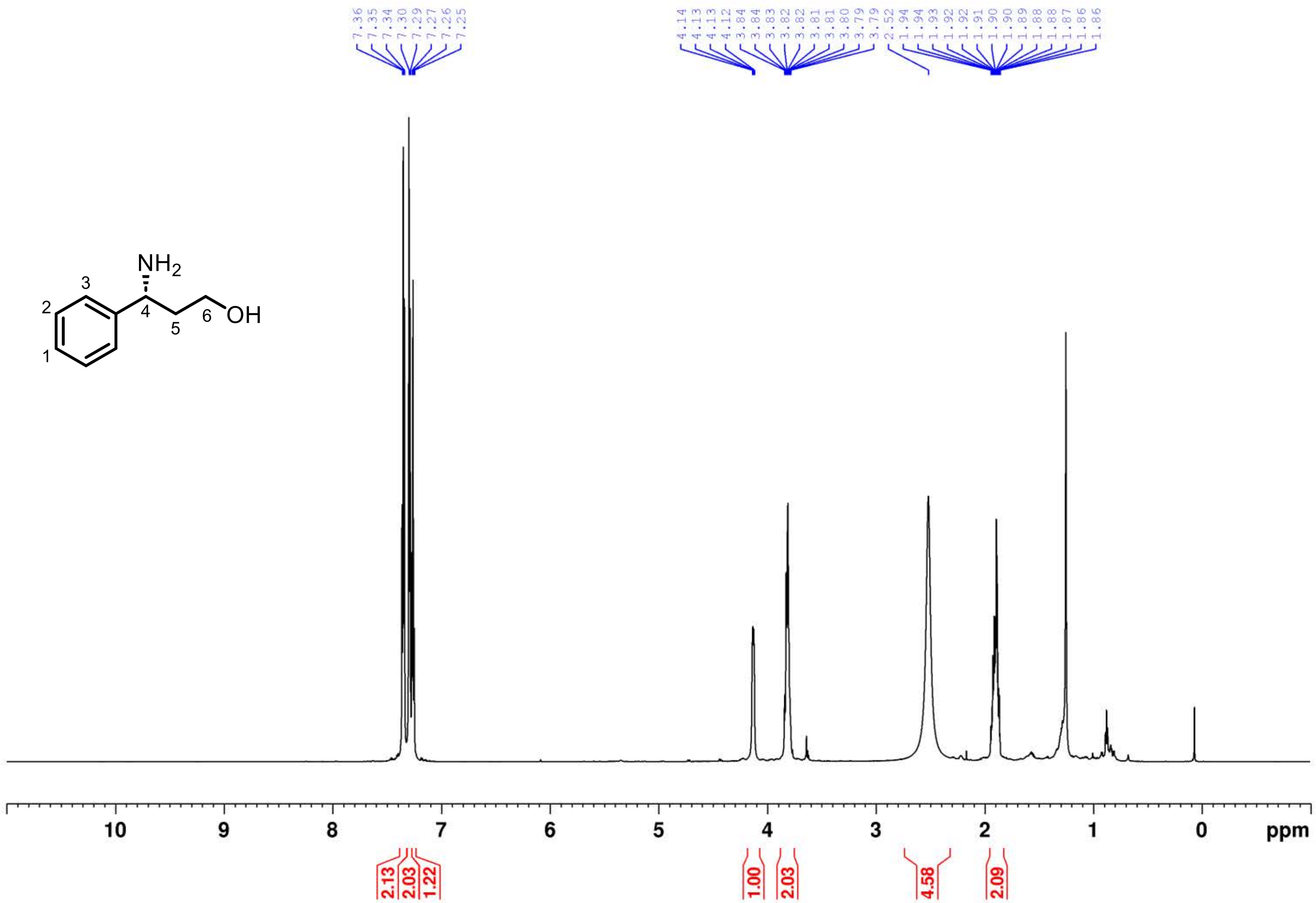

^{13}C NMR (176 MHz, $CDCl_3$) for *(R)-3-amino-3-phenylpropan-1-ol* (**3**)

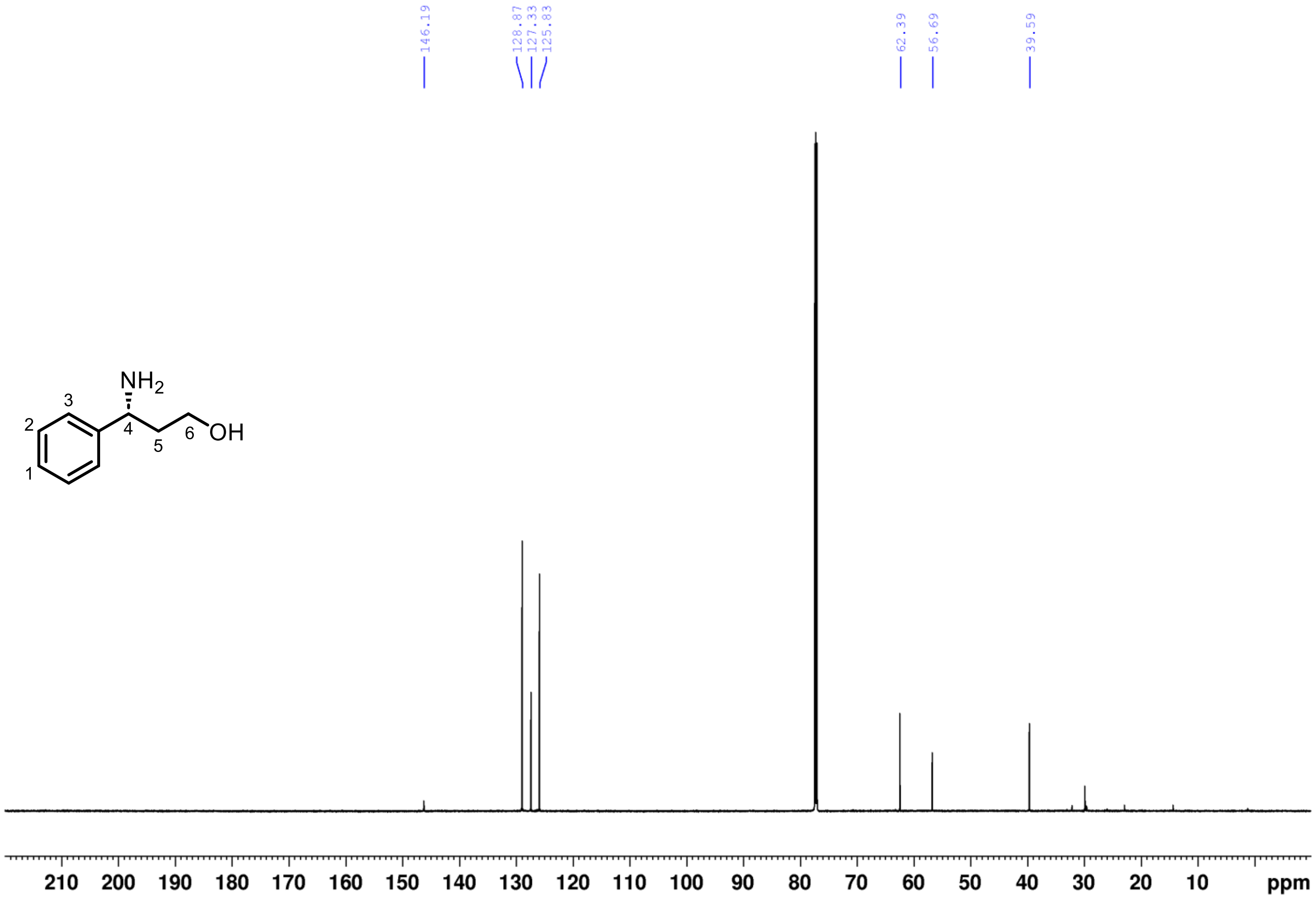

^{1}H NMR (400 MHz, CD_3OD) for *(R)-3-hydroxy-1-phenylpropan-1-aminium chloride* (**3•HCl**)

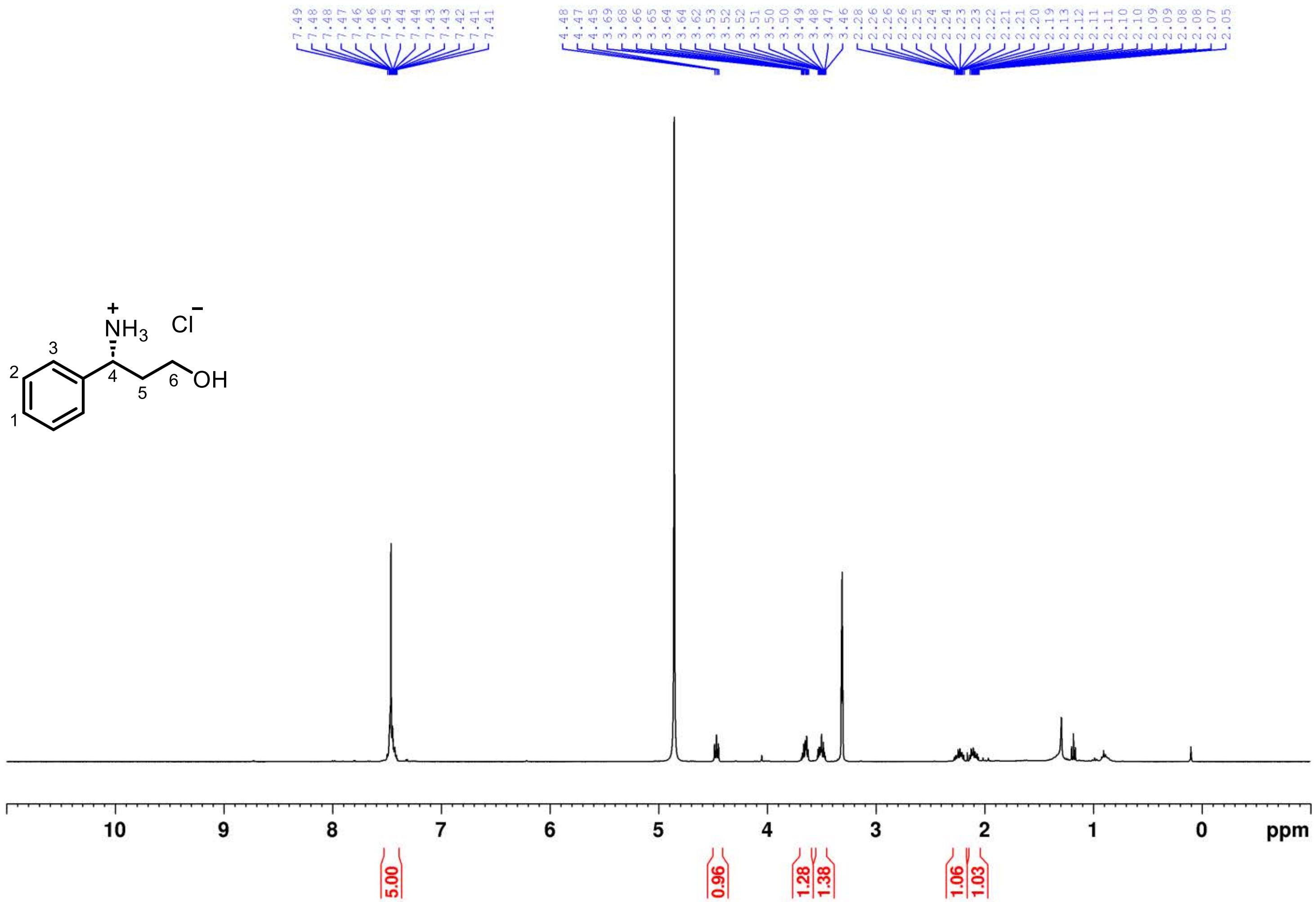

^{13}C NMR (176 MHz, CD_3OD) for *(R)-3-hydroxy-1-phenylpropan-1-aminium chloride* (**3•HCl**)

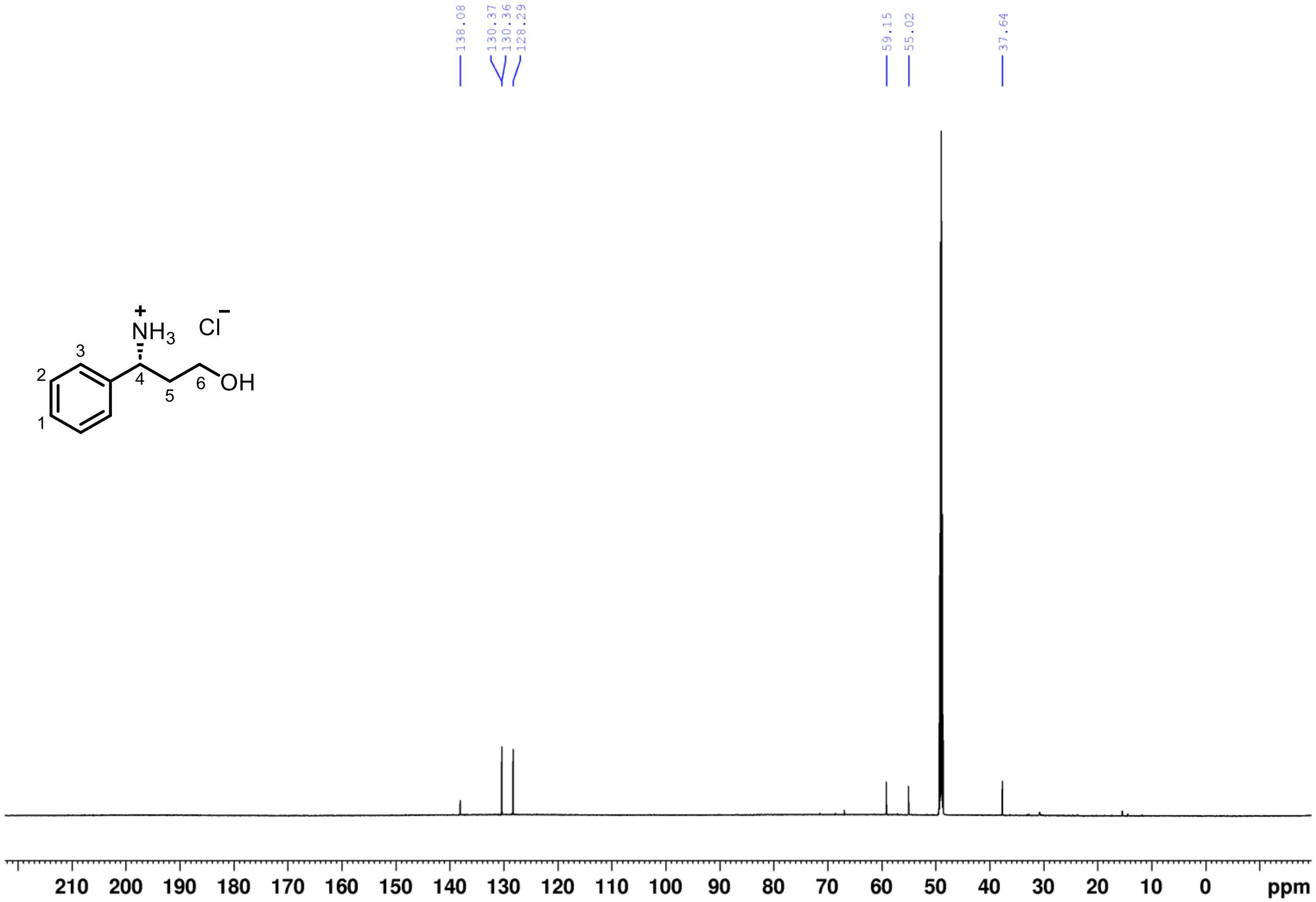

^{1}H NMR (400 MHz, $(CD_3)_2CO$) for *2,2,2-trichloroethyl (R)-(3-hydroxy-1-(thiophen-2-yl)propyl)sulfamate* (**2t**)

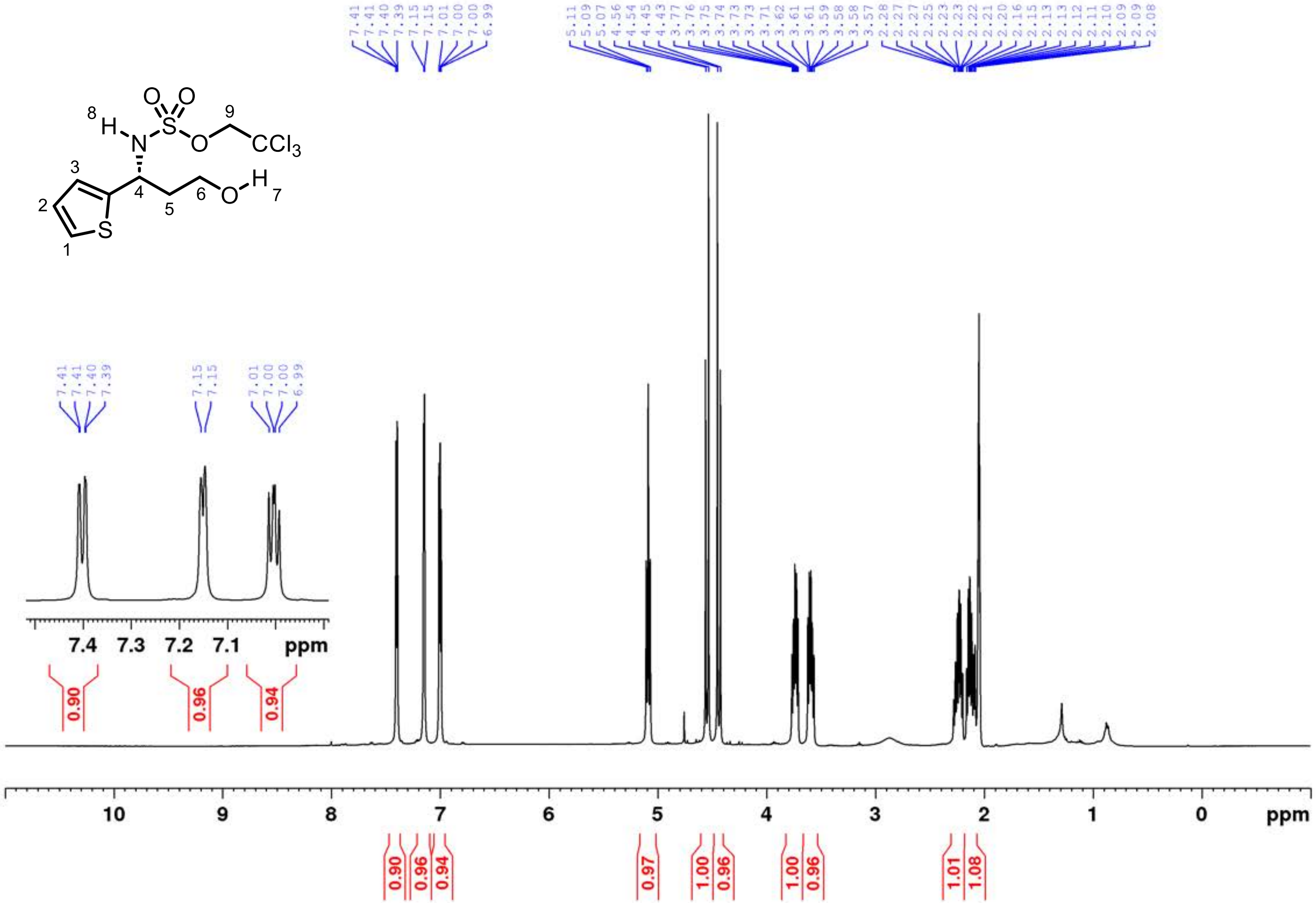

^{13}C NMR (101 MHz, $(CD_3)_2CO$) for *2,2,2-trichloroethyl (R)-(3-hydroxy-1-(thiophen-2-yl)propyl)sulfamate* (**2t**)

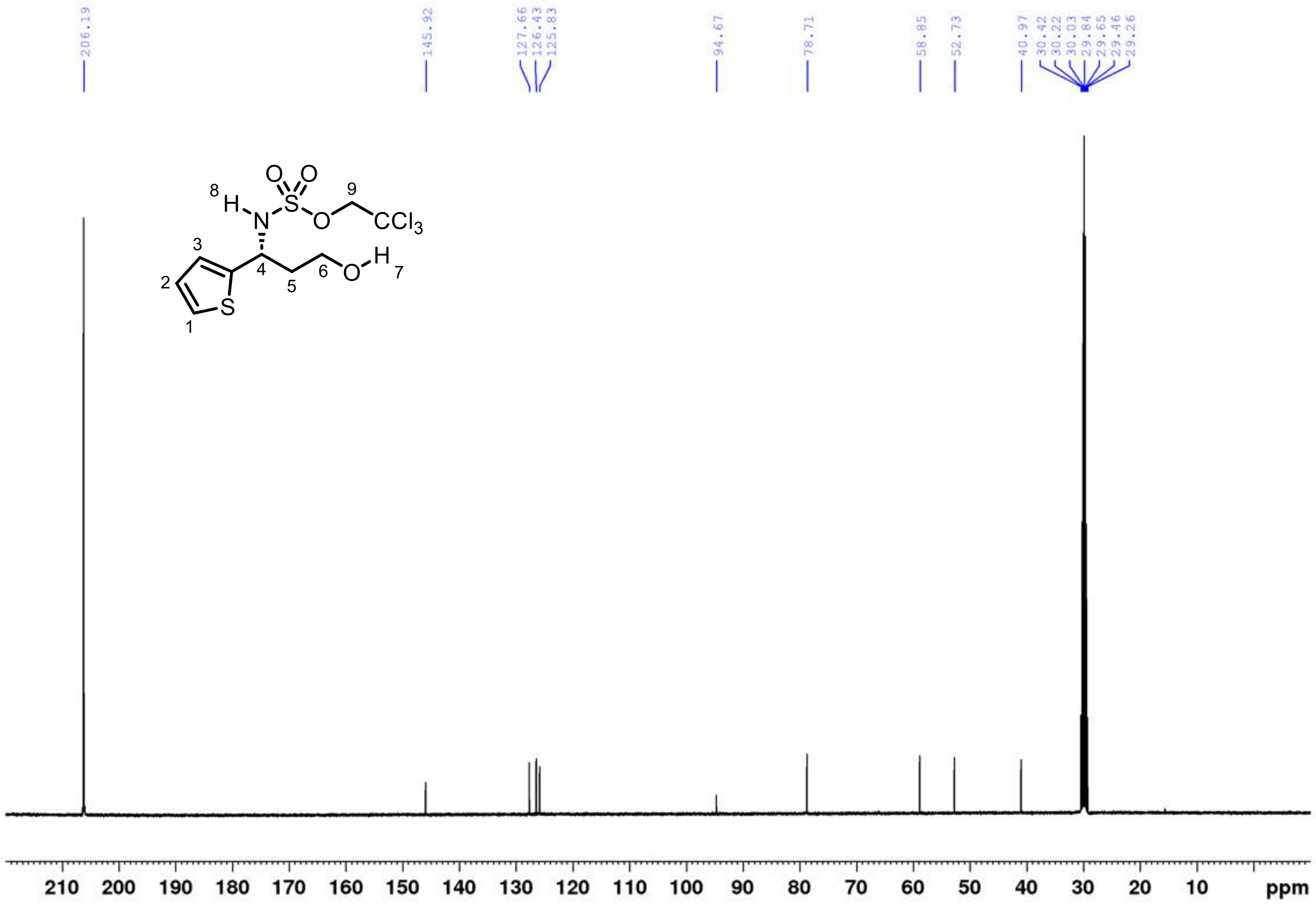

¹H NMR (700 MHz, $(CD_3)_2SO$) for *2,2,2-trichloroethyl (R)-(1-(benzo[b]thiophen-5-yl)-3-hydroxypropyl)sulfamate* (**2u**)

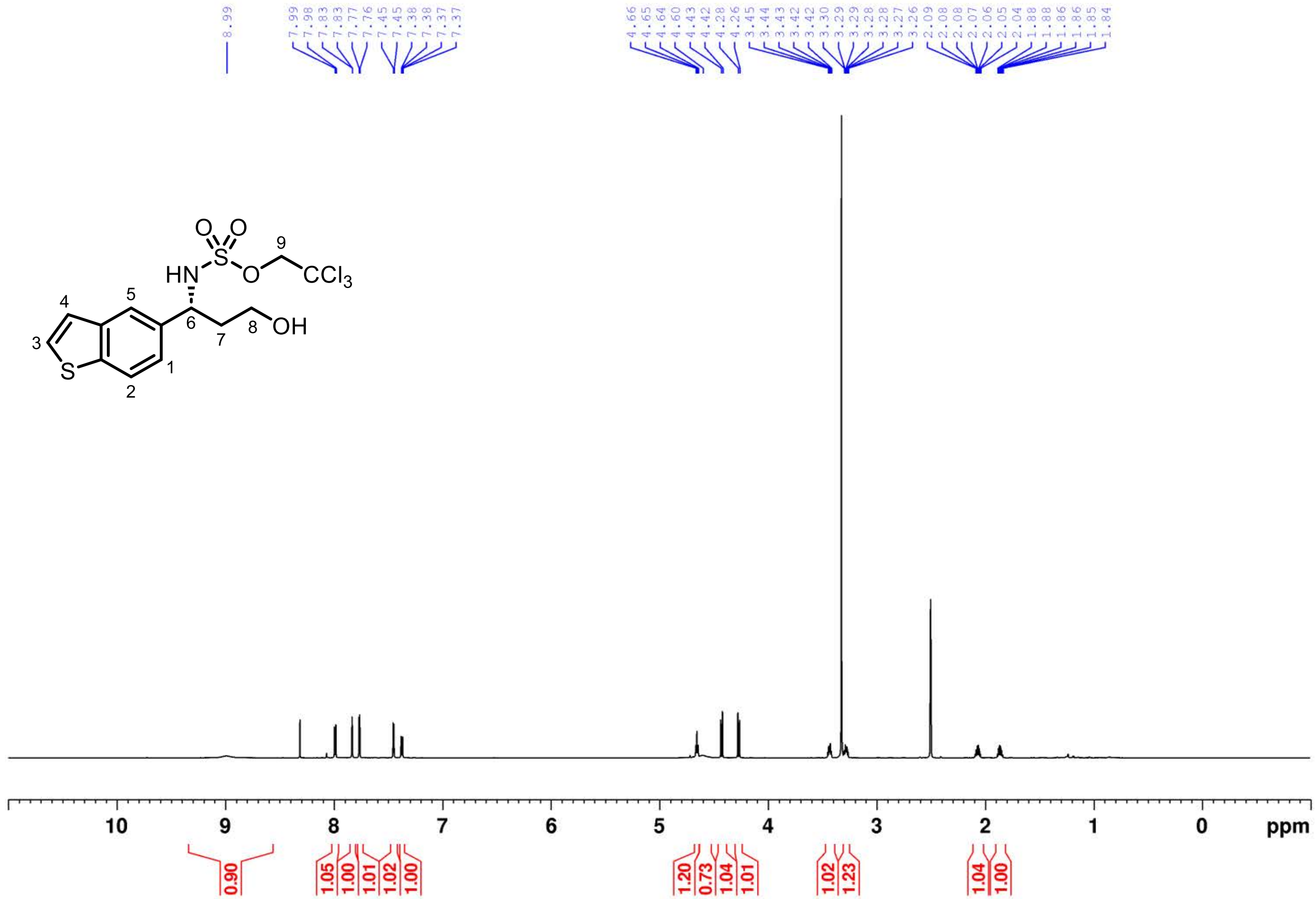

^{13}C NMR (176 MHz, $(CD_3)_2SO$) for *2,2,2-trichloroethyl (R)-(1-(benzo[b]thiophen-5-yl)-3-hydroxypropyl)sulfamate* (**2u**)

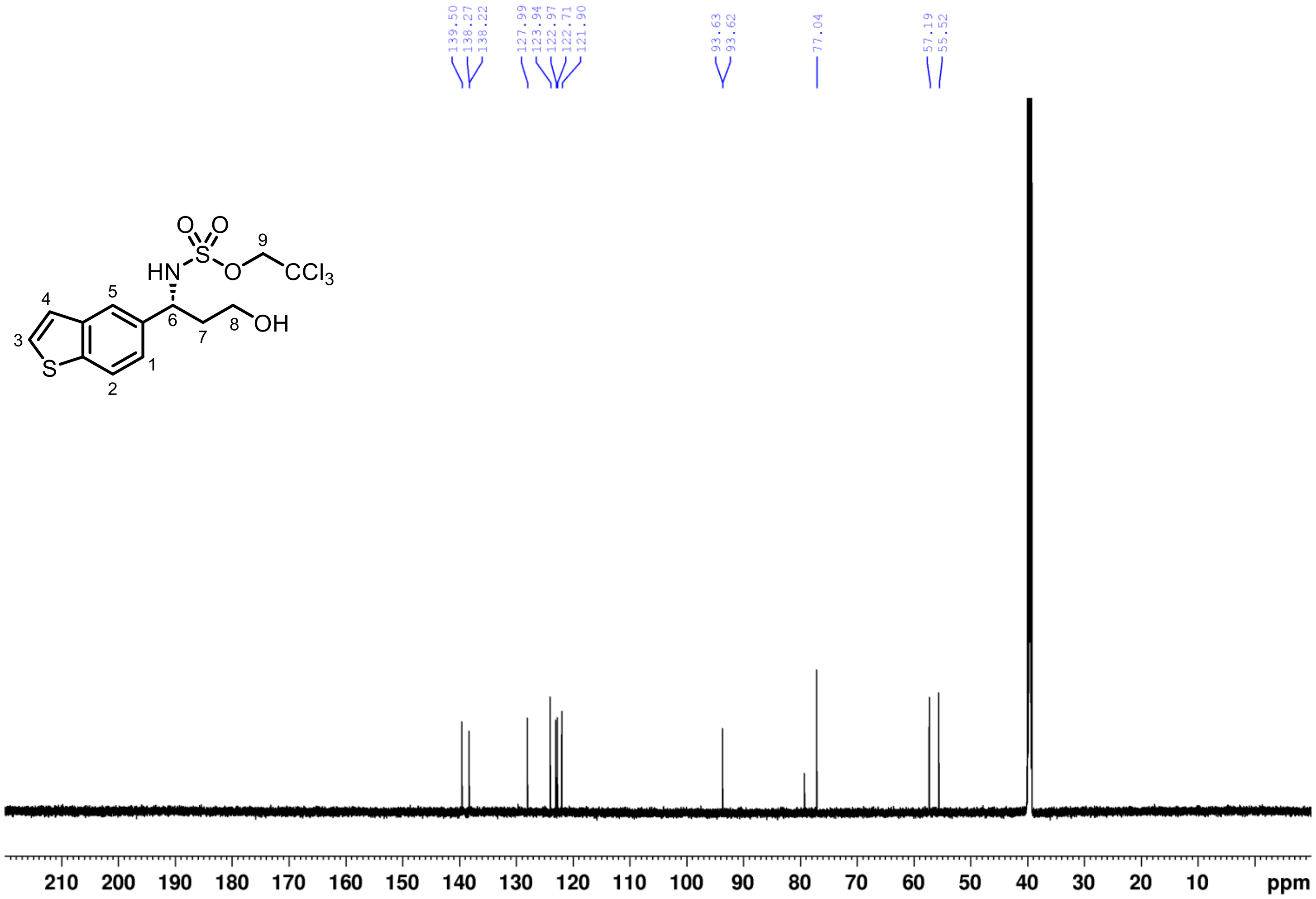

^{1}H NMR (700 MHz, CD_3OD) for *2,2,2-trichloroethyl (R)-(1-(benzo[b]thiophen-2-yl)-3-hydroxypropyl)sulfamate* (**2v**)

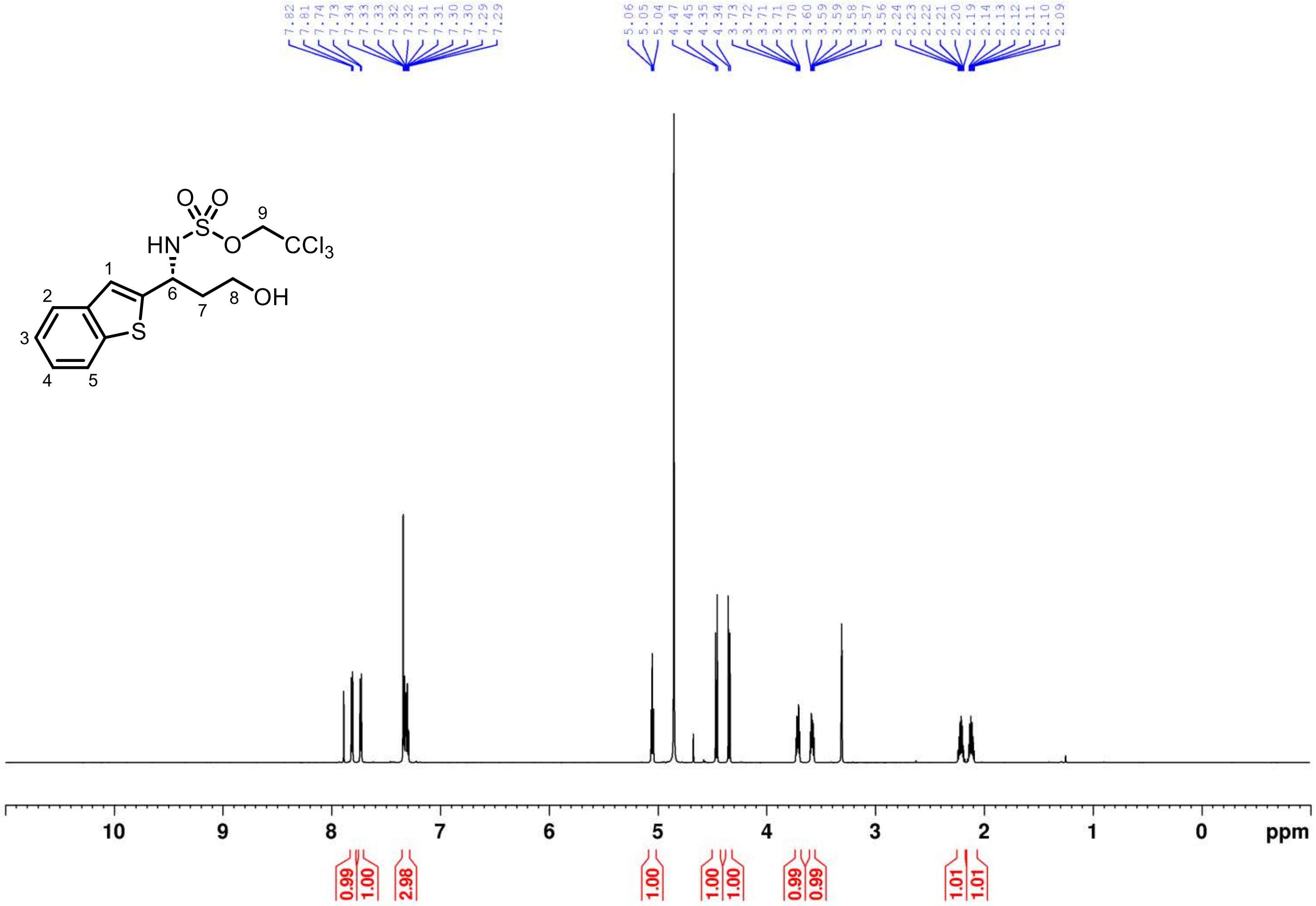

^{13}C NMR (176 MHz, CD_3OD) for *2,2,2-trichloroethyl (R)-(1-(benzo[b]thiophen-2-yl)-3-hydroxypropyl)sulfamate* (**2v**)

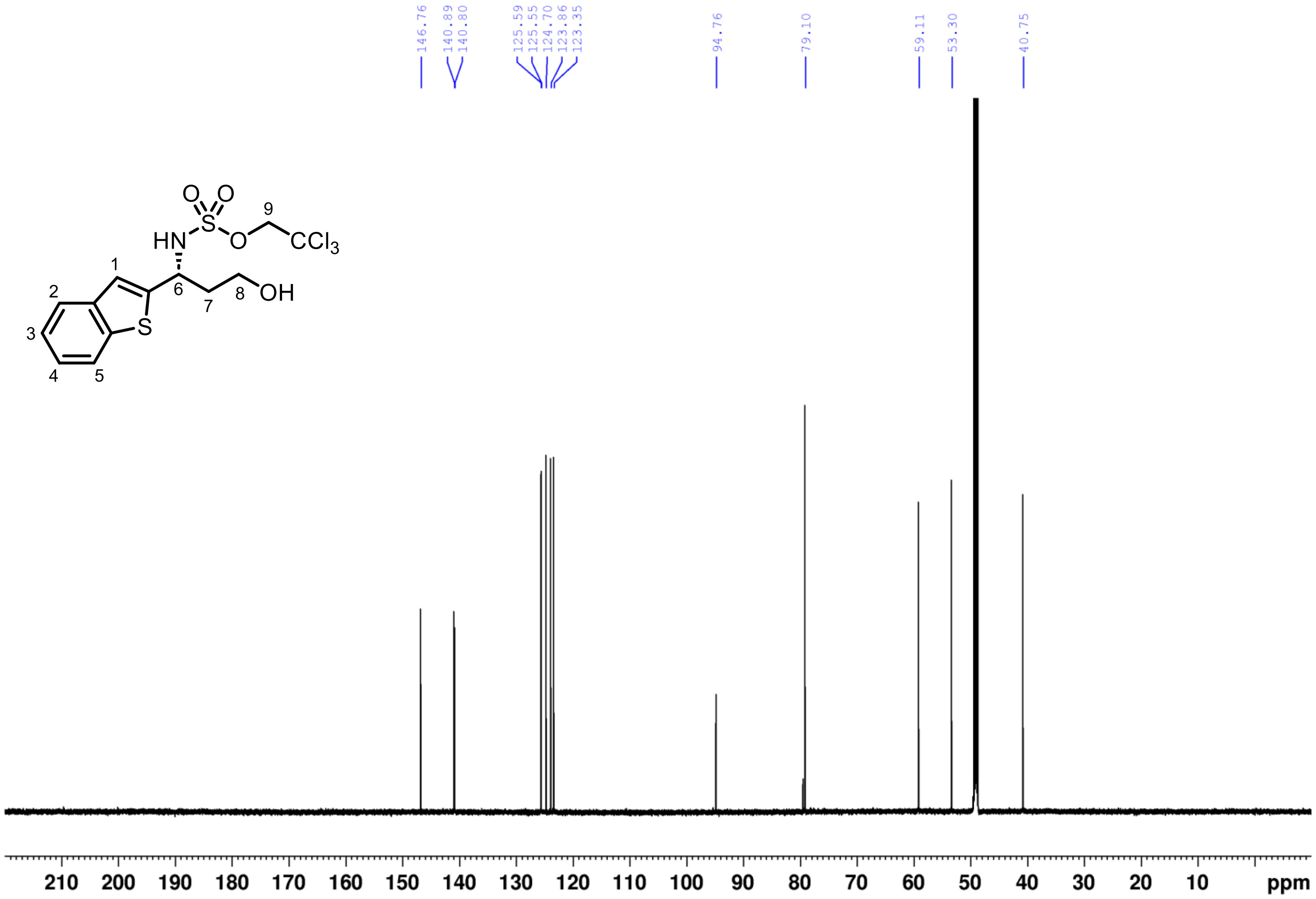

¹H NMR (400 MHz, CDCl₃) for *2,2,2-trichloroethyl (R)-(3-hydroxy-1-(1-(phenylsulfonyl)-1H-pyrrol-3-yl)propyl)sulfamate* (**2w**)

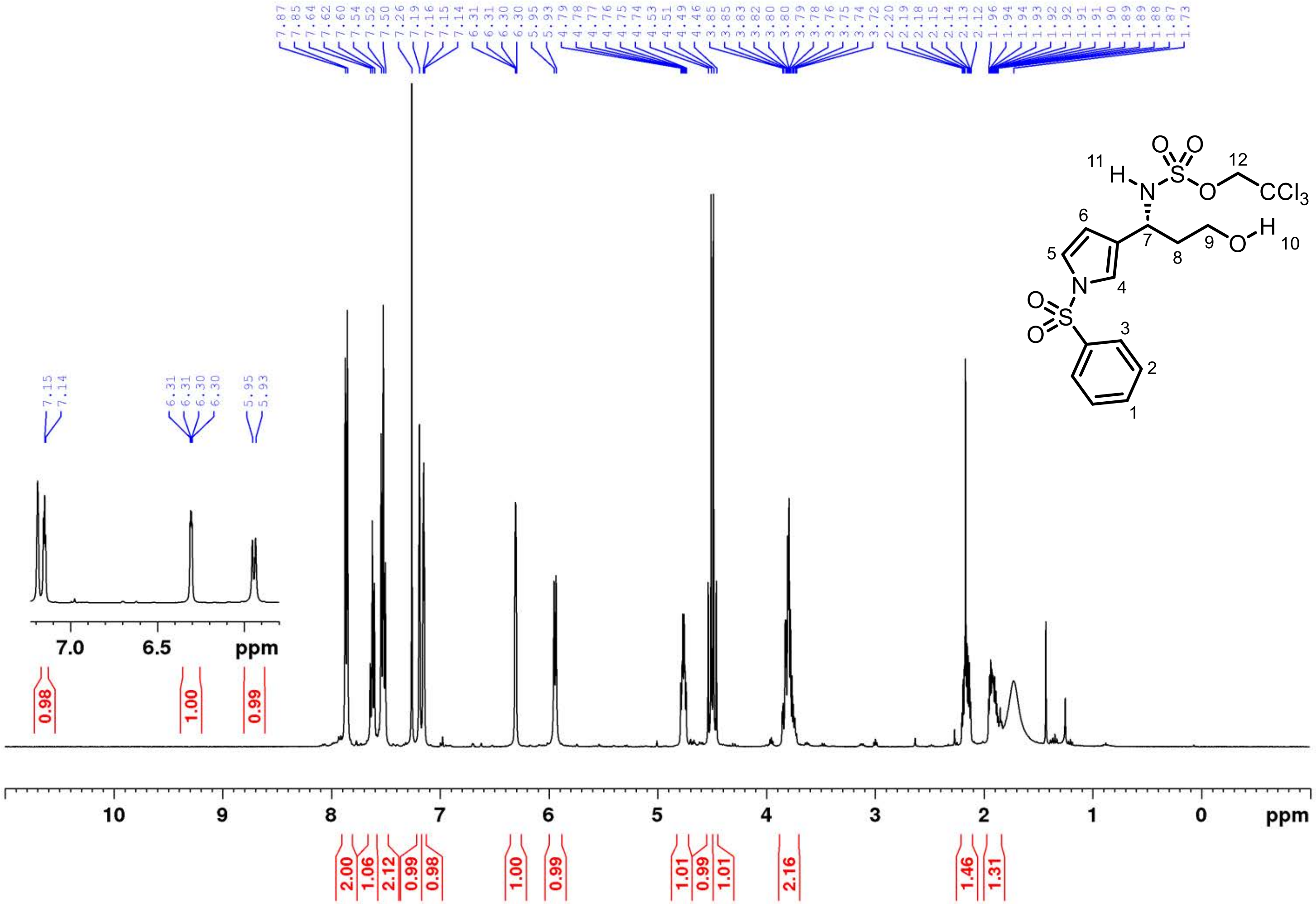

^{13}C NMR (176 MHz, $CDCl_3$) for *2,2,2-trichloroethyl (R)-(3-hydroxy-1-(1-(phenylsulfonyl)-1H-pyrrol-3-yl)propyl)sulfamate* (**2w**)

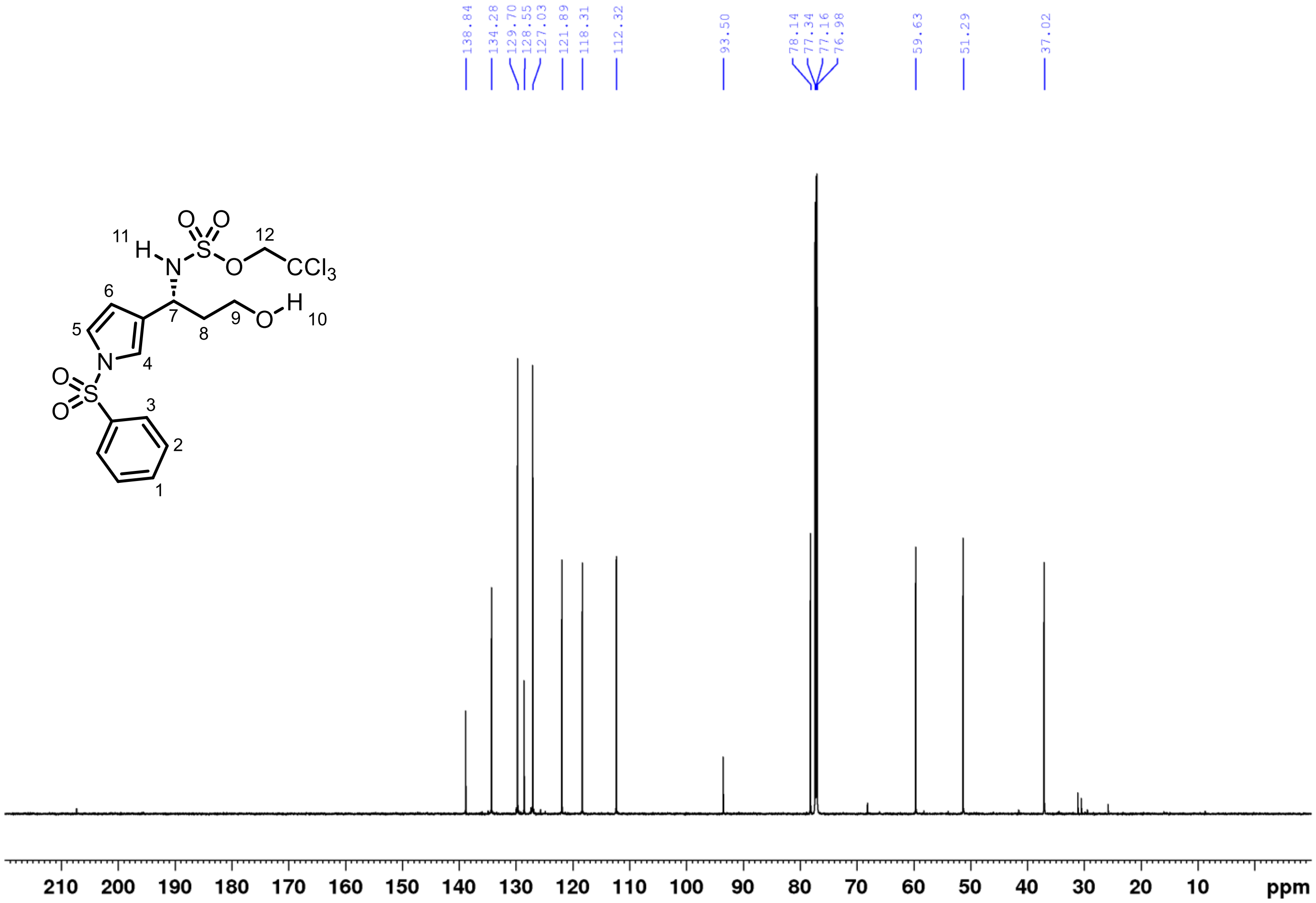

^{1}H NMR (400 MHz, $CDCl_3$) for *2,2,2-trichloroethyl (6-(phenylsulfonyl)-2,3,4,6,7,7a-hexahydropyrano[2,3-c]pyrrol-7-yl)sulfamate* (**4a**)

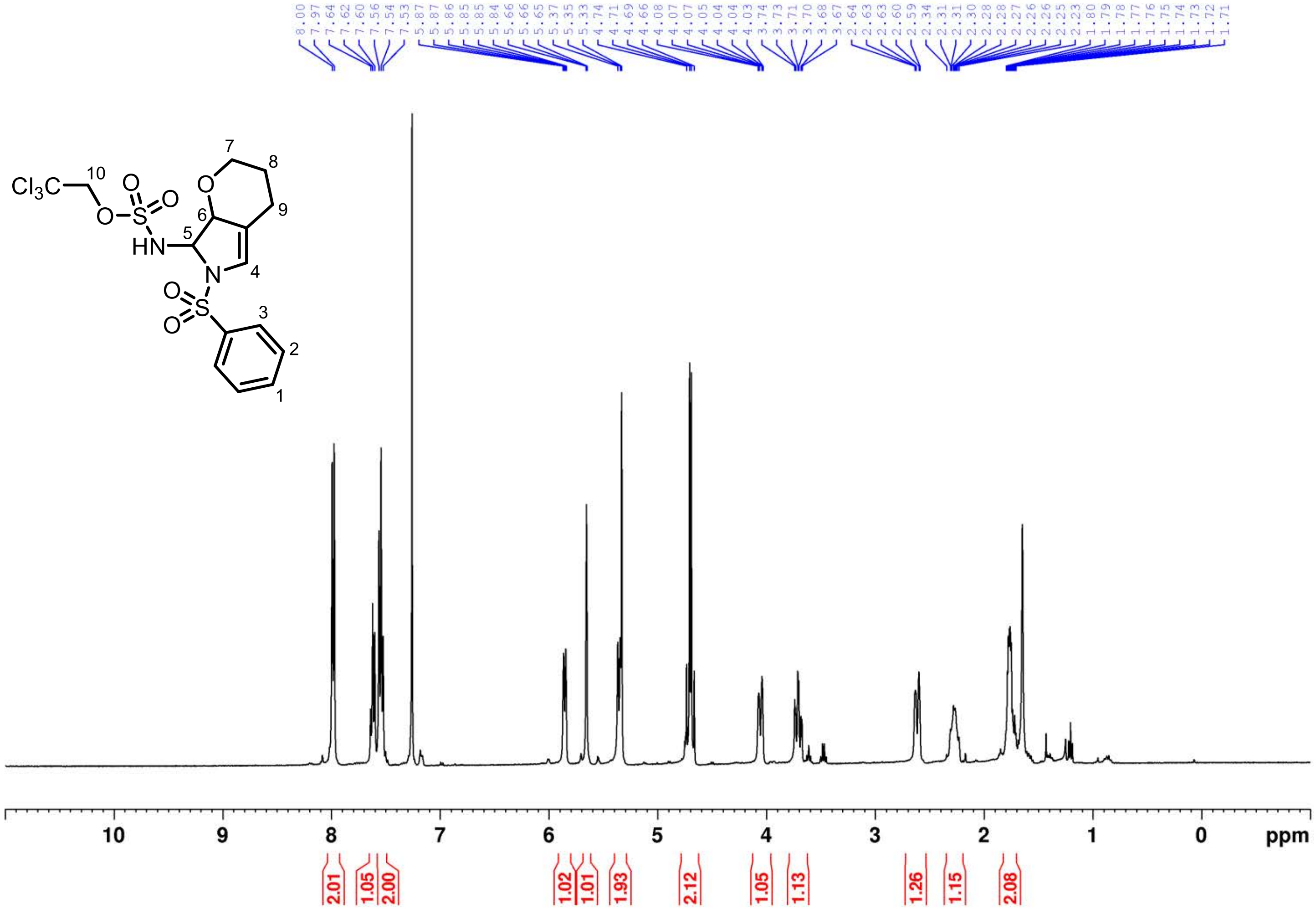

^{13}C NMR (101 MHz, CDCl$_3$) for *2,2,2-trichloroethyl (6-(phenylsulfonyl)-2,3,4,6,7,7a-hexahydropyrano[2,3-c]pyrrol-7-yl)sulfamate* (**4a**)

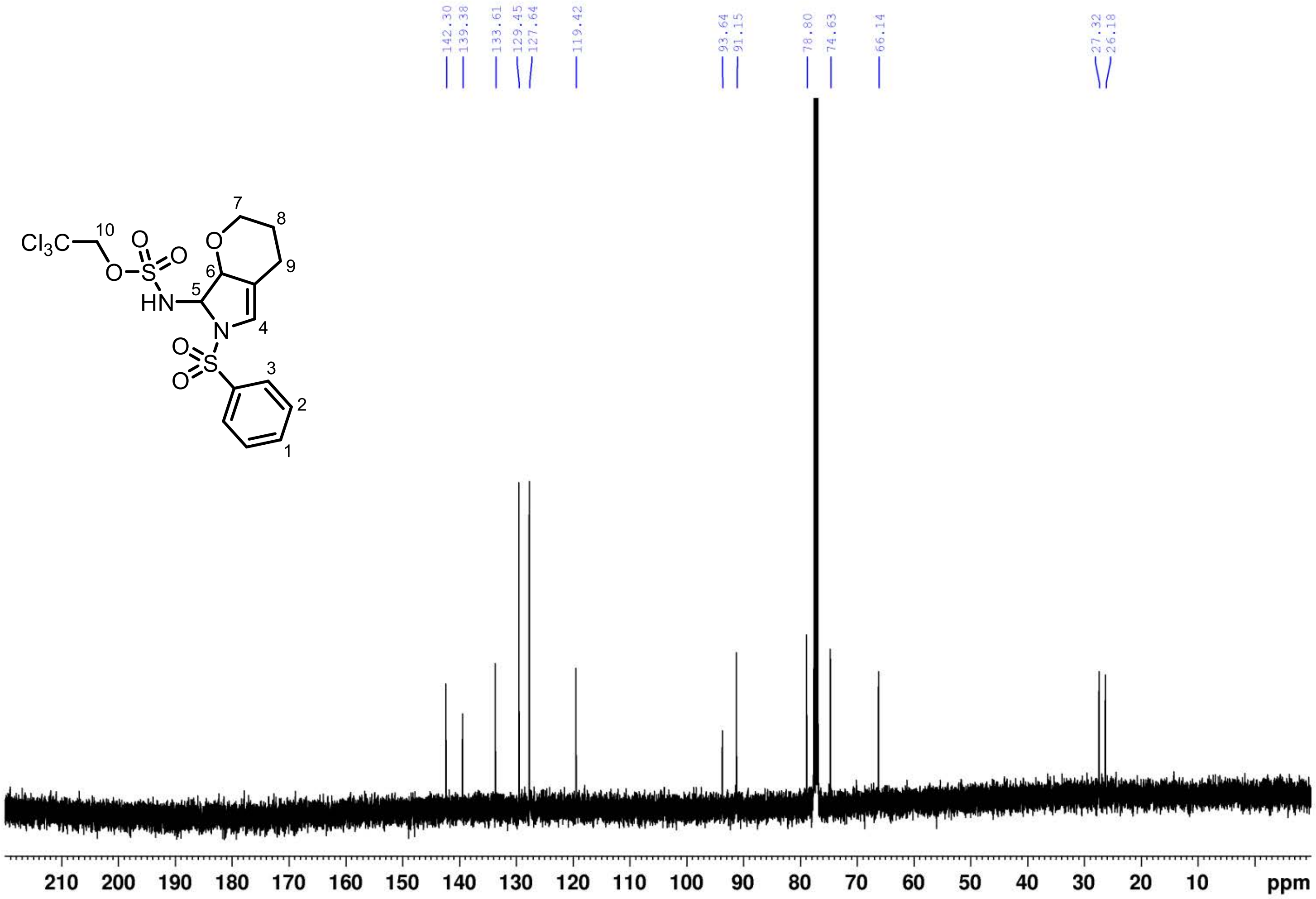

^{1}H NMR (700 MHz, CD_3OD) for *2,2,2-trichloroethyl (R)-(1-(benzofuran-2-yl)-3-hydroxypropyl)sulfamate* (**2x**)

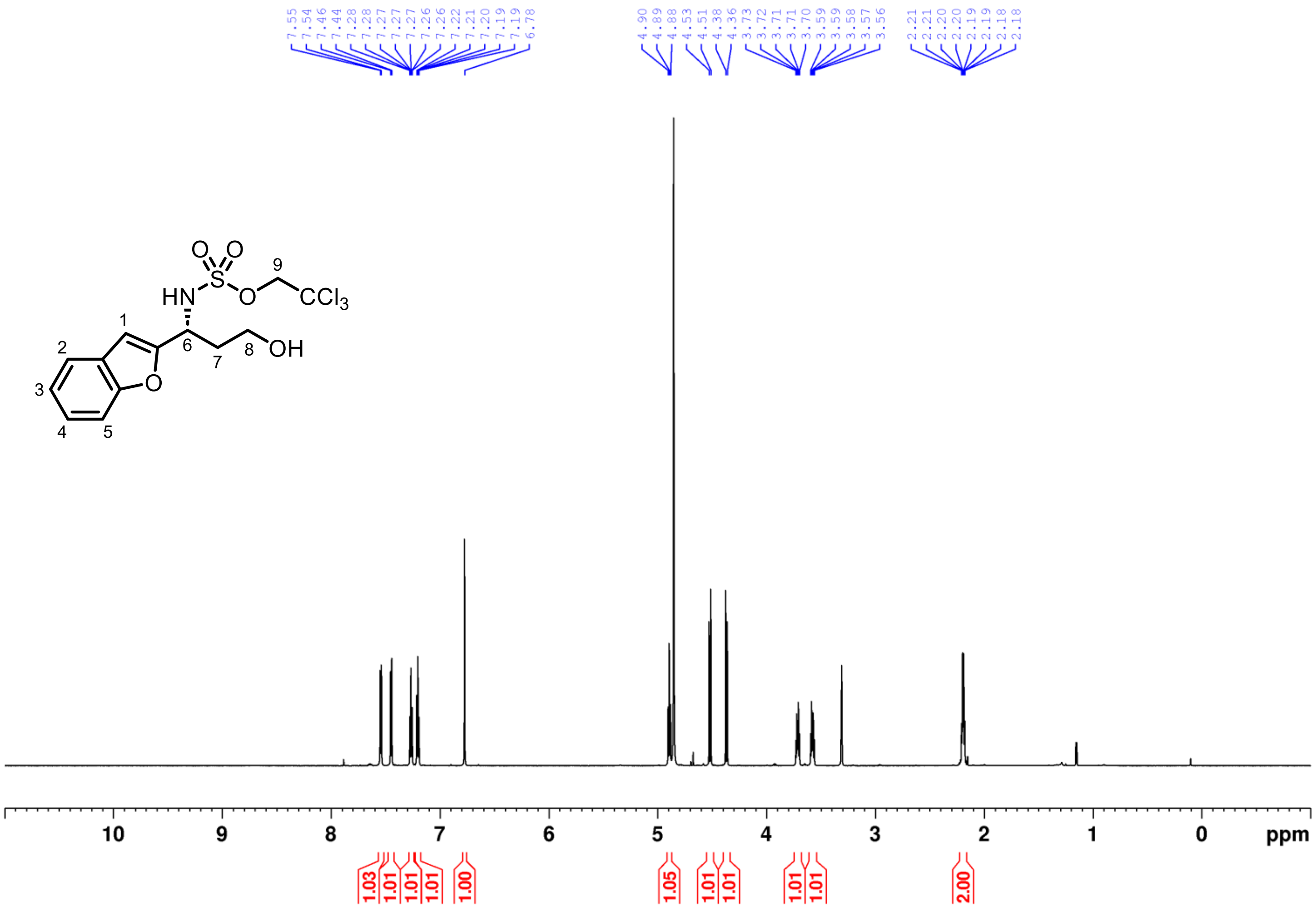

^{13}C NMR (176 MHz, CD_3OD) for *2,2,2-trichloroethyl (R)-(1-(benzofuran-2-yl)-3-hydroxypropyl)sulfamate* (**2x**)

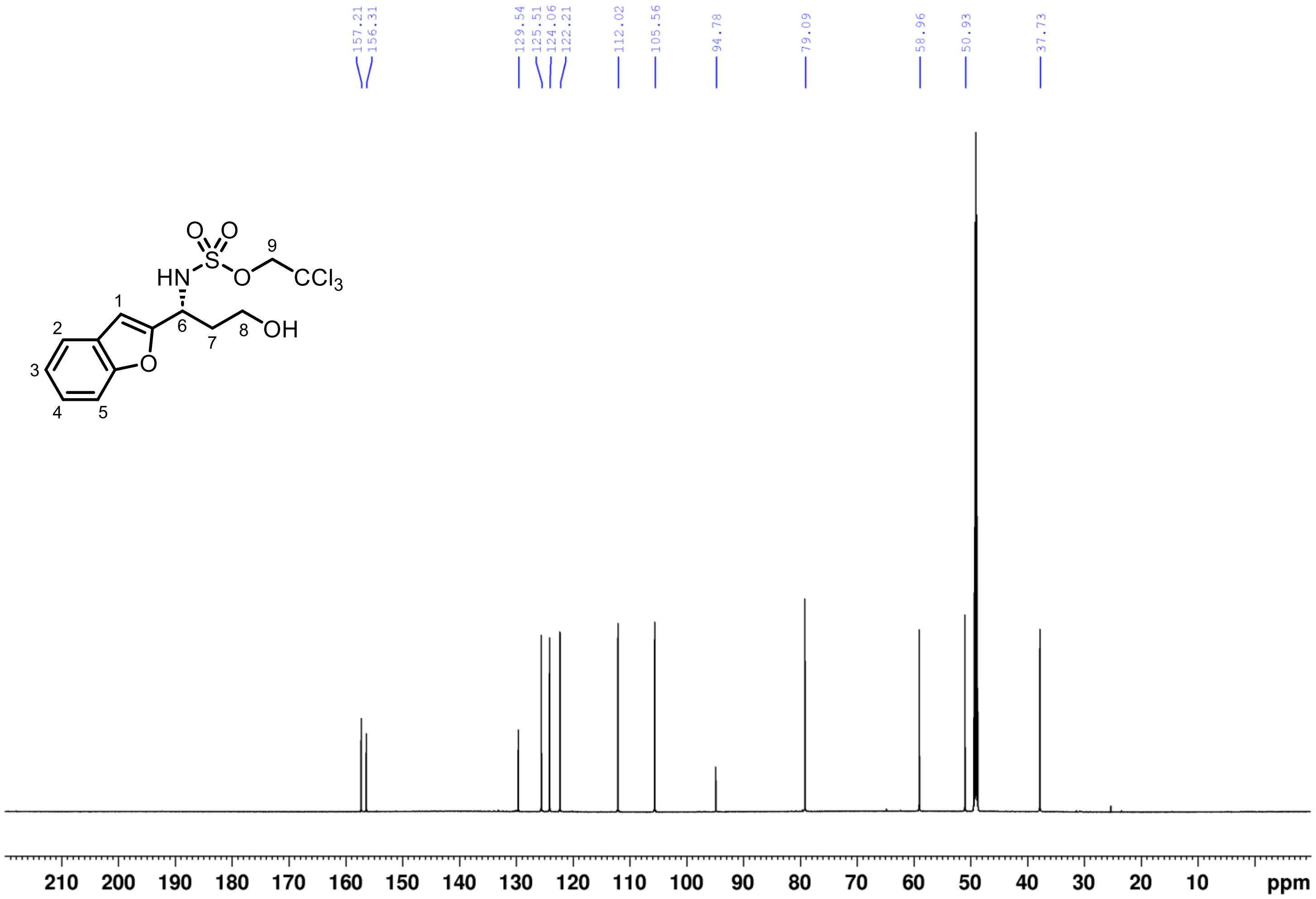

^{1}H NMR (700 MHz, $CDCl_3$) for *2,2,2-trichloroethyl (4',5'-dihydro-3H,3'H-spiro[benzofuran-2,2'-furan]-3-yl)sulfamate* (**4b**)

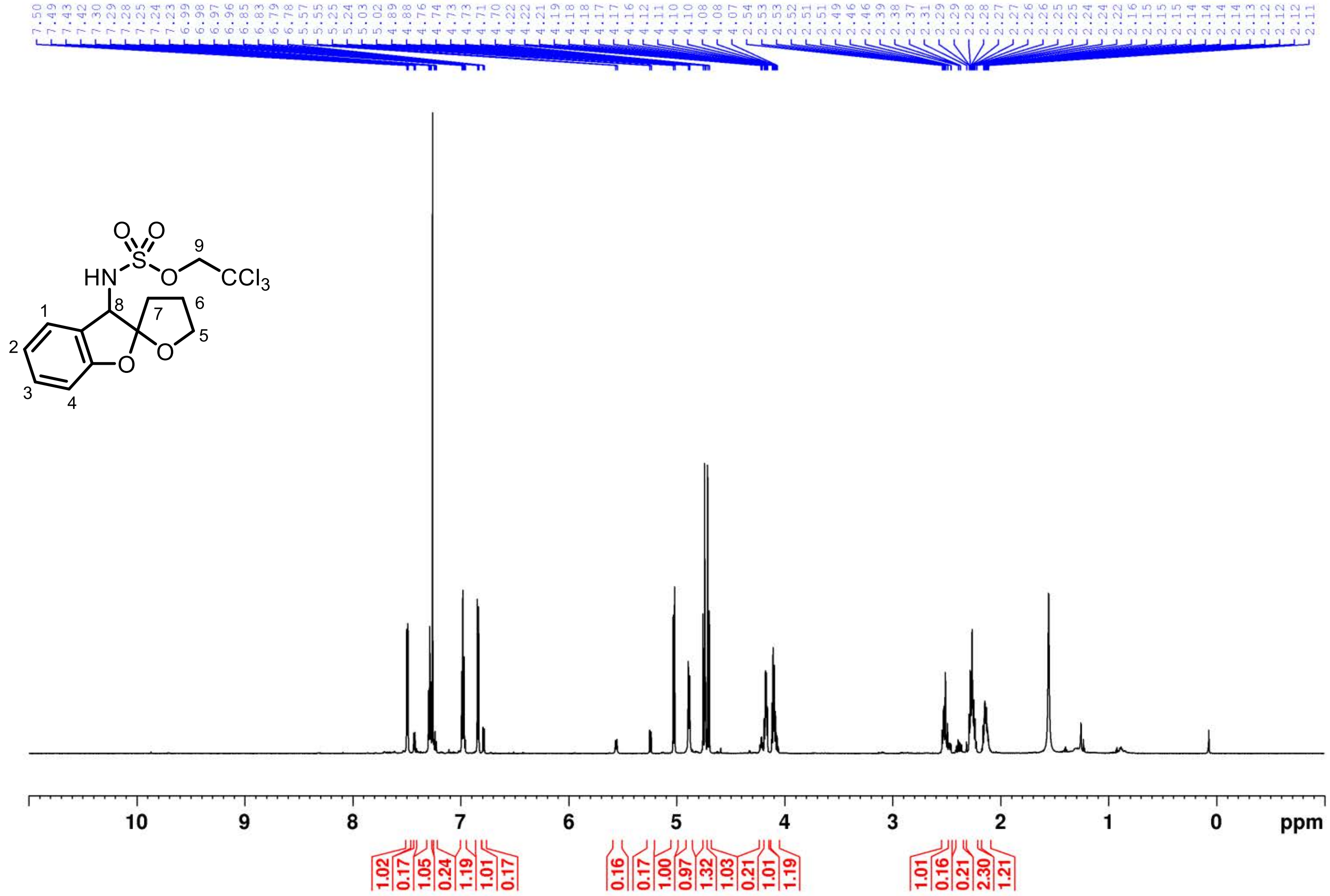

^{13}C NMR (176 MHz, CDCl$_3$) for *2,2,2-trichloroethyl (4',5'-dihydro-3H,3'H-spiro[benzofuran-2,2'-furan]-3-yl)sulfamate* (**4b**)

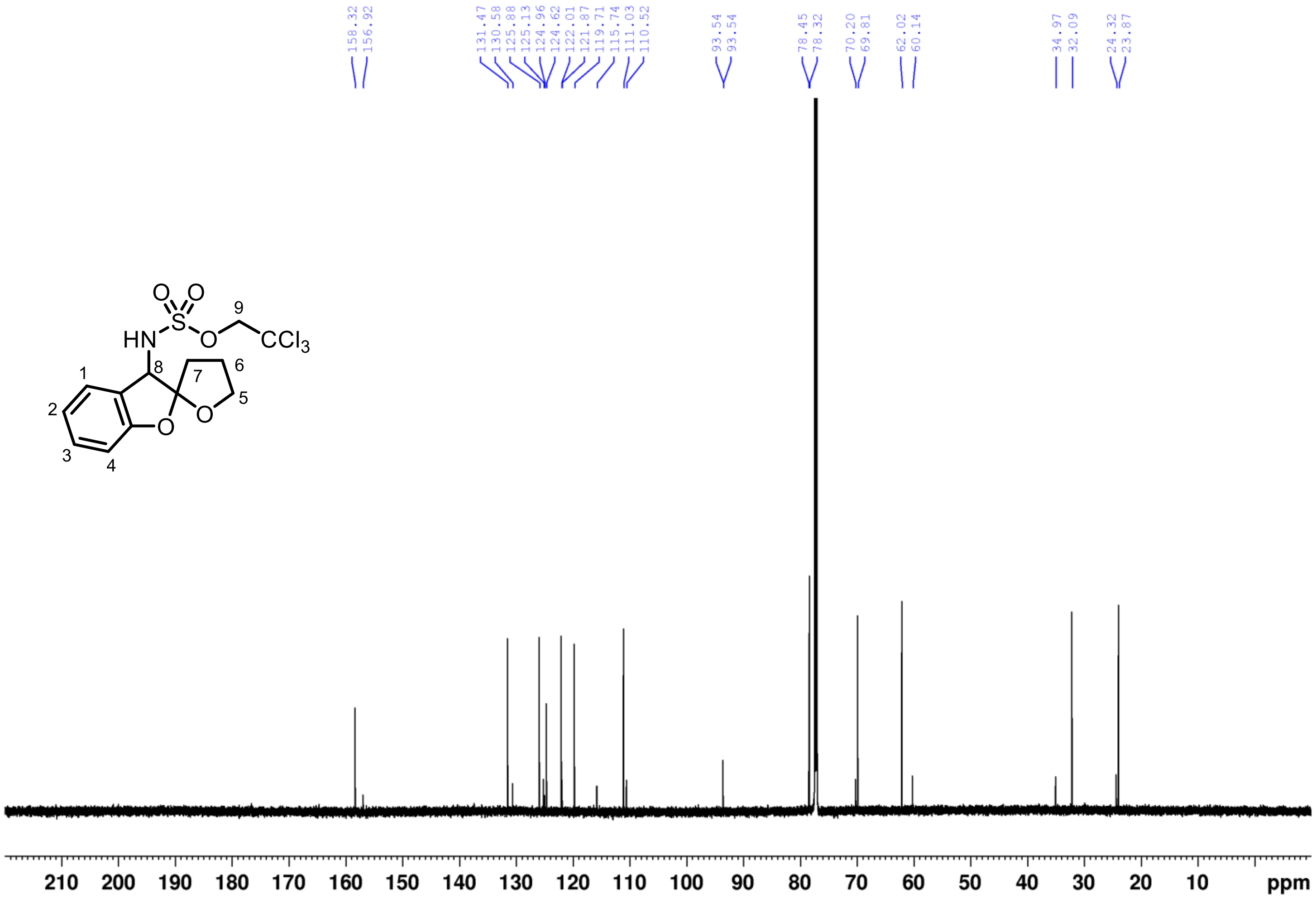

^{1}H NMR (700 MHz, CDCl$_3$) for *methyl (R)-4-(3-hydroxy-1-(((2,2,2-trichloroethoxy)sulfonyl)amino)propyl)furan-2-carboxylate* (**2y**)

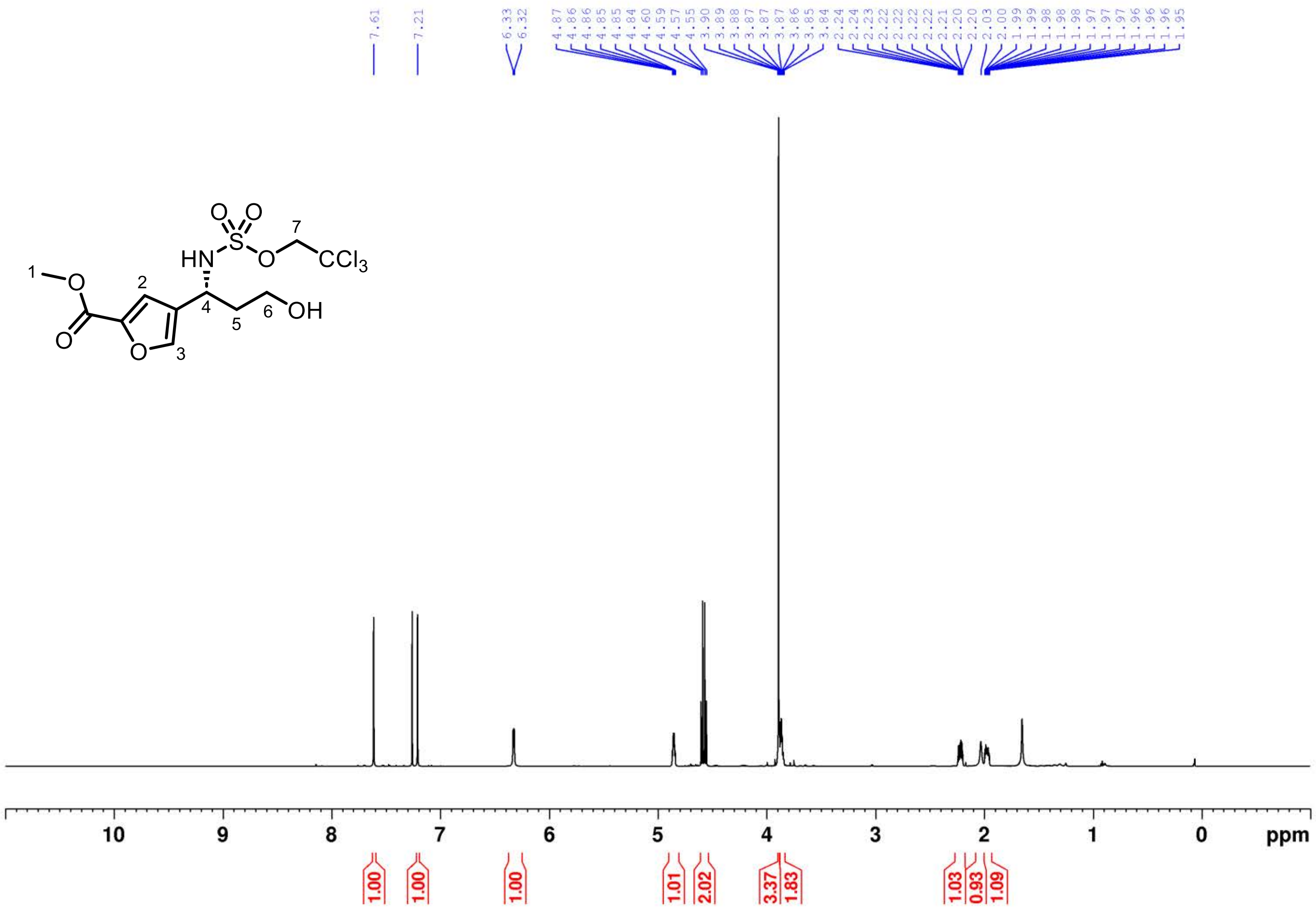

^{13}C NMR (176 MHz, CDCl$_3$) for *methyl (R)-4-(3-hydroxy-1-(((2,2,2-trichloroethoxy)sulfonyl)amino)propyl)furan-2-carboxylate* (**2y**)

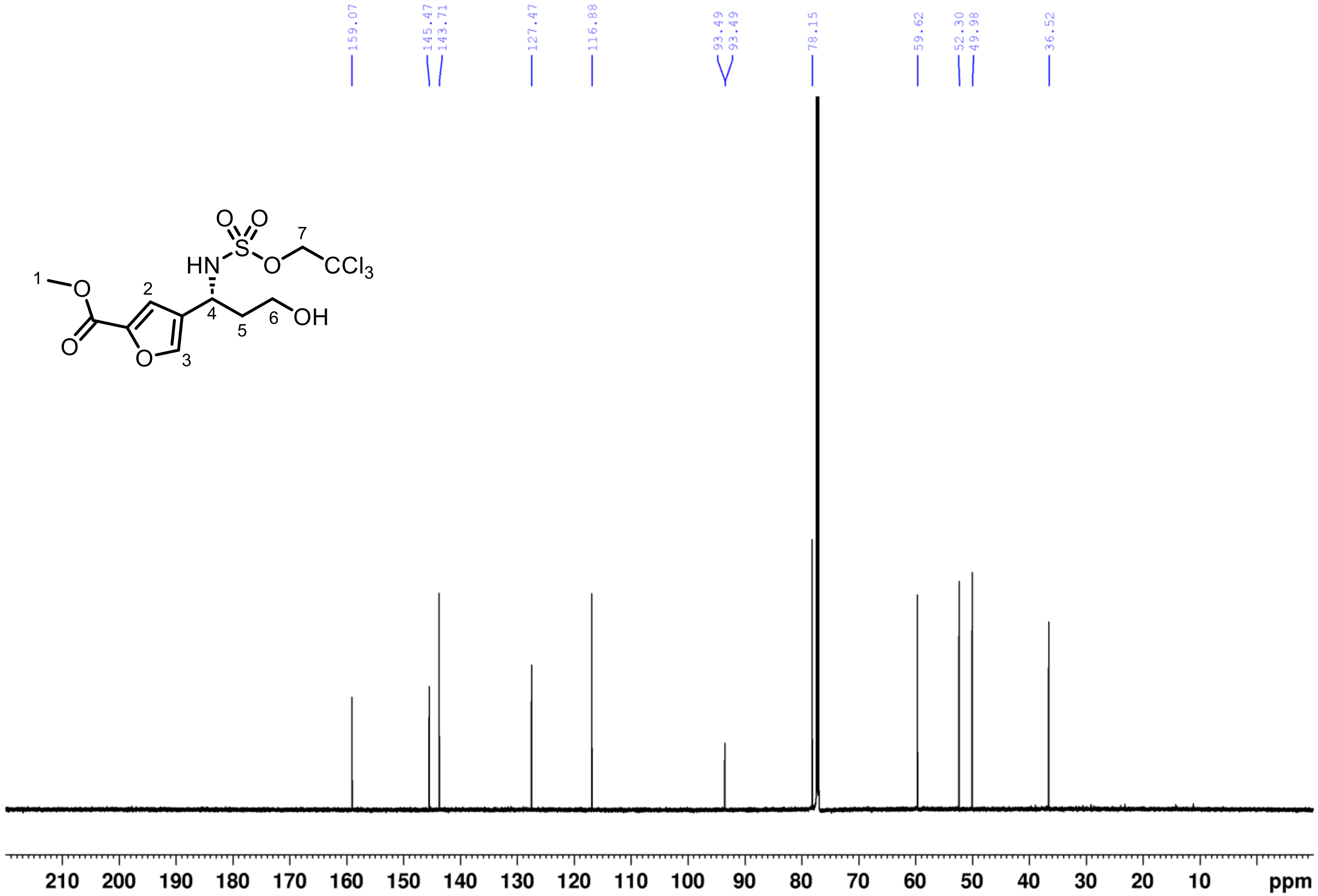

^{1}H NMR (400 MHz, CDCl$_3$) for *methyl 3a-(((2,2,2-trichloroethoxy)sulfonyl)amino)-3a,5,6,7a-tetrahydro-4H-furo[2,3-b]pyran-2-carboxylate* (**4c**)

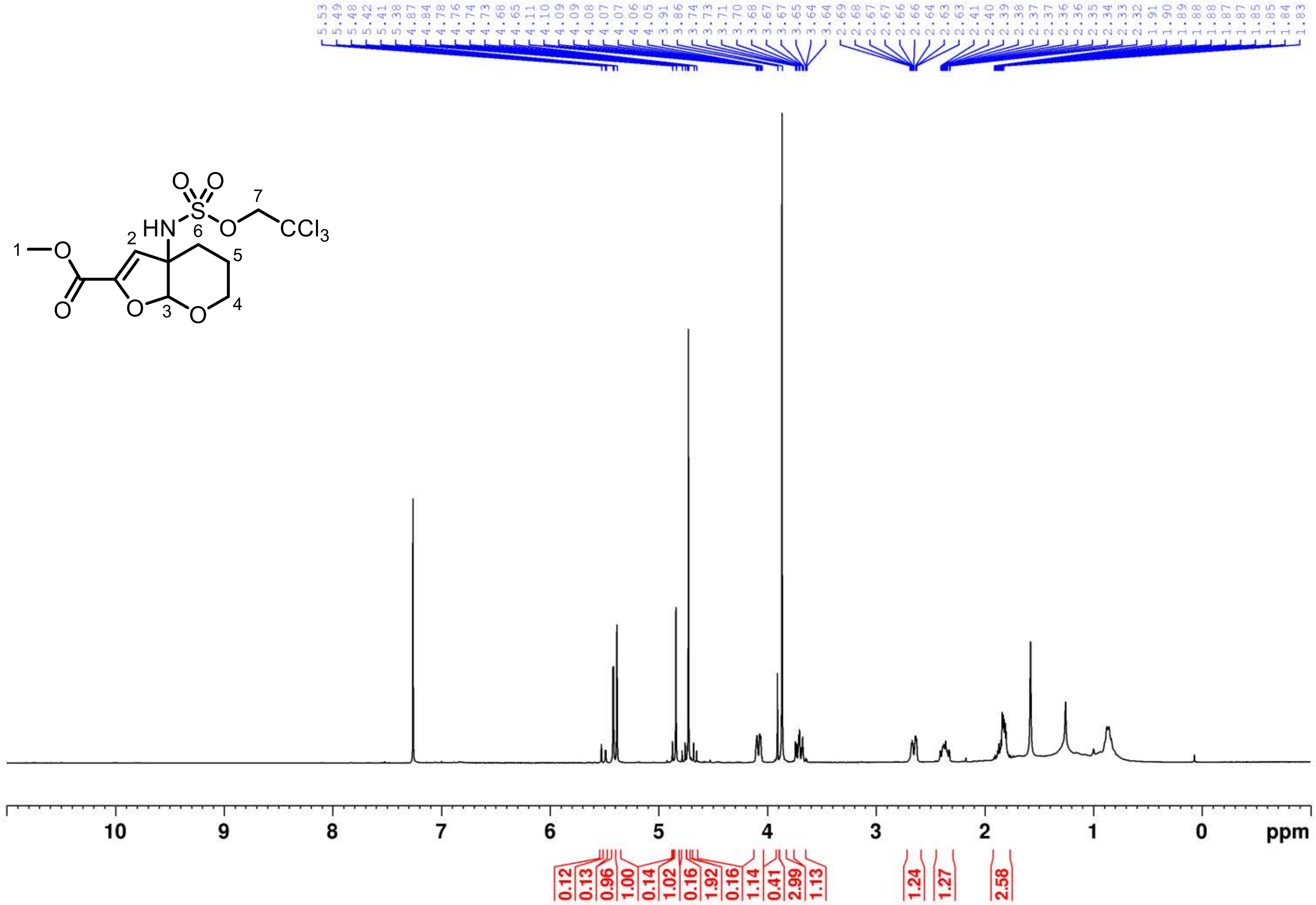

^{13}C NMR (101 MHz, CDCl$_3$) for *methyl 3a-(((2,2,2-trichloroethoxy)sulfonyl)amino)-3a,5,6,7a-tetrahydro-4H-furo[2,3-b]pyran-2-carboxylate* (**4c**)

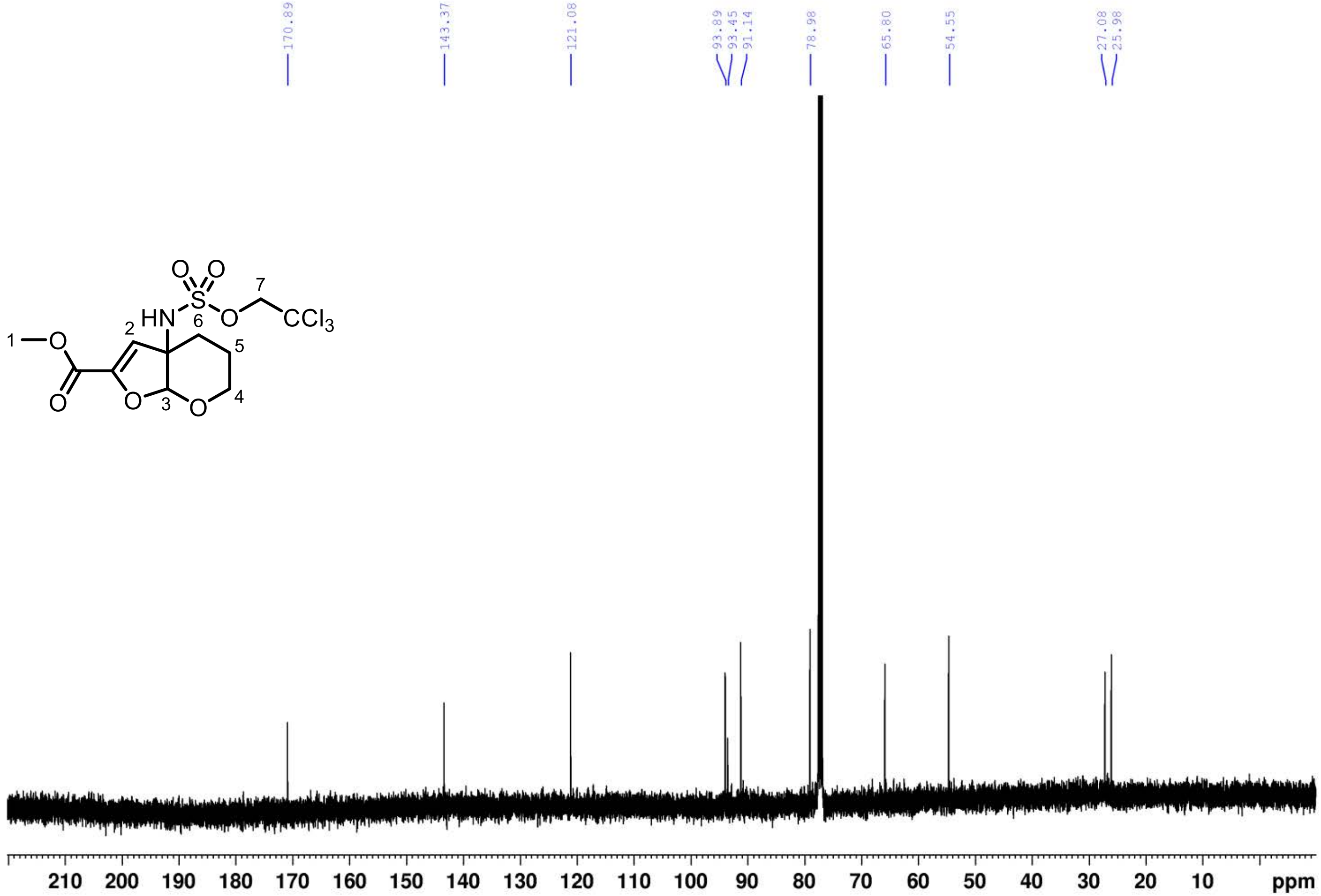

^{1}H NMR (700 MHz, $(CD_3)_2CO$) for *2,2,2-trichloroethyl ((1R,3R)-3-hydroxy-1,5-diphenylpentyl)sulfamate* (**2z**)

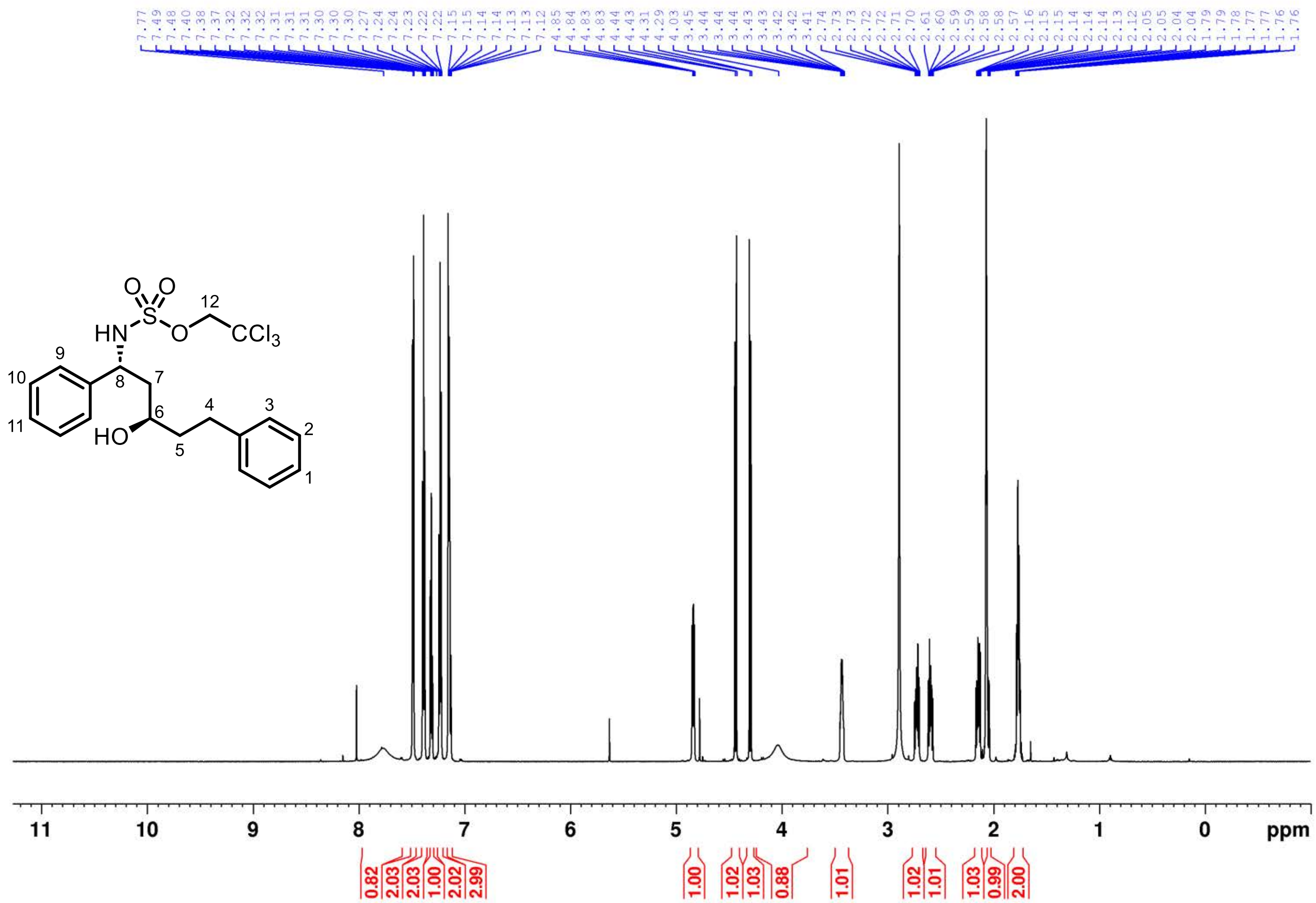

^{13}C NMR (176 MHz, $(CD_3)_2CO$) for *2,2,2-trichloroethyl ((1R,3R)-3-hydroxy-1,5-diphenylpentyl)sulfamate* (**2z**)

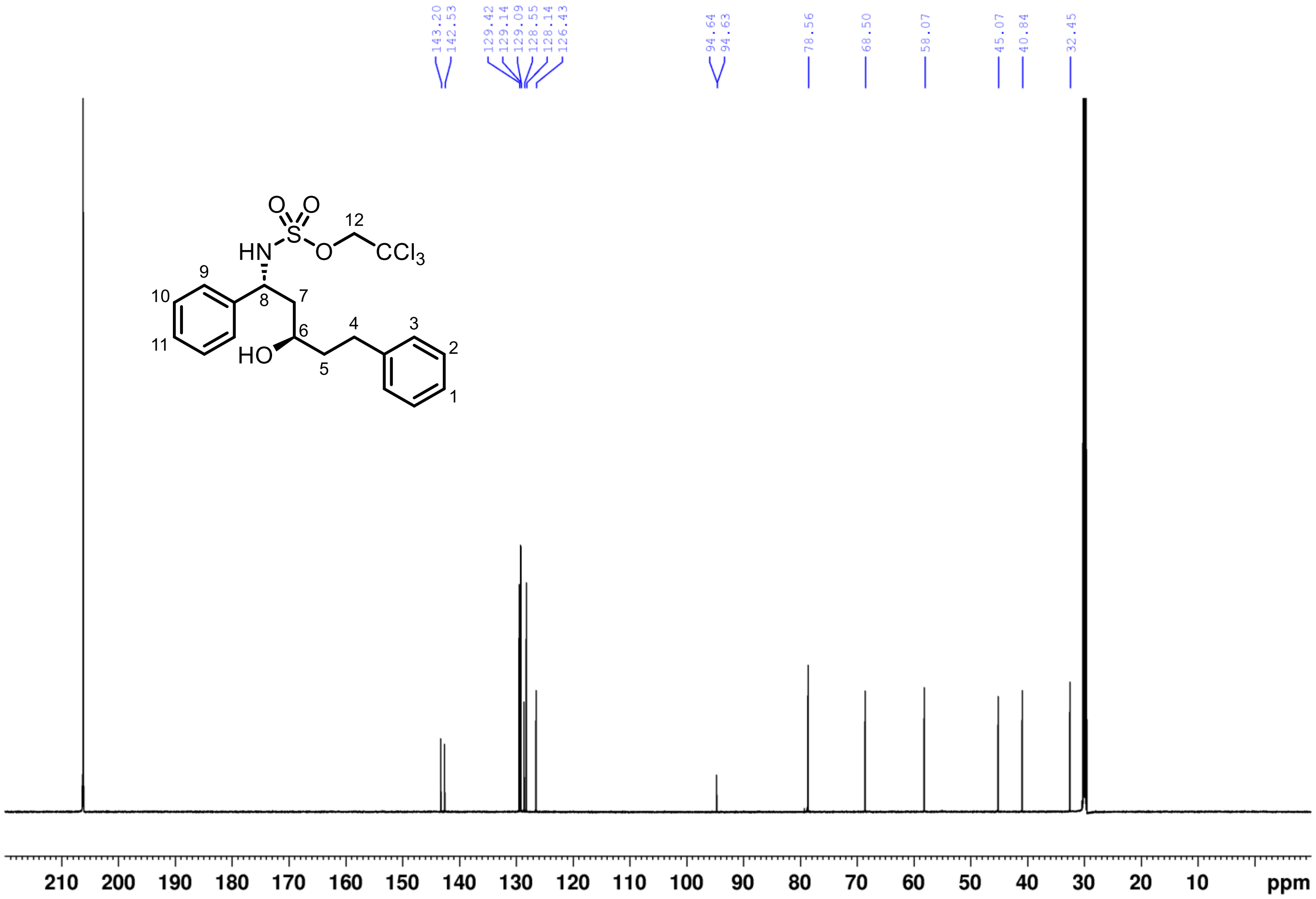

^{1}H NMR (500 MHz, CD_3OD) for *2,2,2-trichloroethyl ((1R,3R)-3-hydroxy-1-phenylbutyl)sulfamate* (**2zaa**)

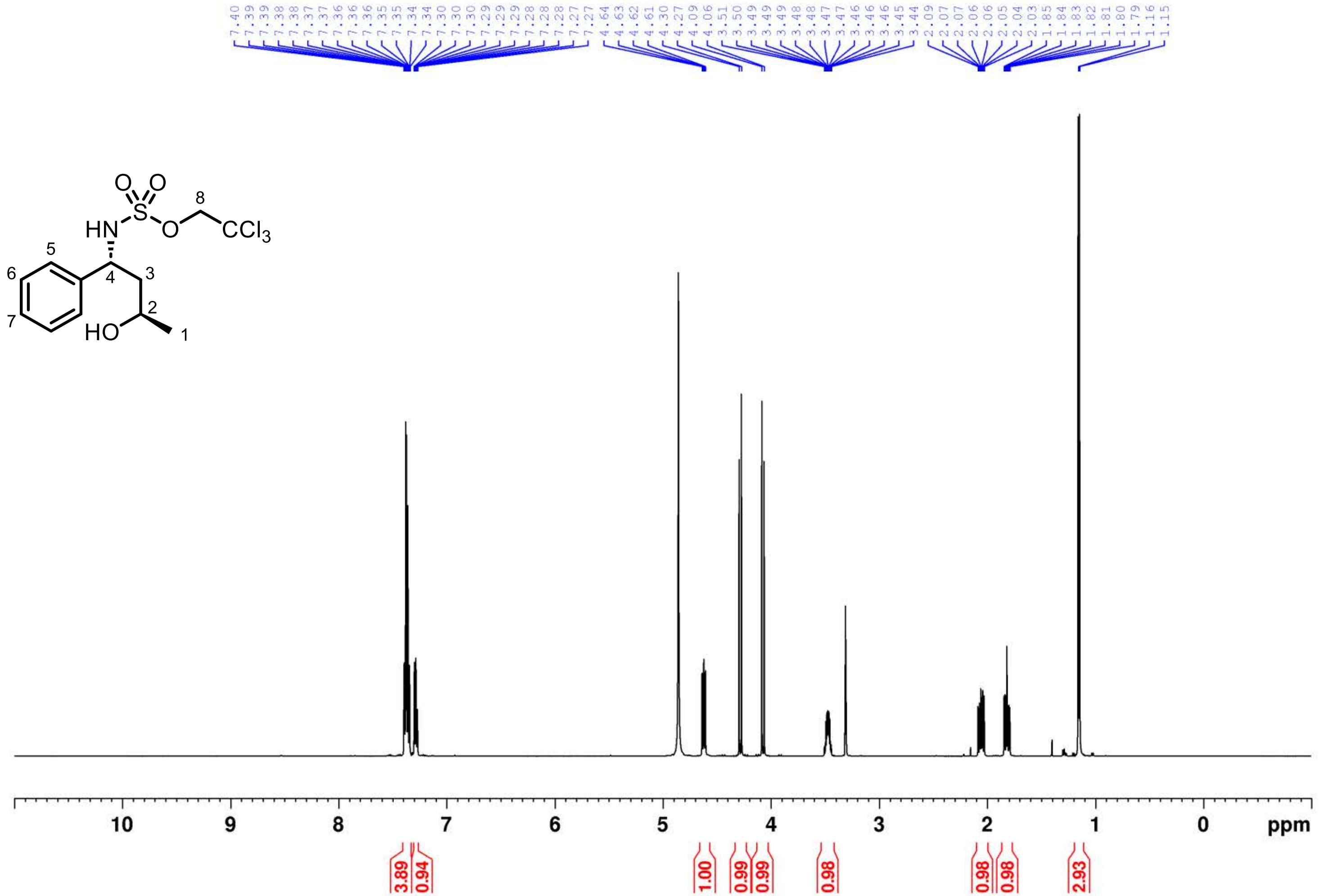

^{13}C NMR (126 MHz, CD_3OD) for *2,2,2-trichloroethyl ((1R,3R)-3-hydroxy-1-phenylbutyl)sulfamate* (**2zaa**)

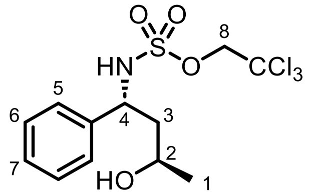

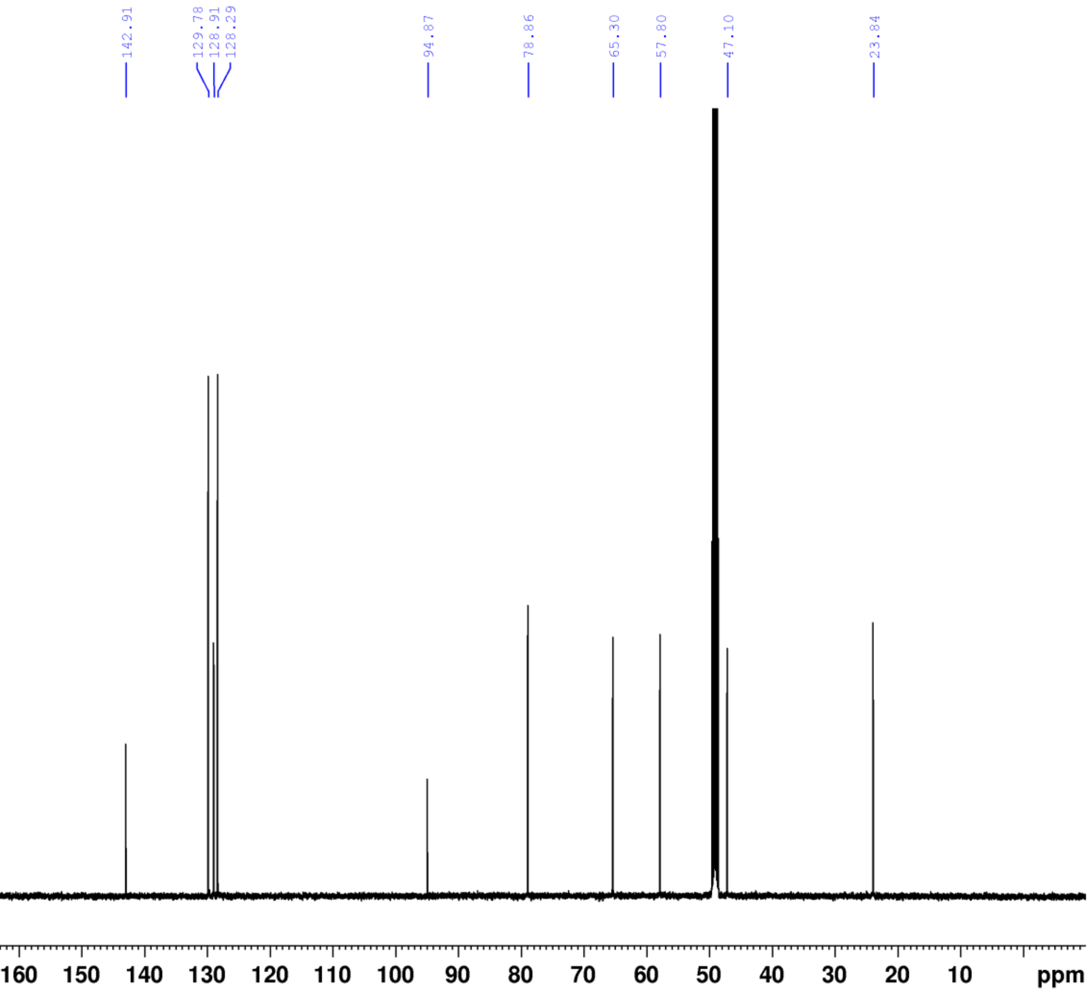

^{1}H NMR (700 MHz, CD_3OD) for *2,2,2-trichloroethyl ((1S,3R)-3-hydroxy-1-phenylbutyl)sulfamate* (**2zab**)

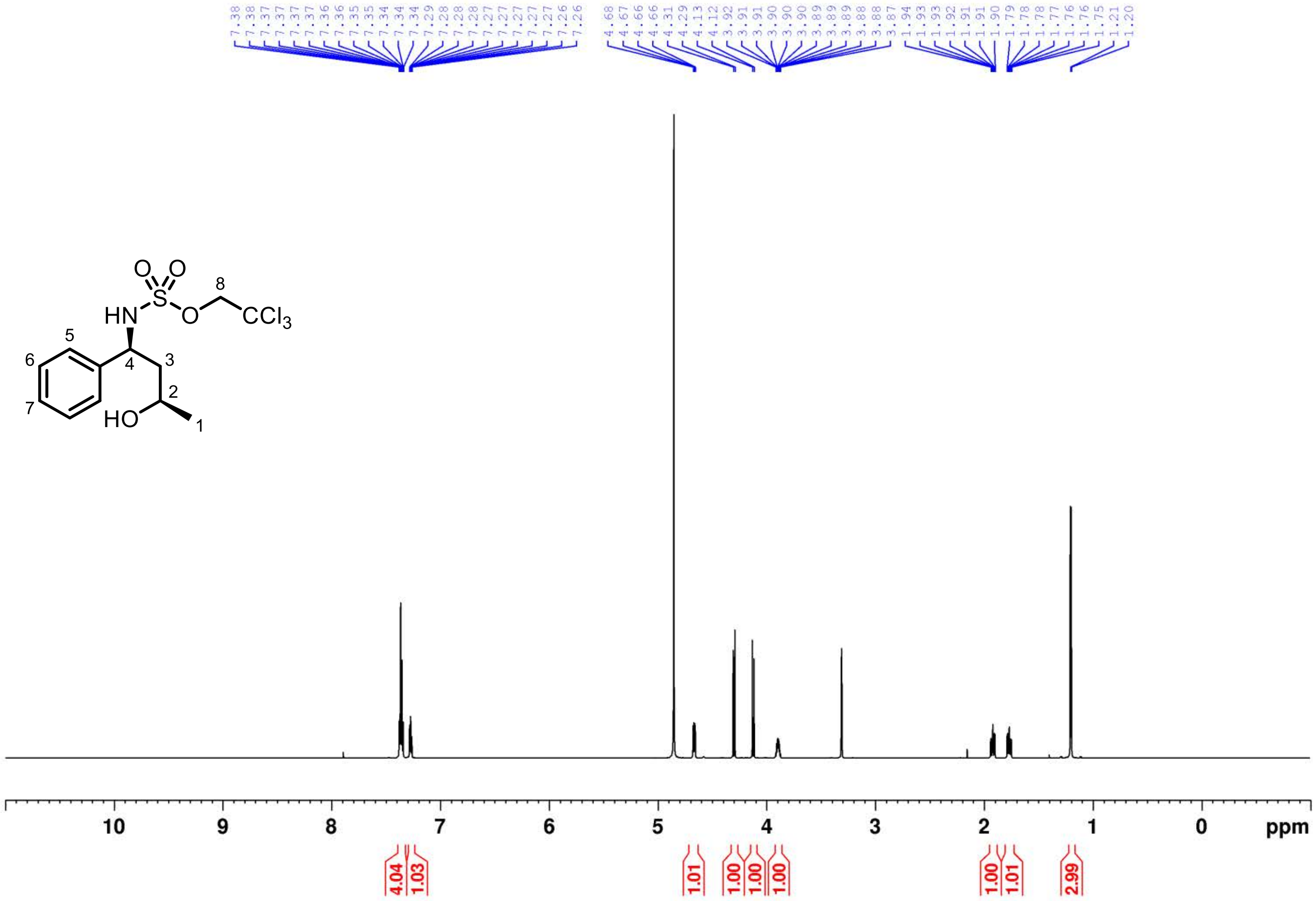

^{13}C NMR (176 MHz, CD_3OD) for *2,2,2-trichloroethyl ((1S,3R)-3-hydroxy-1-phenylbutyl)sulfamate* (**2zab**)

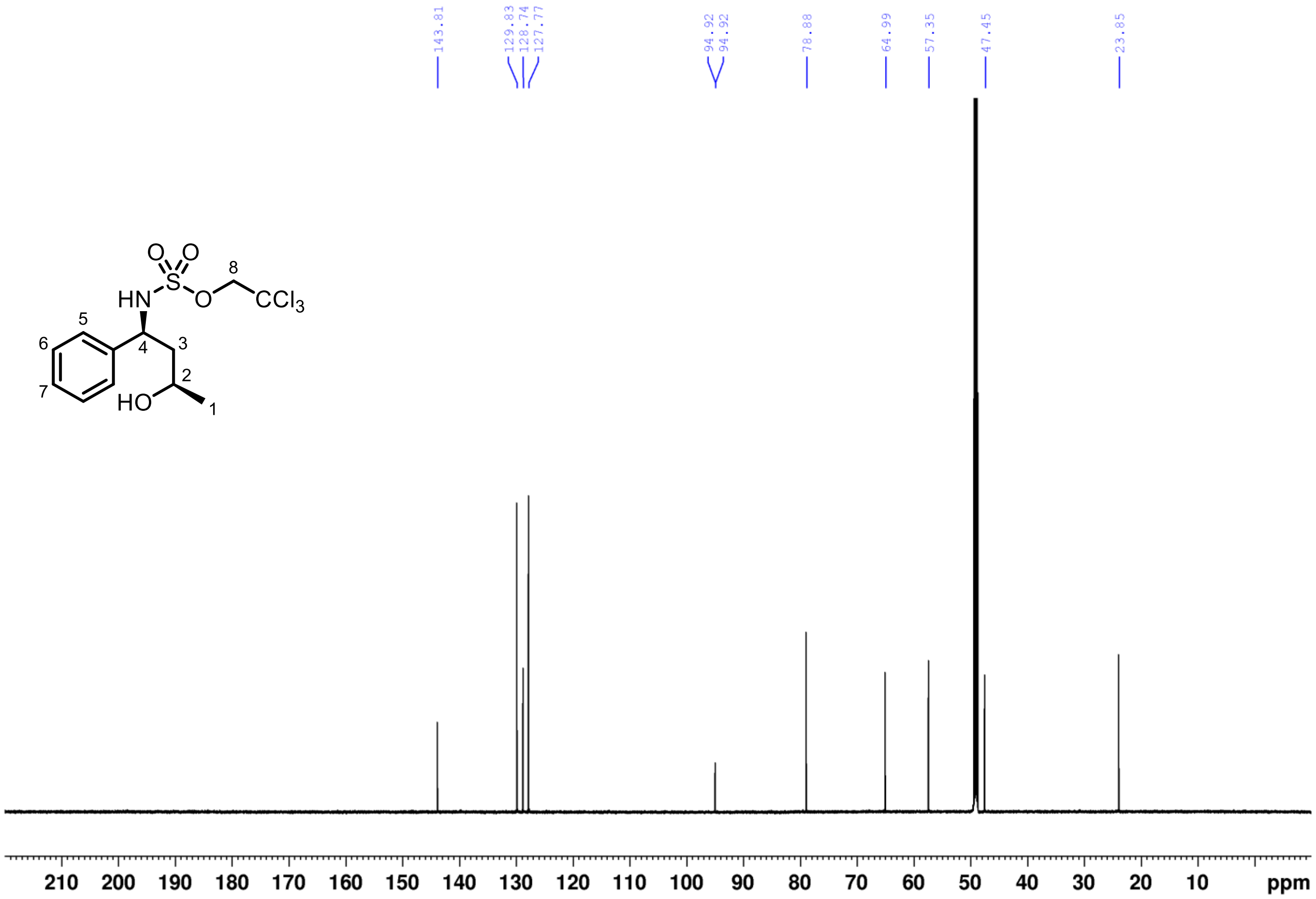

^{1}H NMR (700 MHz, $CDCl_3$) for a mixture of *ethyl (2R,4R)-2-hydroxy-4-phenyl-4-(((2,2,2-trichloroethoxy)sulfonyl)amino)butanoate* (**2zba**) NH868 and *ethyl (2R,4S)-2-hydroxy-4-phenyl-4-(((2,2,2-trichloroethoxy)sulfonyl)amino)butanoate* (**2zbb**)

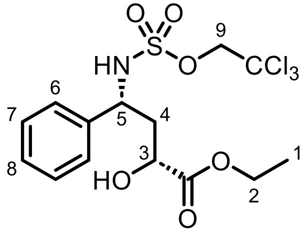

2zba

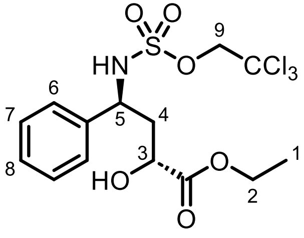

2zbb

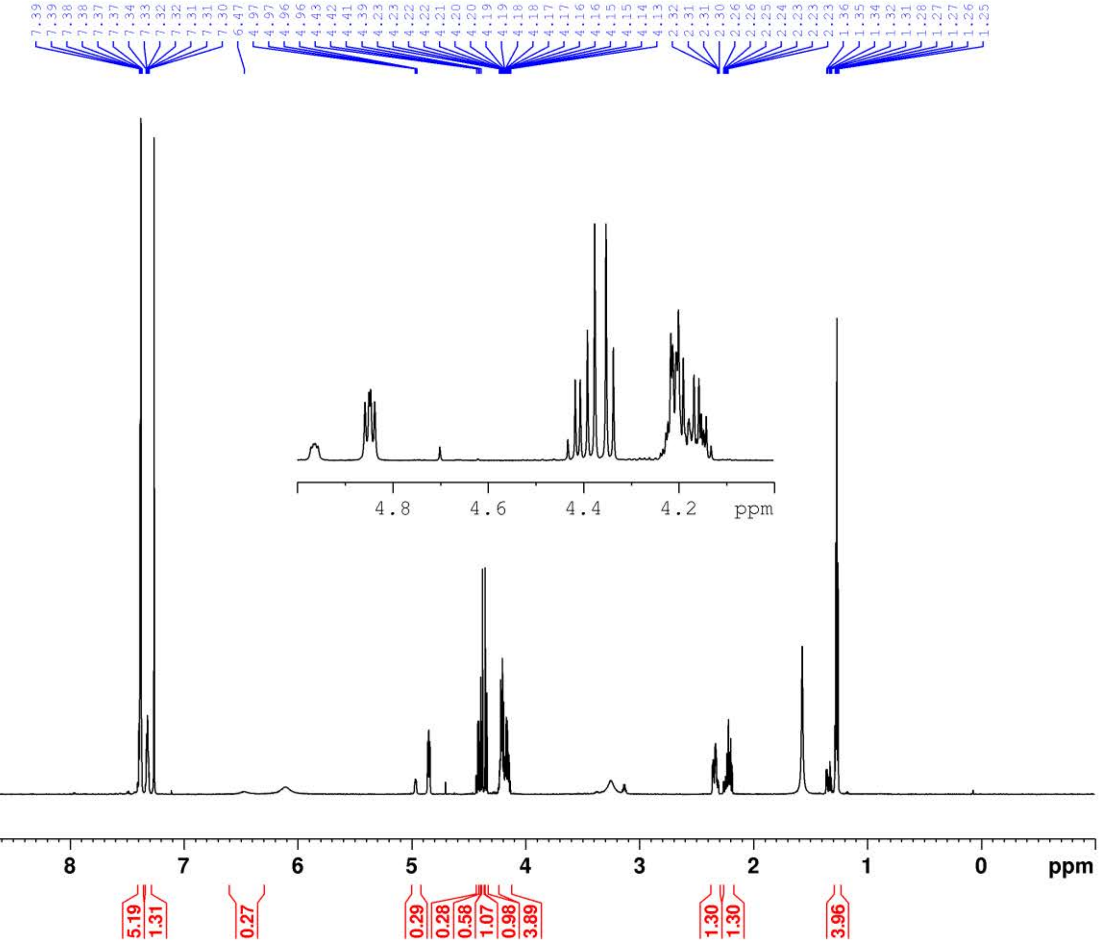

^{13}C NMR (176 MHz, CDCl$_3$) for a mixture of *ethyl (2R,4R)-2-hydroxy-4-phenyl-4-(((2,2,2-trichloroethoxy)sulfonyl)amino)butanoate* (**2zba**) and *ethyl (2R,4S)-2-hydroxy-4-phenyl-4-(((2,2,2-trichloroethoxy)sulfonyl)amino)butanoate* (**2zbb**)

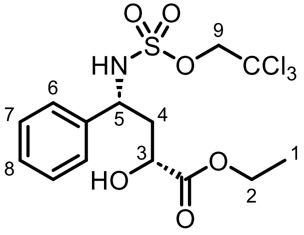

2zba

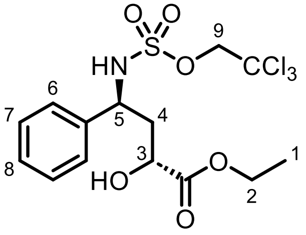

2zbb

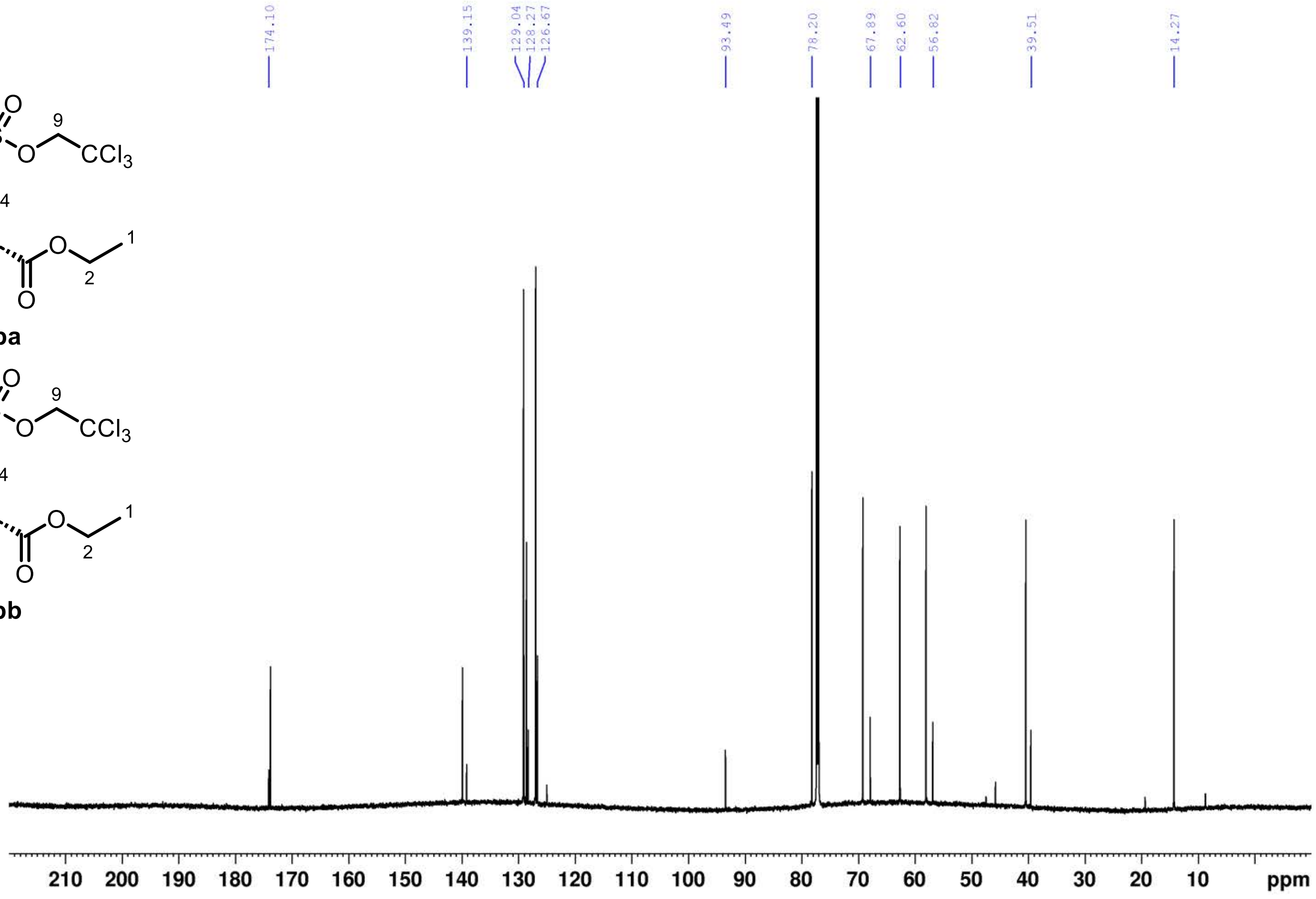

^{1}H NMR (700 MHz, $CDCl_3$) for *ethyl (2R,4S)-2-hydroxy-4-phenyl-4-(((2,2,2-trichloroethoxy)sulfonyl)amino)butanoate* (**2zbb**)

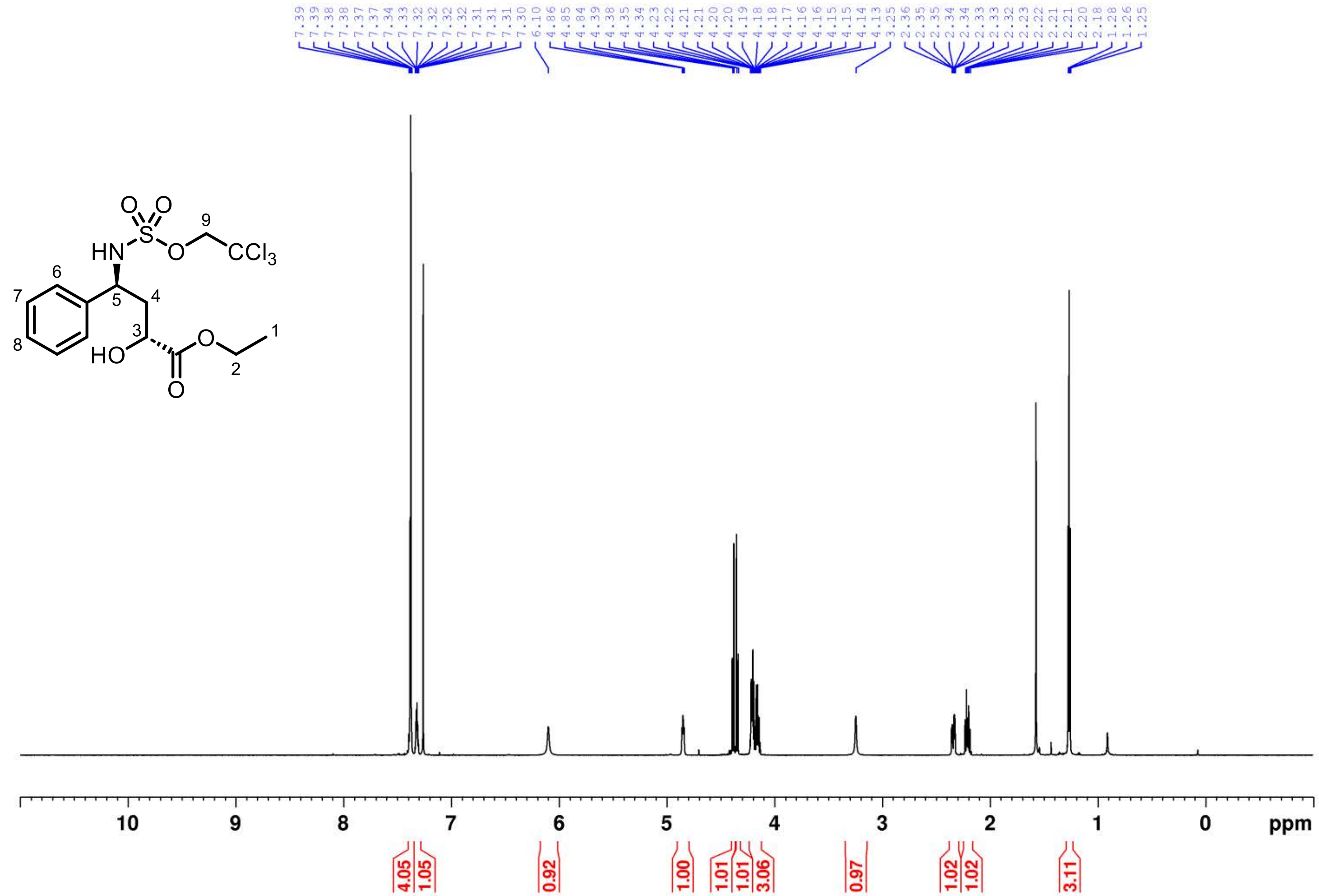

^{13}C NMR (176 MHz, $CDCl_3$) for *ethyl (2R,4S)-2-hydroxy-4-phenyl-4-(((2,2,2-trichloroethoxy)sulfonyl)amino)butanoate* (**2zbb**)

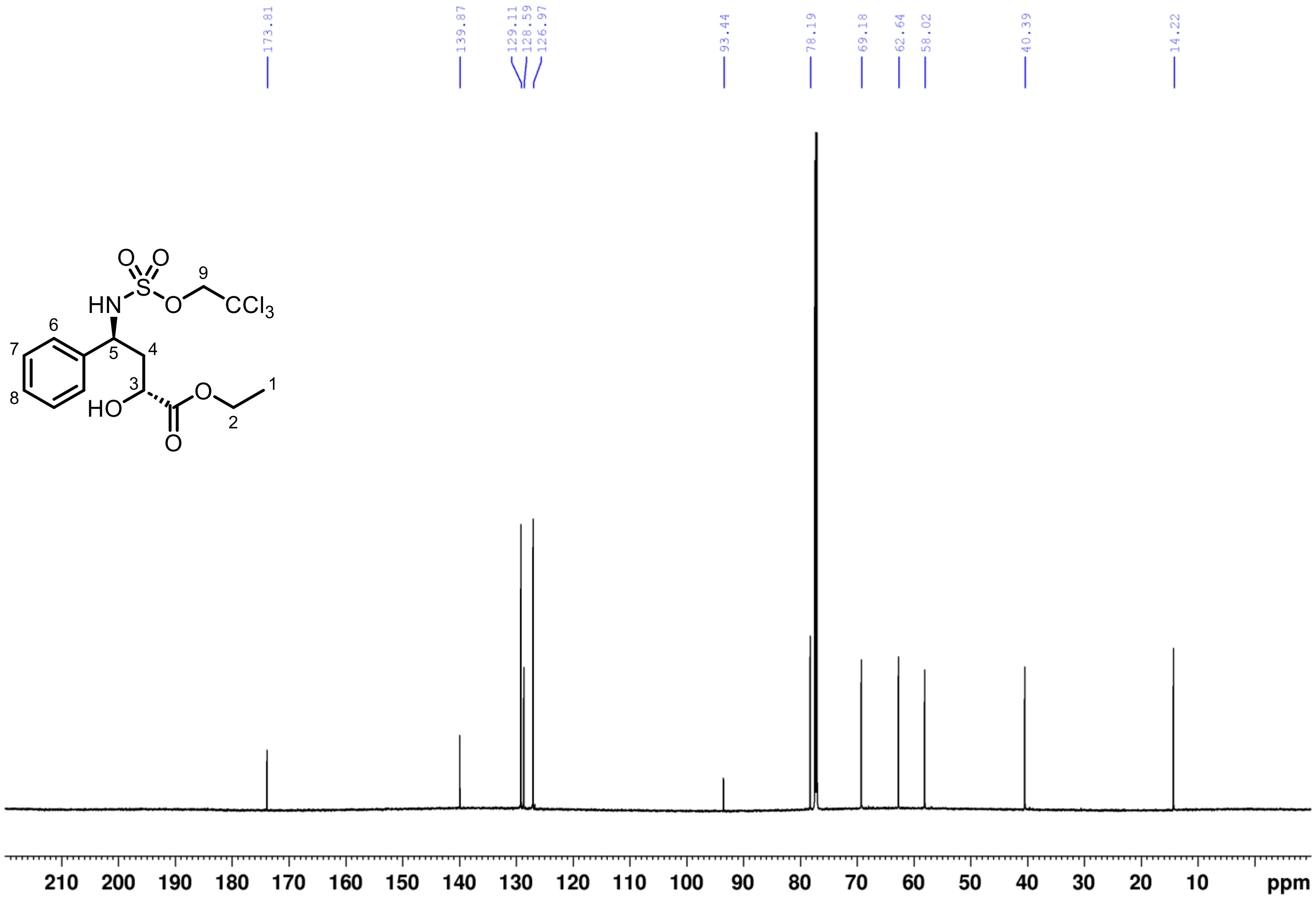

^{1}H NMR (700 MHz, CDCl$_3$) for *2,2,2-trichloroethyl (2S,4R)-2-methyl-4-phenylazetidine-1-sulfonate* (**5a**)

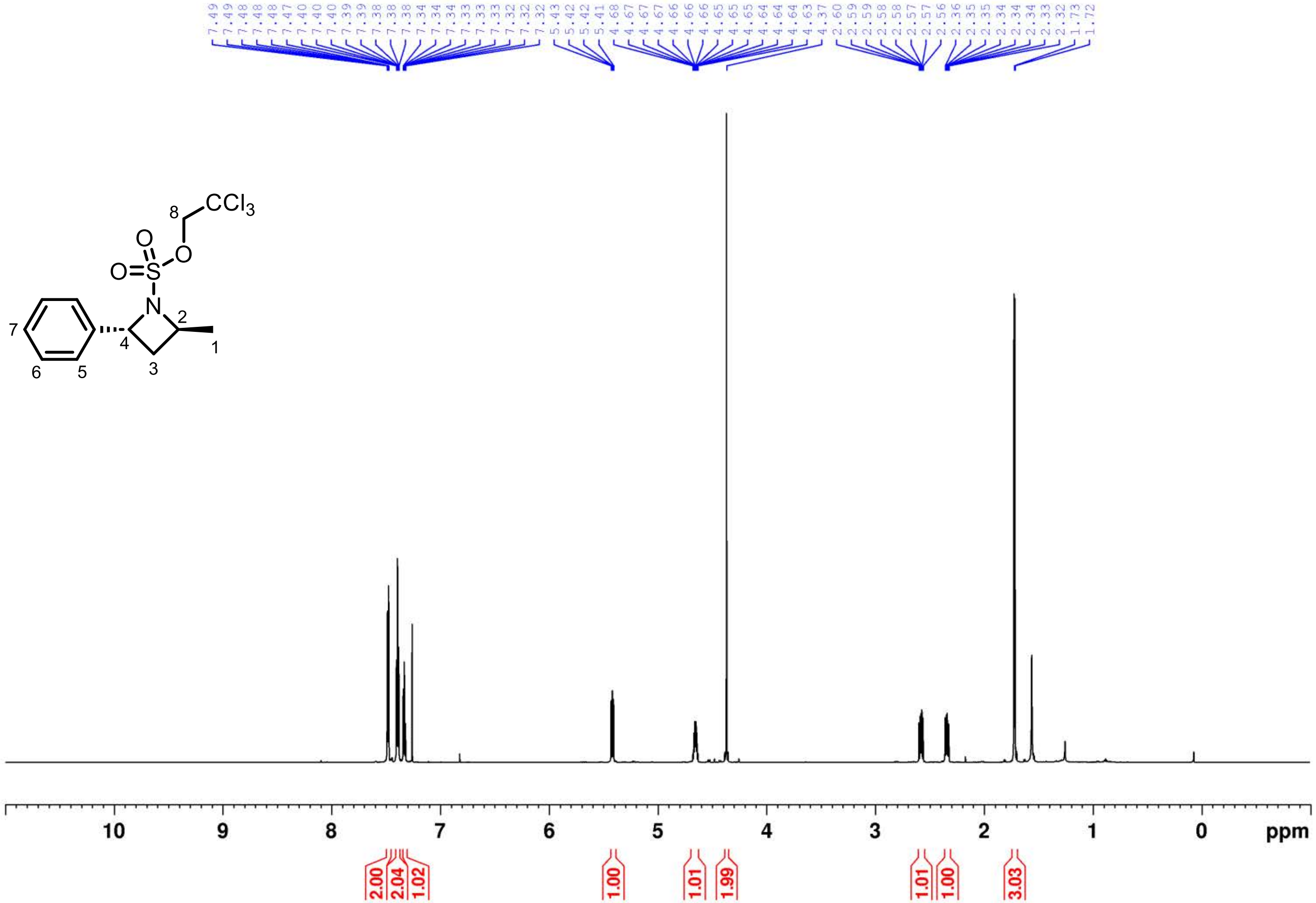

^{13}C NMR (176 MHz, CDCl$_3$) for *2,2,2-trichloroethyl (2S,4R)-2-methyl-4-phenylazetidine-1-sulfonate* (**5a**)

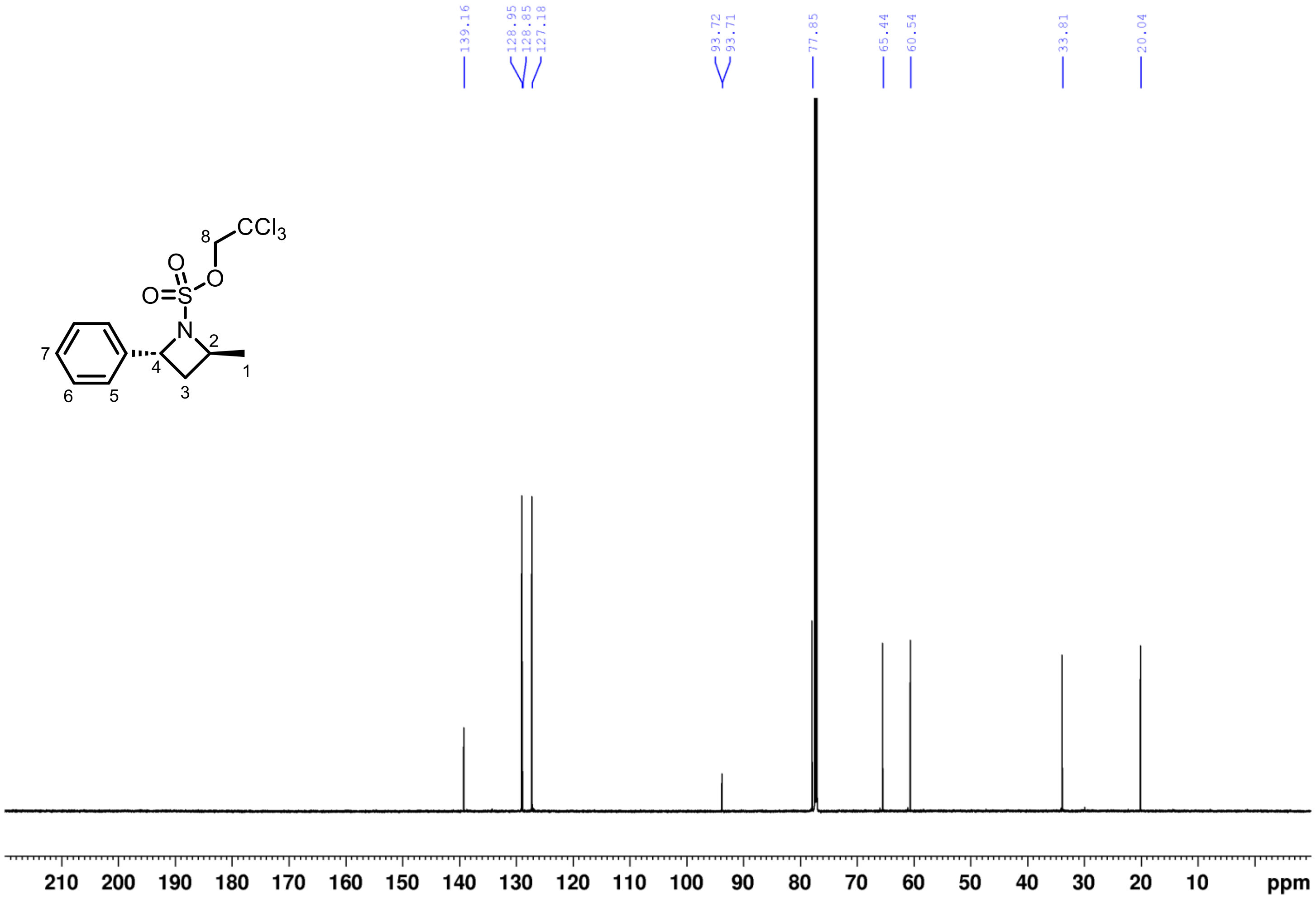

¹H NMR (700 MHz, CDCl₃) for *ethyl (2S,4S)-4-phenyl-1-((2,2,2-trichloroethoxy)sulfonyl)azetidine-2-carboxylate* (**5b**)

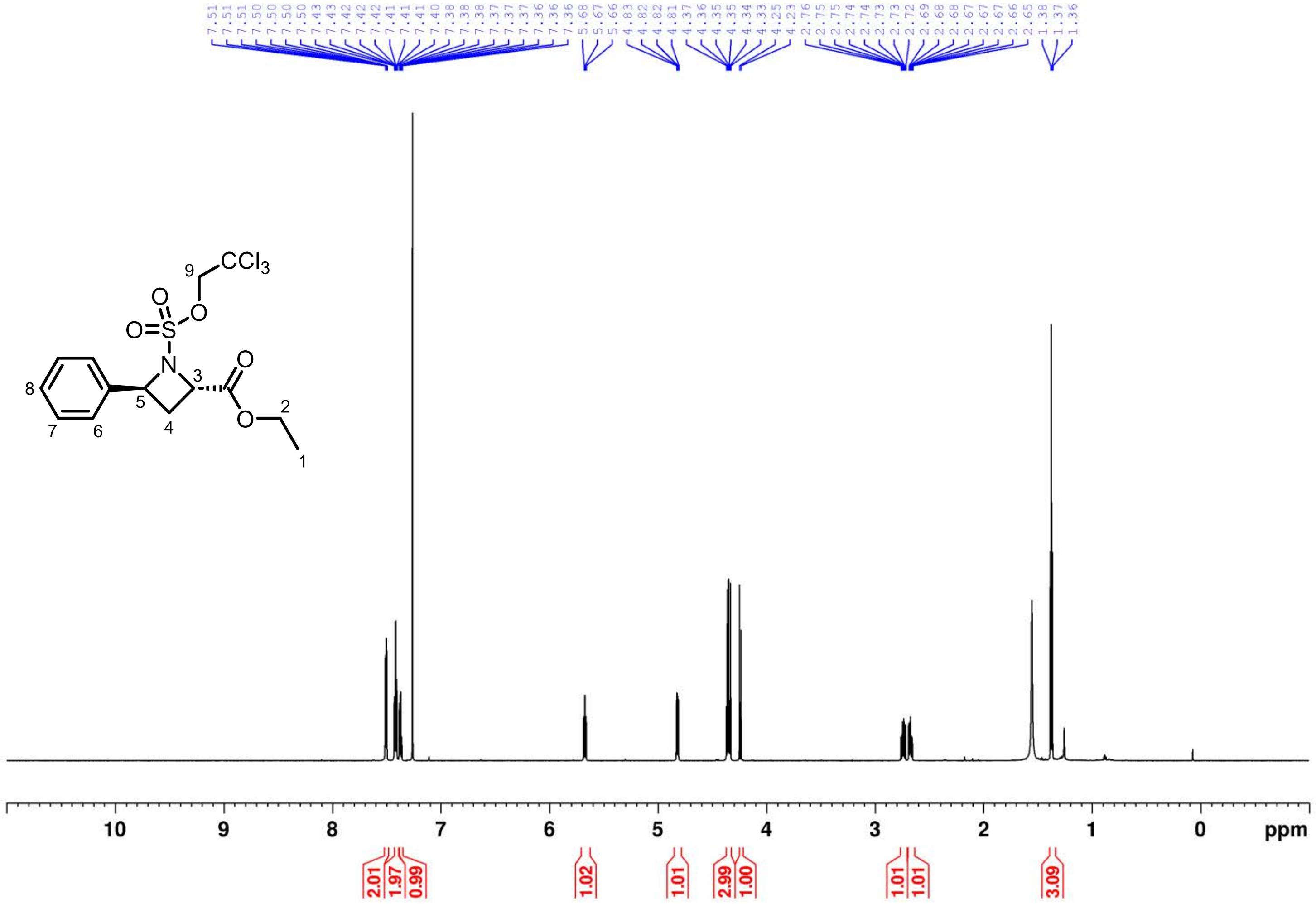

^{13}C NMR (176 MHz, CDCl$_3$) for *ethyl (2S,4S)-4-phenyl-1-((2,2,2-trichloroethoxy)sulfonyl)azetidine-2-carboxylate* (**5b**)

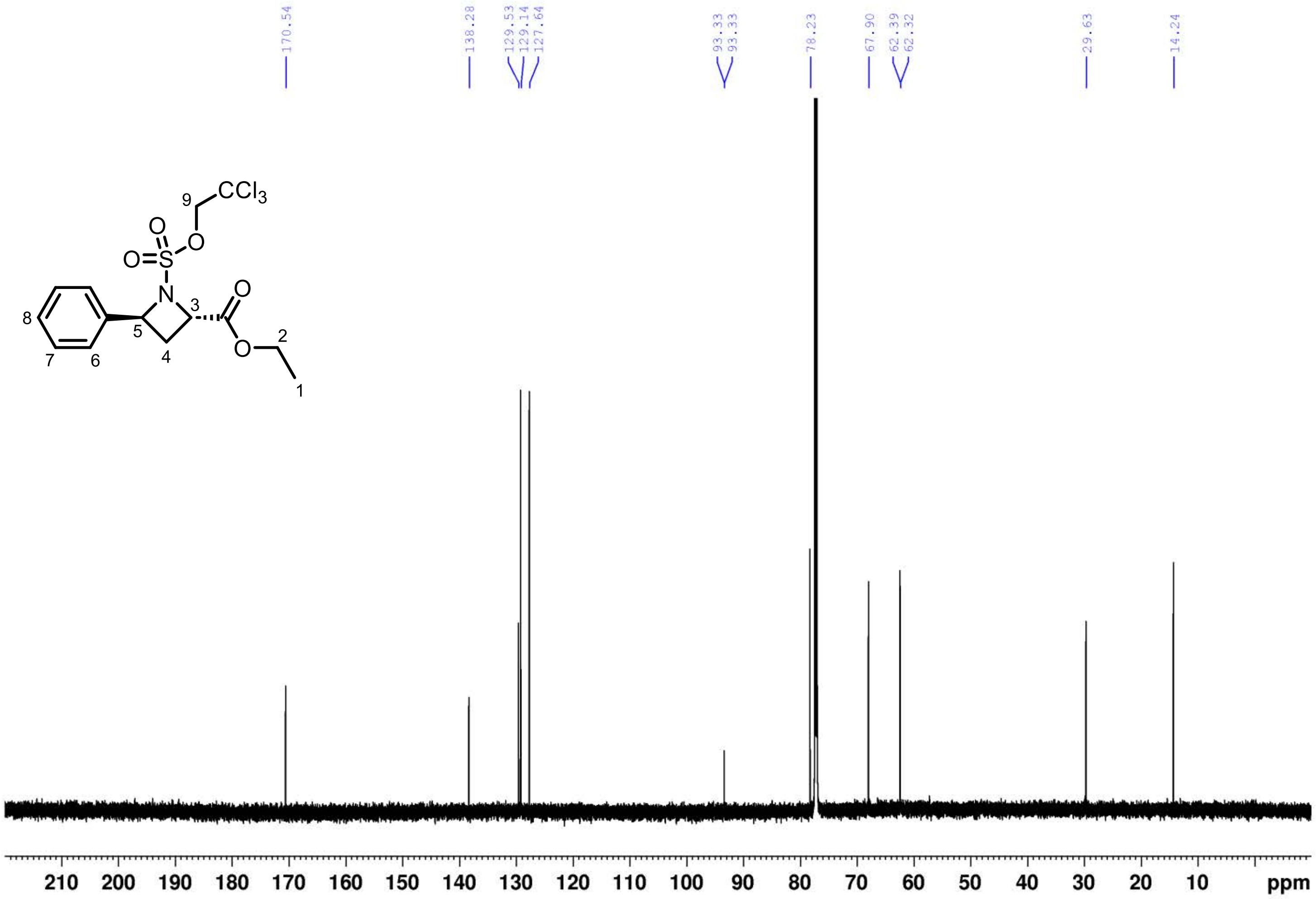

^{1}H NMR (500 MHz, $CDCl_3$) for *2,2,2-trichloroethyl (S)-1-phenyl-2-azaspiro[3.4]octane-2-sulfonate* (**5c**)

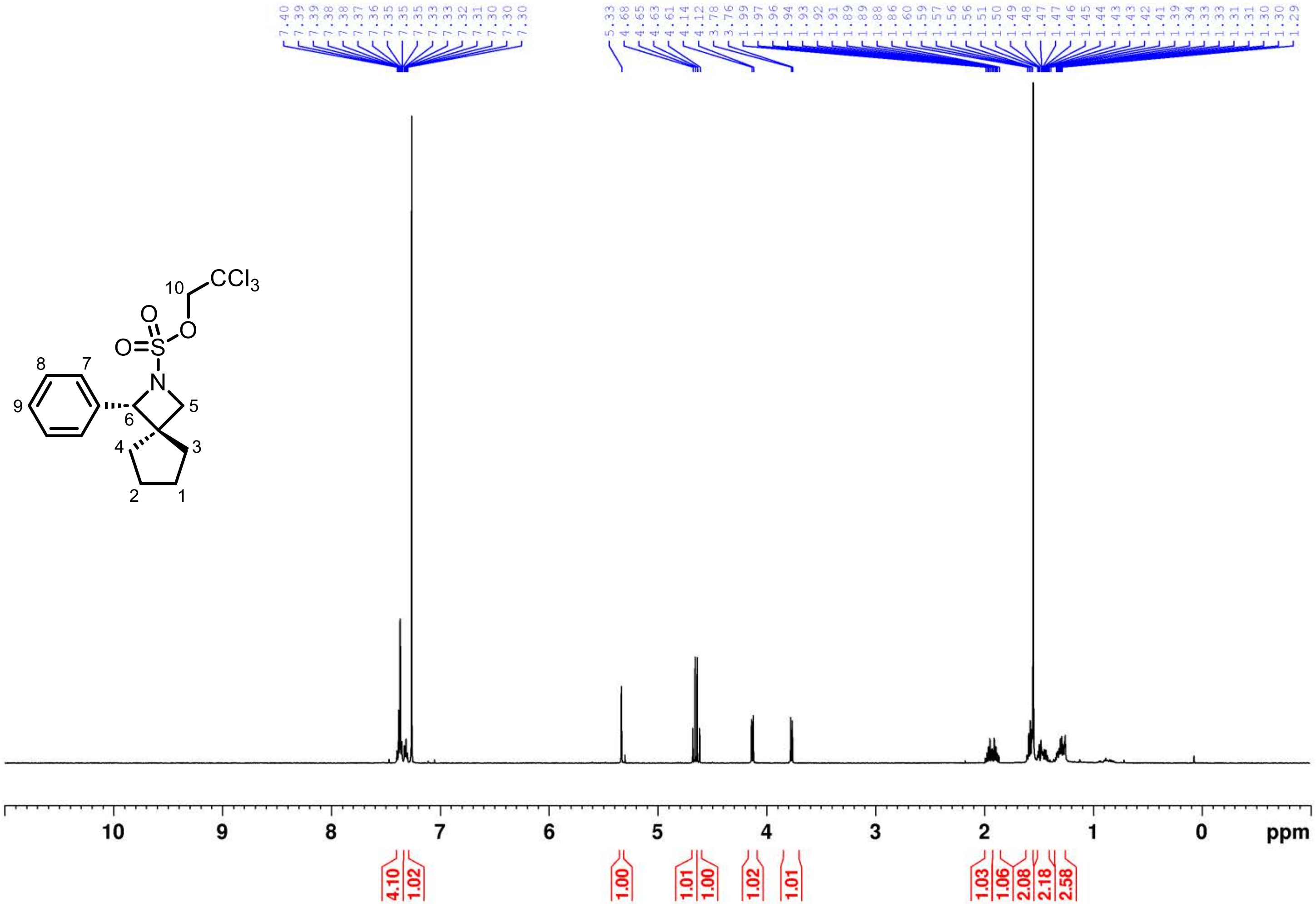

^{13}C NMR (126 MHz, $CDCl_3$) for *2,2,2-trichloroethyl (S)-1-phenyl-2-azaspiro[3.4]octane-2-sulfonate* (**5c**)

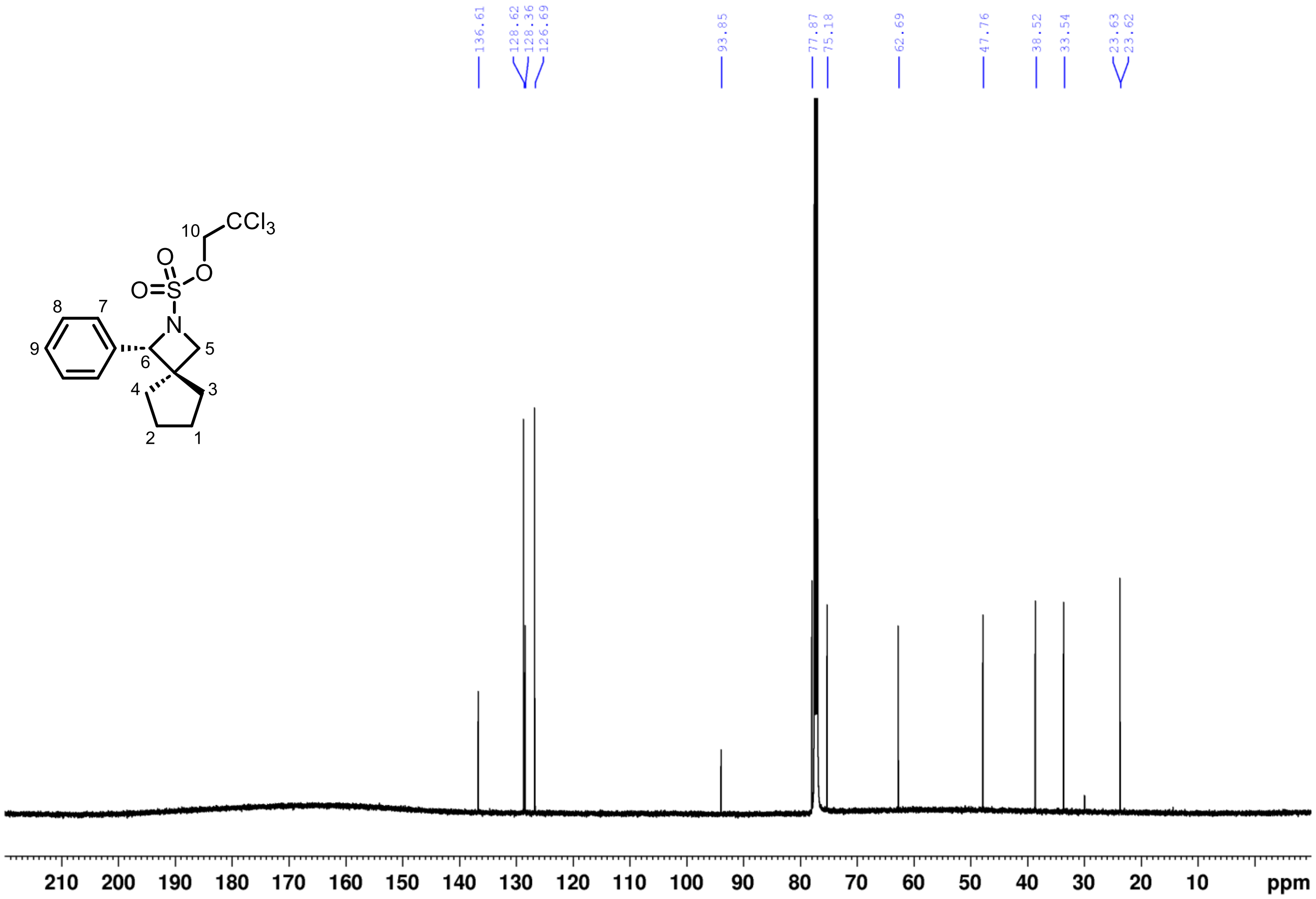

^{1}H NMR (700 MHz, CDCl$_3$) for *2,2,2-trichloroethyl (2S,4R)-2-phenethyl-4-phenylazetidine-1-sulfonate* (**5d**)

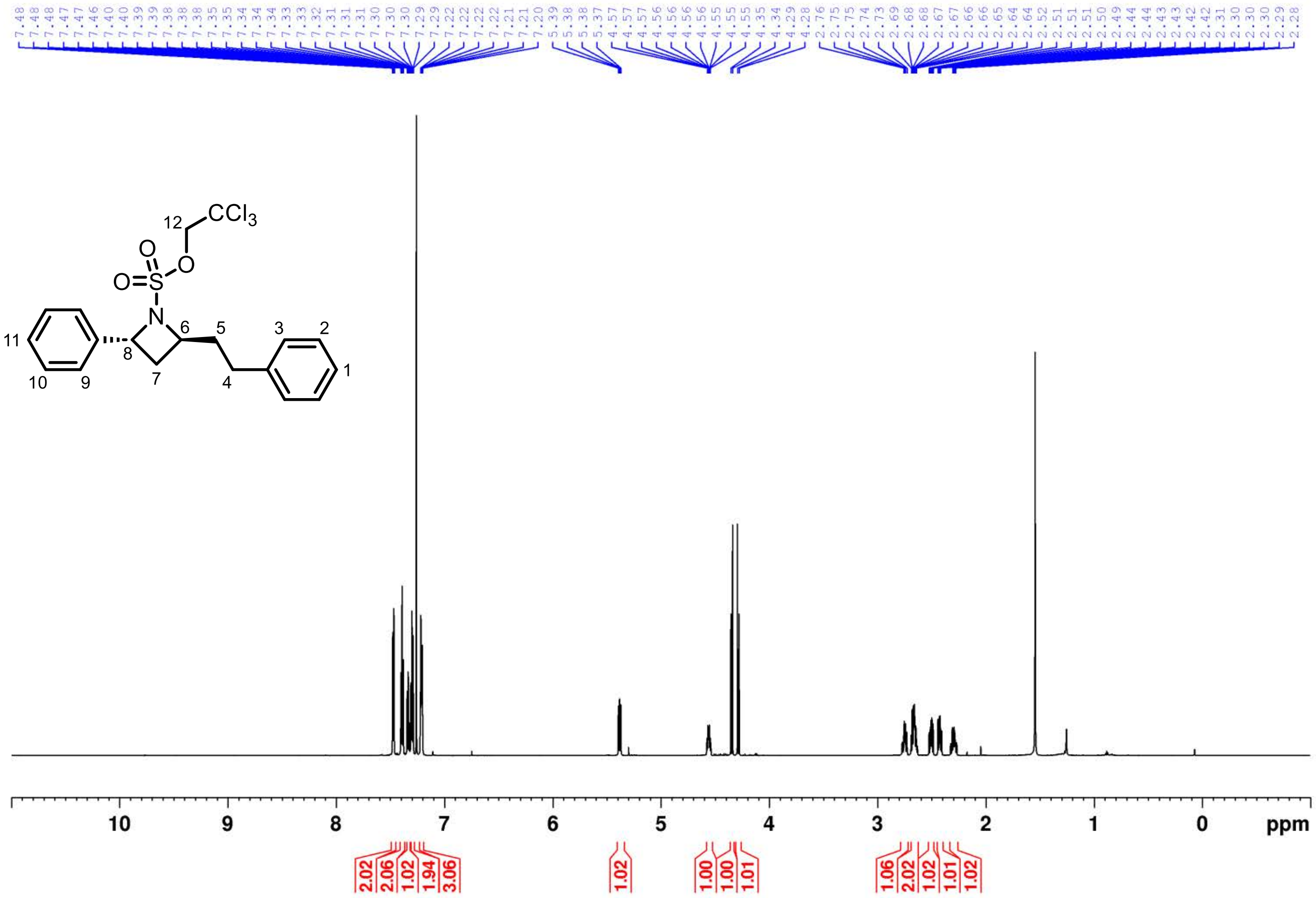

^{13}C NMR (176 MHz, CDCl$_3$) for *2,2,2-trichloroethyl (2S,4R)-2-phenethyl-4-phenylazetidine-1-sulfonate* (**5d**)

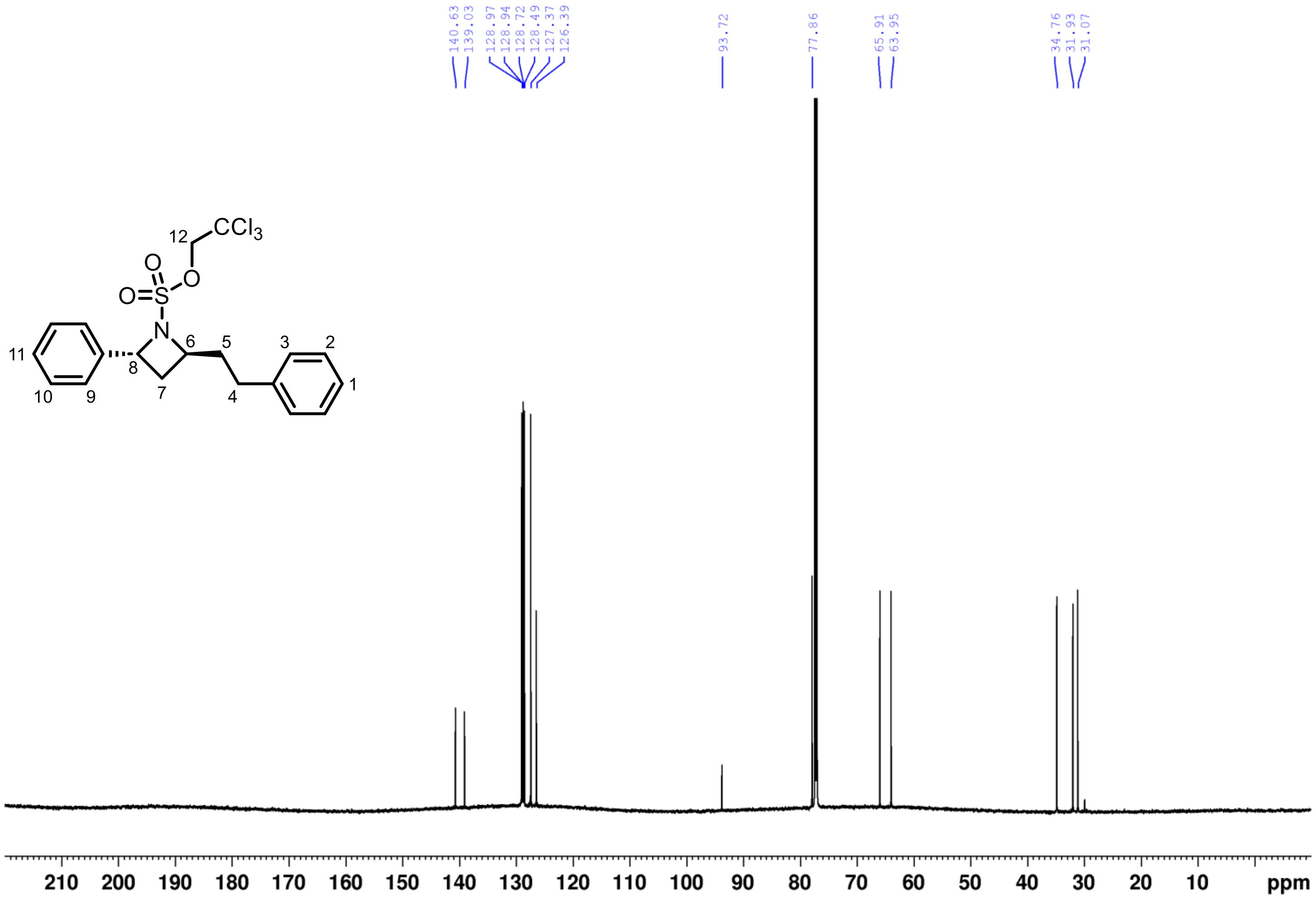

^{1}H NMR (700 MHz, CDCl$_3$) for *2,2,2-trichloroethyl (R)-2-phenyl-1-azaspiro[3.3]heptane-1-sulfonate* (**5e**)

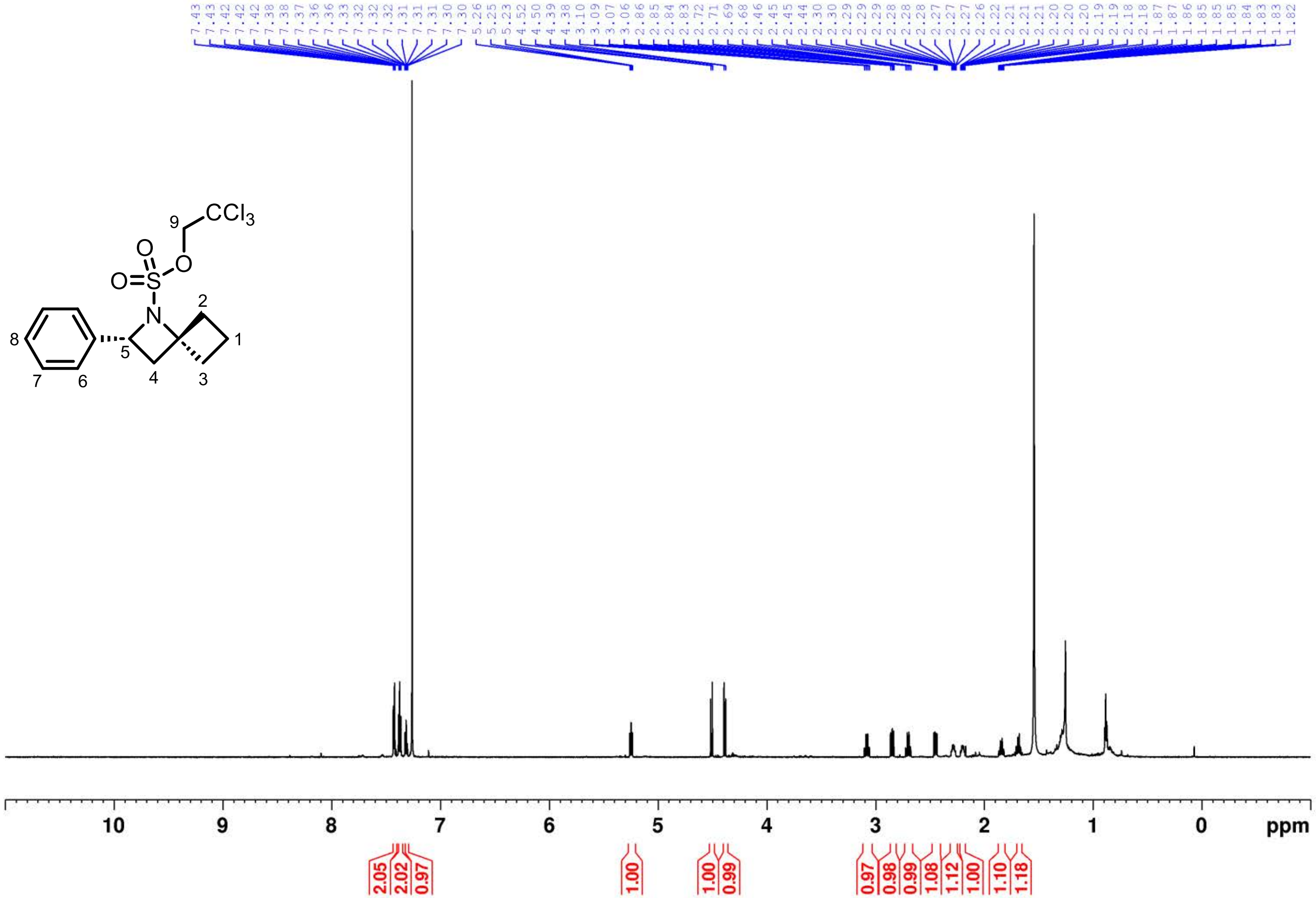

^{13}C NMR (176 MHz, $CDCl_3$) for *2,2,2-trichloroethyl (R)-2-phenyl-1-azaspiro[3.3]heptane-1-sulfonate* (**5e**)

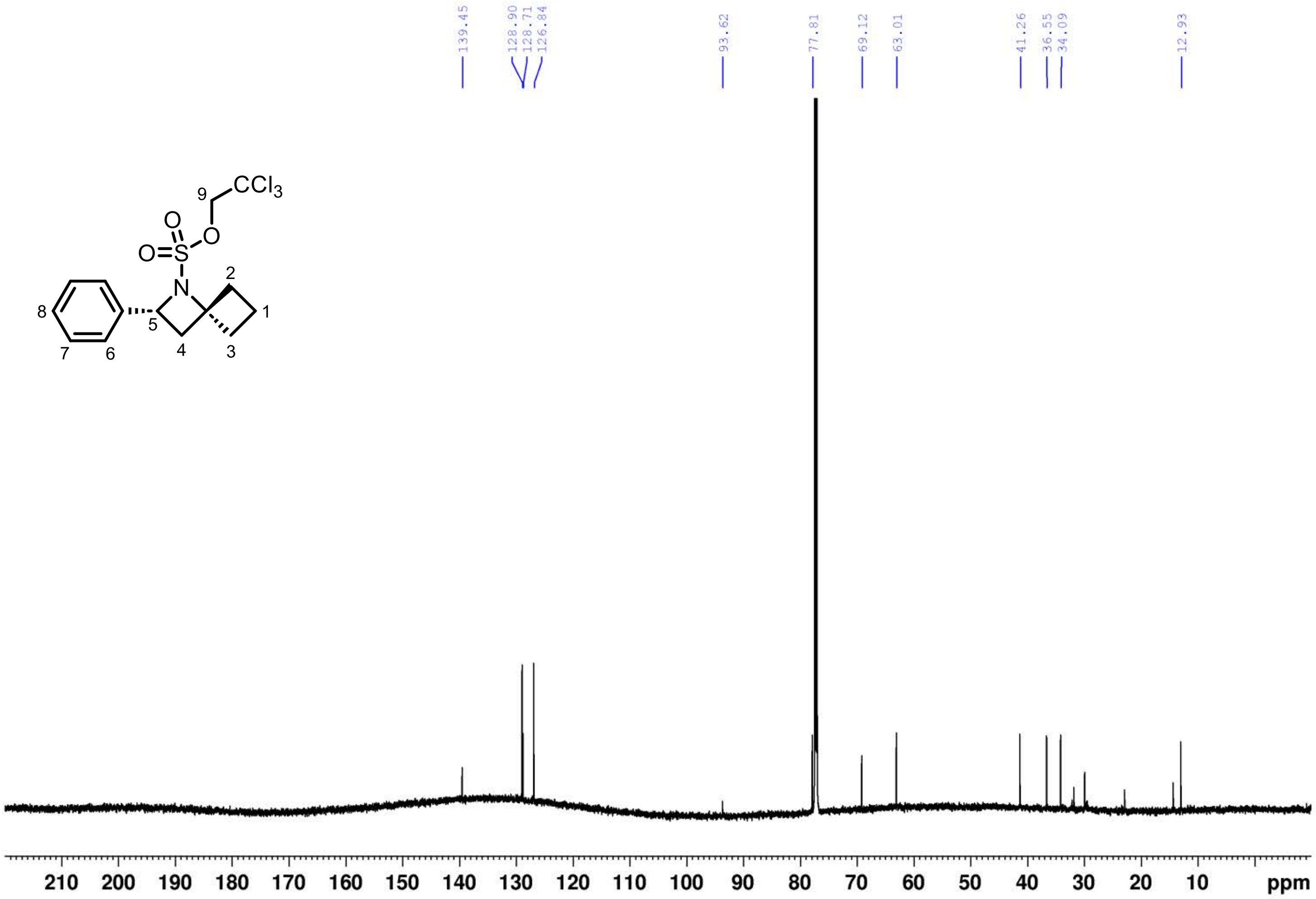

^{1}H NMR (400 MHz, CDCl$_3$) for *2,2,2-trichloroethyl (R)-(4-hydroxy-1-phenylbutyl)sulfamate* (**7a**)

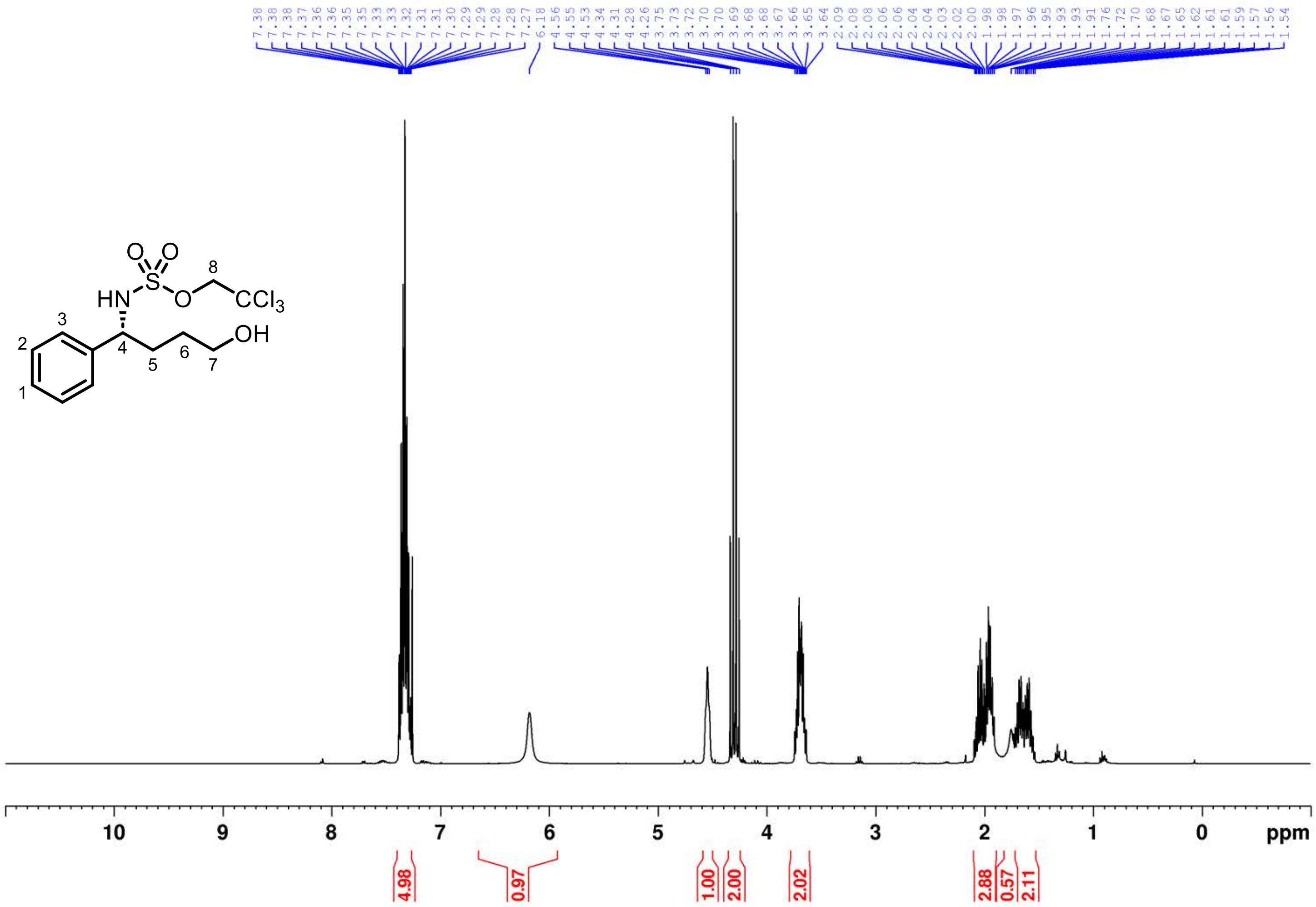

^{13}C NMR (101 MHz, $CDCl_3$) for *2,2,2-trichloroethyl (R)-(4-hydroxy-1-phenylbutyl)sulfamate* (**7a**)

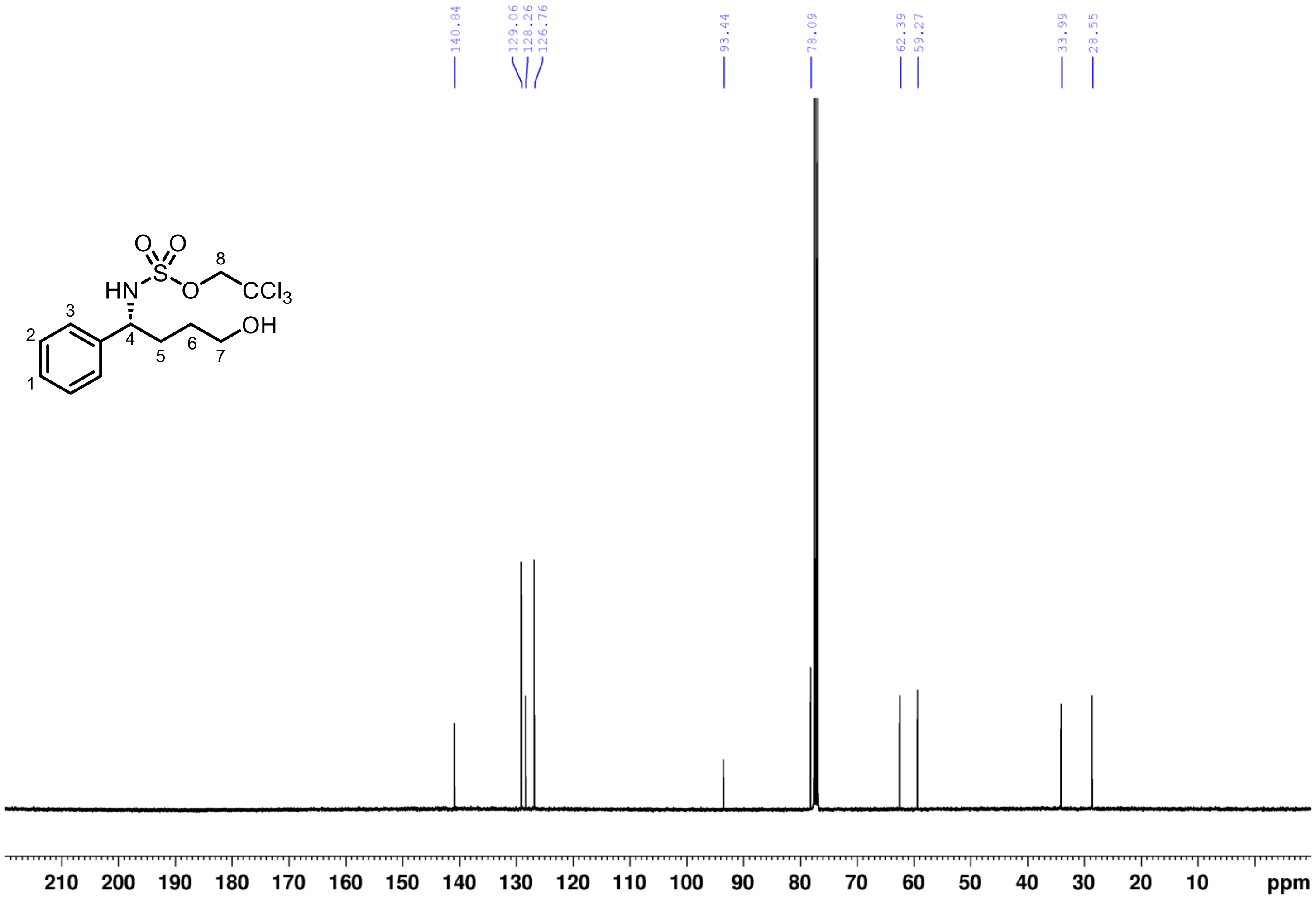

^{1}H NMR (700 MHz, $CDCl_3$) for *ethyl (R)-3-(4-hydroxy-1-(((2,2,2-trichloroethoxy)sulfonyl)amino)butyl)benzoate* (**7b**)

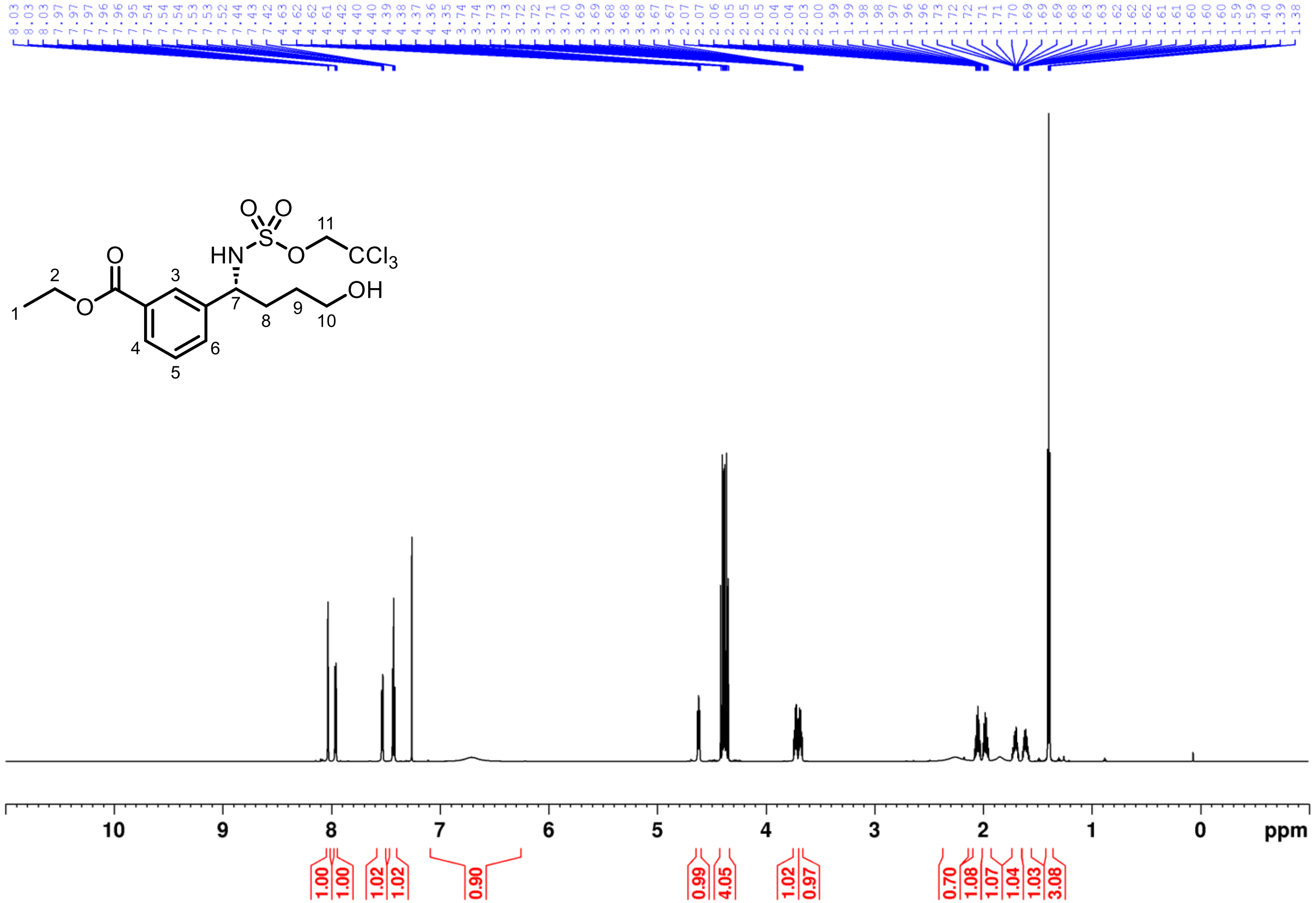

^{13}C NMR (176 MHz, $CDCl_3$) for *ethyl (R)-3-(4-hydroxy-1-(((2,2,2-trichloroethoxy)sulfonyl)amino)butyl)benzoate* (**7b**)

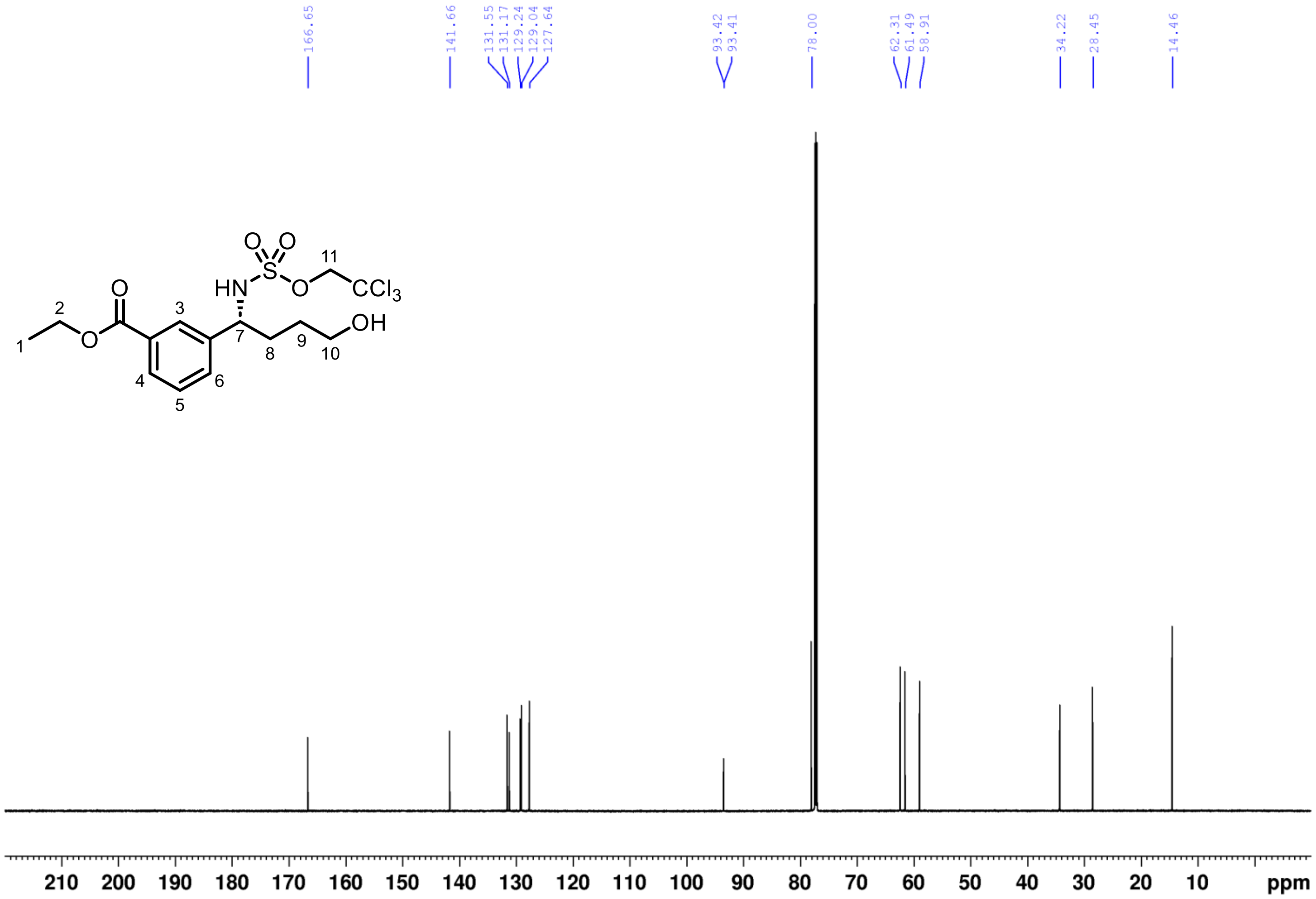

^{1}H NMR (700 MHz, CD_3OD) for *2,2,2-trichloroethyl (R)-(1-(3-chloro-2-methylphenyl)-4-hydroxybutyl)sulfamate* (**7c**)

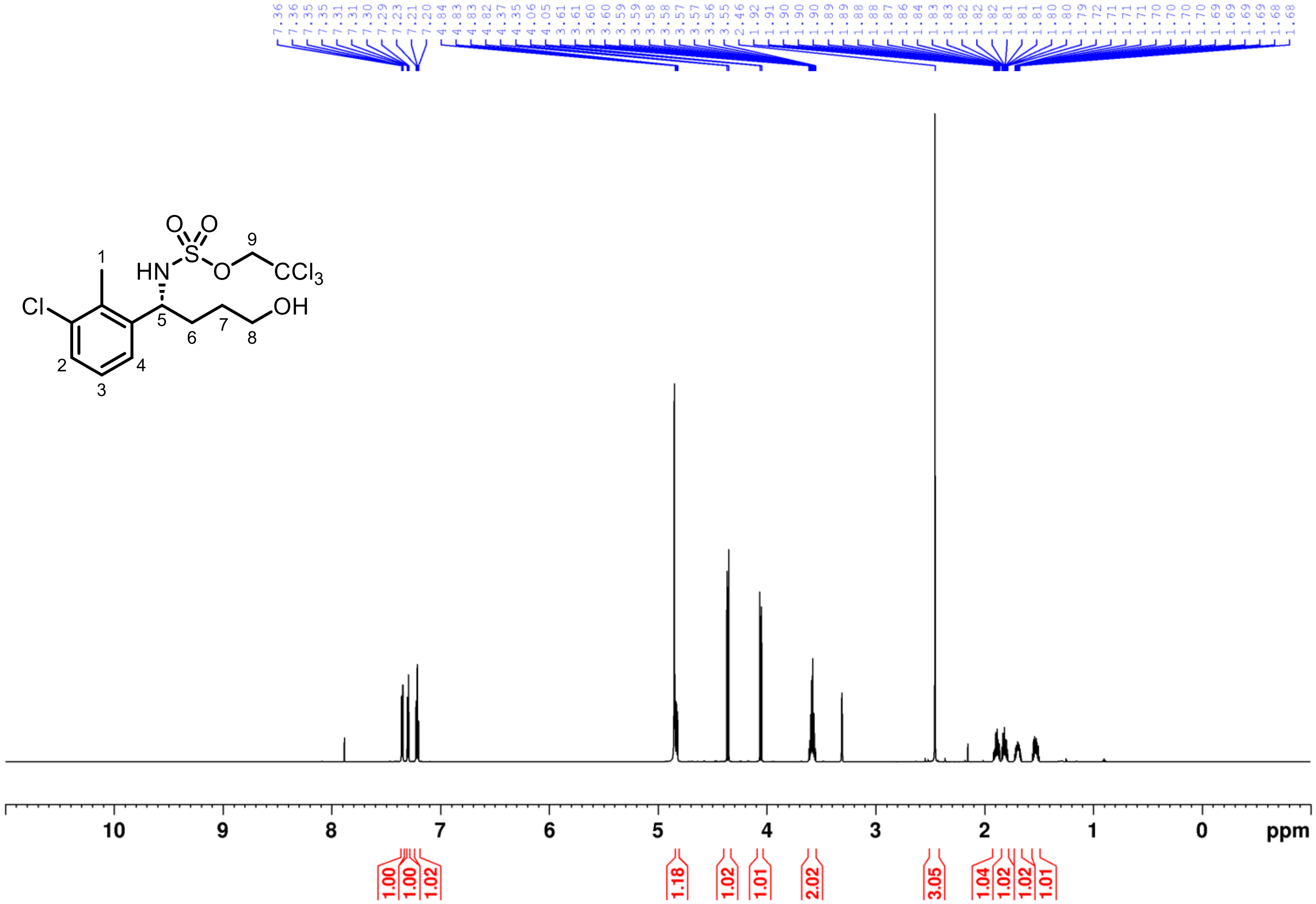

^{13}C NMR (176 MHz, CD_3OD) for *2,2,2-trichloroethyl (R)-(1-(3-chloro-2-methylphenyl)-4-hydroxybutyl)sulfamate* (**7c**)

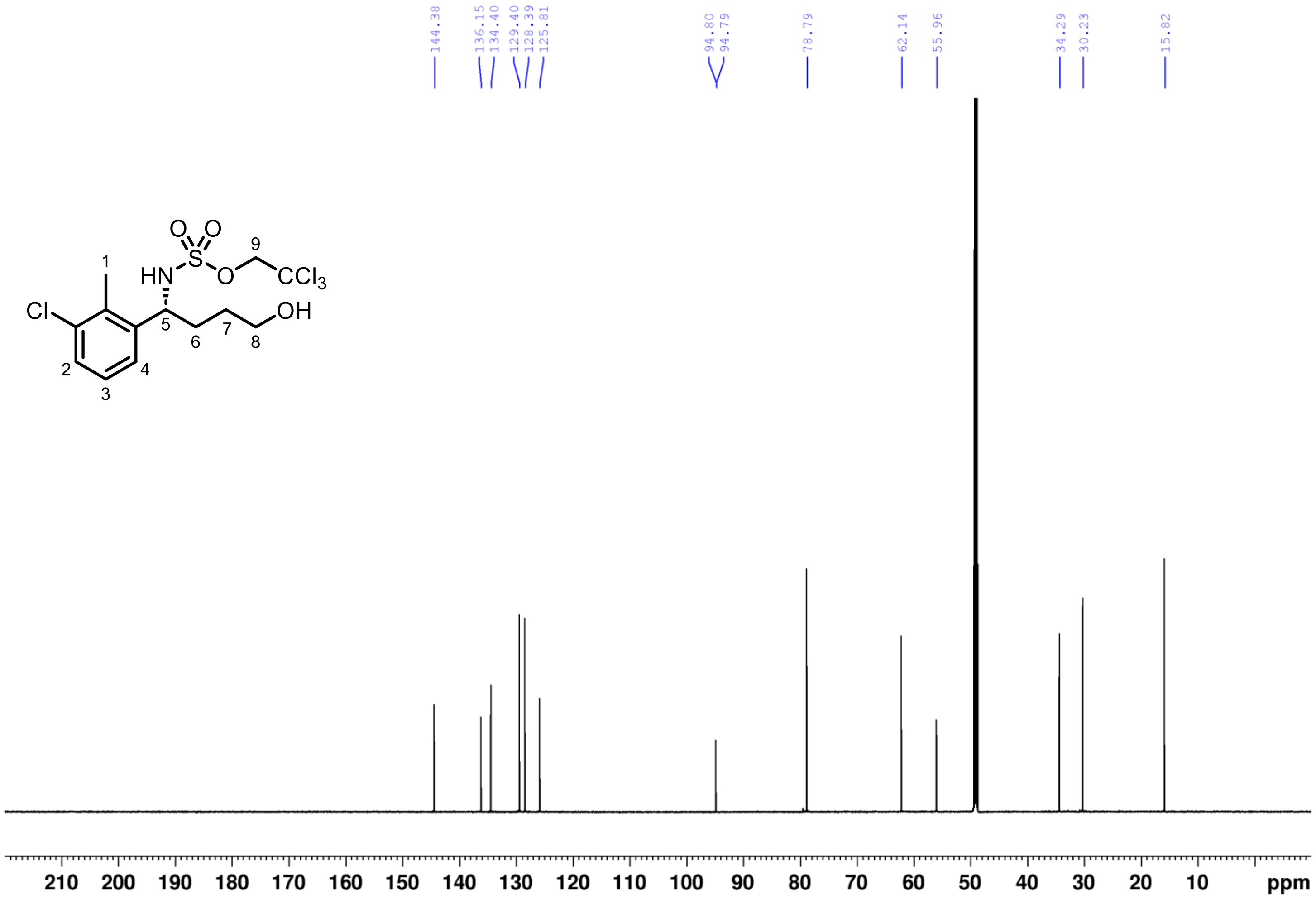

^{1}H NMR (400 MHz, CDCl$_3$) for *2,2,2-trichloroethyl (R)-(5-hydroxy-1-phenylpentyl)sulfamate* (**7d**)

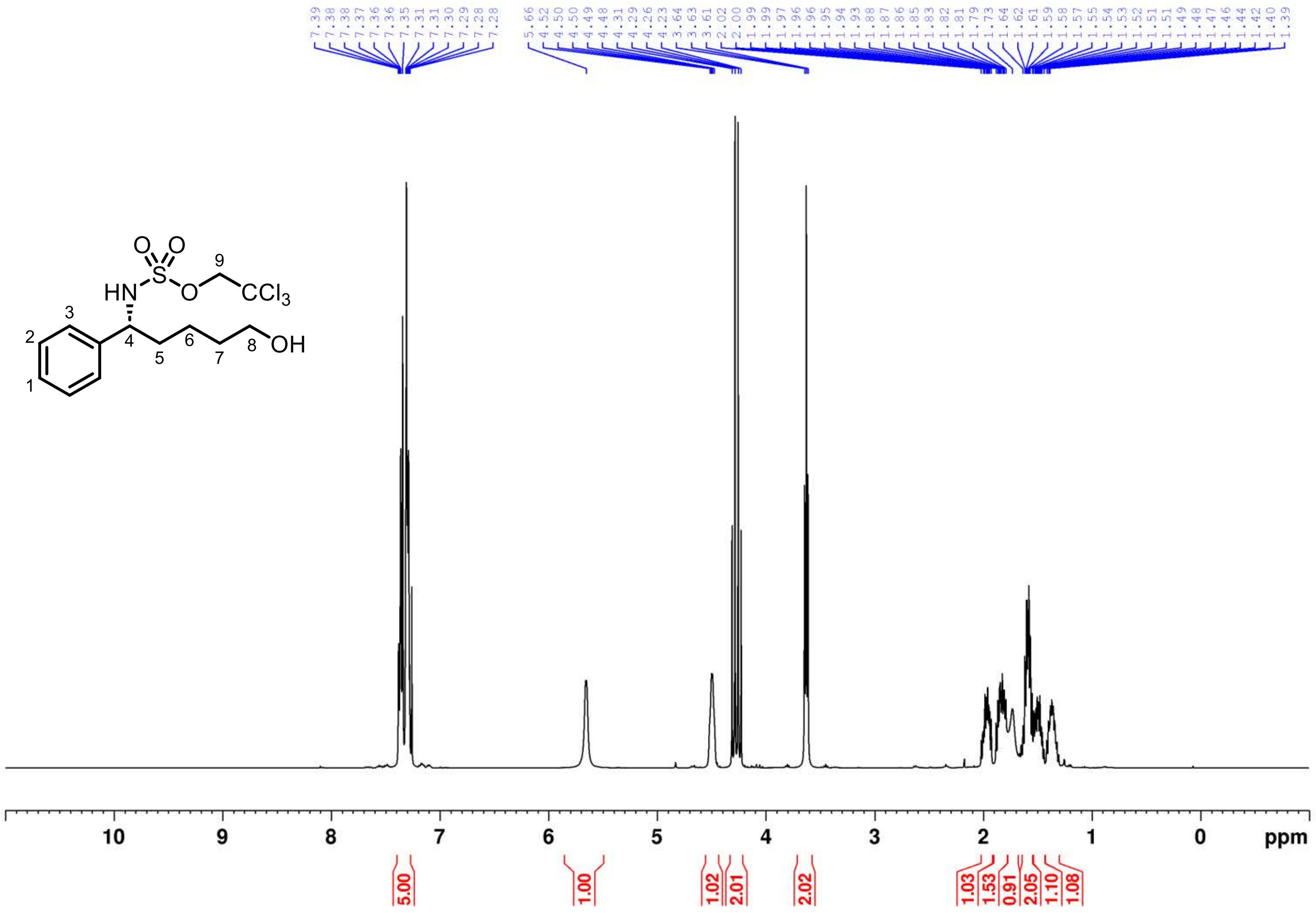

^{13}C NMR (101 MHz, CDCl$_3$) for *2,2,2-trichloroethyl (R)-(5-hydroxy-1-phenylpentyl)sulfamate* (**7d**)

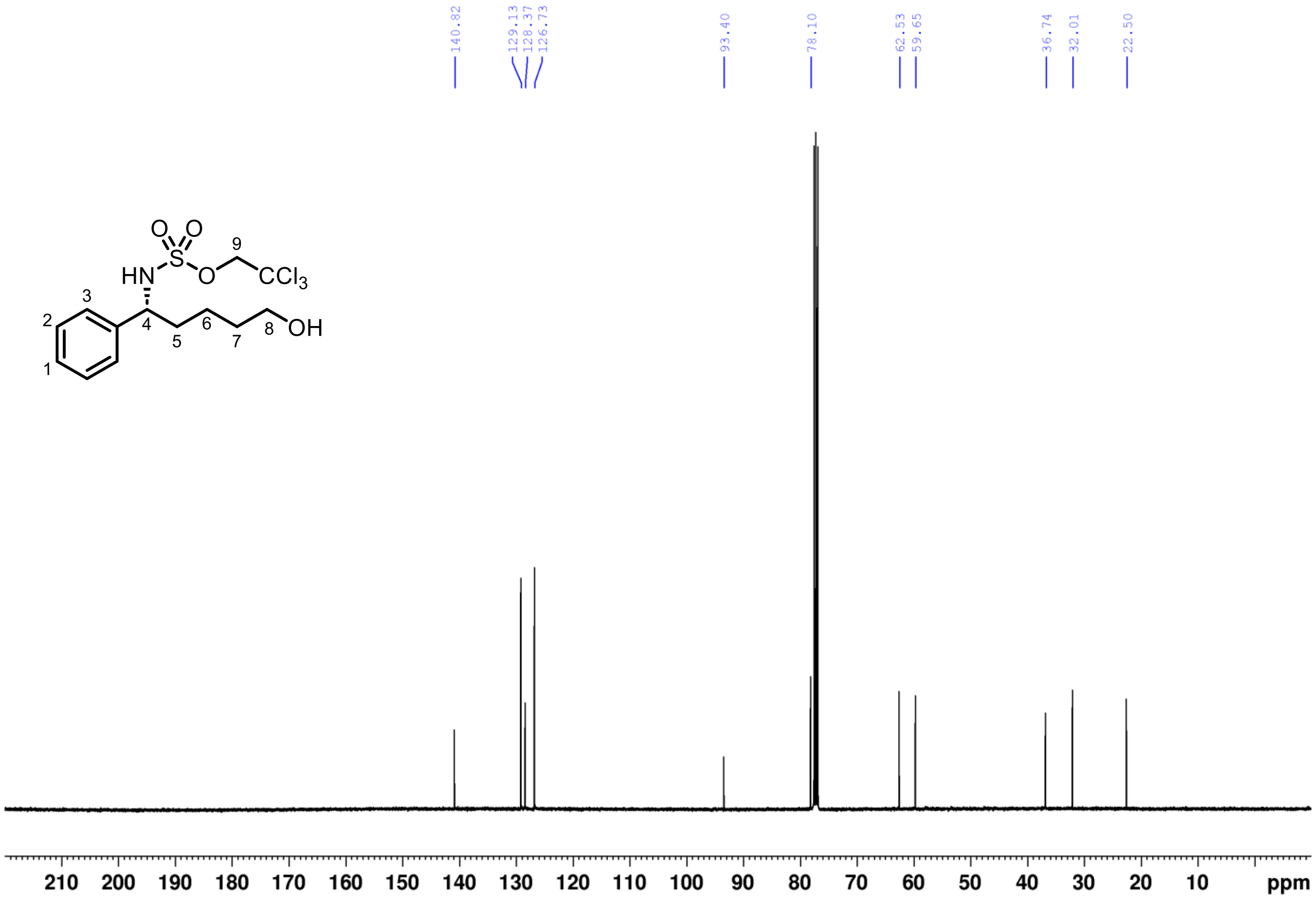

^{1}H NMR (700 MHz, $CDCl_3$) for *2,2,2-trichloroethyl (R)-(1-(4-(tert-butyl)phenyl)-5-hydroxypentyl)sulfamate* (**7e**)

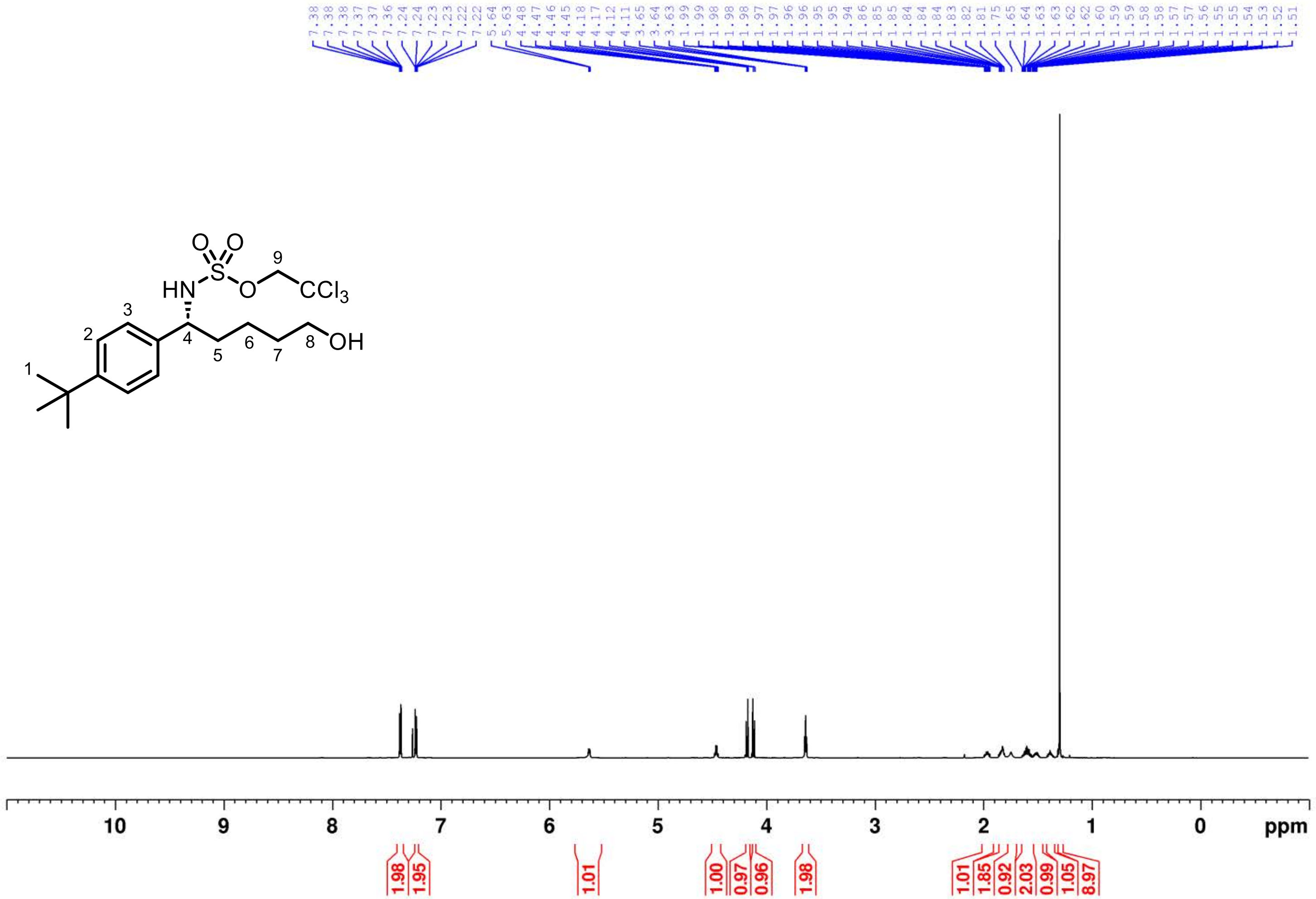

^{13}C NMR (176 MHz, $CDCl_3$) for *2,2,2-trichloroethyl (R)-(1-(4-(tert-butyl)phenyl)-5-hydroxypentyl)sulfamate* (**7e**)

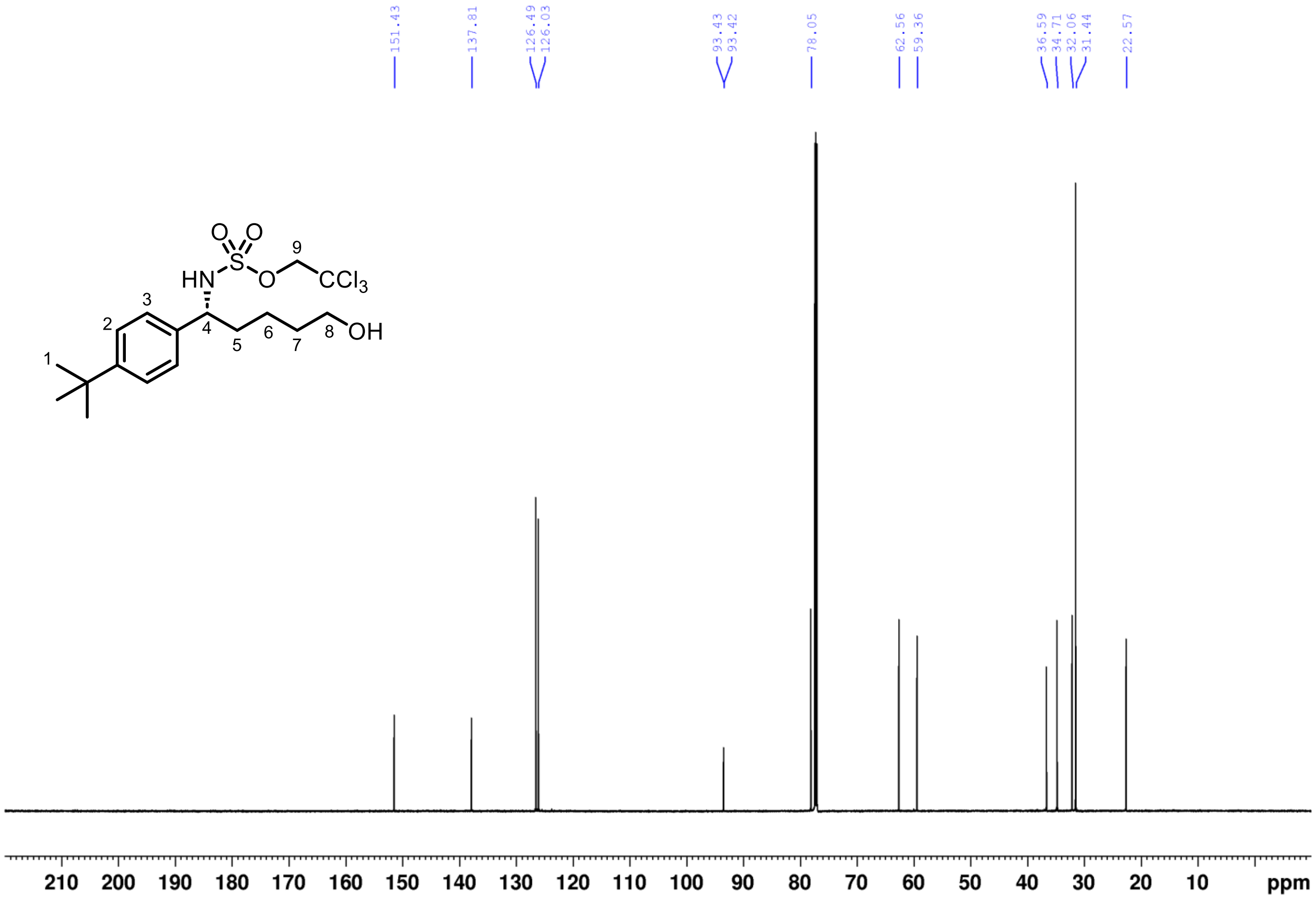

^{1}H NMR (700 MHz, $CDCl_3$) for *2,2,2-trichloroethyl (R)-(1-(3-fluoro-2-methylphenyl)-5-hydroxypentyl)sulfamate* (**7f**)

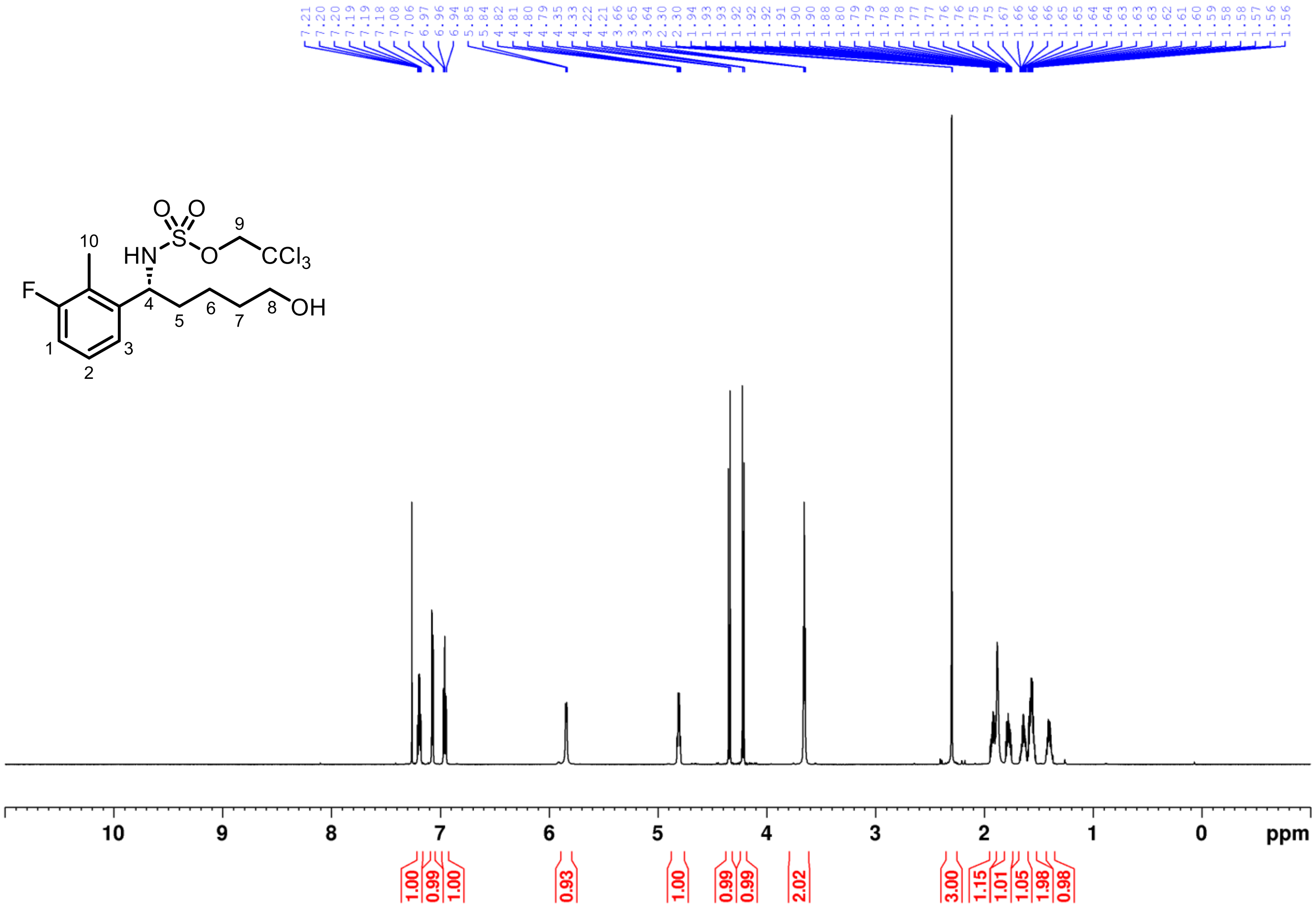

^{13}C NMR (176 MHz, $CDCl_3$) for *2,2,2-trichloroethyl (R)-(1-(3-fluoro-2-methylphenyl)-5-hydroxypentyl)sulfamate* (**7f**)

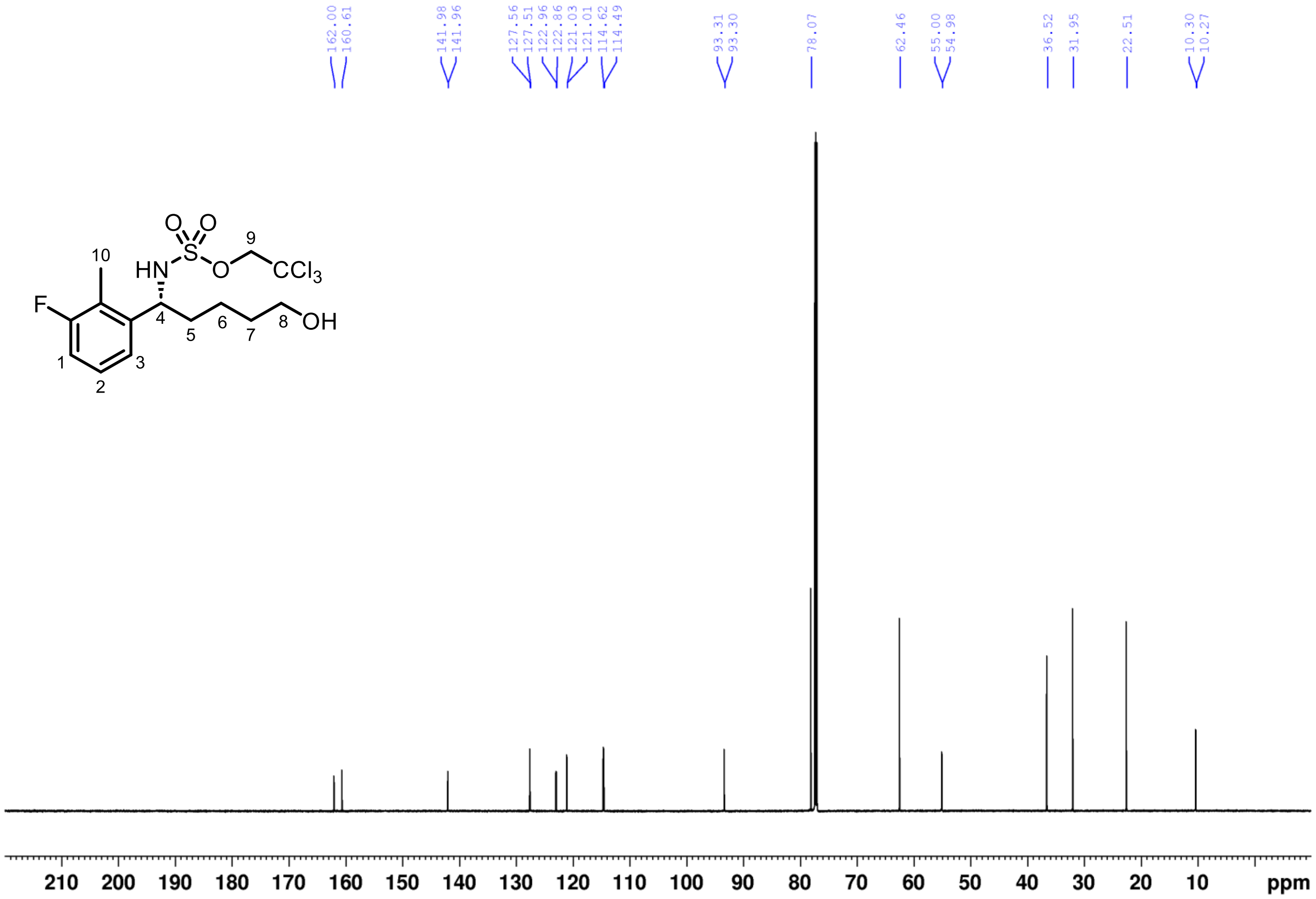

^{19}F NMR (376 MHz, $CDCl_3$) for *2,2,2-trichloroethyl (R)-(1-(3-fluoro-2-methylphenyl)-5-hydroxypentyl)sulfamate* (**7f**)

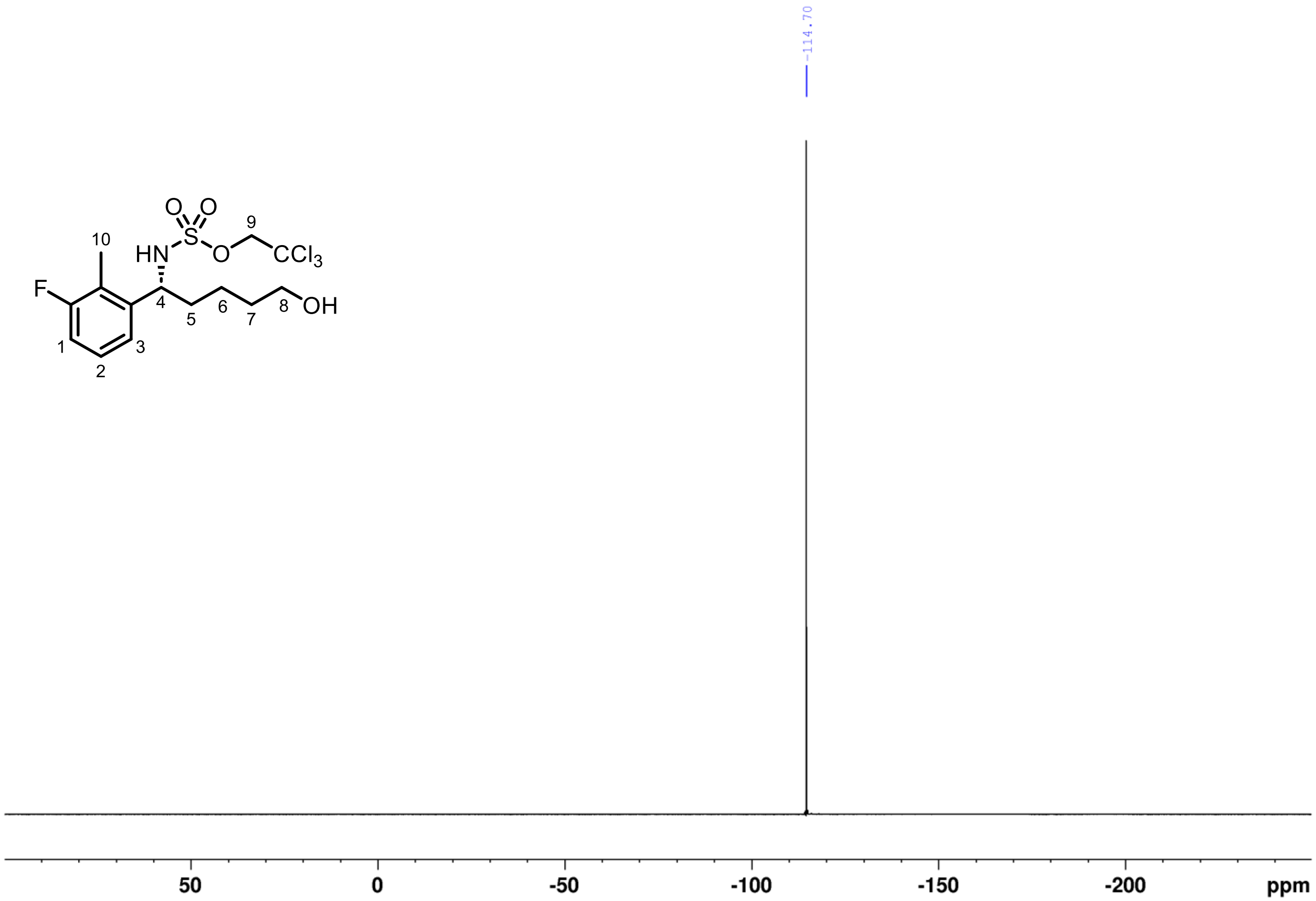

^{1}H NMR (500 MHz, CDCl$_3$)** for *2,2,3,3,3-pentafluoropropyl (S)-3-(hydroxymethyl)-2,2-dimethylaziridine-1-sulfonate* (**9a**)

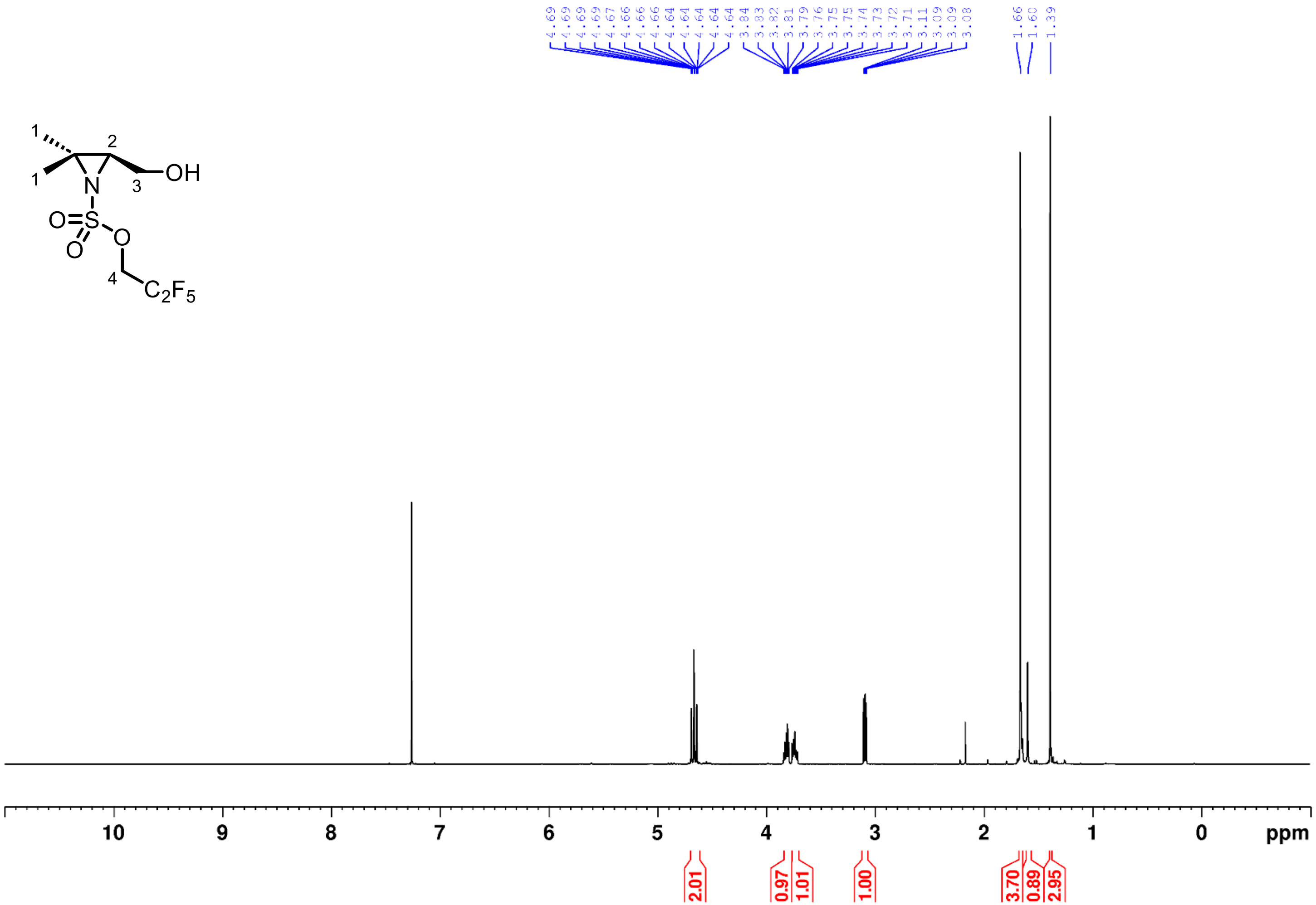

^{13}C NMR (126 MHz, CDCl$_3$) for *2,2,3,3,3-pentafluoropropyl (S)-3-(hydroxymethyl)-2,2-dimethylaziridine-1-sulfonate* (**9a**)

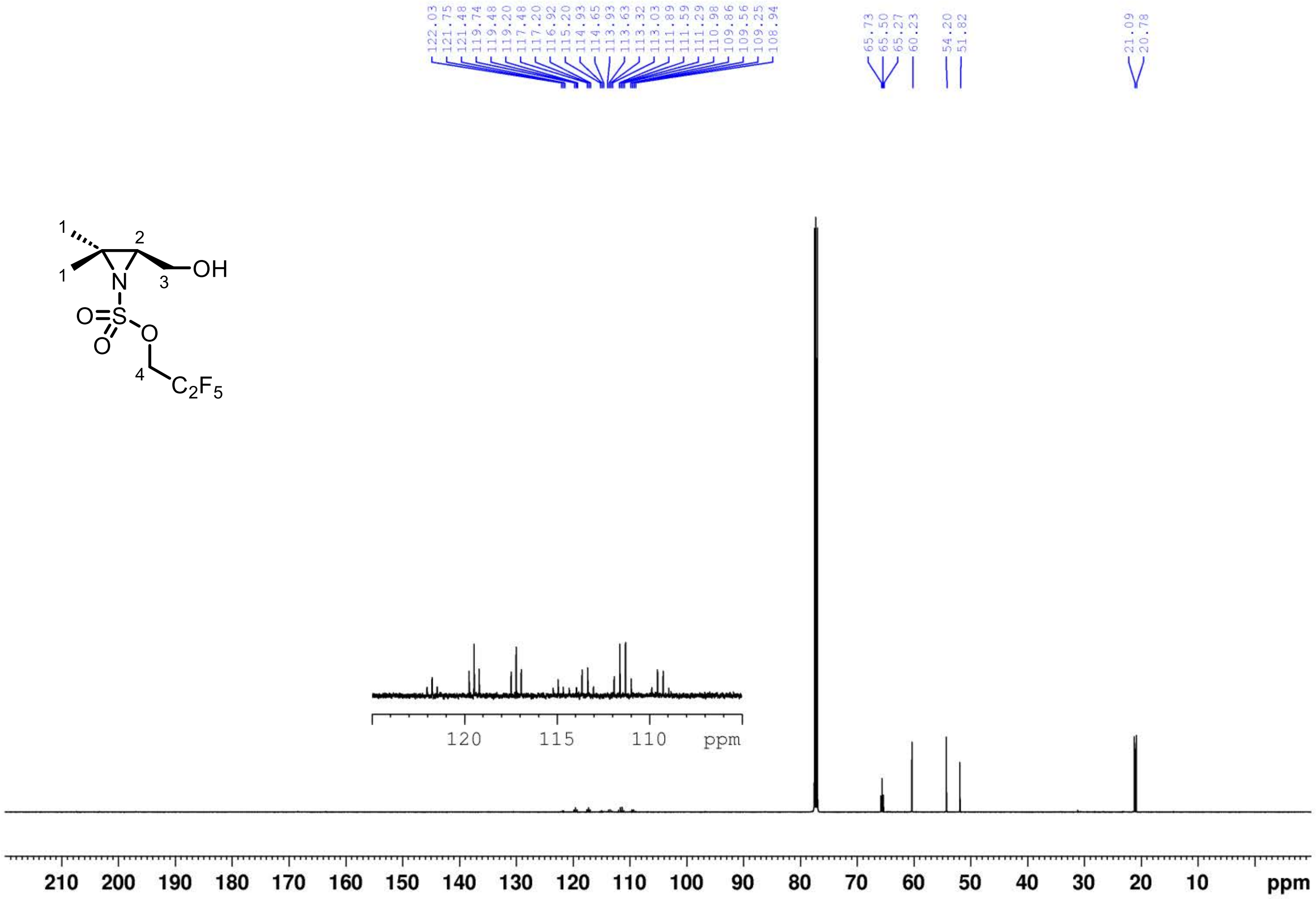

^{19}F NMR (376 MHz, CDCl$_3$) for *2,2,3,3,3-pentafluoropropyl (S)-3-(hydroxymethyl)-2,2-dimethylaziridine-1-sulfonate* (**9a**) NH668

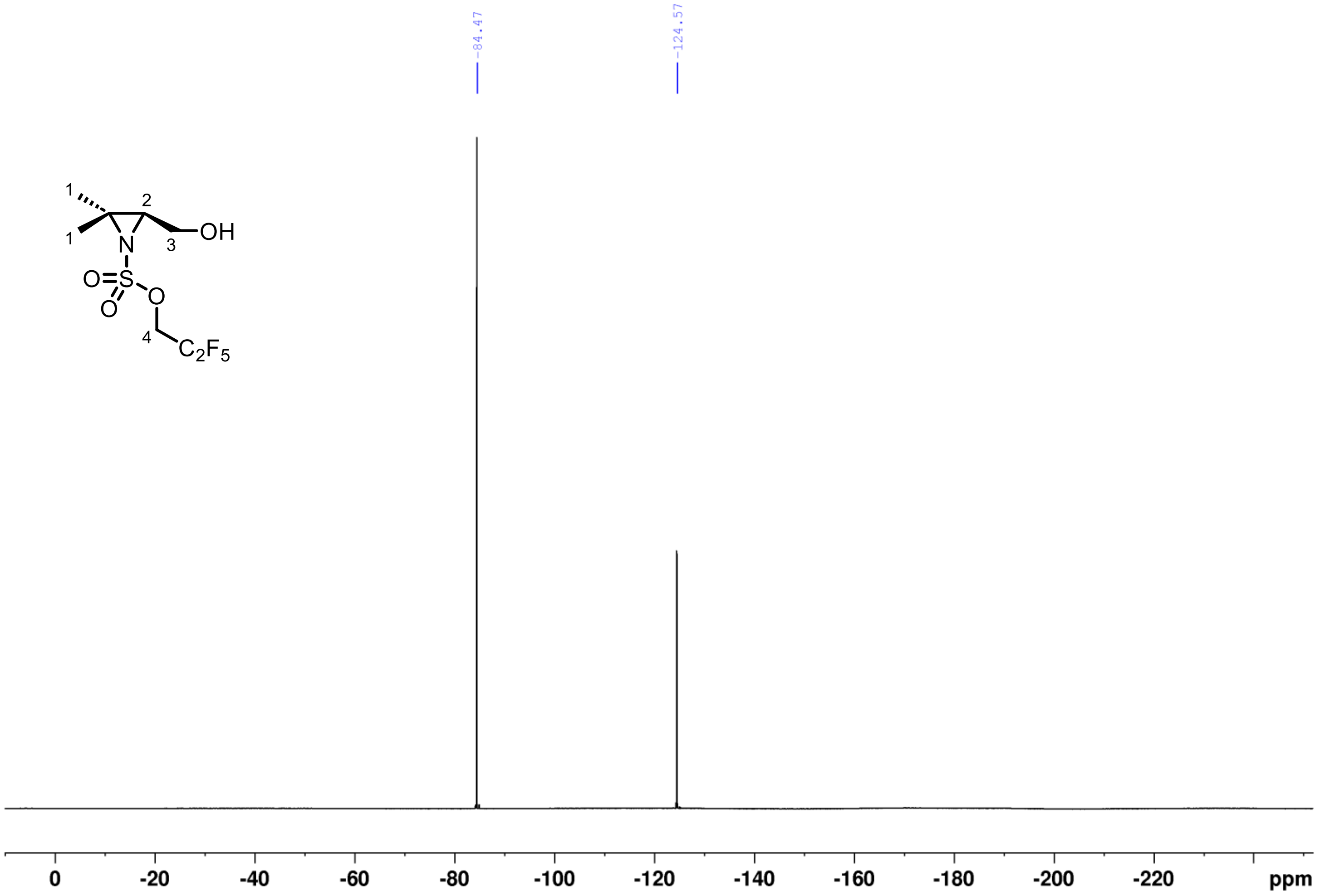

^{1}H NMR (700 MHz, $CDCl_3$) for *2,2,3,3,3-pentafluoropropyl (R)-(1-hydroxy-3-methyl-3-(phenylthio)butan-2-yl)sulfamate* and *2,2,3,3,3-pentafluoropropyl (R)-(4-hydroxy-2-methyl-3-(phenylthio)butan-2-yl)sulfamate.* **Obtained following derivatisation of 9a**

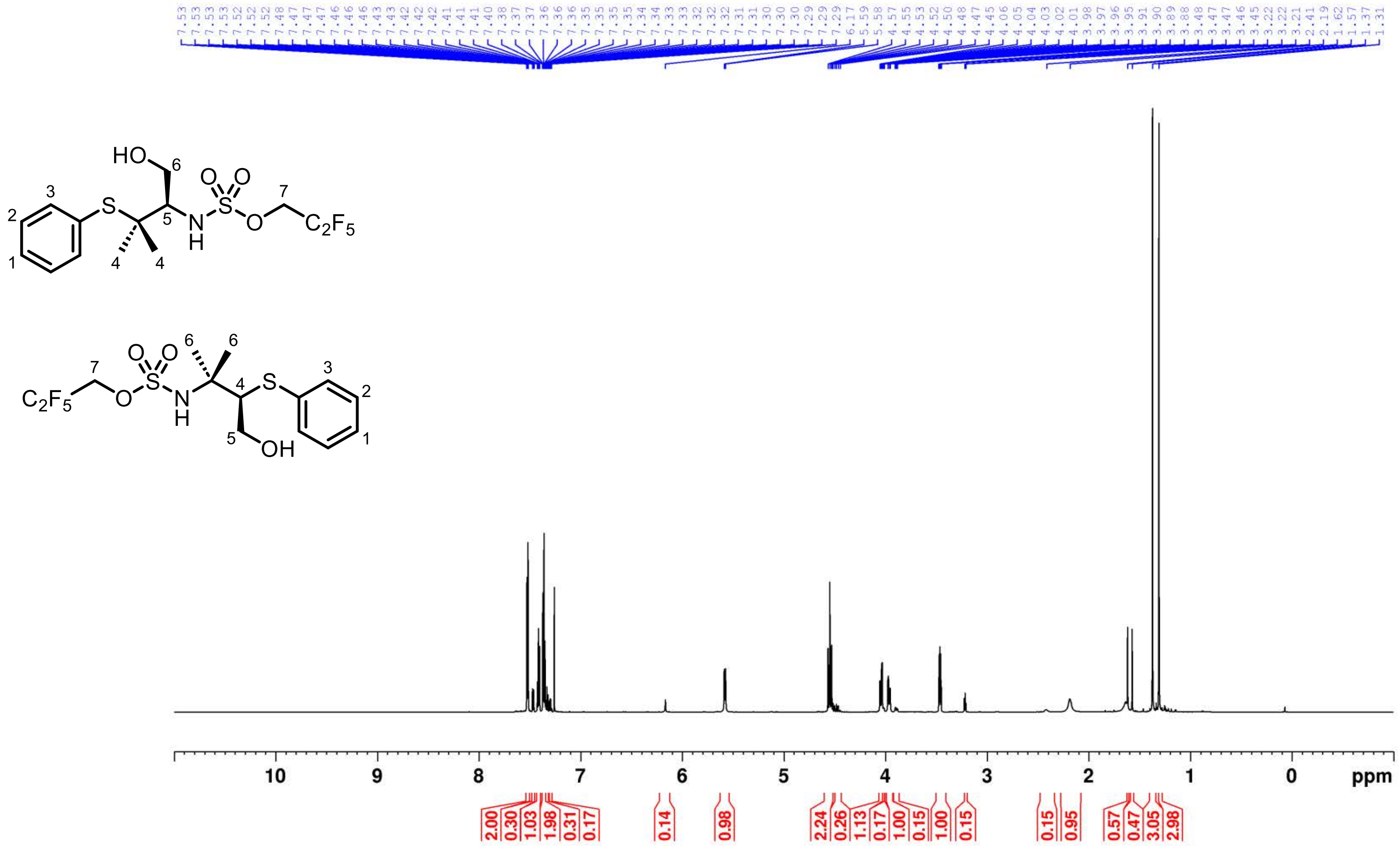

^{13}C NMR (176 MHz, $CDCl_3$) for *2,2,3,3,3-pentafluoropropyl (R)-(1-hydroxy-3-methyl-3-(phenylthio)butan-2-yl)sulfamate* and *2,2,3,3,3-pentafluoropropyl (R)-(4-hydroxy-2-methyl-3-(phenylthio)butan-2-yl)sulfamate.* **Obtained following derivatisation of 9a**

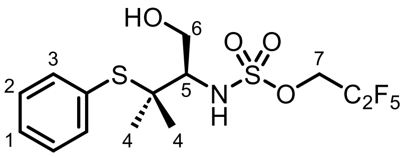

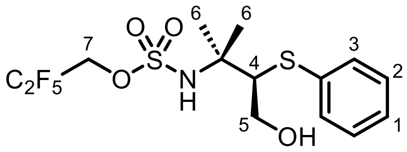

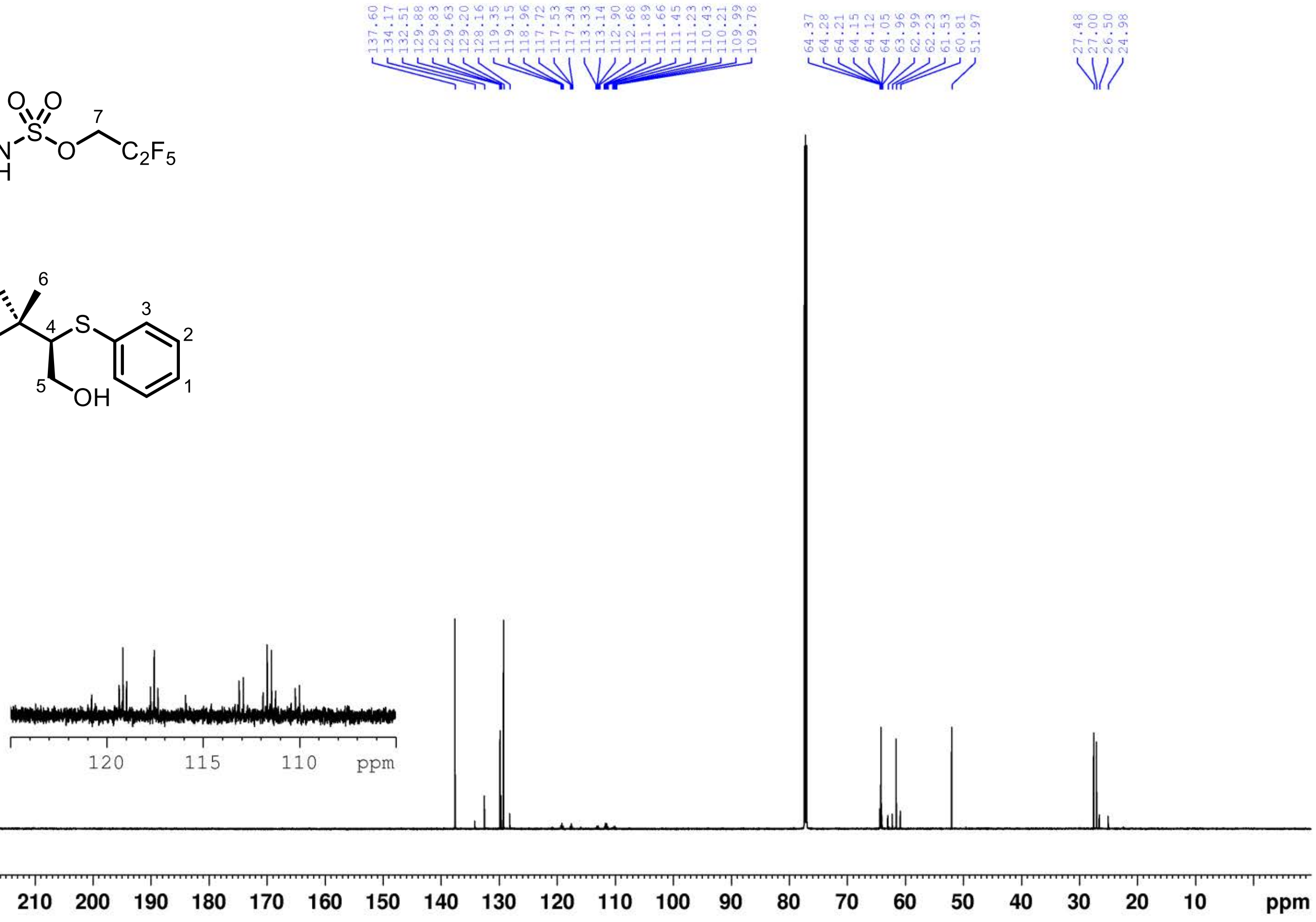

^{19}F NMR (376 MHz, CDCl$_3$) for *2,2,3,3,3-pentafluoropropyl (R)-(1-hydroxy-3-methyl-3-(phenylthio)butan-2-yl)sulfamate* and *2,2,3,3,3-pentafluoropropyl (R)-(4-hydroxy-2-methyl-3-(phenylthio)butan-2-yl)sulfamate.* **Obtained following derivatisation of 9a**

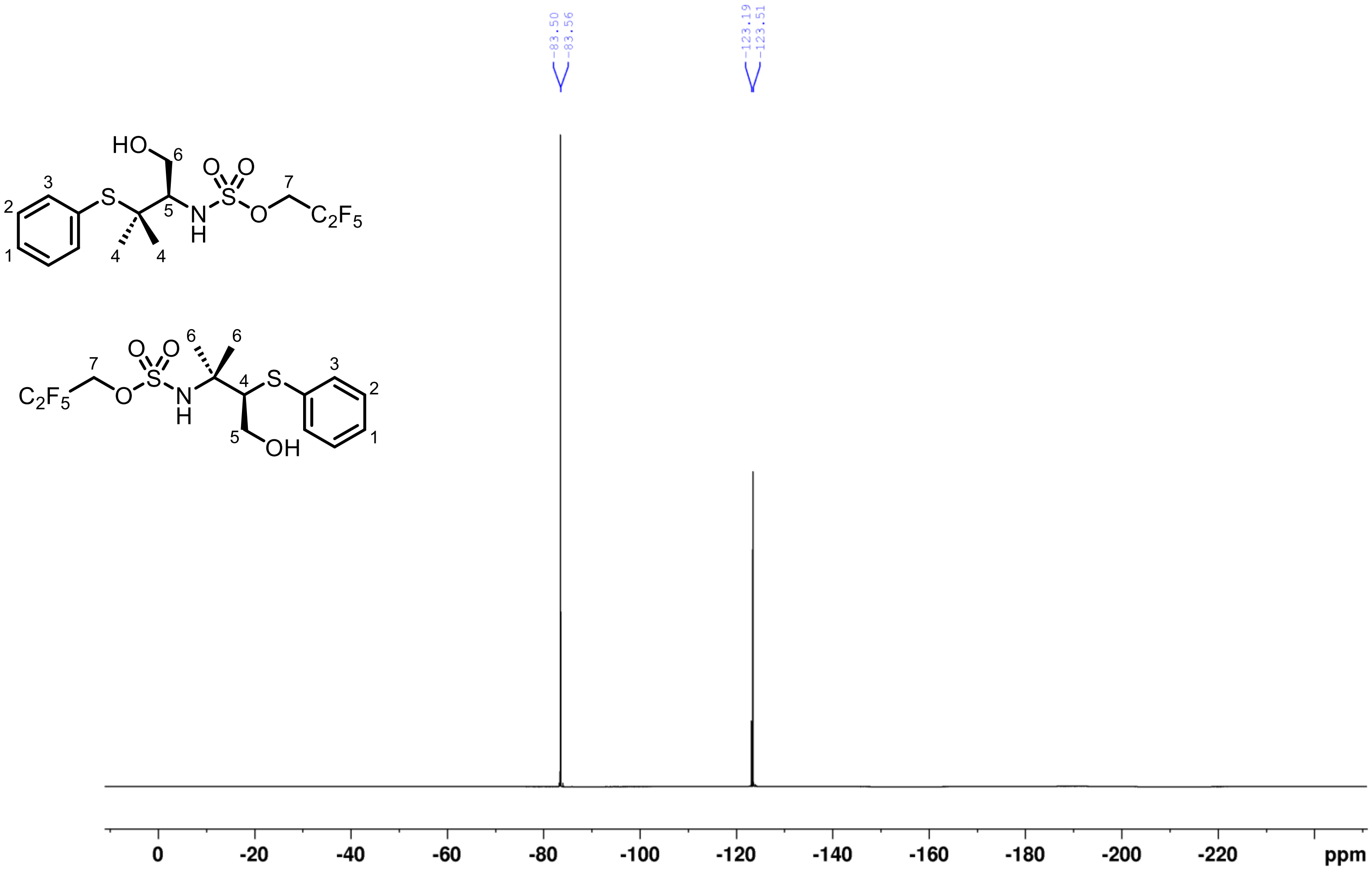

^{1}H NMR (500 MHz, $CDCl_3$) for *2,2,3,3,3-pentafluoropropyl (S)-2,2-dibutyl-3-(hydroxymethyl)aziridine-1-sulfonate* (**9b**)

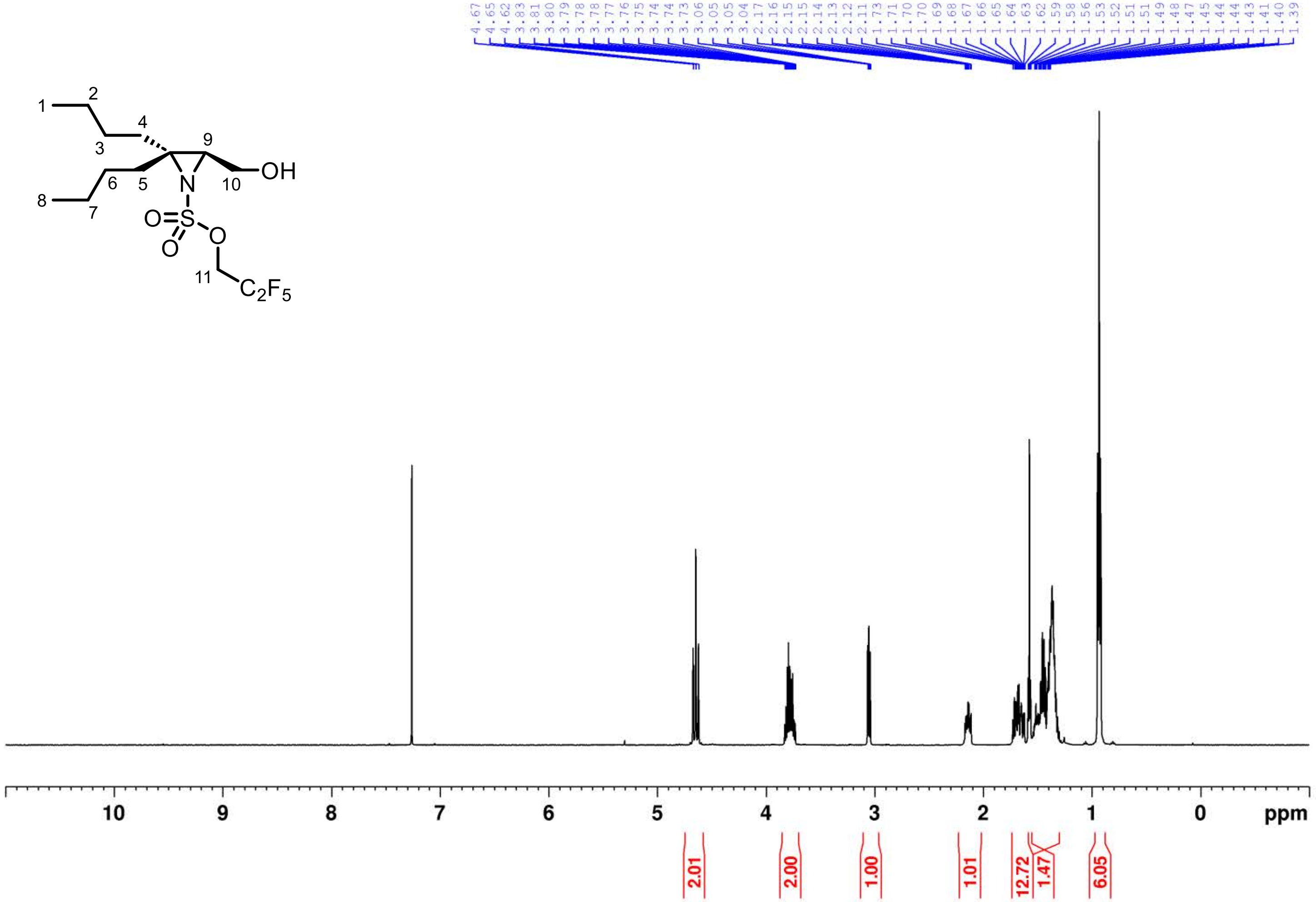

^{13}C NMR (126 MHz, CDCl$_3$) for *2,2,3,3,3-pentafluoropropyl (S)-2,2-dibutyl-3-(hydroxymethyl)aziridine-1-sulfonate* (**9b**)

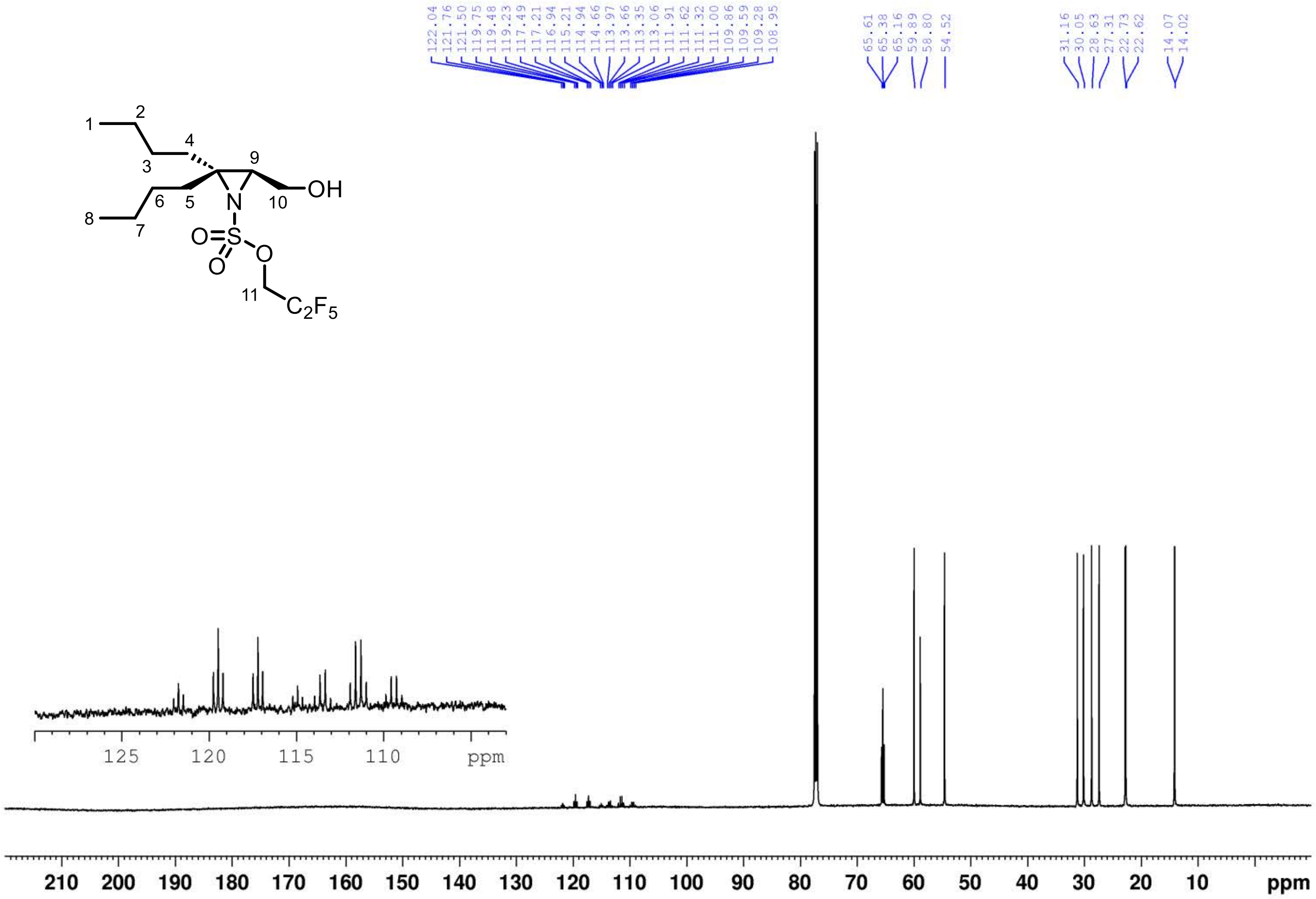

¹⁹F NMR (376 MHz, CDCl$_3$) for *2,2,3,3,3-pentafluoropropyl (S)-2,2-dibutyl-3-(hydroxymethyl)aziridine-1-sulfonate* (**9b**)

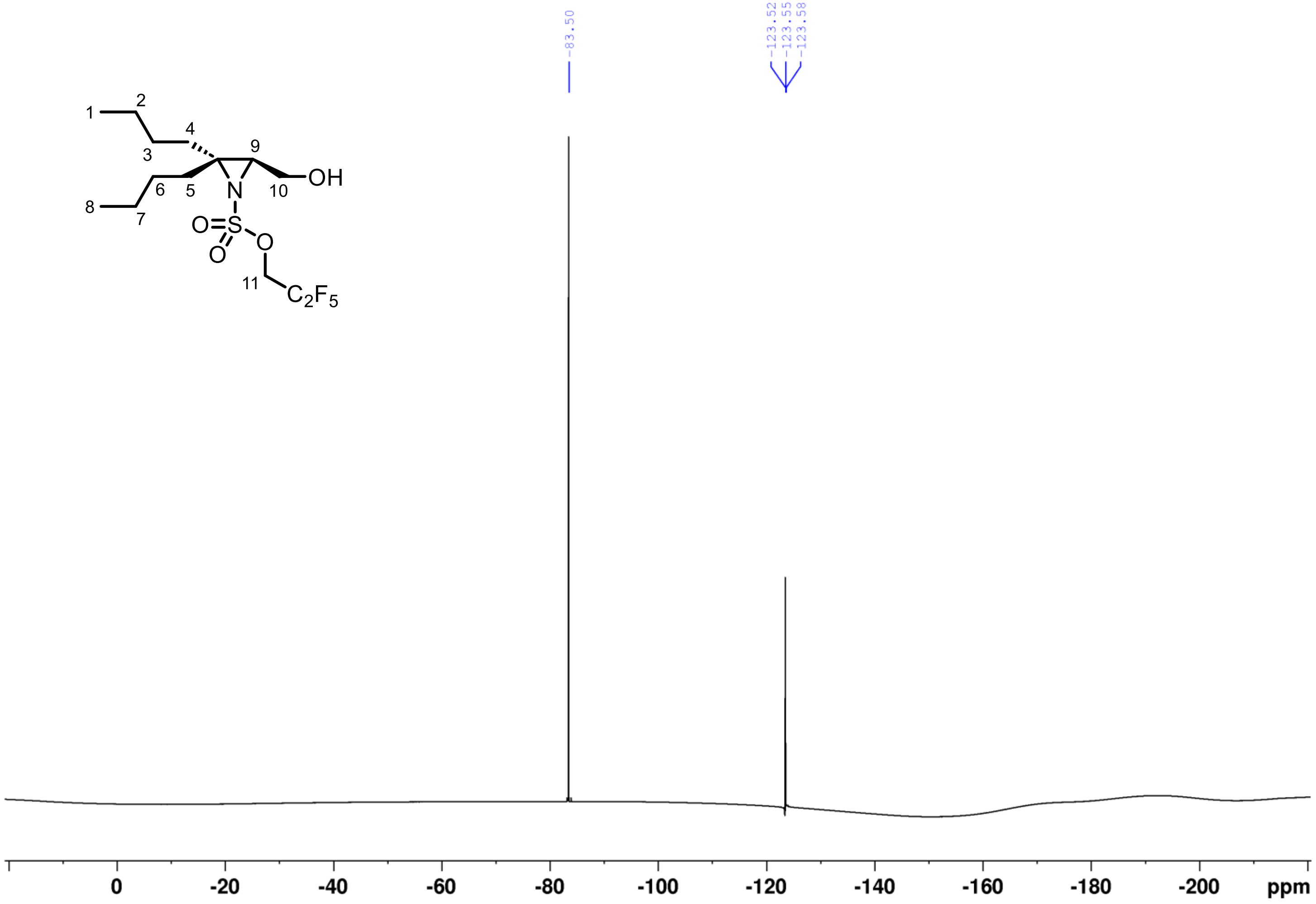

^{1}H NMR (700 MHz, $CDCl_3$) for *2,2,3,3,3-pentafluoropropyl (R)-(3-butyl-1-hydroxy-3-(phenylthio)heptan-2-yl)sulfamate.* **Obtained following derivatisation of 9b**

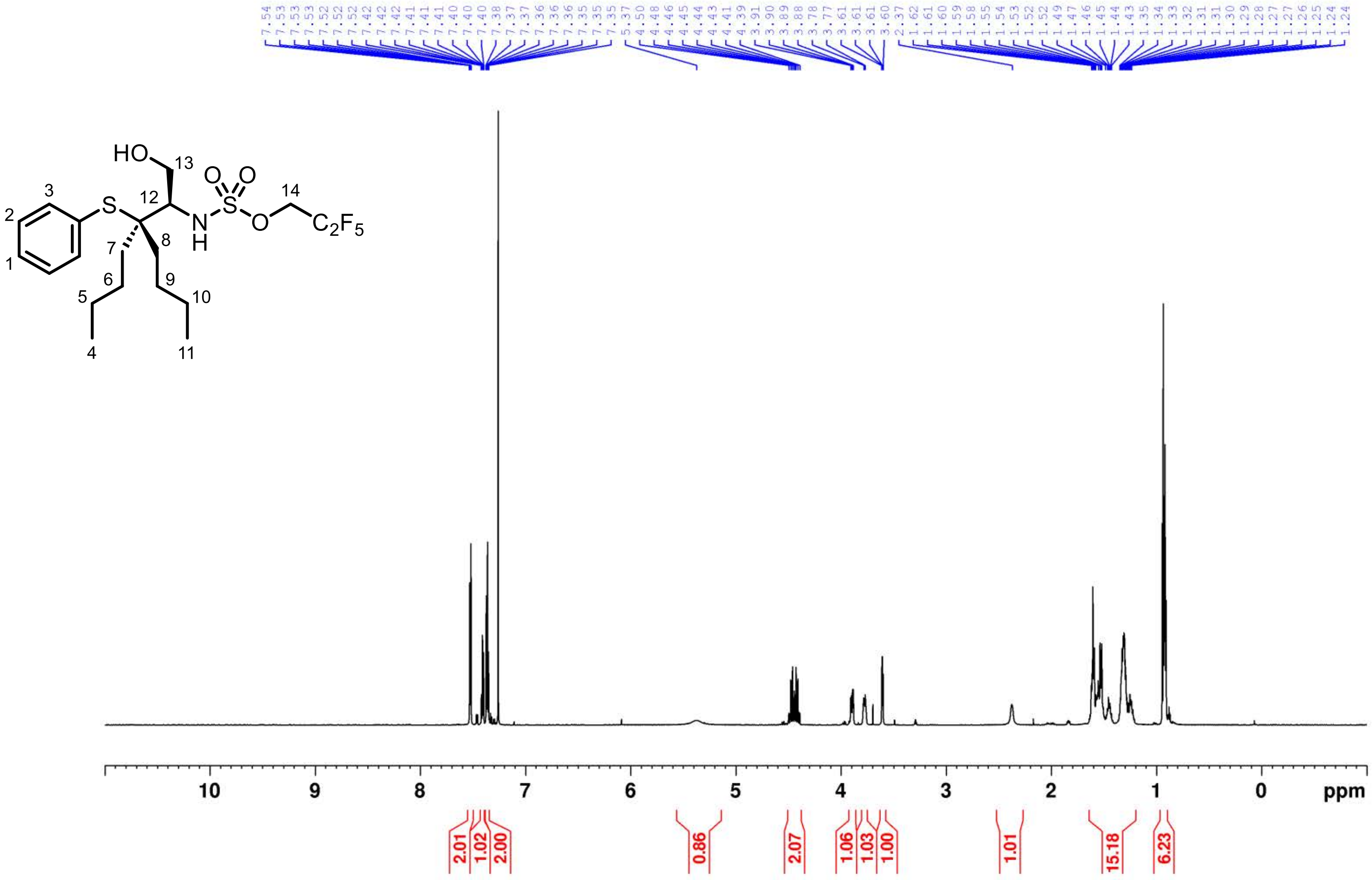

^{13}C NMR (176 MHz, CDCl$_3$) for *2,2,3,3,3-pentafluoropropyl (R)-(3-butyl-1-hydroxy-3-(phenylthio)heptan-2-yl)sulfamate.* **Obtained following derivatisation of 9b**

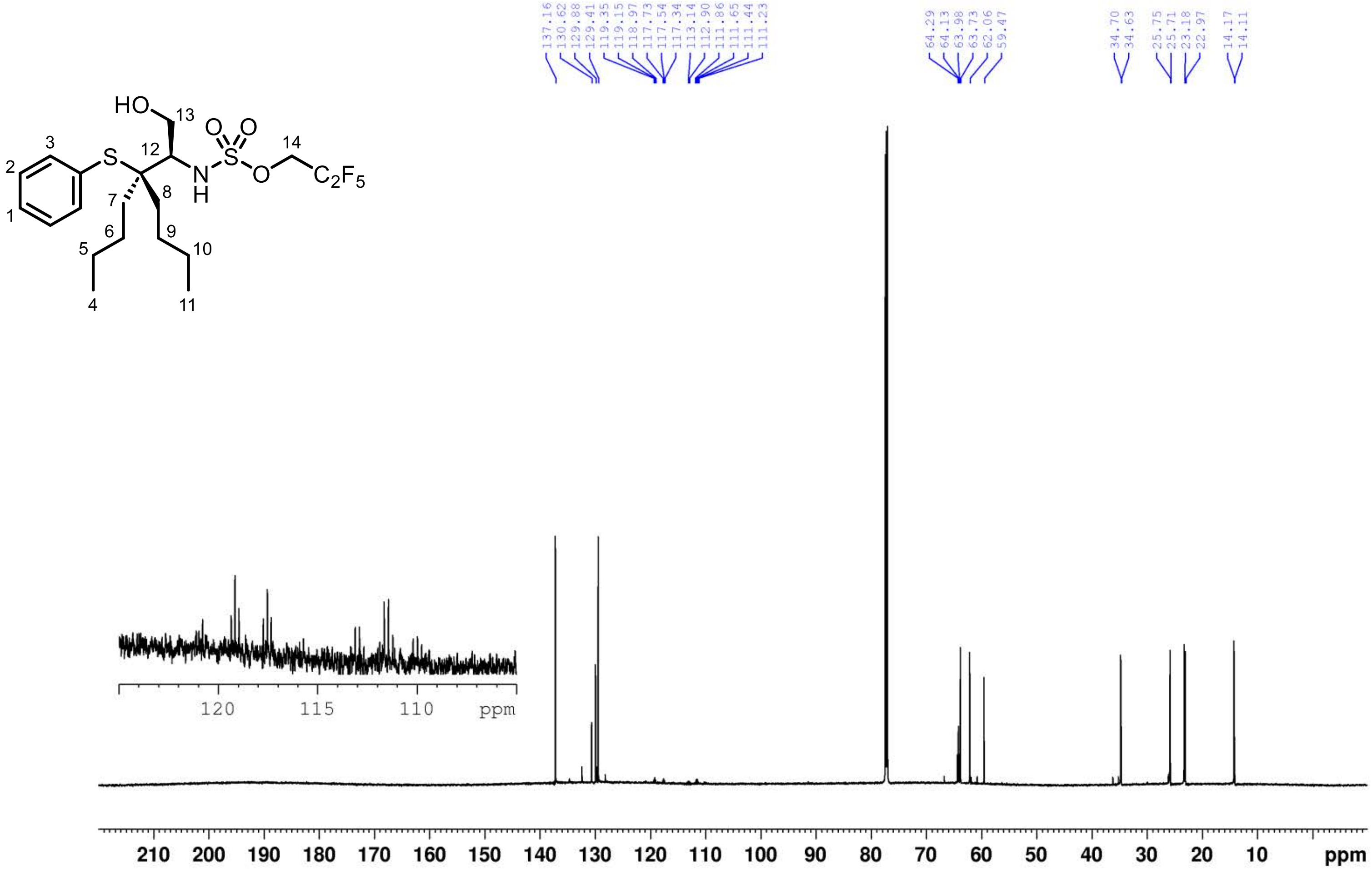

^{19}F NMR (376 MHz, $CDCl_3$) for *2,2,3,3,3-pentafluoropropyl (R)-(3-butyl-1-hydroxy-3-(phenylthio)heptan-2-yl)sulfamate.* **Obtained following derivatisation of 9b**

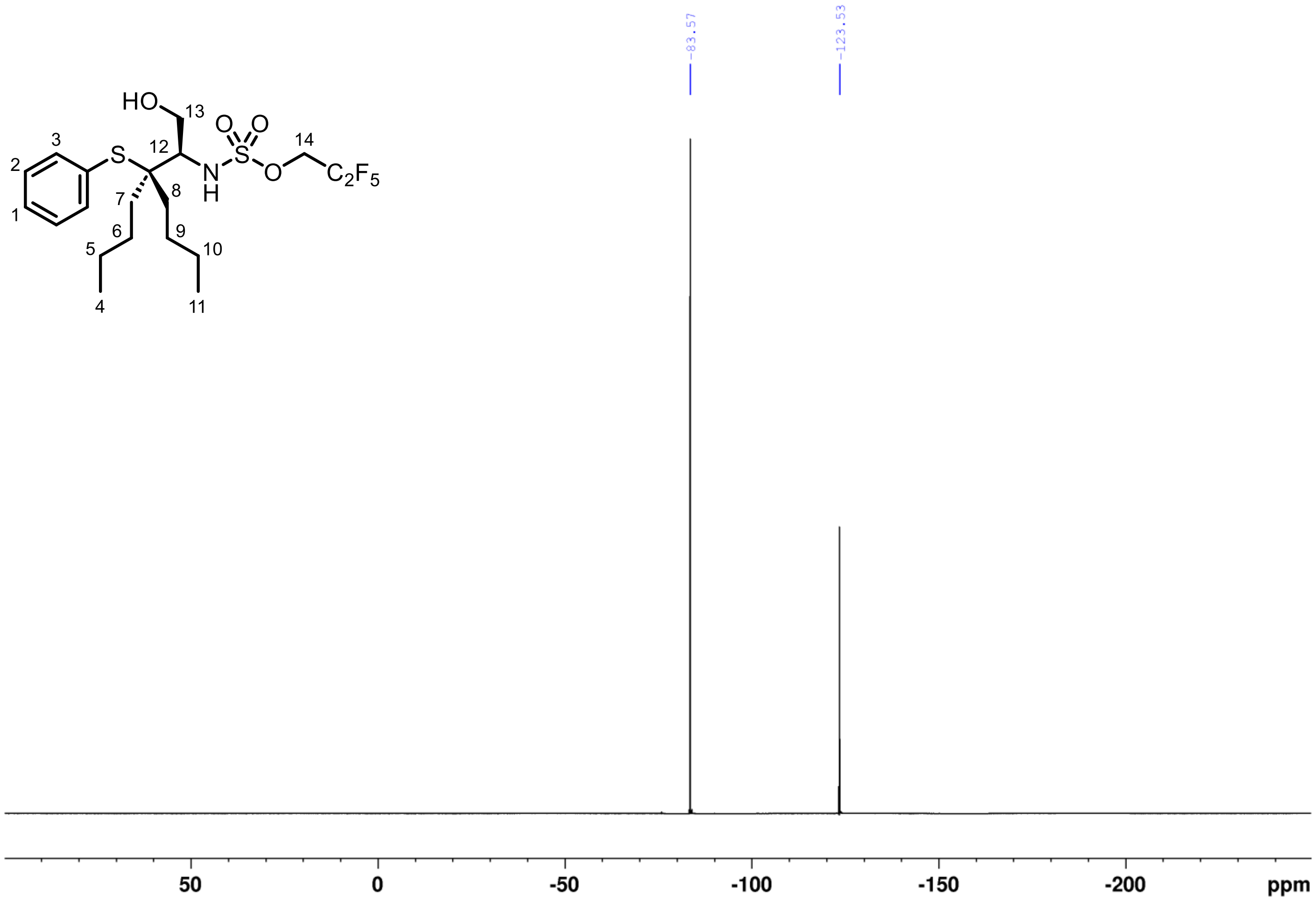

^{1}H NMR (700 MHz, $CDCl_3$) for *2,2,3,3,3-pentafluoropropyl (S)-2-(hydroxymethyl)-1-azaspiro[2.5]octane-1-sulfonate* (**9c**)

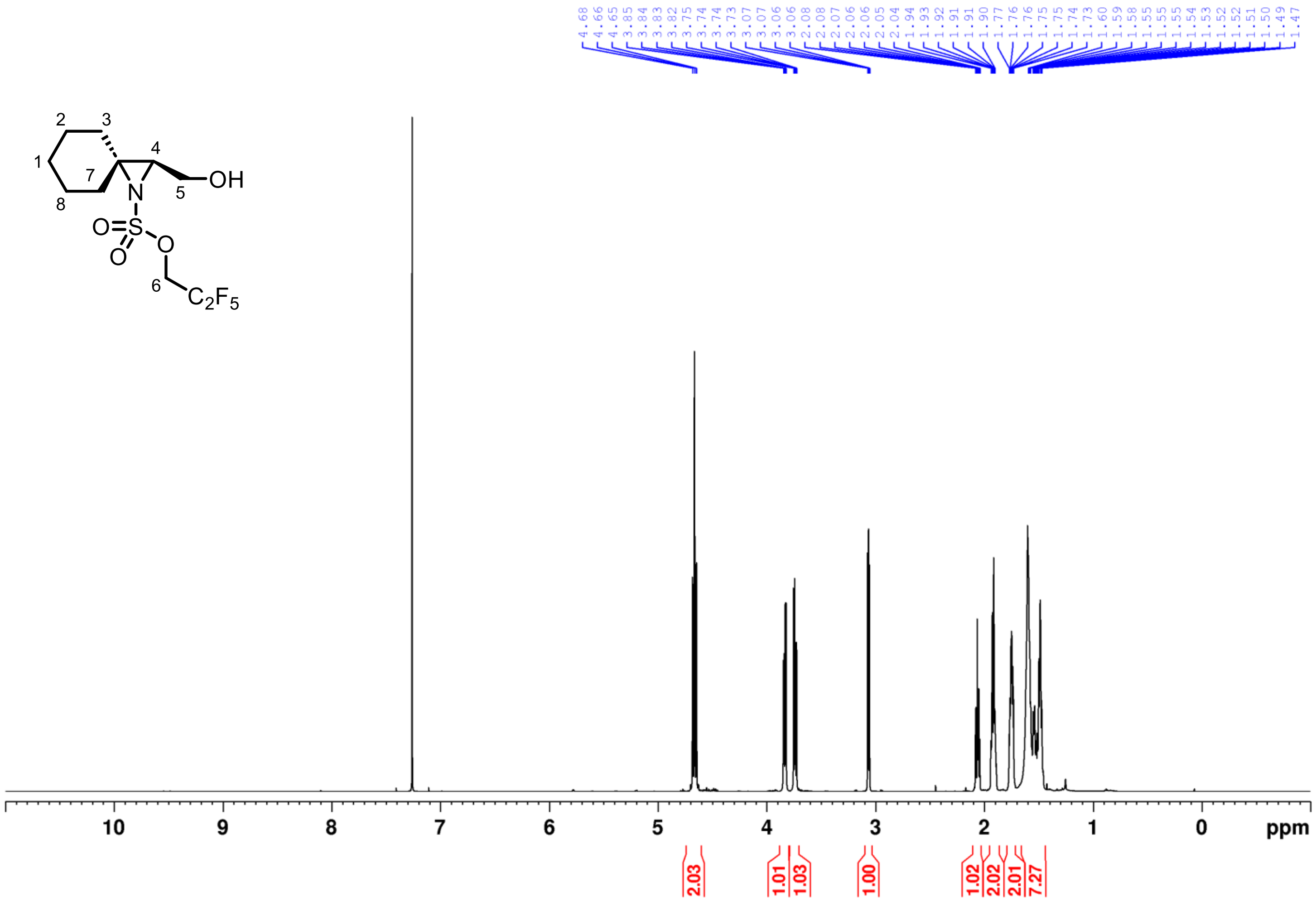

^{13}C NMR (176 MHz, CDCl$_3$) for *2,2,3,3,3-pentafluoropropyl (S)-2-(hydroxymethyl)-1-azaspiro[2.5]octane-1-sulfonate* (**9c**)

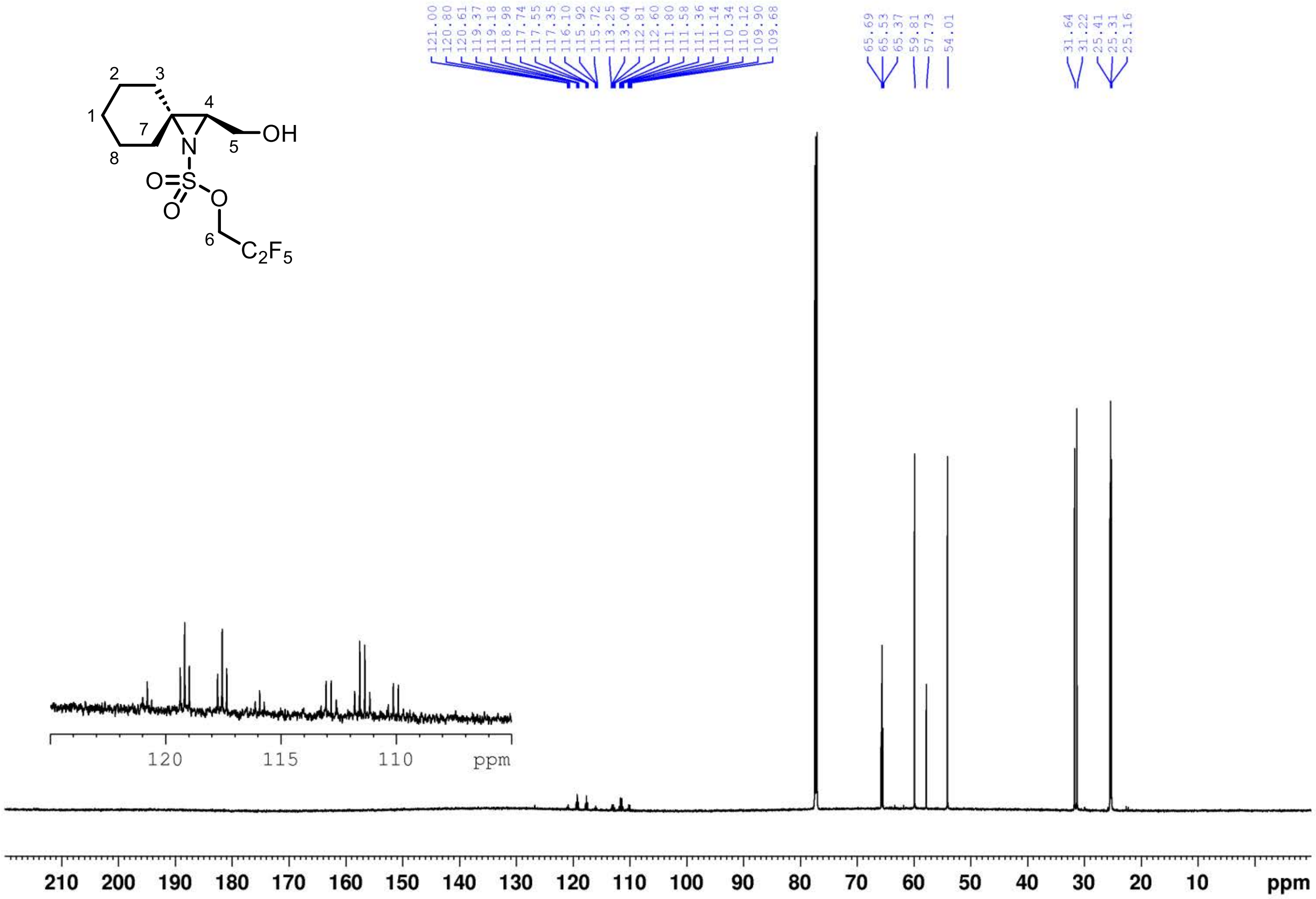

^{19}F NMR (376 MHz, CDCl$_3$) for *2,2,3,3,3-pentafluoropropyl (S)-2-(hydroxymethyl)-1-azaspiro[2.5]octane-1-sulfonate* (**9c**)

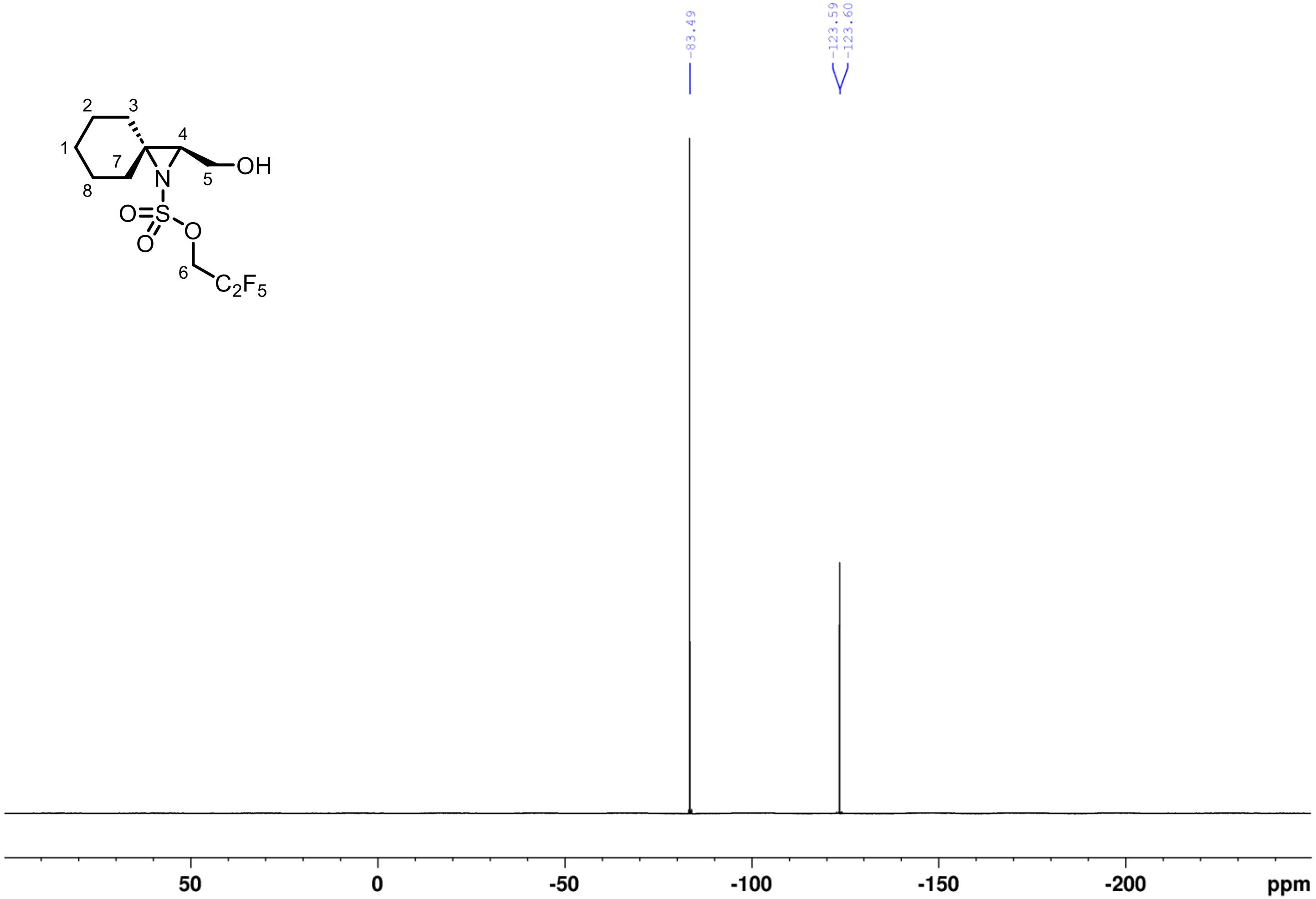

^{1}H NMR (700 MHz, $CDCl_3$) for *2,2,3,3,3-pentafluoropropyl (R)-(2-hydroxy-1-(1-(phenylthio)cyclohexyl)ethyl)sulfamate* and *2,2,3,3,3-pentafluoropropyl (R)-(1-(2-hydroxy-1-(phenylthio)ethyl)cyclohexyl)sulfamate.* **Obtained following derivatisation of 9c**

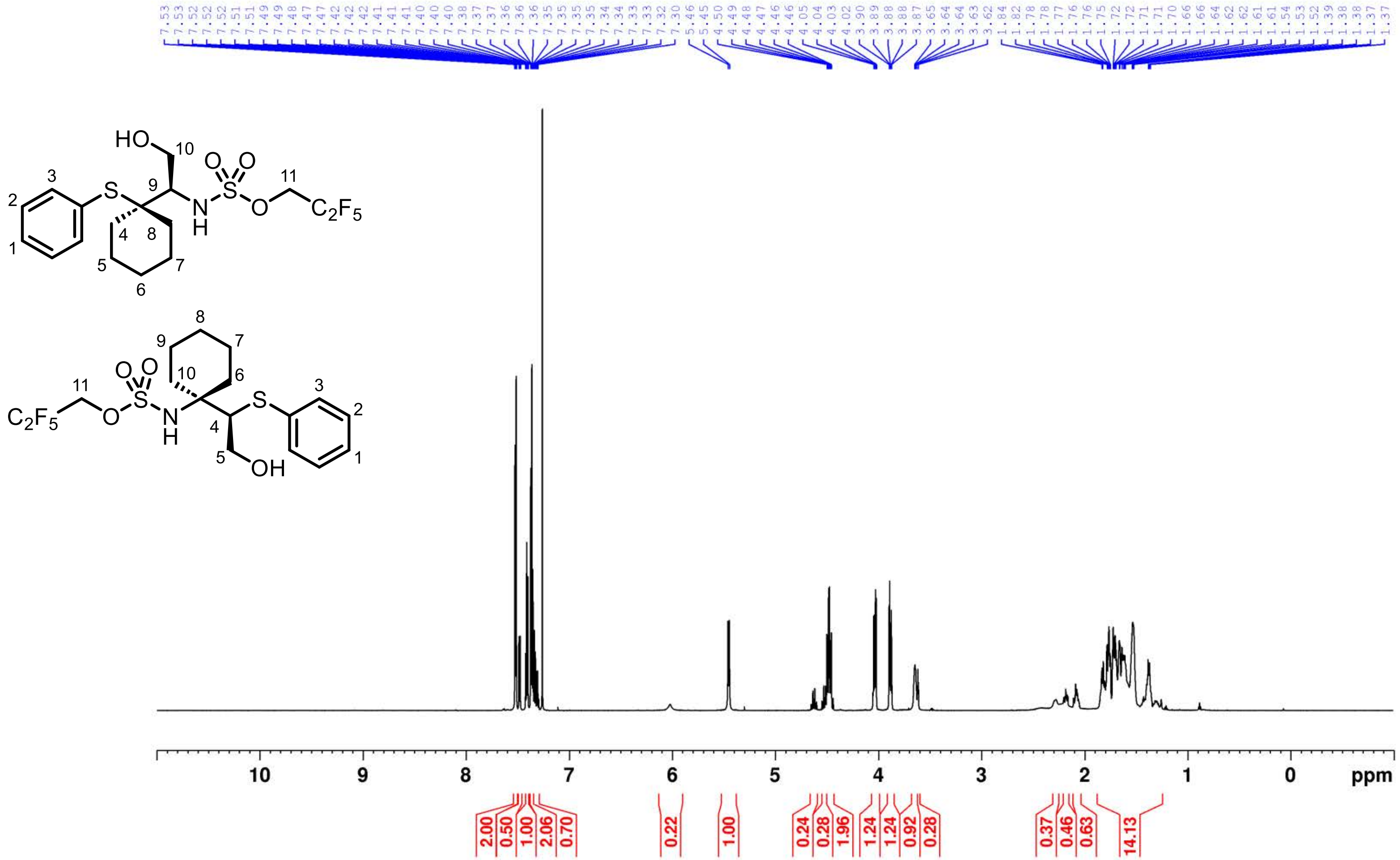

^{13}C NMR (176 MHz, CDCl$_3$) for *2,2,3,3,3-pentafluoropropyl (R)-(2-hydroxy-1-(1-(phenylthio)cyclohexyl)ethyl)sulfamate* and *2,2,3,3,3-pentafluoropropyl (R)-(1-(2-hydroxy-1-(phenylthio)ethyl)cyclohexyl)sulfamate.* **Obtained following derivatisation of 9c**

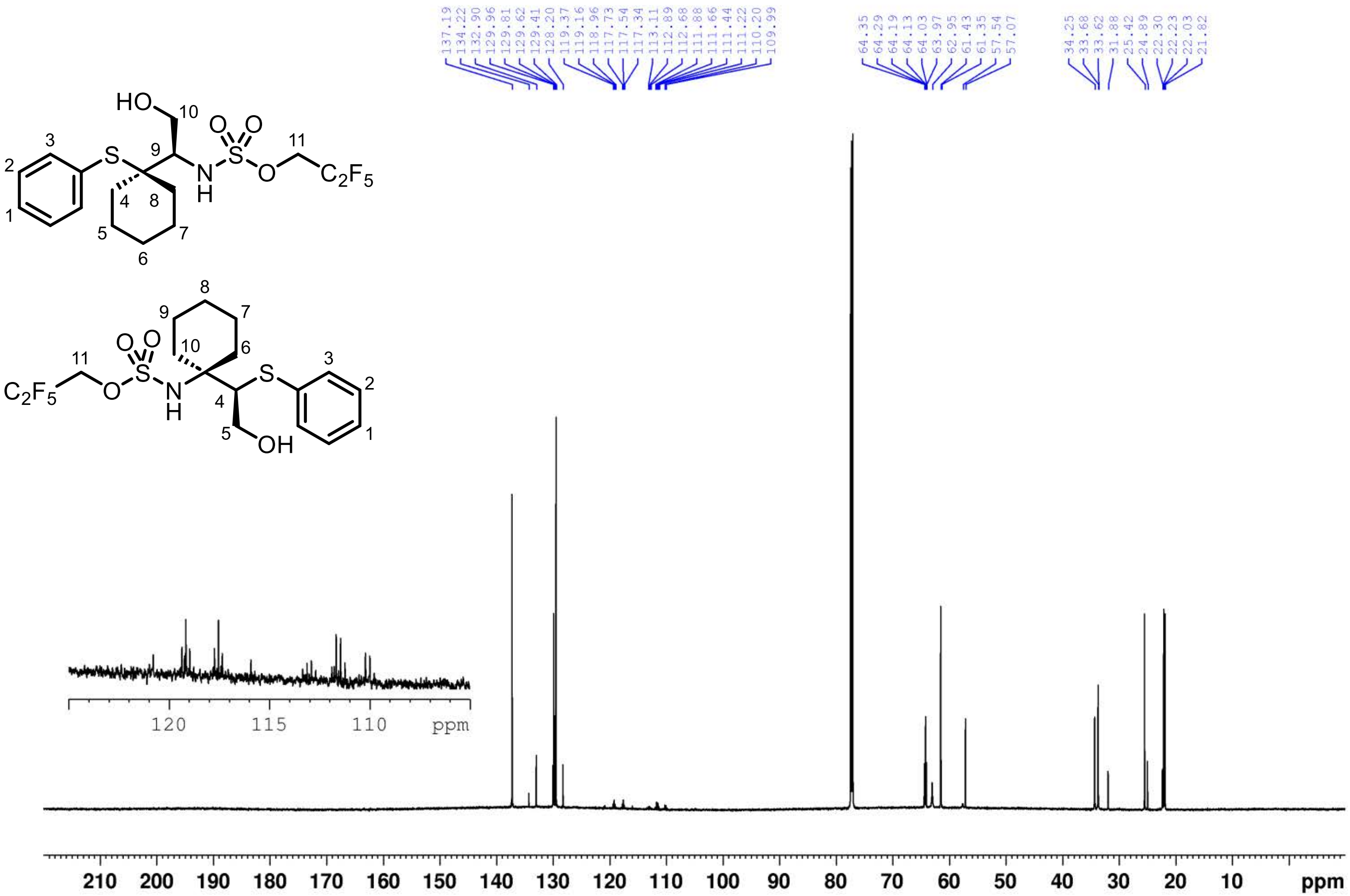

^{19}F NMR (376 MHz, $CDCl_3$) for *2,2,3,3,3-pentafluoropropyl (R)-(2-hydroxy-1-(1-(phenylthio)cyclohexyl)ethyl)sulfamate* and *2,2,3,3,3-pentafluoropropyl (R)-(1-(2-hydroxy-1-(phenylthio)ethyl)cyclohexyl)sulfamate.* **Obtained following derivatisation of 9c**

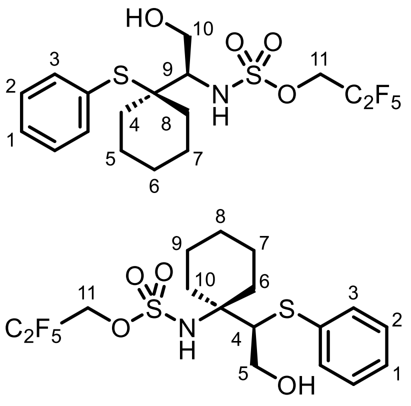

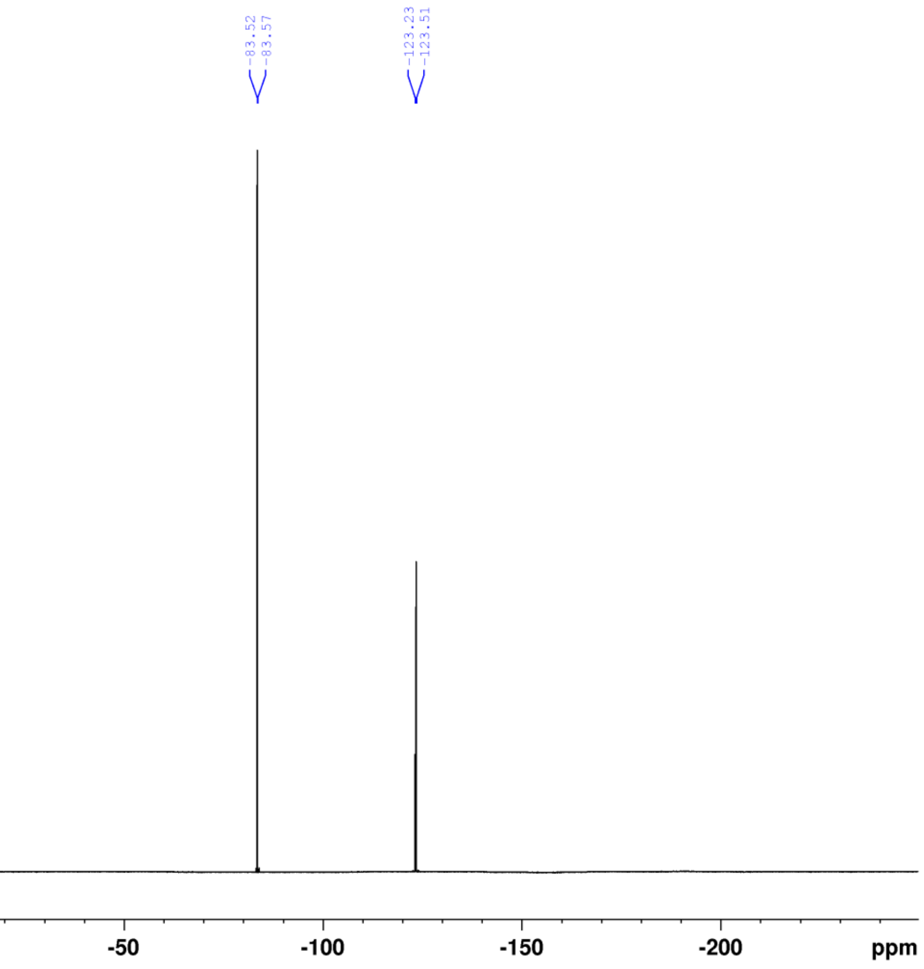

^{1}H NMR (500 MHz, CDCl$_3$) for *2,2,3,3,3-pentafluoropropyl (S)-2-(hydroxymethyl)-1-azaspiro[2.11]tetradecane-1-sulfonate* (**9d**)

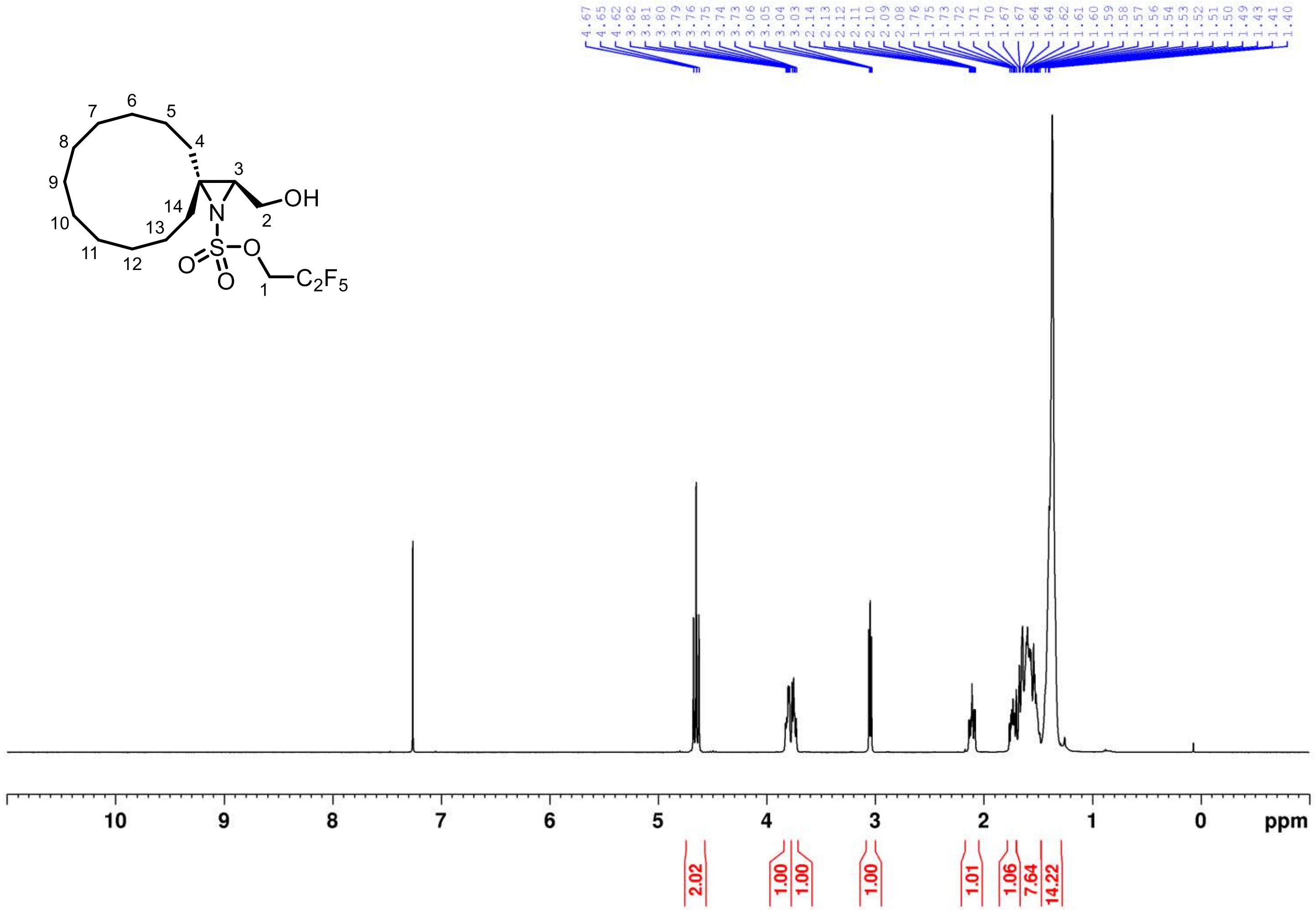

^{13}C NMR (126 MHz, $CDCl_3$) for *2,2,3,3,3-pentafluoropropyl (S)-2-(hydroxymethyl)-1-azaspiro[2.11]tetradecane-1-sulfonate* (**9d**)

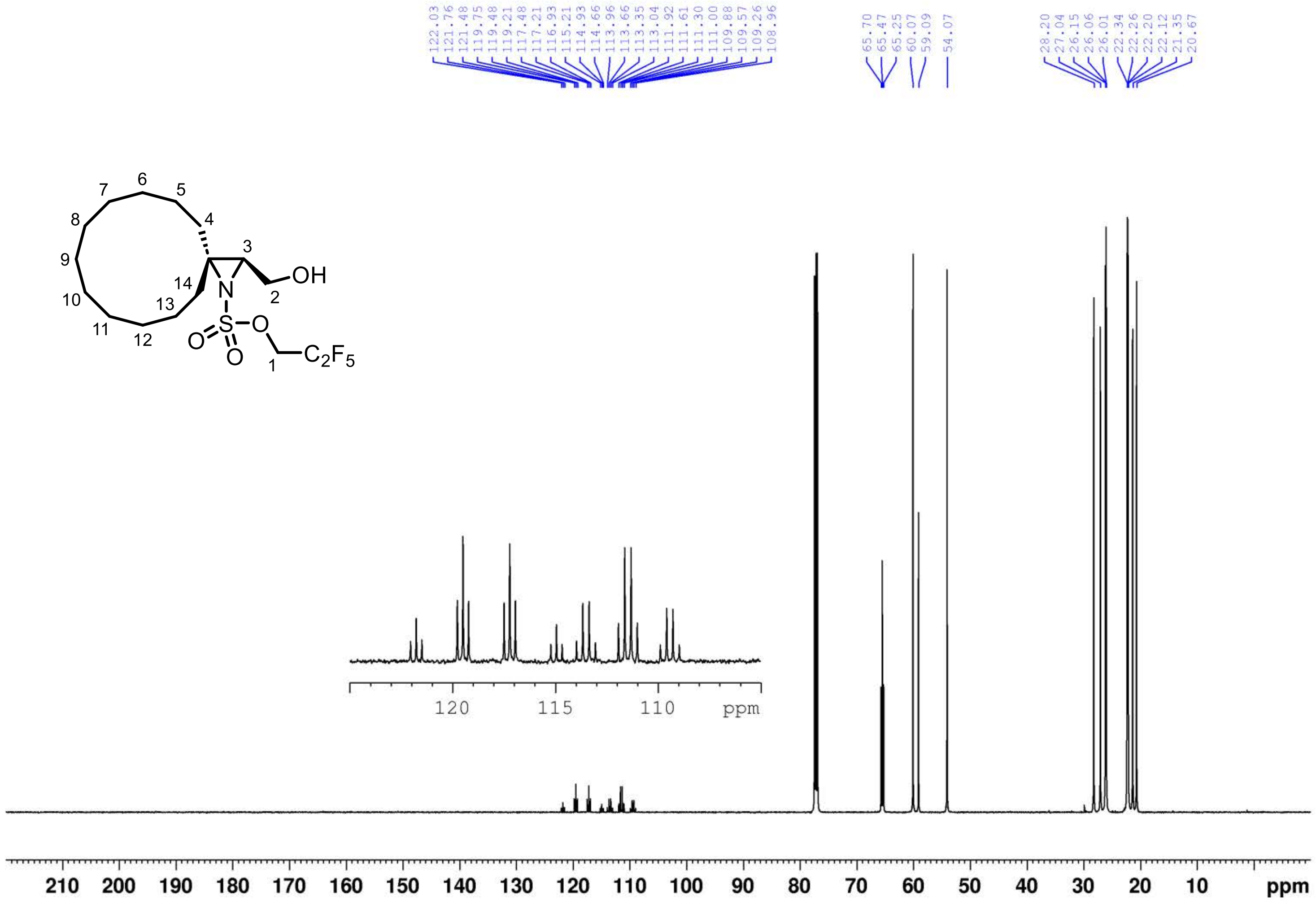

^{19}F NMR (376 MHz, CDCl$_3$) for *2,2,3,3,3-pentafluoropropyl (S)-2-(hydroxymethyl)-1-azaspiro[2.11]tetradecane-1-sulfonate* (**9d**)

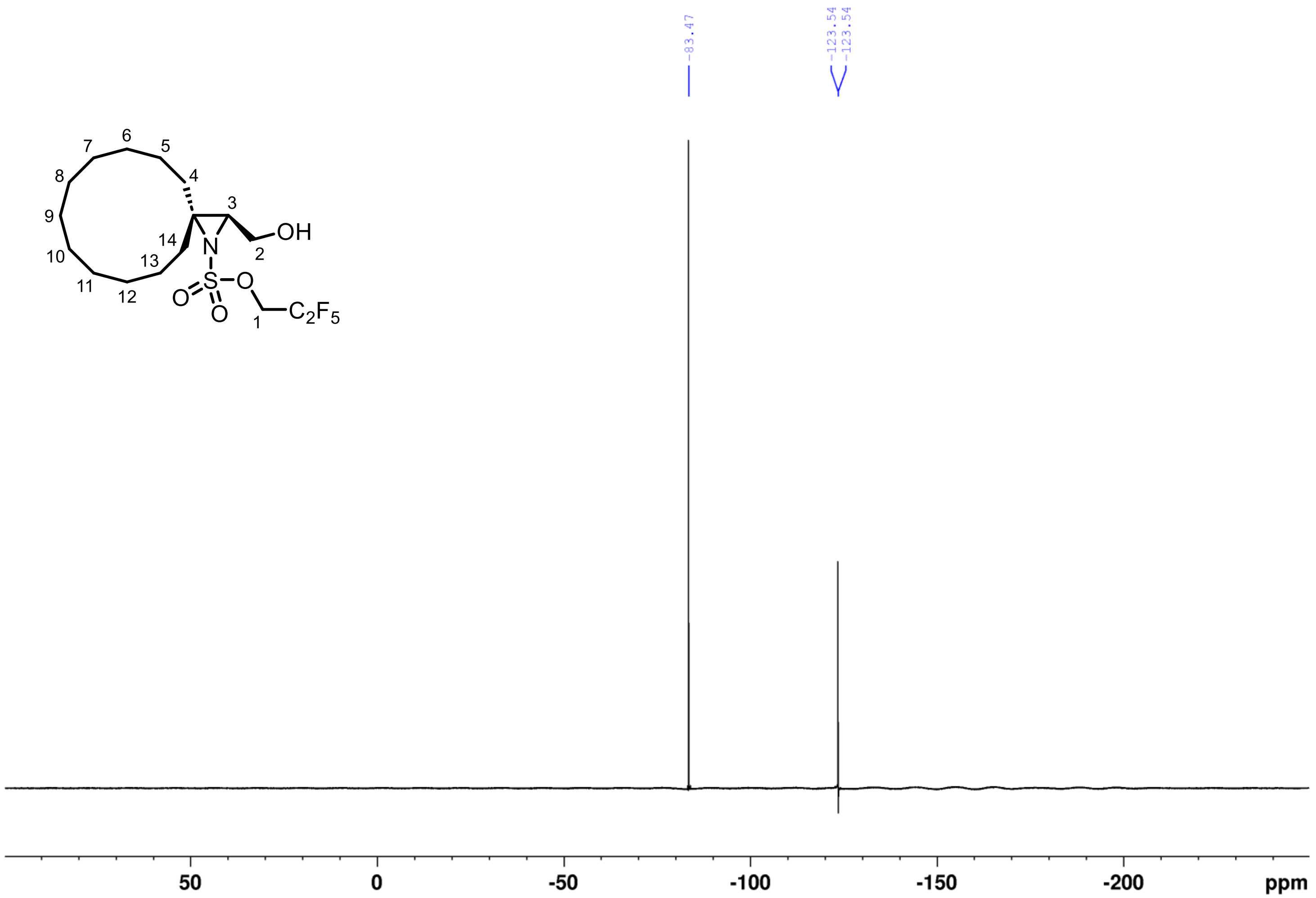

¹H NMR (500 MHz, $CDCl_3$) for *2,2,3,3,3-pentafluoropropyl (R)-(2-hydroxy-1-(1-(phenylthio)cyclododecyl)ethyl)sulfamate* and *2,2,3,3,3-pentafluoropropyl (R)-(1-(2-hydroxy-1-(phenylthio)ethyl)cyclododecyl)sulfamate.* **Obtained following derivatisation of 9d**

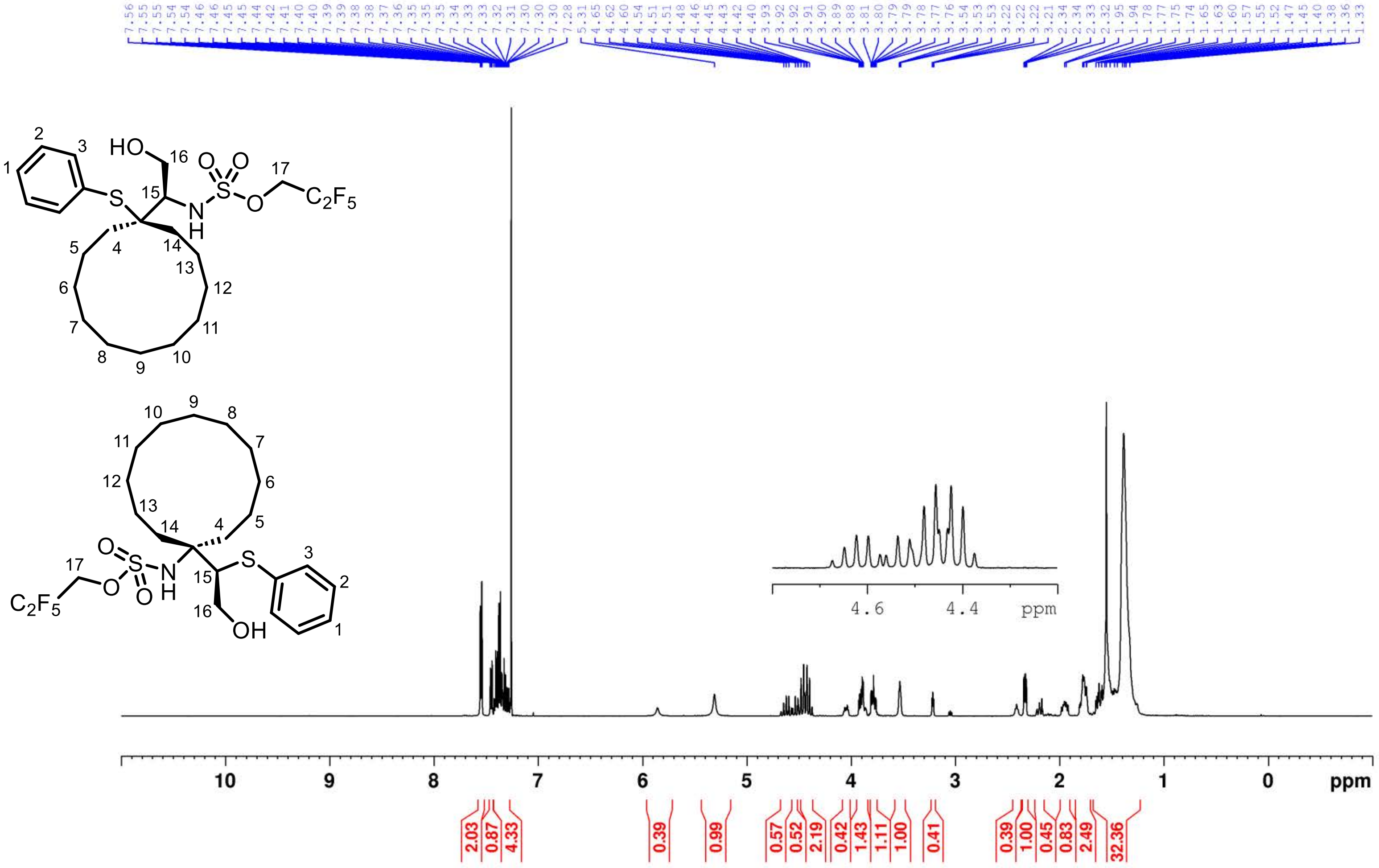

^{13}C NMR (126 MHz, $CDCl_3$) for *2,2,3,3,3-pentafluoropropyl (R)-(2-hydroxy-1-(1-(phenylthio)cyclododecyl)ethyl)sulfamate* and *2,2,3,3,3-pentafluoropropyl (R)-(1-(2-hydroxy-1-(phenylthio)ethyl)cyclododecyl)sulfamate.* **Obtained following derivatisation of 9d**

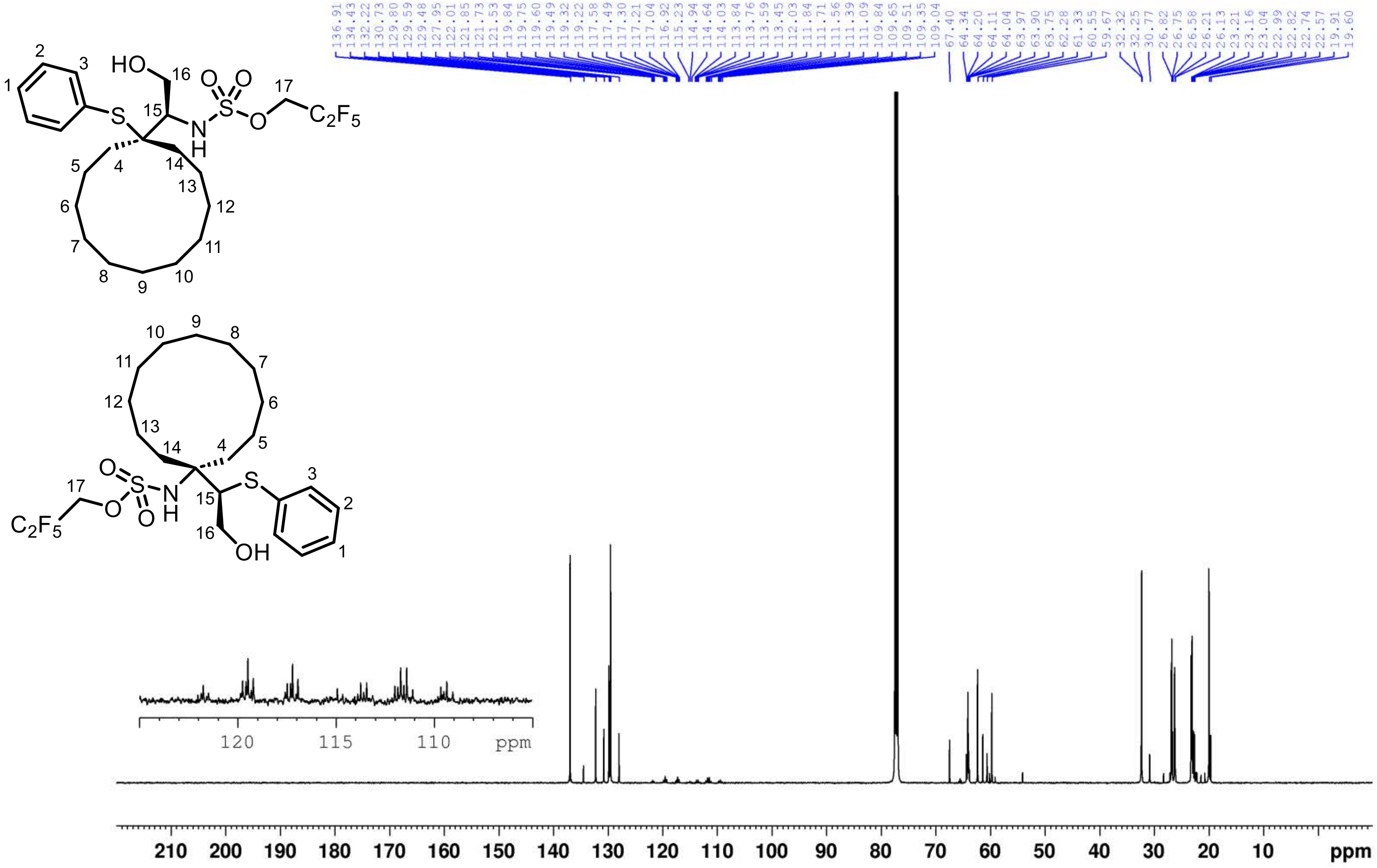

^{19}F NMR (376 MHz, CDCl$_3$) for *2,2,3,3,3-pentafluoropropyl (R)-(2-hydroxy-1-(1-(phenylthio)cyclododecyl)ethyl)sulfamate* and *2,2,3,3,3-pentafluoropropyl (R)-(1-(2-hydroxy-1-(phenylthio)ethyl)cyclododecyl)sulfamate.* **Obtained following derivatisation of 9d**

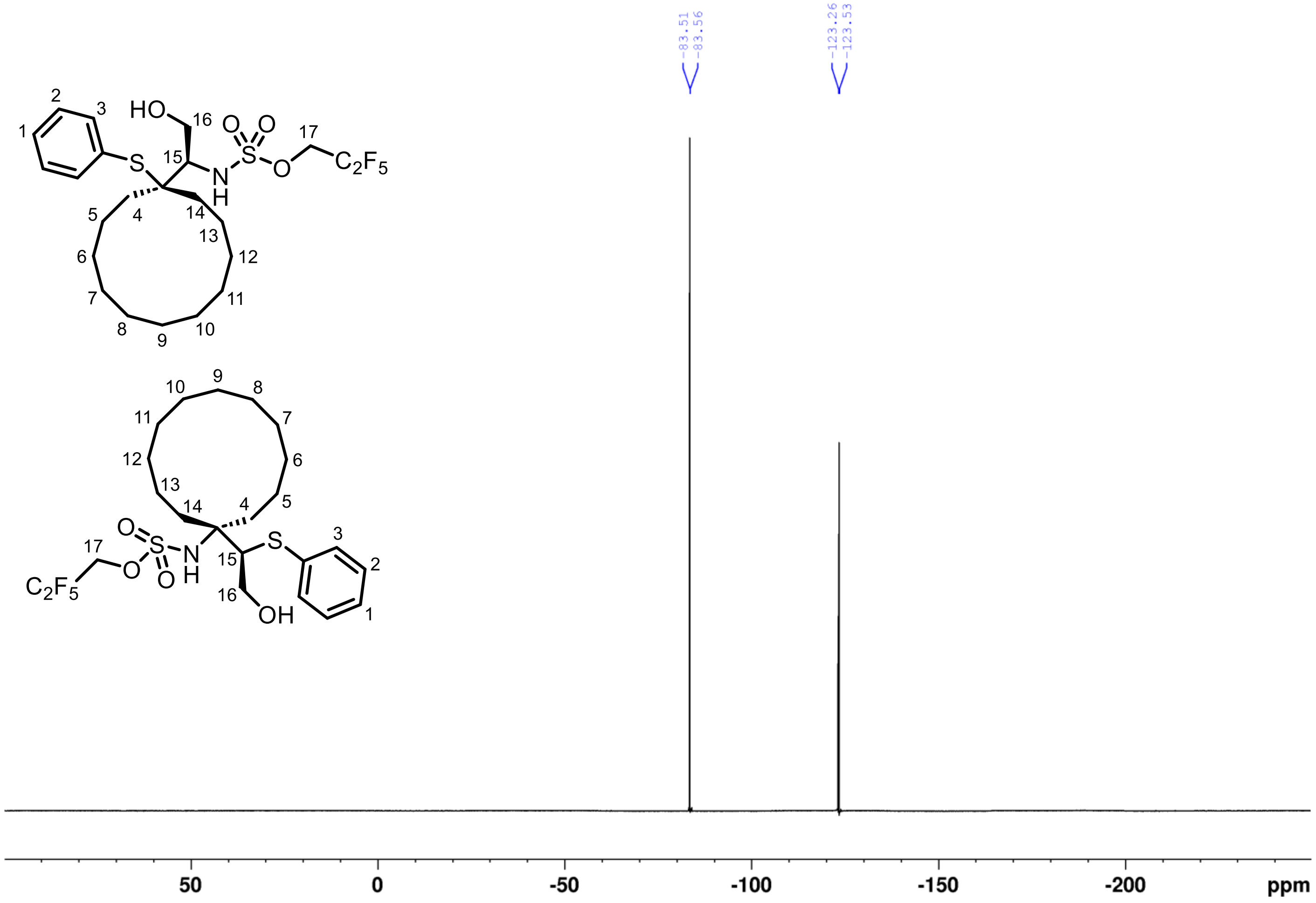

^{1}H NMR (500 MHz, $CDCl_3$) for *2,2,3,3,3-pentafluoropropyl (S)-6,6-difluoro-2-(hydroxymethyl)-1-azaspiro[2.5]octane-1-sulfonate* (**9e**)

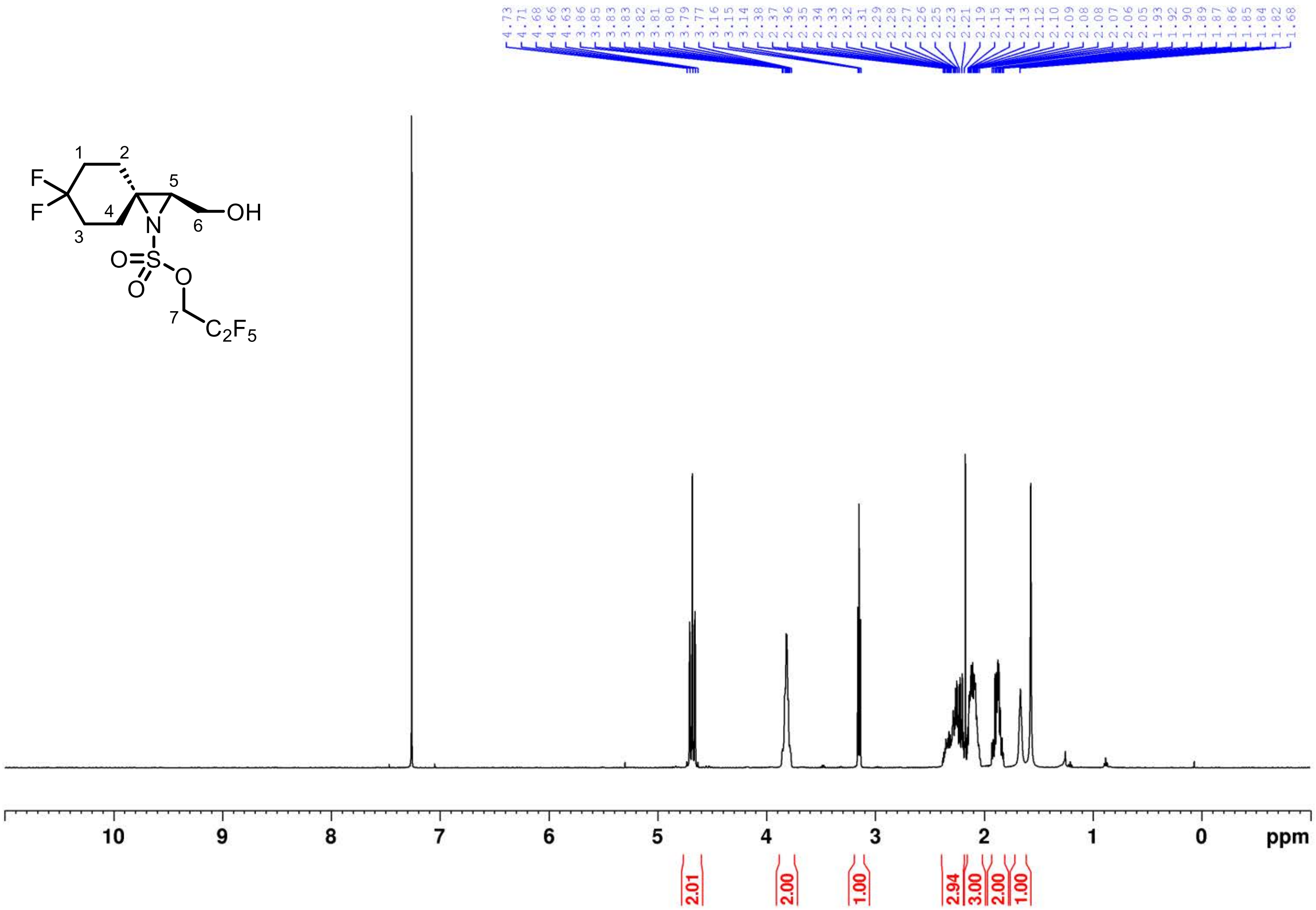

^{13}C NMR (126 MHz, $CDCl_3$) for *2,2,3,3,3-pentafluoropropyl (S)-6,6-difluoro-2-(hydroxymethyl)-1-azaspiro[2.5]octane-1-sulfonate* (**9e**)

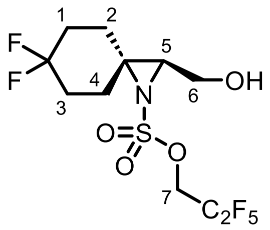

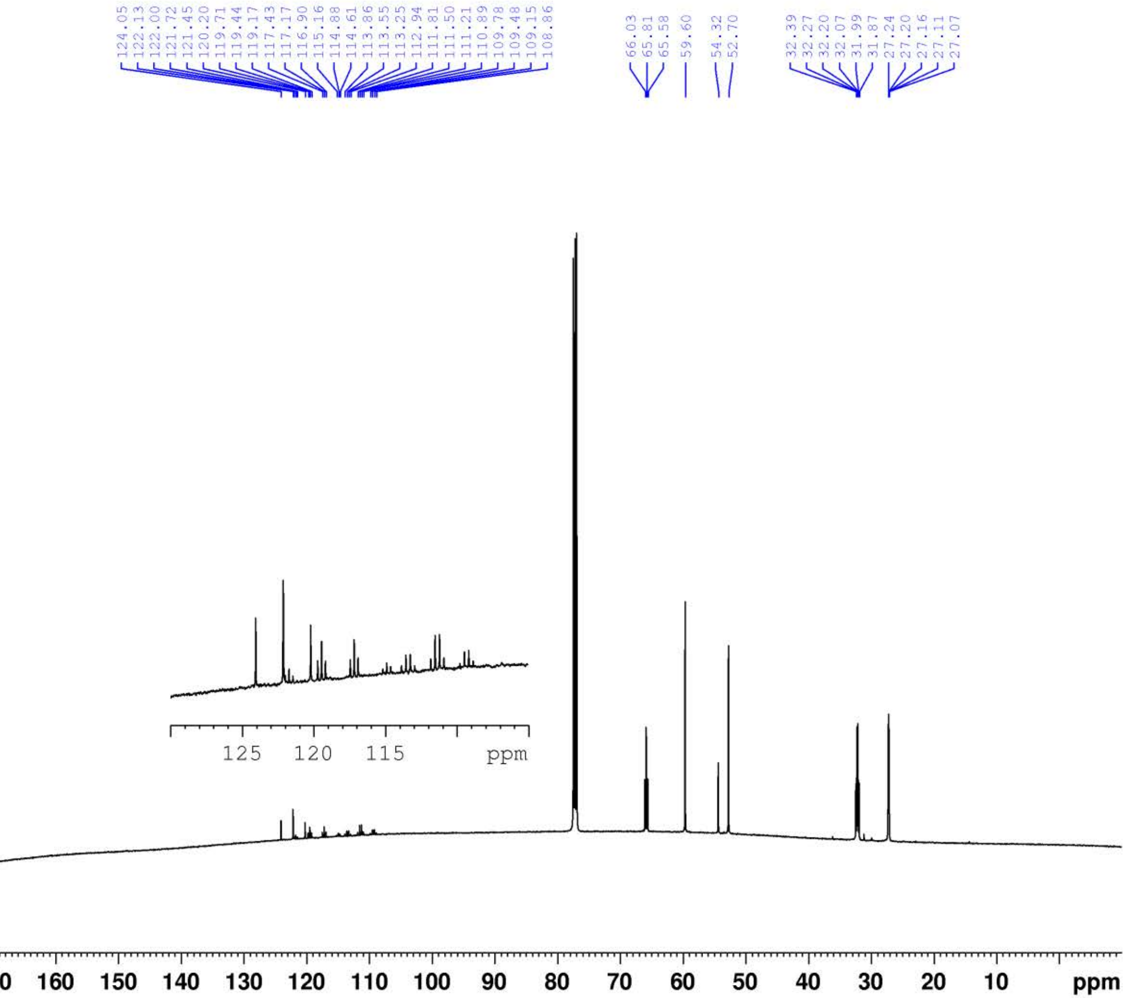

^{19}F NMR (376 MHz, CDCl$_3$) for *2,2,3,3,3-pentafluoropropyl (S)-6,6-difluoro-2-(hydroxymethyl)-1-azaspiro[2.5]octane-1-sulfonate* (**9e**)

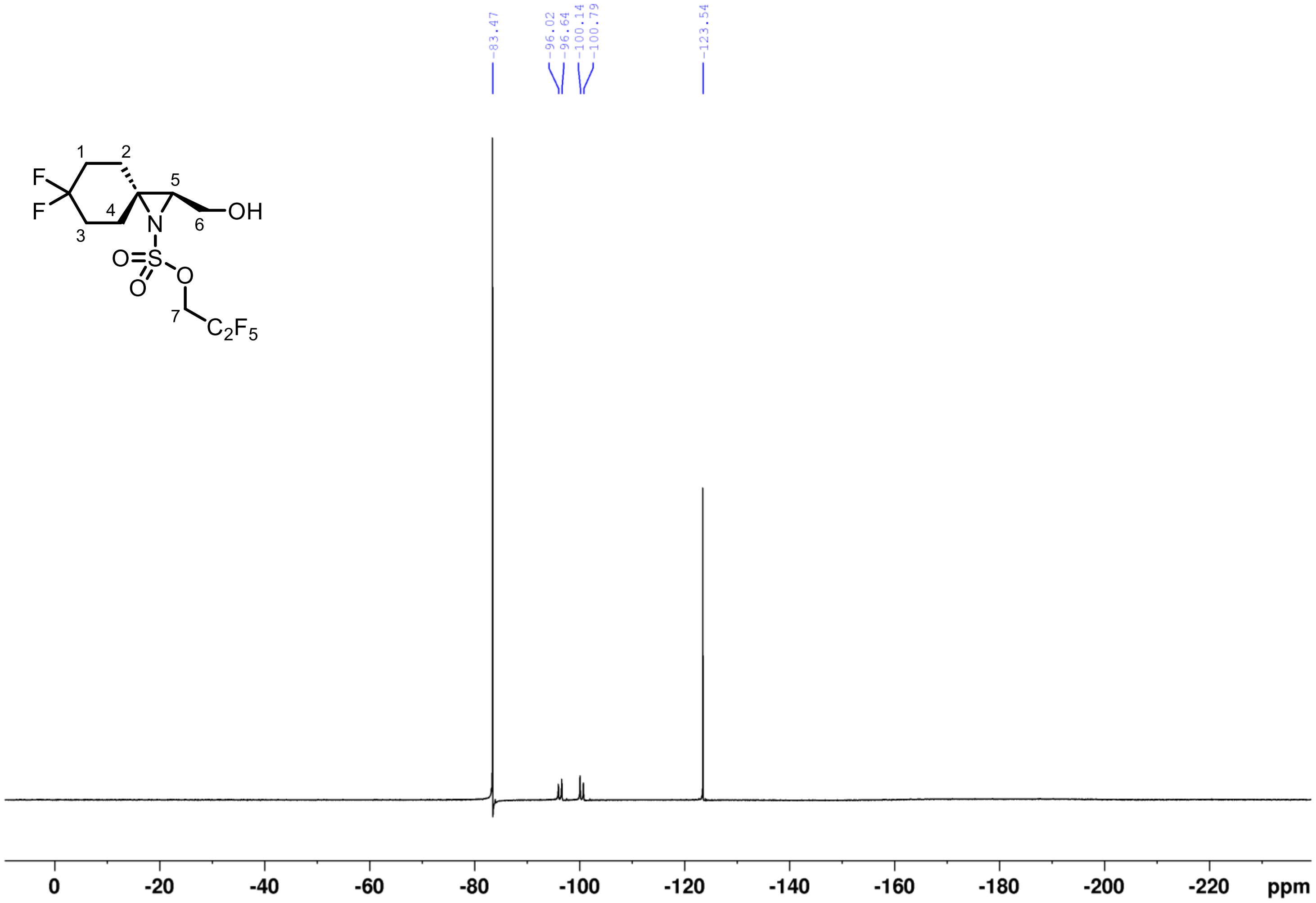

^{1}H NMR (700 MHz, CDCl$_3$) for *2,2,3,3,3-pentafluoropropyl (R)-(4,4-difluoro-1-(2-hydroxy-1-(phenylthio)ethyl)cyclohexyl)sulfamate* and *2,2,3,3,3-pentafluoropropyl (R)-(1-(4,4-difluoro-1-(phenylthio)cyclohexyl)-2-hydroxyethyl)sulfamate.* **Obtained following derivatisation of 9e**

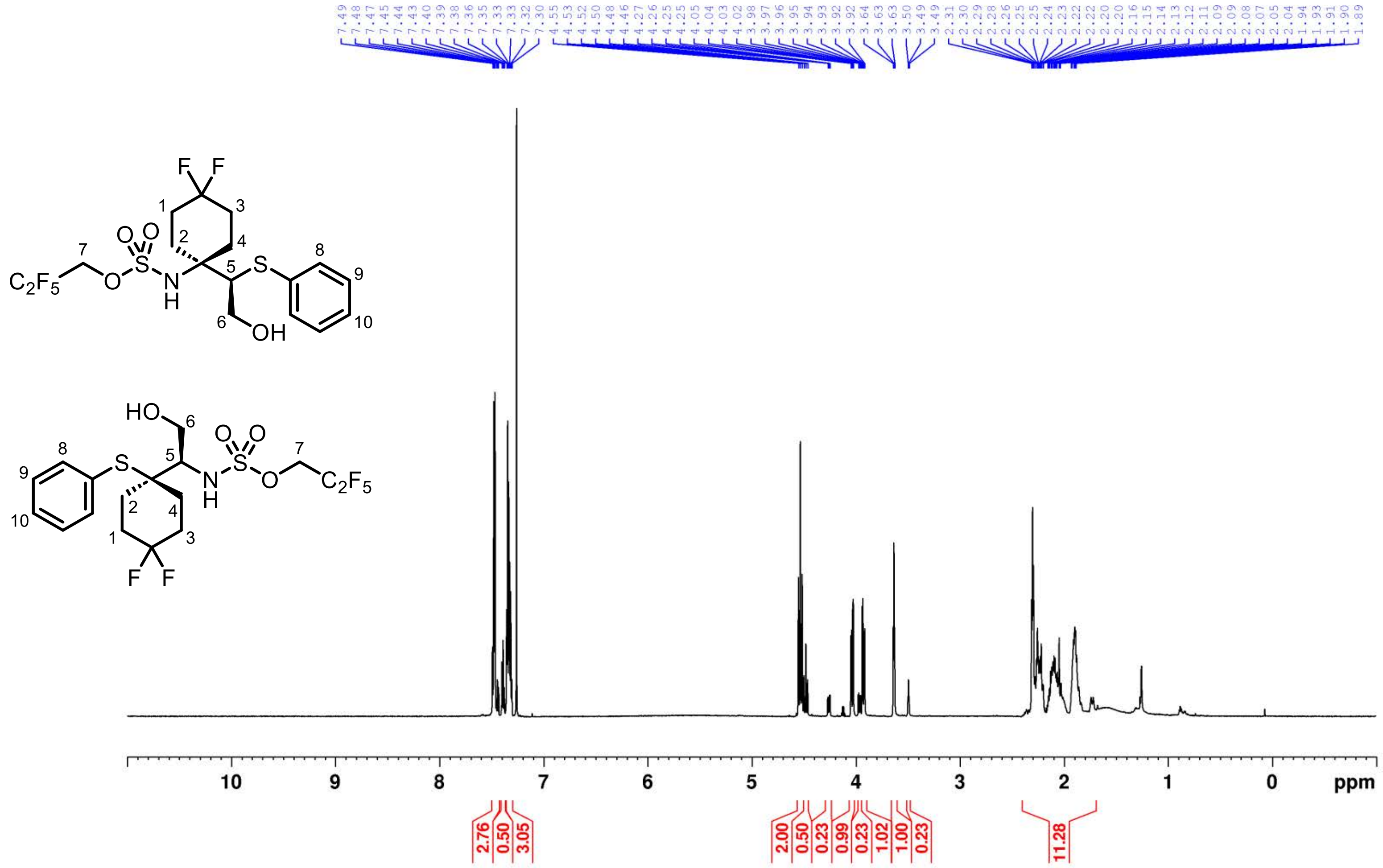

^{13}C NMR (176 MHz, $CDCl_3$) for *2,2,3,3,3-pentafluoropropyl (R)-(4,4-difluoro-1-(2-hydroxy-1-(phenylthio)ethyl)cyclohexyl)sulfamate* and *2,2,3,3,3-pentafluoropropyl (R)-(1-(4,4-difluoro-1-(phenylthio)cyclohexyl)-2-hydroxyethyl)sulfamate.* **Obtained following derivatisation of 9e**

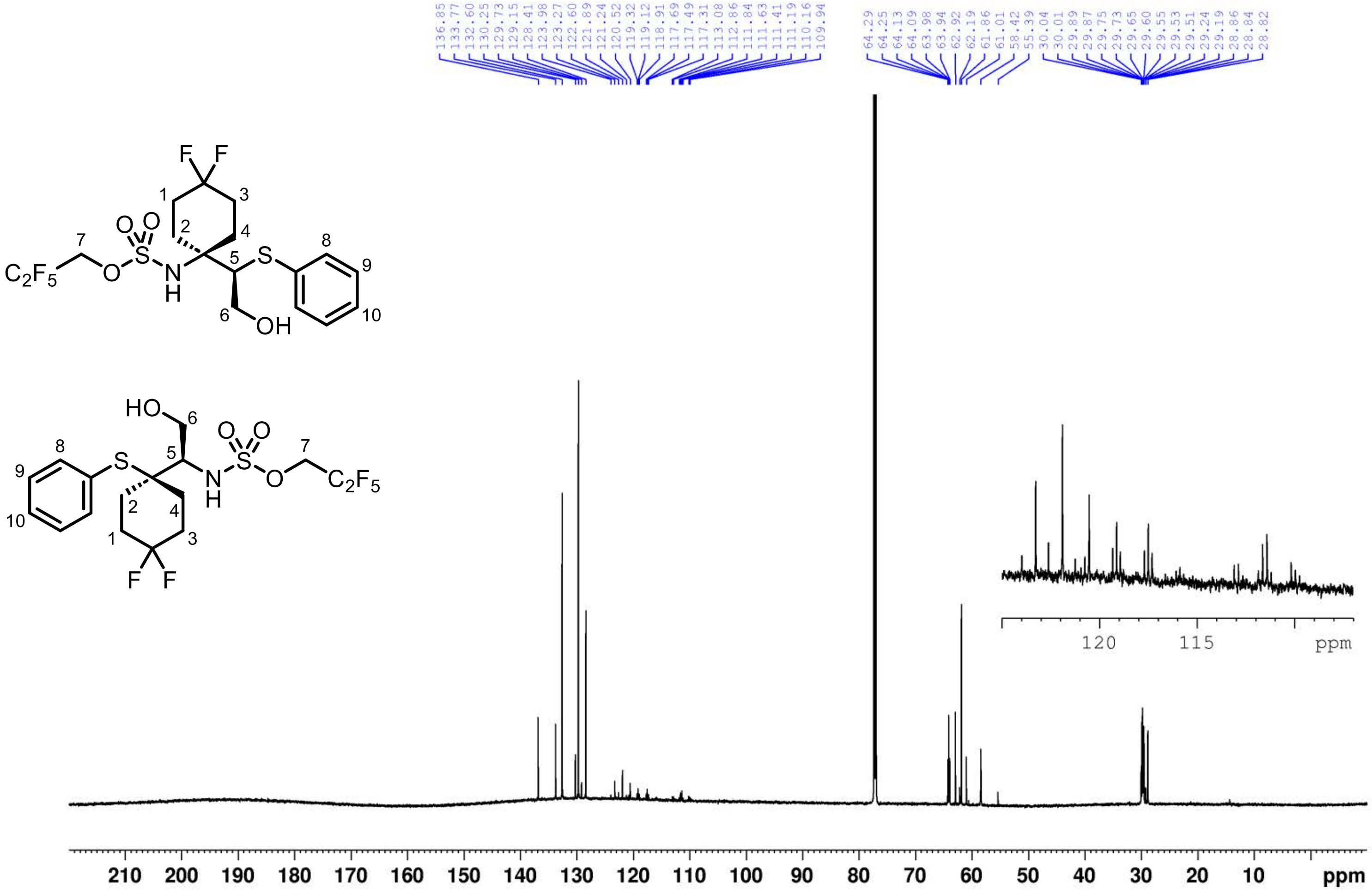

^{19}F NMR (376 MHz, $CDCl_3$) for *2,2,3,3,3-pentafluoropropyl (R)-(4,4-difluoro-1-(2-hydroxy-1-(phenylthio)ethyl)cyclohexyl)sulfamate* and *2,2,3,3,3-pentafluoropropyl (R)-(1-(4,4-difluoro-1-(phenylthio)cyclohexyl)-2-hydroxyethyl)sulfamate.* **Obtained following derivatisation of 9e**

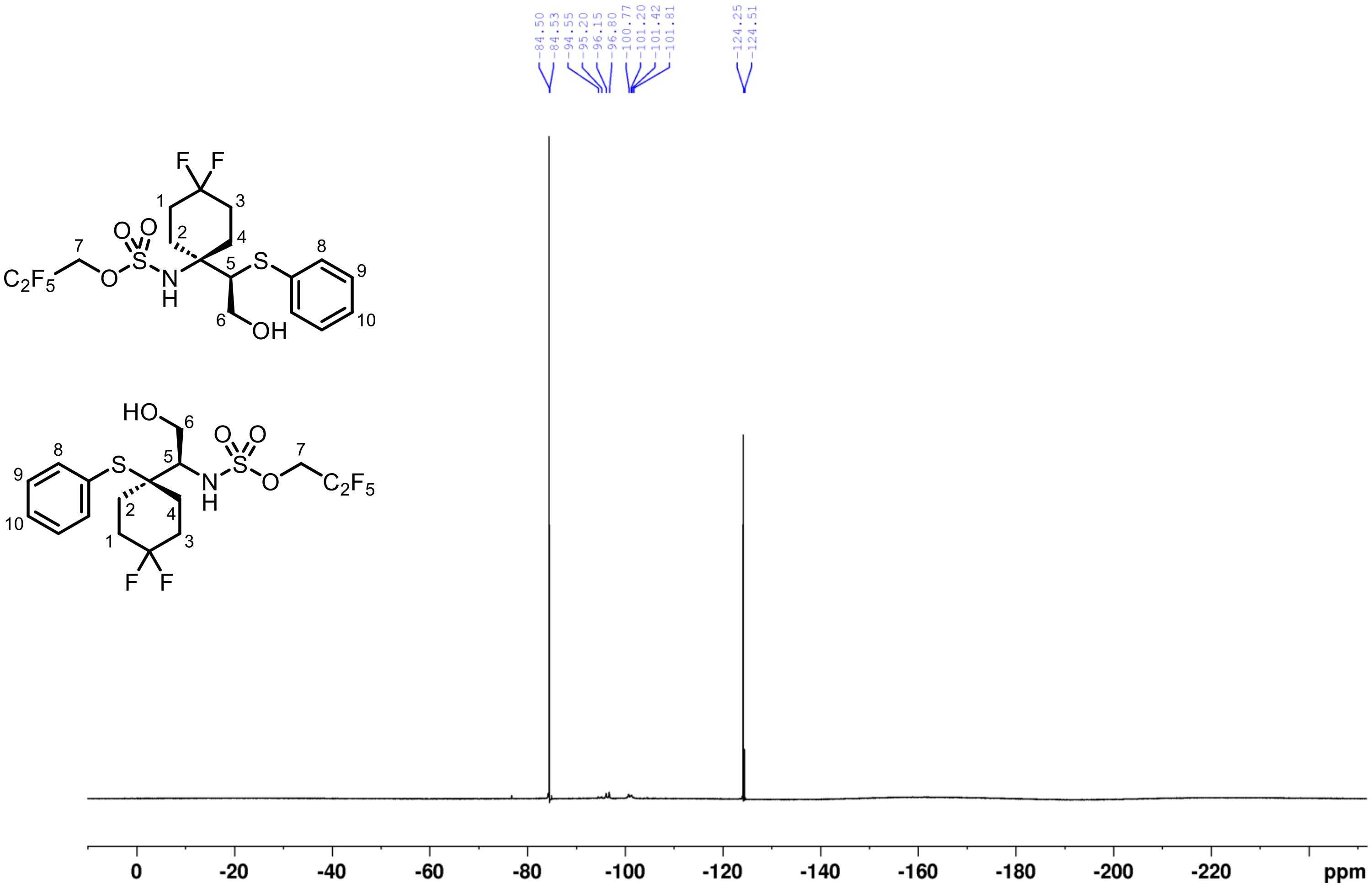

¹H NMR (500 MHz, $CDCl_3$) for *2,2,3,3,3-pentafluoropropyl (S)-2-(hydroxymethyl)-6-oxa-1-azaspiro[2.5]octane-1-sulfonate* (**9f**)

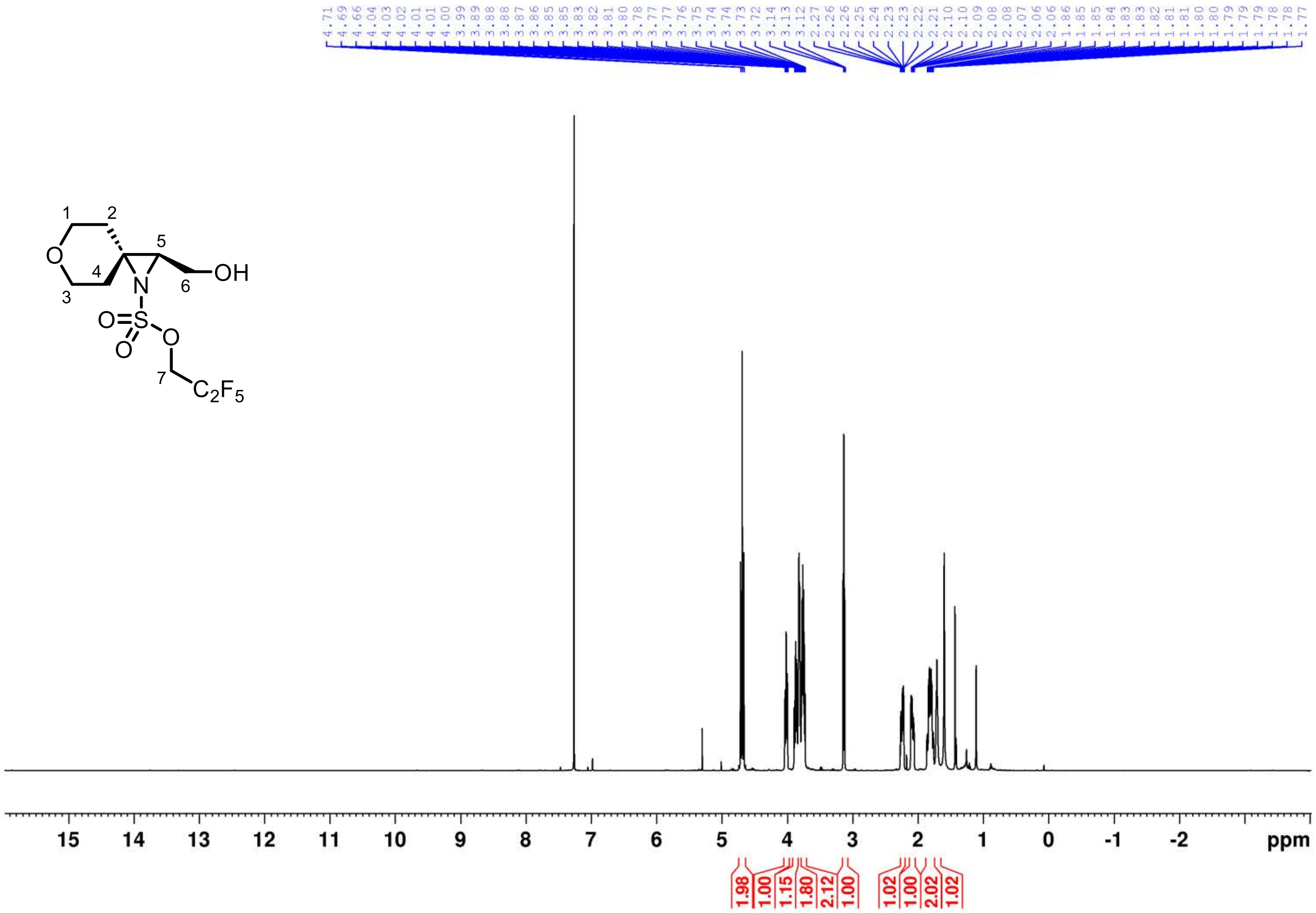

^{13}C NMR (126 MHz, CDCl$_3$) for *2,2,3,3,3-pentafluoropropyl (S)-2-(hydroxymethyl)-6-oxa-1-azaspiro[2.5]octane-1-sulfonate* (**9f**)

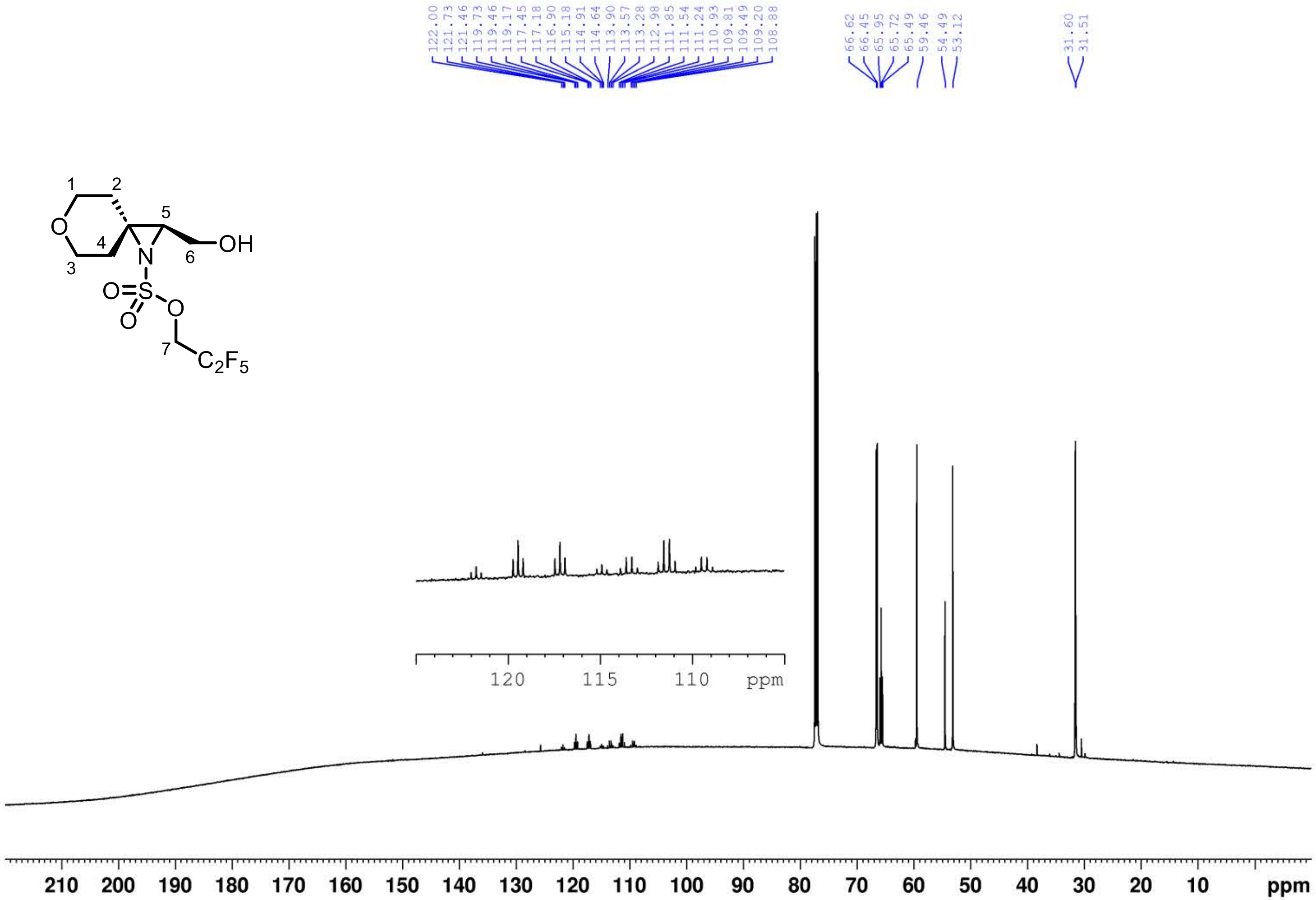

^{19}F NMR (376 MHz, CDCl$_3$) for *2,2,3,3,3-pentafluoropropyl (S)-2-(hydroxymethyl)-6-oxa-1-azaspiro[2.5]octane-1-sulfonate* (**9f**)

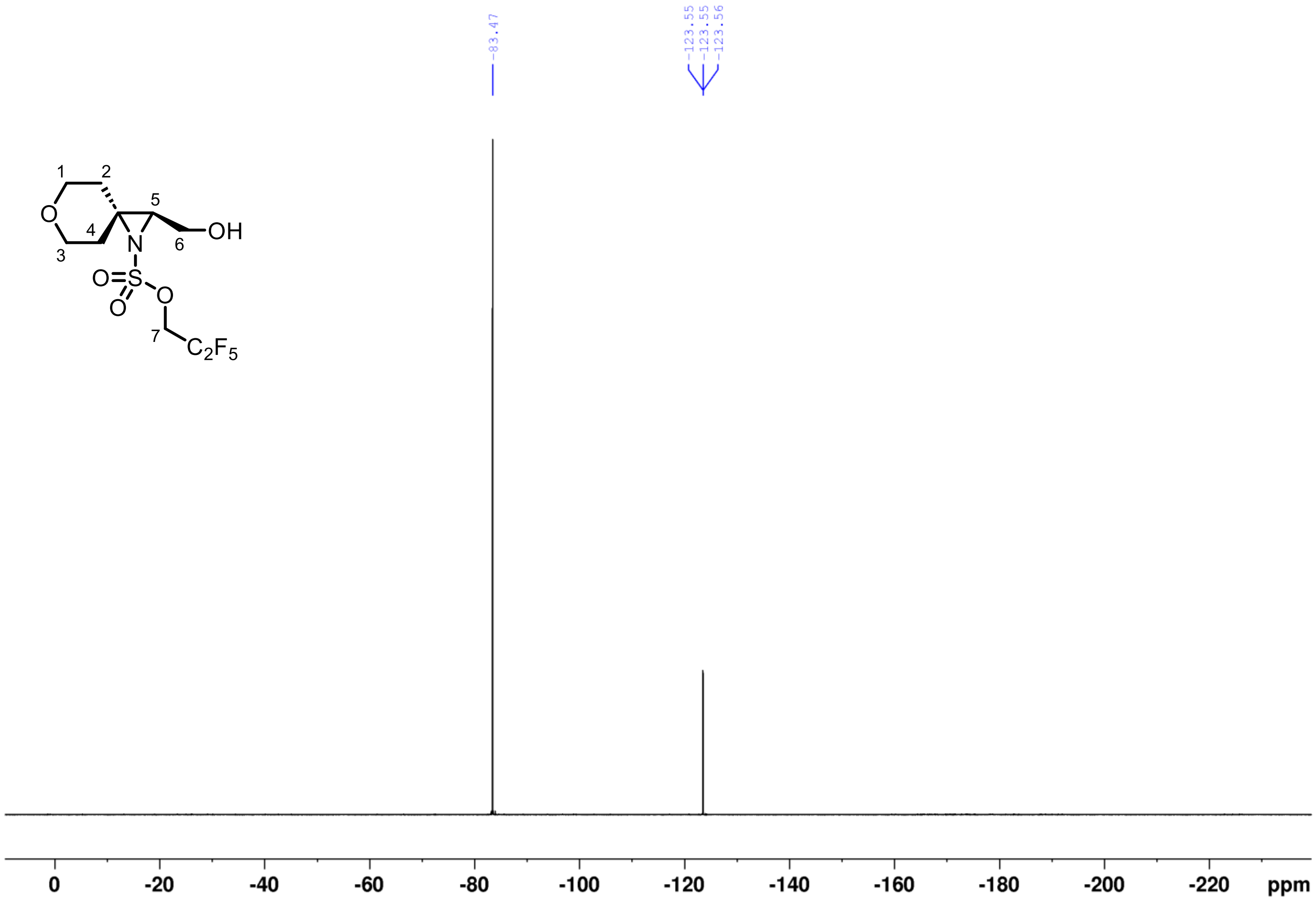

^{1}H NMR (700 MHz, $CDCl_3$) for *2,2,3,3,3-pentafluoropropyl (R)-(2-hydroxy-1-(4-(phenylthio)tetrahydro-2H-pyran-4-yl)ethyl)sulfamate* and *2,2,3,3,3-pentafluoropropyl (R)-(4-(2-hydroxy-1-(phenylthio)ethyl)tetrahydro-2H-pyran-4-yl)sulfamate.* **Obtained following derivatisation of 9f**

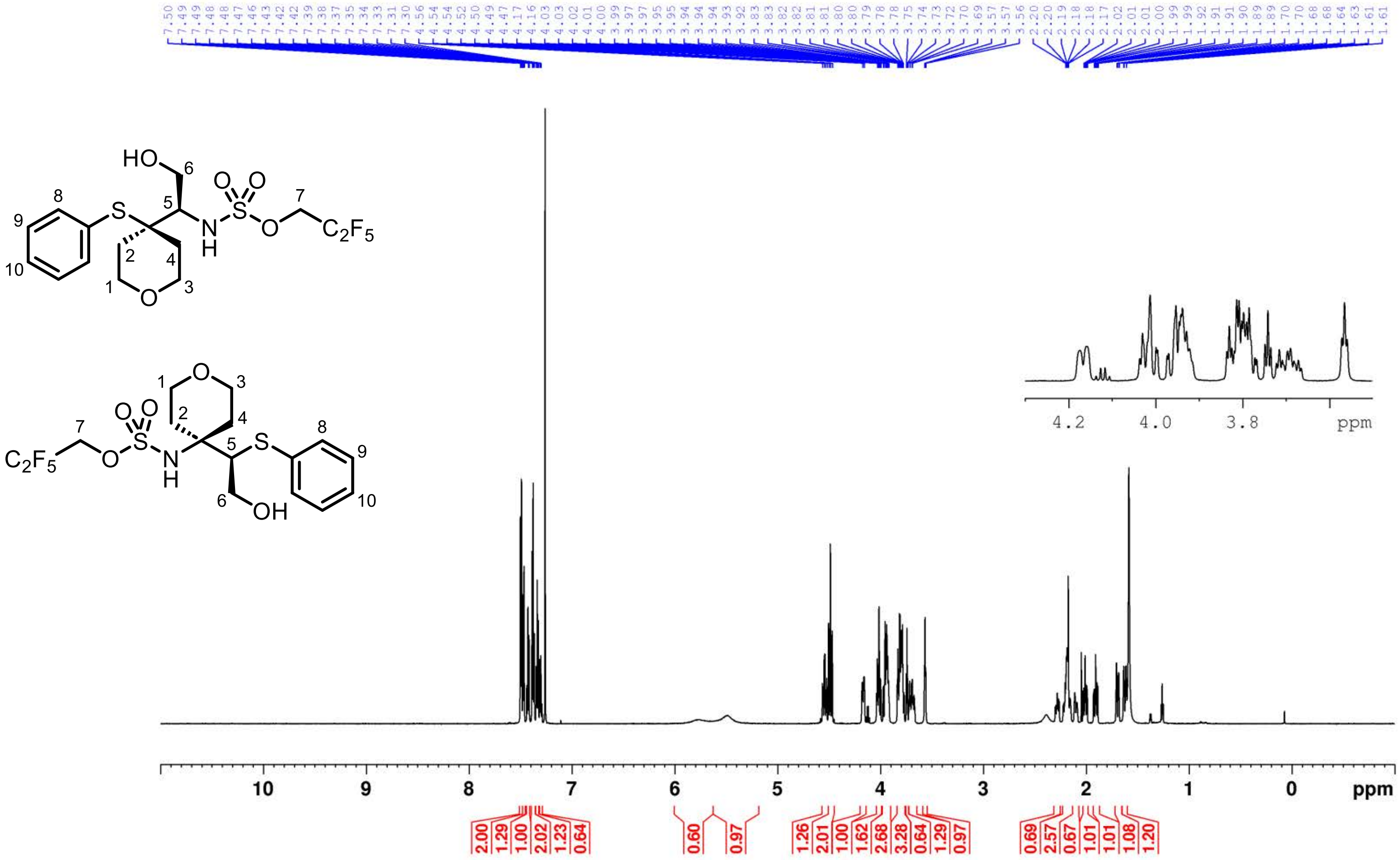

^{13}C NMR (176 MHz, $CDCl_3$) for *2,2,3,3,3-pentafluoropropyl (R)-(2-hydroxy-1-(4-(phenylthio)tetrahydro-2H-pyran-4-yl)ethyl)sulfamate* and *2,2,3,3,3-pentafluoropropyl (R)-(4-(2-hydroxy-1-(phenylthio)ethyl)tetrahydro-2H-pyran-4-yl)sulfamate.* **Obtained following derivatisation of 9f**

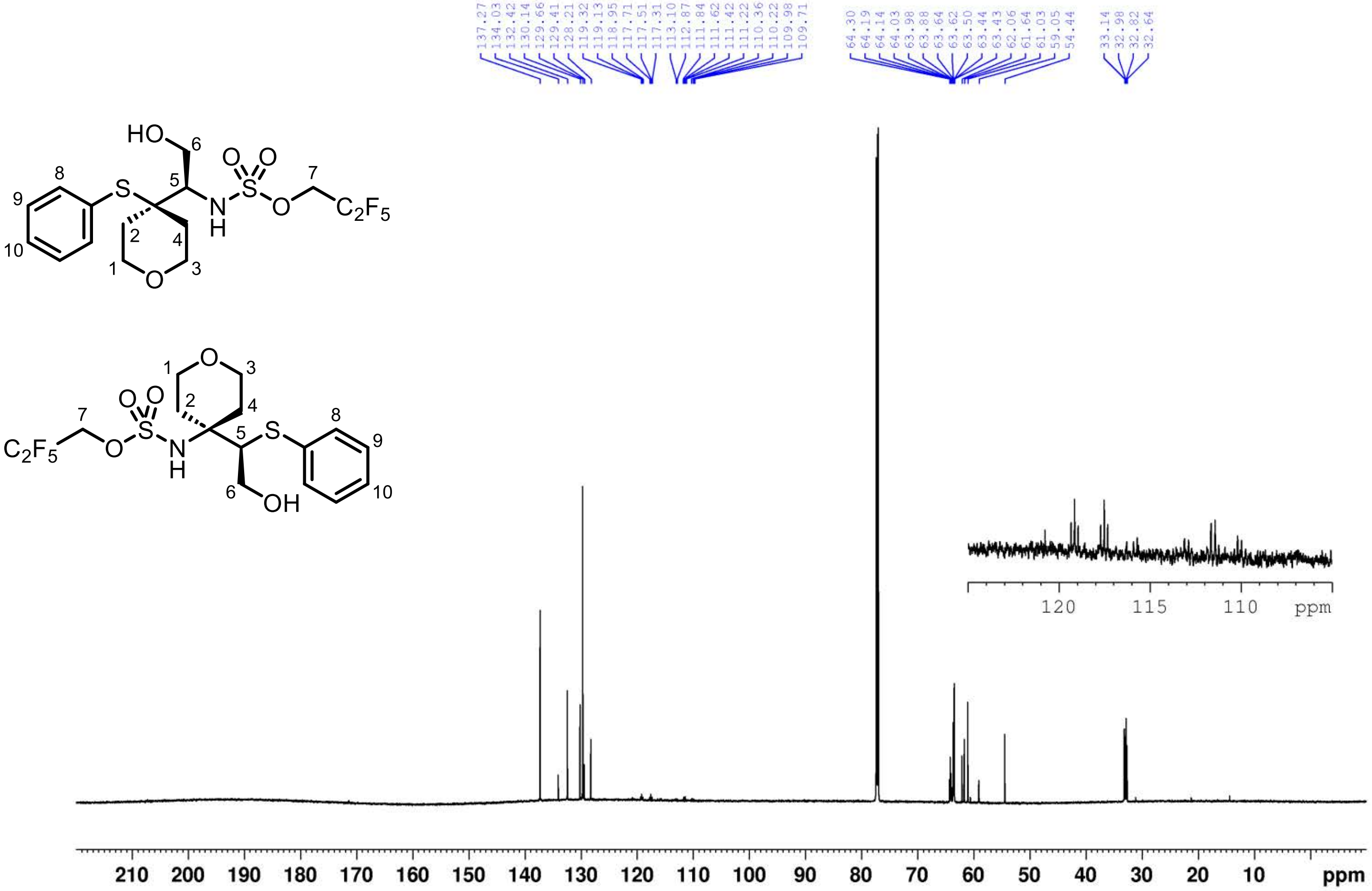

^{19}F NMR (376 MHz, CDCl$_3$) for *2,2,3,3,3-pentafluoropropyl (R)-(2-hydroxy-1-(4-(phenylthio)tetrahydro-2H-pyran-4-yl)ethyl)sulfamate* and *2,2,3,3,3-pentafluoropropyl (R)-(4-(2-hydroxy-1-(phenylthio)ethyl)tetrahydro-2H-pyran-4-yl)sulfamate.* **Obtained following derivatisation of 9f**

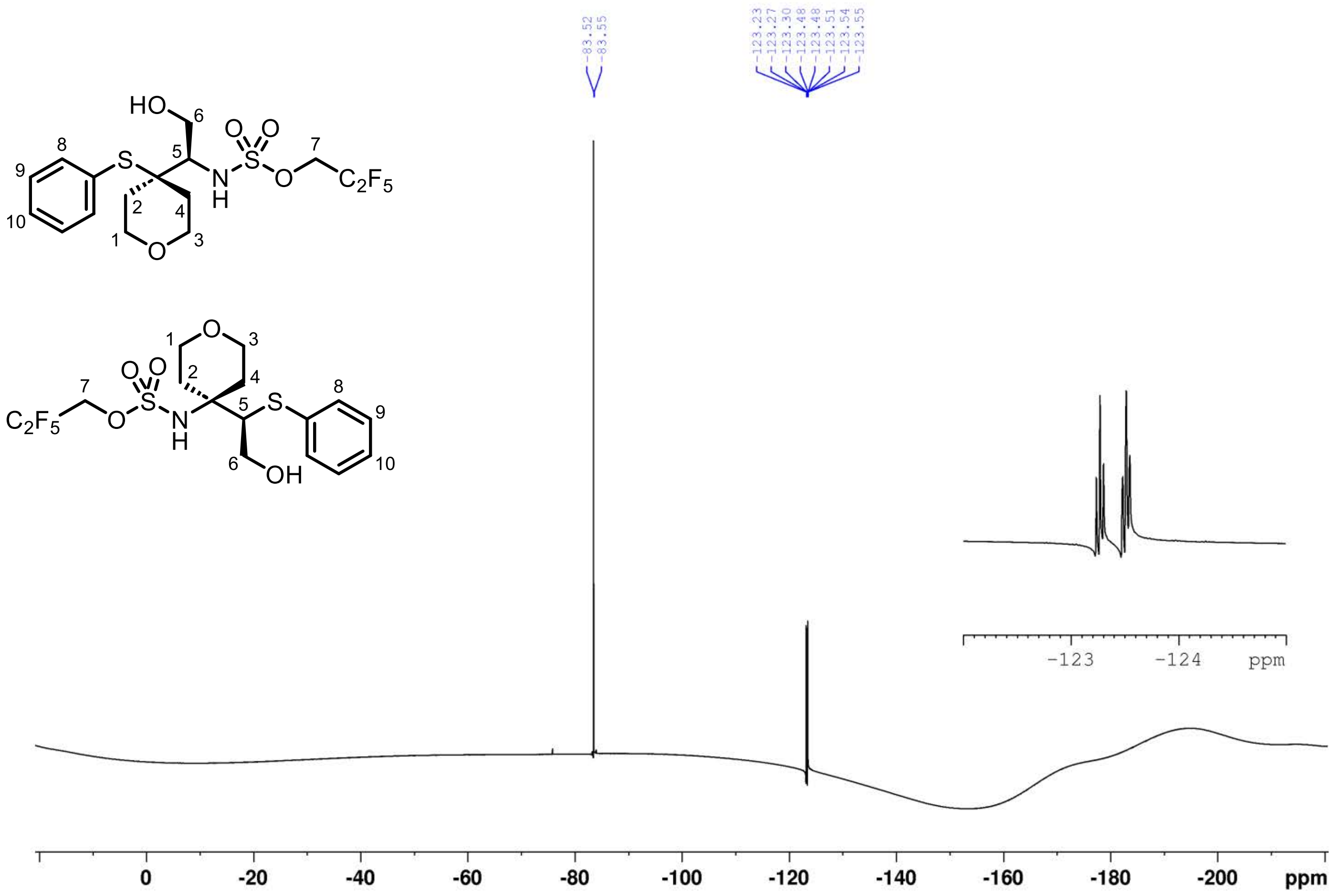

^{1}H NMR (500 MHz, CDCl$_3$) for *2,2,3,3,3-pentafluoropropyl (S)-2-(hydroxymethyl)-7,10-dioxa-1-azadispiro[2.2.4^{6}.2^{3}]dodecane-1-sulfonate* (**9g**)

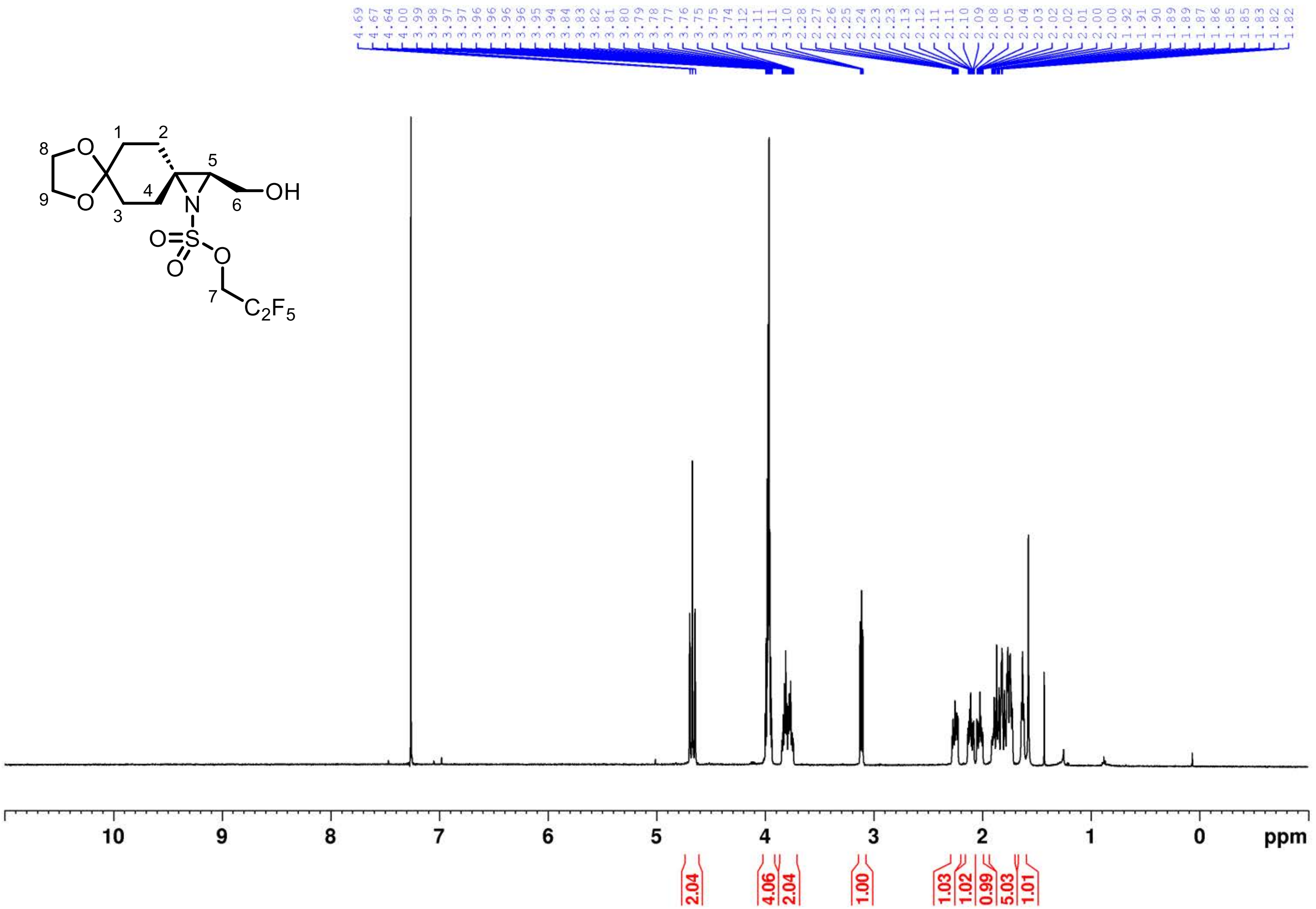

^{13}C NMR (176 MHz, CDCl$_3$) for *2,2,3,3,3-pentafluoropropyl (S)-2-(hydroxymethyl)-7,10-dioxa-1-azadispiro[2.2.4^6.2^3]dodecane-1-sulfonate* (**9g**)

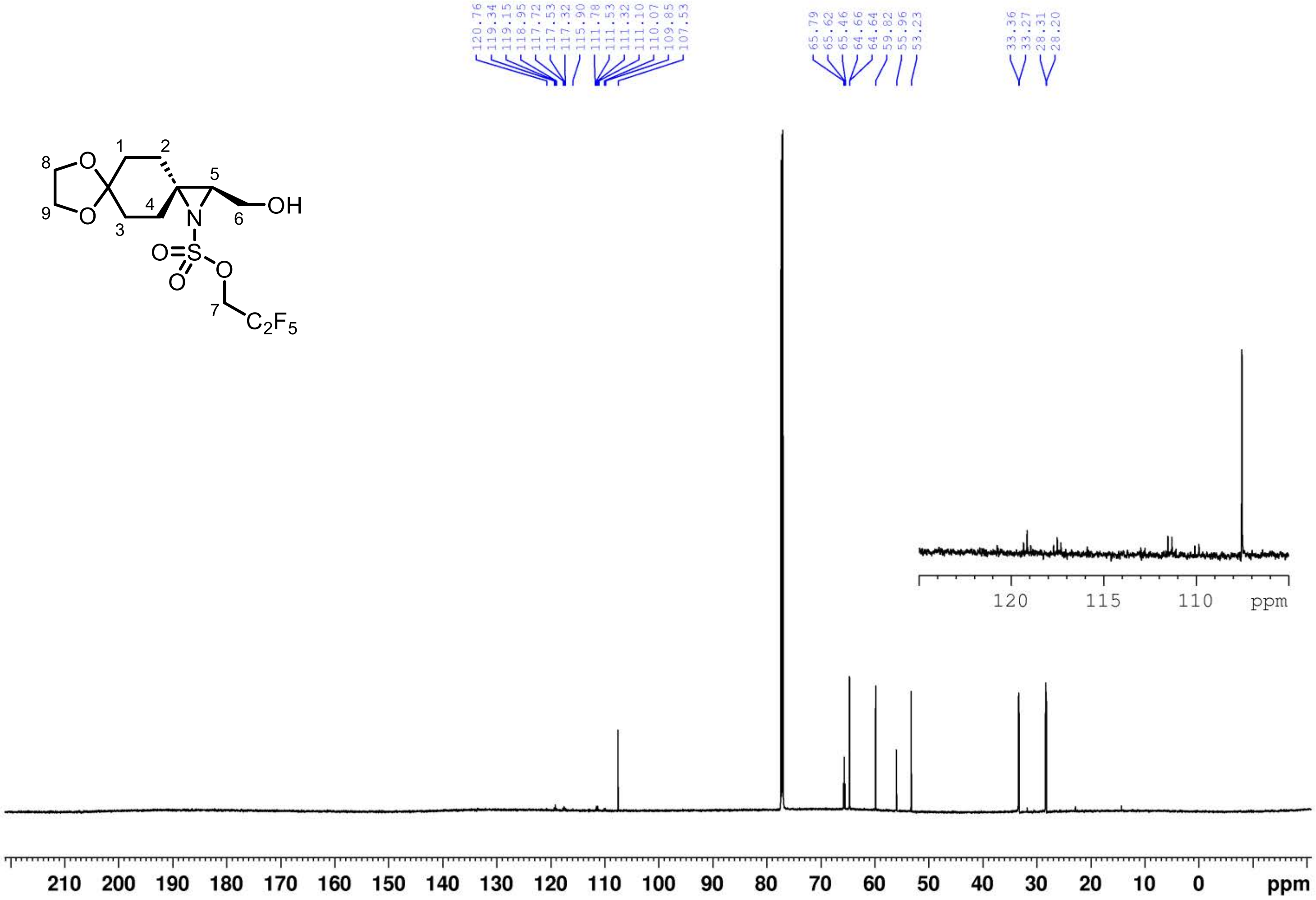

^{19}F NMR (376 MHz, CDCl$_3$) for *2,2,3,3,3-pentafluoropropyl (S)-2-(hydroxymethyl)-7,10-dioxa-1-azadispiro[2.2.4^6.2^3]dodecane-1-sulfonate* (**9g**)

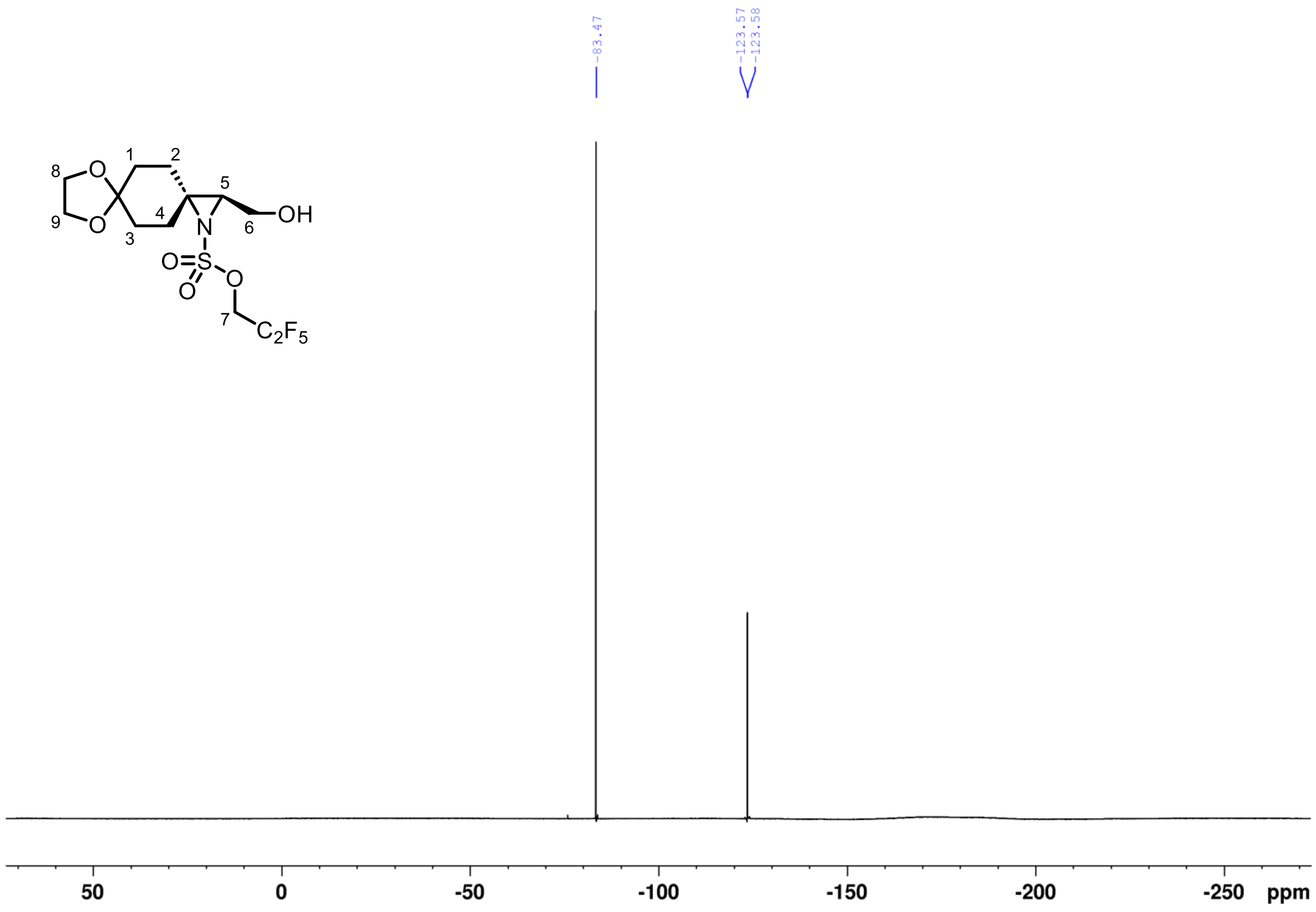

^{1}H NMR (700 MHz, $CDCl_3$) for *2,2,3,3,3-pentafluoropropyl (R)-(2-hydroxy-1-(8-(phenylthio)-1,4-dioxaspiro[4.5]decan-8-yl)ethyl)sulfamate* and *2,2,3,3,3-pentafluoropropyl (R)-(8-(2-hydroxy-1-(phenylthio)ethyl)-1,4-dioxaspiro[4.5]decan-8-yl)sulfamate.* **Obtained following derivatisation of 9g**

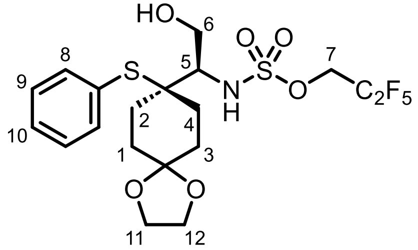

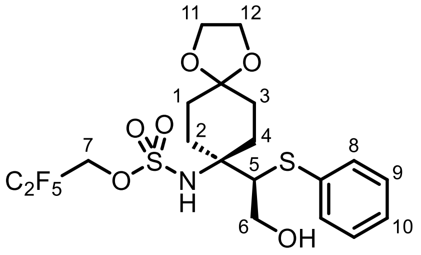

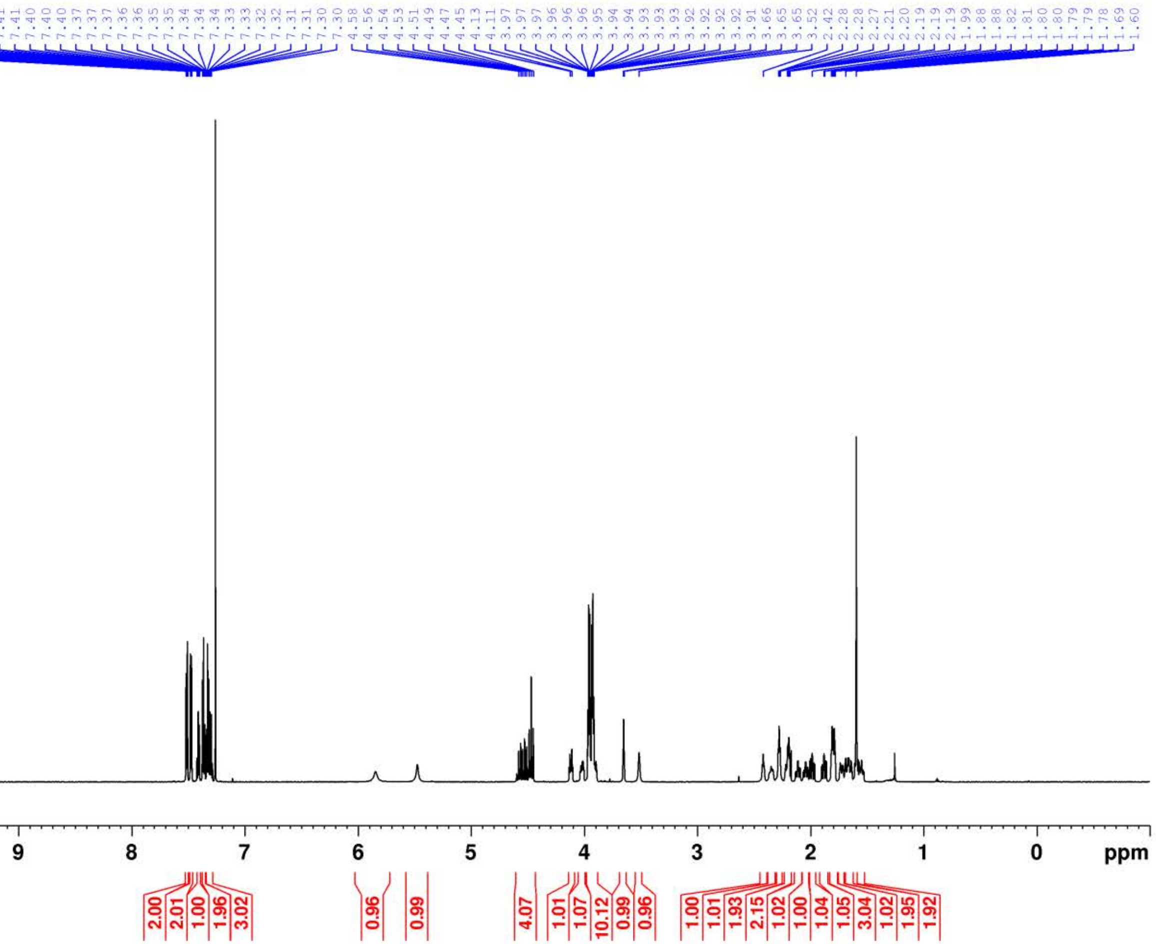

^{13}C NMR (176 MHz, CDCl$_3$) for *2,2,3,3,3-pentafluoropropyl (R)-(2-hydroxy-1-(8-(phenylthio)-1,4-dioxaspiro[4.5]decan-8-yl)ethyl)sulfamate* and *2,2,3,3,3-pentafluoropropyl (R)-(8-(2-hydroxy-1-(phenylthio)ethyl)-1,4-dioxaspiro[4.5]decan-8-yl)sulfamate.* **Obtained following derivatisation of 9g**

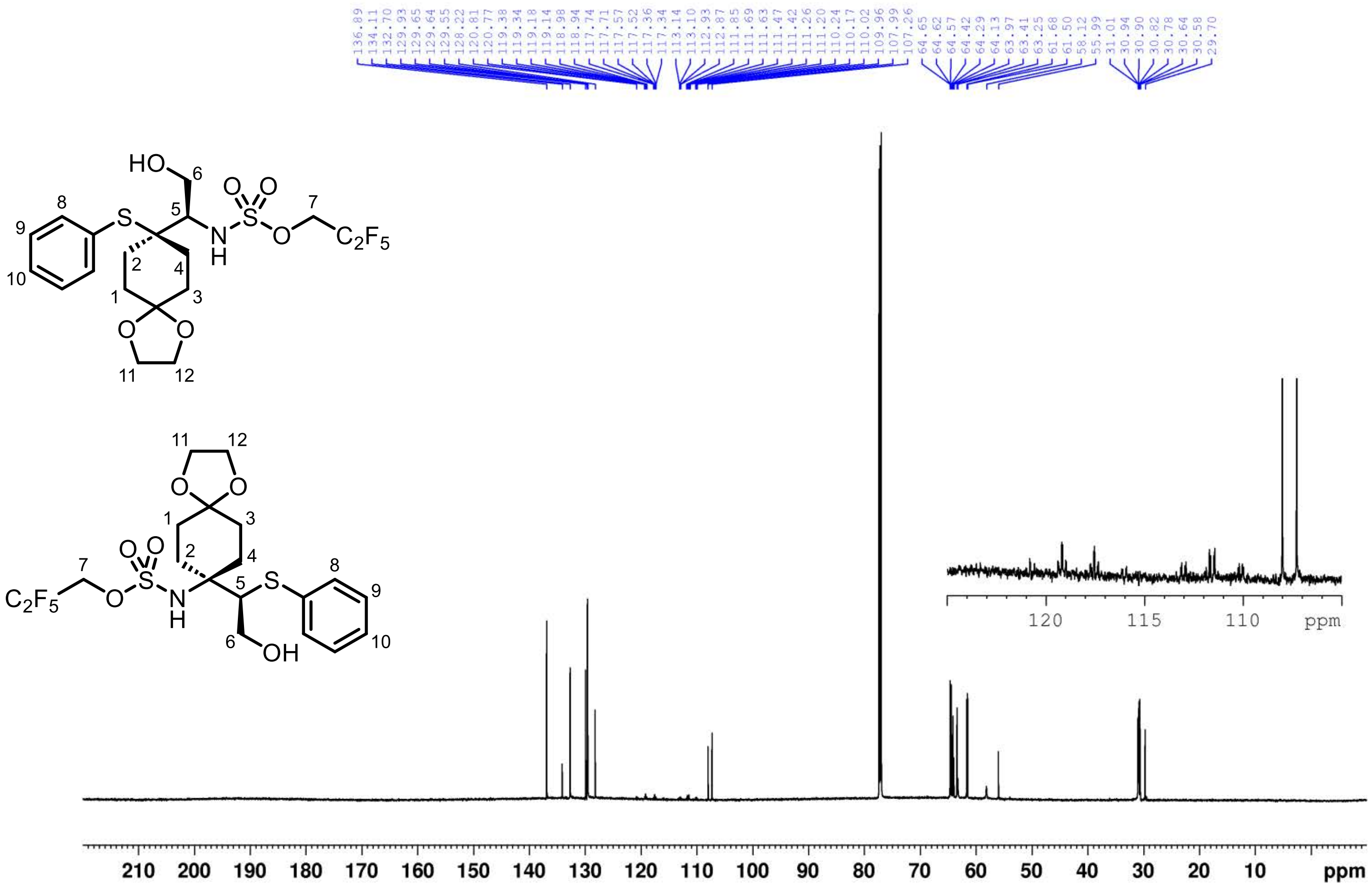

^{19}F NMR (376 MHz, $CDCl_3$) for *2,2,3,3,3-pentafluoropropyl (R)-(2-hydroxy-1-(8-(phenylthio)-1,4-dioxaspiro[4.5]decan-8-yl)ethyl)sulfamate* and *2,2,3,3,3-pentafluoropropyl (R)-(8-(2-hydroxy-1-(phenylthio)ethyl)-1,4-dioxaspiro[4.5]decan-8-yl)sulfamate.* **Obtained following derivatisation of 9g**

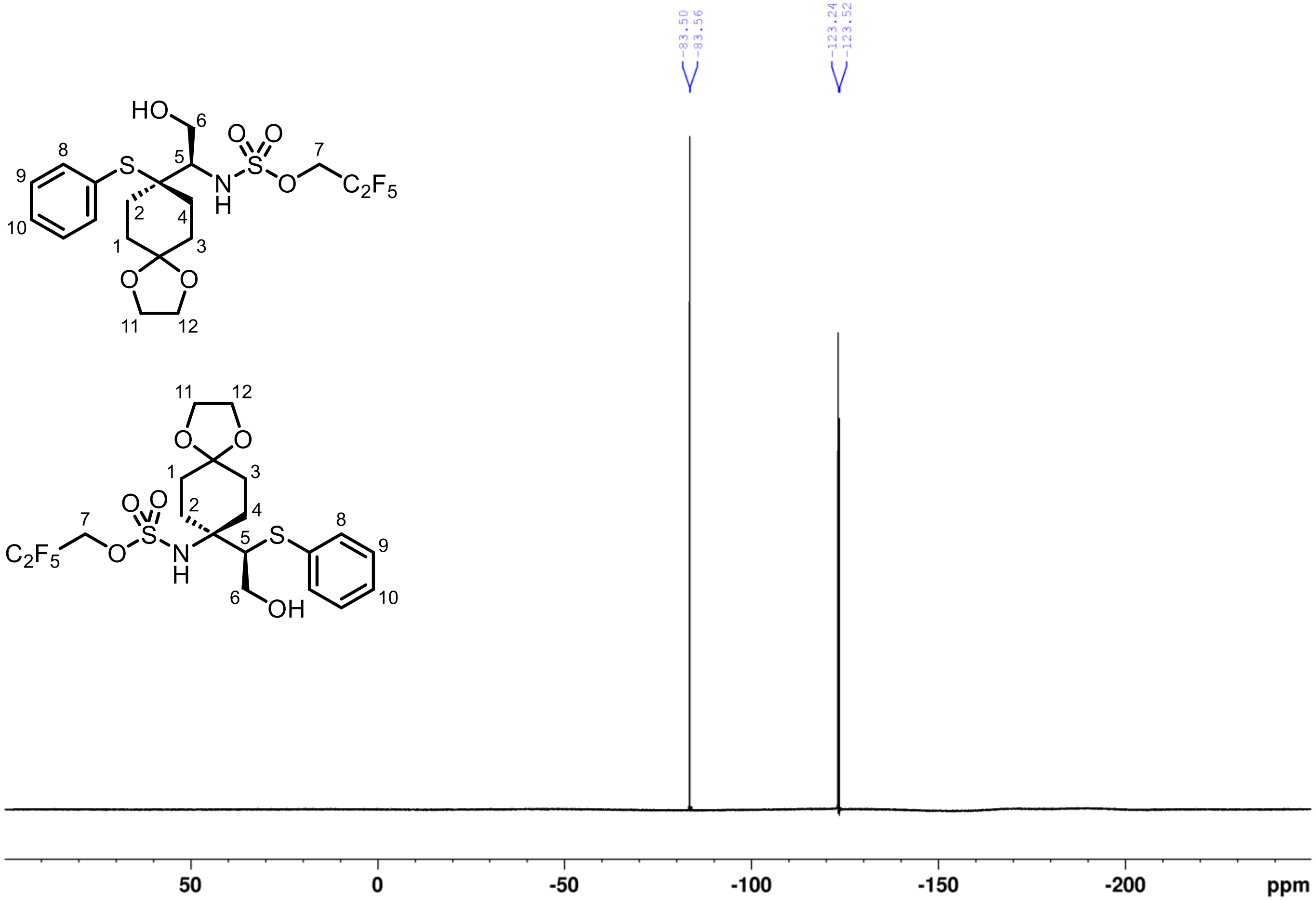

^{1}H NMR (400 MHz, $CDCl_3$) for *2,2,3,3,3-pentafluoropropyl (2S,3R)-2-(hydroxymethyl)-3-propylaziridine-1-sulfonate* (**9h**)

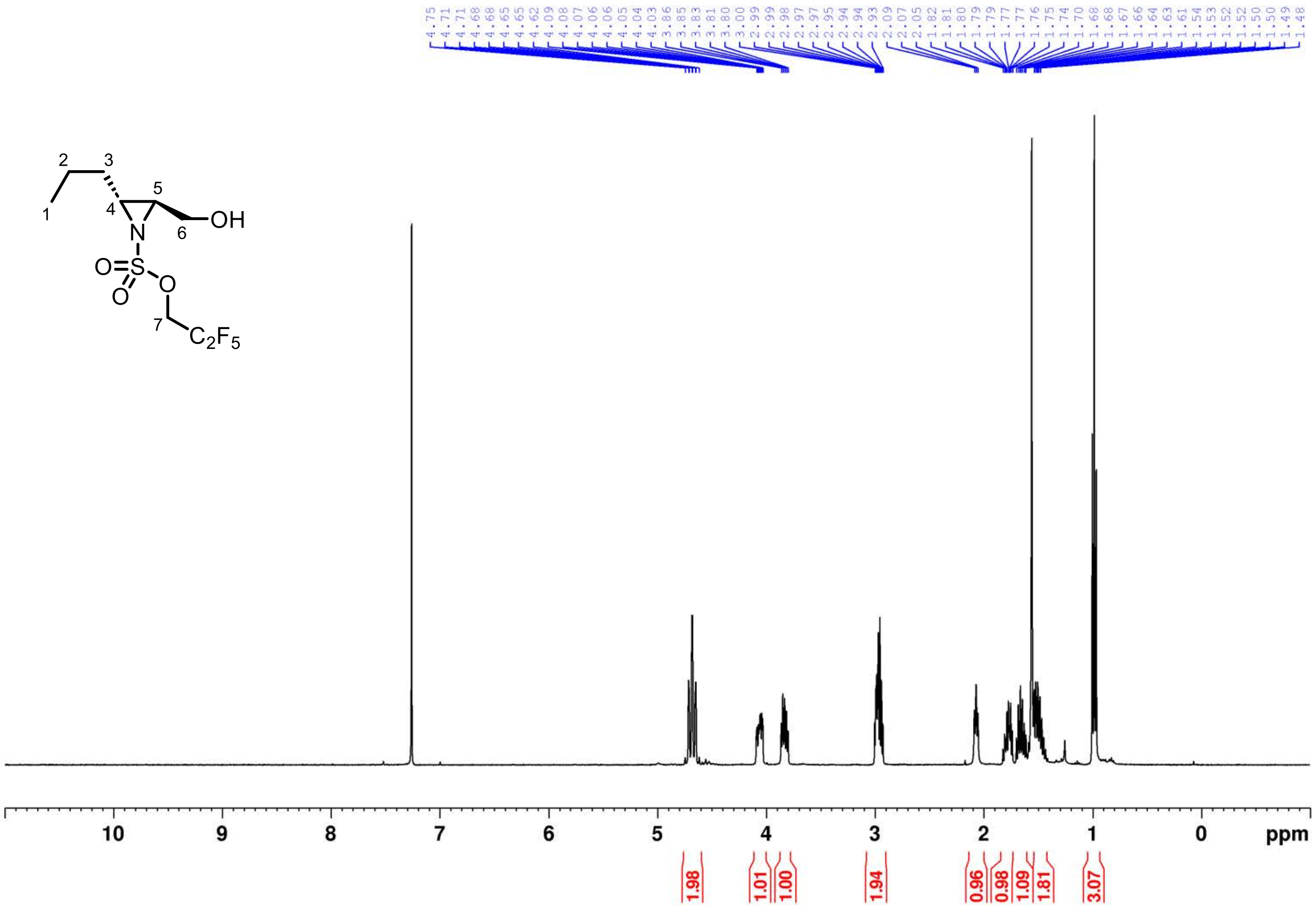

^{13}C NMR (176 MHz, CDCl$_3$) for *2,2,3,3,3-pentafluoropropyl (2S,3R)-2-(hydroxymethyl)-3-propylaziridine-1-sulfonate* (**9h**)

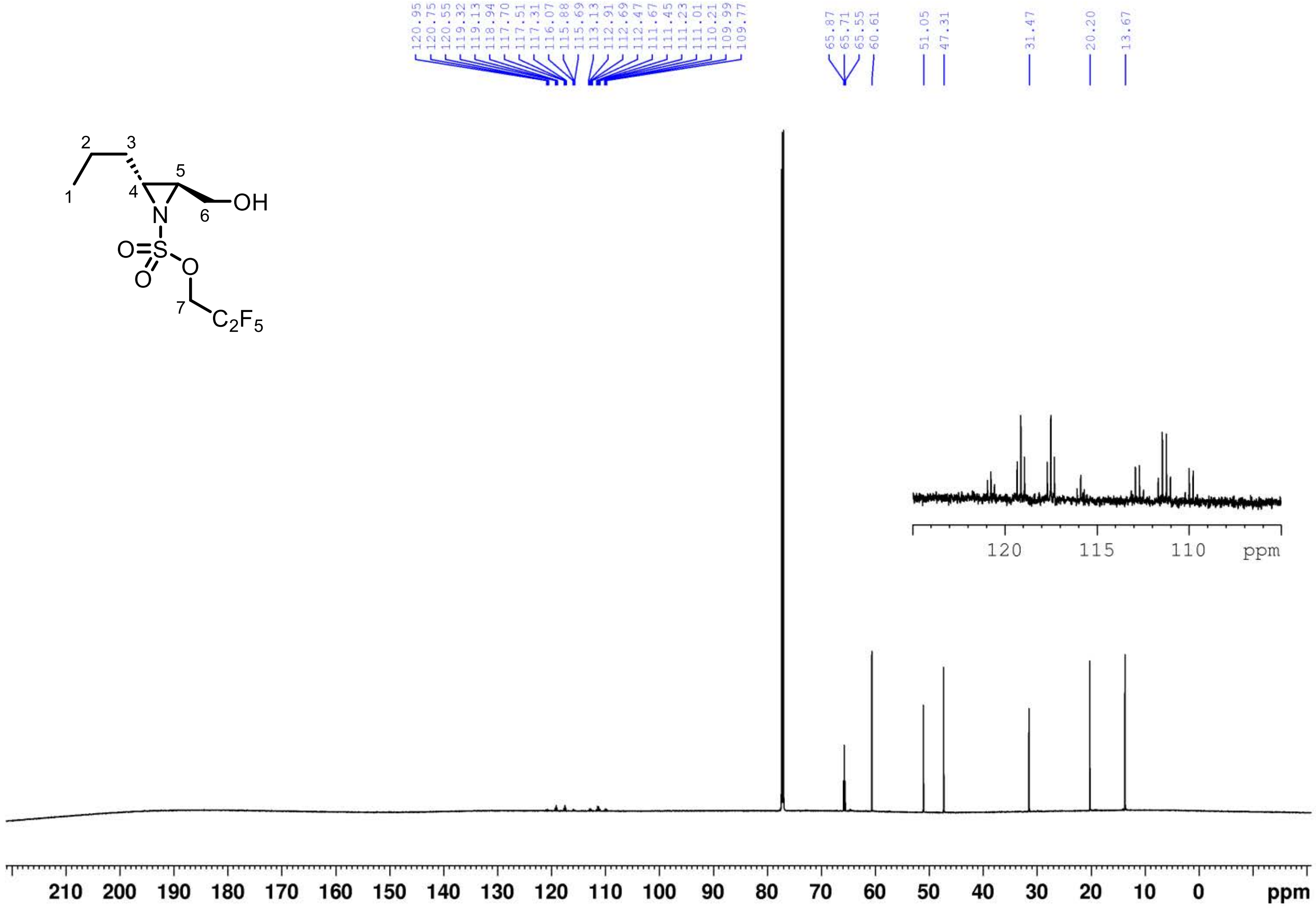

^{19}F NMR (376 MHz, $CDCl_3$) for *2,2,3,3,3-pentafluoropropyl (2S,3R)-2-(hydroxymethyl)-3-propylaziridine-1-sulfonate* (**9h**)

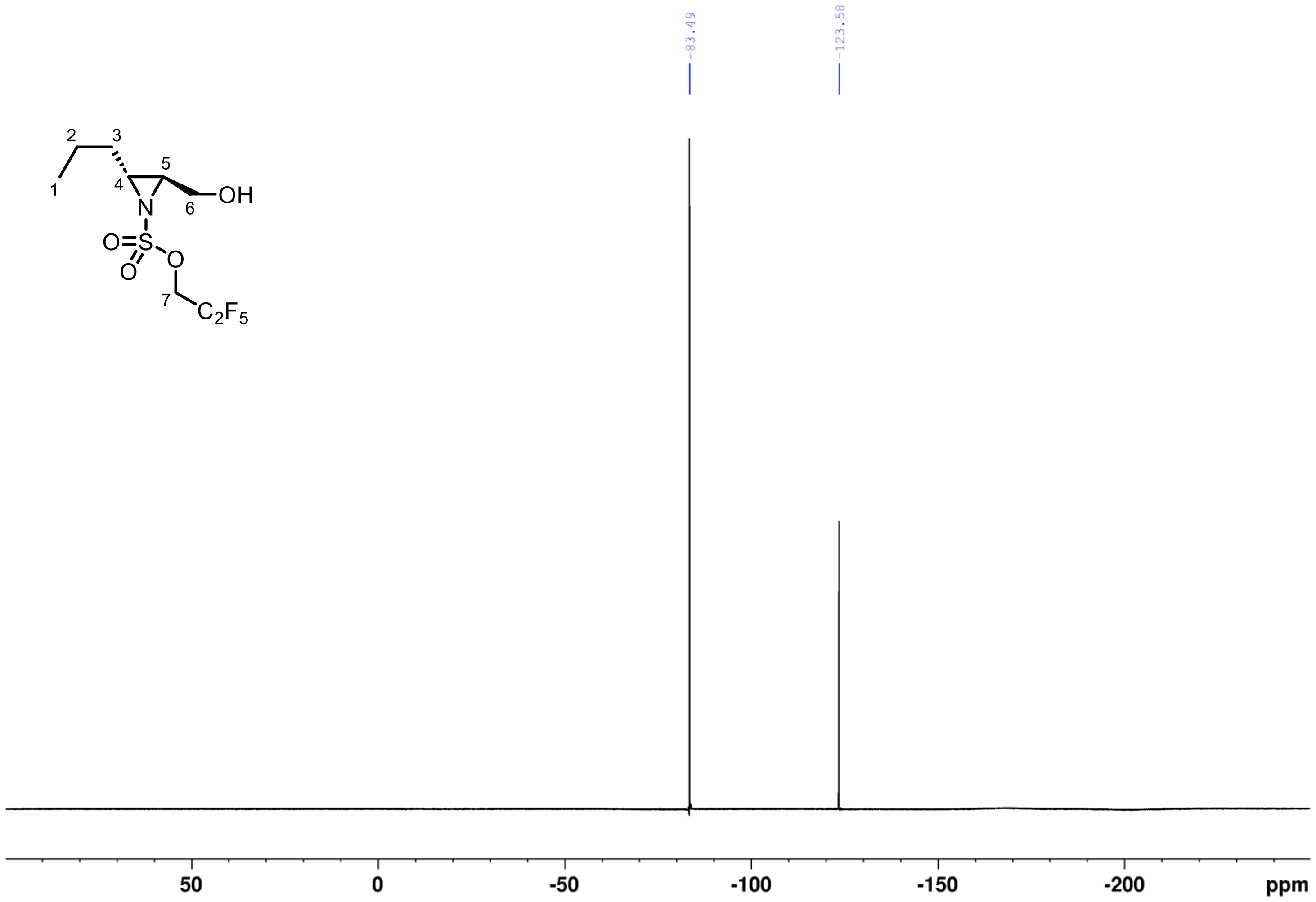

^{1}H NMR (700 MHz, CDCl$_3$) for *2,2,3,3,3-pentafluoropropyl ((2R,3S)-1-hydroxy-3-(phenylthio)hexan-2-yl)sulfamate.* **Obtained following derivatisation of 9h**

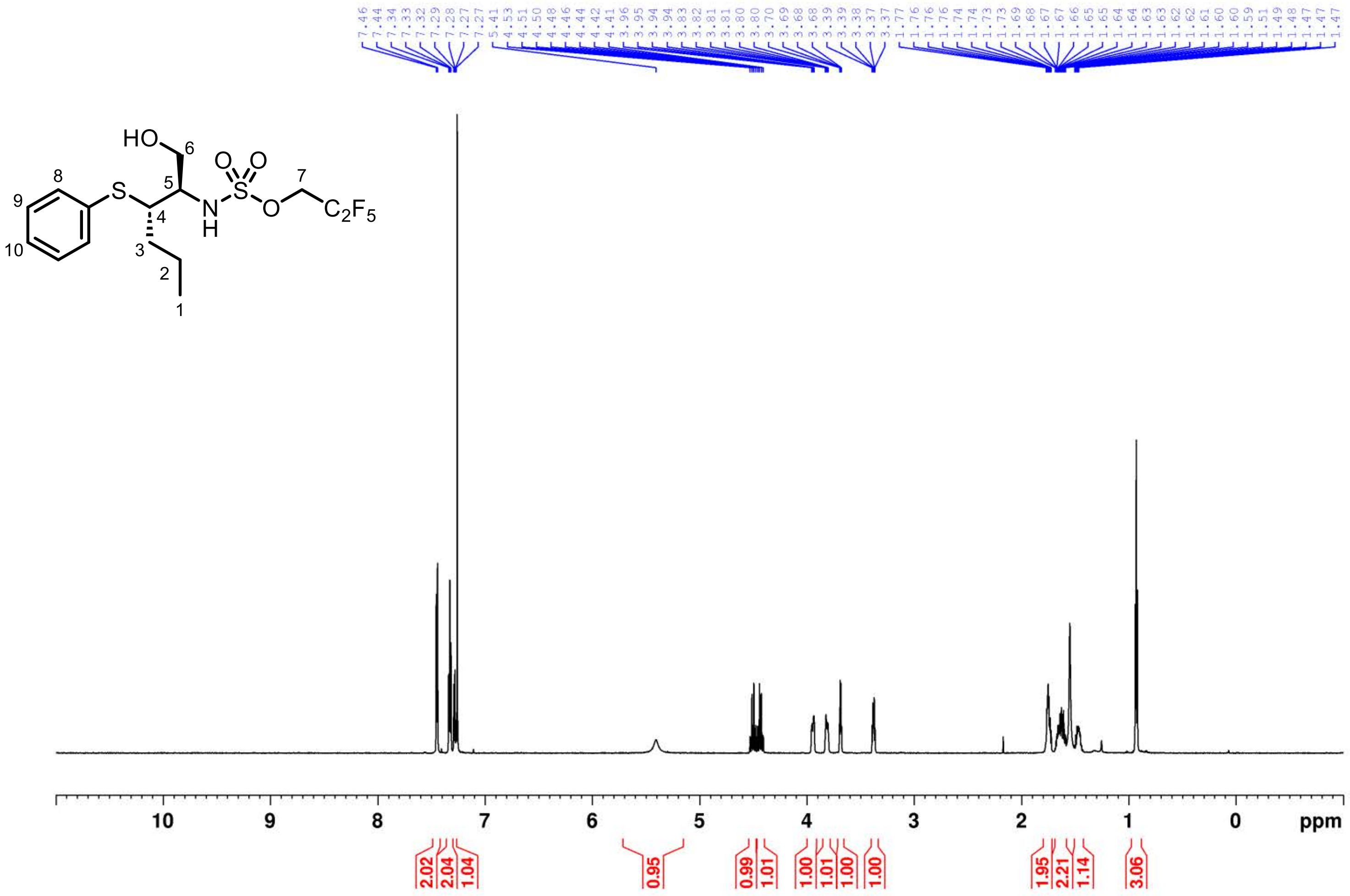

^{13}C NMR (176 MHz, $CDCl_3$) for *2,2,3,3,3-pentafluoropropyl ((2R,3S)-1-hydroxy-3-(phenylthio)hexan-2-yl)sulfamate.* **Obtained following derivatisation of 9h**

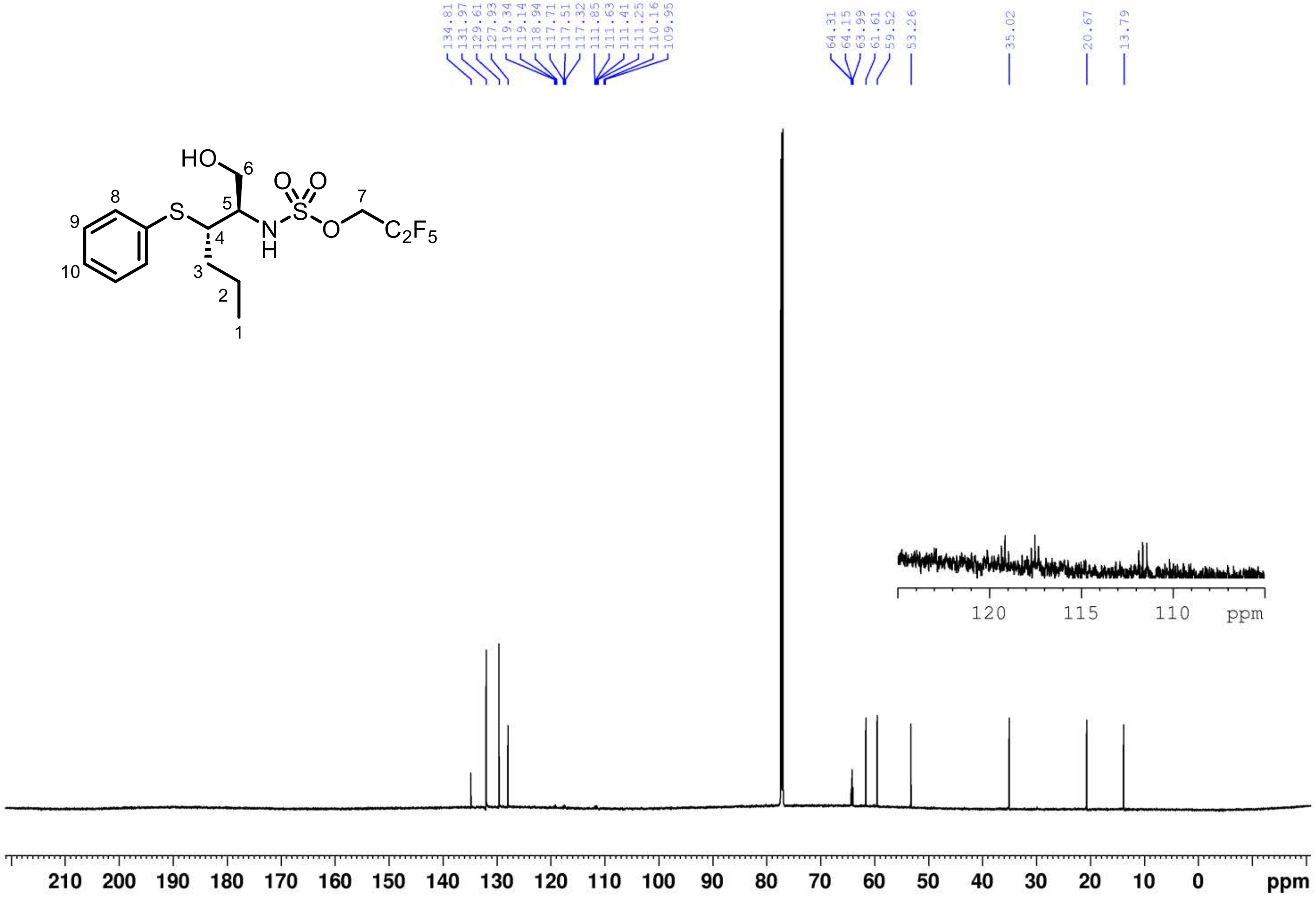

^{19}F NMR (376 MHz, CDCl$_3$) for *2,2,3,3,3-pentafluoropropyl ((2R,3S)-1-hydroxy-3-(phenylthio)hexan-2-yl)sulfamate.* **Obtained following derivatisation of 9h**

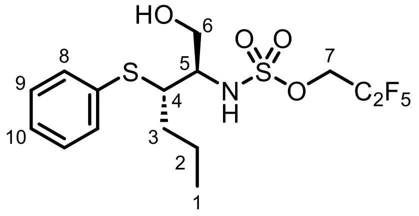

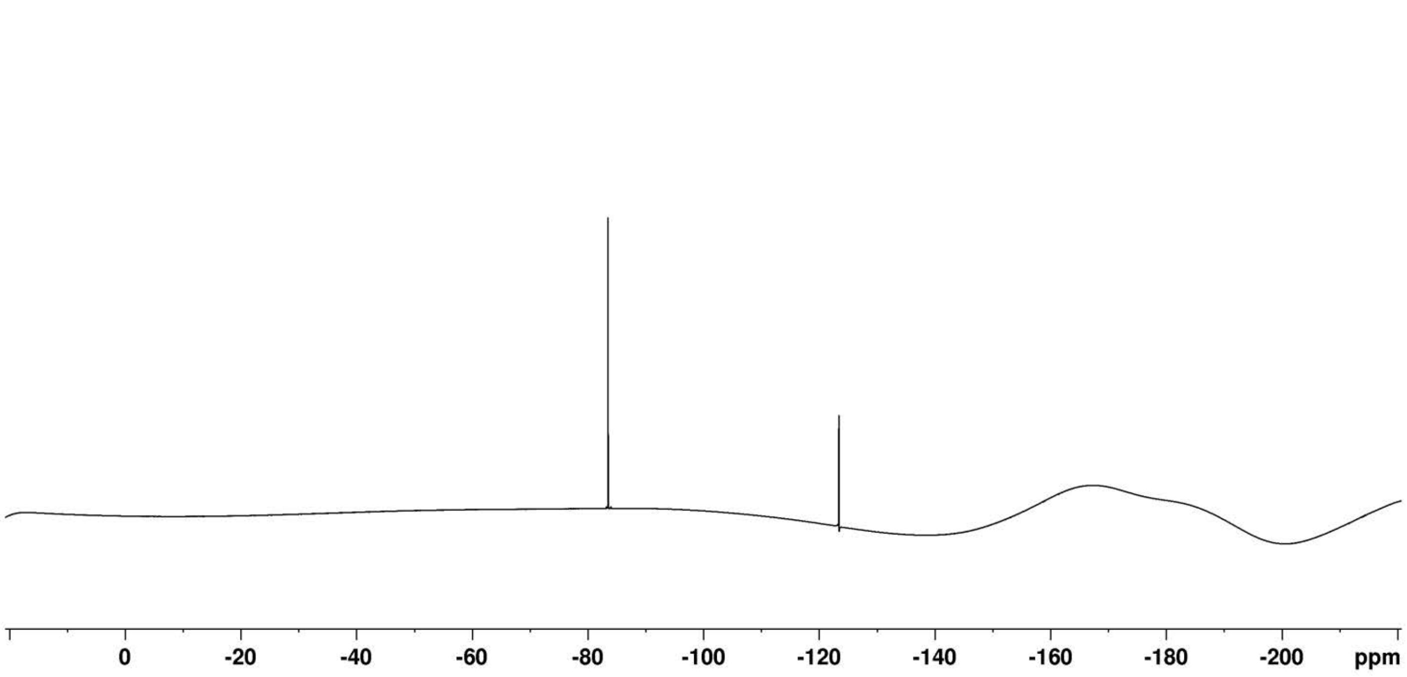

^{1}H NMR (500 MHz, $CDCl_3$) for *2,2,3,3,3-pentafluoropropyl (R)-(2-hydroxy-1-(1-(phenylthio)cyclopentyl)ethyl)sulfamate* (**10a**)

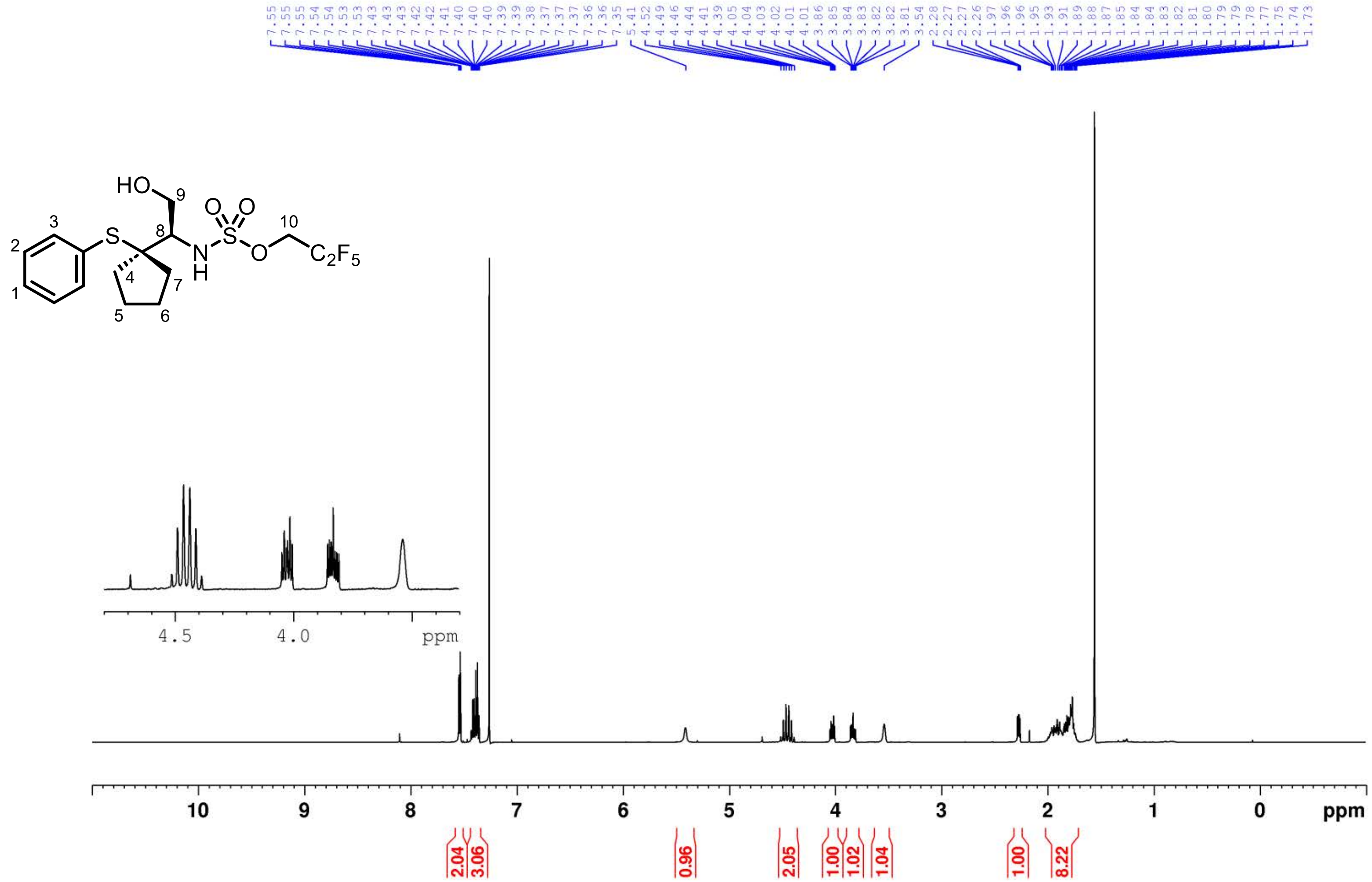

^{13}C NMR (126 MHz, CDCl$_3$) for *2,2,3,3,3-pentafluoropropyl (R)-(2-hydroxy-1-(1-(phenylthio)cyclopentyl)ethyl)sulfamate* (**10a**)

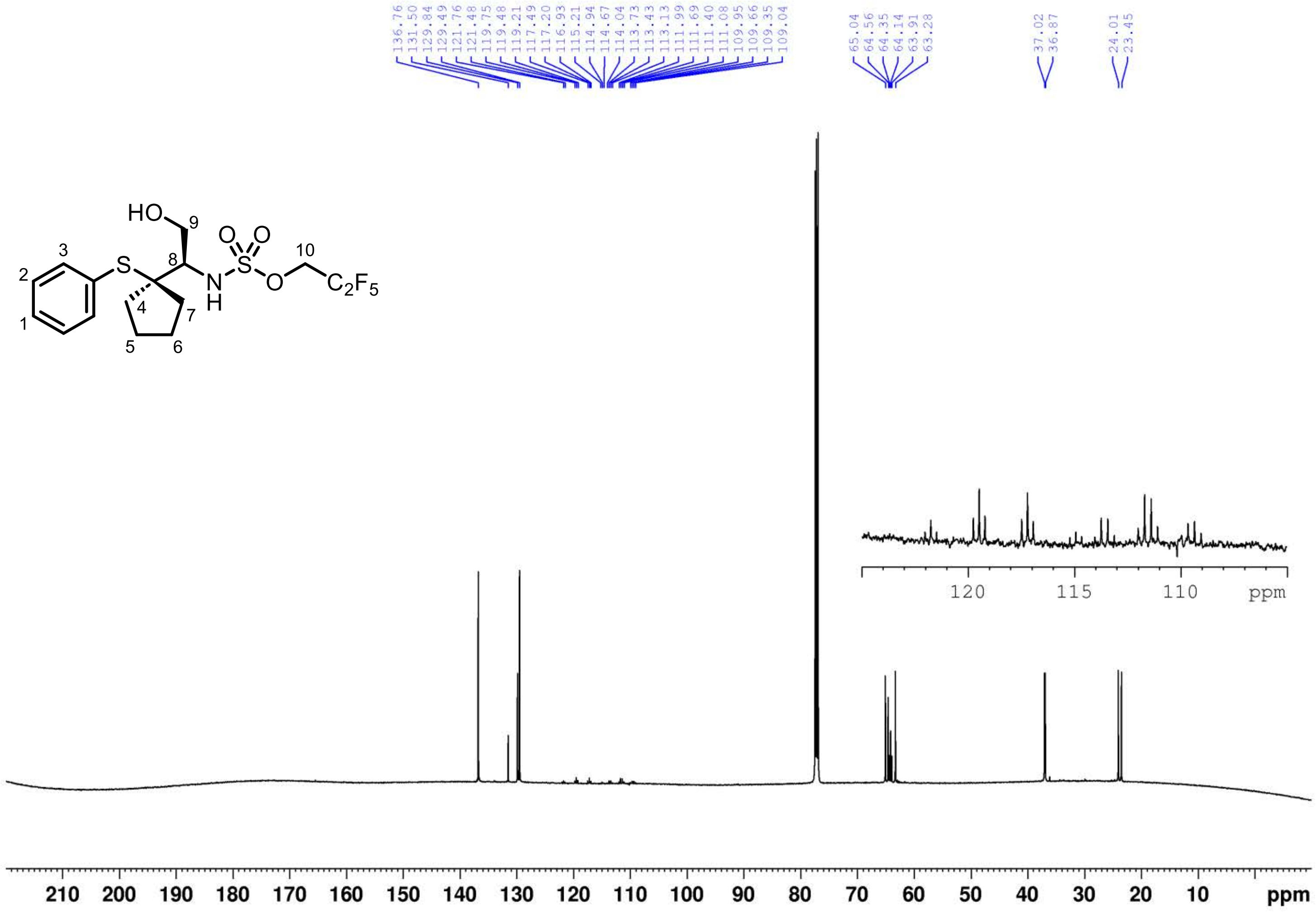

^{19}F NMR (376 MHz, CDCl$_3$) for *2,2,3,3,3-pentafluoropropyl (R)-(2-hydroxy-1-(1-(phenylthio)cyclopentyl)ethyl)sulfamate* (**10a**)

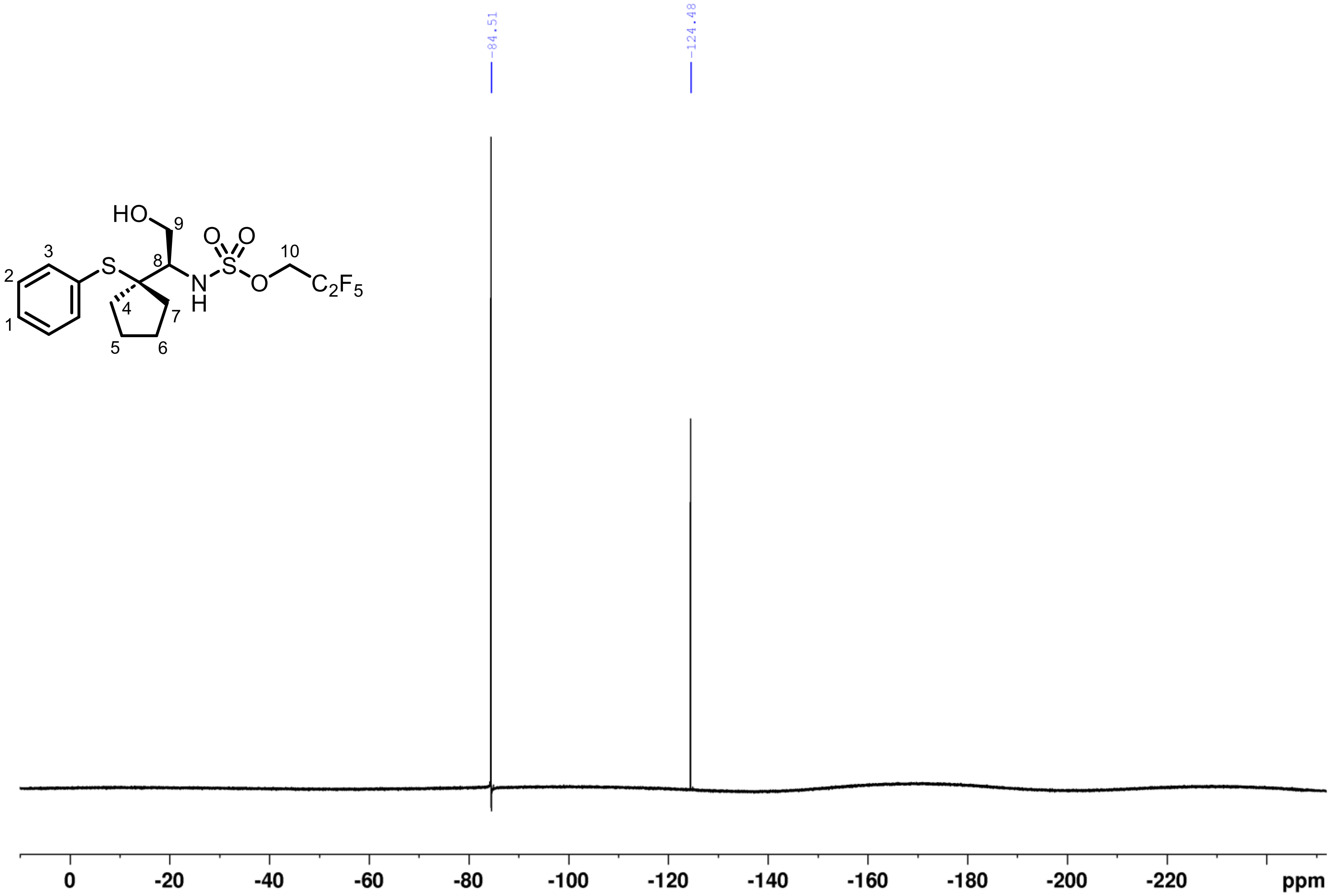

^{1}H NMR (700 MHz, CDCl$_3$) for *2,2,3,3,3-pentafluoropropyl (R)-(2-hydroxy-1-(1-(phenylthio)cycloheptyl)ethyl)sulfamate* (**10b**)

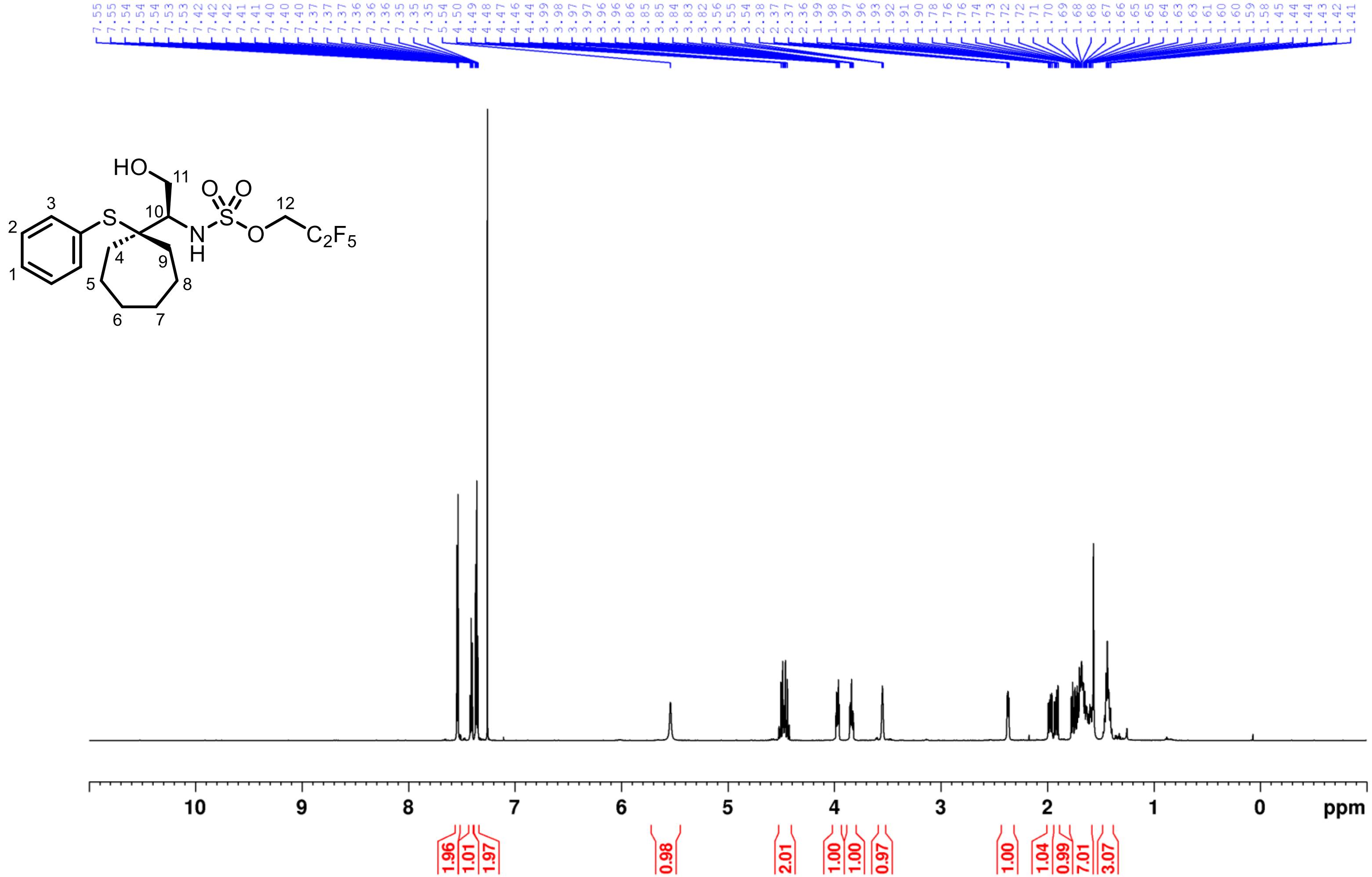

^{13}C NMR (176 MHz, CDCl$_3$) for *2,2,3,3,3-pentafluoropropyl (R)-(2-hydroxy-1-(1-(phenylthio)cycloheptyl)ethyl)sulfamate* (**10b**)

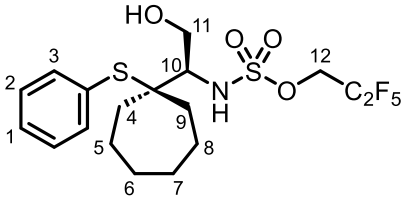

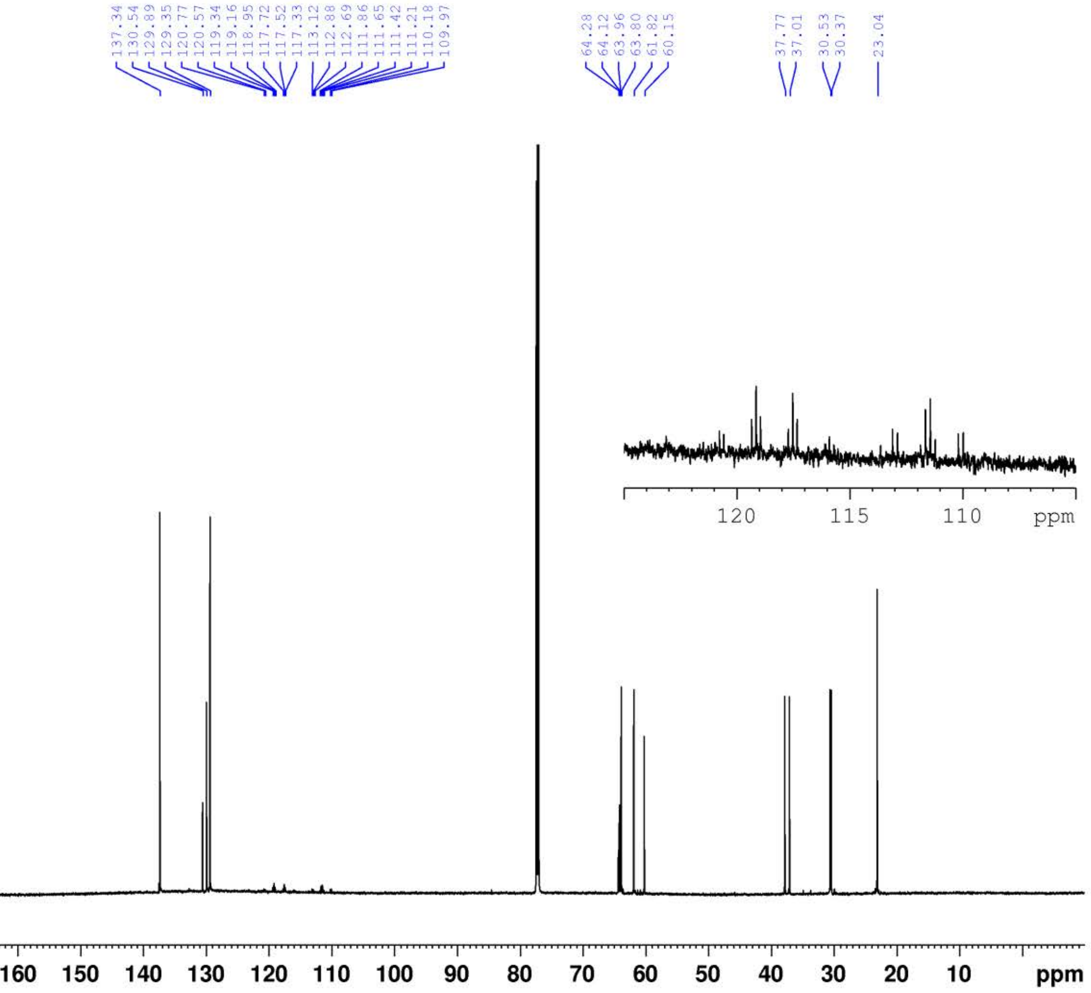

^{19}F NMR (376 MHz, CDCl$_3$) for *2,2,3,3,3-pentafluoropropyl (R)-(2-hydroxy-1-(1-(phenylthio)cycloheptyl)ethyl)sulfamate* (**10b**)

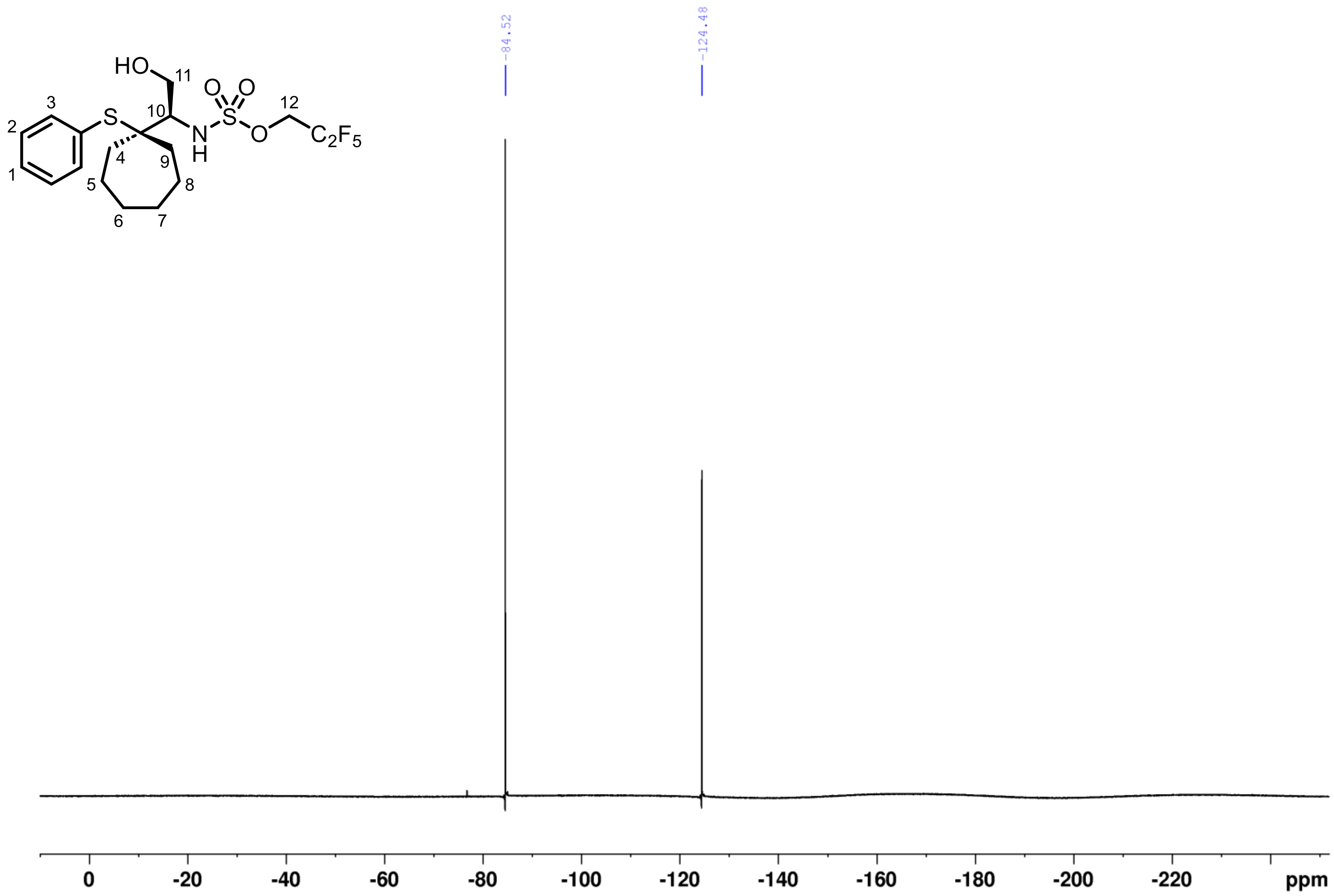

¹H NMR (500 MHz, CDCl₃) for *2,2,3,3,3-pentafluoropropyl (2R,3S)-3-(hydroxymethyl)-2-methyl-2-(4-methylpent-3-en-1-yl)aziridine-1-sulfonate* **(9i)**

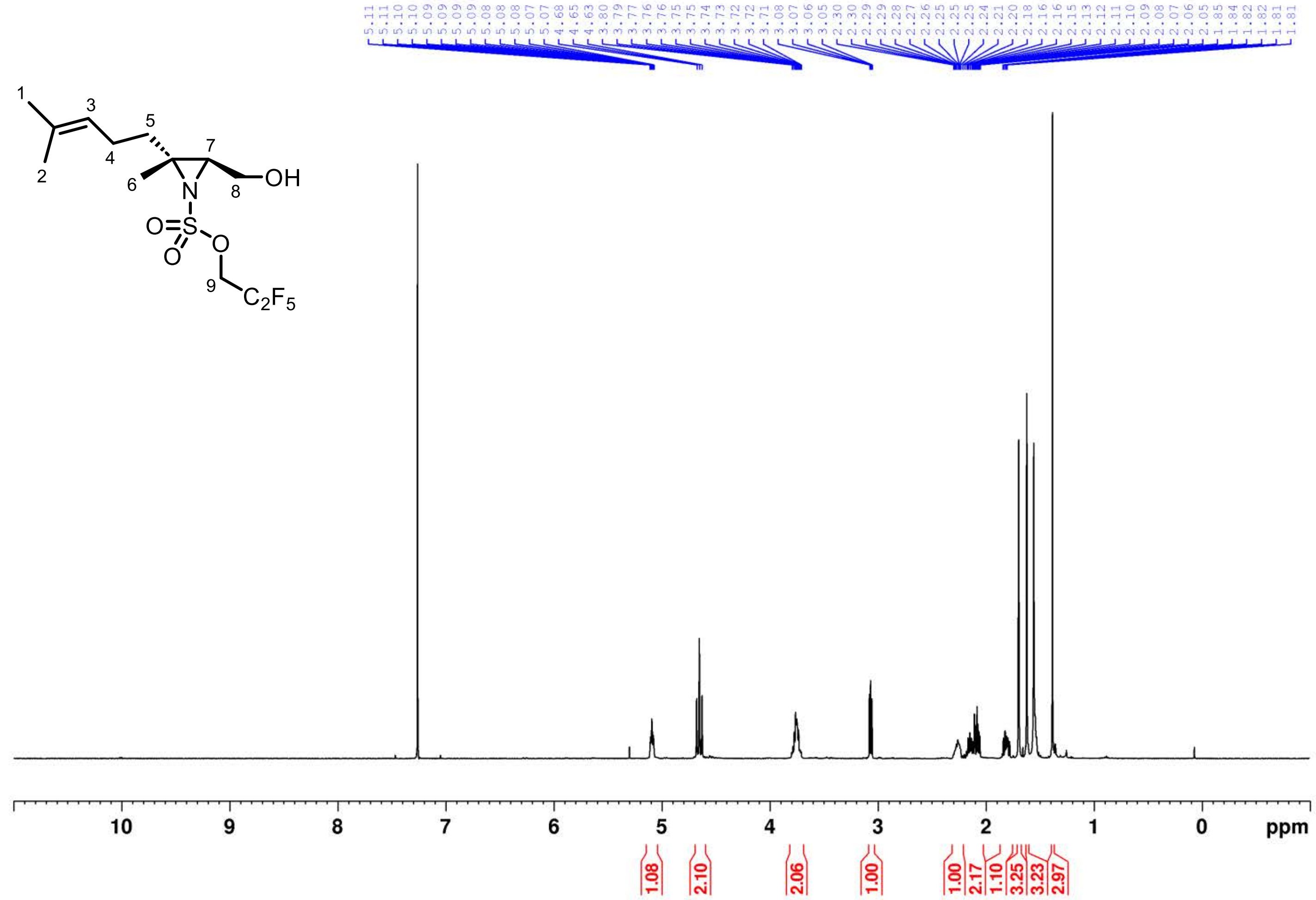

^{13}C NMR (126 MHz, CDCl$_3$) for *2,2,3,3,3-pentafluoropropyl (2R,3S)-3-(hydroxymethyl)-2-methyl-2-(4-methylpent-3-en-1-yl)aziridine-1-sulfonate* (**9i**)

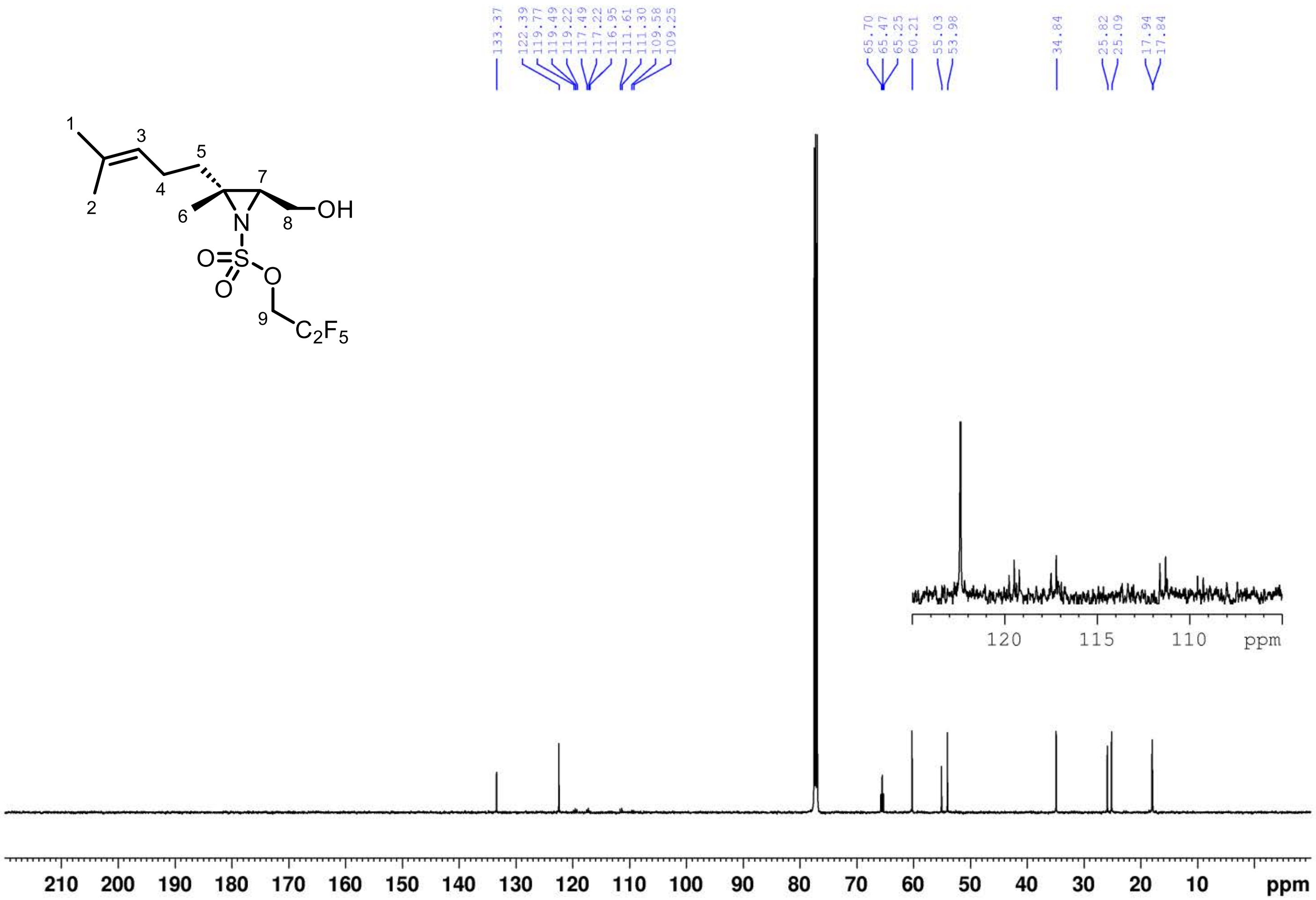

^{19}F NMR (376 MHz, CDCl$_3$) for *2,2,3,3,3-pentafluoropropyl (2R,3S)-3-(hydroxymethyl)-2-methyl-2-(4-methylpent-3-en-1-yl)aziridine-1-sulfonate* (**9i**)

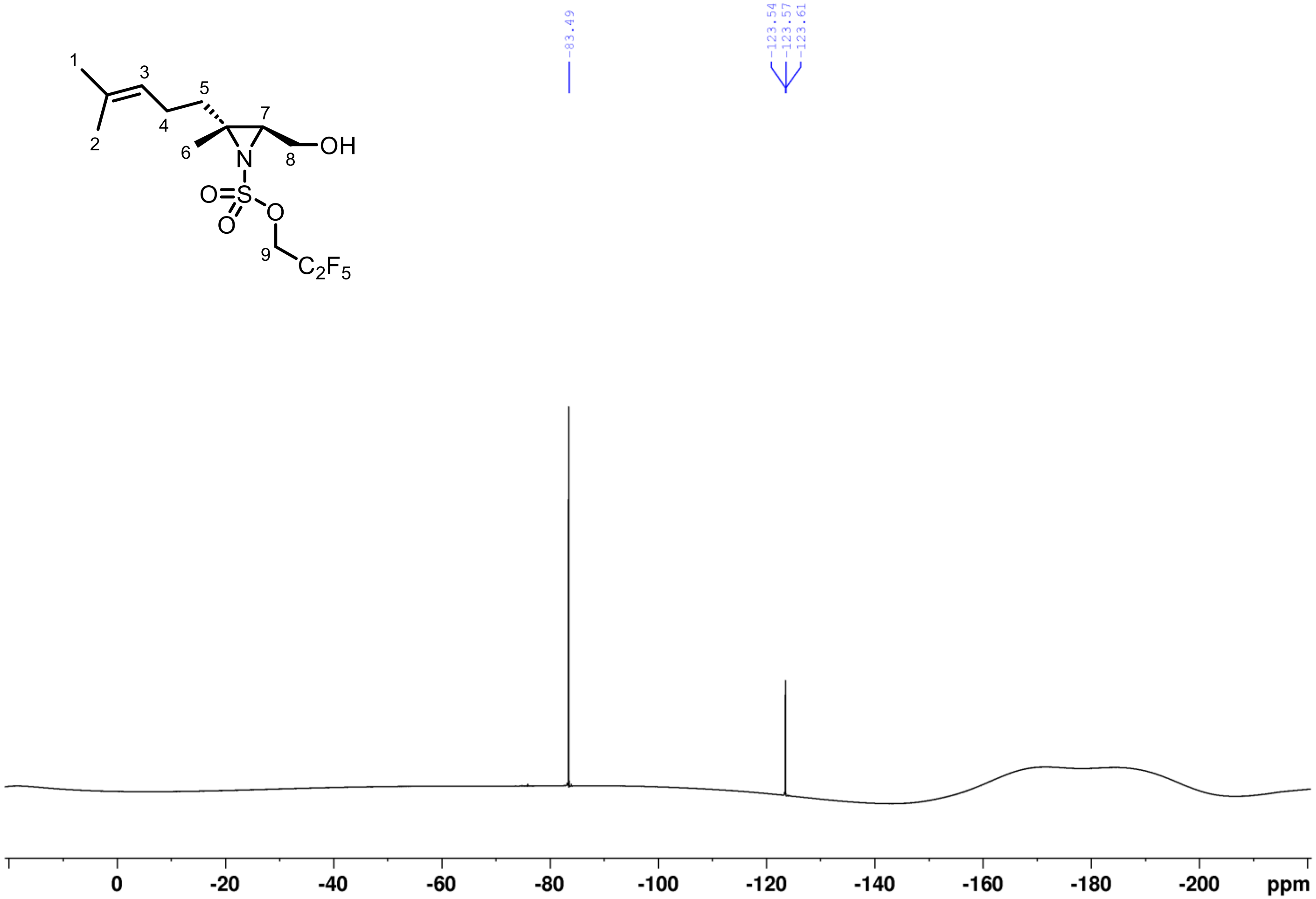

^{1}H NMR (700 MHz, CDCl$_3$) for *2,2,3,3,3-pentafluoropropyl ((2R,3S)-1-hydroxy-3,7-dimethyl-3-(phenylthio)oct-6-en-2-yl)sulfamate.* **Obtained following derivatisation of 9i**

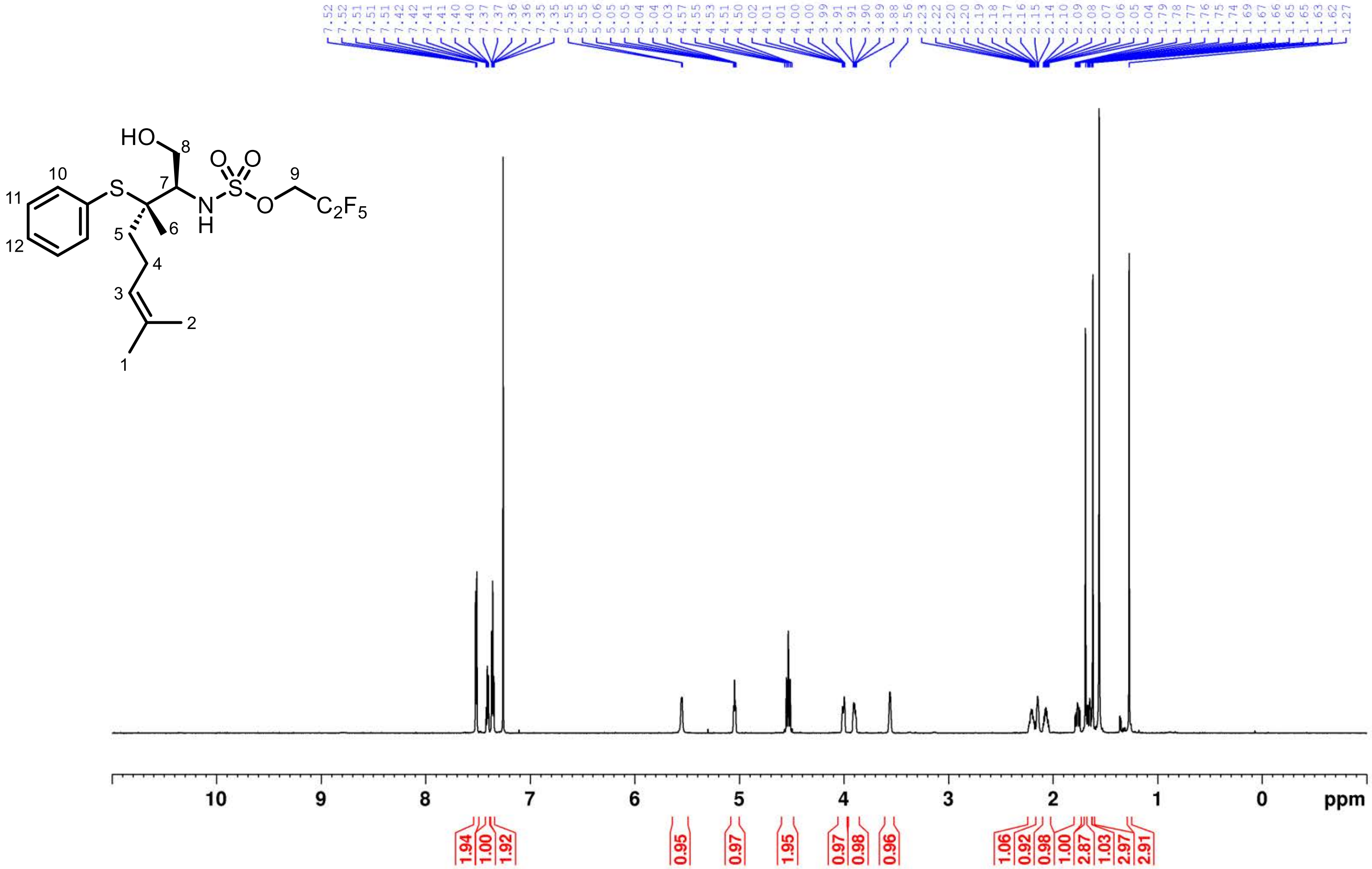

^{13}C NMR (176 MHz, CDCl$_3$) for *2,2,3,3,3-pentafluoropropyl ((2R,3S)-1-hydroxy-3,7-dimethyl-3-(phenylthio)oct-6-en-2-yl)sulfamate.* **Obtained following derivatisation of 9i**

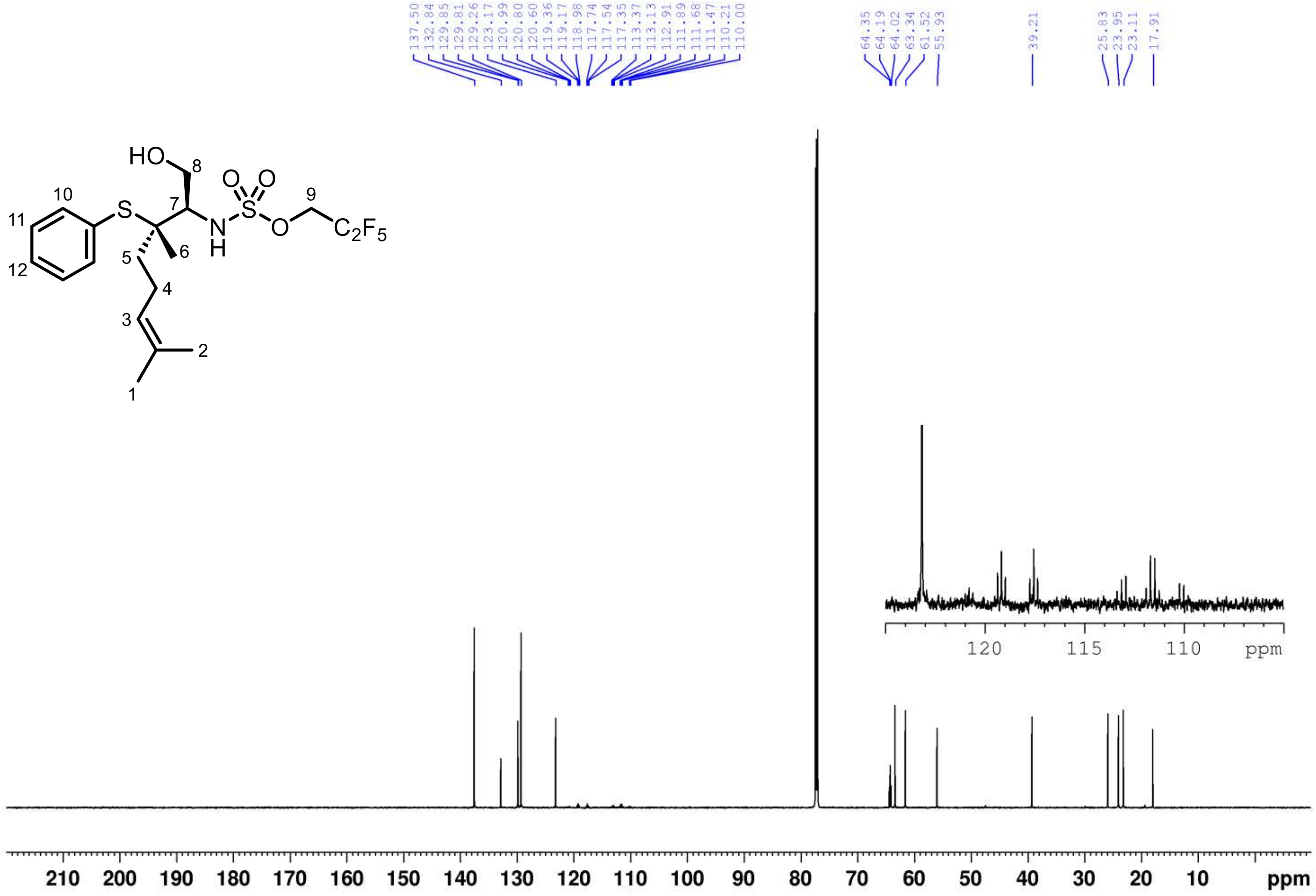

^{19}F NMR (376 MHz, $CDCl_3$) for *2,2,3,3,3-pentafluoropropyl ((2R,3S)-1-hydroxy-3,7-dimethyl-3-(phenylthio)oct-6-en-2-yl)sulfamate.* **Obtained following derivatisation of 9i**

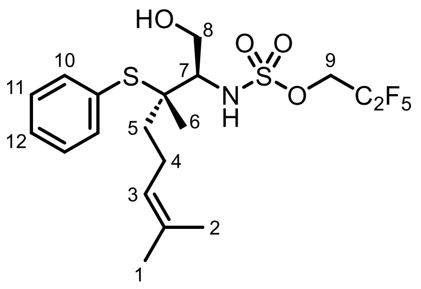

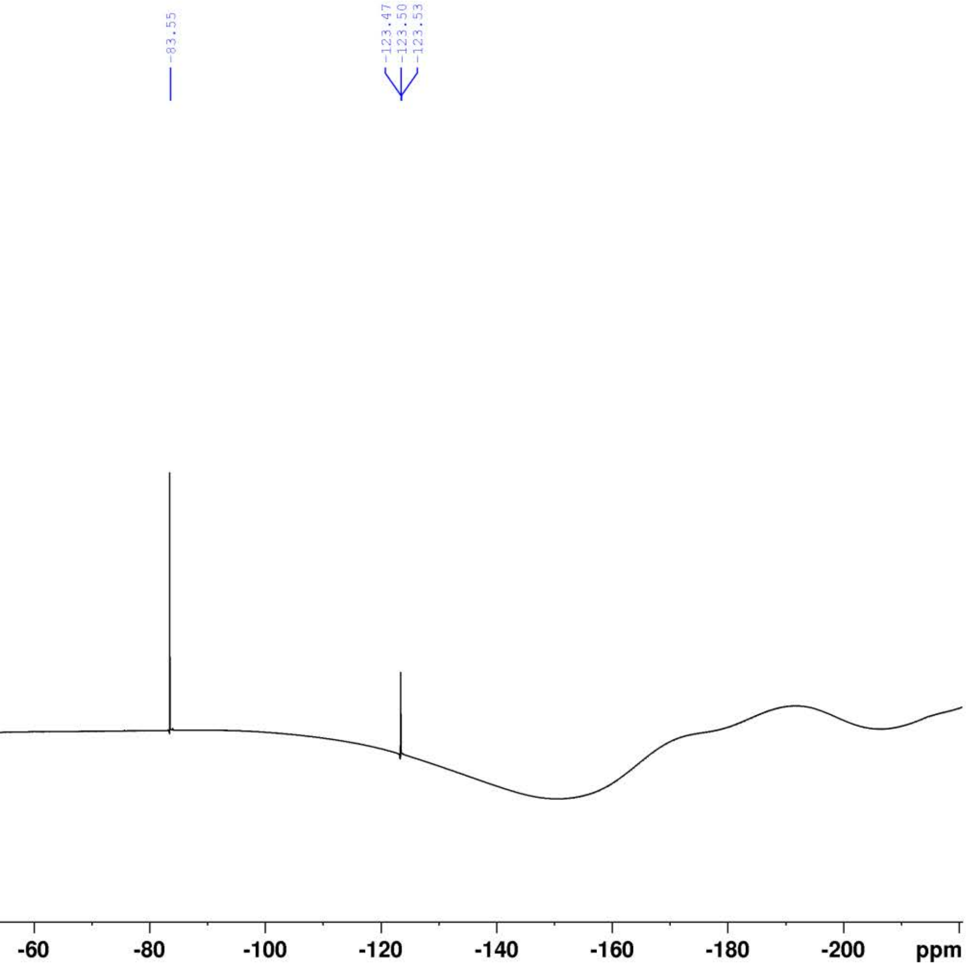

^{1}H NMR (700 MHz, CDCl$_3$) for *2,2,3,3,3-pentafluoropropyl (2S,3S)-3-(hydroxymethyl)-2-methyl-2-(4-methylpent-3-en-1-yl)aziridine-1-sulfonate* (**9j**)

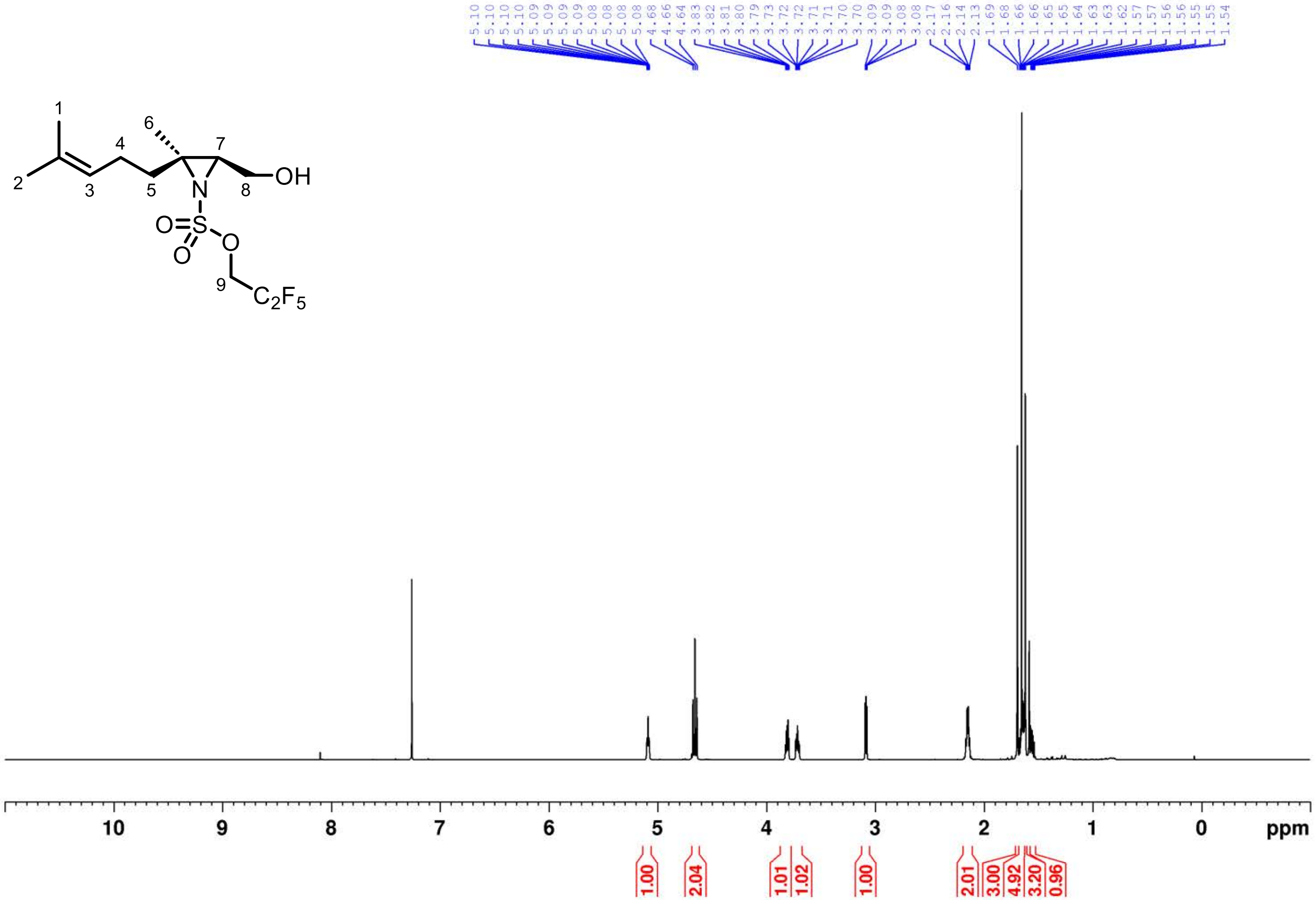

^{13}C NMR (176 MHz, $CDCl_3$) for *2,2,3,3,3-pentafluoropropyl (2S,3S)-3-(hydroxymethyl)-2-methyl-2-(4-methylpent-3-en-1-yl)aziridine-1-sulfonate* (**9j**)

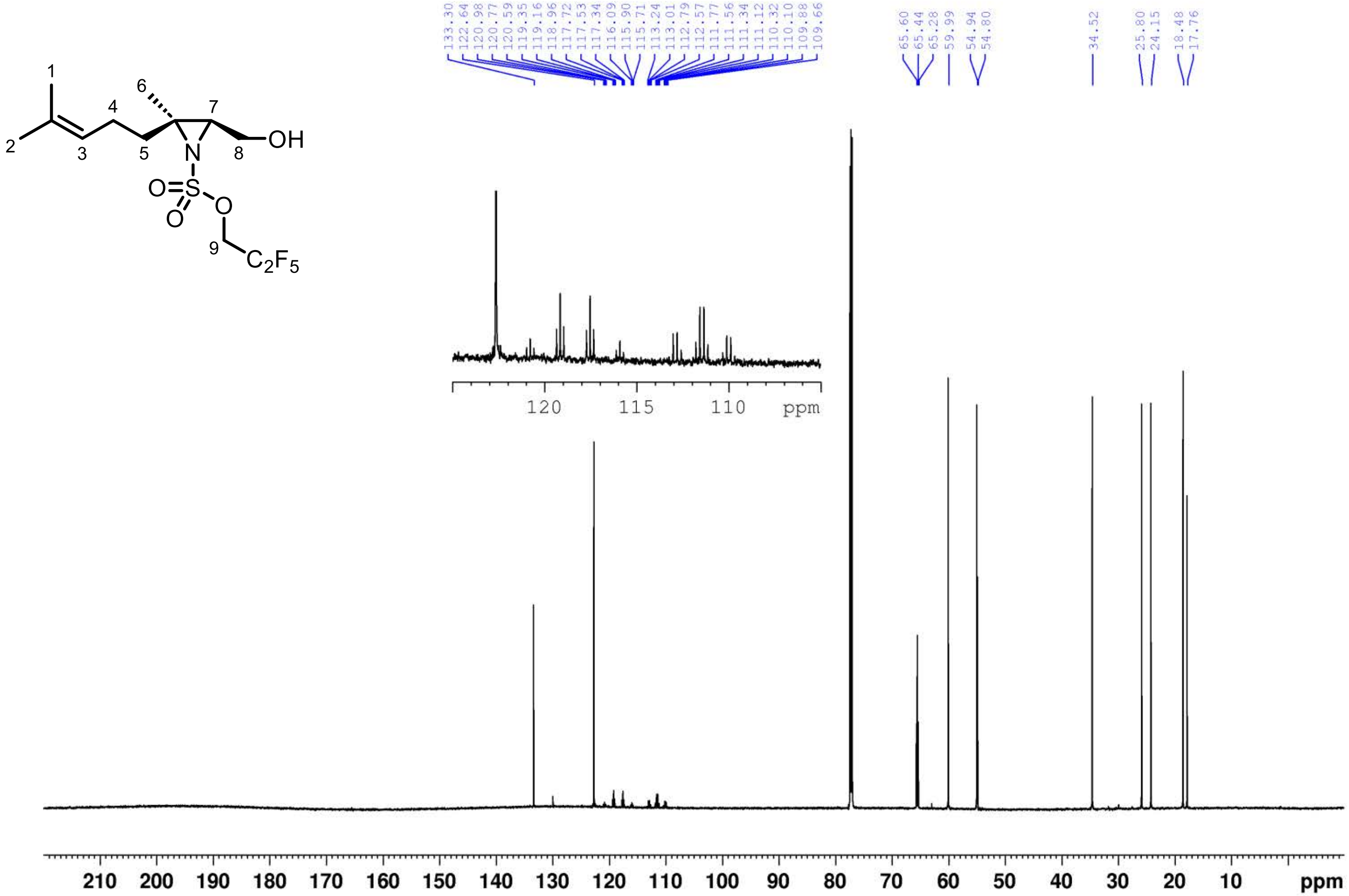

^{19}F NMR (376 MHz, $CDCl_3$) for *2,2,3,3,3-pentafluoropropyl (2S,3S)-3-(hydroxymethyl)-2-methyl-2-(4-methylpent-3-en-1-yl)aziridine-1-sulfonate* (**9j**)

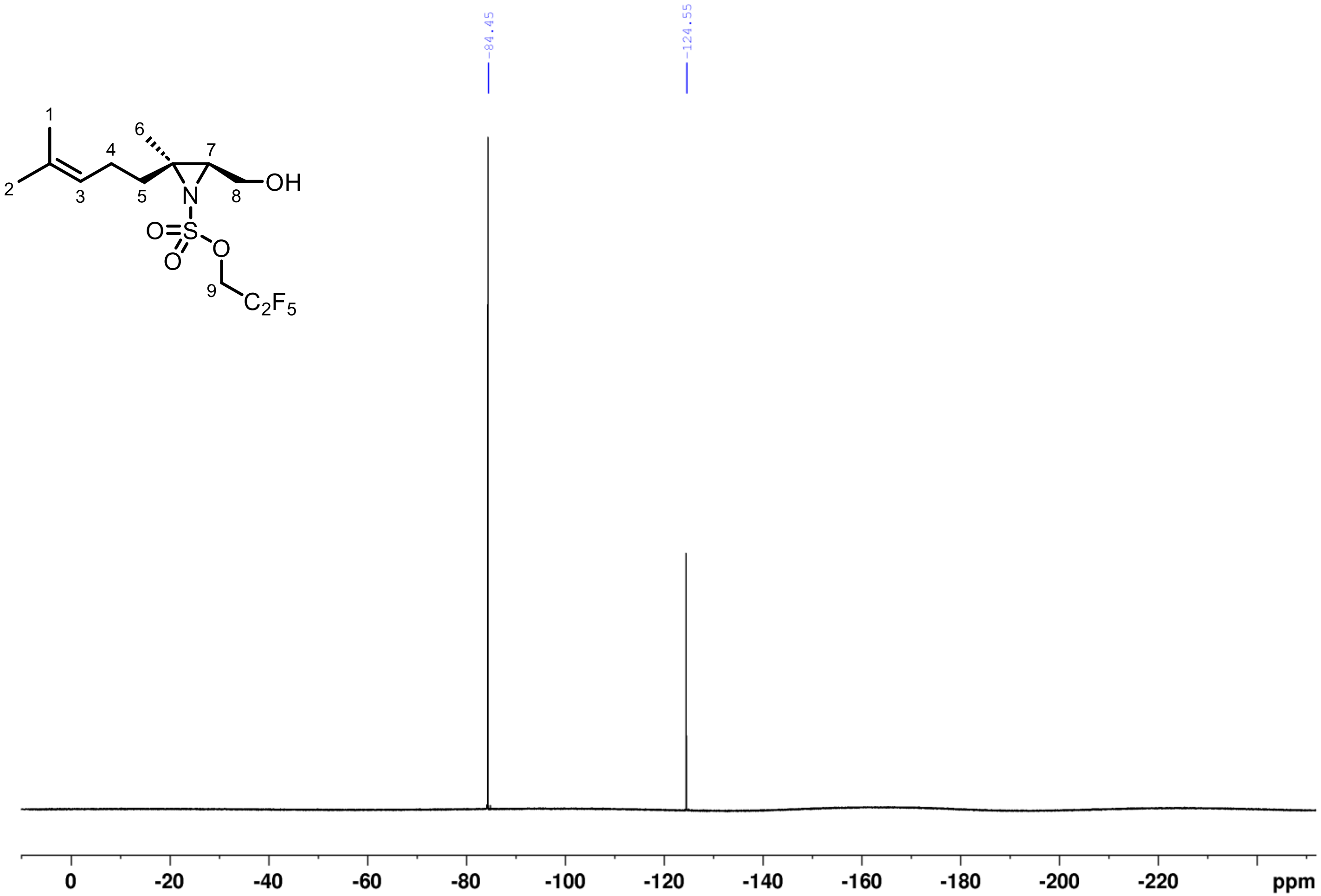

^{1}H NMR (700 MHz, $CDCl_3$) for a semi-purified sample of *2,2,3,3,3-pentafluoropropyl (Z)-3-(5-hydroxy-3-methylpent-3-en-1-yl)-2,2-dimethylaziridine-1-sulfonate*

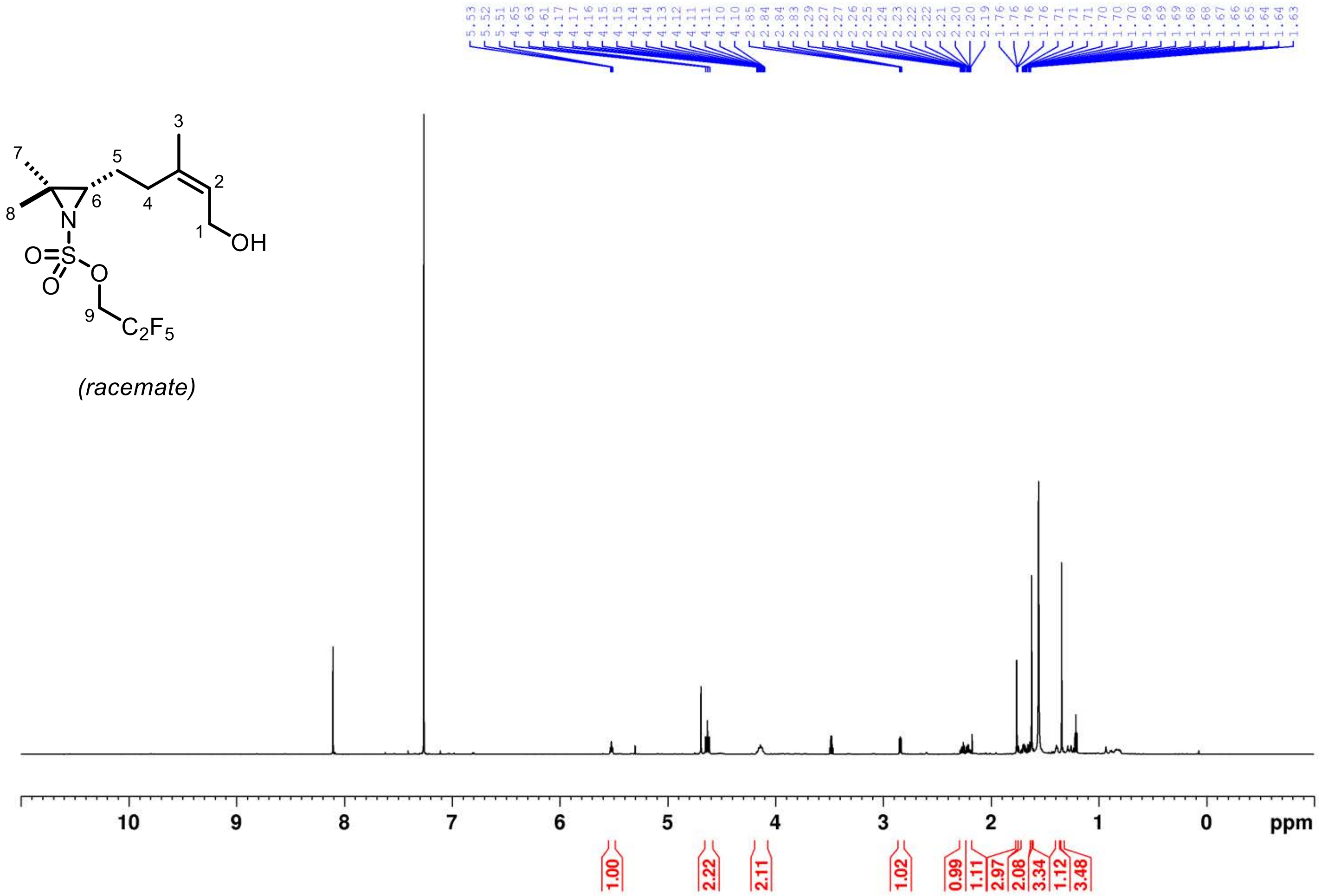

^{13}C NMR (176 MHz, $CDCl_3$) for a semi-purified sample of *2,2,3,3,3-pentafluoropropyl (Z)-3-(5-hydroxy-3-methylpent-3-en-1-yl)-2,2-dimethylaziridine-1-sulfonate*

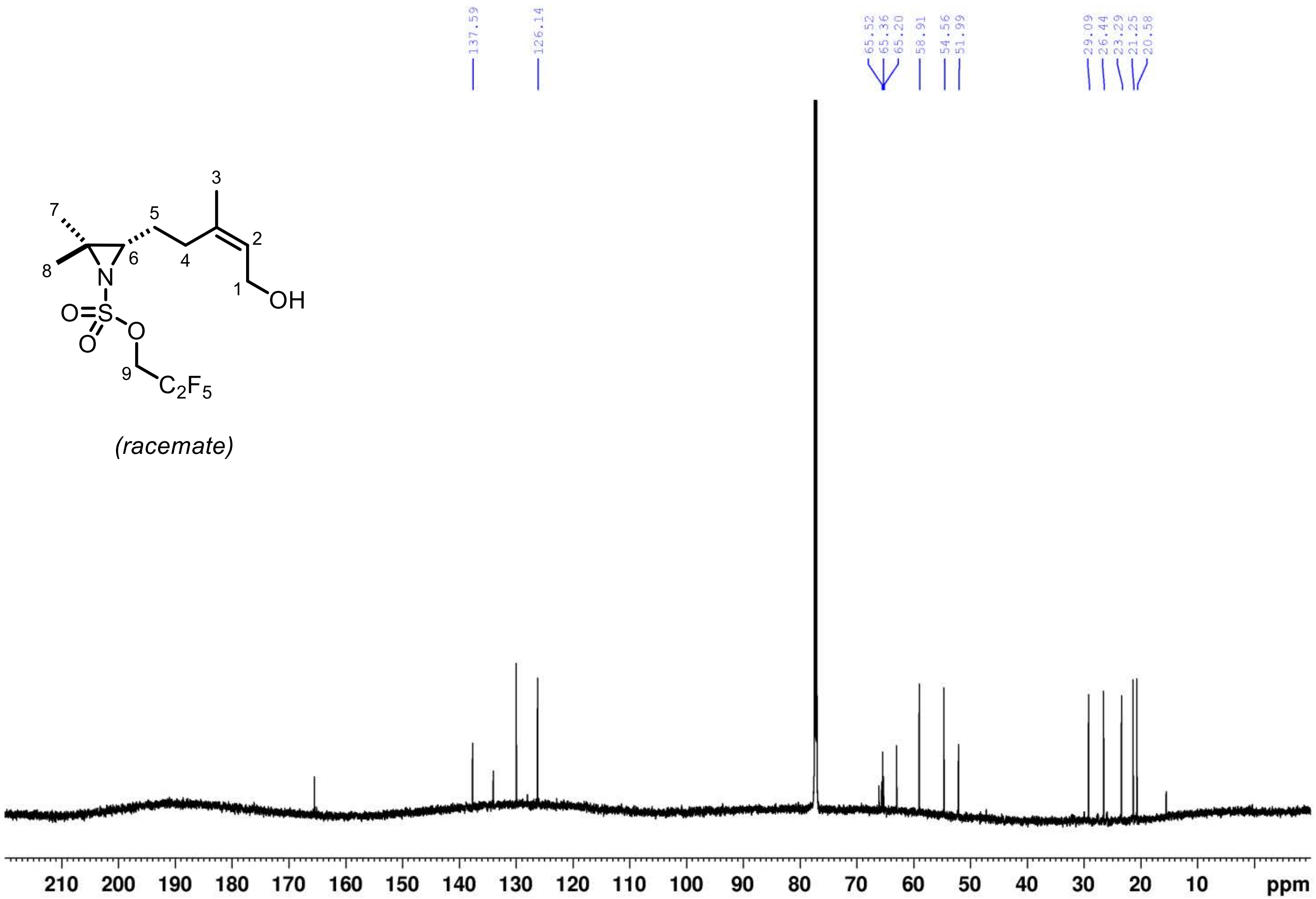

^{19}F NMR (376 MHz, $CDCl_3$) for a semi-purified sample of *2,2,3,3,3-pentafluoropropyl (Z)-3-(5-hydroxy-3-methylpent-3-en-1-yl)-2,2-dimethylaziridine-1-sulfonate*

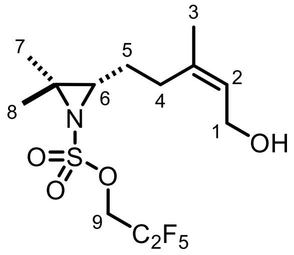

(racemate)

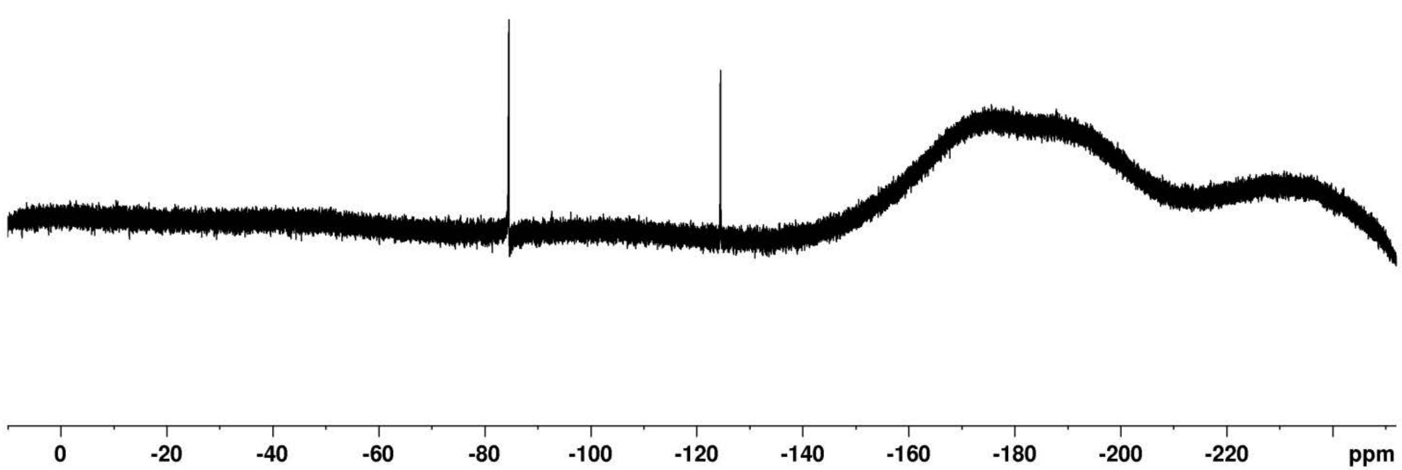

^{1}H NMR (700 MHz, $CDCl_3$) for *2,2,3,3,3-pentafluoropropyl ((2R,3R)-1-hydroxy-3,7-dimethyl-3-(phenylthio)oct-6-en-2-yl)sulfamate* and *2,2,3,3,3-pentafluoropropyl ((2R,3S)-1-hydroxy-3,7-dimethyl-2-(phenylthio)oct-6-en-3-yl)sulfamate.* **Obtained following derivatisation of 9j**

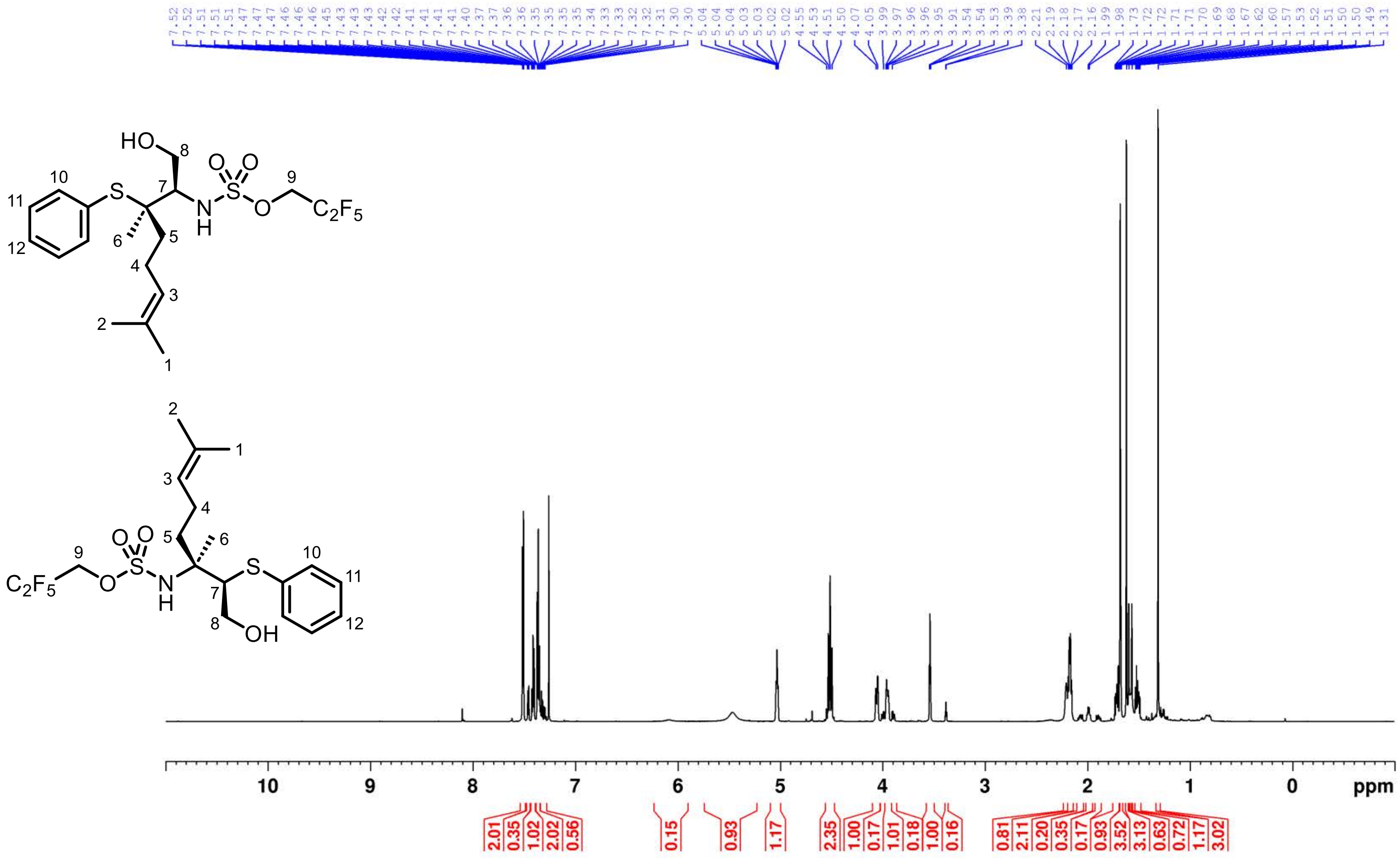

^{13}C NMR (176 MHz, CDCl$_3$) for *2,2,3,3,3-pentafluoropropyl ((2R,3R)-1-hydroxy-3,7-dimethyl-3-(phenylthio)oct-6-en-2-yl)sulfamate* and *2,2,3,3,3-pentafluoropropyl ((2R,3S)-1-hydroxy-3,7-dimethyl-2-(phenylthio)oct-6-en-3-yl)sulfamate.* **Obtained following derivatisation of 9j**

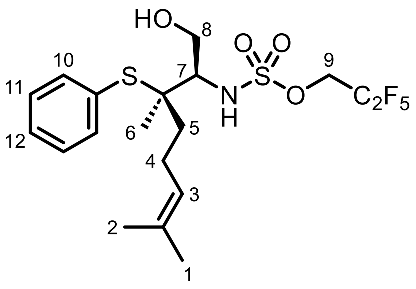

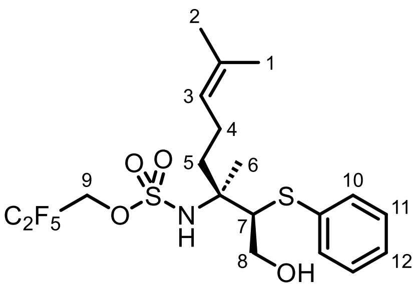

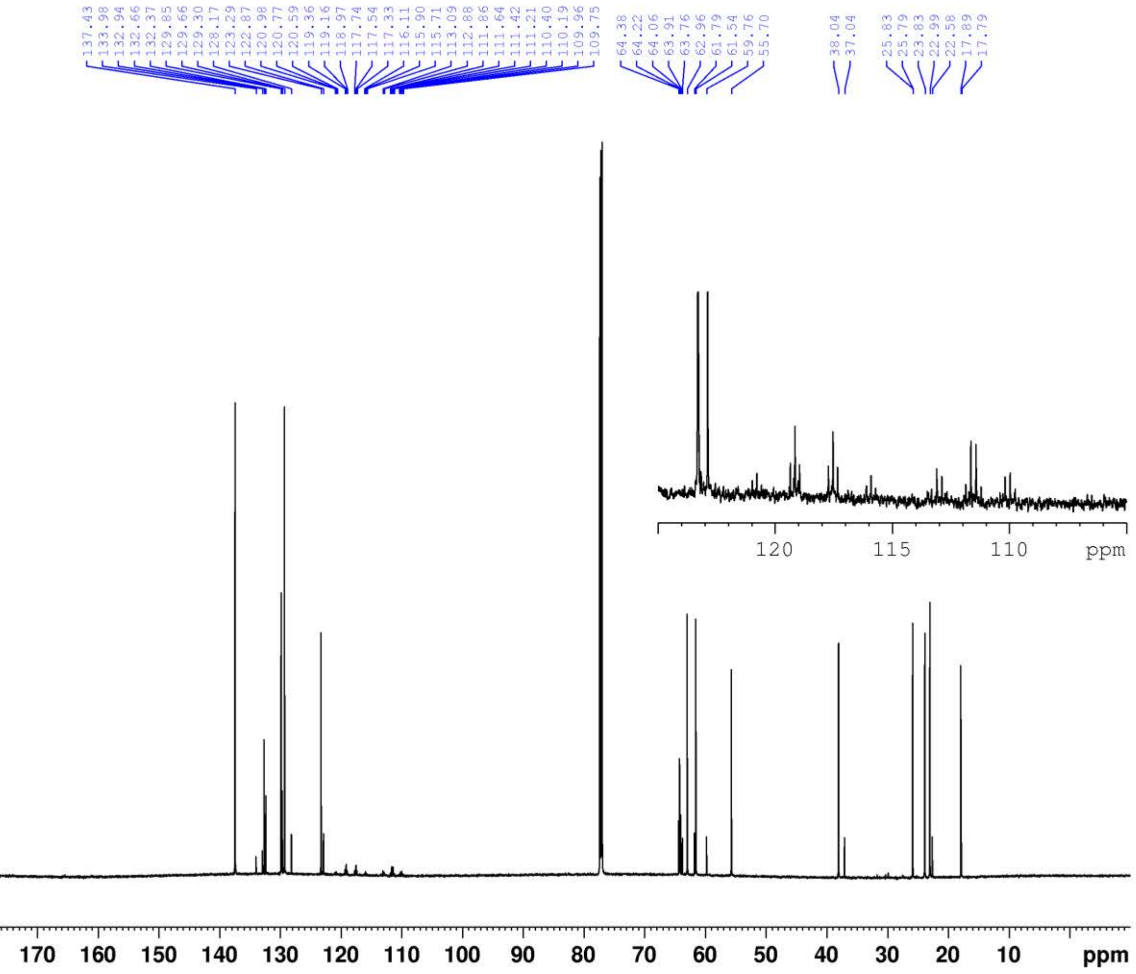

^{19}F NMR (376 MHz, $CDCl_3$) for *2,2,3,3,3-pentafluoropropyl ((2R,3R)-1-hydroxy-3,7-dimethyl-3-(phenylthio)oct-6-en-2-yl)sulfamate* and *2,2,3,3,3-pentafluoropropyl ((2R,3S)-1-hydroxy-3,7-dimethyl-2-(phenylthio)oct-6-en-3-yl)sulfamate.* **Obtained following derivatisation of 9j**

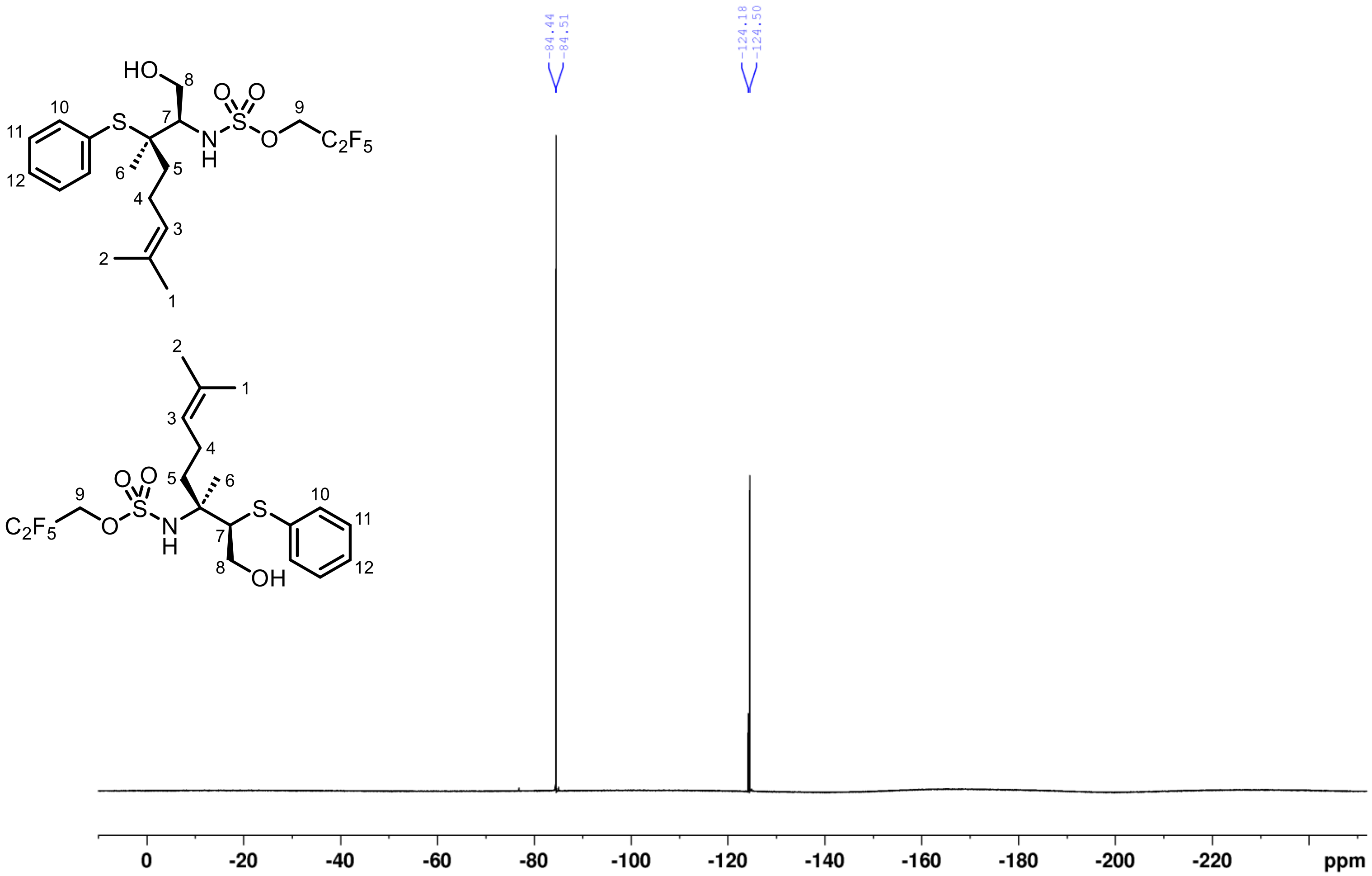

^{1}H NMR (700 MHz, $CDCl_3$) for *2,2,2-trichloroethyl (R)-(1-hydroxy-3-methyl-3-(phenylthio)butan-2-yl)sulfamate* and *2,2,2-trichloroethyl (R)-(4-hydroxy-2-methyl-3-(phenylthio)butan-2-yl)sulfamate*. **Obtained following derivatisation of S2**

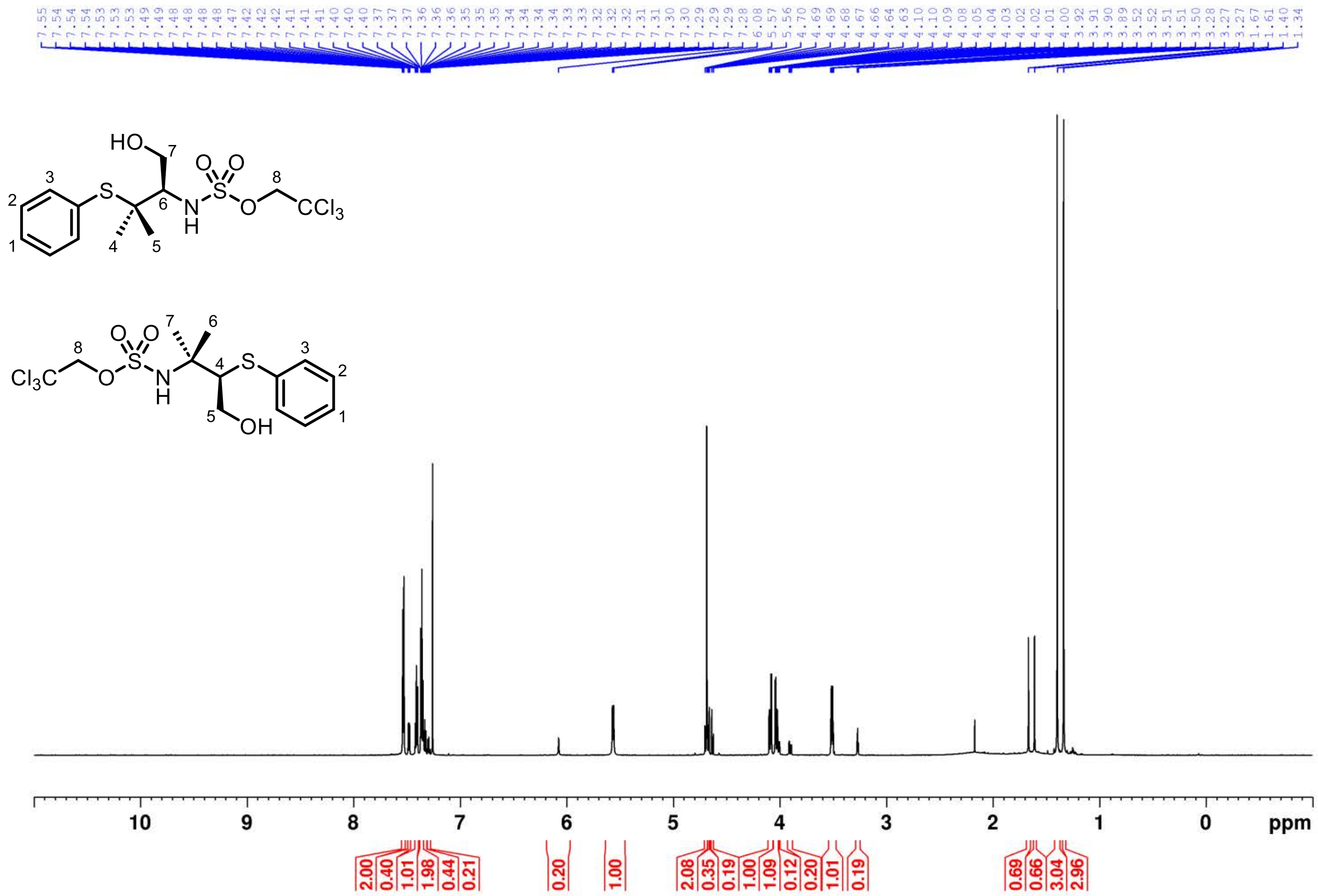

^{13}C NMR (176 MHz, CDCl$_3$) for *2,2,2-trichloroethyl (R)-(1-hydroxy-3-methyl-3-(phenylthio)butan-2-yl)sulfamate* and *2,2,2-trichloroethyl (R)-(4-hydroxy-2-methyl-3-(phenylthio)butan-2-yl)sulfamate*. **Obtained following derivatisation of S2**

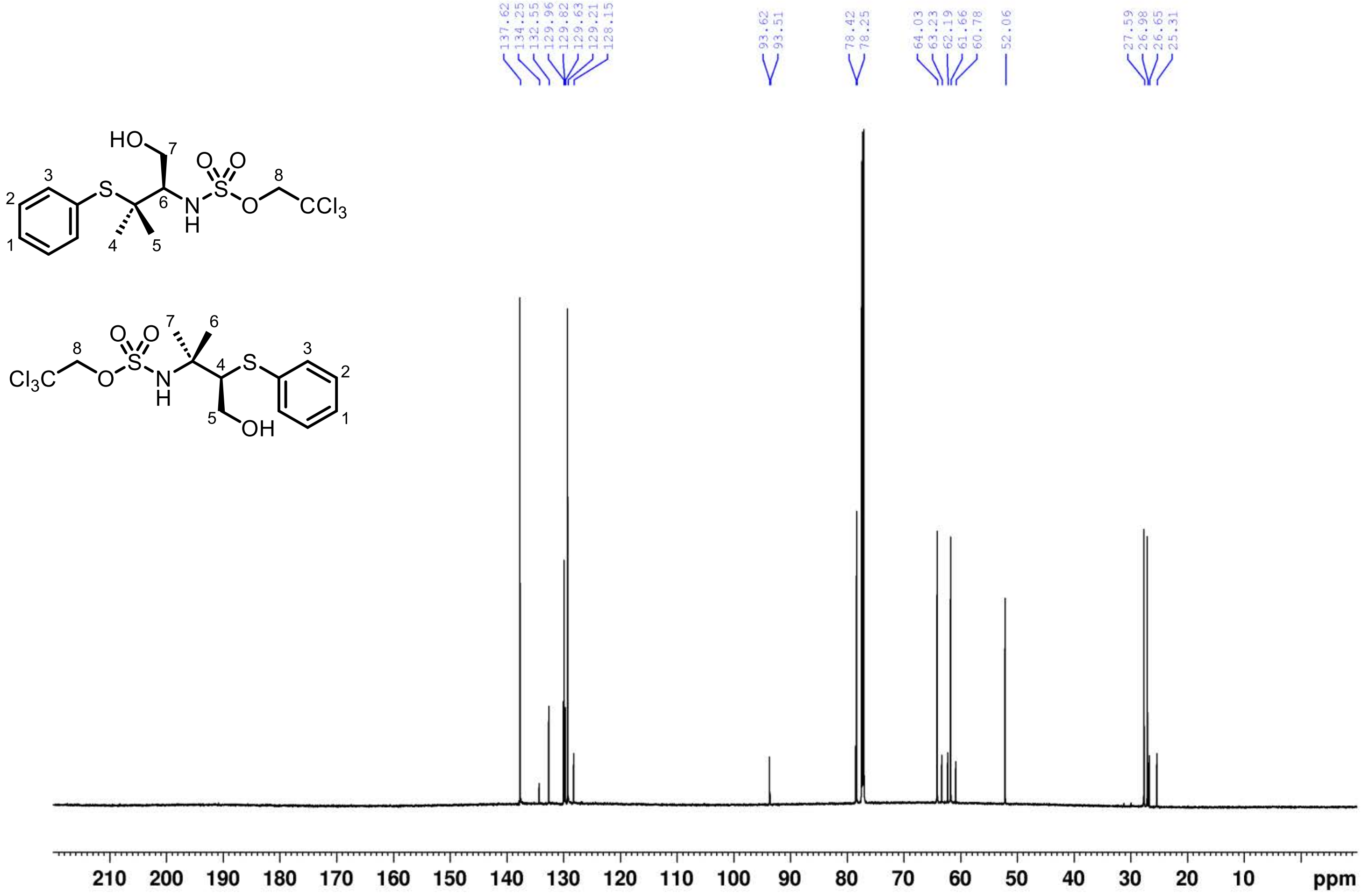

^{1}H NMR (700 MHz, $CDCl_3$) for *2,2,3,3,3-pentafluoropropyl ((2R,3R)-1-hydroxy-3-(phenylthio)hexan-2-yl)sulfamate* and *2,2,3,3,3-pentafluoropropyl ((2R,3S)-1-hydroxy-2-(phenylthio)hexan-3-yl)sulfamate*. **Obtained following aziridination and derivatisation of a *cis*-alkyl allylic alcohol substrate**

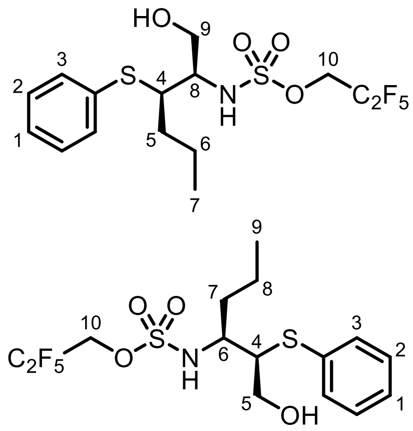

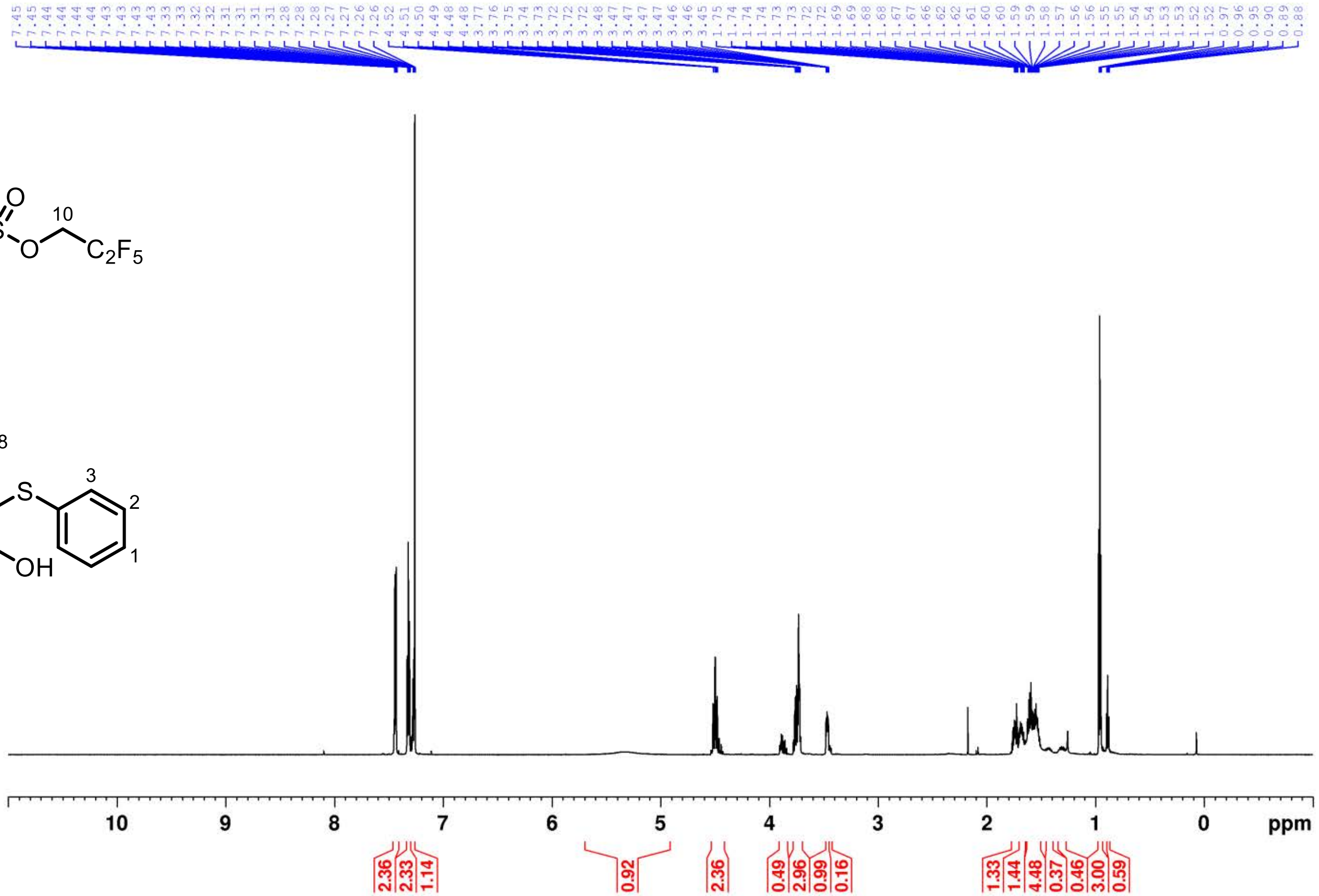

^{13}C NMR (176 MHz, $CDCl_3$) for *2,2,3,3,3-pentafluoropropyl ((2R,3R)-1-hydroxy-3-(phenylthio)hexan-2-yl)sulfamate* and *2,2,3,3,3-pentafluoropropyl ((2R,3S)-1-hydroxy-2-(phenylthio)hexan-3-yl)sulfamate*. **Obtained following aziridination and derivatisation of a *cis*-alkyl allylic alcohol substrate.**

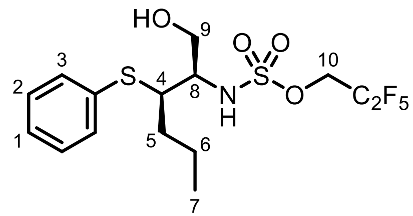

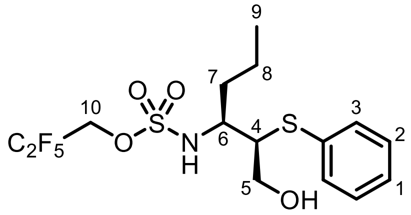

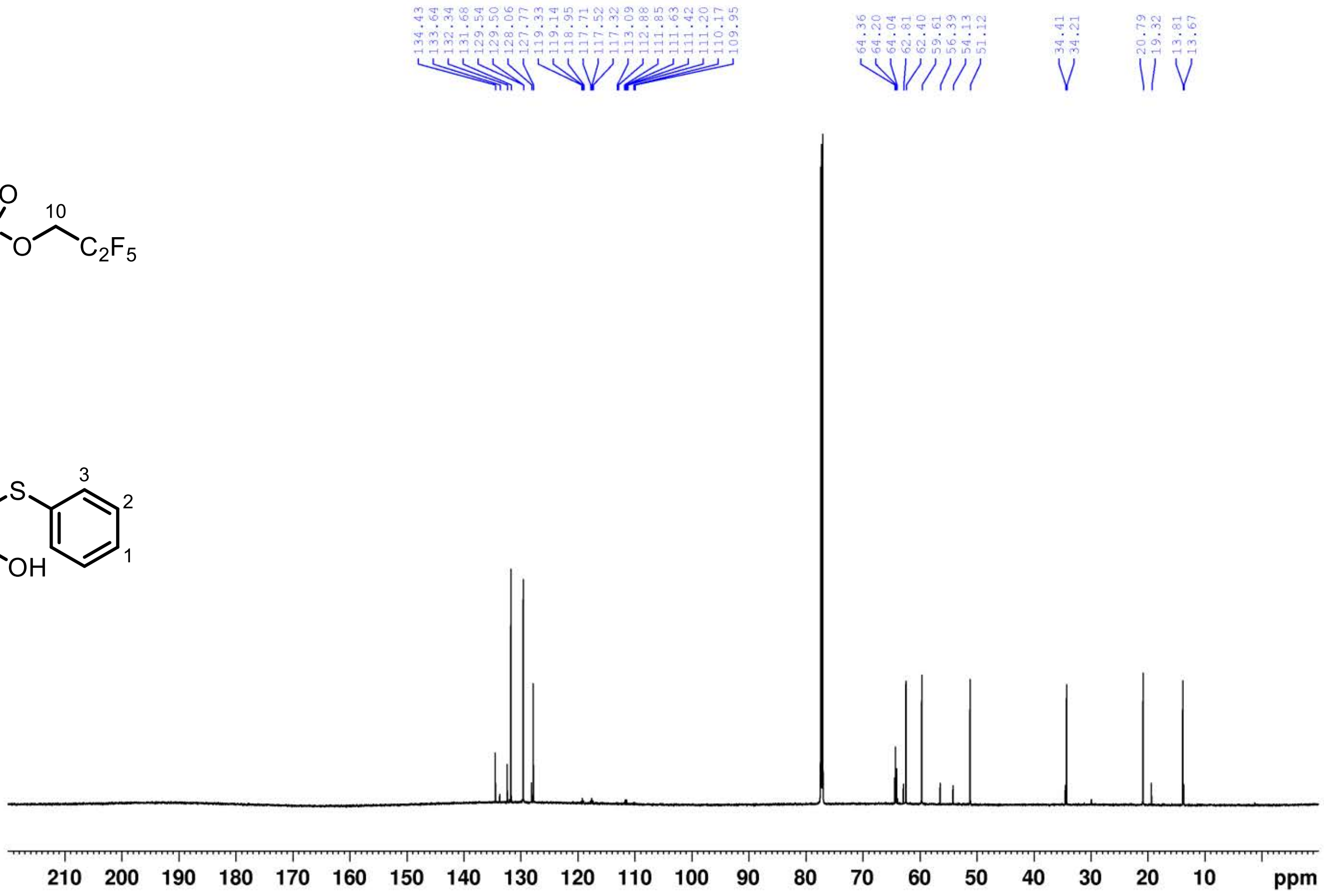

^{19}F NMR (376 MHz, $CDCl_3$) for *2,2,3,3,3-pentafluoropropyl ((2R,3R)-1-hydroxy-3-(phenylthio)hexan-2-yl)sulfamate* and *2,2,3,3,3-pentafluoropropyl ((2R,3S)-1-hydroxy-2-(phenylthio)hexan-3-yl)sulfamate*. **Obtained following aziridination and derivatisation of a *cis*-alkyl allylic alcohol substrate.**

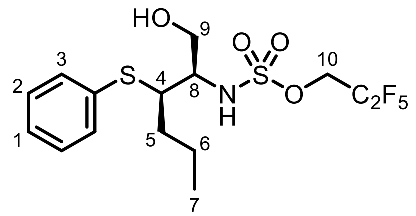

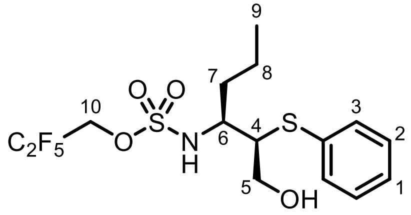

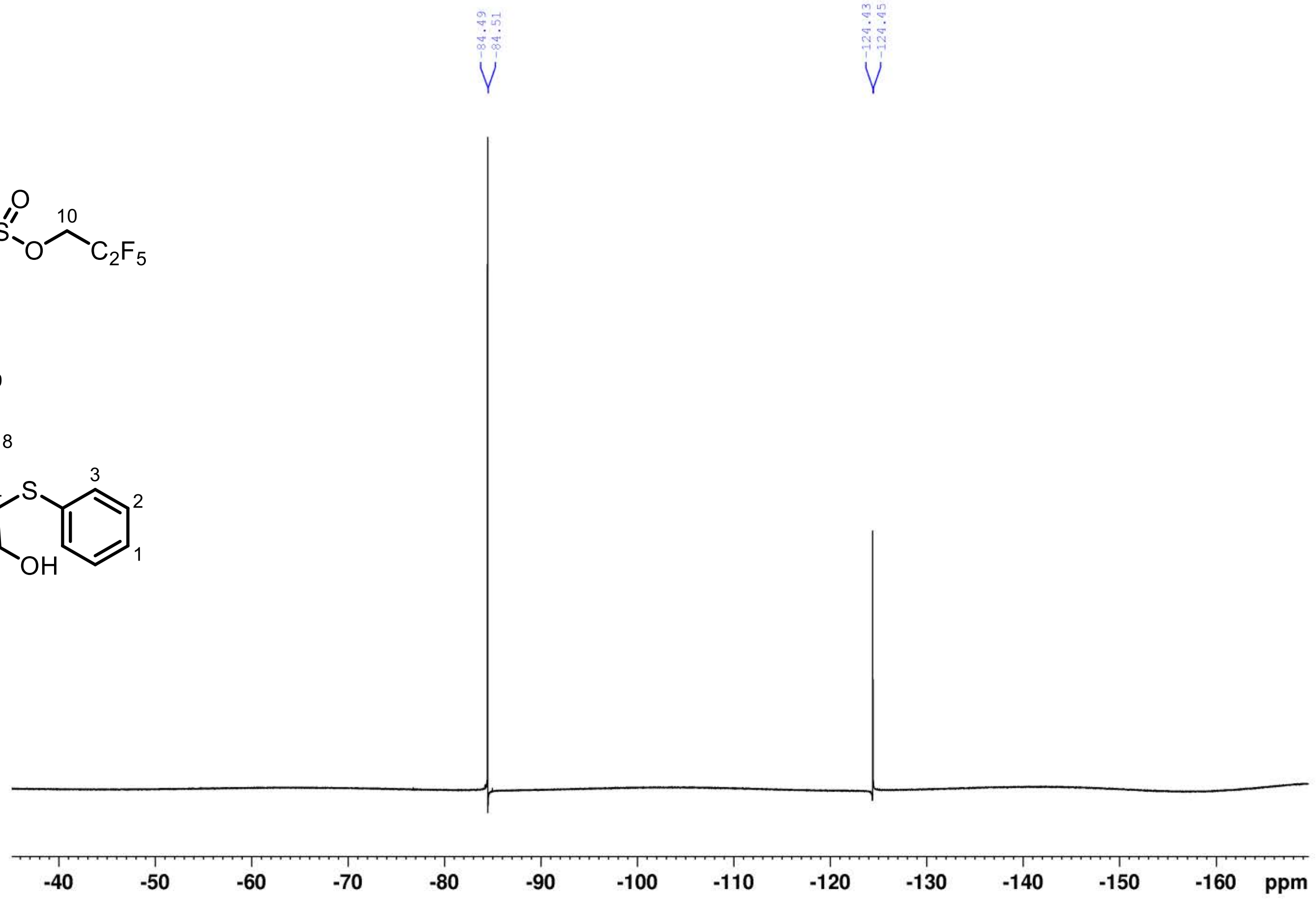

^{1}H NMR (700 MHz, CDCl$_3$) for *2,2,3,3,3-pentafluoropropyl (S)-(1-hydroxy-3-methylbutan-2-yl)sulfamate* (**S5**)

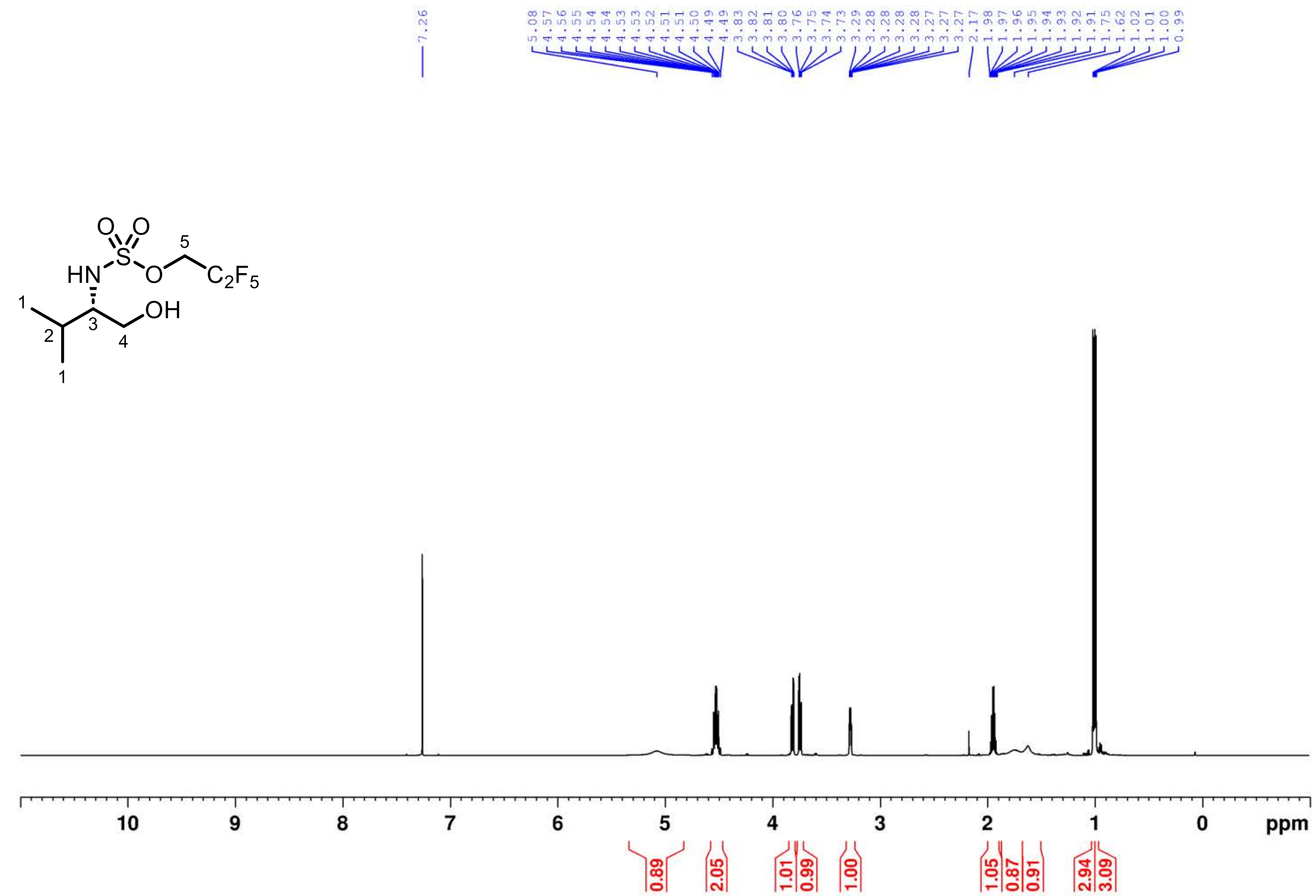

^{13}C NMR (176 MHz, CDCl$_3$) for *2,2,3,3,3-pentafluoropropyl (S)-(1-hydroxy-3-methylbutan-2-yl)sulfamate* (**S5**)

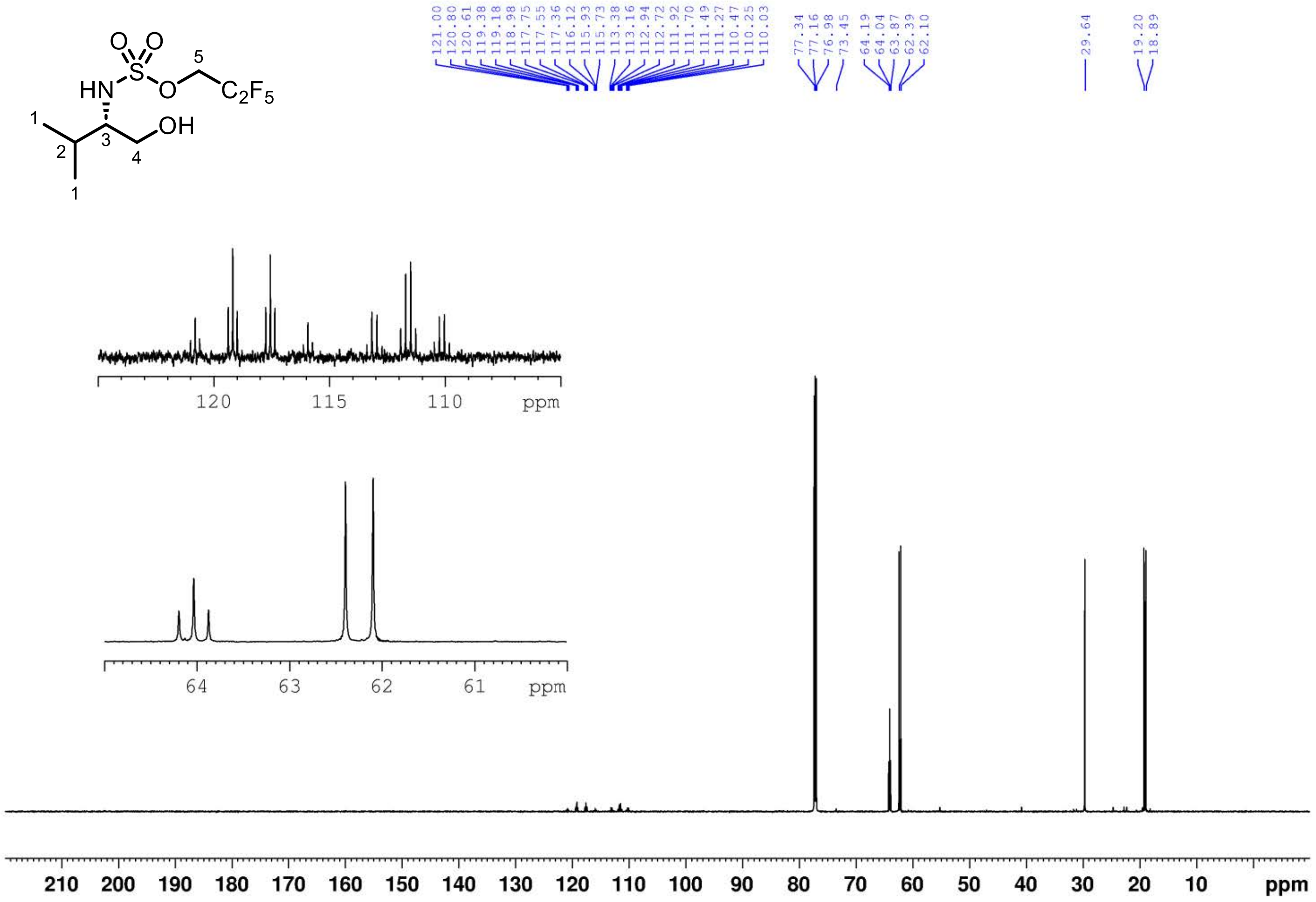

^{19}F NMR (376 MHz, $CDCl_3$) for *2,2,3,3,3-pentafluoropropyl (S)-(1-hydroxy-3-methylbutan-2-yl)sulfamate* (**S5**)

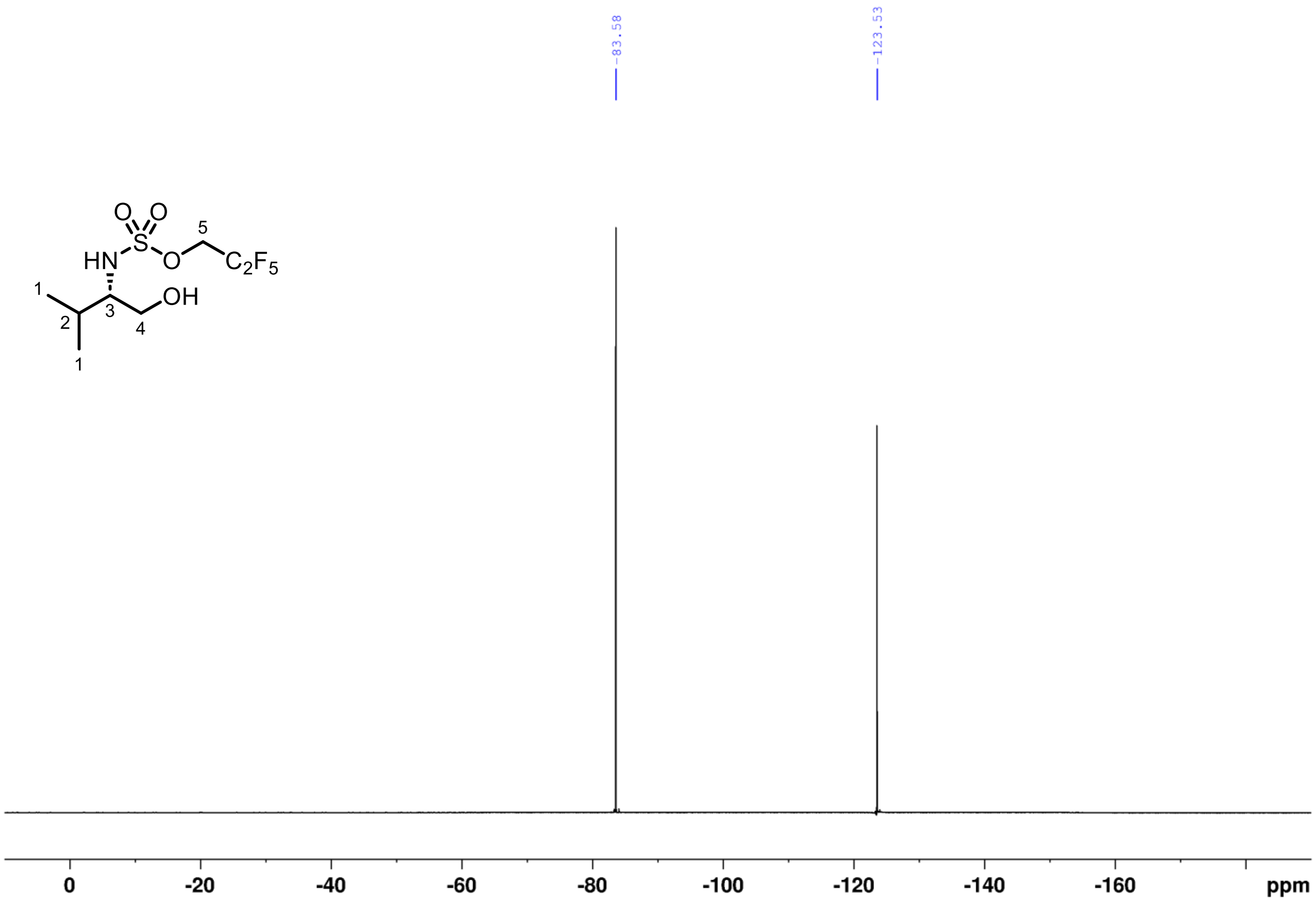

Supplement: Supplementary file 1 — ja4c07117_si_001.pdf [file ja4c07117_si_001.pdf]
